# Host circadian behaviors exert only weak selective pressure on the gut microbiome under stable conditions but are critical for recovery from antibiotic treatment

**DOI:** 10.1371/journal.pbio.3001865

**Published:** 2022-11-09

**Authors:** Chi Zhao, Kevin Kelly, Maria Luísa Jabbur, Marcell Paguaga, Megan Behringer, Carl Hirschie Johnson

**Affiliations:** Department of Biological Sciences & Vanderbilt Microbiome Initiative, Vanderbilt University, Nashville, Tennessee, United States of America; Charité - Universitätsmedizin Berlin, GERMANY

## Abstract

The circadian rhythms of hosts dictate an approximately 24 h transformation in the environment experienced by their gut microbiome. The consequences of this cyclic environment on the intestinal microbiota are barely understood and are likely to have medical ramifications. Can daily rhythmicity in the gut act as a selective pressure that shapes the microbial community? Moreover, given that several bacterial species have been reported to exhibit circadian rhythms themselves, we test here whether a rhythmic environment is a selective pressure that favors clock-harboring bacteria that can anticipate and prepare for consistent daily changes in the environment. We observed that the daily rhythmicity of the mouse gut environment is a stabilizing influence that facilitates microbiotal recovery from antibiotic perturbation. The composition of the microbiome recovers to pretreatment conditions when exposed to consistent daily rhythmicity, whereas in hosts whose feeding and activity patterns are temporally disrupted, microbiotal recovery is incomplete and allows potentially unhealthy opportunists to exploit the temporal disarray. Unexpectedly, we found that in the absence of antibiotic perturbation, the gut microbiome is stable to rhythmic versus disrupted feeding and activity patterns. Comparison of our results with those of other studies reveals an intriguing correlation that a stable microbiome may be resilient to one perturbation alone (e.g., disruption of the daily timing of host behavior and feeding), but not to multiple perturbations in combination. However, after a perturbation of the stable microbiome, a regular daily pattern of host behavior/feeding appears to be essential for the microbiome to recover to the original steady state. Given the inconsistency of daily rhythms in modern human life (e.g., shiftwork, social jet-lag, irregular eating habits), these results emphasize the importance of consistent daily rhythmicity to optimal health not only directly to the host, but also indirectly by preserving the host’s microbiome in the face of perturbations.

## Abstract 2

What happens to our intestinal microbiome if the daily feast/fast cycle driven by our eating habits is disrupted? This study unexpectedly reveals that major disruption to the daily cycle by itself does not have a major effect upon our intestinal microbiome, but full recovery from antibiotic treatment is dramatically improved by a consistently cycling gut environment.

## Introduction

The relationship between host and its microbiota has become one of the most exciting topics in basic and applied biological sciences with unexpected translational implications, e.g., for disorders such as obesity, inflammatory bowel disease, and bacterial vaginosis [1,2]. The temporal dimension of this relationship warrants serious examination, especially given the ubiquity of circadian rhythms in animal hosts [3–6]. At the very least, because most animal hosts (including humans) eat on a daily schedule, the environment experienced by gut microbiota undergoes approximately 24 h transformation in nutrient availability [5,7]. A gut bacterium that can anticipate the arrival of fresh nutrients might more readily metabolize those resources and thus gain the upper hand over time-ignorant competitors. Our knowledge that cyanobacteria harbor circadian timekeepers opens the possibility that bacteria of the microbiome may have endogenous timekeepers that optimize their adaptation to the host [5]. This blossoming area of enquiry has already identified bacteria that are known to inhabit mammalian intestines (*Klebsiella aerogenes* and *Bacillus subtilis)* and which appear to express daily rhythms when isolated outside of the host [8–10]. If gut microbiota are capable of daily timekeeping, what might be the environmental cue(s) whereby their rhythm is entrained to the daily cycle? Within the gut, the microbiota of mammals may not be strongly exposed to the most common entraining agent for known circadian rhythms, namely the solar light/dark (LD) cycle. Nevertheless, because of their host’s behavior and physiology, these intestinal residents encounter daily patterns of nutrient availability, gastrointestinal hormones, body temperature, and levels of host-delivered antimicrobial peptides and gut mucosal antibodies [11,12].

The composition of the fecal microbiota has been reported to show time-of-day differences [4,6,13,14]. There is a bidirectional relationship between circadian rhythms of the host and the intestinal microbiota. The first “direction” is in terms of the effects of the host on its microbiome. Circadian/daily aspects of the host that have been reported to affect the microbiome include the rhythmicity/genotype, diet, jet-lag, and sex/gender. Several studies have reported that the daily patterning of the microbiome’s composition is altered in arhythmic mouse models (e.g., knockout strains of *Bmal1*, *Cry1/2*, or *Per1/2*) [7,13,14]. Daily time restriction of high-fat diet appears to rescue the deleterious effects of high-fat diet ad libitum [14–16]. Mice undergoing a simulated “jet-lag” have been reported to experience an altered gut microbiome that approaches dysbiosis [14,17]. Finally, the daily changes in microbiotal composition are enhanced in female mice as compared with males [13]. The second “direction” in this bidirectional relationship is the effect of the gut microbiome on the host’s circadian system. Circadian transcriptomic changes in the liver and intestine appear to be regulated by fluctuations of microbiota-derived metabolites, including lipids, amino acids, carbohydrates, vitamins, nucleotides, and xenobiotics, as well as other mechanistic linkages [18–20].

Our approach here differs from those of the previous studies; we will consider the host as providing an environment (temporally rhythmic or temporally disrupted) that can exert selective pressures on its microbiota that shape the diversity and composition of the microbial community. Specifically, we test whether rhythmic versus disrupted environments have distinct effects on the microbiome composition either (i) directly without any additional perturbation, or (ii) during the recovery from a strong disturbance induced by antibiotics. Our hypothesis is that the composition of the gut microbiome is maintained under active selection by the host’s daily feeding patterns. We report here that established, stable microbiomes are robust and compositional differences that result due to the distinct selective pressures of strongly rhythmic versus temporally inconsistent feeding/activity patterns of the host are limited to a small number of species. However, microbiomes that have been destabilized by antibiotic perturbation are dramatically influenced by the circadian rhythmicity of the host as the final recovery kinetics and ultimate steady state of many species differ greatly between strongly rhythmic and temporally inconsistent environments. Our investigation provides a novel assessment of real-time population dynamics and selection within the microbiome in response to perturbations of environmental conditions, with clear translational import.

## Results

### Feeding and activity patterns of WT versus Per1/2-dko mice in LD and RR

**Fig 1:**
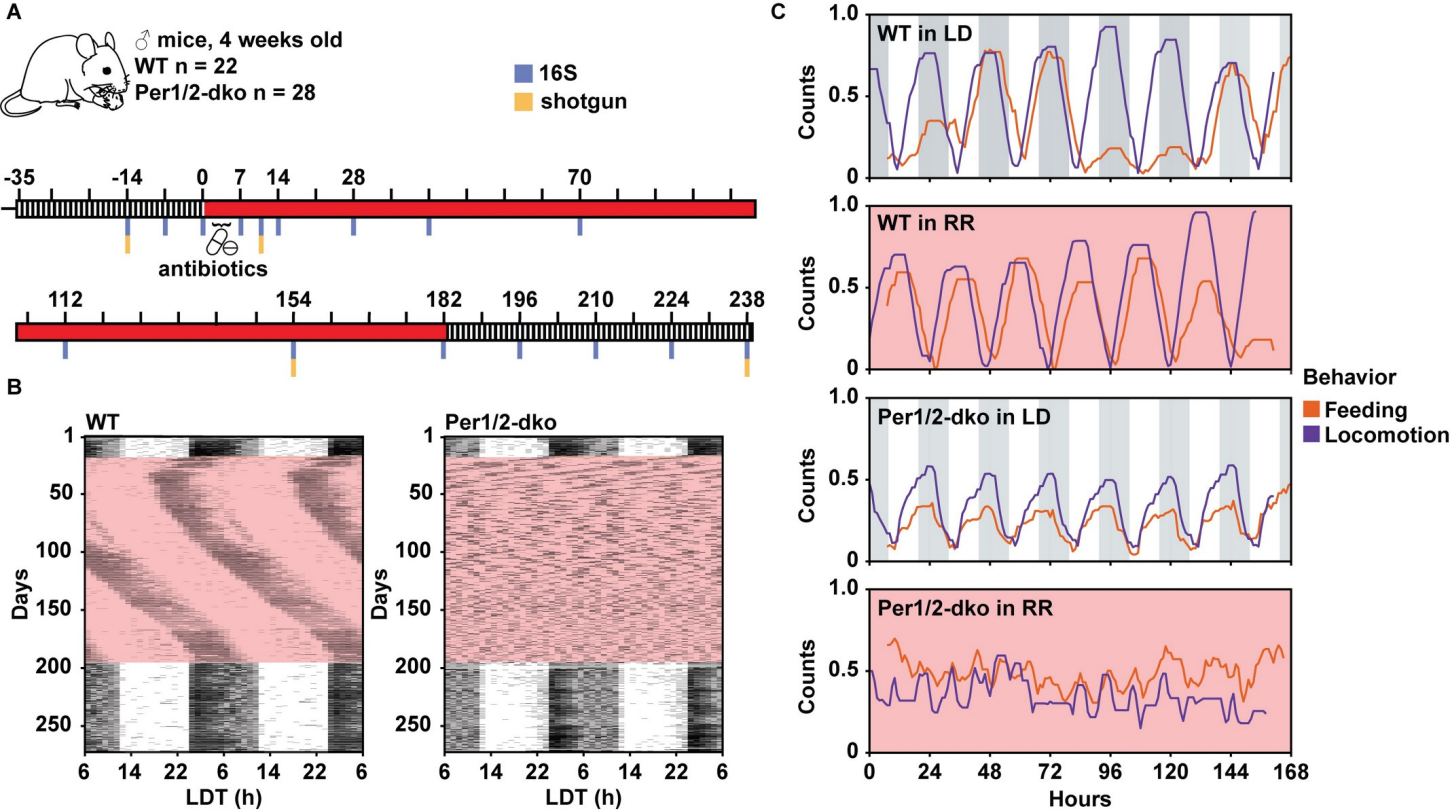
Protocol and characterization of the Per1/2-dko mouse model in RR. (A) The LD/RR/LD regimen of this experiment started with entrainment to LD 12:12 for 35 days, then RR for 182 days, and concluded with a return to LD for an additional 56 days. Black/white stripes indicate LD conditions and red indicates RR. Vertical blue bars indicate the time points of stool collection for 16S sequencing, and the additional yellow bars show the 4 samples that were additionally processed for shotgun sequencing. For the fecal collection samples, there were 22 WT and 28 Per1/2-dko mice, all starting at 4 weeks of age. On the first day of RR (Day 0) and for 5 days thereafter, half of the mice were treated with an antibiotic cocktail in their drinking water (*n* = 11 for WT, *n* = 13 for Per1/2-dko). The samples were labeled as: WT(UT) and Per(UT) for the untreated WT and Per1/2-dko groups, respectively, and WT(T) and Per(T) for the groups treated with antibiotics. (B) Wheel-running locomotor activity of singly housed WT and Per1/2-dko mice. The mice used for activity and feeding experiments were a separate group from those used for stool collection, but were exposed to the same regimen as shown in panel A (the data of the 2 mice shown in panel B are representative examples of each corresponding group). Both genotypes of mice were entrained well by LD at the beginning. In RR, WT mice were still rhythmic (left panel), whereas Per mice had severely disrupted patterns (right panel). Both genotypes could still be entrained in LD after the exposure to RR. See S1 Table for statistical analyses and S1 Data File for the data that are analyzed in S1 Table. (C) Feeding and locomotor activities of representative individual mice were recorded for 7 days interspersed throughout the varying LD/RR conditions (see S2 Table). Locomotor activity is represented by the purple lines and the feeding activity shown by orange lines. “Counts” on the ordinate is locomotor activity or feeding behavior events organized in 1-h bins and normalized as described in Methods. See for statistical analyses and S1 Data File for the data that are analyzed in S2 Table. LD, light/dark; Per1/2-dko, Per1/Per2 double knockout mice; RR; red light; WT, wild-type mice.

To test our hypothesis that the gut microbiome is under active selection by the host’s daily feeding patterns, we developed a novel model that combines a clock-knockout strain with constant dim red light (RR) for disrupted rhythmicity of feeding under conditions in which fecal samples could be easily collected without disturbing the light/dark conditions of the experiment. Our protocol for fecal collection comprised LD entrainment for 5 weeks, then RR for 26 weeks, and concluded with a return to LD for an additional 8 weeks (Fig 1A). We used mice in which 2 central circadian clock genes *Period1* and *Period2* were both knocked out (Per1/2-dko [21]). In the standard circadian mouse C57 background, these Per1/2-dko mice express robustly rhythmic nocturnal activity patterns in LD and arhythmicity and/or highly disrupted activity patterns in constant darkness (DD; [22]), whereas wild-type (WT) mice maintain robust nocturnal activity rhythms in LD and free-running rhythmicity in constant DD conditions with a period approximately 24 h. Per1/2-dko mice are fertile and healthy, unlike another commonly used clock-disrupted mouse strain, the *Bmal1*-ko mouse, that suffers from a variety of health problems (short lifespan, arthropathy, infertility, etc. [23–26]), and has been used in some other microbiome studies [13]. Moreover, the microbiome responses in Per1/2-dko mice to time-restricted feeding and jet-lag paradigms have already been characterized [14,27].

This study is a longitudinal analysis where each mouse genotype serves as its own control on the basis of comparisons pretreatment versus posttreatment. The treatments include both alterations in the light/dark conditions and/or antibiotics included in the drinking water. We tested the temporal patterns of locomotor activity and feeding of both strains in both LD cycles and uninterrupted constant conditions. For constant conditions, we used dim red light (RR) conditions rather than constant darkness (DD) to visually facilitate fecal sample collection (constant *white* light is known to suppress rhythmicity, even in WT). As shown in Fig 1B, the wheel-running locomotor activity of WT mice is strongly rhythmic with a 24-h period in LD 12:12, and sustains approximately 24 h but slightly longer period free-running rhythms in RR (period approximately 24.5 h, S1 Table). On the other hand, while the locomotor activity of Per1/2-dko mice remains highly rhythmic in LD (period = 24 h), it is dramatically disrupted in RR, where a variety of fragmented patterns are observed (often in the same mouse) from arhythmic to ultradian to very short period (ostensibly averaging 13.3 h, but note the dramatic loss of power that indicates arhythmicity and/or highly disrupted patterns, S1 Table).

In parallel experiments, we used infrared video recordings to quantify temporal feeding patterns of the WT and Per1/2-dko mice and found that feeding behavior under the LD and RR regimens corresponded closely to the locomotor activity patterns. Under a regimen of LD for 6 weeks, dim RR for 23 weeks and then back to LD for the remaining 10 weeks, we confirmed strong daily rhythms of both locomotor activity and feeding in LD for both strains (Fig 1C); in RR, WT mice retain robust daily rhythms of both kinds of activity, while Per1/2-dko mice exhibit highly disrupted patterns of both activity and feeding behavior in RR that are ultradian or arhythmic (Fig 1C and further analyzed in S2 Table; note the pronounced loss of power in the Per1/2-dko mice in RR). There were no obvious differences in the locomotor activity or feeding behavior between the antibiotic-treated and the untreated groups of mice.

### Response of gut microbiome to rhythmic versus arhythmic host behaviors

**Fig 2:**
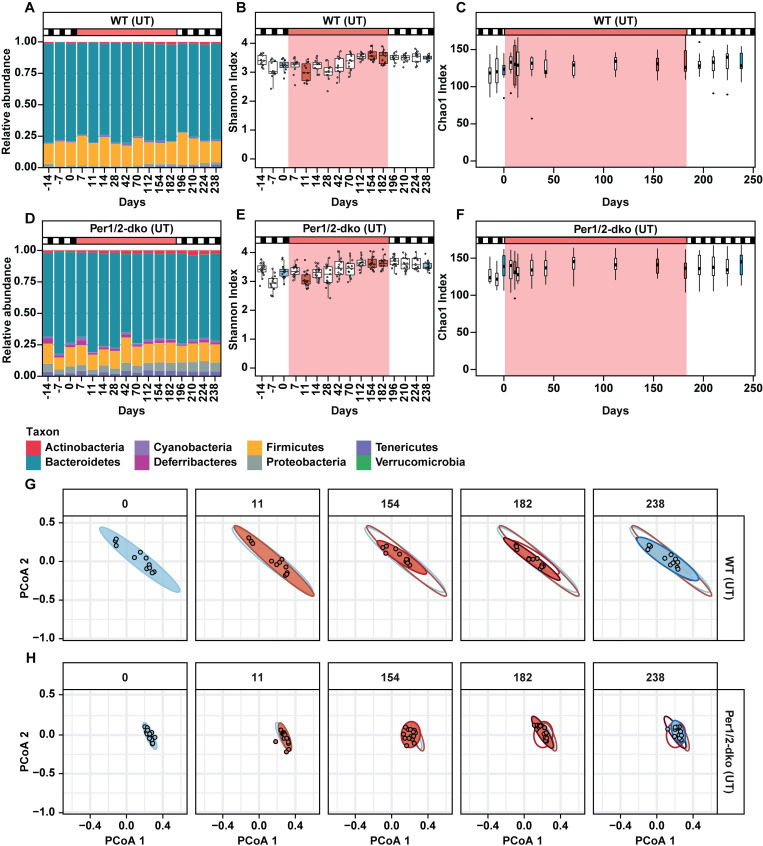
Alpha- and beta-diversity characterizations of the responses of gut microbiome to rhythmic vs. non-rhythmic host behaviors. Sample groups are labeled as in Fig 1A: WT(UT) and Per(UT) for the untreated WT and Per1/2-dko groups that were transferred from LD to RR on Day 0 and back to LD on Day 182. The data used to compile this figure are included in S1 Data File. (A and D) Quantification of the average relative abundances of the microbiota at the phylum level based on 16S sequences at various time points during the regimen in the 4 groups. The upper abscissae for panels A–F indicate LD conditions (black/white boxes) and RR (red). (B and E) Shannon index analyses of alpha-diversity of the microbiota at various time points during the regimen in the 4 groups. The Shannon diversity index is a measure of biodiversity that deals with richness and evenness of species [28]. The data of this diversity index are plotted with the time points equally spaced along the abscissae to mostly emphasize the significance level of each data point. Colored boxes refer to the time points shown in panels G and H (blue for LD time points and red/pink for RR time points). (C and F) Chao1 diversity analyses of alpha-diversity of the microbiota at various time points during the regimen in the 4 groups to assess richness [30]. The data of this diversity index are plotted with the time points on a linear timescale along the abscissae to clearly emphasize the time-dependent changes in alpha-diversity. Colored boxes refer to the time points shown in panels G and H (blue for LD time points and red/pink for RR time points). (G and H) Beta-diversity analyses for WT(UT) and Per(UT) samples. The day of stool collection is labeled along the top; 5 key time points are shown in this figure, and all 16 time points are shown in S1 Video and S2 Fig. The axes are Principal Component 1 (PCoA1, abscissa) and Principal Component 2 (PCoA2, ordinate). Individual data points are shown for each new time point (open circles), and data of previous time points are shown as shaded ellipses that are color-coded as blue for LD time points and red/pink for RR time points. LD, light/dark; Per1/2-dko, Per1/Per2 double knockout mice; RR; red light; WT, wild-type mice.

Based upon the temporal patterns of feeding and locomotion in the 2 mouse strains, we tested whether temporally disrupted versus rhythmic behaviors (feeding, etc.) select for differing microbiotal composition and communities using the LD/RR/LD protocol shown in Fig 1A. Prior to treatments, there were no significant differences in the microbiome diversity, neither between the WT versus Per1/2-dko genotypes nor among the pretreatment time points (Table 1; based on the Shannon diversity index that assesses richness and evenness of species [28]). Thus, the pretreatment samples within each genotype serve as controls for the treatment- and time-dependent changes in the microbiomes in this longitudinal design. Quantification of the microbiota using 16S sequence analyses indicated that the relative abundances at the phylum level were not obviously changed by the LD to RR to LD transitions alone (the antibiotic-untreated or “UT” samples, Fig 2A and 2D). The total bacterial load in the UT stool samples did not change significantly among these LD/RR/LD transitions, although there was a downward trend in bacterial load over approximately 8 month timecourse (S1 Fig). To refine our assessments of microbiotal diversity, evenness, and richness of operational taxonomic units (OTUs; from phylum to species), we applied the commonly used Shannon and Chao1 alpha-diversity indices [28–30]. The Shannon diversity index is a measure of biodiversity that deals with richness (the breadth of differences among OTUs) and evenness (abundance of specific OTUs), while the Chao1 index focuses upon richness [28–30]. The alpha-diversity Shannon and Chao1 indices suggest a slight destabilization of microbiome diversity at the beginning of the RR treatment that stabilizes later in RR and continues in the final LD segment (“UT” samples, Fig 2B and 2E for Shannon, Fig 2C and 2F for Chao1). Assessments of diversity with the Chao1 metric (Fig 2C and 2F, plotted on a linear time scale) largely agrees with the Shannon diversity metric (Fig 2B and 2E, plotted on an equidistance timescale). Importantly, while there are some minor differences in the relative abundances at the phylum level between the WT(UT) and Per(UT) samples (Fig 2A versus 2D), there is no obvious difference in the long-term response of the microbiome to rhythmic {WT(UT)} versus arhythmic {Per(UT)} behaviors in RR as indicated by the alpha-diversity indices (compare Fig 2B and 2C with 2E and 2F).

**Table 1:** Mann–Whitney U test for Shannon indices of pretreatment samples.

Samples	*P* value	Sample size(s)	Medians	Difference: Actual	Difference: Hodges–Lehmann
**Genotype (WT vs. Per1/2-dko)**	0.9752	22, 28	3.524, 3.521	−0.003648	0.003251
**Time for WT mice**	0.0845	22	3.564	−0.1679	−0.1168
**Time for Per1/2-dko mice**	0.3405	28	3.569	−0.09584	−0.06275

“Genotype” compares the pretreatment samples (Days −14 to 0) between WT and Per1/2-dko mice.

**Fig 3:**
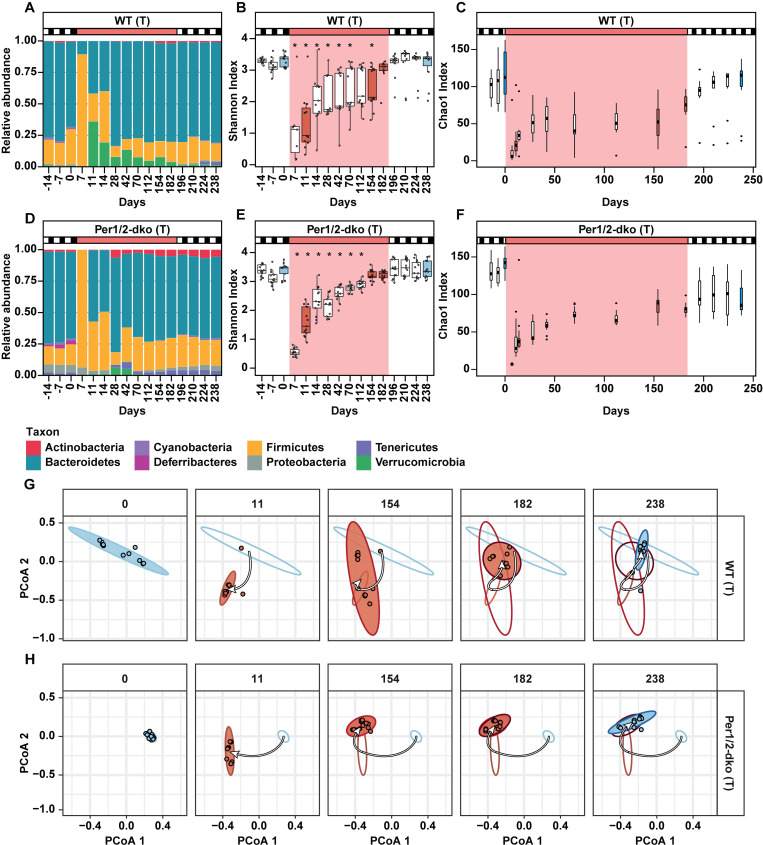
Alpha- and beta-diversity characterizations of the responses of gut microbiome to rhythmic vs. non-rhythmic host behaviors after brief perturbation from antibiotics. Sample groups are labeled as in Fig 1A: WT(T) and Per(T) for the groups treated with antibiotics on Days 0–5 (transferred from LD to RR on Day 0, and back to LD on Day 182). Data are plotted as in Fig 2. The data used to compile this figure are included in S1 Data File. (A and D) Quantification of the average relative abundances of the microbiota at the phylum level based on 16S sequences at various time points during the regimen in the 4 groups. The upper abscissae for panels A–F indicate LD conditions (black/white boxes) and RR (red). (B and E) Shannon index analyses of alpha-diversity of the microbiota at various time points during the regimen in the 4 groups, plotted as in Fig 2. (C and F) Chao1 diversity analyses of alpha-diversity of the microbiota, plotted as in Fig 2. (G and H) Beta-diversity analyses for WT(T) and Per(T) samples, plotted as in Fig 2. White arrows indicate the trajectory of the center of the ellipses through the time points shown. The day of stool collection is labeled along the top; 5 key time points are shown in this figure, and all 16 time points are shown in S1 Video and S3 Fig. LD, light/dark; Per1/2-dko, Per1/Per2 double knockout mice; RR; red light; WT, wild-type mice.

“Time” compares among the pretreatment samples (Days −14 to 0) for WT and Per1/2-dko separately. The data for this table are tabulated in S1 Data File (tabs for Figs 2B, 2E, 3B, and 3E, columns for Day −14 and Day 0).

We then proceeded to a complementary assessment, namely beta-diversity, which quantifies the compositional dissimilarity between treatments/environments that in our case are the temporal changes in the gut environment. Differences in microbiotal composition can be visualized by the Bray–Curtis beta-diversity index [29,31] and graphed by principal coordinates analysis (PCoA) dimensionality. Fig 2G and 2H summarize the PCoA analysis for 5 key time points; all 16 time points are shown in S1 Video and S2 Fig (S1 Video is organized as a video animation). By this complementary beta-diversity assay, the long-term microbiotal compositions are not changing significantly in the WT(UT) samples as the mice transition from LD to RR and back to LD (rhythmic behavior throughout; Figs 1, 2G, and S2). Surprisingly, neither is the microbiotal composition changing in the Per(UT) samples that are undergoing a transition from rhythmic behavior (first LD) to temporally disrupted behavior (RR) and back to rhythmic behavior (second LD) (Figs 1, 2H, and S2). Based on the results of prior short-term studies with other arhythmic mouse models, “jet-lag” simulations, and other tests of circadian disruption [13–17], this is an unexpected result, the implications of which will be addressed in the Discussion. Note that these alpha- and beta-diversity indices do not necessarily indicate that microbiotal composition was completely unchanged by the LD/RR/LD transitions. This issue will be addressed and confirmed by our shotgun sequencing analyses below.

### Response of microbiome to rhythmicity versus arhythmicity after antibiotic perturbation

In addition, we performed a perturbation analysis by testing whether rhythmic versus disrupted feeding alters the recovery of the microbiome from a brief antibiotic treatment. This perturbation analysis was accomplished by treating approximately half of the mice in each group (*n* = 11 for WT, *n* = 13 for Per1/2-dko) for the first 5 days of RR with an antibiotic cocktail that dramatically knocks down both gram+ and gram- bacteria (vancomycin, streptomycin, and metronidazole [32,33]), hereinafter referred to as the WT(T) and Per(T) groups. This knockdown is clearly indicated by the decrease of total bacterial load in response to the antibiotic treatment (S1E and S1F Fig). The 16S sequence analyses confirmed that the antibiotic treatment dramatically changed the relative proportions among the bacterial phyla (Fig 3A and 3D). For example, in both WT(T) and Per(T) samples, Bacteroidetes bacteria were strikingly diminished by antibiotics and thereafter gradually recovered over the next weeks in RR. Interestingly, Verrucomicrobia bacteria became a significantly larger proportion of the microbiome in the WT(T) samples after the antibiotic knockdown, but this effect was much less prominent in the Per(T) samples. Tables that detail the specific genera and species that respond to the antibiotic treatment are included in the Supporting information (S3 Table for genera and S4 Table for species). As in the case for the UT samples (Fig 2), we then applied Shannon and Chao1 alpha-diversity indices [28–30] to assess microbiotal diversity, evenness, and richness of OTUs. These indices highlighted the dramatic perturbation by the antibiotics in the treated samples (Fig 3B, 3C, 3E and 3F). By these alpha-diversity analyses, both the WT(T) and the Per(T) samples appeared to recover over 6 months from the antibiotic knockdown during the RR treatment. Changes in the taxonomic composition will be addressed by our shotgun sequencing analyses below.

The complementary beta-diversity index [29,31] was enlightening towards visualizing the differences between the WT(T) versus Per(T) samples as they recovered from the brief perturbation. Fig 3G and 3H shows 5 key time points in which arrows track the recovery trajectories (and all 16 time points are animated in S1 Video and graphed in S3 Fig). Just before the antibiotic/RR treatment (e.g., Day 0), the beta-diversity of both WT and Per1/2-dko mice is essentially the same (the WT(T) samples are somewhat more distributed in the PCoA1 dimension than the Per(T) samples), and their response to the antibiotic knockdown is comparable (Day 11, which is 6 days after the antibiotic treatment ended). Remarkably, the microbiome diversity of WT mice that feed with approximately 24-h rhythms in RR (Fig 1) largely recovers from the antibiotic perturbation by Day 182 in RR, and finally recovers to its original PCoA values in the final LD {WT(T), Day 238 in Figs 3G and S3}. In contrast, the beta-diversity of the microbiome from the temporally disrupted Per1/2-dko environment never entirely recovers to its initial starting diversity; even 8 further weeks in LD (microbiomes in now rhythmically behaving hosts) allowed minimal further progress towards recovery {Per(T), Day 238 in Figs 3H and S3}. Therefore, even though the alpha-diversity metrics suggested that both WT(T) and Per(T) microbiomes recover over 6 months from the antibiotic knockdown during the RR treatment, the beta-diversity metrics reveal that this “recovery” is associated with major compositional changes in the communities cultivated in the Per(T) environment.

It is as though the disrupted feeding patterns lock the microbiotal composition into a new steady state that remains largely intransient. There appear to be no major differences between the 2 mouse strains in terms of how their microbiotal diversity responds to LD/RR transitions (Fig 2G and 2H) or initially to antibiotic perturbations (Fig 3B, 3C and 3E–3H, Day 11; also see S3 and S4 Tables). However, in the mice treated with antimicrobials, the longitudinal recovery patterns of the microbiota differ significantly between mice that consistently maintain rhythmicity {WT(T)} versus temporally disrupted behavior {Per(T)}. Here, the abundance and presence/absence of microbial species in Per(T) mice failed to recover to its initial state following the return to LD (PERMDISP: Bray–Curtis: *P* = 2.38 × 10^−10^; Jaccard: *P* = 3.08 × 10^−9^), whereas the microbiota of WT(T) mice largely recovers in this timeframe (PERMDISP: Bray–Curtis: *P* = 0.46701; Jaccard *P* = 0.49837) [29,34–36]. Note that antibiotic treatment not only affects microbial abundance and diversity, but also has short-term effects on the gut epithelium, including the characteristics of circadian rhythmicity in intestinal epithelial cells [19]. However, as the perturbation inflicted in this study is a brief 5-day treatment with antibiotics, and the mucosal epithelial layer is reported to recover from antibiotic treatment within 3 weeks [37], any effects on the epithelium should not significantly affect the microbiome on the long-term time scale that we are analyzing. An unavoidable consequence of our long-term protocol is that the mice are aging for approximately 9 months during the procedure and this might have influenced the recovery from antibiotics, but this factor is also true for the untreated groups that exhibit minimal changes over time (Fig 2) and therefore aging appears to not be a major factor by itself.

In the WT(T) samples, we observed an interesting bifurcation in the kinetics of the return in RR to the initial PCoA coordinates. As seen in S4A Fig, at Day 42 the 4 mice in one of the group-housed cages (Cage#4) have dissociated from the main group and are practically back to the initial conditions, whereas it takes until Day 182 for the singly housed mice and the other group-housed cage to eventually return to the PCoA region of the initial LD samples (note that the microbiome of the singly housed mouse in Cage#1 was unexpectedly not influenced by the antibiotics in the drinking water). This bimodality of return is consistent with the conclusion that the microbiomes of animals that are housed together tend to be similar [38,39], and that the microbiomes of 1 or more mice in Cage#4 reverted to initial LD conditions more quickly and influenced the more rapid restoration of the microbiome diversity of the other mice in that cage. Our experimental design of including both types of housing therefore confirms that our perturbation analysis yields the same final results independently of the housing conditions. Interestingly, no such bifurcation was observed in the Per(T) samples—both singly and group-housed mice recovered partially (but only partially) towards the initial conditions in coherent distributions (S4B Fig).

### Analyses at species/genera levels based on shotgun sequencing

**Fig 4:**
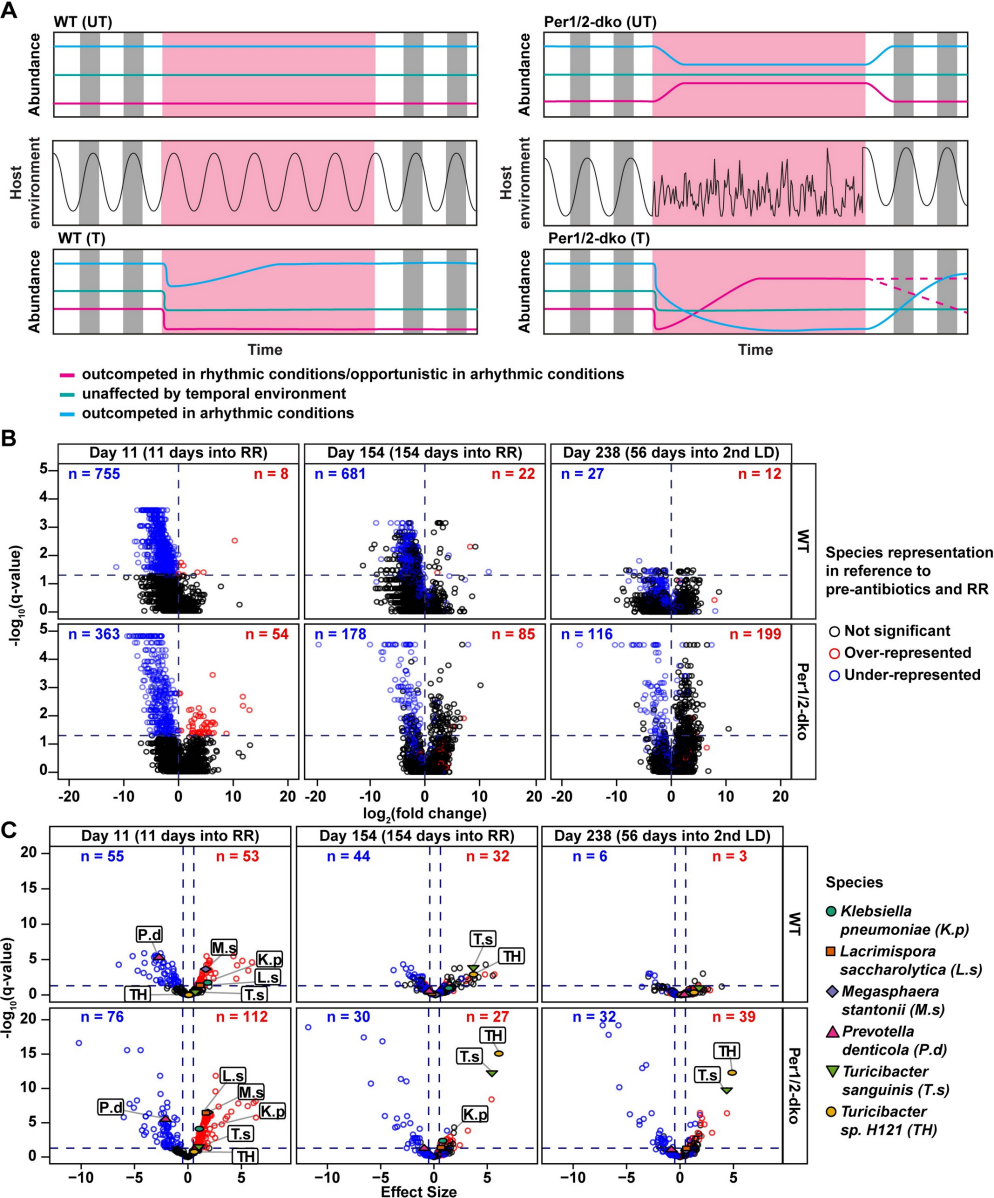
Predictions based on temporal control of the microbiome and volcano plots for changes in species composition during recovery from antibiotic perturbation. (**A**) Predictions based on temporal control of the microbiome based on transitions between LD (vertical gray/white bars) and RR (pink) conditions for the microbiomes of WT (left panels) or Per1/2-dko (right panels). The middle panels indicate whether the host environments under these conditions will be strongly rhythmic versus temporally disrupted. Predictions of the changes in abundances of bacteria are illustrated in response to these environmental transitions in the absence of an antibiotic perturbation (“UT,” upper panels) versus when a brief antibiotic perturbation is included at the beginning of RR (“T,” lower panels). The hypothetical bacterial responses are indicated in the key below the lower left panel. See S5 and S7 Tables for experimental data relating to these predictions. (**B**) Volcano plots depicting the changes in representation for the 5,421 species identified in our WT and Per1/2-dko individuals that were treated with antibiotics. For this analysis, all species were considered, even those with very low abundance. The q-value reflects the results of our Mann–Whitney *U* test followed by a *p*-value adjustment for false discovery rate [40,45,46] in which each experimental day shown (Day 11/154/238) was compared to Day −14 (a time point prior to antibiotic treatment and transfer to RR). Numbers in blue and red refer to the number of species that were under- and overrepresented, respectively, for each day specified in comparison to Day −14. For all days, circles in blue and red follow the same color-coding but represent only the species that were differentially expressed on Day 11 in comparison to Day −14. The ordinate is the significance level and the horizontal dashed line represents the cutoff significance level of *p* < 0.05. Abscissa is the log2-fold change in abundance relative to the value on Day −14. Raw data for this panel are tabulated in S1 Data File and S7 Table. (**C**) Volcano plots showing the differences in representation for different species in samples treated with antibiotics analyzed by the linear mixed-effects model. The cutoff was 5,000 total reads per species, yielding the 258 species that are shown here. As compared with S6 Fig, the axes on this figure are expanded to highlight interesting species, especially *Turicibacter* species that were highly impacted by antibiotic treatment. Similarly to S6 Fig, all differences refer to comparisons between the day described in the label above the plot and Day −14, and show the results of a linear mixed-effects model analysis. Ordinate is the significance level and the horizontal dashed line represents the cutoff significance level of *p* < 0.05. Abscissa is the effect size relative to the value on Day −14 and a cutoff significant effect size below 0.5. While all selected species are labeled in the first panel (Day 11), in subsequent panels only the selected species remain labeled that fall within our criteria of significance. Data for this panel are tabulated in S1 Data File. LD, light/dark; RR; red light; WT, wild-type.

To further refine our analyses to changes in the gut microbiome at the species/genera levels, we undertook shotgun sequencing of samples at 4 key time points that we identified as pivotal nodes in the beta-diversity data, namely Days −14, 11, 154, and 238. These time points correspond to initial conditions (Day −14), 11 days after transfer to RR (and antibiotics for treated mice, Day 11), 154 days after transfer to RR (Day 154), and 55 days after transfer back to LD (Day 238, see amber marks in Fig 1A). As expected, there was a large variety of differential responses among the identified species/genera to the changing environmental conditions (S5 Table). The data were analyzed at both genus and species levels by 4 different methods that have been applied to microbiome data and the results were consistent among all tests: (i) non-parametric Mann–Whitney *U* test; (ii) linear regression; (iii) linear mixed-effects model; and (iv) feature volatility [40–47]. We predicted that bacteria that adapt optimally to a temporally cyclic environment—perhaps due to resonance with an internal timekeeper [5]—might be differentially affected by arhythmic conditions as compared with rhythmic conditions. In the untreated (UT) mice, such a hypothetical bacterium would be predicted to be unaffected by the LD/RR/LD transitions in WT(UT) mice (consistent rhythmic behaviors), but show a disruption by RR in Per(UT) mice (arhythmic in RR) that recovers in LD (Fig 4A). Interestingly, that pattern was observed in the species *Corynebacterium casei* and *Corynebacterium stationis* (both species increase in RR in Per(UT) mice; S5 Table). A variant on these predictions was observed in 6 different species of *Lactobacillus* (*L*. *acidophilus*, *L*. *amylovorus*, *L*. *acetotolerans*, *L*. *amylolyticus*, *L*. *helveticus*, and *L*. *crispatus*), namely that the transfer to RR significantly knocked down the abundance of these *Lactobacillus* species (Day 11) and they thereafter recovered partially by Day 154 in RR, and recovered more completely in LD on Day 238 (S5 Table).

For the antibiotic-treated samples, Fig 4B depicts a “volcano plot” of the abundance and significance changes at the *species* level of the WT(T) and Per(T) microbiomes analyzed by Mann–Whitney *U* test followed by a *p*-value adjustment for false discovery rate [40,45,46]. These plots show changes in abundance where each datum is for a different species as identified by the shotgun sequencing. For the WT(T) samples, the abundance of many species is knocked down by the antibiotic treatment by Day 11 (755 species are underrepresented, blue points in Fig 4B), but there is considerable recovery in RR (Day 154) and essentially complete recovery by Day 238, as indicated by the absence of significant differences in the upper right panel of Fig 4B, which depicts the species representations on Day 238 (at which time only 27 species are underrepresented). On the other hand, the pattern for the bacteria residing in Per1/2-dko mice is quite different. Soon after antibiotic treatment, there are many bacterial species that are knocked down by the antibiotics and transfer to RR (363 species become underrepresented) as well as a few that become overrepresented (54 species, red data points in Fig 4B), as shown in the lower left panel (Day 11) of Fig 4B. At Day 154 in RR, some species remain repressed (blue data points), but there are species that were previously (Day 11) not knocked down by the antibiotics that now have become overrepresented (black data points in the upper right quadrant of the middle bottom panel of Fig 4B). This effect appears to be even more obvious on Day 238 (lower right panel of Fig 4B, where 116 are still underrepresented and 199 are overrepresented). Some of these species may be “opportunists” that have taken advantage of the antibiotic knockdown and the RR conditions to enhance their abundance relative to the total composition. Another way of depicting this volcano plot is to color-code on the basis of the final distribution (S5 Fig); this alternative depiction makes even more clear that most of the microbiota that are overrepresented on Day 238 in the Per(T) samples were not significantly knocked down by the antibiotic treatment and grew to an overrepresented abundance progressively during the recovery period. These volcano plots for the Per(T) samples imply that a new steady state microbial composition has been achieved in LD after the RR conditions that differs from the initial LD conditions, which is another way of visualizing the effects shown in Fig 3 for the Per(T) samples.

An alternative statistical test for longitudinal timecourse samples of microbiomes uses a linear mixed-effects model [42–44], for which the results of 258 most abundant species in the WT(T) and Per(T) samples are visualized in the volcano plot of Fig 4C. The same linear mixed-effects model was applied to genera, and the results of that analysis identified 182 most abundant genera that are depicted in S6 Fig. The overall trends between the WT(T) and Per(T) samples are similar among Figs 4B, 4C, and S6, but in addition, several genera of particular interest are highlighted in S6 Fig. As explained above, we predicted that bacteria that adapt optimally to a temporally cyclic environment—perhaps due to resonance with an internal timekeeper [5]—might recover more slowly in arhythmic conditions after perturbation than in rhythmic conditions (Fig 4A). Such a hypothetical bacterium would show a pattern after perturbation by antibiotics of (i) recovery in RR in WT(T) but more slowly in Per(T) samples (rhythmic environment in 1 situation but not the other), and (ii) recovery in the second LD in both WT(T) and Per(T) samples (rhythmic environments in both situations). Interestingly, the species *Bacteroides_sp*.*_PHL_2737*, *Neobacillus mesonae*, *Mucilaginibacter rubeus*, *Porphyromonas gingivalis*, and *Labilibaculum antarcticum* conform to that predicted pattern (S5 Table), but *Klebsiella aerogenes* that has been suggested to harbor a circadian system [9,10] does not conform to that pattern. Most species of *Clostridium* seem to be relatively unaffected by all the conditions. *Prevotella* species are knocked down by antibiotics and generally recover in RR more rapidly in WT(T) than in Per(T), while species of *Alistipes* are knocked down by antibiotics, but mostly recover in RR in both WT(T) and Per(T).

*Akkermansia muciniphila* abundance is enhanced after the antibiotic treatment but eventually recovers to initial conditions in the second LD for WT(T) {but does not recover in Per(T)}, suggesting that it develops an “opportunistic” reaction to the knocked-down genera and grows in relative abundance (S6 Fig and S5 Table). Another particularly robust example of an opportunistic pattern that stood out from the other genera after the “volatility test” [41] was the genus *Turicibacter*. This genus was knocked down by the antibiotics, but recovered to much higher relative abundance in RR for both WT(T) and Per(T) (off the scale for the Per(T)/RR plot in S6 Fig), and was finally suppressed to initial conditions by the second LD in WT(T) but remained dramatically elevated in Per(T) (S5 Table). The mixed model results for species show this same large effect for species of *Turicibacter*, now plotted on an expanded scale in Fig 4C. One interpretation of these data is that *Turicibacter* is not strongly suppressed by the antibiotic treatment but exploits the altered gut environment to expand in an opportunistic pattern. This expansion is ultimately contained in the case of the rhythmic WT(T) mice, but is not brought back to preconditions in the temporally disrupted Per(T) mice; the volatility test indicated that *Turicibacter* is indeed an important contributor to the lack of full recovery of the microbiome of feeding-disrupted Per1/2-dko mice. Interestingly, *Turicibacter* shows the same effects in the untreated samples in RR, suggesting that *Turicibacter* is particularly adept in exploiting temporally disrupted gut environments, even in the absence of an antibiotic perturbation (S5 Table). Finally, while Figs 4 and S6 show the results of our Mann–Whitney *U* and linear mixed-effects model tests, we have also analyzed the data by a linear regression test [47], which yielded equivalent conclusions.

### Functional pathways

As a complementary approach, we analyzed whether these rhythmic versus disrupted daily patterns of environment have consequences for the functional capacities of the intestinal microbial community based on our shotgun metagenomic sequencing reads. We first filtered the shotgun sequencing reads with Bowtie2 to remove the mouse DNA and then we used HUMAnN3 to analyze the microbial metabolic pathway contributions. The data from Days 11, 154, and 238 are compared relative to the baseline (Day −14) data in S7 Fig, which depicts 24 pathways based on strength of signal and that include a variety of metabolic pathways, including carbohydrate, fatty acid, amino acid biosynthesis, and nucleotide metabolism. These 24 pathways were chosen for inclusion in S7 Fig on the basis of greatest significance and for illustrating (i) changes that occurred between T-samples and (ii) UT samples, and (ii) differences between WT(T) and Per(T) samples. As was the case for microbial abundance and diversity, the microbiota of untreated mice (either WT(UT) or Per(UT) mice) had stable pathway abundances that were not significantly affected by transitions between rhythmic and temporally disrupted conditions. This lack of response in metabolic pathways given the stability of richness and diversity of composition is not trivial, as other studies have found changes in metabolic pathways even when OTU diversity was not significantly affected—for example, in the case of disrupted and phase-shifted sleep/wake cycles in humans [48]. For the microbiomes from the treated mice, many pathways were dramatically down- or up-regulated by the brief 5-day antibiotic perturbation as expected (e.g., data for Day 11) [49], and under continuous rhythmic conditions {WT(T)}, almost all of the pathways had recovered by Day 238. However, for the pathways recovering in the temporally disrupted conditions {Per(T)}, many of these pathways had not returned to initial conditions, even by Day 238 (S7 Fig). Not surprisingly, the antibiotics knocked down bacterial species so that fundamental metabolic pathways such as aerobic respiration and gluconeogenesis were reduced, but these recovered completely only under rhythmic conditions but not disrupted conditions. Amino acid and nucleotide biosynthetic pathways exhibited blunted recovery from antibiotics under disrupted conditions. Sulfate reduction and seleno-amino acid biosynthesis were up-regulated by the brief antibiotic treatment (S7 Fig); increases in sulfur metabolism pathways are associated with antibiotic treatments [48,50]. The microbiotal recovery from this up-regulation is similarly protracted in the temporally disrupted environment (S7 Fig). Together, this analysis of functional pathways reflects the common themes observed with the richness and diversity analyses; the temporally disrupted environments (untreated groups) have limited consequences, whereas after perturbation the stably rhythmic environment significantly facilitates recovery.

## Discussion

The gut environment is influenced by host behavior and physiology, which is not constant over the time of day. We report here that the relationship between host circadian rhythms and the gut microbiome on the 24-h timescale is remarkably interactive. On the one hand, in the absence of the antibiotic perturbation, the microbiome is surprisingly stable in the face of disrupted feeding/activity patterns. While there are some differences between the WT(UT) and Per(UT) microbiomes in our study when individual, low-abundance species are considered (S5 and S7 Tables), the results of the alpha-diversity and beta-diversity analyses and the Bray–Curtis/Jaccard metrics indicate that the stability of the microbiome’s composition was not dramatically disturbed by the loss of consistent temporal patterns in the Per(UT) mice in RR in the absence of perturbation by antimicrobials (Figs 2 and S2 and S1 Video; with the exception of *Turicibacter*, considered below). Previous literature has reported varying conclusions regarding the relative stability of the microbiome to disrupted feeding/activity/sleep patterns. For example, studies of human and rodent microbiomes in response to sleep deprivation or shifts of the sleep/wake cycle found—as did we in the present study—practically no overt changes in the richness or composition of the microbiome [48,51]. On the contrary, a different prior study reported jet-lag regimens can affect the microbiotal composition of mice and humans [14]. That investigation also reported that the microbiome of the Per1/2-dko mouse was significantly different from that of WT mice under LD conditions. The results of that study [14] naturally led to a prediction that disruption of the daily temporal environment would provide a selective pressure (or the relaxation of a rhythmic selective pressure) that could result in long-term transformation of microbiotal diversity/composition. In contrast, we observed that—in the absence of any other factor—disruption of regular daily changes in the gut environment did not lead to large changes.

On the other hand, in the face of an antibiotic perturbation, daily rhythmicity of the gut can be a stabilizing influence. When that environment is temporally erratic, perturbations can be more impactful and undermine full recovery. Despite a dramatic alteration of the composition of the gut microbiome by the 5-day treatment with antibiotics, mice that continued to experience approximately 24-h rhythmicity of their behavior in constant conditions (WT mice) exhibited an almost complete recovery of their gut microbiomes to the initial pretreatment diversity. Comparing the groups that were housed in different cages, it is clear that the kinetics of the recovery in constant conditions (RR) can differ (S4 Fig), but for mice that are able to maintain consistent daily rhythmicity (WT mice), the recovery was essentially complete by Days 182 to 196 (S3 Fig and S1 Video). In contrast, microbiomes of mice that cannot maintain consistent cyclic behavior in constant conditions (Per1/2-dko mice, Fig 1B and 1C) never recovered completely after antibiotic exposure (Figs 3, 4B, 4C, and S6). By the time that LD conditions and therefore approximately 24-h rhythmicity was restored to the Per(T) mice, it appears that the altered microbiome composition had reached a new stable state that was not reversible.

We have primarily interpreted these data in terms of a disruption of daily rhythms of feeding and/or activity/sleep (Fig 1) because these seem to be the most obvious daily patterns that would directly affect the gut environment [7]. However, given the circadian control of daily rhythms of gastrointestinal hormones, bile acid levels, body temperature, immune system function, levels of host-delivered antimicrobial peptides, and gut mucosal antibodies [12,52], more investigation is needed to pinpoint which of the multiple clock-controlled factors is most relevant to the inability of Per(T) mice to recover their gut microbiome to its initial pretreatment composition. Moreover, we cannot exclude the possibility that the incomplete recovery from antibiotics in the Per1/2-dko mice is due to a disruption of the gut environment—e.g., destabilization of the intestinal epithelium—by a direct action of PER1/PER2 proteins that is independent of circadian clock phenotypes. If the antibiotic treatment impaired the gut directly, we would expect that damage to manifest most strongly in the first 4 to 6 weeks of the recovery since the gut epithelium recovers within a few weeks after short antibiotic treatments (as is the case for our 5-day treatment). If gut epithelial recovery were significantly different between the WT and Per1/2-dko hosts in a way that directly affected the resident microbiota, we would predict that difference to be revealed early in the recovery period. Therefore, we performed a quadratic regression fit of the recovery kinetics in the first 6 weeks (i.e., to Day 42) using both the Shannon and Chao1 indices. We found that there was no significant difference between the 2 strains, both for the Shannon and the Chao1 alpha indexes (*p* = 0.22 and *p* = 0.59, respectively). This result strongly implies that the intestinal epithelia recoveries of the 2 host strains from the antibiotics are not different.

The discrepancy between the prediction that microbiotal composition/diversity is easily disturbed by temporal disruptions and our UT results might be explained by considering that an additional factor is necessary to enable a large microbiotal change in response to a disrupted temporal environment. In our study, that second factor is the antibiotic perturbation, but in other studies, altered diet could exacerbate the effect of temporal disruption. For example, in a different investigation of the possible influence of jet lag on the gut microbiome, Voigt and colleagues found that weekly phase reversals of the LD cycle did not alter the microbiome in mice that were consistently fed standard chow; however, mice fed a high-fat, high-sugar diet in conjunction with phase shifts in the LD cycle had significantly altered microbiota [17]. In fact, the comparison of those various studies with ours reveals an intriguing correlation: When mice are maintained on a standard diet, disruptions of daily timing of host behavior, feeding, sleep, etc. have little effect upon a stable microbiome [17,48,51]. However, after a perturbation of the stable microbiome by antibiotics (our results) or a high-fat/carbohydrate diet (e.g., [17]), a regular daily pattern of host behavior/feeding is essential for the microbiome to reconstitute its original steady state. By different mechanisms, both antibiotics and high-fat diet can dramatically alter the luminal environment of the gut [53,54]. Therefore, we suggest that the conjunction of temporal disruption in association with other changes in the gut environment synergize to exacerbate changes in the gut microbiome with consequences that may lead to long-term alteration of the microbiotal steady state.

Another interesting conclusion from our results is that neither of the 2 bacteria that have been suggested to display circadian rhythmicity under laboratory conditions outside of the host, namely *Klebsiella aerogenes* and *Bacillus subtilis* [8–10], exhibit a pattern that appears to respond to the disparate temporal patterns of rhythmic in RR (WT) versus disrupted in RR (Per) (S5 and S7 Tables). A simple prediction would have been that a timekeeping bacterium would “resonate” in a rhythmic environment, whereas it might be selected against in an arhythmic environment [5]. However, we previously speculated that expression of circadian rhythmicity in bacteria may be highly dependent upon the specific environmental conditions, and this prediction has been supported by recent data on *Bacillus* [5,8]. A gut bacterium that anticipates when fresh nutrients are entering the digestive tract could have a competitive advantage. A gut bacterium could also benefit from a daily timekeeper when it attempts to survive after excretion in a similar manner to bacteria that are always exposed to the external world. Therefore, bacterial cells that switch between environments might flip into circadian mode in one environment but not another [5]. Consequently, even if *Klebsiella aerogenes* and *Bacillus subtilis* express rhythms in the lab under conditions that might be somewhat akin to the external environment [8–10], we cannot conclude that they will be rhythmic in the gut environment. Another interesting vignette from our study relates to probiotic supplements. These supplements often include the bacteria *Lactobacillus* and *Bifidobacterium*. If our experiments with mice are translatable to humans, however, we infer that probiotic supplements would not change the endpoint recovery from antibiotics because both of these genera recovered by themselves to initial conditions.

The particular case of *Turicibacter* stands out as an opportunist *par excellence*. At both the genera and species levels, *Turicibacter* increases in relative abundance as other bacteria are struggling to recover from the antibiotic perturbation (Figs 4C and S6 and S3 Table). Even in the case of the untreated samples, *Turicibacter* is a stand-out example of bacteria that exploit the altered temporal conditions (free-running in RR for WT, temporal disruption for Per1/2-dko); *Turicibacter* was one of very few species/genera to show such differences in the UT samples (penultimate line in S3 Table). This observation might have broad implications. For example, changes in *Turicibacter* level have been correlated with particular disease states including inflammation and cancer [55]. A fascinating study has reported that *Turicibacter sanguinis* expresses a neurotransmitter sodium symporter-related protein with homology to the mammalian serotonin transporter (aka SERT) [56]. Moreover, the authors found that *T*. *sanguinis* is competent to import serotonin and that increases in the relative abundance of *T*. *sanguinis* modulate host lipid metabolism and abundance [56]. A further implication is that serotonin is a chemical precursor of the circadian-regulated hormone melatonin, and Paulose and colleagues reported that the expression of rhythmicity by *Klebsiella aerogenes* is affected by melatonin, which is known to reach high levels in the gut [10]. Serotonin is strongly implicated in mood disorders, and it is tempting to suggest that the disruptions in the temporal order that lead to enhanced *T*. *sanguinis* as we observed here might explain to some degree the influence of circadian misalignment/disruption on mood and endocrinology [57–61].

In summary, this study reveals that disruptions of daily timing of host behavior, feeding, sleep, etc. have little effect upon a stable microbiome, nor are bacteria that are thought to harbor circadian systems (*Klebsiella* and *Bacillus*) significantly influenced by the rhythmic environmental created by the host’s behavior patterns. However, after a perturbation of the stable microbiome, a regular daily pattern of host behavior/feeding is essential for the microbiome to reconstitute to its original steady state. These exciting results indicate that daily rhythmic patterns dramatically influence microbiome recovery dynamics after perturbation. Significant health-related implications include how the gut of a patient might recover after treatment with high-dose broad spectrum antibiotics; consistent daily eating/behavior patterns make a difference in the recovery from antibiotics. This conclusion is highly relevant to our public health because it is estimated that currently up to 30% of the working population perform shift work and about one-third of adults sleep less than 6 h per night [12,61]. Circadian “misalignment” and disruption are associated with other aspects of modern life, including social jet lag [60], increased nighttime light exposure [58], and irregular daily eating patterns [57,59]. Therefore, rapid recovery of the gut microbiome to pretreatment conditions is another of the many health benefits of harmonic alignment of our internal circadian clock with the environmental day/night cycle [59,61].

## Materials and methods

### Ethics statement

All animal experiments were approved by the Vanderbilt University Institutional Animal Care and Use Committee and were conducted according to that committee’s guidelines under Animal Welfare Assurance Number A3227-01 (IACUC protocol # M1600221) that adhere to those published in “The Guide for the Care and Use of Laboratory Animals (8th Edition).”

### Animals

Adult C57/BL6J background WT or Per1/2 double knockout (“Per1/2-dko” or “Per”) mice were bred to create age-matched cohorts of 3 to 4 weeks of age at the beginning of the experiment. Our Per1/2-dko mice were generated as described by Bae and colleagues [21] and had been introgressed into the C57 background (>8 generations of backcrossing). This mouse strain entrains to LD 12:12 cycles with a consolidated nocturnal activity pattern and exhibits arhythmicity and/or highly disrupted activity patterns in DD [22]. Prior to the study, the parental mice were housed in the same facility and bedding was periodically switched (once every week) between WT and Per1/2-dko mice cages to promote a similar microbiome composition. Bedding exchanges between and among cages of the 2 genotypes were also performed on the experimental mice up until the start of the experiment to homogenize the microbiomes prior to transfer to RR. Mice were provided with a standard lab diet (LabDiet Rodent 5001) ad libitum throughout the study. Because there are sex differences in the gut microbiome [13], we focused upon a single sex to reduce experimental variability, and males were chosen because their circadian wheel-running behavior tends to be more precise because it is not perturbed by the estrous cycle.

### Light/dark/red conditions and fecal collections

A total of 22 WT mice and 28 Per1/2-dko mice were used for the experimental protocol depicted in Fig 1A under both singly and group housed conditions so that their responses could be systematically compared (see S6 Table for housing conditions organized by cage number). Briefly, mice were under an LD 12:12 (12 h light/12 h dark, lights-on at 6:00 AM, cool-white light intensity approximately 400 lux) cycle for 5 weeks (35 days). Mice were then placed under constant dim red light (“RR” approximately 30 lux, see S8 Fig for spectrum of the light source) for 26 weeks (182 days) and then reintroduced to the original LD cycle for the remaining 8 weeks (56 days) of the experiment. Regular chow was provided ad libitum throughout the entire experiment. Fresh fecal samples were collected from all mice weekly during the first LD cycle, twice a week during the first 2 weeks of RR, once every other week for the remainder of the RR treatment, and once a week for the final LD treatment. All fecal collections occurred only between 2:00 PM to 4:00 PM clock time (LDT 8 to 10 for mice under LD conditions). All fecal samples were immediately put on ice after collection and placed in a −80°C freezer for long-term storage. Samples for quality control included: (i) empty tubes that were exposed to the air in the animal care facility during stool collection but which did not receive stool samples; (ii) samples of the chow; and (iii) samples of the water provided to the mice. These quality-control samples were also extracted for DNA and processed along with the stool samples to confirm that bacteria were not introduced to the analysis by artifactual exposure during sample collection.

### Treatment with antibiotics

During the first 5 days of constant dim red light vancomycin (0.5 g/L), streptomycin (2 g/L), and metronidazole (0.75 g/L) were added to the drinking water for the “Treated” mice in both the WT and Per1/2-dko groups (*n* = 11 for WT, *n* = 13 for Per1/2-dko) [32,33]. The antibiotic treatment did not cause diarrhea or any other detectable ill effects, but significantly decreased total bacterial load (S1 Fig) and affected many identifiable genera and species (S3 and S4 Tables). The remaining “Untreated” mice had no antibiotic treatment and experienced no significant effect of the LD/RR/LD transitions on total bacterial load (S1 Fig).

### Locomotor and feeding activity analysis

Locomotor activity and feeding activity were measured in mice in singly housed cages that underwent the same LD/RR/LD regimen as the mice from whom feces were collected (Fig 1 and S1 and S2 Tables). Both locomotor and feeding activity were monitored simultaneously for 1-week intervals during the second LD 12:12 (LD2) and the RR conditions. These 1-week intervals were dispersed throughout the light condition. Activity of WT and PER1/2 double knockout mice with or without antibiotic treatment was recorded (*n* = 4 for each mouse strain). Locomotor activity was measured as wheel-running activity and analyzed with Clocklab software by Actimetrics. Feeding activity was recorded using an infrared motion detecting camera (PYLE PLCM22IR Flush Mount Rear View Camera with 0.5 lux night vision, Pyle Audio, Brooklyn, New York, United States of America) directed at the chow feeder and recorded on a DVR (AMCREST 4 Channel Video Security Recorder, AMDV10814-H5) under infrared illumination. The video recordings were assessed for feeding activity by an independent observer who scored the behavior in accordance with the criteria outlined by Pendergast and co-researchers [62]. Specifically, feeding behavior was defined as a mouse reaching into the chow feeder and visibly eating a pellet with its mouth or holding a chow pellet in its paws and chewing for at least 3 s. Feeding behavior was logged as feeding events in 1-min intervals (multiple feeding events within 1 min were still only considered as 1 feeding event), and the data were then collected in 1-h bins. Therefore, the maximum number of feeding events that could be logged in a 1-h bin was 60. To better compare rhythmic activity between treatment groups, feeding and activity data of individual mouse data were then normalized by dividing by the hour of maximum recorded activity followed by smoothing with a 4-point (= 4 h) moving average and plotted as “counts” in Fig 1C. The feeding activity and locomotor activity were aligned by time using the timestamp on the video recording for comparison. There were no obvious differences in the locomotor activity or feeding behavior between the antibiotic-treated and the untreated groups of mice. Moreover, there were no obvious differences in the weight gain of the mice among any of the groups although this was not quantified.

### DNA extraction, 16S, and shotgun sequencing

DNA from fecal samples was extracted using a DNeasy PowerSoil Kit from Qiagen (Hilden, Germany). A total of 800 DNA samples (stool samples from the same 50 mice collected at 16 key time points) were processed for 16S rDNA sequencing by the Vanderbilt Vantage Genomic Resource Core. The 16S Amplicon sequencing was performed with Illumina MiSeq (PE 250) on V4 regions [63]. Approximately 200 DNA samples (from the 800 total) at 4 nodal time points (Days −14, 11, 154, and 238) were sent for shotgun sequencing by the Vanderbilt Vantage Genomic Resource Core with the Illumina NovaSeq 6000 (PE150) up to 10 M-reads coverage for each sample. Except as indicated otherwise, for data analyses, we used 5,000 reads per taxa as the cutoff for both 16S amplicon sequencing data (5,000 total reads/sample) and the shotgun sequencing data (5,000 total reads/species or genus) after summing up the total reads per taxa for all 200 samples.

### Bioinformatics and functional pathway analyses

QIIME2 was used for analysis of 16s rRNA sequencing [64] (QIIME2 was run online from https://cgc.sbgenomics.com/ with the embedded GreenGenes 16S database). Kraken2 was used for Metagenomic shotgun sequencing [65]. The shotgun sequencing reads were first screened against the mouse reference genome (GRCm38) with Bowtie2 to remove the host DNA [66]. Then, HUMAnN3 was applied to analyze the microbial metabolic pathway contributions [67]. Kraken2, Bowtie2, and HUMAnN3 analyses were all performed with the use of the Advance Computing Center for Research and Education (ACCRE) at Vanderbilt University.

### Statistical analyses

Period analyses were performed with the Lomb–Scargle periodogram in Clocklab (Actimetrics, Illinois, USA) and R. A Wilcoxon rank sum test was performed on the power values of WT and Per1/2-dko mice to determine significant changes in power of the period for each condition. The OTU tables obtained from QIIME2 were uploaded to MicrobiomeAnalyst (https://www.microbiomeanalyst.ca/MicrobiomeAnalyst/home.xhtml) for statistics [29]. The raw sequencing reads for the 800 samples span from 950 to 47,573 reads. Samples with sequencing depths below 5,000 total reads were excluded from data analyses. For any feature retained, at least 20% of the values contained a minimal of 4 counts. Data were rarefied and scaled through total sum scaling (TSS). Shannon and Chao1 indices were applied to calculate alpha-diversities followed by Kruskal–Wallis tests for significance [28–30]. In order to identify whether the early stages of recovery after antibiotic treatment were different between WT and Per1/2-dko mice, we selected the alpha index data in which we observe the fastest rate of antibiotic recovery (Days 7 to 42) and fit quadratic regressions. This was done using the “stats” R package [68]. After this, we compared the coefficients obtained for the WT and Per1/2-dko regressions [69] in order to obtain the z-score between the 2 means. The *p*-values for the WT and Per1/2-dko regressions were then calculated through this z-score, and we found that there was no significant difference between the 2 strains, both for the Shannon and the Chao1 alpha indexes (*p* = 0.22 and *p* = 0.59, respectively). Bray–Curtis and Jaccard indices were used for beta-diversity calculations [29,31,36] followed by PERMANOVA and PERMDISP tests for significance [34,35]. PCoA was performed on all the samples together, but plotted separately for samples at 5 representative time points in Figs 2 and 3, and then for all samples as a gif video file in S1 Video and as graphs in S2 and S3 Figs. The “feature volatility” analysis with the shotgun data was applied with the q2-longitudinal plugin of QIIME2. The machine learning regressor (random forest by default) was used to learn the data structures and then to identify the important features [41]. For shotgun sequencing data, volcano plots at both genera and species levels were generated by 3 different statistical methods that are commonly used to analyze microbiome data [45] and the results were consistent among all 3 tests: (i) Mann–Whitney *U* test followed by a *p*-value adjustment for false discovery rate ([40,45,68], used by ref. [46]); (ii) a linear regression model ([70] used by ref. [47]); and (iii) a linear mixed-effects model (used by refs. [42–44]). In addition to the volcano plots, we used the data analyzed by the Mann–Whitney U test to identify species that conform to the predicted patterns of abundance changes described in Fig 4A. In order to avoid undefined values when calculating log2-fold changes, any zeroes that would end up in the numerator or denominator were changed to be effectively zero (specifically, to 1e-10, a value one order of magnitude smaller than the smallest value we obtained for our relative abundances). The data used for this analysis can be found in S5 Table, where we highlight log2-fold changes that are significant, so that patterns can be more readily identified. For the linear mixed-effects modeling of functional pathways, the samples (mice) were considered as the random effects, whereas the time points and cages were considered to be the fixed effects. The pathway relative abundances of mice on Days 11, 154, and 238 were compared with their values on Day −14 (reference time point) after a natural logarithm transformation and plotted as a heatmap in S7 Fig.

### Ethical approval and consent to participate

No human experiments, tissues, or data were included in this study.

All animal experiments were approved by the Vanderbilt University Institutional Animal Care and Use Committee and were conducted according to that committee’s guidelines under Animal Welfare Assurance Number A3227-01 (IACUC protocol # M1600221) that adhere to those published in “The Guide for the Care and Use of Laboratory Animals (8th Edition).”

## Supporting information

S1 Fig Bacterial Load plotted as (amount of bacterial DNA)÷(stool weight). For panels A–D, the data are plotted as the median, with the box representing the interquartile range (first and third quartile/25th–75th percentiles), while the whiskers indicate variability outside the interquartile range, and represent minimum and maximum as defined by Q1 (quartile 1) - 1.5*IQR (interquartile range) and Q3 + 1.5*IQR. Data for this figure are tabulated in S1 Data File. A. WT(T) samples. B. Per(T) samples. C. WT(UT) samples. D. Per(UT) samples. E. Normalized bacterial load for the WT treated samples {WT(T)}. Because there was a gradual decrease in the level of bacterial load in the untreated samples (panel C) that might be attributed to aging of the mice, normalization of the treated samples was performed by dividing the mean values of the bacterial load in panel A by the mean values of the bacterial load in panel C and the result is plotted in panel E. F. As in panel E, except for the Per1/2-dko samples {Per(T)} using the mean values from panel B divided by the mean values from panel D.
(PDF)

S2 Fig Beta-diversity analyses for WT(UT) and Per(UT) samples, showing all 16S-analyzed time points. The day of stool collection is labeled along the top. Blue ellipses indicate collections and points that were performed during LD conditions, while red ellipses and points indicate collections performed in RR. The axes are Principal Component 1 (PCoA1, abscissa) and Principal Component 2 (PCoA2, ordinate). The pink squares show the centroid of the initial ellipse (Day −14) to allow for easier assessment of how each time point compares to initial conditions. Data for this figure are tabulated in S1 Data File.
(PDF)

S3 Fig Beta-diversity analyses for WT(T) and Per(T) samples, showing all of 16S-analyzed time points. The day of stool collection is labeled along the top. Blue ellipses indicate collections and points that were performed during LD conditions, while red ellipses and points indicate collections performed in RR. The axes are Principal Component 1 (PCoA1, abscissa) and Principal Component 2 (PCoA2, ordinate). The pink squares show the centroid of the initial ellipse (Day −14) to allow for easier assessment of how each time point compares to initial conditions and whether recovery from antibiotics + RR perturbation has occurred. Data for this figure are tabulated in S1 Data File.
(PDF)

S4 Fig Bimodality in the kinetics of recovery of beta-diversity from antibiotics in the WT(T) and Per1/2-dko samples. **(A)** WT(T) samples: 11 mice were split among 5 cages: 3 mice were singly housed in Cages 1, 2, and 5, whereas Cages 3 and 4 contained 4 group-housed mice each. **(B)** Per(T) samples: 13 mice were split into 4 cages: 1 mouse was singly housed in Cage 1, whereas Cages 2, 3, and 4 group-housed 4 mice each. Dots with the same color and shape indicate individual mice from the same cage. Representative time points are: Day 0 (the day of transfer to RR and onset of antibiotic treatment), 14 days in RR also 9 days after removing antibiotics; 42 days in RR, 154 days in RR, and 182 days (the last day) in RR before transferring them back to LD. The colors of the ellipses indicate whether the samples were taken in LD (blue ellipse) or in RR (red ellipses). Housing conditions for all mice are tabulated in S6 Table; data for this figure are tabulated in S1 Data File.
(PDF)

S5 Fig Volcano plots depicting the changes in representation for the 5,421 species identified in our WT and Per1/2-dko individuals that were treated with antibiotics; alternative color-coding to that of Fig 4B. For this analysis, all species were considered, even those with very low abundance. The q-value reflects the results of our Mann–Whitney U test followed by a *p*-value adjustment for false discovery rate [40,45,46] in which each experimental day shown (Day 11/154/238) was compared to Day −14 (a time point prior to antibiotic treatment and transfer to RR). Blue and red dots indicate, respectively, species that were under- and overrepresented on Day 238 in comparison with Day −14, thus highlighting how the species that were differentially represented at the end of the experiment behaved throughout the timecourse. The ordinate is the significance level and the horizontal dashed line represents the cutoff significance level of *p* < 0.05. Abscissa is the log2-fold change in abundance relative to the value on Day −14. Raw data for this figure are tabulated in S6 Table.
(PDF)

S6 Fig Volcano plots showing the differences in representation for different genera in WT and Per1/2-dko mice based on analysis by a linear mixed-effects model [42–44] with a cutoff of 5,000 total reads per genus, yielding the 182 genera shown here. The top 3 panels are for the WT(T) samples, and the bottom 3 panels are for the Per(T) samples. All differences refer to comparisons between the day described in the label above the plot and Day −14 (before antibiotic treatment and onset of RR). Blue and red circles represent genera that were under- (blue) or over- (red) represented on Days 11/154/238 in comparison to Day −14. For WT(T), the underrepresented genera decline from 31 on Day 11 to 23 on Day 154 and to only 1 genus by Day 238, whereas the overrepresented genera decline from 39 (Day 11), to 12 (Day 154), and to 0 on Day 238. For Per(T), the underrepresented genera are 45 (Day 11), 19 (Day 154), and 15 on Day 238, while the overrepresented genera are 72 (Day 11), 22 (Day 154), and 29 on Day 238. Ordinate is the significance level and the horizontal dashed line represents the cutoff significance level of *p* < 0.05. Abscissa is the effect size relative to the value on Day −14 and a cutoff significant effect size below 0.5. While all selected genera are labeled in the first panel (Day 11), in subsequent panels, only the selected genera remain labeled that fall within our criteria of significance. Fig 4C replots these same data with expanded scales to show species. Raw data for this figure are tabulated in S8 Table.
(PDF)

S7 Fig Heatmap representation of metabolic pathways in response to altered host behavior and/or antibiotic treatment. Representative metabolic pathways are identified along the right side of the figure, and panels are from left to right: WT(T), Per1/2-dko(T), WT(UT), and Per1/2-dko(UT). The data from Days 11, 154, and 238 are depicted relative to the baseline (Day −14) data as blue rectangles for decreased values relative to Day −14 (darker blue means a larger decrease, see scale to the left) and as red rectangles for increased values relative to Day −14 (darker red means a larger decrease, see scale to the left). White rectangles mean no change for that time point relative to Day −14. Data for this figure are tabulated in S1 Data File.
(PDF)

S8 Fig Spectrum of the red light source used in the RR experiments. Light intensity in the light tight box was approximately 30 lux.
(PDF)

S1 Table Lomb–Scargle periodogram analyses of WT and Per1/2-dko individual mouse locomotor activity assayed by wheel-running behavior as in Fig 1B. The time frame for analyzing period were as follows: first 12:12 LD cycle (LD1): approximately 15 days, RR: approximately 80 days, second 12:12 LD cycle (LD2): approximately 80 days, with few exceptions. The increase of power in both genotypes under LD2 compared with LD1 is due to the longer time frame analyzed. Note the dramatic loss of power in the Per1/2-dko mice in RR as compared with the WT mice, indicating practically arhythmic and/or highly disrupted patterns. A Wilcoxon rank sum test found that this loss of power in the Per1/2-dko mice during the RR condition was statistically significant compared to WT (*p* = 0.001). No significant difference was observed between the powers of the 2 genotypes under LD1 (*p* = 1) and LD2 (*p* = 0.8). Data for this figure are tabulated in S1 Data File (Fig 1B tab).
(PDF)

S2 Table Lomb–Scargle periodogram analyses of WT and Per1/2-dko individual mouse locomotor activity and feeding behavior as in Fig 1C. Locomotor activity was measured as wheel-running and feeding activity was video-recorded as described in the Methods. These simultaneous recordings were taken during approximately 7-day periods. Note the dramatically lower power for the Per1/2-dko mice in RR as compared with the WT mice for both behaviors, indicating practically arhythmic and/or highly disrupted patterns. A Wilcoxon rank sum test found a significant change in power during the RR condition between the WT and Per1/2-dko power levels for both locomotor activity (*p* = 0.01) and feeding activity (*p* = 0.01). A significant difference in power between the 2 genotypes was not observed during the LD2 condition for locomotor activity (*p* = 0.8) and feeding activity (*p* = 0.8). Data for this figure are tabulated in S1 Data File (Fig 1C tab).
(PDF)

S3 Table Effect size and *p*-value for genera responding to the antibiotic treatment (Day 11) and at the endpoint (Day 238). Columns are: “effect.size” is the effect size of the bacterial abundance changes based on the linear mixed-effects model, “neg.log10q” is the negative log q-value (q-value is the adjusted *p*-value), “mouse.strain” indicates either WT or Per1/2-dko {Per(T) in the table}, and “over.or.under” codes “over” (marked in red) to indicate bacteria at the chosen time point recovered (increased) to values beyond (over) the reference values, while “under” (marked in blue) indicates bacteria that did not recover (decreased) to values that were equivalent to the reference values, whereas “ns” means the changes were not significant. For both WT(T) and Per(T), neg.log10q values are sorted in descending order on Day 11.
(PDF)

S4 Table Log2-fold change and *p*-value for species responding to the antibiotic treatment (Day 11) and at the endpoint (Day 238). Columns are: “log2FC” is the log2-fold change of the bacterial abundances, “neg.log10q” is the negative log q-value (q-value is the adjusted *p*-value), “mouse.strain” indicates either WT or Per1/2-dko {Per(T) in the table}, and “over.or.under” where “over” (marked in red) indicates bacteria at the chosen time point that recovered (increased) to values beyond (over) the reference values and “under” (marked in blue) indicates bacteria that did not recover (decreased) to values that were equivalent to the reference values. For both WT(T) and Per(T), neg.log10q values are sorted in descending order on Day 11.
(PDF)

S5 Table Changes in representation for the 5,421 species analyzed. Values within cells show the log2-fold changes observed when we compare the day indicated for each column (i.e., Days 11, 154, and 238) and Day −14 (prior to antibiotic treatment and/or transfer to RR) and were calculated as the ratio between the average relative abundance at the day indicated and Day −14. Cells highlighted in blue and red are those in which there is a significant decrease (blue) or increase (red) in abundance, as determined by a Mann–Whitney U test followed by a *p*-value adjustment for false discovery rate (q-value < 0.05). Cells that are not highlighted are those in which there were no significant changes in abundance for the time point specified.
(PDF)

S6 Table Cage allocation of mice for each group. All groups distribute both groups and single mice among the individual cages.
(PDF)

S7 Table Relative bacterial abundances at the species level (total sequencing reads for each species are divided by total sequencing reads per sample). Each value here is the mean relative abundance of the chosen species from all the biological replicates at a given time point for the WT(T), Per(T), WT(UT), or Per(UT) samples, which were used to derive the volcano plots in Fig 4B.
(PDF)

S8 Table Relative bacterial abundances at the genus level (total sequencing reads for each genus are divided by total sequencing reads per sample). Each value here is the mean relative abundance of the chosen genus from all the biological replicates at a given time point for the WT(T), Per(T), WT(UT), or Per(UT) samples, which were used to derive the volcano plots in S6 Fig. Columns are tabulated as in S7 Table.
(PDF)

S1 Data File Full listing of data as plotted in text and supplemental figures. (XLSX)

S1 Video Beta-diversity characterizations: Responses of gut microbiome to antibiotic treatment and rhythmic vs. non-rhythmic feeding patterns displayed as a GIF animation of all 16 time points. Timescale along the top is the timecourse of sample collection as in Fig 1A, which identifies each time point progressively. The axes are Principal Component 1 (PCoA1, abscissa) and Principal Component 2 (PCoA2, ordinate). Antibiotic-treated samples are the top 2 panels {left top = WT(T), right top = Per(T)}; untreated samples are the bottom panels {left bottom = WT(UT), right bottom = Per(UT)}. Data of each new time point are shown as colored dots/lines, and data of previous time points are illustrated as gray dots/lines. Note that this S1 Video is a movie that is included as a separate supplemental video file. This movie version can be stepped time point by time point as frame by frame.
(MOV)

## Abbreviations

DD: constant darkness
LD: light/dark cycle (LD 12:12 = 12-h light/12-h dark cycle)
LDT: time in the LD cycle, where LDT0 is lights-on
OTU: operational taxonomic unit
Per: Per1/2-dko = mice in which two central circadian clock genes *Period1* and *Period2* were both knocked out
RR: constant dim red light (~30 lux in this study)
TSS: total sum scaling
WT: wild-type mice
