# Supplementary material for: Host circadian behaviors exert only weak selective pressure on the gut microbiome under stable conditions but are critical for recovery from antibiotic treatment
Source: PLoS Biol. 2022 Nov 9;20(11):e3001865. doi: 10.1371/journal.pbio.3001865 (PMC9645659; doi:10.1371/journal.pbio.3001865)
Supplement: S1 Fig — For panels A–D, the data are plotted as the median, with the box representing the interquartile range (first and third quartile/25th–75th percentiles), while the whiskers indicate variability outside the interquartile range, and represent minimum and maximum as defined by Q1 (quartile 1) - 1.5*IQR (interquartile range) and Q3 + 1.5*IQR. Data for this figure are tabulated in S1 Data File. A. WT(T) samples. B. Per(T) samples. C. WT(UT) samples. D. Per(UT) samples. E. Normalized bacterial load for the WT treated samples {WT(T)}. Because there was a gradual decrease in the level of bacterial load in the untreated samples (panel C) that might be attributed to aging of the mice, normalization of the treated samples was performed by dividing the mean values of the bacterial load in panel A by the mean values of the bacterial load in panel C and the result is plotted in panel E. F. As in panel E, except for the Per1/2-dko samples {Per(T)} using the mean values from panel B divided by the mean values from panel D. (PDF) [file pbio.3001865.s001.pdf]

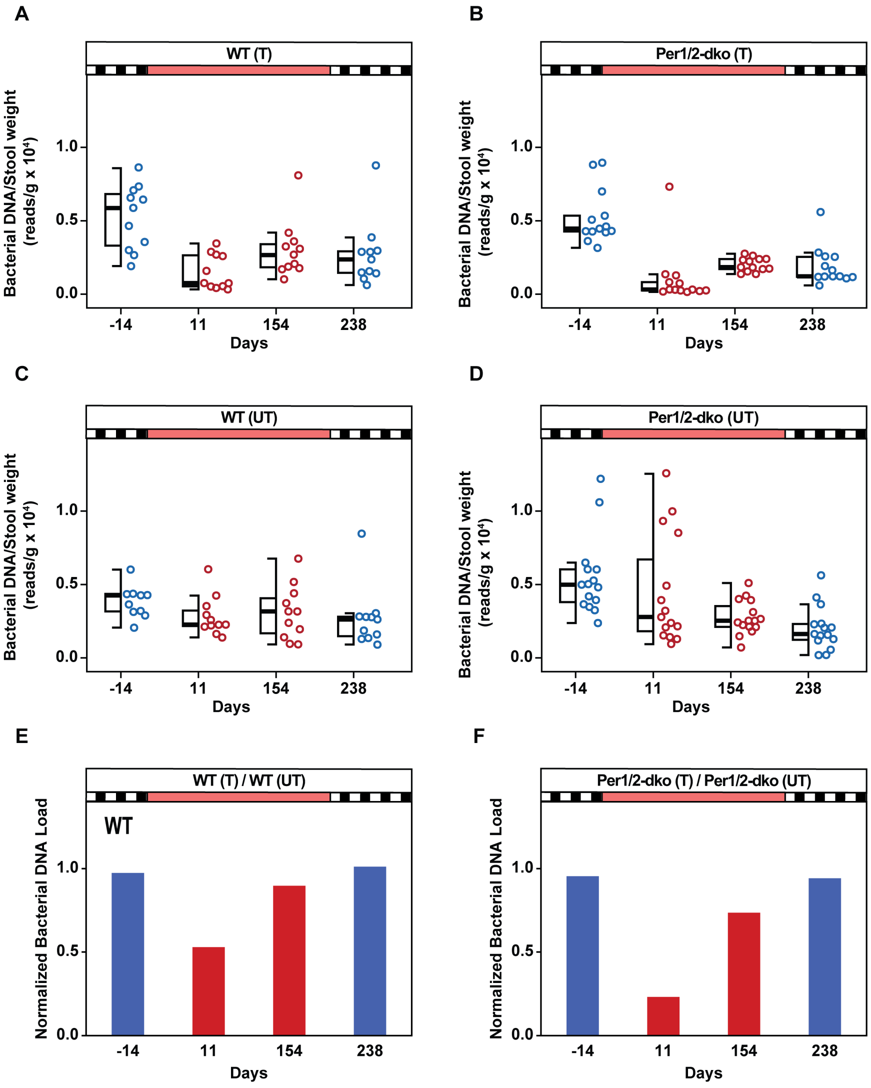


**S1 Fig**. Bacterial Load plotted as (amount of bacterial DNA)÷(stool weight). For panels A-D, the data are plotted as the median, with the box representing the interquartile range (first and third quartile/25th-75th percentiles), while the whiskers indicate variability outside the interquartile range, and represent minimum and maximum as defined by Q1 (quartile 1) - 1.5*IQR (interquartile range) and Q3 + 1.5*IQR. Data for this figure are tabulated in S1 Data File.

A. WT(T) samples
B. Per(T) samples
C. WT(UT) samples
D. Per(UT) samples
E. Normalized bacterial load for the WT treated samples {WT(T)}. Because there was a gradual decrease in the level of bacterial load in the untreated samples (panel C) that might be attributed to aging of the mice, normalization of the treated samples was performed by dividing the mean values of the bacterial load in panel A by the mean values of the bacterial load in panel C and the result is plotted in panel E.
F. As in panel E, except for the Per1/2-dko samples {Per(T)} using the mean values from panel B divided by the mean values from panel D.
