# Supplementary material for: Host circadian behaviors exert only weak selective pressure on the gut microbiome under stable conditions but are critical for recovery from antibiotic treatment
Source: PLoS Biol. 2022 Nov 9;20(11):e3001865. doi: 10.1371/journal.pbio.3001865 (PMC9645659; doi:10.1371/journal.pbio.3001865)
Supplement: S2 Fig — The day of stool collection is labeled along the top. Blue ellipses indicate collections and points that were performed during LD conditions, while red ellipses and points indicate collections performed in RR. The axes are Principal Component 1 (PCoA1, abscissa) and Principal Component 2 (PCoA2, ordinate). The pink squares show the centroid of the initial ellipse (Day −14) to allow for easier assessment of how each time point compares to initial conditions. Data for this figure are tabulated in S1 Data File. (PDF) [file pbio.3001865.s002.pdf]

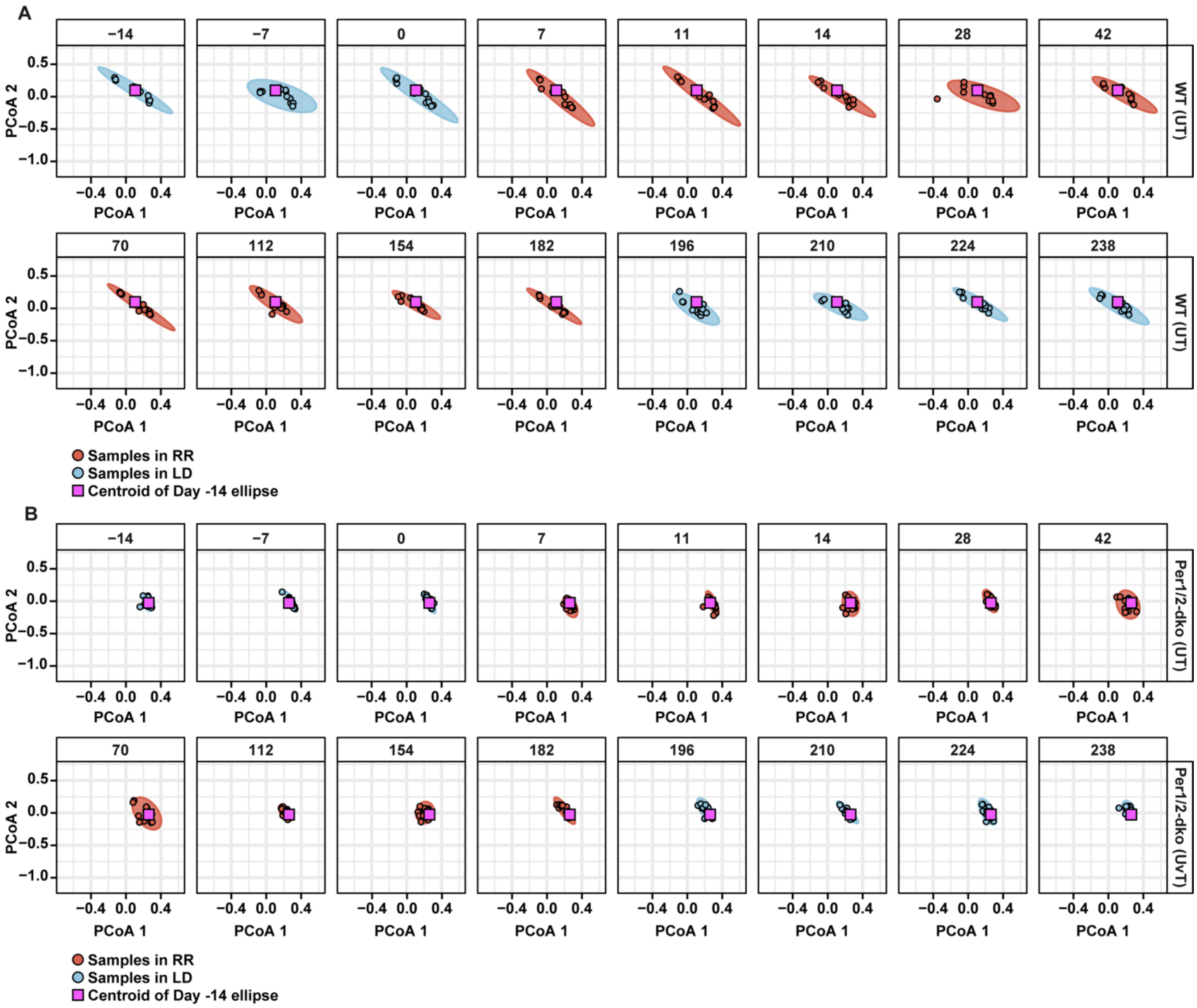

S2 Fig. Beta-Diversity analyses for WT(UT) and Per(UT) samples, showing all 16S-analyzed timepoints. The day of stool collection is labeled along the top. Blue ellipses indicate collections and points that were performed during LD conditions, while red ellipses and points indicate collections performed in RR. The axes are Principal Component 1 (PCoA1, abscissa) and Principal Component 2 (PCoA2, ordinate). The pink squares show the centroid of the initial ellipse (day -14) to allow for easier assessment of how each timepoint compares to initial conditions. Data for this figure are tabulated in S1 Data File.
