# Supplementary material for: Host circadian behaviors exert only weak selective pressure on the gut microbiome under stable conditions but are critical for recovery from antibiotic treatment
Source: PLoS Biol. 2022 Nov 9;20(11):e3001865. doi: 10.1371/journal.pbio.3001865 (PMC9645659; doi:10.1371/journal.pbio.3001865)
Supplement: S4 Fig — (A) WT(T) samples: 11 mice were split among 5 cages: 3 mice were singly housed in Cages 1, 2, and 5, whereas Cages 3 and 4 contained 4 group-housed mice each. (B) Per(T) samples: 13 mice were split into 4 cages: 1 mouse was singly housed in Cage 1, whereas Cages 2, 3, and 4 group-housed 4 mice each. Dots with the same color and shape indicate individual mice from the same cage. Representative time points are: Day 0 (the day of transfer to RR and onset of antibiotic treatment), 14 days in RR also 9 days after removing antibiotics; 42 days in RR, 154 days in RR, and 182 days (the last day) in RR before transferring them back to LD. The colors of the ellipses indicate whether the samples were taken in LD (blue ellipse) or in RR (red ellipses). Housing conditions for all mice are tabulated in S6 Table; data for this figure are tabulated in S1 Data File. (PDF) [file pbio.3001865.s004.pdf]

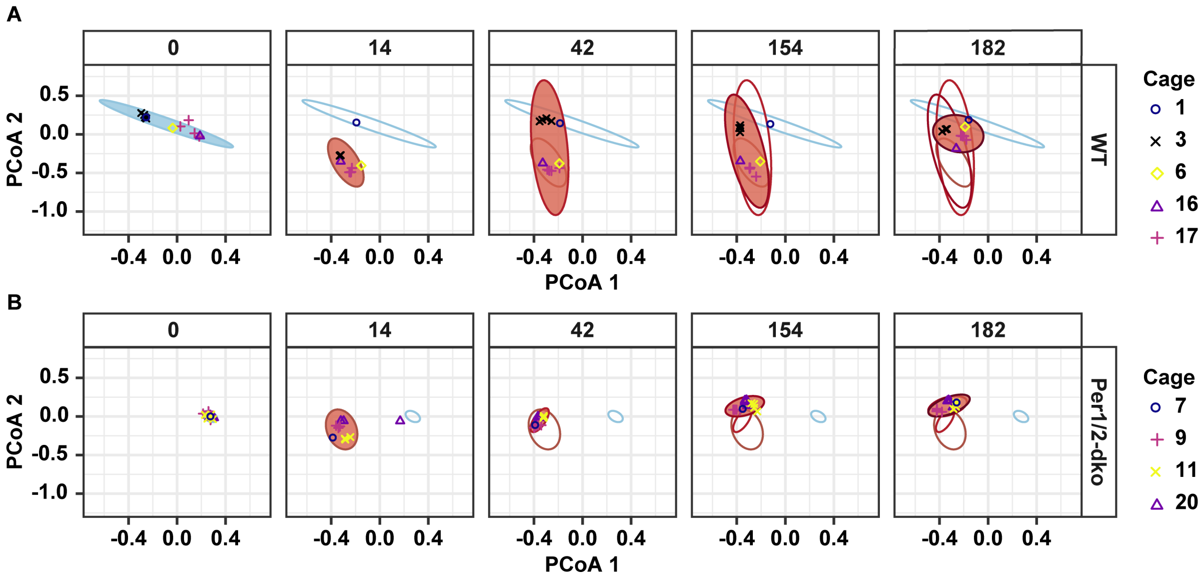

S4 Fig. Bimodality in the kinetics of recovery of Beta Diversity from antibiotics in the WT(T) and Per1/2-dko samples. A. WT(T) samples: 11 mice were split among 5 cages: 3 mice were singly housed in Cages 1, 2 and 5, whereas Cages 3 and 4 contained 4 group-housed mice each. **B.** Per(T) samples: 13 mice were split into 4 cages: 1 mouse was singly housed in Cage 1, whereas Cages 2, 3 and 4 group-housed 4 mice each. Dots with the same color and shape indicate individual mice from the same cage. Representative time points are: Day 0 (the day of transfer to RR and onset of antibiotic treatment), 14 days in RR also 9 days after removing antibiotics; 42 days in RR, 154 days in RR and 182 days (the last day) in RR before transferring them back to LD. The colors of the ellipses indicate whether the samples were taken in LD (blue ellipse) or in RR (red ellipses). Housing conditions for all mice are tabulated in S6 Table S6, data for this figure are tabulated in S1 Data File.
