# Supplementary material for: Host circadian behaviors exert only weak selective pressure on the gut microbiome under stable conditions but are critical for recovery from antibiotic treatment
Source: PLoS Biol. 2022 Nov 9;20(11):e3001865. doi: 10.1371/journal.pbio.3001865 (PMC9645659; doi:10.1371/journal.pbio.3001865)
Supplement: S5 Fig — For this analysis, all species were considered, even those with very low abundance. The q-value reflects the results of our Mann–Whitney U test followed by a p-value adjustment for false discovery rate [40,45,46] in which each experimental day shown (Day 11/154/238) was compared to Day −14 (a time point prior to antibiotic treatment and transfer to RR). Blue and red dots indicate, respectively, species that were under- and overrepresented on Day 238 in comparison with Day −14, thus highlighting how the species that were differentially represented at the end of the experiment behaved throughout the timecourse. The ordinate is the significance level and the horizontal dashed line represents the cutoff significance level of p < 0.05. Abscissa is the log2-fold change in abundance relative to the value on Day −14. Raw data for this figure are tabulated in S6 Table. (PDF) [file pbio.3001865.s005.pdf]

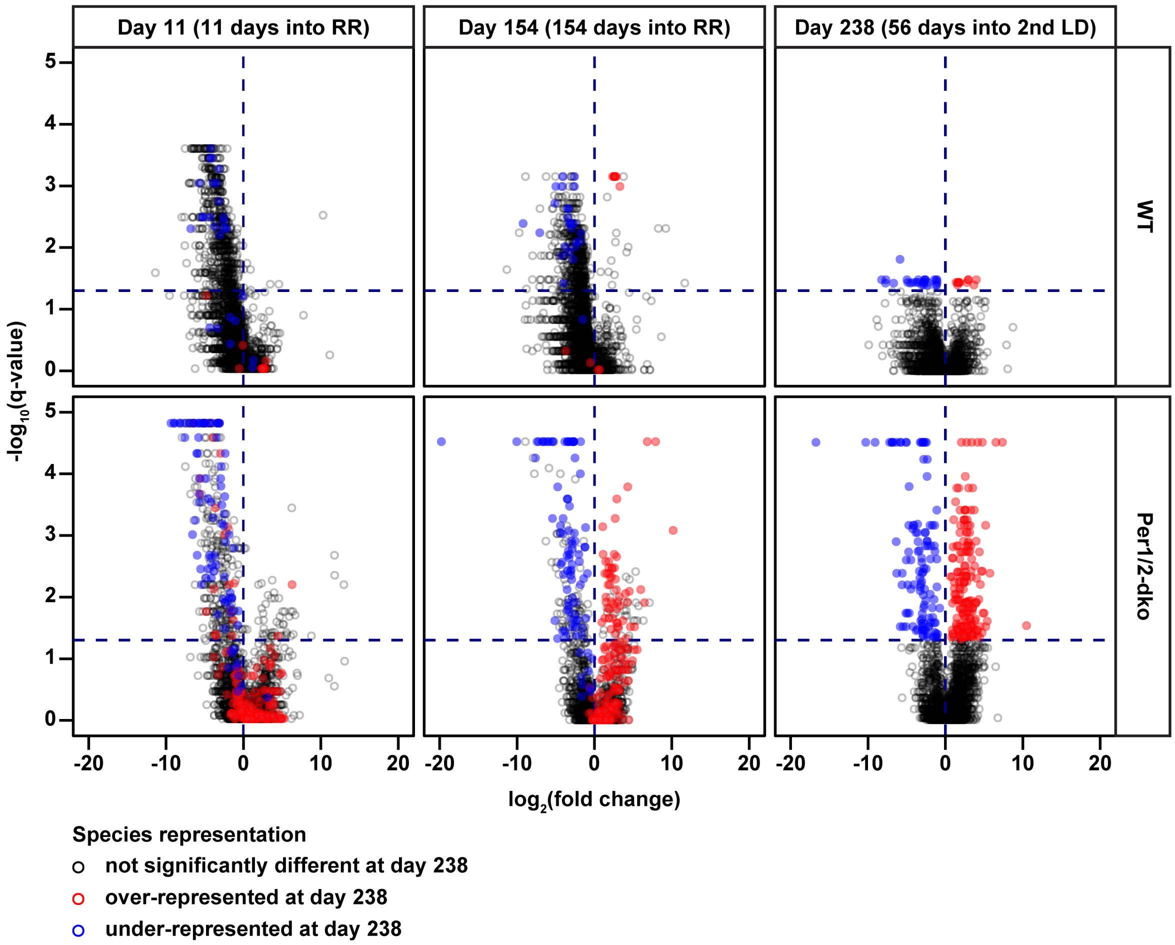

S5 Fig. Volcano plots depicting the changes in representation for the 5421 species identified in our WT and Per1/2-dko individuals that were treated with antibiotics; alternative color-coding to that of Fig 4B. For this analysis, all species were considered, even those with very low abundance. The q-value reflects the results of our Mann-Whitney U test followed by a p-value adjustment for false discovery rate [40,45,46] in which each experimental day shown (Day 11/154/238) was compared to Day -14 (a timepoint prior to antibiotic treatment and transfer to RR). Blue and red dots indicate, respectively, species that were under and over-represented on day 238 in comparison with day -14, thus highlighting how the species that were differentially represented at the end of the experiment behaved throughout the timecourse. The ordinate is the significance level and the horizontal dashed line represents the cutoff significance level of $p<0.05$. Abscissa is the log2-fold change in abundance relative to the value on Day -14. Raw data for this figure are tabulated in S6 Table.
