# Supplementary material for: Host circadian behaviors exert only weak selective pressure on the gut microbiome under stable conditions but are critical for recovery from antibiotic treatment
Source: PLoS Biol. 2022 Nov 9;20(11):e3001865. doi: 10.1371/journal.pbio.3001865 (PMC9645659; doi:10.1371/journal.pbio.3001865)
Supplement: S6 Fig — The top 3 panels are for the WT(T) samples, and the bottom 3 panels are for the Per(T) samples. All differences refer to comparisons between the day described in the label above the plot and Day −14 (before antibiotic treatment and onset of RR). Blue and red circles represent genera that were under- (blue) or over- (red) represented on Days 11/154/238 in comparison to Day −14. For WT(T), the underrepresented genera decline from 31 on Day 11 to 23 on Day 154 and to only 1 genus by Day 238, whereas the overrepresented genera decline from 39 (Day 11), to 12 (Day 154), and to 0 on Day 238. For Per(T), the underrepresented genera are 45 (Day 11), 19 (Day 154), and 15 on Day 238, while the overrepresented genera are 72 (Day 11), 22 (Day 154), and 29 on Day 238. Ordinate is the significance level and the horizontal dashed line represents the cutoff significance level of p < 0.05. Abscissa is the effect size relative to the value on Day −14 and a cutoff significant effect size below 0.5. While all selected genera are labeled in the first panel (Day 11), in subsequent panels, only the selected genera remain labeled that fall within our criteria of significance. Fig 4C replots these same data with expanded scales to show species. Raw data for this figure are tabulated in S8 Table. (PDF) [file pbio.3001865.s006.pdf]

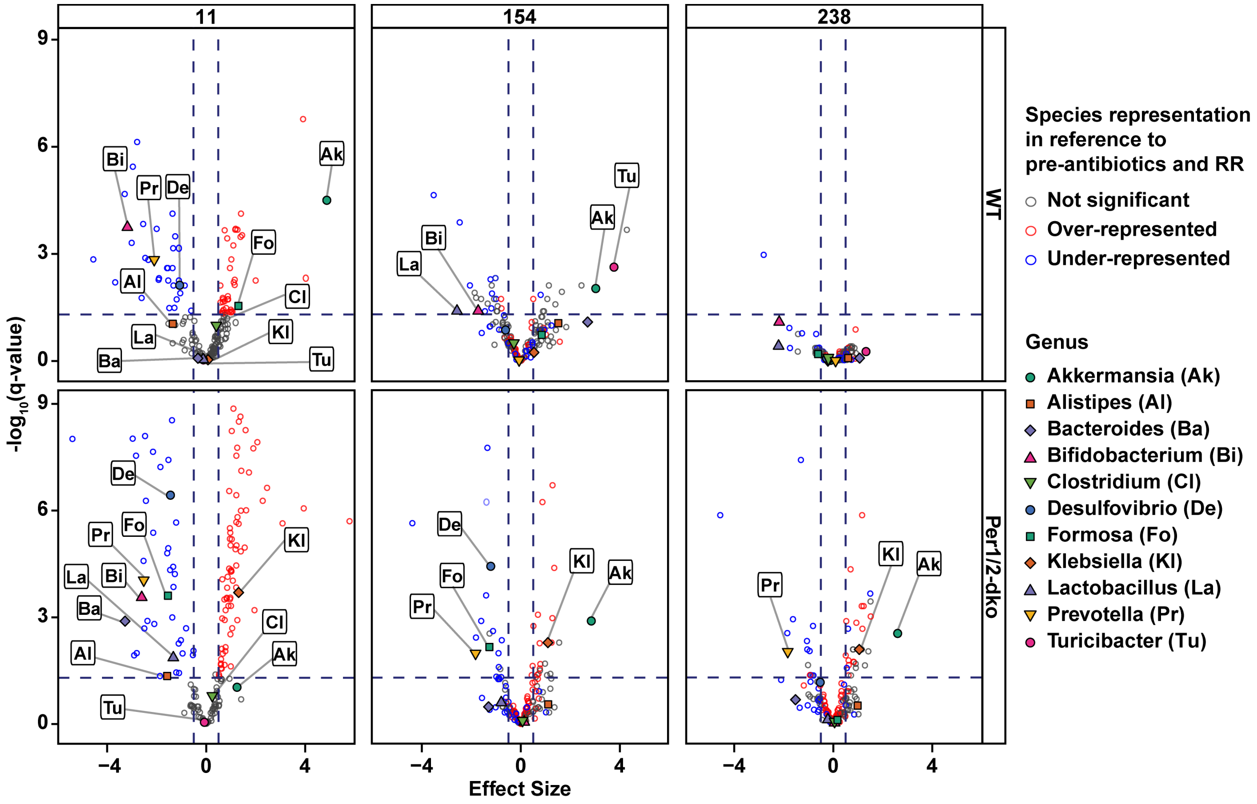

S6 Fig. Volcano plots showing the differences in representation for different genera in WT and Per1/2-dko mice based on analysis by a linear mixed-effects model [42-44] with a cutoff of 5000 total reads per genus, yielding the 182 genera shown here. The top three panels are for the WT(T) samples, and the bottom three panels are for the Per(T) samples. All differences refer to comparisons between the day described in the label above the plot and Day -14 (before antibiotic treatment and onset of RR). Blue and red circles represent genera that were under- (blue) or over- (red) represented on Days 11/154/238 in comparison to Day -14. For WT(T), the under-represented genera decline from 31 on Day 11 to 23 on Day 154 and to only 1 genus by Day 238, whereas the over-represented genera decline from 39 (Day 11), to 12 (Day 154), and to 0 on Day 238. For Per(T), the under-represented genera are 45 (Day 11), 19 (Day 154), and 15 on Day 238, while the over-represented genera are 72(Day 11), 22 (Day 154), and 29 on Day 238. Ordinate is the significance level and the horizontal dashed line represents the cutoff significance level of p<0.05. Abscissa is the effect size relative to the value on Day -14 and a cutoff significant effect size below 0.5. While all selected genera are labeled in the first panel (Day 11), in subsequent panels only the selected genera remain labeled that fall within our criteria of significance. Fig 4C replots these same data with expanded scales to show species. Raw data for this figure are tabulated in S8 Table.
