# Supplementary material for: Host circadian behaviors exert only weak selective pressure on the gut microbiome under stable conditions but are critical for recovery from antibiotic treatment
Source: PLoS Biol. 2022 Nov 9;20(11):e3001865. doi: 10.1371/journal.pbio.3001865 (PMC9645659; doi:10.1371/journal.pbio.3001865)
Supplement: S7 Fig — Representative metabolic pathways are identified along the right side of the figure, and panels are from left to right: WT(T), Per1/2-dko(T), WT(UT), and Per1/2-dko(UT). The data from Days 11, 154, and 238 are depicted relative to the baseline (Day −14) data as blue rectangles for decreased values relative to Day −14 (darker blue means a larger decrease, see scale to the left) and as red rectangles for increased values relative to Day −14 (darker red means a larger decrease, see scale to the left). White rectangles mean no change for that time point relative to Day −14. Data for this figure are tabulated in S1 Data File. (PDF) [file pbio.3001865.s007.pdf]

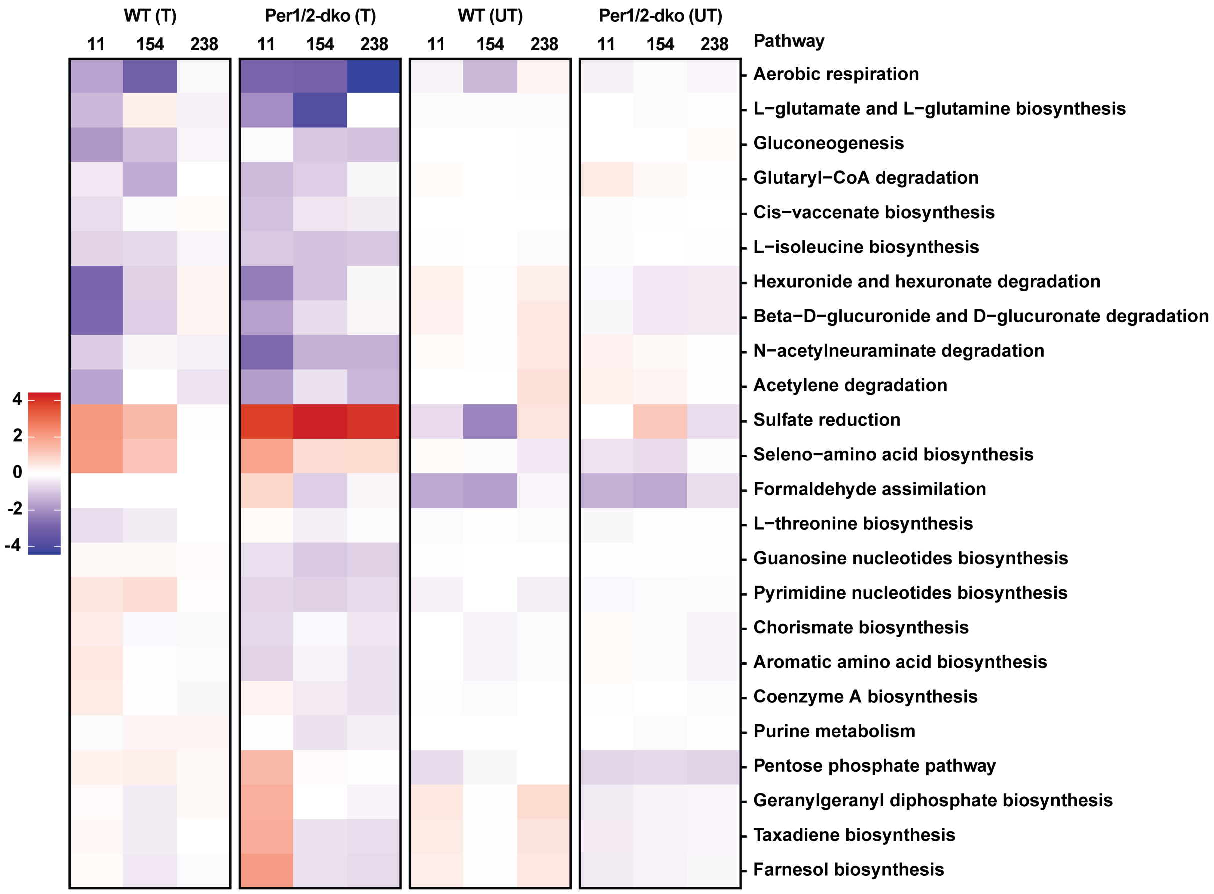

S7 Fig. Heatmap representation of metabolic pathways in response to altered host behavior and/or antibiotic treatment. Representative metabolic pathways are identified along the right side of the figure, and panels are from left to right: WT(T), Per1/2-dko(T), WT(UT), and Per1/2-dko(UT). The data from Days 11, 154, and 238 are depicted relative to the baseline (Day -14) data as blue rectangles for decreased values relative to Day -14 (darker blue means a larger decrease, see scale to the left), and as red rectangles for increased values relative to Day -14 (darker red means a larger decrease, see scale to the left). White rectangles mean no change for that timepoint relative to Day -14. Data for this figure are tabulated in S1 Data File.
