# Supplementary material for: Host circadian behaviors exert only weak selective pressure on the gut microbiome under stable conditions but are critical for recovery from antibiotic treatment
Source: PLoS Biol. 2022 Nov 9;20(11):e3001865. doi: 10.1371/journal.pbio.3001865 (PMC9645659; doi:10.1371/journal.pbio.3001865)
Supplement: S8 Fig — Light intensity in the light tight box was approximately 30 lux. (PDF) [file pbio.3001865.s008.pdf]

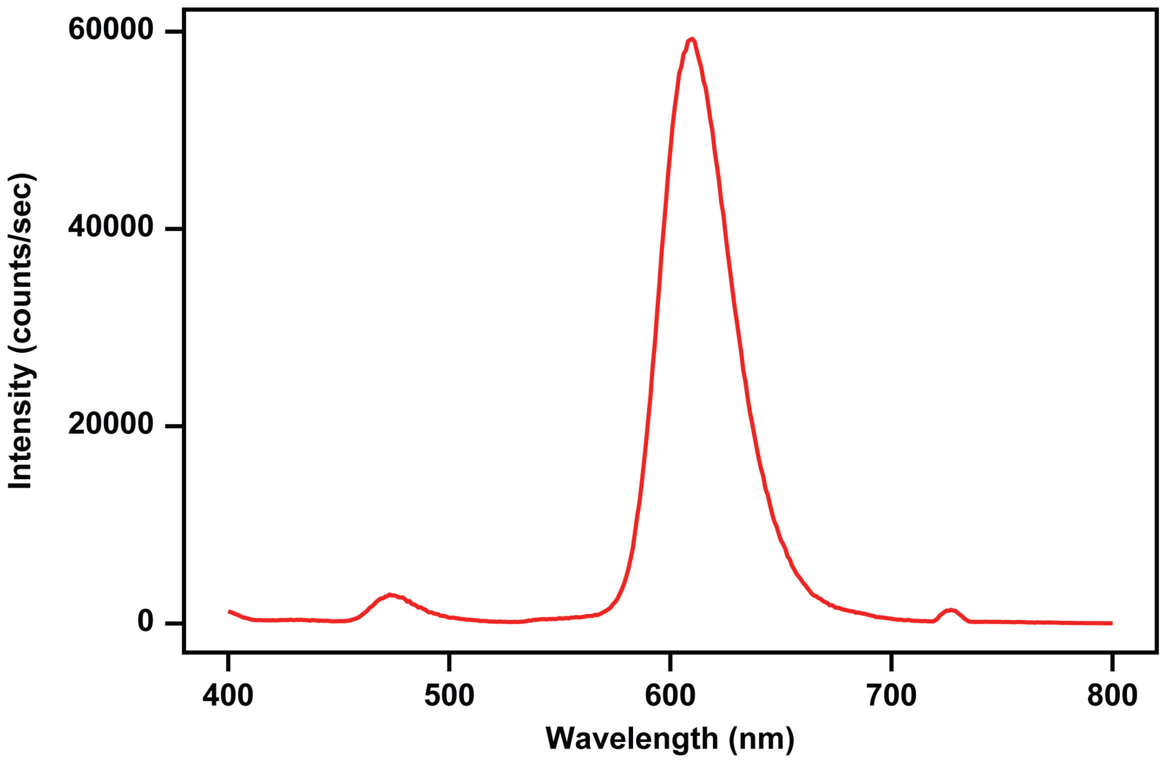

S8 Fig. Spectrum of the red-light source used in the RR experiments. Light intensity in the light-tight box was ~30 lux.
