# Supplementary material for: Host circadian behaviors exert only weak selective pressure on the gut microbiome under stable conditions but are critical for recovery from antibiotic treatment
Source: PLoS Biol. 2022 Nov 9;20(11):e3001865. doi: 10.1371/journal.pbio.3001865 (PMC9645659; doi:10.1371/journal.pbio.3001865)
Supplement: S1 Table — The time frame for analyzing period were as follows: first 12:12 LD cycle (LD1): approximately 15 days, RR: approximately 80 days, second 12:12 LD cycle (LD2): approximately 80 days, with few exceptions. The increase of power in both genotypes under LD2 compared with LD1 is due to the longer time frame analyzed. Note the dramatic loss of power in the Per1/2-dko mice in RR as compared with the WT mice, indicating practically arhythmic and/or highly disrupted patterns. A Wilcoxon rank sum test found that this loss of power in the Per1/2-dko mice during the RR condition was statistically significant compared to WT (p = 0.001). No significant difference was observed between the powers of the 2 genotypes under LD1 (p = 1) and LD2 (p = 0.8). Data for this figure are tabulated in S1 Data File (Fig 1B tab). (PDF) [file pbio.3001865.s009.pdf]

	LD1 (~15 day window)			RR (~80 day window[a])			LD2 (~80 day window[b])		
**Genotype**	***N***	**Period**	**Power**	***N***	**Period**	**Power**	***N***	**Period**	**Power**
WT	6	24.0 ± 0.038	96 ± 26	6	24.5 ± 0.328	279 ± 150	6	24.0 ± 0	259 ± 148
Per1/2-dko	6	24.0 ± 0.042	88 ± 38	6	13.3 ± 2.86	32 ± 32	4	24.0 ± 0	277 ± 128

[a] Except for two Per1/2-dko mice that had a window of ~30 days
[b] Except for one Per1/2-dko mouse that had a window of 53 days

S1 Table. Lomb-Scargle periodogram analyses of WT and Per1/2-dko individual mouse locomotor activity assayed by wheel-running behavior as in Fig 1B. The time frame for analyzing period were as follows: first 12:12 LD cycle (LD1): ~15 days, RR: ~80 days, second 12:12 LD cycle (LD2): ~80 days, with few exceptions. The increase of power in both genotypes under LD2 compared with LD1 is due to the longer time frame analyzed. Note the dramatic loss of power in the Per1/2-dko mice in RR as compared with the WT mice, indicating practically arhythmic and/or highly disrupted patterns. A Wilcoxon rank sum test found that this loss of power in the Per1/2-dko mice during the RR condition was statistically significant compared to WT ($p = 0.001$). No significant difference was observed between the powers of the two genotypes under LD1 ($p = 1$) and LD2 ($p = 0.8$). Data for this table and Fig 1B are tabulated in S1 Data File (Fig 1B tab).
