# Supplementary material for: Host circadian behaviors exert only weak selective pressure on the gut microbiome under stable conditions but are critical for recovery from antibiotic treatment
Source: PLoS Biol. 2022 Nov 9;20(11):e3001865. doi: 10.1371/journal.pbio.3001865 (PMC9645659; doi:10.1371/journal.pbio.3001865)
Supplement: S2 Table — Locomotor activity was measured as wheel-running and feeding activity was video-recorded as described in the Methods. These simultaneous recordings were taken during approximately 7-day periods. Note the dramatically lower power for the Per1/2-dko mice in RR as compared with the WT mice for both behaviors, indicating practically arhythmic and/or highly disrupted patterns. A Wilcoxon rank sum test found a significant change in power during the RR condition between the WT and Per1/2-dko power levels for both locomotor activity (p = 0.01) and feeding activity (p = 0.01). A significant difference in power between the 2 genotypes was not observed during the LD2 condition for locomotor activity (p = 0.8) and feeding activity (p = 0.8). Data for this figure are tabulated in S1 Data File (Fig 1C tab). (PDF) [file pbio.3001865.s010.pdf]

	RR (~7 day window)						LD2 (~7 day window)					
	Locomotor Activity			Feeding Activity			Locomotor Activity			Feeding Activity		
Genotype	*N*	Period	Power	*N*	Period	Power	*N*	Period	Power	*N*	Period	Power
WT	4	24.5 ± 0.302	33.0 ± 18.3	4	24.5 ± 0.321	32 ± 11	4	24.0 ± 0.117	31 ± 17	4	22.8 ± 2.33	12 ± 9
Per1/2-dko	3	18.6 ± 0.737	3.0 ± 0.9	3	19.2 ± 0.716	4 ± 1	4	24.0 ± 0.139	28 ± 9	4	24.3 ± 1.03	14 ± 9

S2 Table. Lomb-Scargle periodogram analyses of WT and Per1/2-dko individual mouse locomotor activity and feeding behavior as in Fig 1C. Locomotor activity was measured as wheel-running and feeding activity was video-recorded as described in the Methods. These simultaneous recordings were taken during ~seven-day periods. Note the dramatically lower power for the Per1/2-dko mice in RR as compared with the WT mice for both behaviors, indicating practically arhythmic and/or highly disrupted patterns. A Wilcoxon rank sum test found a significant change in power during the RR condition between the WT and Per1/2-dko power levels for both locomotor activity ($p = 0.01$) and feeding activity ($p = 0.01$). A significant difference in power between the two genotypes was not observed during the LD2 condition for locomotor activity ($p = 0.8$) and feeding activity ($p = 0.8$). Data for this table and Fig 1C are tabulated in S1 Data File (Fig 1C tab).
