# Supplementary material for: Host circadian behaviors exert only weak selective pressure on the gut microbiome under stable conditions but are critical for recovery from antibiotic treatment
Source: PLoS Biol. 2022 Nov 9;20(11):e3001865. doi: 10.1371/journal.pbio.3001865 (PMC9645659; doi:10.1371/journal.pbio.3001865)
Supplement: S3 Table — Columns are: “effect.size” is the effect size of the bacterial abundance changes based on the linear mixed-effects model, “neg.log10q” is the negative log q-value (q-value is the adjusted p-value), “mouse.strain” indicates either WT or Per1/2-dko {Per(T) in the table}, and “over.or.under” codes “over” (marked in red) to indicate bacteria at the chosen time point recovered (increased) to values beyond (over) the reference values, while “under” (marked in blue) indicates bacteria that did not recover (decreased) to values that were equivalent to the reference values, whereas “ns” means the changes were not significant. For both WT(T) and Per(T), neg.log10q values are sorted in descending order on Day 11. (PDF) [file pbio.3001865.s011.pdf]

S3 Table. Effect Size and p-value for genera responding to the antibiotic treatment (Day 11) and at the endpoint (Day 238). Columns are: “effect.size” is the effect size of the bacterial abundance changes based on the linear mixed-effects model, “neg.log10q” is the negative log q-value (q-value is the adjusted p-value), “mouse.strain” indicates either WT or Per1/2-dko {Per(T) in the table}, and “over.or.under” codes "over" (marked in red) to indicate bacteria at the chosen time point recovered (increased) to values beyond (over) the reference values, while "under" (marked in blue) indicates bacteria that did not recover (decreased) to values that were equivalent to the reference values, whereas "ns" means the changes were not significant. For both WT(T) and Per(T), neg.log10q values are sorted in descending order on Day 11.

S3 Table

Effect size and p-value for genera responding to the antibiotic treatment(Day11) and at the end point(Day238) compared to the reference(Day-14). Columns are: "effect.size" is the effect size of the bacterial abundance changes based on linear mixed effects model, "neg.log10q" is negative log10 q-value (q-value is the adjusted p-value), "mouse.strain" indicates either WT(T) or Per(T), "over.or.under" codes "over"(marked in red) to mean bacteria at the chosen time point recovered (increased) to values beyond (over) the reference values and "under" (marked in blue) means bacteria that did not recover (decreased) to values that were equivalent to the reference values whereas "ns" means the changes were not significant. For both WT(T) and Per(T), neg.log10q values are sorted in descending order on Day11.

	Day11				Day238			
genus	effect.size	neg.log10q	mouse.strain	over.or.under	effect.size	neg.log10q	mouse.strain	over.or.under
Enterococcus	3.93	6.77	WT(T)	over	0.75	0.26	WT(T)	ns
Petrimonas	-2.78	6.13	WT(T)	under	0.22	0.03	WT(T)	ns
Adlercreutzia	-2.95	5.44	WT(T)	under	-1.26	0.77	WT(T)	ns
Candidatus_Arthromitus	-3.28	4.68	WT(T)	under	-2.81	2.97	WT(T)	under
Akkermansia	4.89	4.50	WT(T)	over	-0.11	0.02	WT(T)	ns
Mediterraneibacter	1.42	4.13	WT(T)	over	-0.23	0.08	WT(T)	ns
Eggerthella	-1.35	4.13	WT(T)	under	-0.41	0.26	WT(T)	ns
Barnesiella	-2.53	3.84	WT(T)	under	0.27	0.03	WT(T)	ns
Bifidobacterium	-3.17	3.74	WT(T)	under	-2.19	1.08	WT(T)	ns
Lachnoclostridium	1.20	3.70	WT(T)	over	-0.17	0.07	WT(T)	ns
Chlorobaculum	-1.99	3.70	WT(T)	under	0.00	0.00	WT(T)	ns
Megasphaera	1.28	3.68	WT(T)	over	0.07	0.02	WT(T)	ns
Selenomonas	1.19	3.68	WT(T)	over	0.08	0.02	WT(T)	ns
Pseudomonas	0.76	3.66	WT(T)	over	0.25	0.26	WT(T)	ns
Clostridioides	1.47	3.52	WT(T)	over	0.06	0.02	WT(T)	ns
Mucilaginibacter	-1.24	3.49	WT(T)	under	0.26	0.09	WT(T)	ns
Streptococcus	1.40	3.48	WT(T)	over	0.23	0.07	WT(T)	ns
Streptomyces	0.86	3.43	WT(T)	over	0.25	0.21	WT(T)	ns
Faecalibacterium	1.11	3.24	WT(T)	over	-0.30	0.16	WT(T)	ns
Arabia	-1.09	3.16	WT(T)	under	-0.69	0.75	WT(T)	ns
Gordonibacter	-1.32	3.16	WT(T)	under	-0.64	0.36	WT(T)	ns
Candidatus_Saccharimonas	-2.45	2.88	WT(T)	under	-0.37	0.03	WT(T)	ns
Roseburia	1.03	2.84	WT(T)	over	-0.07	0.02	WT(T)	ns
Prevotella	-2.08	2.84	WT(T)	under	0.09	0.02	WT(T)	ns
Muribaculum	-4.54	2.84	WT(T)	under	0.73	0.03	WT(T)	ns
Candidatus_Nanosynbacter	-2.32	2.83	WT(T)	under	-0.30	0.03	WT(T)	ns
Aminipila	0.97	2.60	WT(T)	over	-0.02	0.01	WT(T)	ns
Alloprevotella	-1.34	2.60	WT(T)	under	0.22	0.03	WT(T)	ns
Proteiniphilum	-1.53	2.60	WT(T)	under	0.51	0.20	WT(T)	ns
Candidatus_Izimaplasma	-1.57	2.60	WT(T)	under	0.08	0.02	WT(T)	ns
Limosilactobacillus	4.04	2.32	WT(T)	over	0.88	0.07	WT(T)	ns
Acholeplasma	-1.89	2.32	WT(T)	under	0.08	0.02	WT(T)	ns
Sutterella	-1.91	2.27	WT(T)	under	-1.77	0.92	WT(T)	ns
Desulfotomaculum	1.17	2.27	WT(T)	over	0.51	0.26	WT(T)	ns
Mycoplasma	-1.09	2.25	WT(T)	under	0.08	0.02	WT(T)	ns
Blautia	2.01	2.25	WT(T)	over	0.23	0.02	WT(T)	ns
Fibrobacter	-1.52	2.25	WT(T)	under	0.05	0.02	WT(T)	ns
Amedibacterium	1.13	2.20	WT(T)	over	-0.30	0.08	WT(T)	ns
Duncaniella	-3.66	2.20	WT(T)	under	0.44	0.02	WT(T)	ns
Paenibacillus	1.17	2.13	WT(T)	over	0.17	0.02	WT(T)	ns
Desulfovibrio	-1.06	2.12	WT(T)	under	-0.27	0.07	WT(T)	ns
Nitrosomonas	-0.85	2.11	WT(T)	under	-0.57	0.37	WT(T)	ns
Bacillus	0.59	2.11	WT(T)	over	-0.01	0.00	WT(T)	ns
Runella	-1.31	2.11	WT(T)	under	0.16	0.02	WT(T)	ns
Alkaliphilus	-1.03	1.90	WT(T)	under	0.07	0.02	WT(T)	ns
Enterocloster	0.89	1.81	WT(T)	over	-0.15	0.02	WT(T)	ns
Faecalibaculum	-2.59	1.77	WT(T)	under	-1.75	0.36	WT(T)	ns

Ruminococcus	0.68	1.77	WT(T)	over	0.19	0.07	WT(T)	ns
Acetobacterium	0.88	1.73	WT(T)	over	-0.03	0.02	WT(T)	ns
Acinetobacter	0.90	1.73	WT(T)	over	0.00	0.00	WT(T)	ns
Christensenella	0.70	1.72	WT(T)	over	-0.18	0.07	WT(T)	ns
Hymenobacter	-1.18	1.72	WT(T)	under	0.37	0.08	WT(T)	ns
Trueperella	0.76	1.68	WT(T)	over	0.12	0.02	WT(T)	ns
Acutalibacter	0.68	1.65	WT(T)	over	-0.13	0.03	WT(T)	ns
Deinococcus	0.59	1.64	WT(T)	over	0.20	0.08	WT(T)	ns
Formosa	1.32	1.54	WT(T)	over	-0.61	0.19	WT(T)	ns
Cyclobacterium	-1.27	1.49	WT(T)	under	0.36	0.07	WT(T)	ns
Monoglobus	0.82	1.48	WT(T)	over	0.04	0.02	WT(T)	ns
Mucinivorans	-1.48	1.48	WT(T)	under	0.17	0.02	WT(T)	ns
Geobacillus	0.72	1.43	WT(T)	over	0.61	0.37	WT(T)	ns
Massilistercora	1.01	1.43	WT(T)	over	-0.09	0.02	WT(T)	ns
Chitinophaga	-0.59	1.41	WT(T)	under	0.20	0.08	WT(T)	ns
Ethanoligenens	0.67	1.39	WT(T)	over	0.29	0.13	WT(T)	ns
Exiguobacterium	1.03	1.39	WT(T)	over	-0.13	0.02	WT(T)	ns
Desulfosporosinus	1.08	1.38	WT(T)	over	0.76	0.26	WT(T)	ns
Hungatella	0.94	1.38	WT(T)	over	0.09	0.02	WT(T)	ns
Blattabacterium	1.01	1.36	WT(T)	over	0.11	0.02	WT(T)	ns
Acidaminococcus	0.77	1.36	WT(T)	over	0.29	0.08	WT(T)	ns
Actinomyces	0.66	1.34	WT(T)	over	-0.05	0.02	WT(T)	ns
Mesorhizobium	0.70	1.33	WT(T)	over	0.90	0.88	WT(T)	ns
Helicobacter	-9.28	18.86	Per(T)	under	-9.92	19.82	Per(T)	under
Adlercreutzia	-3.00	15.65	Per(T)	under	0.44	1.10	Per(T)	ns
Acidaminococcus	1.69	10.73	Per(T)	over	0.18	0.38	Per(T)	ns
Arabia	-1.61	10.73	Per(T)	under	0.60	2.67	Per(T)	over
Arcobacter	-1.95	9.77	Per(T)	under	-0.82	2.75	Per(T)	under
Microbacterium	1.63	9.50	Per(T)	over	1.17	5.87	Per(T)	over
Acholeplasma	-3.26	9.50	Per(T)	under	-0.91	1.37	Per(T)	under
Streptomyces	1.10	8.87	Per(T)	over	0.53	2.89	Per(T)	over
Geobacter	1.38	8.64	Per(T)	over	0.33	0.92	Per(T)	ns
Campylobacter	-1.38	8.54	Per(T)	under	-1.31	7.42	Per(T)	under
Pseudomonas	1.32	8.50	Per(T)	over	0.50	1.92	Per(T)	over
Acinetobacter	1.59	8.26	Per(T)	over	0.17	0.27	Per(T)	ns
Aeromonas	1.03	8.24	Per(T)	over	0.70	4.34	Per(T)	over
Gemella	-2.47	8.10	Per(T)	under	-0.80	1.36	Per(T)	under
Candidatus_Saccharimonas	-2.97	8.02	Per(T)	under	0.04	0.02	Per(T)	ns
Phocaeicola	-5.40	8.02	Per(T)	under	-4.57	5.87	Per(T)	under
Massilia	2.07	7.92	Per(T)	over	1.23	3.31	Per(T)	over
Geobacillus	1.25	7.77	Per(T)	over	0.31	0.78	Per(T)	ns
Mesorhizobium	1.90	7.75	Per(T)	over	1.15	3.31	Per(T)	over
Sphingobacterium	-2.15	7.65	Per(T)	under	-0.95	2.05	Per(T)	under
Aminipila	1.22	7.54	Per(T)	over	0.11	0.18	Per(T)	ns
Candidatus_Nanosynbacter	-2.83	7.54	Per(T)	under	-0.10	0.05	Per(T)	ns
Fusobacterium	-1.53	7.43	Per(T)	under	-0.49	1.15	Per(T)	ns
Paludibacter	-1.85	7.23	Per(T)	under	0.01	0.01	Per(T)	ns
Serratia	1.42	7.12	Per(T)	over	0.62	1.78	Per(T)	over
Thermus	1.73	7.07	Per(T)	over	0.20	0.24	Per(T)	ns
Enterococcus	2.46	6.64	Per(T)	over	-0.43	0.39	Per(T)	ns
Paenibacillus	0.93	6.55	Per(T)	over	0.11	0.22	Per(T)	ns
Massilistercora	1.22	6.43	Per(T)	over	-0.19	0.33	Per(T)	ns
Desulfovibrio	-1.44	6.43	Per(T)	under	-0.53	1.17	Per(T)	ns
Limosilactobacillus	2.28	6.28	Per(T)	over	-0.94	1.37	Per(T)	under
Candidatus_Izimaplasma	-2.44	6.28	Per(T)	under	-0.92	1.18	Per(T)	ns
Haemophilus	3.94	6.06	Per(T)	over	0.49	0.22	Per(T)	ns
Flintibacter	1.61	6.01	Per(T)	over	-0.31	0.40	Per(T)	ns
Blautia	1.40	6.00	Per(T)	over	0.13	0.16	Per(T)	ns
Clostridioides	1.26	5.82	Per(T)	over	0.33	0.62	Per(T)	ns
Rodentibacter	5.79	5.70	Per(T)	over	0.27	0.05	Per(T)	ns
Mediterraneibacter	1.21	5.68	Per(T)	over	0.12	0.16	Per(T)	ns
Blattabacterium	-1.21	5.66	Per(T)	under	-0.02	0.02	Per(T)	ns
Ligilactobacillus	3.08	5.64	Per(T)	over	-1.19	1.08	Per(T)	ns
Actinomyces	1.31	5.63	Per(T)	over	0.93	2.84	Per(T)	over
Megasphaera	0.96	5.50	Per(T)	over	0.19	0.39	Per(T)	ns
Lacrimispora	0.97	5.38	Per(T)	over	0.15	0.26	Per(T)	ns

Dysgonomonas	-2.14	5.38	Per(T)	under	-1.63	2.94	Per(T)	under
Selenomonas	0.96	5.21	Per(T)	over	0.33	0.82	Per(T)	ns
Faecalitalea	1.03	5.20	Per(T)	over	0.07	0.08	Per(T)	ns
Amedibacterium	0.98	5.02	Per(T)	over	-0.08	0.10	Per(T)	ns
Flavonifractor	1.22	5.02	Per(T)	over	-0.37	0.64	Per(T)	ns
Citrobacter	1.55	4.96	Per(T)	over	0.36	0.42	Per(T)	ns
Brachyspira	-1.53	4.94	Per(T)	under	-1.06	2.33	Per(T)	under
Intestinimonas	1.18	4.91	Per(T)	over	-0.36	0.62	Per(T)	ns
Streptococcus	1.40	4.85	Per(T)	over	0.03	0.02	Per(T)	ns
Fibrobacter	-1.57	4.80	Per(T)	under	-1.05	2.09	Per(T)	under
Petrimonas	-2.53	4.59	Per(T)	under	-0.58	0.39	Per(T)	ns
Desulfosarcina	1.21	4.46	Per(T)	over	0.00	0.00	Per(T)	ns
Leptotrichia	-1.33	4.42	Per(T)	under	-0.59	1.00	Per(T)	ns
Treponema	0.93	4.37	Per(T)	over	-0.01	0.02	Per(T)	ns
Cloacibacillus	1.00	4.33	Per(T)	over	-0.22	0.33	Per(T)	ns
Draconibacterium	-1.46	4.33	Per(T)	under	-0.27	0.27	Per(T)	ns
Deinococcus	1.02	4.29	Per(T)	over	0.63	1.65	Per(T)	over
Vibrio	0.99	4.28	Per(T)	over	0.09	0.11	Per(T)	ns
Runella	-1.27	4.22	Per(T)	under	0.04	0.03	Per(T)	ns
Hungatella	0.87	4.14	Per(T)	over	-0.11	0.16	Per(T)	ns
Synechococcus	0.65	4.07	Per(T)	over	0.33	1.14	Per(T)	ns
Prevotella	-2.52	4.05	Per(T)	under	-1.84	2.04	Per(T)	under
Neisseria	1.08	4.03	Per(T)	over	0.62	1.36	Per(T)	over
Parolsenella	-1.32	3.85	Per(T)	under	1.50	3.66	Per(T)	over
Burkholderia	1.21	3.84	Per(T)	over	0.83	1.74	Per(T)	over
Faecalibacterium	0.85	3.83	Per(T)	over	-0.12	0.16	Per(T)	ns
Klebsiella	1.31	3.70	Per(T)	over	1.05	2.09	Per(T)	over
Formosa	-1.54	3.61	Per(T)	under	0.17	0.11	Per(T)	ns
Desulfotomaculum	0.98	3.55	Per(T)	over	0.17	0.21	Per(T)	ns
Bifidobacterium	-2.61	3.55	Per(T)	under	0.05	0.02	Per(T)	ns
Christensenella	0.83	3.55	Per(T)	over	-0.04	0.03	Per(T)	ns
Anaerostipes	0.83	3.52	Per(T)	over	-0.13	0.18	Per(T)	ns
Caproiciproducens	0.96	3.51	Per(T)	over	0.05	0.04	Per(T)	ns
Bradyrhizobium	0.73	3.50	Per(T)	over	0.22	0.44	Per(T)	ns
Monoglobus	1.00	3.32	Per(T)	over	-0.23	0.28	Per(T)	ns
Lachnoclostridium	0.90	3.32	Per(T)	over	-0.24	0.36	Per(T)	ns
Staphylococcus	1.95	3.20	Per(T)	over	0.04	0.02	Per(T)	ns
Erysipelatoclostridium	0.78	3.20	Per(T)	over	-0.01	0.01	Per(T)	ns
Alkaliphilus	-1.37	3.00	Per(T)	under	-0.60	0.65	Per(T)	ns
Ruminococcus	0.64	2.97	Per(T)	over	0.01	0.01	Per(T)	ns
Desulfitobacterium	0.75	2.92	Per(T)	over	-0.39	0.82	Per(T)	ns
Peptoniphilus	1.26	2.92	Per(T)	over	1.51	3.02	Per(T)	over
Odoribacter	-2.37	2.90	Per(T)	under	-0.78	0.40	Per(T)	ns
Bacteroides	-3.28	2.89	Per(T)	under	-1.52	0.68	Per(T)	ns
Paraprevotella	-2.10	2.81	Per(T)	under	-0.62	0.34	Per(T)	ns
Thermaerobacter	1.05	2.69	Per(T)	over	0.23	0.22	Per(T)	ns
Gordonibacter	-0.81	2.69	Per(T)	under	0.39	0.65	Per(T)	ns
Butyricimonas	-2.50	2.69	Per(T)	under	-1.03	0.52	Per(T)	ns
Chryseobacterium	0.98	2.52	Per(T)	over	0.61	0.92	Per(T)	ns
Capnocytophaga	-1.02	2.35	Per(T)	under	-0.56	0.70	Per(T)	ns
Pedobacter	-1.11	2.26	Per(T)	under	0.50	0.52	Per(T)	ns
Ethanoligenens	0.78	2.16	Per(T)	over	0.12	0.11	Per(T)	ns
Eubacterium	0.63	2.11	Per(T)	over	-0.13	0.16	Per(T)	ns
Flavobacterium	-0.51	2.06	Per(T)	under	0.02	0.02	Per(T)	ns
Sphingomonas	0.77	2.05	Per(T)	over	0.82	1.74	Per(T)	over
Muribaculum	-2.81	1.99	Per(T)	under	0.85	0.26	Per(T)	ns
Mahella	-0.95	1.98	Per(T)	under	-0.48	0.52	Per(T)	ns
Oscillibacter	0.67	1.98	Per(T)	over	-0.33	0.52	Per(T)	ns
Eggerthella	-0.54	1.94	Per(T)	under	0.55	1.57	Per(T)	over
Parabacteroides	-2.91	1.93	Per(T)	under	-1.36	0.45	Per(T)	ns
Lactobacillus	-1.33	1.86	Per(T)	under	-0.22	0.11	Per(T)	ns
Dysosmobacter	0.60	1.82	Per(T)	over	-0.41	0.76	Per(T)	ns
Acutalibacter	0.59	1.70	Per(T)	over	-0.53	1.08	Per(T)	ns
Corynebacterium	0.74	1.66	Per(T)	over	1.18	2.67	Per(T)	over
Roseburia	0.59	1.66	Per(T)	over	-0.27	0.39	Per(T)	ns
Rhodococcus	0.99	1.62	Per(T)	over	0.41	0.32	Per(T)	ns

Arthrobacter	0.60	1.56	Per(T)	over	0.17	0.18	Per(T)	ns
Rhizobium	0.63	1.56	Per(T)	over	0.54	0.92	Per(T)	ns
Alloprevotella	-1.21	1.45	Per(T)	under	-0.13	0.05	Per(T)	ns
Cyclobacterium	-1.10	1.43	Per(T)	under	-1.86	2.55	Per(T)	under
Bacillus	0.37	1.39	Per(T)	over	0.02	0.02	Per(T)	ns
Enterocloster	0.51	1.39	Per(T)	over	-0.33	0.52	Per(T)	ns
Alistipes	-1.58	1.35	Per(T)	under	0.99	0.51	Per(T)	ns
Candidatus_Arthromitus	-1.87	1.35	Per(T)	under	-2.11	1.23	Per(T)	ns
Herbinix	0.52	1.33	Per(T)	over	-0.07	0.06	Per(T)	ns
Mycoplasma	-0.63	1.11	Per(T)	ns	-0.85	1.36	Per(T)	under
Akkermansia	1.24	1.03	Per(T)	ns	2.60	2.54	Per(T)	over
Hymenobacter	0.33	0.49	Per(T)	ns	0.91	1.69	Per(T)	over
Salmonella	-0.32	0.40	Per(T)	ns	-1.02	1.65	Per(T)	under
Peptoclostridium	0.24	0.36	Per(T)	ns	0.72	1.36	Per(T)	over
Enterobacter	0.18	0.31	Per(T)	ns	0.80	2.09	Per(T)	over
Libanicoccus	-0.13	0.12	Per(T)	ns	1.15	2.09	Per(T)	over
Fibrella	-0.13	0.09	Per(T)	ns	1.28	1.64	Per(T)	over
Collinsella	0.06	0.07	Per(T)	ns	0.80	2.09	Per(T)	over
Chryseolinea	0.09	0.06	Per(T)	ns	1.42	2.04	Per(T)	over
Olsenella	0.06	0.05	Per(T)	ns	1.51	3.44	Per(T)	over
Turicibacter	-0.07	0.05	Per(T)	ns	4.45	10.15	Per(T)	over
Spirosoma	0.03	0.02	Per(T)	ns	0.91	1.57	Per(T)	over
