# Supplementary material for: Host circadian behaviors exert only weak selective pressure on the gut microbiome under stable conditions but are critical for recovery from antibiotic treatment
Source: PLoS Biol. 2022 Nov 9;20(11):e3001865. doi: 10.1371/journal.pbio.3001865 (PMC9645659; doi:10.1371/journal.pbio.3001865)
Supplement: S4 Table — Columns are: “log2FC” is the log2-fold change of the bacterial abundances, “neg.log10q” is the negative log q-value (q-value is the adjusted p-value), “mouse.strain” indicates either WT or Per1/2-dko {Per(T) in the table}, and “over.or.under” where “over” (marked in red) indicates bacteria at the chosen time point that recovered (increased) to values beyond (over) the reference values and “under” (marked in blue) indicates bacteria that did not recover (decreased) to values that were equivalent to the reference values. For both WT(T) and Per(T), neg.log10q values are sorted in descending order on Day 11. (PDF) [file pbio.3001865.s012.pdf]

S4 Table. Log2 fold change and p-value for species responding to the antibiotic treatment (Day 11) and at the endpoint (Day 238). Columns are: “log2FC” is the log2-fold change of the bacterial abundances, “neg.log10q” is the negative log q-value (q-value is the adjusted p-value), “mouse.strain” indicates either WT or Per1/2-dko {Per(T) in the table}, and “over.or.under” where "over" (marked in red) indicates bacteria at the chosen time point that recovered (increased) to values beyond (over) the reference values and "under" (marked in blue) indicates bacteria that did not recover (decreased) to values that were equivalent to the reference values. For both WT(T) and Per(T), neg.log10q values are sorted in descending order on Day 11.

S4 Table

Log2 fold change and p-value for species responding to the antibiotic treatment(Day11) and at the end point(Day238) compared to the reference(Day-14). Columns are: "log2FC" is the log2 fold change of the bacterial abundance changes, "neg.log10q" is the negative log10 q-value (q-value is the adjusted p-value), "mouse.strain" indicates either WT(T) or Per(T),"over.or.under" codes "over"(marked in red) to mean bacteria at the chosen time point recovered (increased) to values beyond (over) the reference values and "under" (marked in blue) means bacteria that did not recover (decreased) to values that were equivalent to the reference values whereas "ns" means the changes were not significant. For both WT(T) and Per(T), neg.log10q values are sorted in descending order on Day11.

	Day11				Day238			
**Species**	**log2FC**	**neg.log10q**	**mouse.strain**	**over.or.under**	**log2FC**	**neg.log10q**	**mouse.strain**	**over.or.under**
Paraprevotella_xylaniphila	-2.11	3.61	WT(T)	under	-0.35	0.15	WT(T)	ns
Eggerthella_sp._YY7918	-2.70	3.61	WT(T)	under	-0.67	0.26	WT(T)	ns
Lactiplantibacillus_plantarum	-2.94	3.61	WT(T)	under	-0.38	0.21	WT(T)	ns
Treponema_socranskii	-3.17	3.61	WT(T)	under	-0.04	0.00	WT(T)	ns
Paraflavitalea_soli	-3.62	3.61	WT(T)	under	0.72	0.64	WT(T)	ns
Eggerthella_lenta	-4.23	3.61	WT(T)	under	-1.09	1.48	WT(T)	under
Barnesiella_viscericola	-3.82	3.61	WT(T)	under	-0.23	0.05	WT(T)	ns
Alistipes_sp._dk3624	-3.83	3.61	WT(T)	under	0.20	0.34	WT(T)	ns
Porphyromonas_cangingivalis	-3.95	3.61	WT(T)	under	-0.35	0.22	WT(T)	ns
Alloprevotella_sp._E39	-4.03	3.61	WT(T)	under	-0.10	0.00	WT(T)	ns
Gluconobacter_oxydans	-4.15	3.61	WT(T)	under	-1.77	0.95	WT(T)	ns
Sulfuritortus_calidifontis	-4.17	3.61	WT(T)	under	-1.48	0.57	WT(T)	ns
Proteiniphilum_saccharofermentans	-4.20	3.61	WT(T)	under	0.12	0.00	WT(T)	ns
Chryseobacterium_shandongense	-4.25	3.61	WT(T)	under	-0.41	0.15	WT(T)	ns
Hymenobacter_sp._PAMC_26628	-4.26	3.61	WT(T)	under	1.05	0.42	WT(T)	ns
Cyanobacterium_aponinum	-4.30	3.61	WT(T)	under	-1.31	1.13	WT(T)	ns
Pontibacter_sp._SGAir0037	-4.40	3.61	WT(T)	under	0.02	0.00	WT(T)	ns
Spirosoma_sp._TS118	-4.58	3.61	WT(T)	under	0.63	0.00	WT(T)	ns
Bifidobacterium_dentium	-4.58	3.61	WT(T)	under	-0.74	0.35	WT(T)	ns
Chlorobaculum_limnaeum	-4.62	3.61	WT(T)	under	-0.06	0.00	WT(T)	ns
Fibrobacter_succinogenes	-4.77	3.61	WT(T)	under	-0.61	0.24	WT(T)	ns
Streptococcus_ratti	-4.80	3.61	WT(T)	under	-2.23	1.13	WT(T)	ns
Muribaculum_sp._TLL-A4	-4.92	3.61	WT(T)	under	-0.02	0.01	WT(T)	ns
Corynebacterium_sp._2184	-4.93	3.61	WT(T)	under	-0.34	0.01	WT(T)	ns
Prevotella_dentalis	-4.95	3.61	WT(T)	under	-0.34	0.16	WT(T)	ns
Niastella_koreensis	-5.06	3.61	WT(T)	under	0.02	0.00	WT(T)	ns
Prevotella_denticola	-5.09	3.61	WT(T)	under	0.00	0.00	WT(T)	ns
Methylococcus_sp._IM1	NA	3.61	WT(T)	ns	-2.80	1.43	WT(T)	under
Cruoricaptor_ignavus	-5.13	3.61	WT(T)	under	0.01	0.00	WT(T)	ns
Prevotella_oris	-5.27	3.61	WT(T)	under	-0.57	0.03	WT(T)	ns
Chryseobacterium_taklimakanense	-5.53	3.61	WT(T)	under	-0.49	0.11	WT(T)	ns
Variovorax_paradoxus	-5.63	3.61	WT(T)	under	0.81	0.21	WT(T)	ns
Sphingobacterium_sp._ML3W	-5.68	3.61	WT(T)	under	-0.12	0.01	WT(T)	ns
Mucilaginibacter_ginsenosidivorans	-5.72	3.61	WT(T)	under	0.07	0.00	WT(T)	ns
Chitinophaga_sp._MD30	-5.87	3.61	WT(T)	under	-1.43	0.42	WT(T)	ns
Salinivirga_cyanobacteriivorans	-5.92	3.61	WT(T)	under	1.29	0.24	WT(T)	ns
Prevotella_fusca	-6.05	3.61	WT(T)	under	-1.74	0.24	WT(T)	ns
Limihaloglobus_sulfuriphilus	-6.23	3.61	WT(T)	under	-0.14	0.00	WT(T)	ns
Spirochaeta_africana	-6.27	3.61	WT(T)	under	-1.17	0.42	WT(T)	ns
Chryseobacterium_sp._cx-624	-6.36	3.61	WT(T)	under	-0.58	0.16	WT(T)	ns
Prevotella_ruminicola	-6.43	3.61	WT(T)	under	-1.04	0.16	WT(T)	ns
Prevotella_enoeca	-6.45	3.61	WT(T)	under	0.11	0.00	WT(T)	ns
Prevotella_sp._oral_taxon_299	-6.49	3.61	WT(T)	under	0.67	0.00	WT(T)	ns
Algoriphagus_sanaruensis	-6.69	3.61	WT(T)	under	-0.73	0.27	WT(T)	ns
Xanthomonas_oryzae	-6.73	3.61	WT(T)	under	-0.54	0.01	WT(T)	ns
Shewanella_amazonensis	-6.79	3.61	WT(T)	under	-0.07	0.00	WT(T)	ns
Ottowia_sp._oral_taxon_894	-7.55	3.61	WT(T)	under	-1.78	1.13	WT(T)	ns
Adlercreutzia_sp._8CFCBH1	-4.09	3.45	WT(T)	under	-1.15	1.39	WT(T)	under
Adlercreutzia_equolifaciens	-4.30	3.45	WT(T)	under	-1.19	1.39	WT(T)	under

Sorangium_cellulosum	-3.01	3.45	WT(T)	under	-0.55	0.16	WT(T)	ns
Thiocystis_violascens	-3.05	3.45	WT(T)	under	0.47	0.14	WT(T)	ns
Actinomyces_qiguomingii	-3.23	3.45	WT(T)	under	-0.38	0.05	WT(T)	ns
Pontibacter_russatus	-3.38	3.45	WT(T)	under	-0.82	0.75	WT(T)	ns
Chitinophaga_pinensis	-3.48	3.45	WT(T)	under	0.10	0.05	WT(T)	ns
Tannerella_forsythia	-3.94	3.45	WT(T)	under	-0.26	0.01	WT(T)	ns
Nibribacter_sp._BT10	-3.96	3.45	WT(T)	under	-0.38	0.03	WT(T)	ns
Petrimonas_mucosa	-4.15	3.45	WT(T)	under	-0.36	0.05	WT(T)	ns
Hymenobacter_jejuensis	-4.25	3.45	WT(T)	under	-0.11	0.00	WT(T)	ns
Campylobacter_gracilis	-4.37	3.45	WT(T)	under	-0.06	0.00	WT(T)	ns
Hymenobacter_swuensis	-4.45	3.45	WT(T)	under	0.31	0.11	WT(T)	ns
Sphingobacterium_sp._DR205	-4.48	3.45	WT(T)	under	-0.41	0.11	WT(T)	ns
Gallaecimonas_mangrovi	-4.49	3.45	WT(T)	under	-0.03	0.00	WT(T)	ns
Flavihumibacter_sp._SB-02	-4.53	3.45	WT(T)	under	0.71	0.31	WT(T)	ns
Euzebyella_marina	-4.53	3.45	WT(T)	under	-0.11	0.00	WT(T)	ns
Pontibacter_korlensis	-4.54	3.45	WT(T)	under	-0.06	0.00	WT(T)	ns
Chlorobaculum_parvum	-4.55	3.45	WT(T)	under	-0.91	0.66	WT(T)	ns
Ereboglobus_luteus	-4.87	3.45	WT(T)	under	0.05	0.00	WT(T)	ns
Arachidicoccus_soli	-5.12	3.45	WT(T)	under	-0.90	0.24	WT(T)	ns
Cytophaga_hutchinsonii	-5.28	3.45	WT(T)	under	-0.20	0.00	WT(T)	ns
Flavobacterium_alkalisoli	-5.32	3.45	WT(T)	under	-0.23	0.00	WT(T)	ns
Saccharopolyspora_coralli	-4.19	3.31	WT(T)	under	-0.74	0.22	WT(T)	ns
Arthrobacter_sp._FB24	-4.44	3.31	WT(T)	under	-0.04	0.00	WT(T)	ns
Bacteriovorax_stolpii	-4.51	3.31	WT(T)	under	-0.28	0.12	WT(T)	ns
Thiomicrorhabdus_sp._G2	-4.77	3.31	WT(T)	under	-0.16	0.00	WT(T)	ns
Empedobacter_brevis	-4.99	3.31	WT(T)	under	0.27	0.00	WT(T)	ns
Dokdonia_sp._MED134	-5.32	3.31	WT(T)	under	0.34	0.01	WT(T)	ns
Arabia_massiliensis	-3.16	3.28	WT(T)	under	-1.07	1.43	WT(T)	under
Alistipes_indistinctus	-2.35	3.28	WT(T)	under	0.03	0.00	WT(T)	ns
Parolsenella_catena	-2.55	3.28	WT(T)	under	-0.19	0.27	WT(T)	ns
Candidatus_Nitrospira_inopinata	-2.57	3.28	WT(T)	under	0.18	0.00	WT(T)	ns
Hymenobacter_oligotrophus	-2.96	3.28	WT(T)	under	-0.06	0.00	WT(T)	ns
Pelobacter_acetylenicus	-3.05	3.28	WT(T)	under	-0.35	0.01	WT(T)	ns
Chitinophaga_sp._XS-30	-3.05	3.28	WT(T)	under	-0.33	0.07	WT(T)	ns
Chryseolinea_soli	-3.38	3.28	WT(T)	under	0.50	0.35	WT(T)	ns
Chryseobacterium_sp._6424	-3.87	3.28	WT(T)	under	-0.09	0.00	WT(T)	ns
Mucilaginibacter_sp._F39-2	-4.02	3.28	WT(T)	under	-0.74	0.27	WT(T)	ns
Alkalitalea_saponilacus	-4.07	3.28	WT(T)	under	-0.13	0.00	WT(T)	ns
Candidatus_Planktophila_vernalis	-4.18	3.28	WT(T)	under	0.06	0.00	WT(T)	ns
Flavisolibacter_tropicus	-4.19	3.28	WT(T)	under	-1.17	0.28	WT(T)	ns
Prevotella_scopos	-4.22	3.28	WT(T)	under	-0.47	0.08	WT(T)	ns
Duncaniella_sp._C9	-4.29	3.28	WT(T)	under	-0.13	0.00	WT(T)	ns
Rhodoferax_ferrireducens	-4.42	3.28	WT(T)	under	-0.32	0.00	WT(T)	ns
Halomonas_sp._KO116	-4.43	3.28	WT(T)	under	-0.20	0.00	WT(T)	ns
Candidatus_Saccharimonas_aalborgensis	-4.47	3.28	WT(T)	under	-0.43	0.00	WT(T)	ns
Hymenobacter_sp._NBH84	-4.50	3.28	WT(T)	under	0.25	0.00	WT(T)	ns
Pedobacter_sp._HDW13	-4.89	3.28	WT(T)	under	-1.01	0.40	WT(T)	ns
Prevotella_melaninogenica	-4.97	3.28	WT(T)	under	-2.32	0.71	WT(T)	ns
Acetilactobacillus_jinshanensis	-4.05	3.19	WT(T)	under	-0.48	0.00	WT(T)	ns
Candidatus_Nanosynbacter_lyticus	-4.06	3.19	WT(T)	under	-0.31	0.00	WT(T)	ns
Nonlabens_sp._Hel1_33_55	-4.11	3.15	WT(T)	under	-1.73	1.03	WT(T)	ns
Lutibacter_profundi	-3.47	3.15	WT(T)	under	-0.21	0.00	WT(T)	ns
Desulfovibrio_alaskensis	-3.49	3.15	WT(T)	under	-0.39	0.00	WT(T)	ns
Stenotrophomonas_sp._ZAC14D2_NAIMI4_6	-3.51	3.15	WT(T)	under	-0.07	0.00	WT(T)	ns
Sideroxydans_lithotrophicus	-3.61	3.15	WT(T)	under	0.81	0.11	WT(T)	ns
Runella_sp._SP2	-3.66	3.15	WT(T)	under	-1.15	0.61	WT(T)	ns
Dysgonomonas_sp._HDW5B	-3.71	3.15	WT(T)	under	0.56	0.16	WT(T)	ns
Duncaniella_sp._B8	-3.98	3.15	WT(T)	under	0.75	0.74	WT(T)	ns
Bernardetia_litoralis	-4.10	3.15	WT(T)	under	-0.19	0.00	WT(T)	ns
Pedobacter_suwonensis	-4.16	3.15	WT(T)	under	-0.65	0.06	WT(T)	ns
Desulfoglaeba_alkanexedens	-3.71	3.13	WT(T)	under	-2.01	1.21	WT(T)	ns
Eggerthella_sp._HF-1101	-3.10	3.13	WT(T)	under	-0.94	1.13	WT(T)	ns
Robiginitalea_biformata	-2.88	3.13	WT(T)	under	-0.27	0.01	WT(T)	ns
Hymenobacter_sp._BT182	-2.88	3.13	WT(T)	under	-0.53	0.11	WT(T)	ns
Mucinivorans_hirudinis	-2.90	3.13	WT(T)	under	-0.19	0.02	WT(T)	ns
Dyadobacter_fermentans	-2.98	3.13	WT(T)	under	-0.26	0.16	WT(T)	ns

Roseimaritima_ulvae	-3.24	3.13	WT(T)	under	0.12	0.00	WT(T)	ns
Rhodopseudomonas_palustris	-3.24	3.13	WT(T)	under	-0.25	0.00	WT(T)	ns
Porphyromonas_crevioricanis	-3.45	3.13	WT(T)	under	1.15	0.48	WT(T)	ns
Hyphobacterium_sp._CCMP332	-3.48	3.13	WT(T)	under	-0.76	0.21	WT(T)	ns
Leuconostoc_citreum	-3.48	3.13	WT(T)	under	0.22	0.00	WT(T)	ns
Pusillimonas_sp._DMV24BSW_D	-3.52	3.13	WT(T)	under	-0.97	0.40	WT(T)	ns
Hymenobacter_sp._BRD128	-4.10	3.13	WT(T)	under	-0.59	0.03	WT(T)	ns
Spirosoma_pollinicola	-4.11	3.13	WT(T)	under	-0.27	0.00	WT(T)	ns
Gramella_fulva	-4.43	3.13	WT(T)	under	0.04	0.00	WT(T)	ns
Rodentibacter_heylii	-3.94	3.05	WT(T)	under	-5.87	1.81	WT(T)	under
Pseudoleptotrichia_goodfellowii	-3.44	3.05	WT(T)	under	-2.73	1.48	WT(T)	under
Flaviflexus_sp._H23T48	-5.71	3.05	WT(T)	under	-3.05	1.39	WT(T)	under
Thermus_oshimai	-3.72	3.05	WT(T)	under	-1.77	1.10	WT(T)	ns
Proteus_mirabilis	-5.48	3.05	WT(T)	under	-2.27	1.10	WT(T)	ns
Hymenobacter_sp._BT18	-2.77	3.05	WT(T)	under	0.32	0.00	WT(T)	ns
Hymenobacter_sp._APR13	-3.04	3.05	WT(T)	under	0.69	0.14	WT(T)	ns
Cyclobacterium_marinum	-3.06	3.05	WT(T)	under	-0.29	0.03	WT(T)	ns
Tenuifilum_thalassicum	-3.10	3.05	WT(T)	under	-0.19	0.05	WT(T)	ns
Mucilaginibacter_gotjawali	-3.67	3.05	WT(T)	under	-1.74	0.87	WT(T)	ns
Hymenobacter_nivis	-3.14	3.05	WT(T)	under	-0.92	0.35	WT(T)	ns
Desulfovibrio_vulgaris	-3.22	3.05	WT(T)	under	-1.07	0.11	WT(T)	ns
Hymenobacter_sp._DG25B	-3.25	3.05	WT(T)	under	-0.69	0.35	WT(T)	ns
Hymenobacter_sp._PAMC_26554	-3.28	3.05	WT(T)	under	0.13	0.00	WT(T)	ns
Porphyromonas_asaccharolytica	-3.34	3.05	WT(T)	under	0.08	0.00	WT(T)	ns
Desulfovibrio_sp._86	-3.43	3.05	WT(T)	under	-0.76	0.08	WT(T)	ns
Athalassotoga_saccharophila	-3.49	3.05	WT(T)	under	-0.24	0.01	WT(T)	ns
Hymenobacter_sp._DG01	-3.49	3.05	WT(T)	under	-0.22	0.00	WT(T)	ns
Photobacterium_damselae	-3.53	3.05	WT(T)	under	0.05	0.00	WT(T)	ns
Draconibacterium_sp._M1	-3.86	3.05	WT(T)	under	-0.33	0.00	WT(T)	ns
Chryseobacterium_balustinum	-3.88	3.05	WT(T)	under	0.23	0.00	WT(T)	ns
Duncaniella_dubosii	-3.90	3.05	WT(T)	under	-0.13	0.00	WT(T)	ns
Bifidobacterium_choerinum	-4.33	3.05	WT(T)	under	-2.15	0.75	WT(T)	ns
Microbacterium_pygmaeum	-4.59	3.05	WT(T)	under	-0.31	0.03	WT(T)	ns
Gemmatimonas_aurantiaca	-5.53	3.05	WT(T)	under	-1.11	0.45	WT(T)	ns
Dyella_sp._G9	-5.56	3.05	WT(T)	under	-1.20	0.48	WT(T)	ns
Chryseobacterium_joostei	-5.75	3.05	WT(T)	under	0.33	0.00	WT(T)	ns
Pseudomonas_sihuiensis	-6.53	3.05	WT(T)	under	0.25	0.00	WT(T)	ns
Colwellia_sp._Arc7-635	-6.72	3.05	WT(T)	under	-1.67	0.71	WT(T)	ns
Hypericibacter_terrae	-6.87	3.05	WT(T)	under	-0.56	0.07	WT(T)	ns
Aquimarina_sp._AD1	-7.01	3.05	WT(T)	under	-1.25	0.11	WT(T)	ns
Nonlabens_sp._MJ115	-7.04	3.05	WT(T)	under	-0.34	0.00	WT(T)	ns
Sutterella_faecalis	-3.64	3.01	WT(T)	under	-1.78	0.61	WT(T)	ns
Gloeomargarita_lithophora	-3.61	2.95	WT(T)	under	-0.04	0.01	WT(T)	ns
Synechococcus_sp._JA-3-3Ab	-2.97	2.94	WT(T)	under	0.12	0.00	WT(T)	ns
Deinococcus_geothermalis	-3.07	2.94	WT(T)	under	-0.31	0.05	WT(T)	ns
Spirochaeta_thermophila	-3.29	2.94	WT(T)	under	0.60	0.11	WT(T)	ns
Spirosoma_montaniterrae	-3.31	2.94	WT(T)	under	-0.73	0.61	WT(T)	ns
Corynebacterium_sphenisci	-3.67	2.94	WT(T)	under	-0.55	0.14	WT(T)	ns
Haliscomenobacter_hydrossis	-3.82	2.94	WT(T)	under	0.01	0.00	WT(T)	ns
Candidatus_Phytoplasma_australiense	-3.94	2.94	WT(T)	under	-1.22	0.19	WT(T)	ns
Sphingobacterium_mizutaii	-3.98	2.93	WT(T)	under	-1.66	0.75	WT(T)	ns
Fibrella_sp._ES10-3-2-2	-4.11	2.93	WT(T)	under	0.87	0.11	WT(T)	ns
Phaeobacter_inhibens	-5.00	2.93	WT(T)	under	-0.53	0.10	WT(T)	ns
Nodularia_spumigena	-5.02	2.93	WT(T)	under	0.50	0.00	WT(T)	ns
Nitrosomonas_communis	-5.43	2.93	WT(T)	under	-0.88	0.11	WT(T)	ns
Erythrobacter_aureus	-3.01	2.91	WT(T)	under	-0.52	0.10	WT(T)	ns
Rubrobacter_sp._SCSIO_52909	-3.08	2.91	WT(T)	under	-1.21	0.48	WT(T)	ns
Riemerella_anatipestifer	-3.27	2.91	WT(T)	under	-0.80	0.24	WT(T)	ns
Wenzhouxiangella_sp._AB-CW3	-3.36	2.91	WT(T)	under	-1.21	0.52	WT(T)	ns
Kangiella_sediminilitoris	-3.96	2.91	WT(T)	under	-1.33	0.23	WT(T)	ns
Gordonibacter_urolithinfaciens	-2.95	2.91	WT(T)	under	-0.80	0.87	WT(T)	ns
Candidatus_Arthromitus_sp._SFB-rat-Yit	-4.51	2.91	WT(T)	under	-2.49	0.81	WT(T)	ns
Desulfomicrobium_orale	-2.69	2.91	WT(T)	under	-1.37	0.75	WT(T)	ns
Acinetobacter_radioresistens	-2.74	2.91	WT(T)	under	-0.49	0.22	WT(T)	ns
Treponema_azotonutricium	-2.74	2.91	WT(T)	under	-0.63	0.24	WT(T)	ns
Gordonibacter_pamelaeae	-2.95	2.91	WT(T)	under	-0.85	0.48	WT(T)	ns

Staphylococcus_pseudintermedius	-3.62	2.91	WT(T)	under	-1.65	0.55	WT(T)	ns
Bifidobacterium_pseudolongum	-4.54	2.91	WT(T)	under	-2.01	0.66	WT(T)	ns
Bacteroides_sp._HF-162	-4.93	2.91	WT(T)	under	-1.24	0.01	WT(T)	ns
Kiritimatiella_glycovorans	-2.99	2.83	WT(T)	under	0.36	0.00	WT(T)	ns
Kushneria_marisflavi	-3.32	2.83	WT(T)	under	-0.15	0.00	WT(T)	ns
Candidatus_Desulfovibrio_trichonymphae	-3.62	2.83	WT(T)	under	-0.89	0.16	WT(T)	ns
Moraxella_bovoculi	-4.94	2.83	WT(T)	under	0.00	0.00	WT(T)	ns
Cyclobacterium_amurskyense	-4.96	2.83	WT(T)	under	0.02	0.00	WT(T)	ns
Microbulbifer_sp._SH-1	-3.65	2.75	WT(T)	under	0.66	0.00	WT(T)	ns
Candidatus_Cyclonatronum_proteinivorum	-4.06	2.75	WT(T)	under	-0.78	0.24	WT(T)	ns
Geobacter_sp._DSM_9736	-5.16	2.75	WT(T)	under	-0.68	0.11	WT(T)	ns
Wolinella_succinogenes	-3.15	2.75	WT(T)	under	-2.78	1.39	WT(T)	under
Flavobacterium_sediminis	-3.00	2.75	WT(T)	under	-0.24	0.01	WT(T)	ns
Fibrella_aestuarina	-3.25	2.75	WT(T)	under	0.02	0.00	WT(T)	ns
Spirosoma_sp._CJU-R4	-3.42	2.75	WT(T)	under	1.15	0.66	WT(T)	ns
Mucilaginibacter_ginsenosidivorax	-3.30	2.75	WT(T)	under	0.58	0.02	WT(T)	ns
Anseongella_ginsenosidimutans	-3.48	2.75	WT(T)	under	0.55	0.30	WT(T)	ns
Corynebacterium_sp._NML93-0612	-4.51	2.75	WT(T)	under	-0.28	0.01	WT(T)	ns
Bifidobacterium_animalis	-3.82	2.74	WT(T)	under	-3.06	1.13	WT(T)	ns
Tannerella_sp._oral_taxon_HOT-286	-2.34	2.74	WT(T)	under	-0.06	0.00	WT(T)	ns
Desulfovibrio_marinus	-2.37	2.74	WT(T)	under	0.03	0.01	WT(T)	ns
Prevotella_intermedia	-2.97	2.74	WT(T)	under	0.21	0.11	WT(T)	ns
Haloactinobacterium_sp._RN3S43	-2.95	2.74	WT(T)	under	-0.79	0.11	WT(T)	ns
Sphingobacterium_lactis	-3.04	2.74	WT(T)	under	0.07	0.00	WT(T)	ns
Niabella_ginsenosidivorans	-3.07	2.74	WT(T)	under	0.22	0.01	WT(T)	ns
Propionibacterium_freudenreichii	-3.27	2.74	WT(T)	under	-1.27	0.59	WT(T)	ns
Vibrio_campbellii	-3.94	2.71	WT(T)	under	0.00	0.00	WT(T)	ns
Elizabethkingia_bruuniana	-4.07	2.71	WT(T)	under	0.07	0.00	WT(T)	ns
Muricauda_sp._501str8	-4.45	2.71	WT(T)	under	0.10	0.00	WT(T)	ns
Aeromonas_encheleia	-4.56	2.71	WT(T)	under	-1.49	0.46	WT(T)	ns
Komagataeibacter_medellinensis	-4.62	2.71	WT(T)	under	-1.69	0.46	WT(T)	ns
Corynebacterium_diphtheriae	-4.70	2.71	WT(T)	under	0.43	0.03	WT(T)	ns
Aequorivita_sublithincola	-4.81	2.71	WT(T)	under	-0.42	0.00	WT(T)	ns
Elizabethkingia_anophelis	-2.83	2.67	WT(T)	under	-0.43	0.01	WT(T)	ns
Bergeyella_cardium	-3.04	2.67	WT(T)	under	-0.40	0.00	WT(T)	ns
Desulfovibrio_salexigens	-2.97	2.66	WT(T)	under	-0.65	0.01	WT(T)	ns
Mucilaginibacter_rubeus	-4.65	2.66	WT(T)	under	-0.43	0.00	WT(T)	ns
Polynucleobacter_necessarius	-3.08	2.63	WT(T)	under	-0.44	0.02	WT(T)	ns
Vibrio_mediterranei	-5.15	2.62	WT(T)	under	-2.92	0.83	WT(T)	ns
Paraburkholderia_graminis	-3.68	2.62	WT(T)	under	-1.79	0.71	WT(T)	ns
Spirosoma_rigui	-1.89	2.62	WT(T)	under	-0.34	0.11	WT(T)	ns
Enterococcus_mundtii	-2.09	2.62	WT(T)	under	-0.16	0.01	WT(T)	ns
Rhodothermus_marinus	-2.47	2.62	WT(T)	under	0.15	0.00	WT(T)	ns
Corallococcus_coralloides	-2.92	2.62	WT(T)	under	-0.56	0.18	WT(T)	ns
Alkaliphilus_metalliredigens	-3.03	2.62	WT(T)	under	-0.22	0.00	WT(T)	ns
Arenibacter_algicola	-3.80	2.62	WT(T)	under	-0.02	0.00	WT(T)	ns
Campylobacter_sp._RM6914	-4.36	2.62	WT(T)	under	-0.16	0.00	WT(T)	ns
Marinobacter_sp._Arc7-DN-1	-4.65	2.62	WT(T)	under	0.10	0.00	WT(T)	ns
Beggiatoa_leptomitoformis	-5.08	2.62	WT(T)	under	-0.94	0.07	WT(T)	ns
Candidatus_Solibacter_usitatus	-2.68	2.58	WT(T)	under	0.01	0.00	WT(T)	ns
Rubinisphaera_brasiliensis	-2.76	2.58	WT(T)	under	-0.40	0.13	WT(T)	ns
Cronobacter_sakazakii	-3.10	2.58	WT(T)	under	-0.85	0.15	WT(T)	ns
Algibacter_sp._L3A6	-3.83	2.53	WT(T)	under	-0.77	0.11	WT(T)	ns
Thermanaerovibrio_velox	-3.90	2.53	WT(T)	under	-0.97	0.18	WT(T)	ns
Xanthomonas_citri	-4.28	2.53	WT(T)	under	-1.31	0.27	WT(T)	ns
Gemella_sanguinis	-4.32	2.53	WT(T)	under	0.01	0.00	WT(T)	ns
Enterococcus_gallinarum	10.29	2.53	WT(T)	over	7.89	0.42	WT(T)	ns
Denitratisoma_sp._DHT3	-2.91	2.51	WT(T)	under	-2.07	0.95	WT(T)	ns
Parvimonas_micra	-2.71	2.51	WT(T)	under	-0.56	0.08	WT(T)	ns
Acidobacterium_capsulatum	-2.89	2.51	WT(T)	under	-0.74	0.05	WT(T)	ns
Desulfovibrio_fairfieldensis	-3.16	2.51	WT(T)	under	-0.88	0.12	WT(T)	ns
Salmonella_enterica	-2.42	2.50	WT(T)	under	1.03	0.01	WT(T)	ns
Carnobacterium_sp._CP1	-2.57	2.49	WT(T)	under	-3.50	1.48	WT(T)	under
Herbaspirillum_rubrisubalbicans	-4.26	2.49	WT(T)	under	-4.88	1.44	WT(T)	under
Streptococcus_urinalis	-5.27	2.49	WT(T)	under	-4.55	1.42	WT(T)	under
Methylomonas_methanica	-5.29	2.49	WT(T)	under	-6.69	1.42	WT(T)	under

Candidatus_Arthromitus_sp._SFB-mouse-NL	-7.61	2.49	WT(T)	under	-3.58	1.13	WT(T)	ns
Candidatus_Arthromitus_sp._SFB-mouse	-6.53	2.49	WT(T)	under	-3.58	1.10	WT(T)	ns
Gloeothece_citriformis	-5.79	2.49	WT(T)	under	-2.31	0.74	WT(T)	ns
Burkholderia_gladioli	-3.72	2.49	WT(T)	under	-2.81	0.72	WT(T)	ns
Francisella_persica	-7.97	2.49	WT(T)	under	-3.86	0.66	WT(T)	ns
Prevotella_jejuni	-1.54	2.49	WT(T)	under	-0.05	0.00	WT(T)	ns
Paludibaculum_fermentans	-2.16	2.49	WT(T)	under	-0.64	0.53	WT(T)	ns
Akkermansia_glycaniphila	-2.56	2.49	WT(T)	under	-0.23	0.01	WT(T)	ns
Petrotoga_mobilis	-2.60	2.49	WT(T)	under	0.18	0.00	WT(T)	ns
Candidatus_Izimaplasma_sp._HR1	-2.62	2.49	WT(T)	under	-0.07	0.00	WT(T)	ns
Acholeplasma_hippikon	-2.65	2.49	WT(T)	under	-1.01	0.05	WT(T)	ns
Lacipirellula_parvula	-2.69	2.49	WT(T)	under	-0.44	0.03	WT(T)	ns
Paludibacter_propionicigenes	-2.72	2.49	WT(T)	under	0.39	0.26	WT(T)	ns
Acholeplasma_palmae	-2.72	2.49	WT(T)	under	2.36	0.16	WT(T)	ns
Kaistella_daneshvariae	-2.82	2.49	WT(T)	under	-0.61	0.39	WT(T)	ns
Vibrio_atlanticus	-2.88	2.49	WT(T)	under	-0.52	0.02	WT(T)	ns
Bacteroides_sp._PHL_2737	-2.89	2.49	WT(T)	under	-0.54	0.11	WT(T)	ns
Winogradskyella_sp._J14-2	-2.89	2.49	WT(T)	under	-0.55	0.05	WT(T)	ns
Anaerolinea_sp._'rifampicinis'	-2.98	2.49	WT(T)	under	-0.41	0.18	WT(T)	ns
Frigoriglobus_tundricola	-3.00	2.49	WT(T)	under	0.24	0.00	WT(T)	ns
Myxococcus_xanthus	-3.11	2.49	WT(T)	under	-1.95	0.46	WT(T)	ns
Mesobacillus_jeotgali	-3.55	2.49	WT(T)	under	-1.34	0.48	WT(T)	ns
Granulosicoccus_antarcticus	-3.65	2.49	WT(T)	under	-0.28	0.01	WT(T)	ns
Haematospirillum_jordaniae	-3.70	2.49	WT(T)	under	-1.12	0.07	WT(T)	ns
Opitutus_sp._GAS368	-4.06	2.49	WT(T)	under	-0.73	0.14	WT(T)	ns
Flavisolibacter_ginsenosidimutans	-4.36	2.49	WT(T)	under	-1.44	0.45	WT(T)	ns
Streptococcus_sp._A12	-4.66	2.49	WT(T)	under	-0.58	0.00	WT(T)	ns
Streptomyces_sp._So13.3	-4.78	2.49	WT(T)	under	-0.43	0.01	WT(T)	ns
Cupriavidus_necator	-5.15	2.49	WT(T)	under	-1.67	0.52	WT(T)	ns
Rhizobium_sp._ACO-34A	-5.36	2.49	WT(T)	under	-1.19	0.25	WT(T)	ns
Melittangium_boletus	-5.40	2.49	WT(T)	under	-0.87	0.01	WT(T)	ns
Pseudolysobacter_antarcticus	-5.56	2.49	WT(T)	under	-0.39	0.00	WT(T)	ns
Corynebacterium_sp._NML98-0116	-5.57	2.49	WT(T)	under	-1.78	0.46	WT(T)	ns
Micrococcus_luteus	-5.71	2.49	WT(T)	under	-0.91	0.13	WT(T)	ns
Hydrogenophilus_thermoluteolus	-6.52	2.49	WT(T)	under	-0.36	0.00	WT(T)	ns
Aquimarina_sp._TRL1	-6.61	2.49	WT(T)	under	-0.03	0.00	WT(T)	ns
Methylomonas_sp._GJ1	-2.50	2.46	WT(T)	under	-0.74	0.11	WT(T)	ns
Sutterella_megalosphaeroides	-2.96	2.46	WT(T)	under	-1.98	0.95	WT(T)	ns
Fastidiosipila_sanguinis	-2.69	2.46	WT(T)	under	-0.10	0.00	WT(T)	ns
Nesterenkonia_sp._NBAIMH1	-3.11	2.46	WT(T)	under	0.12	0.00	WT(T)	ns
Lacimicrobium_alkaliphilum	-2.69	2.45	WT(T)	under	-0.84	0.35	WT(T)	ns
Desulfovibrio_piger	-2.63	2.45	WT(T)	under	-1.00	0.11	WT(T)	ns
Draconibacterium_orientale	-2.19	2.42	WT(T)	under	0.21	0.01	WT(T)	ns
Clostridium_estertheticum	-2.37	2.42	WT(T)	under	-0.74	0.11	WT(T)	ns
Rhodobacter_sphaeroides	-2.37	2.42	WT(T)	under	0.10	0.01	WT(T)	ns
Campylobacter_pinnipediorum	-2.54	2.42	WT(T)	under	-3.53	1.44	WT(T)	under
Kosakonia_sp._SMBL-WEM22	-2.53	2.42	WT(T)	under	1.69	1.02	WT(T)	ns
Succinivibrio_dextrinosolvens	-2.38	2.42	WT(T)	under	-1.31	1.02	WT(T)	ns
Bacillus_sp._FJAT-45348	-3.77	2.42	WT(T)	under	-2.21	0.87	WT(T)	ns
Acinetobacter_gyllenbergii	-3.02	2.42	WT(T)	under	-1.90	0.83	WT(T)	ns
Mixta_theicola	-2.66	2.42	WT(T)	under	-1.21	0.52	WT(T)	ns
Bifidobacterium_catenulatum	-2.22	2.42	WT(T)	under	-0.43	0.11	WT(T)	ns
Turicibacter_sp._H121	-2.27	2.42	WT(T)	under	4.36	0.00	WT(T)	ns
Aequorivita_sp._H23M31	-2.29	2.42	WT(T)	under	0.26	0.00	WT(T)	ns
Pseudobacter_ginsenosidimutans	-2.31	2.42	WT(T)	under	-0.51	0.18	WT(T)	ns
Labilibaculum_antarcticum	-2.41	2.42	WT(T)	under	-0.01	0.00	WT(T)	ns
Pelobacter_propionicus	-2.83	2.42	WT(T)	under	1.20	0.31	WT(T)	ns
Hymenobacter_sedentarius	-2.98	2.42	WT(T)	under	0.16	0.00	WT(T)	ns
Adhaeribacter_swui	-3.05	2.42	WT(T)	under	-0.03	0.00	WT(T)	ns
Vibrio_vulnificus	-3.29	2.42	WT(T)	under	0.31	0.00	WT(T)	ns
Pseudoalteromonas_ulvae	-3.42	2.42	WT(T)	under	-0.54	0.11	WT(T)	ns
Staphylococcus_haemolyticus	-3.59	2.42	WT(T)	under	-0.02	0.00	WT(T)	ns
Leptotrichia_sp._oral_taxon_212	-4.43	2.42	WT(T)	under	1.32	0.11	WT(T)	ns
Thermoanaerobacter_kivui	-3.66	2.42	WT(T)	under	-2.89	1.13	WT(T)	ns
[Enterobacter]_lignolyticus	-3.46	2.42	WT(T)	under	0.81	0.21	WT(T)	ns
Bradyrhizobium_sp._CCBAU_53421	-3.49	2.42	WT(T)	under	-1.12	0.24	WT(T)	ns

Nocardia_wallacei	-1.87	2.42	WT(T)	under	-1.18	0.50	WT(T)	ns
Planctomyces_sp._SH-PL62	-2.06	2.42	WT(T)	under	0.11	0.00	WT(T)	ns
Alcaligenes_faecalis	-1.96	2.40	WT(T)	under	0.75	0.26	WT(T)	ns
Campylobacter_coli	-3.72	2.34	WT(T)	under	-3.78	1.44	WT(T)	under
Mesobacillus_foraminis	-2.28	2.34	WT(T)	under	-0.34	0.00	WT(T)	ns
Agrobacterium_vitis	-3.47	2.34	WT(T)	under	-0.37	0.01	WT(T)	ns
Fimbriimonas_ginsengisoli	-3.67	2.34	WT(T)	under	-1.24	0.39	WT(T)	ns
Rufibacter_sp._DG15C	-2.56	2.34	WT(T)	under	-1.11	0.42	WT(T)	ns
Buchnera_aphidicola	-2.57	2.34	WT(T)	under	-1.25	0.42	WT(T)	ns
Clostridium_cellulovorans	-2.85	2.33	WT(T)	under	-1.91	0.61	WT(T)	ns
Porphyromonas_gingivalis	-1.56	2.33	WT(T)	under	-0.12	0.00	WT(T)	ns
Proteus_vulgaris	-1.67	2.32	WT(T)	under	-0.40	0.02	WT(T)	ns
Allochromatium_vinosum	-2.96	2.32	WT(T)	under	-0.53	0.10	WT(T)	ns
Thiomonas_intermedia	-3.32	2.32	WT(T)	under	-0.59	0.03	WT(T)	ns
Gemmobacter_sp._HYN0069	-3.40	2.32	WT(T)	under	-1.18	0.40	WT(T)	ns
Peptoniphilus_ivorii	-1.65	2.31	WT(T)	under	-0.89	0.42	WT(T)	ns
Desulfotomaculum_ferrireducens	-2.13	2.31	WT(T)	under	0.16	0.00	WT(T)	ns
Mucilaginibacter_paludis	-2.16	2.31	WT(T)	under	-1.18	1.48	WT(T)	under
Synechococcus_sp._A15-24	-2.56	2.31	WT(T)	under	-4.21	1.43	WT(T)	under
Leuconostoc_suionicum	-6.81	2.31	WT(T)	under	-7.75	1.42	WT(T)	under
Denitrobacterium_detoxificans	-2.13	2.31	WT(T)	under	-1.04	1.10	WT(T)	ns
Sphingobacterium_daejeonense	-5.76	2.31	WT(T)	under	-4.99	1.10	WT(T)	ns
Muribaculum_intestinale	-2.65	2.31	WT(T)	under	0.60	0.61	WT(T)	ns
Candidatus_Promineofilum_breve	-0.77	2.31	WT(T)	under	0.88	0.19	WT(T)	ns
Desulfovibrio_ferrophilus	-1.79	2.31	WT(T)	under	0.25	0.01	WT(T)	ns
Phocaeicola_salanitronis	-2.31	2.31	WT(T)	under	-0.08	0.00	WT(T)	ns
Propionibacterium_australiense	-2.40	2.31	WT(T)	under	-0.46	0.35	WT(T)	ns
Candidatus_Cytomitobacter_indipagum	-2.71	2.31	WT(T)	under	-0.11	0.00	WT(T)	ns
Terriglobus_albidus	-2.99	2.31	WT(T)	under	-0.50	0.01	WT(T)	ns
Hirschia_baltica	-3.09	2.31	WT(T)	under	-1.27	0.11	WT(T)	ns
Pseudomonas_furukawaii	-3.57	2.31	WT(T)	under	-0.80	0.24	WT(T)	ns
Sphingopyxis_sp._113P3	-3.81	2.31	WT(T)	under	0.76	0.02	WT(T)	ns
Mycobacteroides_chelonae	-4.06	2.31	WT(T)	under	0.62	0.22	WT(T)	ns
Georgenia_sp._Z443	-4.71	2.31	WT(T)	under	0.25	0.00	WT(T)	ns
Chitinophaga_sp._H33E-04	-5.28	2.31	WT(T)	under	-1.77	0.00	WT(T)	ns
Pseudodesulfovibrio_sp._SRB007	-5.30	2.31	WT(T)	under	0.82	0.14	WT(T)	ns
Streptomyces_ficellus	-5.41	2.31	WT(T)	under	-0.07	0.00	WT(T)	ns
Streptobacillus_moniliformis	-5.67	2.31	WT(T)	under	0.19	0.00	WT(T)	ns
Sedimenticola_thiotaurini	-6.10	2.31	WT(T)	under	-0.73	0.00	WT(T)	ns
Lactobacillus_brevis	-2.28	2.24	WT(T)	under	-1.34	0.48	WT(T)	ns
Chryseobacterium_sp.	-1.78	2.24	WT(T)	under	0.39	0.00	WT(T)	ns
Chryseobacterium_manosquense	-3.24	2.24	WT(T)	under	-0.22	0.00	WT(T)	ns
Flammeovirga_pectinis	-3.07	2.24	WT(T)	under	0.20	0.00	WT(T)	ns
Hyphomonas_sp._Mor2	-3.28	2.24	WT(T)	under	-0.13	0.00	WT(T)	ns
Methylocella_silvestris	-3.56	2.24	WT(T)	under	-0.96	0.11	WT(T)	ns
Crassaminicella_sp._SY095	-0.85	2.23	WT(T)	under	-0.54	0.01	WT(T)	ns
Oryzomicrobium_terrae	-2.22	2.23	WT(T)	under	-0.93	0.42	WT(T)	ns
Neobacillus_mesonae	-2.90	2.22	WT(T)	under	-0.58	0.11	WT(T)	ns
Martelella_endophytica	-3.13	2.22	WT(T)	under	-0.18	0.01	WT(T)	ns
Desulfocurvibacter_africanus	-3.04	2.20	WT(T)	under	-2.61	1.48	WT(T)	under
Clostridium_baratii	-1.20	2.20	WT(T)	under	-0.46	0.08	WT(T)	ns
Bifidobacterium_adolescentis	-1.79	2.20	WT(T)	under	-0.29	0.01	WT(T)	ns
Escherichia_coli	-2.04	2.20	WT(T)	under	1.30	0.00	WT(T)	ns
Oligotropha_carboxidovorans	-2.85	2.20	WT(T)	under	-0.84	0.46	WT(T)	ns
Olivibacter_sp._SDN3	-2.77	2.20	WT(T)	under	-1.06	0.12	WT(T)	ns
Chlorobaculum_tepidum	-2.95	2.20	WT(T)	under	-0.24	0.00	WT(T)	ns
Luteolibacter_sp._G-1-1-1	-2.97	2.20	WT(T)	under	-0.97	0.14	WT(T)	ns
Clostridium_sp._DL-VIII	-1.43	2.20	WT(T)	under	-0.89	0.94	WT(T)	ns
Hathewaya_histolytica	-1.81	2.20	WT(T)	under	-0.98	0.87	WT(T)	ns
Pontibacter_actiniarum	-1.80	2.20	WT(T)	under	0.58	0.13	WT(T)	ns
Libanicoccus_massiliensis	-1.67	2.20	WT(T)	under	0.68	0.00	WT(T)	ns
Brochothrix_thermosphacta	-1.93	2.20	WT(T)	under	-0.72	0.16	WT(T)	ns
Paraoceanicella_profunda	-2.10	2.20	WT(T)	under	0.02	0.00	WT(T)	ns
Streptomyces_koyangensis	-2.35	2.20	WT(T)	under	-0.58	0.27	WT(T)	ns
Faecalibaculum_rodentium	-2.56	2.20	WT(T)	under	-0.82	0.42	WT(T)	ns
Eikenella_corrodens	-3.11	2.20	WT(T)	under	0.01	0.00	WT(T)	ns

Xenorhabdus_nematophila	-3.42	2.20	WT(T)	under	-0.09	0.01	WT(T)	ns
Aromatoleum_aromaticum	-2.83	2.18	WT(T)	under	0.21	0.03	WT(T)	ns
Butyricicoccus_sp._GAM44	-2.85	2.18	WT(T)	under	-0.89	0.01	WT(T)	ns
Chitinolyticbacter_meiyuanensis	-2.85	2.18	WT(T)	under	0.15	0.00	WT(T)	ns
Sphingosinithalassobacter_sp._CS137	-3.00	2.18	WT(T)	under	-0.29	0.00	WT(T)	ns
Gilvibacter_sp._SZ-19	-3.06	2.18	WT(T)	under	-0.69	0.21	WT(T)	ns
Mariprofundus_ferrinatatus	-4.02	2.18	WT(T)	under	-0.07	0.00	WT(T)	ns
Chryseobacterium_sp._NEB161	-4.50	2.18	WT(T)	under	1.10	0.24	WT(T)	ns
Chondrocystis_sp._NIES-4102	-2.84	2.18	WT(T)	under	-0.13	0.00	WT(T)	ns
Methylorubrum_populi	-2.88	2.18	WT(T)	under	1.20	0.10	WT(T)	ns
Flexistipes_sinusarabici	-3.53	2.18	WT(T)	under	-0.70	0.00	WT(T)	ns
Sinorhizobium_fredii	-1.63	2.17	WT(T)	under	-0.37	0.01	WT(T)	ns
Anaerococcus_mediterraneensis	-3.11	2.13	WT(T)	under	-2.44	0.71	WT(T)	ns
Schlegelella_thermodepolymerans	-2.32	2.13	WT(T)	under	-0.77	0.24	WT(T)	ns
Lysobacter_soli	-2.40	2.13	WT(T)	under	-0.98	0.35	WT(T)	ns
Jeotgalicoccus_saudimassiliensis	-3.26	2.13	WT(T)	under	0.83	0.03	WT(T)	ns
Muricauda_ruestringensis	-2.65	2.13	WT(T)	under	0.55	0.16	WT(T)	ns
Sporolactobacillus_terrae	-2.75	2.13	WT(T)	under	0.18	0.00	WT(T)	ns
Runella_sp._HYN0085	-2.89	2.12	WT(T)	under	-0.12	0.00	WT(T)	ns
Thermoleptolyngbya_sp._PKUAC-SCTA183	-3.01	2.12	WT(T)	under	-1.34	0.39	WT(T)	ns
Runella_slithyformis	-3.22	2.12	WT(T)	under	0.05	0.00	WT(T)	ns
Dehalobacter_restrictus	-2.24	2.12	WT(T)	under	-1.19	0.61	WT(T)	ns
Ensifer_adhaerens	-2.21	2.12	WT(T)	under	-0.17	0.00	WT(T)	ns
Lacibacter_sp._S13-6-6	-0.94	2.11	WT(T)	under	0.12	0.00	WT(T)	ns
Parabacteroides_sp._CT06	-1.80	2.11	WT(T)	under	-0.32	0.01	WT(T)	ns
Opitutus_terrae	-2.36	2.11	WT(T)	under	0.12	0.00	WT(T)	ns
Geovibrio_thiophilus	-2.42	2.11	WT(T)	under	0.00	0.01	WT(T)	ns
Pedobacter_sp._G11	-2.66	2.11	WT(T)	under	0.36	0.01	WT(T)	ns
Blattabacterium_cuenoti	-2.80	2.11	WT(T)	under	-1.06	0.24	WT(T)	ns
Erysipelothrix_piscisicarius	-3.04	2.11	WT(T)	under	-1.10	0.24	WT(T)	ns
Stackebrandtia_nassauensis	-3.84	2.11	WT(T)	under	0.22	0.00	WT(T)	ns
Thioalkalivibrio_versutus	-4.28	2.11	WT(T)	under	-0.55	0.10	WT(T)	ns
Hymenobacter_sp._TS19	-2.13	2.10	WT(T)	under	-0.05	0.01	WT(T)	ns
Microlunatus_soli	-2.39	2.10	WT(T)	under	-0.43	0.12	WT(T)	ns
Bifidobacterium_longum	-2.02	2.10	WT(T)	under	-1.21	1.10	WT(T)	ns
Clostridium_bornimense	-1.12	2.10	WT(T)	under	-3.10	0.61	WT(T)	ns
Parascardovia_denticolens	-2.36	2.10	WT(T)	under	-1.52	0.59	WT(T)	ns
Moorella_thermoacetica	-2.14	2.10	WT(T)	under	1.04	0.45	WT(T)	ns
Desulfovibrio_sulfodismutans	-2.08	2.10	WT(T)	under	-0.49	0.11	WT(T)	ns
Cutibacterium_avidum	-2.09	2.10	WT(T)	under	-0.37	0.11	WT(T)	ns
Erysipelatoclostridium_ramosum	-2.13	2.10	WT(T)	under	-0.62	0.02	WT(T)	ns
Alistipes_megaguti	-2.28	2.10	WT(T)	under	0.25	0.01	WT(T)	ns
Mucilaginibacter_sp._HYN0043	-2.54	2.10	WT(T)	under	-0.27	0.05	WT(T)	ns
Lactobacillus_paragasseri	-2.73	2.10	WT(T)	under	-1.37	0.21	WT(T)	ns
Maribacter_sp._1_2014MBL_MicDiv	-3.62	2.10	WT(T)	under	-5.10	1.17	WT(T)	ns
Nonomuraea_nitratireducens	-2.27	2.10	WT(T)	under	-1.79	0.56	WT(T)	ns
Radiobacillus_deserti	-2.33	2.05	WT(T)	under	0.17	0.00	WT(T)	ns
Pandoraea_sp._XY-2	-2.56	2.05	WT(T)	under	0.33	0.01	WT(T)	ns
Hymenobacter_sp._S2-20-2	-3.41	2.03	WT(T)	under	-0.25	0.00	WT(T)	ns
Chryseobacterium_indoltheticum	-5.70	2.03	WT(T)	under	-5.71	1.13	WT(T)	ns
Denitrovibrio_acetiphilus	-2.77	2.03	WT(T)	under	-2.48	0.75	WT(T)	ns
Phoenicibacter_congonensis	-2.45	2.03	WT(T)	under	-1.93	0.61	WT(T)	ns
Nostoc_sp._NIES-4103	-6.19	2.03	WT(T)	under	-2.66	0.60	WT(T)	ns
Tenacibaculum_sp._SZ-18	-7.59	2.03	WT(T)	under	-4.49	0.60	WT(T)	ns
Mobilicoccus_sp._NJES-13	-4.64	2.03	WT(T)	under	-1.92	0.52	WT(T)	ns
Coriobacterium_glomerans	-0.05	2.03	WT(T)	under	-0.14	0.01	WT(T)	ns
Hymenobacter_sp._BRD67	-0.55	2.03	WT(T)	under	-0.22	0.00	WT(T)	ns
Geobacter_bremensis	-1.79	2.03	WT(T)	under	0.03	0.00	WT(T)	ns
Desulfarculus_baarsii	-1.95	2.03	WT(T)	under	-0.82	0.14	WT(T)	ns
Desulfuromonas_sp._DDH964	-1.97	2.03	WT(T)	under	-0.54	0.01	WT(T)	ns
Aquisphaera_giovannonii	-1.97	2.03	WT(T)	under	-0.49	0.16	WT(T)	ns
Nitratireductor_sp._OM-1	-2.11	2.03	WT(T)	under	-0.35	0.01	WT(T)	ns
Alistipes_dispar	-2.17	2.03	WT(T)	under	0.00	0.00	WT(T)	ns
Sulfuricaulis_limicola	-2.18	2.03	WT(T)	under	-0.40	0.11	WT(T)	ns
Alistipes_finegoldii	-2.24	2.03	WT(T)	under	0.35	0.01	WT(T)	ns
Thermocrinis_minervae	-2.31	2.03	WT(T)	under	-0.79	0.11	WT(T)	ns

Desulfotalea_psychrophila	-2.31	2.03	WT(T)	under	-0.54	0.11	WT(T)	ns
Verrucomicrobium_sp._GAS474	-2.57	2.03	WT(T)	under	0.53	0.01	WT(T)	ns
Sphingomonas_sanxanigenens	-2.82	2.03	WT(T)	under	-0.58	0.14	WT(T)	ns
Enterobacter_cloacae	-2.88	2.03	WT(T)	under	8.04	0.03	WT(T)	ns
Rhodocytophaga_sp._172606-1	-3.01	2.03	WT(T)	under	-0.82	0.21	WT(T)	ns
Rufibacter_sp._DG31D	-3.07	2.03	WT(T)	under	0.17	0.01	WT(T)	ns
Gemmatirosa_kalamazoonesis	-3.12	2.03	WT(T)	under	-0.76	0.03	WT(T)	ns
Streptomyces_sp._CCM_MD2014	-3.31	2.03	WT(T)	under	-1.49	0.34	WT(T)	ns
Dehalogenimonas_formicexedens	-3.50	2.03	WT(T)	under	1.15	0.02	WT(T)	ns
Corynebacterium_segmentosum	-3.57	2.03	WT(T)	under	0.11	0.00	WT(T)	ns
Xanthomonas_hortorum	-3.61	2.03	WT(T)	under	0.72	0.11	WT(T)	ns
Flavobacterium_anhuiense	-3.87	2.03	WT(T)	under	-1.30	0.24	WT(T)	ns
Sporosarcina_ureae	-3.89	2.03	WT(T)	under	-1.17	0.33	WT(T)	ns
Rickettsia_typhi	-4.01	2.03	WT(T)	under	-1.39	0.14	WT(T)	ns
Nocardioides_sp._78	-5.63	2.03	WT(T)	under	-1.82	0.11	WT(T)	ns
Paenisporosarcina_antarctica	-5.69	2.03	WT(T)	under	-1.52	0.30	WT(T)	ns
Alistipes_communis	-2.12	2.03	WT(T)	under	0.34	0.01	WT(T)	ns
Alistipes_onderdonkii	-2.29	2.03	WT(T)	under	0.30	0.01	WT(T)	ns
Polynucleobacter_asymbioticus	-1.24	2.00	WT(T)	under	1.84	0.28	WT(T)	ns
Sulfuritalea_hydrogenivorans	-1.68	1.99	WT(T)	under	-3.89	1.19	WT(T)	ns
Hyphomicrobium_denitrificans	-2.45	1.99	WT(T)	under	-1.43	0.46	WT(T)	ns
Helicobacter_canadensis	-3.61	1.99	WT(T)	under	-2.22	0.42	WT(T)	ns
Pseudomonas_corrugata	-3.11	1.99	WT(T)	under	0.37	0.00	WT(T)	ns
Candidatus_Phycorickettsia_trachydisci	-3.62	1.99	WT(T)	under	-0.66	0.05	WT(T)	ns
Geobacter_sp._M18	-3.81	1.99	WT(T)	under	-0.54	0.01	WT(T)	ns
Neisseria_bacilliformis	-3.83	1.99	WT(T)	under	-0.72	0.00	WT(T)	ns
Inhella_inkyongensis	-1.98	1.97	WT(T)	under	-2.09	1.15	WT(T)	ns
Clostridium_sp._JN-9	-0.98	1.97	WT(T)	under	-0.76	0.14	WT(T)	ns
Thauera_sp._MZ1T	-2.06	1.97	WT(T)	under	-0.40	0.35	WT(T)	ns
Geobacter_anodireducens	-2.18	1.97	WT(T)	under	-1.99	1.04	WT(T)	ns
Clostridium_intestinale	-1.17	1.97	WT(T)	under	-0.83	0.48	WT(T)	ns
Pedobacter_ginsengisoli	-1.72	1.97	WT(T)	under	-0.71	0.24	WT(T)	ns
Azospirillum_brasilense	-2.30	1.97	WT(T)	under	-0.61	0.03	WT(T)	ns
Chlorobium_phaeobacteroides	-1.46	1.96	WT(T)	under	0.05	0.00	WT(T)	ns
Actinopolymorpha_singaporensis	-2.40	1.96	WT(T)	under	-1.70	0.75	WT(T)	ns
Clostridium_taeniosporum	-2.37	1.96	WT(T)	under	-2.21	0.76	WT(T)	ns
Pandoraea_apista	-3.79	1.96	WT(T)	under	-1.69	0.45	WT(T)	ns
Candidatus_Endomicrobium_trichonymphae	-1.78	1.96	WT(T)	under	-1.23	0.42	WT(T)	ns
Planctomyces_sp._SH-PL14	-1.26	1.96	WT(T)	under	-1.26	0.35	WT(T)	ns
Thermomonospora_curvata	-1.77	1.96	WT(T)	under	-0.54	0.00	WT(T)	ns
Streptomyces_tirandamycinicus	-1.87	1.96	WT(T)	under	-0.58	0.11	WT(T)	ns
Microbulbifer_aggregans	-3.37	1.96	WT(T)	under	-0.37	0.01	WT(T)	ns
Echinicola_strongylocentroti	-3.61	1.96	WT(T)	under	0.41	0.00	WT(T)	ns
Lactobacillus_rossiae	-5.66	1.96	WT(T)	under	-3.54	0.33	WT(T)	ns
Gemella_haemolysans	-2.52	1.95	WT(T)	under	-2.20	0.75	WT(T)	ns
Actinobaculum_sp._313	-2.36	1.95	WT(T)	under	0.92	0.13	WT(T)	ns
Nibricoccus_aquaticus	-2.93	1.95	WT(T)	under	0.02	0.00	WT(T)	ns
Sphingomonas_sp._HDW15A	-4.21	1.95	WT(T)	under	0.42	0.00	WT(T)	ns
Blastochloris_tepida	-2.70	1.95	WT(T)	under	-2.07	0.54	WT(T)	ns
Slackia_heliotrinireducens	-1.30	1.95	WT(T)	under	-0.42	0.07	WT(T)	ns
Selenomonas_sp._oral_taxon_920	-1.31	1.95	WT(T)	under	-0.93	0.06	WT(T)	ns
Rhodococcus_sp._X156	-1.85	1.95	WT(T)	under	-0.02	0.00	WT(T)	ns
Flavobacterium_magnum	-2.63	1.95	WT(T)	under	-0.44	0.11	WT(T)	ns
Chryseobacterium_carnipullorum	-2.91	1.95	WT(T)	under	-0.86	0.01	WT(T)	ns
Sphingomonas_sp._LMO-1	-2.96	1.95	WT(T)	under	-0.24	0.00	WT(T)	ns
Pelosinus_sp._UFO1	-3.36	1.95	WT(T)	under	-1.46	0.01	WT(T)	ns
Bacteroides_sp._CACC_737	-3.75	1.95	WT(T)	under	-1.47	0.02	WT(T)	ns
Hydrocarboniclastica_marina	-3.43	1.93	WT(T)	under	-1.87	0.60	WT(T)	ns
Nitrosomonas_ureae	-3.63	1.93	WT(T)	under	0.04	0.00	WT(T)	ns
Staphylococcus_vitulinus	-2.88	1.89	WT(T)	under	0.15	0.00	WT(T)	ns
Deinococcus_maricopensis	-2.16	1.89	WT(T)	under	-0.78	0.11	WT(T)	ns
Veillonella_rodentium	-2.44	1.89	WT(T)	under	-1.16	0.42	WT(T)	ns
Thermovibrio_ammonificans	-0.81	1.89	WT(T)	under	1.59	0.14	WT(T)	ns
Neisseria_elongata	-1.37	1.89	WT(T)	under	0.37	0.00	WT(T)	ns
Thermosulfurimonas_marina	-1.83	1.89	WT(T)	under	-0.33	0.01	WT(T)	ns
Oceanobacillus_sp._160	-4.92	1.88	WT(T)	under	-1.04	0.00	WT(T)	ns

Listeria_ivanovii	-1.37	1.87	WT(T)	under	-1.09	0.46	WT(T)	ns
Streptomyces_sp._NHF165	-2.18	1.87	WT(T)	under	-0.92	0.39	WT(T)	ns
Pseudarthrobacter_chlorophenolicus	-2.25	1.87	WT(T)	under	-0.68	0.24	WT(T)	ns
Fusobacterium_ulcerans	-2.75	1.87	WT(T)	under	-1.39	0.24	WT(T)	ns
Marinobacter_sp._JH2	-2.48	1.87	WT(T)	under	-0.94	0.16	WT(T)	ns
Klebsiella_aerogenes	-2.50	1.87	WT(T)	under	1.21	0.03	WT(T)	ns
Acholeplasma_axanthum	-1.98	1.85	WT(T)	under	-3.16	0.60	WT(T)	ns
Nitrosomonas_stercoris	-3.09	1.85	WT(T)	under	-1.80	0.47	WT(T)	ns
Desulfobulbus_propionicus	-2.36	1.85	WT(T)	under	0.21	0.00	WT(T)	ns
Verminephrobacter_eiseniae	-3.12	1.85	WT(T)	under	0.18	0.00	WT(T)	ns
Anaerohalosphaera_lusitana	-2.77	1.85	WT(T)	under	1.29	0.10	WT(T)	ns
Aerococcus_urinae	-0.89	1.84	WT(T)	under	-0.77	0.39	WT(T)	ns
Pedobacter_heparinus	-3.20	1.84	WT(T)	under	1.73	1.13	WT(T)	ns
Chitinophaga_caeni	-2.28	1.84	WT(T)	under	1.32	0.59	WT(T)	ns
Candidatus_Reidiella_endopervernicosa	-2.34	1.84	WT(T)	under	-0.47	0.01	WT(T)	ns
Brachyspira_intermedia	-5.68	1.83	WT(T)	under	-4.37	0.80	WT(T)	ns
Chryseobacterium_sp._POL2	-4.46	1.83	WT(T)	under	-2.14	0.39	WT(T)	ns
Paenibacillus_naphthalenovorans	-1.96	1.83	WT(T)	under	0.75	0.14	WT(T)	ns
Lactobacillus_paracollinoides	-5.05	1.83	WT(T)	under	1.38	0.18	WT(T)	ns
Leptospirillum_ferrooxidans	-3.88	1.81	WT(T)	under	-0.71	0.01	WT(T)	ns
Shewanella_khirikhana	-2.24	1.78	WT(T)	under	-0.14	0.00	WT(T)	ns
Thermodesulfobacterium_geofontis	-1.47	1.78	WT(T)	under	-1.84	0.36	WT(T)	ns
Actinotignum_schaalii	-1.73	1.78	WT(T)	under	-0.37	0.00	WT(T)	ns
Sphingosinicella_microcystinivorans	-2.03	1.78	WT(T)	under	-0.35	0.14	WT(T)	ns
Parvibaculum_lavamentivorans	-4.22	1.77	WT(T)	under	-2.60	0.51	WT(T)	ns
Mucilaginibacter_sp._G2-14	-1.66	1.77	WT(T)	under	-0.80	0.06	WT(T)	ns
Burkholderia_pseudomallei	-3.71	1.77	WT(T)	under	1.34	0.03	WT(T)	ns
Caulobacter_flavus	-4.14	1.77	WT(T)	under	0.10	0.00	WT(T)	ns
Citrobacter_portucalensis	-3.40	1.76	WT(T)	under	-3.74	1.03	WT(T)	ns
Jeotgalibaca_arthritidis	-2.00	1.76	WT(T)	under	-1.32	0.01	WT(T)	ns
Pseudodesulfovibrio_sp._zrk46	-2.12	1.76	WT(T)	under	-0.31	0.00	WT(T)	ns
Corynebacterium_sp._4H37-19	-2.54	1.76	WT(T)	under	-0.27	0.00	WT(T)	ns
Neisseria_animaloris	-2.89	1.76	WT(T)	under	0.29	0.00	WT(T)	ns
Acidihalobacter_aeolianus	-3.18	1.76	WT(T)	under	-1.48	0.25	WT(T)	ns
Polaribacter_sp._KT25b	-1.94	1.75	WT(T)	under	-3.95	1.15	WT(T)	ns
Defluviitoga_tunisiensis	-2.07	1.75	WT(T)	under	-3.21	1.10	WT(T)	ns
Staphylococcus_lentus	-7.35	1.75	WT(T)	under	-7.80	1.10	WT(T)	ns
Mycoplasma_bovigenitalium	-3.31	1.75	WT(T)	under	-3.33	0.78	WT(T)	ns
Shewanella_sp._Scap07	-2.05	1.75	WT(T)	under	-2.26	0.61	WT(T)	ns
Pseudodesulfovibrio_aespoeensis	-2.07	1.75	WT(T)	under	-1.49	0.48	WT(T)	ns
Fusobacterium_hwasookii	-1.25	1.75	WT(T)	under	-2.62	0.42	WT(T)	ns
Lactobacillus_coryniformis	0.45	1.75	WT(T)	over	0.88	0.11	WT(T)	ns
Zobellia_galactanivorans	-0.82	1.75	WT(T)	under	1.71	0.18	WT(T)	ns
Brevibacillus_laterosporus	-0.85	1.75	WT(T)	under	0.56	0.13	WT(T)	ns
Flavobacterium_album	-1.23	1.75	WT(T)	under	0.46	0.00	WT(T)	ns
Halanaerobium_hydrogeniformans	-1.49	1.75	WT(T)	under	-1.03	0.27	WT(T)	ns
Paenibacillus_baekrokdamisoli	-1.53	1.75	WT(T)	under	0.29	0.00	WT(T)	ns
Alistipes_shahii	-1.88	1.75	WT(T)	under	0.50	0.01	WT(T)	ns
Aminipila_sp._CBA3637	-0.43	1.74	WT(T)	under	-0.87	0.95	WT(T)	ns
Bifidobacterium_angulatum	-1.42	1.74	WT(T)	under	0.06	0.00	WT(T)	ns
Mycobacteroides_abscessus	-1.63	1.74	WT(T)	under	-0.08	0.01	WT(T)	ns
Romboutsia_ilealis	-1.84	1.74	WT(T)	under	-0.68	0.11	WT(T)	ns
Oscillibacter_valericigenes	-1.54	1.73	WT(T)	under	-0.92	0.42	WT(T)	ns
Bifidobacterium_pseudocatenulatum	-0.32	1.73	WT(T)	under	-0.18	0.00	WT(T)	ns
Desulfitobacterium_hafniense	-0.69	1.73	WT(T)	under	-0.36	0.03	WT(T)	ns
Clostridium_sp._BNL1100	-1.03	1.73	WT(T)	under	-0.51	0.03	WT(T)	ns
Bacillus_cereus	-2.24	1.73	WT(T)	under	-0.98	0.11	WT(T)	ns
Lancefieldella_parvula	-2.47	1.73	WT(T)	under	-1.44	0.42	WT(T)	ns
Dysosmobacter_welbionis	-1.54	1.71	WT(T)	under	-0.86	0.46	WT(T)	ns
Desulfovibrio_hydrothermalis	-4.86	1.70	WT(T)	under	-0.97	0.00	WT(T)	ns
Peptoniphilus_harei	-1.85	1.69	WT(T)	under	-3.20	1.22	WT(T)	ns
Syntrophomonas_wolfei	-2.50	1.68	WT(T)	under	-0.70	0.11	WT(T)	ns
Paenibacillus_sp._FSL_R5-0345	-2.78	1.68	WT(T)	under	-2.23	0.27	WT(T)	ns
Tepiditoga_spiralis	-1.30	1.68	WT(T)	under	-0.51	0.03	WT(T)	ns
Halomonas_elongata	-2.39	1.68	WT(T)	under	-0.39	0.01	WT(T)	ns
Methylocaldum_marinum	-2.91	1.68	WT(T)	under	-1.18	0.25	WT(T)	ns

Corynebacterium_mustelae	-3.00	1.68	WT(T)	under	-1.10	0.11	WT(T)	ns
Mixta_calida	-1.87	1.67	WT(T)	under	0.53	0.00	WT(T)	ns
Niabella_soli	-2.11	1.67	WT(T)	under	-0.04	0.00	WT(T)	ns
Geobacter_bemidjiensis	-2.75	1.66	WT(T)	under	0.61	0.00	WT(T)	ns
Micropruina_glycogenica	-4.40	1.66	WT(T)	under	1.36	0.42	WT(T)	ns
Sphingomonas_alpina	-3.83	1.66	WT(T)	under	-0.36	0.02	WT(T)	ns
Rhizobium_jaguaris	-4.97	1.66	WT(T)	under	0.32	0.00	WT(T)	ns
Thermoanaerobacterium_aotearoense	-0.96	1.66	WT(T)	under	-1.11	0.52	WT(T)	ns
Melioribacter_roseus	-1.61	1.66	WT(T)	under	-1.15	0.48	WT(T)	ns
Brachyspira_pilosicoli	-3.89	1.66	WT(T)	under	-2.27	0.42	WT(T)	ns
Luteitalea_pratensis	-2.02	1.66	WT(T)	under	-0.20	0.01	WT(T)	ns
Chryseobacterium_indologenes	-2.26	1.66	WT(T)	under	0.23	0.00	WT(T)	ns
Rhizobium_grahamii	-2.37	1.66	WT(T)	under	-0.48	0.26	WT(T)	ns
Mesotoga_infera	-3.19	1.66	WT(T)	under	0.83	0.10	WT(T)	ns
Pseudomonas_syringae	-1.55	1.65	WT(T)	under	-1.16	0.58	WT(T)	ns
Cellulosilyticum_lentocellum	-1.46	1.65	WT(T)	under	-0.78	0.14	WT(T)	ns
Megamonas_hypermegale	-1.83	1.65	WT(T)	under	-1.58	1.10	WT(T)	ns
Alcaligenes_aquatilis	-1.30	1.65	WT(T)	under	-3.87	1.10	WT(T)	ns
Streptomyces_sp._CRXT-Y-14	-2.10	1.65	WT(T)	under	-2.57	0.77	WT(T)	ns
Campylobacter_iguaniorum	0.04	1.65	WT(T)	over	-0.21	0.00	WT(T)	ns
Acidipropionibacterium_virtanenii	-0.42	1.65	WT(T)	under	-0.03	0.00	WT(T)	ns
Sphaerochaeta_globosa	-1.58	1.65	WT(T)	under	-0.18	0.00	WT(T)	ns
Actinopolyspora_erythraea	-1.64	1.65	WT(T)	under	0.27	0.00	WT(T)	ns
Chryseobacterium_glaciei	-1.69	1.65	WT(T)	under	-0.54	0.02	WT(T)	ns
Bacillus_subtilis	-1.92	1.65	WT(T)	under	0.11	0.00	WT(T)	ns
Synechococcus_sp._CB0101	-1.92	1.65	WT(T)	under	-0.29	0.00	WT(T)	ns
Streptococcus_pyogenes	0.91	1.64	WT(T)	over	0.80	0.14	WT(T)	ns
Bacillus_thuringiensis	-1.44	1.64	WT(T)	under	-0.48	0.18	WT(T)	ns
Streptococcus_oralis	-4.71	1.64	WT(T)	under	-4.00	0.40	WT(T)	ns
Jannaschia_sp._CCS1	-2.61	1.62	WT(T)	under	-2.39	0.52	WT(T)	ns
Staphylococcus_simiae	-2.39	1.61	WT(T)	under	-3.41	0.74	WT(T)	ns
Alysiella_filiformis	-4.01	1.61	WT(T)	under	-2.96	0.68	WT(T)	ns
Lactobacillus_bifermentans	-4.03	1.61	WT(T)	under	-3.10	0.46	WT(T)	ns
Asticcacaulis_excentricus	-2.32	1.61	WT(T)	under	-0.54	0.01	WT(T)	ns
Leptotrichia_trevisanii	-2.92	1.61	WT(T)	under	-0.56	0.08	WT(T)	ns
Candidatus_Symbiobacter_mobilis	-3.74	1.61	WT(T)	under	1.20	0.14	WT(T)	ns
Lactobacillus_dextrinicus	-2.36	1.61	WT(T)	under	-2.71	0.83	WT(T)	ns
Peribacillus_asahii	-2.01	1.61	WT(T)	under	-0.09	0.00	WT(T)	ns
Thermobispora_bispora	-1.78	1.60	WT(T)	under	0.01	0.00	WT(T)	ns
Pseudopuniceibacterium_sp._HQ09	-1.94	1.60	WT(T)	under	-0.11	0.00	WT(T)	ns
Treponema_sp._Marseille-Q4132	-2.36	1.59	WT(T)	under	-1.22	0.27	WT(T)	ns
Thioalkalivibrio_sulfidiphilus	-2.09	1.59	WT(T)	under	-0.72	0.10	WT(T)	ns
Methylacidiphilum_fumariolicum	-4.68	1.59	WT(T)	under	-5.67	0.83	WT(T)	ns
Spiroplasma_corruscae	-5.00	1.59	WT(T)	under	-3.36	0.48	WT(T)	ns
Biomaibacter_acetigenes	-1.92	1.59	WT(T)	under	-1.25	0.42	WT(T)	ns
Bacteroides_coprosuis	-1.38	1.59	WT(T)	under	-0.17	0.01	WT(T)	ns
Clostridium_isatidis	-1.40	1.59	WT(T)	under	0.02	0.00	WT(T)	ns
Bradyrhizobium_sp._BTAi1	-3.57	1.59	WT(T)	under	-0.59	0.00	WT(T)	ns
Teredinibacter_turnerae	-4.19	1.59	WT(T)	under	2.29	0.15	WT(T)	ns
Corynebacterium_timonense	-4.86	1.59	WT(T)	under	-2.01	0.08	WT(T)	ns
Streptomyces_violaceusniger	-4.89	1.59	WT(T)	under	-0.13	0.00	WT(T)	ns
Vibrio_fluvialis	-4.91	1.59	WT(T)	under	0.16	0.00	WT(T)	ns
Anaerococcus_prevotii	-4.96	1.59	WT(T)	under	1.27	0.20	WT(T)	ns
Arcobacter_venerupis	-5.31	1.59	WT(T)	under	-2.08	0.24	WT(T)	ns
Maritalea_myrionectae	-5.55	1.59	WT(T)	under	-1.25	0.01	WT(T)	ns
Pedobacter_sp._PACM_27299	-5.64	1.59	WT(T)	under	-1.31	0.03	WT(T)	ns
Sandaracinus_amylolyticus	-6.19	1.59	WT(T)	under	-1.27	0.01	WT(T)	ns
Nonlabens_marinus	-6.30	1.59	WT(T)	under	-0.58	0.01	WT(T)	ns
Helicobacter_hepaticus	-11.38	1.59	WT(T)	under	-5.18	0.24	WT(T)	ns
Streptomyces_fulvissimus	-1.70	1.59	WT(T)	under	-0.54	0.42	WT(T)	ns
Bacillus_circulans	-1.90	1.59	WT(T)	under	-0.94	0.35	WT(T)	ns
Campylobacter_cuniculorum	-1.58	1.59	WT(T)	under	-0.42	0.01	WT(T)	ns
Skermanella_pratensis	-1.79	1.59	WT(T)	under	-0.29	0.16	WT(T)	ns
Kyrpidia_spormannii	-2.42	1.59	WT(T)	under	-0.12	0.00	WT(T)	ns
Salinibacter_ruber	-0.61	1.58	WT(T)	under	0.80	0.35	WT(T)	ns
Megamonas_funiformis	-2.30	1.57	WT(T)	under	-0.48	0.00	WT(T)	ns

Limnoglobus_roseus	-2.12	1.57	WT(T)	under	0.66	0.11	WT(T)	ns
Candidatus_Thiodictyon_syntrophicum	-1.93	1.56	WT(T)	under	-1.37	0.42	WT(T)	ns
Gramella_forsetii	-4.01	1.56	WT(T)	under	-3.06	0.60	WT(T)	ns
Nocardioides_daphniae	-3.15	1.56	WT(T)	under	1.13	0.21	WT(T)	ns
Cedecea_sp._FDAARGOS_727	-3.68	1.56	WT(T)	under	-1.02	0.00	WT(T)	ns
Suicoccus_acidiformans	-3.79	1.56	WT(T)	under	0.85	0.02	WT(T)	ns
Pseudoxanthomonas_spadix	-4.32	1.56	WT(T)	under	-1.58	0.05	WT(T)	ns
Streptococcus_pantholopis	-2.50	1.56	WT(T)	under	-1.25	0.08	WT(T)	ns
Mariniflexile_sp._TRM1-10	-3.29	1.55	WT(T)	under	-2.14	0.57	WT(T)	ns
Gracilibacillus_sp._SCU50	-4.08	1.55	WT(T)	under	-3.09	0.24	WT(T)	ns
Pseudoxanthomonas_suwonensis	-4.22	1.55	WT(T)	under	1.57	0.45	WT(T)	ns
Chryseobacterium_sp._StRB126	-1.90	1.54	WT(T)	under	-3.27	1.17	WT(T)	ns
Xylella_fastidiosa	-0.65	1.54	WT(T)	under	-1.06	0.46	WT(T)	ns
Cyanobium_gracile	-1.19	1.54	WT(T)	under	-0.79	0.02	WT(T)	ns
Bacillus_sp._X1(2014)	-1.96	1.53	WT(T)	under	-1.06	0.42	WT(T)	ns
Corynebacterium_glaucum	-2.44	1.53	WT(T)	under	-0.35	0.00	WT(T)	ns
Pusillimonas_sp._ye3	-1.71	1.52	WT(T)	under	-1.52	0.60	WT(T)	ns
Photobacterium_gaetbulicola	-3.83	1.52	WT(T)	under	-2.47	0.42	WT(T)	ns
Vulgatibacter_incomptus	-2.27	1.52	WT(T)	under	0.19	0.00	WT(T)	ns
Bradyrhizobium_sp._6(2017)	-3.62	1.52	WT(T)	under	-0.01	0.00	WT(T)	ns
Antarctobacter_heliothermus	-2.04	1.52	WT(T)	under	-0.88	0.24	WT(T)	ns
Phascolarctobacterium_faecium	-0.72	1.51	WT(T)	under	0.02	0.00	WT(T)	ns
Bifidobacterium_pullorum	-5.91	1.50	WT(T)	under	-1.91	0.01	WT(T)	ns
Clostridium_botulinum	-1.15	1.50	WT(T)	under	-0.88	0.60	WT(T)	ns
Comamonas_testosteroni	-2.83	1.50	WT(T)	under	-1.68	0.45	WT(T)	ns
Pseudonocardia_sp._HH130630-07	-2.06	1.50	WT(T)	under	-0.25	0.00	WT(T)	ns
Fusobacterium_gonidiaformans	-2.51	1.50	WT(T)	under	-0.19	0.00	WT(T)	ns
Exiguobacterium_sp._S3-2	-3.91	1.50	WT(T)	under	-4.98	1.13	WT(T)	ns
Bartonella_kosoyi	-3.20	1.50	WT(T)	under	-3.76	0.75	WT(T)	ns
Acaryochloris_marina	-1.79	1.50	WT(T)	under	-1.05	0.42	WT(T)	ns
Desulfosarcina_widdelii	-1.48	1.50	WT(T)	under	-0.15	0.00	WT(T)	ns
Blastomonas_sp._RAC04	-2.25	1.50	WT(T)	under	0.59	0.05	WT(T)	ns
Celeribacter_ethanolicus	-2.64	1.50	WT(T)	under	0.79	0.05	WT(T)	ns
Hydrogenophaga_sp._BPS33	-2.87	1.50	WT(T)	under	-0.53	0.01	WT(T)	ns
Terriglobus_saanensis	-2.89	1.50	WT(T)	under	-1.18	0.20	WT(T)	ns
Dokdonella_koreensis	-2.77	1.49	WT(T)	under	-2.51	0.74	WT(T)	ns
Streptococcus_gallolyticus	-1.63	1.49	WT(T)	under	-1.38	0.42	WT(T)	ns
Haliangium_ochraceum	-1.25	1.49	WT(T)	under	-1.20	0.35	WT(T)	ns
Christensenella_minuta	-2.19	1.49	WT(T)	under	-2.28	0.59	WT(T)	ns
Ruminiclostridium_sp._MA18	-1.28	1.48	WT(T)	under	-0.70	0.14	WT(T)	ns
Actinomarinicola_tropica	-2.97	1.47	WT(T)	under	-0.13	0.00	WT(T)	ns
Rummeliibacillus_stabekisii	-1.55	1.46	WT(T)	under	-0.02	0.00	WT(T)	ns
Sulfurospirillum_cavolei	-3.49	1.46	WT(T)	under	-0.78	0.18	WT(T)	ns
Corynebacterium_rouxii	-2.25	1.45	WT(T)	under	-0.11	0.01	WT(T)	ns
Oceanihabitans_sp._IOP_32	-2.72	1.44	WT(T)	under	-4.64	0.87	WT(T)	ns
Nonlabens_spongiae	-2.81	1.44	WT(T)	under	1.89	0.58	WT(T)	ns
Frondihabitans_sp._PAMC_28766	-3.89	1.44	WT(T)	under	1.34	0.34	WT(T)	ns
Amycolatopsis_methanolica	-1.57	1.44	WT(T)	under	-1.01	0.27	WT(T)	ns
Fusobacterium_varium	-1.52	1.44	WT(T)	under	-0.17	0.00	WT(T)	ns
Bosea_sp._RAC05	-1.81	1.44	WT(T)	under	-0.38	0.02	WT(T)	ns
Zobellella_denitrificans	-1.81	1.44	WT(T)	under	0.44	0.01	WT(T)	ns
Alkalihalobacillus_krulwichiae	-1.85	1.44	WT(T)	under	0.27	0.00	WT(T)	ns
Rhodanobacter_denitrificans	-1.96	1.44	WT(T)	under	-0.76	0.24	WT(T)	ns
Phytobacter_ursingii	-2.41	1.44	WT(T)	under	0.05	0.00	WT(T)	ns
Dechloromonas_sp._HYN0024	-2.59	1.44	WT(T)	under	1.41	0.18	WT(T)	ns
Komagataeibacter_hansenii	-2.62	1.44	WT(T)	under	0.60	0.00	WT(T)	ns
Sphingobium_sp._YG1	-2.86	1.44	WT(T)	under	1.12	0.00	WT(T)	ns
Finegoldia_magna	-1.23	1.43	WT(T)	under	-0.89	0.31	WT(T)	ns
Olsenella_sp._oral_taxon_807	-1.44	1.42	WT(T)	under	0.18	0.00	WT(T)	ns
Chryseobacterium_taihuense	-1.45	1.42	WT(T)	under	-0.95	0.19	WT(T)	ns
Acetoanaerobium_sticklandii	-2.10	1.42	WT(T)	under	-3.12	0.87	WT(T)	ns
Caloranaerobacter_azorensis	-1.39	1.42	WT(T)	under	-1.65	0.87	WT(T)	ns
Candidatus_Syntrophocurvum_alkaliphilum	-1.10	1.42	WT(T)	under	-1.49	0.61	WT(T)	ns
Halobacillus_halophilus	-3.62	1.42	WT(T)	under	-3.25	0.47	WT(T)	ns
Pseudomonas_coronafaciens	-2.00	1.42	WT(T)	under	-0.86	0.35	WT(T)	ns
Legionella_pneumophila	-2.60	1.42	WT(T)	under	-1.22	0.25	WT(T)	ns

Phycisphaera_mikurensis	-1.36	1.42	WT(T)	under	-1.43	0.25	WT(T)	ns
Chondromyces_crocatus	-1.45	1.42	WT(T)	under	-0.04	0.00	WT(T)	ns
Micavibrio_aeruginosavorus	-1.69	1.42	WT(T)	under	-0.63	0.08	WT(T)	ns
Desulfurispirillum_indicum	-1.70	1.42	WT(T)	under	-0.02	0.00	WT(T)	ns
Marinobacter_sp._LV10R510-11A	-2.08	1.42	WT(T)	under	-0.34	0.00	WT(T)	ns
Phenylobacterium_zucineum	-2.55	1.41	WT(T)	under	-3.10	0.77	WT(T)	ns
Candidatus_Phytoplasma_mali	-2.73	1.41	WT(T)	under	-4.16	0.68	WT(T)	ns
Massilia_sp._LPB0304	-1.96	1.41	WT(T)	under	-2.19	0.35	WT(T)	ns
Capnocytophaga_endodontalis	-2.49	1.41	WT(T)	under	0.05	0.27	WT(T)	ns
Akkermansia_muciniphila	4.58	1.41	WT(T)	over	0.31	0.02	WT(T)	ns
Enterococcus_casseliflavus	3.46	1.41	WT(T)	over	0.42	0.00	WT(T)	ns
Chlorobium_chlorochromatii	-1.43	1.41	WT(T)	under	2.34	0.10	WT(T)	ns
Candidatus_Desulforudis_audaxviator	-1.51	1.41	WT(T)	under	-0.46	0.11	WT(T)	ns
Lysinibacillus_sphaericus	-1.61	1.41	WT(T)	under	-1.60	0.11	WT(T)	ns
Mucilaginibacter_xinganensis	-1.69	1.41	WT(T)	under	0.64	0.01	WT(T)	ns
Desulfovibrio_carbinolicus	-2.14	1.41	WT(T)	under	-0.07	0.00	WT(T)	ns
Bacillus_sp._DSL-17	-2.21	1.41	WT(T)	under	-1.59	0.19	WT(T)	ns
Candidatus_Doolittlea_endobia	-2.25	1.41	WT(T)	under	0.55	0.00	WT(T)	ns
Defluviimonas_alba	-3.66	1.41	WT(T)	under	-0.89	0.08	WT(T)	ns
Herbaspirillum_sp._meg3	-4.38	1.41	WT(T)	under	-1.57	0.17	WT(T)	ns
Siansivirga_zeaxanthinifaciens	-4.82	1.41	WT(T)	under	0.29	0.00	WT(T)	ns
Gloeocapsa_sp._PCC_7428	-2.19	1.40	WT(T)	under	0.16	0.00	WT(T)	ns
Staphylococcus_kloosii	-2.17	1.40	WT(T)	under	-3.64	1.10	WT(T)	ns
Aggregatibacter_actinomycetemcomitans	-3.04	1.40	WT(T)	under	-3.40	0.51	WT(T)	ns
Thiospirochaeta_perfilievii	-1.12	1.37	WT(T)	under	-2.15	1.10	WT(T)	ns
Fictibacillus_arsenicus	-1.36	1.37	WT(T)	under	-0.78	0.15	WT(T)	ns
Rhodococcus_fascians	-1.52	1.37	WT(T)	under	-2.17	1.05	WT(T)	ns
Staphylococcus_cohnii	-1.04	1.37	WT(T)	under	-1.35	0.46	WT(T)	ns
Pontibacter_akesuensis	-1.78	1.37	WT(T)	under	1.26	0.24	WT(T)	ns
Ilyobacter_polytropus	-0.85	1.37	WT(T)	under	0.08	0.00	WT(T)	ns
Sphingomonas_sp._NBWT7	-2.08	1.37	WT(T)	under	0.12	0.00	WT(T)	ns
Paraburkholderia_sp._7MH5	-2.17	1.37	WT(T)	under	-0.48	0.15	WT(T)	ns
Amphibacillus_xylanus	-0.69	1.36	WT(T)	under	0.09	0.00	WT(T)	ns
Gemmatimonas_sp._TET16	-3.37	1.36	WT(T)	under	0.40	0.00	WT(T)	ns
Neisseria_sp._KEM232	-2.46	1.35	WT(T)	under	-2.02	0.24	WT(T)	ns
Tessaracoccus_flavus	0.02	1.35	WT(T)	over	-0.81	0.14	WT(T)	ns
Arcticibacterium_luteifluviistationis	-2.89	1.35	WT(T)	under	-0.39	0.01	WT(T)	ns
Enterococcus_saigonensis	-1.13	1.35	WT(T)	under	-0.03	0.00	WT(T)	ns
Jonquetella_anthropi	-1.81	1.35	WT(T)	under	-0.39	0.00	WT(T)	ns
Arthrobacter_sp._QXT-31	-1.48	1.34	WT(T)	under	0.97	0.01	WT(T)	ns
Aggregatibacter_aphrophilus	-2.80	1.34	WT(T)	under	-1.22	0.11	WT(T)	ns
Lactobacillus_manihotivorans	-0.40	1.34	WT(T)	under	-0.56	0.00	WT(T)	ns
Desulfobulbus_oralis	-1.98	1.34	WT(T)	under	0.03	0.00	WT(T)	ns
Ligilactobacillus_salivarius	-1.51	1.33	WT(T)	under	-0.46	0.01	WT(T)	ns
Carboxydothermus_hydrogenoformans	-1.46	1.33	WT(T)	under	-1.38	0.74	WT(T)	ns
Geobacillus_stearothermophilus	-1.08	1.32	WT(T)	under	-0.33	0.01	WT(T)	ns
Euzebya_sp._DY32-46	0.93	1.31	WT(T)	over	1.40	1.10	WT(T)	ns
Lactobacillus_pentosus	-1.74	1.31	WT(T)	under	-1.12	0.35	WT(T)	ns
Bacillus_safensis	-4.88	1.30	WT(T)	under	-4.71	0.95	WT(T)	ns
Corynebacterium_minutissimum	-0.98	1.30	WT(T)	under	-1.39	0.77	WT(T)	ns
Verrucosispora_sp._NA02020	-2.18	1.30	WT(T)	under	-2.63	0.74	WT(T)	ns
Caldicellulosiruptor_owensensis	-0.36	1.30	WT(T)	under	-1.66	0.36	WT(T)	ns
Bifidobacterium_bifidum	-0.65	1.30	WT(T)	under	-0.71	0.11	WT(T)	ns
Zunongwangia_profunda	-1.10	1.30	WT(T)	under	0.11	0.00	WT(T)	ns
Granulicella_mallensis	-1.37	1.30	WT(T)	under	0.11	0.00	WT(T)	ns
Aquimarina_sp._BL5	-4.79	1.22	WT(T)	ns	2.79	1.43	WT(T)	over
Aquaspirillum_sp._LM1	-0.11	1.21	WT(T)	ns	-2.22	1.43	WT(T)	under
Lactobacillus_iners	-1.70	0.87	WT(T)	ns	-2.30	1.38	WT(T)	under
Selenomonas_sp._oral_taxon_126	-1.03	0.81	WT(T)	ns	-1.42	1.48	WT(T)	under
Lactobacillus_helveticus	-4.36	0.70	WT(T)	ns	-8.25	1.48	WT(T)	under
Lactobacillus_gasseri	-3.33	0.69	WT(T)	ns	-7.69	1.48	WT(T)	under
Lactobacillus_crispatus	-1.69	0.44	WT(T)	ns	-4.96	1.48	WT(T)	under
Terriglobus_roseus	-0.07	0.41	WT(T)	ns	2.93	1.48	WT(T)	over
Pantoea_rwandensis	NA	0.36	WT(T)	ns	3.68	1.39	WT(T)	over
Lactobacillus_johnsonii	1.28	0.17	WT(T)	ns	-2.59	1.48	WT(T)	under
Labrenzia_sp._THAF82	2.89	0.16	WT(T)	ns	2.97	1.48	WT(T)	over

Lactobacillus_mucosae	1.18	0.05	WT(T)	ns	-2.90	1.43	WT(T)	under
Nitrosospira_briensis	-0.56	0.04	WT(T)	ns	4.00	1.48	WT(T)	over
Bacteroides_sp._HF-5141	2.63	0.04	WT(T)	ns	1.76	1.44	WT(T)	over
Bacteroides_ovatus	2.33	0.04	WT(T)	ns	1.38	1.44	WT(T)	over
Bacteroides_sp._CBA7301	2.85	0.04	WT(T)	ns	1.86	1.43	WT(T)	over
Bacteroides_caccae	2.61	0.04	WT(T)	ns	1.73	1.43	WT(T)	over
Bacteroides_caecimuris	2.50	0.04	WT(T)	ns	1.62	1.43	WT(T)	over
Bacteroides_sp._M10	2.49	0.04	WT(T)	ns	1.59	1.43	WT(T)	over
Bacteroides_xylanisolvens	2.50	0.04	WT(T)	ns	1.58	1.39	WT(T)	over
Bacteroides_cellulosilyticus	-3.09	4.82	Per(T)	under	-1.38	3.17	Per(T)	under
Fibrobacter_succinogenes	-3.11	4.82	Per(T)	under	-1.09	2.17	Per(T)	under
Arabia_massiliensis	-3.13	4.82	Per(T)	under	1.08	3.26	Per(T)	over
Prevotella_denticola	-3.23	4.82	Per(T)	under	-1.35	2.74	Per(T)	under
Prevotella_enoeca	-3.33	4.82	Per(T)	under	-1.99	3.17	Per(T)	under
Prevotella_intermedia	-3.39	4.82	Per(T)	under	-2.34	4.24	Per(T)	under
Adlercreutzia_equolifaciens	-3.45	4.82	Per(T)	under	0.99	2.81	Per(T)	over
Flavihumibacter_sp._SB-02	-6.52	4.82	Per(T)	under	2.06	4.51	Per(T)	over
Chryseobacterium_indologenes	-3.99	4.82	Per(T)	under	-0.54	0.30	Per(T)	ns
Cyanobacterium_aponinum	-4.05	4.82	Per(T)	under	-0.35	0.73	Per(T)	ns
Prevotella_fusca	-4.20	4.82	Per(T)	under	-2.91	4.51	Per(T)	under
Bacteroides_sp._HF-162	-4.33	4.82	Per(T)	under	-3.25	4.51	Per(T)	under
Pedobacter_heparinus	-4.42	4.82	Per(T)	under	2.63	0.43	Per(T)	ns
Paludibacter_propionicigenes	-4.42	4.82	Per(T)	under	0.40	0.15	Per(T)	ns
Moorea_producens	-4.58	4.82	Per(T)	under	-0.90	0.93	Per(T)	ns
Adlercreutzia_sp._8CFCBH1	-4.84	4.82	Per(T)	under	0.99	2.63	Per(T)	over
Bacteroides_sp._CACC_737	-5.05	4.82	Per(T)	under	-5.03	4.51	Per(T)	under
Desulfovibrio_fairfieldensis	-4.98	4.82	Per(T)	under	-2.11	1.66	Per(T)	under
Sphingomonas_sp._CL5.1	NA	4.82	Per(T)	ns	-5.10	4.51	Per(T)	under
Scandinavium_goeteborgense	-4.98	4.82	Per(T)	under	2.92	0.35	Per(T)	ns
Phocaeicola_dorei	-5.86	4.82	Per(T)	under	-5.77	4.51	Per(T)	under
Bacteroides_uniformis	-5.21	4.82	Per(T)	under	-5.82	4.51	Per(T)	under
Helicobacter_pylori	-5.11	4.82	Per(T)	under	-2.60	2.83	Per(T)	under
Phocaeicola_vulgatus	-6.98	4.82	Per(T)	under	-6.32	4.51	Per(T)	under
Helicobacter_bilis	NA	4.82	Per(T)	ns	-6.75	4.51	Per(T)	under
Acetilactobacillus_jinshanensis	-5.38	4.82	Per(T)	under	-0.14	0.01	Per(T)	ns
Helicobacter_apodemus	-8.27	4.82	Per(T)	under	-6.91	4.51	Per(T)	under
Draconibacterium_orientale	-5.41	4.82	Per(T)	under	0.54	0.37	Per(T)	ns
Bacteroides_sp._A1C1	-6.49	4.82	Per(T)	under	-6.92	4.51	Per(T)	under
Sphingobacterium_lactis	-5.52	4.82	Per(T)	under	-0.97	0.87	Per(T)	ns
Streptomyces_harbinensis	-5.78	4.82	Per(T)	under	-7.21	4.51	Per(T)	under
Helicobacter_hepaticus	-8.96	4.82	Per(T)	under	-9.09	4.51	Per(T)	under
Helicobacter_cinaedi	-8.17	4.82	Per(T)	under	-10.30	4.51	Per(T)	under
Calditerrivibrio_nitroreducens	-6.34	4.82	Per(T)	under	-4.16	3.17	Per(T)	under
Helicobacter_typhlonius	-9.36	4.82	Per(T)	under	-16.73	4.51	Per(T)	under
Bifidobacterium_bifidum	-6.34	4.82	Per(T)	under	-0.42	0.09	Per(T)	ns
Capnocytophaga_sp._oral_taxon_864	-6.52	4.82	Per(T)	under	-1.26	0.87	Per(T)	ns
Pelobacter_propionicus	-6.87	4.82	Per(T)	under	3.04	0.61	Per(T)	ns
Helicobacter_cholecystus	NA	4.82	Per(T)	ns	-4.69	3.79	Per(T)	under
Candidatus_Saccharimonas_aalborgensis	-6.94	4.82	Per(T)	under	0.29	0.80	Per(T)	ns
Acholeplasma_brassicae	-7.01	4.82	Per(T)	under	-0.34	0.08	Per(T)	ns
Prevotella_scopos	-7.42	4.82	Per(T)	under	-1.68	3.17	Per(T)	under
Candidatus_Phytoplasma_australiense	-7.44	4.82	Per(T)	under	-0.21	0.02	Per(T)	ns
Helicobacter_canis	-7.68	4.82	Per(T)	under	NA	4.51	Per(T)	ns
Candidatus_Nanosynbacter_lyticus	-7.79	4.82	Per(T)	under	0.15	0.49	Per(T)	ns
Algoriphagus_sanaruensis	-8.93	4.82	Per(T)	under	-1.47	1.96	Per(T)	under
Candidatus_Protochlamydia_naegleriophila	NA	4.82	Per(T)	ns	-3.60	3.18	Per(T)	under
Rufibacter_sp._DG31D	NA	4.82	Per(T)	ns	2.93	3.17	Per(T)	over
Campylobacter_pinnipediorum	NA	4.82	Per(T)	ns	-4.07	3.17	Per(T)	under
Streptomyces_chartreusis	NA	4.82	Per(T)	ns	-2.49	2.46	Per(T)	under
Arcobacter_cryaerophilus	NA	4.82	Per(T)	ns	-1.44	1.58	Per(T)	under
Bacteroides_zoogleoformans	-3.48	4.59	Per(T)	under	-2.44	4.51	Per(T)	under
Alkaliphilus_metalliredigens	-2.95	4.59	Per(T)	under	0.14	0.01	Per(T)	ns
Petrimonas_mucosa	-3.19	4.59	Per(T)	under	-0.57	0.87	Per(T)	ns
Lactobacillus_johnsonii	-3.65	4.59	Per(T)	under	-0.08	0.01	Per(T)	ns
Hymenobacter_swuensis	-3.92	4.59	Per(T)	under	1.94	2.73	Per(T)	over
Winogradskyella_forsetii	-3.98	4.59	Per(T)	under	-0.89	0.65	Per(T)	ns

Spirosoma_sp._CJU-R4	-4.80	4.59	Per(T)	under	3.08	0.61	Per(T)	ns
Alkalitalea_saponilacus	-5.02	4.59	Per(T)	under	0.51	0.18	Per(T)	ns
Flavisolibacter_tropicus	-5.80	4.59	Per(T)	under	-2.64	3.05	Per(T)	under
Brachyspira_murdochii	-7.58	4.59	Per(T)	under	-4.73	3.17	Per(T)	under
Acholeplasma_palmae	-7.94	4.59	Per(T)	under	-0.43	0.11	Per(T)	ns
Phocaeicola_salanitronis	-2.36	4.33	Per(T)	under	-1.43	2.83	Per(T)	under
Parolsenella_catena	-2.97	4.33	Per(T)	under	2.85	2.83	Per(T)	over
Bifidobacterium_animalis	-3.64	4.33	Per(T)	under	-0.05	0.01	Per(T)	ns
Candidatus_Izimaplasma_sp._HR1	-4.64	4.33	Per(T)	under	-0.45	0.24	Per(T)	ns
Leptotrichia_trevisanii	-4.71	4.33	Per(T)	under	-1.05	1.16	Per(T)	ns
Sulfurimonas_autotrophica	-5.57	4.33	Per(T)	under	NA	4.51	Per(T)	ns
Seonamhaeicola_sp._S2-3	-5.93	4.33	Per(T)	under	-2.78	2.47	Per(T)	under
Capnocytophaga_canimorsus	-6.04	4.33	Per(T)	under	-3.36	2.42	Per(T)	under
Polynucleobacter_necessarius	-4.40	4.17	Per(T)	under	0.34	0.01	Per(T)	ns
Deferribacter_desulfuricans	-4.87	4.17	Per(T)	under	-2.07	1.01	Per(T)	ns
Bacteroides_heparinolyticus	-2.85	4.12	Per(T)	under	-2.60	4.51	Per(T)	under
Paraprevotella_xylaniphila	-2.40	4.12	Per(T)	under	-0.39	0.29	Per(T)	ns
Desulfovibrio_piger	-3.25	4.12	Per(T)	under	-0.06	0.04	Per(T)	ns
Mycoplasma_anseris	-7.50	4.12	Per(T)	under	-1.72	1.26	Per(T)	ns
Labilibaculum_antarcticum	-3.74	4.07	Per(T)	under	-1.06	0.91	Per(T)	ns
Sphingobacterium_sp._CZ-2	-4.03	4.02	Per(T)	under	0.77	0.61	Per(T)	ns
Muricauda_ruestringensis	-5.56	3.92	Per(T)	under	2.63	3.41	Per(T)	over
Desulfocurvibacter_africanus	-4.83	3.92	Per(T)	under	-3.62	3.05	Per(T)	under
Bacteroides_coprosuis	-2.94	3.92	Per(T)	under	-0.75	1.82	Per(T)	under
Desulfovibrio_vulgaris	-3.65	3.92	Per(T)	under	-0.06	0.01	Per(T)	ns
Cedecea_neteri	-5.06	3.92	Per(T)	under	0.30	0.04	Per(T)	ns
Pedobacter_sp._KBS0701	NA	3.92	Per(T)	ns	-3.54	2.90	Per(T)	under
Colwellia_sp._PAMC_21821	-5.70	3.92	Per(T)	under	-3.00	2.39	Per(T)	under
Candidatus_Doolittlea_endobia	-6.19	3.92	Per(T)	under	-0.30	0.13	Per(T)	ns
Desulfurella_acetivorans	NA	3.92	Per(T)	ns	-3.54	2.74	Per(T)	under
Helicobacter_pullorum	NA	3.92	Per(T)	ns	-3.05	2.56	Per(T)	under
Candidatus_Nitrosoglobus_terrae	NA	3.92	Per(T)	ns	-2.88	2.41	Per(T)	under
Aerosticca_soli	NA	3.92	Per(T)	ns	-2.69	2.13	Per(T)	under
Helicobacter_acinonychis	NA	3.92	Per(T)	ns	-2.86	2.04	Per(T)	under
Shewanella_pealeana	NA	3.92	Per(T)	ns	-2.16	1.52	Per(T)	under
Acetobacter_pasteurianus	NA	3.92	Per(T)	ns	-1.02	1.40	Per(T)	under
Haematospirillum_jordaniae	-3.81	3.87	Per(T)	under	-0.69	0.52	Per(T)	ns
Prevotella_ruminicola	-2.91	3.80	Per(T)	under	-2.81	4.24	Per(T)	under
Microbulbifer_aggregans	-3.65	3.79	Per(T)	under	0.04	0.07	Per(T)	ns
Anaerolinea_sp._'rifampicinis'	-6.00	3.79	Per(T)	under	0.65	0.85	Per(T)	ns
Gallaecimonas_mangrovi	-6.28	3.79	Per(T)	under	1.81	0.39	Per(T)	ns
Acinetobacter_radioresistens	-5.40	3.67	Per(T)	under	0.30	0.01	Per(T)	ns
Hymenobacter_sp._NBH84	-5.67	3.67	Per(T)	under	1.46	2.83	Per(T)	over
Sphingobacterium_sp._ML3W	-5.70	3.67	Per(T)	under	-1.57	1.35	Per(T)	under
Mycoplasma_bovirhinis	-6.84	3.67	Per(T)	under	-1.39	0.84	Per(T)	ns
Desulfovibrio_salexigens	-3.37	3.64	Per(T)	under	0.82	0.19	Per(T)	ns
Butyricimonas_faecalis	-2.42	3.63	Per(T)	under	-1.11	2.62	Per(T)	under
Campylobacter_upsaliensis	-4.62	3.60	Per(T)	under	-4.24	3.11	Per(T)	under
Thauera_aromatica	-4.45	3.60	Per(T)	under	-1.04	1.30	Per(T)	ns
Gluconobacter_oxydans	-3.49	3.58	Per(T)	under	0.30	0.08	Per(T)	ns
Lactobacillus_coryniformis	-4.95	3.58	Per(T)	under	-0.22	0.01	Per(T)	ns
Winogradskyella_sediminis	-3.40	3.55	Per(T)	under	0.42	0.04	Per(T)	ns
Thermomonas_brevis	-4.19	3.54	Per(T)	under	-3.37	2.76	Per(T)	under
Gemella_sanguinis	-5.31	3.54	Per(T)	under	0.11	0.01	Per(T)	ns
Mycoplasma_bovis	-5.55	3.54	Per(T)	under	-0.41	0.20	Per(T)	ns
Crateriforma_conspicua	-5.72	3.54	Per(T)	under	-3.30	2.31	Per(T)	under
Flavobacterium_alkalisoli	-6.03	3.54	Per(T)	under	1.37	0.30	Per(T)	ns
Clostridium_tyrobutyricum	-3.64	3.50	Per(T)	under	-0.93	0.40	Per(T)	ns
Desulfosporosinus_youngiae	-2.79	3.50	Per(T)	under	-2.35	2.91	Per(T)	under
Mannheimia_varigena	6.25	3.45	Per(T)	over	-1.00	0.04	Per(T)	ns
Paraflavitalea_soli	-3.63	3.45	Per(T)	under	2.33	1.35	Per(T)	over
Francisella_sp._Scap27	-5.24	3.43	Per(T)	under	-1.27	1.00	Per(T)	ns
Campylobacter_fetus	-4.64	3.40	Per(T)	under	-0.98	0.71	Per(T)	ns
Neobacillus_mesonae	-3.23	3.37	Per(T)	under	-0.66	0.77	Per(T)	ns
Prevotella_oris	-2.50	3.35	Per(T)	under	-2.37	3.96	Per(T)	under
Bacillus_cereus	-2.22	3.35	Per(T)	under	-1.16	1.18	Per(T)	ns

Blattabacterium_cuenoti	-2.35	3.35	Per(T)	under	0.63	0.04	Per(T)	ns
Saccharophagus_degradans	-4.66	3.35	Per(T)	under	1.42	0.75	Per(T)	ns
Dysgonomonas_sp._HDW5B	-4.56	3.32	Per(T)	under	-0.23	0.04	Per(T)	ns
Azoarcus_communis	-3.92	3.29	Per(T)	under	-3.07	2.63	Per(T)	under
Chlorobaculum_limnaeum	-3.25	3.29	Per(T)	under	1.63	0.71	Per(T)	ns
Polaribacter_sp._ALD11	-4.02	3.29	Per(T)	under	-0.98	0.87	Per(T)	ns
Pontibacter_akesuensis	-4.55	3.29	Per(T)	under	0.70	0.06	Per(T)	ns
Pseudarthrobacter_chlorophenolicus	-4.79	3.28	Per(T)	under	0.78	0.04	Per(T)	ns
Corynebacterium_sp._sy039	NA	3.25	Per(T)	ns	-6.37	2.90	Per(T)	under
Arcobacter_defluvii	NA	3.25	Per(T)	ns	-4.47	2.60	Per(T)	under
Porphyromonas_gingivalis	-1.24	3.25	Per(T)	under	-0.26	0.18	Per(T)	ns
Bifidobacterium_adolescentis	-2.37	3.25	Per(T)	under	0.58	0.01	Per(T)	ns
Clostridium_estertheticum	-2.77	3.25	Per(T)	under	-0.27	0.04	Per(T)	ns
Fusobacterium_nucleatum	-3.60	3.25	Per(T)	under	-0.26	0.01	Per(T)	ns
Brachyspira_intermedia	-6.20	3.25	Per(T)	under	-2.98	1.47	Per(T)	under
Chryseobacterium_sp._StRB126	-6.41	3.25	Per(T)	under	-0.69	0.28	Per(T)	ns
Halorhodospira_halochloris	NA	3.25	Per(T)	ns	-3.07	2.25	Per(T)	under
Bartonella_henselae	NA	3.25	Per(T)	ns	-4.08	2.20	Per(T)	under
Helicobacter_cetorum	NA	3.25	Per(T)	ns	-3.02	1.58	Per(T)	under
Fibrella_sp._ES10-3-2-2	NA	3.25	Per(T)	ns	2.98	1.53	Per(T)	over
Winogradskyella_sp._PC-19	NA	3.25	Per(T)	ns	-2.17	1.52	Per(T)	under
Salinimonas_sp._KX18D6	NA	3.25	Per(T)	ns	-2.33	1.40	Per(T)	under
Mycoplasma_mycoides	NA	3.25	Per(T)	ns	-2.31	1.35	Per(T)	under
Spirosoma_aerolatum	-4.22	3.24	Per(T)	under	3.05	0.39	Per(T)	ns
Fimbriimonas_ginsengisoli	-3.88	3.24	Per(T)	under	1.02	0.60	Per(T)	ns
Treponema_pedis	-2.76	3.19	Per(T)	under	-2.29	2.83	Per(T)	under
Stackebrandtia_nassauensis	-3.00	3.19	Per(T)	under	-0.41	0.58	Per(T)	ns
Prevotella_sp._oral_taxon_299	-2.54	3.16	Per(T)	under	-1.30	1.58	Per(T)	under
Denitrobacterium_detoxificans	-2.04	3.14	Per(T)	under	0.79	1.35	Per(T)	over
Chlorobium_phaeobacteroides	-1.85	3.09	Per(T)	under	-0.34	0.01	Per(T)	ns
Brachyspira_pilosicoli	-4.44	3.09	Per(T)	under	0.88	0.22	Per(T)	ns
Desulfopila_sp._IMCC35005	-2.30	3.07	Per(T)	under	0.54	0.36	Per(T)	ns
Paenibacillus_baekrokdamisoli	-2.17	3.03	Per(T)	under	-0.86	1.03	Per(T)	ns
Mixta_theicola	-4.98	3.03	Per(T)	under	1.07	0.37	Per(T)	ns
Bacteroides_fragilis	-1.86	3.01	Per(T)	under	-1.11	3.41	Per(T)	under
Gordonibacter_pamelaeae	-2.45	3.01	Per(T)	under	0.91	1.90	Per(T)	over
Desulfovibrio_sp._86	-3.22	3.01	Per(T)	under	0.04	0.01	Per(T)	ns
Desulfovibrio_alaskensis	-4.21	3.01	Per(T)	under	0.07	0.01	Per(T)	ns
Sphaerotilus_natans	-4.73	3.01	Per(T)	under	NA	2.90	Per(T)	ns
Campylobacter_helveticus	-6.58	3.01	Per(T)	under	-2.61	1.47	Per(T)	under
Capnocytophaga_sputigena	-3.42	2.98	Per(T)	under	-1.09	0.91	Per(T)	ns
Duncaniella_sp._B8	-0.96	2.93	Per(T)	under	1.79	0.35	Per(T)	ns
Gloeomargarita_lithophora	-2.45	2.91	Per(T)	under	-0.16	0.27	Per(T)	ns
Bifidobacterium_choerinum	-2.53	2.91	Per(T)	under	0.36	0.18	Per(T)	ns
Candidatus_Desulfovibrio_trichonymphae	-3.25	2.91	Per(T)	under	-0.25	0.08	Per(T)	ns
Leptotrichia_sp._oral_taxon_212	-3.55	2.91	Per(T)	under	-0.72	0.25	Per(T)	ns
Brevibacillus_laterosporus	-3.91	2.91	Per(T)	under	-0.25	0.16	Per(T)	ns
Bacteroides_helcogenes	-2.30	2.89	Per(T)	under	-2.79	4.51	Per(T)	under
Bifidobacterium_pseudolongum	-2.56	2.89	Per(T)	under	0.35	0.18	Per(T)	ns
Bacillus_sp._THAF10	-3.42	2.88	Per(T)	under	-1.37	0.35	Per(T)	ns
Tenacibaculum_jejuense	-4.65	2.88	Per(T)	under	-0.63	0.33	Per(T)	ns
Lacibacter_sp._S13-6-6	-5.00	2.88	Per(T)	under	1.21	0.04	Per(T)	ns
Nautilia_sp._PV-1	-5.45	2.88	Per(T)	under	-0.32	0.25	Per(T)	ns
Thiosulfatimonas_sediminis	-3.40	2.84	Per(T)	under	0.69	0.45	Per(T)	ns
Caldisericum_exile	-4.02	2.84	Per(T)	under	-1.20	0.51	Per(T)	ns
Lawsonia_intracellularis	-2.77	2.83	Per(T)	under	0.64	0.06	Per(T)	ns
Clostridium_sp._DL-VIII	-2.33	2.81	Per(T)	under	0.23	0.01	Per(T)	ns
Bacteroides_xylanisolvens	-0.65	2.80	Per(T)	under	-0.30	0.07	Per(T)	ns
Bacteroides_intestinalis	-1.20	2.79	Per(T)	under	-2.87	4.51	Per(T)	under
Bacteroides_caecimuris	0.29	2.79	Per(T)	over	-0.07	0.07	Per(T)	ns
Bacteroides_sp._HF-5141	0.08	2.79	Per(T)	over	0.15	0.07	Per(T)	ns
Bacteroides_sp._M10	-0.28	2.79	Per(T)	under	-0.20	0.07	Per(T)	ns
Bacteroides_ovatus	-0.46	2.79	Per(T)	under	-0.07	0.07	Per(T)	ns
Bacteroides_thetaiotaomicron	-0.63	2.79	Per(T)	under	-0.37	0.13	Per(T)	ns
Bacteroides_sp._HF-5287	-1.37	2.79	Per(T)	under	-0.32	0.18	Per(T)	ns
Bacteroides_caccae	-1.48	2.79	Per(T)	under	0.21	0.08	Per(T)	ns

Bacteroides_sp._CBA7301	-1.76	2.79	Per(T)	under	0.31	0.07	Per(T)	ns
Draconibacterium_sp._M1	-2.62	2.79	Per(T)	under	-0.13	0.06	Per(T)	ns
Breoghania_sp._L-A4	-4.13	2.79	Per(T)	under	3.51	0.85	Per(T)	ns
Rhizobium_jaguaris	-4.79	2.79	Per(T)	under	1.07	0.04	Per(T)	ns
Formosa_sp._L2A11	-3.38	2.76	Per(T)	under	-0.93	0.27	Per(T)	ns
Cytophaga_hutchinsonii	-2.63	2.75	Per(T)	under	0.66	0.01	Per(T)	ns
Mycoplasma_sp._NEAQ87857	-2.35	2.73	Per(T)	under	-0.23	0.66	Per(T)	ns
Listeria_ivanovii	-2.87	2.73	Per(T)	under	-1.18	0.15	Per(T)	ns
Desulfopila_sp._IMCC35004	-3.46	2.73	Per(T)	under	1.57	0.11	Per(T)	ns
Phoenicibacter_congonensis	-3.64	2.72	Per(T)	under	-0.23	0.01	Per(T)	ns
Spirosoma_sp._PL0136	NA	2.68	Per(T)	ns	-5.57	2.39	Per(T)	under
Neisseria_sp._KEM232	-4.39	2.68	Per(T)	under	-3.24	2.17	Per(T)	under
Glaesserella_parasuis	11.77	2.68	Per(T)	over	3.42	0.37	Per(T)	ns
Mycoplasmopsis_californica	-3.70	2.68	Per(T)	under	-2.55	1.94	Per(T)	under
Desulfitobacterium_dichloroeliminans	-2.89	2.68	Per(T)	under	-0.85	0.53	Per(T)	ns
Bradyrhizobium_cosmicum	-5.98	2.68	Per(T)	under	-2.81	1.58	Per(T)	under
Chlamydia_sp._2742-308	NA	2.68	Per(T)	ns	-3.03	1.65	Per(T)	under
Mycobacterium_sp._JS623	NA	2.68	Per(T)	ns	-3.18	1.62	Per(T)	under
Persephonella_marina	NA	2.68	Per(T)	ns	-2.73	1.47	Per(T)	under
Clostridium_formicaceticum	-3.61	2.65	Per(T)	under	-0.91	0.35	Per(T)	ns
Sphingobacterium_sp._B29	-4.22	2.64	Per(T)	under	-5.06	2.60	Per(T)	under
Bacteroides_sp._PHL_2737	-2.04	2.64	Per(T)	under	-1.23	1.29	Per(T)	ns
Chlorobium_chlorochromatii	-4.01	2.64	Per(T)	under	-0.76	0.33	Per(T)	ns
Erysipelothrix_inopinata	-4.47	2.64	Per(T)	under	-0.15	0.13	Per(T)	ns
Bifidobacterium_catenulatum	-2.06	2.64	Per(T)	under	-0.39	0.01	Per(T)	ns
Gramella_forsetii	-1.55	2.60	Per(T)	under	1.33	0.34	Per(T)	ns
Streptobacillus_moniliformis	-2.78	2.58	Per(T)	under	-0.20	0.22	Per(T)	ns
Geovibrio_thiophilus	-2.42	2.57	Per(T)	under	-1.25	0.72	Per(T)	ns
Gracilibacillus_sp._SCU50	-4.29	2.57	Per(T)	under	0.92	0.07	Per(T)	ns
Nitratiruptor_sp._SB155-2	-3.55	2.53	Per(T)	under	-4.76	2.73	Per(T)	under
Pedobacter_sp._JBR3-12	-0.71	2.53	Per(T)	under	-0.48	0.18	Per(T)	ns
Clostridium_cellulovorans	-2.24	2.53	Per(T)	under	0.37	0.01	Per(T)	ns
Syntrophomonas_wolfei	-3.20	2.52	Per(T)	under	-0.89	0.14	Per(T)	ns
Sulfurimonas_sp._NW10	-4.06	2.51	Per(T)	under	-2.90	1.38	Per(T)	under
Alistipes_indistinctus	-1.37	2.50	Per(T)	under	0.57	0.35	Per(T)	ns
Clostridium_argentinense	-3.11	2.48	Per(T)	under	-0.85	0.30	Per(T)	ns
Candidatus_Koribacter_versatilis	-4.60	2.46	Per(T)	under	-4.86	2.20	Per(T)	under
Paraglaciecola_psychrophila	-5.54	2.46	Per(T)	under	-5.61	2.20	Per(T)	under
Halocella_sp._SP3-1	-2.72	2.46	Per(T)	under	-0.13	0.01	Per(T)	ns
Flavobacterium_nackdongense	-4.34	2.46	Per(T)	under	0.37	0.18	Per(T)	ns
Staphylococcus_chromogenes	-3.79	2.42	Per(T)	under	-2.25	1.22	Per(T)	ns
Sphingobacterium_daejeonense	-3.81	2.42	Per(T)	under	-2.99	1.73	Per(T)	under
Lactobacillus_futsaii	-2.24	2.39	Per(T)	under	-1.39	0.76	Per(T)	ns
Acaryochloris_marina	-2.49	2.38	Per(T)	under	-0.93	0.60	Per(T)	ns
Runella_sp._SP2	-2.64	2.38	Per(T)	under	0.24	0.04	Per(T)	ns
Romboutsia_hominis	-0.61	2.37	Per(T)	under	-1.28	0.05	Per(T)	ns
Rodentibacter_pneumotropicus	11.79	2.35	Per(T)	over	1.80	0.02	Per(T)	ns
Burkholderia_gladioli	-2.45	2.33	Per(T)	under	-4.46	2.13	Per(T)	under
Oleiphilus_messinensis	-4.15	2.31	Per(T)	under	-2.95	1.47	Per(T)	under
Alcaligenes_aquatilis	-4.86	2.31	Per(T)	under	-2.99	1.52	Per(T)	under
Leptotrichia_shahii	-4.91	2.31	Per(T)	under	-1.19	0.38	Per(T)	ns
Turneriella_parva	3.72	2.28	Per(T)	over	0.66	0.01	Per(T)	ns
Clostridium_thermarum	-1.02	2.27	Per(T)	under	-0.85	0.06	Per(T)	ns
Photobacterium_damselae	-2.56	2.27	Per(T)	under	0.32	0.01	Per(T)	ns
Thalassotalea_sp._PS06	-4.64	2.27	Per(T)	under	-0.52	0.52	Per(T)	ns
Fusobacterium_ulcerans	-2.54	2.26	Per(T)	under	-0.37	0.04	Per(T)	ns
Niastella_koreensis	-2.44	2.25	Per(T)	under	0.95	0.13	Per(T)	ns
Coriobacterium_glomerans	-1.19	2.22	Per(T)	under	2.42	2.24	Per(T)	over
Verrucomicrobium_spinosum	2.33	2.22	Per(T)	over	1.32	0.18	Per(T)	ns
Gemmobacter_sp._HYN0069	-3.99	2.21	Per(T)	under	0.16	0.06	Per(T)	ns
Phytobacter_ursingii	-4.33	2.21	Per(T)	under	-0.43	0.07	Per(T)	ns
Ehrlichia_ruminantium	-5.06	2.21	Per(T)	under	0.25	0.01	Per(T)	ns
Inhella_inkyongensis	NA	2.20	Per(T)	ns	2.87	1.77	Per(T)	over
Arcobacter_cloacae	NA	2.20	Per(T)	ns	-4.67	1.74	Per(T)	under
Campylobacter_curvus	NA	2.20	Per(T)	ns	-4.91	1.74	Per(T)	under
Haemophilus_influenzae	12.98	2.20	Per(T)	over	2.40	0.18	Per(T)	ns

Halomicronema_hongdechloris	6.29	2.20	Per(T)	over	4.41	1.35	Per(T)	over
Acidaminococcus_fermentans	1.94	2.20	Per(T)	over	0.34	0.01	Per(T)	ns
Eggerthella_lenta	-1.85	2.20	Per(T)	under	0.98	1.40	Per(T)	over
Arthrobacter_citreus	-2.22	2.20	Per(T)	under	0.58	0.35	Per(T)	ns
Campylobacter_cuniculorum	-2.75	2.20	Per(T)	under	-1.83	1.09	Per(T)	ns
Flavobacterium_album	-2.99	2.20	Per(T)	under	0.76	0.01	Per(T)	ns
Sphingobacterium_thalpophilum	-3.09	2.20	Per(T)	under	0.65	0.25	Per(T)	ns
Anaeromyxobacter_sp._Fw109-5	-3.94	2.20	Per(T)	under	0.37	0.01	Per(T)	ns
Pandoraea_sp._XY-2	-4.09	2.20	Per(T)	under	-1.16	0.62	Per(T)	ns
Pseudodesulfovibrio_profundus	-4.22	2.20	Per(T)	under	-2.74	1.25	Per(T)	ns
Helicobacter_sp._MIT_01-6242	-4.50	2.20	Per(T)	under	NA	1.92	Per(T)	ns
Roseivirga_pacifica	-5.00	2.20	Per(T)	under	0.89	0.10	Per(T)	ns
Lactococcus_piscium	-5.11	2.20	Per(T)	under	0.50	0.01	Per(T)	ns
Sphingobacterium_hotanense	-5.45	2.20	Per(T)	under	-3.78	1.46	Per(T)	under
Legionella_sainthelensi	-6.26	2.20	Per(T)	under	-0.03	0.01	Per(T)	ns
Polaribacter_sp._KT25b	NA	2.20	Per(T)	ns	-4.20	1.52	Per(T)	under
Flavobacterium_johnsoniae	NA	2.20	Per(T)	ns	-3.62	1.48	Per(T)	under
Bacillus_flexus	-2.54	2.19	Per(T)	under	0.20	0.01	Per(T)	ns
Sinorhizobium_fredii	-2.11	2.17	Per(T)	under	2.08	0.91	Per(T)	ns
Chitinophaga_sp._H33E-04	-2.14	2.15	Per(T)	under	0.61	0.08	Per(T)	ns
Mucilaginibacter_sp._HYN0043	-3.80	2.13	Per(T)	under	2.49	3.41	Per(T)	over
Pontibacter_korlensis	-4.20	2.13	Per(T)	under	0.95	0.01	Per(T)	ns
Helicobacter_himalayensis	-1.71	2.07	Per(T)	under	-2.36	2.91	Per(T)	under
Serpentinomonas_mccroryi	3.94	2.07	Per(T)	over	2.28	0.01	Per(T)	ns
Sinorhizobium_meliloti	-3.28	2.07	Per(T)	under	-1.48	0.42	Per(T)	ns
Hirschia_baltica	-3.66	2.07	Per(T)	under	0.03	0.18	Per(T)	ns
Microvirga_thermotolerans	3.17	2.03	Per(T)	over	1.08	0.01	Per(T)	ns
Mucilaginibacter_rubeus	-2.57	2.03	Per(T)	under	-1.12	1.13	Per(T)	ns
Hymenobacter_sp._BRD128	-2.24	2.00	Per(T)	under	1.77	0.71	Per(T)	ns
Salmonella_enterica	-1.54	1.99	Per(T)	under	-1.88	2.83	Per(T)	under
Desulfotomaculum_nigrificans	3.60	1.98	Per(T)	over	0.88	0.01	Per(T)	ns
Phycisphaera_mikurensis	-1.50	1.98	Per(T)	under	-1.89	1.58	Per(T)	under
Streptomyces_lunaelactis	4.58	1.98	Per(T)	over	1.32	0.24	Per(T)	ns
Herbaspirillum_seropedicae	4.03	1.98	Per(T)	over	-0.35	0.01	Per(T)	ns
Odoribacter_splanchnicus	-1.49	1.98	Per(T)	under	0.49	0.35	Per(T)	ns
Clostridium_fermenticellae	-1.85	1.98	Per(T)	under	0.42	0.01	Per(T)	ns
Dolosigranulum_pigrum	-1.91	1.98	Per(T)	under	-0.07	0.04	Per(T)	ns
Lactobacillus_allii	-4.24	1.98	Per(T)	under	-2.45	0.97	Per(T)	ns
Sphingosinithalassobacter_sp._CS137	-4.26	1.98	Per(T)	under	0.95	0.37	Per(T)	ns
Candidatus_Hamiltonella_defensa	-4.77	1.98	Per(T)	under	NA	1.92	Per(T)	ns
Bacillus_ciccensis	-4.79	1.98	Per(T)	under	-0.52	0.32	Per(T)	ns
Shewanella_sp._YLB-08	-5.00	1.98	Per(T)	under	NA	1.92	Per(T)	ns
Solitalea_canadensis	-5.05	1.98	Per(T)	under	-1.19	0.19	Per(T)	ns
Xanthomonas_hortorum	-2.63	1.98	Per(T)	under	-1.50	0.83	Per(T)	ns
Flavobacterium_columnare	-3.63	1.97	Per(T)	under	-0.20	0.39	Per(T)	ns
Lactobacillus_dextrinicus	-2.62	1.97	Per(T)	under	-2.74	1.87	Per(T)	under
Echinicola_rosea	-1.61	1.96	Per(T)	under	1.25	0.20	Per(T)	ns
Elizabethkingia_sp._JS20170427COW	-2.05	1.94	Per(T)	under	-2.59	3.05	Per(T)	under
Denitrovibrio_acetiphilus	-2.42	1.92	Per(T)	under	-3.82	3.05	Per(T)	under
Niabella_soli	-2.40	1.90	Per(T)	under	1.23	0.29	Per(T)	ns
Bacillus_subtilis	-1.53	1.90	Per(T)	under	-0.12	0.01	Per(T)	ns
Ruminiclostridium_cellulolyticum	-2.85	1.89	Per(T)	under	-0.50	0.01	Per(T)	ns
Bdellovibrio_bacteriovorus	2.87	1.89	Per(T)	over	2.18	0.44	Per(T)	ns
Paraoceanicella_profunda	-3.04	1.88	Per(T)	under	1.93	0.51	Per(T)	ns
Acetoanaerobium_sticklandii	-2.34	1.86	Per(T)	under	0.50	0.27	Per(T)	ns
Orientia_tsutsugamushi	-3.97	1.86	Per(T)	under	-2.75	1.08	Per(T)	ns
Syntrophobacter_fumaroxidans	-2.15	1.84	Per(T)	under	0.76	0.37	Per(T)	ns
Granulosicoccus_antarcticus	-3.83	1.84	Per(T)	under	1.62	0.68	Per(T)	ns
Prevotella_melaninogenica	-1.68	1.84	Per(T)	under	-3.12	4.51	Per(T)	under
Mahella_australiensis	-2.48	1.82	Per(T)	under	-0.46	0.04	Per(T)	ns
Limosilactobacillus_reuteri	2.36	1.82	Per(T)	over	-1.26	0.64	Per(T)	ns
Flavisolibacter_sp._17J28-1	-1.88	1.80	Per(T)	under	0.66	0.01	Per(T)	ns
Ruthenibacterium_lactatiformans	-1.24	1.80	Per(T)	under	0.39	0.01	Per(T)	ns
Sulfurimonas_denitrificans	-3.35	1.80	Per(T)	under	-0.81	0.01	Per(T)	ns
Cyclobacterium_amurskyense	-2.72	1.79	Per(T)	under	0.93	0.02	Per(T)	ns
Exiguobacterium_sp._Helios	-2.88	1.79	Per(T)	under	-1.40	0.56	Per(T)	ns

Erysipelothrix_rhusiopathiae	-3.00	1.79	Per(T)	under	-0.62	0.07	Per(T)	ns
Turicibacter_sp._H121	-1.65	1.77	Per(T)	under	7.37	4.51	Per(T)	over
Staphylococcus_pseudintermedius	-0.64	1.77	Per(T)	under	-1.92	2.62	Per(T)	under
Campylobacter_jejuni	-1.15	1.77	Per(T)	under	1.16	0.23	Per(T)	ns
Finegoldia_magna	-1.37	1.77	Per(T)	under	-0.36	0.16	Per(T)	ns
Sphingomonas_sp._HDW15A	NA	1.77	Per(T)	ns	3.51	1.82	Per(T)	over
Paenisporosarcina_sp._K2R23-3	NA	1.77	Per(T)	ns	-5.47	1.52	Per(T)	under
Myroides_odoratus	NA	1.77	Per(T)	ns	-5.84	1.52	Per(T)	under
Rodentibacter_sp._DSM_111151	6.38	1.77	Per(T)	over	0.58	0.01	Per(T)	ns
Curtobacterium_sp._MR_MD2014	6.11	1.77	Per(T)	over	6.49	0.87	Per(T)	ns
Octadecabacter_sp._SW4	5.33	1.77	Per(T)	over	2.38	0.18	Per(T)	ns
Dehalogenimonas_lykanthroporepellens	5.04	1.77	Per(T)	over	1.13	0.04	Per(T)	ns
Lactobacillus_pentosus	-1.29	1.77	Per(T)	under	-0.27	0.01	Per(T)	ns
Chryseobacterium_taihuense	-1.42	1.77	Per(T)	under	-0.14	0.01	Per(T)	ns
Chitinophaga_sp._MD30	-1.88	1.77	Per(T)	under	-1.45	1.22	Per(T)	ns
Bacillus_sonorensis	-2.39	1.77	Per(T)	under	-1.25	0.86	Per(T)	ns
Jeotgalibacillus_malaysiensis	-2.95	1.77	Per(T)	under	-0.06	0.01	Per(T)	ns
Wenzhouxiangella_sp._AB-CW3	-3.46	1.77	Per(T)	under	1.38	0.24	Per(T)	ns
Acidobacterium_sp._4Y35	-3.86	1.77	Per(T)	under	-1.58	0.52	Per(T)	ns
Streptococcus_parasanguinis	-3.91	1.77	Per(T)	under	-0.23	0.08	Per(T)	ns
Saprospira_grandis	-3.92	1.77	Per(T)	under	1.27	0.01	Per(T)	ns
Hymenobacter_sp._DG25A	-4.38	1.77	Per(T)	under	3.16	0.73	Per(T)	ns
Pectobacterium_parmentieri	-4.72	1.77	Per(T)	under	0.06	0.07	Per(T)	ns
Aquimarina_sp._AD1	-4.81	1.77	Per(T)	under	1.99	1.32	Per(T)	over
Dyella_sp._G9	-4.82	1.77	Per(T)	under	1.03	0.05	Per(T)	ns
Fusobacterium_hwasookii	-5.27	1.77	Per(T)	under	-2.99	0.78	Per(T)	ns
Laribacter_hongkongensis	-5.69	1.77	Per(T)	under	-0.92	0.18	Per(T)	ns
Bacillus_amyloliquefaciens	-1.60	1.75	Per(T)	under	-1.69	1.83	Per(T)	under
Lactobacillus_vaginalis	2.54	1.74	Per(T)	over	-0.53	0.01	Per(T)	ns
Kosakonia_arachidis	-3.32	1.72	Per(T)	under	-2.89	1.38	Per(T)	under
Flavobacterium_sp._MDT1-60	-2.19	1.72	Per(T)	under	-1.99	1.29	Per(T)	ns
Sulfurospirillum_sp._UCH001	-3.64	1.72	Per(T)	under	-2.88	1.02	Per(T)	ns
Crassaminicella_sp._SY095	-1.77	1.72	Per(T)	under	0.03	0.01	Per(T)	ns
Lactobacillus_sp.	6.67	1.71	Per(T)	over	-0.76	0.22	Per(T)	ns
[Mannheimia]_succiniciproducens	5.92	1.69	Per(T)	over	-1.37	0.01	Per(T)	ns
Methylocystis_parvus	4.70	1.69	Per(T)	over	4.86	1.21	Per(T)	ns
Flexistipes_sinusarabici	-2.21	1.69	Per(T)	under	-1.07	0.47	Per(T)	ns
Aequorivita_sublithincola	-3.58	1.69	Per(T)	under	0.74	0.23	Per(T)	ns
Anaerolinea_thermophila	-1.88	1.68	Per(T)	under	-0.36	0.16	Per(T)	ns
Campylobacter_hominis	-1.60	1.67	Per(T)	under	NA	4.51	Per(T)	ns
Cupriavidus_necator	-3.00	1.65	Per(T)	under	-2.49	1.08	Per(T)	ns
Amniculibacterium_sp._G2-70	-3.82	1.65	Per(T)	under	-1.97	0.69	Per(T)	ns
Chryseobacterium_taklimakanense	-1.45	1.64	Per(T)	under	1.29	0.75	Per(T)	ns
Hydrogenophaga_sp._BPS33	-3.25	1.64	Per(T)	under	-0.28	0.08	Per(T)	ns
Beggiatoa_leptomitoformis	-3.03	1.64	Per(T)	under	0.68	0.18	Per(T)	ns
Muribaculum_intestinale	-1.30	1.63	Per(T)	under	1.47	3.77	Per(T)	over
Propionibacterium_australiense	2.61	1.63	Per(T)	over	2.23	0.67	Per(T)	ns
Desulfobulbus_oralis	-2.08	1.62	Per(T)	under	-0.01	0.01	Per(T)	ns
Paenibacillus_sp._FSL_R5-0345	-2.27	1.62	Per(T)	under	0.12	0.01	Per(T)	ns
Thermoanaerobacterium_thermosaccharolyticum	-2.79	1.62	Per(T)	under	-1.05	0.36	Per(T)	ns
Thermus_oshimai	1.95	1.62	Per(T)	over	0.95	0.04	Per(T)	ns
Pseudomonas_sp._URMO17WK12:I11	4.30	1.60	Per(T)	over	0.90	0.01	Per(T)	ns
Vibrio_campbellii	-2.79	1.60	Per(T)	under	0.11	0.07	Per(T)	ns
Jeotgalicoccus_saudimassiliensis	-3.51	1.57	Per(T)	under	0.98	0.13	Per(T)	ns
Paenibacillus_sp._FSL_H7-0357	-1.75	1.56	Per(T)	under	-1.72	1.06	Per(T)	ns
Flavobacterium_sediminis	-2.21	1.56	Per(T)	under	0.65	0.01	Per(T)	ns
Komagataeibacter_oboediens	5.20	1.56	Per(T)	over	1.37	0.08	Per(T)	ns
Streptococcus_sp._I-P16	5.09	1.56	Per(T)	over	-1.49	0.01	Per(T)	ns
Hydrogenophaga_sp._PBL-H3	4.67	1.56	Per(T)	over	3.20	0.46	Per(T)	ns
Bacillus_wiedmannii	-4.03	1.56	Per(T)	under	-2.24	0.78	Per(T)	ns
Streptococcus_porcinus	-1.42	1.55	Per(T)	under	-1.21	0.73	Per(T)	ns
Erysipelothrix_sp._HDW6C	-1.64	1.55	Per(T)	under	-0.95	0.48	Per(T)	ns
Mucilaginibacter_ginsenosidivorax	-2.36	1.55	Per(T)	under	1.13	0.39	Per(T)	ns
Muricauda_lutaonensis	-0.71	1.54	Per(T)	under	-1.96	2.05	Per(T)	under
Fusobacterium_mortiferum	-1.91	1.54	Per(T)	under	0.53	0.01	Per(T)	ns
Streptococcus_sp._KS_6	-0.80	1.54	Per(T)	under	-0.36	0.01	Per(T)	ns

Marinomonas_sp._MWYL1	-3.93	1.52	Per(T)	under	-0.98	0.32	Per(T)	ns
Peribacillus_asahii	-2.25	1.52	Per(T)	under	-0.94	0.19	Per(T)	ns
Limnoglobus_roseus	-3.46	1.52	Per(T)	under	-0.29	0.04	Per(T)	ns
Clostridium_saccharobutylicum	-2.64	1.50	Per(T)	under	-1.43	0.13	Per(T)	ns
Chryseobacterium_sp.	-3.06	1.50	Per(T)	under	0.78	0.12	Per(T)	ns
Amphibacillus_xylanus	-3.07	1.50	Per(T)	under	1.47	0.04	Per(T)	ns
Colwellia_sp._20A7	-3.46	1.50	Per(T)	under	NA	1.92	Per(T)	ns
Candidatus_Arthromitus_sp._SFB-mouse	-5.24	1.50	Per(T)	under	-4.59	0.78	Per(T)	ns
Altibacter_sp._ALE3EI	-1.30	1.49	Per(T)	under	0.74	0.18	Per(T)	ns
Rhodococcus_sp._X156	2.62	1.49	Per(T)	over	3.18	0.65	Per(T)	ns
Planococcus_sp._PAMC_21323	-2.43	1.49	Per(T)	under	-3.27	2.10	Per(T)	under
Kushneria_konosiri	0.16	1.49	Per(T)	over	-0.39	0.04	Per(T)	ns
Bifidobacterium_breve	-0.99	1.49	Per(T)	under	-0.10	0.01	Per(T)	ns
Geobacter_pickeringii	-1.85	1.48	Per(T)	under	-0.99	0.44	Per(T)	ns
Ferriphaselus_amnicola	0.48	1.48	Per(T)	over	-0.57	0.13	Per(T)	ns
Pseudoarcobacter_acticola	-0.10	1.48	Per(T)	under	-0.56	0.04	Per(T)	ns
Neisseria_zalophi	-0.18	1.48	Per(T)	under	-0.62	0.01	Per(T)	ns
Staphylococcus_agnetis	-0.54	1.48	Per(T)	under	-0.47	0.04	Per(T)	ns
Viridibacillus_sp._JNUCC-6	-2.34	1.48	Per(T)	under	-0.43	0.18	Per(T)	ns
Streptomyces_olivoreticuli	5.56	1.43	Per(T)	over	-0.29	0.01	Per(T)	ns
Gordonia_sp._HY186	4.02	1.43	Per(T)	over	-1.49	0.01	Per(T)	ns
Salmonella_bongori	3.71	1.43	Per(T)	over	-0.39	0.01	Per(T)	ns
Metabacillus_sp._KUDC1714	-3.44	1.43	Per(T)	under	0.02	0.01	Per(T)	ns
Hydrogenophaga_sp._NH-16	2.93	1.42	Per(T)	over	0.00	0.01	Per(T)	ns
Streptococcus_canis	3.54	1.41	Per(T)	over	0.04	0.01	Per(T)	ns
Veillonella_parvula	-2.26	1.41	Per(T)	under	-0.64	0.11	Per(T)	ns
Thermochromatium_tepidum	2.50	1.41	Per(T)	over	2.64	0.51	Per(T)	ns
Chitinophaga_caeni	-1.65	1.41	Per(T)	under	2.80	0.43	Per(T)	ns
Hymenobacter_sp._BT18	-2.05	1.41	Per(T)	under	1.09	0.33	Per(T)	ns
Melioribacter_roseus	-1.91	1.40	Per(T)	under	0.39	0.01	Per(T)	ns
Alcaligenes_faecalis	-3.65	1.40	Per(T)	under	3.05	2.83	Per(T)	over
Glaesserella_sp._15-184	6.44	1.40	Per(T)	over	1.02	0.08	Per(T)	ns
Avibacterium_volantium	5.59	1.40	Per(T)	over	1.21	0.01	Per(T)	ns
Weissella_soli	-3.07	1.40	Per(T)	under	-0.22	0.01	Per(T)	ns
Actinomyces_howellii	-3.74	1.40	Per(T)	under	-0.19	0.24	Per(T)	ns
Chryseobacterium_balustinum	NA	1.37	Per(T)	ns	2.68	2.34	Per(T)	over
Mucilaginibacter_ginsenosidivorans	-1.45	1.37	Per(T)	under	1.88	1.95	Per(T)	over
Bradyrhizobium_sp._CCBAU_53421	-3.56	1.37	Per(T)	under	2.24	1.74	Per(T)	over
Flavobacterium_sp._LPB0248	-3.53	1.37	Per(T)	under	-3.94	1.18	Per(T)	ns
Actinobacillus_porcitonsillarum	8.78	1.37	Per(T)	over	0.17	0.01	Per(T)	ns
Neisseria_sp._10022	5.42	1.37	Per(T)	over	2.55	0.18	Per(T)	ns
Agrobacterium_larrymoorei	4.99	1.37	Per(T)	over	NA	0.01	Per(T)	ns
Actinoplanes_friuliensis	4.76	1.37	Per(T)	over	3.27	0.72	Per(T)	ns
Paracoccus_sp._Arc7-R13	3.95	1.37	Per(T)	over	1.50	0.04	Per(T)	ns
Actinoplanes_sp._N902-109	3.64	1.37	Per(T)	over	1.50	0.18	Per(T)	ns
Laceyella_sacchari	2.63	1.37	Per(T)	over	-0.45	0.01	Per(T)	ns
Sulfurimonas_gotlandica	-3.81	1.37	Per(T)	under	0.04	0.01	Per(T)	ns
Winogradskyella_schleiferi	-3.83	1.37	Per(T)	under	-2.81	0.72	Per(T)	ns
Lactobacillus_lindneri	-3.95	1.37	Per(T)	under	-0.36	0.01	Per(T)	ns
Haloactinobacterium_sp._RN3S43	-4.03	1.37	Per(T)	under	0.35	0.01	Per(T)	ns
Solibacillus_sp._R5-41	-4.36	1.37	Per(T)	under	-0.55	0.01	Per(T)	ns
Oligella_urethralis	4.44	1.37	Per(T)	over	3.88	1.91	Per(T)	over
Pelistega_sp._NLN63	6.11	1.37	Per(T)	over	2.96	0.45	Per(T)	ns
Mycobacterium_sp._KMS	3.69	1.37	Per(T)	over	-1.22	0.01	Per(T)	ns
Pusillimonas_sp._T7-7	3.61	1.37	Per(T)	over	3.18	0.78	Per(T)	ns
Sphingobacterium_sp._DR205	-1.69	1.37	Per(T)	under	1.24	0.12	Per(T)	ns
Buttiauxella_sp._3AFRM03	-3.18	1.37	Per(T)	under	1.31	0.27	Per(T)	ns
Xanthomonas_sacchari	-2.52	1.36	Per(T)	under	-0.72	0.55	Per(T)	ns
Ralstonia_solanacearum	-2.17	1.35	Per(T)	under	2.53	0.35	Per(T)	ns
Tetragenococcus_halophilus	-0.98	1.34	Per(T)	under	0.46	0.01	Per(T)	ns
Candidatus_Arthromitus_sp._SFB-mouse-NL	-5.02	1.33	Per(T)	under	-4.68	1.14	Per(T)	ns
Colwellia_sp._Arc7-635	-1.69	1.32	Per(T)	under	0.26	0.34	Per(T)	ns
Candidatus_Symbiobacter_mobilis	-1.82	1.32	Per(T)	under	2.78	0.37	Per(T)	ns
Moorella_thermoacetica	-0.31	1.31	Per(T)	under	1.03	1.08	Per(T)	ns
Hymenobacter_nivis	-2.07	1.31	Per(T)	under	2.26	1.18	Per(T)	ns
Leptospirillum_ferrooxidans	-2.90	1.31	Per(T)	under	-0.06	0.04	Per(T)	ns

Paeniclostridium_sordellii	-1.42	1.31	Per(T)	under	0.59	0.11	Per(T)	ns
Geobacter_sp._M21	2.33	1.31	Per(T)	over	0.69	0.01	Per(T)	ns
Avibacterium_paragallinarum	3.48	1.18	Per(T)	ns	2.47	2.25	Per(T)	over
Campylobacter_coli	-1.16	1.16	Per(T)	ns	-1.38	1.43	Per(T)	under
Mucilaginibacter_paludis	-1.84	1.13	Per(T)	ns	2.30	1.90	Per(T)	over
Ectothiorhodospira_sp._BSL-9	-1.60	1.13	Per(T)	ns	-2.81	1.83	Per(T)	under
Ottowia_sp._oral_taxon_894	-2.27	1.10	Per(T)	ns	1.90	2.13	Per(T)	over
Pseudoleptotrichia_goodfellowii	-1.44	1.09	Per(T)	ns	-3.14	2.21	Per(T)	under
Acinetobacter_baylyi	4.01	1.06	Per(T)	ns	4.31	1.96	Per(T)	over
Jeotgalibaca_dankookensis	NA	1.03	Per(T)	ns	2.73	1.92	Per(T)	over
Nesterenkonia_sp._NBAIMH1	-3.79	1.03	Per(T)	ns	2.52	1.35	Per(T)	over
Nibricoccus_aquaticus	2.64	0.98	Per(T)	ns	2.18	1.35	Per(T)	over
Rhodopseudomonas_palustris	3.60	0.93	Per(T)	ns	2.82	1.76	Per(T)	over
Lactobacillus_gasseri	-0.76	0.93	Per(T)	ns	-1.64	1.67	Per(T)	under
Sulfurifustis_variabilis	3.37	0.91	Per(T)	ns	4.01	1.91	Per(T)	over
Pontibacter_pudoricolor	-0.91	0.87	Per(T)	ns	-1.25	1.36	Per(T)	under
Glaciecola_nitratireducens	-2.72	0.87	Per(T)	ns	3.08	1.94	Per(T)	over
Vibrio_mediterranei	1.76	0.86	Per(T)	ns	2.39	1.38	Per(T)	over
Marinomonas_primoryensis	3.41	0.86	Per(T)	ns	3.26	1.62	Per(T)	over
Eggerthella_sp._YY7918	-0.91	0.85	Per(T)	ns	0.90	2.63	Per(T)	over
Flavobacterium_sangjuense	-1.95	0.85	Per(T)	ns	-5.09	1.52	Per(T)	under
Eggerthella_sp._HF-1101	-0.61	0.82	Per(T)	ns	1.13	1.35	Per(T)	over
Olsenella_umbonata	-0.79	0.79	Per(T)	ns	2.82	3.26	Per(T)	over
Chryseobacterium_manosquense	4.95	0.77	Per(T)	ns	4.77	4.51	Per(T)	over
Pseudorhodobacter_sp._S12M18	4.78	0.75	Per(T)	ns	2.40	1.52	Per(T)	over
Wolinella_succinogenes	-3.25	0.74	Per(T)	ns	2.93	1.89	Per(T)	over
Brevundimonas_naejangsanensis	0.00	0.73	Per(T)	ns	2.44	1.54	Per(T)	over
Peptoniphilus_ivorii	1.03	0.73	Per(T)	ns	2.17	2.73	Per(T)	over
Lactobacillus_amylolyticus	-0.68	0.73	Per(T)	ns	-1.22	1.41	Per(T)	under
Veillonella_rodentium	-1.12	0.73	Per(T)	ns	1.89	1.36	Per(T)	over
Desulfotomaculum_ferrireducens	1.34	0.72	Per(T)	ns	1.41	1.52	Per(T)	over
Hymenobacter_sp._DG25B	-1.14	0.72	Per(T)	ns	1.63	2.41	Per(T)	over
Spirosoma_montaniterrae	-0.78	0.72	Per(T)	ns	1.65	1.95	Per(T)	over
Geobacillus_stearothermophilus	2.53	0.69	Per(T)	ns	2.10	1.36	Per(T)	over
Streptococcus_intermedius	-0.88	0.61	Per(T)	ns	2.35	2.91	Per(T)	over
Capnocytophaga_gingivalis	-0.94	0.61	Per(T)	ns	1.52	1.48	Per(T)	over
Lactobacillus_acidophilus	-0.27	0.55	Per(T)	ns	-1.14	1.35	Per(T)	under
Casimicrobium_huifangae	2.76	0.50	Per(T)	ns	2.73	2.83	Per(T)	over
Alistipes_sp._dk3624	-0.95	0.47	Per(T)	ns	0.88	2.33	Per(T)	over
Alistipes_onderdonkii	-0.47	0.47	Per(T)	ns	2.36	1.41	Per(T)	over
Massilia_sp._NR_4-1	3.30	0.47	Per(T)	ns	2.52	3.41	Per(T)	over
Chryseobacterium_shandongense	3.48	0.47	Per(T)	ns	1.88	3.41	Per(T)	over
Mucilaginibacter_sp._F39-2	4.00	0.47	Per(T)	ns	1.96	3.17	Per(T)	over
Flavobacterium_gilvum	-0.70	0.47	Per(T)	ns	-6.30	2.39	Per(T)	under
Actinotignum_schaalii	2.40	0.47	Per(T)	ns	1.80	2.24	Per(T)	over
Robiginitalea_biformata	-0.71	0.47	Per(T)	ns	1.24	1.95	Per(T)	over
Neisseria_animaloris	4.26	0.47	Per(T)	ns	4.86	1.74	Per(T)	over
Chryseobacterium_lactis	4.74	0.47	Per(T)	ns	5.21	1.52	Per(T)	over
Porphyromonas_asaccharolytica	-0.33	0.46	Per(T)	ns	1.62	2.83	Per(T)	over
Hymenobacter_sp._APR13	1.51	0.46	Per(T)	ns	3.04	1.82	Per(T)	over
Tannerella_forsythia	-0.07	0.46	Per(T)	ns	1.15	2.24	Per(T)	over
Methylorubrum_populi	-0.99	0.42	Per(T)	ns	3.96	2.91	Per(T)	over
Gemmatimonas_aurantiaca	-2.11	0.42	Per(T)	ns	2.68	3.05	Per(T)	over
Collinsella_aerofaciens	-0.33	0.42	Per(T)	ns	1.48	1.66	Per(T)	over
Virgibacillus_phasianinus	-0.52	0.39	Per(T)	ns	1.92	1.83	Per(T)	over
Paenibacillus_mucilaginosus	0.82	0.38	Per(T)	ns	1.99	1.52	Per(T)	over
Actinobacillus_pleuropneumoniae	3.12	0.37	Per(T)	ns	-5.11	1.74	Per(T)	under
Magnetospirillum_gryphiswaldense	1.80	0.37	Per(T)	ns	2.37	1.71	Per(T)	over
Xanthomonas_oryzae	-1.18	0.35	Per(T)	ns	2.27	1.81	Per(T)	over
Streptosporangium_roseum	-0.79	0.35	Per(T)	ns	2.53	1.90	Per(T)	over
Chlorobaculum_parvum	3.81	0.32	Per(T)	ns	1.94	2.33	Per(T)	over
Chryseobacterium_sp._6424	3.32	0.31	Per(T)	ns	1.91	1.80	Per(T)	over
Fibrella_aestuarina	0.00	0.31	Per(T)	ns	1.98	1.53	Per(T)	over
Burkholderia_pseudomallei	2.24	0.31	Per(T)	ns	1.80	2.15	Per(T)	over
Hymenobacter_sp._TS19	-0.58	0.31	Per(T)	ns	2.52	1.90	Per(T)	over
Olsenella_uli	-0.40	0.29	Per(T)	ns	2.74	4.51	Per(T)	over

Paludibaculum_fermentans	-1.38	0.27	Per(T)	ns	2.98	3.77	Per(T)	over
Flavobacterium_magnum	0.27	0.27	Per(T)	ns	3.34	2.33	Per(T)	over
Olsenella_sp._LZLJ-2	-0.30	0.25	Per(T)	ns	2.96	2.90	Per(T)	over
Chryseolinea_soli	0.50	0.25	Per(T)	ns	2.52	2.73	Per(T)	over
Cutibacterium_avidum	3.72	0.25	Per(T)	ns	3.39	2.62	Per(T)	over
Blastochloris_tepida	-0.84	0.25	Per(T)	ns	2.68	2.34	Per(T)	over
Lacipirellula_parvula	3.99	0.25	Per(T)	ns	2.31	1.75	Per(T)	over
Flavobacterium_sp._I3-2	NA	0.25	Per(T)	ns	3.65	1.49	Per(T)	over
Jeotgalibaca_arthritidis	1.31	0.25	Per(T)	ns	2.74	1.36	Per(T)	over
Gemmata_massiliana	NA	0.25	Per(T)	ns	2.62	1.36	Per(T)	over
Aeromonas_encheleia	3.30	0.25	Per(T)	ns	2.22	1.41	Per(T)	over
Baekduia_soli	-0.77	0.25	Per(T)	ns	2.60	2.34	Per(T)	over
Pseudomonas_tolaasii	0.84	0.24	Per(T)	ns	1.41	1.57	Per(T)	over
Treponema_brennaborense	-0.17	0.20	Per(T)	ns	2.69	1.92	Per(T)	over
Corynebacterium_ureicelerivorans	3.36	0.20	Per(T)	ns	3.79	1.62	Per(T)	over
Hymenobacter_sp._BT182	1.94	0.20	Per(T)	ns	2.40	3.26	Per(T)	over
Rubinisphaera_brasiliensis	1.09	0.19	Per(T)	ns	4.77	2.42	Per(T)	over
Desulfomonile_tiedjei	0.19	0.19	Per(T)	ns	2.48	3.17	Per(T)	over
Filimonas_lacunae	0.72	0.16	Per(T)	ns	2.39	3.26	Per(T)	over
Dokdonella_koreensis	0.22	0.16	Per(T)	ns	2.15	1.43	Per(T)	over
Nitratifractor_salsuginis	0.69	0.16	Per(T)	ns	3.34	1.94	Per(T)	over
Turicibacter_sanguinis	-0.30	0.15	Per(T)	ns	6.53	4.51	Per(T)	over
Marinobacter_sp._LV10R510-11A	-0.76	0.14	Per(T)	ns	3.34	1.52	Per(T)	over
Caldilinea_aerophila	-0.55	0.14	Per(T)	ns	3.48	1.77	Per(T)	over
Hymenobacter_sedentarius	1.51	0.14	Per(T)	ns	2.78	1.52	Per(T)	over
Metabacillus_litoralis	-0.34	0.13	Per(T)	ns	2.55	1.66	Per(T)	over
Filifactor_alocis	1.12	0.13	Per(T)	ns	2.56	2.51	Per(T)	over
Duncaniella_dubosii	2.51	0.12	Per(T)	ns	1.87	3.05	Per(T)	over
Corynebacterium_ammoniagenes	-1.57	0.12	Per(T)	ns	4.52	2.21	Per(T)	over
Mesorhizobium_ciceri	-1.50	0.12	Per(T)	ns	3.44	1.57	Per(T)	over
Brevibacterium_sp._YB235	4.30	0.12	Per(T)	ns	4.51	1.52	Per(T)	over
Adhaeribacter_swui	1.70	0.12	Per(T)	ns	1.68	1.47	Per(T)	over
Lysobacter_soli	-0.75	0.12	Per(T)	ns	3.20	1.77	Per(T)	over
Oceanicoccus_sagamiensis	-1.51	0.11	Per(T)	ns	2.87	1.59	Per(T)	over
Fastidiosipila_sanguinis	2.16	0.11	Per(T)	ns	2.34	1.36	Per(T)	over
Desulfovibrio_marinus	0.65	0.11	Per(T)	ns	1.57	1.95	Per(T)	over
Pseudonocardia_sp._HH130630-07	0.83	0.10	Per(T)	ns	3.21	1.65	Per(T)	over
Pandoraea_oxalativorans	0.05	0.10	Per(T)	ns	3.64	1.38	Per(T)	over
Succinivibrio_dextrinosolvens	3.00	0.09	Per(T)	ns	1.57	2.41	Per(T)	over
Celeribacter_baekdonensis	1.70	0.08	Per(T)	ns	3.90	1.65	Per(T)	over
Zunongwangia_profunda	-1.67	0.08	Per(T)	ns	2.78	1.52	Per(T)	over
Desulfosporosinus_meridiei	1.42	0.08	Per(T)	ns	1.96	2.41	Per(T)	over
Candidatus_Tachikawaea_gelatinosa	1.54	0.08	Per(T)	ns	3.28	3.41	Per(T)	over
Actinopolymorpha_singaporensis	1.07	0.08	Per(T)	ns	2.94	2.88	Per(T)	over
Saccharopolyspora_coralli	4.63	0.08	Per(T)	ns	3.17	2.74	Per(T)	over
Streptomyces_prasinus	NA	0.08	Per(T)	ns	4.39	2.73	Per(T)	over
Kosakonia_sp._SMBL-WEM22	3.57	0.08	Per(T)	ns	3.78	2.50	Per(T)	over
Streptomyces_sp._NHF165	1.66	0.08	Per(T)	ns	4.64	2.42	Per(T)	over
Proteiniphilum_saccharofermentans	0.47	0.08	Per(T)	ns	1.14	2.14	Per(T)	over
Massilia_sp._LPB0304	2.59	0.08	Per(T)	ns	1.36	1.97	Per(T)	over
Pseudoalteromonas_ulvae	NA	0.08	Per(T)	ns	3.52	1.82	Per(T)	over
Geobacter_bremensis	1.73	0.08	Per(T)	ns	1.90	1.57	Per(T)	over
Thermanaerovibrio_acidaminovorans	2.39	0.08	Per(T)	ns	1.53	1.48	Per(T)	over
Bacillus_coagulans	0.76	0.08	Per(T)	ns	2.92	1.47	Per(T)	over
Sutterella_megalosphaeroides	1.93	0.08	Per(T)	ns	0.88	1.47	Per(T)	over
Shewanella_violacea	3.68	0.08	Per(T)	ns	2.98	1.42	Per(T)	over
Sutterella_faecalis	2.34	0.08	Per(T)	ns	0.96	1.41	Per(T)	over
Mycolicibacterium_sarraceniae	3.80	0.08	Per(T)	ns	4.14	1.38	Per(T)	over
Sphingopyxis_sp._PAMC25046	-1.16	0.08	Per(T)	ns	3.36	1.36	Per(T)	over
Athalassotoga_saccharophila	2.08	0.08	Per(T)	ns	2.78	1.36	Per(T)	over
Staphylococcus_hominis	1.84	0.08	Per(T)	ns	2.57	1.35	Per(T)	over
Ferrovibrio_terrae	-0.41	0.07	Per(T)	ns	2.19	1.52	Per(T)	over
Chryseobacterium_sp._cx-624	3.09	0.07	Per(T)	ns	2.56	3.96	Per(T)	over
Olsenella_timonensis	0.07	0.06	Per(T)	ns	3.14	3.05	Per(T)	over
Libanicoccus_massiliensis	-0.08	0.05	Per(T)	ns	2.33	2.34	Per(T)	over
Pseudobacter_ginsenosidimutans	0.70	0.04	Per(T)	ns	1.86	2.62	Per(T)	over

Akkermansia_muciniphila	5.07	0.04	Per(T)	ns	5.19	3.17	Per(T)	over
Corynebacterium_sp._NML93-0612	5.26	0.03	Per(T)	ns	4.15	4.51	Per(T)	over
Pseudomonas_furukawaii	4.85	0.03	Per(T)	ns	3.39	4.51	Per(T)	over
Paenibacillus_antarcticus	1.00	0.03	Per(T)	ns	3.52	3.77	Per(T)	over
Mucinivorans_hirudinis	1.23	0.03	Per(T)	ns	1.73	3.77	Per(T)	over
Barnesiella_viscericola	1.03	0.03	Per(T)	ns	1.31	3.55	Per(T)	over
Klebsiella_aerogenes	3.43	0.03	Per(T)	ns	3.73	3.41	Per(T)	over
Hymenobacter_sp._DG01	2.87	0.03	Per(T)	ns	2.56	3.26	Per(T)	over
Altererythrobacter_marensis	4.12	0.03	Per(T)	ns	3.20	3.17	Per(T)	over
Olsenella_sp._GAM18	0.01	0.03	Per(T)	ns	2.84	3.05	Per(T)	over
Methylocaldum_marinum	4.90	0.03	Per(T)	ns	3.30	2.83	Per(T)	over
Methylocella_silvestris	4.17	0.03	Per(T)	ns	2.75	2.74	Per(T)	over
Allochromatium_vinosum	0.33	0.03	Per(T)	ns	2.99	2.73	Per(T)	over
Hyphomicrobium_denitrificans	2.86	0.03	Per(T)	ns	2.47	2.50	Per(T)	over
Gemmata_obscuriglobus	1.87	0.03	Per(T)	ns	3.33	2.42	Per(T)	over
Mesotoga_infera	0.92	0.03	Per(T)	ns	2.60	2.41	Per(T)	over
endosymbiont_'TC1'_of_Trimyema_compressum	NA	0.03	Per(T)	ns	5.75	2.39	Per(T)	over
Olsenella_sp._oral_taxon_807	0.59	0.03	Per(T)	ns	2.71	2.33	Per(T)	over
Prevotella_jejuni	0.28	0.03	Per(T)	ns	0.65	2.33	Per(T)	over
Rhodanobacter_denitrificans	0.07	0.03	Per(T)	ns	4.01	2.31	Per(T)	over
Geobacter_sp._DSM_9736	3.13	0.03	Per(T)	ns	1.85	2.25	Per(T)	over
Elizabethkingia_bruuniana	1.77	0.03	Per(T)	ns	2.80	2.25	Per(T)	over
Rhodothermus_marinus	0.76	0.03	Per(T)	ns	2.40	2.24	Per(T)	over
Bergeyella_cardium	1.88	0.03	Per(T)	ns	2.33	2.13	Per(T)	over
Antarcticibacterium_sp._PAMC_28998	2.69	0.03	Per(T)	ns	1.85	2.05	Per(T)	over
Treponema_azotonutricium	0.47	0.03	Per(T)	ns	1.35	2.05	Per(T)	over
Pontibacter_actiniarum	0.66	0.03	Per(T)	ns	1.19	2.05	Per(T)	over
Actinomyces_qiguomingii	1.57	0.03	Per(T)	ns	2.94	1.92	Per(T)	over
Nitrosospira_briensis	0.80	0.03	Per(T)	ns	3.52	1.89	Per(T)	over
Bosea_sp._RAC05	0.53	0.03	Per(T)	ns	3.89	1.89	Per(T)	over
Variovorax_paradoxus	2.34	0.03	Per(T)	ns	2.98	1.83	Per(T)	over
Brochothrix_thermosphacta	NA	0.03	Per(T)	ns	5.00	1.74	Per(T)	over
Chondromyces_crocatus	4.42	0.03	Per(T)	ns	2.58	1.69	Per(T)	over
Brevibacillus_sp._SCSIO_07484	1.07	0.03	Per(T)	ns	3.08	1.65	Per(T)	over
Frondihabitans_sp._762G35	NA	0.03	Per(T)	ns	5.40	1.61	Per(T)	over
Corynebacterium_stationis	0.41	0.03	Per(T)	ns	10.48	1.54	Per(T)	over
Spiroplasma_helicoides	0.52	0.03	Per(T)	ns	3.33	1.54	Per(T)	over
Limihaloglobus_sulfuriphilus	0.25	0.03	Per(T)	ns	2.22	1.52	Per(T)	over
Erythrobacter_seohaensis	-1.16	0.03	Per(T)	ns	4.06	1.47	Per(T)	over
Azospirillum_sp._TSH100	3.19	0.03	Per(T)	ns	3.54	1.47	Per(T)	over
Phaeobacter_gallaeciensis	4.86	0.03	Per(T)	ns	3.03	1.47	Per(T)	over
Streptomyces_sp._ZFG47	1.00	0.03	Per(T)	ns	2.97	1.47	Per(T)	over
Streptomyces_koyangensis	1.56	0.03	Per(T)	ns	4.14	1.46	Per(T)	over
Rubrobacter_sp._SCSIO_52909	0.97	0.03	Per(T)	ns	1.67	1.44	Per(T)	over
Paraburkholderia_sp._SOS3	3.83	0.03	Per(T)	ns	4.75	1.42	Per(T)	over
Sphaerobacter_thermophilus	1.07	0.03	Per(T)	ns	3.76	1.42	Per(T)	over
Corynebacterium_phocae	2.87	0.03	Per(T)	ns	2.70	1.42	Per(T)	over
Rhodoferax_sediminis	1.08	0.03	Per(T)	ns	1.80	1.42	Per(T)	over
Dokdonia_sp._MED134	0.94	0.03	Per(T)	ns	2.24	1.42	Per(T)	over
Spirosoma_radiotolerans	1.26	0.03	Per(T)	ns	3.49	1.40	Per(T)	over
Sphingosinicella_microcystinivorans	-0.97	0.03	Per(T)	ns	3.41	1.39	Per(T)	over
Aquisphaera_giovannonii	1.47	0.03	Per(T)	ns	3.49	1.35	Per(T)	over
