# Supplementary material for: Host circadian behaviors exert only weak selective pressure on the gut microbiome under stable conditions but are critical for recovery from antibiotic treatment
Source: PLoS Biol. 2022 Nov 9;20(11):e3001865. doi: 10.1371/journal.pbio.3001865 (PMC9645659; doi:10.1371/journal.pbio.3001865)
Supplement: S5 Table — Values within cells show the log2-fold changes observed when we compare the day indicated for each column (i.e., Days 11, 154, and 238) and Day −14 (prior to antibiotic treatment and/or transfer to RR) and were calculated as the ratio between the average relative abundance at the day indicated and Day −14. Cells highlighted in blue and red are those in which there is a significant decrease (blue) or increase (red) in abundance, as determined by a Mann–Whitney U test followed by a p-value adjustment for false discovery rate (q-value < 0.05). Cells that are not highlighted are those in which there were no significant changes in abundance for the time point specified. (PDF) [file pbio.3001865.s013.pdf]

S5 Table. Changes in representation for the 5421 species analyzed. Values within cells show the log2 fold-changes observed when we compare the day indicated for each column (i.e. Day 11, 154 and 238) and Day -14 (prior to antibiotic treatment and/or transfer to RR), and were calculated as the ratio between the average relative abundance at the day indicated and Day -14. Cells highlighted in blue and red are those in which there is a significant decrease (blue) or increase (red) in abundance, as determined by a Mann-Whitney U test followed by a p-value adjustment for false-discovery rate (q-value < 0.05). Cells that are not highlighted are those in which there were no significant changes in abundance for the timepoint specified.

log2 fold changes at the species level for each of our experimental groups. All comparisons were made in reference to Day -14 (prior to antibiotic treatment and/or transfer to RR). Cells highlighted in blue are those in which there is a significant decrease in species abundance, while cells highlighted in red are those in which there was a significant increase in species abundance (q-value < 0.05 in the Mann-Whitney U test). Cells that are not highlighted showed no significant changes in abundance at that particular timepoint.

	WT (T) log2 fold-change			Per 1/2-dko (T) log2 fold-change			WT (UT) log2 fold-change			Per 1/2-dko (UT) log2 fold-change		
Species	Day 11	Day 154	Day 238	Day 11	Day 154	Day 238	Day 11	Day 154	Day 238	Day 11	Day 154	Day 238
Helicobacter_hepaticus	-11.38236228	-8.862726025	-5.177684158	-8.95802696	-10.04146372	-9.089891689	-0.247196947	0.852782109	1.983663232	-0.786802169	-0.654002875	-0.877640062
Capnocytophaga_canimorsus	-9.555697545	-1.117425489	-1.426817122	-6.037629058	-4.10305135	-3.358569968	0	8.627369725	9.793234257	-0.192587338	0.113238851	-0.099878551
Francisella_persica	-7.974776548	-3.85070544	-3.859568716	-1.216118188	1.845857436	1.902591659	-8.10567284	0.460727555	2.739817211	-1.405746966	-0.995664188	0.010547442
Capnocytophaga_haemolytica	-7.666002033	-4.401559432	-0.215435125	0.240233051	-0.491837357	-0.352532154	-9.739012655	1.396113023	-2.581898187	1.513884433	1.223923543	0.901560029
Candidatus_Arthromitus_sp._SFB-mouse-NL	-7.610808841	-3.776805082	-3.584744291	-5.021795961	-1.056144956	-4.676731367	-1.770617647	1.42145897	1.493375679	0.193671407	-3.014488726	-1.870687883
Tenacibaculum_sp._SZ-18	-7.586660327	-1.892022339	-4.491102772	-1.103395669	1.100361687	1.226197412	1.08717616	0.550554128	0.228626033	-1.605845327	-0.760307211	0.77218787
Ottowia_sp._oral_taxon_894	-7.546713629	-3.15708229	-1.783293755	-2.267549887	0.682994584	1.897391707	-0.166890634	-0.033708429	1.517640239	0.717685816	0.048341809	0.180807683
Staphylococcus_lentus	-7.354143775	1.210850349	-7.804858727	4.771025804	2.166023291	1.329265826	0.014023489	-0.866671805	-0.806118954	-0.473619591	2.957964715	2.572596595
Nonlabens_sp._MJ115	-7.035728631	-1.669822839	-0.343237666	NA	-3.101695397	1.249119659	-9.076815597	0.691067583	1.860823049	0.083400193	-0.353949768	0.823417325
Aquimarina_sp._AD1	-7.010442222	-0.552881337	-1.248074755	-4.80934378	0.885010371	1.991583886	-0.469794438	0.022086207	-0.430363442	-0.381689731	1.360765874	0.709164164
Calditerrivibrio_nitroreducens	-6.954416986	-5.156499889	-6.077539816	-6.340018249	-4.049264348	-4.159016591	0	0	6.15711649	-0.163877523	0.085881778	-0.476436485
Hypericibacter_terrae	-6.872071042	-4.806976013	-0.556631146	NA	-2.101075594	-3.155307905	0.383328941	1.287341052	0.818093166	0.935987804	1.232433055	0.292705351
Lactobacillus_amylolyticus	-6.838773577	-9.725339715	-8.67994258	-0.675331893	-0.772485127	-1.215798896	-0.274900013	0.697301208	1.421765074	-1.358956848	-0.867246497	-0.452038417
Leuconostoc_suionicum	-6.813016257	-3.992485946	-7.748310568	0.196043654	2.543542573	2.895438509	11.14963282	0	8.48088308	-3.693455496	-2.17678977	-0.472319196
Shewanella_amazonensis	-6.785748466	-1.481474236	-0.066313392	NA	4.019588541	3.522722735	8.246199041	0	9.280348569	0.053189735	-0.227108817	0.977968693
Winogradskyella_sediminis	-6.776400349	-5.342379747	-0.491978389	-3.400334843	-0.50490532	0.424093592	-8.799135887	-8.799135887	-1.642021419	-0.049591237	0.566367948	0.836268867
Xanthomonas_oryzae	-6.73311851	-1.695201574	-0.540718476	-1.178864346	1.516334412	2.268315012	-0.205792245	0.826390592	1.147720115	-0.023692557	0.300186838	1.200058585
Colwellia_sp._Arc7-635	-6.724237289	-1.243867355	-1.669013489	-1.685809358	-1.859069193	0.258659152	0	0	0	-0.738872618	-0.176106073	0.000389409
Algicoccus_marinus	-6.689128221	-2.330193956	-6.127452573	-0.393577715	1.687681698	1.790235703	-1.67901763	0.881439867	1.2792055	-1.048091522	-0.352918995	1.545503891
Algoriphagus_sanaruensis	-6.688751604	-2.011941347	-0.730330707	-8.932715335	-2.02561836	-1.47356519	0.147433455	0.444212818	0.214527422	-0.301998438	-0.06217807	-0.265465239
Aquimarina_sp._TRL1	-6.611255389	-1.884441198	-0.033236574	0.029101119	-0.637932514	0.892040752	-1.016499026	0.25932556	1.1972355	0.251672103	-0.493641388	0.416656304
Lactobacillus_hokkaidonensis	-6.57197715	-3.703711168	-8.872229348	-0.382061661	1.20985792	0.484990587	-9.177646265	-9.177646265	-0.233001546	-0.905192204	-0.902579916	0.700041629
Taylorella_equigenitalis	-6.541434132	-3.684823	NA	0.308954587	1.878791803	2.144162267	0	10.71969684	9.561202011	-2.325131527	-0.283264335	0.769386072
Lactobacillus_apis	-6.537911314	-7.340691582	-5.149886767	-0.329970877	-0.631234896	-0.996595639	-0.288054664	0.565639555	0.279354537	-1.167012535	-0.824375935	-0.287363466
Candidatus_Arthromitus_sp._SFB-mouse	-6.531089871	-3.672660764	-3.580940959	-5.238995141	-1.117086036	-4.585992596	-0.682684148	-0.323545297	0.568087155	0.093287504	-2.88344412	-2.023691088
Pseudomonas_sihuiensis	-6.525469742	-0.94574389	0.253150242	1.594867496	3.160208125	3.606589104	0.064130533	0.638600991	-0.579847756	-0.941108864	-0.023599864	-1.216552617
Hydrogenophilus_thermoluteolus	-6.523409701	-0.609811483	-0.36265709	3.457591299	1.669571394	1.660100605	-9.222246695	-0.840115539	0.496674843	-0.441182843	0.633851696	0.061726072
Bacillus_sp._Y-01	-6.517257379	-4.259650265	-7.208812514	-1.678425464	0.857139628	1.337399504	-0.629608179	-0.146672778	1.056275561	-2.454204954	-2.17491481	1.209297929
Prevotella_sp._oral_taxon_299	-6.486725121	-1.030333932	0.67043499	-2.538830364	-1.399979424	-1.300645762	0.183895216	-0.332165968	-0.246206302	0.021461667	-0.171837631	-0.301636753
Prevotella_enoeca	-6.452137936	-1.652445422	0.112524992	-3.326796002	-2.842275703	-1.993996854	8.502740328	8.559465045	6.15711649	-0.089051005	-0.334674422	-0.372472581
Prevotella_ruminicola	-6.427094849	-2.863389144	-1.036243225	-2.912335683	-3.299957154	-2.813623351	-8.10567284	3.667466366	3.958335593	-0.243333294	-0.531335574	-0.467789497
Chryseobacterium_sp._cx-624	-6.361827056	-1.08614156	-0.576831528	3.085553132	2.224171643	2.564723821	0	0	0	0.064013768	-0.087778666	0.253416531
Nonlabens_marinus	-6.297095697	-4.055122907	-0.581297803	NA	-1.049360171	-1.640128294	-9.222246695	2.630707991	0.40180118	11.0425691	10.98108916	10.65523554
Spirochaeta_africana	-6.271608924	-3.52274521	-1.165700416	-1.897123412	1.437875561	1.672456891	-8.552496194	-1.869824586	1.038771812	1.693372038	0.638821164	0.523883292
Lactobacillus_acetotolerans	-6.251540367	-8.769481758	-6.6682854	-0.434347482	-0.590050114	-1.084532389	-0.656333716	-1.317206171	-0.482292596	-1.315205669	-0.830051279	-0.480607638
Limihaloglobus_sulfuriphilus	-6.230169772	-2.213018277	-0.139728285	0.252748912	2.005633896	2.224655473	0.330021622	0.191283104	0.717067246	-0.30624891	0.682252761	0.967310747
Sphingobacterium_sp._B29	-6.218173757	NA	-4.140376479	-4.224668933	-4.000875156	-5.055107468	0	0	9.477205768	-0.206709574	0.093465124	0.093804901
Sandaracinus_amylolyticus	-6.191984513	-2.110501075	-1.265317884	0.90054018	1.228599199	1.588144918	-0.430037293	1.094296327	-0.648604843	1.586158781	0.585162447	0.339376277
Nostoc_sp._NIES-4103	-6.185649778	-1.6694573	-2.658172772	2.020970422	1.362520141	1.364266957	0.083370495	0.622346791	0.914234252	-0.41478135	0.018291149	-0.539661674
Sedimenticola_thiotaurini	-6.097325383	-1.327256303	-0.727348337	2.676394832	1.129880283	1.166385388	-0.996795903	0.07956615	-0.475911109	0.027680343	0.472773131	0.742443172
Prevotella_fusca	-6.047847734	-3.54881156	-1.735252957	-4.201971954	-3.829911132	-2.91360757	0.969452327	0.617174306	0.174839554	-0.232904516	-0.534973399	-0.485552667
Neomicrococcus_aestuarii	-6.016322594	-2.952996659	-2.054746754	-0.854833632	0.38545232	0.621562511	0	0	7.157114468	0.612975227	-0.033770974	0.327059437
Lactobacillus_amylovorus	-6.00828355	-6.585164678	-6.616967897	-0.500839886	-0.558367997	-1.182758188	0	0	0	-1.393675008	-0.894088262	-0.377365562
Salinivirga_cyanobacteriivorans	-5.919358361	-2.515716022	1.286134339	NA	-3.686802799	-0.508827665	0.584296975	-2.746015034	-0.37450621	0.529471969	0.354134613	0.611376939
Bifidobacterium_pullorum	-5.905355262	-2.137076629	-1.913369756	-0.17363871	0.245781491	1.830749932	-1.66601531	-1.012427578	-1.316142028	0.209618989	0.40204714	0.804486979
Helicobacter_typhlonius	-5.879595027	-8.794677905	-8.147821372	-9.364839462	-19.7825621	-16.73477888	-0.307267872	1.137086666	0.511645976	0.039484494	0.005004344	-0.27194021
Chitinophaga_sp._MD30	-5.868533215	-1.796400611	-1.434947774	-1.878742421	-2.04714751	-1.454083844	-11.40939094	-2.782021211	-3.252271414	-0.162103258	-0.087733	0.34276578
Lysinibacillus_fusiformis	-5.845216113	-3.753682563	-5.4419603	-0.227698404	1.320656438	2.079429401	-0.754185502	0.129050837	-0.537921964	-0.258371725	-0.806621851	0.934329458
Fusobacterium_sp._NSJ-57	-5.82768592	-4.579426867	-0.502454122	NA	-1.637825615	-0.730348723	0.179966119	0.210041161	2.048244952	-0.309183915	0.126626575	0.093789256
Leuconostoc_mesenteroides	-5.803657576	-3.578176973	-4.566909862	-0.420133289	2.775478356	2.632400744	-1.439664038	1.362126463	0.168155952	-0.782223788	-1.951771256	0.43467786
Lactobacillus_jensenii	-5.796202889	-11.32014328	-7.012930607	-0.82190936	-0.84305156	-0.928967043	0.391002826	-0.508155573	-0.067461226	-1.313372116	-0.949933881	-0.580000491
Gloeothece_citriformis	-5.787959324	-1.516455697	-2.309268828	NA	-0.366342314	0.740691333	0	0	8.30544163	-0.141948465	0.55248696	-0.510780702
Prauserella_marina	-5.787205222	-2.257202382	-0.007884294	2.976701751	0.486426962	1.129843178	-0.470971973	0.387542543	0.904483019	-0.034340772	0.535305339	-0.358253512
Sphingobacterium_daejeonense	-5.755986183	-4.474804431	-4.988304569	-3.812952194	-3.363967363	-2.992107522	-0.18453702	-2.497811242	-1.551017001	-0.333779104	-0.177796485	0.206146131
Chryseobacterium_joostei	-5.749869427	0.152392619	0.331281094	0.02134037	0.716452997	0.838536577	0	0	10.52516841	-0.787728158	0.686747147	-0.621504032
Mucilaginibacter_ginsenosidivorans	-5.715990751	-1.348897943	0.07046708	-1.451499667	2.279081129	1.883894417	0	0	0	0.367471889	0.252991325	0.397450489
Flaviflexus_sp._H23T48	-5.713725534	-2.771096911	-3.047890301	NA	-3.450801779	-1.578427065	0.166573936	0.164650843	0.166990792	-0.139407052	-0.746862335	-0.504539488
Micrococcus_luteus	-5.712623997	-3.43945244	-0.913749305	2.662585306	1.490997913	1.456368106	0.336989695	-0.259193997	0.062703519	0.703475849	0.219203664	1.011960626
Chryseobacterium_indoltheticum	-5.700439718	NA	-5.713039755	-3.346485199	-0.198046805	0.963630201	9.163991757	10.35434857	11.14442882	0.174732845	0.574605708	0.474917828
Paenisporosarcina_antarctica	-5.689561254	-2.365380708	-1.51525971	-1.224306572	-0.579806944	1.89127378	-0.817945641	-0.317307249	-1.05451067	0.050516355	1.008475782	0.995338328
Brachyspira_intermedia	-5.681103233	-3.537224259	-4.36659261	-6.200489967	-3.479960676	-2.976488293	-0.278912629	-0.105241291	0.540360981	0.400417209	0.023419605	0.003624943
Sphingobacterium_sp._ML3W	-5.679135564	-1.900897574	-0.120945011	-5.701522261	-2.990395864	-1.569636621	0	9.110423278	9.810864	0.001028146	-0.201047471	0.020054812
Streptobacillus_moniliformis	-5.671218234	-1.546670566	0.191705517	-2.778163876	-0.992879828	-0.201426592	-0.666103992	1.378924672	1.27594783	-0.507669688	-0.094185349	-0.778187371
Lactobacillus_rossiae	-5.663144423	-3.323866912	-3.544733194	-0.777395863	1.427746113	1.045013782	-0.112368453	0.358125059	-0.121349752	-1.28717404	-0.958731751	0.384763314
Caldicellulosiruptor_lactoaceticus	-5.644554662	-2.150297138	NA	-2.889223558	1.622692032	1.625965475	-10.18217085	1.352749637	1.20584643	-1.458102041	-0.627189526	0.98519403
Pedobacter_sp._PACM_27299	-5.642954223	NA	-1.311477402	-1.519645802	0.217786986	1.529872094	-0.183035803	-0.269378204	-0.501973334	1.137503524	-0.266119298	1.609754962
Spirosoma_aerolatum	-5.642438264	2.005839983	1.878280657	-4.217346913	3.14454759	3.0495098	9.901416014	0	0	-0.123870123	1.025166087	0.058319755
Variovorax_paradoxus	-5.63334748	-2.770551306	0.814868946	2.342524999	2.674684	2.975999896	-0.857900292	-1.454562146	0.802487925	0.231079395	0.056934211	0.440625187
Nocardioides_sp._78	-5.629802004	-4.506688137	-1.821097718	1.117382772	-1.224873411	-0.535020413	-1.370491857	1.348031914	0.905482099	0.282321594	0.552505824	0.239823527
Thermosynechococcus_elongatus	-5.598353861	-3.640174037	-1.980562831	1.72173912	2.270535482	1.44640676	-0.049685236	0.661661781	0.850994143	-1.59800553	0.977568601	0.022082484
Corynebacterium_sp._NML98-0116	-5.567079796	0.892408845	-1.775039295	-2.099444158	1.171632333	1.62081556	-0.21951059	1.195107321	-1.082873672	0.868158205	0.835588574	0.297157129
Dyella_sp._G9	-5.562242424	-3.117227578	-1.198655178	-4.818621144	-0.206546798	1.026154777	8.246199041	0	8.451211112	-0.533851711	0.18338635	-0.386866692
Pseudolysobacter_antarcticus	-5.55672882	-0.927711139	-0.393342428	2.272701015	2.222016622	1.861378184	1.942448364	0.32868566	2.773337664	-0.319513916	-0.161932411	-0.245319044
Maritalea_myrionectae	-5.553869243	-1.770871034	-1.252944752	-0.770774565	0.576845173	0.170132539	0.683038148	0.930016824	1.295657489	0.921625503	1.363944542	2.000513658
Trueperella_bialowiezensis	-5.550793361	-2.466827964	NA	-1.30256277	1.075404748	1.318345583	0.037499097	0.74436128	0.385551261	-1.459795749	-1.901966725	0.858132844
Gemmatimonas_aurantiaca	-5.527941726	-5.339084908	-1.114923394	-2.110044283	0.565976882	2.675006865	1.533082725	1.969159336	0.698160579	-1.522125184	-0.324602129	0.244811069
Chryseobacterium_taklimakanense	-5.526881133	-2.074702321	-0.486815162	-1.454687058	1.054655523	1.287681715	0.666886595	2.88280288	1.835042471	0.43298849	0.044649897	0.413908265
Glaesserella_parasuis	-5.513346677	-4.25877385	-5.631991174	11.76534663	4.152104403	3.419589389	9.806477123	9.6794801	6.15711649	1.298653997	-0.743117405	-1.455012835
Proteus_mirabilis	-5.481032657	-1.947752237	-2.267668394	2.901623583	-0.447880756	1.672455661	-0.637904818	-0.031341683	-0.176321636	0.413409412	-1.273075089	-0.225972735
Nitrosomonas_communis	-5.426659419	-2.127242353	-0.880981711	2.792665606	1.092788116	1.894378793	-8.04736934	1.304974294	3.86395523	-0.152917102	0.478564208	1.414437171
Streptomyces_ficellus	-5.41110278	NA	-0.068966931	-0.162227546	NA	0.43061949	0.582546914	-0.456825247	0.162173669	0.307015577	0.305437692	1.111606901
Melittangium_boletus	-5.400648697	-2.221740295	-0.873700132	2.099198605	0.147636177	1.923769313	0	0	0	0.997169698	1.054998869	-0.040154482
Flavobacterium_sp._I3-2	-5.389614902	-4.358453971	NA	NA	2.882244243	3.645160931	-1.72774546	-0.500767674	-3.727748493	-1.025837915	-1.517843329	0.035093226
Rhizobium_sp._ACO-34A	-5.359200485	1.529010025	-1.189275484	NA	NA	NA	-0.187477992	1.078357777	1.537207117	0.789846436	0.894854214	1.612592391
Salinimonas_sp._KX18D6	-5.357826882	NA	-0.566786123	NA	NA	-2.329593286	-1.27794217	0.147527872	-0.212950194	-0.214198142	0.673762855	-0.173138714
Moraxella_catarrhalis	-5.324849517	NA	-5.552450229	-0.354389657	-1.736260292	-1.291907226	-0.460578381	0.491858326	-1.026214931	0.269381041	0.730068921	0.769492708
Flavobacterium_alkalisoli	-5.322516487	-1.541780578	-0.229850505	-6.031947172	1.898513342	1.373797384	-1.009461165	0.126199267	0.118722869	0.27955848	-0.03796082	-0.05615343
Dokdonia_sp._MED134	-5.317633981	-0.600221184	0.33587507	0.938382971	2.132725748	2.238970225	0.513679915	0.267555624	0.015309442	0.663163331	1.247349181	1.322497029
Arcobacter_venerupis	-5.309476354	-2.004098568	-2.075856677	-0.501552467	-0.948915644	-0.343839887	0.007782123	1.566912976	1.16804804	0.232524636	0.788514578	-0.056784521
Pseudodesulfovibrio_sp._SRB007	-5.303035694	-1.967656647	0.816065947	2.052931745	2.761725802	2.388882785	-2.424319707	-1.787104567	0.018851947	0.732145022	1.002010957	0.527819278
Methylomonas_methanica	-5.288339004	-4.216585425	-6.693853729	NA	0.319058691	-0.411901353	-1.180430333	0.56878	0.761410029	1.216160709	1.22517148	0.57503086
Streptomyces_harbinensis	-5.285132833	-7.09388911	-3.811509826	-5.77943422	-6.639930905	-7.210363092	-0.800196175	0.540781908	0.90919071	-0.202877011	-0.538727378	-0.562022457
Cytophaga_hutchinsonii	-5.283538472	-1.720875477	-0.197025475	-2.634220668	-1.182947198	0.655372754	-0.244960108	-0.302415495	0.772517006	0.428996628	0.972210897	0.437785067
Brevibacterium_sandarakinum	-5.283128979	-2.428118471	-0.029882768	NA	NA	NA	0.337908338	-0.925124548	3.796842252	0.690121169	2.692977662	1.046488865
Staphylococcus_auricularis	-5.278682509	-1.879489288	-4.014870161	0.774448622	-0.944290567	0.225403103	-1.036559277	-1.209449642	-1.007236519	0	10.85174904	0
Chitinophaga_sp._H33E-04	-5.277737986	-2.495133919	-1.772869374	-2.136625768	1.060146717	0.614178391	-9.39582543	-0.488934834	1.214867251	-0.209593135	-0.304928894	0.826663507
Microbulbifer_thermotolerans	-5.274166071	-1.287171781	0.641820528	NA	NA	3.112530428	-0.521362107	-0.046057136	1.478523682	0.530981543	1.484735053	0.596747081
Streptococcus_urinalis	-5.268542	-1.500357676	-4.547442812	-2.743696285	-2.444784843	1.092649288	8.246199041	0	0	0.790324543	0.061898836	0.877667087
Prevotella_oris	-5.267279771	-2.191985985	-0.571488266	-2.501864259	-2.630609033	-2.372850065	3.103064234	2.839040837	4.378684337	-0.193644823	-0.407477355	-0.382673469
Neorhizobium_sp._SOG26	-5.251617435	-7.966165563	NA	-0.753230076	NA	-1.695614805	-1.387665995	-0.353499441	-0.490236699	-0.450587981	-0.018009184	0.147553724

Deferribacter_desulfuricans	-5.231956822	-3.727432244	-6.313992591	-4.871060875	-1.642785419	-2.071546871	0	0	7.157114468	0.031569298	0.520860518	0.439924523
Leclercia_sp._29361	-5.196484067	-3.259963124	-0.103411985	NA	NA	NA	-0.629422717	-0.389211771	-0.702798098	3.896133741	3.142405328	0.615276132
Lactobacillus_kefiranofaciens	-5.174399681	-6.958618585	-3.26424589	-1.145820157	-0.846777135	-0.671395477	1.047214865	2.746845594	2.66312805	-1.200964046	-0.710309985	-0.193804536
Geobacter_sp._DSM_9736	-5.163970431	-1.075601849	-0.681082349	3.131614198	1.288610801	1.847831683	0	0	0	-0.306153816	0.463413166	0.832422703
Cupriavidus_necator	-5.151006703	NA	-1.671383081	-3.001229046	-4.364736245	-2.487968011	-0.20273624	0.506055788	-0.020081982	-0.632461415	-1.421672386	-0.594338294
Vibrio_mediterranei	-5.14827687	-3.47155475	-2.922675749	1.762673358	2.024019254	2.391595017	-10.81814275	-0.790734322	-1.954238362	-0.675636769	-0.876620905	0.83303399
Histophilus_somni	-5.146989279	-2.574305108	-4.559990511	6.913927706	1.289292835	1.504754253	0	9.352343634	12.94064762	-1.476475322	-0.595441456	0.846469437
Cruoricaptor_ignavus	-5.131969828	-0.849950455	0.007302336	-1.069711138	2.540896577	2.520256811	1.093062469	0.007642504	0.269866178	-0.515287601	-0.129436167	-0.731036722
Arachidicoccus_soli	-5.116602065	-2.992921979	-0.900130919	-3.488844346	-1.299843479	1.741976697	-0.580507856	-0.538622752	0.443315394	0.71795034	0.621503417	1.05838354
Prevotella_denticola	-5.093114905	-1.552838273	0.000624976	-3.231352527	-1.823264502	-1.351196058	0	0	6.15711649	-0.149004301	-0.352424131	-0.231498295
Beggiatoa_leptomitoformis	-5.081456435	-3.243019422	-0.938917321	-3.025132792	-2.965533801	0.682209463	2.830619454	0.054834119	-1.395381726	0.024223376	0.297547049	0.517788513
Niastella_koreensis	-5.058052713	-1.194238991	0.020215269	-2.443865361	0.750329341	0.950595242	-9.076815597	0.307281711	0.858483548	0.412247362	0.846078516	0.587937743
Lactobacillus_paracollinoides	-5.050698944	1.058560394	1.377137659	-1.050450481	3.981546612	4.256334989	0.104467128	0.583511713	0.426886557	-0.79755548	-0.119617158	-1.303742182
Nodularia_spumigena	-5.018382465	-0.034484663	0.495739795	-0.375546278	2.445093316	3.017348339	-1.427191182	-9.929931509	-3.772815019	-0.989519231	0.802533715	-0.279681321
Spiroplasma_corruscae	-5.000597018	0.187000856	-3.359418508	NA	0.285523361	-1.253819801	-0.76632692	0.139664362	1.501683802	0.740591584	0.638343489	1.089559569
Phaeobacter_inhibens	-4.996536145	-1.206084115	-0.533335236	1.314038155	0.876361343	1.851823175	0.322242642	-1.570652606	-0.356078927	0.526325017	0.561198151	0.201336495
Hippea_maritima	-4.995128152	-3.077898943	-1.200948619	-2.238828862	0.074763689	0.611136554	-0.178642412	-0.272662311	0.216429168	-0.508938617	-0.041196418	1.097567023
Empedobacter_brevis	-4.988351004	-1.542372473	0.270315503	NA	1.63740394	1.332329272	0.653879039	2.8428222	3.364247318	1.683686499	1.389764814	1.439700427
Rhizobium_jaguaris	-4.969484889	-0.167283729	0.323115941	-4.791420586	-0.203002527	1.067544299	0	0	0	-0.743098181	0.675701188	-0.418721038
Prevotella_melaninogenica	-4.967021522	-2.744619167	-2.317045122	-1.679856531	-3.410823106	-3.117956942	-0.562480295	-0.168365018	-0.522471543	-0.258339248	-0.579354013	-0.589306399
Xanthobacter_autotrophicus	-4.966144913	-6.461524521	-1.32319069	-4.898196407	-1.800502383	-3.117031009	0	0	0	-0.275876152	-1.20180113	0.096455802
Anaerococcus_prevotii	-4.96108789	-0.217515701	1.2677308	-3.11284816	0.998321472	1.118722399	0.748943938	1.228044013	0.729771881	-0.05407149	0.476204804	0.801882571
Cyclobacterium_amurskyense	-4.956920605	-1.754796781	0.020717086	-2.721706245	-0.189933414	0.926377137	-1.662181432	-0.272755668	0.955272557	-1.007303185	0.935336718	-0.158513319
Hymenobacter_sp._DG25A	-4.953559901	0.598989127	0.816226299	-4.382984205	3.001439816	3.163870072	-0.100687949	1.244345206	1.047279555	0.352360032	1.45597352	1.052093988
Prevotella_dentalis	-4.953392532	-1.64971231	-0.343235288	-0.736822768	-0.8317083	-0.608401002	-0.899921908	0.597349832	-0.646844589	-0.137793739	-0.339755195	-0.307327515
Moraxella_bovoculi	-4.935327862	-1.02400502	-0.004689762	NA	0.552656489	0.228877195	-7.757316664	-7.757316664	1.567682903	0.241631691	0.517593496	0.370984786
Bacteroides_sp._HF-162	-4.934813142	-1.027796568	-1.236616658	-4.325706429	-3.502355163	-3.249923415	-9.076815597	0.315111043	-0.919696075	-0.163700949	-0.175943022	-0.290666581
Corynebacterium_sp._2184	-4.925465573	-1.96915439	-0.343887436	-1.648906419	1.018677024	1.17443716	8.502740328	8.559465045	8.30544163	-0.007358792	0.086941632	0.255039365
Oceanobacillus_sp._160	-4.924705075	-3.751882512	-1.035323129	-1.507900356	-0.22410739	0.650174927	-0.672599217	-0.113136814	0.301759599	-0.643494407	0.312166524	0.921091262
Muribaculum_sp._TLL-A4	-4.923149888	1.194305414	-0.019902898	-1.178120184	1.72713201	1.45426413	0.180664338	-10.66339799	-1.123182756	0.266541162	0.843195209	0.825996115
Vibrio_fluvialis	-4.914760755	-1.631218829	0.159586586	-2.19064778	0.173486875	0.887058095	0	0	6.15711649	2.172351739	3.674708017	0.613817363
Pedobacter_sp._HDW13	-4.894773996	-1.290539922	-1.007459887	0.369434682	0.928728179	0.616220025	0.610741296	0.016782891	0.344948196	0.730953416	0.266688028	1.216302174
Streptomyces_violaceusniger	-4.89281541	-1.535741764	-0.12758967	-0.12900758	-1.354616714	0.820031608	0	8.559465045	9.104480817	1.688948061	0.127865195	0.401493332
Bacillus_safensis	-4.881208966	-3.700809119	-4.713052715	-0.411806998	1.801017989	1.410606888	0	9.968407603	9.104480817	-0.981256935	-0.302970908	1.382587513
Ereboglobus_luteus	-4.867364031	-0.3695001	0.052743169	-0.443183143	2.900718603	2.049265575	-0.682708721	-0.207827773	-0.372009238	0.385772528	0.199890541	1.08286718
Corynebacterium_timonense	-4.864688097	-3.723644216	-2.012365265	0.322597969	-0.427169197	-1.230332761	-0.054339302	0.055952842	0.5024842	0.79474728	-0.560540131	-0.74760905
Desulfovibrio_hydrothermalis	-4.856867321	-1.725337698	-0.971798167	-2.099671361	-2.728442921	0.183139915	0	0	0	0.167060164	0.091254153	0.699886789
Caldisericum_exile	-4.846197554	-3.253855523	-2.679234966	-4.01693456	-3.524516287	-1.197431099	-0.129908208	0.439507383	1.512661284	0.23190323	0.385965727	0.771118571
Shewanella_violacea	-4.839535328	0.302634444	0.274480932	3.677007175	2.777607579	2.980879533	-1.045967409	-0.458965089	0.937084595	1.005150407	2.312947059	2.621227855
Agrococcus_carbonis	-4.82853321	-1.423391747	-0.56526401	NA	0.210645857	1.18839175	-1.162329925	-0.803222485	0.937073353	-0.468501516	0.4635411	-0.027319882
Siansivirga_zeaxanthinifaciens	-4.816583708	-3.145927459	0.291777391	NA	-3.332635906	-1.546392043	0.023610177	-8.10567284	2.214202463	0.632978243	1.063227524	0.024016881
Aequorivita_sublithincola	-4.806201458	-1.139524308	-0.419081901	-3.575882328	0.542457514	0.743606722	0.118042507	-0.357059243	0.091900916	0.11590026	0.760427636	0.800856362
Streptococcus_ratti	-4.80329359	-2.261703984	-2.227536752	-0.446735061	0.337267989	-1.490826052	0	0	0	-0.373239693	-0.249037443	0.465026925
Mariprofundus_aestuarium	-4.792058735	-5.519374159	-2.006304577	-1.396012674	-1.373095736	NA	0	0	0	1.126840118	1.17142235	2.267899783
Pseudomonas_monteilii	-4.79057347	-3.997024347	2.12556191	3.090141125	3.96727994	3.456956057	0	0	0	-0.30882496	-1.013470815	0.228777807
Aquimarina_sp._BL5	-4.785043715	-0.524476961	2.791322528	NA	-0.552657187	0.806483466	0.49353853	-0.133006533	0.138336987	-0.022242503	0.503140268	1.29789167
Streptomyces_sp._So13.3	-4.784609486	-4.492055993	-0.425694261	1.804922815	-0.562283954	-1.103566082	0.477417675	-8.552496194	1.877956357	-0.073580972	-0.931629716	1.009775335
Formosa_sp._L2A11	-4.782822745	-2.683662822	-2.97225111	-3.382584153	-2.45258539	-0.933456986	0.344132519	-0.239670842	1.613148144	0.269725573	0.730991103	0.623974872
Fibrobacter_succinogenes	-4.774703062	-1.925308956	-0.61283006	-3.111193331	-1.101311156	-1.094376035	0	0	9.154818109	-0.280758965	-0.535049862	-0.553566746
Thiomicrorhabdus_sp._G2	-4.774482982	-2.065284907	-0.16030856	NA	-1.049393752	-0.241426581	8.246199041	0	0	0.487076227	0.555420299	1.742307431
Moraxella_osloensis	-4.740570935	-3.434412892	-3.203273796	-1.108859735	1.062888526	1.096290664	0.211946682	0.550552304	0.522083656	-0.564307428	-0.0641254	1.197401106
Panacibacter_ginsenosidivorans	-4.731743291	-2.864464551	-3.427962543	1.082248412	1.885964757	3.038436182	0.393397263	-9.764871591	-9.764871591	1.262563187	1.275105527	2.087905812
Georgenia_sp._Z443	-4.713073418	-3.317457919	0.252084445	NA	NA	NA	0	0	0	1.784518631	0.325851458	1.43589479
Geodermatophilus_obscurus	-4.709920771	NA	-2.520284443	0.705669464	-0.851096145	-0.018696094	0	8.627369725	6.15711649	-0.563158083	-1.613365282	-0.789679507
Bradyrhizobium_sp._SK17	-4.707962167	-1.253632924	-1.422919141	NA	NA	NA	0.956245608	1.191605234	0.263287233	0	0	0
Streptococcus_oralis	-4.707254856	-3.891828023	-4.002463113	-3.413772387	1.298393884	1.59800031	1.065803271	1.407806648	3.066664726	-1.263671166	-1.317976606	0.594825341
Streptomyces_antibioticus	-4.707091555	-2.641503213	-1.370143943	NA	-1.56726418	-0.046621754	-0.746345224	0.238650892	0.168959299	10.16574867	11.13442632	9.897845456
Corynebacterium_diphtheriae	-4.704405377	-0.834637044	0.433383512	0.23625127	0.811328197	1.571750204	0.726931858	0.272527078	1.616360265	0.239768564	0.854817757	0.381517843
Methylacidiphilum_fumariolicum	-4.67914494	NA	-5.673659401	NA	0.960764837	1.871266686	-0.934124045	0.395127251	1.335900868	0.349281203	1.141423187	0.133757037
Streptococcus_sp._A12	-4.658986381	-1.360566762	-0.578117222	NA	NA	NA	-0.949532922	-0.58982669	-1.13458089	9.392317423	10.57553925	7.539158811
Pradoshia_sp._D12	-4.651828277	-0.307436289	-1.333026283	-1.374395515	1.519005419	0.577190863	-1.85660644	-0.703245719	-1.741963943	1.346454423	-0.673962267	0.62533357
Mucilaginibacter_rubeus	-4.651151654	-1.509771693	-0.432711529	-2.568239709	-2.259631822	-1.115885357	-0.522599072	-1.108317073	-0.842264416	-0.057505422	-0.4937846	-0.134650859
Marinobacter_sp._Arc7-DN-1	-4.645209709	-3.92290152	0.096792609	NA	-4.095086341	-0.216231898	0.416890327	-0.401130761	-0.413296606	1.70386242	0.814710433	1.210428357
Candidatus_Babela_massiliensis	-4.641148597	NA	-0.872964273	1.550411953	0.718790366	1.612055308	-2.604853293	-0.592032688	-1.519688115	-0.255104242	0.093801175	0.191799765
Mobilicoccus_sp._NJES-13	-4.638222393	-3.429209001	-1.92137216	NA	-0.029170902	0.822912962	0	0	0	1.205616709	2.336035665	1.172702086
Mycolicibacterium_smegmatis	-4.63024338	-3.983880335	-1.93134851	0.221564476	-0.289935863	0.422261826	-9.36433386	1.451633761	1.107341354	-0.532374286	0.237535677	-0.905874386
Komagataeibacter_medellinensis	-4.618370099	-1.583238409	-1.692370681	0.749691936	0.930060899	0.686472322	-0.208175147	-0.391827543	0.806430926	-0.095346031	-0.681073742	-0.713189639
Chlorobaculum_limnaeum	-4.616620338	-1.563378686	-0.06087256	-3.24727516	2.0306159	1.626796486	-1.944371647	-2.40788512	-0.448226488	0.37241584	0.288788258	0.501103941
Micromonospora_sp._28ISP2-46	-4.614860825	0.031656347	0.722886857	-2.443347895	0.002585476	-0.35464367	0.085562039	-0.071912906	0.965128319	1.970531589	1.803180911	1.845001087
Microbacterium_pygmaeum	-4.589160045	-0.815340727	-0.310863308	-1.449861467	3.506448682	3.128253868	0.408575093	-0.431471716	-0.270274249	-0.178777133	-0.310250878	0.307086881
Youhaiella_tibetensis	-4.58590145	-2.179058695	-0.161210258	2.628355096	0.947940409	0.327894195	0.587598455	0.400606628	0.472758457	0.69779429	1.006643798	0.848626439
Bifidobacterium_dentium	-4.581921131	-3.946799582	-0.738017082	2.12733179	0.777967019	-0.822756418	0	9.426646347	0	0.692048181	-0.194727902	0.378233678
Spirosoma_sp._TS118	-4.577084322	0.361360301	0.628863413	-1.155225406	0.119729695	0.654135239	-10.76185038	-2.65142449	-2.162952985	0.527921353	0.101138562	0.401809115
Aeromonas_encheleia	-4.55733382	-2.015032472	-1.488146037	3.303969311	1.498390529	2.21762241	-10.83906203	-1.878532946	-0.010782713	1.19753888	0.482300302	1.447789819
Chlorobaculum_parvum	-4.54765054	-1.016262193	-0.906600076	3.80618112	1.837935601	1.937617297	0	0	0	0.13054279	0.47878033	0.302611571
Capnocytophaga_sp._oral_taxon_864	-4.541781677	-1.60472707	-2.361357803	-6.515042724	-1.267906378	-1.262180215	-0.138253479	0.335867885	0.71507638	-0.41453955	-0.646714437	-0.392260441
Pontibacter_korlensis	-4.541435125	-1.859742114	-0.063232625	-4.197810449	-1.280233175	0.951029933	0.029608342	-1.185083852	0.384745216	-0.337480012	-0.211408863	0.470608322
Bifidobacterium_pseudolongum	-4.541177011	-1.372686422	-2.006778667	-2.555211957	0.33849307	0.349239868	0.072706788	0.64861652	0.85725622	0.466221602	0.761284631	0.464512266
Oscillatoria_acuminata	-4.540556475	-3.814400692	-4.579689789	0.71152274	1.805009005	2.380578681	8.157119522	0	0	-0.126593695	-0.021087651	1.228346674
Euzebyella_marina	-4.531679077	-1.482287741	-0.106439145	NA	2.16417034	1.84932924	-0.637613075	0.910295009	1.048309875	0.423460033	-0.077908131	0.206072843
Flavihumibacter_sp._SB-02	-4.529150921	0.022517215	0.708597081	-6.518325308	1.868682276	2.0646203	0.593279204	0.923034689	1.163316145	0.611797518	0.647311232	0.964098556
Brevibacterium_sp._WO024	-4.508908261	-2.186980166	-1.857895368	NA	NA	-1.704314808	-0.244521921	-1.976846681	0.160659419	0	0	0
Corynebacterium_sp._NML93-0612	-4.507972148	-0.726770596	-0.282255083	5.263931545	2.945949379	4.153353002	1.201476944	-0.105208279	-0.007916667	0.175376487	-0.355011946	0.605705344
Bacteriovorax_stolpii	-4.507816813	-1.264782681	-0.276981777	1.276984525	-0.283239609	0.141355849	-0.709654789	1.232020642	1.762017698	-0.007953617	0.73731772	1.029397495
Candidatus_Arthromitus_sp._SFB-rat-Yit	-4.507583665	-2.887636423	-2.488244903	-0.583162543	-0.824685064	-0.703551389	-2.213703571	-0.531933584	-0.247304583	0.334928797	-0.197983685	-0.623142074
Ruegeria_pomeroyi	-4.506307581	NA	-0.990607743	-0.509977786	-0.069673528	1.141944422	8.129283017	8.627369725	9.104480817	0.682586498	1.972589132	1.063603046
Streptococcus_iniae	-4.506025363	-3.938439431	-3.729782036	-1.982488989	-0.096637257	0.880506994	0.221178584	-0.897927105	0.587078452	-0.510619114	-0.331005656	-0.540963335
Chryseobacterium_sp._NEB161	-4.503494503	-2.978626349	1.102602211	0.600071575	-0.826970815	1.418254912	0.958396014	1.551333448	1.904422825	0.423487759	0.655422921	1.806355525
Hymenobacter_sp._NBH84	-4.500471165	-0.915307969	0.250240015	-5.668910124	1.23925889	1.462563774	0.936094109	1.251598766	2.052357384	0.076705831	-0.099904358	0.678623407
Brevibacterium_aurantiacum	-4.491853096	-0.96829114	-2.0489096	3.569155698	1.749487514	0.490295175	-0.715037345	0.46265341	1.554897591	-2.007003495	-2.611741533	0.04265652
Gallaecimonas_mangrovi	-4.487825996	-1.381966545	-0.029602717	-6.280760484	2.369479322	1.806561223	0	0	0	0.52910291	-0.036898506	-0.038974434
Sphingobacterium_sp._DR205	-4.481277261	-1.101020559	-0.410827882	-1.694499754	1.218620273	1.244862898	1.268398387	1.205296092	3.122900013	0.468242319	0.395152	0.635425362
Castellaniella_defragrans	-4.478406399	-0.279210553	1.263714959	2.520059984	NA	1.029199976	-8.04736934	1.579998561	2.670778195	0.834168775	1.223953715	2.460097171
Streptomyces_lunaelactis	-4.476746204	-1.806089955	-1.311839277	4.580533656	2.634940369	1.318607737	0	0	10.87686223	-0.041588807	-0.114244875	-0.550938777
Candidatus_Saccharimonas_aalborgensis	-4.472336633	-1.252942955	-0.432173092	-6.93962964	0.138817775	0.290838742	0.450161478	0.938840044	1.986846239	0.610908497	0.673254357	0.142157371
Conchiformibius_steedae	-4.46760555	-1.016394438	0.387652277	-1.219101743	-0.177423712	-0.660074151	0.31368195	0.231308797	1.388084147	-0.052641024	-0.924748493	0.118421523
Chryseobacterium_sp._POL2	-4.464703348	-1.369855165	-2.143065758	NA	-0.23296812	-1.065567921	1.002262825	-1.067220377	-3.020529775	0.379097704	-0.707346083	0.508997283
Hymenobacter_swuensis	-4.452243012	-0.331942642	0.307131976	-3.923639043	1.772816923	1.939641614	1.313239812	3.737684838	3.706898219	-0.332368481	-0.240938818	-0.332627976
Muricauda_sp._501str8	-4.448503519	-0.453796524	0.100318389	-0.176951395	-1.358516695	0.399476948	-1.972498529	-0.56276661	-0.770038394	-0.508259699	-0.753634371	-0.155190551
Arthrobacter_sp._FB24	-4.435339515	-2.008371064	-0.039231774	NA	2.799808544	2.032242325	0.669175991	2.638641992	0.391194822	0.16668716	0.380937532	0.193879496
Halomonas_sp._KO116	-4.433286321	-6.244429504	-0.197355787	NA	-4.970116092	0.604041257	-0.442970013	0.898000253	0.764044137	-0.382548221	-0.265428561	0.418620641
Leptotrichia_sp._oral_taxon_212	-4.428353253	-0.344251625	1.319636373	-3.547841691	-1.42275303	-0.717890089	0	0	0	-0.057445278	0.230857433	-0.130291742
Psychromonas_sp._CNPT3	-4.426593649	-3.966647792	-4.194534893	-1.731267708	2.101239592	2.314542067	-9.883475721	0	-0.558476154	-0.52240593	0.236752418	0.623451218
Gramella_fulva	-4.425411362	-0.663580698	0.035516828	-1.011752141	2.631146039	2.207632143	1.471115754	2.078774742	0.426317535	0.858598972	0.48195672	0.658905104
Rhodoferax_ferrireducens	-4.424339565	-2.911785559	-0.317892844	-1.821344019	-0.403659719	1.528470352	0	9.426646347	0	-0.698200399	-1.225815609	0.259003799
Micropruina_glycogenica	-4.399318128	0.767155449	1.363947792	1.548541662	2.223383929	2.000598405	0	10.67307052	10.4295009	0.872380988	-0.352499794	0.580058319
Pontibacter_sp._SGAir0037	-4.399262715	-0.50160472	0.020468135	0.447501395	1.643036432	1.583986546	0.790558028	-0.629882474	-0.108085257	0.485146284	0.627772164	0.967486624
Acetobacter_pasteurianus	-4.385038139	-3.327552644	-2.95106545	NA	-3.536888348	-1.016553066	-0.411565614	1.357676313	1.318790003	0.305062894	-0.061262031	-0.03829346
Herbaspirillum_sp._meg3	-4.37934929	-1.484124756	-1.574070687	NA	-1.566353493	-0.533852124	0.332769986	0.645852988	0.210441268	-0.898825189	-0.625308617	0.070930574
Campylobacter_gracilis	-4.367040827	-1.057369931	-0.057808015	NA	1.479271684	1.468366797	-2.063470545	-1.108325309	-2.467072873	1.100958976	0.037480867	0.607822607
Paraliobacillus_sp._X-1125	-4.36475794	-2.930737338	-0.34813411	NA	1.585222563	1.741561781	0.801968202	0.172230362	2.215437305	1.6736881	1.069067839	0.0016949

Lactobacillus_helveticus	-4.363451672	-9.217490385	-8.247337531	-0.457377771	-0.551091078	-1.198860076	-1.713181778	-1.973485082	-0.290050447	-1.372675693	-0.696860727	-0.36464024
Campylobacter_sp._RM6914	-4.36320049	-2.070624649	-0.158280556	NA	-3.653109643	-1.392735523	0.144148204	0.510643319	0.366013904	-0.440886618	-1.185499701	-0.665080146
Flavisolibacter_ginsenosidimutans	-4.355756023	-3.93176163	-1.438520224	NA	-0.67826432	0.489406804	0.138707323	-1.298525123	0.260814821	-0.086849516	-0.341254004	0.367680993
Hydrogenovibrio_marinus	-4.342480541	NA	-3.121881402	0.941828533	-0.711190696	1.556906719	-3.440140667	0.105249696	-0.265540216	1.332276448	1.112644378	1.031820931
Halomonas_meridiana	-4.335009436	-2.353723156	1.599847144	2.826305501	3.411040608	3.215117091	-9.177646265	-9.177646265	-9.177646265	-0.264180809	0.448119823	1.531148889
Bifidobacterium_choerinum	-4.331621685	-1.28208783	-2.146758458	-2.534964061	0.358756509	0.361013167	-1.671696952	-10.81959634	-2.018990832	0.418940159	0.730611829	0.39343182
Pseudoxanthomonas_spadix	-4.323037434	-0.582374002	-1.582645727	0.551388645	0.930280275	2.454783555	9.832602079	9.39192664	10.81523918	0.151826742	1.382320088	0.907477271
Gemella_sanguinis	-4.3216254	-0.526064775	0.011558105	-5.306764747	-1.578743104	0.109864068	0.647049727	1.26993707	1.949745766	-0.672512238	0.456562514	1.053338352
Adlercreutzia_equolifaciens	-4.297837479	-4.1039966	-1.185476906	-3.451033887	0.583070752	0.992358683	-0.515533767	0.043115832	-0.108343197	-0.224774882	-0.43880144	-0.154033121
Cyanobacterium_aponinum	-4.297040805	-1.668241397	-1.313095241	-4.047661155	-1.567334019	-0.349822317	-1.110563791	-0.775739701	-1.593061385	0.627859554	0.924504641	-0.687669951
Duncaniella_sp._C9	-4.287494751	-3.939928711	-0.132375543	-0.73317339	0.929809743	1.549632576	0.119113178	-0.608848037	0.201569576	0.342915421	0.049333611	0.533515429
Leucobacter_sp._HDW9B	-4.280231263	-2.990419669	-0.001151623	-1.671735222	NA	0.433215046	-0.596359212	0.14289246	-0.553698839	-0.429348975	0.002844791	-1.365858738
Xanthomonas_citri	-4.2765279	NA	-1.310469227	NA	-2.13087551	-0.648048915	-1.17024244	-1.66030866	-0.691346034	-1.562291671	0.272938069	-1.844062639
Thioalkalivibrio_versutus	-4.276089193	-2.812792635	-0.552190502	-3.421293025	0.920558015	0.232431856	-0.912537159	-0.069262662	0.622718088	0.377051761	0.285541262	0.641294905
Spartinivicinus_ruber	-4.26668526	-1.234870448	-0.952576669	0.83348728	0.756545963	0.645217595	-8.091313724	0.019112164	0.532732323	1.205043015	0.664559882	0.954461568
Herbaspirillum_rubrisubalbicans	-4.264662404	-3.78790912	-4.877639281	NA	0.657489441	-1.773510354	-1.762304771	-2.175209146	0.590476627	1.259950953	3.226455942	0.474607726
Hymenobacter_sp._PAMC_26628	-4.259633827	-0.411524885	1.047786121	1.617621889	3.448705676	3.36836274	-1.036700308	0.308115644	0.826876633	0.14296134	0.309533973	-0.173375137
Chryseobacterium_shandongense	-4.253863313	-0.229106022	-0.407752347	3.476241013	1.339183069	1.879808554	-0.971059161	0.115610634	1.676027405	-0.097054659	-0.017616533	0.174962579
Hymenobacter_jejuensis	-4.25380408	-1.38177542	-0.107960291	0.481449071	1.879752889	1.409249746	-1.485678971	-0.715180333	0.345061661	-0.234018227	-0.206350392	0.283903239
Legionella_sp._TUM19329	-4.250209357	-6.8008714	-3.401609946	-0.555935277	-2.693887889	-0.343003185	-2.128047125	-0.239114073	-0.002469521	-0.196498992	1.051178724	0.85863396
Corynebacterium_geronticis	-4.24659477	NA	0.512037563	NA	0.934980208	NA	-2.101722103	-0.540371525	1.960726537	-2.703709116	-0.133020234	-0.596684508
Gimesia_maris	-4.242207066	-1.526825608	-0.142671392	0.162122413	1.835099913	1.450490585	0	0	0	1.937312902	1.135023508	0.971318381
Eggerthella_lenta	-4.234112665	-2.59175364	-1.093690143	-1.849114631	0.007213137	0.978789291	-0.81792907	0.260672835	0.102667406	-0.1076566	0.156567261	0.485124943
Pseudoxanthomonas_suwonensis	-4.221386708	0.672916454	1.571125866	0.475269375	2.911952942	2.49822394	0	0	0	0.374109703	0.863386565	-0.384928364
Parvibaculum_lavamentivorans	-4.22107327	0.099945754	-2.600579218	0.653617412	-3.958679898	-3.086513566	0.478374698	0.424720707	0.769591127	2.937647771	2.284533914	2.912565855
Prevotella_scopos	-4.218910227	-1.768680827	-0.471576043	-7.423700698	-1.811371444	-1.679910905	0	10.00842862	7.157114468	-0.317854131	-0.472895142	-0.214011143
Sphingomonas_sp._HDW15A	-4.210359866	0.255626277	0.418727347	NA	2.880100788	3.508762748	8.246199041	0	8.904155902	-1.521914937	0.679084398	-0.033886892
Corynebacterium_uterequi	-4.206714504	-0.497539976	-0.923650527	NA	NA	NA	-8.076815597	-8.076815597	-8.076815597	1.624855228	3.01564796	2.430122666
Proteiniphilum_saccharofermentans	-4.199867999	-1.29575546	0.123195034	0.467900052	0.857088572	1.141642354	-2.401532172	-0.084068907	0.242324018	0.194208	0.568023416	0.665638005
Flavisolibacter_tropicus	-4.193743621	-2.413850584	-1.16571275	-5.795566206	NA	-2.642436447	-0.150931607	-0.510684452	0.353906538	-0.03190752	0.556431485	0.251425164
Saccharopolyspora_coralli	-4.192944557	-1.252416365	-0.739947397	4.634088141	2.879023802	3.16590495	-8.076815597	-8.076815597	-1.919699107	-1.197423874	-0.760761254	-0.501887471
Teredinibacter_turnerae	-4.18662788	-1.355175419	2.286767711	NA	-1.914468047	-0.034498751	1.049781398	0.074873531	0.071549852	-0.039128115	-0.055889324	0.066840145
Candidatus_Planktophila_vernalis	-4.180201287	-2.383318961	0.05909847	NA	-1.151793645	0.257773275	0.173850512	0.005871018	0.621486381	0.755608498	-0.166150068	0.435477096
Lactobacillus_acidophilus	-4.167642925	-6.261517021	-5.553741421	-0.26874073	-0.836476569	-1.137733488	0.208853326	2.22846237	1.948940427	-1.327212363	-0.781684777	-0.41272449
Sulfuritortus_calidifontis	-4.165738789	-5.041080549	-1.477175251	0.157063245	1.56113776	0.713570243	0	0	8.451211112	0.165981586	1.575692856	0.173996889
Pedobacter_suwonensis	-4.1620176	-2.132253891	-0.653209872	NA	2.122496471	2.181156442	-0.877873186	-0.247487261	-0.718257869	0.311522683	1.175509515	-0.133761004
Petrimonas_mucosa	-4.152140225	-1.9012017	-0.356983261	-3.186047875	-0.875992807	-0.566168371	1.339932515	0.779284296	1.96321377	-0.177250369	-0.435522276	-0.241987131
Gluconobacter_oxydans	-4.146286489	-2.770219336	-1.768818351	-3.485259985	-0.118121455	0.298118821	0	0	0	0.062307878	-0.293417671	0.25250691
Caulobacter_flavus	-4.140793099	0.070810526	0.100582426	NA	0.956625458	0.447080516	-0.41856471	0.203933907	-1.077580398	-0.133440804	-0.516160583	0.022758207
Spirosoma_pollinicola	-4.113816608	-1.166510337	-0.266435063	0.719445103	0.405096859	0.267199533	-8.10567284	0.004753048	0.375210239	0.149961921	0.157928499	0.237649962
Nonlabens_sp._Hel1_33_55	-4.110955646	-3.227089233	-1.731929805	NA	1.613559669	1.771394181	0	8.382131157	0	1.157556835	0.531787907	1.421811893
Helicobacter_bizzozeronii	-4.109216629	NA	NA	NA	NA	NA	-2.223339333	-1.182560524	0.166877593	0.30434415	0.182752219	-0.017974129
Fibrella_sp._ES10-3-2-2	-4.108766	-0.131397889	0.86971868	NA	3.08627427	2.97985033	0.913389364	1.338382312	-0.104816474	-0.347674849	-0.172622455	0.371905264
Hymenobacter_sp._BRD128	-4.098035069	-1.325515403	-0.592386128	-2.241015972	1.250327569	1.773546028	1.17826396	0.790198687	1.772752236	-0.403662885	0.411671786	0.022325349
Bernardetia_litoralis	-4.096612806	-0.61949334	-0.193889388	-1.389988453	2.792628962	2.461162595	0.695672288	0.576664267	0.132096379	1.275191359	1.5070516	0.808051119
Adlercreutzia_sp._8CFCBH1	-4.093604389	-4.05736767	-1.148517419	-4.839712443	0.552364108	0.987220064	1.187608577	0.295517639	1.046978767	-0.232746428	-0.410458117	-0.125217436
Leisingera_methylohalidivorans	-4.092096415	NA	-2.77016832	NA	NA	NA	0	0	0	1.072434017	1.857868801	1.375717106
Mesorhizobium_sp._8	-4.089078401	NA	0.794263121	NA	1.058495649	0.387413252	0	0	6.15711649	0.605834327	1.366947032	1.104515404
Streptococcus_sp._NSJ-72	-4.087921495	-2.794662375	0.234396465	NA	0.546123931	-0.526204301	-0.790936107	0.93883445	-2.583426183	1.884306005	-0.467418878	0.297629922
Bacteroides_uniformis	-4.086983803	-1.301468539	-1.736279958	-5.210326772	-5.447882043	-5.819014136	0.005639245	-0.100653575	0.118540375	-0.254751316	-0.285659886	-0.386766187
Myroides_sp._ZB35	-4.079753221	-0.822175537	-1.786181278	NA	NA	1.870116624	0	0	8.451211112	0.65542541	2.298694599	0.191782746
Gracilibacillus_sp._SCU50	-4.078569692	-2.441191531	-3.092769203	-4.285010022	0.195355592	0.918783272	0	9.110423278	0	0.607338382	0.824220515	1.030894551
Alkalitalea_saponilacus	-4.074355998	-2.150411375	-0.128128517	-5.020470131	-0.570720716	0.506437623	0.171410734	-0.792678102	-0.525431928	0.776862766	0.796417254	0.766278339
Bacillus_cytotoxicus	-4.073911007	-1.480533489	1.859017449	0.318437362	-0.960569119	-0.169643253	0	9.3781973	0	0.323923906	0.4730583	1.580098425
Elizabethkingia_bruuniana	-4.069968201	-2.128915137	0.071655957	1.773732261	1.961507575	2.802451741	9.648765614	9.3781973	10.9898486	0.899415419	0.12941283	1.935503896
Staphylococcus_lugdunensis	-4.064388555	-2.22760877	-2.615646661	1.218082259	1.655668498	0.743037286	-0.075756352	1.299854207	0.332364745	-0.389447077	0.64000054	0.606182457
Candidatus_Nanosynbacter_lyticus	-4.06436554	-1.304144594	-0.305582682	-7.792266894	0.384697565	0.148870044	-0.301129318	-9.177646265	-9.177646265	0.640044419	0.540398739	0.067347031
Opitutus_sp._GAS368	-4.063571615	-0.570854518	-0.732781275	-1.210472495	1.520096422	0.87689387	-0.191685297	-2.089688031	0.693051073	1.16121902	0.720945818	0.741504789
Mycobacteroides_chelonae	-4.060265111	-1.186438149	0.618122034	NA	-0.046801441	1.700996265	-1.313459422	-0.704600586	-0.205434697	1.308996702	0.914361604	0.58600405
Candidatus_Cyclonatronum_proteinivorum	-4.059702649	-1.661202838	-0.779907917	1.794262854	0.204395363	0.661690886	-1.086182743	-1.205856311	-1.162300569	-0.111256751	-0.309303556	0.124467724
Acetilactobacillus_jinshanensis	-4.048185377	-1.3200118	-0.478117092	-5.381899116	0.429539639	-0.144029775	-0.678246076	0.282506002	-0.474154758	0.917692654	0.673461866	0.132803916
Paenarthrobacter_ureafaciens	-4.045514659	NA	-3.349064225	1.320089095	-0.858325629	2.502392229	0	8.110425888	8.451211112	0.398875706	0.030767589	-1.558322552
Lactobacillus_lindneri	-4.044394119	NA	-0.137503524	-3.946138416	-2.925528137	-0.358151849	-9.800605508	-9.800605508	-9.800605508	0.212287889	0.106959932	0.399146512
Alloprevotella_sp._E39	-4.032421478	-0.951077792	-0.098432825	0.712908011	-0.338616816	0.129340232	-3.44545148	-1.159192168	-1.777014172	-0.018323368	-0.181236937	-0.076155186
Lactobacillus_bifermentans	-4.027975573	-2.015069396	-3.098264016	1.134736228	0.691779136	0.134301092	0	11.27347509	9.311646154	-1.169794005	-0.36893772	-0.26706836
Mariprofundus_ferrinatatus	-4.024880058	0.053175552	-0.071269666	-0.081932476	-0.322209569	-0.861626813	0	0	0	0.592152507	0.041326153	0.228494853
Mucilaginibacter_sp._F39-2	-4.018520661	-1.01336457	-0.740795245	4.000024693	1.922822945	1.959736459	0	0	0	-0.010943395	0.05667547	0.119068998
Flaviflexus_sp._dk850	-4.018403207	NA	-3.006752848	NA	3.071553261	1.58821915	-0.644359121	0.099392287	0.472824331	-1.207450012	-2.74711306	0.793046641
Agrococcus_sp._SGAir0287	-4.017073513	0.352426206	-1.046570411	NA	NA	NA	0.003795006	-0.433423182	0.506251151	1.116665816	-9.765977233	0.578318675
Gramella_forsetii	-4.014305348	-7.991484387	-3.056273895	-1.550492747	0.790772038	1.32792291	1.893162522	1.960797786	-0.101116062	0.53721375	1.055799146	0.777284008
Rickettsia_typhi	-4.012056018	-2.235289415	-1.385984535	NA	NA	0.00982452	11.43945172	10.98338556	12.18075767	-0.591156258	1.511179817	0.668353906
Alysiella_filiformis	-4.010178893	-2.015232121	-2.962547771	-3.27687506	-0.398817147	-0.598734535	0.733941569	0.828532313	0.81871379	1.868670789	2.836362296	1.809373585
Neisseria_shayeganii	-3.993651908	-3.831431216	-0.866073197	3.210804447	1.936088236	-0.596550475	-3.444641167	-1.485209595	-1.766173331	8.389450655	11.31197531	8.634204809
Crateriforma_conspicua	-3.993300655	-2.46807282	-2.201304487	-5.716462244	-3.039917435	-3.297204278	-0.340488684	-0.389615422	-1.456099631	-0.685924091	-0.290389677	0.007250361
Cellulosimicrobium_sp._BI34T	-3.98011237	-2.202504791	1.565679534	-1.230804091	-0.36679773	-1.470813742	0.234840029	0.919563403	0.203542151	0.382295084	-0.360377264	0.559977604
Rathayibacter_sp._VKM_Ac-2759	-3.978369984	-1.867974921	-0.899825207	-0.477359546	-1.130969394	0.682399272	0	8.110425888	7.745136469	1.009970377	-2.035724034	-0.554771717
Duncaniella_sp._B8	-3.978236026	-0.231551691	0.7500824	-0.962989609	1.338828584	1.786674016	0.145008767	0.560105624	0.40592963	-0.153360854	-0.607490047	0.296653212
Sphingobacterium_mizutaii	-3.97626179	-1.418266337	-1.658829076	-2.977980222	NA	-0.184267908	-10.17311494	-3.490443329	-0.594657329	-0.036689617	0.615275828	0.664182257
Kangiella_sediminilitoris	-3.962697855	NA	-1.327828431	NA	1.242671065	2.099802118	8.502740328	0	0	-0.254809721	0.510228657	0.222558577
Nibribacter_sp._BT10	-3.959306257	-2.253247845	-0.378455724	-2.113149485	-0.724814186	0.432651225	0.787770974	-1.356621795	1.361234246	0.433290664	0.861399822	1.085481562
Muriicola_sp._MMS17-SY002	-3.956376157	-0.329389543	0.042214272	-0.112474729	-1.017392917	-0.349387335	-0.018235583	1.816991513	1.537467482	0.525307575	0.837446387	1.634407925
Porphyromonas_cangingivalis	-3.947764485	-1.424390306	-0.349654405	-0.018319842	-0.148652366	0.453549732	-1.396054757	-0.436515166	-0.150060693	-0.406474436	-0.176247198	-0.172305132
Candidatus_Phytoplasma_australiense	-3.942522292	-2.675277264	-1.219833776	-7.44065292	-0.618015705	-0.212711134	0.57429095	2.28659879	1.054427658	-0.035224886	0.545054823	0.734489218
Vibrio_campbellii	-3.940396769	-0.976922645	-0.003816482	-2.792267778	-2.818774731	0.10823005	-0.235126055	0.458912645	0.202115118	-0.063664549	0.045660108	0.712765039
Rodentibacter_heylii	-3.939830269	-3.445388052	-5.86516347	11.7478038	1.17508936	1.543212609	0	8.607330314	8.451211112	0.907837004	1.300884424	1.623294887
Tannerella_forsythia	-3.935397448	-1.434724078	-0.258364619	-0.069273999	0.513282401	1.146250275	0.869373645	1.142713162	0.925006832	-0.28735717	-0.334335614	-0.081206618
Rhodobacter_sp._CZR27	-3.931476234	NA	-0.289424541	NA	NA	NA	-0.724091909	-0.719198785	-0.822611464	-8.888743249	-0.172208411	1.569324953
Spiroplasma_poulsonii	-3.915111102	-3.137503524	-3.915111102	NA	NA	-1.2574774	-0.115158125	1.412318881	0.259090215	1.267318118	1.612182236	0.867895299
Exiguobacterium_sp._S3-2	-3.912267307	NA	-4.976208184	NA	NA	NA	0.844914063	1.74153867	1.552858428	0	0	0
Microbulbifer_sp._ALW1	-3.905083273	-2.434243982	-0.471434166	2.074787032	-1.116113046	-1.952171475	-0.875817148	-0.620127107	1.043936672	0.171161862	-3.303439692	0.564137015
Paraburkholderia_caledonica	-3.902036175	-1.954710715	-1.066989519	-2.598316128	-1.822257336	-2.136072785	-0.879822556	-0.743999722	-0.338015749	-0.4668422	1.06297381	0.998621285
Thermanaerovibrio_velox	-3.900258991	-4.157109856	-0.969362932	2.82434612	2.167435181	0.505378413	0.208859093	0.3764971	1.127392716	-0.413265606	-0.188770704	-0.470147945
Duncaniella_dubosii	-3.900116482	-1.623410461	-0.134248115	2.505872832	1.770455407	1.87086065	10.0949222	9.627367901	8.30544163	-0.573465527	0.032013238	0.099713763
Brachyspira_pilosicoli	-3.887610745	-4.674813409	-2.273944738	-4.443085221	0.382337619	0.87649225	0.772485893	0.095792242	0.705008627	0.212029684	0.810202879	0.602593726
Streptococcus_troglodytae	-3.886537656	-3.087122208	-6.566962787	-0.518742472	1.18571292	1.67830882	0	9.3781973	0	-2.586481784	-1.486512519	0.217974675
Frondihabitans_sp._PAMC_28766	-3.885671815	1.004002366	1.342558216	-3.799821249	3.454321597	3.420481495	-0.082092385	0.352804195	0.156423296	0.050393545	0.731656973	0.263153582
Sporosarcina_ureae	-3.885287191	-0.553781885	-1.16739839	1.250798259	1.033726726	2.169102191	0.6759447	2.179068655	0.760094752	0.349743487	0.558352295	-0.07590238
Leptospirillum_ferrooxidans	-3.884737453	-3.987220411	-0.713164599	-2.900586611	-2.26772942	-0.055678943	-11.54197746	-11.54197746	-11.54197746	0.281793076	1.748166056	0.576515784
Rhodovulum_sp._P5	-3.884170519	-5.379550127	-0.390615246	-1.568209266	-0.83021903	0.413416311	0.98530122	1.388561367	1.107572258	0.433027851	0.643682944	0.705778604
Colwellia_sp._PAMC_21821	-3.884130693	-1.904980163	-1.152191916	-5.703228352	-3.116800442	-2.995364222	2.35040154	0.334761816	2.052333511	0.249696322	0.493525562	0.350637762
Cupriavidus_campinensis	-3.880936054	-2.372527057	-2.266454467	NA	NA	1.371828047	9.129283017	0	9.673068754	-2.194459409	-0.119317347	-0.353837343
Chryseobacterium_balustinum	-3.879052945	-1.699089006	0.225709052	NA	0.221771881	2.683165752	8.129283017	0	9.695386649	-0.380628214	0.054312835	0.59234624
Flavobacterium_anhuiense	-3.874239809	-4.683613899	-1.299401526	-1.6687151	0.232787991	1.687205882	0.788524909	0.371800255	0.578055146	0.115274878	0.459881174	0.288751151
Chryseobacterium_sp._6424	-3.87291419	-0.803600969	-0.092365051	3.31934087	1.547590932	1.913618658	0	0	0	0.446909914	0.439851205	0.746622483
Neisseria_subflava	-3.871843649	-0.86929996	-0.16177465	NA	0.259762285	-0.619288463	-1.775498301	-9.932617823	1.174834668	1.230933625	2.101885631	0.679070656
Janthinobacterium_sp._17J80-10	-3.86663586	-2.415148131	-1.861802706	NA	-0.269471703	0.181952085	0.363556628	-0.305579597	1.183216332	-9.723375961	-4.250888189	-0.822509153
Lactobacillus_sanfranciscensis	-3.862637358	-2.190212016	0.886813108	-1.201055627	-1.672588012	-0.506868673	-0.842570392	0.2122653	0.131077311	-0.603275581	-1.179004148	-1.401013389
Burkholderia_cenocepacia	-3.861108602	NA	NA	NA	NA	NA	0.430457909	0.838810241	-2.182844483	-8.087462841	-8.087462841	-8.087462841
Leptotrichia_sp._oral_taxon_847	-3.861005967	-1.038674837	-1.851627635	NA	-3.201967471	-1.054961062	0.6814941	-11.19397498	0.336075275	2.026842007	0.770587088	0.993759661
Draconibacterium_sp._M1	-3.858759316	-3.854363429	-0.331374091	-2.622108437	-1.061843358	-0.131272795	-0.312530792	0.565349668	0.456648197	-0.086709118	0.053627414	-0.219005385
Candidatus_Cloacimonas_acidaminovorans	-3.857035974	-4.259884064	-3.711658237	-2.151218807	1.536864224	0.9856906	-9.177646265	-9.177646265	-3.020529775	0.929219987	0.100012289	1.424139764

Mycolicibacterium_confluentis	-3.852101305	-1.371968777	-2.724721998	NA	NA	NA	-0.539727163	-0.765915293	0.468926825	0	0	7.380824669
Stackebrandtia_nassauensis	-3.836185515	-5.049091888	0.224957628	-2.99829894	-1.890730016	-0.413510815	-10.27612441	0.982977387	0.836966578	-0.599472473	0.150132859	-0.464894856
Photobacterium_gaetbulicola	-3.832890014	-2.170750578	-2.466694524	3.386236642	-0.613763358	0.332051066	-1.274494497	0.476205215	1.962697241	0.466829111	-0.757003845	1.321529558
Algibacter_sp._L3A6	-3.831367044	-0.579091985	-0.77352297	1.119169086	1.956009218	1.570576534	-0.67888636	-0.338365877	-0.632840429	-0.771704305	0.035162752	0.166388342
Alistipes_sp._dk3624	-3.829385267	-0.677949555	0.20302269	-0.948825205	0.958982702	0.878360154	-1.484936938	-2.034704339	-2.278745863	0.01614298	0.159256354	0.04259162
Sphingomonas_alpina	-3.82833058	0.965991778	-0.361839601	-1.608412173	2.479762783	1.797514908	-0.736963684	1.094248333	-0.680780882	1.200716908	0.443739523	1.52465368
Neisseria_bacilliformis	-3.825543704	-5.81549004	-0.718394316	NA	-0.991613117	0.892257807	0.354693976	-0.235478344	-1.014979606	0.42246367	0.336506571	-0.147805899
Haliscomenobacter_hydrossis	-3.821654688	-1.072463412	0.012095969	-1.074879165	-0.252146582	0.397217331	0	0	0	-0.735542133	-0.380426535	0.555377202
Barnesiella_viscericola	-3.819698857	-1.100321676	-0.233879398	1.025174441	1.067465345	1.31488711	-1.186936839	0.153734883	-1.519116294	-0.037490377	-0.026521383	0.210351271
Bifidobacterium_animalis	-3.817578742	-1.559319713	-3.059721006	-3.641003221	0.380644552	-0.04509523	0	0	8.624046047	0.464340294	0.744369535	0.504169185
Neisseria_musculi	-3.813332359	-2.010728224	0.327298823	1.479827266	-0.051643828	0.710341617	-1.372676445	-0.666129382	0.285971496	-0.343706972	0.733289519	1.013958808
Geobacter_sp._M18	-3.810090709	-1.342792985	-0.539509494	1.576505646	-0.169824623	0.238875329	1.008505204	1.576472989	0.975422491	-0.535274062	0.531297083	-1.162616375
Sphingopyxis_sp._113P3	-3.80512045	-0.056480323	0.764431401	-3.046301361	-0.060098358	0.643508639	0.978278095	-10.01478766	-0.576752691	-1.04550999	-0.505255752	0.211895346
Arenibacter_algicola	-3.798391361	-1.00046415	-0.021919216	-1.300278072	-0.08126829	-1.923586884	-0.419679968	-0.191233126	1.916908677	-0.146041111	-0.299168997	-0.47754112
Spiroplasma_cantharicola	-3.796561642	NA	NA	0.172781259	NA	NA	10.28455809	0	8.624046047	-0.35633968	-0.231326972	-0.99514818
Thermomonas_sp._HDW16	-3.796445259	NA	0.17581659	-0.585579169	-2.205502672	-2.916168409	-0.316453781	-1.026704679	0.312842615	-3.479276763	-0.592127533	-0.10341317
Massilia_oculi	-3.794909348	-5.582990258	-1.439586133	0.897892218	0.011467085	0.578471985	0	0	12.39387842	1.456685542	1.354543468	2.266996517
Lactobacillus_zeae	-3.791162889	0.60244208	-0.00571042	NA	2.818049523	2.163649676	0.391585611	1.147748595	0.760486156	0.134036516	0.426409572	-2.690443547
Suicoccus_acidiformans	-3.790247093	-0.97627759	0.852559483	0.968975873	-2.011251135	0.039197888	0.524048121	0.023197576	0.969692024	0.375419585	0.623775456	0.284850177
Pelodictyon_luteolum	-3.789564532	0.362106324	0.845364947	NA	2.362782363	2.067964802	-1.225296736	-1.224328488	0.214923451	-0.07814786	0.049962557	0.378921996
Polaribacter_haliotis	-3.788854985	NA	-0.625008154	-1.473180148	-1.744821261	-2.18123778	2.455050236	2.859307782	2.72173792	0.149912862	-0.18135991	-1.005118378
Pandoraea_apista	-3.785926535	-3.834659107	-1.691534165	2.528650128	0.772594184	0.688300777	0.211302657	0.043238525	0.702648714	-0.662916109	0.429752119	0.320177728
Modestobacter_marinus	-3.776288724	-0.449307401	-0.11082813	1.371903848	0.940213278	2.498124454	-0.111373713	1.402205336	1.184030836	1.57049849	1.137504143	1.238210744
Bacillus_sp._FJAT-45348	-3.770281858	-1.649376039	-2.213936177	-2.536787907	1.064760298	0.146051044	-0.934218079	0.224637303	0.999506777	3.308417457	2.870828724	3.288845782
Croceicoccus_marinus	-3.76954303	NA	-0.320784913	0.749593202	1.320219752	-1.535809016	-1.79054177	-0.64528969	1.116261196	-1.824612305	-0.656202141	-0.192419233
Microvirgula_aerodenitrificans	-3.766681107	-2.625637226	2.352091646	NA	-0.214228228	-1.155774513	8.6039638	0	0	-1.114958542	1.382298646	-1.434360762
Arthrobacter_citreus	-3.759691988	-2.984968591	-3.608916293	-2.215566586	0.996241085	0.580292697	0	0	8.84263634	-0.792585372	-0.1332573	0.400039533
Planococcus_antarcticus	-3.753386947	-3.379831047	-5.902305569	0.084331527	2.04706065	1.823947694	-2.549286971	-1.954681444	-2.758208049	-0.40185138	-0.144735093	1.390988237
Bacteroides_sp._CACC_737	-3.752821296	-0.983242586	-1.469391875	-5.046119369	-5.279151268	-5.031624447	0	0	8.904155902	-0.211167227	-0.26023853	-0.364763012
Rhodobaca_barguzinensis	-3.749492692	-2.241083695	-1.223792834	-0.143764984	NA	-0.30091138	-0.306966897	-0.121792573	1.518069487	2.028749278	2.364133664	2.05954466
Blastococcus_saxobsidens	-3.745153619	-4.286705046	1.877425721	4.855292862	5.52927497	4.952640199	-10.99242148	-2.385091165	-0.973845719	0.706170184	0.888502663	0.297098575
Capnocytophaga_stomatis	-3.744833837	NA	-1.174978229	-0.446207026	NA	NA	0	8.382131157	0	-1.303151812	0.187596348	0.656647872
Candidatus_Symbiobacter_mobilis	-3.743558655	1.477031074	1.200251225	-1.818819655	2.968177511	2.778185056	0.187228654	-0.533375657	-0.826668397	-0.60289283	0.493538802	0.419837673
Nitrosomonas_europaea	-3.735706881	1.17025144	1.008788017	3.082453349	3.717227279	3.276034354	1.612184224	0.941962262	0.25890542	0.058500673	0.423536514	-0.157571154
Nautilia_sp._PV-1	-3.729503857	NA	NA	-5.450235227	-6.278851801	-0.323903331	8.502740328	0	0	-0.16960948	1.026153125	0.627646143
Burkholderia_gladioli	-3.72431609	-3.892597262	-2.811984542	-2.454499095	-4.356867776	-4.457429914	-0.461138641	1.529322061	0.972097976	-0.059244152	-0.444503587	0.012350776
Thermus_oshimai	-3.723554675	-4.102203215	-1.771402429	1.947325891	-0.979118803	0.94966134	1.825969533	-10.02236781	0.008795768	0.731032695	0.018303974	-0.48023792
Campylobacter_coli	-3.72250461	-3.20043822	-3.779648517	-1.161327431	-1.620675049	-1.380207221	9.029913869	0	0	0.214238827	0.240963713	0.293878079
Burkholderia_pseudomallei	-3.712228916	-2.637266858	1.3449325	2.239841697	1.1236282	1.800820966	1.887525414	1.968395759	3.312278883	-0.430768621	-0.432722934	0.677212324
Dysgonomonas_sp._HDW5B	-3.712226131	-0.167045495	0.560131421	-4.557881268	-0.190679093	-0.227686249	-0.571198768	-1.725975036	0.335204731	0.622080373	0.354630905	0.378378863
Paracoccus_sp._Arc7-R13	-3.70633963	-1.849949634	-0.412572311	3.95482859	1.527317607	1.496586394	0.181661235	0.537624517	1.975701953	-1.995848357	0.972364244	-0.76048574
Desulfoglaeba_alkanexedens	-3.706222464	-3.086778288	-2.013068602	-1.307756874	-0.791636424	0.660080709	-0.514503442	-0.054769694	-0.220956768	0.363189889	0.228355849	0.729546215
Haematospirillum_jordaniae	-3.698760410	-3.128691819	-1.121157791	-3.813664774	-4.406506569	-0.686328816	0	0	9.561202011	0.315795482	1.084972357	0.256141386
Agrococcus_jejuensis	-3.697971463	-2.748724763	NA	4.147089573	1.586244327	-0.973317957	0.521485515	0.87725629	1.711374954	-0.984652036	-1.644018936	-1.031194622
Dickeya_sp._NCPPB_3274	-3.697836358	-2.349108204	0.433184575	3.618750407	1.921162353	-1.840423687	-0.297656612	-0.565636624	-0.064013936	0.603187512	1.254885961	1.157369455
Marinilactibacillus_sp._15R	-3.683960156	-1.249387898	-0.980767274	0.709336119	0.383895124	1.335546906	-0.152004744	0.007812581	-0.895039043	0.809663266	0.70828774	0.368077234
Paraburkholderia_graminis	-3.682793285	-3.493936468	-1.790640061	NA	0.549120193	2.769423851	0.126419376	-0.181086455	-0.039749976	-0.56415074	-0.887092322	-0.641181528
Cedecea_sp._FDAARGOS_727	-3.681063906	-0.960433372	-1.015658413	-1.525364449	-0.497066454	-0.726330124	-1.822001698	-9.951284715	-2.794170247	-0.47775755	0.705815622	-0.3308688
Corynebacterium_sphenisci	-3.674839205	-2.254602034	-0.549354833	-0.770309667	-1.929142278	0.914394127	-2.320627948	-0.56256681	-0.744891781	0.409810892	-0.192618162	0.638336906
Mucilaginibacter_gotjawali	-3.674404945	-1.946764095	-1.744263968	NA	-1.227519639	0.035703087	9.832602079	8.627369725	6.15711649	-0.15912587	-0.18549965	0.069391582
Fimbriimonas_ginsengisoli	-3.673298939	-2.518335185	-1.243573666	-3.880163918	-0.590827641	1.017076507	-0.060586502	0.374157525	0.147179113	-0.35180696	0.025117158	-0.142962441
Runella_sp._SP2	-3.664689663	-3.624152804	-1.154347393	-2.641594638	-2.274943858	0.242493627	0.712493068	1.863997901	2.068306795	-0.377898234	-0.066182017	0.552647088
Planococcus_versutus	-3.661778098	-2.332205972	1.096028574	NA	NA	1.545204791	-0.669372483	0.319985741	-0.409507073	0.463326088	-2.285148246	0.243843549
Thermoanaerobacter_kivui	-3.659924558	-3.073010192	-2.893606661	-2.129540296	-0.287107551	-0.449050168	8.6039638	9.566400396	6.15711649	2.276317498	2.274874081	1.952329699
Defluviimonas_alba	-3.658543631	-1.47378359	-0.890051533	NA	0.654758121	0.021489423	0	0	0	-0.326183458	-10.4101015	0.087081713
Jeotgalibaca_ciconiae	-3.651012893	-1.078057694	1.96719401	-1.544537218	-1.265411241	-0.213937601	0.862911253	0.533361861	0.129489812	-0.237798846	1.170470655	1.372989101
Microbulbifer_sp._SH-1	-3.650964309	-0.796226738	0.658237191	-3.254773187	0.922022367	1.366421277	-0.760552194	0.339607988	0.220007028	-0.175510576	-0.200761547	0.825662252
Granulosicoccus_antarcticus	-3.645066335	-0.579730235	-0.278242827	-3.831941185	-0.050633209	1.617691815	-1.110626913	0.695466833	1.101474761	-0.480144973	-0.607802511	0.266758596
Rhizobium_tropici	-3.643306487	-1.865698908	1.418469711	0.067263227	-0.055141554	1.62058641	-1.515413988	0.030901242	0.183088243	-0.571156701	1.77069286	1.257628634
Pseudomonas_sp._ATCC_13867	-3.639169575	-4.665930643	NA	NA	NA	NA	0.494783439	0.175048709	0.003331347	-0.192645078	-0.86507364	2.063134913
Sutterella_faecalis	-3.638435503	-4.266615603	-1.779201468	2.337078995	0.487511799	0.960802424	0.33014713	1.363708573	2.83237315	-0.493002365	0.2658021	0.562256555
Streptomyces_sp._SS52	-3.62844554	NA	NA	NA	NA	NA	-10.20011392	-10.20011392	-10.20011392	-7.807354922	1.510961919	-7.807354922
Nitrosomonas_ureae	-3.627141713	-3.114134444	0.041540502	2.276645499	0.18502504	0.797229003	0.602823367	0.173244354	-0.172720088	-0.090366352	0.53517346	0.555879791
Cronobacter_universalis	-3.626596989	-3.666125354	-5.438247393	NA	NA	-2.765192511	-0.794037745	0.335846588	0.101135544	-1.139643505	-0.181997283	1.273121002
Candidatus_Phycorickettsia_trachydisci	-3.62443633	NA	-0.661738475	NA	-3.126173488	0.092541402	-0.119846759	-0.600028746	-0.706294278	0.811919133	1.038466309	0.309592913
Candidatus_Desulfovibrio_trichonymphae	-3.623829836	-4.298753529	-0.886402436	-3.251387063	-2.716177598	-0.248145474	-0.611071367	0.232044396	-0.035831247	0.632837185	0.951703929	0.552102954
Halobacillus_halophilus	-3.622817171	-3.25275747	-3.253858764	-0.017143206	0.981342084	0.545398	-0.907545728	0.571962166	0.195922042	0.191801765	-1.033750244	-0.342416827
Staphylococcus_pseudintermedius	-3.622216379	-3.087421704	-1.645966033	-0.6379733	-2.593397044	-1.917429188	-1.101722103	-1.461616846	1.414081344	0.093558489	-0.169060477	-0.019584496
Sporosarcina_ureilytica	-3.621966391	-1.447458977	0.112319392	-1.796099181	0.908702319	1.154325484	0	9.384097309	6.15711649	0.05577168	0.207932869	0.499899996
Maribacter_sp._1_2014MBL_MicDiv	-3.618780784	-2.842014182	-5.0977652	NA	-4.596952574	-1.009355975	-1.813588499	-1.203472317	-2.459501391	-0.300076794	-0.898631178	-0.280694967
Paraflavitalea_soli	-3.616393597	-0.041952164	0.717084734	-3.630407449	1.747750953	2.330864169	0.291753212	-0.240995128	-0.173037803	0.508442417	0.637428242	0.981079138
Bradyrhizobium_sp._6(2017)	-3.615698538	-0.745301957	-0.009560369	1.808830305	-0.043770713	1.275216106	0.438603188	1.166263827	0.880455957	-0.767762693	-0.994749613	-0.961685515
Echinicola_strongylocentroti	-3.61474477	-0.797789227	0.406279641	-0.627197721	-0.532573819	-0.257893613	-2.238176948	-1.256265897	-0.821095635	0.326235172	0.012189409	-0.334368352
Gloeomargarita_lithophora	-3.610967896	-1.352003759	-0.039293965	-2.44888639	-1.318547918	-0.163608755	0	9.3781973	10.82104847	-0.077599092	0.610998746	-0.57789002
Sideroxydans_lithotrophicus	-3.610709972	0.939364071	0.814381191	-0.203101365	2.606767304	2.506248095	0.209194588	-0.290800596	-0.430723578	0.388204615	1.278078968	0.534829929
Helicobacter_canadensis	-3.607924832	-2.542036896	-2.221139666	NA	-0.105540928	-0.693607225	2.628102597	1.917102938	-8.04736934	-0.310745109	-0.492119833	-0.160386303
Xanthomonas_hortorum	-3.60698658	-1.066379619	0.719783516	-2.632084014	-1.814973792	-1.502474756	-8.10567284	-8.10567284	0.493224552	0.850466122	-0.107357778	1.34021192
Prosthecochloris_aestuarii	-3.597232364	-0.632273169	1.526903272	3.322719325	3.979646938	4.003035396	-8.624046047	1.255258439	1.775669801	0.518528924	-0.892743339	0.257921758
Anoxybacillus_kamchatkensis	-3.594485275	-5.328269622	-5	NA	NA	NA	0.076361821	0.349016473	0.737952767	0.175297002	-2.609392782	-0.590244039
Frondihabitans_sp._762G35	-3.59239909	-1.90877253	0.044731237	NA	6.469804836	5.402271987	-1.008776683	0.352726156	-0.407543628	-0.496482339	1.952011124	3.611720877
Staphylococcus_haemolyticus	-3.586508206	-1.81591387	-0.015829565	-0.824554529	-0.600172921	1.02750072	2.178534566	2.018003227	-0.380754681	0.106581604	-0.039150568	-0.024430593
Lactobacillus_gallinarum	-3.582008511	-5.487588162	-5.796553071	-1.003928581	-1.112168896	-0.980783967	-1.150363108	0.78920069	0.646150976	-1.325344957	-0.761038379	-0.434228133
Streptomyces_sp._Tue_6075	-3.581200582	-1.90087723	-0.455787111	2.450244927	2.047030312	-0.34126458	0	0	0	0.288431183	1.605834327	0.336049203
Pseudorhodobacter_sp._S12M18	-3.5789886	-4.005998741	-1.852193405	4.776583798	2.231072448	2.395234124	0.455370987	0.334761816	-8.04736934	0.920230276	1.04832108	0.184830224
Exiguobacterium_sp._Helios	-3.574837427	-3.908115294	-4.380005895	-2.884653559	-0.925458606	-1.402164679	0	0	0	-1.058364163	-0.506124735	0.050479451
Corynebacterium_segmentosum	-3.572465708	-3.383608891	0.106236608	-1.158011828	-3.307608424	-0.12507306	-8.799135887	-2.116464279	-1.642021419	2.117533441	2.417540006	3.522013306
Bradyrhizobium_sp._BTAi1	-3.569031312	-1.129047456	-0.590864068	-0.933556253	0.372638915	1.61976693	0.430399688	0.371035854	0.449932401	0.61094538	1.735703359	2.251808909
Pseudomonas_furukawaii	-3.567048835	-1.599859991	-0.796326064	4.846521957	2.716188817	3.390920139	-0.654106513	0.574650922	0.479788841	-0.67846698	-0.285784622	-0.038814344
Arthrobacter_sp._YN	-3.562242424	NA	-4.842511367	NA	NA	2.773892163	0.578049073	0.770274607	1.759472012	9.871392428	7.222389201	8.398029648
Tatumella_citrea	-3.560500443	-1.132941317	-3.67914494	0.306590772	NA	-0.381167288	-3.495786639	0.381639144	0.63793226	0	0	0
Methylocella_silvestris	-3.558477711	-1.301724445	-0.963178559	4.168052624	1.927726526	2.753934367	-1.320095624	-10.48179943	0.67244275	0.268707614	-0.710493383	0.958831709
Mesobacillus_jeotgali	-3.551709606	-1.433270882	-1.342638028	-1.133336166	1.307028721	0.854776688	-1.850746984	-1.21773897	-0.584850763	-0.029054959	0.609746812	0.818566548
Streptomyces_sp._WAC_01529	-3.550746785	NA	-2.038994132	NA	1.70862248	-1.015320799	1.766394651	0.584961421	-0.347924776	1.838285866	-1.0748477	2.416056095
Halanaerobium_praevalens	-3.549379004	-2.127361099	-2.32145477	-1.695536357	1.044846684	1.144812773	-1.067051304	-0.044811971	0.557513348	-0.323686876	0.165681953	0.53787455
Photobacterium_damselae	-3.533079151	-2.172172625	0.046817448	-2.562532147	-1.273474478	0.317780941	-0.558060736	0.552696125	0.040220535	0.220791103	-0.258360944	0.141340042
Flexistipes_sinusarabici	-3.528708317	-2.368540863	-0.702520558	-2.214822306	-2.026571043	-1.072596863	-1.456485479	-1.33907266	-2.123804222	0.286368472	0.477305314	0.313428699
Turneriella_parva	-3.526499239	-1.444350198	-1.465803307	3.718366467	1.232552288	0.65678068	0.427002284	0.505615854	0.203889574	-1.859684805	0.85544929	-0.480752651
Pusillimonas_sp._DMV24BSW_D	-3.520400268	-1.744517219	-0.973085055	1.588051123	0.503772927	0.234882604	0	0	0	-0.158825939	0.018440762	0.943901374
Stenotrophomonas_sp._ZAC14D2_NAIMI4_6	-3.50751223	-2.282761821	-0.069598717	NA	-2.08081477	0.398036481	-0.716652602	0.622223019	0.063848457	0.112941176	-0.605502826	-0.561417475
Paraburkholderia_tropica	-3.499321642	-1.927724625	-1.972208173	0.724907177	2.014932656	1.370837695	0.638561852	0.418218776	-0.57888642	0	9.8008999	0
Dehalogenimonas_formicexedens	-3.497094958	1.261942925	1.151587313	0.366593308	2.382196721	2.403672651	-0.393329638	-0.316483135	-0.111538701	0.070243264	0.023698525	1.169870115
Desulfovibrio_alaskensis	-3.493558357	-2.834606051	-0.393174289	-4.207307894	-1.086596447	0.09508303	10.65610304	9.384097309	0	0.314458608	0.706870181	0.553488426
Sulfurospirillum_cavolei	-3.49292324	-2.865488728	-0.78450905	-1.558774689	1.249487874	-1.184424571	2.470096009	1.699075082	2.993727597	-0.063949443	0.732212923	0.29193665
Bradyrhizobium_sp._CCBAU_53421	-3.489192019	-2.916006686	-1.115424992	-3.558327374	-0.69043091	2.23830583	0.421165017	-0.104766478	0.228701649	-0.599295448	-1.121080582	-0.951153876
Hymenobacter_sp._DG01	-3.486406752	-0.966963167	-0.222510728	2.872114599	1.964989462	2.555385322	-1.03767594	0.791052633	-0.703662739	0.070748387	0.772672134	1.096599764
Athalassotoga_saccharophila	-3.485009899	-3.486598205	-0.239394122	2.081980776	2.125264874	2.783712267	8.502740328	8.607330314	10.75883115	0.063349495	-0.73750436	-0.197524223
Leuconostoc_citreum	-3.484435625	-0.906777925	0.215460749	NA	0.320947289	0.29820274	-0.148435967	-0.117888811	0.105664395	1.133545429	1.04870064	0.335455643
Anseongella_ginsenosidimutans	-3.482919229	-0.516612827	0.554428133	-0.78518466	0.514564613	1.29420305	0.151902742	-0.08045498	0.08625524	0.457036743	0.85471029	1.07990511
Chitinophaga_pinensis	-3.482703861	-0.478185012	0.101807002	-0.940417297	1.356017435	1.510273035	-0.844998939	-1.856731726	-3.198732576	0.101555671	0.167198161	-0.034786415
Hyphobacterium_sp._CCMP332	-3.479703014	-1.772816716	-0.761852546	NA	1.728157137	1.020608662	-0.863695133	-1.045583938	-0.393240419	0.105535968	-0.111777811	0.556452665
Agrobacterium_vitis	-3.471297761	-2.442462123	-0.372118113	-0.094909351	0.793814064	1.428750347	0.087823584	-0.36729403	-2.908724602	0.523282399	-0.064688599	0.348559443

Lutibacter_profundi	-3.469691261	-2.869973034	-0.207359272	NA	-0.561131837	1.345855225	0.570789239	1.374549865	0.787059168	-0.353004394	-0.163865046	1.072672377
Escherichia_marmotae	-3.462478494	0.765982274	0.378043292	-2.20701432	2.500057165	3.11978965	0.070707958	0.172921829	0.386063642	1.655595539	2.553464386	1.676540505
Corynebacterium_camporealensis	-3.461710758	0.567780539	-1.681491966	-1.051365632	NA	-1.030070854	0.44881047	-0.226376453	0.992821023	9.990572019	10.34132979	11.15987134
Sulfurimonas_sp._S2-6	-3.460403832	-2.175001613	-2.86124745	-1.760534065	0.031463816	1.450203163	-0.151280045	1.119180707	1.834593499	-2.09314436	-2.497790932	-0.031545917
Phytohabitans_flavus	-3.458722503	-2.317678622	1.462394694	2.868224129	0.535672125	-0.177998302	-1.557125995	0.420886575	2.010114849	-0.811771276	-0.071788447	-1.16842423
[Enterobacter]_lignolyticus	-3.457736751	0.841792337	0.807666374	NA	2.308462843	3.120292638	10.72668649	12.38532318	12.47086728	-0.223075991	0.46860365	0.694028908
Bacillus_sp._KH172YL63	-3.455348517	-1.537535883	-0.351707383	1.295072018	-0.716387097	-0.253821228	0.380314782	0.073592531	-0.241736731	-0.227316828	0.489460338	0.989989934
Porphyromonas_crevioricanis	-3.449341563	0.872489508	1.151501176	0.672053485	2.89375251	2.822469277	-8.04736934	0.559960973	2.347479165	0.001826477	0.573345189	0.318914576
Calothrix_sp._PCC_7507	-3.443054785	-3.07350867	1.142512746	-1.656382988	-1.423261409	0.283233285	-3.121103189	-0.896509005	0.042807494	-0.095209973	-0.295310008	-1.170851924
Acholeplasma_laidlawii	-3.440162093	-0.536498423	2.348269669	NA	0.086624235	0.575068668	-0.204125873	-0.797600866	0.511059575	-0.133861763	0.61097443	1.287437286
Pseudoleptotrichia_goodfellowii	-3.439547127	-3.453724155	-2.733584015	-1.44325055	-2.391185724	-3.144212171	-1.079659922	1.384630713	0.395717175	-0.3835428	-0.32634044	-0.839285752
Desulfovibrio_sp._86	-3.427450163	-2.245773853	-0.760683068	-3.217654021	-0.199897898	0.041834156	-0.898000813	-1.554538152	-1.613614748	0.846094713	0.776307214	0.572323427
Hydrocarboniclastica_marina	-3.426264755	-3.440466953	-1.869047244	0.41053008	0.451069052	1.65638556	0.086443161	-2.283741609	-1.148920259	-0.549763699	-0.329700342	-0.390317282
Pseudoalteromonas_ulvae	-3.42037306	-1.727204584	-0.541170749	NA	3.30323721	3.52438649	0	0	0	1.820556841	2.697496681	2.711235062
Spirosoma_sp._CJU-R4	-3.420161991	0.934332151	1.148217038	-4.801650677	2.986883218	3.084834886	-0.938813172	1.516343945	-0.081672612	-0.314972674	0.491201	0.177969051
Xenorhabdus_nematophila	-3.415037499	-3.506276297	-0.090125546	1.133867714	-0.19291522	0.133760006	0.143830172	-0.999308859	0.143207766	1.326026243	1.150144502	2.120554323
Hymenobacter_sp._S2-20-2	-3.410650182	-0.998645821	-0.247012388	NA	0.395682197	1.972643234	11.05971624	12.81578354	11.44346161	-0.230822189	0.46751262	0.517529723
Novosphingobium_sp._Gsoil_351	-3.408464845	-0.797837461	-6.176018759	NA	NA	NA	-1.151553538	-0.172305319	-0.194948309	-1.968793564	0.89054074	-9.389452806
Mycoplasma_gallisepticum	-3.406097862	-1.217162144	-3.611903672	2.278535499	1.014766711	2.317170646	0.44536423	0.994222029	1.028379676	8.990577692	7.222389201	0
Sulfuriflexus_mobilis	-3.40599236	NA	0.070821337	NA	1.056166711	0.290631964	0	9.426646347	7.157114468	-1.123847008	-0.646526682	-0.431198513
Desulfurobacterium_thermolithotrophum	-3.404520222	NA	-1.197809423	-0.253870653	1.01328322	0.43783866	0.15743454	1.259662728	1.241370725	-1.960350923	0.151109507	-0.804008894
Agarilytica_rhodophyticola	-3.404514513	-3.808411455	0.322772764	NA	-1.096974113	-0.394725612	-1.502744941	-1.328369802	-1.959695898	0.30467806	0.972960183	1.272228098
Hyphomonas_sp._CACIAM_19H1	-3.401946124	0.08589391	-0.171925688	1.931689327	-1.873785862	-1.686158859	-0.09596483	1.628122364	-0.069653431	-0.425204585	0.899116643	1.587404214
Gemmobacter_sp._HYN0069	-3.401405296	-2.240243555	-1.177243557	-3.993881891	-1.481470623	0.155441841	-1.600148615	-0.160846202	-0.055494755	1.087599404	0.896667642	0.871013378
Citrobacter_portucalensis	-3.395337091	-3.640072501	-3.743555861	NA	NA	NA	-0.477431773	0.32318034	0.616878971	0	0	0
Caminibacter_mediatlanticus	-3.39386621	-2.776723303	-2.755661688	-1.791691263	-3.070013713	-2.381664867	0	8.382131157	0	0.463854571	0.695097902	0.325235224
Campylobacter_concisus	-3.391784768	-2.195997703	-1.821811442	1.174063307	0.026607835	NA	-1.144238623	-0.749693826	-0.163387275	1.266812172	1.654527697	1.165457777
Laribacter_hongkongensis	-3.387794412	-2.676807884	-0.613975093	-5.685208547	-1.640814427	-0.922950215	-0.189619644	-0.452884577	0.379716059	0.101432334	-0.191218203	-0.041148279
Methylocystis_parvus	-3.385369949	NA	2.378192042	4.702593592	5.027212583	4.862446437	-8.799135887	1.393156927	-0.642016365	0.056408827	0.143524264	-1.302815476
Pontibacter_russatus	-3.380969032	-0.899775187	-0.820783389	3.451416012	1.106011415	1.270249049	-0.516976854	-0.3761963	-0.019327795	0.050860496	0.044537974	0.101369014
Roseicitreum_antarcticum	-3.377328249	NA	-2.18398436	3.4034277	0.789531421	1.25629398	-10.48088106	0.28323582	0.387941494	2.745699767	2.28643901	2.580461108
Chryseolinea_soli	-3.377179161	-0.16139144	0.502618433	0.496448578	2.54067576	2.523768806	-0.585244641	0.986976596	0.599263648	0.28728095	0.533824024	0.668418673
Gemmatimonas_sp._TET16	-3.371029114	-1.394981727	0.395349935	-0.948248025	1.440120773	0.447395396	1.171300501	0.513953089	0.908374266	0.150051765	-0.643407544	-0.016086599
Microbulbifer_aggregans	-3.366249608	-1.316420433	-0.373218904	-3.648472271	-0.190500821	0.039980398	0.449526643	2.064465328	-2.051895298	0.117513361	-0.333466044	0.34420806
Pelosinus_sp._UFO1	-3.364333715	-1.916769843	-1.462500258	-0.860138503	0.81415109	0.980719989	-0.720674621	-0.453536966	-0.424250136	0.414885372	-0.339692307	0.360354204
Wenzhouxiangella_sp._AB-CW3	-3.357946579	-8.916721268	-1.206415472	-3.45774195	-0.729471058	1.375039431	0.214123555	3.039036873	3.209496746	0.503508203	0.995705949	1.194907234
endosymbiont_'TC1'_of_Trimyema_compressum	-3.347900136	-1.254344823	-0.257083252	NA	4.380109166	5.750041804	-0.590869364	0.556687045	-1.248666273	-0.987556002	0.287999068	-0.224450362
Porphyromonas_asaccharolytica	-3.335326744	-0.566429958	0.078854425	-0.334665191	1.457297375	1.623265307	-1.856455128	-0.326414559	0.825506053	-0.269064604	-0.138451868	0.373207043
Lactobacillus_gasseri	-3.334052321	-7.069335266	-7.685522858	-0.761538728	-0.490686447	-1.641490542	-1.213515337	0.509986715	0.668983197	-1.186343251	-0.756491646	-0.163155734
Rhodococcus_sp._P1Y	-3.332030498	-1.880418384	0.588314309	NA	0.326432834	1.777741852	-0.208964583	0.889548597	1.631526078	0.108764914	-0.177214012	0.331281941
Thiomonas_intermedia	-3.324330585	-1.866065252	-0.589049118	4.203396094	2.487787387	2.753194849	-0.736461462	-1.423257825	-0.409633767	0.167619061	0.690404092	0.065318463
Kushneria_marisflavi	-3.320502929	-1.9130707	-0.147479097	-0.647698256	2.306283009	2.140966783	1.946000582	3.570660456	-0.175089841	0.498464278	0.489223448	0.032310816
Spirosoma_montaniterrae	-3.314539632	-1.952885457	-0.728282453	-0.775376789	0.799109212	1.654454106	0.171962811	0.675610668	0.839831649	-0.535746132	-0.451138077	-0.405924987
Streptomyces_sp._CCM_MD2014	-3.309363457	-4.118737547	-1.489666694	-0.916476644	0.219535722	NA	-8.799135887	-8.799135887	-1.642021419	0.399368043	-2.064759124	-0.890467463
Mycoplasma_bovigenitalium	-3.306816955	-1.117960137	-3.326114114	NA	0.422731171	-0.270089163	2.890609971	1.949121383	0.758231549	2.377624172	-8.186525318	-0.060683107
Mucilaginibacter_ginsenosidivorax	-3.300686947	-1.669335396	0.577057303	-2.358965474	1.610443161	1.126177106	0	9.695386649	11.7028826	-0.393333524	-0.342342596	-0.213087375
Marinifilexile_sp._TRM1-10	-3.290608734	-3.520284543	-2.138816576	-2.897457097	0.73801428	1.560583525	-0.656216818	1.801584701	1.359899431	-1.063613753	0.822306916	1.292608966
Vibrio_vulnificus	-3.290068196	-2.090383242	0.313311939	-0.004048963	0.94580004	1.483964005	0	0	0	0.067701929	-0.662882459	0.458198986
Spirochaeta_thermophila	-3.289489663	-0.035919574	0.599385776	-0.964692478	2.377536412	2.641370844	-0.170853763	1.653771266	-0.499086957	-0.544673569	0.3830061	-0.096624773
Pediococcus_pentosaceus	-3.287198674	-2.816873191	-2.562833117	-0.418089372	-0.928920854	-1.315417334	0.684365017	0.939220469	1.021046174	-1.16779506	-0.423876255	-0.098201351
Hymenobacter_sp._PAMC_26554	-3.280544174	-0.855803965	0.133101882	-0.646363045	1.448735798	1.075390009	-9.334172707	2.750818331	2.379682028	-0.669365671	-0.907776109	-0.365474182
Hyphomonas_sp._Mor2	-3.279966472	-2.897760365	-0.127422469	2.248814226	1.013470165	-0.985265533	-0.292095525	1.479928168	0.899488752	-0.330629473	0.310316499	0.645959921
Chthonomonas_calidirosea	-3.278421057	-3.205514376	-1.161181231	2.892497427	2.913385775	2.66423527	0.695233264	0.440777386	-0.79333747	0.814963711	0.384412336	-0.44321503
Aerosticca_soli	-3.27694064	-5.244760234	-3.184170255	NA	-3.28386343	-2.693124891	-0.927376042	0.537072499	0.73854552	-0.439828284	-0.567184576	-0.088964778
Riemerella_anatipestifer	-3.270673342	-2.25959903	-0.798120685	-1.373528428	0.568434237	2.181043436	0.254211426	-0.275047174	-0.753680042	0.160152449	0.083541892	0.55574666
Maribacter_sp._MJ134	-3.270601514	-2.414070221	0.346107907	0.475647521	1.256353431	1.769818306	0.213984499	1.064301033	2.664667531	-1.344585617	-0.360560754	-0.759951309
Bordetella_sp._H567	-3.267251002	-2.47049538	-4.408694524	2.767440553	1.697527283	NA	0.285359234	-0.622957116	1.051651403	8.878562894	9.607330314	10.71424552
Propionibacterium_freudenreichii	-3.265567341	-1.14389101	-1.265264667	1.510091901	1.056559544	1.917968792	-0.308223947	0.416807229	1.686077998	-0.26604071	0.505742744	0.488621077
Planctopirus_limnophila	-3.258943309	NA	NA	NA	NA	NA	0	8.559465045	9.052320788	0.196201343	0.602664502	-0.442607157
Jeotgalicoccus_saudimassiliensis	-3.255109719	3.061495538	0.831439179	-3.513062044	1.221519896	0.976387045	-0.716647731	-1.020599827	0.617357779	-0.938570706	2.252733151	2.316430733
Fibrella_aestuarina	-3.251841822	-0.748083836	0.022728599	-0.002923494	1.85472277	1.979459405	-0.205842807	0.362882658	0.068363586	-0.135788349	-0.276292249	-0.279255055
Microbacterium_sp._Se63.02b	-3.245756414	NA	0.547792708	-0.496153234	-0.951744831	NA	-2.154294187	-0.079650863	-0.739999663	0.541938072	1.219002525	2.042133363
Hymenobacter_sp._DG25B	-3.245198223	-1.395802872	-0.685093184	-1.143393176	-0.268875284	1.625238998	0.762138998	-10.99820256	-1.546991445	-0.54468391	-0.40805746	0.307362754
Chryseobacterium_manosquense	-3.241459927	-0.694241678	-0.221341649	4.950050813	4.006353679	4.76707795	-8.552496194	-8.552496194	-8.552496194	-0.572458788	0.546347944	1.33468216
Rhodopseudomonas_palustris	-3.238617694	-1.526268077	-0.252966056	3.60105537	2.929419807	2.8191533	8.6039638	0	0	-0.213743004	0.386045325	0.00328493
Roseimaritima_ulvae	-3.237909614	-0.37424971	0.115214351	-2.176348409	2.002081465	1.744189793	-0.860870398	-1.490943237	-1.600552313	-0.245049955	-0.001686513	0.669813611
Simonsiella_muelleri	-3.23723093	-4.565500552	-2.295333885	NA	NA	NA	0.58025506	0.618716825	1.483725457	0.624855228	1.479315191	-7.741466986
Bacillus_sp._HD4P25	-3.23690751	-1.718645411	0.57199853	-1.665698685	2.226094139	2.572734746	0.143609044	-0.563114876	0.27377569	-0.107194883	0.51703936	-0.68020346
Actinomyces_qiguomingii	-3.234598127	-1.622821075	-0.375953977	1.566532675	2.860446393	2.937916836	-2.610766968	-1.341240143	-1.753104411	0.54805581	1.051783789	0.257140448
Emcibacter_sp._MEBiC09520	-3.230764719	-1.587165031	1.129998387	2.149488414	0.229773155	0.595585467	1.02130409	-2.951313512	1.640550282	0.900942179	1.708184671	0.908305404
Brachyspira_murdochii	-3.230095866	-4.408344914	-6.852511023	-7.576839039	-5.418881713	-4.730556388	-0.159090086	-0.291467557	0.554148547	-0.003216986	-0.050839936	-0.790742385
Bartonella_vinsonii	-3.221835289	NA	-2.202469964	NA	NA	NA	-3.113789725	-1.552620409	0.657237019	0	0	0
Candidatus_Kinetoplastibacterium_desouzaii	-3.221671614	-2.450805614	-1.159472596	2.620961009	0.741368056	0.752087409	-0.394961497	-0.200334762	-0.162054835	-0.049230216	-0.07531867	0.453020257
Thiomicrorhabdus_aquaedulcis	-3.219459938	-5.93884122	-0.491666944	-0.705843077	0.640508492	0.498847343	-10.17311494	-3.490443329	-4.015998448	0	0	0
Runella_slithyformis	-3.219339766	-0.737246623	0.053475661	-1.230595243	-0.64442279	0.622565685	-10.00077468	-0.620608025	-1.695333047	0.294886803	0.473966609	0.525286803
Campylobacter_peloridis	-3.219273079	-1.9435709	1.244754352	0.347139996	2.167170582	3.313220115	0.266119748	0.411265429	1.357526908	0.348120556	1.831740397	0.643466422
Desulfovibrio_vulgaris	-3.218661527	-1.745052309	-1.06852697	-3.6489427	-1.326035037	-0.059926309	0.204809722	0.691928617	-0.799459768	0.900056357	1.124030783	0.718649638
Pseudonocardia_dioxanivorans	-3.213147038	NA	-2.081163243	-0.933273886	-3.384577624	-0.753093979	0	9.742080338	0	-0.702701351	-2.147489874	-1.106987532
Bythopirellula_goksoyri	-3.212340772	-2.109177703	-2.017301598	NA	NA	NA	-0.119805667	-0.680781379	-1.412033387	0.56760639	-0.747506872	0.947868332
Rhodococcus_rhodochrous	-3.19813044	-2.0364426	-4.339573962	NA	NA	NA	9.129283017	9.648765614	7.157114468	-2.033951526	-1.595913469	-1.50502306
Pedobacter_heparinus	-3.197355906	1.483200459	1.727606576	-4.419266086	2.771874451	2.627075709	-3.664799929	-2.4840697	-2.276874276	-0.027231116	0.753615281	0.018267564
Bartonella_kosoyi	-3.196140385	-1.68358638	-3.756098879	NA	-0.159782873	-1.527611818	-8.637284195	2.598077886	1.325874883	8.366322214	0	0
Mesotoga_infera	-3.191890899	-3.075462224	0.827313567	0.915412498	0.910978248	2.595403854	-8.799135887	-8.799135887	-8.799135887	-0.293365337	1.037569986	0.733724018
Thalassotalea_sp._PS06	-3.188635528	NA	-0.08641202	-4.643388086	-2.109838884	-0.516279693	2.563975486	4.388650906	3.674312182	-0.743508303	-0.260470173	-1.336543336
Acidihalobacter_aeolianus	-3.178380321	-1.890491302	-1.481078944	NA	-2.101330084	-1.133817937	-0.033107295	1.510875348	1.682003097	-0.160125781	0.15547692	0.628011225
Devosia_sp._1566	-3.173635775	-1.316643492	0.737389076	1.194087052	-1.13289427	-0.012483224	0.982035754	1.415082218	1.155991818	-1.081993372	-0.639792507	-0.149642601
Halomonas_sp._PA16-9	-3.171176798	-1.498751456	0.927513299	NA	NA	NA	0.18794247	-0.324021167	-0.239423566	-0.077102039	1.883843506	0.418077194
Rhodococcus_sp._MTM3W5.2	-3.166454897	-4.193215966	-2.372755814	NA	-1.452905685	NA	-0.242250068	-0.81826927	-0.092402036	2.284710519	1.79190823	-0.201633525
Treponema_socranskii	-3.165510469	-0.884640781	-0.038218111	-1.346446073	-0.18280246	0.721383603	0.763271533	2.442500654	2.844788035	0.377150167	0.685367196	0.262576237
Variovorax_sp._HW608	-3.164535772	-1.399285834	0.946932532	1.158515647	0.429470121	1.99972103	-2.460265291	0.473329707	-0.11013525	-0.603122363	0.196961005	0.705705904
Arabia_massiliensis	-3.163059267	-2.590756426	-1.067755597	-3.132307523	-0.086583479	1.076853369	-0.362044964	-0.3131542	0.801610278	0.066323035	0.035519159	0.483404321
Desulfovibrio_fairfieldensis	-3.159528906	-4.120359507	-0.882215024	-4.975648517	-2.985643428	-2.108593235	0	0	6.15711649	1.29174761	1.484469987	1.130649167
Bradyrhizobium_sp._CCBAU_51765	-3.156818013	NA	-1.080582427	-0.524915117	-1.226003675	0.412781525	-10.35334535	-0.768040999	-1.902134236	-9.550746785	0.850132651	0.275801702
Leptospira_tipperaryensis	-3.15573112	-2.966874302	-1.567409607	2.109776258	0.123667559	0.561436291	-3.747722044	-2.512914926	-1.010296607	-0.694053291	0.100820931	0.674404259
Wolinella_succinogenes	-3.154789723	NA	-2.782514544	-3.247927513	1.414286185	2.926100043	-1.280108504	-1.402684169	1.582227626	-0.467422619	0.660245409	0.901728622
Nocardioides_daphniae	-3.150286981	-0.324202563	1.132059502	-1.698925607	1.372244188	1.105850771	9.029913869	0	8.451211112	-0.210858752	-0.028652096	0.531326978
Azospirillum_baldaniorum	-3.150103561	-1.310568233	-3.268748057	-0.724634792	NA	NA	0.423175207	0.166404645	0.766771966	7.303780748	0	0
Aeromicrobium_sp._A1-2	-3.146841388	-2.235967279	1.762042339	NA	NA	NA	-1.086368542	1.221249015	0.85965744	10.0962803	11.02283245	10.15734694
Hymenobacter_nivis	-3.13762242	-1.099212195	-0.921471659	-2.073227502	1.989003302	2.256597492	0.296038821	0.242989911	0.754272613	0.413471905	0.494151133	0.43655486
Nocardiopsis_gilva	-3.137503524	-3.874469118	2.76545438	4.505056262	5.321797882	4.786332149	0	0	6.15711649	-0.686614272	1.247946253	-0.462922945
Frigoribacterium_sp._NBH87	-3.137088419	-0.406070205	-0.919268728	0.95951011	1.655098302	2.289899784	-0.036366615	1.420310026	1.252762202	1.781385407	2.705194294	0.393562421
Sphingopyxis_sp._QXT-31	-3.136046459	-2.81105699	-0.722286451	NA	NA	1.373689523	0	0	10.87686223	-1.136552537	0.122367742	1.350633902
Martelella_endophytica	-3.133749569	-0.876434824	-0.184703326	-1.118768007	1.708150028	1.288585297	-0.20029865	1.305322046	1.053046958	-0.653909333	0.51313316	0.523175012
Psychrobacillus_glaciei	-3.130138253	-1.697500839	-0.520068118	-2.071698489	0.137843371	0.719853135	-1.03612908	-0.44977425	-4.171962246	-0.226365023	0.339057078	1.110667187
Gemmatirosa_kalamazoonesis	-3.124611443	-3.423388281	-0.758486135	-1.153357199	-0.219859493	-0.083682637	0.487541228	0.958862834	0.599736124	0.371985291	1.481750143	0.505892209
Lactobacillus_buchneri	-3.123139823	-4.214770299	0.401386823	NA	-1.007772695	-2.379028502	-9.72047685	-0.340310198	1.911032188	-0.165174602	-0.592594869	1.108729667
Verminephrobacter_eiseniae	-3.119841378	1.085647382	0.179718903	-0.862022059	1.122878446	1.864346262	-2.259713677	-0.525092304	-0.697570707	0.49173104	0.848256837	1.109074734
Pseudomonas_corrugata	-3.111358226	-0.869923357	0.371544199	-1.684654556	0.454713776	0.974044595	-0.343256175	0.619048285	-0.308282573	0.028145208	0.281268749	0.066651669
Bartonella_henselae	-3.110730205	-1.256945241	-1.171015062	NA	-2.809728091	-4.077705078	0	9.384097309	10.49276451	-0.633385199	0.65750867	-0.013620467
Eikenella_corrodens	-3.110007868	-3.494586879	0.012792734	1.110058913	1.97713665	2.13741715	8.876516947	9.695386649	8.157119522	0.398119096	0.754830639	0.457200069
Anaerococcus_mediterraneensis	-3.107612047	-3.01134793	-2.436516291	-1.254010634	1.343866103	0.7908046	-0.228535239	0.3362875	0.318565574	0.466435343	1.23105907	1.167227347

Myxococcus_xanthus	-3.105981918	-2.008119122	-1.946582096	0.60522134	0.742699981	0.566103473	-1.208705825	-10.23861969	0.925375835	-0.879716989	-0.385990462	-0.990800889
Nesterenkonia_sp._NBAIMH1	-3.10528606	-0.693420482	0.117860554	-3.793032517	1.768505956	2.519013627	2.240397348	2.652077093	2.314379014	1.096812885	0.700875159	0.591742083
Eggerthella_sp._HF-1101	-3.104069182	-2.627193308	-0.938181814	-0.607877237	0.342112198	1.132965942	-0.712883148	0.834903652	0.233893045	-0.001532139	-0.080946279	0.490765334
Actinoplanes_teichomyceticus	-3.102693365	-2.474662143	-0.55348089	2.64077818	1.211835079	0.566231227	-0.587846451	1.143900043	-0.295738907	0.940987926	1.395162864	0.499821036
Tenuifilum_thalassicum	-3.100155965	-0.88242277	-0.191887306	-0.969494163	0.445205262	0.365602676	-0.314181951	1.452366377	1.844889839	-0.008108723	0.529450908	0.981360609
Cronobacter_sakazakii	-3.097302119	-1.342612344	-0.848018051	-0.300685283	0.837701091	-0.40812913	1.333361115	0.373458554	1.04638192	0.795024386	0.388198375	0.726901066
Hirschia_baltica	-3.094977925	-3.905707574	-1.268678927	-3.660939415	-3.327426347	0.025433607	9.502742316	11.47812749	11.85684956	-0.136102465	0.429333412	0.603546849
Fuerstia_marisgermanicae	-3.091664869	-0.156828686	-0.355444661	-2.649743182	1.156695452	0.401951158	-0.457236796	-0.256071758	-0.025612326	0.779304378	-1.569364972	0.474578376
Nitrosomonas_stercoris	-3.088830213	-4.469267784	-1.795246744	1.778977278	0.110814866	0.992349253	9.029913869	0	10.44228004	-0.690614657	-0.271300292	0.518918951
Scandinavium_goeteborgense	-3.087901577	1.709816943	1.774932875	-4.98077472	3.101076518	2.919140612	-10.86812032	-1.394599235	-1.276852318	-0.374743391	0.732640678	-0.00970647
Martelella_mediterranea	-3.084828981	-2.015505162	-0.483187742	0.518646043	0.092753666	-1.792727308	-0.464200941	-0.509396617	-0.546748793	-0.170332837	0.373929408	0.903094376
Oceanobacillus_iheyensis	-3.083031832	-0.416293187	-1.05147162	1.085321814	0.414374344	1.174632004	0.089462997	2.903973761	0.404067483	0.478973508	0.419119104	0.666034116
Polynucleobacter_necessarius	-3.080919995	-2.51537281	-0.444084714	-4.402484038	-1.435793853	0.335260647	0.732835419	-1.048984713	1.676786178	0.000196285	-0.767662583	-0.409497268
Rubrobacter_sp._SCSIO_52909	-3.079269809	-2.576855469	-1.205404936	0.966937086	0.846878972	1.666518993	-1.127036461	-0.656791853	0.647419984	0.615272335	1.357776113	1.268575523
Flammeovirga_pectinis	-3.0739482	-0.130256386	0.204591869	-1.946900097	0.638468854	0.512642384	-0.273449316	-0.142671589	0.964412979	-0.555873082	-0.437000137	0.343561939
Niabella_ginsenosidivorans	-3.073123429	-1.64652577	0.223634745	-2.250174299	-0.442349978	1.51502211	-0.931447224	1.306904777	3.692402445	-0.675157255	-0.001588496	0.430268862
Rufibacter_sp._DG31D	-3.072355134	-0.300426794	0.168088662	NA	2.772373728	2.926002792	0.174937189	-0.31141121	1.225881407	-0.20743364	0.459080459	0.596980753
Olleya_aquimaris	-3.069589545	-4.101152659	NA	-2.449196512	0.851416683	1.559511071	-0.373784033	-0.933380043	-0.345520215	-0.644755886	-1.112067135	0.269832508
Deinococcus_geothermalis	-3.068529349	-1.173383603	-0.310272443	-0.768876781	3.256749026	2.674102376	0.621424985	-0.387448336	-1.027829269	0.54030804	0.828801009	0.260508197
Gilvibacter_sp._SZ-19	-3.06002319	-1.354779907	-0.685008069	0.561606344	0.784184596	-0.294635749	0	7.68267863	6.15711649	0.646274095	1.26694454	1.701797844
Cyclobacterium_marinum	-3.05996513	-0.705396442	-0.294083654	0.071537412	-1.322542376	-1.257453098	-9.681078494	-1.07374818	0.129785506	0.100778209	-0.183221209	-0.291035733
Nostoc_sp._CENA543	-3.0590551	1.61000339	1.54727116	NA	3.176630025	3.32302538	0.075696057	-0.635284575	1.290829126	-0.122647409	0.544547683	0.039396721
Allopseudarcicella_aquatilis	-3.055639996	-3.009421442	-6.350087354	NA	0.409402443	1.351168335	-0.105612255	-0.086814132	-0.362771644	-1.494113149	-1.065599762	0.759544123
Adhaeribacter_swui	-3.05435754	-0.491880054	-0.025517223	1.700330545	1.491877625	1.68241167	-1.901815853	-1.635167906	-0.24171172	0.695793651	0.544642883	0.420513775
Thiocystis_violascens	-3.052614626	0.080371738	0.47216549	-0.186704063	2.726809904	2.781704797	1.603628229	1.714362021	3.269208635	0.023479341	0.696721592	1.268341922
Chitinophaga_sp._XS-30	-3.047138788	-0.310173194	-0.326107551	0.523334816	1.167704025	1.433357682	1.008348036	2.423211951	1.693338662	0.188003289	0.097073358	0.362777282
Pelobacter_acetylenicus	-3.046625675	-1.229688684	-0.350680404	0.704923125	0.054082251	-0.469485283	0	0	8.598897393	0.389006594	0.044747763	0.156947386
Pantoea_sp._SO10	-3.045642666	NA	0.746915255	NA	NA	NA	10.85614537	0	9.366322214	0.382195504	0.453217329	0.431525622
Desulfocurvibacter_africanus	-3.044725525	-3.249752148	-2.609129537	-4.82892358	-4.749674941	-3.620999315	-1.079726749	2.154331079	-2.051895298	-0.166744494	0.141370417	-0.291812975
Flavobacterium_sp._xlx-214	-3.042714486	NA	-0.603346651	-1.885731431	NA	-2.03536751	-1.483917096	-2.231068957	-0.303621444	0.688131501	0.859293914	1.356564135
Aggregatibacter_actinomycetemcomitans	-3.041415474	-3.350780851	-3.398795821	3.647454343	0.633120705	0.281552107	-0.075932692	0.378749061	1.073104758	-0.235645222	0.116119031	1.359003213
Bergeyella_cardium	-3.040422472	-0.780254665	-0.396099672	1.880597793	1.359610256	2.325811424	0.073013163	-0.363542937	0.73873945	-0.013433414	0.116661393	0.31530858
Erysipelothrix_piscisicarius	-3.039764528	-1.689080367	-1.101026917	-1.964368304	0.963629648	0.253746294	-9.760342386	-1.200877341	0.164199958	-1.147682353	0.754872196	-0.632525051
Sphingobacterium_lactis	-3.039074799	-1.047050828	0.072798817	-5.521378535	-1.833928298	-0.967377024	-11.87197452	-1.681927647	-0.643702957	-0.317522087	-0.313380575	-0.293082061
Hymenobacter_sp._APR13	-3.038174638	-0.787442175	0.686538516	1.509350865	3.092834774	3.036191847	-1.109833961	-0.722966776	-0.90618687	-0.108269491	0.399524282	0.138606132
Alkaliphilus_metalliredigens	-3.031635515	-2.811424446	-0.220173086	-2.950709387	-1.180448328	0.139361171	0.990544067	1.215985857	1.98385017	0.142460491	-0.406590116	-0.347953356
Sulfuricella_denitrificans	-3.031116124	-2.369918038	-1.184362385	2.962362343	1.243030931	0.661334664	0	0	0	10.21270308	10.07503785	10.40585412
Staphylococcus_hyicus	-3.024874669	-2.054021014	1.256339753	NA	NA	NA	10.07438234	11.3836071	8.800605508	-0.505890625	0.929355928	1.058312285
Acinetobacter_gyllenbergii	-3.024722039	-1.796366733	-1.903810191	NA	1.383264175	1.415520895	-1.359995685	1.884273558	1.603875506	8.417852515	10.96222365	10.46760555
Rhodocytophaga_sp._172606-1	-3.01230451	-0.630351257	-0.824300814	3.115306549	1.217721373	1.626510991	0	0	6.15711649	0.355563168	-0.115939045	1.098924492
Sorangium_cellulosum	-3.009844731	-2.586161528	-0.550862201	-0.411513818	-0.531410992	0.613037452	-2.763954927	-1.343227195	-0.818531364	-0.071623315	0.236346666	0.153616666
Thermoleptolyngbya_sp._PKUAC-SCTA183	-3.005045195	-0.340823269	-1.335613933	-0.057178147	0.363815317	-0.09427453	-1.067486634	-0.288283221	-0.292009584	0.305119923	-0.020951133	-0.953864248
Erythrobacter_aureus	-3.00503559	-1.823988656	-0.51601012	2.279381771	0.358645044	1.152590929	0.035333073	-3.558119724	-0.686551686	0.701097346	0.548538942	-0.343675108
Flavobacterium_sediminis	-3.004627397	-1.715427247	-0.244555718	-2.213577651	0.414129771	0.648951201	1.503486633	3.224360919	2.419831345	0.084987971	0.229388767	0.857198524
Weissella_hellenica	-3.004187797	-1.318447526	-1.08216701	-1.656567242	0.339837504	0.656396008	-2.99288532	-0.86098977	-3.58047114	0.465524014	-1.08850808	0.328876582
Frigoriglobus_tundricola	-3.00341871	-1.791922384	0.237375451	-1.618321098	0.743193994	2.214124805	0.743755405	0.756134904	1.411181624	0.098139831	0.045480299	0.854600243
Corynebacterium_mustelae	-3.001188545	NA	-1.103841722	2.635327225	1.602012551	2.266138887	-0.798362244	-1.531287077	-0.274713002	0.17585576	0.619204462	0.538560244
Sphingosinithalassobacter_sp._CS137	-2.999494766	-2.130768165	-0.287722249	-4.26013546	0.633545278	0.953272581	-0.720400668	0.897145654	1.199156042	-0.083944042	-0.971889773	0.411601978
Streptomyces_rutgersensis	-2.992938336	NA	-4.273207279	NA	NA	NA	0.15743454	-0.221356478	-0.001555312	-0.565796976	-1.881292906	-9.593702973
Nostoc_linckia	-2.988859442	NA	-0.124514542	NA	-1.576626642	0.861600485	0	0	6.15711649	1.477128992	1.785039485	2.071847709
Terriglobus_albidus	-2.987842386	-2.970792611	-0.495227227	NA	-0.229935068	1.826651682	0.392589285	0.699282948	1.131129697	-0.069359835	0.273368283	-0.524999741
Kiritimatiella_glycovorans	-2.986109612	-0.979728523	0.356943236	0.771186672	0.272669322	1.50843378	9.029913869	0	0	-0.03861776	0.142079698	0.303692619
Agarivorans_gilvus	-2.97946869	NA	NA	NA	NA	NA	0.759435914	0.089796911	-0.511720882	-1.099733784	-0.685193655	-1.253933389
Hymenobacter_sedentarius	-2.979231135	-0.870332316	0.159467729	1.509767189	2.253456687	2.781397879	-1.055968101	-0.840115539	0.995601979	0.291307037	0.52120525	0.404868795
Anaerolinea_sp._'rifampicinis'	-2.97670338	-2.356265819	-0.409304559	-5.998148983	0.109137982	0.64653294	-0.493941189	0.84063084	0.689837047	0.373558145	0.28690353	0.066941623
Dyadobacter_fermentans	-2.975685562	-1.340653727	-0.25733885	0.915692885	-0.041558615	0.751499661	-9.734401406	-0.350304097	0.02594098	-0.242563987	-0.055438845	0.203070938
Prevotella_intermedia	-2.973242287	-0.398979787	0.209822694	-3.39279997	-2.711772184	-2.341743421	-0.047835429	0.292376816	0.930323311	0.026461367	-0.295642804	-0.397800505
Streptococcus_mitis	-2.973121616	NA	-2.878544417	-1.141259484	-2.773851713	-1.220134505	0	8.382131157	0	-0.477543091	-0.130198088	-0.031463631
Synechococcus_sp._JA-3-3Ab	-2.973067084	-1.048496549	0.119017315	0.588277178	1.399330702	1.139697064	-0.832540296	1.598278965	0.229938586	1.1770783	0.406263923	0.456348137
Luteolibacter_sp._G-1-1-1	-2.973023527	-3.163500768	-0.973023527	1.795412931	0.311419433	0.43527796	0.390154779	-0.088999861	0.107669114	0.407150257	0.315020807	2.771164851
Actinomarinicola_tropica	-2.972445894	-1.397693036	-0.128988593	2.279381771	-0.430695679	-0.101325008	2.35825711	2.353495029	1.433204424	0.286735533	1.405649966	0.664634936
Desulfovibrio_salexigens	-2.970731157	-2.899199018	-0.651424556	-3.373209659	-1.537075366	0.82140488	-0.099402641	0.24700572	0.247981286	0.981783527	2.139333898	1.78674476
Renibacterium_salmoninarum	-2.968424242	-1.468463006	-0.606329624	-1.331278063	1.637669551	1.015572804	-9.177646265	-9.177646265	-2.020531797	0.279668281	-0.162740087	0.293949123
Acinetobacter_johnsonii	-2.966687255	1.250948088	1.089443853	6.194777117	2.524336142	NA	-0.879810467	0.767074425	0.808377316	1.780606741	2.78330814	-1.000003091
Chryseobacterium_gleum	-2.965968371	-2.613702031	-0.082515468	NA	1.69921968	0.49140006	8.129283017	0	0	1.456093033	0.850510626	1.005915521
Allochromatium_vinosum	-2.962947449	-0.778077987	-0.528942576	0.332051196	0.814668549	2.987983035	0.903802304	0.599998399	0.833927394	-0.064487318	-0.258089209	0.025151379
Lactobacillus_sp._BHWM-4	-2.961188847	-0.769657389	-4.790076931	NA	4.340385	2.803368962	0.1656857	0.294309821	0.438737443	0.47509362	1.497919828	1.503070277
Hymenobacter_oligotrophus	-2.959493708	-0.590284412	-0.064174744	1.44408763	2.246382235	2.106754842	-0.839957172	-1.19824085	1.919024903	-0.58307232	-0.219001092	0.102155579
Sutterella_megalosphaeroides	-2.958598095	-3.352057342	-1.978450967	1.925465314	0.071021801	0.878370105	2.843313346	0.536056001	1.22033243	-0.583872511	-0.057415136	0.4364019
Vagococcus_carniphilus	-2.958441728	-1.932319303	0.016404795	-0.975615841	-1.327860425	0.231342413	-0.195281699	0.724366391	1.146985828	0.880157445	-0.445088404	-0.959528483
Neisseria_mucosa	-2.956107587	-1.7009173	-3.842151398	2.695347848	0.871061096	2.276280709	-0.618282896	-0.147861681	2.87416869	-0.89662756	-1.27530873	0.819826234
Sphingomonas_sp._LMO-1	-2.955952641	-1.127423314	-0.235016029	1.115038508	-1.081121054	1.620014517	-0.570192367	-1.634871008	-2.709612628	0.966209274	1.340983966	0.922824414
Gordonibacter_pamelaeae	-2.954709816	-2.311572842	-0.852952406	-2.450834626	0.044856157	0.912990107	10.12576215	8.906890596	12.93663794	0.229920818	0.013379945	0.409872167
Chlorobaculum_tepidum	-2.954533378	-2.769954811	-0.243765013	NA	-1.309471048	0.021712144	0.270166137	0.865434242	0.549451151	1.6359588	1.196962384	1.952345342
Haloactinobacterium_sp._RN3S43	-2.953912318	-3.074525532	-0.787033503	-4.032045727	-0.305543712	0.3505783	-0.356276542	-0.05417941	0.568818945	0.686877657	-0.002513959	0.034779875
Gordonibacter_urolithinfaciens	-2.952298755	-3.07334975	-0.803736573	-0.066900959	-0.07906163	0.330566723	9.029913869	0	0	-0.002397166	0.2727138	0.271062313
Lactiplantibacillus_plantarum	-2.938438197	-0.972531678	-0.375022801	4.270089163	0.909144153	0.781581128	0.348142871	-0.580001107	0.013598757	-0.479718002	-0.678275607	-0.2832682
Frankia_sp._QA3	-2.927717006	-5.347909117	0.427081072	-1.051675678	-0.592980083	-0.803687307	0.631777419	1.072427849	2.762399441	0.76599116	1.737804467	1.439683819
Lysinibacillus_sp._YS11	-2.927264386	-4.651462684	-1.757339384	NA	-0.936353279	-0.863077397	1.384389953	1.363172572	-0.128676691	-0.286532886	0.48658684	-0.11003143
Nibricoccus_aquaticus	-2.926502854	-1.201372144	0.02396061	2.637388913	0.69774338	2.180538475	0	6.682671608	0	0.270540911	0.578668048	0.527084685
Leptotrichia_trevisanii	-2.922880579	-3.162057919	-0.561570495	-4.711202099	-1.679036337	-1.050848131	0	0	0	-0.453278779	-0.285048809	-1.004103668
Echinicola_rosea	-2.920627144	-2.115606605	-1.11299235	-1.612342936	1.13195303	1.246114565	0	0	0	0.329178626	0.558612908	1.026505046
Corallococcus_coralloides	-2.919479779	-0.635044955	-0.559527453	-0.280266141	2.017566372	1.759801318	0	0	7.157114468	0.776919529	0.227767763	0.399929097
Methylocaldum_marinum	-2.911471467	-0.455142231	-1.179467025	4.898429017	2.891623839	3.303187729	-8.392072318	-7.887720454	-6.815846228	-0.311660403	0.156234732	0.53312214
Chryseobacterium_carnipullorum	-2.910929875	-0.928735917	-0.855708267	0.647590119	-1.0251879	-1.346778746	0.6055028	-1.325739521	0.261665128	1.209945723	0.595147992	1.079988042
Denitratisoma_sp._DHT3	-2.910847622	-1.217288952	-2.073857292	NA	-1.285696826	-0.309215885	-0.478178074	1.450299498	2.081562054	-1.303890892	-0.640943331	0.153645334
Spirosoma_radiotolerans	-2.908654282	2.060577759	1.309358981	1.256970798	3.85019333	3.488111976	0.239232752	2.063632297	0.98676368	1.595585365	0.764097425	1.547704705
Dyella_sp._M7H15-1	-2.907527406	0.970411815	2.79658236	1.584353512	0.823400169	1.597289848	0.665264741	2.393473134	3.11491606	-0.667106509	-0.449595148	0.646567485
Paludisphaera_borealis	-2.906093306	-2.960924347	-0.704632731	0.463400521	-1.071482693	-0.289506617	-1.142351815	-11.34580994	-1.196761481	-0.887881369	-0.139881375	-0.074021423
Sphingobium_sp._CAP-1	-2.906054008	NA	0.113725248	-0.967195152	-0.946584873	-0.373609225	-0.184071744	0.487340503	0.842291026	-9.700439718	-9.700439718	-1.510615159
Kitasatospora_sp._NA04385	-2.903823031	-1.410685475	-3.182660599	-1.775293713	-1.036621797	1.584130976	-0.625420529	-0.798460184	-1.293010504	-7.741466986	-7.741466986	-7.741466986
Neobacillus_mesonae	-2.902555385	-1.236924013	-0.582545562	-3.225834146	-2.174979627	-0.656205216	0	9.627367901	7.157114468	0.510681872	0.686692679	0.812662062
Microvirga_ossetica	-2.900866808	NA	0.611873655	0.880614097	2.650700298	1.02924723	-0.558773042	1.695169863	-0.816320948	0.789546692	0.718827865	0.444458071
Roseobacter_denitrificans	-2.896332404	NA	0.156235647	3.392427809	0.266636	-1.224454519	-10.50815131	1.144693667	-0.056940195	-1.014390529	0.341005183	-2.597128969
Mucinivorans_hirudinis	-2.8955431	-0.033918923	-0.192555793	1.231053	1.343204431	1.726353513	-0.241303875	0.12414127	-0.136072024	0.338904027	0.316440276	0.498983238
Acidobacterium_capsulatum	-2.89216312	-2.124838308	-0.736045768	-0.996457202	-0.878384591	-0.247301453	-0.47905811	-0.426250562	0.193221187	0.212496592	-0.225663466	0.355141466
Arcticibacterium_luteifluviistationis	-2.889971803	-2.914258225	-0.393487912	-0.161078872	2.033024504	2.507220945	-0.240981327	-0.059347789	0.736576375	0.029283578	0.025497521	-0.171424213
Winogradskyella_sp._J14-2	-2.88943715	-1.186717474	-0.554756609	NA	NA	NA	-0.746873369	0.310295634	0.150784816	0	8.630813152	0
Neisseria_animaloris	-2.888833384	-0.411414324	0.293538908	4.263635404	4.338947294	4.859439535	-9.95921229	-0.58101499	-9.95921229	-3.335006432	-2.865946906	-1.979479493
Runella_sp._HYN0085	-2.887827584	-1.009586049	-0.121341336	NA	1.549439419	2.078204161	0.199419064	0.474090473	0.737320977	1.289260881	1.167200103	1.453015509
Bacteroides_sp._PHL_2737	-2.886473081	-0.919278478	-0.542269994	-2.044197031	-2.05898755	-1.234607958	0.444735125	0.865982553	1.236324272	0.221991253	0.207595309	-0.000749941
Terriglobus_saanensis	-2.885342263	-0.444928977	-1.176699676	-0.461580805	0.187627003	0.452754395	-0.080897628	-0.669476804	0.204305525	-0.022970288	0.427399974	-0.066879892
Amycolatopsis_sp._Hca4	-2.882381222	-1.400489331	-0.466571609	2.084373557	NA	1.220048481	0.160848362	1.421703519	1.042619134	8.27301694	9.956714465	10.39446269
Pluralibacter_gergoviae	-2.882150634	4.361802853	1.160981272	-5.079853308	-0.475955618	-0.4501413	-1.456207678	-0.026219804	-0.068225504	0.794153548	0.939440563	0.635703153
Vibrio_atlanticus	-2.880692374	-0.862895025	-0.521124024	0.617492554	-2.410592597	-1.493462326	0.371491856	-1.373404952	0.214867522	-0.896870525	-0.328242315	-0.988871004
Methylorubrum_populi	-2.880439487	0.686894871	1.204910252	-0.985242484	3.690483535	3.964065183	1.276462945	2.074992311	1.128962745	-0.177462397	0.805885558	0.750601066
Staphylococcus_vitulinus	-2.880418384	-0.41324837	0.152989214	0.923663292	0.936158683	1.174772212	-3.614340918	-0.617101986	-1.64998256	-0.086743991	1.741320529	1.363222853
Hymenobacter_sp._BT182	-2.87877931	-0.724790523	-0.527866283	1.944026466	1.927567587	2.404172688	0.971798804	0.082746742	0.181026972	0.607135876	0.315342406	0.691776328
Enterobacter_cloacae	-2.878201472	11.67337059	8.042365089	-1.515586981	-2.289091059	-1.721569858	0	0	9.280348569	0.594775455	-0.038314485	0.553601359
Robiginitalea_biformata	-2.876299131	-0.687939405	-0.268260588	-0.710168347	1.120651769	1.237442008	0	11.0109756	6.15711649	0.220180046	-0.109907846	0.241475064
Hydrogenophaga_sp._BPS33	-2.869968012	-3.099102012	-0.534983765	-3.254796594	NA	-0.277920504	-0.470027037	-2.171686446	-1.291291314	0.733190598	1.906010891	0.688415882

Sphingobium_sp._YG1	-2.86499129	-1.676134473	1.118520587	-1.774707688	-1.297998078	-1.823317632	-0.211341457	0.55787738	0.794467331	0.692960531	0.797686386	0.886716086
Metabacillus_litoralis	-2.861243312	-3.70195305	-2.258184648	-0.341483504	2.624164992	2.548848735	-0.116922086	-1.586247935	-1.841541842	0.144702674	0.882194276	1.388899421
Chitinolyticbacter_meiyuanensis	-2.851924136	-1.062399633	0.147907315	NA	-0.601833653	0.901152239	8.157119522	0	8.624046047	0.837192243	-0.37549339	0.101575915
Clostridium_cellulovorans	-2.85138909	-1.486522309	-1.906416326	-2.237565191	0.003755277	0.370381845	-0.600482204	-0.17059665	-1.41834838	0.433547158	0.566309554	0.355398192
Butyricicoccus_sp._GAM44	-2.849791862	-2.262137765	-0.886006913	0.12714528	-0.260931432	-0.539914018	0	0	9.748955934	-0.28914845	-0.000514833	0.104489706
Oligotropha_carboxidovorans	-2.84962708	-0.129378169	-0.841438368	NA	NA	NA	0.207740789	-0.046373413	-0.997854283	0.398549376	2.045360891	-1.113656782
Bradyrhizobium_sp._CCBAU_51753	-2.842260537	NA	-2.723616041	NA	NA	NA	-0.011027815	-0.19875825	0.392493645	0	9.539158811	6.544320001
Chondrocystis_sp._NIES-4102	-2.8372705	-0.893245019	-0.132644415	0.451891223	-0.784831905	1.276090559	0.168700517	0.417158466	-0.007252432	2.652632327	2.363699714	2.435148279
Comamonas_testosteroni	-2.83467261	-4.456854138	-1.683313146	-1.035169631	-2.662653238	-1.203144698	0	0	0	0.275950081	-0.060495585	0.798426724
Aromatoleum_aromaticum	-2.834623534	-1.950100751	0.208853132	0.028160199	1.870154061	2.704371762	0	6.682671608	0	2.083151313	1.54493084	2.012327435
Pelobacter_propionicus	-2.834532765	1.14791907	1.201542406	-6.871554964	3.149867165	3.040338455	-1.601972287	-0.613014843	0.263322283	-0.002466806	1.039654387	-0.003952902
Elizabethkingia_anophelis	-2.831581087	-1.262321544	-0.428280151	1.11876354	-0.429863897	0.18440154	-3.120634518	-1.420595471	-1.722263945	0.059866637	0.189370279	0.744068363
Phreatobacter_cathodiphilus	-2.831307244	NA	-0.681560124	2.600328233	3.006032183	2.451349272	-1.178589664	0.708205915	0.390554718	9.978238539	0	6.544320001
Pectobacterium_parmentieri	-2.830359953	-2.785468495	1.180089466	-4.72476339	-1.273753364	0.064253282	0.234051604	0.366071178	0.610637416	-0.700289013	-0.758672464	-0.425400359
Pseudomonas_migulae	-2.829300813	NA	-2.213706693	NA	0.021247734	1.391297778	-9.177646265	-9.177646265	-9.177646265	-8.948367232	-8.948367232	-0.53614884
Sphingomonas_sp._IC081	-2.826548487	0.461625481	-0.482252579	NA	-0.59187087	-1.925386939	-0.089301719	0.705700796	-0.564238891	-9.12756364	0.147939342	-2.583243639
Staphylococcus_sp._693-2	-2.82336724	NA	NA	NA	NA	NA	1.424240822	1.235528595	2.454891353	0.909369767	-0.175092191	-0.005164281
Sphingomonas_sanxanigenens	-2.822076428	-0.524342138	-0.580672038	-3.993406141	1.945428854	1.71427884	0.176803037	0.653311852	0.812929374	0.799901569	1.148176314	0.077499568
Paracoccus_aminophilus	-2.81901535	-0.426182587	2.172649271	NA	-0.995253421	-0.940493652	0.350701355	0.422118967	1.048133493	-1.065705491	0.824439942	0.364779432
Kaistella_daneshvariae	-2.818481621	-1.525741051	-0.614765712	-0.604299205	-0.235746049	0.800683077	-1.789101705	-0.618257937	0.062854117	0.033375868	0.901164602	0.054544394
Flavivirga_eckloniae	-2.81451586	-2.71493696	-4.530028345	0.029599787	1.332079889	1.590865785	-0.008472304	0.636345354	1.195340851	-0.287259297	0.018063625	0.48133039
Nonlabens_spongiae	-2.813108449	-0.446128474	1.886632646	2.012218779	2.012946547	2.337201034	0	9.3781973	7.745136469	-0.707580853	0.077585011	-0.072682447
Kocuria_sp._TGY1127_2	-2.81124359	-1.287393852	1.262756991	NA	NA	-1.142533894	0	9.627367901	0	0.802721272	-0.639429336	0.995364969
Microlunatus_phosphovorus	-2.808468542	NA	-4.620118946	NA	NA	NA	0.198890239	0.085604745	0.192058206	8.442934496	8.215935782	0
Bacillus_sp._PAMC26568	-2.804956426	-0.847516888	-1.284802221	NA	-1.892494375	-2.70561624	-2.496103926	0.692183846	1.000962012	-0.935373534	-10.38658465	-1.079764202
Aggregatibacter_aphrophilus	-2.802257054	-2.336126785	-1.222449166	2.663642493	-0.94612958	-1.146817187	-2.198474091	1.408640865	1.807443769	-0.109709081	0.205419054	0.147639635
Sulfuriferula_sp._AH1	-2.801800433	-3.433155839	-0.680365278	2.890916122	0.563248771	3.283364079	1.219071893	-0.409730814	1.338553255	-0.291707555	2.245045411	3.458534118
Blattabacterium_cuenoti	-2.800187812	-1.905267162	-1.06073407	-2.35499026	-0.092017276	0.63481626	0.110189934	-0.232044978	1.030123143	0.096163761	0.422429895	0.447386308
Ensifer_alkalisoli	-2.789547192	-3.793549123	-0.490174253	-2.015918091	1.978200987	-0.75501943	-10.56207066	-0.678594944	-4.404954175	0.834093566	3.172784656	1.499465521
Bacillus_horikoshii	-2.788481704	-2.740852479	-2.447121276	0.65554786	1.32316265	2.171100792	0	0	0	-0.895717431	-0.293656177	0.490026358
Aerococcus_urinaeequi	-2.787161349	1.677930679	1.014612541	-0.997195927	3.486478993	3.125750147	0.100538248	0.34652865	0.674514467	1.266139751	3.36250615	2.637509204
Herbaspirillum_seropedicae	-2.784188498	-1.040563905	0.366980056	4.02696781	0.652645648	-0.35071141	-1.658776921	-1.675946171	0.085060787	-0.232877624	1.696582314	0.921179998
Paenibacillus_sp._FSL_R5-0345	-2.780193236	-2.698694672	-2.22757664	-2.270196596	0.28080389	0.124166608	0	0	6.15711649	0.134576031	-0.056355056	0.296706007
Ralstonia_mannitolilytica	-2.778532087	NA	NA	1.38017769	2.558404289	NA	0	0	0	-9.681823454	-9.681823454	-9.681823454
Bacillus_vietnamensis	-2.773944365	-1.908993511	-2.224431567	-0.39686961	1.99579552	1.726000592	-3.431168799	-0.734269291	-0.214531373	-1.494170117	-0.627151771	0.760651647
Dokdonella_koreensis	-2.772723146	-5.128710405	-2.512235077	0.220329955	0.949534933	2.152386258	0	0	0	-0.368240309	-0.465485139	-0.662169722
Olivibacter_sp._SDN3	-2.771998445	-0.731012347	-1.063617421	-1.501774821	0.247818381	0.303481513	-0.513910493	-0.657262308	0.153652188	0.447696305	0.945957915	0.715878274
Bordetella_bronchialis	-2.771731012	-3.807354922	-2.077620897	NA	-1.089522096	-1.863040787	0.531333648	0.689739449	0.662926929	-0.258922981	-0.107322991	-0.152007778
Anaerohalosphaera_lusitana	-2.770663291	-0.721601992	1.29119482	-1.322158908	-0.583347845	-0.951213495	-4.26623287	-3.226416444	-3.337619047	0.681098371	0.500525398	0.896790006
Muricauda_lutaonensis	-2.768077458	-0.250968902	0.943836565	-0.70501497	-0.571614369	-1.959640509	-1.527121379	-0.57705819	-0.479026931	0.018226889	0.339237617	-1.054626464
Hymenobacter_sp._BT18	-2.767916953	-0.492192473	0.317098731	-2.052692772	0.776984157	1.091898371	-10.33213728	0.131203173	0.187675863	0.544235531	0.321484864	0.395558129
Denitrovibrio_acetiphilus	-2.766957523	-3.759715989	-2.484481875	-2.415242321	-2.383150411	-3.818223704	0.186918258	0.06723369	0.748622884	0.102442289	0.077570436	-0.250049579
Streptomyces_broussonetiae	-2.759888183	NA	NA	NA	NA	NA	-0.968064484	1.454321479	0.208191479	0	0	0
Salipiger_profundus	-2.757660583	-2.823058307	-1.206264419	NA	0.666349052	2.179290915	0.293687821	-1.011560277	0.052495984	-0.350713416	1.786846244	1.841969111
Rubinisphaera_brasiliensis	-2.755598446	-0.273652265	-0.396207095	1.089190021	4.64478367	4.774342166	0.498290959	-8.10567284	3.118769054	2.563950972	0.830157772	2.558827442
Sporolactobacillus_terrae	-2.754054468	-2.195985114	0.184071864	3.102032745	0.661003496	1.593594105	0.089381995	-0.792044093	-1.650944241	0.897029362	1.271827949	1.651524881
Sphingomonas_sp._HMP9	-2.751544059	-1.402815905	-2.333691544	0.568660688	-2.83924063	-2.130388672	0	0	11.76656618	-0.142563867	-1.548590111	-0.230258267
Corynebacterium_efficiens	-2.751524205	-3.508595371	-1.847031536	2.269008646	1.88587529	0.635882644	0	6.682671608	13.04597632	0.007756162	0.522805902	1.320063664
Fusobacterium_ulcerans	-2.750269374	-1.617568582	-1.387225425	-2.542233576	-0.847243303	-0.368448815	-0.480164672	1.746808796	-0.013238148	0.245527901	0.806006265	0.634796166
Synechococcus_sp._MIT_S9220	-2.748391201	-7.181880135	0.138259372	1.841010476	-0.205761747	-0.245577209	-3.406667961	-0.73495263	-1.517746217	2.376612974	2.298846764	2.119261363
Croceicoccus_naphthovorans	-2.745186101	NA	1.223562965	NA	NA	-0.572498535	-9.177646265	2.136058747	2.108489831	2.753407104	-0.678803612	2.82130485
Geobacter_bemidjiensis	-2.745029869	-2.121343543	0.6119224	2.322251896	0.580962969	0.713458841	0	10.42283218	6.15711649	1.165925578	1.065962551	1.65719633
Treponema_azotonutricium	-2.741678429	-1.06193394	-0.628506978	0.474459523	1.266882414	1.345793946	-2.339175715	-0.180452492	0.328582129	0.614894602	0.259955339	0.156534657
Acinetobacter_radioresistens	-2.738031395	-1.312073514	-0.489732164	-5.397050649	0.53866691	0.30468899	-1.308232991	0.836026827	0.767974327	0.392885806	0.612965016	0.609911933
Candidatus_Phytoplasma_mali	-2.734874194	-2.127613026	-4.164002741	NA	0.035943762	-0.468023152	-0.348874065	0.117175027	1.454334578	2.158089865	2.709655545	1.057195402
Planococcus_sp._PAMC_21323	-2.733420887	-6.899456744	-0.824761199	-2.429601474	-4.185426095	-3.271242062	-9.069511529	0.357134819	-9.069511529	-0.236351251	0.278751041	-0.879137738
Lysinibacillus_sp._SGAir0095	-2.732548855	-1.143439003	-1.93916128	NA	-2.297450337	-0.443841951	-0.498311696	1.253629831	0.379574163	0.140978368	0.4712024	0.070062459
Aureimonas_altamirensis	-2.729928635	-0.867835654	-0.478777221	NA	NA	NA	-0.733397342	0.514630014	0.625975124	-0.354081348	-0.023101523	1.530473199
Lactobacillus_paragasseri	-2.728961584	-3.714227386	-1.369857672	-0.271656719	-0.468153137	-0.546747877	0.84143262	1.984173987	2.975889328	-0.959331725	-0.52169196	-0.257070166
Acholeplasma_palmae	-2.72390438	-1.641323956	2.356896515	-7.940395916	-0.998255682	-0.42606935	-1.564416109	0.921271181	0.772298912	-0.005047645	0.565065342	0.737366424
Methylomicrobium_album	-2.722047609	-1.568754261	0.133535233	3.586057941	2.136668937	0.969124968	-0.528710487	-0.999043302	-0.701400339	-1.263384228	-0.364479816	-0.351819076
Lentibacillus_amyloliquefaciens	-2.72103156	-2.345700802	-0.467541481	1.269054721	-1.831453334	-0.912701862	-0.118219863	-0.230111741	-0.876044659	0.738207125	0.755953164	0.899012534
Paludibacter_propionicigenes	-2.717091637	-0.852676427	0.392406409	-4.420196197	0.018822893	0.398581357	0.753353397	0.339098527	-0.605549586	0.197723002	-0.082260988	0.389210018
Oceanihabitans_sp._IOP_32	-2.716324274	-2.302973151	-4.635825994	NA	0.241470565	0.901813557	-2.256090823	1.113843729	2.465060447	-0.477448305	-0.603779129	-2.709158098
Dietzia_timorensis	-2.71596199	-0.150479053	0.73627925	3.349807127	1.024423474	1.538477598	-0.012105345	0.827161867	0.63749569	-0.351674702	0.049204734	0.57610326
Parvimonas_micra	-2.714034966	-1.241139527	-0.560358796	-0.661997899	1.130915956	1.732802827	-0.090200863	-0.110306739	0.33983068	0.844647491	0.802666799	-0.00546708
Vibrio_ponticus	-2.712019237	NA	-0.946484491	NA	NA	-1.416353227	-0.576107623	1.191563088	0.924979212	-1.883196927	-0.608260836	0.122936333
Candidatus_Cytomitobacter_indipagum	-2.711109787	-1.119939251	-0.112757909	NA	4.389989059	4.004483486	-4.385885187	-0.590590086	-1.557319579	0.272611452	0.489541115	0.192768166
Pelagibacterium_halotolerans	-2.707413484	-1.460083979	0.341791914	-1.184074195	-0.695869614	-0.728141309	-0.51563405	0.203442403	0.546423283	0.249284749	0.217471363	-0.139822256
Flavobacterium_faecale	-2.70692764	0.105249666	-0.507255295	-1.910600915	2.458832617	2.264474735	0.389606795	0.414765685	0.909851249	0.812864668	2.266444307	2.071824461
Schaalia_meyeri	-2.704948721	-0.103558251	-0.361052651	NA	-1.759283207	0.699724588	-1.36291163	-0.353677541	2.415637548	2.004427712	1.926520574	2.648262681
Devosia_sp._D6-9	-2.703188582	-4.691314482	-1.295622332	0.182467047	0.326810316	-1.802915857	0.065424109	1.622183051	2.382585185	0.82498968	1.089459997	0.31984478
Virgibacillus_sp._6R	-2.701921685	-1.302912225	0.177324684	-3.219183579	0.657171049	0.38028971	-8.925892636	1.038579642	-8.925892636	1.082319556	0.677095	0.635677518
Eggerthella_sp._YY7918	-2.699465712	-1.855694255	-0.673357492	-0.913688136	0.282653249	0.902724458	-0.962879951	0.982077684	2.25758203	0.581988341	0.359280118	0.418780273
Blastochloris_tepida	-2.696909044	NA	-2.067688624	-0.839719304	1.331161096	2.67501025	-9.222246695	-9.222246695	-1.477110226	0.492885385	0.782080905	0.992328173
Lacimicrobium_alkaliphilum	-2.693109954	-1.051813262	-0.840528779	1.005316103	-0.367277082	0.992776302	-0.182456049	0.785942395	0.793621153	-0.440638702	-1.165594799	-0.209249979
Lacipirellula_parvula	-2.692100063	-1.191986315	-0.443294273	3.991462317	1.891064158	2.306211878	0	0	0	0.801369958	0.695109766	1.432315491
Fastidiosipila_sanguinis	-2.690589566	-2.128891774	-0.102511765	2.159196208	1.810789436	2.335997535	9.330101211	9.557724072	9.929258409	0.387782945	0.623949209	0.433944013
Desulfomicrobium_orale	-2.688046881	-2.858401398	-1.374122148	0.077430208	-1.721583649	-0.625792098	9.870925659	10.05970948	10.62736973	0.940450464	0.369951502	-0.002067924
Kurthia_sp._11kri321	-2.684324554	-3.259446328	-1.094163147	-0.062825133	-0.356672493	1.846463959	1.061768615	1.595749073	0.714757122	-0.154103706	1.270145072	1.836463385
Candidatus_Solibacter_usitatus	-2.6764676	-0.380240607	0.01493574	1.254624699	1.028128619	1.150089618	0.501385666	0.092026177	0.208126937	0.350281808	0.813064191	0.055839565
Streptomyces_sp._RTd22	-2.676414086	0.015543345	-0.859127313	0.819940621	NA	2.286092574	-10.42664215	-10.42664215	-1.707718905	0.544024533	0.777895354	0.037564256
Salinibacterium_sp._CGMCC_1.16371	-2.674135144	0.3715005	-1.503984704	0.620100503	-1.09329912	-1.333893326	-0.91888464	0.244679488	1.210666168	0.579598647	0.756373075	0.046095745
Devosia_ginsengisoli	-2.671424968	-3.253459326	-1.964192061	3.433224594	-1.424616848	-0.597453445	0.668174383	0.992367491	0.619444188	10.37721053	8.607330314	9.040747255
Achromobacter_sp._AONIH1	-2.666677151	-1.401962261	-0.448904129	-0.874469118	-1.172692952	0.882348411	0.993200873	0.480021559	1.383947856	-8.739216921	-1.923295717	-1.200058109
Pedobacter_sp._G11	-2.65877071	-0.857415975	0.358963701	-0.726108996	-0.402437462	0.055853235	-2.177166586	-0.176608778	0.434776585	0.435868868	-0.462556394	-0.384445106
Mixta_theicola	-2.656851972	-1.037797854	-1.211363268	-4.978121882	0.185859615	1.066042528	-8.552496194	-8.552496194	-8.552496194	-0.796349622	-0.526493215	-0.070914687
Acholeplasma_hippikon	-2.65455091	-0.920635348	-1.012983324	0.059928215	0.273910371	0.728935158	0.138095632	0.037713074	1.313207549	-0.229803202	1.360592945	0.499953118
Sulfitobacter_sp._SK011	-2.654230418	-1.937584808	-1.303931003	NA	NA	0.607581995	-0.235989583	-0.021079495	0.904828428	-1.02039604	1.519195259	0.543245491
Muricauda_ruestringensis	-2.647301275	0.660587807	0.550090557	-5.559260493	2.592003607	2.629870991	0.199334218	0.118977268	-0.393013428	0.551716049	0.565125365	0.668537038
Muribaculum_intestinale	-2.647063565	-0.286041601	0.599601773	-1.298656547	0.984505028	1.465971052	-2.526511894	-0.747364017	-2.315336394	-0.246835051	-0.144750009	0.377842683
Labilithrix_luteola	-2.643616718	-1.376596965	0.34888095	0.25098244	0.782235313	1.032653217	-0.034377859	-2.494974656	0.721023137	-1.538154324	-0.208673099	-0.193373528
Zhongshania_aliphaticivorans	-2.642051693	-0.394598828	NA	NA	NA	NA	0	9.380166652	9.069511529	-7.807354922	-2.334867151	-7.807354922
Vibrio_aphrogenes	-2.641046048	-4.667807116	-3.339537494	NA	-1.550790012	NA	1.746901633	2.639683586	3.92261412	0.540142876	-1.134797454	-3.070019927
Celeribacter_ethanolicus	-2.640057814	-2.49068019	0.788035838	-2.457939653	-0.572717303	0.68677103	-2.193603998	-11.07012094	-11.07012094	0.284502337	1.783628982	1.786813889
Pedobacter_steynii	-2.639975396	-2.46285195	0.056445557	NA	NA	2.095014345	0.110435865	-0.052417678	-0.5559074	0.712613455	0.58780525	1.129344047
Burkholderia_cepacia	-2.634675559	-4.366148154	-0.155721382	-1.343222873	0.246202034	0.770190107	11.07499104	9.627367901	10.66662346	-0.740159366	-1.47712439	1.235980206
Mycobacterium_sp._KMS	-2.631494514	NA	-1.350916509	3.691245618	0.230626333	-1.220035076	0	0	0	-1.067573876	0.311326499	-0.402290388
Flavobacterium_magnum	-2.631358296	-0.017573713	-0.444948632	0.2661451	3.299082321	3.343651053	-0.357103891	1.230673357	1.242308336	1.348283069	1.147361278	1.318569318
Lactobacillus_sp._CBA3605	-2.628874	-1.164067272	-0.636057559	0.811005539	0.279992138	2.20257487	-0.377988986	0.186763598	0.341599571	-0.536102371	-0.097601294	0.304754899
Vibrio_crassostreae	-2.627664478	NA	-1.687671787	-1.011541622	-1.495810123	NA	9.648765614	10.60901248	9.905523897	-7.700439718	-7.700439718	1.45606561
Desulfovibrio_piger	-2.627550383	-2.987624298	-1.003566703	-3.254120327	-2.082300891	-0.056188774	0	0	0	0.66280565	1.055349843	0.664258488
Xanthomonas_sp._GW	-2.627487117	-1.886089277	NA	NA	NA	NA	-0.17835122	0.638070087	0.018302174	-7.709655951	-7.709655951	-7.709655951
Pseudescherichia_vulneris	-2.624752531	NA	0.354339805	0.227886084	-0.270760848	0.193358401	-0.415914189	-1.159036821	0.966069158	0.191080615	1.601085023	2.126024639
Miniimonas_sp._S16	-2.622761871	-3.71030193	-2.387324029	-3.092881081	-4.02674598	-4.084441662	0.106624349	-0.088226546	0.32129517	-0.719043686	-0.558368396	-1.852690515
Mycolicibacterium_rutilum	-2.622569221	-1.536815583	0.567636158	-2.238795963	-0.169583656	0.834123266	1.253118416	-8.637284195	-2.480167705	0.3995092	-0.777625809	-1.320033864
Candidatus_Izimaplasma_sp._HR1	-2.619958784	-2.33498653	-0.073604154	-4.640953558	-1.037520768	-0.454202358	-0.100584011	-0.217436021	-0.276393621	-0.070458904	0.375074952	0.589879431
Thiolapillus_brandeum	-2.619905606	-1.174667869	0.373508404	-0.663350399	-0.192645078	0.395640931	1.591883784	1.407977938	2.449599385	-0.626973121	0.321985098	0.209984796
Komagataeibacter_hansenii	-2.615948005	-0.554535301	0.601809751	-2.130663326	0.608022224	0.486167228	0	0	0	0.078658386	-0.636412424	0.289379184
Lactobacillus_fuchuensis	-2.612224581	NA	0.161466767	-2.319963908	-0.693087766	-0.721974727	-0.165505113	0.282552557	0.966570259	-0.941272365	-0.785707731	0.662984629
Jannaschia_sp._CCS1	-2.609303943	-2.839656123	-2.385532437	NA	1.235216462	1.25055168	-0.666142067	0.864094701	0.533284928	0.727877754	-0.149105639	-0.502335753

Myroides_phaeus	-2.60533187	-1.622585551	-2.498416666	NA	0.858284041	1.819678854	9.029913869	8.960529086	8.157119522	-0.047121251	-0.407179484	2.14500554
Anoxybacillus_amylolyticus	-2.603800057	-2.127566781	-0.16208182	0.66744249	-0.304939533	0.995178154	0.023026046	0.544233473	-0.152011153	2.035061803	2.031032585	3.235187144
Petrotoga_mobilis	-2.602462314	-0.737473541	0.178546865	-2.393565969	1.316927414	1.434137598	-0.010654584	0.514206827	-1.511937713	-0.294467861	-1.321929943	1.390212124
Legionella_pneumophila	-2.60125613	-1.93030235	-1.216762735	-0.248605778	-1.363360177	-0.080665227	-2.463367048	0.32228499	-1.948399356	0.433227145	1.075718888	0.273129641
Dechloromonas_sp._HYN0024	-2.589662931	1.465149124	1.414499269	NA	3.294688003	3.484525664	0	8.627369725	8.30544163	-0.003414891	0.204884697	0.566390372
Stappia_indica	-2.588685587	NA	-3.260062839	NA	NA	NA	-0.338653039	-0.048407618	0.410029419	0	0	0
Lactococcus_raffinolactis	-2.588229158	-3.944672003	-1.948865016	0.099576583	-1.492562806	-1.073645353	0.403413436	0.780809741	-0.645176498	-1.727465418	-0.185322776	-0.559022157
Cupriavidus_nantongensis	-2.578676238	-1.514873203	-0.120385541	NA	NA	NA	-8.10567284	2.727652131	4.687005628	0	5.472487771	7.539158811
Actinomyces_sp._oral_taxon_171	-2.574936783	NA	-1.676329115	NA	-2.760280761	-1.163211657	0.479089682	1.179663836	1.262970311	-1.68876544	1.777516741	0.338456871
Buchnera_aphidicola	-2.571183476	-2.765202262	-1.246545184	-1.077217067	-1.384680852	-0.14304688	-10.5099531	0.717223587	1.090001568	-0.051451261	0.557972852	0.187976409
Carnobacterium_sp._CP1	-2.570609187	-2.489608978	-3.503957854	-0.437810115	0.54488371	0.60478123	-9.797661526	0.085814195	-2.640547057	-0.076967244	-0.141229462	1.433595628
Roseovarius_indicus	-2.569069532	-3.378443621	-2.980211302	-1.24061694	0.884119288	1.208493518	9.561202011	8.627369725	0	10.52552081	9.612254807	10.76983784
Verrucomicrobium_sp._GAS474	-2.568918581	-3.270041044	0.530785622	-1.675338911	0.324921104	0.501158744	0.739802524	1.355316396	1.020689717	0.93664781	0.343788974	1.558759601
Spiroplasma_syrphidicola	-2.568729474	-1.056175469	-0.679460172	NA	NA	NA	-1.418232243	-0.180178596	0.073929697	1.230297619	-2.227951947	-1.156119717
Streptomyces_pluripotens	-2.568032105	-3.557172715	-0.17663097	NA	-0.740199431	-1.606932901	1.039325772	0.485695155	0.62430825	-2.202321291	-2.556042176	-0.906571918
Candidatus_Nitrospira_inopinata	-2.567972451	0.513799056	0.179566572	-0.728933247	3.715370187	3.717875387	0	0	9.819597937	0.701862217	1.44926498	1.038140367
Ilumatobacter_coccineus	-2.56521324	2.204850043	0.224350211	NA	NA	NA	0	0	8.30544163	3.288244969	2.64385619	3.068844408
Thiosulfativibrio_zosterae	-2.562936149	-1.775608947	0.931828497	-0.506977273	-1.277623544	0.295524807	0.138898734	-0.77848357	1.136805796	0.294029572	0.530364538	1.648440534
Akkermansia_glycaniphila	-2.560752515	-0.339785447	-0.226187595	-0.699431663	1.533836447	1.390932815	-0.349546696	0.036948522	0.023659975	1.254004656	0.94966729	1.22638453
Bradymonas_sediminis	-2.558479725	-0.440240613	-1.057490218	-0.934863277	-0.131514587	1.521691089	0.534914622	0.23548227	0.099258126	-0.205639955	0.297084009	-1.331340006
Faecalibaculum_rodentium	-2.557985624	-2.164858448	-0.817768878	3.721713953	0.963889473	-1.094478935	-0.282068611	-0.155390177	-1.058540554	0.016081921	0.711130115	0.41302061
Rufibacter_sp._DG15C	-2.556638112	-1.26423108	-1.106835194	1.292939189	-0.406433894	-0.383216833	0	0	0	0.645203382	0.616737762	-5.21E-05
Synechococcus_sp._A15-24	-2.556433703	NA	-4.205343121	NA	NA	NA	-1.311398484	0.988326796	0.990721661	-10.79928162	-0.176012813	-0.539538358
Mycoplasma_putrefaciens	-2.555793224	-1.777623958	-3.27600259	NA	-0.699382411	-0.053019366	-0.268961792	1.520306618	-0.613079353	8.94055733	5.472487771	7.63420844
Pandoraea_sp._XY-2	-2.555150629	-2.995513643	0.332242461	-4.089512012	-3.499196277	-1.160974375	0.982544529	-8.04736934	2.007424883	-0.080766062	0.105307347	0.576493018
Parolsenella_catena	-2.552644403	-0.290708756	-0.188283746	-2.969282635	-0.632252708	2.852693301	-0.863735378	-0.454592064	0.704514088	0.277810286	0.575974245	1.795666665
Phenylobacterium_zucineum	-2.545415453	0.052936424	-3.099436923	2.033220273	0.261702096	-0.361898793	0.758740265	0.703174072	-0.368965959	-0.161788098	0.051456749	0.212326513
Bradyrhizobium_sp._ORS_278	-2.544678905	NA	-0.847137136	NA	NA	NA	0	0	0	-1.369516398	-0.789420627	-0.801165843
Mucilaginibacter_sp._HYN0043	-2.540399032	-0.561721302	-0.268216143	-3.800853356	2.294296976	2.494428331	0.299822096	1.633807209	1.701557571	-0.517246604	-0.593130098	0.68818271
Corynebacterium_sp._4H37-19	-2.539093184	-4.280604184	-0.272678613	-0.534065789	-1.385856268	-0.641389052	0	0	7.745136469	0.258961556	1.172632592	1.167040419
Anaplasma_marginale	-2.538367473	-2.180425414	0.071961748	NA	NA	NA	0.118743289	1.863058818	0.353094551	0	0	0
Campylobacter_pinnipediorum	-2.538204316	-2.941567852	-3.532359195	NA	-3.372514298	-4.07311461	-2.352459442	-1.377753069	-4.604733888	-0.14495251	0.150172822	-0.939191345
Dickeya_poaceiphila	-2.536638053	-0.759369972	0.757874584	NA	NA	NA	0.367899194	0.253072254	1.378761533	0	5.472487771	8.266786541
Roseovarius_sp._MME-070	-2.535275377	-0.862850035	-3.81554432	NA	-0.070883486	-1.448357572	-1.298311091	-11.61613068	-2.07591544	2.082601361	-8.186525318	-0.647366507
Acidipropionibacterium_jensenii	-2.531270396	-3.270023717	0.181553634	1.087412296	0.473656199	0.909237326	-0.920982026	-0.955099135	-0.242302235	0.190256166	0.550197083	0.708761875
Kosakonia_sp._SMBL-WEM22	-2.529330932	-2.772071479	1.688187249	3.573871554	4.247408512	3.777072355	1.156735061	1.681933072	0.29698578	0.030059518	-0.444247997	-0.701599029
Phyllobacterium_zundukense	-2.526505804	-0.533299536	-0.429392792	NA	-0.11189288	0.935713052	-0.147732396	-9.177646265	-1.432509796	-0.027562578	-0.414059234	0.998949096
Gemella_haemolysans	-2.524649161	-2.530317911	-2.204171269	NA	0.64951997	1.639651736	-8.624046047	-8.624046047	0.70095352	-0.660892843	-0.088425309	-0.548208021
Luteimicrobium_xylanilyticum	-2.524175925	0.05235467	0.998947322	NA	2.750739968	1.342314738	0.627201945	-0.311066186	-0.114271959	-1.249818387	-0.946453193	-0.26711521
Nocardiopsis_alba	-2.522780242	-0.93498244	-0.984934262	3.486682983	1.143887141	3.495133548	-0.185158364	0.599700614	1.008729259	1.182790168	1.922562986	0.603158245
Vagococcus_penaei	-2.520312345	-0.65615218	-3.182890673	NA	NA	-0.133726331	-0.541754942	-0.715941299	-0.533787643	0.760867265	0.209568594	1.335961745
Synechococcus_lividus	-2.51473899	-0.225006263	-0.782756804	3.131421514	0.916196244	1.802228234	0.613084639	0.199879932	0.912421347	-0.307484358	-0.647489832	-1.701181914
Francisella_tularensis	-2.514320931	-4.179776658	0.395660243	NA	1.749678781	1.6711314	0	0	6.15711649	-1.726020102	-0.107192836	-0.367946371
Pandoraea_thiooxydans	-2.512400257	-1.291387085	-1.035962213	0.125360341	0.975232095	-2.301608111	10.6190553	8.566400396	0	-1.264847907	1.010311653	1.696952584
Pseudomonas_prosekii	-2.509440848	-3.028034539	-0.998150259	NA	-3.594073551	-1.640149422	-9.764871591	-0.372944951	-9.764871591	-0.12197738	-0.511868875	0.310506916
Virgibacillus_sp._Bac332	-2.508673217	-1.001461361	-2.691224148	NA	1.030442098	1.844008804	-1.784415618	-0.291784784	0.657968256	0.778904383	2.315512541	1.275880643
Brevundimonas_sp._LM2	-2.507049639	-2.816925074	0.190930113	NA	NA	NA	0	0	7.157114468	-1.032654086	-0.376473918	-0.677052693
Pectinatus_frisingensis	-2.506239159	-2.471000892	-1.617892436	-0.415933824	0.715552818	0.6682166	-0.106573181	-0.187824735	1.4040456	-0.11942368	-0.287412595	0.586150793
Fusobacterium_gonidiaformans	-2.505249874	-0.717158823	-0.188329582	-1.550049476	-0.09745581	1.305759666	0.885371039	0.431345018	-0.417236603	0.580578073	0.941147901	0.940038424
Streptococcus_pantholopis	-2.504251043	-3.215793289	-1.251611008	0.056093852	1.59650112	0.929552049	0	0	9.819597937	0.550531766	0.044206967	0.612060716
Raoultella_terrigena	-2.499475254	-2.830066183	-0.295629006	NA	-4.363302256	0.80286817	0	0	0	1.091090817	1.329220587	1.628042512
Erwinia_amylovora	-2.499023798	-2.518038176	-3.715133378	-2.432767189	1.456019853	NA	0	0	0	0.369234494	-1.27581842	-9.087462841
Helicobacter_pullorum	-2.498871678	-2.469396784	-2.251466681	NA	-4.450728007	-3.048090607	-0.528781182	-2.141996107	-0.706294857	-1.288535008	-0.818082861	-0.936978833
Methylomonas_sp._GJ1	-2.497964781	-0.015442185	-0.737919625	-2.7589919	2.653709466	2.728967023	0.362021596	2.012340142	2.780187728	0.052369604	-0.23217634	1.852918252
Klebsiella_aerogenes	-2.497363928	3.99026959	1.210212313	3.431747894	3.200165887	3.726127944	0	10.72125302	8.157119522	-1.092191478	0.367379592	0.607145805
Syntrophomonas_wolfei	-2.497215909	-1.186830147	-0.698248552	-3.204746666	-0.100816791	-0.886733248	-0.666794291	1.656764776	0.358309147	-0.173738792	-0.604099113	-1.225908882
Leptospira_biflexa	-2.490405862	-2.483322239	-3.692039723	NA	0.020160259	-0.440210105	-9.932617823	-9.932617823	-9.932617823	0.366495019	-8.087462841	1.868670789
Pelodictyon_phaeoclathratiforme	-2.488965522	-0.764166771	0.122315673	-1.020846047	-0.771583791	0.295647502	0	0	0	0.851634819	0.371844189	-0.173302162
Lactobacillus_kullabergensis	-2.488011931	-4.983873107	-4.266411925	-0.557287747	-0.62840373	-1.166722615	0.067519669	0.841062009	-0.667394868	-1.084560433	-0.440304258	-0.33519226
Capnocytophaga_endodontalis	-2.486002409	-0.794892503	0.054407288	-1.565775481	-0.011935498	1.990309943	0	0	8.624046047	0.188479965	0.36745258	1.104679147
Thiomicrospira_sp._S5	-2.484402792	NA	-0.209079935	1.942543127	1.566538836	2.044543466	-0.148233054	0.253842779	0.341468117	0.240967789	-1.628928599	1.088503153
Sulfurifustis_variabilis	-2.484033592	NA	1.704401551	3.374410725	3.841995393	4.007102799	0.237097763	1.393149572	1.845060872	-1.958179824	-0.628031223	1.732181395
Pedobacter_cryoconitis	-2.480174774	-6.901850376	-2.292728602	-0.855906667	1.403836597	2.303544243	-0.133936034	0.515791469	0.30017535	1.536943825	0.087607786	0.566947161
Marinobacter_sp._JH2	-2.478979739	-0.087275057	-0.944633107	NA	NA	1.472184433	0.176890274	0.813533529	1.064246375	11.49285462	5.472487771	6.930737338
Caldicellulosiruptor_obsidiansis	-2.473263892	-1.798670877	-0.203988546	2.167509028	0.629860674	1.464387416	-9.562936888	0.690804252	0.929827623	0.318043809	-0.956992421	-1.116863758
Rhodothermus_marinus	-2.468517631	-0.903839286	0.145163348	0.75939703	2.193717841	2.396469266	0.381808621	-8.933959107	2.870317145	0.011301071	0.267072795	0.202125201
Streptococcus_porcinus	-2.468313229	-2.340200296	0.108563441	-1.415559256	-1.190698142	-1.207638005	-2.717197127	-10.88347572	-0.254447023	0.219719876	0.682264557	1.060937616
Vibrio_sp._SM1977	-2.468027279	-1.545749575	-1.674866239	NA	NA	NA	0	0	10.13687579	0.097214528	1.095229636	0.533682564
Lancefieldella_parvula	-2.466341993	-2.172262828	-1.443538284	-0.689903894	-0.128791703	-0.261601976	0.006475405	0.709254996	0.533150186	1.079400566	1.014608721	1.219681996
Lactobacillus_zymae	-2.464141695	-2.644259309	-2.070765569	2.137460323	1.897100092	0.975075745	-0.298966946	1.056394351	-1.635639108	0.259947936	1.277600171	1.609356275
Mycoplasmopsis_glycophila	-2.464010153	-1.275153336	-2.790991476	NA	-0.612377958	-2.196821597	8.129283017	8.110425888	9.793234257	0	0	9.994353437
Neisseria_sp._KEM232	-2.460210349	-3.771201811	-2.018905125	-4.389014504	-2.419908512	-3.243751556	-1.244374329	-0.456411489	0.346197134	0.289004143	0.67519579	0.105794317
Staphylococcus_equorum	-2.460075018	-0.935959162	0.052922222	0.961189793	-0.026854454	-0.431878164	0	8.559465045	6.15711649	-0.293625387	0.396100449	1.195185947
Basfia_succiniciproducens	-2.457396787	NA	-2.124328135	2.632910176	2.236908063	1.842167438	-0.548213679	-0.258439687	0.673369701	-0.625192346	-0.481745202	1.365505913
Chroococcidiopsis_thermalis	-2.447012944	-3.23388806	-1.314993028	NA	0.87021248	-2.013420516	0	0	0	-0.988684687	-0.218293338	-0.174903496
Archangium_gephyra	-2.446348785	-3.579420062	0.035470401	-0.795426491	-0.650748279	-1.058138001	-0.22121056	-0.140748071	-0.740380755	0.86132981	0.582187634	1.528669401
Hyphomicrobium_denitrificans	-2.445430529	-1.874643807	-1.433380976	2.856618357	2.145037612	2.472643277	0.733006525	-9.222246695	1.67125962	-0.149556388	-0.147936984	0.146128044
Phoenicibacter_congonensis	-2.445237737	-2.296315007	-1.93056368	-3.642701572	-1.305882629	-0.231614345	0.982544529	-8.04736934	1.531088268	0.785409091	0.725092615	0.112770479
Burkholderia_lata	-2.444528796	NA	-2.575773329	NA	-0.039235163	-1.519609763	1.243709498	1.218186739	1.902710949	0.516575526	-0.758651304	-1.120679062
Corynebacterium_glaucum	-2.443827266	-2.12475773	-0.348736078	-3.457070801	2.909817434	3.022098798	0	0	0	-0.172830171	0.857261795	1.046698879
Pseudomonas_agarici	-2.439689399	-3.324580921	-0.229329533	-0.15589701	0.229596214	NA	-0.211352443	-0.20627761	-0.25476307	-0.341936259	0.246949823	1.641337673
Veillonella_rodentium	-2.439039002	-0.087964561	-1.160249455	-1.118466216	1.464601166	1.892256144	0	9.426646347	10.36233838	0.676127917	-0.016349013	0.716590901
Methylocystis_sp._SC2	-2.436247747	NA	1.268917819	0.493985518	1.634639388	-0.83213559	-1.33139345	-0.65314237	0.164067862	-0.271217815	-0.842850394	0.650938896
Syntrophothermus_lipocalidus	-2.43411218	-0.929244905	-0.071253821	-1.895113428	0.576264395	2.130498828	0.504111216	-0.22467555	-0.132939601	1.146773064	-0.038783377	-2.332193854
Deinococcus_sp._D7000	-2.432959407	-3.811471031	-1.782002082	NA	-3.070991832	-0.367958504	0.088475532	-9.573302566	-2.416188097	10.09364204	7.222389201	9.884678808
Kingella_oralis	-2.432128601	-0.877380713	-0.823932202	NA	NA	NA	1.104796144	-8.637284195	-8.637284195	-0.240542344	2.008474708	1.66187811
Campylobacter_hyointestinalis	-2.424266285	-0.918435832	-2.024281442	2.789872767	0.193930464	1.421980077	-1.130727515	0.291199791	0.613547718	0.877672603	-0.822895964	0.458442379
Geovibrio_thiophilus	-2.424157922	-1.624624736	0.000267705	-2.415691744	-0.723371285	-1.251841257	0	0	0	0.75312074	0.644976707	0.355948443
Paraburkholderia_phytofirmans	-2.422064766	NA	1.324449555	NA	NA	NA	0	0	0	0	5.472487771	7.63420844
Kyrpidia_spormannii	-2.419560058	-1.535650797	-0.116783063	-0.399355877	0.615330284	0.306472623	0	9.590416761	6.15711649	0.39286938	0.250933551	0.111349143
Salmonella_enterica	-2.416579814	4.472145125	1.027380258	-1.543169115	-2.729440903	-1.87578459	-0.972207023	1.05704298	-10.63398512	-0.343384178	0.026731524	0.139782557
Phytobacter_ursingii	-2.41057312	-2.402242627	0.052312285	-4.327566171	-2.44994017	-0.42900424	-1.778772264	-2.027919485	-4.49817272	-0.680937701	-0.295230136	0.186225818
Labilibaculum_antarcticum	-2.409879141	-0.085657674	-0.00881307	-3.740054921	-3.028290235	-1.064357161	0.170528027	0.38579376	1.357999516	0.551957692	0.552612124	-0.028430008
Sodalis_glossinidius	-2.409868966	-1.079330765	-0.613440845	1.493616225	0.809197409	0.873580025	0	0	8.624046047	0.928146592	1.381301943	0.684484445
Gibbsiella_quercinecans	-2.404176507	-6.587806423	-0.491584701	NA	-0.44633363	0.54308162	0.453589471	1.315335775	0.966194478	1.985139965	1.51163851	1.464332796
Actinopolymorpha_singaporensis	-2.402844076	-4.51887331	-1.701402775	1.07295526	1.134435917	2.939302752	0.103843594	-0.507018418	-0.372481955	-0.679536915	-0.223003138	1.256220982
Lysobacter_soli	-2.401187156	-0.062324135	-0.982371634	-0.754522237	2.93486001	3.200158049	-1.648520923	-2.709435531	-0.364345086	1.847883696	1.397727346	1.286415258
Burkholderia_sp._KK1	-2.400079276	-2.133014993	-0.705705558	2.07446474	NA	0.732387423	-1.090607553	-0.379249518	1.370443738	-0.622102791	-0.607757716	0.263704743
Syntrophobacter_fumaroxidans	-2.396195457	-0.365752393	0.677156199	-2.14758658	-3.57780322	0.75731988	0.472036961	0.704541927	1.534146975	0.78567706	1.297499862	1.437280038
Propionibacterium_australiense	-2.395316166	-2.576633066	-0.463356949	2.607362176	-0.240691828	2.226381075	-0.327607426	1.233683564	1.528506173	-0.43555546	-0.936801994	-0.664793702
Bacillus_sp._FJAT-18017	-2.390498266	-4.247651038	-4.847067633	-0.637899567	0.727326567	0.327095837	0	0	0	-0.979111213	-1.326514132	0.283859226
Nostoc_sp._TCL26-01	-2.388819979	-1.268190314	-0.104044882	-3.1480892	-1.373839431	0.381115113	-9.177646265	-9.177646265	-9.177646265	0.458946647	1.611422152	0.720809826
Microlunatus_soli	-2.387676162	0.08039097	-0.433490789	1.1684148	3.402566489	3.191275345	1.514015994	2.509798419	2.45293328	0.344402753	0.489059402	1.243806305
Pseudomonas_asturiensis	-2.387481777	-4.414242845	-4.199132181	NA	NA	NA	0.102882782	-0.438365392	-0.247339672	-7.745956618	-0.930035414	-7.745956618
Staphylococcus_simiae	-2.386548942	-1.405761794	-3.408312401	-0.323795078	0.7868425	0.823513802	0.557397017	0.111360598	0.235992864	0.778247633	1.093516846	-0.24817685
Halomonas_elongata	-2.386333659	-0.573077425	-0.39350405	1.193786534	1.87735752	0.768556765	-0.259755734	0.779218336	1.146693166	-0.281825884	-0.19817181	-0.91619185
Agromyces_sp._LHK192	-2.382163202	NA	-3.67914494	-1.622587573	-1.562287063	-1.269333144	-1.582206892	1.003537641	-0.780930696	-8.77478706	-1.552397859	1.505983711
Candidatus_Erwinia_haradaeae	-2.377637527	-0.94260463	0.712889811	NA	0.923267802	0.258753778	-0.260062839	-0.264279528	0.072769899	0.080266007	0.287988988	0.583546011
Succinivibrio_dextrinosolvens	-2.376915086	-1.682576101	-1.310148128	3.004591096	1.796760825	1.565787205	-2.145087465	-2.139523133	-1.041560008	-0.37890726	-0.108391875	0.245294915
Kingella_kingae	-2.37679003	-1.42569963	1.021903956	1.95081675	1.026291858	0.972462038	1.281542165	-0.645603677	-0.4115495	-1.378514018	0.594555459	1.387021949
Saccharomonospora_cyanea	-2.37609707	NA	-0.543163039	NA	2.910596028	NA	-0.052586314	0.275953133	-0.192645078	0	8.91607949	0

Rhodobacter_sphaeroides	-2.374708752	0.108495071	0.098991587	-0.920079087	0.126153294	2.075131707	-9.64385619	-1.016486465	-1.898719721	0.681669454	0.646563588	1.264998003
Desulfovibrio_marinus	-2.374388545	-0.499449337	0.03109096	0.653877531	1.24017474	1.565822622	0	0	0	-0.138024095	-0.08543525	-0.236641563
Aeromonas_sp._CA23	-2.371510124	-3.361456459	-0.500879844	-1.985852515	-2.639462363	0.944445151	-1.913058539	0.309333883	0.293509673	9.936637939	10.49558525	7.380824669
Clostridium_estertheticum	-2.370276585	-2.396601875	-0.743179095	-2.765678142	-0.391952464	-0.268991325	-9.076815597	3.098281676	2.573989343	0.34982941	0.419722563	0.786791871
Rhizobium_grahamii	-2.370224575	0.134226868	-0.484889062	-1.521822355	2.788315566	2.714634908	0.793828517	0.692799184	1.11826138	0.5749093	-0.185441304	1.050697765
Aureimonas_sp._AU20	-2.36881663	-6.473374976	-1.95701543	1.705901582	0.724964497	-0.124755031	0.166311453	0.131879635	0.66915406	9.557143918	0	8.520944699
Clostridium_taeniosporum	-2.365427979	-1.529750838	-2.209797243	0.308673602	1.755681911	2.342058911	0.712484887	0.332568482	0.134988721	0.545094726	0.540164667	0.11043872
Opitutus_terrae	-2.363409099	-0.662620806	0.116412472	1.195672423	2.646934903	2.064435944	0.126986354	-0.399265891	0.362429254	0.460281953	0.14350411	-0.134742062
Legionella_israelensis	-2.36212162	-2.838296502	-0.8481539	-1.143632688	-2.814568412	-2.268080059	-0.404714924	1.104862062	1.144240362	0.189339277	-0.609647557	0.209405176
Lactobacillus_dextrinicus	-2.361260196	-2.549163238	-2.712139664	-2.624611577	NA	-2.737379508	-8.091313724	1.300612916	2.070385046	-0.527804621	-0.195412316	-0.579566049
Actinobaculum_sp._313	-2.36067231	-3.415889206	0.923046945	2.209697395	2.796773094	2.364127277	1.375990169	1.469563279	-0.167539777	-0.50081855	-0.191785246	0.527465038
Parascardovia_denticolens	-2.359455292	-2.831056822	-1.51852716	2.364849515	0.608180448	0.408042611	0.320350016	0.341727366	0.857906747	0.236635433	0.431619077	-0.359627256
Desulfobulbus_propionicus	-2.35914879	-1.685410406	0.206243266	1.799373095	2.290082107	1.920183194	0.339758464	0.486516899	-0.324930583	0.094225116	0.130023885	-0.268061334
Treponema_sp._Marseille-Q4132	-2.355084146	-2.624029877	-1.221424131	-2.172748675	-0.979592657	-0.008284986	-0.493149433	-1.242326444	1.593367506	0.695163198	0.03535707	0.465002264
Alistipes_indistinctus	-2.353972294	-0.242274988	0.032704165	-1.374968048	0.544810141	0.57011066	-0.957394013	0.428029534	0.657964115	0.076601912	-0.002909677	-0.126424574
Actinoalloteichus_hoggarensis	-2.352443832	-0.923719156	-0.612515824	NA	0.497252263	1.42170123	0.217723321	3.23233488	1.822133613	7.129283017	5.472487771	10.23681547
Pseudovibrio_sp._FO-BEG1	-2.351812375	-2.871186534	-0.110374198	NA	1.051250084	-1.793052339	0	0	0	-0.627849868	-2.084945604	-8.900866808
Streptomyces_koyangensis	-2.351660802	0.008444689	-0.58371409	1.557309556	4.042525436	4.144418126	0	0	0	1.88915976	2.011629121	1.381203904
Plantactinospora_sp._KBS50	-2.350309896	0.932885804	-2.874469118	NA	NA		0.105087485	-0.078063489	1.162685311	0.22862282	-0.44646821	0.081448092
Lactobacillus_ginsenosidimutans	-2.347019076	NA	-3.460178033	-1.243532501	NA	-0.855915003	0.610279269	1.32536459	0.619986891	-0.629400007	0.552922757	0.599634562
Phreatobacter_stygius	-2.33888816	-1.050730802	-3.007840753	-1.525416558	1.624450791	-2.766424658	-1.995420176	-3.479027161	-10.16169877	-1.732488088	-0.694982566	0.21661441
Candidatus_Reidiella_endopervernicosa	-2.338334829	0.006479648	-0.474226806	0.426464101	3.852248851	3.855457477	0	0	6.15711649	1.823373297	1.526998888	1.47143134
Tannerella_sp._oral_taxon_HOT-286	-2.336627387	-0.215602455	-0.064947559	0.408360424	0.360568774	0.407588	-0.546045754	-0.116605554	1.335464348	0.096922002	-0.080136754	-0.056089083
Radiobacillus_deserti	-2.331	-2.123999629	0.169073603	-0.145110833	0.976492487	1.417718316	-0.700061143	-0.412324776	-0.727268824	0.840432582	1.124305723	1.318653532
Solitalea_canadensis	-2.329695554	-8.025054169	-2.231611994	-5.048940909	-0.517072133	-1.191737437	0	0	0	-0.150500632	-0.124806456	-0.03825874
Desulfomonile_tiedjei	-2.328258194	-1.096911669	0.751621257	0.194995741	1.593230117	2.478693415	-0.905682964	0.010576293	1.543190925	1.243713108	1.536221907	1.843813106
Elusimicrobium_minutum	-2.327802338	-2.597883097	-2.254273303	1.040989664	NA	-0.046799124	10.16056477	10.40552443	0	0.757628763	0.304880631	0.963340657
Asticcacaulis_excentricus	-2.322548475	-2.487571996	-0.537796849	-1.237584056	-1.802209106	-0.35460237	-1.677159781	-0.732021137	-1.092686343	1.890498211	1.948593876	2.370797091
Schlegelella_thermodepolymerans	-2.3151651	0.094814494	-0.769391528	-0.468924196	3.121257967	2.979664308	-0.553124177	-0.092916902	-0.456334243	1.46695444	0.770584103	0.639296971
Desulfotalea_psychrophila	-2.313709818	-1.368592929	-0.535126984	-1.673400465	0.69810499	-0.112822965	-10.00077468	-0.126677368	0.924445939	-0.127377972	0.098437755	0.436423236
Phocaeicola_salanitronis	-2.310790312	-0.045541679	-0.084524627	-2.362450563	-1.791131841	-1.42946019	-9.552494273	-0.125847926	-9.552494273	0.129165615	-0.061721098	-0.215055709
Pseudobacter_ginsenosidimutans	-2.30964488	-0.594447101	-0.510264605	0.702306717	0.599612021	1.863797835	-1.911451397	0.181845146	-0.723366379	0.460241258	0.215029702	0.637508853
Thermocrinis_minervae	-2.309363457	-1.300462405	-0.7946894	-2.878731145	1.79687102	1.542163401	-0.937341785	1.892939503	1.342454618	0.485045846	1.596568038	1.195647291
Bacillus_vallismortis	-2.306918901	-2.964077752	-1.045566939	-1.769719506	-0.423068982	0.503667445	-0.800256834	0.416504974	0.660497378	-0.626143169	0.619789424	-8.992465383
Celeribacter_marinus	-2.306352289	-1.547382394	2.958169029	3.033903185	4.21647237	4.003926809	0	0	0	0.651417049	0.399657919	-0.122553182
Serratia_liquefaciens	-2.305912762	-4.373603256	-2.677434692	NA	NA	2.116944116	0	10.19004687	10.98143885	0.990417877	0.972525605	0.966646442
Azospirillum_brasilense	-2.303848837	-3.440513454	-0.606806134	-0.318363396	0.083504928	0.721332305	-0.329739248	0.05376892	0.325407181	-1.604657596	-0.17693372	-0.125104908
Ralstonia_solanacearum	-2.303305051	0.545297282	1.058994227	-2.167061267	2.922718929	2.528977246	0.464519311	-0.890517417	0.416288608	-0.217174518	0.701969618	-1.467192952
Campylobacter_blaseri	-2.29997008	NA	-4.400659218	2.885938549	NA	-1.052156996	-0.706741784	-0.751981693	-0.25050456	-0.046837216	-1.684501395	0.611434056
Megamonas_funiformis	-2.29644664	-2.040801443	-0.479494336	-0.883263364	0.779246305	1.500924289	-2.261677445	-0.759668224	-1.067818794	0.145047111	0.172897661	0.117429825
Alistipes_onderdonkii	-2.293982558	0.468098557	0.303713795	-0.474172874	2.497001659	2.36091636	-1.102324978	0.820824694	0.773653853	0.250930565	0.616827584	0.177918343
Staphylococcus_carnosus	-2.29198578	-0.856429261	1.816425653	1.641492505	0.606078857	1.070417728	0.038948248	-0.118792937	0.751505126	1.313812538	2.358818981	3.079852419
Aequorivita_sp._H23M31	-2.287878825	-1.234258986	0.258321769	-0.555602726	1.740841204	1.215147221	-0.229059014	0.58925588	0.840142368	0.849148206	0.458789055	0.206880961
Mycobacterium_basiliense	-2.28771238	NA	-3.567981323	NA	NA		0	9.426646347	8.451211112	9.367779474	7.811644421	9.0750325
Janthinobacterium_sp._HH102	-2.285805713	-3.881298882	0.99434078	0.045485957	1.910767074	1.74874727	0.375128135	0.497494103	0.173271271	1.378101758	0.412115436	-1.057890959
Mesobacillus_foraminis	-2.283429173	-2.429615964	-0.344940427	-2.829468574	-0.150620818	-0.112822965	-1.027333306	-1.09671809	0.029247356	-0.028975442	0.181940615	-0.532083243
Alistipes_megaguti	-2.281961158	0.48096394	0.252141318	-0.328823088	2.692692423	2.565413462	0.887481474	1.640662761	1.385957613	0.169478502	0.522306246	-0.040545128
Lactobacillus_brevis	-2.281919778	-2.224592721	-1.339646293	2.199411572	0.366867239	-0.578985194	-0.853829391	-2.045315756	-1.991749888	-0.021684293	0.943871335	0.797150055
Roseococcus_sp._NIBR12	-2.280705432	-1.127976819	0.247367745	1.539452924	0.668525501	1.371725478	0	0	0	-0.053864948	1.370212706	-8.992465383
Chelatococcus_daeguensis	-2.279610581	-1.323379645	0.601120854	-0.731651794	NA	-0.996809372	0.286691929	-1.075539119	0.134435004	2.788617415	2.062233098	4.07311548
Nostoc_carneum	-2.275312736	NA	1.544669795	NA	NA	0.560681866	9.502742316	10.88069318	11.30129041	-0.380015966	0.145677455	0.36833856
Chitinophaga_caeni	-2.275145628	1.406518059	1.317046906	-1.649122195	2.876248968	2.804065601	-0.759082006	0.98054721	1.066157521	-0.271636015	0.365314717	-0.150225576
Turicibacter_sp._H121	-2.268328281	4.364659042	4.356779031	-1.649811745	7.90329582	7.368394268	-8.448613965	-2.847183484	-3.443545463	2.366468034	7.18482746	6.262507387
Vulgatibacter_incomptus	-2.266679879	-3.586328474	0.189346511	2.658937482	-0.073252982	0.804554478	-10.25158892	-10.25158892	-1.001074945	-0.530192543	-1.066769012	0.706192322
Nonomuraea_nitratireducens	-2.266628553	-1.861355525	-1.786482609	1.001467065	-1.561024859	-1.767068608	8.246199041	0	0	0.624366355	0.146556719	-0.228480329
Chlamydia_sp._2742-308	-2.265554398	0.635000004	-2.192764978	NA	-3.243247233	-3.033840013	0	0	0	0.100534849	0.936382355	0.737297221
Candidatus_Amoebophilus_asiaticus	-2.263470991	-1.753796617	-0.657700028	2.280925121	2.130942407	0.990493958	0	0	9.154818109	-0.301221741	1.571236363	0.557374711
Chloracidobacterium_thermophilum	-2.262675124	-1.910788558	-0.587659968	2.876264632	1.996402255	2.479382635	-0.469573941	0.189709236	1.772620632	0.927303581	2.463317119	3.379292706
Pseudonocardia_autotrophica	-2.259743264	0.044037484	0.362308556	2.253516525	0.048877934	-2.182008478	10.61069268	8.559465045	12.17849368	1.605739262	2.58118217	-1.587425925
Listeria_welshimeri	-2.259743264	NA	-0.955962516	2.239025013	0.941583314	0.266326847	-2.654806735	-1.257277451	-0.934093925	8.282314694	7.222389201	9.444325417
Geobacillus_sp._44C	-2.259743264	NA	1.106578951	NA	NA		0.057251878	-3.479027161	-0.453711615	9.571121208	9.225609977	0
Gordonia_sp._HY186	-2.259743264	NA	NA	4.020250446	1.42343994	-1.485966253	8.246199041	9.384097309	6.15711649	0	5.472487771	0
Chryseobacterium_indologenes	-2.257286843	-0.07211513	0.23234814	-3.992656245	-1.729244007	-0.53798904	-0.525320453	-1.332114361	-0.48106207	0.322210252	0.28155279	0.187881635
Arenimonas_daejeonensis	-2.255257055	-1.688851521	1.699153703	3.383576439	4.3783552	3.695190272	8.6039638	8.566400396	0	-0.140085999	-0.134357641	-0.301071138
Pseudarthrobacter_chlorophenolicus	-2.252073206	0.129768294	-0.683724702	-4.785186103	1.446466562	0.778427949	-0.279696133	-0.666986371	-1.108220219	0.046426219	-0.239529731	0.233568552
Corynebacterium_rouxii	-2.250888758	-0.346179147	-0.10666062	0.964947013	4.835137505	5.026827181	-0.763857758	-2.266280232	0.175317174	1.376412742	-0.960852687	0.238943401
Marinobacterium_aestuarii	-2.250447016	-1.086449283	0.264292509	-1.941970747	0.588425716	-0.351893208	0	0	0	0.168574795	0.092771971	-0.380513418
Blastomonas_sp._RAC04	-2.249146191	-0.3190723	0.593039282	1.355255704	0.09753808	1.476286994	8.129283017	9.110423278	9.154818109	0.95986694	1.202041937	1.205702897
Paraburkholderia_hospita	-2.248665145	-2.897176302	-0.8687945	0.463347849	2.041573657	1.646269845	-0.151492954	0.174011366	0.064714871	-0.509756559	0.606988325	1.109153194
Altererythrobacter_sp._ZODW24	-2.247927513	NA	-1.209008524	-0.366127899	NA	1.014026885	0.323314419	-0.399763592	0.191672307	8.442943496	0	0
Candidatus_Doolittlea_endobia	-2.247715103	-1.701504899	0.545809768	-6.191367713	-0.653906619	-0.298951388	-0.246248781	-1.205518603	-0.269442057	-0.20813469	0.534228868	1.231075034
Candidatus_Fonsibacter_ubiquis	-2.245100346	-1.381461054	-1.09527655	NA	2.568528043	2.693960538	0.993593128	-2.028191503	1.402919711	2.66964191	2.420925474	3.291075028
Dehalobacter_restrictus	-2.243814961	-2.16617273	-1.185703	-0.450218729	0.991368311	1.058273708	-8.637284195	-8.637284195	-0.186073083	0.30557457	0.522224525	0.448544836
Alistipes_finegoldii	-2.241704489	0.397592553	0.345763008	0.724468683	2.959848761	2.739402957	-0.731467485	0.208121092	-0.204023349	0.217581085	0.608049323	0.040392288
Geobacter_pickeringii	-2.23916923	0.000224876	-1.219396947	-1.852872425	0.27120786	-0.993928681	-12.88451779	-3.257149892	0.096433965	0.401397629	-0.100115607	-0.494476803
Shewanella_khirikhana	-2.238091863	-2.549605295	-0.139841812	NA	-4.108157592	0.457696086	-0.021398888	-0.142163956	0.473337569	-0.668585315	0.996714191	-0.024205819
Bacillus_cereus	-2.238071474	-1.22304154	-0.983726721	-2.224459311	-0.826864253	-1.159058496	0	8.627369725	0	0.705229599	0.232290882	-0.184473952
Corynebacterium_provencense	-2.237605321	-0.07331433	-0.906828689	1.905999768	3.218020506	2.291008642	8.502740328	0	7.157114468	0.690408779	-4.772268499	0.862022567
Agromyces_sp._MF30-A	-2.232875396	-1.215825621	-2.21482894	NA	NA	NA	-0.267584544	-0.088828153	0.170270699	-8.723377667	0.411048653	0.234690685
Lactococcus_sp._KACC_19320	-2.232735561	NA	-1.472968771	-3.27362229	-1.107838417	-0.46731023	8.6039638	0	0	-1.20349171	-0.121475912	-0.838874091
Oceanicola_sp._D3	-2.226174584	-1.740349167	-1.52938862	NA	-0.508261326	-1.405117328	9.315767728	0	11.44181336	-1.727659367	0.106361033	0.418637608
Oryzomicrobium_terrae	-2.223323117	-0.636054366	-0.927535389	-2.585143007	1.038381523	1.639047021	-0.49476303	-1.941374438	0.516898726	0.415038259	0.642801799	0.095044221
Scytonema_sp._NIES-4073	-2.221587121	0.732609189	1.405034531	NA	NA	NA	0.132188448	1.104500608	0.894148829	8.868307104	7.894817763	8.853830391
Corynebacterium_pseudopelargi	-2.221587121	2.084645493	2.721660275	NA	NA	NA	-1.815050958	-11.22444189	-1.119961077	-1.420491385	0.677395177	0.120076996
Rhodoferax_sediminis	-2.218044958	-2.468451756	-0.353941979	1.078446959	0.63055823	1.800979088	-8.552496194	-8.552496194	-8.552496194	0.891635517	0.867280337	1.617886842
Bifidobacterium_catenulatum	-2.215636744	-1.521212741	-0.433692283	-2.055860024	-0.035091886	-0.388209411	0.359759078	-0.098383669	0.398084723	-0.333509164	-0.605720698	-1.09878362
Ensifer_adhaerens	-2.210896782	-2.893174028	-0.165112738	-0.544686056	-0.511287474	0.054072089	-1.020526743	-2.499474656	-9.177646265	0.818936786	-0.356525875	1.323402301
Actinoplanes_sp._OR16	-2.210359866	-4.068208429	0.995796674	2.331676071	1.335072362	-0.496349414	-2.995970114	-11.15308964	-1.872741067	1.374850743	0.63699714	0.578900407
Roseiflexus_castenholzii	-2.209869588	-1.95697002	-0.704050972	0.787496097	0.570692744	0.804051795	1.946622184	-8.552496194	3.315272886	0.532775526	0.595773744	-0.436609987
Synechococcus_sp._PCC_6312	-2.209309831	-6.375345687	-1.914181795	2.271616793	-0.178645349	1.197285907	-0.467959101	-1.027957711	-0.248238579	0.12540959	1.440572591	1.12541446
Bacillus_sp._DSL-17	-2.208089373	-0.618862266	-1.592613809	NA	-0.091975359	1.206230265	0	0	7.745136469	-0.133521309	0.848789281	-0.01864524
Flagellimonas_maritima	-2.202510879	-2.013654062	0.050822573	NA	NA	2.428353859	-0.116545322	0.326748395	1.112180045	0.893570216	-0.339572457	-0.411551712
Lactobacillus_harbinensis	-2.201265109	-2.733818871	-0.270197638	-0.471806521	-1.553362167	-1.220884509	0	0	0	0.460831609	0.553589438	0.387268941
Pseudomonas_fuscovaginae	-2.200791657	-0.834997677	NA	NA	NA	NA	-1.302766915	-0.601348228	-2.27776396	0	5.472487771	0
Porphyrobacter_sp._CACIAM_03H1	-2.196489248	NA	-1.900096245	0.830912752	-0.653609848	0.335805402	0.383833798	0.459730604	0.901630507	-0.480602232	1.207482778	1.573302896
Draconibacterium_orientale	-2.194625127	-0.476844811	0.20963239	-5.412303185	0.659200118	0.541981937	-1.057462048	-1.054398595	-0.583815515	0.482449506	0.689182042	0.733605343
Gloeocapsa_sp._PCC_7428	-2.193203609	-2.112103054	0.155097983	NA	1.856140981	2.848957423	-3.549691592	-0.317814421	-11.70681111	0.883931254	-0.08383627	0.532542142
Christensenella_minuta	-2.190313114	-2.830812151	-2.284467031	0.006546013	0.806107801	0.494802323	0	0	0	-0.283710369	-0.57323167	0.60053955
Devosia_sp._A16	-2.186532716	-4.070023656	-0.457698651	0.055059018	NA	0.317961012	0.341408208	0.559434089	0.662639582	-0.517422484	0.79630098	0.073472
Pseudomonas_sp._13159349	-2.185897692	-2.240781599	-2.999169672	-3.73079457	NA	-1.075256575	0.815911341	0.536403071	0.821151274	-3.028063918	-1.225427545	-0.420381905
Verrucosispora_sp._NA02020	-2.1820897	-2.946579005	-2.626018178	-4.682737716	0.483598895	-0.678345947	-0.345729749	0.774678668	0.469179291	1.126249995	0.286143137	1.566763125
Marinobacter_salinus	-2.181566408	-2.274675812	-3.04542972	NA	NA	NA	0	0	0	-1.314758222	1.675524569	-8.648655978
Geobacter_anodireducens	-2.179808585	-3.141344025	-1.989784415	1.130699203	1.402298703	0.869121855	-0.02347889	-0.410365828	-0.193450386	0.150787001	0.875293655	-0.450242364
Streptomyces_sp._NHF165	-2.17929261	-0.849629208	-0.923324045	1.663290861	4.071163098	4.642121436	0	0	0	0	7.811644421	0
Erythrobacter_flavus	-2.179268914	0.535699578	-0.953265239	NA	NA	NA	1.901219745	-7.757316664	1.146839237	8.45395786	0	0
Sulfuricaulis_limicola	-2.177318907	-1.778904863	-0.395503698	-3.213726614	1.546039807	2.107651569	-7.757316664	3.493194944	1.974002367	1.350723925	0.453844948	1.7636988
Cutibacterium_granulosum	-2.176996491	NA	0.00318225	1.841127726	-1.935788097	-1.411914105	-2.064630545	0.022559873	1.308785999	1.118869575	1.244864136	1.387991367
Candidatus_Walczuchella_monophlebidarum	-2.175613309	-2.318738251	-0.969803771	-2.279884966	1.002079561	-0.124916154	-2.477691528	0.668033469	0.379969635	-0.034886393	0.688670333	-0.733738087
Corynebacterium_ulcerans	-2.175072294	-1.459655033	0.396035903	0.926532028	-0.193869068	0.739143952	-0.792691551	-0.407797567	0.220788882	-1.146151412	-1.063130958	-0.333661493
Staphylococcus_kloosii	-2.173457872	-0.665825436	-3.639652122	0.641752403	0.916172456	0.593303166	-0.479114274	-1.371384584	-1.900607659	-0.102288463	0.548197274	1.243863648
Desulfovibrio_gigas	-2.173449194	-2.181484855	-0.856098917	-0.809991404	-0.819569188	-0.47387619	0	0	0	0.308397473	0.345608924	-0.061707213

Paraburkholderia_sp._7MH5	-2.17197866	-0.148378301	-0.476289342	-0.522905784	2.157847644	3.011565356	0.48682835	-9.384097309	1.873396611	3.643678201	3.454766274	3.113401397
Vibrio_metoecus	-2.168397532	2.444909595	0.541523239	NA	NA		-0.136152267	0.112634628	-0.559801098	-7.807354922	2.063626202	4.189118567
Alistipes_dispar	-2.168127321	0.385314193	-0.000126886	-0.055443805	2.947758671	2.81496489	0.009396218	-0.113241339	-0.114528295	0.476959364	0.725197007	0.101215912
Aeromonas_simiae	-2.164171474	0.136935645	0.734080203	0.290693744	-0.430263974	-0.619741772	-9.59551112	-9.59551112	0.224086817	-0.626816426	0.685604388	0.31885431
Elizabethkingia_meningoseptica	-2.161165354	-2.025493599	-0.674532686	-3.372139541	NA	-0.986996151	9.790275179	9.791755477	10.57244444	1.028203964	1.316224816	2.30244021
Staphylococcus_sp._17KM0847	-2.160814774	0.345035998	-0.65797904	2.165298009	1.359409907	1.020639766	-0.459670998	-0.112469865	-0.455028792	0.558347004	0.60990423	1.107397411
Mucilaginibacter_paludis	-2.15958812	-1.753237305	-1.175897566	-1.838536577	1.887311484	2.300733873	9.029913869	0	6.15711649	-0.627906134	-0.253218575	0.041224709
Flavobacterium_crassostreae	-2.15911448	-1.523940533	0.249251261	-1.431593198	-0.731977119	NA	-0.13012922	-0.159150463	-0.226406637	0.168362408	0.236783514	2.081659561
Croceibacter_atlanticus	-2.158445505	-2.967819594	-4.807354922	NA	2.967900845	3.665522228	-2.138652161	1.054783945	0.472584031	2.076223459	0.753572379	2.415574036
Deinococcus_maricopensis	-2.158358588	0.11564794	-0.775389753	1.163199138	1.330374958	1.608259121	8.129283017	8.627369725	10.77912016	1.601328691	0.760172279	0.759221469
Paludibaculum_fermentans	-2.157599933	-0.8682768	-0.637332277	-1.382214971	2.68428328	2.984536817	9.315767728	0	6.15711649	-0.195460094	0.209073279	0.247055104
Bacillus_methanolicus	-2.151195765	-0.962338948	0.688369235	-3.201715533	-1.644570977	0.359572412	0.264554613	-9.488205685	-1.036994573	0.805248137	0.994457561	1.158407412
Planctopirus_ephydatiae	-2.146646108	-0.639760853	-1.753464192	NA	NA		1.684602339	2.299851588	3.180988875	7.129283017	0	9.306820443
Moorella_thermoacetica	-2.142064915	-0.53511551	1.037721887	-0.313355134	0.347598909	1.033908218	0	0	9.30543935	0.262874166	0.05569861	0.027847377
endosymbiont_of_unidentified_scaly_snail_isolate_Monju	-2.141852633	-1.987987482	-0.312609686	-0.871911238	-0.810276448	-0.028544575	-1.541427151	1.667278162	0.186318309	1.176342378	1.709699791	0.957129714
Tsukamurella_paurometabola	-2.140351886	NA	0.195807287	1.430899217	-0.663491486	0.980924865	-0.066702976	0.036271479	0.459734076	-0.030257821	-0.63340356	0.810206383
Olleya_sp._Bg11-27	-2.13863372	0.866580398	-0.68373176	NA	NA	-2.683334241	0.903566406	3.997056022	3.998787606	-0.086787373	-2.176052639	0.644833957
Desulfovibrio_carbinolicus	-2.138076636	-1.500156772	-0.070211709	1.227357877	-2.94287536	-0.071941642	0	0	0	0.594196978	0.590823621	0.486568316
Bradyrhizobium_canariense	-2.137503524	-2.946877613	-0.49453542	3.646578023	NA		0.051467605	-8.552496194	1.369344743	0	6.815921204	6.544320001
Erysipelatoclostridium_ramosum	-2.134544799	1.624455945	-0.619163544	2.868642655	0.944596914	0.405902962	-0.440274079	0.24778301	0.719832078	0.31402245	0.106915204	0.474618141
Candidatus_Hoaglandella_endobia	-2.130879802	NA	-0.784555053	-1.386581053	0.882545626	-1.645114067	-2.950457436	-3.249422484	-0.911453262	0	9.45532722	7.544323092
Denitrobacterium_detoxificans	-2.129317133	-1.575658444	-1.037071834	-2.03850916	0.426274797	0.794404196	-8.637284195	1.392624155	0.595899253	0.277558654	0.193711201	0.38112634
Hymenobacter_sp._TS19	-2.129238358	0.019769756	-0.048558524	-0.584161048	2.430903278	2.523306327	0	0	0	0.460324695	-0.04640511	0.593851453
Paracoccus_contaminans	-2.127917475	-0.352301744	0.545069774	NA	NA	NA	-0.195786394	0.677047041	1.420108299	1.118586394	-7.741466986	1.72885227
Bradyrhizobium_cosmicum	-2.127111918	-3.336220182	-1.479560538	-5.982112455	-2.487915418	-2.812187453	0.648678963	2.004929995	3.170899717	-0.412970547	0.024833173	-0.522711443
Belliella_baltica	-2.126917422	NA	-0.407818249	-0.360108842	-0.51120143	0.122974045	0.623319865	-0.349954375	0.008417306	1.668874106	-3.430390766	1.76482735
Desulfotomaculum_ferrireducens	-2.126214234	-1.566997665	0.162223892	1.337038795	1.270062413	1.411628953	-0.380398317	0.309073713	-0.005468028	0.853199569	1.046205296	0.687909031
Limnoglobus_roseus	-2.122869036	-2.638991366	0.660197517	-3.456800605	-0.893560077	-0.287890514	0	0	11.45167991	0.100827061	0.668281729	1.286539142
Brevibacillus_sp._SCSIO_07484	-2.121304163	-0.064847791	0.336371254	1.073912735	2.865282838	3.084859616	0.46282356	1.025424322	0.35876791	1.42902376	2.502570935	1.81059977
Actinoplanes_sp._N902-109	-2.120929487	-4.208766123	-0.149522924	3.638006967	2.015344078	1.497479255	-1.30954258	-1.394575751	-1.600325611	-2.93558421	-1.22247791	-1.936546809
Alistipes_communis	-2.119049766	0.436782624	0.344732224	0.61246529	2.413066323	2.08960383	-0.4345244	0.280441332	-1.905679443	-0.010944117	0.292653388	-0.057311931
Pseudodesulfovibrio_sp._zrk46	-2.118469668	-1.319450822	-0.310133343	0.942492416	0.337815896	0.01352114	-0.114270198	0.549553985	1.613367909	1.104454751	1.079128614	0.699514422
Luteimonas_chenhongjianii	-2.115310826	0.348832279	0.310299862	-0.038228053	NA	0.591452368	0.309563884	-0.028669568	-0.037035334	0.500927389	0.793478479	1.55266795
Halomicronema_hongdechloris	-2.115247909	-2.361685804	0.207276218	6.285796748	4.396159489	4.411426246	9.029913869	0	0	0.248845217	-0.518494026	-0.647339039
Nitratireductor_sp._OM-1	-2.113985972	0.383336696	-0.350895671	-1.225447221	2.941953435	2.654685577	8.876516947	9.426646347	0	1.405790982	1.094340725	0.433753719
Lactobacillus_saerimneri	-2.113430115	-1.202556006	-0.833322196	2.494465056	NA	3.301976938	-0.240039428	-0.919536743	-0.669210712	-1.089287488	0.84615499	0.988768458
Alcanivorax_xenomutans	-2.112894056	-0.453465063	NA	3.579823777	1.160059648	2.907147052	0.561592653	0.542831055	0.539367204	1.057864148	-0.448892094	2.075251448
Paracoccus_sp._AK26	-2.11273445	-1.212906373	-1.468830765	0.119581616	0.381249186	1.457162975	-0.112584558	0.269984827	0.509740939	-7.741466986	-7.741466986	1.459840957
Microbacterium_oleivorans	-2.111682789	3.337113433	0.289321569	0.67818353	2.897602987	2.324545283	0	0	6.15711649	0.541199552	0.225545867	-0.30493897
Neorhizobium_galegae	-2.111124724	-6.088303763	0.237591051	1.742584667	3.391647025	1.650316239	-8.04736934	2.573354515	1.881889068	-8.750428971	1.19754833	0.011122262
Niabella_soli	-2.107692461	0.229785673	-0.039094774	-2.400388465	0.829697675	1.227843597	9.690633595	10.6064885	11.08105729	0.154144044	0.217722873	0.385664462
Paraprevotella_xylaniphila	-2.106085826	-0.152977347	-0.353729536	-2.400754515	-0.332546439	-0.390930018	10.21895065	9.384097309	9.742080338	-0.087903639	-0.197455097	-0.115256805
Halomonas_sp._SH5A2	-2.104571263	-3.094517599	-0.644589453	0.600997851	0.612210563	0.106915204	-2.049675081	-10.2067946	-2.461658135	0	8.033423002	8.89683794
Paraoceanicella_profunda	-2.101429354	-1.608071576	0.024500339	-3.042121263	0.7704103	1.930466965	0.649738754	0.783327148	1.570716287	-0.273828512	0.24528934	0.389615563
Candidatus_Cardinium_hertigii	-2.101152468	-2.150518545	-2.123382416	1.179164587	-0.205284592	-0.23501442	-0.106865479	0.068727123	-0.288181993	0.212033997	0.272920543	0.435581452
Acetoanaerobium_sticklandii	-2.100686939	-2.86439639	-3.119339613	-2.336803094	0.626253663	0.503179956	-0.69911663	0.240383562	0.595505615	-0.207534673	0.220884072	1.121294138
Streptomyces_sp._CRXT-Y-14	-2.098459136	-3.907833226	-2.567634029	NA	-1.179245629	1.429563613	-0.987408134	0.048530443	1.674307097	0.987632774	0.572070255	0.424519061
Cutibacterium_avidum	-2.091077559	-0.283856856	-0.369291407	3.71884728	3.061895224	3.390605284	0.542459913	0.578020819	-1.099741315	0.08840663	0.762726194	0.531729535
Thioalkalivibrio_sulfidiphilus	-2.090673826	-2.362478442	-0.724969979	0.584559344	1.025055536	0.337276827	-10.74895593	-1.10019032	1.422733786	0.372515483	-0.435025943	-0.556486706
Enterococcus_mundtii	-2.090571309	-1.476563657	-0.161370475	3.582277368	2.096781626	1.784295515	-0.844183361	-1.934862888	-1.298352309	0.31007807	0.462925275	0.118963183
Listeria_weihenstephanensis	-2.089045171	-1.392191915	-0.482036945	-1.24376021	0.42923042	-0.457689692	9.932617823	0	9.466123378	0.312865842	0.476433027	0.255971923
Sphingomonas_sp._NBWT7	-2.084886226	0.271050091	0.118844021	-2.394435141	1.16818007	1.54979702	-0.22708985	-0.684040293	0.20931147	1.621886175	1.802187266	1.022786696
Marinobacter_sp._LV10R510-11A	-2.084225952	0.066638968	-0.343364848	-0.763325264	3.060212033	3.341348246	1.534222807	0.003323679	-2.466929557	0.800490115	0.330023182	-0.169146584
Desulfovibrio_sulfodismutans	-2.083809625	-0.610232562	-0.487501828	-0.495944879	0.816061765	0.93148524	0	0	0	0.706516347	1.055719123	0.273615217
Pararhodospirillum_photometricum	-2.081842022	-1.601351461	0.259835524	NA	NA	NA	-0.359008675	-2.825218777	1.097312256	-0.663731015	-10.02421162	-0.131415348
Anoxybacillus_caldiproteolyticus	-2.079770564	-1.974277435	-1.681944641	0.311870957	0.122081338	0.113464188	0.972557974	2.102173219	-0.036701492	0.556397567	1.714854506	1.550407789
Mycobacterium_avium	-2.076386047	-1.128474558	-1.321119636	NA	NA	NA	-11.85188956	-0.430492665	-1.181233308	8.45395786	0	0
Variovorax_sp._WDL1	-2.076167049	-0.763671556	-0.746479034	-0.632227782	0.391084613	-0.732121622	0.127932413	0.328027974	0.197090096	-2.263459565	-2.141897019	-0.872546452
Streptomonospora_sp._M2	-2.073153112	-2.133459527	0.850423644	NA	1.523748074	NA	0.294795536	-1.761460705	-0.282005535	8.45395786	9.150762744	9.306820443
Rhodococcus_ruber	-2.071349844	0.631726067	-1.675558381	2.166269663	1.851753352	3.063783983	0.662017627	-0.118166719	-0.323513792	1.037158909	0.62943713	0.443968022
Pseudodesulfovibrio_aespoeensis	-2.070005785	-1.460555437	-1.49458536	0.74378686	1.765949076	1.495058744	0	0	7.157114468	0.786251714	0.678951998	0.088866936
Defluviitoga_tunisiensis	-2.068831135	-3.265536913	-3.212084796	0.09145018	0.065978085	-0.629874923	1.874611071	1.025187225	1.681725804	-0.007892497	0.106622496	0.130106603
Pseudonocardia_sp._HH130630-07	-2.061889942	0.271159484	-0.2543922	0.827801371	2.892192658	3.214222051	0	0	8.451211112	0.799268043	0.002640024	0.21562986
Microbulbifer_hydrolyticus	-2.061596209	-2.154705614	1.157168816	4.28661917	1.299598327	2.045832224	0.48706325	2.186877252	2.32878863	2.449370203	3.849400824	3.037420895
Planctomyces_sp._SH-PL62	-2.061122688	-5.190405705	0.106015712	-0.551238364	-0.597277285	1.372154559	0	0	0	0.530907898	0.813327191	1.395030415
Thauera_sp._MZ1T	-2.058173162	-0.152650985	-0.400039607	-1.977317199	2.660891814	2.442360687	-0.506658356	-0.057666432	0.103348851	0.65990695	0.519393569	0.10004907
Microbacterium_sp._CBA3102	-2.057774267	-2.394767188	0.565311779	1.245531571	0.438176649	0.406850315	-2.631377	-3.092431751	-1.84527801	-0.791327883	0.815695413	1.032964763
Rahnella_aquatilis	-2.057464437	-1.578114967	-1.139252913	NA	-1.306582122	0.366244674	0.924241029	0.838971879	-8.10567284	-1.901706505	-2.25702915	-2.311697609
Kosmotoga_olearia	-2.056198142	-0.459527981	1.940993887	-1.95419631	-1.828960127	-0.774872611	0.563266951	1.624296385	0.488499353	0.009726449	0.923649359	1.333811938
Azotobacter_vinelandii	-2.054942451	-3.206488923	-2.360522551	2.641683476	1.380623425	1.937171174	-10.55772216	-0.502927934	-4.400605667	-0.747942203	-0.19874696	0.834036831
Bacterioplanes_sanyensis	-2.053925882	-0.162142178	0.5282161	1.77284936	-1.230584515	0.710404588	1.119200282	1.891448949	-1.600200174	-0.301609261	0.900495759	1.597070401
Nissabacter_sp._SGAir0207	-2.053647577	-1.409422661	-0.251834891	1.762855462	0.89016482	1.114958542	-8.799135887	2.314248165	-0.318252808	-0.9460754	1.124105375	-0.204341449
Bacteroides_helcogenes	-2.052772177	-0.444688024	-0.850717375	-2.296317451	-3.062746608	-2.794866848	0.541212359	1.620408229	0.74518462	-0.140630049	-0.204198043	-0.263687811
Shewanella_sp._Scap07	-2.048392655	-3.441131487	-2.255362207	2.193592972	0.105912467	0.355165642	-1.51913942	-0.229471053	0.646451149	-0.063064073	0.643027077	0.523494062
Rothia_mucilaginosa	-2.048363022	-2.173160361	-1.375344344	0.16957623	1.283764943	2.120257617	-9.177646265	-1.067220377	-0.073165448	1.318871536	1.054223715	0.826732751
Escherichia_coli	-2.042915478	3.929271788	1.304376576	-0.355981262	1.222943849	1.054775119	-0.294402421	0.023355434	0.208430183	0.116562627	0.142679525	0.662852784
Changchengzhania_lutea	-2.039110161	-1.062505729	-0.916806064	0.468302649	1.991463318	2.001063542	0.361553383	0.482722586	-0.499043561	-2.989065838	0.847861398	0.287955327
Thiomicrorhabdus_sp._G1	-2.037406515	-0.54138133	0.196244084	1.235245152	0.489184977	0.498681159	10.31781959	9.426646347	9.561202011	-0.400102935	-0.088298334	0.12283901
Antarctobacter_heliothermus	-2.036931502	-0.918435352	-0.881005026	2.425349328	0.457339442	0.293542909	-0.198195544	-0.173251586	-0.192873334	0.331924589	0.862842176	-0.587774234
Lactobacillus_terrae	-2.035877036	-0.774357044	-2.753938673	-2.037105557	-3.192311082	0.076278568	0.261815964	0.299314151	-0.28583913	-1.291534569	0.527095165	1.494325614
Anoxybacter_fermentans	-2.034154154	-1.469831119	-1.29550437	-0.537565232	-1.345854596	-1.400218495	0.103164591	-0.710412208	-0.396951443	0.845804192	1.226656596	0.120025864
Sphingosinicella_microcystinivorans	-2.034100644	-0.269638963	-0.352569929	-0.974062491	3.128959869	3.409888823	0	0	0	1.584271398	0.836547688	1.047008996
Flavobacterium_pallidum	-2.030714809	-0.310398509	0.05907554	0.641374162	1.410947422	1.544751321	-1.300966429	-0.322556821	1.17304736	1.049730148	-0.015385344	1.250028799
Acidothermus_cellulolyticus	-2.02424101	-0.89359908	-0.059172237	0.728500159	1.032011679	0.064112523	-0.449425411	-1.346646868	-0.511716551	-0.02024488	-0.400270254	-0.008670506
Serpentinomonas_raichei	-2.020430009	NA	-3.462178814	-1.172692952	1.476141732	0.545990015	-0.521789674	0.175972001	0.777951924	0.057376203	-0.322788933	0.729023589
Luteitalea_pratensis	-2.019673507	-0.305386479	-0.198068099	0.581194687	2.127669443	3.054042693	0.308065755	-0.200272429	0.271785191	0.373302613	-0.249346873	-0.07478611
Bifidobacterium_longum	-2.015737272	-2.023157899	-1.210620723	-0.868901774	0.518633359	0.229499519	0.21861334	-1.378508855	0.250269191	0.399172291	0.70733467	0.448417616
Propionibacterium_sp._oral_taxon_193	-2.01272156	-3.329070164	-0.471404673	3.110621567	1.567878346	1.379748749	0	8.906890596	0	0.324054844	0.157766148	-1.951663842
Streptomyces_sp._SUK_48	-2.01155669	NA	-0.947425352	NA	NA	1.727289005	0	0	0	-0.508877845	-0.491355018	0.96517087
Neisseria_meningitidis	-2.009051445	NA	-5.657960863	-3.073695291	-0.982700759	-0.894615575	0	9.3781973	0	-1.170257794	-0.951346008	0.605107834
Haemophilus_parainfluenzae	-2.008620483	-2.366662784	-1.664434035	4.412796524	-1.518948244	-0.843167111	1.011775991	0.726578882	0.763616624	3.492499007	1.718265302	-0.505235308
Chryseobacterium_camelliae	-2.008138179	-0.264185834	-0.932121377	-1.906491756	-0.030787263	0.478634827	0	0	0	0.131451083	0.38358758	0.867426152
Peribacillus_asahii	-2.007060942	-1.199343852	-0.091911423	-2.248197874	-0.688471351	-0.954758415	4.365540028	2.681633913	2.243663684	0.334112093	0.608516903	0.748311642
Arachidicoccus_sp._B3-10	-2.00341351	0.227324242	1.194287996	NA	0.43993722	0.208847439	-0.64186749	0.440354573	0.125962195	-0.024129656	0.384113938	0.858208699
Jeotgalibaca_arthritidis	-2	-2.293279208	-1.323023357	1.312156382	3.06789597	2.743530291	0.345212653	0.890414504	0.197527702	-1.254261389	0.09508546	1.202682267
Collimonas_fungivorans	-1.999495473	NA	-0.028693293	3.577054132	0.310340121	2.972325042	-9.084082873	-9.084082873	-9.084082873	1.535981245	0.63360821	2.180657369
Pseudomonas_coronafaciens	-1.998709208	-0.131080559	-0.863268659	0.866766846	2.990050041	3.405998867	-0.791482286	-0.511304297	0.867849773	0.560787236	1.12644085	0.986217297
Corynebacterium_sp._ATCC_6931	-1.998601362	NA	-2.706047869	2.845759236	1.73851272	0.922083483	-0.825563907	-0.041228521	0.187699156	-0.863138964	-2.858858971	1.465199574
Teredinibacter_sp._Bs08	-1.991906318	-4.386242908	0.970532688	0.716102313	-0.57088147	1.371872981	-3.825873059	-2.874500911	-3.264788818	2.093933772	2.320009018	1.849528257
Leptolyngbya_sp._PCC_7376	-1.991037668	0.807152121	-1.142622831	-1.568491491	-1.895453224	-1.632418818	0.159361856	1.72613124	1.684251292	0.798905051	0.68830445	0.216455623
Bacillus_sp._Y1	-1.989107769	-2.050867819	-0.023224277	-3.512372836	-1.269279137	0.235219364	0.892113944	-0.295293254	0.291132089	0.816981001	-0.114353872	0.3429046
Pseudomonas_tolaasii	-1.98583527	-1.876639412	-0.607515434	0.835398878	3.631381917	1.409705962	0.334224706	0.626575736	0.580122232	-1.755765588	-1.298851449	-0.9792962
Acholeplasma_axanthum	-1.983689952	-2.156860993	-3.160528601	-4.8573748	0.186508816	-0.170547248	0.052656143	0.178829398	0.405365655	1.056843435	0.662965013	1.081368876
Flavobacterium_haoranii	-1.983182382	NA	-3.501856065	NA	-0.720815201	-0.293955057	-1.290541926	3.064779919	2.094801235	0.247686442	0.443382686	0.266337751
Corynebacterium_riegelii	-1.98112199	-0.221199196	-0.422364559	0.362185617	0.291392943	1.433087539	-0.349220821	0.406995587	0.574876072	0.187702747	-0.246143883	0.940950021
Curtobacterium_sp._MR_MD2014	-1.979724892	-3.184583683	2.206343921	6.113839696	7.109894222	6.487644356	-0.378655168	-1.208105963	0.993525519	0.714356336	1.973964557	-2.362887196
Desulfobulbus_oralis	-1.979243278	-1.970897398	0.030992678	-2.076137749	-0.917792839	-0.012256042	-9.773889213	0.980388688	-0.46224306	1.10324312	1.370359935	0.766916275
Inhella_inkyongensis	-1.976136524	-1.519728083	-2.085149647	NA	1.277270936	2.868886152	0.347964578	0.651506024	0.867555432	-0.465008845	-0.054536893	1.054158444
Aquisphaera_giovannonii	-1.973855303	0.020678855	-0.488096302	1.46661892	1.547794487	3.4855887	0	0	11.37276714	0.819705968	0.743003123	0.266762745
Chlorobium_phaeovibrioides	-1.972777006	-1.829150177	-2.610824142	-0.185431937	1.130552426	0.574784302	-8.637284195	-0.077819149	0.205352145	-1.538209788	-0.360658979	1.525306073
Acidovorax_avenae	-1.972247797	-4.403722186	-2.321928095	-3.090490534	-2.077666494	1.042908591	-0.759082769	-2.221381714	-0.172634884	0.42530617	0.507942164	0.501466538

Synechococcus_sp._A15-28	-1.970922767	-2.033207045	0.083085945	NA	-0.282066506	0.67652974	1.021252215	1.580564479	0.519832801	2.527016287	2.704453145	2.066059129
Nocardioides_marinisabuli	-1.969816995	-2.213271031	0.178372707	0.618784168	1.484268501	3.592597014	8.876516947	12.53470168	7.157114468	-0.915630256	0.658959168	-3.718561768
Flavobacterium_nitrogenifigens	-1.96889634	-2.950849884	-0.622162561	NA	NA	-1.087160801	0.130486666	-0.426970842	-0.304605467	-0.779102282	-0.890877509	-0.75702905
Chryseobacterium_nakagawai	-1.968153891	-2.071842225	-5.325088436	NA	-1.278301162	1.714647215	-9.72047685	-9.72047685	-3.56336036	1.109404238	-2.119750513	1.219454598
Desulfuromonas_sp._DDH964	-1.965412034	-1.124858307	-0.537189597	-0.124378109	0.407232097	1.043792138	-0.17349618	0.111537814	0.077027542	-0.533670566	-0.353807428	-0.377390491
Paenibacillus_naphthalenovorans	-1.964722765	-0.520598485	0.747404494	-1.196695168	-0.487592833	0.002992243	-0.399315043	0.143756206	-0.427187728	-0.251448619	-0.489260545	0.332952685
Bacillus_sp._X1(2014)	-1.962979612	-1.537532054	-1.063893277	2.453881479	1.71229447	1.999671181	-0.532327544	-0.746246769	-0.224432254	2.109896972	1.193993866	1.077248384
Alcaligenes_faecalis	-1.960747761	0.187196567	0.75492719	-3.653873657	2.632721364	3.05290641	-0.386089934	-0.454969777	-0.329686516	0.282718415	0.686971622	0.798967958
Rhodanobacter_denitrificans	-1.960184661	-0.29401831	-0.761259048	0.071684968	3.49547796	4.01207106	0	0	0	0.95605978	0.163845367	0.172484286
Pseudomonas_alcaliphila	-1.958457738	-1.88836844	0.199094401	2.041396622	2.127588055	-0.212544635	0.568245445	0.888317488	2.148949354	0.167192075	0.786315699	0.49372609
Massilia_sp._LPB0304	-1.957758415	-4.348728154	-2.187472748	2.587271357	0.2248855	1.355621259	-0.839458523	-0.970582362	0.47319519	-0.719706068	0.802671471	0.665395793
Methylacidiphilum_infernorum	-1.95455928	-4.609854842	-2.024301678	-2.831235116	0.837319927	-2.527128944	-1.222823394	-0.007770142	1.198606941	0.32483132	-0.43104055	0.093433715
Myroides_profundi	-1.9510904	0.154843045	0.422126924	NA	NA	NA	0	0	10.86741775	8.35167691	0	0
Desulfarculus_baarsii	-1.950439099	-2.977890785	-0.817233663	0.799030269	0.062187332	0.216182146	-0.211450609	-0.175041554	-0.07803282	-0.089762334	-0.033445784	0.5369995
Cystobacter_fuscus	-1.948317083	-2.541943463	-0.698161045	-0.103948485	-0.055962499	-0.273728621	-0.354757834	-0.681338841	0.006927799	0.256154148	-0.232568114	-0.524763832
Streptomyces_paludis	-1.944931348	-2.097246342	-3.606259989	-0.606861982	-0.710970386	NA	-0.668552605	-1.030905537	1.200386326	-0.009475964	-10.47843595	-1.543763182
Pseudopuniceibacterium_sp._HQ09	-1.944524673	-1.020091041	-0.110905483	0.088148913	3.132833995	2.039672868	9.147899389	9.426646347	6.15711649	1.204189566	0.712197689	0.086457359
Aeromonas_media	-1.943583141	-6.044442266	0.157433359	0.658476787	0.700413624	-0.648060134	-1.122872409	-0.727995503	0.644969525	0.010782524	-1.938900815	-0.293442504
Sphingomonas_sp._C33	-1.941504351	-0.507483748	0.841070849	NA	NA	NA	-0.034969574	1.202777293	1.578277197	-0.891127936	-0.734682848	-2.967250802
Polaribacter_sp._KT25b	-1.939213424	-4.748587514	-3.951981617	NA	-5.119331212	-4.197333724	-0.588591101	-0.279589741	-0.22282202	0.730608867	-0.025477682	-0.037147112
Crocosphaera_subtropica	-1.93633849	NA	-3.742165942	NA	-1.28408777	-1.792006958	8.157119522	11.55729137	6.15711649	1.187488462	-0.536099547	1.557247459
Candidatus_Thiodictyon_syntrophicum	-1.933881587	-2.120309998	-1.373603434	-0.891745764	-2.011094692	-0.998549438	0	0	8.48088308	-0.492391135	-0.697785726	-0.269183656
Azoarcus_pumilus	-1.932752659	NA	-3.166560199	-0.794018368	NA	-3.425723507	-2.105189304	-1.816726492	-0.592455707	2.49262004	-2.334867151	2.826848072
Acetohalobium_arabaticum	-1.929769708	-2.708309136	-3.612269847	-0.340885364	1.196579451	0.770165338	-0.573618103	0.119730791	0.880271118	-0.643789936	-0.105938017	0.809047405
Brochothrix_thermosphacta	-1.928065692	-1.119900949	-0.716708875	NA	4.918259007	4.999639281	-0.012838773	-0.303778999	0.687013506	1.084794325	1.989866853	-0.056406388
Stenotrophomonas_sp._LM091	-1.925999419	-2.124545098	NA	NA	NA	NA	-0.970860808	0.449498378	-0.30934869	10.44432127	9.033423002	8.658211483
Rhodococcus_sp._W8901	-1.925999419	NA	-1.807354922	NA	NA	NA	0	0	0	0	7.902875524	0
Microbacterium_wangchenii	-1.925232232	-0.892098488	0.304013861	-0.407319586	1.473533149	3.015668177	0	9.252665432	9.014782079	-0.931740294	-1.039367049	1.246188656
Candidatus_Tachikawaea_gelatinosa	-1.922809966	0.651350837	4.582432507	1.540355779	2.402432075	3.281689182	-1.069019756	-0.23798951	0.786554589	0.766271142	1.494982527	0.998733182
Saccharopolyspora_sp._ASAGF58	-1.922277705	-2.731651794	-4.689831619	0.195664322	NA	NA	9.877911389	0	0	-0.288162861	0.489128895	-0.597996374
Mycobacterium_sp._DL90	-1.921673255	-1.464708017	-1.27048671	-1.798452422	1.86596509	-1.82281697	1.328916238	0.625012721	0.697625444	0.319750183	-0.926379195	-0.962454969
Biomaibacter_acetigenes	-1.921352413	-2.975393693	-1.25058986	-0.754138757	0.883033677	0.821093222	-0.354183439	-9.384097309	0.521426588	-0.369234661	-0.140513638	0.336304957
Pleurocapsa_minor	-1.920952504	-5.150198597	-0.322944117	-1.066207203	-4.191542053	-2.44130841	2.225124221	0.145508852	0.166648397	1.583335543	1.640718491	1.230162736
Synechococcus_sp._CB0101	-1.919924904	-1.291964998	-0.293534851	-1.724207884	-2.424497829	1.931771464	-9.624047875	-9.624047875	-9.624047875	0.072038539	-1.088557224	-0.586759178
Bacillus_subtilis	-1.916343723	-2.067920551	0.10656888	-1.528383971	-0.607028796	-0.122073345	1.200551035	-0.61818122	1.805739293	0.371565717	0.10537077	0.056451728
Halomonas_sp._YLGW01	-1.915365255	-1.770265112	-0.107291561	0.543533533	-0.675759063	0.22187922	2.27252446	2.984439579	1.957722241	2.417601002	2.220986028	2.282470108
Synechococcus_sp._MVIR-18-1	-1.909405254	-3.302144086	0.116981153	NA	-4.067272787	-0.910136847	9.147899389	11.38508046	9.540215237	1.57334007	2.148673944	0.594963538
Sulfitobacter_pseudonitzschiae	-1.908369525	-2.717743614	-0.270773869	NA	1.067694407	0.649753516	0.056814114	0.286740347	0.963708171	0.409890976	0.612088001	0.82750805
Bacillus_circulans	-1.903517304	-2.856063384	-0.937512496	-1.179766789	1.197889776	1.533858861	-0.27285004	-0.161030389	0.816833998	0.261559104	1.583886962	1.529792238
Chryseobacterium_sp._StRB126	-1.90008771	NA	-3.274968083	-6.412836591	-4.879617665	-0.692905239	-2.755884572	-2.732478646	-0.08425344	-0.084016201	0.803633799	1.048916669
Corynebacterium_variabile	-1.896714155	-0.831119112	-1.355057294	-0.714148319	1.987076229	1.611486124	0	0	0	-2.213421926	-1.991962476	-1.878066316
Shewanella_benthica	-1.895373779	-4.158937712	NA	NA	1.024423474	NA	1.7895467	3.287190608	2.634195916	0	9.853828832	9.399455246
Mycolicibacterium_goodii	-1.894883294	NA	-3.593374741	NA	NA	NA	0	11.26390882	9.695386649	0.134101112	-0.316458267	1.040050947
Deinococcus_ficus	-1.892660349	-2.364906772	-2.702546114	0.058909409	1.049636463	0.674481336	-0.495706733	0.451363776	0.672570603	1.468879479	-0.222251816	1.389699956
Tessaracoccus_defluvii	-1.891180058	-2.779975833	0.743195161	-0.53652964	1.737918602	1.424933923	0.441544347	-0.901608874	0.308630061	-0.658901317	2.013597443	1.076959808
Janthinobacterium_sp._LM6	-1.890683306	NA	NA	NA	NA	NA	0.345770282	0.455974251	0.448857672	-9.247927513	-2.43200631	0.164288761
Spirosoma_rigui	-1.889246019	-0.569602157	-0.336173228	0.017094935	0.652481266	0.906067621	-2.575931594	-2.418922135	-2.50443945	-0.261408785	-0.242178595	-0.184463815
Rhodobacter_blasticus	-1.887084392	-0.557525633	-0.647217304	-2.129575327	1.106455833	-0.529346069	0.210857577	0.198560121	0.787150907	0.893333342	2.446314487	0.793297332
Paracoccus_zhejiangensis	-1.88400088	-2.00816622	0.122870597	NA	NA	NA	-0.360739752	0.234521665	0.345437541	0.108981579	-0.516831217	0.75450067
Alistipes_shahii	-1.880767206	0.592778738	0.498604597	-0.8819181	2.313662374	2.103239854	-1.741274133	0.97644722	1.083223117	-0.225067292	0.471121646	-0.025751138
Nocardia_wallacei	-1.874927626	NA	-1.177087462	-2.21201794	0.524495327	2.20612547	0	0	6.15711649	-0.307780926	-0.176030364	-0.185790483
Streptomyces_tirandamycinicus	-1.871347394	-2.461363122	-0.576850886	NA	-0.587608608	-0.640790889	0	0	0	0.496896514	-0.152829558	1.066078318
Anaeromyxobacter_sp._K	-1.870519923	-2.681663106	-0.187098287	NA	NA	NA	-0.283303642	0.809305332	-0.018129093	0.910007533	1.099678021	-1.889441625
Mixta_calida	-1.869939459	-0.621351399	0.530700611	-4.627341103	1.552001637	2.290164395	-0.965726104	0.812302534	-9.995639973	-0.38088678	-0.048323142	0.807243568
Neisseria_brasiliensis	-1.868029582	NA	-5.82709314	NA	NA	-1.896569368	0.8668169	1.190002334	1.483406406	-0.412186298	0.256362034	0.547350241
Rouxiella_badensis	-1.867362231	NA	-3.679012635	NA	NA	NA	-10.9306043	-1.503957949	-1.369402285	8.45395786	6.815921204	8.89683794
Geobacter_daltonii	-1.865184233	-1.469770036	0.257693018	-0.286907902	-0.289357993	0.712786064	0	0	0	0.369414423	0.850989537	0.342834826
Salinicola_tamaricis	-1.856379818	-5.833558857	-3.617080061	3.443955563	-2.107668294	0.052851882	-0.407066932	0.516710181	0.542758819	1.140274294	0.938165768	-0.110045421
Pseudarthrobacter_sp._BIM_B-2242	-1.855220247	-2.966654504	-0.700849168	-0.315753298	-1.332575339	0.617593505	0.048795704	-1.052279267	0.002565005	-2.806467844	-0.128877858	-0.445881021
Erysipelothrix_sp._HDW6A	-1.852532451	-2.28109938	-1.144047445	2.634696989	0.367507939	0.324303687	9.602277592	11.62902414	8.742080338	0.175207098	0.738271421	0.856430425
Alkalihalobacillus_krulwichiae	-1.852435351	-2.695881013	0.274670724	-0.592306443	-1.020657988	-0.17471365	-1.07550377	-0.420283218	-1.968913114	0.389115279	0.32348292	0.336063088
Baekduia_soli	-1.852154532	-1.135947498	-1.3107813	-0.773305304	0.230984179	2.601207831	0.452434581	-0.960145786	1.605688673	0.055978694	1.081169845	1.695844134
Peptoniphilus_harei	-1.849263662	-2.923117835	-3.198148307	1.058246596	0.751769198	2.167766893	0	0	0	1.538144755	0.639988347	2.612936695
Rhodococcus_sp._PBTS_1	-1.847814529	1.780530435	1.780264966	-0.738812627	3.718022237	3.753451126	-0.953205789	0.863284987	1.339976828	0.689836911	1.342572831	-0.513781159
Rhodococcus_sp._X156	-1.847202111	-0.396827678	-0.022876808	2.624565246	2.946546894	3.180345577	0.612628914	1.952808466	-1.432509796	1.50059796	1.298273844	1.071162264
Virgibacillus_sp._MSP4-1	-1.846414136	-2.891401591	-0.581008826	-0.17990909	3.302898867	3.081402321	9.790275179	8.627369725	9.810864	1.521984405	2.42265342	1.001650564
Staphylococcus_warneri	-1.845514316	-0.499785226	-0.821987832	1.993121822	NA	-1.76176568	0.014898415	-0.191732962	-0.659454754	-1.183680396	1.273832241	-0.555830527
Listeria_seeligeri	-1.84006871	-2.362236084	-0.058861214	-1.027831971	-0.529864018	-0.733352187	0.300048064	0.502455379	0.383513789	1.003964935	0.427318379	0.446796172
Romboutsia_ilealis	-1.839659212	7.341316688	-0.67955922	-1.505323564	0.282291295	-0.372179498	0.342919198	-1.119473878	0.745120804	-0.529695692	1.06191392	1.336646267
Pseudomonas_chlororaphis	-1.839495079	-1.899929103	0.114800552	-0.47977959	0.350791498	0.757077346	-0.691438568	0.114324481	0.362237088	0.593715276	0.394537988	1.115539844
Dictyoglomus_turgidum	-1.837308144	-0.516995157	-1.785777844	NA	-0.978148602	-0.434477932	-1.688233566	-0.363798964	0.726960452	-1.735369366	-0.176583488	-0.172299024
Corynebacterium_glyciniphilum	-1.835047752	-1.540414151	-0.594893571	NA	0.978148602	NA	8.6039638	10.05724718	0	0.233663478	-0.641288111	0.011980931
Bacillus_flexus	-1.834505797	-1.19648899	-0.554202392	-2.539779192	-0.443854772	0.202945672	0.405506659	-1.149556281	-1.926529679	-0.239620503	0.827830082	1.078509963
Acidimicrobium_ferrooxidans	-1.834025537	-1.845859955	-1.29463988	-0.570878728	0.081675682	0.511473934	-0.570525002	-0.186149777	0.366564883	-0.934133502	-0.351184533	-0.049817606
[Mannheimia]_succiniciproducens	-1.832445903	0.348684553	-2.832445903	5.919475757	NA	-1.371968777	0.089316559	0.818839207	0.884434031	0	8.979187942	8.380820341
Burkholderia_contaminans	-1.827684451	NA	-1.684978896	NA	-0.813955333	0.549406131	0	10.02990835	10.82104847	-1.028428408	-0.962945262	0.525091045
Thermosulfurimonas_marina	-1.826408039	-1.785242295	-0.328749361	-0.862991838	0.658958758	-0.585676112	-1.263190622	-0.030225557	0.825043137	1.234966728	0.291114056	0.139989663
Megamonas_hypermegale	-1.825727743	-1.446966741	-1.581068585	-0.880457708	0.852239945	1.449706496	0.583751629	-0.721845253	-0.124400075	-0.069078657	0.306330364	0.558412874
Pseudomonas_fulva	-1.823956319	NA	-4.795968782	0.643947352	0.755309503	-0.911598995	0	0	0	0.03722703	0.604855396	-0.802209737
Rubrobacter_radiotolerans	-1.819500915	-1.487467943	-0.784910576	-0.755072769	-0.905274734	1.114108535	-9.222246695	-11.59487697	-9.222246695	0.474188898	0.475504146	-0.436028044
Streptomyces_sp._S8	-1.816339973	NA	-0.960729882	-2.17698918	0.156480537	-1.82637833	0.725587649	0.68214919	0.254629777	-0.465472412	-4.430389259	-0.08909584
Hathewaya_histolytica	-1.813977477	-1.228840963	-0.979419627	0.221512013	0.613654688	1.323091366	-0.09953736	-0.668486638	-1.381088959	0.376964458	0.7077787	0.119144201
Corynebacterium_suranareeae	-1.813969503	-2.623343592	-3.242114517	2.469376927	-0.56010212	1.490552283	0.225544417	-1.418474352	-9.800605508	-0.202218687	0.895107505	1.087946736
Zobellella_denitrificans	-1.812466336	-0.877935602	0.439565193	0.148519764	-0.720803604	-0.505063287	0.234232362	0.077062843	0.123687229	1.013829134	0.426634756	1.183959919
[Brevibacterium]_frigoritolerans	-1.812314254	-6.891716802	-1.764523528	NA	-0.165445022	0.179313781	0.449717584	-0.859160751	1.407715138	0.339373229	-9.682994584	-9.682994584
Agromyces_sp._KACC_19306	-1.810823306	0.129147567	-0.364672716	NA	NA	NA	-0.179029042	0.483409698	0.571720896	0	7.902875524	7.63420844
Jonquetella_anthropi	-1.809984475	-2.107373345	-0.392216719	1.197377006	-1.317920018	-0.682699912	-0.184277034	-1.260058905	-1.330485322	0.829856399	0.608436997	0.507220306
Bosea_sp._RAC05	-1.806238563	0.223866541	-0.376993587	0.53148744	3.191686151	3.89150272	0	0	0	0.947432654	0.475495297	-0.051724377
Azoarcus_sp._M9-3-2	-1.804497533	-4.795732168	-1.56564294	0.949255201	-0.858099721	-0.082071133	9.029193869	8.960529086	9.929258409	0.32481652	-0.717341388	0.238003935
Halobacillus_mangrovi	-1.80415604	-0.127482773	0.72086392	-0.524420959	-0.15795236	1.821461868	0.298977016	1.536306596	1.260288447	-0.438040612	-0.713726447	0.980347991
Parabacteroides_sp._CT06	-1.802914491	-1.107220387	-0.323867137	0.691202991	-1.318767754	-0.58749679	-0.574268903	0.292318401	0.987153836	0.107384241	0.114633122	0.040765113
Polaromonas_vacuolata	-1.799760721	NA	-3.079071571	NA	-0.944007216	-2.326463325	-0.427877118	0.244348255	0.222543885	-1.083181765	-0.814773397	0.112637835
Pontibacter_actiniarum	-1.798617232	-0.330805272	0.582393713	0.6582829	0.753489826	1.185764926	-10.8786104	0.517218238	0.153814204	0.291304289	0.518276247	0.926481893
Mycetocola_sp._JXN-3	-1.797523061	-1.512568037	-1.083288615	2.180572246	1.207476429	2.579180148	-0.129301751	-0.305059597	0.163599214	-0.502174596	-0.225333697	0.240329876
Bifidobacterium_adolescentis	-1.792959235	-2.690135771	-0.294571713	-2.374787079	0.204182332	0.579645885	8.409390936	9.822499133	0	0.250642691	0.436541601	0.769011183
Desulfovibrio_ferrophilus	-1.792903099	-0.721090227	0.246160587	-0.93597372	0.579580994	1.406342522	0	0	0	0.596493932	0.537543569	0.42442352
Lactobacillus_malefermentans	-1.792799086	0.981653211	1.306996509	-1.522448049	2.836768684	2.449265548	0.971267199	0.299944166	0.416396204	1.642972585	1.135388083	-1.538852624
Denitratisoma_oestradiolicum	-1.791376038	-1.319018457	-0.127615319	-0.180177473	0.420800308	-0.003557819	-0.487490262	-0.149393986	-0.912369385	0.574610747	0.361523959	-0.712002276
Desulfovibrio_desulfuricans	-1.791006113	-3.34606855	-0.599497129	-1.482224189	-1.749065816	-0.371822086	0	0	0	0.718216757	1.049887732	0.499499313
Sulfurimonas_sp._NW10	-1.790074454	-2.94050259	-3.34071205	-4.059222337	-3.045204137	-2.898850343	1.249478559	-9.062167594	0.832374333	-0.139831984	0.431236235	-0.124449703
Skermanella_pratensis	-1.790038526	-0.30259583	-0.288445991	-0.566729145	3.266637596	3.286048996	-0.660991392	-0.35825768	-0.652569437	2.269955519	2.205742733	1.897559736
Auraticoccus_monumenti	-1.788901165	NA	-1.715499692	3.056181106	0.808493143	0.978317006	1.150690409	-0.295364832	2.37516439	-0.336971376	-0.110695521	-0.224988915
Acaryochloris_marina	-1.788445599	-0.978144726	-1.047814848	-2.485415631	-0.750614762	-0.92596583	-0.144142839	-0.455532141	1.049014671	0.326427567	0.82081678	0.202450705
Geobacter_bremensis	-1.785006494	-1.35065062	0.030004462	1.729786528	2.022828298	1.900938031	0	0	0	-0.256946321	-0.108800407	0.054194231
Pontibacter_akesuensis	-1.782524482	0.436672713	1.260811903	-4.549632262	0.129998418	0.702783483	-0.678558964	-0.090408891	-0.786691461	-0.182082915	0.578168589	0.41339368
Candidatus_Endomicrobium_trichonymphae	-1.777619601	-1.170514217	-1.229478944	-2.241667316	0.668735625	1.553674931	1.088442937	1.064365474	-0.819698317	0.340946949	1.209268559	0.405068045
Thermobispora_bispora	-1.777194944	-3.313287452	0.005190593	3.378599372	0.231705753	1.209843847	-0.664088314	-0.051344823	-1.12622726	-0.512261417	-0.089724061	0.367312733
Chryseobacterium_sp.	-1.775694293	-0.697251241	0.385141584	-3.060541542	0.2099874	0.776268842	-8.076815597	0.033610291	-1.919699107	0.486908003	0.263857494	0.492488733
Thermomonospora_curvata	-1.772852565	-4.115148458	-0.537898631	0.317788365	-0.029833467	0.325584819	0.137534942	0.74473319	0.767715661	0.291459481	0.188127439	0.246236319
Streptococcus_gordonii	-1.77226061	-2.54390941	-0.693947664	-1.386147656	-0.114332675	-0.877023083	-9.898669402	-9.898669402	-9.898669402	2.028210753	0.592649861	-0.310359473
Candidatus_Sulcia_muelleri	-1.771821009	0.140519262	0.68589141	-1.238808384	-0.120890966	0.473868138	0.393371392	-0.052306636	0.277709512	-0.04961395	0.076148614	0.309351499

Streptomyces_niveus	-1.771710423	-2.241483986	0.542993345	-1.672625953	-0.524464926	-2.652305969	0	0	0	0.527060322	1.573866764	2.677506002
Burkholderia_seminalis	-1.770663793	0.157336233	0.043918573	4.306437809	1.775490423	1.982827477	0.576885268	0.398037131	-0.204155181	-0.592143169	0.98374237	0.830076917
Rhodoplanes_sp._Z2-YC6860	-1.764347343	-0.091075583	0.71530292	1.498965068	1.615681086	0.697092211	0.46849176	0.884285754	-0.524483982	1.637795307	2.097707444	1.702603762
Halomonas_sp._BC-M4-5	-1.763253528	-0.305991376	-0.528564733	NA	NA	NA	-0.063171799	1.488331796	-2.607757122	-10.91438513	-1.340737945	-2.393440433
Nocardia_yunnanensis	-1.762466405	-0.221592746	-3.839383776	-3.139301773	NA	-1.406741358	1.953092752	0.30531556	2.765102502	1.996709784	1.807354922	2.065816181
Pseudomonas_parafulva	-1.760812336	-4.066798887	0.742253656	NA	0.760099684	-0.703955333	0	0	0	0.261161759	0.015910684	-0.265845343
Echinicola_vietnamensis	-1.757714523	-1.722748477	-0.323839885	-0.415064064	-4.000557964	-1.445236511	-0.650362408	-0.452412813	0.230844969	-0.074462098	0.473397725	-0.22770282
Bacteroides_sp._A1C1	-1.756183546	-0.40360775	-1.167618995	-6.488170401	-7.226022928	-6.916403776	0	0	0	-0.187431598	-0.253642595	-0.371638991
Parasaccharibacter_apium	-1.753644335	-0.43171624	0.993768046	NA	-0.2386497	0.764237423	-1.233840686	-0.164455759	0.912637848	1.230389662	1.337680723	-0.042114203
Streptococcus_equi	-1.750281593	-2.356829906	-1.218256252	-0.477951707	-0.212041828	0.77323553	-1.498256087	1.143262792	1.212648128	0.400027862	0.071983375	0.096672964
Curtobacterium_pusillum	-1.750221691	NA	-2.637820948	NA	NA	NA	-0.164584582	-0.784816575	-0.518916265	0	7.369230901	0
Paraburkholderia_fungorum	-1.748361568	NA	NA	NA	NA	-0.508726915	-10.27718277	0.783756293	1.145171138	0.038134143	0.22485522	0.144581951
Sulfurimicrobium_lacus	-1.748221004	-1.559364187	-0.175416118	1.046878994	0.058486665	-0.955256508	1.412522212	-0.208345178	0.698091297	0.858370604	0.018719346	0.958321039
Lactobacillus_pentosus	-1.743655543	-1.47049692	-1.124997759	-1.288785111	-0.068861858	-0.269774552	-0.365614401	0.054836546	0.636874171	-0.487120894	-0.347669453	0.070120897
Rheinheimera_sp._LHK132	-1.742680597	-0.734159042	0.519748286	NA	-2.801271732	-1.335560894	0.051467605	-0.442070306	0.602321915	0.4174845	0.718761968	0.779002564
Mycobacterium_sp._EPa45	-1.74139784	NA	-0.004432246	NA	-1.234992458	-1.698648663	0	0	8.30544163	8.35167691	7.59792591	9.833944757
Xanthomonas_fragariae	-1.74139784	NA	0.194303666	NA	NA	NA	-1.101314723	-2.306656562	-1.007691514	-8.202941691	-8.202941691	-8.202941691
Flavobacterium_sp._KBS0721	-1.740643571	-1.413470574	-0.196496622	-3.658568967	0.057002445	-0.87524094	0.448120463	0.011550015	1.438625834	0.720137452	0.917417413	2.004571445
Breoghania_sp._L-A4	-1.736288749	0.924965727	1.406873473	-4.126919528	3.649890443	3.514718317	-1.199196313	0.099766486	0.278373011	-0.946890937	0.016574675	0.15802924
Lactobacillus_crustorum	-1.735930503	-6.162047448	-1.613149174	-0.181679092	-2.522895293	-1.457950564	0	0	0	1.039031812	1.011260116	-0.379834111
Thermus_scotoductus	-1.729751245	-0.837017961	-0.503447754	0.181577774	1.777345056	0.71319085	0.89456142	0.497541311	1.506765366	3.295760128	2.39663182	3.101227621
Altererythrobacter_sp._BO-6	-1.728420485	-2.299252657	0.951779812	0.429392792	0.934411658	0.719548541	-2.128490762	0.241329031	-0.973964131	-1.65027586	-0.013504798	-1.020301705
Actinotignum_schaalii	-1.726744253	-2.100928909	-0.370126054	2.403141743	1.103340767	1.798881847	-1.162568013	-0.12632677	-0.349975867	0.825364189	0.659783143	0.62068135
Enterococcus_thailandicus	-1.722592493	-2.09910215	-3.468676144	3.443332591	1.305562988	1.505548639	-0.691813799	0.378210748	-0.1144378	-0.144886965	-0.495175095	0.588583319
Granulicella_sp._5B5	-1.721548571	-5.698727611	NA	NA	-1.496409679	-0.321815071	0.7448899	0.040427233	-0.733976101	0.033192622	-1.478113247	-1.016444622
Cytobacillus_kochii	-1.721213929	-1.607600304	-0.870371469	0.844450021	1.538524445	2.006164098	-0.581325719	-0.266652417	-0.167606461	1.575560873	1.069558831	1.476117504
Salaquimonas_pukyongi	-1.720091776	-2.239465935	-1.665229841	-0.117594373	-0.946719917	NA	-0.240881378	0.21224855	0.108342957	-0.185854892	-4.168115437	-2.050969656
Acidisarcina_polymorpha	-1.719577103	-1.98932913	-1.414680529	0.731078278	-0.79997901	-0.257736099	1.856085049	2.460081761	0.856742074	0.182996053	0.388933075	-0.121666334
Jeotgalibaca_dankookensis	-1.719008528	-0.177697774	0.04267008	NA	1.915070572	2.730388054	0.056671766	0.220275691	-0.090396272	-0.049724721	1.70745468	1.847371545
Pseudanabaena_sp._ABRG5-3	-1.71788129	-2.326905822	-1.720949456	-1.193122361	1.440513986	1.736000258	1.705390957	-8.04736934	2.582488129	-0.696937568	-0.408173028	1.130604607
Pedobacter_ginsengisoli	-1.716859007	-1.495672349	-0.710000894	0.252241259	1.367388397	-0.488304225	-1.749070931	0.017072788	-2.085657756	1.381487787	0.128085207	-0.246091123
Streptomyces_griseus	-1.71525952	-1.827472584	0.269959699	1.510843086	-0.36713886	-1.465248945	-0.736471478	-1.374453188	0.791347115	0.882543472	0.199522901	-0.824279575
Caulobacter_rhizosphaerae	-1.71242341	-2.584962501	-1.271545906	1.209263149	NA	NA	-0.968575052	0.265138044	0.037784686	9.003751198	9.621562905	9.678306113
Kyrpidia_tusciae	-1.711611318	-2.324537959	-1.066706277	-1.369546115	0.656838071	-0.620239831	1.05951099	-0.263567987	1.644984387	1.281519641	0.827215039	-0.575608333
Pusillimonas_sp._ye3	-1.709291098	-4.728938555	-1.523203478	-0.006961911	-0.680894813	0.700650162	9.248359308	0	0	-0.128877906	-0.589385745	-0.352835915
Burkholderia_multivorans	-1.709177104	-2.101270523	-0.16445432	1.405521337	3.044000755	2.225313362	0	0	8.451211112	2.741617471	2.655228414	2.904726955
Wolbachia_endosymbiont_of_Litomosoides_sigmodontis	-1.707819249	0.250360576	NA	NA	NA	1.807354922	-9.222246695	-1.539568065	-2.065132227	-9.478431906	-1.880505997	-9.478431906
Brevundimonas_vesicularis	-1.707819249	NA	-1.644645051	NA	NA	NA	0.473260046	0.430746348	1.733306185	8.27301694	0	0
Ehrlichia_canis	-1.707819249	NA	-0.383145777	NA	NA	NA	8.157119522	10.42283218	8.451211112	0	0	9.108524457
Streptomyces_galilaeus	-1.707819249	NA	0.187852679	NA	NA	NA	-1.043415021	0.066519716	0.439051375	-9.247927513	-9.247927513	-9.247927513
Chryseobacterium_lactis	-1.707819249	NA	4.639137641	4.742318829	5.524261332	5.207422626	0	0	0	0.659300406	-1.139065733	-0.750020591
Clostridium_saccharoperbutylacetonicum	-1.70226512	-2.813771965	-1.809507663	-0.489903388	0.295097064	0.936090423	-9.372270887	-9.372270887	-1.627134418	0.425000087	0.465941704	0.5148471
Lactobacillus_iners	-1.702053595	-3.091713434	-2.302077062	-0.064176921	-0.728493028	-0.870648175	-0.232732071	-0.087902523	0.262101679	-0.72984695	-0.293991218	-0.02889964
Flavobacterium_sp._140616W15	-1.70183595	NA	-5.463904325	NA	-2.335074378	-1.278183042	-1.649175289	0.169773399	-0.345629882	0.085012604	0.302352086	0.459926039
Desulfurispirillum_indicum	-1.701382209	-0.017182788	-0.016690757	1.038861715	1.442941964	0.983847384	0	0	9.561202011	0.546069293	0.407403742	0.248315074
Streptomyces_fulvissimus	-1.70070057	0.180412671	-0.54090987	4.406218291	2.180748825	2.605966105	0.423994357	0.270089163	0.34801411	0.2719925	-0.808239554	-0.223568269
Azospirillum_lipoferum	-1.695726043	0.288693374	0.448983669	1.649541451	-0.360360407	1.450963418	-1.529498473	-0.449578343	-1.016914017	1.101771318	-0.432720196	1.807359544
Calothrix_parasitica	-1.693202049	-2.481169498	-1.249790838	-1.359136165	-0.625423549	-2.960437403	0.197343626	1.304354753	1.421504448	-0.118100783	0.670927988	-0.183658894
Lactobacillus_crispatus	-1.6887413	-5.126811341	-4.964675142	-0.855671968	-0.912738704	-0.99187564	-1.020149802	0.716651956	-0.331788162	-1.451533398	-0.824122699	-0.488543358
Chryseobacterium_glaciei	-1.688148978	-0.877331124	-0.538136018	NA	NA	NA	-0.587870606	0.35414712	1.8113406	0	9.713099494	10.09803208
Mucilaginibacter_xinganensis	-1.687996909	-0.941382858	0.644260279	-1.184159485	-3.718887683	-0.968001359	-0.628030313	-8.75731333	-8.75731333	0.337406479	0.566399665	0.531326754
Micavibrio_aeruginosavorus	-1.687462261	-0.18967001	-0.6332524	-0.311628894	1.039281336	1.193569586	0.23343263	1.491780263	2.234135203	0.598380547	0.852484899	1.470104913
Acidovorax_sp._16-35-5	-1.682479007	-1.907362142	-0.008827707	0.23877907	-0.579915701	0.612729377	-1.321928095	1.286268633	1.227868672	2.826142368	2.342503127	2.814465478
Acholeplasma_brassicae	-1.682425687	-1.909020812	0.547160468	-7.010634852	-1.077835995	-0.339876035	-0.525656963	0.019176607	-0.008773144	-0.022317986	0.57264065	0.871200363
Arcobacter_suis	-1.681698288	0.351798624	-0.83208853	NA	NA	NA	-10.33315535	-10.33315535	-0.427631454	1.677362879	1.680204978	0.683741791
Mycoplasma_edwardii	-1.681018329	-2.073757161	-3.589595302	NA	-1.514394537	0.363264651	-0.785001122	-0.71874092	1.059909914	3.082909355	2.636966353	3.084428781
Lawsonia_intracellularis	-1.678701819	-4.373947894	-1.261430385	-2.771318821	-3.314073826	0.63604787	0.02924009	2.683358043	-1.97534038	1.046234372	1.696172386	1.113201917
Nitrosococcus_watsonii	-1.676750842	-1.939978425	-2.307088708	NA	1.972815171	2.762703156	0.352116676	0.83308785	-0.306190357	9.410805357	5.472487771	9.020516091
Sulfuritalea_hydrogenivorans	-1.675133816	-2.030353678	-3.894157931	-3.046028531	1.004186534	0.52779806	0	0	0	1.324398874	0.90100281	0.951140596
Proteus_vulgaris	-1.672534509	-1.189917671	-0.402149815	NA	0.832185872	0.726463758	-1.468619903	1.427919627	0.638291618	0.606084939	0.154317605	0.142841509
Dietzia_lutea	-1.667895027	-1.473530066	-0.670766062	-3.753750178	-0.203740277	-1.273998575	0.330656832	0.63767985	0.705659905	2.153564067	0.672056961	1.670395007
Libanicoccus_massiliensis	-1.667505443	-0.131601266	0.676012508	-0.081705125	-0.384037816	2.331869844	-8.04736934	-8.04736934	-8.04736934	0.400777807	0.762491318	1.625201145
Lactobacillus_delbrueckii	-1.667206426	-2.850622376	-1.232689878	0.361448193	-0.540629553	-0.6045779	-0.190934565	-0.65680967	1.090260384	-0.907323203	-0.581478424	-0.406167317
Microlunatus_sp._KUDC0627	-1.664607612	-1.369660706	-0.02918655	-1.367035036	0.527002034	0.66208035	-0.322748394	0.123962742	0.732040403	-1.522931858	-2.302940898	1.261524521
Candidatus_Pelagibacter_sp._RS39	-1.663495903	NA	-2.191099025	NA	0.717406817	-1.751695839	0.714967525	0.272023851	0.675937321	3.038775514	3.687577567	2.850951327
Mucilaginibacter_sp._G2-14	-1.661877652	-1.629399355	-0.800935836	-2.627503654	-0.70879413	0.308667897	8.6039638	8.110425888	6.15711649	-0.136581417	0.231848617	0.663426653
Serpentinomonas_mccroryi	-1.659658175	0.297678568	0.054629533	3.944653021	2.646339509	2.283227979	9.029913869	9.426646347	8.863904389	0.691379148	0.642716419	1.1634532
Corynebacterium_genitalium	-1.653268514	-0.694198778	1.174976734	NA	NA	NA	0.587634456	0.488169761	0.131832108	2.027966296	-8.044394119	2.190028163
Arcobacter_molluscorum	-1.653268514	-0.326103771	-1.431164372	-1.653521197	NA	-2.151722386	0.8747875	0.532830309	1.853402232	0.392901666	0.844818012	1.285924508
Pseudomonas_alcaligenes	-1.652330849	-0.991132762	0.969437093	NA	1.413244217	1.952171475	-1.000397194	-0.030628645	-0.480035814	-1.801454321	0.761586981	-8.930737338
Cellulomonas_flavigena	-1.652330849	NA	-1.117665911	NA	NA	NA	0	0	6.15711649	-1.557767376	-3.980097368	-0.931640441
Calothrix_sp._NIES-2098	-1.648769658	-2.565604199	-0.732057867	-2.412857061	1.853720509	-0.744538814	0.953098272	0.30531556	0.888968688	2.145279537	1.313352431	-0.721974159
Saccharomonospora_marina	-1.646556231	1.507494691	1.83403649	NA	NA	NA	0	0	0	0.472682393	0.825416534	0.7659583
Peptoniphilus_ivorii	-1.645956922	-2.904057369	-0.891334999	1.033457549	1.377926451	2.16861139	0	0	7.157114468	-0.010459166	-0.113579268	0.413975313
Thermodesulfobacterium_commune	-1.644259684	-2.038596275	-1.911739995	NA	0.004001931	2.695498977	0.689424412	1.061997336	1.334722719	-0.368747769	-1.465273836	-0.531162539
Providencia_rettgeri	-1.643306487	0.314873337	1.823600252	NA	NA	0.062862117	0.439531402	1.205349002	1.100706701	0.771211898	1.341725421	-0.221401836
Entomoplasma_somnilux	-1.643306487	0.314873337	NA	1.365723213	-1.431601344	-0.66430375	-1.91043726	-0.748167994	-0.954030668	0.563360989	-0.48726675	3.586876021
Mycobacterium_shinjukuense	-1.640244936	0.032180406	1.929610672	0.774139315	1.159563511	-1.593546347	0.505247347	-0.403956729	-0.477727609	0.677345848	1.071782704	0.829056356
Synechococcus_sp._A15-60	-1.640244936	NA	-0.905535316	NA	NA	NA	-1.306872481	0.590000887	0.923149513	8.45395786	0	0
Microbacterium_sp._HY82	-1.640244936	NA	0.214623447	NA	NA	NA	0	0	0	-0.595890689	0.164470571	1.467820839
Thioflavicoccus_mobilis	-1.638762465	-3.030714216	-0.332659337	-1.549406131	-2.266916348	0.117605714	-0.561065309	-0.220320401	1.062701645	0.410341105	-1.251538767	1.372765482
Actinopolyspora_erythraea	-1.637694744	-1.914820197	0.274399932	1.238553626	-1.352098932	-1.515781342	-1.296597991	0.249717239	-3.016000469	0.945609772	-0.030654831	0.037195186
Geitlerinema_sp._PCC_7407	-1.637238721	-1.125165887	0.273750189	0.780911882	1.271585545	0.910146002	0	0	0	0.560877789	0.368181065	1.533505679
Mycobacteroides_abscessus	-1.634369757	0.259734605	-0.082487927	-0.204177272	2.981514711	2.842370078	-0.809471636	-0.726135101	0.424644952	1.848465597	1.469849149	1.08188552
Caldicellulosiruptor_kronotskyensis	-1.633744874	-1.90781333	-0.781302062	NA	0.896906507	2.239348876	-10.12106722	-0.742869921	2.080165359	-0.247159713	0.808582839	-2.682744589
Candidatus_Phaeomarinobacter_ectocarpi	-1.631291552	-1.442434734	-0.593454731	0.379825468	1.316547636	1.147235062	0.649290109	0.693370884	0.621860635	0.587739934	0.165284727	-2.305344684
Streptococcus_gallolyticus	-1.63000546	-1.984029424	-1.381278891	-0.339797931	1.019123248	0.846290585	0.556263001	0.971129431	0.475594938	-0.018101588	-0.363815975	0.182790613
Gemmatimonas_phototrophica	-1.629017681	-4.135624544	NA	-0.766498793	-0.886080408	-0.362983163	-0.760844268	-0.488766038	0.201159603	0.224982426	-0.450108604	3.147952785
Virgibacillus_necropolis	-1.626020908	-1.173816292	-0.202470683	NA	4.288801054	4.23956666	-1.671322491	0.707545798	2.028395699	-1.419177746	1.055250837	1.524458015
Sinorhizobium_fredii	-1.625663037	-0.686750483	-0.369539811	-2.111883717	1.602155199	2.084915274	0.397067487	2.97538445	2.590503594	0.455237255	0.11248767	0.034866172
Saccharophagus_degradans	-1.623454348	NA	2.179714872	-4.658798382	0.828068775	1.415526399	8.409390936	0	0	0.35082255	0.233253942	0.636743977
Neisseria_dentiae	-1.619926173	-0.199045429	0.426170242	NA	1.286492506	0.693454015	-0.795580776	-2.812091619	-3.765403038	-0.900843748	-0.555605298	0.45591085
Caldicellulosiruptor_changbaiensis	-1.619029085	-1.206986061	-0.971076267	NA	1.542395639	2.243195145	0.281039622	0.279874716	0.654988493	0.533499027	0.931358334	0.290387333
Ruegeria_sp._THAF33	-1.61739079	-1.024786068	-1.565021891	NA	NA	NA	-0.628883177	-1.572160448	0.396203346	0.182292893	-0.284488991	1.368027962
Anaerolinea_thermophila	-1.614876387	-2.690580184	0.365647999	-1.884815173	-0.192485394	-0.362761723	1.064415597	2.929117152	1.783633952	-0.04118966	0.183624626	0.799806379
Pandoraea_vervacti	-1.613721494	-2.597673552	-1.460750473	0.748174956	0.074505436	0.399444565	-0.871930079	0.371514282	1.029097033	-0.173286917	0.368357062	1.029783082
Campylobacter_avium	-1.60899257	-0.100583573	-1.275923918	NA	-3.597562538	-3.911377987	0.062644813	1.140218755	1.690117585	-0.724381853	-0.659399345	-1.315813186
Brevibacterium_linens	-1.608441568	0.824240957	-0.166099642	2.622607275	1.378511623	1.493694443	2.427604934	2.345772495	3.207373227	-0.896250603	0.910770526	0.0436277
Melioribacter_roseus	-1.608015375	-3.121758857	-1.145828339	-1.90554841	-0.26169222	0.392499404	-1.028597236	-0.708470248	-1.136714853	-0.022864108	-0.193941883	-0.336796826
Moritella_marina	-1.607379967	-2.334695391	0.111560839	NA	0.021247734	1.165637643	0.210342523	-2.337101109	-1.019824651	-2.321342154	-1.487992024	-0.285366411
Corynebacterium_pseudotuberculosis	-1.606988807	-1.26573951	-2.609007975	NA	4.331621491	2.083962311	1.835246492	0.673555222	-0.785874739	9.379378367	9.067883368	0
Flavobacterium_sp._CJ74	-1.606864868	0.256661406	-0.982323951	0.315495794	-0.001013722	0.539787034	-0.164106196	-0.314645369	-1.450889096	1.02758994	-0.663234878	1.155011611
Lysinibacillus_sphaericus	-1.605028124	-1.156734215	-1.596687076	NA	-0.985475759	-0.49470862	-0.487265977	-2.134751709	0.034054873	0.719194691	0.960909668	-0.140115954
Serratia_odorifera	-1.603767219	-1.285144269	-0.553163914	NA	NA	NA	-0.644908396	1.559382593	1.879640109	1.789194474	2.808000042	2.940939518
Deinococcus_peraridilitoris	-1.602868945	-2.933785823	-1.732531402	2.228391162	-1.415037499	2.118248967	8.6039638	8.110425888	10.93596796	1.030227198	2.673554245	-0.879570838
Enterobacter_sp._E76	-1.600461787	-3.181287949	0.272661809	NA	NA	-1.567057275	0	0	10.89454496	0.562486491	0.631203973	1.629740891
Celeribacter_baekdonensis	-1.597020918	1.42710229	2.498812455	1.697996542	4.505028978	3.904202874	-0.535924971	0.995729183	-0.142502494	2.069398125	2.188558415	0.911811402
Acidiferrobacter_sp._SPIII_3	-1.597018518	-3.586964854	0.503794501	0.560783963	2.276430835	0.936178082	-2.514867145	-1.108343025	-0.252940954	0.04354548	0.408392864	2.027120509
Pseudomonas_cedrina	-1.595016165	-5.572195205	-1.300382565	2.278600663	1.252562802	3.367856272	0	0	0	0.712490874	1.248353234	-0.202308175
Nostoc_sp._PCC_7524	-1.594485275	2.675399972	4.070615296	0.765283646	-0.696975863	-0.486494304	0	0	0	8.81164121	0	8.996239023
Brevibacterium_sp._YB235	-1.594266491	0.226526695	-0.481259527	4.302107187	5.170299484	4.507290787	0.250735073	0.551430831	0.698106105	2.488013232	2.921449491	2.99519964

Bacillus_cohnii	-1.591757557	-2.61900668	-0.451836333	NA	-2.029179414	-0.449242934	-3.202975386	-1.18001234	0.592204822	-0.701760371	0.054385742	-0.866248801
Pseudomonas_sp._11K1	-1.589174752	NA	1.535037275	NA	NA	NA	0.491677203	0.619462385	1.261743177	7.333897756	0	0
Weissella_paramesenteroides	-1.587407057	-4.871504997	1.941428547	-3.197672743	-1.184733687	0.219556377	-0.642981149	-1.122858724	-0.082636909	-0.24082975	1.281549412	1.438222898
Marinithermus_hydrothermalis	-1.585394898	-3.206883275	-0.562228914	-0.037023271	0.827461758	-0.209150349	1.545639795	0.822639168	0.462023285	1.289647194	1.532524516	1.130227935
Phaeobacter_gallaeciensis	-1.582313044	-0.941342349	-1.163755989	4.855574275	2.830130987	3.028756953	0	0	0	-0.230368919	-0.49356453	0.433146684
Xenorhabdus_bovienii	-1.581932689	-2.243553158	-4.230842106	-2.151613123	-2.460602162	-2.941527597	-7.757316664	-7.757316664	-7.757316664	-0.671500891	-0.664954773	-2.031631457
Sphaerochaeta_globosa	-1.578982526	-1.054785276	-0.181404133	2.438969499	0.96913491	1.203345035	-3.236920254	-0.295974177	1.097107423	-0.157044499	-0.225387788	-0.066696632
Colwellia_sp._PAMC_20917	-1.577992946	-1.865569536	-2.550446619	NA	-2.649970217	0.159418819	-0.305961494	0.365121859	0.627346101	-1.918329839	0.215759696	-1.444164862
Campylobacter_cuniculorum	-1.57737022	-1.100623554	-0.421635173	-2.754928636	-1.387092734	-1.82840323	-0.250802783	-0.498554451	-0.189901319	-0.756693398	-0.417615464	-0.521842869
Arcanobacterium_phocae	-1.576574715	-1.832600873	-1.707819249	3.078002512	NA	-2.373855887	0.968134174	1.399081137	-0.042952254	0	0	7.63420844
Vibrio_anguillarum	-1.576574715	1.095604622	1.445213974	NA	NA	-0.520414747	-10.78953364	-2.16216392	-0.726138564	-7.544323092	-7.544323092	-7.544323092
Streptomyces_cacaoi	-1.576574715	NA	0.057715498	NA	NA	1.086414752	-1.079676365	-0.34796627	-1.678515368	-9.855907186	-0.997926191	-0.903652257
Pantoea_stewartii	-1.57569334	0.727176003	-0.25215669	-2.426988696	0.798920915	1.950424719	0.051467605	1.974443122	0.926548391	2.178389997	0.889346166	1.159399822
Acinetobacter_sp._WCHAc010034	-1.569749407	-0.388079682	-0.384437296	NA	0.147654454	-0.498653618	0.397067487	1.808031897	1.219326726	0.295476637	0.385293144	-0.139344534
Amycolatopsis_methanolica	-1.569563341	-1.576921598	-1.009167532	-0.007399991	-0.465654073	0.920655864	-4.231708818	-1.658730335	-2.549393701	0.365601817	-0.28613689	-0.084680079
Kutzneria_albida	-1.568919658	-1.664310004	-0.299914883	NA	-1.505474971	0.794348938	-0.331294625	0.236761885	0.01434868	0.444740809	0.681014715	0.943158693
Thalassotalea_sp._LPB0316	-1.56855959	-1.399458562	-2.017097318	0.740058792	-0.339630097	-0.321928095	-1.080758254	-0.259655544	-1.141345718	1.962170401	2.207478796	1.327746147
Alteromonas_mediterranea	-1.566687442	0.079765278	1.133286565	-2.308368831	-1.047923431	-1.548640987	0	0	6.15711649	0.382227225	-0.25573679	1.084567748
Alcanivorax_sp._N3-2A	-1.56632725	-4.135840733	-1.091701132	-1.38966118	-1.105749078	-1.272403881	-0.35187458	0.123439209	-0.013268149	-1.755514094	-1.299626744	-0.35814698
Porphyromonas_gingivalis	-1.56427717	-0.695539167	-0.117660595	-1.241462203	-0.799583285	-0.260981452	0.185261069	0.174585944	1.159133422	-0.219597824	-0.274603127	-0.112314553
Lactobacillus_rhamnosus	-1.560664949	-5.002502507	0.399135544	0.04536482	-0.47783193	-1.542828607	0.582783864	0.744411191	0.198853922	-0.21446552	0.352696766	0.658833715
Bordetella_genomosp._9	-1.56019579	-0.759216462	-1.963417989	NA	-1.300238506	-0.218480026	0	0	7.157114468	-1.115354042	-1.900442647	-1.190391572
Pasteurella_multocida	-1.558787782	-4.736132681	-1.265776926	-1.14127457	-0.941914473	-1.243749979	-1.621316436	0.760680893	1.869765088	-1.703903697	-1.430425751	-0.604002779
Nostoc_edaphicum	-1.556433703	-2.365807793	-2.883415026	-0.776205679	NA	-2.042992219	-8.04736934	-1.364697732	3.727829389	0	0	0
Arcobacter_lanthieri	-1.556284366	-1.911026666	-3.331843564	-5.86941589	-0.064654784	0.017994016	-10.57501918	1.021338324	-0.614490092	2.222203842	2.297172169	1.051927288
Lactobacillus_vaginalis	-1.553563969	-0.531748499	-1.764640263	2.544555787	-0.263549194	-0.52757251	1.443869495	1.712430676	1.701890675	-0.23984535	-0.724036202	-0.353705952
Pannonibacter_phragmitetus	-1.551832446	-3.299429953	-0.395076504	-0.376337231	1.143488723	0.49306919	-0.195746854	1.197121289	1.272082864	-0.17230689	-0.670287564	0.013034188
Pseudomonas_syringae	-1.550386138	-1.56609553	-1.163616364	1.498419666	-0.782636148	-0.497086322	-1.122975767	-0.572846366	0.13213278	0.781427771	1.336075143	1.468503787
Odoribacter_splanchnicus	-1.550199171	-1.309062756	-0.705084109	-1.492685317	0.160711036	0.48548047	8.6039638	0	10.32090206	0.46325622	0.855461873	0.411294412
Rummeliibacillus_stabekisii	-1.549208753	-1.797857015	-0.016444416	NA	-0.764193566	-0.768374789	0	0	6.15711649	0.831478445	1.444166189	0.001349114
Shewanella_piezotolerans	-1.547415991	-1.050826119	NA	NA	NA	2.092331881	0	0	0	-10.30833903	-10.30833903	-2.769180219
Lactococcus_lactis	-1.546311725	-2.585261203	-2.725644877	1.090619676	1.267741519	1.495077967	-1.961392378	0.218239168	-0.884424001	-0.124844116	-0.296634311	0.592262072
Mycobacterium_colombiense	-1.545415453	-1.588838855	0.036854195	2.018774074	1.246588052	0.731139458	0.846007406	0.487493031	0.459894842	0.355480655	-0.360450028	-0.349294
Rhodopirellula_baltica	-1.544973432	-2.412735448	-0.915440267	-1.482819284	0.518699609	1.601780119	-9.575022961	0.216732516	-2.417908492	8.27301694	7.894817763	10.23202512
Acetobacter_aceti	-1.54434776	-0.402214038	0.100690326	-0.029428508	-0.99310421	1.626171255	0.121612459	-0.702407422	-0.422250337	-0.616881835	0.744522914	0.535362575
Corynebacterium_imitans	-1.544010092	-1.012534679	-1.220778946	NA	2.294928833	1.937856946	-0.336285842	-0.007842508	0.547793884	-8.044394119	1.256340516	-8.044394119
Rhodospirillum_rubrum	-1.542930413	-1.322113543	0.549672338	-1.514982971	-0.592871639	0.015334686	-0.09583238	-0.845472363	0.823628261	1.237845433	0.436173461	0.658891666
Oceanithermus_profundus	-1.541905793	-0.592497884	-0.697481389	1.644341048	0.828467741	-0.001455553	3.070156886	2.375462836	5.259888401	-0.135699642	-1.341739092	-0.234847484
Gloeothece_verrucosa	-1.540568381	-1.997228251	-1.21420804	0.515371039	-1.407282153	-1.403217075	10.91574499	10.42283218	10.89454496	1.777564921	1.348540282	1.403900765
Serratia_sp._ATCC_39006	-1.539402046	-1.981602804	-0.873786573	-2.076611926	1.005307494	-0.321027871	0.683013491	0.652358512	-2.006102952	0.512132146	-1.297317615	0.18182214
Achromobacter_xylosoxidans	-1.539397539	-2.496958396	0.08358145	1.218084535	0.269164542	2.617387824	-2.000577307	-1.042533576	-0.325453014	-1.298974894	0.668267616	0.282099913
Prevotella_jejuni	-1.537947166	-0.522994189	-0.048214606	0.275945511	0.415596402	0.653945631	-1.176810851	-0.282487917	-0.367889899	-0.146190284	-0.169716037	0.013341792
Dysosmobacter_welbionis	-1.53771181	-2.44548356	-0.8602343	0.287963733	-0.166937885	-0.053561448	0.439531402	-9.222246695	0.614663654	0.352145447	0.200302155	0.058521422
Oscillibacter_valericigenes	-1.536707701	-2.298857211	-0.923171334	0.077625418	-0.058075761	0.248250009	-8.10567284	-1.423001232	1.371532928	0.307660898	0.10717351	-0.055647307
Paenibacillus_baekrokdamisoli	-1.534488929	-4.027266179	0.287433134	-2.174465221	-1.218714366	-0.864149153	-8.552496194	-8.552496194	-8.552496194	-0.440028347	0.23094978	-0.229971266
Streptosporangium_roseum	-1.533926415	-0.463623396	1.308723995	-0.786654819	0.58755821	2.526777688	-0.72705302	1.229365609	1.669447597	1.728413991	1.908801426	2.733416824
Pontibacillus_sp._HMF3514	-1.529427824	-0.679190707	-0.828832902	-2.626211552	-2.319098488	-1.911906556	1.922775196	2.623391763	2.311125254	1.167600273	0.327777857	0.363361127
Acinetobacter_guillouiae	-1.522951861	-2.22397022	-0.632751443	NA	-3.051507862	0.819119189	0.012116093	0.595402183	1.984966769	-0.760192349	0.931453978	0.174299158
Vibrio_navarrensis	-1.521676256	NA	-0.594699512	NA	-2.037474705	NA	-1.766936254	0.537474973	0.826909674	1.839267932	2.215819767	-8.044394119
Fusobacterium_varium	-1.518992974	-0.87633913	-0.168209062	-2.293899396	0.032987422	1.148363839	-10.50364688	-0.854881267	-10.50364688	0.701072525	0.717501814	0.190500699
Actinomyces_sp._oral_taxon_897	-1.517450682	-2.761178541	-1.142411251	-0.600052311	-0.171244153	2.144457526	0.250179779	-1.268041634	1.411910019	-8.988684687	2.120708523	0.657573393
Rhodococcus_fascians	-1.516744272	-1.957857044	-2.174792809	-0.742122028	0.970658557	0.05341252	-11.73978061	-2.970392336	-0.073960618	9.960967751	11.03158405	11.15481811
Candidatus_Desulforudis_audaxviator	-1.514697484	-0.271587096	-0.460710206	0.114778359	0.737179295	1.280189632	0	0	8.451211112	0.02527845	0.487468444	-0.357648067
Campylobacter_ureolyticus	-1.513496635	-0.639449473	-2.664379189	-3.005687153	-3.318926301	-0.993005505	-0.622950173	-0.398715733	1.196912809	-0.008178641	-0.305330469	-0.885262864
Orientia_tsutsugamushi	-1.512985718	-7.373047182	-0.82986202	-3.971662469	-3.335387877	-2.745366553	-0.606251507	-0.856964169	0.213892124	0.485685865	0.780487736	-0.193795365
Chryseobacterium_bernardetii	-1.51288942	-3.125259308	-0.589726929	0.038054812	-1.700786117	0.569330508	-0.489249772	-0.783418771	-0.018168281	0.403338084	0.560596589	1.00503389
Lactobacillus_mali	-1.512441389	-2.446314794	-4.926643753	-0.953836788	-0.859281556	1.706147449	-1.090837488	-0.039572396	-2.19252579	0.35829101	0.92302222	-2.212050477
Bombella_sp._KACC_21507	-1.512061954	NA	0.37036845	0.15298086	NA	NA	0	0	0	-0.918751496	-0.394594663	1.659324619
Pseudohongiella_spirulinae	-1.509238496	1.203056437	-0.369769756	NA	NA	NA	1.187276815	0.004753048	2.370617704	-8.818048457	-3.345560686	-0.405830065
Ligilactobacillus_salivarius	-1.508336004	-1.969056678	-0.45595258	-0.031063179	0.352995236	0.184239113	0	8.627369725	0	0.525704871	0.831885593	1.16100628
Pseudomonas_litoralis	-1.505400231	NA	0.280396586	NA	NA	NA	0	10.35434857	8.548995253	9	9.214319121	9.306820443
Mycobacterium_mantenii	-1.504923003	NA	0.690724563	NA	NA	NA	-2.262647918	1.003897707	0.423308244	2.194517675	0.226068079	-0.173146482
Serratia_marcescens	-1.504333475	2.187087068	-0.331606368	1.560457391	0.190718762	0.412262788	10.77912016	11.23753862	10.75200424	-0.349599251	0.181767621	-0.36091769
Actinobacillus_porcitonsillarum	-1.504009593	-2.550973073	0.730797896	8.780524485	-2.234992458	0.172113768	10.02614993	11.48042166	12.66551802	3.451605591	3.034783245	3.11547474
Pontibacter_pudoricolor	-1.500972991	0.024134345	0.686114727	-0.912193719	-0.308575773	-1.247723683	0.080858239	0.588086892	1.347288103	0.872845088	1.038691744	1.072314205
Oxynema_sp._AP17	-1.498302004	1.361603868	2.207609477	-1.99212644	NA	-0.522263142	3.722185743	4.783779951	4.452800627	-0.355031496	1.380875079	0.820477696
Staphylococcus_hominis	-1.497330054	-0.775001083	0.399819311	1.835421748	1.705538937	2.56714435	0.865458122	1.479059767	2.116993707	-1.398189983	0.464899487	-0.71677605
Massilia_flava	-1.497133801	-2.842942667	-0.810867201	NA	0.213281371	0.947713698	-3.180925203	-1.139411865	-1.307832919	10.21026952	10.70159237	10.50051328
Virgibacillus_halodenitrificans	-1.494377067	-1.686678907	-1.515792101	NA	-1.905113096	-1.976311549	0	0	8.742080338	0.870682776	1.006735256	0.436765245
Halomonas_sp._N3-2A	-1.493879615	-4.718053431	-1.274482103	-1.332193753	-0.951783131	-1.251444032	-0.524128233	-0.239622361	0.939401706	-1.662338543	-1.44242525	-0.723614157
Tumebacillus_algifaecis	-1.49080445	-1.043153925	-2.200884294	1.125558144	1.227263275	0.71871389	0.087967553	0.271406328	0.386331171	1.032781271	1.824110896	1.675768869
Nocardioides_seonyuensis	-1.489700508	0.200848682	0.22688724	NA	NA	NA	0	8.559465045	9.069511529	0	9.707359132	0
Aneurinibacillus_sp._XH2	-1.487560635	-1.482746834	-0.16949518	1.165447974	0.386871768	1.49894619	-1.035695581	1.124883075	-0.34073538	0.822306594	0.726902559	1.427823809
Spiroplasma_helicoides	-1.487027747	-1.395533188	-1.418678525	0.521865665	1.555003598	3.327036708	0.144890785	0.677799237	0.504866969	0.077726326	1.556057117	2.064070675
Pseudomonas_oryzae	-1.486651966	-1.432253069	-1.062568296	-0.012722077	0.059790423	-0.62123284	-9.59551112	-0.485087842	0.095914867	2.43890158	1.807554359	2.622088758
Halanaerobium_hydrogeniformans	-1.485704095	-2.552545644	-1.026369533	NA	-0.76125577	-0.887994508	-1.913016698	-10.78953364	-0.818507485	0.979637856	0.451280555	-0.065992719
Bacillus_sp._NSP9.1	-1.484881831	-0.380498708	-0.721603209	0.955880708	0.276319185	1.526652131	-0.435920353	0.303311245	0.859680089	0.895375393	1.23034679	0.898979071
Enterobacter_oligotrophicus	-1.48235567	0.138356085	-0.328129677	-0.913180658	2.589429821	2.98507102	0.737385782	0.099886225	-1.138352397	2.554968721	1.431912027	2.330357788
Exiguobacterium_sp._N4-1P	-1.478629817	-4.097653464	-1.136897781	0.51143526	-1.040596863	-0.976503373	-0.125046422	0.278314507	0.028535984	-0.889451679	-1.150811355	-0.498654664
Desulfosarcina_widdelii	-1.477524676	-1.513577867	-0.154357979	0.030864033	0.794204432	-0.322813055	1.375537605	1.077051943	-0.278576871	0.115856378	0.544501224	-0.254151693
Gordonia_bronchialis	-1.477178674	-1.539462953	0.814427651	-0.296420574	1.533232169	-0.876566059	4.34673425	0.069612343	1.450747777	1.37178755	0.891217599	0.646592384
Arthrobacter_sp._QXT-31	-1.475689816	-0.907355906	0.96531401	-1.879973039	NA	0.694844078	-0.048833241	0.786247781	0.113478288	0.19754156	0.582355155	0.698439036
Thermodesulfobacterium_geofontis	-1.470372481	-0.505508087	-1.837513932	-4.38243951	-0.151276879	-0.734366965	-0.187214829	-0.435326195	0.847411533	0.022331858	-0.532469174	-0.716711316
Kocuria_palustris	-1.469234794	0.318667766	0.049379024	-0.210035215	-1.665626812	2.578076115	-0.540052108	1.605490979	-0.379031613	1.643101702	-2.172763483	1.182904965
Methylobacterium_durans	-1.468148836	-4.777309635	-1.674599713	2.696258695	1.437580068	1.368378517	0	0	9.233183447	1.508600496	1.69066072	2.252596292
Streptomyces_xinghaiensis	-1.462778452	-5.777273583	-1.833456418	-1.112077664	-1.283955393	-2.040663331	0.202827987	0.879803512	0.658325145	-1.219100835	-1.337316337	-1.275623957
Cellulosilyticum_lentocellum	-1.461748388	-1.974022987	-0.781415438	-0.955679585	0.02784337	-0.884959392	1.74692689	1.668216373	1.585753146	0.160082927	-0.261394021	-0.35692051
Chlorobium_phaeobacteroides	-1.46061493	-2.284118453	0.050650423	-1.845761272	0.535746603	-0.338738104	1.556105257	2.629502363	0.860111444	-0.384562888	0.304678594	0.237056284
Gordonia_terrae	-1.459981811	NA	-0.034139439	NA	NA	NA	1.197187943	1.192746936	0.103198047	0	7.712410067	10.15987134
Carboxydothermus_hydrogenoformans	-1.458453702	-2.811146667	-1.381126861	0.671448377	-1.176877762	-0.513886755	-8.04736934	3.220265939	2.854048182	0.149134402	0.816078458	0.173405525
Brachybacterium_ginsengisoli	-1.458364931	-2.898379096	2.385934707	-0.940763611	-0.195690992	-1.492052022	-0.022558473	0.099107313	0.422428437	0.401530017	-0.230208713	0.252953155
Salinispora_tropica	-1.457714562	-4.704765938	-0.962210663	NA	NA	NA	8.166278594	8.607330314	0	1.588056071	-0.708083811	0.479538437
Chryseobacterium_taihuense	-1.454206749	-1.907831813	-0.953524691	-1.424396069	0.350163984	-0.142513869	-2.924269589	-1.429504731	-0.528384194	0.481470436	0.276736976	0.572678996
Microbacterium_endophyticum	-1.453050825	0.822766303	0.340228912	NA	NA	NA	-11.22334411	-1.796697762	-11.22334411	0.382248036	1.446110276	1.508686342
Hydrogenovibrio_crunogenus	-1.451481614	-0.602792734	-1.08683661	3.668754409	2.629853556	3.232828254	9.288762279	0	0	0.907559018	0.619409513	0.344952581
Bacillus_sonorensis	-1.446041655	-0.47859459	-0.030784135	-2.393621136	-2.589481884	-1.250146476	0.139790393	-0.354649532	-0.3017668	0.378411253	0.612690804	0.728665841
Chondromyces_crocatus	-1.445613019	-0.553331844	-0.040732342	4.415883122	2.556080009	2.576268965	-9.222246695	-0.59487697	-9.222246695	-0.183321544	0.097073504	0.162208922
Sphingomonas_sp._MK52	-1.444496563	-2.253870653	-3.50844348	NA	NA	NA	0.385649343	0.33056507	0.280714671	0	0	7.539158811
Carnobacterium_sp._17-4	-1.444496563	-0.674340259	-5.206564938	-1.733606582	NA	-2.83344185	10.61069268	8.6794801	6.15711649	1.054868469	1.789212045	1.615701527
Mycoplasma_haemocanis	-1.444389584	-2.171705008	-2.949312587	NA	NA	NA	-1.276613117	-0.581386691	-0.030390398	0	0	0
Turicibacter_sanguinis	-1.442142509	4.15736153	4.262786605	-0.296657682	6.827578947	6.528556321	-9.316698068	-2.77318585	-3.386418086	1.998521829	6.86803096	5.916826795
Bacillus_thuringiensis	-1.441528588	-1.805798003	-0.483829814	-0.947528962	0.080955911	0.286538174	-1.621472854	-0.734054131	-0.171018023	0.124529224	0.000461404	-0.0798111
Jeotgalibaca_sp._PTS2502	-1.441235916	-0.855339935	0.54716199	3.646777093	-0.075013354	2.067838717	-1.997133059	-11.15883687	-11.15883687	0.092174364	0.396741768	-0.013969266
Lactococcus_garvieae	-1.440724852	-1.936410371	-1.661189825	3.920928429	0.111031312	0.45206823	-0.09696887	1.241578679	0.785786386	10.01402047	10.38197548	10.42906146
Olsenella_sp._oral_taxon_807	-1.440458118	-0.74189091	0.18279127	0.587572261	-0.116571598	2.714793991	-1.597858621	-10.76185038	-3.604735909	0.334847731	0.730411036	1.889977903
Metabacillus_sp._KUDC1714	-1.435476732	-2.392338039	-0.272216991	-3.444149007	-0.053081212	0.020786533	-1.793952468	1.336711655	1.054073345	0.197929517	1.080390257	0.802442213
Nocardioides_sp._S-1144	-1.434739414	NA	0.750069825	1.148863386	NA	NA	0	0	9.104480817	7.303780748	0	9.201307944
Acidobacterium_sp._4Y35	-1.43423416	-0.435288633	-2.163956473	-3.856485642	-1.199071347	-1.577288573	0.550791583	-2.075057648	1.213852193	1.003008235	0.681920687	-0.858028505
Clostridium_sp._DL-VIII	-1.433848135	-1.51484812	-0.892489256	-2.331990679	0.618994723	0.225060203	-0.01568066	0.419442516	0.423936581	0.870890007	0.90194099	0.020398547
Amycolatopsis_orientalis	-1.433728025	NA	0.144298429	2.592149041	NA	1.910847622	0	10.96381366	11.19060869	-0.946921576	-0.098622083	-0.65941245
Roseivirga_pacifica	-1.432043263	-1.668003734	-0.750883659	-4.998702029	0.366802206	0.885699119	0.416607003	-0.33551633	-10.02614993	0.603273881	1.751473064	1.293919326

Azotobacter_salinestris	-1.431494227	0.090965307	0.354881718	NA	NA	NA	-1.440574055	-0.894476323	-0.984703631	2.098317774	2.665068418	1.496286647
Pedobacter_roseus	-1.429667174	-2.509859021	-0.23241288	0.049509277	0.52310979	-1.000073748	0.982544529	0.897275379	2.478689687	-0.436968336	0.27403191	-0.403786923
Chlorobium_chlorochromatii	-1.428093652	-1.433957041	2.341811217	-4.007038748	-1.105276922	-0.763955749	0.849629726	1.555212683	-0.642016365	0.133674879	0.556329547	0.506011159
Rathayibacter_sp._VKM_Ac-2760	-1.42739019	-2.154705614	-0.561750322	NA	NA	-0.353050612	0	0	11.45097923	-8.900866808	-8.900866808	-8.900866808
Streptomyces_anulatus	-1.426264755	-0.22659241	-0.276517635	-0.497901155	-0.55892464	1.131971306	-2.063009798	1.830074999	2.67354676	-1.366541962	1.344869457	-0.066234909
Desulfococcus_oleovorans	-1.42322193	-3.302655165	-1.059975205	0.027055236	-0.792849521	0.567230102	-0.999275071	0.688407049	0.46286036	0.946699355	1.017140543	0.923859255
Microbacterium_sp._LKL04	-1.423211431	-1.51662237	-0.851880796	-0.337972716	1.121629444	0.797995028	0	9.110423278	6.15711649	-2.694524402	-1.245046257	0.239325907
Clavibacter_michiganensis	-1.421688064	-4.609434989	-1.900211577	0.008583238	0.419156674	1.543849004	-0.203813337	1.201688907	-0.799456639	0.242335273	0.489699923	0.04342545
Streptomyces_sp._NA00687	-1.420620021	NA	-3.985661828	NA	0.724318218	-2.48758709	-10.14442882	-10.14442882	-10.14442882	-2.033098432	-0.05610841	-9.33687918
Bifidobacterium_angulatum	-1.417348894	-1.422498623	0.058878292	-0.045780279	0.841214795	0.805685709	8.246199041	10.75351763	10.80647712	0.164875311	-0.482689968	-0.147040786
Clostridium_tyrobutyricum	-1.415450987	-1.772852206	-1.393747486	-3.6447984	-2.267536868	-0.927350111	-0.414187053	0.373189357	0.281574719	0.087144088	0.314763686	-0.243111463
Methylosinus_sp._C49	-1.415289697	-1.181985119	-0.134291866	NA	NA	NA	-1.102458233	0.08663947	-0.181532468	8.6794801	0	0
Candidatus_Frankia_datiscae	-1.411905228	-3.017032791	0.089925679	1.238983597	-1.359412347	-0.566758474	-0.491206502	-1.386429618	-0.179931598	0.940747014	1.13032056	0.213193095
Caldilinea_aerophila	-1.411716159	0.30846263	0.003903609	-0.549933973	2.656089875	3.478023682	10.19565518	10.65934484	8.451211112	0.30677718	0.316010244	0.548085417
Bacillus_atrophaeus	-1.41147968	-2.114497942	1.425767617	-0.919080005	0.971733029	-2.013939191	1.803811255	-8.04736934	-8.04736934	2.167611461	1.042836815	-1.66474811
Paenibacillus_sp._Cedars	-1.410837511	NA	-3.589174752	NA	NA	NA	0	0	0	9.367779474	0	0
Mageeibacillus_indolicus	-1.410580539	-3.485579259	-1.291423273	-0.692141707	0.008580133	0.378722791	-10.7998725	-4.117200895	-3.054736034	0.46415453	0.022882683	0.335199628
Pediococcus_damnosus	-1.408624215	-1.742234115	0.664643198	NA	NA	NA	-0.717266958	0.785710781	0.630829889	-2.695757081	-3.15001589	-0.380477362
Bordetella_hinzii	-1.408301168	NA	-0.036468167	NA	NA	2.128805488	0.487830722	1.697205393	1.270117093	-9.049848549	-1.45192264	2.23169323
Lactobacillus_helsingborgensis	-1.407041618	-4.983867904	-2.27528118	-1.315658318	-0.40549952	-1.12496962	0.001101973	-2.107422786	0.606823726	-0.662260091	-0.106722025	0.408341968
Paenibacillus_sp._JNUCC-31	-1.404721617	-3.097784115	-3.731185066	0.028333629	1.709757246	1.625826888	1.642899476	-0.118141606	0.248179915	-1.109714177	0.457710098	1.084827634
Aeromonas_hydrophila	-1.402403169	-1.865712144	-1.67487215	0.479314817	0.80248954	0.208638632	-0.69561121	-0.220582932	1.199999437	0.127301487	-0.320994667	-0.983397062
Curtobacterium_flaccumfaciens	-1.40198783	-4.646741896	-1.239508853	-1.305276054	-1.124680215	-1.419912164	-0.512008859	-0.230230925	-0.196355286	-1.725612724	-1.49224198	-0.70518815
Mycoplasma_orale	-1.401840197	0.710694245	-0.83482349	NA	NA	1.501546493	-1.85175254	-0.675921586	-1.037539351	1.873930775	-0.121641576	0.831824056
Clostridium_isatidis	-1.401602768	-0.73721083	0.018432781	-2.242984028	-0.161964098	0.398179551	0.377278144	-1.292177153	0.509839778	0.450530214	0.497455306	0.919824616
Thauera_aromatica	-1.396251481	NA	-0.142494889	-4.446770147	NA	-1.043158255	0.546645541	0.768762263	0.042881051	0.336016915	0.157136608	0.273194711
Schaalia_cardiffensis	-1.394810654	-2.762853975	-0.806547231	-0.353361136	1.262671052	0.873940573	0	0	0	0.468444944	1.024699572	0.448574286
Bartonella_bacilliformis	-1.392459651	-0.637682781	-4.588607973	-3.875871154	NA	NA	-0.604693619	-1.494676489	0.327193936	-0.798101938	0.626846582	-0.953286604
Tardibacter_chloracetimidivorans	-1.390490679	-0.848535757	-0.083571778	NA	NA	NA	0	8.559465045	0	1.004286288	1.731803889	-1.263034921
Caloranaerobacter_azorensis	-1.390022519	-1.703546598	-1.648784153	-1.313586171	0.226325495	0.500494758	10.26016683	10.75200424	7.745136469	0.431666133	0.294887869	0.144735432
Vagococcus_sp._HDW17B	-1.38881372	-0.835789301	-0.670338231	NA	NA	NA	-0.301129318	-0.55027654	0.530340889	-1.2326773	-1.495825286	0.238830778
Thermus_caldilimi	-1.385878347	-2.033032845	0.095238977	3.483978875	1.84679388	0.873278813	0.485175731	1.01990146	-0.356294808	0.600708305	1.472789939	1.261094295
Bacillus_infantis	-1.382279961	-1.438835007	-0.161697058	0.063681615	0.462822627	0.559848531	-0.367894374	-1.683926115	-1.005162608	0.789828759	0.861693504	-0.002175703
Bdellovibrio_sp._KM01	-1.378511623	-2.519867472	-3.153373825	0.887048228	-0.723790205	1.533788117	0	0	0	-0.802080694	-1.642727284	0.345068863
Corallococcus_macrosporus	-1.377291585	-2.244696665	0.519629601	0.595653931	-0.539749145	-0.9648054	0	0	0	-1.985416677	0.834515725	0.528741869
Bacteroides_coprosuis	-1.37520617	-0.251714085	-0.174899582	-2.944719919	-0.911816784	-0.746266652	-1.235711666	0.828344965	1.089537806	-0.086306498	-0.321894523	-0.296917729
Granulicella_mallensis	-1.370829343	0.171538935	0.114955407	NA	1.752576133	2.160989242	0.096271341	-9.552494273	-1.807357804	-0.299856094	0.584749665	0.651263445
Syntrophus_aciditrophicus	-1.369730111	-1.315314587	0.123225853	-0.980956082	1.20432677	2.218993203	0.06200978	0.53894258	1.348275641	0.473450414	1.099449109	0.509604824
Neisseria_elongata	-1.367394183	-0.994680062	0.374846777	1.102395189	2.39794139	1.532956121	-1.162186807	-0.034350459	-1.294044665	1.188353838	0.928977118	1.669539874
Ktedonosporobacter_rubrisoli	-1.366817846	-2.105234406	-0.115870897	2.100876612	1.622648877	-0.909989834	-8.909620116	-0.799194228	1.079575672	3.600979978	2.881801822	4.012585381
Listeria_ivanovii	-1.365129528	-3.259524317	-1.091697781	-2.870804454	-0.075204384	-1.179482716	-0.173533823	-0.795780663	0.819818458	-0.38808645	-0.291072842	-0.435088666
Nocardioides_sp._SB3-45	-1.363547184	-0.938690624	-5.210137269	NA	NA	NA	-2.042994396	-1.089690641	-0.508687932	0.931213823	0.625718792	-1.322962431
Phycisphaera_mikurensis	-1.360832658	-1.992090501	-1.432559874	-1.500316154	-2.8833875	-1.887847148	0.512534685	-0.050651666	0.682828072	0.812835605	0.092991245	-0.245621885
Planktomarina_temperata	-1.358978492	-0.79720375	-0.121389103	1.209844736	-0.26459492	0.533938498	-0.301129318	-9.177646265	-3.020529775	-0.110692525	-0.060101623	-0.533967199
Fictibacillus_arsenicus	-1.357816759	-2.443502324	-0.776668391	0.681465485	1.104820535	1.131235301	0.875310218	2.406161773	1.597245802	0.190380123	-0.081419958	0.746337333
Rhodomicrobium_vannielii	-1.357552005	1.663427934	1.464221977	0.363126085	0.74768644	1.861213371	0.016666816	-0.108828983	-0.031217888	0.376887592	2.232148853	0.717253515
Comamonas_thiooxydans	-1.357552005	1.84212034	0.864035117	NA	NA	NA	-1.474556309	1.011213188	0.88255659	0	10.49385545	11.63481105
Hyphomicrobium_sp._MC1	-1.357552005	NA	1.670353992	4.179499122	NA	NA	10.86812032	10.94929417	11.11397652	-2.47681308	1.461156739	-1.281164014
Microbacterium_sp._BH-3-3-3	-1.356884574	NA	-4.151528445	1.461496121	NA	-1.191637883	0.239232752	1.550513239	-1.480169726	-0.67276501	-2.402391537	-0.98517254
Streptomyces_lividans	-1.356750406	-4.768009393	-1.537920165	NA	-1.152003093	-0.710493383	0	0	6.15711649	-0.257349822	1.268432424	1.186002131
Stigmatella_aurantiaca	-1.354832203	-2.279899648	0.011978666	0.729951454	1.952502466	2.049546259	-0.063878811	0.048000684	0.856235135	-0.574222095	-0.171512868	-0.776472938
Nitratifractor_salsuginis	-1.354202076	0.379098246	0.255894661	0.689000592	3.130763463	3.339813429	0.399802858	1.891448949	1.879143016	0.444119853	0.422001447	0.456509425
Methylocystis_rosea	-1.353142702	-1.831785247	-1.041150536	-0.763283863	1.464851894	-0.123831433	0.745423663	-7.757316664	2.171941744	1.319020308	-0.037661005	0.553344861
Treponema_succinifaciens	-1.348790803	-1.780751374	0.137692042	0.817264888	-1.09671964	-0.495114115	0.101778437	-0.117927338	-0.549883194	0.159943702	0.205045255	-0.211022036
Comamonas_aquatica	-1.348728154	-0.856875058	NA	NA	2.136520761	1.346571833	-8.799135887	-8.799135887	-1.642021419	0.375717106	0.923741343	1.315974359
Cyanothece_sp._PCC_7425	-1.347808442	-1.662838666	0.448385266	1.690973477	-0.403402799	0.304298201	10.3781973	0	7.745136469	0.759156695	0.72056425	1.384257511
Nocardia_cyriacigeorgica	-1.346337053	-0.805213895	0.432783049	-3.555251195	-3.01241417	-0.65738398	-8.076815597	-8.076815597	-8.076815597	1.228110146	0.34013269	1.956410731
Pseudomonas_putida	-1.34324359	-3.667012151	-1.704205186	-0.995334316	-2.474531484	-1.360430128	9.248359308	6.682671608	0	-0.243969485	0.36853265	0.497719609
Bifidobacterium_scardovii	-1.338548885	-2.134275501	-0.33074229	0.651895645	-0.685963067	-0.494920769	-0.591034736	1.768745697	-1.600198862	0.66904146	-0.361171663	0.038781859
Geobacillus_kaustophilus	-1.337500899	NA	NA	2.152003093	NA	NA	-0.812559268	-0.275398214	-0.060776983	7.129283017	0	0
Ornithinimicrobium_pratense	-1.336782746	-3.002238472	-0.675198963	2.629427705	NA	1.693384961	-1.748377627	0.38436491	1.291514763	-0.167168503	-1.33795113	1.169508803
Streptacidiphilus_sp._P02-A3a	-1.335160351	-5.358016965	-0.43983671	NA	NA	NA	-0.722083693	0.161075551	-1.237371797	-0.432038254	-8.705055194	1.031909837
Kocuria_sp._TGY1120_3	-1.333423734	-0.78153863	-0.982297998	-0.825510282	NA	-0.022971304	0.08876141	0.447041435	-0.743289886	1.628988141	1.466876252	1.85812214
Olsenella_sp._GAM18	-1.332657385	0.064231125	0.44334905	0.009963317	-0.249151254	2.835892247	-0.611643702	0.117705393	0.292516922	0.338326657	0.676026134	1.810277804
Chloroherpeton_thalassium	-1.330628509	-2.39788123	-0.508520151	-1.316267015	-0.179294936	0.73798232	0	0	0	0.127960021	-0.806243081	-0.154853274
Pelobacter_carbinolicus	-1.330329837	-4.472535643	0.193153612	0.964912462	1.005436989	0.770575073	-9.624047875	0.003320026	-9.624047875	1.11514624	1.907796039	2.113148813
Roseobacter_ponti	-1.330266647	-1.834672794	-1.830258629	NA	-1.012149083	-0.477885278	8.502740328	10.21232615	11.38654693	-0.361650717	0.865921424	-1.22131888
Bacillus_pumilus	-1.32911092	-2.955199756	-2.115242078	-1.092421778	1.70725881	1.379663169	-8.799135887	-8.799135887	-1.642021419	0.157316089	0.23646213	0.9192892
Pedobacter_sp._KBS0701	-1.327649299	0.597847176	3.359253132	NA	-4.390965952	-3.540269896	3.195696655	-0.289119001	4.414926422	1.023222536	0.58138756	-0.077198906
Avibacterium_paragallinarum	-1.326762598	NA	-1.988383068	3.476077079	1.560153641	2.474517607	0.115025589	0.596569887	1.01351912	0.239670542	1.173880561	1.577408643
Candidatus_Vampirococcus_archaeovorus	-1.321350901	-2.578396145	1.266734057	1.157668737	0.08260884	0.121476632	-7.757316664	-7.757316664	-7.757316664	1.641739797	0.627863201	0.598794774
Carnobacterium_inhibens	-1.318893251	-0.831985549	-1.861275963	-1.68363041	-2.172370752	-0.987374694	0	0	10.55249619	1.42940426	1.37246606	1.419889708
Capnocytophaga_sputigena	-1.317501138	0.78140002	0.239984562	-3.424602855	-3.71026017	-1.089673759	0	0	0	0.766276663	0.423025088	0.795053832
Actinomadura_sp._NAK00032	-1.316896408	-1.510737916	0.177868284	-2.711953706	-1.208127968	-0.379544127	-0.132308968	1.067629953	0.601309758	0.4684191	1.026218888	-2.622388811
Glaciecola_amylolytica	-1.315789645	1.072730866	2.082884791	NA	-1.308358396	-2.910613682	0	0	0	1.460176115	2.408141882	1.72345675
Chania_multitudinisentens	-1.313815449	0.12845927	1.55513606	NA	-0.5299378	-0.894866451	8.876516947	8.110425888	8.598897393	0.817028927	1.283040343	2.533776575
Lactobacillus_sakei	-1.312738923	-4.120093845	0.630631748	-0.293066322	0.02596453	0.409497044	-0.265794037	0.241280767	0.325859281	0.888129934	0.497249107	0.118130935
Aerococcus_christensenii	-1.311524356	-2.478047297	-0.159713793	-0.724323744	0.222553693	-2.132469595	-9.346312052	2.941137733	0.385006979	0.609080153	0.117122787	0.102488716
Truepera_radiovictrix	-1.310628256	-1.385240179	0.424907334	0.42259485	NA	-1.511113471	-0.565808194	0.199782252	0.846061648	0.603687296	0.708972675	0.20535613
Cryobacterium_arcticum	-1.310557574	-3.701527313	-1.789929715	-1.935384311	-0.514012878	-0.969840111	0.100004222	1.197007794	1.652677988	-0.002284957	0.007844084	-0.795288662
Thiohalobacter_thiocyanaticus	-1.310177389	-6.287356428	0.065615465	0.974368674	0.334803912	0.106090131	-10.15135909	-10.15135909	-0.533974277	-0.294229363	-0.794064633	-0.021221596
Selenomonas_sp._oral_taxon_920	-1.309317225	-2.452223721	-0.932500774	-0.648473335	0.525295328	0.129996214	8.246199041	0	10.52516841	0.335369733	-0.368559238	0.152092634
Fusobacterium_pseudoperiodonticum	-1.306683139	-6.261494365	-1.063076369	-1.433445713	0.127555393	1.594194303	-0.096876248	-0.188835995	0.002162525	0.547759056	0.220565068	0.603710845
Kytococcus_sedentarius	-1.306615195	-1.198672503	0.19803723	-0.558915858	2.213544273	1.999729942	-1.310458092	-11.11693522	-11.11693522	0.653002827	1.439270258	2.441760884
Streptomyces_sp._RPA4-2	-1.305492321	-2.705869514	-0.871491418	NA	-0.979414228	NA	0.051467605	2.249575823	0.698017783	-1.657689859	-2.640135868	0.704878749
Tepiditoga_spiralis	-1.304696147	-2.274709271	-0.510464738	NA	0.586558252	0.480361009	0.008707738	-1.442068385	-0.075288505	0.080439198	-0.169966866	-0.069372241
Streptococcus_mutans	-1.304436385	-0.715790629	-0.480741596	-3.551272564	0.0767205	-1.016472858	0	0	0	0.086751102	0.450763018	-1.219805238
Novosphingopyxis_sp._pb63	-1.303113908	-1.535302923	-0.382450954	NA	-2.639378192	-0.307261653	8.876516947	9.384097309	8.48088308	1.666465285	1.606717568	2.726818178
Alcaligenes_aquatilis	-1.302890395	-5.612745657	-3.871698207	-4.856425529	-1.539012915	-2.992239384	-1.721786281	-1.084064265	0.462353641	-0.103879051	-0.063378782	0.397426227
Slackia_heliotrinireducens	-1.300879199	-2.068694069	-0.421381135	-0.010491699	-0.202125405	0.294093286	0.102321517	0.917726012	-0.583154362	0.3501868	0.214504512	0.126342714
Saccharothrix_syringae	-1.300650617	-5.463433549	-1.963764799	1.038551259	1.701566325	0.463067735	0.109750182	1.336727968	2.072518515	0.104158914	0.356950088	0.117294301
Staphylococcus_saprophyticus	-1.298923359	-1.13013086	-0.974145294	2.636535799	-1.521477825	-0.775609176	0	8.627369725	0	0.09949583	0.938556739	0.946444756
Bordetella_bronchiseptica	-1.298740336	-3.288686671	0.861963261	-0.411754915	-1.072444742	-1.218700039	0.011764674	0.071407293	-0.115857243	0.196332252	-1.078910569	-2.566061046
Prosthecochloris_sp._GSB1	-1.297784304	-1.117992493	0.978326937	-1.866176923	0.073989828	1.261043628	-2.009156561	-0.558945769	-0.004577313	0.0278695	0.03023583	-0.124499772
Selenomonas_sp._oral_taxon_478	-1.295929977	-1.223370409	0.298337693	0.285908872	-0.463105516	0.650675701	-0.206108525	1.919572396	2.366137108	1.267807312	0.834548072	1.375306934
Mycolicibacterium_tokaiense	-1.295455884	1.173446375	0.318800924	NA	2.233037598	3.321731261	0	0	8.30544163	-1.133835356	-0.704151856	0.083014628
Sphingomonas_sp._AP4-R1	-1.294061301	2.256411137	0.387925654	NA	NA	NA	-0.057051637	-0.283108743	0.351662317	8.366322214	7.29523629	9.678306113
Brucella_intermedia	-1.292988454	-1.122709776	-0.589102231	0.879946793	-2.435302146	-0.470717829	0	9.499120372	9.740548156	-0.901410002	-0.437955094	-0.644179343
Saccharomonospora_azurea	-1.292041219	0.889925609	-1.062887706	NA	-1.894621758	-0.791758067	-8.552496194	-8.552496194	-2.395379704	-2.62056535	0.23180296	-0.799592236
Actinoplanes_friuliensis	-1.289644161	-1.068700674	-0.801949539	4.760740228	1.57554847	3.267060704	0	6.682671608	10.95921229	1.666573131	-1.392773898	2.205681896
Alicyclobacillus_acidocaldarius	-1.289026893	-2.513907084	-0.058097434	2.76159395	0.939468913	0.149891418	-0.132689903	0.273574611	0.320951484	1.183589189	1.222222115	1.085574498
Lacticaseibacillus_paracasei	-1.287211889	-0.516345889	0.216119052	1.230617107	-1.35371868	0.095681381	0.407068617	-2.202809801	0.155565191	0.606145369	1.013841084	0.978093087
Thalassospira_indica	-1.284134933	-1.515045738	-0.143137117	0.539083277	1.098420245	-0.215717901	1.332575339	0.30531556	2.143236183	0.029920729	-1.122230878	0.427158166
Neisseria_zalophi	-1.282922715	-1.289506617	-0.179815065	-0.180807961	0.909857312	-0.618282849	0	0	0	-0.798820523	-0.042947118	0.121989842
Desulfosporosinus_meridiei	-1.282354233	-2.198738872	0.044945439	1.418800113	1.623034112	1.961438189	0	8.382131157	8.624046047	-0.067075429	-0.169807924	0.401742804
Treponema_pedis	-1.279518649	0.105823078	-0.519564831	-2.758162634	-1.524455814	-2.285379501	0.956959827	1.749553207	2.361302439	0.876528481	0.109802678	-0.220575177
Ureibacillus_thermosphaericus	-1.27886849	-1.342298096	-1.078346987	1.243474149	1.096077621	1.513866518	-0.685512419	1.027415181	1.123272351	1.261514733	0.298464525	1.057939428
Staphylococcus_sp._SDB_2975	-1.278791592	-0.617593505	-0.237450796	NA	NA	NA	-8.10567284	-8.10567284	-8.10567284	0	11.29523514	10.39802751
Bradyrhizobium_sp._CCBAU_051011	-1.277393811	-2.782388007	-0.899265577	NA	NA	NA	0.043623233	0.402703336	0.649808254	-7.544323092	-2.071835321	-7.544323092
Ruminiclostridium_sp._MA18	-1.277315128	-1.996186147	-0.7000871	-0.260618203	-0.872577165	0.278109952	1.142858878	1.926286024	1.000224461	1.045964908	0.726519718	-0.032865885
Streptococcus_intermedius	-1.276918755	-0.974426646	-1.006991294	-0.884700047	2.593872781	2.346190305	2.76414886	-3.679287921	-1.772906134	1.544899665	0.284890689	0.241556353

Oscillatoria_nigro-viridis	-1.27526048	-2.374494836	-0.968436886	0.930191527	1.777529067	1.777345856	-1.092770792	-1.912832489	-1.438391597	0.154972266	0.937135242	1.419671549
Halobacteroides_halobius	-1.270794477	-0.683972507	-0.975188245	NA	0.858968123	0.473505669	-0.742957562	-0.684953678	-1.094232462	1.678851687	1.630968331	1.794970682
Capnocytophaga_sp._oral_taxon_878	-1.270710446	-3.006614844	-0.736525681	NA	NA	NA	0	0	9.819597937	2.177655276	1.608481951	0.567603523
Oscillibacter_sp._PEA192	-1.268392374	-2.107135062	-0.958368372	0.075550134	-0.120275084	0.261859393	-9.372270887	-0.141268167	0.658892694	0.278045738	0.16925567	-0.118789228
Microlunatus_sp._Gsoil_973	-1.26326693	-0.606631151	-2.238368411	2.313029296	0.6923624	1.558050332	1.6759447	1.968070266	3.122746333	0.647576835	0.419086734	0.799667785
Planktothrix_agardhii	-1.258824613	0.077478753	0.774927969	-1.561241232	0.96268893	0.888145696	0	9.39192664	8.157119522	0.976118747	0.437863127	1.086662657
Planctomyces_sp._SH-PL14	-1.255944981	-1.067549376	-1.257157839	-0.058394164	-0.600304636	-0.464504767	3.058310081	3.178537602	3.911741286	0.918391726	0.560381847	1.182605421
Streptococcus_sp._Z15	-1.255718404	-3.429853823	-0.311243121	0.216332234	-3.975978848	-0.106619204	0.366016153	1.042323487	-0.006504064	0.219157165	-0.460944702	-0.139018392
Pseudarthrobacter_equi	-1.255607388	-1.905436998	-0.07196086	NA	NA	NA	2.662964177	1.858645726	1.888825854	8.333902227	9.491853096	10.23442228
Parabacteroides_distasonis	-1.254228051	-0.531522224	-0.478605588	1.099917056	-0.727208101	-0.378855763	-0.631864228	-0.305425172	-0.695993813	0.146733059	0.170997403	-0.070076394
Pseudonocardia_sp._Gen01	-1.25269897	-0.558117901	0.329789085	0.633163548	-0.445870672	0.200336735	0.455013754	-2.29378736	0.210642356	-0.769525902	-0.651800429	0.517832754
Fusobacterium_hwasookii	-1.251018136	-2.230964909	-2.619111852	-5.26873692	-3.097143098	-2.990287461	0.322437401	0.600429661	0.219117572	1.396496448	1.201673542	0.119942644
Magnetospirillum_magneticum	-1.249160604	-2.112759637	-0.160604686	2.444923325	2.06987221	0.630822739	0.762241866	0.64427394	1.363246276	0.119255571	0.431217929	0.201634117
Labrenzia_sp._PHM005	-1.246753704	-4.565500552	-4.350389888	-0.963207969	1.630377959	0.775831954	-10.76185038	-1.854959782	-0.840009441	0.821065124	1.281667168	2.456159288
Lactobacillus_pontis	-1.246240435	-2.951166698	0.751721956	-0.20557038	-0.120958152	1.034320073	-0.073771439	0.200914042	0.658173084	-0.465475231	0.525181272	-0.497690069
Haliangium_ochraceum	-1.245938999	-1.793242731	-1.199113674	1.073189381	0.268097573	0.645219985	0.474486004	-0.001285953	0.845627421	0.548656716	-0.631182029	0.07855562
Megasphaera_elsdenii	-1.245668594	-0.69217324	-0.564591029	-0.346110218	0.633357336	0.592214013	-0.488946635	-0.766358188	0.157228728	0.835231447	0.431738397	0.33752222
Tenacibaculum_mesophilum	-1.245379244	NA	-0.875319542	NA	-0.658837269	0.842941349	-10.60059127	-0.177759093	-10.60059127	0.278616835	-0.514394863	-0.505529451
Crinalium_epipsammum	-1.241438177	1.741688004	0.206766186	0.513339573	-0.625892765	0.558185795	-0.069663792	0.141812732	-0.893974974	10.24237623	11.09319966	9.586214923
Polynucleobacter_asymbioticus	-1.240292947	-2.573988273	1.840641743	-0.623425314	0.436682493	2.045867579	-8.552496194	0.006968851	-8.552496194	0.832026934	1.194692085	1.335560956
Maricaulis_maris	-1.239217044	-0.058520369	-1.001793282	NA	0.547352519	0.917588233	0	0	6.15711649	0.252490962	-0.093896753	0.524318819
Erwinia_sp._J780	-1.236733444	-3.891984389	-0.128413702	NA	-0.463330236	-0.814912882	-2.653560263	-1.025680954	-1.833131064	1.004936808	1.102325981	0.569641492
Finegoldia_magna	-1.23372639	-1.302672858	-0.892469552	-1.368040448	-0.829679884	-0.361590043	0.009041717	0.217224387	0.450721957	0.538971695	0.579244893	0.507066555
Lactobacillus_kunkeei	-1.231587083	-2.477372323	-0.85241596	NA	0.256740558	1.279541588	-0.481102695	-0.746228546	0.298245442	-1.225499653	0.199035452	-0.372612641
Flavobacterium_album	-1.230312882	-0.461620791	0.456444591	-2.993926383	1.246189899	0.761087135	0.801630248	-0.20961168	1.425549859	-0.295901616	0.919877595	0.849248773
Shimwellia_blattae	-1.227153454	-1.273762663	-0.232242331	1.017437692	0.815293955	0.883447412	-2.124686776	-2.017432923	-1.088168883	-2.525847794	-0.423486076	-10.42066556
Agromyces_flavus	-1.218640286	-0.148731457	-1.797461722	NA	NA	1.032789935	0.680903809	0.681163055	1.176563427	-1.35310975	-9.247927513	-9.247927513
Leptospira_santarosai	-1.217771797	-1.934993268	-0.520446092	-1.65790795	1.0620989	2.061948815	-2.179688883	-3.368793308	-4.455278317	-0.756286541	-0.454874	-0.419034309
Paracoccus_sanguinis	-1.216725453	-2.5561796	-0.833146972	-0.411897792	NA	-1.017073513	-1.694081614	-2.303170626	0.578884949	0.924365013	1.957070529	0.851629991
Endomicrobium_proavitum	-1.214597328	-0.005590678	-1.192595452	1.92533735	0.831534024	0.828520082	0.215739087	0.523416513	0.869240248	2.28104362	-1.028572759	2.229949773
Azoarcus_olearius	-1.214052727	0.194023566	-0.036217874	-3.204352412	3.254477861	3.271439015	-1.030206172	0.905582719	1.298682378	-0.559544066	0.385604495	-0.203323566
Pseudomonas_entomophila	-1.206156119	-2.482285838	-1.798733603	-1.232018212	-2.007189913	-0.867917421	-0.491109581	0.955534945	1.027045843	-0.562500879	-0.613247623	-0.03077012
Listeria_sp._PSOL-1	-1.205788663	-1.850308008	-1.799353482	-1.281924676	-3.24827002	-0.951877017	-0.968969252	0.363017951	-0.126521075	0.780183642	1.254804613	1.022883447
Brevirhabdus_pacifica	-1.203942669	-2.651755205	-1.226148464	NA	NA	NA	-1.062283	1.355181032	1.860078463	-7.544323092	1.671615115	-0.613585755
Cellulomonas_sp._H30R-01	-1.202896246	NA	NA	NA	-0.739900885	0.085391491	-0.332605011	0.11251546	1.452792089	8.45395786	7.996236198	6.544320001
Sulfurospirillum_sp._UCH001	-1.202772082	-2.173243194	-2.039517371	-3.637915606	-1.134642974	-2.882680726	1.580904181	1.458631495	-0.015563917	-0.258477254	-0.04446127	-0.698257003
Clostridium_baratii	-1.199480437	-1.07312985	-0.457456299	0.578094883	1.540077986	1.470670263	-0.160969718	1.285529886	1.312653305	1.078640466	1.047338117	0.814883448
Cedecea_neteri	-1.199122642	-1.869033657	-1.011009491	-5.058769847	-0.127525072	0.304058202	-1.049630165	-11.36334084	-1.327167232	-0.24632209	0.415496796	-0.078379255
Sphingomonas_sp._NIC1	-1.199117476	NA	-1.975666351	NA	NA	NA	0.183817825	0.308059595	0.431860404	-1.071327116	-9.489179631	-1.854971191
Olsenella_sp._LZLJ-2	-1.198943549	0.164728318	0.53120221	-0.297032778	-0.275414528	2.955307955	0.793638988	0.798489767	0.846562332	0.335108018	0.633591831	1.970239916
Paenibacillus_alvei	-1.196818696	-2.715513995	-1.60913375	0.223583255	0.893298608	-1.028383847	-2.597870778	-0.324940128	-0.46607958	0.597165978	0.882200955	-0.484252212
Ferrovibrio_terrae	-1.196775752	-1.97166623	0.332336775	-0.407073074	2.033148632	2.188323388	0.334866997	0.453268411	1.074076881	1.209663295	1.190097701	0.571503776
Mucilaginibacter_sp._14171R-50	-1.187971457	-1.445769214	1.077163248	NA	NA	-0.120521284	0	0	0	-0.532998452	0.531823711	0.407973602
Sphingomonas_paucimobilis	-1.187075367	-2.131187784	0.619202355	2.393206786	0.844533051	1.43103607	0.263529706	-0.187249462	1.233918655	1.338345174	1.737402708	-1.698619283
Cyanobium_gracile	-1.186387555	-2.415828667	-0.793242661	-1.977402191	-1.057148141	-0.043259273	1.076236503	0.569433114	1.328624179	1.330539255	1.645000566	1.281236653
Bradyrhizobium_oligotrophicum	-1.183247697	-2.820346402	-1.382064186	-0.114987388	2.003816267	-2.306723223	-2.106666054	1.539377081	2.67552297	1.503879638	-0.210718144	3.100565118
Mycolicibacterium_sediminis	-1.181574919	-1.991526953	0.351560704	-0.216852304	0.735299402	0.843026304	-1.68979527	-9.846914793	1.175765108	0.434577889	0.925041197	0.074488713
Thermus_aquaticus	-1.1805017	-0.839906678	-0.78113927	-0.122416756	0.398032983	-1.628602143	9.315767728	0	7.745136469	1.228102212	0.422586491	-0.025289263
Stanieria_cyanosphaera	-1.180461741	-1.094414	-1.926220453	-3.030170918	-2.009560638	1.238366875	0.486943787	0.754611221	0.528881055	1.825125783	2.088880427	2.096602288
Lactobacillus_paralimentarius	-1.180077718	-1.054292145	-5.34298971	-0.949398939	-1.539975046	-1.719422822	-0.080775438	0.533201234	0.285707763	-0.670678756	-0.805011172	0.785624888
Alkalilimnicola_ehrlichii	-1.179189427	-1.13118656	0.084888898	-1.322351732	-0.172097456	0.447910719	-10.41324711	-10.41324711	-4.256130619	-0.066183982	-0.610743723	-0.716188979
Thermaerobacter_marianensis	-1.178200555	-1.608672308	-0.719225586	1.629360773	0.103504576	1.070669921	0	9.617384813	7.745136469	0.830728311	0.784874368	0.971315268
Bifidobacterium_eulemuris	-1.175301726	0.404896061	0.356498122	2.615597012	0.013943485	-0.35324626	0	0	11.84566646	1.248402221	2.205061661	1.289095031
Frankia_alni	-1.172824892	NA	-0.346928725	-0.800966594	-0.275465966	-1.019336485	-0.947007037	-0.071778322	-0.180341274	-0.182860308	0.424420112	-0.951926731
Georgenia_sp._Z294	-1.171941347	-2.837397073	-0.523170216	0.753045956	-0.733880814	1.497885618	0	0	0	-0.280900827	1.47581822	0.748023536
Lactobacillus_koreensis	-1.170867367	-1.456294837	0.290289414	-2.271167936	1.14395336	-0.572792455	-0.274894956	0.310339406	-0.339933928	1.213672121	1.081362642	0.234511669
Clostridium_intestinale	-1.169440355	-1.652630777	-0.827264896	-3.142828171	-0.201726338	0.952502835	-1.567579842	-0.420880198	-0.57916913	0.008335105	-0.138240182	-0.133911451
Deinococcus_metallilatus	-1.166581558	-0.926903478	1.402007973	1.711534178	0.348867922	1.174572154	0	0	0	0.977686811	1.794995657	1.440337904
Cutibacterium_acnes	-1.16510676	0.44302144	0.427453889	2.155948792	2.603133006	2.884081355	0	10.42283218	8.904155902	0.208232296	-0.116252692	-0.214006489
Treponema_caldarium	-1.164411397	0.487665299	1.656120988	-1.013939191	-2.411415366	-1.097613517	-1.644293741	-1.711407364	-1.052065958	0.149617732	0.145024793	0.231450059
Sulfitobacter_sp._BSw21498	-1.162774548	-3.59708606	-0.058123627	4.287781726	2.754658157	2.720306652	-1.092963678	-9.222246695	-1.477110226	0.994526312	2.060563373	1.959879147
Bacillus_sp._1NLA3E	-1.161258016	-2.497541404	1.262383757	3.012028194	1.755914099	0.488544354	-0.031581736	-1.984900199	-0.675039275	-0.803861176	0.632649136	-0.266057369
Arcobacter_skirrowii	-1.161049667	-3.081578285	-1.473314685	NA	-1.646731858	0.918535332	-0.073362487	0.223192922	-0.84735009	-0.973944822	0.163976713	-0.441197043
Brachybacterium_sp._P6-10-X1	-1.160703557	-5.137882636	-0.926541039	0.250363345	-0.292019367	1.401915229	-0.144297794	0.246387267	0.555403194	1.016615575	1.175113495	0.723392251
Roseitalea_porphyridii	-1.159349166	-0.747847067	0.662061352	NA	-2.035552243	0.449034832	-0.951657168	-0.500951935	-1.037676324	1.216681332	0.810868297	0.750864435
Bacteroides_thetaiotaomicron	-1.15670254	-1.147049947	-2.04076222	-0.633714837	-0.063390873	-0.370567658	-7.757316664	-7.757316664	2.273846916	-0.303537373	-0.475669996	-0.42905098
Roseiflexus_sp._RS-1	-1.155692853	-2.061660123	1.471142601	0.797307863	2.594408425	0.339017165	0.311742139	1.154213907	1.475006611	1.488610414	1.907088891	2.235925986
Bacillus_amyloliquefaciens	-1.153363575	-0.835602912	0.869442016	-1.597110002	-3.222011249	-1.692696777	-1.516500217	-9.925891153	0.98202099	-0.116320393	0.154439192	0.098988405
Clostridium_botulinum	-1.150394648	-1.5800486	-0.881565081	-0.151921082	0.475339089	0.483281157	0.647701779	-0.520741542	0.996489305	0.587990453	0.443068994	0.633008452
Streptomyces_sp._YIM_121038	-1.149878862	-0.440665244	-0.705637454	NA	0.925410684	2.3670676	-10.17311494	0.210978052	0.035899704	0.689010937	-7.700439718	-7.700439718
Tepidiforma_bonchosmolovskayae	-1.148496428	-2.408131294	-0.92043585	-1.07521281	-0.408899482	0.21632903	-0.745056279	-1.084781189	-1.422201307	0.23167413	-0.483449919	-0.31585942
Zymomonas_mobilis	-1.147293431	-1.400684901	-0.089467125	0.716935401	0.13951685	-0.670416617	-0.346600515	0.218330188	0.661835357	0.058451715	0.696556191	-0.71794664
Rhodoferax_koreense	-1.147153694	1.614816905	1.651877965	2.821438695	1.976591779	0.69805628	0.982544529	-8.04736934	1.057111477	-2.069099446	0.828728573	0.590693174
Hyphomonas_neptunium	-1.146874092	0.158699654	0.484968901	NA	0.494205375	1.61356942	-1.620221375	-0.614546575	-1.030998518	0.456128533	1.756881094	1.331669988
Persicimonas_caeni	-1.144730198	-0.802071068	1.083691606	1.535406315	2.756059166	2.263680991	0.003913885	-0.009739476	-0.036034913	-0.330959934	-0.036220172	0.486580307
Bermanella_marisrubri	-1.139551352	NA	2.181249196	2.602400193	NA	-2.704318808	9.288762279	0	11.69617643	0.819998304	-1.965453227	-0.766299648
Aeromonas_veronii	-1.138997195	-2.281093456	-0.566795956	0.979470249	0.622030287	1.03019935	0.201728017	-0.500731227	0.440034658	-0.252217801	0.060619918	0.80564514
Oxalobacter_formigenes	-1.138565236	-1.992946994	-0.654341586	-1.086932768	-0.528467808	-1.068232463	-0.354645853	-0.544883665	-1.029894074	0.766754578	0.709381293	1.06176821
Arcobacter_anaerophilus	-1.133954457	NA	-0.6116815	NA	-2.552541023	0.840963315	0.41737127	0.454497577	-0.334191964	2.296956692	3.685650071	4.141768905
Hahella_sp._KA22	-1.133954457	NA	2.83251592	1.64716539	0.593285149	0.852057528	-0.504928845	0.545299428	-0.322174596	0.673528062	1.999180203	2.71232544
Microbacterium_foliorum	-1.128298833	-1.701023758	-1.867839431	0.339192746	0.440203946	-1.000700507	0.711575068	0.981673109	0.884619203	-0.366903648	0.480405734	0.803186158
Enterococcus_saigonensis	-1.125745271	-1.559245176	-0.03132612	1.79056023	0.366175951	0.33477295	-0.70556279	0.373891678	0.769472018	0.380376028	0.298628335	-0.140305542
Rothia_dentocariosa	-1.125538082	-0.345470454	1.276840205	0.750021747	0.627108488	3.1099287	0.837517453	1.910376717	-0.183661889	1.376150389	-1.681544553	1.35752139
Hahella_chejuensis	-1.125387409	-2.204322317	-0.272737539	-0.778747844	-0.921196023	0.064077776	1.230772462	1.413190258	2.587411038	0.677514798	-0.230260824	0.662823042
Planococcus_dechangensis	-1.122798617	-1.871202884	-0.18314689	NA	-1.098723983	-0.466290453	-0.094769175	-1.208646965	-0.332135266	0.98236552	0.443290733	0.141967508
Clostridium_bornimense	-1.121643596	-1.105330663	-3.095558827	-0.794722855	1.177182719	1.22506485	0.189911562	3.370429333	3.787871083	0.47228357	0.617291848	-0.081550838
Brachybacterium_avium	-1.118941073	-2.212050477	0.487155641	3.43460528	2.402677731	0.892901554	0.397067487	0.663715434	4.014801578	-1.095068257	0.160743335	0.522059839
Chryseobacterium_sp._3008163	-1.116760076	-1.72471579	-0.183315745	NA	-0.808819073	-1.114276972	0	10.38409299	6.15711649	-0.440620419	-0.545427658	-0.267055486
Bacillus_mycoides	-1.115865689	-1.568269148	-0.818574315	0.695421486	0.54470601	-0.652831222	0.556594459	3.356223267	1.576678535	0.621436104	0.193182068	0.852288401
Thiospirochaeta_perfilievii	-1.115513097	-1.839682628	-2.154436945	-1.087232505	0.039624653	-0.146240398	0	0	0	0.246999483	0.922895806	0.210021653
Sinorhizobium_medicae	-1.104902183	-1.932561202	0.594848075	0.155081665	NA	1.289594167	0.350130289	0.431047439	1.108698497	-0.612183969	1.595412194	1.254772037
Thermobacillus_composti	-1.104697379	-0.520894417	-0.384619239	0.853267402	2.123193546	1.522000671	-1.811363867	-9.940646884	-9.940646884	0.325024569	0.604040077	-0.424609423
Peribacillus_simplex	-1.103163069	-3.093109404	-0.512159966	3.614583475	1.542018861	3.523831141	0.022764145	0.043953405	-0.07697609	-0.46375782	-0.859970054	1.978532816
Zunongwangia_profunda	-1.100962962	-0.582834686	0.106411616	-1.674547514	2.522322248	2.78113761	-1.242999036	0.341965059	0.127259956	0.820941115	0.573054846	-0.458878489
Bacteroides_heparinolyticus	-1.099199398	0.007630116	-0.62105484	-2.852442812	-2.737700725	-2.597659244	-0.06142799	0.057972303	0.568291451	-0.084414411	-0.255803963	-0.308839926
Thermodesulfobium_acidiphilum	-1.098459136	-0.757209838	-0.902538926	NA	0.397335498	-1.297937148	0.209802271	0.654164517	1.467403931	1.258135603	0.504612706	-2.424689757
Candidatus_Syntrophocurvum_alkaliphilum	-1.098152831	-2.339395328	-1.489960083	-2.967346783	0.823528387	1.43440717	9.292949655	0	9.819597937	0.08210192	0.38290885	1.36891795
Treponema_brennaborense	-1.09662366	0.323845609	1.23079852	-0.165514526	2.528940915	2.688555647	-2.414322694	-1.554035821	0.200820514	1.048921222	1.588492806	1.405693821
Intestinibaculum_porci	-1.095697745	-1.172946353	-0.089317556	1.050459969	0.159268166	0.427597508	-0.369188341	0.217558412	0.823048386	0.357550647	0.707256012	0.188768809
Polaromonas_naphthalenivorans	-1.095583006	-1.047201656	0.60601092	-0.042125476	0.53658965	1.231998109	-0.433828267	-0.724782585	0.12148665	-2.252199004	0.26326986	-0.550720232
Granulicella_tundricola	-1.094487374	-2.409985895	-0.111816129	0.373509016	1.409323043	-1.939260842	-0.325144061	0.463652613	0.34512852	0.371373278	-0.028309975	-1.49058411
Pseudomonas_stutzeri	-1.092836031	-1.931575913	-0.316040126	1.432685135	1.564109764	1.731954996	0.51663184	-8.799135887	0.652075224	0.747737495	0.61124044	0.265604548
Pedobacter_sp._CJ43	-1.090800924	-2.789653433	-1.187942082	NA	0.524425941	1.613791467	-0.210348091	0.097132833	0.142269332	1.247912217	0.886305253	0.240771743
Prochlorococcus_marinus	-1.089132188	-1.412731564	-1.394227958	-1.366320787	0.020771612	0.127577458	1.557499311	1.357181637	1.17250358	0.679462465	1.309827134	1.070708085
Spiroplasma_eriocheiris	-1.086385935	-3.013529593	-1.195361424	-0.58340283	0.767231954	-1.273842334	-0.562401784	-1.442609252	-0.028635627	-0.943822963	-1.882502953	-1.389551359
Olsenella_umbonata	-1.080357572	-0.485848114	0.062943792	-0.792568173	-0.236144688	2.816201051	0.439905281	1.955636606	1.83348269	0.33968661	0.717783296	1.892468592
Geobacillus_stearothermophilus	-1.075828412	-1.40757529	-0.330546077	2.531865738	0.711492364	2.098335983	-0.0348554	1.132293954	1.168574213	-0.141699735	-0.908595487	0.075261269
Oscillibacter_sp._NSJ-62	-1.07415906	-2.242548244	-0.857892697	0.846034556	-0.106156952	0.119990518	0.138121463	-9.177646265	-0.458723015	0.366546418	0.175358038	-0.02322318
Carnobacterium_maltaromaticum	-1.072728785	-2.083696695	-0.280279179	-0.420448227	1.196919325	0.1799548	-0.40877129	0.236221213	-0.20551532	0.025245059	1.303036263	1.010462179
Spirosoma_sp._KUDC1026	-1.072394537	-0.94105358	-0.10889198	-0.277315558	1.456526642	1.1612413	8.129283017	11.01287591	8.71892325	1.124258054	1.111770596	1.018178698
Antarcticibacterium_flavum	-1.071221929	-0.59828352	-1.152799111	0.739894925	-0.404002208	NA	-0.054589247	0.862836452	0.805154853	0.690220969	-0.649654643	0.477219549

Geobacillus_sp._46C-IIa	-1.071216799	-0.252484486	0.508928686	1.5941014	1.578977072	-1.45246834	9.71268347	0	9.466123378	-0.646083864	-0.433539229	-0.88086001
Tetragenococcus_koreensis	-1.070426827	-1.256112853	-1.281549668	0.375303631	1.254984776	0.22629955	0	0	8.624046047	0.517755802	0.093852517	0.22304638
Dolosigranulum_pigrum	-1.0704241	-0.751761401	1.17276836	-1.910705062	0.217620185	-0.074091375	-1.031150386	-1.3105557	-0.506151696	-0.019082561	0.553343955	1.31196856
Collimonas_arenae	-1.069211533	-1.305902772	-0.523063874	NA	-1.537269768	-0.662280291	-0.66985287	0.579061413	-0.493694257	-0.734053429	0.528668568	0.883078648
Chryseobacterium_sp._T16E-39	-1.068947354	0.954072202	1.064882852	NA	NA	-2.204413378	10.79323751	10.46889891	6.15711649	-0.245404792	0.905444385	-2.02893741
Glutamicibacter_creatinolyticus	-1.068556558	-0.535601765	-0.280607035	1.463630536	-0.264993993	1.026594981	0.045869366	-0.921550713	-0.606970385	-0.071781117	1.141766982	0.106407084
Plesiomonas_shigelloides	-1.066911157	-1.323705908	-0.16708505	2.806313202	1.458934528	0.024095676	0.770844106	1.48474392	2.434542397	0.376596203	0.623701343	0.889942051
Campylobacter_showae	-1.064451466	-2.176051104	-0.427224493	-3.59049415	-0.961861178	-0.170668412	0.333871261	0.896156584	0.76845826	1.146742596	1.953010005	1.514260275
Alkalihalobacillus_clausii	-1.064398507	-1.387143247	0.781015817	0.496119836	1.05267909	1.100633455	-0.222812114	1.117010319	0.739434945	1.383875996	1.780141318	1.580553122
Kozakia_baliensis	-1.063098341	0.477177595	-0.534821857	-0.838177846	0.826331454	0.649194828	-0.467177233	0.751133141	-0.724528027	0.805190252	0.325707162	1.028297622
Caulobacter_sp._K31	-1.062860024	NA	0.445469395	1.239811875	0.555250167	1.794453715	0.820896131	0.210056478	2.466241013	0.588182493	0.696289049	0.698847768
Immundisolibacter_cernigliae	-1.06012682	-2.39007309	-0.287286595	-2.793884282	0.66383986	0.13922497	0	8.110425888	9.561202011	1.210308521	1.305767608	0.484558382
Costertonia_aggregata	-1.059984322	-3.70208578	-0.26140587	2.585821759	-0.028646511	2.69417814	1.247309018	0.27125389	-1.089919998	-0.7957186	-0.506488137	0.255340967
Acetobacter_persici	-1.05878582	-3.630327805	-0.309602457	-3.482500467	-2.798684579	-4.295622332	-0.144899157	-0.506175951	0.237612916	-0.891283131	-0.513741736	-2.299517257
Luteipulveratus_mongoliensis	-1.049582029	-5.026761068	-2.46529227	NA	NA	0.705193248	0	8.559465045	11.36831001	-0.159740408	-1.885898197	-0.534063283
Yersinia_mollaretii	-1.044699807	-1.854073897	-0.447014455	NA	2.032510038	0.957534992	-0.466228103	0.461736056	-0.186120184	9.873444113	10.98156728	8.587466258
Tetragenococcus_halophilus	-1.04348271	-0.356919752	0.500882496	-0.979894741	-0.72640508	0.455208216	0.233652571	-0.663134284	1.021148823	0.628208502	0.974623291	1.11595804
Celeribacter_indicus	-1.042252487	0.132309096	-1.089870507	-0.79655439	2.276522484	1.502598446	-0.28162782	0.485635782	0.763689823	-0.052151282	1.301102725	-9.985841937
Salinispora_arenicola	-1.042162412	NA	-0.202174142	NA	NA	NA	1.433139486	1.303351055	2.378141654	1.412231434	-2.273468846	0.443867941
Agrobacterium_rhizogenes	-1.040574743	-0.587317922	-0.581319181	2.758791957	2.598388447	1.597807301	-1.545968369	-0.492755063	-3.351578685	-9.125844794	0.14406031	1.069736574
Staphylococcus_cohnii	-1.039511985	-0.579518529	-1.35258907	2.4018271	1.505385319	-0.050660453	-0.810282424	-0.819531733	-0.128242933	0.650078035	1.874787493	1.790146741
Thermosynechococcus_sp._CL-1	-1.038664099	NA	-1.911415518	0.519633912	-0.033026991	0.307593424	0.251625787	-0.671169469	0.158350961	2.520297529	0.561783388	2.042850733
Bacillus_marisflavi	-1.034740728	-0.863257985	-1.050474895	-0.89653184	-0.14153307	-0.45175926	1.110395973	-0.74646859	-0.845052669	0.189161446	0.365507242	0.637869163
Clostridium_novyi	-1.034463264	-1.087189567	-0.799763925	-0.755795318	-0.158202697	0.613540386	1.870985513	1.675127413	3.142551327	0.132551778	0.241023512	0.288719053
Lelliottia_amnigena	-1.033810939	-1.687843833	-0.561287945	2.023651268	0.134610176	1.161510179	0.564592934	1.382495889	0.087807324	1.167786091	1.331979043	0.558354721
Nakamurella_sp._s14-144	-1.033087388	-2.856517073	0.056959035	NA	-2.356934545	0.225917209	-8.04736934	0.512095705	-8.04736934	0.60694087	-1.128986441	-1.2255597
Selenomonas_sp._oral_taxon_126	-1.032369017	-1.812654526	-1.420021794	-1.606384513	0.244441521	0.212813701	8.502740328	8.627369725	7.745136469	1.014131999	0.767345994	1.400478197
Clostridium_sp._BNL1100	-1.031954341	-1.877863533	-0.506225409	-1.026190412	0.787938416	0.654205473	0.109750182	-8.04736934	0.403841772	1.235497338	0.954310792	1.056750931
Devosia_sp._S02	-1.03058832	-2.178018683	0.261099427	6.321022775	1.622895525	0.839929625	-0.624276096	-0.180072301	-0.175527412	0.868135423	0.087708843	0.709747283
Carnobacterium_divergens	-1.029560291	-2.132033554	0.006102159	-0.545925123	1.184712851	-0.331219859	0.111867894	-0.14931268	0.412775234	1.230223888	0.246673984	0.921613623
Lachnoclostridium_phytofermentans	-1.028657663	-2.17348416	-1.047155097	-0.060605354	0.30233833	-0.009081018	1.916263911	0.211388413	0.849508373	0.558443382	0.185962117	0.129566881
Magnetospirillum_gryphiswaldense	-1.02393466	-1.332915319	1.458328256	1.798880377	3.294093219	2.372862733	-0.599785779	-0.223943867	-0.113687697	2.311004677	2.155977743	0.241554238
Anaerotignum_propionicum	-1.02349642	-2.246603935	-1.383377806	-0.563834691	-0.281643188	-0.56310352	-10.15135909	0.749371892	-3.994242601	0.154890867	0.708330108	0.267481654
Caproiciproducens_sp._NJN-50	-1.020977081	-1.952011642	-0.691817719	0.887622597	0.287359635	0.564358285	0	0	0	0.218624081	0.214580728	-0.192402106
Eubacterium_callanderi	-1.02082811	-1.436928237	-0.321764509	0.394454621	-0.646599315	-0.411488151	-1.506879095	-1.50354262	0.34722474	0.370826626	0.005842007	0.065660981
Acetobacter_sp._KACC_21233	-1.020012227	-1.260198043	-1.888052638	5.123433167	2.19583421	2.770624906	-10.09251931	-0.708422006	0.201469543	-0.090354738	0.327269908	1.877892586
Janthinobacterium_sp._Marseille	-1.01768392	-1.278643997	-0.605243532	NA	1.310046472	1.020896712	-2.207822587	0.992498259	-1.611385288	-1.796912328	0.645190732	0.957437504
Chelativorans_sp._BNC1	-1.012981652	-0.733921822	0.831866616	NA	NA	0.255051948	-0.690683355	-0.882965322	0.641095734	0.758191165	0.719494019	1.374918763
Desulfococcus_multivorans	-1.012481035	-1.136798222	-0.572023687	0.309983721	-0.395640931	0.645029597	-9.929931509	-0.503285162	-0.452725741	0.042417953	-0.005457043	0.54097363
Brevibacillus_brevis	-1.009725308	-1.444872505	0.002027259	2.201380947	0.326373876	0.504893388	-0.84367887	-0.258937676	-0.377875737	1.175398652	0.354186045	0.110785291
Natranaerobius_thermophilus	-1.005081707	0.167623512	0.049856206	-1.583611979	-2.210953082	-0.534837374	9.330101211	8.110425888	7.745136469	0.550471861	0.909152724	0.184168438
Bacillus_velezensis	-1.004600209	-0.986181751	-0.474480869	2.96437328	1.214210094	1.018543606	0	0	9.540215237	0.665463428	1.182570135	1.335920969
Geosporobacter_ferrireducens	-1.003818104	-1.700326398	-0.786606361	0.636877634	0.772466282	0.654763537	-8.091313724	1.335332623	-0.934199256	0.316089611	0.582251106	0.204924974
Olsenella_uli	-1.003106395	-0.64015078	0.01475375	-0.404449561	0.020576117	2.744507143	-1.92854051	-2.309599407	-1.575957491	0.462314591	0.716257648	1.893028553
Vitreoscilla_filiformis	-1.003049217	-2.74019982	-0.955018618	2.708951218	0.792147387	0.956026085	-1.627041321	-0.095873028	-4.07388863	0.28602669	0.33929989	0.590134356
Bacillus_anthracis	-1.001985816	-0.154827372	-0.583970617	2.89877795	-0.401616984	0.554990421	0	0	0	-0.342895031	0.359627068	-1.89308922
Tessaracoccus_aquimaris	-0.997539322	-1.314068654	-0.036412214	NA	-0.040404426	0.86422778	-0.858219243	0.762816204	-0.558550407	-0.178725676	-0.920783987	-1.221194664
Aneurinibacillus_soli	-0.99753148	-2.000772279	-0.960851462	2.646090294	-0.762753026	0.169763377	-0.286117074	-0.364119035	-0.357193058	1.121160473	-0.182940649	0.767059191
Mycolicibacterium_fluoranthenivorans	-0.994725058	-2.12254061	-2.156572239	-2.19356658	0.440603445	-0.868424161	-0.668613215	-0.92789891	-1.40640057	-0.735712676	-1.855260011	0.383975443
Erysipelothrix_rhusiopathiae	-0.994723176	-2.107718815	-0.110639392	-2.996961905	-0.632352254	-0.618032582	0.88444306	0.86534687	0.592555678	-0.04469088	0.457206082	0.235689013
Fusobacterium_necrophorum	-0.992729558	-0.812527077	-0.918523513	-1.162739989	0.342580238	-0.602771867	-0.525244617	-0.75708218	-0.051386532	0.843655594	1.438443021	0.540384356
Salinicoccus_halodurans	-0.992163506	0.855486015	-1.203914429	-1	0.043144492	1.578938713	0.143887759	0.865197872	-0.65511164	3.067898286	2.457137002	0.976379825
Desulfitobacterium_metallireducens	-0.991656766	-2.913120237	-0.262619063	2.534180263	0.797651048	0.377454408	0.192335421	0.37691587	0.09351909	-0.128866336	0.481926987	-0.134134328
Anaerocolumna_sp._CBA3638	-0.990412922	-2.389345617	-1.094528926	-0.681810404	0.494981322	0.411732616	-1.233311718	0.860814318	0.495635396	0.381482235	0.314603673	0.591271207
Sulfitobacter_sp._AM1-D1	-0.99034983	1.033496912	-0.839790153	-1.251965537	-0.513293621	NA	-9.076815597	-2.394143989	-2.919699107	-7.709655951	0.647896054	-1.16533595
Octadecabacter_sp._SW4	-0.988684687	0.527015152	3.613086102	5.332767931	2.476910218	2.379596182	1.691875469	2.60052877	1.953922526	-0.913972925	0.341215517	1.038735875
Streptococcus_sp._I-P16	-0.988684687	NA	0.789392443	5.086595007	0.97566155	-1.494033535	0.813314545	0.4408839	0.641742994	1.666264774	1.736307317	2.658439108
Rhodococcus_hoagii	-0.987842386	NA	-0.379529398	0.922893826	0.131365603	1.217773866	0	0	0	1.290485377	1.163572433	0.767731847
Butyrivibrio_hungatei	-0.987747792	-1.94654463	-0.998096708	0.446360211	0.31836148	0.26141431	-0.381948342	0.8724701	0.224356692	0.310224358	0.061795653	0.333292983
Clostridium_chauvoei	-0.984692747	-1.215728691	-0.222445855	-1.641906748	0.566581007	0.896532683	0.127989384	-0.305603759	0.966668854	0.223347518	0.573337669	0.353886644
Corynebacterium_minutissimum	-0.983227433	-2.329293671	-1.391467348	-2.174732845	2.002397927	2.294236361	-1.405817366	-0.935567163	-0.938890841	-1.914029758	1.703845304	1.957053151
Clostridium_sp._JN-9	-0.982718313	-1.367181078	-0.760965848	-0.330223067	1.07490524	1.43318462	0	9.384097309	11.81159546	0.964667829	0.766887876	0.381169479
Caulobacter_mirabilis	-0.981681971	0.109424642	-0.450285934	3.03103775	NA	1.387743151	-0.358295718	-0.224756561	0.182564507	-0.201051112	0.396524814	-0.321923444
Iodobacter_sp._H11R3	-0.977316563	-3.374156703	-0.133654247	-1.171012597	1.511235279	0.346861613	0	10.07438234	0	1.415143379	0.52835762	0.80853539
Sphingobacterium_hotanense	-0.977210135	-1.943072932	0.097449777	-5.450772017	-3.82928364	-3.77750999	1.146718187	1.173882028	1.232881473	-0.161884456	0.80993532	0.921707065
Pigmentiphaga_aceris	-0.974984033	-3.785935206	-1.206383945	NA	0	0	0	0	0	-0.174237106	-8.890771943	-1.351613132
Bdellovibrio_bacteriovorus	-0.97403902	-2.083176431	-1.367278424	2.868770215	2.307985295	2.183966107	0	8.627369725	0	-0.242613186	1.207292842	1.734621233
Rubrobacter_sp._SCSIO_52915	-0.972006941	-2.261948498	-0.514652353	1.366708269	0.838918518	0.454805592	0	0	6.15711649	0.390500848	0.549208161	-0.135264374
Rubrobacter_indicoceani	-0.968516443	-1.385164273	-0.619042021	-1.784694325	-2.021089605	-1.222868323	0.677827959	-0.128122228	1.165023964	-0.031112726	0.0285247	-0.662371246
Clostridium_beijerinckii	-0.968306883	-1.93486344	-0.749146445	-1.087663047	1.190426967	1.184776865	0.395194171	0.381187171	0.525711404	0.659932885	0.05364582	-0.10962339
Simiduia_agarivorans	-0.967938791	-1.517664111	0.180393447	1.290541017	-3.942994569	-1.931440343	0.849137077	0.612322438	1.599817226	-0.334117504	-0.103007245	-1.11297346
Corynebacterium_argentoratense	-0.966654852	-2.43786301	-0.278651726	1.353124816	-0.463608717	1.149350809	-0.356699566	0.683260775	0.454808664	0.720616028	1.550808668	1.249277972
Pseudonocardia_sp._CGMCC_4.1532	-0.965928995	-1.822527063	-0.552907841	-1.68182404	-1.074638801	0.195535286	0	0	0	-0.336058871	0.473759168	1.016022281
Anaerostipes_hadrus	-0.965397625	-2.454718137	-1.708880521	-0.315678591	0.094636874	-0.05760628	-0.962567628	0.93123761	0.315010827	0.230552742	-0.032659828	0.38539387
Flavisolibacter_sp._17J28-1	-0.964431023	-0.149736524	0.155002913	-1.875572741	0.773318192	0.664465337	-0.005610779	0.874371562	1.017774702	-0.252134098	-0.054233611	0.270631909
Staphylococcus_condimenti	-0.963723064	0.009922524	-1.568236404	-0.181577959	-0.444612364	-1.567621148	8.246199041	8.382131157	6.15711649	0.381483082	1.14851651	0.877288222
Desulfomicrobium_baculatum	-0.963627321	-1.902475757	-0.47264358	0.328353755	-0.557546398	0.1849342	0.094634466	-0.069941199	-0.558483209	1.051823875	1.243724472	0.056486964
Microbacterium_sediminis	-0.96074057	-1.688055994	1.891352139	NA	NA	NA	-0.378403414	-0.113405455	-1.454191688	0	0	0
Bacillus_licheniformis	-0.958825557	-1.866298539	0.650986965	1.277107521	-0.710978	-1.235456327	0	0	0	0.558997955	0.432595608	-0.01777856
Thermoanaerobacterium_aotearoense	-0.958565934	-1.192426378	-1.113377349	-6.861313905	0.343137954	0.454369542	-1.548372794	-0.041836556	-0.480529573	1.201352267	1.316930809	1.056736181
Mycolicibacterium_litorale	-0.955815073	-2.619896291	-1.0617007	NA	3.181860499	-0.352588648	-10.26657574	1.686034478	-2.10945622	-0.170184585	1.341801735	0.871434504
Spirosoma_sp._PL0136	-0.953438154	1.532408195	1.043974895	NA	-3.303566605	-5.570957416	-2.981504268	-1.249603595	-1.396543452	0.302524018	-0.080947003	0.73764997
Wolbachia_endosymbiont_of_Dirofilaria_(Dirofilaria)_immitis	-0.952828796	-1.60807974	0.229189057	NA	NA	NA	9.029913869	9.426646347	0	-1.449705478	0.297886893	0.436455724
Fusobacterium_nucleatum	-0.952511642	-0.384430423	1.19926074	-3.603284386	-0.794855367	-0.256531262	0.39875013	1.379981249	1.298367496	-0.019911524	0.401077316	0.657761351
Enterococcus_avium	-0.952264982	-1.4774973	-0.249439529	0.988271983	-0.16518671	-0.177411656	0.391861056	0.969991608	1.713591035	0.509258529	0.630141069	0.823013495
Ligilactobacillus_agilis	-0.949590695	-1.754304662	0.238322824	4.93269198	-0.540297489	-2.067214061	1.772238549	0.453792205	-8.10567284	0.201071002	-0.474287328	-1.143444037
Clostridium_fermenticellae	-0.943876313	-0.998849889	-1.213306191	-1.853897508	-0.555085861	0.415999969	-8.552496194	-0.170365038	-8.552496194	0.543120935	0.962212729	1.188500293
Deinococcus_deserti	-0.943416472	-1.411195433	0.479418718	-1.173915862	1.058149566	1.408713529	-0.084147883	0.349010358	0.426306338	0.594538076	0.413712449	-0.797160845
Lacibacter_sp._S13-6-6	-0.94333722	-1.028089676	0.123101398	-5.000352177	-0.017358603	1.211333363	-0.931447224	0.206451044	1.521691937	0.918171933	0.867788069	1.143141614
Diaphorobacter_sp._HDW4B	-0.943020383	-0.045590117	0.765664569	NA	NA	NA	0.317992865	-0.243315669	-1.279887728	-10.3383687	-2.189978554	-0.8082882
Sulfurovum_sp._NBC37-1	-0.942621411	-1.915697657	-0.34630707	1.319149339	2.368113987	0.444484416	-8.552496194	0.825701106	1.418529966	0.614455262	0.310315657	1.491681597
Eubacterium_maltosivorans	-0.941641583	-2.228927165	0.053211701	0.049747083	0.123489998	-0.179557059	-1.396184021	-0.768119723	-0.11951898	1.572488272	1.05681841	0.380864753
Spiroplasma_chrysopicola	-0.941504351	NA	NA	NA	NA	-1.036525876	-0.312028032	-0.74646859	-1.228470219	9.398031787	7.712410067	0
Arthrobacter_sp._PAMC_25486	-0.937807467	-3.913065393	-1.040526302	2.220528875	2.592507071	1.612066642	0.234573825	0.157919956	0.981211433	2.047824338	0.729317385	1.955901848
Cryobacterium_soli	-0.936147374	-2.901931659	-1.669722379	2.90473168	0.47139413	0.381619765	-0.844670369	0.233528117	-1.161865834	-0.558624454	-0.955668585	0.006687834
Alteromonas_sp._RKMC-009	-0.934887371	0.903122782	-1.555795836	1.75765411	NA	1.22274259	-1.201708902	0.866472796	0.80256879	7.129283017	10.50713061	10.39088905
Thermacetogenium_phaeum	-0.933021837	-2.182274485	0.558883201	0.718491761	0.402467404	0.805258454	0.803792581	2.209390947	3.713964684	-0.09276512	0.130683306	0.526981738
Spirosoma_sp._KCTC_42546	-0.93191939	0.637936218	2.005012946	-0.08933663	0.720194452	2.092854956	0	0	0	2.405816932	3.862096693	4.514345723
Pseudomonas_fluorescens	-0.930917222	-1.581384529	-0.009317761	1.311115555	0.900721928	1.388005801	-1.725114936	0.05893855	-0.50368582	-0.778068725	-0.289160598	0.332290531
Anaplasma_ovis	-0.930737338	3.39351823	1.974148122	NA	NA	NA	0.915820161	0.340308924	1.900561994	0.441812562	0.339942392	-1.41642404
Diaphorobacter_ruginosibacter	-0.929422809	-2.789189492	0.422743258	NA	-1.298109475	1.519453945	0.726726526	1.360474271	1.01660825	-0.580372934	1.627223229	0.55713059
Streptomyces_sp._S1D4-11	-0.925999419	0.167109395	-0.64422845	-0.657045515	-0.175142711	NA	-0.998422806	0.211059669	0.784592975	-0.980103344	-3.183321952	-2.048424375
Desulfotomaculum_nigrificans	-0.920004862	-2.662340916	-0.209723491	3.601809814	0.726391985	0.884607445	0.069135789	-2.657583539	-0.034815797	0.458483887	1.331748369	0.742149974
Thermaerobacter_sp._FW80	-0.919093127	0.169733767	0.175078776	0.752363779	1.613539038	1.556961049	-0.409503589	0.793402707	0.217066941	1.007744553	1.234156746	1.093044383
Corynebacterium_maris	-0.918863237	-1.06586565	1.247550945	NA	-0.121094239	NA	-2.936419007	-0.953879008	-0.503891844	0.791671446	1.068760212	0.252788395
Hydrogenophaga_sp._PAMC20947	-0.918326214	-1.212423385	-2.488923212	0.319811154	-0.222568714	0.916728788	-9.222246695	1.72506149	0.215788271	1.078908302	-0.044730745	0.860253544
Paraburkholderia_caribensis	-0.918012528	1.568854834	-0.678712771	NA	NA	NA	-0.782094992	-0.738329887	-0.276017624	1.799019076	1.072331317	2.093948968
Chryseobacterium_sp._IHB_B_17019	-0.917389078	-1.896052616	-0.682944898	NA	0.068616612	1.638389233	0.370183385	-0.525328764	-0.510164871	-1.217017564	0.684423249	0.068512058
Lactobacillus_curvatus	-0.916463207	-1.075019394	0.346232975	2.256552086	1.202030806	3.721358814	-0.139123417	0.715638857	-0.393752284	-0.153103389	0.884294122	-1.108922241
Citrobacter_freundii	-0.916003515	4.354009345	1.274913414	-2.782388813	-3.089109754	-0.468409472	-0.850687697	-1.400617622	0.754045242	-0.002744075	0.934935024	1.304289512
Enterobacter_asburiae	-0.915859702	3.09077775	-0.214689098	-1.444075204	NA	0.511055313	1.083978462	0.440899813	0.449420462	0.655129224	-0.9460115	-3.560714561

Desulfallas_gibsoniae	-0.910105718	-1.94965359	-0.599988753	1.628460991	0.711554716	0.629353179	-0.307574238	1.484834671	1.347421217	0.671790275	0.405643739	0.700575545
Paenibacillus_sp._RUD330	-0.91004173	1.251817882	1.388642621	-0.548951262	3.064089157	3.091169447	-1.215151364	-9.372270887	2.261301091	0.099524953	0.945617154	-0.294945371
Mucilaginibacter_sp._PAMC_26640	-0.903084217	-1.208122598	1.796574188	4.340142522	4.830564961	4.79143661	0	0	0	0.410169482	0.177237779	0.28757659
Xanthomonas_euvesicatoria	-0.902112639	-1.093887327	0.398093536	-1.100790594	-0.228481702	0.241732295	0	0	10.24132777	0.032075113	0.698232696	0.791602415
Parashewanella_spongiae	-0.901146613	-0.647698256	1.40676437	NA	NA	-2.807354922	0.438179993	0.889080529	0.859125993	1.132808077	-0.249086802	-7.544323092
Streptomyces_cyaneochromogenes	-0.901146613	0.209176802	-0.418452164	NA	NA	NA	0	0	0	0.572577222	-7.700439718	0.820504981
Streptomyces_sp._2323.1	-0.901146613	NA	1.441084095	0.899306	NA	1.572299265	-9.482717218	0.681272023	1.031730559	-1.960402109	-2.59986473	-2.480009159
Cupriavidus_taiwanensis	-0.900327129	-3.298307901	0.420832494	2.278433946	-0.781216369	0.742825378	9.409390936	8.110425888	11.98823377	0.184056865	-0.213085366	0.231915606
Chryseobacterium_sp._G0201	-0.897676177	-1.513019637	0.078303372	NA	1.85045465	1.044684798	0.081824772	-0.185866269	-0.235885976	-0.246234902	0.381052419	0.436242958
Hungateiclostridium_clariflavum	-0.897584314	-1.586818655	-0.894660212	-1.144537982	-0.856805541	-0.993375993	0.174120148	0.144573626	0.046955996	0.86997372	0.715212689	0.654895341
Citrobacter_werkmanii	-0.894368675	3.344970448	0.317501598	NA	-0.081427599	-0.6794801	0.477417675	2.154707615	1.696937295	8.45395786	8.809501266	0
Marinovum_algicola	-0.892134054	-3.098193632	-0.695089475	-1.573883055	-0.281584055	0.35460377	0.555176007	-1.881412257	1.489530297	0.650247018	1.94574764	0.611170852
Thermobifida_fusca	-0.891498545	-3.428281811	1.079129764	1.851123491	0.452280018	0.625045453	0.510929159	1.474562806	0.546065866	0.730206548	-0.43097264	1.518630175
Methylobacterium_sp._17Sr1-43	-0.890683306	2.099535674	-2.702333709	NA	NA	NA	0	0	0	0	0	0
Thermaerobacter_sp._PB12/4term	-0.890380162	-1.507947665	-0.11106438	0.636017591	0.610290948	0.113771713	-0.181438178	0.333609647	-0.035194804	0.254896283	1.039302157	-0.118539933
Olsenella_timonensis	-0.889909448	-0.048159285	0.589150458	0.073332154	0.027590159	3.137918862	-11.03929607	-1.159991585	-0.917050447	0.612853866	0.972606945	2.094792733
Neisseria_sp._ZJ785	-0.888916359	-1.677660939	0.854433324	0.862804467	1.491604746	1.65318984	-0.816615968	-1.879879838	-3.350135095	0.031946842	0.112275445	-0.696673578
Aerococcus_urinae	-0.886276624	-1.426705073	-0.773630869	-0.70523628	-1.567194931	0.232514893	0.924238128	-0.995244334	0.947889172	0.435498865	-0.522737929	-0.033191619
Saccharomonospora_sp._31sw	-0.884033982	NA	-1.676801368	0.476186155	NA	NA	0	0	6.15711649	8.442943496	0	0
Bacillus_thermoamylovorans	-0.881480834	-0.064398245	0.65572502	-1.233790215	0.018881021	1.383987082	-1.958228037	-0.987519923	0.145244155	-0.013399966	0.05204089	-0.185129013
Ammonifex_degensii	-0.880709825	-2.370937537	-0.80745833	1.196942611	0.481932504	-0.049560049	-0.445249373	0.710908992	0.777190439	-0.044775075	-0.285399923	-0.52171879
Candidatus_Koribacter_versatilis	-0.880288519	-1.084440786	-0.173880014	-4.60439879	-1.918481995	-4.858444412	-0.228142935	-0.21234838	-0.214260092	-0.215334674	-0.026712687	0.138412545
Desulfovibrio_carbinoliphilus	-0.878532313	-1.709816013	0.020096979	1.122472105	0.424608795	0.236685206	0	0	0	0.297253616	0.153629841	0.344598471
Hydrogenophaga_sp._NH-16	-0.877752679	-1.382366735	-0.904700598	2.931534445	0.469236166	0.004173699	-1.588698353	-0.1642176	-1.243435013	0.494590381	0.254100541	0.803488873
Deinococcus_radiodurans	-0.876569934	-2.577810384	-1.187312144	1.930360169	0.40183196	0.889477978	2.840961083	2.864398973	3.685070832	2.859402644	3.277984827	2.234849609
Mycobacterium_gordonae	-0.875799364	-2.416582416	-0.764942966	-2.089360796	-2.274017107	0.337733605	-2.768764872	-0.330253083	-3.180984889	-3.049366077	0.293114855	0.58004957
Lactobacillus_allii	-0.866733469	-2.469996787	-1.411053985	-4.240267784	NA	-2.454436575	-1.843107216	0.957062278	0.252747526	-0.09430488	0.417327017	0.047379627
Acetobacterium_woodii	-0.866226101	-2.235452032	-0.827668194	-0.577626788	0.862897354	0.985335552	-0.417043666	-0.775143075	0.465269384	0.383759754	0.440206931	0.439687052
Corynebacterium_endometrii	-0.862923752	NA	-0.56930501	NA	NA	NA	0.265663252	0.049436122	-1.209993553	0	5.472487771	0
Peptoclostridium_acidaminophilum	-0.859997354	-1.634166483	-0.520181337	-0.864162603	1.055645794	1.065551139	9.029913869	0	8.451211112	0.764402256	0.631354566	0.599586426
Actinomadura_amylolytica	-0.859189321	-3.250159061	-0.358101435	1.242791186	1.094992724	1.107964095	-0.247132196	0.2468668	0.279789171	0.381084968	1.328622148	1.340970886
Lichenihabitans_psoromatis	-0.856056676	-0.74524989	0.227550026	1.434262067	NA	NA	8.129283017	11.09191797	9.069511529	-0.947195166	-9.247927513	0.254572164
Herbinix_luporum	-0.855222155	-2.42023644	-1.158337027	0.044327531	0.185774607	-0.031487125	-0.314515465	-0.018539574	0.325274523	0.483726968	0.100347087	0.27820779
Brevibacillus_laterosporus	-0.85455348	-1.796849711	0.561544931	-3.91134366	-0.921560056	-0.252517938	-1.20488969	-0.767772311	2.373225253	0.082307403	0.65252249	0.934088028
Mycolicibacterium_duvalii	-0.85431068	0.830626141	0.053591978	2.940183715	1.288737006	0.321928095	-9.769386621	-0.259431538	0.540192048	2.320820727	1.767245394	2.291141277
Ilyobacter_polytropus	-0.85246065	-1.153323769	0.082008984	-0.710333029	0.886264725	1.256193235	0	6.682671608	0	0.093740067	0.565433336	1.187269168
Crassaminicella_sp._SY095	-0.848837095	-1.881957792	-0.544500748	-1.765225473	-0.255302409	0.034531076	0.846647135	3.420893772	3.77007237	1.058772863	1.224674226	0.396679102
Erysipelothrix_sp._HDW6C	-0.847531078	-0.76372047	0.829673301	-1.6405352	-1.113015475	-0.947866278	-0.135940036	-0.538851129	0.70338191	1.067508258	0.778641676	0.812685229
Streptococcus_australis	-0.844386475	0.527300656	-0.862197751	1.100521406	1.201930641	2.054426714	-0.098758518	0.09987835	0.910418457	1.785088372	-2.295880704	0.471783138
Cellulomonas_sp._40-2	-0.842816667	-1.097535377	1.346217157	-1.989095224	-1.097348114	0.116992035	10.50815131	9.352343634	11.66783892	1.037494828	1.184107616	1.734382127
Eubacterium_sp._NSJ-61	-0.840530075	-1.933096971	-0.915450732	-0.512683808	-0.154468233	0.205849698	0	0	0	0.23533738	0.02443632	0.225633382
Tenacibaculum_maritimum	-0.840320896	-3.170710569	-0.443857987	0.524612845	NA	0.374165218	0	0	0	-0.262032304	-0.619661673	0.147373141
Shewanella_algae	-0.837872689	-1.391005709	-0.397831184	0.548269876	-0.13018324	1.876916152	-11.00906579	-0.273890585	-1.124895269	0.366769425	0.665543597	-0.605277943
Pseudopedobacter_saltans	-0.837159882	-1.302538802	-0.444027076	0.433441084	1.951621715	1.577686407	0.515254206	-2.485931614	0.229396331	0.701611863	0.443975306	0.890562933
Paenibacillus_sp._UniB2	-0.837101094	-0.514717751	-0.061095837	-0.127620972	1.442539246	1.324457742	-1.248499093	-9.751239421	1.006833852	1.244214801	1.363085559	1.252949614
Phycicoccus_sp._HDW14	-0.835613182	NA	1.411924887	NA	NA	NA	9.752760297	7.68267863	13.67497565	-7.807354922	-7.807354922	-7.807354922
Lactobacillus_curieae	-0.8344821	0.780433623	0.960424488	NA	-1.489827527	-0.36871081	0.008624638	1.601856422	1.272160711	-0.168665898	-3.070966521	-1.084160957
Intestinimonas_butyriciproducens	-0.833795749	-2.173672939	-0.570298986	1.167972628	-0.227024567	-0.044178029	0	9.964472278	6.15711649	0.472437201	0.224857516	0.027072132
Ornithinimicrobium_flavum	-0.832217272	1.596626027	-1.164269584	NA	NA	1.011495639	0.247474324	1.345056061	1.969148788	1.85916558	0.415039109	0.60486347
Echinicola_sp._LN3S3	-0.830940002	-5.468691917	-0.264229454	0.588223883	0.745615373	-0.506665426	0.183465709	-0.016575757	0.821657489	-0.822494057	-0.835435065	-0.541716088
Staphylococcus_simulans	-0.830702015	0.170106988	-3.486992856	2.343898069	-0.053538107	-1.359507313	-0.085279083	0.799825262	0.752406349	0.694392468	1.926331999	2.61026903
Tepidanaerobacter_acetatoxydans	-0.829557642	-1.324117153	-1.067161237	1.49780685	1.66341467	1.591059599	1.39898165	0.408953139	1.230883473	1.619126392	1.448845684	1.492951016
Paenibacillus_swuensis	-0.82845001	-1.95601709	-0.904502326	-0.168110502	0.275406026	-1.194606605	8.876516947	9.384097309	0	0.330500987	-0.524403351	-0.650829138
Pseudomonas_mucidolens	-0.827833048	NA	0.218219533	NA	NA	-0.348563221	0.036522146	-0.944370945	0.774206984	0	10.22367595	7.539158811
Streptomyces_glaucescens	-0.826539598	NA	0.696583648	NA	1.216631383	NA	8.129283017	6.682671608	0	1.500868226	-0.478050517	-0.769702381
Streptomyces_sp._CRPJ-33	-0.826463745	-2.14839184	-0.569587275	NA	NA	NA	-0.064349421	1.310259264	1.507899252	7.894817763	0	8.412218392
Mycolicibacterium_poriferae	-0.826132129	0.159304664	-2.832445903	1.236476194	0.275365208	-1.286757317	8.876516947	9.648765614	0	8.878562894	10.30910114	10.21916852
Janthinobacterium_lividum	-0.820268241	NA	-2.751320887	NA	-0.17513836	NA	-10.44745522	0.513733984	1.296154835	-0.641504475	-2.622054517	-1.082892485
Proteus_columbae	-0.817346694	NA	-0.614018534	NA	NA	NA	0.190102883	0.833759576	1.117039421	0	0	0
Shinella_sp._HZN7	-0.817346694	NA	0.429348975	-0.124258103	-0.748391201	0.43503299	1.221553472	2.125999261	0.193233833	-0.082660056	0.187660724	-0.574890969
Zobellia_galactanivorans	-0.816324576	-1.05864366	1.709312658	0.372358916	-3.030082227	-1.354063184	-0.578381153	-0.070670557	-0.252409852	-0.335783298	0.083356704	0.681277631
Lacinutrix_sp._5H-3-7-4	-0.815461311	-1.148449959	-1.197044579	NA	NA	NA	9.029913869	6.682671608	9.052320788	-0.674236309	-1.633024709	-3.490983519
Thermovibrio_ammonificans	-0.813543573	-1.160990743	1.58788562	0.41190461	1.07272214	1.755312432	-0.652651978	0.160846216	-0.324475486	2.666613756	3.648458105	3.707577133
Paeniclostridium_sordellii	-0.811577181	-1.286537288	-0.788375655	-1.416063233	0.905954085	0.591146029	-2.1101115	0.684599896	0.996537268	0.42103303	0.329499869	0.471920928
Anoxybacillus_flavithermus	-0.807245465	NA	-2.368650136	1.311445251	1.332659337	0.876544725	-2.347009137	-0.582893183	-0.655631951	0.791030322	-0.074044464	0.386340726
Mobiluncus_curtisii	-0.80702757	-1.231002938	-0.153539812	NA	-0.298327736	1.060976078	0	0	9.324999567	1.421674129	1.256013337	1.014462466
Streptomyces_sp._NA04227	-0.803602787	-0.462034979	0.701506406	-2.942861482	0.776172867	0.816445658	0	9.965782846	0	-0.427570821	-0.332638655	-0.843139128
Brevefilum_fermentans	-0.802245904	-2.719093245	-0.082901425	1.304095993	-0.073698098	1.524311473	1.564814222	0.961075606	1.34809474	0.265065202	1.796448367	1.540493564
Novibacillus_thermophilus	-0.799482748	-1.402313955	-1.908800868	1.739652069	0.961794409	1.008451323	0	6.682671608	11.8329612	-1.031237866	-0.039373751	-0.323420392
Bacteroides_cellulosilyticus	-0.799053278	0.27402453	-0.008939637	-3.091646973	-1.227775676	-1.379592885	1.542699234	0.44832747	1.944584533	-0.055814854	-0.221030372	-0.24046198
Rhizobium_daejeonense	-0.798341824	-1.482261183	-0.540720914	0.746784655	0.738372415	3.380540255	0	0	6.15711649	1.109231096	2.990345057	-1.364448903
Clostridium_sp._AWRP	-0.798309782	-1.547348209	-0.781724322	1.514905068	0.155787023	0.051998936	9.550746785	0	6.15711649	0.6309425	0.987040488	1.275371228
Vibrio_cyclitrophicus	-0.797274612	-4.440120865	-0.516501243	-1.106695465	0.619308404	-0.360836622	-9.72047685	-1.093107124	-2.563362381	2.20003839	1.966128967	0.915862852
Paenibacillus_sp._FSL_H7-0357	-0.791708559	-1.589806635	-1.43208803	-1.749751352	-1.762105942	-1.718503958	-8.10567284	-8.10567284	-1.94855635	-0.168040723	-0.192398398	-0.382751477
Desulfofarcimen_acetoxidans	-0.791614983	-2.366534572	-0.910557542	-0.306486004	-0.105562716	-1.270126119	0.177269955	-0.382042004	-1.248894633	0.385487714	-0.199441728	-0.975927652
Brenneria_goodwinii	-0.790830353	NA	0.623702619	2.990776937	0.423499078	1.108934372	0.591194454	-9.588714636	-9.588714636	-0.675493361	1.106357909	-0.470875045
Pyruvatibacter_mobilis	-0.790622762	-2.495625311	-0.702042586	-0.889005526	-0.223885122	-0.743278641	9.163991757	0	0	0.266518144	0.560911026	0.320285357
Tissierellia_sp._JN-28	-0.789515475	-1.461908605	-0.730737239	0.74946623	0.827177302	0.877805123	-1.445906081	-10.72203049	-0.911166486	-0.129288265	0.353822322	0.09033423
Actinobacillus_succinogenes	-0.7855725	-4.762751539	-1.334123889	3.033308676	-0.189652968	-1.362427457	-0.078888235	-0.468500728	0.240078906	-0.065310656	-0.090041385	-0.48429518
Marinobacter_sp._CP1	-0.785464112	NA	-1.055915989	2.686295536	2.327547167	0.413277041	0.371966353	2.729056873	2.39634936	-0.325297376	-0.148030708	2.52550641
Polynucleobacter_difficilis	-0.784401481	-2.962420164	-3.133315659	1.018338643	-0.49255661	1.348951826	-0.074873986	0.427723794	0.534604354	0.409430707	1.232312915	1.5787667
Erythrobacter_sp._KY5	-0.782643169	NA	-1.370534385	NA	NA	NA	-1.295261932	-2.190904042	-0.398094294	0	0	0
Clostridium_butyricum	-0.780801195	-2.077945947	-0.543590329	-1.335259994	0.248411819	0.434291264	0	0	0	0.369495713	0.460192826	0.481803098
Corynebacterium_matruchotii	-0.779613475	-2.52867337	-0.46302369	-0.676302812	-0.566678321	0.116301154	0.045492139	-1.079435065	0.856308736	2.196094977	0.703133116	0.457155759
Epibacterium_mobile	-0.778781297	0.429338582	-1.59182269	-1.378118036	-0.070586162	1.936115667	-0.233261525	-0.661211861	1.132373494	3.616026152	0.969824149	3.74806863
Cyanobium_sp._NS01	-0.775379478	-1.962582471	0.895798052	-1.685592855	1.140573803	0.138415465	-0.334099727	0.210168403	-0.94522168	0.673927576	2.398001944	0.978221956
Collinsella_aerofaciens	-0.774128501	-1.315891104	-0.233923091	-0.332477751	0.05107726	1.481480335	0	0	6.15711649	0.52971034	0.39670643	1.285571994
Candidatus_Promineofilum_breve	-0.772495622	0.533175195	0.881083332	2.050411244	2.076593903	2.039310461	1.405862303	0.628619386	0.742276168	-0.71866142	0.475328047	-0.001704561
Deinococcus_proteolyticus	-0.771080914	-1.937666994	-0.637497759	2.026172575	1.518894165	0.898405826	0	0	0	-0.663747598	0.98812965	0.910710467
Desulfovibrio_sp._IOR2	-0.768857227	-2.310885109	-0.468464519	-1.166851406	-1.070908196	-0.650768852	-0.816861153	-0.712967112	-0.34136741	1.054085784	0.774192093	0.674760256
Massilia_sp._NR_4-1	-0.767314021	-2.159001033	-0.085149361	3.302454693	1.580056532	2.520558794	0	0	0	-0.631363994	0.017544983	0.714131364
Desulfosporosinus_acidiphilus	-0.76631745	-1.257512956	-0.252193158	0.978723122	-0.312187342	0.318453054	-8.076815597	1.307281711	2.495628843	0.034753524	0.685179279	0.276051868
[Clostridium]_innocuum	-0.762599967	-2.081548123	-0.991704481	0.0908584	0.287002606	0.23863137	0.31980981	0.394384547	1.406513573	0.345368189	0.01415869	0.091085136
Mycobacterium_xenopi	-0.76044039	NA	-1.682973659	-1.036899583	-1.293966265	-0.432959407	0.037969293	2.570464374	-0.934199256	0.958763809	0.066414806	-8.849664685
Staphylococcus_arlettae	-0.759414235	-1.570885266	0.121813615	0.106827686	-0.891182586	-1.500638189	-0.872845051	0.911847022	-0.927417661	2.73462487	2.062350143	2.147143065
Thermodesulfobacterium_sp._TA1	-0.759080756	NA	-1.806777498	NA	NA	NA	-0.213543658	-0.270223901	0.432297305	-1.411221932	-1.496018498	-0.076539057
Nocardioides_dokdonensis	-0.758877849	-0.620462237	0.513534889	NA	-0.445601479	0.846671118	9.315767728	0	0	0.645446943	1.339660178	2.746679243
Arcanobacterium_sp._2701	-0.756442969	-1.938323103	4.055257499	NA	-0.130969394	NA	-0.194378412	0.81401694	-2.819537022	0.47555331	-0.831878822	-7.890768904
Desulfocapsa_sulfexigens	-0.752625341	-1.769351711	-0.234762733	-0.327025963	0.896603566	0.524715183	9.315767728	0	6.15711649	0.984645407	1.629455958	1.121322037
Brachybacterium_saurashtrense	-0.752527574	-1.361411896	0.060222848	2.701468273	0.248229196	0.705135709	-0.216329653	0.020585239	0.335571798	-0.722391328	1.031582957	0.673856301
Clostridiales_bacterium_Choco116	-0.752002003	-2.402606144	-1.00154526	1.563787199	1.37492535	1.813058012	0	11.42378299	9.550746785	0.403758188	0.158679546	0.05809115
Kosakonia_arachidis	-0.750717207	2.265447612	0.741948446	-3.318848702	NA	-2.893084796	-1.035346256	-1.039645438	0.007429171	0.100401284	-0.172854339	0.228506439
Pseudomonas_graminis	-0.750279947	-1.484064294	3.430590838	0.360870337	1.743829146	-1.399370903	-0.105127545	-0.150934679	-0.238021711	1.380627029	2.560639425	-0.799059089
Leptotrichia_hongkongensis	-0.748211167	-0.542986029	0.548930838	NA	NA	NA	0	0	0	0.972581904	1.425563434	1.981012654
Actinomyces_sp._299	-0.748060422	-1.668127227	0.36403823	3.577203176	1.032467081	1.552797525	0	0	0	0.002527342	0.81922917	-0.293064243
Xanthomonas_campestris	-0.747715204	-1.784182729	-0.529133486	0.919829651	-0.246033325	-1.151065974	0	0	0	-0.48911538	-1.4185496	-1.708167817
Deinococcus_sp._AJ005	-0.747213397	1.098154134	0.747097622	-0.33604156	1.662044563	1.123898214	-0.295154435	1.009890594	2.12612459	0.0840422	0.152042659	0.780124543
Streptomyces_griseorubiginosus	-0.74579884	NA	NA	1.709253913	-1.298109475	-0.00343499	-7.757316664	1.599036262	0.399802858	8.45395786	10.00281502	8.266786541
Staphylococcus_succinus	-0.742901373	-1.122053459	-0.769547809	0.462490963	0.842552489	-0.771961675	-0.009426659	-0.020828428	-0.077100968	-0.338918811	-0.07963005	0.695355811
Cetia_pacifica	-0.74139784	-2.844107762	NA	-2.198035708	NA	0.650587232	0.448205767	0.721056833	1.264352999	2.453471144	1.65278269	0.795996143
Butyrivibrio_fibrisolvens	-0.739299291	-2.386901031	-1.163842408	-0.50605719	0.379699375	-0.347095114	-0.454845281	-2.575660633	1.549544056	0.234152126	-0.187515346	0.094704868

Rubrivivax_gelatinosus	-0.738180216	-3.248970922	-1.749269395	-0.409496657	0.695895288	0.341365587	-0.384424229	0.91869739	1.214506533	1.064992599	1.215576405	0.85558856
Salegentibacter_salegens	-0.737484456	-4.351805136	-2.55264478	-0.738388604	-3.153426104	NA	-0.166403208	0.137171198	0.980298953	0.291956015	1.137095908	2.440095281
Trueperella_pyogenes	-0.736554957	-2.224626792	-0.043939378	-0.220576459	-0.865230652	-0.765555613	-9.72977537	-9.72977537	0.357329405	0.180057692	-0.13476084	-0.125772785
Abiotrophia_defectiva	-0.736292852	0.927910601	1.344362161	2.823071229	0.270056461	1.112173019	0.33221318	1.091361903	0.594418213	-1.781464623	-0.094520911	-2.566677135
Streptococcus_macedonicus	-0.728697978	NA	NA	NA	NA	NA	-1.24660124	-1.811911603	0.452452225	8.389450655	0	0
Flavobacterium_johnsoniae	-0.725230338	1.407997826	0.088446495	NA	NA	-3.619197235	0.221012074	-0.234774652	0.13367357	0.801445785	1.001715406	-0.21416638
Massilia_umbonata	-0.72224687	NA	-1.224220006	NA	-2.48209413	-2.626910683	0	0	0	-1.14991355	0.742596416	0.492504893
Aeromonas_schubertii	-0.721196474	-1.166865043	-0.708253752	-1.306551017	2.243535408	2.598696762	0	8.607330314	6.15711649	0.728693137	0.209599352	0.625117282
Phascolarctobacterium_faecium	-0.719443726	-1.901557402	0.022690298	0.321304729	0.90165907	1.234241129	2.308574729	-0.24191489	1.270495881	0.322229985	0.157073165	-0.49488577
Acidaminococcus_fermentans	-0.714640253	-1.939256784	-0.465032714	1.93968624	0.644300069	0.337647736	0.858106233	-2.462645211	0.966453808	0.472194082	0.503942136	0.283425254
Sinorhizobium_meliloti	-0.712833276	0.015129937	-0.13407853	-3.279683035	-3.047854084	-1.475974046	9.315767728	8.110425888	0	1.182160426	0.32125072	0.883660688
Campylobacter_lari	-0.711113878	-4.241860744	-1.103123034	NA	-2.536349241	-1.736909155	0.454268462	0.668878904	-0.564051252	-0.017696491	0.001950726	-0.256460419
Clostridium_sp._'deep_sea'	-0.708051877	-0.966180098	0.630115599	-0.080680713	-0.583448131	0.483149981	-0.360069408	0.504465573	0.955804238	-0.31458612	0.775876479	0.818709699
Caproiciproducens_sp._7D4C2	-0.706303501	-1.931898291	-0.283540709	0.811210817	0.131455509	0.129146285	-9.376223085	-2.693551477	-1.070781455	0.353635451	0.197593042	0.059972736
Prosthecochloris_sp._CIB_2401	-0.704778794	0.056999165	0.326764988	-3.810036014	-3.977146	0.114281654	0	0	10.02362962	-1.005677571	0.118986515	0.671762181
Parageobacillus_genomosp._1	-0.704340447	-0.947794484	1.294165307	3.171151887	1.665869106	1.06796755	9.380166652	0	8.624046047	-1.383037635	-2.925866631	-0.970783226
Staphylococcus_agnetis	-0.703079882	-2.230451944	-0.100204982	-0.543429001	1.563116256	-0.467480147	-0.800241961	0.377264912	0.533578312	1.010069778	1.069068196	1.093161107
Actinomyces_sp._dk561	-0.702917512	NA	-1.073642306	1.373834951	0.03437763	-0.106744739	-0.134988131	-0.833807717	0.9378699	2.036643284	2.025946334	1.610822673
Pedobacter_sp._JBR3-12	-0.702707055	-0.894706759	-0.541487646	-0.714001913	0.641644388	-0.48389731	0	0	8.30544163	0.785231531	-0.426086013	-0.707110782
Glaciihabitans_sp._INWT7	-0.699683643	-1.354187077	-0.245866422	-0.651345169	1.033748791	1.159071176	-10.83260524	-0.312792104	-0.538616386	1.148330554	1.548891698	0.416391937
Cedecea_lapagei	-0.69800907	-1.728809191	0.740177407	1.840249389	1.582015969	0.684817021	-9.062167594	-0.454837281	2.887130226	-0.199601213	0.509606438	1.677743028
Moorea_producens	-0.697631716	-2.076464298	0.04811788	-4.577483562	-3.477164028	-0.898513519	0.564591343	-3.751581303	-0.179436865	0.075240778	0.364745425	-0.177659346
Lachnospira_eligens	-0.697381539	-1.878112129	-1.242336767	-0.378966942	0.315325498	0.252620703	1.515326496	1.442156048	0.951074623	0.490012785	0.114182314	0.131500176
Thermoanaerosceptrum_fracticalcis	-0.697321174	-1.296263428	-0.755521043	0.518803597	0.86160845	0.527300518	-8.10567284	1.808031897	-8.10567284	-0.153067282	0.270371643	-0.049570337
Clostridium_septicum	-0.696545883	-0.396807613	-0.702058176	-0.152101156	0.99006888	0.832901073	0.822543359	6.235235689	4.97520036	1.588755718	1.816305089	1.193830556
Anabaena_cylindrica	-0.695048978	-1.80870576	-0.738316432	-1.249508549	2.903198813	0.884917771	-1.462120296	-1.755435456	-0.301264022	1.590676865	2.746341562	-0.268196111
Desulfitobacterium_hafniense	-0.69489082	-2.044691985	-0.357401697	-0.783826224	-0.144360739	-0.194345004	-0.694049927	-0.306423932	0.518089192	1.233489743	0.854660327	0.335368056
Jiangella_alkaliphila	-0.694460866	-0.275066961	-0.012032555	NA	1.003152055	-1.998738351	-0.255309802	1.418454375	1.535193157	2.105194042	2.445668278	-7.709655951
Faecalitalea_cylindroides	-0.693831666	-2.011134072	-1.005868273	0.617521328	0.335137496	0.44481275	2.678542192	4.108024466	4.357918406	0.623938825	0.201940953	0.652012236
Brevibacillus_sp._JNUCC-41	-0.693071683	-1.752795594	-2.754719239	1.132864629	0.765398521	2.000231554	0	0	0	-1.323991699	-0.355329666	0.658615383
Weissella_cibaria	-0.692431629	-1.593293775	0.518665614	0.677138597	-1.062541466	1.489989434	-2.109350428	-0.416368745	-1.350297919	-0.030872093	0.467974468	2.212337864
Amphibacillus_xylanus	-0.691101854	-1.643495921	0.08561346	-3.073850626	-0.28767157	1.465961572	0.152730181	0.834108627	1.726059742	1.874401548	1.267632433	1.233530369
Brevibacillus_formosus	-0.68812557	NA	-3.337034987	NA	0.163218171	-1.036234981	-0.84674919	0.382694782	0.718567951	0.853866026	1.403166692	0.56401116
Lautropia_mirabilis	-0.687303075	-2.868184046	-1.541225795	1.395016163	0.099229011	1.524028492	-0.141042585	-0.190200832	0.208322826	-0.615376625	-0.005956943	0.05730321
Corynebacterium_jeikeium	-0.685858943	-3.1382718	-0.84633371	2.044797614	0.970853654	2.446663426	0.06711398	-0.028208649	-0.306189761	2.178987868	2.747697728	2.519880951
Mycoplasma_yeatsii	-0.683107495	-0.494250678	-4.332016913	NA	NA	NA	0	0	0	-8.95419631	1.258506767	0.469270511
Mycetocola_sp._449	-0.681698288	-0.124433776	1.38280036	NA	NA	NA	-1.01489812	-0.700298848	-0.369029489	-0.979535897	-2.906893816	-2.378857396
Vibrio_gazogenes	-0.681533616	1.456538971	0.655165926	-0.100298166	-0.78900661	-2.394495606	0	0	0	1.279586467	1.371882132	0.169094007
Ruminococcus_bicirculans	-0.675994274	-1.64235595	0.316772538	-0.011318877	0.413246444	0.559459739	-2.144992134	-3.066277304	-0.262573397	0.551913351	0.261616839	0.016408708
Salicibibacter_halophilus	-0.673624477	-1.404353864	1.912981746	3.471819774	0.138539943	-2.594115236	0.397888093	-0.025768011	0.385812582	1.277645465	1.322376123	0.892756404
Listeria_monocytogenes	-0.672476868	-1.779168093	-0.305864133	0.996203181	0.775826815	-0.66786635	-0.412788943	-0.862826233	-0.496064759	0.458445236	0.161511442	-1.24338404
Hungateiclostridium_thermocellum	-0.670223648	-1.20309542	-0.091331606	-0.410163431	0.676051665	0.060636901	1.813926917	2.829302143	2.069134118	0.135775114	0.545022129	0.206883359
Cupriavidus_gilardii	-0.667128996	-1.208385563	-2.781522629	NA	0.86019736	0.3623999	9.029913869	8.607330314	9.924542344	-0.714136758	-8.048034513	-8.048034513
Streptomyces_formicae	-0.665335917	NA	1.269386972	3.75038611	-0.645542566	NA	-0.64462393	0.364420577	0.179127293	7.303780748	0	9.026061374
Methylomusa_anaerophila	-0.66486034	-1.355248342	-0.423220746	-0.770493004	-0.192297439	-0.05521075	-1.630642967	-0.087254028	0.405197309	1.132611865	0.729630874	0.468522127
Sphingomonas_sp._CL5.1	-0.66484375	-3.774185744	-4.306733694	NA	-6.059020236	-5.095329799	-1.404430641	-0.680968377	0.15460031	0.275675639	0.838379168	0.289641848
Micromonospora_purpureochromogenes	-0.662858811	-1.472232901	-0.346996717	0.282875318	1.930947863	NA	-11.58530529	-11.58530529	-0.018038411	0.166070823	-0.016727046	-0.131971409
Corynebacterium_xerosis	-0.662418641	NA	0.370079717	-2.191655085	-1.047767944	-1.778378043	-0.477920621	-0.571560568	0.212703028	-0.240542344	-7.544323092	0.206102528
Acidihalobacter_prosperus	-0.661583782	-1.839602466	-0.983511877	-1.207488414	NA	-0.372233177	-1.952250785	0.704045026	-0.188765558	9.312882955	7.222389201	9.398479853
Thermoanaerobacterium_sp._RBIITD	-0.658741626	-1.538210301	-0.962490436	-0.114649701	1.02797605	0.925010104	-0.359610221	-0.626841093	0.456803563	1.483999778	0.41431937	0.398944723
Clostridium_cochlearium	-0.656150363	-0.454510268	0.180540136	-1.231784892	-0.16791472	0.893548359	0.416298045	0.633819294	0.034953338	0.610229711	0.762829824	0.557620509
Rhizobium_pseudoryzae	-0.654108662	0.007089425	-0.264456481	0.815575429	0.600269754	-2.805912948	-8.091313724	-8.091313724	-8.091313724	-2.445530595	-0.180722446	-3.204991342
Frankia_sp._EAN1pec	-0.654108662	0.007089425	NA	2.744686189	2.260099115	1.649194828	0.414062214	0.420798346	0.940921966	-7.890768904	1.462377922	-1.346448903
Synechococcus_sp._WH_7803	-0.654108662	0.534336428	1.514573173	NA	NA	NA	0	0	0	-2.75330393	-10.05708468	-0.401255251
Campylobacter_hominis	-0.650700298	-2.640646633	-1.038422044	-1.603604639	NA	NA	0.053436118	0.545332618	-1.057676343	-1.255795405	-1.146120945	-1.401796357
Xylella_fastidiosa	-0.649271188	-3.116718153	-1.060802743	-0.754015704	-0.941160973	-0.973278043	0.408652061	0.615227042	1.430328815	1.069906022	0.901871442	0.510312313
Campylobacter_geochelonis	-0.648127502	-2.151788518	-0.397759508	-3.366771212	-2.192494186	-2.951188991	-0.272566268	0.687217619	-0.145515079	0.938384698	0.94889155	-0.605316373
Bacillus_altitudinis	-0.647783283	-2.643154908	-1.262830362	1.203856958	1.303484469	-0.499297469	0.490298799	2.067471977	0.438738066	0.961631648	-0.236900274	1.601756684
Gudongella_oleilytica	-0.6471209	1.07093758	-0.044394119	-0.730998375	-0.959302085	0.742410147	0.255280604	0.68337676	1.330859806	0.371541549	0.34008749	0.401445508
Rhodococcus_sp._WB9	-0.646218189	NA	-4.623454659	NA	NA	NA	0.807356187	-1.05246578	0.619960823	-8.841303302	1.273310988	-8.841303302
Flavobacterium_gilvum	-0.645288744	2.288382628	1.601745183	-0.695737516	NA	-6.298663442	0.49974448	0.549631303	1.101378107	0.791257082	0.186242618	0.802685524
Bifidobacterium_bifidum	-0.645052399	-1.623605423	-0.708115993	-6.343438904	0.352229107	-0.420575111	11.24782073	10.60901248	10.58444582	0.368900781	0.859346826	0.492591853
Desulfosporosinus_orientis	-0.644900558	-1.532845138	0.819542807	0.113874256	0.426501446	-0.457501592	8.6039638	11.04736934	9.944646183	0.915616676	0.28333699	0.189191342
Geobacter_metallireducens	-0.643520361	0.63114719	0.995867715	-1.607133882	0.121402051	1.182019823	-0.007902284	-0.793158317	-0.094558532	0.152091337	0.937384122	1.047443906
Veillonella_dispar	-0.641623844	-1.712772085	-0.958061721	NA	2.481869008	4.502939228	-2.347921641	0.176926633	0.494953407	-10.83973131	1.19895795	0.05559403
Lactobacillus_graminis	-0.640787369	-0.585393735	0.497571519	3.948514924	1.697630682	1.243557031	0	0	9.104480817	1.537910654	1.783213722	2.041402476
Bacillus_megaterium	-0.640734664	-2.07345473	0.265728902	-1.028960824	-0.187054711	0.116047319	8.502740328	10.5340402	9.202346702	0.668643946	1.079892408	0.83590684
Burkholderia_sp._PAMC_28687	-0.640705053	NA	NA	NA	NA	NA	0.091011796	0.842021007	0.601422373	-9.768184325	-9.768184325	-0.712901889
Streptococcus_pseudoporcinus	-0.639531443	-2.1568802	-2.531775263	-2.742145833	0.698768136	0.544054557	1.083252224	2.415034226	2.367850278	-0.978821674	0.744881607	1.55763558
Pseudomonas_sp._Os17	-0.637691444	NA	NA	NA	NA	NA	0	0	6.15711649	0	0	0
Dickeya_zeae	-0.637079294	-0.976230867	0.052868911	0.524290598	0.505997062	1.072504207	0	6.682671608	8.863904389	0.66287055	1.077364685	1.638107065
Flintibacter_sp._KGMB00164	-0.633183622	-2.110235069	-0.628528999	2.078605378	0.032333398	0.131998253	-8.799135887	0.581030764	-0.318252808	0.346169754	0.227183269	0.035822568
Algoriphagus_sp._Y33	-0.632596545	-4.691336009	-0.696453495	-2.204600082	-1.94370142	1.428631628	-1.925112298	-0.188253113	-0.271116392	0.89175945	1.36172822	0.889228161
Pelolinea_submarina	-0.629206639	-2.049740354	-0.571909474	1.417906607	0.609937356	0.602825643	-0.604666449	0.411813105	0.088626852	0.158696186	0.087341942	0.058860918
Neptunomonas_phycophila	-0.629017681	-1.14839184	-1.862825221	NA	NA	2.220644759	0	0	0	0	7.996236198	9.700439718
Streptomyces_sp._CRXT-G-22	-0.629017681	NA	0.871507718	1.028854863	-0.24911203	2.243779849	-10.04984855	-0.188761643	0.464599227	0.314999971	-0.644747887	-0.098761923
Thalassospira_marina	-0.624570834	-0.088182723	0.300382565	NA	NA	NA	-0.783889889	-1.096038837	-0.970083312	0.142654098	0.525956856	2.103584533
Acidovorax_carolinensis	-0.621608421	-1.0283663	-0.523293768	NA	-0.464262217	-0.157541277	-0.812902697	-0.296669919	-0.314877386	0.180890272	0.041469803	1.373386284
Streptomyces_chartreusis	-0.620657048	1.099208049	0.149470258	NA	-3.343174057	-2.49416792	-0.932188767	-2.007664131	-0.872735276	0.464485406	-0.015803825	0.704746658
Desulfitobacterium_dichloroeliminans	-0.619763424	-1.917710749	1.483528872	-2.8870044	-1.063395081	-0.850606386	-0.31132517	-0.128229183	-0.458698994	0.17271049	0.855396978	0.839774686
Acutalibacter_muris	-0.618777944	-2.185234013	-0.679038676	0.374446742	-0.084140359	-0.381124746	-9.177646265	-9.177646265	-2.020531797	0.355100355	0.094919149	-0.127508909
Acidovorax_sp._1608163	-0.618656116	-2.078190009	0.278566006	2.449394701	2.144740376	2.135071391	-8.637284195	-8.637284195	-8.637284195	-0.120627182	0.656013206	0.944511437
Francisella_marina	-0.618282574	-2.011021406	-0.897860896	NA	-1.103352743	0.992764034	0.982544529	2.416900709	2.419675732	0.063590317	-0.262713031	0.015739645
Erwinia_persicina	-0.616232651	0.789188533	1.749808273	-0.884679317	0.986712598	0.753153086	0.271421318	0.85735663	1.720386627	10.18156227	6.815921204	7.380824669
Leucobacter_muris	-0.610957709	NA	NA	NA	NA	NA	-2.608360236	-10.77463883	0.429375914	-8.709659397	-8.709659397	-1.778922059
Corynebacterium_lactis	-0.610053482	NA	1.761627648	4.199929624	NA	2.996618946	-0.340841006	-9.797661526	-1.346450414	1.801385473	1.979875004	1.062315732
Salinibacter_ruber	-0.609462867	-0.334278214	0.804726787	3.199151478	3.824334544	3.335753599	0.48423999	-2.365864656	-0.734210206	0.016726247	0.244293537	-0.089249633
Vibrio_sp._Sal10	-0.608169892	2.321928095	0.164229396	NA	NA	NA	0.07496487	-8.091313724	-0.346177255	-3.511602548	-2.517701219	-10.8153833
Geobacillus_sp._E263	-0.608169892	NA	0.204744556	NA	NA	NA	-8.624046047	-8.624046047	-8.624046047	0	0	0
Treponema_sp._OMZ_838	-0.60791724	-1.959683593	-0.688899688	1.321659562	0.741148254	0.493802938	-1.13929079	0.860320113	0.100106866	0.224435357	0.470743006	-0.193711106
Polaribacter_vadi	-0.606937875	-1.83877321	-1.733741324	-0.189978997	1.108351127	-1.075369316	0.35267047	-0.118864948	-0.444207258	-0.326784863	-0.25710117	-3.055454451
Methylobacterium_nodulans	-0.606585015	-2.485923537	-1.760930585	-0.36300959	0.140576358	2.177084793	0.126374241	1.650203762	0.301769006	0.286384512	0.129417475	-0.490429597
Chryseobacterium_jeonii	-0.603313379	-1.376728474	-1.862342004	-1.2518723	2.928899217	2.843418001	-0.945079925	-0.370500362	-1.819503941	-0.576699747	-2.076062821	-0.744010509
Candidatus_Deianiraea_vastatrix	-0.603299806	-3.889842302	0.948389796	NA	-1.701088558	-1.408134639	-1.3341349	-1.116437671	-1.562396023	9.422064766	9.673604122	8.938600435
Peptoniphilus_sp._ING2-D1G	-0.600613258	-1.448060428	-1.709443257	-0.577572116	2.929647352	3.137663573	0	0	0	0.772205344	0.429004198	0.33302562
Clostridium_ljungdahlii	-0.599792569	-0.694717827	-0.410554093	NA	NA	NA	0	0	9.929258409	-8.739216921	-0.836341397	-0.613374709
Aminomonas_paucivorans	-0.599436771	-1.767850928	-0.310260808	0.999097676	0.242821198	0.414641427	-0.118548862	-0.114589614	-1.361572923	0.282876498	0.303817964	0.215789979
Luteimonas_granuli	-0.595747171	-2.781127837	-1.318376749	3.021886178	1.767374812	2.682599891	0.422679974	-0.283477764	0.090225867	0.432155609	-3.4602201	1.321529478
Klebsiella_variicola	-0.595683925	-0.526351504	0.582958293	NA	0.104903223	-0.631281269	-1.206488569	-0.3124916	0.107111047	-0.656912343	-0.071492241	-0.408984829
Bacillus_beveridgei	-0.593836656	-1.85439813	-1.327549273	-0.954696638	0.987691741	-0.238826879	0.530618377	1.60732733	1.663563707	1.465761767	0.59916478	-10.24475627
Massilia_violaceinigra	-0.592216022	-1.679164442	-1.419777813	-1.366400669	0.59533898	-0.245732505	0	0	10.83439994	-0.564553639	0.543952025	0.408502771
Planococcus_faecalis	-0.591956294	-2.754735839	-1.408178078	-0.495756512	0.745528818	-0.971985624	8.166278594	9.913704737	10.58786188	2.590929813	3.35196847	3.237579735
Actinomyces_sp._432	-0.589739641	-0.067114196	-0.317463847	2.71904252	-0.506469243	1.723002862	-8.799135887	-8.799135887	-1.642021419	1.464108106	1.493379713	1.265894953
Azoarcus_sp._DN11	-0.58025786	-5.453932323	-3.618702713	2.931445962	-1.05119343	2.530871019	-0.500996545	0.186765857	0.409319359	0.246875009	2.39087086	1.463711346
Tumebacillus_avium	-0.579444301	-0.85164424	-1.148491523	0.607656362	-0.472249761	0.326785377	0.264473212	-0.704821992	-0.301045943	-0.241918401	0.138840658	-0.608413013
Desulfatibacillum_aliphaticivorans	-0.57932855	-1.469748087	-0.739119649	-1.175348283	-0.596642059	-0.923297703	-0.164001398	0.633699396	0.377425996	0.087041784	0.245213641	-0.341187916
Ruminococcus_sp._JE7A12	-0.578798191	-1.943711652	-0.528280592	0.071155237	0.640430564	0.486132085	0	0	0	-0.85291087	-0.860130549	-0.937013721
Thiomicrorhabdus_indica	-0.577949312	-1.014559824	-0.298228042	-0.248486097	-0.459397772	-0.726085422	9.502742316	6.682671608	9.451211112	-0.33498125	0.043683537	-0.922595379
Scardovia_inopinata	-0.576720487	-1.528790528	-0.001236508	0.455347441	-0.124837112	0.495036821	-9.384097309	-9.384097309	-0.665174059	0.57489155	0.161892994	0.868080407
Butyricimonas_faecalis	-0.57599722	0.682125637	0.174128986	-2.418205053	-1.226626509	-1.106137864	-1.22994141	-0.90238866	-3.256122297	0.345361192	0.059968975	-0.146849507
Pseudomonas_sp._HLS-6	-0.574160803	-1.307767385	1.485378424	0.947057429	-1.637204904	-2.926944872	3.438972929	3.905593368	3.094586409	2.068883983	2.322595655	0.526589113

Helicobacter_mustelae	-0.570832172	NA	-0.902854777	-2.36979695	-0.714202631	-4.578432075	-10.31679063	0.98709284	0.15857803	-1.438499307	-0.034540172	0.170072901
Megasphaera_stantonii	-0.569818393	-1.275085055	-0.35970905	0.621516666	0.587209962	0.474542956	0	0	0	0.165798976	0.026337781	0.223234875
Caldicellulosiruptor_saccharolyticus	-0.568244449	-0.807839512	-1.467863859	-0.570233586	-1.414829002	-0.586109773	-9.177646265	0.202520387	-3.020529775	-0.034711162	0.597082094	0.343672463
Thermus_sp._CCB_US3_UF1	-0.567883525	-2.402452268	-0.461542025	-0.131327557	1.844602899	0.733649253	1.378344575	-0.59487697	0.102752871	1.08732921	-0.922032121	0.037279118
Frederiksenia_canicola	-0.567475074	NA	NA	NA	NA	NA	-0.365643674	0.012904696	0.125498796	-0.072187412	-0.370294404	2.544963711
Bacillus_sp._OxB-1	-0.567118828	-3.128659066	-0.172676002	1.139381767	1.953459624	0.269312852	9.029913869	0	0	-0.088227183	0.609424432	0.301902568
Arthrobacter_sp._TES	-0.565941088	-1.989255068	-0.167543317	0.189467221	0.977827706	-1.404487329	0	0	0	0.008831957	-0.872694491	-0.232652657
Deinococcus_wulumuqiensis	-0.564994084	-2.682081283	0.112523233	1.797251104	0.818462459	-0.161794547	0.780311314	0.35343316	1.185371325	-0.060304593	-0.007165839	-0.331941038
Pseudobutyrivibrio_xylanivorans	-0.564445922	-1.803838595	-0.632146062	-0.614540281	0.207522488	-0.244926266	-0.817461791	-0.806675384	1.605302023	0.901560112	0.50635966	0.215651842
Klebsiella_michiganensis	-0.563013528	0.493886339	0.632567532	2.047257537	2.19002913	2.573634121	-0.414770684	0.851511391	0.911574675	0.108072376	-0.274106457	-0.471403705
Nitrosospira_briensis	-0.562096003	0.685398452	3.995497223	0.801304883	2.112829455	3.516736421	-10.24727483	-10.24727483	-10.24727483	0.104433479	0.805717094	0.386703648
Shewanella_aestuarii	-0.561944158	-0.953755815	-1.712631026	-3.10575077	-0.948759024	0.129927084	-0.803503306	-1.303759403	-1.751947135	-0.745857922	0.289973737	-0.530431349
Mariniblastus_fucicola	-0.561911432	-0.791045431	-1.265633734	NA	1.257496222	-0.433954713	-1.056250747	1.675261389	3.397337021	0	0	8.520944699
Desulfolutivibrio_sulfoxidireducens	-0.560560949	-2.197545873	-0.539418629	1.284045007	-0.535395761	0.166796797	-0.286846698	0.560415954	0.658364002	0.862069003	0.968344339	0.802349594
Streptococcus_sobrinus	-0.559883308	-3.811646079	-0.470832357	0.720798391	-1.49625585	-1.442084375	0.35974974	-0.030933018	0.284867792	0.487748953	-1.718738657	-1.491180558
Ethanoligenens_harbinense	-0.557884213	-1.446733712	-0.173315809	0.76222908	0.699951612	0.540270017	0.503437409	0.094356891	1.875375742	0.46057338	0.272731468	0.307460232
Mannheimia_varigena	-0.557878622	NA	2.679254388	6.245254608	0.548853416	-1.00314999	1.287404779	1.334388649	-0.04434317	1.113732994	0.572813418	-0.980472631
Cellvibrio_sp._PSBB023	-0.557016693	-1.584677291	-0.51856621	-2.084099403	0.511994105	-0.407735656	0.287547482	0.378694622	2.549437389	0.463224979	1.998089502	2.246519648
Hymenobacter_sp._BRD67	-0.55366823	-2.340541803	-0.224937113	0.258227802	0.939033127	0.350391642	0.081676307	1.067621777	0.346893389	0.099563551	-0.693864587	-0.588767657
Roseimicrobium_sp._ORNL1	-0.553133019	0.707462058	0.964305661	0.167099649	2.748050072	2.361445016	-0.612335627	0.161230579	0.895472748	-0.152962251	0.463322038	0.43843173
Planomicrobium_glaciei	-0.551785375	-2.09966468	-1.844449467	-1.526698667	0.034881866	-0.150153169	-3.202854259	-1.704769375	-1.304728846	1.06852361	1.420872483	2.073175142
Nocardiopsis_dassonvillei	-0.548310078	-0.53276629	-0.09352193	-2.367748702	0.313618283	0.79765799	10.81012976	12.38532318	12.73315094	-0.277937826	-0.367614345	1.23036059
Streptococcus_infantarius	-0.546882155	-4.334583396	NA	NA	NA	NA	-0.153728493	0.219987922	-0.363379946	-8.924812504	-1.702423303	-8.924812504
Nocardioides_ungokensis	-0.546844166	-0.77193515	-1.887467332	NA	NA	NA	0	0	0	-2.477578965	-9.781359714	-9.781359714
Clostridium_pasteurianum	-0.545572273	-2.170466403	-1.918515921	-0.607019044	0.701241941	0.543955147	-0.224092675	1.994034687	-0.102936579	0.054209864	-0.210061551	0.157374788
Chitinibacter_sp._2T18	-0.540568381	NA	-1.231204924	NA	NA	2.824060823	1.132953776	1.808559805	1.449462225	-0.724461957	1.207728936	0.789311147
Bacillus_freudenreichii	-0.537970211	-0.334735871	-1.237675447	1.396791861	0.741307328	0.879908311	0.05246742	2.277532974	0.54723045	0.527860888	0.919255261	-0.309688245
Parachlamydia_acanthamoebae	-0.532375539	NA	-4.031813002	NA	NA	1.897829549	-0.234961178	0.556360763	0.687584221	-0.422887402	1.187360868	1.678067743
Lachnoanaerobaculum_umeaense	-0.531505497	-2.467207284	-1.906216793	-0.54796949	0.69097579	0.4072898	-10.20011392	-10.20011392	-0.336209529	-0.165102826	-0.405640476	0.420588885
Acetobacterium_sp._KB-1	-0.529331467	-1.051806172	-0.456146173	-0.43458368	0.124779131	0.184135945	-0.273455825	-0.781637747	0.496114189	0.624043854	0.571608191	0.5050055
Leptolyngbya_boryana	-0.526320833	-1.63253824	-3.813668696	NA	NA	NA	0	0	0	0.746393832	-0.218327271	1.923091495
Pedobacter_sp._SW-16	-0.523816072	-0.208598942	0.449773218	NA	1.256662323	0.543466598	-0.138990117	-1.418194057	-0.040099854	0.39704138	0.078863619	2.007029835
Limosilactobacillus_fermentum	-0.523669075	-1.809809567	-0.468739935	3.484239488	0.77495623	0.576094118	-0.568495557	-0.200096977	0.25587671	0.499437391	0.59915911	-0.022062167
Thermotoga_caldifontis	-0.523280647	-0.009484021	1.147494668	NA	NA	NA	10.17990909	10.05970948	9.863904389	-8.186525318	-2.714037547	-8.186525318
Auricoccus_indicus	-0.523123247	-2.310564791	0.30597407	-3.314016704	0.766800824	-0.245238426	-0.841989491	-1.121575093	-0.424465685	1.802731862	1.519177315	1.016297709
Clostridium_thermarum	-0.522412837	-2.271113314	-0.459723172	-1.015729496	0.195335302	-0.850232609	9.315767728	10.25051161	9.104480817	0.150470816	-0.204384749	-0.101170922
Desulfitobacterium_dehalogenans	-0.521926243	-1.430021285	-0.118558502	0.138625366	-0.005443686	-0.337129613	-0.513773043	0.662162922	0.746774774	1.130134704	0.989095603	0.682093205
Agrobacterium_sp._T29	-0.521254906	-0.572957722	-0.640457613	NA	NA	NA	-0.138292174	0.207747839	0.72713441	1.142874614	-1.961061165	-8.776982369
Methylocystis_heyeri	-0.52059167	-2.110896785	1.170416634	3.614815877	4.534813144	4.098525029	1.233641464	1.767271986	2.840383015	-0.348938411	0.455872751	0.396653936
Arcobacter_thereius	-0.520280659	-4.132004228	NA	NA	-0.940004035	0.442344845	-1.342146726	-2.447045726	-1.046204734	-0.753838214	-0.389383491	-1.104332885
Rahnella_sp._Y9602	-0.518904837	-0.913241428	0.4996397	NA	-0.68251781	1.011055189	1.405862303	-1.941374438	1.91352182	0.645254698	-0.266497854	0.213031637
Arachidicoccus_sp._BS20	-0.517160258	-1.742252503	0.941535546	-1.602158116	-0.581547836	0.744467216	0.474817342	0.643107792	0.09343802	-1.223577006	0.765321804	2.007375957
Chlorobium_limicola	-0.517052847	-0.4106679	-0.838691041	0.219120539	0.923004965	0.741961328	-1.170200049	-0.125731581	-0.168950338	0.734113849	1.007810382	1.823663448
Bacillus_sp._N1-1	-0.513888051	-3.416077222	1.52829902	NA	-1.421460743	-0.758842054	0	10.96381366	9.6039638	-0.470051337	1.060349423	1.543779441
Flavonifractor_plautii	-0.510529768	-2.261414661	-0.644212541	1.328353078	-0.188654027	-0.048872178	-3.515838909	-0.754670415	-0.049847653	0.48526652	0.213260182	0.004264088
Confluentimicrobium_sp._EMB200-NS6	-0.510452223	-0.904980163	1.296902699	NA	NA	NA	0	0	9.598899253	8.366322214	8.945639889	8.986789195
Streptococcus_suis	-0.51026552	-1.537772195	0.07733226	0.277231284	0.148796162	-0.030533222	-1.098723126	-0.379898507	-0.617804962	0.446030985	0.160264132	0.268695758
Thermincola_potens	-0.507463191	-1.206659629	0.330818429	-0.720400868	0.521271608	0.024508547	-0.946379242	-0.594516916	-0.751788977	0.507262918	0.39721054	0.081948732
Symbiobacterium_thermophilum	-0.505459899	-1.718078748	-0.583958747	0.98835716	-0.067857087	0.551682284	0	0	0	-0.050526403	0.162598565	0.275643431
Sulfurihydrogenibium_azorense	-0.504631232	-1.1222762	-0.062134661	-0.331449586	-0.337065568	-0.205735942	0	0	0	0.696664338	0.789811078	-0.157165303
Corynebacterium_ureicelerivorans	-0.503330072	-2.506861763	2.470867109	3.35846399	4.106238185	3.786451156	0.278888926	-0.319618534	-0.484586837	-0.046036832	0.775734687	-3.950865476
Staphylococcus_pasteuri	-0.501868563	-1.364977343	-2.495875109	1.481074298	0.967857716	0.509298231	-1.300781116	1.485246315	-0.208123563	1.173628043	1.920914443	1.032992817
Desulfobacter_postgatei	-0.500063728	-1.378557709	-0.502020367	-0.81300024	NA	0.153430798	0.341375944	0.01134109	-0.107413429	-0.282142875	-0.202783352	0.458002291
Nitrospira_japonica	-0.497125856	0.783861219	1.882822572	1.308981548	-2.963058422	-1.266364344	-9.177646265	-0.55027654	-9.177646265	-0.172163654	-0.009479774	-1.265197029
Congregibacter_litoralis	-0.496805355	-0.434849607	-0.289244285	1.806692923	NA	-0.949917201	0	0	0	-1.645504043	1	-8.77478706
Mesorhizobium_opportunistum	-0.496233582	2.120829762	3.863723994	4.303633467	4.087462841	3.922704281	-9.471675214	1.986080142	-0.629038875	-2.366125072	-2.504913166	-1.74755323
Bacteroides_intestinalis	-0.496172898	1.292696063	0.350932236	-1.201844692	-1.939355483	-2.873652236	0.456510862	0.521458497	0.772159127	0.327038724	0.0229035	-0.21765685
Streptococcus_pasteurianus	-0.494164769	-0.595263445	0.440872515	-0.735531886	-1.443604552	-1.055691664	0	9.965784285	0	0.164683682	0.324787367	-0.482370261
Rhizobacter_gummiphilus	-0.490654546	NA	0.132891815	1.172180975	0.527812374	1.213392093	-0.721325551	-2.06856079	-1.152342028	1.626370419	0.78998472	-1.616157122
Blautia_hansenii	-0.486905858	-2.029954317	-0.965787683	0.288136455	0.411609651	0.156634871	-0.703598539	0.330784051	0.641417147	0.514470281	0.308943947	0.271837392
Roseovarius_sp._THAF9	-0.486543464	0.070787917	0.734172379	NA	NA	NA	-0.927440023	0.786977621	1.39266392	1.049788426	-2.418281133	-7.890768904
Chamaesiphon_minutus	-0.484587644	-2.108034874	-0.215692587	NA	-0.117470133	-0.357958424	0	0	6.15711649	1.233513438	0.73477289	-0.061417433
Micromonospora_sp._HM134	-0.48454201	NA	NA	NA	0.680778288	NA	0.303598097	-0.116845003	1.215356226	7.129283017	8.41672576	0
Virgibacillus_sp._SK37	-0.483282425	-3.337197816	0.047696742	NA	-1.187543402	-1.203995709	0.772944465	1.934160004	-0.527820344	0.287802421	0.940535788	0.821612205
Geobacter_lovleyi	-0.479437426	-1.304192764	-0.27096575	0.402784032	-0.25475146	-0.561001841	0	0	0	0.424446482	0.615847627	0.216331253
Mycoplasma_felis	-0.477618926	NA	-0.850777641	NA	NA	NA	-0.220471121	1.034452431	0.384593339	0	0	8.89683794
Kosmotoga_pacifica	-0.477260014	-2.084316286	-0.060469544	NA	0.308122295	-2.460488925	-0.614040674	2.311561338	0.56723895	-3.25153516	0.281912497	-0.876992439
Lysobacter_antibioticus	-0.476753284	0.241610259	-0.715378732	2.873061198	NA	4.149834326	0.74182258	1.070628231	1.396089618	10.81378119	8.118941073	9.696967526
Leptotrichia_shahii	-0.475869643	-2.826366891	-0.915086196	-4.907566399	-0.708958643	-1.192714023	-3.362725596	-2.339315962	-2.282813819	0.105631325	1.13701345	0.714782696
Herbaspirillum_huttiense	-0.475234851	0.626102843	1.484932368	0.965167428	0.183322857	-0.008283439	-8.552496194	1.251047301	-0.807359725	-2.090490534	-1.816287455	-3.017923269
Anaerobutyricum_hallii	-0.474349018	-2.206558614	-0.978673159	0.40905979	0.435375862	0.191699213	-0.036114917	-0.252199702	-0.106066387	0.079834476	-0.078578294	0.315374354
Providencia_alcalifaciens	-0.471973396	-1.47606615	-0.78133298	0.589211382	0.688974203	-1.313209667	1.123828587	-0.081440946	-0.144324653	0.923613953	0.746359186	0.753757257
Desulfopila_sp._IMCC35004	-0.471772224	-0.858795347	-0.365330527	-3.45649289	-1.200641371	1.568939512	0.324366879	0.331513895	-0.167437645	0.578733418	1.318141746	1.538637404
Chryseobacterium_gallinarum	-0.470978249	-0.536030731	-1.367249137	3.836562214	1.287145414	1.736198627	0	0	0	0.31056248	0.250344103	0.881356577
Synechococcus_sp._BIOS-U3-1	-0.46661995	-3.482561493	-0.373572475	0.436373961	2.001421764	-0.778872546	2.052858097	2.825056924	3.800046634	1.677733091	0.605804945	-0.681906169
Mycolicibacillus_koreensis	-0.466147138	-1.410793582	-0.784979844	NA	NA	NA	-2.529722323	-0.258481427	-1.257628169	-8.044394119	-2.571906348	-1.113656782
Limnochorda_pilosa	-0.464800177	-2.580976195	-0.283819377	1.020945172	-0.097548801	-0.031724324	-2.412485887	0.453423743	1.025829602	0.619868129	0.173424783	0.413364518
Rivularia_sp._PCC_7116	-0.463615925	-3.289634692	-1.051628102	1.05707266	-0.149342045	1.100611545	9.029913869	0	9.477205768	-0.530221954	0.595414108	0.305051428
Paenibacillus_psychroresistens	-0.463406181	-0.173033579	0.431658273	-0.987821333	1.24839783	1.255409607	-0.646532944	-0.159856865	0.174932176	-0.901830768	-0.189848684	-0.308785757
Atlantibacter_hermannii	-0.46317402	-0.289097781	-0.687417237	3.286715362	2.642237306	1.613443525	3.086613877	-0.980890481	2.087460507	-0.626445811	-1.490262994	0.8027474
Selenomonas_ruminantium	-0.462171996	-1.267574396	-0.423198601	1.306456686	0.151830305	0.344669768	-0.235843369	-0.055193261	0.151832661	0.123524721	0.171100459	0.259367975
Caloramator_sp._E03	-0.459406934	-1.87598392	-0.791029442	0.142987507	0.542113626	0.902357336	-0.190854391	-0.07133901	0.274174222	0.471164579	0.444969755	0.051926299
Enterobacter_cancerogenus	-0.458615034	1.067624117	-0.284874374	2.384305379	2.94111684	1.680554559	0	10.02990835	6.15711649	-0.147190222	-0.42241751	-1.077731001
Treponema_phagedenis	-0.458205615	-1.607957455	0.604056858	-0.584001951	0.435892414	-0.521217002	2.025574036	-1.111820807	1.258634365	0.37921201	0.252637414	0.167428292
Pseudomonas_koreensis	-0.457303226	NA	3.209760355	NA	-1.354767491	-2.252433184	-9.177646265	2.565197871	2.9096044	0.99206475	3.019444418	1.876193798
Chromobacterium_sp._ATCC_53434	-0.456788512	-1.953946477	-1.615261101	NA	NA	NA	-1.369323071	0.217973306	0.198257259	7.894817763	8.91607949	8.539158811
Bordetella_petrii	-0.456756889	1.502701905	0.01194432	0.730523383	1.241648911	-0.568981715	0	10.05479422	9.624047875	-0.254032941	0.315570455	0.291623254
Weissella_diestrammenae	-0.453365618	-4.430544657	0.264507295	NA	-0.075013354	3.669026766	0.355406122	1.58240075	0.498013681	0.476454981	-0.019095015	2.391084082
Ruthenibacterium_lactatiformans	-0.453144671	-1.943910969	1.504197793	-1.239158614	-0.776576802	0.385122662	-0.29542833	-0.253455552	0.990048801	0.301317351	0.354751093	0.803235571
Lysinibacillus_timonensis	-0.451153461	0.757830837	0.315567713	-1.665708259	-0.107973987	-0.484822894	-0.709032782	-0.191027646	0.149387936	-0.386758792	1.18405002	-0.75354845
Blautia_sp._LZLJ-3	-0.448069651	-1.840271139	-0.847499511	0.426463368	0.436502008	0.25927391	0.380805971	1.484007399	-0.259853043	0.291321651	-0.050102084	0.168302726
Aerococcus_viridans	-0.446584881	-1.339085577	1.149577328	-0.019954606	1.538647308	0.588920514	0.340672423	-1.597928183	1.363616389	1.149822964	1.509881283	1.403278069
Desulfosarcina_ovata	-0.446198941	-0.382136046	-0.010547777	1.433208375	1.099649208	1.110400401	-0.445411148	1.799721973	2.939579214	-0.030195014	0.687372029	0.152056187
Thauera_chlorobenzoica	-0.446167397	NA	-2.94560486	NA	NA	NA	-0.49476303	1.761029019	1.830911204	0	0	0
Kocuria_varians	-0.444389584	NA	-2.886138389	1.23599057	NA	0.623115324	10.49911838	10.08166984	10.78285082	9.300734635	9.650572508	8.958068353
Roseobacter_litoralis	-0.444389584	NA	0.320860355	NA	NA	NA	0	0	0	1.799975392	0.969627447	1.662964335
Clostridium_autoethanogenum	-0.443547978	-2.155636212	-0.925304758	-2.236811481	2.568932391	2.239934723	0	0	0	1.906085961	0.780495222	0.234670575
Microbacterium_sp._SGAir0570	-0.442943496	NA	NA	NA	NA	NA	0.237511801	0.087460323	-0.402981238	0	0	0
Cryobacterium_sp._LW097	-0.441590306	-1.840491423	1.10332881	NA	2.688665336	2.669346319	-2.580374568	1.085475206	-0.153080441	-0.820712242	0.53783927	-9.46216073
Enterococcus_cecorum	-0.439924337	-1.628508563	1.832088866	3.743090657	0.139109691	1.744615425	-2.535188209	-0.70472118	-0.683016959	0.758306433	1.315362682	0.575681845
Clostridium_sp._SY8519	-0.439892015	-2.022432499	-0.820763487	0.491157696	0.052495707	-0.211379052	9.029913869	8.110425888	0	0.243373154	-0.008228589	-0.073623735
Leptospira_mayottensis	-0.439314661	-1.219027924	-0.609486484	NA	2.448530645	2.041662639	-6.564335086	-5.168484849	-5.060569303	-9.376487917	-2.560566713	0.291214423
Monoglobus_pectinilyticus	-0.438998118	-1.91812237	-0.478805332	0.668661751	-0.165224255	-0.148675305	0	0	0	0.566210376	0.312375887	-0.212695595
Desulfobacca_acetoxidans	-0.438104399	-1.758453957	-0.949347128	0.232757585	0.620919378	1.187016362	0	0	6.15711649	0.345767206	-0.116939335	0.346453139
Propioniciclava_sp._HDW11	-0.437535907	NA	-0.180022501	-0.052443577	1.766973051	0.764132913	-8.925892636	-0.366427591	-8.925892636	10.2463487	12.04575966	11.00187963
Pelobacter_sp._SFB93	-0.437344348	-1.297487347	0.324761628	0.917012069	0.188716405	-0.507522213	-0.439247871	-0.354832027	0.290897418	0.638126352	1.128444147	0.409786853
Cloacibacillus_porcorum	-0.43537489	-2.317164672	-0.422964664	0.642762415	-0.203311923	-0.045309611	-1.216872198	0.522886174	-0.912952543	0.662548732	0.199698496	0.133211881
Duganella_sp._AF9R3	-0.434704533	-0.678630116	-0.479717588	0.553231143	-1.522840789	-1.198296746	0.167159889	1.234410086	0.205593401	0.52176991	-1.597466775	1.718799978
Arthrobacter_sp._YC-RL1	-0.434079947	NA	0.554779495	NA	NA	2.012106603	1.343841939	1.179144398	0.607753319	2.281774604	0.689519528	-1.54314284
Desulfopila_sp._IMCC35005	-0.430202489	-2.728161936	-2.252695615	-2.300622085	-5.441755793	0.544697184	0.034505053	-0.844780277	1.629922779	0.22638726	1.392840044	1.049151803
Bacillus_filamentosus	-0.428206173	-0.663287777	0.015684859	-0.539552773	-1.23855297	-1.179082931	2.171538967	1.34983075	1.064129176	0.467080698	1.018001914	0.229015061

Aminipila_sp._CBA3637	-0.427477517	-1.668650755	-0.873901578	-0.124153571	-0.636303699	-0.201497971	0.734397869	2.371481504	1.287201622	0.491442367	0.580801887	0.314364188
Yersinia_canariae	-0.426931178	-0.361555169	-0.944236728	NA	NA	NA	2.07423625	0.276458316	-0.948558372	8.366322214	5.472487771	7.539158811
Spiroplasma_litorale	-0.426931178	0.919626584	1.010621116	-1.51564144	-0.618759671	0.529723864	-1.843573941	-10.34631427	0.364802019	0.347461793	-0.553178561	-0.317694744
Paenibacillus_sp._FSL_R7-0331	-0.421247677	-2.116960359	-0.376930476	0.14940761	-0.491108498	-1.1107774	0.462759653	-1.059540638	0.761733253	-0.22805966	-0.199272107	-0.212035463
Chlamydia_suis	-0.421076416	-0.453883045	0.214623447	NA	NA	NA	-9.209009766	0.398320548	-0.068064993	-0.081941614	-4.461202883	-9.933690655
Caldicellulosiruptor_kristjanssonii	-0.416381417	NA	-3.065963262	NA	NA	NA	0.444584794	0.693652553	0.611475428	8.86005338	9.975375111	0
Acidipropionibacterium_virtanenii	-0.41514968	-2.512315396	-0.032157633	1.080339412	2.305352692	2.653419381	0.432122377	0.183584406	0.102862135	0.403072978	-1.286910566	-0.310020047
Lactobacillus_casei	-0.412823078	0.22235443	-2.362380139	0.811004352	-0.559608257	-1.831220752	0.516235767	-0.336361786	-0.22830838	-0.104999586	0.094862474	1.120224067
Xenorhabdus_doucetiae	-0.412498288	-1.533699746	-1.378511623	-0.173237735	-0.391697131	1.667833113	-0.606677226	0.493645221	-0.21137412	1.518381297	2.761438025	3.50164009
Bacillus_sp._SB49	-0.4117354	-1.189832609	-0.459286725	0.439187275	0.378675699	-0.184844791	0	0	10.39290769	1.488453541	0.048677105	0.145299223
Streptomyces_sp._RPA4-5	-0.411325907	-0.218680829	0.151222444	NA	NA	NA	-0.774279264	-0.973941186	0.446888168	2.608002239	1.964740637	2.075516637
Clostridium_tetani	-0.411067376	-1.094478052	0.043686825	1.832551538	0.347655721	-0.049730035	-10.20011392	-0.81601661	0.108429896	0.108995656	0.200326231	0.437824327
Georgenia_faecalis	-0.410912237	-2.482473773	0.092449414	1.555442134	2.14779964	1.01036317	-0.103639108	0.058026805	0.092352294	7.303780748	7.712410067	9.410805357
Candidatus_Sodalis_pierantonius	-0.410837511	-0.874399018	-2.702333709	2.318682435	0.601082166	2.485233837	0	6.682671608	0	10.47573343	8.906890596	9.678306113
Jeotgalibacillus_malaysiensis	-0.409042498	-3.643630927	1.75331803	-2.949959318	-1.962870016	-0.062575842	0.157240012	0.164519984	1.773850855	0.092137248	0.724191134	0.970082897
Streptococcus_parauberis	-0.408854319	-1.195611645	-1.376842242	-2.254010966	-2.825238082	-2.751017817	-9.552494273	-2.869822665	-9.552494273	1.185924489	1.094480559	0.714218198
Azorhizobium_caulinodans	-0.408535933	NA	-0.675727965	NA	NA	NA	-0.734085048	-0.172468614	-0.383957663	-9.376487917	-9.376487917	-0.718276434
Novosphingobium_tardaugens	-0.408246454	-4.725400043	-1.522661304	-0.78878213	-2.178212973	-0.581425037	-0.544677705	-1.63799458	-1.070343712	1.252503549	0.716434266	0.986627178
Komagataeibacter_oboediens	-0.403307192	-2.799183539	-1.657025543	5.204754405	1.956672568	1.370610033	0.652240455	0.939716918	-0.182675128	-0.203591981	0.362511734	-0.417158019
Rhizobium_leguminosarum	-0.399673608	-1.271378599	0.531425473	-1.950071187	0.357933544	0.86357717	-0.189195057	-9.346312052	-1.040870422	0.189488853	0.37628025	0.593623959
Lactobacillus_manihotivorans	-0.399304573	-1.861361586	-0.557010569	0.865337609	0.070645403	0.319925162	-0.276814394	0.891791367	0.263960735	0.253294308	0.691258342	-0.081497383
Escherichia_albertii	-0.399171094	0.719769979	-0.483172242	2.19468887	-0.227618722	-0.65941245	0.703717711	0.405587931	0.013551019	-1.944146765	0.05372186	0.947653854
Paenibacillus_chitinolyticus	-0.398804487	-2.021982619	-0.408723859	-0.23059801	0.458964753	-1.063819642	9.648765614	9.627367901	7.157114468	0.56296833	-0.494992298	-0.463320207
Sinorhizobium_americanum	-0.397783818	NA	-1.151662795	NA	NA	NA	0	0	0	0	5.472487771	10.4415644
Romboutsia_sp._CE17	-0.396017303	0.332404742	-1.145138486	-0.466343383	0.18159298	1.604629534	0.30073765	0.256555924	1.413663569	0.392042598	1.180573572	1.435451913
Burkholderia_thailandensis	-0.386568745	-2.761994389	0.506872462	-1.083081741	0.63102212	-0.063691628	0	9.968407603	9.154818109	1.63315877	1.686377204	3.085839908
Chelatococcus_sp._CO-6	-0.386269457	NA	-4.109814536	2.164386818	NA	0.9278942	-0.644749796	-0.66343279	-1.272582992	1.52550641	-0.687066535	-7.745956618
Ramlibacter_tataouinensis	-0.385624013	-0.023083613	0.112643402	1.637374665	3.078104293	2.783285303	-0.506168834	-1.981610557	-2.144055214	0.090426164	0.143873391	1.015001432
Brachyspira_hampsonii	-0.38466385	-1.847635827	-1.962571593	NA	-0.957144521	-2.557673279	0	0	0	0.653300588	1.834077927	1.511879479
Yersinia_pestis	-0.384630593	1.105032572	0.763067697	1.515274912	-0.196352219	0.111151292	0.047171403	-0.375406632	1.55395224	-0.812476769	-1.136201276	-0.397439808
Indioceanicola_profundi	-0.38210446	-2.679503063	-0.192155282	0.524408025	-0.418814783	1.947800811	0.938318078	1.727047477	1.944029388	-0.534472029	0.090049577	0.744171086
Herbaspirillum_frisingense	-0.382087077	-1.376513233	-0.973112311	1.291302131	-1.113008962	NA	0.29966365	0.565308046	-0.640135343	-8.202941691	1.799873325	2.973231458
Magnetospira_sp._QH-2	-0.378839811	-1.946741069	-0.317850214	-2.175939965	0.742632436	-0.391597715	-1.084702434	0.024652869	-0.288925222	1.049024476	0.289346166	0.137870842
Francisella_halioticida	-0.377423207	-1.340956266	-0.947727027	-1.866248611	0.510961919	-1.293864176	2.233252176	1.021640215	1.092579592	0.636467964	3.829337451	4.025061922
Bartonella_apis	-0.37597738	-1.670667124	0.479833406	0.774247427	0.838377764	0.090331163	0.416088547	0.668214513	0.469872697	1.565398442	0.818186556	1.162899964
Christensenella_sp._Marseille-P3954	-0.372863691	-1.945378049	-0.381340711	0.436724574	0.128779981	0.132834265	0.645598434	1.748363338	0.757438665	0.502173962	0.096884112	-0.281977184
Roseibacterium_elongatum	-0.370229704	-0.404457184	-1.148467693	-0.785449019	0.352331174	0.218350549	0	0	0	1.302004665	-0.1646908	0.608882118
Helicobacter_cinaedi	-0.370003386	-1.477092344	-0.235010677	-8.166304838	-7.41668407	-10.29713391	0.707473626	0.351817274	0.484959627	-1.341780656	-1.124349535	-1.21273012
Altererythrobacter_atlanticus	-0.36923381	-2.727757056	-1.862924371	-1.407276883	0.871920186	3.870803611	-0.049816648	-0.365476932	-0.603596889	-2.520877008	-1.178655956	-0.181094058
Aquiflexum_balticum	-0.368922828	-0.924859481	-1.236979958	1.470086281	0.840269545	0.677378135	0.784986322	0.252042086	0.356246616	-0.462444133	1.722299588	1.473471092
Lactobacillus_sp._3B(2020)	-0.368696748	-1.256007526	-1.336286711	2.509351208	0.064075685	-0.882373021	-0.008793666	0.54645098	-0.483887709	-1.588122677	-0.999473757	-0.609111919
Citrobacter_sp._FDAARGOS_156	-0.366782331	NA	NA	NA	NA	NA	1.263435099	-9.076815597	0.654503434	7.894817763	0	6.930737338
Butyrivibrio_proteoclasticus	-0.366244897	-1.97590406	-0.995939849	-0.897026968	-0.107486571	-0.386284862	-1.055968101	-9.222246695	-9.222246695	0.133782582	-0.103234336	-0.143270987
Bacteroides_fragilis	-0.366015512	0.510056678	0.162036754	-1.861855289	-1.146381277	-1.110857373	0.719316004	-0.728911882	0.474165676	0.05705558	-0.118262492	-0.247195112
Cohaesibacter_sp._ES.047	-0.361707997	-2.358522763	-0.01652667	3.597213631	4.118698604	3.561979183	11.07742327	0	0	0.891929866	1.245670673	-0.003667311
Dactylococcopsis_salina	-0.36154538	-2.777940853	-0.179707057	-0.264278419	0.75830659	-0.199910741	0	0	0	0.535072288	0.010262165	-0.842340147
Thermanaerovibrio_acidaminovorans	-0.361162587	-1.6099548	-0.617373325	2.385063775	0.622621844	1.526149211	1.352355289	0.973641755	1.772569568	0.597004327	0.463831737	0.431785142
Mesorhizobium_sp._M9A.F.Ca.ET.002.03.1.2	-0.360920087	-2.575064146	-0.434991042	3.456601475	1.467987859	-1.576228131	0.363055626	0.78051323	-0.612264041	-0.472083054	0.103319922	-0.118245358
Mixta_gaviniae	-0.359990218	-2.43082148	-1.021430544	NA	0.528171055	1.218793494	0	0	8.451211112	0.518502819	0.489155167	0.095722727
Caldicellulosiruptor_owensensis	-0.358510971	-2.415284515	-1.664942323	NA	-1.738854351	-2.395249991	-0.228354541	-0.308618721	-1.857132267	0.506038719	0.931314596	1.174011597
Yersinia_pseudotuberculosis	-0.357404356	-0.879201856	-0.309773234	0.040091719	0.509351687	NA	-0.679693595	1.702306814	1.343876916	1.459434382	-1.420448427	1.29575889
Desulfosarcina_alkanivorans	-0.35464826	-1.441581523	0.186742973	1.705333454	0.857023315	0.860394702	0.386637355	0.549216289	0.477030432	1.07190789	0.608717351	0.756962014
Salimicrobium_jeotgali	-0.354639603	-0.825262856	-0.432620207	-0.859730786	0.793467005	1.117558848	0.547854606	-2.334456838	-1.559470999	0.42356704	0.403829537	0.578157919
Lactobacillus_acidipiscis	-0.353636955	-0.524137614	-0.284547498	1.331993134	0.853597262	2.13321635	-4.030313	-7.731627596	-6.747799525	-1.545993406	0.115226183	0.789517661
Campylobacter_sp._CCUG_57310	-0.353345177	-0.487441704	-1.963692996	NA	NA	-1.031645627	-0.808652152	-0.600147254	-0.5050204	-8.743713548	-0.595323403	2.281426014
Xylanimonas_cellulosilytica	-0.348428608	-3.850221572	-1.049177913	1.176016615	2.60717378	-1.085937525	0.477374375	0.272786507	0.152526491	0.51796848	0.947113831	0.378222024
Pseudoclostridium_thermosuccinogenes	-0.346168385	-1.39967377	0.11498976	-0.640609327	0.059224037	0.427408042	8.246199041	0	8.548995253	0.739725326	0.760947556	0.262364875
Ruminococcus_champanellensis	-0.34596654	-1.527988942	-0.334582705	0.117908417	0.039827395	0.069444003	-1.341797113	-0.42311874	0.208353614	-0.020726063	-0.238814458	-0.365530062
Faecalibacterium_prausnitzii	-0.345645565	-1.936041902	-0.858313914	0.381215327	0.186854199	0.1275065	1.338689288	0.063056548	1.897276843	0.421299882	0.092998015	-0.001885823
Streptomyces_albus	-0.345323232	-0.978273513	-0.654604784	1.469393827	1.48312036	1.001645222	0.300528613	0.394074724	1.190038537	0.33212364	-0.56374941	-0.504399991
Acinetobacter_nosocomialis	-0.340572273	-1.853791701	-0.777902669	-2.009150628	0.152591468	-0.370705797	1.20392317	2.496673559	2.998352313	1.542710622	1.832249838	2.726981506
Methylobacterium_sp._WL1	-0.339101544	-3.35320106	-1.183704501	NA	NA	NA	0.21467259	0.207791211	-0.443098888	-1.500073603	-1.813435416	-0.041890362
Desulfobacterium_autotrophicum	-0.338511929	-2.154667471	0.317480386	-2.689774983	0.884790364	0.619357848	-0.553468381	-1.497404781	-1.508577156	0.412998285	-0.876979408	0.932403524
Spiroplasma_clarkii	-0.337500809	0.323697188	-0.627628966	0.409255147	NA	1.579823777	9.029913869	10.49099381	9.718921538	0.662499433	0.724026208	0.480996866
Corynebacterium_halotolerans	-0.336214602	-0.837943242	-0.422086262	1.679934649	0.333068652	-0.085425588	2.144075519	-9.222246695	0.984547908	1.887759815	0.864936574	0.002159188
Caldanaerobacter_subterraneus	-0.336107804	-1.900851522	-0.999811876	-0.370174428	1.205349142	-0.886097245	0.023610177	2.184138575	2.554483541	0.423819547	0.296417209	-0.982017912
Acinetobacter_sp._ACNIH2	-0.335854471	NA	-0.586877685	2.093647529	NA	0.086508993	-0.398571244	-0.898479215	-0.750794865	-7.890768904	1.022120432	-7.890768904
Thermoclostridium_stercorarium	-0.334885155	-0.82099958	-0.301207493	0.3502282	-0.055556947	-0.079796055	0	0	9.328062269	0.007669791	0.096675964	0.149759531
Arthrobacter_sp._ERGS1:01	-0.333700686	-3.840974744	0.227996611	0.895955118	0.146389487	-0.94054615	0.715855614	0.71708521	0.335938694	0.813764345	-0.412040497	-1.30256202
Clostridium_formicaceticum	-0.332111497	-0.8036865	0.379167709	-3.612891715	-1.293109447	-0.905191384	-1.850111744	0.042489867	1.265385131	0.030318609	0.327101457	0.507855966
Desulfurivibrio_alkaliphilus	-0.32800839	-2.880562517	-0.043311917	2.174341766	-0.661540735	-0.527205737	-0.386652004	-0.238746461	-0.07849182	0.581791291	0.957433082	0.849678321
Bifidobacterium_breve	-0.323967014	-1.988375003	-0.774305736	-0.994035286	0.39147546	-0.095285626	-0.183520572	0.907468684	1.068078882	0.438165666	0.658805824	0.259995843
Nocardioides_sp._HDW12B	-0.322886378	NA	-1.013474234	NA	NA	NA	-2.615552347	-1.051863493	-1.993411279	1.831683648	-7.807354922	2.256040159
Fusobacterium_mortiferum	-0.322639997	-1.225396748	-0.052310503	-1.907832022	-0.421681528	0.527445735	-0.811922227	0.23729924	0.13442152	0.201148121	0.108785801	0.792066942
Bifidobacterium_pseudocatenulatum	-0.322459116	-1.850035425	-0.176519332	-0.157048289	-0.877390888	-0.968189122	0.268587984	-0.159273775	0.444620903	0.40909246	0.119938958	0.178958173
Sporosarcina_pasteurii	-0.322096723	-0.23220556	-0.294536442	-1.24765918	-0.296041512	2.149908378	-7.757316664	-7.757316664	2.497499382	0.127902833	1.834177426	1.71112472
Janthinobacterium_sp._SNU_WT3	-0.321928095	-1.286845636	-0.802037858	1.75748084	0.142019005	0.455233074	1.041816905	-0.050512031	0.041041606	-0.389409214	0.932416288	0.995073837
Thermosulfidibacter_takaii	-0.319311398	-1.915670471	-0.142446866	NA	0.915864665	-2.629293167	-0.597090314	-0.437656713	-0.735435457	0.640779333	3.353797217	3.339112066
Rhodoferax_saidenbachensis	-0.316106856	NA	-1.190979469	NA	NA	NA	-10.92925841	-0.429233192	0.995958498	-2.067239337	-0.215983531	0.117695043
Oricola_sp._MEBiC13590	-0.314462645	-0.918161708	2.09503779	-1.386278506	-2.971241007	-0.169925001	-0.289824254	1.403216865	2.792807642	1.619727369	1.491852547	2.43544002
Microbacterium_sp._A18JL241	-0.310340121	-1.546649322	-0.682599891	NA	NA	NA	0.525421772	-0.114520471	1.659847431	7.333897756	0	0
Streptococcus_sp._KS_6	-0.30956129	-1.612034372	-0.270370908	-0.804203914	0.862007676	-0.363370021	-1.444019549	-9.573302566	1.028133969	1.508939333	0.850581784	1.031301558
Sphingomonas_panacis	-0.307667402	-1.150941898	-0.752709579	NA	1.173237735	0.780092842	0.341229274	1.094736764	0.881843107	2.583912122	0.200811891	1.290079229
Magnetococcus_marinus	-0.307049261	-0.878825159	-1.647871667	-0.274908506	-1.917647338	-2.919510082	-12.05293819	0.466430239	0.778315609	1.703417156	1.720122816	0.631795756
Ectothiorhodospira_sp._BSL-9	-0.306673614	0.556976326	-0.143205791	-1.602673621	-2.198707127	-2.81191671	0.314546738	2.456919295	1.035626174	0.232232635	0.227866334	0.111193879
Anaeromyxobacter_dehalogenans	-0.305782054	-2.806671432	0.102431954	-0.08008709	-0.46793191	0.574172301	-1.22407411	-0.252274856	0.581819833	0.006842506	0.293725289	0.913417758
Rhodococcus_sp._ABRD24	-0.304224171	-1.571156701	-1.815575429	-1.921671639	0.07539307	-0.480481852	-1.513014318	-0.399334396	-0.901428533	2.809809463	1.2882188	1.337303321
Staphylococcus_muscae	-0.303249131	-1.862420093	-2.075018075	-1.616480256	-0.493332025	1.065329514	-1.084889722	3.152969684	2.817325386	0.80062374	1.080659153	1.15064753
Achromobacter_sp._B7	-0.302351781	-3.799893185	-2.015621876	-2.556071522	-2.003652394	0.511697686	0.023610177	1.841632422	1.371532928	-0.934274191	-10.83815604	-0.858016459
Anaerocolumna_cellulosilytica	-0.300327513	-1.67012819	-0.562907704	-0.301377513	-0.001133321	-0.25208265	0	0	0	0.38127618	0.227065074	0.060716935
Staphylococcus_sciuri	-0.299656715	-2.47111068	1.823376393	4.774007746	0.165303688	-0.150678912	0.004214491	0.258069772	-0.035738452	-0.088130132	1.925808711	1.263217384
Sebaldella_termitidis	-0.299264414	-1.1144257	-0.219964567	0.022666041	0.245559439	-0.360371088	0.366540072	0.627974581	0.596110855	0.1410106	0.533998904	0.203469277
Thermodesulfatator_indicus	-0.297176506	-0.352323499	0.539843955	NA	-0.18126655	0.502402341	-9.062167594	0.364478753	0.561880281	1.695165732	-0.609367186	1.167040462
Veillonella_parvula	-0.294147004	-0.604040999	-1.897586358	-2.257964507	-2.58548084	-0.64048505	1.397587047	-2.462368656	-0.763261658	0.701201403	0.732567273	0.788369255
Clostridium_carboxidivorans	-0.293118154	-1.224647049	-0.047553447	-0.696093313	-0.243565609	1.160608673	0.305680087	0.73421285	1.27291049	0.602926251	0.660804973	0.23476649
Mogibacterium_diversum	-0.29264358	-1.470156968	-0.178542375	-0.182864057	0.866353138	0.071537416	-1.479952949	-10.86601158	0.1063149	0.148590344	-0.276951286	-0.410254475
Lactobacillus_murinus	-0.292156516	-2.892490751	-2.661178275	5.936328959	-0.738805339	-1.50204698	1.024338991	2.175673074	1.596280736	-0.365269228	-1.048707341	-0.67380717
Clostridium_perfringens	-0.28824827	-1.550456359	-0.627166455	-0.081137546	0.502082229	0.690859258	0	0	6.15711649	0.442477612	0.253185998	-0.012699896
Marmoricola_scoriae	-0.285770515	0.225515399	-1.057001078	1.163135836	-0.460248264	0.73812625	-1.341331727	-10.21784867	0.201637548	-0.659778962	1.007735649	0.527651457
Pseudoalteromonas_tunicata	-0.285557422	NA	-1.280119744	NA	-2.168945242	-2.250543462	0	0	6.15711649	9.963859889	6.470320477	6.544320001
Filifactor_alocis	-0.281503199	-1.002830457	-0.27415707	1.121903054	1.148868904	2.562557311	-0.565940994	0.9925542	0.98044857	-0.020405921	0.390934911	0.387903303
Paenibacillus_sp._BIHB4019	-0.280187702	-0.870404278	-0.39067206	1.481619656	0.200234536	0.155216401	0.974815687	1.378138187	2.830049437	0.924913124	0.912479677	0.334826924
Tatumella_sp._TA1	-0.279957709	4.103945341	2.770844453	NA	NA	NA	-9.661778098	2.184425284	3.398395978	0.303521785	0.975527192	1.70660082
Oceanispirochaeta_crateris	-0.279806665	-1.68373512	-0.718941278	0.747089811	0.724038742	1.582451719	0	0	0	0.627678735	1.461492761	-1.123134483
Bacillus_paralicheniformis	-0.279214121	-2.112573561	0.52161613	1.731220919	0.456585878	0.072423075	0	0	0	-0.77689942	0.02696253	-1.169174726
Shewanella_chilikensis	-0.278088752	NA	-0.254672087	NA	NA	NA	-1.839736084	-10.44369988	0.584330478	-8.705055194	1.727904213	-0.046843711
Lactobacillus_animalis	-0.276918466	-2.757273293	-2.764500727	5.782013859	-0.792932825	-1.494049235	-1.532177113	-9.778376154	-9.778376154	-0.423251292	-1.084736115	-0.663603852
Candidatus_Atelocyanobacterium_thalassa	-0.276290503	-3.437894113	0.209770337	NA	1.559211506	2.069813517	0.985560784	-0.277432343	-1.584698997	1.057667112	-0.761890838	-1.040575365
Actinomyces_israelii	-0.274964616	-5.226698639	-2.121401586	NA	-0.116229405	0.619513048	-1.54213857	-0.019389553	1.376500027	-0.867124622	0.252173449	0.883501417
Staphylospora_marina	-0.274581316	-0.731037162	0.17932712	-1.608587447	0.563686751	0.453618658	0.59166512	2.145067429	0.141330657	1.176620542	1.164930978	0.240400156
Paenibacillus_mucilaginosus	-0.274580108	-1.646580446	0.101389261	0.822974453	1.626350203	1.994587171	8.157119522	0	9.104480817	0.678404352	0.46616342	0.917138391

Halocella_sp._SP3-1	-0.273857213	-1.15067809	-0.107644513	-2.717272698	-0.074081784	-0.128276259	-0.81298833	-0.270288381	-0.21839402	0.28962088	1.101473804	0.915499763
Rhizobium_sp._IE4771	-0.27308807	NA	NA	NA	NA	NA	-0.907901331	-0.342443618	1.465082267	0	5.472487771	0
Trichodesmium_erythraeum	-0.272624336	-0.734057422	0.861865599	0.630266477	-0.131929899	1.128568271	0.533016069	1.718647284	1.070492919	-0.219024841	0.272621524	0.780542545
Burkholderia_glumae	-0.272447439	-2.537028024	-1.373403011	4.069320105	2.98169589	3.538825879	-1.847775951	0.464594964	-0.122389041	-1.29578496	0.651599344	0.449069128
Variovorax_sp._RA8	-0.271397879	NA	-2.561799468	NA	3.043754201	2.152618747	0.255769813	0.877374319	1.385468108	0.684744162	-9.255814636	-9.255814636
Lachnoclostridium_phocaeense	-0.269782185	-2.040109186	-1.080157386	0.252439639	0.147515861	-0.186319318	0.537996432	-0.255463966	0.405852538	0.49579901	0.162239857	0.079292517
Lactobacillus_sp.	-0.268929962	-2.507996052	-2.795330366	6.673465554	-0.721601013	-0.763192726	-1.304403673	0.042100744	-0.265567415	-0.841724194	-1.498706644	-0.921521663
Sulfuriferula_plumbiphila	-0.268769021	-2.327883728	-1.151954178	0.833714537	0.713204325	1.159645037	-0.768255329	2.044049598	1.936330255	0.097116143	1.069030053	0.320846246
Parasedimentitalea_marina	-0.266951754	NA	0.844409355	-3.808924946	-1.097698673	0.382585708	0.753158597	-0.773467571	1.380917146	1.411246976	2.26372229	0.494189851
Arsenicicoccus_sp._oral_taxon_190	-0.266393418	-4.243572457	-0.317740298	2.836986496	-0.808182393	0.543185578	-10.17311494	-10.17311494	1.425361001	0.426781526	2.948638377	1.093323582
Salicibibacter_kimchii	-0.264492977	-2.831783046	0.638710964	-0.539931115	1.64138336	-1.052009953	0.297782567	-0.292299588	0.193530915	0.256085757	-0.329962438	-0.581503927
Legionella_anisa	-0.263604754	-1.310568233	NA	NA	NA	NA	0	0	10.2067946	1.449307401	1.86814918	1.977866395
Aminipila_sp._JN-18	-0.263346233	-1.408340842	-0.345091597	1.189954462	0.667953482	0.793566287	-0.433596072	0.421662182	-0.278041525	0.36094776	0.259821261	0.155400977
Selenomonas_sp._Marseille-Q3039	-0.261891833	-2.182019207	-0.213175988	-0.024926176	0.68133635	-0.101885051	1.325386415	1.685326654	1.453798331	0.220310048	0.055362978	0.3040578
Flavobacterium_sp._Sr18	-0.260734623	-0.96098634	-0.409816013	1.850228719	0.191581607	1.749206576	0	0	0	0.214042183	0.467302544	-0.471226047
Serratia_sp._KUDC3025	-0.259835062	-2.344382946	-0.217526666	NA	NA	NA	0	0	0	0	0	0
Enterocloster_bolteae	-0.259267117	-2.021013571	-0.753444537	0.041472238	-0.203002653	-0.244388435	-0.140482806	0.530244249	0.076117357	0.445078575	0.038666295	-0.032602299
Paenibacillus_beijingensis	-0.258863938	-0.733233834	0.017123396	-0.892955485	0.466389127	-0.020359575	0	0	9.140944773	-0.183608049	0.1519889	-0.589469484
Murdochiella_vaginalis	-0.256468028	-1.185953746	0.445780685	-0.529463584	0.356583275	0.712471195	0.184330157	-10.71502677	1.19117989	0.692543141	0.323470342	0.166047589
Carboxydocella_thermautotrophica	-0.256363951	-1.978491843	0.587247399	1.294660656	0.300089564	0.182120732	0	0	0	0.733814022	0.191769701	0.887615646
Spiribacter_curvatus	-0.253020126	-1.711162876	-0.873847401	-0.999103638	0.243247962	-0.608921327	-1.00064687	-3.217875738	-1.069481711	0.747419952	1.137503524	0.887391353
Pelosinus_fermentans	-0.252383539	-1.108897154	-0.718570741	0.897399921	0.237465912	0.312921707	0.875061226	0.161850613	1.643764882	0.055219733	0.210961337	0.199184386
Lactobacillus_bombi	-0.252245899	-0.869819009	0.708745721	0.757886246	2.215573899	0.956808469	0.417284775	1.894416062	0.819328015	-1.875677563	-2.99879819	0.233657357
Clostridium_aceticum	-0.248475894	-1.841417496	-1.540077398	-0.599342812	0.26215954	0.009669492	-0.938678074	0.388030111	-2.761666663	0.485718056	0.801028325	1.002768443
Vibrio_coralliilyticus	-0.246869586	-1.068130536	-2.24606608	1.483177949	-0.110775202	-0.039799061	-9.222246695	-9.222246695	-9.222246695	1.339084452	1.379189515	0.927919198
Egicoccus_halophilus	-0.243634289	-1.17790379	-0.537325226	-5.524326703	0.629050255	0.631190875	-1.917189508	-0.337929253	-1.273181106	0.114814519	-0.760118669	-0.167638958
Petrocella_atlantisensis	-0.242724433	-1.395921459	-1.002058347	0.14694788	0.284772539	0.740595296	-0.185950675	0.421730747	1.721857101	0.86317142	0.487615057	0.436141009
Mycobacteroides_immunogenum	-0.241897219	-0.231656217	0.32105874	-0.747307538	NA	-0.197363627	0	0	0	-1.0903633	-2.728399956	-3.000001159
Marichromatium_purpuratum	-0.240939136	-1.358419931	0.367219629	1.74539493	1.639069906	0.923579195	-2.272719617	1.137179908	-10.51891866	0.383735706	-1.273272248	-2.108411921
Rhizobium_pusense	-0.23667121	1.852154532	1.176985852	1.339630097	2.443966756	1.045293387	0	0	9.941980727	1.314901077	1.385946786	0.276023388
Thermoactinomyces_vulgaris	-0.23644686	-1.789139957	0.179450051	-1.138324596	-0.23728183	0.688700028	-8.076815597	0.033610291	3.129423463	-0.537554126	0.382366952	0.209075948
Aeribacillus_pallidus	-0.236082249	-2.535302599	1.311571688	-0.758438661	0.449588809	1.15304178	-0.779882435	-1.338966304	-0.478154436	2.609874071	0.833291711	1.628199595
Paraglaciecola_sp._L1A13	-0.234996169	-1.757604277	-0.400819954	-1.825931578	NA	-1.456108793	0.388168233	0.237956976	0.678313034	-3.246966037	-0.019628543	-10.55074679
Glutamicibacter_halophytocola	-0.234642805	-0.105801225	0.727578732	NA	NA	0.535493824	1.680489473	-0.230318276	-1.332340308	0.342688961	-1.287814989	-0.990889891
Nocardia_arthritidis	-0.231421387	-3.276009604	-2.504583655	2.448103196	1.129341154	1.201014432	0	8.607330314	8.904155902	-0.899160943	1.769176528	1.432473532
Staphylococcus_epidermidis	-0.231139503	0.490136047	-1.26066331	0.346933712	-0.258733295	-0.216687606	0	0	8.157119522	0.395905047	0.726809666	0.957431395
Herbaspirillum_hiltneri	-0.223892728	-2.447336294	-3.229156124	0.847996907	NA	-0.274086674	0.532074365	-0.055518608	-0.201538772	1.039968434	3.32783671	-0.346445812
Paenibacillus_sp._FSL_R7-0273	-0.223301175	-1.340346888	-0.558671447	0.584212434	0.41152903	0.50911186	-8.10567284	-8.10567284	0.518373206	0.491395283	0.503782504	0.681456466
Actinomyces_slackii	-0.221820476	-0.982108165	-0.001107635	2.126892447	1.396115481	1.457944808	-0.389323304	0.429597923	1.344399855	-0.828665428	-0.303578602	0.015255623
Blautia_sp._SC05B48	-0.221646245	-1.705372161	-0.784802348	0.744819379	0.49770001	0.60487256	0.051593675	-0.271378772	-0.093483887	0.432307551	0.405138385	0.436361497
Methylobacterium_radiotolerans	-0.219623332	0.233633489	-0.717692664	NA	NA	NA	0	0	9.782849183	-0.475277423	-0.715703261	-0.357211242
Catenulispora_acidiphila	-0.218243763	-1.571643818	-0.497187018	2.735916582	1.7062866	1.267718038	-9.714245518	-9.714245518	1.555518192	1.892691441	2.274889085	0.320900231
Pandoraea_norimbergensis	-0.216905235	0.940997801	-1.137607693	NA	-0.784096042	-1.277447064	-0.508001178	-0.009914469	3.78219676	-0.781931242	0.189041732	0.871978612
Propionibacterium_acidifaciens	-0.216193811	-3.451716654	0.413014269	-0.615634196	-1.686500527	-2.55721751	-0.238352938	0.057325698	0.018405018	2.040922368	1.554884712	1.283949553
Bacillus_cellulosilyticus	-0.214668398	-0.712446328	-0.942726822	NA	-1.957464636	-2.292339339	0	9.426646347	0	1.580243134	0.933089975	-0.495539055
Dehalobacter_sp._DCA	-0.210375457	NA	-1.717962627	2.131012197	1.831076522	1.195425374	0.803458352	0.053828124	0.27101057	-7.890768904	2.994927469	-7.890768904
Sphaerotilus_natans	-0.208094038	NA	1.229880272	-4.731601885	NA	NA	-0.422512116	0.851216507	0.112003934	-0.057548062	0.324574917	0.352917796
Paenalcaligenes_hominis	-0.207941265	NA	NA	NA	NA	NA	0.445626506	-0.017973164	0.379570955	-9.489179631	-1.776769564	-0.901713373
Ruminococcus_albus	-0.205385177	-1.214029742	-0.669282265	0.211526956	0.626406572	0.600719219	-8.637284195	2.419969752	2.794594665	0.424974623	0.363882792	0.267041726
Humibacter_sp._WJ7-1	-0.203176319	-1.154502323	0.299166255	-1.876709328	1.244605255	1.303379279	0.302354733	-0.088151483	0.064516508	-1.87790559	-2.294331986	-0.245201178
Paenibacillus_sp._PAMC21692	-0.203105976	-1.41278418	0.307506665	-1.51750593	-0.949563576	-0.003096775	0.399802858	1.826279514	1.793430121	1.559446344	0.711409463	-0.033776364
Halomonas_socia	-0.202896246	NA	-2.589174752	NA	NA	NA	0.680081599	0.333675744	2.098640359	-8.820178962	0.394140158	-0.553392422
Streptomyces_sp._Sge12	-0.202896246	NA	NA	NA	NA	NA	0	6.682671608	6.15711649	-9.662965606	-2.604075524	-9.662965606
Enterobacter_sp._SA187	-0.202556006	-1.12544708	-2.140271727	NA	-0.014727286	-2.744499267	-0.206340857	-0.746537647	-0.048428374	1.36340967	0.867039637	2.196430561
Oceanisphaera_profunda	-0.200939312	0.949092705	-0.43383965	NA	4.329235742	4.209656433	0	0	0	1.704102798	1.320560862	2.720746674
Bacillus_coagulans	-0.198105696	-1.31739553	-1.234474597	0.763789677	2.506312389	2.921399116	-0.730208907	2.128702717	2.087460869	0.003030682	-0.122533795	-0.962938998
Kocuria_turfanensis	-0.197661059	-3.515174171	-0.657193175	3.264716965	0.873388366	1.120222929	0	0	0	-0.166115562	-2.506296522	-0.400533977
Azospirillum_thermophilum	-0.197195986	-3.125516054	-0.857242099	-1.125186687	1.450107166	0.766202815	-0.708936349	-0.329330824	-0.911512288	1.335795099	0.935254693	-0.182360598
Kribbella_flavida	-0.197178803	-1.393287737	-3.359709145	-0.256397499	-0.609862025	-1.045401395	0.477417675	-8.552496194	1.569749429	-0.252195328	-1.177245072	-0.646091491
Paenibacillus_polymyxa	-0.195739319	-2.544867619	-1.079538765	-1.495551597	-0.881499958	-0.383149255	0.350102065	0.194191496	0.031181094	0.609636761	0.112094037	-0.004783078
Paenibacillus_crassostreae	-0.193204732	-0.829722735	-0.912262898	-0.165252	-0.064727337	-0.526803529	8.876516947	8.906890596	0	1.568656895	0.60372949	0.931097271
Paraburkholderia_sp._7Q-K02	-0.187177729	-1.561194335	1.457687653	NA	1.777942738	2.292124884	-1.005432913	-0.481364996	0.424641666	1.866482264	0.168086974	0.78060899
Mahella_australiensis	-0.184346226	-2.093638087	-2.059413087	-2.477691335	-0.336549006	-0.45558874	-0.1070773	-0.00039391	0.68737483	0.193826027	-0.097709739	0.062930847
Bordetella_sp._N	-0.182610815	-1.809801551	-1.213456782	-2.133445307	0.172180975	-0.14923489	0	0	6.15711649	2.026529483	1.646625521	3.146399814
Dickeya_dianthicola	-0.181477987	-2.409013712	-0.157715414	-1.736965594	0.277054876	0.909293086	-0.697970451	0.226808265	0.806666854	0.712490874	1.582864349	2.048609401
Bacillus_glycinifermentans	-0.180396862	-1.437750827	-0.296130076	0.672934081	-0.999474077	-0.496551074	-1.158326491	-0.462483378	-1.750314229	0.520871256	0.560216928	-0.404321173
Corynebacterium_sp._1959	-0.180087917	-3.001090335	NA	NA	NA	NA	-1.187934805	1.588994935	-0.246822514	-8.904884456	-1.682495255	-1.270676016
Lacrimispora_sphenoides	-0.179537784	-1.809543528	-0.599453708	0.333839294	0.49882384	0.294026482	0.126162054	1.548658658	2.176588797	0.479949435	0.2614044	0.288134694
Brevibacillus_agri	-0.179413588	-1.352205969	-0.341857623	-0.615522012	0.416985951	-0.365335829	-0.658051767	-0.41441399	-2.16919862	1.035480426	0.408148249	0.442321815
Bordetella_flabilis	-0.177719159	0.699163561	0.705956723	2.281480837	0.860285933	1.951547331	-0.154715094	0.091285379	-0.148953313	1.761780719	1.349851255	2.067036714
[Eubacterium]_cellulosolvens	-0.175196818	-1.60289918	-0.524171606	0.655368025	0.209257908	0.00137465	0.77756243	0.461192546	1.554200247	0.583427519	0.21561278	0.145937705
Hydrogenobacter_sp._T-8	-0.171000436	0.280487293	-3.670437899	NA	NA	NA	-0.768255329	1.176702306	0.517740384	0	0	0
Arthrobacter_alpinus	-0.170296252	-1.728173569	1.315976597	1.795970749	1.363622123	1.093133867	0.255004172	-0.124721951	0.26222126	-0.103362892	0.546256705	0.217110349
Geobacillus_sp._44B	-0.170081514	-1.611424775	-1.374475011	NA	-0.828352377	1.23026583	-1.074105738	-0.619422101	1.918267948	1.850060732	0.61725178	1.364846771
Paenibacillus_sp._Y412MC10	-0.169775878	-1.461819693	-0.110613806	2.310150927	-0.982512573	1.555004722	-0.353903191	0.49808306	0.218639481	1.963871017	1.930612059	2.785545775
Leuconostoc_pseudomesenteroides	-0.168819064	NA	-0.295073737	-2.068683006	-0.185866545	-1.350253363	-0.078061223	-0.059842818	0.076116478	0.462256655	0	0.180254744
Sneathia_amnii	-0.167304695	0.292215431	-0.946220285	-2.097154264	2.498103217	1.353713308	-0.860562181	-0.695991105	0.370681527	0.635602568	1.286514365	0.67502503
Thioalkalivibrio_paradoxus	-0.166319884	-1.623355483	1.056072537	NA	1.078855421	NA	-0.063804748	1.339585142	1.121923582	-0.933213808	-1.767260535	-0.413840847
Streptococcus_respiraculi	-0.165507185	-2.229447585	-1.6936558	1.73361562	1.297626999	1.07362715	0	0	0	-0.162226183	-0.738458908	0.363982575
Nocardioides_sp._603	-0.162129641	-0.569413674	1.624128667	-0.890205164	NA	-1.504959958	-9.36433386	3.806217175	1.169706336	-0.135164359	0.053837795	-2.900415438
Erysipelothrix_inopinata	-0.159284283	-2.104826373	2.141326471	-4.469719087	-0.865482574	-0.146250962	-0.807771283	-0.134794864	-0.97448782	0.063354724	0.763907489	1.442161782
Pseudomonas_sabulinigri	-0.157143237	NA	0.948122229	NA	NA	NA	-0.192090145	0.451736671	-1.27450719	-0.601562712	0.454586855	-0.699065516
Meiothermus_ruber	-0.155403924	-2.259689256	-0.947357019	1.165405798	1.217805152	0.842643672	-9.222246695	-0.66278165	1.174550718	0.662373333	1.207535753	-0.364969176
Devosia_sp._I507	-0.153225758	-1.496132824	0.189967083	1.896693304	2.551577872	2.243485498	0.246427517	0.560719701	0.615624967	-2.255729605	0.491254087	0.560342534
Agrobacterium_tumefaciens	-0.152813369	0.770402694	1.846586134	0.078804304	1.031637233	1.37790957	-0.061011755	0.684925659	0.095184523	0.087718002	1.002251271	0.117376001
Kosakonia_cowanii	-0.148917621	1.989268274	-0.766611467	0.686583405	NA	2.891825637	0.829054546	-1.12943041	-1.953494138	-9.054404697	-10.71119994	-16.18368771
Streptomyces_fodineus	-0.136229621	1.254085449	-1.609382866	2.746169421	2.191562651	1.50589093	-0.401027859	-9.910982942	-0.858662153	-2.235441425	-2.249185878	-1.201637922
Virgibacillus_sp._Bac330	-0.133523673	-3.825680786	0.569965322	-0.504076884	0.610327298	-0.768184325	0	0	0	0.635693116	-1.809011606	-0.166752934
Kineococcus_radiotolerans	-0.129559634	NA	-1.571308439	5.31517976	3.684369929	2.50589093	0.146246129	-2.395785269	0.477103669	-7.890768904	-1.0748477	0.648389907
Acinetobacter_soli	-0.129559634	NA	2.64137581	NA	NA	NA	0.645145137	0.412942009	0.393729155	0	0	0
Vibrio_metschnikovii	-0.128324097	0.676792353	4.93107277	NA	NA	NA	-0.659411046	1.725182038	1.055472965	10.52813174	7.29523629	9.073249875
Pectobacterium_carotovorum	-0.128324097	2.121368181	1.697603488	1.932026801	-1.119581616	0.791340513	0.057553537	1.126126768	1.061135791	-0.96608811	-0.218668159	-9.332410325
Reinekea_forsetii	-0.127890001	-0.574936926	0.702751538	NA	1.296870637	0.823491438	8.166278594	0	0	0.909634767	2.183597362	2.183597362
Borreliella_burgdorferi	-0.120827917	NA	0.39062816	NA	-0.553691039	1.63809692	-0.531423702	-0.359676894	-1.078256717	1.121412743	1.58735991	0.614013334
Paenibacillus_antarcticus	-0.120294234	0.64377558	2.858896701	1.001826362	2.113547997	3.519310407	0	0	9.624047875	-0.363240633	0.897204431	0.693409228
Corynebacterium_nuruki	-0.120038382	NA	0.382515704	0.280650388	-1.465276074	-3.799239234	0.302853787	1.491344485	0.506666581	-0.446544981	-0.467940444	-0.168498424
Sphingobacterium_sp._CZ-2	-0.119502343	0.791905536	1.015201259	-4.030047748	0.136345938	0.773556237	10.17990909	9.384097309	0	-0.094663334	0.051701464	-0.007989689
Gemmata_obscuriglobus	-0.117482845	0.267625044	2.484833014	1.868216008	2.623823638	3.325136524	1.815739563	2.305941065	-1.642021419	0.843473863	1.736074542	1.633027092
Aquaspirillum_sp._LM1	-0.112494832	-3.363172317	-2.218266189	-1.782817666	-0.815919066	-1.644674508	-8.076815597	0.033610291	0.642107653	0.075775374	-0.667848672	-1.0050829
Synechococcus_sp._BMK-MC-1	-0.108338682	NA	-3.378511623	2.489818596	NA	-0.140321288	-0.731070458	0.222112374	0.111276458	8.366322214	0	8.125842211
Tessaracoccus_flavescens	-0.108321513	-2.469705458	0.085065013	0.938617727	-1.077255606	0.637099692	0	0	10.20345813	0.815799348	-0.954883908	-0.014740307
Geobacillus_subterraneus	-0.10752728	-0.727286034	1.384511507	0.039770071	-0.984170072	0.017178732	8.409390936	11.0109756	0	0.881098779	0.561902692	-0.095324959
Streptococcus_anginosus	-0.103516369	-0.542775429	-0.957351044	2.465039134	0.387379933	0.449782605	0	0	0	0.227273536	-1.029056211	0.315640156
Clostridium_kluyveri	-0.101969372	-1.514127242	-0.018350001	-0.312123621	0.370620898	0.069038955	0.522187602	0.781907849	0.879436349	0.540986726	0.163018331	0.112884695
Sulfurimonas_denitrificans	-0.101146723	-1.601398803	-1.586090047	-3.345643769	0.094364487	-0.813355078	-1.104995548	-0.19935583	-1.391642225	-0.589555711	-0.358862308	-0.56240415
Streptococcus_cristatus	-0.101024779	-2.063927898	0.271443761	0.233881746	0.260911429	0.440272492	-8.624046047	-0.513620159	0.70095352	-0.248093141	0.240036009	0.474089091
Oleiphilus_messinensis	-0.097372712	0.319545086	-0.553912578	-4.148039038	NA	-2.949297885	0.616475766	1.357565931	1.207071417	0.160810466	0.890367299	-0.594315468
Bradyrhizobium_icense	-0.093982776	NA	0.576898226	2.90490141	0.144644735	-0.807737245	2.248112549	1.361208543	3.082727644	-0.941218503	1.211534199	0.164520017
Bacillus_sp._S3	-0.091893831	-1.446691908	0.264213708	NA	0.194134286	0.83829607	0	0	0	1.809717609	1.66820654	0.742929379
Acinetobacter_baumannii	-0.090151443	-0.33852609	-0.306442911	3.353328555	1.065691514	-0.379321958	-0.480356252	-0.293548771	-0.257838889	0.569491656	0.234182714	0.669453136
Arcobacter_mytili	-0.090131752	-1.335128534	-4.212528383	NA	1.160059648	3.733209702	-1.133130833	-0.539863387	0.006368884	-1.411147823	-2.461775562	1.420872891

Staphylococcus_nepalensis	-0.089538836	1.572098089	-6.849082061	3.930256	0.463075368	-1.174329773	-9.084082873	-9.084082873	1.644917498	-2.334264407	1.313229891	1.817053314
Roseburia_intestinalis	-0.087991912	-1.84088048	-0.735410846	-0.087623555	0.068914598	-0.372171102	-0.885956179	-0.477396921	-0.408836332	0.494639478	0.100468625	-0.054105655
Egibacter_rhizosphaerae	-0.087884251	-0.086620392	1.154598244	-2.965727983	0.281829086	2.079732036	0.912147329	-1.670192349	-10.05232351	0.071001179	0.331113248	1.740416883
Moorella_glycerini	-0.086894521	-1.682534155	0.224405712	0.398730438	-0.851891874	-0.651326732	1.514948037	0.296722212	0.435832705	-0.067588226	0.356800078	0.319900734
Nocardioides_sp._JQ2195	-0.086271452	-0.739178317	-0.812130344	-0.178538132	NA	0.809413234	8.409390936	8.110425888	10.15020424	-2.122484007	1.442040806	0.701304044
Gillisia_sp._Hel1_33_143	-0.085696581	-1.648895107	0.859911123	NA	0.504055301	0.18564169	1.42208209	-8.624046047	-0.878909578	3.785888754	2.702940779	2.550812736
Bacillus_smithii	-0.08519465	-1.685218981	-1.374146431	-2.00728572	-1.839152886	-0.972940141	1.733314165	-0.69468444	-9.076815597	-0.212740878	-0.609904815	-0.242172323
Streptococcus_agalactiae	-0.083911092	-1.418076124	-0.486210831	1.823685229	0.166923892	-0.040592009	-2.09199347	0.219095009	0.074929868	0.409715268	0.334440811	-0.323091061
Marinobacter_fonticola	-0.082694684	-1.556587116	0.277237501	NA	NA	NA	0	6.682671608	6.15711649	-0.549189898	-0.308409771	-0.711042249
Geobacter_sp._M21	-0.081117994	-1.853974451	0.734996044	2.329353947	1.548077031	0.69177682	0.336616838	-0.890651509	0.275756136	-0.067417463	0.961447781	0.260013621
Weissella_sp._HDW19	-0.078627243	-2.23843874	-1.502500341	NA	0.011714799	-1.520209708	-1.661340508	-11.07073144	-11.07073144	1.21826634	2.420507715	2.365871424
Treponema_sp._OMZ_804	-0.077647942	-2.369642717	0.212578925	0.296984622	-0.278018853	-0.015335364	0	10.18555687	9.999492713	0.115719414	0.809474476	1.555241153
Raineyella_sp._CBA3103	-0.076049806	-1.520588588	-1.041042233	-3.337096076	1.153453184	0.922207036	0	0	6.15711649	0.909787089	0.807493442	1.822049617
Bacteroides_zoogleoformans	-0.075570601	0.802188517	0.275562458	-3.476584929	-2.516988175	-2.444707391	-0.332407046	-0.21552429	-0.394007028	0.056708896	-0.143938617	-0.238871388
Microvirga_sp._17_mud_1-3	-0.07490897	-0.087545795	0.894003353	-0.861198505	NA	-0.025943267	0	0	9.052320788	-0.014666625	-1.342010554	-0.021562906
Terriglobus_roseus	-0.073401473	0.477154537	2.934226066	0.704680675	0.508164618	1.623335204	0	0	0	0.80196884	1.374188103	1.336943205
Comamonas_serinivorans	-0.073321992	NA	-2.276667691	0.770215176	-2.526545814	-1.811439654	2.722606478	1.004750437	2.7881823	-1.311522459	-3.14193433	1.24469199
Cupriavidus_metallidurans	-0.073146116	-0.763468963	-0.28595096	-1.499598958	-0.243072674	0.89451805	1.030589007	0.857127847	1.738204406	-0.462824888	0.24458134	-0.18047118
Aminipila_butyrica	-0.072087615	-0.791210659	-0.667840206	1.281978961	0.670729603	0.474272776	0.261160852	0.599779748	0.268286795	0.313379966	0.236548659	-0.333138687
Halocynthiibacter_arcticus	-0.066791319	NA	-3.315162807	1.024423474	0.865484333	1.480391237	0	0	0	1.406832561	-0.898310674	2.199460661
Defluviicoccus_vanus	-0.059370747	-1.887834696	0.274948236	NA	0.866084379	1.77413077	1.302601196	0.406093273	-0.237649239	-0.604980394	0.010843261	-0.914543224
Clostridium_sp._MF28	-0.05930485	-2.870090677	NA	NA	NA	NA	0.69441368	0.149039763	-1.042619893	0	0	0
Sediminispirochaeta_smaragdinae	-0.056570241	-2.504783174	0.811704774	1.945328296	-1.261254041	-0.922483069	-0.050894686	0.006900716	0.077740223	0.862850789	-0.238195637	-1.049886787
Oenococcus_kitaharae	-0.054283869	-2.578371443	0.671129439	-4.425312638	-3.117780764	-0.318420273	0.006326984	-0.246216056	0.59689668	0.275940429	1.294800196	3.437730045
Anaeromyxobacter_sp._Fw109-5	-0.054134457	-2.047669793	-0.533831414	-3.936094921	-0.525380466	0.372599115	0.443743596	1.044953069	2.989156754	0.219287794	-0.252962996	0.367869195
Stappia_sp._ES.058	-0.053475945	NA	1.012583129	-1.304002813	0.691265927	-2.63133933	-5.359702785	-2.6016597	-3.812722909	0.389813753	0.464979145	-0.098032314
Coriobacterium_glomerans	-0.052648714	-1.04591685	-0.139503554	-1.190009071	0.384148406	2.424073296	0	0	0	0.091480793	0.632043677	1.505874491
Bifidobacterium_actinocoloniiforme	-0.052603851	0.542299453	0.489742812	-1.499371232	2.310749195	2.824792486	0	8.627369725	7.157114468	-0.733820182	1.203070661	0.011674208
Anaerostipes_rhamnosivorans	-0.049717402	0.67333356	1.000103623	0.167176608	-0.001744321	-0.296614958	1.208556033	1.372879317	1.429513347	0.666260453	0.610147267	0.502182841
Altibacter_sp._ALE3EI	-0.049716357	-1.760059745	-0.499568757	-1.298999385	0.795049627	0.744628623	0.598200855	0.303101638	-0.065186524	0.271382705	0.247639597	0.386106816
Actinomyces_howellii	-0.046936314	-1.482646332	-2.122678876	-3.74176658	-0.743548786	-0.193786722	0.178134552	1.238615271	-1.065825552	-0.087907183	0.782257912	-0.243460728
Bifidobacterium_lemurum	-0.044174077	-2.054573571	-0.861113714	-0.532190992	-0.356778307	1.096793561	0.349963913	1.173019545	1.39249289	0.344713516	0.170216721	0.176117624
Pseudopropionibacterium_propionicum	-0.04285891	-2.070447844	-0.094507948	0.477797265	-0.675690446	0.905421272	-0.3862176	-0.442070306	-2.395379704	0.223902108	1.126652853	-0.050030104
Alkaliphilus_oremlandii	-0.042146305	-1.078281949	-0.959987496	-0.200469499	-0.529660508	-0.1330659	-9.105670222	-9.105670222	-9.105670222	1.368919815	1.385921711	1.514477714
Candidatus_Pelagibacter_sp._FZCC0015	-0.041762524	1.848214688	0.328967553	NA	1.776805859	2.933572638	0.352733051	-11.36929744	-1.140478745	1.159029663	0.939686772	-8.725649145
Treponema_denticola	-0.039257259	-1.918455506	-0.221978496	1.961686226	0.295497629	0.277121178	-11.58402294	-2.1920963	0.788249035	0.153299693	0.435269575	-0.193315369
Kushneria_konosiri	-0.039064473	-1.193218533	-0.000590325	0.162586317	1.450884367	-0.388296198	-3.511623233	-1.662926698	-1.257499616	0.71402939	0.220717796	0.347809381
Planococcus_rifietoensis	-0.038550306	-1.090106916	-1.133648343	NA	-0.168764146	1.253713778	0	0	9.672264444	8.442943496	9.432681779	10.21270308
Pantoea_vagans	-0.037124026	-3.30621452	-0.292590986	1.183163303	1.629966866	-0.261759244	-0.464001483	0.124003437	0.476785714	1.603223678	1.315294427	0.764468831
Geobacter_uraniireducens	-0.035997096	-0.323769045	-0.136322405	0.093698713	0.753392771	0.649657559	0	0	0	0.930042223	1.097106373	0.991825922
Bifidobacterium_asteroides	-0.034396106	-1.043616108	-0.390971939	1.670194722	-1.113499181	0.509800605	0	0	0	0.170325402	0.170699337	0.567347494
Clostridium_drakei	-0.03390809	-0.801905675	-0.165433878	-0.182769535	0.44317798	-0.305626283	8.502740328	0	0	-0.957178027	0.662803419	0.227262818
Hafnia_paralvei	-0.033900407	-2.023846742	-3.682809824	-1.341201618	NA	0.099041515	1.184336005	-0.541412141	-0.981500396	8.876516947	9.54432116	0
Streptococcus_salivarius	-0.031138512	-0.604902806	-1.261714801	1.86318331	1.664169769	0.91753784	-0.14287637	1.112486059	1.827426734	1.320074177	0.538570117	-1.835783942
Amedibacterium_intestinale	-0.028570637	-1.717422078	-0.796353085	0.739430561	0.266074288	0.107155836	0.411340468	0.677736159	0.639053784	0.299405841	0.277613113	0.350135641
Rubrobacter_xylanophilus	-0.026065056	-1.905037252	-0.675187017	1.887229288	1.366847509	2.254779136	-0.544346043	-0.303154338	-0.205781487	-0.372610511	0.822766977	0.674146065
Legionella_hackeliae	-0.024523898	0.073530752	-0.744168004	NA	-1.076541555	0.18928383	2.290548265	1.209833891	1.488975695	2.038860209	2.161649887	1.032272549
Gimesia_benthica	-0.020316805	-0.90577023	-1.008092421	NA	0.123028917	-1.537928967	-10.00332114	-1.892895248	-1.846201613	1.406117943	0.867237254	2.163067912
Lactobacillus_fructivorans	-0.019982245	-2.2193308	0.665052396	-0.27368632	1.670631753	-0.85896642	0	0	9.913704737	0.653738687	-0.320686068	-1.162363123
Longibaculum_sp._KGMB06250	-0.019556006	-1.691644523	-1.408272353	-0.12165057	0.904207713	1.040863784	-2.704753421	-1.873507352	0.674119822	0.58089653	0.069324589	-0.029565633
Chromobacterium_violaceum	-0.014580233	-1.358317475	-1.206860443	-0.354171831	2.196287822	1.378789269	3.458119611	3.332947061	3.001806687	1.960620159	-0.036147927	-0.713361577
Massilistercora_timonensis	-0.010250521	-1.85685819	-0.759709264	0.915209157	0.145646741	-0.108568951	-2.383786814	-0.122663699	-0.716489414	0.419873632	0.157363614	0.004319456
Sphingobium_sp._SYK-6	-0.010231923	-1.933433612	-0.90886157	-3.045696779	-3.15532127	-0.312254251	0.271624746	-0.394705818	0.024509682	0.17047286	0.257711099	-0.8811301
Rodentibacter_sp._DSM_111151	-0.007442646	-1.375662259	-1.614368014	6.381728336	1.670322406	0.58221189	0	0	0	9.904885961	11.09055575	12.18508293
Lachnoclostridium_sp._YL32	-0.00447912	-1.835726629	-0.802218028	0.356567444	0.068922741	-0.216075221	0.025855873	-0.188352185	3.125897321	0.553911451	0.10657696	0.048782727
Curtobacterium_sp._Csp2	-0.002418601	NA	NA	NA	NA	NA	-8.10567284	0.460727555	0.051446682	-1.149542056	-1.624601482	0.536821162
Corynebacterium_flavescens	-1.44E-15	-2.195816821	-0.224099416	0.491650171	1.288909867	1.406702851	1.37106838	2.218734659	0.644516301	0.66304599	0.637800836	1.359359076
Ruminiclostridium_cellulolyticum	0.005672575	-1.32966727	-0.472663087	-2.854223589	-0.54545017	-0.495471627	0.543292169	0.321446873	-0.522875545	0.798597425	1.144716898	0.623660581
Lawsonella_clevelandensis	0.007079485	1.404097338	-2.170939198	0.692285764	-0.221916363	-1.025943267	1.513832671	-8.04736934	-8.04736934	-0.563609233	0.7415474	-1.576384941
Snodgrassella_alvi	0.012212189	-1.988893748	-0.404373859	0.491137883	NA	-3.147122844	0.706197623	-0.115669953	-0.180606892	-1.204024423	0.673439392	-0.551069262
Pseudorhodoplanes_sinuspersici	0.0131155	-0.891500668	-3.329227616	NA	NA	NA	-8.552496194	-8.552496194	-8.552496194	9.271463028	5.472487771	0
Arcobacter_pacificus	0.014993317	0.723388871	0.042653677	NA	2.166982803	-0.132019915	-0.75061877	-2.228548487	-1.111425226	11.47269084	11.09671515	12.16469641
Tessaracoccus_flavus	0.0156348	0.246254941	-0.807755994	NA	-0.767923949	-0.045295241	-0.326088935	-0.333773081	1.506248159	1.167575993	0.603273363	-0.641953068
Yersinia_enterocolitica	0.016158915	4.821924384	1.43720965	-1.911716502	0.315667499	-1.209162663	-0.157069265	0.095208093	0.007882283	0.840073101	2	0.773087002
Streptococcus_canis	0.021288409	-2.137202148	-0.588996553	3.539005434	0.086670004	0.037435941	0.28077402	0.083500194	-0.421777766	0.22382737	-0.501141562	0.267072382
Pseudanabaena_sp._PCC_7367	0.02266706	-0.947581052	-0.92657634	-0.954535968	0.528255799	0.245952806	-0.346416635	-0.144705011	-0.368033061	1.897495416	4.442233609	4.53675647
Clostridium_acetobutylicum	0.025448147	-1.087868934	-0.867931862	NA	0.077188005	-0.411130522	1.63176014	2.83332584	1.029148339	1.25959975	1.871478712	1.881278601
Polymorphum_gilvum	0.026158142	-1.134118289	0.279398465	-0.811304508	0.016557842	2.912139947	-0.665166413	0.079222433	-0.619812957	1.119205244	0.593126011	2.10103209
Candidatus_Pelagibacter_sp._RS40	0.026402999	-1.074200942	-0.327649299	NA	NA	NA	-0.626196233	-2.727699498	-0.277225411	0.179796896	2.529436672	-8.186525318
Alienimonas_californiensis	0.028142382	-2.394110705	-0.021408042	-0.449549483	0.338546607	1.050409037	0.766216195	0.533225529	1.296084125	0.762437203	-0.002022811	0.496513919
Burkholderia_ambifaria	0.0302696	-2.501876202	-1.530445355	NA	NA	0.463504306	9.602277592	8.110425888	9.624047875	-3.332881803	0.165733795	-1.608334429
Pediococcus_acidilactici	0.033743153	-2.360731825	-1.878457726	0.451077995	-1.292442963	-1.077579041	-0.20340974	-1.978596675	-1.066190068	1.655252719	1.284788958	0.017167283
[Ruminococcus]_gnavus	0.037399437	-1.605228248	-0.871021427	0.816862043	0.454658056	0.324344024	-0.273621893	0.611637479	0.631883739	0.563914523	0.133476336	-0.137529919
Nonomuraea_sp._ATCC_55076	0.037428185	1.01327352	0.155011617	2.987425307	4.125701418	3.923107279	-8.637284195	-8.637284195	-8.637284195	0.489295246	0.024937902	-0.408315674
Peribacillus_muralis	0.039520844	-2.170453762	-0.366072251	0.872511434	0.249195498	0.32674052	-0.951758658	-0.924707574	0.044935973	0.747115656	0.073749679	0.404390255
Planococcus_plakortidis	0.040470456	-0.566197818	0.439884228	-2.625661816	1.993212017	1.666867184	0.9565638	0.205983211	0.169626078	-0.639591524	-0.627500943	-0.154171205
Gallibacterium_anatis	0.041789506	-2.409323848	-0.46231936	0.875246941	-0.904599997	0.032638817	0.36882916	-0.059192986	0.249709315	-0.177193162	0.915243307	1.039840525
Octadecabacter_arcticus	0.042019052	-2.024572042	-1.399459357	-3.939137774	-3.385471404	-0.620929566	-2.645355813	-2.167308516	-4.61752234	2.139904169	0.729554172	2.371254268
Campylobacter_iguaniorum	0.043021277	-1.63085168	-0.205325686	NA	-0.355689818	0.034168824	-3.11809747	-2.287235157	-2.17544814	0.104636678	0.060770781	-0.210239662
Streptomyces_kanamyceticus	0.043721377	-2.234204156	-1.035956878	NA	NA	-0.649869193	9.029913869	0	0	-1.001776755	-1.161590049	-0.840657585
Klebsiella_pneumoniae	0.044309599	4.570503311	1.236086654	-1.689403677	0.113900624	0.836969409	-0.651878101	0.519091254	-1.346964948	1.019776535	0.561674144	1.641064689
Burkholderia_mallei	0.045018916	NA	-0.69412105	-0.419066642	-0.750498923	-0.781736968	-0.19526421	0.186139494	1.303191641	-0.791208639	-2.18168552	1.689340428
Massilia_lutea	0.045116092	0.068904579	-2.046572638	0.222068838	0.926833587	-2.573682576	8.876516947	0	7.157114468	8.714245518	10.46488265	7.63420844
Dehalobacterium_formicoaceticum	0.047479263	-1.911027841	-0.767868105	-0.295925768	-0.002929255	0.250600551	-0.744181711	-0.323747662	-0.408472136	0.703000934	0.613635267	0.33906048
Ezakiella_massiliensis	0.047977507	-1.982300631	-2.298589242	-0.656741842	2.976562285	2.674153268	-1.095261033	0.276449617	-0.18897557	0.786558057	0.594075994	-1.133550799
Azospirillum_thiophilum	0.049695902	NA	-1.020367798	3.965481665	0.601082166	1.346571833	1.310621551	1.513268072	0.682048434	-2.072707169	1.781282163	-1.837329106
Streptomyces_antimycoticus	0.053893008	NA	0.582186485	NA	2.585548843	NA	0.701426325	0.929669738	1.153963577	1.553125682	1.099535674	-7.807354922
Xenorhabdus_poinarii	0.054940435	-2.768557577	-0.387761093	NA	-0.42241318	-2.787227088	0	8.110425888	8.598897393	0.897945507	-0.171777047	0.603012978
Bacillus_sp._CS13	0.055305144	-1.675537084	-0.793951885	NA	2.279797406	2.44507206	-10.17311494	-0.086620406	-1.692231858	-0.324883436	0.327300265	-0.303638951
Synechococcus_sp._NIES-970	0.056269122	-2.13436956	0.010693381	-0.755125515	0.84489391	-0.316804824	0	0	6.15711649	-0.64518097	0.107442799	0.350176968
Thermomonas_sp._SY21	0.056532899	-1.294030168	-0.133113675	NA	NA	NA	2.035444641	1.693765712	3.745785295	-8.087462841	-8.087462841	-8.087462841
Dermacoccus_nishinomiyaensis	0.057181661	-1.005844822	1.073903362	0.258778182	-0.09212898	0.041567959	-1.071099321	-0.003700049	-1.103112317	0.048270729	-0.919221173	0.331709498
Polaribacter_sp._SA4-10	0.062541466	-1.241838428	0.157541277	NA	0.941604691	1.636413385	-1.395371415	-0.002555083	-0.041923934	1.831711938	1.708550798	-0.735769843
Streptococcus_pluranimalium	0.063321092	-1.642714823	-1.28002737	-0.336340753	1.325251354	1.849430285	0	8.382131157	0	0.908948859	0.545777332	0.426104686
Vibrio_parahaemolyticus	0.064053371	1.755818184	1.471897591	-1.095884435	NA	-0.523128839	-2.949354835	0.450968392	-2.336557513	0.440754475	0.003013595	-0.94839615
Mesorhizobium_sp._NBSH29	0.065571206	0.281422696	0.803582707	NA	NA	NA	-2.713035539	-2.34407438	-1.782487631	0.487166046	2.536889643	1.923012287
Lacrimispora_saccharolytica	0.066246626	-1.782535776	-0.52737248	0.556533665	0.519620087	0.422883495	1.012173487	-0.170365038	0.898714918	0.554067636	0.326292071	0.213352014
Sphingobacterium_sp._dk4302	0.069394197	-0.735874709	-0.000311833	-1.427386844	0.218827217	0.846001477	-1.359110248	1.293332121	0.637938922	1.464315595	1.7627271	0.691588107
Anaerostipes_caccae	0.072119736	2.804464234	2.808920959	0.814842738	0.108430463	0.060237299	-0.696601879	-0.475010896	-0.663960159	0.433732493	0.254599988	0.231858462
Kurthia_zopfii	0.074000581	0.053009685	0.248904077	1.68574875	0.462626958	1.859193854	0.775566075	0.006968851	-0.071613114	1.619667827	-0.236390092	1.756096508
Hymenobacter_sp._17J36-26	0.074074494	-0.446630472	-0.343618033	NA	2.194341882	1.463826063	0.293462158	-0.041897766	-1.457065409	2.633235394	1.689568972	1.0682488
Acetobacter_tropicalis	0.078951341	-2.468217537	-0.147430364	NA	NA	NA	-0.804454404	0.916266502	0.513038082	0	0	0
Gottschalkia_acidurici	0.079010668	-0.879714679	0.014741298	-0.456583256	0.900899343	0.914301864	-1.347296661	-1.432565811	-0.773246731	1.243485191	0.671985259	0.434290554
Bradyrhizobium_erythrophlei	0.079116863	-1.145092603	1.36879543	-1.925411443	-1.690890345	-0.924108952	9.147899389	9.889020195	12.38703721	1.065840428	1.322549812	0.852252895
Peptacetobacter_hiranonis	0.080872002	-1.34917756	-1.248538264	-0.642823896	0.327903315	0.660729032	-0.858856097	0.039096419	0.599464455	0.846738279	0.916016836	0.877210103
Corynebacterium_sp._2019	0.08305574	-1.384723222	1.229701848	NA	NA	NA	-0.508001178	-8.637284195	3.59071373	0	0	0
Paenibacillus_thiaminolyticus	0.086706628	-2.466596191	-0.218843403	0.228068993	0.921213465	0.166745222	1.464405132	0.976992323	-0.728241116	0.399311203	0.013645881	-0.321885516
Bifidobacterium_subtile	0.086958688	-1.579266733	-0.840816753	0.717201509	0.588971103	0.88879406	0	0	0	-0.280561667	0.169508421	1.13274725
Mixta_intestinalis	0.088035202	-1.913702319	-1.21647584	NA	-3.192700736	-1.435572103	0	0	0	-2.536466964	0.123606777	-0.521636565
Corynebacterium_callunae	0.088437965	1.229481846	2.813265193	0.195130341	2.174707624	NA	0	0	7.157114468	-8.725649145	2.646127499	1.868675459
Enterococcus_faecium	0.089483269	-1.575175391	-0.688205731	1.558680294	0.74762741	0.987660693	1.566577727	3.1537156	3.301474175	0.412692606	0.34764976	-0.053639202

Paenibacillus_brasilensis	0.090147059	-0.644888986	-0.589613531	1.156092728	-0.36099766	1.49625156	-1.853048456	-3.489493059	0.698447306	-0.468425162	0.016050596	-1.268670269
Arcobacter_butzleri	0.091630475	NA	-1.351249967	NA	NA	0.603612558	-0.736235004	-0.07031372	1.132913372	-0.641316855	-0.094782401	-1.305190154
Ectothiorhodospira_haloalkaliphila	0.093239324	1.638914022	0.029764313	NA	1.590003505	0.845145474	-10.36831001	0.390521136	-0.304914933	-2.716207034	-0.326698064	-0.094014909
Cellvibrio_sp._PSBB006	0.093340793	-1.812192397	-1.547133685	NA	1.837127153	3.186585572	-8.799135887	-2.116464279	-8.799135887	11.17200864	10.97393409	10.19639311
Thermus_brockianus	0.094546072	-0.53111152	0.023620896	1.072692014	0.757356006	1.729425073	-1.330182422	-0.885418658	0.266511409	0.258232241	0.93564843	-1.786607623
Paraburkholderia_caffeinilytica	0.095937031	-2.894009304	NA	NA	NA	0.047205843	-0.719903497	-0.691566773	-0.236998791	10.12240095	6.470320477	0
Polaribacter_sp._BM10	0.095937031	NA	-1.664763212	NA	NA	NA	0	0	0	-8.681825211	-8.681825211	-8.681825211
Pigmentiphaga_sp._H8	0.095937031	NA	0.958433507	NA	NA	NA	-2.130844025	0.199281806	0.463223094	8.27301694	0	10.4415644
Thermosynechococcus_sp._NK55a	0.095991913	NA	0.543119694	NA	NA	NA	0.25277776	-0.230522222	0.107755096	-8.809501266	-1.587112065	-8.809501266
Acidipropionibacterium_acidipropionici	0.096352225	-1.975947845	-0.25332522	3.480843675	0.844865405	0.987593987	-0.475917571	0.525276622	0.781577389	0.391189497	-0.096418205	0.691662467
Enterocloster_clostridioformis	0.098787022	-1.816475007	-1.058451095	0.123274064	0.142787896	-0.01907493	-10.25158892	1.506105263	0.021355725	0.391624334	-0.00391771	0.016076274
Morganella_morganii	0.099221532	-0.580878609	-0.384271269	-0.436744441	0.889524819	1.620430352	-9.177646265	-9.177646265	2.08172039	0.185796838	0.398309285	0.68578687
Sporomusa_termitida	0.100532036	-1.226410396	0.048475073	0.83195001	0.328076449	-0.160155808	8.409390936	10.36831001	9.819597937	0.900572931	0.923608754	0.51245238
Roseburia_hominis	0.101674906	-1.773088093	-0.519735723	-0.054136086	0.136310082	-0.315573872	0	0	0	0.54256919	0.214667564	-0.022386693
Gemmata_massiliana	0.10373741	-0.11372858	0.913231369	NA	2.346225257	2.620958719	-2.391369888	-0.528599926	0.498597684	-1.229274701	-0.460075439	-0.236673114
Erwinia_gerundensis	0.104391391	0.559637527	NA	0.672979036	-1.900690295	-1.166257967	0	0	0	-1.54424	1.093752334	0.702933966
Paenibacillus_durus	0.104724564	-1.858850118	-0.054267191	0.069636958	-0.382973798	0.173405752	-0.561550998	0.437810098	1.436193828	0.498507426	0.065444628	-0.121220041
Helicobacter_suis	0.105674042	-2.069304187	-0.490429016	NA	1.013144267	-1.469861524	-1.207618779	-1.855401492	-0.529545494	0.699061734	0.069089766	1.288841149
Heliorestis_convoluta	0.108878312	-1.670210216	-0.336866232	0.337634615	-0.321619684	0.532190413	-0.322134442	0.719572294	1.00773018	0.390471163	1.352088954	1.959541352
Bartonella_sp._HY038	0.111112025	-1.604158498	0.816973959	NA	-0.01573456	-0.559662752	-11.22991232	2.161035825	0.373208623	3.405335411	2.856601259	1.656229494
Streptomyces_seoulensis	0.112613059	-2.175592541	-0.428524096	2.17846739	1.7811788	1.969838029	-0.979105128	-1.78526527	-1.325732559	-0.032161925	0.215395915	1.818218641
Mycolicibacterium_sarraceniae	0.117951245	-2.819772073	2.788948254	3.803824802	4.477622946	4.138484806	-10.85967455	0.340445505	-0.700837679	0.299259021	1.202212711	-2.001206782
Paenibacillus_donghaensis	0.12019487	0.309334975	-0.815906668	1.565442872	0.959368256	1.27514795	-9.105670222	-9.105670222	-9.105670222	0.183392394	0.698633905	0.340795465
Pseudoarcobacter_acticola	0.121904627	-1.421248128	0.621829981	-0.104219752	1.047909006	-0.555339233	-1.569605812	-4.381402717	-0.582724665	1.058229914	-0.120420087	1.137728245
Thermochromatium_tepidum	0.128293401	-7.046126393	1.551146837	2.498369627	2.965623231	2.63691458	0.528784882	0.873240908	-0.469205483	1.254813899	1.965325778	1.650700298
Blastochloris_viridis	0.133071277	NA	2.428116078	NA	NA	-0.886203885	0.98433448	1.930229245	2.549398775	2.880902267	1.610489018	2.036013229
Saccharibacillus_brassicae	0.133869151	-0.824665935	-0.361618249	1.088539789	0.743231381	0.12941757	-0.437587132	0.27859235	0.218484572	-0.155698184	0.490595036	1.132769905
Clostridium_argentinense	0.139027444	-0.894366134	-0.343820701	-3.110079923	-1.298063624	-0.854921455	-0.646190613	0.733727777	-0.063801918	0.848812223	1.665507926	0.819193189
Clostridioides_difficile	0.140234689	-1.560589599	-0.590624923	0.813845097	0.438510967	0.631211305	-0.620870211	-9.936637939	0.779950055	0.490381491	0.129695033	-0.13781413
Chryseobacterium_sp._7-3A	0.140283028	-1.665119668	-0.306972997	1.068099092	0.450939307	-0.634735093	8.157119522	9.384097309	8.548995253	0.049159959	0.392712609	1.205671252
Cryptobacterium_curtum	0.140673413	-1.467686126	0.692699148	NA	1.197794524	0.109647454	1.014500394	0.534833161	1.102834069	1.54204132	1.550490824	0.894179544
Paenibacillus_xylanexedens	0.142279163	-0.289817481	1.216385355	1.975519554	0.701918647	1.489347197	-0.681175666	1.109408212	1.624298925	2.058733825	1.518165443	0.955356085
Chromohalobacter_salexigens	0.143124942	-0.046774759	2.440153278	0.911385613	-0.331552514	1.6123274	-1.751457534	0.228166737	-0.719447833	-9.201307944	-1.832077043	0.997542223
Desulfosporosinus_youngiae	0.143659283	1.384858104	0.825346781	-2.790741658	-3.439780745	-2.349436218	-1.625660424	-0.358204268	-0.251518577	0.545049961	0.256771723	-0.338197607
Pseudomonas_sp._ABC1	0.145565796	-1.839327312	-2.158605828	-0.901000246	-0.219882949	-1.161410547	0	0	9.944646183	0	8.86005338	8.539158811
Synechococcus_sp._HK01-R	0.147452001	NA	-0.731296844	NA	0.262634269	2.60916337	0	0	0	0.721456523	1.859419128	-0.437446293
Pantoea_sp._At-9b	0.147879996	-1.806474017	-0.836436158	0.699057106	-0.052901431	-2.281112543	0	10.35434857	9.477205768	0.539531942	-0.815130181	0.243616503
Desulfovibrio_magneticus	0.149083119	-2.000209496	-0.548072043	-1.670797797	-1.132286692	0.815924127	0	0	0	0.560707376	0.770651923	-0.614651746
Lonsdalea_britannica	0.149502284	-0.312543506	-0.058380774	3.186379656	-0.49683159	-1.82769281	-0.326125391	-9.987903489	-0.846958716	0.417656033	2.615733793	1.163380662
Streptomyces_bingchenggensis	0.150050798	-2.64001071	-0.422931414	NA	NA	NA	-0.266175525	-1.095996505	-0.986460703	2.511560658	3.535092042	2.895397842
Melissococcus_plutonius	0.151117733	2.687445458	2.181678183	0.449952575	0.957472996	0.858327764	-3.097893674	-0.785363334	0.240800692	1.198538253	0.919918897	0.82512104
Romboutsia_hominis	0.15418325	-0.366059435	-0.676124058	-0.611419541	-0.586294393	-1.28092252	0	10.56467349	6.15711649	0.251024267	1.091293812	1.083894919
Streptomyces_subrutilus	0.156616992	-1.705087899	-0.971164362	NA	NA	NA	-1.533024926	-0.7593953	0.326078357	1.979356093	-8.681825211	1.13195598
Nitratiruptor_labii	0.157047092	-0.496479695	-1.358589338	-0.104307745	0.774262347	0.292797486	-1.470090722	-0.489348993	-0.844948659	-2.39192745	-0.719707687	-0.445173279
Meiothermus_silvanus	0.158080929	-1.902480455	-0.137371798	2.056113958	-0.014183645	-0.38658304	-0.260354282	-0.822692327	-0.370579056	0.172845762	1.471576833	-0.361233632
Nocardia_otitidiscaviarum	0.164386818	1.168081301	1.853972711	NA	NA	NA	0	9.384097309	0	1.258533014	1.595412194	-0.202308175
Cobetia_sp._L2A1	0.165759527	-5.049008902	-1.659721079	NA	1.77909107	0.78599393	1.796922982	-7.757316664	-1.600200174	-0.587814036	-9.399455246	-9.399455246
Gramella_flava	0.167369297	-0.096347396	-0.439934372	-0.288293963	2.071532715	1.543363532	0.702321773	0.63530316	-0.709857933	0.432590559	0.492841441	-1.196534575
Ornithinimicrobium_sp._HY006	0.17319366	-2.833490653	-2.834980271	NA	0.593369488	-0.590619692	1.138137401	3.005661404	1.720448453	0.935415367	-0.078534315	-2.497928219
Thermovirga_lienii	0.174936912	-4.188720305	-1.989800717	-4.745735804	-0.513324627	0.802851188	0.224960837	-0.211731607	0.618882303	0.424564106	-1.139508328	0.915803345
Cellulosilyticum_sp._WCF-2	0.175445071	-2.921490523	0.025760432	-1.08980568	-0.571295072	-0.310703202	0.109750182	2.30697923	1.492845897	1.750904647	0.588492713	-0.351634887
Lactobacillus_suantsaii	0.18191018	-3.127236479	-2.156561375	2.186872577	1.172961283	2.220437147	-0.061733159	0.50144859	0.797960446	0.355735558	0.577352067	1.367673296
Novosphingobium_aromaticivorans	0.184424571	-3.73617094	1.631113905	NA	-1.347019076	-0.438442104	-0.415370596	-2.20038764	-1.632766784	10.1581931	8.979187942	9.801978283
Lactobacillus_kefiri	0.187085553	NA	-0.280900827	-0.234770554	-1.701088558	NA	-0.820831154	-0.30783844	-0.983833769	-1.139088425	-3.42645503	-0.489716275
Advenella_mimigardefordensis	0.193935047	-1.177894019	-0.039384977	0.102131762	0.069291557	-0.13625227	-10.81814275	-0.107026464	-1.873498033	-0.105616195	0.529680065	0.66505589
Acidaminococcus_intestini	0.194299319	-1.435016461	0.047142983	-0.460574168	-0.040808246	1.095328442	-0.484718085	0.053123371	1.275512075	0.652374451	0.420498026	0.526804815
Pantoea_agglomerans	0.197252569	-0.691917704	-0.509493712	2.53201033	-1.009360728	-2.47109768	-0.68170626	-0.398463399	-0.727693315	3.34175541	1.867948063	3.254587333
Ligilactobacillus_ruminis	0.201476573	-1.215669286	-0.858973045	0.070231296	-0.826930112	-0.490763297	1.187939031	0.909213717	3.037098377	0.814065728	0.455568128	0.125862682
Rhizobium_hidalgonense	0.201766468	-0.422989623	-4.750927817	NA	NA	NA	-1.291741609	-0.691955786	0.601452473	0	0	0
Enterobacter_sp._N18-03635	0.202648599	0.913245619	0.210033483	-0.221412036	1.906890596	1.671936597	-0.191906512	-1.314759747	-0.351829907	1.350863037	1.741679918	-0.530512206
Pectobacterium_odoriferum	0.204229638	-2.230954435	0.27437195	NA	-0.978752266	-2.442408471	0.17824597	-0.525761734	-0.264319644	0.871551635	1.464410758	0.684735635
Phocaeicola_dorei	0.204478956	2.346851532	0.945157977	-5.862847972	-5.855671299	-5.770310855	0	8.110425888	8.48088308	0.538480571	0.04931189	-0.321699441
Diaphorobacter_sp._HDW4A	0.208119522	-0.2955611	-0.884814702	-2.061622836	1.524406352	0.897247288	-0.001794937	-1.008793016	-1.297041657	0.055674631	1.638631332	-0.027705478
Emcibacter_congregatus	0.208362932	-1.859492304	-0.124226934	2.387486552	0.505192328	1.705119448	-0.664662196	-0.083779936	0.358508618	0.679690713	1.010298727	0.684345802
Streptomyces_sp._SM18	0.211330712	NA	-0.936975713	NA	1.676971871	1.902661882	0	8.110425888	11.09251931	9.379378367	9.420661347	6.930737338
Sterolibacterium_denitrificans	0.211422285	0.744684879	-1.19123149	-2.790288889	NA	-2.816924016	-1.803763451	0.883521241	1.021814349	0.234165439	-0.803186999	0.007985436
Cylindrospermum_stagnale	0.21592181	0.193904713	-0.763867051	-1.395436765	-0.331306427	1.291318408	1.063037566	-0.378314569	-1.159964369	-0.026114527	-1.492792069	0.340839166
Polynucleobacter_acidiphobus	0.21602234	-3.85263861	-2.996568048	-2.428383525	-0.079885709	-1.937603463	0.254254784	-0.859042984	-0.778180725	-0.221190315	1.592165187	1.031422751
Sulfuricurvum_kujiense	0.221484209	-0.664157159	1.379651901	0.803868746	2.100309237	-0.03921674	0	9.426646347	6.15711649	0.066013878	-0.552826732	0.379989004
Shewanella_polaris	0.222056168	NA	-0.858863827	NA	NA	NA	0	8.110425888	8.742080338	-8.709659397	-8.709659397	-8.709659397
Halomonas_titanicae	0.222056168	NA	0.419736836	-0.152585792	2.512100687	3.058635538	0.018243764	0.478671953	-0.076720757	0.617554812	-0.137865964	-0.161978454
Streptomyces_venezuelae	0.222129972	-1.197141937	0.078011301	2.220112353	1.15865228	0.809894752	0	0	0	1.006934279	-0.105919746	0.444225234
Plantibacter_flavus	0.222533289	NA	-2.793614865	2.36975662	NA	0.270220946	-0.322948327	0.388041481	0.864710475	9.350203286	0	6.930737338
Paenibacillus_odorifer	0.223279895	-1.35944859	1.226364055	0.23342343	-0.468484826	0.233683192	-10.3229534	-0.0875973	-3.165838928	0.358925103	0.2594873	-0.181071311
Nostoc_flagelliforme	0.225488335	NA	-1.925999419	0.49839761	NA	-0.764871591	0.138474207	2.258679956	2.039140756	1.371033415	-8.803055861	-8.803055861
Paenibacillus_yonginensis	0.225671928	-0.707223086	-0.820445539	2.495230147	2.986823072	2.896801883	-1.508891953	0.001549623	0.050015347	0.435696376	0.725920315	-0.589606778
Legionella_oakridgensis	0.228866927	-1.017389387	-1.016575914	-1.866248611	1.183599938	-1.205658406	-0.008112086	0.320660475	-0.583192168	9.933690655	11.98797452	11.9183676
Mycoplasma_sp._1654_15	0.229133999	-0.58024009	-0.146756924	0.641879253	-1.358120747	0.865223321	-0.197718263	-0.015148506	0.348751713	-2.675839382	-0.337213622	-1.873823822
Caldithrix_abyssi	0.230099751	-0.130421684	0.267195132	1.446635054	0.889225926	0.326119424	0.200919369	0.598886844	0.512450389	0.101673902	-0.121212119	0.169395165
Kosakonia_sacchari	0.230688129	-2.038495611	-0.131922236	NA	NA	NA	0.538304538	0.292261298	-1.18537481	-7.544323092	2.684976416	1.960827488
Borrelia_coriaceae	0.233059346	-3.096861539	-0.191040003	NA	NA	NA	0.193637646	0.310969274	-0.074400273	-7.745956618	-7.745956618	0.84150964
Bradyrhizobium_lablabi	0.233718839	NA	3.382500303	2.177291464	-0.502820548	-3.096520063	0	9.593814998	10.50590085	-0.0413649	0.789567467	0.003310173
Streptococcus_thermophilus	0.2411543	-0.532703889	-0.248345269	1.621656278	-1.358247325	-0.300395431	0.447487243	0.32060413	1.414483167	-0.32328918	-0.478567406	0.364725568
Vibrio_cholerae	0.241804226	-2.1098191	0.541169071	-1.284989608	-1.938599455	-0.158006929	0.118527352	-0.066691098	0.639988721	-0.507925509	0.032510162	-0.351472371
Deinococcus_sp._NW-56	0.244545564	-1.928326722	0.843183001	-0.87731748	-0.840928824	0.471333708	-0.213981963	-0.120955314	-0.052922674	-0.515667532	2.537178046	1.754317068
Mesorhizobium_sp._M6A.T.Cr.TU.016.01.1.1	0.244905675	0.056244111	-0.873366561	NA	NA	1.557689571	-0.212165087	0.126075219	-0.697005644	-1.294100684	-2.655416491	-1.618229687
Pectobacterium_polaris	0.250520409	0.27438907	2.532593264	NA	NA	-1.383283611	-0.056119566	0.303288209	0.681859177	0.724653789	0.294938763	-1.892687672
Peribacillus_butanolivorans	0.251389644	-0.642381319	0.957119543	NA	-0.292799369	-1.328639138	0.319750909	1.231353757	0.984753551	1.025961186	0.369532524	-1.27488856
Synechococcus_sp._TAK9802	0.253942628	-6.269342976	-1.504836689	NA	2.134301092	0.276169808	0.014676129	0.779995269	0.736518569	0.396072221	-1.435115909	0.027254422
Rhizobium_sp._11515TR	0.254813899	1.075539396	-0.882331236	NA	NA	NA	-0.111818068	0.299250926	0.670404231	7.333897756	0	0
Candidatus_Blochmannia_vafer	0.26021231	NA	NA	NA	NA	NA	0	0	0	8.366322214	7.222389201	8.380820341
Bradyrhizobium_arachidis	0.261421104	-2.728525231	0.396813613	NA	NA	NA	-1.749254921	-0.2277175	-2.465464021	10.19311874	7.29523629	10.47099435
Phocaeicola_vulgatus	0.262254484	2.4792655	0.996173939	-6.977403685	-6.360307018	-6.321179357	0	0	0	0.561962117	0.045698907	-0.333886854
Parvularcula_bermudensis	0.263858254	-1.483669853	0.382605096	NA	0.217707	1.137678619	-1.143695123	-1.955754772	0.20549179	-0.917143006	-0.090425202	-0.74888329
Brevundimonas_subvibrioides	0.265053573	-0.815866422	0.741081703	NA	-1.602929047	NA	0.077341832	-1.767485501	1.091151299	-0.270853197	-0.989404885	-0.32015683
Lysobacter_sp._H23M47	0.269580296	-3.040949628	-0.685087081	NA	NA	NA	-0.536888838	0.662527008	0.745906823	0	0	0
Anaerocolumna_sp._CTTW	0.269880551	-1.972469377	-1.085379497	-0.312912468	0.179988349	0.06610687	-0.903110947	-0.255987052	-0.721330127	0.663250156	0.282692495	0.255940425
Microbacterium_hominis	0.27188844	-1.141895506	1.35841466	0.780953607	2.174780396	2.981004325	-0.95108762	0.42988586	0.41023278	-1.324027629	0.898947234	1.462010253
Eubacterium_limosum	0.273991525	-2.046838149	-0.321576175	0.158103065	0.003259821	-0.102110191	1.13054516	0.743013061	2.20139847	0.290519686	0.257314585	0.12949788
Lactobacillus_frumenti	0.276190069	-2.256563322	-0.02266372	1.517854991	1.235391651	0.795815475	0	8.110425888	9.324999567	0.230177461	-0.931404263	-1.190069745
Paenibacillus_sp._DCT19	0.276637984	-2.623694895	-3.746551643	2.103706456	-1.048209093	-1.438252213	8.876516947	0	0	-0.598941464	-0.850868103	-1.416531246
Liquorilactobacillus_nagelii	0.281373542	-2.037215391	-0.138627758	-2.968319562	NA	0.737619137	0.920070823	0.854569376	0.934929508	-0.541944012	0.370845501	0.378210607
Phytohabitans_suffuscus	0.281981218	-0.289345683	0.887838424	NA	NA	NA	0	0	6.15711649	9.158188052	8.776982369	11.16532786
Citricoccus_sp._SGAir0253	0.283811729	-1.569806355	0.373329349	NA	0.085881805	0.846767362	-0.724072465	0.451731376	0.361253823	0.976367409	1.945163909	0.336769652
Aeromicrobium_marinum	0.284536708	0.559605942	-0.840434593	1.877105311	2.521258933	1.175793856	-0.254573003	2.622850074	-0.133267284	0.238902743	0.85330512	2.769415789
Bacillus_sp._WP8	0.285031508	-1.65637856	0.209281448	NA	NA	NA	0.161039653	0.174038063	-0.48084562	-2.949676767	-4.780969743	0.310691975
Cyanobium_sp._NIES-981	0.286022184	-4.342553922	-0.066550751	1.802925397	2.117381137	-0.00620515	-0.967270424	-0.219698747	1.450911469	0.128591141	1.158459815	0.473324291
Rufibacter_tibetensis	0.289506617	1.132589511	1.352083498	-1.204222061	2.797801152	2.28258313	-1.040830469	-0.244570346	-0.060764549	1.845983526	1.804960441	1.018854029
Geoalkalibacter_subterraneus	0.290097613	-1.546466964	-0.835050594	0.353915748	-0.736598237	-1.14491738	-0.328691432	0.852405989	0.27370437	-0.201274466	0.000339345	-0.087334292
Treponema_primitia	0.296548852	-1.509582777	0.095657338	1.373866015	0.341215191	0.420953309	-1.956603335	0.031924906	0.335100259	1.214807497	0.889385902	0.072199208
Bacillus_weihaiensis	0.296770738	-1.066764948	-1.022174797	-1.902123136	0.508867531	-1.193726694	-10.25803597	0.796751474	1.633576881	3.202512928	4.22623841	3.344669239

Saccharopolyspora_pogona	0.297529466	-3.064848217	-0.394388087	3.05246742	0.894727957	-0.35161978	-9.944646183	-0.065341698	-3.787529693	-9.768184325	0.561242044	-1.578359766
Stenotrophomonas_maltophilia	0.298929871	0.237386761	0.734694825	2.285659899	0.548465258	0.855658178	-3.524543081	-3.927087225	-2.476426261	0.242293581	0.272264803	0.864051748
Kinneretia_sp._DAIF2	0.304379894	-1.626672753	0.298009922	NA	NA	NA	0	0	6.15711649	1.279049105	1.987133824	1.279049105
Enterococcus_hirae	0.305640501	-1.304139207	-1.420260246	-0.462495515	-1.858346988	-0.954870142	0.525058835	1.595210336	1.357559405	0.329818355	0.604868131	-0.096459942
Synechococcus_elongatus	0.308308486	1.812877206	-0.498069332	NA	NA	NA	0	10.05724718	6.15711649	0	0	0
Methylobacterium_brachiatum	0.309714543	-2.17938591	0.951151178	-2.408822958	-0.883163892	1.442670671	-10.32907874	-0.902432388	-1.424922834	0.321926796	0.478816325	1.027857516
Weissella_koreensis	0.314184897	-0.015291652	4.274651632	NA	-0.045321979	-0.214661828	1.949188631	2.306508845	1.955795314	0.615408687	0.484844514	1.386977349
Hungatella_hathewayi	0.31671517	-1.513579383	-0.469045839	0.505452083	0.260969568	0.009709025	-9.091316369	-2.40864476	-2.934199879	0.450305838	0.093904023	-0.048421678
Synechococcus_sp._RCC307	0.323153314	-2.709045509	-1.665623228	NA	NA	NA	0	0	0	0	0	0
Corynebacterium_phocae	0.323803552	-0.080559141	-0.381035614	2.866423179	1.569796053	2.703830307	0.023553338	-0.513167652	-2.292712384	-0.179323699	-0.180557413	1.131599105
Vibrio_alginolyticus	0.324163099	NA	NA	-0.207324973	NA	-0.022900402	0	0	0	0	0	8.952254929
Paenibacillus_bovis	0.324244131	-1.779562185	-1.047253628	-3.667202678	-0.118095656	0.143262172	0	0	0	0.872482337	1.077326714	0.539577031
Mogibacterium_pumilum	0.326110817	-1.746676007	-0.057479188	-0.147065583	-0.055137198	-1.026255719	-0.574021583	-0.876272052	-1.709835324	0.924773467	0.063540076	-0.36822702
Synechococcus_sp._JA-2-3B'a(2-13)	0.327374047	-0.276124405	1.02387305	1.34263427	-0.764732835	1.61557486	8.409390936	9.39192664	0	0.434161462	1.004453424	-0.625699265
Nocardioides_humi	0.328622747	-2.857848563	-0.291892479	-3.690480692	0.155723136	-0.238532503	-0.205364794	1.026096523	-0.557605007	-0.500562252	0.8294792	-9.456697335
Amniculibacterium_sp._G2-70	0.32900207	2.41247446	0.989998128	-3.817090645	NA	-1.973420559	-1.778761415	0.320210461	1.070578615	0.623042619	0.750275558	0.694449301
Amycolatopsis_sp._AA4	0.333565181	-1.692441457	0.79898487	0.378987839	NA	3.021128916	1.006262372	1.048649163	0.96452875	-8.95419631	-8.95419631	-2.409876309
Neisseria_gonorrhoeae	0.336525471	NA	1.070098198	0.201370182	-0.43408997	2.001102135	0	0	8.157119522	0.914636611	-0.217827666	-0.123786672
Enterococcus_durans	0.338152684	-0.68232878	-0.979002958	3.468444374	-0.99740929	0.057854066	-0.21465511	0.885909036	3.176190579	1.31356854	1.188162679	-0.187906339
Shewanella_sp._YLB-06	0.341036918	NA	NA	-2.366961849	NA	-0.398084366	1.08080165	-1.098583878	3.177539853	1.845246506	1.872539091	1.962385708
Lysobacter_sp._II4	0.342248079	-3.63493096	-0.562122524	NA	-1.037474705	NA	1.071556529	-0.26670679	-1.049773444	9.599912842	0	9.201307944
Pectobacterium_versatile	0.344115713	-1.743685008	0.61790529	NA	-0.386850273	0.584962501	-0.1123138	0.613872925	0.83349404	7.420659242	0	9.699282671
Candidatus_Desulfofervidus_auxilii	0.347082218	-1.493734535	0.633406969	-0.718301227	0.877776083	-1.039392734	-0.478118076	0.553868204	0.270951865	0.674253084	-0.049963563	-1.93217331
Corynebacterium_sp._2183	0.348684553	1.129729665	-0.599246727	-0.932706887	2.590855069	0.466749071	1.539269171	-0.348594819	2.283209872	0.758047808	-9.445703946	-9.445703946
Mycoplasma_cynos	0.350522755	-3.623190554	-4.400798132	0.625886231	NA	NA	-0.517790367	-0.879503524	0.090839355	0.235925173	-0.56411959	-0.798284995
Fictibacillus_phosphorivorans	0.356203365	-0.842195877	0.384947666	-1.962280334	-2.576626642	-2.660975911	0.27917286	-0.193473005	0.366964511	-0.986385295	0.317138219	1.059036755
Methylobacterium_sp._4-46	0.357641996	-4.119171701	-4.193172282	-0.778052786	-1.437015868	-0.422326483	0.071383908	-9.778376154	-3.621259664	-0.147009938	0.095008369	0.648122376
Actinomyces_sp._oral_taxon_414	0.364039967	0.050169596	1.617678076	0.997612968	1.304652658	-0.964401945	0.138121463	-0.067222987	2.45303495	0.256212132	1.351510087	0.90089817
Serratia_plymuthica	0.36413624	-0.92568605	0.170969298	-0.603534607	-1.193603998	0.814049575	0.876302722	0.138055571	0.956282395	0.555561584	0.51927795	0.735297423
Pauljensenia_hongkongensis	0.364152716	-2.72221547	-0.483017041	1.426103443	-0.05343108	1.311311677	0.81747711	0.93966553	-0.039474991	-0.133019095	-0.662634225	-0.287865388
Dehalococcoides_mccartyi	0.369470276	-1.263722758	1.407075343	0.747947968	0.625209542	0.916579897	0.380088943	2.036494288	2.685056651	0.25650462	0.280807052	0.127574246
Bacillus_gobiensis	0.370976276	-0.753692681	-0.491154915	-1.854960454	-2.765782867	-0.929787896	-1.232853764	3.10073973	2.961366281	-0.004167151	0.726902972	-9.852790092
Protaetiibacter_intestinalis	0.37278698	-0.188727762	0.104179626	NA	NA	1.093314785	0	10.46334045	9.477205768	-1.951481025	-1.144606146	-0.261873606
Anabaena_sp._WA102	0.373775534	0.032180406	2.898283528	NA	NA	NA	-1.055445252	0.206690979	0.539340806	-8.044394119	1.493470728	0.913674233
Microbulbifer_agarilyticus	0.381597281	-1.118407588	-2.187663279	1.819247968	1.243805728	3.032331587	-10.58444582	-2.202314662	1.769902753	1.8567042	2.027480736	0.624492365
Thermoanaerobacterium_thermosaccharolyticum	0.385567871	-0.396880222	0.31771143	-2.7906905	-1.458688605	-1.045208335	-1.110993561	0.308242521	-0.555085228	1.815376317	0.954921007	-0.022208847
Planococcus_sp._Y42	0.387231233	-1.455015725	-0.081635522	0.50803231	-0.537120858	-0.040384731	0	0	0	1.196014446	0.731859192	0.839737386
Paenibacillus_xylanilyticus	0.387607706	-1.465827014	-0.439467835	0.202740595	1.531816694	-0.443891056	1.194545109	0.725536776	1.096279845	0.714970415	0.051838932	-0.630457686
Rhodococcus_erythropolis	0.387925654	-1.63493096	NA	NA	NA	NA	0	9.3781973	0	0	0	0
Paenibacillus_lycopersici	0.390520462	-1.108888458	0.515275493	0.348123068	-0.409573597	-0.58091627	-1.346796389	1.317704087	0.675533006	0.677998159	0.713905769	0.347801896
Sphaerobacter_thermophilus	0.39070817	NA	0.184328067	1.068115902	3.262462081	3.760444968	0	0	0	0.832300182	0.687175513	0.140394449
Thermus_thermophilus	0.391370937	-0.684741696	-0.585936834	0.483458889	0.934667751	0.870458844	-0.582968089	-1.002001574	-1.616274162	0.819166748	1.590559046	0.364519486
Listeria_innocua	0.394971876	-0.749790293	-1.784074515	-1.846223977	-1.013103587	1.489706444	1.7133768	1.075178261	1.755365908	1.259774907	2.856681275	2.774293704
Calothrix_parietina	0.396342238	-1.381947234	-0.077491959	1.718944187	1.243557031	-0.438442104	0	0	0	9.405141463	8.425144554	0
Arthrobacter_dokdonellae	0.396460175	-1.098481303	-1.012358265	0.640382068	-0.954029496	-0.412814891	0	0	0	-0.439652591	0.985934837	1.132599828
Cardiobacterium_hominis	0.397425597	-5.286542496	0.50345786	1.971953821	-1.11473403	0.486816372	0	0	0	0.33579137	1.706933143	1.916806686
Pseudomonas_sp._SNU_WT1	0.400993932	-0.313353359	1.885955449	4.588826618	2.0545092	3.079834174	-0.472851797	1.495628843	0.303351055	-1.866907978	0.62462817	-0.57627173
Helicobacter_bilis	0.401302474	0.250360576	-2.589174752	NA	NA	-6.749620768	-8.091313724	2.070385046	1.58095072	-1.16784893	-1.098947325	-1.317352629
Gramella_echinicola	0.402665752	0.41355477	0.805624924	3.066629794	4.204780618	4.422145531	-0.705657903	-1.384017941	-0.846129087	1.070428134	0.647313327	0.505066038
Clostridium_sp._CT4	0.404182806	-1.160375577	-0.288485464	1.28798605	0.80836801	1.345363188	0.357341475	0.302059332	0.058772465	1.228624875	0.929923424	0.711478573
Phenylobacterium_sp._HYN0004	0.404616876	-2.274903835	-0.509963892	-1.15830188	-0.421826665	1.98398523	-0.199309337	-0.329795638	-0.450313422	0.872424771	0.911975252	-0.216746809
Staphylococcus_aureus	0.407908433	1.265895913	-0.720035198	3.067563381	0.968817245	0.800513798	-0.14518906	0.269148819	-1.141691041	-0.546931574	1.046162542	1.327061009
Nitrospira_defluvii	0.409953746	-0.95199287	0.214291456	NA	1.950787026	0.413277041	-2.471308253	-1.037654382	-0.242236769	1.695937632	-7.741466986	1.810562266
Ahniella_affigens	0.415683145	NA	-4.292523456	NA	NA	NA	-1.005617292	1.236575039	1.435075391	0	0	0
Maribacter_cobaltidurans	0.417423106	-0.931800949	-0.351781142	NA	NA	NA	0.475364986	1.890668333	2.183138656	-9.810571635	-1.322462377	-0.581752944
Caulobacter_sp._FWC26	0.418159541	-2.680574412	-1.38126462	2.486290113	2.826797004	0.608941364	-1.254798046	-2.458141604	0.659517424	-1.901089401	-1.042704653	-1.141777648
Halothermothrix_orenii	0.420542863	-2.916788163	0.367809654	-1.134420647	-0.445677688	-0.686191376	0.225272905	1.436072665	1.402899209	2.491359015	2.02210179	0.690414487
Selenomonas_sputigena	0.421886651	-0.790991685	-0.346046354	-0.01551518	1.150729135	1.060516671	10.43235398	9.074385014	8.742080338	0.570557684	0.187841618	-0.053900917
Syntrophobotulus_glycolicus	0.422759964	-1.745099763	-1.467436118	-0.795142574	-0.733614336	-0.437788901	0	0	8.624046047	0.834099539	0.314371226	0.062002477
Mycobacterium_parmense	0.424331614	NA	1.218633664	-0.039052777	NA	NA	-8.637284195	-8.637284195	-8.637284195	7.303780748	5.472487771	8.412218392
Formosa_agariphila	0.431157165	-3.160001602	-1.646463732	NA	1.274085656	-0.262786754	1.230772462	0.787874613	1.447070281	0.647983669	3.917043539	1.446708895
Bacillus_sp._BD59S	0.431950882	-3.223300062	1.470033373	NA	1.678174775	-1.040900661	0.175522196	-9.868120324	0.386152463	3.181715536	2.513988091	1.341822879
Burkholderia_sp._Bp7605	0.435467508	NA	1.284657218	1.941693015	0.010323443	0.420047143	0	0	6.15711649	1.527933212	2.225935657	2.390333001
Bacillus_miscanthi	0.439599284	-3.082001156	0.046566061	0.228310827	1.477193717	1.227285406	0	0	0	3.296223512	2.979623967	2.418903313
Ochrobactrum_sp._MT180101	0.440190977	2.255159083	2.019204211	NA	NA	NA	0.497106765	1.288035107	1.180204118	7.129283017	0	8.89683794
Leptotrichia_sp._oral_taxon_498	0.44577087	-1.032091509	-1.593596747	0.00011475	0.807944968	-0.054015312	-1.046973908	-4.391710729	-1.333834182	-0.213284259	0.408064978	-0.827520802
[Clostridium]_scindens	0.448171973	-1.697174988	-0.633049632	0.842730557	0.019377891	-0.18582178	0	0	0	0.449307356	0.259416064	0.028412676
Candidatus_Methylopumilus_universalis	0.449556641	-0.815363993	-1.109056056	3.358459611	1.971233437	2.097051966	0	0	0	0.809508256	0.910994994	0.926577981
Rhizobium_sp._ADMK78	0.450274679	NA	-0.476041232	NA	-1.910732662	-0.295316365	0.018663439	-1.166467524	0.29699135	-1.966833136	0.602009699	0.87467041
Lactobacillus_coryniformis	0.451344605	-0.968872226	0.87940588	-4.947566973	-1.247012968	-0.220515823	-9.714245518	-9.714245518	-1.090199471	-0.048763181	0.515111739	1.451973438
Bisgaardia_hudsonensis	0.453606516	2.059177936	0.343092705	2.364024578	NA	-0.15738629	0	0	8.904155902	0.617120523	0.660153885	-0.267912627
Helicobacter_himalayensis	0.453835364	-1.317103374	-0.185494678	-1.705279931	-2.491491115	-2.361958303	-0.371573456	0.610698923	0.574324307	-0.987921848	-1.004475459	-1.041368074
Roseateles_depolymerans	0.455492981	-0.185968187	-0.606759298	NA	-0.431957404	-0.888943085	9.502742316	0	10.8917837	-1.485853352	-1.615969858	-2.315599807
Undibacterium_sp._KW1	0.456659369	NA	1.576631226	NA	NA	NA	0.317504674	0.226099482	1.48273329	8.86005338	0	8.634240809
Leifsonia_xyli	0.457633868	-1.155374049	-0.962930745	-0.602421976	-1.687401265	-0.961870566	-8.624046047	-8.624046047	-8.624046047	-7.807354922	2.756155982	0.826849887
Neoasaia_chiangmaiensis	0.459569814	-2.168253721	-1.73039294	2.732920382	NA	2.449957484	-8.637284195	-8.637284195	-8.637284195	1.99892563	2.062598201	3.229278906
Corynebacterium_marinum	0.459819826	-2.812660527	0.312157533	1.899306	NA	3.299053629	-0.375840956	-0.404291751	0.415133239	-9.620830138	-1.62459394	-0.314009695
Alcanivorax_dieselolei	0.463372461	NA	-1.820178962	NA	NA	NA	0.536217513	1.391354463	0.480747185	0	0	0
Aeromicrobium_sp._592	0.467778961	-0.341595128	NA	NA	NA	NA	0.785203223	3.533148201	3.826804127	-0.037968519	-9.489179631	-1.854971191
Rippkaea_orientalis	0.469070477	NA	-4.874593547	-3.580197553	NA	-1.733354341	-9.177646265	1.206446725	2.363890838	0.094594932	0.604210385	0.294538617
Acetobacter_orientalis	0.475380405	NA	2.827580751	NA	-1.231403663	0.265128955	0.322198223	2.797735552	2.892955165	1.114058909	-8.705055194	-0.184110495
Microbacterium_sp._ABRD_28	0.480562895	-2.27364808	-0.621778921	-0.703324697	1.603507796	1.638840869	0.163105498	0.083464501	0.747751904	0	0	0
Oerskovia_sp._KBS0722	0.481371146	-1.109243227	0.408249077	NA	-2.661839666	-2.189191443	-0.428433568	1.114259793	1.407659949	0.532968683	-0.648607737	0.175627644
Cellulomonas_sp._Y8	0.48679437	-2.287515133	0.187138498	0.582992951	NA	NA	-0.365248943	1.724797779	0.150461341	1.109231096	0.01210662	-7.890768904
Pectobacterium_brasiliense	0.487445995	-1.819427754	0.371968777	2.86078767	-1.750819603	NA	0	0	7.157114468	-1.846692136	0.164982141	-0.655373825
Weissella_confusa	0.487982532	0.440895667	1.849133334	-2.608979382	-2.31006794	-0.708329726	-1.209224281	0.091958836	-2.755848943	-7.745956618	0.47965091	1.867525309
Leuconostoc_gelidum	0.489376319	-2.095010194	-2.854206561	-3.266235368	-4.180437495	-1.272436707	-1.608511539	-0.75599602	1.230949439	0.166258628	0.03865318	-0.605814609
Candidatus_Planktophila_versatilis	0.489843305	-1.088341781	NA	NA	-0.195594196	1.598349807	0.795613506	0.644365391	1.30485384	-0.369270763	-0.308900052	-0.18085989
Saccharospirillum_mangrovi	0.490747159	-0.355268509	-0.276027244	-3.247136345	NA	NA	0	10.35147857	0	0.005548243	-0.350487397	0.609357568
Streptomyces_tsukubensis	0.495285829	NA	-0.234193713	1.943524535	NA	1.501546493	-0.637624033	0.040323827	-0.253375573	-0.202815777	-9.869336279	0.158569718
Limosilactobacillus_reuteri	0.49660488	-1.132978435	-0.41790417	2.360488113	-1.012024671	-1.261683921	-0.346722939	0.354114907	0.119274107	-0.926476853	-0.800466687	-0.492881478
Streptomyces_spectabilis	0.499643667	1.713280561	-0.269688679	NA	NA	NA	8.157119522	6.682671608	9.140994473	2.061773004	1.860818371	3.610495017
Adhaeribacter_sp._KUDC8001	0.500528021	-1.414080488	-0.035863262	NA	1.34788228	1.547729595	2.788772429	0.074873531	0.81382602	1.008399325	-0.515684648	0.848067117
Halomonas_sp._1513	0.500685858	-2.255654875	0.200203503	NA	NA	NA	0.106050347	1.580161335	1.402766433	-8.044394119	2.586782936	2.026169631
Tatumella_ptyseos	0.503832674	-1.003529223	2.793621749	-4.198800994	-3.460129078	-1.16244853	8.157119522	0	9.561202011	-0.698179479	0.942501518	0.908221188
Klebsiella_oxytoca	0.505059613	0.578851383	-1.13920515	1.425919617	2.206285989	0.694466343	-0.070336769	0.449935804	-0.49171688	1.297261002	0.824197235	0.584962501
Paucibacter_sp._KCTC_42545	0.505516366	-1.657174126	-1.077161065	-0.11808607	-0.632659243	0.915880998	-0.595490642	-0.518906382	1.090560829	-2.216893717	0.087982333	-0.301098563
Streptococcus_merionis	0.507045288	-2.942212248	-0.43638365	-0.276221838	-0.512792704	-0.250421813	0.029362068	1.118380004	1.155166138	-0.307939383	-0.40662464	-0.621854825
Thermogutta_terrifontis	0.508178387	NA	-2.334068658	NA	-0.13289427	2.113700499	-0.115054909	-0.21099415	0.080061853	8.35167691	0	9.306820443
Blautia_argi	0.50993869	-1.651603227	-0.980154118	1.044474481	0.518711003	0.422992286	10.19565518	10.47629054	8.800605508	0.522773284	0.215860524	-0.006291416
Neobacillus_thermocopriae	0.510255878	2.083738882	1.573420879	1.812748181	2.94773269	4.368449239	10.77762519	10.54197746	8.863904389	-10.39945952	-1.467540128	-0.151532005
Acidovorax_monticola	0.51104545	-0.720867091	0.583374504	NA	2.584446423	1.248253362	-0.177517188	1.250430555	0.486189554	-8.739216921	-1.51682772	-8.739216921
Pseudomonas_psychrotolerans	0.512525344	-1.258073176	-2.615989396	2.634505828	-0.954668924	0.90592802	0	0	0	1.438258991	0.964601565	-0.182324298
Scytonema_sp._HK-05	0.512569651	-1.405551378	0.570966966	-0.884432926	1.48156251	0.742852845	-1.881546389	0.811303594	-0.130637834	0.907806346	-0.440534441	-0.85895388
Thioalkalivibrio_sp._K90mix	0.515130808	-0.548703901	1.160807105	-0.431831685	-3.224650514	-0.0453816	0.589699951	0.037872313	-1.115272989	-0.542836377	0.781052926	1.847413273
Neisseria_zoodegmatis	0.516015147	-3.461163892	-0.899695094	1.153709415	NA	-0.477254825	0	8.566400396	8.742080338	9.550746785	0	8.651051691
Acinetobacter_calcoaceticus	0.522711407	-2.385750752	-0.375468477	-2.791701658	0.574180164	1.818021847	2.573275226	1.448565993	1.087739273	0.301873328	1.588752773	2.96270781
Deinococcus_gobiensis	0.52899903	-3.715864112	-1.575987697	0.235929176	1.102462034	0.251734042	-0.123108154	-0.137067059	0.212590596	0.180207654	-0.278822296	0.65914963
Saliniradius_amylolyticus	0.5342092	-1.946159239	-0.150025444	NA	NA	NA	-0.540012693	-0.241270562	1.169565531	8.366322214	9.505150581	0
Thioalkalivibrio_nitratireducens	0.535687791	-0.512649579	-2.988687694	0.303993723	-0.653234522	-3.016925141	-0.340305027	-0.577081039	-0.644620885	0.22862282	-0.568660688	1.762960803
Paenibacillus_sabinae	0.536011752	-0.951693803	-0.610849232	-0.894311031	0.388585633	0.560055077	-0.205934738	2.051412494	3.035460325	0.561281491	-0.025177352	-0.672276281

Pseudoxanthomonas_mexicana	0.537681514	-0.622236137	0.43984226	-1.147708813	-0.32851314	0.806150592	0	0	6.15711649	-0.385026197	-0.422725765	0.457342914
Rothia_aeria	0.537689201	-3.195459219	-0.944772778	NA	-1.162667447	0.436496746	0	0	0	0.076849591	-2.312196557	-2.184646507
Variovorax_sp._PMC12	0.538273191	-2.347662971	-2.226275856	NA	1.740480078	2.027015919	-2.241975134	-0.810409556	0.677076793	0.907247238	1.48732353	0.162705366
Paenibacillus_sp._URB8-2	0.539487494	-2.878577134	0.398263072	-1.082478254	-0.146602008	0.335164172	-2.043831921	-10.17311494	-0.362250938	0.541190206	0.249365789	0.529380279
Ferrimonas_balearica	0.539879099	0.864950403	-1.196892049	1.444387098	1.281174753	-0.824932492	-0.039985822	-10.056028	-10.056028	-0.175964435	-0.435010976	1.620441259
Pseudarthrobacter_sp._YJ56	0.540907321	0.373775534	0.509502183	NA	0.878883283	1.265733555	-2.311849358	-1.678411222	-1.722242927	-7.709655951	-7.709655951	-7.709655951
Blautia_producta	0.541742663	-1.179349062	-0.727977382	1.548771539	0.514395503	0.393028878	0.684057629	0.144334753	-0.412660878	0.540302905	0.278438871	0.139776316
Francisella_frigiditurris	0.546634382	-2.443311953	-1.631384301	NA	-1.466216223	-2.801564834	0.654295968	0.831601114	1.998250591	1.890533622	1.677235492	0.024096623
Dehalobacter_sp._CF	0.548317407	-3.128676715	-1.355518111	3.374876975	NA	0.235812937	-1.701380492	-1.487255687	-0.089276525	0.832122827	0.171541662	2.669851398
Brevibacterium_sp._o2	0.548893246	-2.525405656	-2.878158877	NA	NA	NA	0	0	0	-8.202941691	-8.202941691	0.538525296
Kluyvera_intermedia	0.550197083	4.110002238	1.828544544	5.830660974	3.965938164	3.155317482	8.502740328	0	9.624047875	0.257443255	0.577128528	1.041937074
Salinimonas_sediminis	0.554037928	-3.755122871	2.004699337	0.997668685	0.265555753	0.902639596	-0.49476303	-8.624046047	-0.318604416	0.674040372	-0.043918934	0.154369332
Pseudomonas_resinovorans	0.561047603	2.317750997	0.53139207	NA	NA	NA	-8.637284195	-8.637284195	1.276420543	1.184055336	0.550203947	0.563201288
Erysipelothrix_sp._HDW6B	0.56318321	-2.206830984	-0.961366587	0.971791085	-1.654764532	0.423627319	-0.377919474	0.648552584	2.651548217	-0.890971088	-0.099499147	-0.195073743
Propionimicrobium_sp._Marseille-P3275	0.572818937	-2.031395826	-0.714582778	0.729238222	0.790440675	-0.428432751	0.342858543	-10.14442882	0.60338653	0.597237518	0.095766841	-0.51781419
Pseudomonas_sp._IzPS32d	0.575228618	NA	NA	1.663290861	-0.202408047	-0.537756658	-1.049753945	-1.170363116	-1.395374751	0	5.472487771	0
Paenibacillus_sp._FSL_H7-0737	0.575732265	-1.877224956	0.806722669	-0.463105851	0.621699776	-0.509645707	-0.086768284	-0.001557022	-0.194719035	1.363522324	-0.001533424	0.261204971
Acinetobacter_sp._Marseille-Q1620	0.578923584	-0.823901231	-0.552782095	1.877482392	NA	-0.541171792	9.85826391	12.1089393	10.87581845	0.9954927	-1.923295717	0.567603523
Halioglobus_pacificus	0.579119755	-5.109169072	0.234050891	1.985018608	0.915291744	1.667976551	1.236786782	0.307598558	0.169329508	-0.66786459	0.347923303	-2.044391543
Sulfurihydrogenibium_sp._YO3AOP1	0.580135935	-0.522190674	-0.642117224	-2.090751245	0.323605944	-2.30487605	0	0	0	0.81619357	1.290169736	1.838877584
Arthrobacter_crystallopoietes	0.588002955	-0.777085903	-0.167448719	-0.613636809	0.374786029	-1.071862217	0	8.559465045	9.782849183	0.058284736	-1.241506852	0.01992364
Paenibacillus_jilunlii	0.589696644	-1.665728131	-0.387992676	0.026583848	0.845220736	-0.490481366	-1.457129246	0.258528204	0.206404521	0.38747877	-0.297823338	0.516319892
Geobacter_sulfurreducens	0.59018831	-0.241742586	-0.58078712	2.279459954	1.3113401	1.954215008	-1.48181048	-0.212283655	-2.481815534	0.042401047	-0.258512555	-1.839414306
Dechloromonas_aromatica	0.593225218	-3.224966365	-0.086552336	1.367589002	1.373365906	1.668042817	0	0	0	-0.666129117	0.551958972	1.361754075
Vagococcus_teuberi	0.59396309	-4.478373182	1.153997537	NA	0.655088587	1.782504973	0.737185083	0.785023017	-0.794639898	1.228422632	0.585898346	0.644834582
Corynebacterium_pelargi	0.596277814	1.760179028	2.782804783	0.964749153	NA	-1.605000254	8.166278594	10.48546708	7.745136469	2.16117933	0.31765526	-0.488648416
Magnetospirillum_sp._XM-1	0.597682892	-2.287733175	0.039902406	1.759311753	-0.017702002	2.646949388	0.515995006	0.216065729	0.948343119	1.737293962	-0.390521046	0.236096675
Desulfohalobium_retbaense	0.598832058	-1.177309341	0.704707508	1.179248655	0.765519059	1.387394347	0.76426301	0.749529742	1.532747269	0.448717732	0.600317413	0.650933002
Staphylococcus_delphini	0.599156382	0.071553261	-3.049753035	NA	NA	NA	0	8.607330314	0	-7.544323092	-7.544323092	-7.544323092
Pseudomonas_knackmussii	0.599658414	-2.380689116	0.589113636	NA	NA	NA	-0.214607925	-1.485189001	-10.09251931	8.86005338	10.32942637	0
Achromobacter_sp._MFA1_R4	0.60419872	NA	0.316106856	NA	NA	NA	-0.585356374	-2.933494327	-0.640101132	9.713099494	7.29523629	9.306820443
Stenotrophomonas_rhizophila	0.607930612	NA	-2.728391838	1.740055073	1.657689018	-2.471305719	-5.576634814	-2.516000507	-3.805374478	11.10415972	11.15211369	10.37213591
Geobacter_sp._FeAm09	0.607931524	-1.423553204	0.502652147	0.565800651	0.520330135	0.196664656	0	0	0	0.872877297	0.903255713	0.20412295
Pseudodesulfovibrio_indicus	0.608888793	-0.872886838	-0.173349177	1.826868847	0.239761491	1.784409937	-1.342515595	-9.588714636	-3.431598146	0.186196797	-0.306962714	0.303977583
Solibacillus_silvestris	0.615409179	-1.03788476	-0.931540898	0.692764411	-1.431166854	-0.910560204	9.564669681	10.65528921	6.15711649	0.738664382	1.892132028	1.226939768
Leptotrichia_wadei	0.615931378	-2.330131774	0.365693426	-1.611655122	0.830219624	1.015781689	-0.411371551	1.094830112	-0.458263372	1.050264795	0.330829441	0.62976231
Paenibacillus_borealis	0.622235268	-1.621500412	0.507232315	1.001346426	1.336803444	1.627000754	-1.003872512	1.21051924	-0.421228405	1.208798166	1.978822399	0.020356172
Frateuria_aurantia	0.622362473	NA	-1.300233024	0.511252815	0.780092842	-0.187442763	-0.336527373	0.395573989	-0.416632747	1.220551075	-0.991433718	3.085946609
Actinomyces_radicidentis	0.623173659	-1.097434151	0.286266712	0.040445526	0.172562815	1.994260249	0	0	0	0.2114915	0.902980323	1.411853454
Clostridium_saccharobutylicum	0.627674721	-1.343945437	-0.640255406	-2.637097441	-0.975252667	-1.431697121	0.629530817	0.934273745	0.299034181	1.315616239	1.362508886	1.068127873
Streptomyces_katrae	0.628489876	-2.03739262	0.150442579	NA	NA	NA	8.166278594	0	9.759585302	0	9.257387843	0
Salinispira_pacifica	0.630379756	-0.496932116	0.90624711	0.040043385	-0.942333248	1.774911921	1.893048071	-2.191156269	0.837617081	0.433548808	0.115581938	1.09976143
Catenovulum_sediminis	0.63205841	-2.436660311	-0.167319178	-2.004241976	0.14878033	-0.96237325	1.272597205	0.850013649	2.802146469	1.776469368	1.453968154	0.896462588
Dickeya_dadantii	0.635611659	-1.651407388	-1.559744558	NA	NA	NA	0.653890804	0.827962054	0.631188657	8.442943496	9.196398033	6.930737338
Heliobacterium_modesticaldum	0.648946281	-0.790380874	0.915430049	0.810178048	1.366652947	1.333370014	-0.003480723	0.186325835	0.266340314	0.87343171	1.052365014	0.646977817
Acidithiobacillus_thiooxidans	0.650039231	-0.145864103	0.769810051	NA	1.403801623	0.386540858	1.575335016	1.047738232	3.421508443	0.019421234	0.817325211	-0.403024343
Psychrobacillus_sp._AK_1817	0.651398154	-1.363565385	-1.1138199	NA	NA	NA	0	8.110425888	9.069511529	-8.681825211	-0.456217684	-0.094358953
Saccharomonospora_viridis	0.655819098	-0.083328384	0.488577964	NA	3.227016448	0.939304068	0	0	0	1.522391217	1.922455071	1.677570204
Tardiphaga_robiniae	0.655845987	NA	-0.781637752	NA	NA	-0.533269267	-0.229530289	-0.011212566	-1.111517472	-9.478431906	-4.005944135	-0.543759137
Maribius_sp._THAF1	0.656468466	0.134301092	1.700439718	NA	0.213281371	4.018223759	-10.21784867	-10.21784867	-0.302783468	0	7.222389201	0
Providencia_heimbachae	0.659284181	-0.416911999	-0.184753041	NA	NA	NA	0.769975089	-9.543735591	1.304605668	7.333897756	8.912889336	10.27301461
Leptotrichia_buccalis	0.659700468	1.738734687	1.850146562	NA	-2.812874362	-1.163846258	-7.540972677	-6.280604924	-15.70725127	-0.664849611	0.557756958	1.516134723
Deinococcus_puniceus	0.660502106	-3.137917942	0.669683405	0.755177799	1.05397268	-0.938732757	0	0	8.598897393	-1.280701525	-0.685362637	-0.595614463
Kitasatospora_sp._MMS16-BH015	0.670314468	-1.443311953	-0.750289707	-0.407319586	0.174707624	3.084142483	-1.20488969	1.117975852	0.814875756	2.118765771	2.186840931	0.436252669
Edaphobacter_sp._4G125	0.671250639	-2.557995453	0.805798617	-0.069934291	-0.219550791	0.966621442	-0.624058602	0.180126858	-0.720014487	0.869207931	0.591308887	2.758716533
Allofrancisella_frigidaquae	0.672242826	1.137715047	-0.25447812	NA	-2.18977461	-2.880297298	0.957458888	1.533998337	2.163311397	0.765463188	0.542415812	0.463473563
Streptomyces_sp._WAC00288	0.67766841	NA	-3.971241007	NA	NA	NA	9.832602079	8.382131157	0	0	0	0
Trueperella_sp._19M2397	0.683397474	-3.230954435	1.452430119	0.368582054	0.080630016	0.772148618	0.023610177	-8.10567284	-1.94855635	-0.163658259	-0.284578528	0.17197151
Shewanella_halifaxensis	0.686707509	NA	NA	NA	NA	NA	0	0	0	-7.741466986	-7.741466986	-7.741466986
Yokenella_regensburgei	0.689154114	-0.688730387	0.333913274	-2.620852959	0.447192428	-0.174596729	-0.275398927	1.118281988	1.124675916	1.535022246	1.004262027	0.915402095
Cohnella_sp._KS_22	0.690950772	-1.107711517	0.493370649	0.546645739	0.724187471	-0.950655745	10.67065625	9.426646347	7.745136469	1.38266296	0.094192114	0.489939319
Kibdelosporangium_phytohabitans	0.690988282	NA	0.974793398	-0.28125428	0.565736536	0.465149799	0	6.682671608	0	0.95156198	-0.575909075	-1.022769299
Thermobaculum_terrenum	0.69128802	-1.912767162	-1.762937107	-3.654709401	-0.827043823	-0.892290628	-9.482717218	-9.482717218	-9.482717218	-0.939720142	-0.723706488	0.307846842
Nocardioides_sp._ZJ1313	0.694399612	0.486414186	2.45985058	NA	NA	NA	-0.145155163	1.880910011	0.938757446	-0.88274457	-0.19028912	1.120295125
Streptomyces_glomeroaurantiacus	0.696710858	0.453256822	0.260611744	NA	-0.065963262	NA	0	8.110425888	11.45167991	0	0	0
Anaplasma_phagocytophilum	0.702726082	-2.441050736	-1.989995397	NA	NA	NA	0.306058316	1.359933827	1.48587757	0.087336674	-3.337013494	2.330900361
Streptococcus_sanguinis	0.703136417	-1.327075426	0.024109687	-0.406957197	-0.526338587	0.38836633	0	9.426646347	0	0.273833003	0.285315421	0.215948936
Mycoplasma_nasistruthionis	0.707973391	NA	-2.448145811	NA	NA	NA	-2.806803198	-2.271549415	-2.910752504	-8.044394119	-8.044394119	-8.044394119
Desulfobacula_toluolica	0.708263679	-1.594536104	0.972249975	-0.330751945	-0.23001877	-0.601046224	-2.145460246	-0.241063652	-0.4011994	0.797705142	1.742416395	1.591426233
Paraclostridium_bifermentans	0.712676906	-1.24150807	-1.759247234	-0.466353032	0.909834394	1.309193779	1.6208077	1.353749383	2.526035731	0.595629073	0.968981078	0.735742453
Cohnella_candidum	0.716538339	-2.331584167	-0.525951572	-0.485699059	0.307377606	-0.531814725	-0.439015546	-2.012642958	0.676550722	0.30365332	-0.226592101	-0.18416254
Candidatus_Aquiluna_sp._UB-MaderosW2red	0.717964632	1.694352075	-1.235938953	0.442575723	-0.019941876	-1.099318056	-10.60059127	-10.60059127	-2.855454801	-0.866133624	1.743300995	0.569439946
Exiguobacterium_antarcticum	0.724539968	NA	0.184336195	0.008814304	-3.374924315	-0.307951269	1.291536136	1.503455227	1.629315867	-0.678822164	0.593707092	0.267697186
Leptolyngbya_sp._O-77	0.728756094	-1.873394078	-1.212315349	0.242267057	2.645238186	2.514118279	9.029913869	6.682671608	9.432351895	-9.781359714	0.689634632	-2.147151273
Micromonospora_viridifaciens	0.732171445	-0.557197975	1.009087873	0.734527673	0.392159241	0.647439098	0	8.627369725	0	-0.028338376	-2.057241741	-2.509154846
Paenibacillus_cellulositrophicus	0.733931518	-2.363428838	-1.711352141	-1.055897906	0.868019701	0.651690852	-1.994127114	0.328738428	-0.657286753	0.210762323	0.446718725	0.879615196
Mycolicibacterium_frederiksbergense	0.736965594	NA	0.623145695	NA	NA	NA	0.146531158	-0.032128311	0.85268386	0	0	7.539158811
Mycetohabitans_rhizoxinica	0.740332445	-1.134136673	1.95550054	3.050559741	3.021953441	2.022900402	-0.695299511	-0.113511327	0.12694973	-0.804984018	-9.247927513	-1.613719073
Actinoplanes_derwentensis	0.743491997	-1.003881475	1.231964718	NA	NA	NA	-0.703815292	0.280513812	-0.808211409	0.558062489	1.141368488	0.373279529
Selenomonas_sp._oral_taxon_136	0.744711159	-0.754131888	-0.143562731	0.66513779	0.707957278	0.897304222	1.149570694	0.840812876	1.308536579	0.535672535	-0.224450617	-0.514044283
Treponema_putidum	0.744771968	-2.534569132	0.922134938	-0.736418601	-1.465022829	0.72251576	0.825812475	1.638887466	1.64470332	0.452087595	-0.943363404	0.616623367
Comamonas_piscis	0.746688934	-0.477254825	-0.962681652	-0.339981931	0.584610667	0.072046819	-8.091313724	-8.091313724	-8.091313724	-7.709655951	0.002754116	1.597164492
Sphaerochaeta_coccoides	0.752072487	-1.38132636	0.422433311	0.117232802	-0.060184766	-0.536819077	-8.04736934	1.462585742	1.648017308	0.233782622	0.251278859	-0.512966362
Streptococcus_pneumoniae	0.755366386	-3.892478966	0.368248972	-2.803077098	-1.20694529	-1.680144076	0.402823256	0.404749752	-10.00077468	-1.112360353	-1.141072215	-0.935709706
Mycobacterium_lacus	0.757967753	-1.266497488	-0.27182914	-2.100006297	-0.148773883	0.456234066	-1.728475411	-0.658369008	1.790886689	0.26534161	0.350855296	1.605419938
Azospirillum_sp._TSH100	0.760850949	-0.797905369	1.808111823	3.187408	4.299137509	3.542158669	0	8.382131157	8.451211112	0.320864998	0.508141538	0.917671659
Frischella_perrara	0.761840263	-1.1635945	0.871636928	NA	-0.120229436	-0.183476454	-8.925892636	1.03463645	-0.474681524	1.610928916	-0.123663173	1.579848184
Jeotgalibaca_porci	0.766841878	0.448862377	-0.732681708	NA	0.443807738	0.782229668	0.295390575	0.455662354	1.105679588	0.410812515	1.092934978	0.589751021
Chloroflexus_aggregans	0.771638172	-2.706953025	1.032662147	-2.100334729	-0.079504807	-0.466262823	0	0	0	-0.393357027	-0.6339033	-1.238013782
Mycobacterium_saskatchewanense	0.775466764	-0.642573224	-2.338278527	NA	NA	NA	-0.503597057	-0.189392044	0.95888014	0	0	7.380824669
Spiroplasma_monobiae	0.781195217	-0.234058363	-0.241588758	-2.460884923	0.590954009	1.473675513	10.25696346	9.499120372	8.548995253	0.291566877	0.576709126	1.013101951
Halomonas_beimenensis	0.782271335	-2.356350693	0.398148368	1.515427308	-0.680095957	-0.944248298	-0.312085968	0.597064883	0.114960959	-0.319312576	0.49852818	0.174895013
Desulfotomaculum_reducens	0.788080235	-0.574436878	0.349548418	-0.125010037	-0.246011653	0.516892933	-1.357032833	1.10432847	0.37946038	0.043482538	0.009501123	-0.48199355
Acidovorax_sp._HDW3	0.788754032	-1.592955291	-0.673786366	2.917641329	2.541502752	2.786596362	0.923337997	0.282774855	0.85824392	0	8.803055861	7.539158811
Ochrobactrum_pseudogrignonense	0.788774617	-2.968096596	NA	1.33905879	2.796901712	0.211303243	-0.524176488	-0.006602273	0.109548825	-10.70217269	-0.251652171	-1.058316496
Desulfotomaculum_ruminis	0.790133474	-1.743027347	-0.063417998	0.242480614	0.319751243	0.218833423	-8.637284195	-8.637284195	-8.637284195	0.140500886	-0.050238438	-0.317696892
Halobacillus_litoralis	0.797956224	NA	-2.033166864	-1.542072893	-3.87739447	-1.738766217	0	0	6.15711649	0.266362573	0.460106681	0.2633898
Erysipelothrix_larvae	0.798081175	0.276149074	0.06611114	0.090254941	0.388397689	0.644308611	-9.177646265	-0.795515108	1.0878633	1.745795641	2.333127403	1.867667231
Thermosediminibacter_oceani	0.798130406	-1.767884999	0.686563993	-1.818428458	1.608911877	1.90449627	0.91720771	0.770382436	1.074234417	1.625200215	0.79682227	1.871810418
Erythrobacter_mangrovi	0.799806874	-2.170235025	1.315092137	NA	-0.064174232	0.814447526	-0.528772905	0.456855635	1.950970222	0.954813424	0.606889482	0.582850019
Hungateiclostridium_saccincola	0.801260667	-0.940070925	0.181076249	0.639648562	0.521764432	0.849920664	-0.102689969	0.94109246	-0.170455306	0.857126767	0.548901914	0.403429211
Vagococcus_sp._HDW17A	0.802439432	1.476265091	-0.048514828	2.510285814	2.745502276	NA	1.018924143	-1.06326387	3.073672	0	10.7153872	7.63420844
Synechococcus_sp._PROS-U-1	0.804509369	0.490747159	-0.462232667	NA	-2.814130225	-3.022808712	0	10.99499685	8.863904389	0.787731954	0.478801193	-1.569427692
Deinococcus_irradiatisoli	0.8045557	-2.760026383	-0.498790476	-0.508259852	2.22415465	0.071529839	0.383555746	1.786167396	1.076626522	-1.745909792	-1.232890338	-0.48244316
Streptacidiphilus_bronchialis	0.805275111	-1.347923303	0.101796952	4.637607581	1.487793842	2.798324681	1.356809488	0.668052501	-0.333982901	0.941897045	1.978415863	-0.421077626
Xanthomonas_cassavae	0.806799291	-0.600601624	1.535292846	NA	-0.645542566	1.13504996	0.55125485	1.062914171	1.8025408	1.382145618	-7.930737338	1.659226468
Mesorhizobium_amorphae	0.811599555	-2.737285589	-1.010597474	NA	0.400122825	NA	-0.446812838	-0.4515007	-1.12420071	0.611051022	-0.013544597	1.058509368
Streptomyces_sp._SAT1	0.816104955	NA	-0.367116869	0.722336292	0.07912865	1.925323314	9.849760062	10.10686388	11.82864031	-1.290594328	-0.408556302	-8.624492083
Rhizobium_rhizoryzae	0.816463514	3.678959991	-1.9510904	4.223265459	2.496735318	1.979064237	-1.178885383	0.609081752	-0.940212843	-0.967394782	0.118962122	-0.351444589
Comamonas_sp._NLF-7-7	0.816463514	NA	NA	NA	NA	NA	0	0	6.15711649	7.333897756	7.369230901	6.930737338
Nocardioides_baekrokdamisoli	0.819556508	-1.57469838	-0.728997975	NA	NA	1.196965917	-0.697904836	-0.776999926	0.164935646	0.334972256	0.543817029	1.381991385

Comamonas_kerstersii	0.821249364	-1.15329641	-0.582637448	-0.675320711	1.649292376	1.758000603	0.733033062	1.427422167	2.338056753	3.219497869	4.50725225	4.542200181
Halomonas_subglaciescola	0.828519532	1.054052904	-0.930626297	-2.289006706	NA	NA	0.265482345	-0.072172185	0.850165307	0.230257066	-0.751442117	1.158947113
Streptococcus_himalayensis	0.840144857	-3.038553241	-0.728697978	1.273184233	NA	-0.788932933	0.148697616	1.005903482	-0.17316173	0.188496445	-0.471585188	-0.480674679
Yoonia_vestfoldensis	0.840907482	NA	1.912762562	NA	1.433159518	NA	0.651153789	1.350024212	2.518279304	0.729070226	0.098144072	1.395967056
Clostridium_diolis	0.841152812	-1.502817133	-0.507955856	NA	-0.246485972	-0.445598348	0.710247261	-0.290650426	-0.140447344	2.486512785	3.275849124	2.598762281
Paraburkholderia_sp._PGU19	0.841554495	-0.552782095	2.247737197	NA	NA	NA	1.110540188	-1.26138189	1.129408063	0	0	0
Synechococcus_sp._NOUM97013	0.841554495	NA	-2.920513879	-0.207488414	-0.186878135	NA	0	0	0	-10.10852446	-2.296880036	-3.564204456
Hydrogenobaculum_sp._Y04AAS1	0.841554495	NA	-2.920513879	NA	NA	NA	9.509955083	0	6.15711649	0.754256146	0.8232035	-8.990577692
Brucella_inopinata	0.841554495	NA	NA	NA	NA	NA	-0.370446559	-1.552970326	-0.508578105	0	0	0
Mycoplasma_phocicerebrale	0.841554495	NA	NA	NA	NA	NA	-0.153056452	-0.102257119	-0.062740905	-0.227409461	-9.087462841	-9.087462841
Streptomyces_sp._CB04723	0.841554495	NA	NA	NA	NA	NA	1.875121652	1.508819167	3.237437419	1.116207142	0.727447597	-0.708768702
Mycobacterium_paragordonae	0.845131573	-0.808256888	0.908721427	NA	NA	NA	-0.063433338	0.866310782	0.477439909	7.333897756	9.471132578	0
Mycobacterium_sp._YC-RL4	0.847996907	-1.026311277	-0.080418682	NA	0.190952105	0.292067948	9.029913869	9.952608764	9.069511529	-0.6355539	-0.600073948	-0.621052527
Dermatophilus_congolensis	0.847996907	0.048236186	-2.13289427	NA	NA	-1.663146222	9.965784285	9.252665432	11.0877015	-7.890768904	0.920017994	0.95681154
Leifsonia_sp._21MFCrub1.1	0.858626201	-1.542863368	0.543692666	NA	NA	NA	-0.848844562	-0.087165851	0.217925729	0	0	6.930737338
Spirosoma_sp._I-24	0.860808784	-2.021524707	1.340030462	NA	NA	0.257419239	0	8.382131157	6.15711649	-9.442943496	0.498202439	-9.442943496
Mesorhizobium_japonicum	0.862062735	-1.218566695	-0.031789172	NA	NA	NA	0.411201029	1.125133008	0.377143748	0	0	8.996239023
[Clostridium]_hylemonae	0.862373485	-1.509576391	-0.714049454	2.045100704	0.645102159	0.397934237	-1.096297786	0.475325191	-0.637337848	0.542724666	0.313949693	0.189739837
Stenotrophomonas_sp._364	0.867407783	2.47886701	-0.553935605	0.660149997	4.473931188	1.986579484	0.987657144	1.172651356	1.091686005	-0.958179824	1.249416339	-8.087462841
Campylobacter_sp._CGEMS	0.868903789	-0.62099081	0.542738219	1.749927955	-0.221688496	-0.008625779	0	0	11.29137794	0.090840286	0.612353052	-0.018771351
Paenibacillus_sp._FSL_R5-0912	0.874027713	-1.931048706	-0.50540554	4.273565073	2.527129345	2.113591269	-10.99049402	-0.255318817	-0.611310625	2.190240947	1.697324238	0.915153877
Paenibacillus_sp._JNUCC-32	0.876740364	-3.65672767	-1.847346965	-1.932885804	-0.268865448	0.681603596	9.877911389	8.607330314	6.15711649	-0.490107457	-0.011025256	1.054128084
Cohnella_abietis	0.879480435	-2.600507645	-0.995419743	0.324060332	-1.149436898	-0.370487723	-0.770691639	-9.800605508	-0.957969169	-0.609679074	0.112933157	-0.056563133
Lactococcus_sp._1JSPR-7	0.881085747	0.577217624	0.5920968	NA	NA	0.56853153	-8.624046047	0.003323679	-8.624046047	0.407605546	1.006480354	1.622186726
Cloacibacterium_normanense	0.884224819	0.181858247	0.515161908	0.598886317	0.640253953	1.388257115	-1.149076457	-0.573739254	-0.82118733	1.184424571	1.781117904	1.178150322
Actinomyces_sp._Chiba101	0.884975819	NA	-0.046661439	1.720663782	NA	0.939304068	-8.624046047	-8.624046047	-0.075050794	-0.42916987	-0.010718836	-0.680614248
Alkalihalobacillus_halodurans	0.889036625	-2.036833323	-1.048565126	-3.27273045	0.293636095	-1.153112164	-4.085533957	-2.474142615	-3.18789904	2.84238806	3.5986704	3.534551903
Luteimonas_sp._MC1825	0.891404987	0.320813847	1.059706246	NA	NA	NA	0.064889268	-0.3137603	0.11433072	0	9.199672345	0
Profundibacter_amoris	0.891741013	-2.566901523	0.499891528	1.736334632	0.462353389	1.489116932	1.151680794	0.742530949	0.618601915	-0.081560775	-1.400134263	0.271910903
Kordia_antarctica	0.892521353	3.47694838	2.074915707	-1.435187711	-2.022217745	-2.490543936	-0.493062063	-0.584297438	-2.219722759	0.294285097	-0.135352766	0.054070718
Aerococcus_urinaehominis	0.899772552	-1.714548128	3.640638007	NA	NA	NA	-0.250486507	0.164806449	-0.046111806	0.398549376	-0.822004918	1.372050871
Microbacterium_testaceum	0.899863329	2.738689069	3.466678483	0.935180532	1.09422854	1.377345209	-0.413413879	-1.290829052	-0.476170484	-1.141440859	-0.013501318	-0.249523458
Streptococcus_pyogenes	0.906085916	-0.284155248	0.798837422	0.024254287	0.344797799	0.053464259	0	0	0	0.492803077	0.347919887	0.39106721
Pseudomonas_silesiensis	0.908296046	-1.035877036	-4.078002512	NA	NA	NA	0.038710516	-0.222502742	0.605396799	-9.247927513	1.202593	-1.867102844
Streptococcus_acidominimus	0.918641754	-1.317732508	1.127786575	-0.598816427	-0.005140155	0.385581795	0.410353841	-0.225737653	2.274970329	0.729340633	0.137089136	-0.090108745
Microvirga_thermotolerans	0.919354894	-2.120327831	0.062752196	3.168250092	-1.33345243	1.077511576	0.318077212	-8.091313724	3.285403168	1.1111922	2.278480769	1.667393397
Nocardioides_anomalus	0.920464836	-2.05622779	0.888150157	-0.010965559	-0.822730148	-1.623338238	0	0	0	0.90985042	-1.360708694	-1.094683906
Campylobacter_helveticus	0.920548271	-2.296537458	-1.405009247	-6.584517815	-1.673365192	-2.613348426	-0.338382723	0.661112246	0.96404207	-1.377007067	-0.842865627	-1.299691776
Corynebacterium_sp._Marseille-Q3630	0.921251605	2.347675864	-3.982441629	0.058245141	NA	3.272459419	10.85967455	8.382131157	11.08226882	-0.583993364	-1.740197849	-2.416974819
Marisediminicola_antarctica	0.921532867	-0.964031687	1.594346719	0.276388818	-0.279896929	-0.856366391	11.42139689	8.912344481	13.50352763	-0.783589278	-0.807575648	-1.953098927
Veillonella_atypica	0.921544361	-3.162530341	-2.589417558	0.5893321	-0.416496978	-0.29877502	-0.535284137	1.513856339	-0.382355952	1.246025163	-0.873815124	0.554587952
Synechococcus_sp._BIOS-E4-1	0.925633144	-0.686446166	-1.010475444	NA	1.006651441	0.552904621	0	10.11516073	13.08996186	0.873529422	0.350494671	0.645501466
Euzebya_sp._DY32-46	0.927105331	-0.936991691	1.401039136	1.89895675	3.107619425	2.88281745	1.609213178	2.095187483	0.491576407	0.524357821	1.173831995	1.330909895
Kroppenstedtia_pulmonis	0.927263248	-0.31836148	1.569841939	1.445837621	2.176640429	1.773300744	0.227039498	-1.635534379	-0.638757903	0.742021301	0.029470132	-0.202378187
Marinobacter_hydrocarbonoclasticus	0.930892198	-1.713317983	-0.109304891	5.205758059	0.493040011	1.482455261	-0.28531068	-11.98661714	-1.443759384	-8.884680334	-2.06875913	-0.758838123
Halomonas_sp._GFAJ-1	0.938821084	3.081151698	2.283440169	NA	NA	NA	-0.147505464	0.355848554	0.221048795	0.844387774	-0.065187299	0.683756009
Serratia_sp._MYb239	0.940428862	-1.351270231	-0.726522139	NA	-3.902948034	-2.264162834	1.272597205	3.463829365	2.753991255	1.177249384	-9.645058536	1.685483059
Cellulophaga_sp._L1A9	0.941720011	-2.142785661	0.429566791	-3.084949872	-0.154912431	0.421061287	10.17990909	7.68267863	7.745136469	10.26209485	10.65403627	11.61654884
Sphaerochaeta_pleomorpha	0.943534743	-1.33078108	-0.025400694	1.576308582	-0.367499519	0.809049873	1.540025041	0.705541694	0.793819995	1.093033701	0.404882595	0.596643961
Acetomicrobium_mobile	0.944303506	-2.246890641	0.49104955	2.342774756	1.001834323	1.271422635	-0.498853354	0.846594119	0.410766162	1.670996069	1.662908694	1.03718626
Acinetobacter_lwoffii	0.94600739	-1.160005848	0.256833894	NA	NA	NA	0.028930396	-1.453563411	-0.286026918	0.080706828	0.882526737	-0.428350127
Meiothermus_taiwanensis	0.950353982	0.382093549	0.53696914	2.451783983	2.341440751	0.522939012	-9.681078494	-2.998406885	1.672266854	2.004287894	1.879845773	3.251989539
Microchaete_diplosiphon	0.961639526	2.227191411	-2.702333709	NA	3.530358842	2.53456155	0.149796117	-0.036765576	1.413039558	-0.793969653	-1.952691223	-0.351089574
Verrucomicrobium_spinosum	0.961917071	-0.591939471	1.488446349	2.328185251	1.252047116	1.319128968	-0.990564683	-2.075828313	-0.556202982	0.356509987	1.585453242	0.42410849
Pseudomonas_aeruginosa	0.962284272	0.20044401	0.239854873	0.695967838	0.173207835	0.451817065	-0.345107412	-0.040615724	-0.580919187	0.274794416	0.164830291	0.392324528
Kroppenstedtia_eburnea	0.963474124	-0.904993846	-0.617744935	1.436140212	-0.425265912	-0.215081878	0.662100806	1.035634242	2.56535355	0.424870406	0.788084753	0.925581262
Nostoc_punctiforme	0.963510533	-0.41078385	1.040947641	NA	2.266992419	1.45927147	0	9.879304485	8.451211112	0.546028539	0.930185642	0.385196158
Niveispirillum_cyanobacteriorum	0.966052668	0.277904925	1.034112377	0.171880716	-1.191501806	-0.03648482	0	0	0	-1.261332352	1.864262478	-0.347772911
Neisseria_chenwenguii	0.970648802	-1.214314994	0.953292863	1.50424106	0.650419236	0.97054683	-0.452803349	0.592882262	-1.177275588	0.421732245	-0.02126503	0.557024175
Staphylococcus_xylosus	0.979782874	-4.614510233	0.225459381	5.134839493	-1.57623027	-0.504873949	-8.091313724	-8.091313724	0.650766614	0.12459644	0.448310465	0.51909352
Boseongicola_sp._CCM32	0.986972974	-1.619424301	0.384957447	0.795959641	1.48393258	-0.037598512	-1.338087583	-0.867488549	0.738362612	-0.133333404	-1.084521994	-0.853362814
Corynebacterium_sp._sy039	0.987509056	2.736343609	2.175442006	NA	NA	-6.366016461	-8.10567284	-1.423001232	1.529963667	0.271190615	-0.597866135	-0.419852284
Swingsia_sp._F3b2	1	NA	1.487672805	-0.781191916	NA	-1.038087185	1.863625736	2.461014918	1.392149989	1.62796346	-2.28901182	0.397173031
Leptothrix_cholodnii	1.004607954	-2.175910168	-0.4975316	-3.760523884	-1.563487037	1.303638326	0	0	0	10.67771964	11.67830523	7.539158811
Campylobacter_upsaliensis	1.005568968	0.147532389	1.5848137	-4.615222206	-2.937792466	-4.240402815	0.574834338	0.189377154	-0.432575588	-1.044246499	-0.881636972	-0.967576462
Mannheimia_granulomatis	1.006734585	-2.726813404	-1.034344727	-0.666295313	-0.227712753	-0.835079504	0.393723846	1.002060836	1.003551451	3.445255197	3.955762383	3.439194147
Ignavibacterium_album	1.010804277	-1.55677746	-0.815261184	NA	-0.658371872	-1.161419621	-0.88469103	-1.83795599	0.879335095	1.215383126	2.279267571	2.12030431
Enterococcus_gilvus	1.015804741	-1.883280511	0.571747423	1.40381497	0.103122517	0.431462253	0	0	0	0.958894949	0.703059635	0.669889389
Pseudomonas_xinjiangensis	1.027680246	0.850512504	1.055566369	NA	NA	NA	-9.388017285	-9.388017285	1.123290634	7.129283017	0	0
Nitrosomonas_eutropha	1.032332502	-1.021061616	-0.961806846	1.18759185	2.0114842	0.361448668	0	10.68187966	9.554239647	-0.096969242	1.007683721	-2.617443016
Paenibacillus_kribbensis	1.037494185	NA	-1.417318441	NA	3.609931886	2.212282394	0.410176248	0.16907485	0.799711724	-0.813080011	-0.938891694	-0.167619061
Candidatus_Puniceispirillum_marinum	1.04336104	NA	-2.155794673	NA	NA	1.478462879	-0.359565862	0.925619712	-0.352252463	-8.818048457	-0.358616839	2.345179795
Thermoanaerobacterium_xylanolyticum	1.044455745	-1.452533612	1.062874212	0.019929684	2.032348168	1.577244588	-10.33722048	1.49538081	0.251915663	1.008255045	0.985586314	1.17677327
Ochrobactrum_pituitosum	1.046129171	NA	-0.841905673	0.989208642	-0.202478838	-0.034715472	-1.170904112	-0.403530047	0.491594769	-1.54896615	-1.066749868	0.026613631
Vogesella_sp._LIG4	1.046612093	-2.450067043	0.461842298	5.363470357	1.36975662	2.520386932	0.294171442	-1.429239578	0.966207601	0.528039255	0.785845052	2.017921264
Actinomyces_sp._ZJ34	1.04761802	NA	2.790310225	0.787307887	NA	0.945220371	0.643288463	0.011561184	1.545136495	1.319321525	-2.045229185	-1.85699242
Gardnerella_vaginalis	1.059210656	-1.39595805	1.338376135	-0.265252491	-0.82730718	-1.14709953	0.859766962	0.377602244	2.181040841	0.378159912	0.381346904	0.145054209
Streptomyces_sp._Z423-1	1.059734665	-0.334601925	1.790674784	0.296856073	1.357156583	NA	0.687038657	-9.995639973	-0.080574767	1.144549548	0.702828432	2.279127205
Streptomyces_hundungensis	1.059734665	NA	-0.700965577	3.087069637	-1.14343543	-0.67406733	1.210537683	0.118913915	1.448219642	0.355480655	1.660730008	-8.087462841
Corynebacterium_urealyticum	1.059734665	NA	1.527761945	NA	-0.580453435	-0.93105812	-1.06209173	-0.075800588	0.490968328	0.007271068	-0.680768959	0.351871756
Tatlockia_micdadei	1.059734665	NA	NA	NA	NA	NA	0.558501995	1.823524207	3.870168094	0	0	0
Desulfuromonas_soudanensis	1.063529006	-1.406635004	0.456240981	1.038918025	-0.408035954	0.98597953	-0.573972029	-0.148758431	0.357161585	-0.08657746	0.630242001	1.083034422
Conexibacter_woesei	1.069513286	-2.209913221	-0.158374628	1.461851135	0.082653094	0.847185156	1.09761134	-8.091313724	1.159200253	1.368306137	1.087359604	0.946844552
Chryseobacterium_sp._JV274	1.071949842	-0.039892727	-0.756404769	0.468170755	NA	-0.773196061	0.596532758	-0.206794633	0.305855547	-2.48520995	0.611888738	-0.240810344
Streptococcus_dysgalactiae	1.079616852	-1.964265117	0.608914696	0.620832457	0.022986459	0.400181212	0.23699726	2.468840255	2.452764297	0.650602053	-0.131418001	-0.053461245
Actinoplanes_missouriensis	1.080533298	-1.659611206	0.780402958	4.835636472	2.569571193	NA	-2.053565458	-1.279935832	-0.19377004	1.89205944	0.904343404	1.064379228
Tessaracoccus_timonensis	1.083367182	-0.619261651	0.62214005	-0.166608166	4.046467069	3.528750156	-0.029736769	-0.091054715	-0.292644526	-0.246422583	-1.513802568	-0.350303836
Pseudomonas_sp._LAB-08	1.088242794	-1.631384301	-0.980968739	NA	NA	NA	-0.851697391	0.808651042	0.857364904	-7.807354922	-7.807354922	2.159395645
Caldimicrobium_thiodismutans	1.089637212	-1.26811656	1.448678323	1.657584239	-0.921256885	1.503534901	-0.030544324	0.080334295	-2.189197584	0	10.37837073	0
Cupriavidus_basilensis	1.101328955	0.822654058	3.42099715	NA	-0.44633363	-0.909989834	-8.799135887	0.453529545	-1.642021419	9	9.330916878	9.642652841
Paenibacillus_sp._JZ16	1.102763447	0.083515954	0.952689695	-0.957735904	0.886062969	2.077372612	8.129283017	7.68267863	9.578457609	-0.039167374	0.108361123	0.038364899
Dickeya_paradisiaca	1.103019208	-1.793877418	0.784311017	-0.168858314	NA	2.995725345	1.893505867	2.944881098	4.651973807	1.944128096	2.741728694	-0.543139749
Komagataeibacter_europaeus	1.103629472	1.168388748	-0.470442295	-0.900685446	1.580533656	0.592680414	-0.463024972	-0.85962519	-11.62071927	-2.077041757	-9.380822505	-9.380822505
Streptomyces_nitrosporeus	1.114634561	-3.797589479	-1.009831995	NA	0.23624526	0.352406511	0	0	0	1.957815684	2.741008235	1.496937753
Ndongobacter_massiliensis	1.118342897	-0.990425473	-0.201261379	0.382706846	0.549737006	0.74389004	-8.799135887	-8.799135887	0.481212682	0.158492118	0.157614797	-0.112832483
Corynebacterium_choanis	1.119062502	-1.099677524	-2.092602007	-1.42370236	0.998649541	0.185201862	0	9.426646347	0	0.820962678	1.409610249	0.972516824
Candidatus_Protochlamydia_amoebophila	1.122049871	0.22521294	-0.598198328	NA	NA	NA	0.951005051	0.02598371	1.3665915	-2.724093967	-4.585503951	-3.127254385
Serinicoccus_chungangensis	1.123148231	-4.854030808	0.209304875	1.408951157	1.147323573	1.556019545	-0.057081803	0.693060001	0.878805028	0.50425516	1.094270309	0.37621024
Prosthecochloris_sp._HL-130-GSB	1.123735368	1.685454869	1.51473466	NA	NA	NA	0.534909897	0.64209218	0.744819081	-0.002482506	-2.539782178	0.282951067
Vibrio_casei	1.133071277	NA	NA	NA	NA	NA	-8.091313724	-8.091313724	0.532732323	7.333897756	0	10.71424552
Calothrix_sp._NIES-3974	1.134441288	NA	0.203644077	-1.351299524	NA	-1.083583112	-0.094041275	0.68915815	0.852893537	-0.869455536	1.18335059	-8.902878538
Paenibacillus_larvae	1.14888658	-1.259039624	0.07664353	0.337409332	0.874538341	1.18069766	0.044674571	0.219362608	1.352682745	1.821876677	2.177057784	1.118760699
Shewanella_pealeana	1.149337941	NA	1.776174662	NA	NA	-2.158330192	-0.397224328	-0.330616556	-0.292962655	0.736151299	-0.169099951	0.477157969
Paenibacillus_barcinonensis	1.154119089	0.088094538	1.769104704	1.510857757	-0.512852208	-1.246399092	0	0	0	-0.346861144	0.494583134	-0.264036587
Nitrosospira_multiformis	1.163611155	1.635945221	-0.895698037	-1.421635337	1.638744605	2.501275362	-1.004972121	-1.987101514	-0.090142639	1.960661593	2.215489584	2.95940493
Frankia_inefficax	1.164038158	NA	1.590992583	NA	0.531654614	-0.331901655	0	0	10.03116358	1.131630213	-0.832610287	-0.573112783
Paenibacillus_sp._E222	1.17490869	0.44576728	0.359618915	0.32553262	-0.211069542	0.301785536	-0.66960915	-1.151655913	-1.029361336	0.093498108	0.412020347	0.094983932
Candidatus_Kuenenia_stuttgartiensis	1.176322773	-1.324028088	0.241859539	0.173226365	-0.383571157	-0.633167081	-0.024666014	0.265214974	-0.081661654	0.97934974	1.087776191	0.81985159
Lactobacillus_mucosae	1.180673172	-2.602632105	-2.895229953	-0.268541034	-2.449959384	-0.681957688	0	0	0	-0.351679159	-0.60668572	0.117746924
Cupriavidus_pinatubonensis	1.184527159	-1.463283944	0.085877835	NA	-1.583623158	-2.28808464	-1.006238	0.500274137	0.17147079	-1.527010789	-1.003820738	0.464968935
Virgibacillus_phasianinus	1.184539208	-0.496720137	1.84399083	-0.519179082	1.376162192	1.922386456	0.041612386	1.125549994	1.210927591	1.639823524	2.167047973	1.624772917

Bacillus_mobilis	1.198253725	0.080151939	1.946573593	NA	1.917173569	1.575125317	0	0	6.15711649	11.63026713	11.85216664	10.89532535
Cellulomonas_gilvus	1.200397354	NA	-1.887916202	NA	NA	NA	-0.003388217	0.264160496	-0.109825153	7.303780748	0	0
Bacillus_lentus	1.200990957	-1.715958593	0.576531614	-0.399935588	0.116387252	-0.028612186	0	0	10.78135971	0.781099636	-0.147454504	0.738679981
Thermosipho_melanesiensis	1.201633861	-0.178782425	1.761872152	-2.56399588	-4.53416065	-2.980469611	0.467383103	0.470387359	0.769283263	0.108609887	0.710441044	-0.149567632
Serratia_fonticola	1.201784591	-1.47343771	-0.567730943	2.030099548	0.167537479	1.008185823	-0.705103744	-0.06382359	-0.170744814	1.43224963	1.374692221	-1.424626534
Paenibacillus_graminis	1.202348665	-1.090169245	-0.205769902	1.356560695	0.394731147	0.161297577	0.405867823	-8.624046047	-2.466929557	0.938302276	1.365645771	0.009353244
Variovorax_sp._PBS-H4	1.202525675	-1.068161624	-0.350458968	NA	-0.540345726	2.024288369	-10.27400535	-10.27400535	0.162612512	-0.078107442	1.293458074	0.383637451
Pandoraea_pnomenusa	1.207394417	-0.643218073	-0.144389909	0.551177653	0.688635809	NA	0	0	0	10.8008999	8.803055861	0
Mycolicibacterium_helvum	1.213636492	NA	0.816692787	NA	0.120473718	NA	0	6.682671608	0	-0.742681343	-0.112128009	0.713491252
Laceyella_sacchari	1.218377568	-3.428782201	-0.752671812	2.625227498	-0.038454234	-0.454879729	-1.164808572	-1.211502207	-0.841045015	1.942527135	2.218241916	1.453674545
Streptomyces_cyaneogriseus	1.225941488	1.644539145	2.355852288	NA	NA	NA	1.029610659	-0.620821472	-12.32448717	8.81164121	0	0
Clostridium_sp._JN-1	1.230398131	-1.338362902	0.036379248	0.358722685	0.176300039	1.024491235	0	0	6.15711649	0.775080632	0.892564945	1.102388888
Aeromonas_caviae	1.232194116	-1.853207964	0.869872104	1.388479288	4.103940617	2.911835571	0.609781296	0.907636142	-9.681078494	1.614555914	1.396736316	1.481829044
Halomonas_sp._THAF5a	1.238372352	-2.028932492	-0.883931745	NA	NA	NA	-0.219684415	0.622909373	-0.468871414	8.27301694	8.425144554	9.306820443
Mycobacterium_sp._ELW1	1.243298188	0.79113457	0.443376023	1.722968443	1.243557031	0.32788555	0	10.35434857	8.944644719	8.282314694	0	0
Yersinia_aldovae	1.243979859	-0.624999806	-2.097675931	-4.743110334	-1.530806731	0.087514367	1.972449008	-9.49367335	0.628572274	1.623813421	-0.491136129	1.334106102
Flavobacterium_nackdongense	1.25023813	-3.719219553	1.93474755	-4.344768071	0.317590979	0.36684399	-1.338371683	0.585287606	0.004855401	1.722069627	1.691682392	0.915393187
Gloeobacter_kilaueensis	1.252371732	-0.862988909	1.123689713	2.233248582	1.267728282	0.499177797	8.409390936	0	0	0.121913067	-0.344015425	-0.202205358
Frankia_casuarinae	1.258175674	NA	-2.605901838	NA	NA	NA	0.307567451	0.951695744	2.539665343	-0.626956589	0.982151999	1.907415546
Starkeya_sp._ORNL1	1.262630911	-2.072472572	0.954133841	2.374974329	0.878883283	0.939304068	0	0	6.15711649	-2.330852666	-0.756101381	0.880302435
Acinetobacter_wuhouensis	1.262630911	1.263639436	4.203684675	NA	-1.986130493	-0.360163084	-1.009668675	-0.289274898	0.254118407	0.455340047	0.541978348	1.205766162
Undibacterium_parvum	1.262630911	NA	1.329075424	NA	0.477217327	-1.206969553	-0.276431779	0.040130092	0.634595001	-0.09716251	1.767846691	-0.621496071
Streptococcus_equinus	1.26281916	-1.608828993	-0.458216717	0.048078573	1.006217492	1.021408893	-2.495832212	1.150745371	0.841211145	1.054597676	2.119294712	1.511742986
Stella_humosa	1.263321538	-1.336068033	0.110520714	3.577907743	3.086220388	2.298515269	-13.6188443	-4.234746993	-5.313402671	2.791323378	2.096028077	-0.198517832
Paenibacillus_sp._32O-W	1.265750606	-1.210245064	-0.738719544	-1.110488887	0.422513542	0.401950521	0	0	0	0.948235107	0.327028077	-0.534990812
Labrys_sp._KNU-23	1.271238864	-2.847702208	0.426778724	-0.338256265	-2.447880756	-3.288304443	10.37226653	10.35434857	10.83834516	9.332410325	7.712410067	8.741466986
Nitratiruptor_sp._SB155-2	1.273815716	-0.235616772	-0.597468914	-3.546397934	NA	-4.75961977	-9.384097309	0.873938658	-2.22698284	0.071302673	0.096842806	0.159938068
Lactobacillus_johnsonii	1.275643152	-2.348961813	-2.592374912	-3.654822421	-1.338953849	-0.07651424	0.910375631	2.080055317	1.667592693	-0.885673223	-0.502929898	-0.024382963
Sphingomonas_rhizophila	1.291766124	2.227712753	1.166973949	NA	NA	NA	0	0	9.598899253	-2.210423405	-0.222393065	-1.354496601
Pimelobacter_simplex	1.294034089	2.790742466	3.238743504	1.790716256	0.313216726	-1.897572187	-0.068597277	-9.098511147	-1.353374678	-0.84200595	-0.420262414	-3.047680977
Anaerobacillus_isosaccharinicus	1.296524386	0.182101196	0.153302805	-1.653076001	1.062078844	0.500842293	-3.146351136	-0.317761829	-0.762245917	0.037959169	1.433412272	1.005721885
Sporosarcina_sp._PTS2304	1.304276921	-1.305695526	-0.253494379	1.945905739	0.750113012	1.890817304	-9.739012655	0.63523351	-3.581896165	-0.424224302	0.260662633	-0.249935933
Corynebacterium_glutamicum	1.308671998	1.361836816	1.822357085	-1.440117296	-0.352044048	-0.896093356	0	7.68267863	0	-0.278992709	1.904517817	1.699701535
Arsenophonus_endosymbiont_of_Aphis_craccivora	1.309756525	-1.688124705	0.182171888	NA	NA	NA	0.38400292	-0.083667904	-0.267403325	-9.741466986	-0.751646766	-0.305448854
Saccharomonospora_glauca	1.310819309	-0.053226613	-0.37064338	1.7589919	0.86106381	0.410914742	0	0	0	0.726594624	0.595559061	-0.719446733
Undibacterium_sp._YM2	1.319408103	0.037281016	2.16964522	NA	NA	-2.078533403	-0.025569975	-0.778545251	0.154172792	0.737615639	1.288911406	3.217018487
Campylobacter_ornithocola	1.325974928	-2.566440345	1.492933362	1.919931728	0.980576737	-0.19970584	-5.407275492	-3.563518249	-3.746368084	3.368972542	2.621011712	1.926844197
Novosphingobium_resinovorum	1.340682055	NA	-0.654004145	1.514045261	-0.017702002	1.287746159	1.451423393	-3.100177574	2.374841962	1.560424552	2.297676182	0.321928095
Zhihengliuella_sp._ISTPL4	1.342248079	0.874469118	-1.425305835	NA	NA	NA	0	9.426646347	9.591268006	0	6.470320477	6.930737338
Blochmannia_endosymbiont_of_Colobopsis_nipponica	1.342248079	1.460919125	0.164229396	NA	NA	-0.72635192	-0.316351343	-0.65577443	0.386351025	0	8.297682077	0
Candidatus_Hepatoplasma_crinochetorum	1.342248079	NA	2.282197921	-1.368542575	-1.835722595	-1.587572426	-0.311040812	0.245272688	0.796315119	-0.745838468	-2.397017275	-2.768486294
Nocardioides_sp._S-713	1.342248079	NA	NA	NA	NA	NA	-9.098511147	1.629715508	-9.098511147	0	0	0
Candidatus_Riesia_pediculicola	1.35938118	-0.054123517	0.790757416	NA	NA	-0.124755031	0	0	8.548995253	-1.846649223	-9.741466986	-0.12921218
Granulibacter_bethesdensis	1.359695403	1.505439364	0.933277362	0.781085552	1.460097222	2.067535128	0.392629675	1.062839899	2.584961293	0.425992531	0.027363498	-0.167207462
Sulfurivermis_fontis	1.359992611	-0.588349906	0.353254429	1.621019304	1.978508926	1.84017594	0.960268798	2.723058656	0.493100633	0.903148705	0.458626768	1.216056465
Micromonospora_echinaurantiaca	1.360402243	-1.805063111	-0.719487379	NA	NA	NA	0	0	0	0.748742762	-7.930737338	-7.930737338
Sulfurovum_lithotrophicum	1.36306679	-0.142430205	NA	-0.321928095	2.23789198	1.667033634	-1.493949239	-1.392105535	-2.453568692	0.236438369	1.895516778	0.277780003
Mycoavidus_cysteinexigens	1.367371066	-0.293358943	NA	4.510345616	3.708315389	2.613496336	0.015521825	0.916306354	0.42699241	-1.586991195	-8.890771943	-2.346451942
Rhodospirillum_centenum	1.370500547	-0.258389792	0.884441745	NA	0.919450087	NA	0.429339082	-1.289471682	-0.430510724	1.245911861	-0.29971445	0.661375616
Microbacterium_sp._XT11	1.37888398	NA	-0.176414759	0.568194287	1.492280498	-0.596550475	10.09850588	10.19899746	11.09312041	0	0	9.399455246
Paenibacillus_sp._37	1.389293635	-0.29224714	-1.702677875	2.913685735	0.357634153	0.422735624	3.909801545	3.943028762	4.210896782	-0.626204404	1.024607904	0.086566727
Cohnella_sp._MFER-1	1.389755723	-1.814773682	-0.48337836	1.91636141	0.553651215	-0.096445117	-8.04736934	-8.04736934	1.004951448	0.876417908	0.679760696	0.760137237
Streptomyces_sp._11-1-2	1.392647144	NA	-2.510292664	0.493647334	NA	-0.676687582	8.502740328	0	8.48088308	-0.517475197	-1.909234536	0.039275149
Glutamicibacter_mishrai	1.393994874	-0.542447971	-0.468270382	1.249832949	1.84491549	-0.250727181	0.477604829	0.271878775	2.269942988	-8.087462841	0.6212762	-0.54830403
Campylobacter_jejuni	1.39817593	0.337924277	0.901704087	-1.150087221	0.359969788	1.162038836	-0.547712139	0.197100071	0.187042957	-0.744785258	-0.911855101	-1.13604612
Chromobacterium_vaccinii	1.399158217	-0.342622671	1.561911753	2.240416103	2.242856524	2.478540442	-0.237960057	-1.182736335	0.034778588	-0.584209496	-1.111357362	-2.78135339
Sphingopyxis_fribergensis	1.410550108	NA	-1.273466606	NA	NA	NA	1.146407881	-8.909620116	-0.005464214	-7.930737338	-7.930737338	1.056051857
Pandoraea_sputorum	1.410993502	NA	-0.401305417	NA	NA	NA	-9.384097309	0.125857774	-0.479941407	0	9.650572508	10.80896417
Synechocystis_sp._PCC_6714	1.418888875	-1.09845117	-0.020314935	-0.082936341	1.755782752	1.703536296	0	0	6.15711649	0.84359161	1.901169186	-1.03492321
Bacteroides_sp._HF-5287	1.431063524	1.645742515	0.895032897	-1.36809328	-0.352027345	-0.324089812	-0.090212586	0.423685679	1.159071549	-0.150608652	-0.385282743	-0.368780435
Paenibacillus_glucanolyticus	1.436784079	-1.716021002	0.269358416	NA	1.031595807	-1.389997753	-10.8709272	-10.8709272	0.577473073	1.150044273	1.246531881	1.336588578
Nocardia_mangyaensis	1.439815351	-2.478252794	0.369410116	NA	-1.582549967	-0.660552479	8.246199041	0	0	2.045483639	-1.04833397	-8.64625988
Basilea_psittacipulmonis	1.44287887	0.090436313	1.305871601	NA	NA	-1.09036804	0.395617306	-0.050504224	0.32742358	-8.77478706	-8.77478706	2.921602404
Neorhizobium_sp._NCHU2750	1.44909641	2.110571804	1.004673457	0.183307213	2.610486707	2.947515344	0	0	0	1.493972171	1.750130716	2.479454321
Rhizobium_etli	1.454745331	0.627494006	1.595438834	0.980244705	1.858219766	1.331590576	0	8.382131157	0	1.125590545	1.059825767	0.9563723
Streptomyces_sp._W1SF4	1.465080709	NA	-0.264814412	NA	NA	NA	-9.557724072	0.913951143	0.213164425	0	0	0
Corynebacterium_atypicum	1.466804761	-1.208510109	0.321557243	0.734971544	-0.762880293	0.998338483	-0.249374417	1.792744975	2.806253985	0.15739044	2.169439402	0.622869509
Friedmanniella_sagamiharensis	1.4669831	-0.812569589	0.637285989	3.019016027	3.543744705	2.349864971	0.105350736	0.107894854	0.81338819	-7.741466986	0.161408537	0.431795394
Caulobacter_vibrioides	1.48288601	-3.326230564	-0.036385685	NA	NA	1.383914982	10.38016882	11.57158201	9.691425987	0.881870811	0.669437247	0.941040588
Bacillus_sp._FJAT-22090	1.486411327	-0.695981279	0.350964135	-0.093044673	0.984772496	0.197685404	-0.714289311	0.457595823	-1.302151421	-1.499984034	0.598515092	-0.768280423
Paenibacillus_sp._IHBB_10380	1.488556856	-2.690541257	-0.909375859	-2.03996017	-0.623069132	-1.159485423	-0.334020268	0.777722057	0.081527546	0.105663877	-0.68158762	-0.559476761
Dialister_massiliensis	1.489513258	-1.772467982	0.584406843	2.375257397	0.111793	0.653608005	1.104920267	0.748448551	0.457916459	1.966093634	2.176298516	0.5324175
Pediococcus_inopinatus	1.492701921	-2.082694684	-0.298243353	NA	NA	NA	-0.959143489	-1.372993513	-0.034036464	0.465662018	-2.334867151	2.07120644
Arcanobacterium_sp._JY-X040	1.49801458	0.36421718	1.584632855	-0.766480879	-0.394170849	-0.235058833	0.190031498	0.454031631	1.025849523	2.522925393	2.161180996	1.394785369
Paenibacillus_ihbetae	1.498290967	-2.2898139	-0.266314608	0.282991104	0.672748577	0.741130936	0.397067487	1.320973507	1.897015617	0.596105133	0.494358953	0.134922356
Rahnella_sp._ERMR1:05	1.503677047	-1.327396308	0.927243658	2.631052512	-0.379835804	0.777522732	-0.912537159	-9.321928095	-9.321928095	-10.35388563	-0.995454203	0.87212308
Sphaerospermopsis_kisseleviana	1.504231229	NA	-2.702333709	NA	NA	NA	0	0	0	-0.503574174	-7.807354922	-0.268196111
Acidovorax_cattleyae	1.505345333	NA	-2.499437464	NA	NA	NA	-0.009770796	-1.395159217	2.601100943	2.236081497	-0.930035414	-7.745956618
Paracoccus_liaowanqingii	1.509441751	-0.828151749	-0.616460147	2.186318317	-0.403759431	NA	-0.065771566	0.225132243	0.178950571	1.09860802	1.228716633	0.806988406
Hydrogenophaga_sp._BA0156	1.51009458	0.185271582	-0.682314436	NA	NA	NA	-0.817264466	-0.675499068	-1.678437055	0	6.815921204	0
Enterococcus_wangshanyuanii	1.517028529	0.687843701	1.741268391	3.090576804	-0.259692813	0.14774838	8.409390936	12.34353829	11.4799621	0.159355858	1.424190829	1.583190157
Actinomadura_sp._WMMA1423	1.521679848	-1.783722077	0.750021747	NA	0.857256881	0.314765005	-0.348174246	-0.897665238	0.880966806	1.539252376	1.174860135	0.655762427
Salinibacterium_sp._ZJ70	1.532100935	-3.36379407	-1.643758871	NA	NA	NA	-0.977302619	-0.634168682	-0.783127659	9.020516091	0	9.254242075
Leptotrichia_hofstadii	1.541170131	-0.140877969	-0.669550492	-3.14362172	NA	-1.759706738	0	0	0	1.142180011	-0.443950775	0.786095962
Staphylococcus_felis	1.544377214	1.435822991	-0.771101934	NA	NA	NA	-1.517847766	-9.927238702	-9.927238702	1.932134293	0.287885711	0.114106486
Mycoplasmopsis_californica	1.546135199	-1.357233717	1.089714582	-3.699540512	-2.853551696	-2.545520331	0.477414076	-0.286984708	2.122179108	0.462901383	0.600578817	0.505727786
Nitratireductor_sp._SY7	1.553512862	-2.04342228	-0.320956256	NA	NA	NA	0	0	0	0.701476509	0.330354664	-1.197146985
Aeromicrobium_sp._zg-629	1.565207883	0.540364862	0.931394153	NA	2.672048117	2.692427198	0	10.22005178	10.99628281	0	6.815921204	9.833944757
Sagittula_sp._P11	1.579837469	NA	-2.419820296	NA	4.045886158	3.040336504	-1.713980529	-0.229221637	-0.712693863	-0.612836879	-8.64625988	-8.64625988
Formosa_sp._Hel1_31_208	1.580992038	2.714229334	1.405767908	-1.983781489	1.16447806	2.329912694	0.937515158	-0.571498251	1.208016949	-0.656689342	-0.708848454	0.976995216
Paenibacillus_protaetiae	1.581049629	-1.649716909	0.164671958	0.9453631	0.472694464	1.16037225	-1.676451303	-0.746108782	-0.11105271	1.895382654	1.839807936	0.713820154
Pantoea_ananatis	1.581834745	-0.280732936	0.718412809	0.642289207	-0.325144641	1.181336825	-0.166897956	0.997060098	0.239602157	-0.778489871	-0.538554144	-0.111566505
Mannheimia_ovis	1.584399608	1.819603613	1.360246728	-1.402668942	0.331478888	1.173666382	-2.767321853	-0.265928206	-1.305336865	-1.035404566	-0.228530064	0.535591413
Streptacidiphilus_sp._PB12-B1b	1.610249608	-1.491853509	-1.12186886	1.953165914	0.044306153	NA	-0.26005116	1.227588947	0.418131747	-1.258371211	-3.417407354	-2.253519391
Neokomagataea_tanensis	1.614464174	-0.37223179	-1.702138765	NA	-0.903554883	1.768674454	0	0	0	-7.741466986	-7.741466986	-7.741466986
Streptomyces_sp._M2	1.621799269	-4.675288698	-3.465663572	NA	NA	-2.47158792	-1.794250492	-0.311763472	0.028666702	0.156403293	0.576862085	-1.004704619
Candidatus_Aquiluna_sp._15G-AUS-rot	1.623399844	-0.873543898	-2.422998297	-1.105309348	0.858204509	1.805398585	-8.637284195	-8.637284195	-8.637284195	8.930737338	8.660591394	11.33166156
Desulfovibrio_sp._G11	1.628235209	NA	-2.867549704	NA	NA	-5.677165207	0	8.382131157	8.624046047	0.931231375	1.54252889	1.243522458
Chryseobacterium_sp._F5649	1.629712007	NA	-2.52466199	NA	NA	NA	-8.624046047	-8.624046047	-1.466931578	0.355480655	2.793651119	-8.087462841
Paenibacillus_guangzhouensis	1.631643807	-1.496229639	-0.508077542	0.487261519	0.239394317	0.451271662	0	9.426646347	8.84263634	-0.500842713	1.03734225	0.008848141
Photobacterium_profundum	1.638993287	-1.246371677	2.556899839	4.076253033	5.134552417	4.437522125	-0.930615216	-9.960529086	-1.411533833	-1.166143901	1.411460336	1.914594359
Neisseria_sp._10022	1.644630006	-0.190496611	0.010070089	5.420347653	3.161780215	2.549258439	-9.685863079	-9.685863079	-9.685863079	0.843274496	-1.271541637	1.784955024
Pseudolabrys_sp._FHR47	1.654929825	-1.405243416	-0.644158293	-2.176426567	NA	-0.485945876	0.31757319	0.272270053	2.583756045	1.741555161	-7.709655951	-0.075447511
Paracoccus_denitrificans	1.666219483	0.092847072	1.210955538	NA	NA	0.680775653	1.696146869	0.303451995	-0.443178235	0.00773514	-3.192644776	-0.613055694
Dehalogenimonas_lykanthroporepellens	1.667384725	-2.722660122	1.694289809	5.043327432	3.61427891	1.127874823	-0.340461839	0.083739897	-0.853675067	2.2864146	2.14518966	2.072548657
Streptomyces_sp._Rer75	1.690728923	-0.753839413	2.299953405	NA	NA	1.817366304	0.160627241	1.448435066	-3.57265888	0	9.869953123	9.649854332
Anoxybacillus_gonensis	1.694124644	0.442954573	2.577232033	NA	0.827493004	1.150594428	1.878437624	4.154732613	4.641132805	3.47819952	2.890570792	2.494447218
Dictyoglomus_thermophilum	1.694928911	0.986264975	-1.707819249	1.405974877	1.141121095	-0.117718208	0.444648908	-0.369282227	0.038698155	0.62316722	-0.237140725	1.060493056
Pseudodesulfovibrio_piezophilus	1.696890209	-2.712478234	1.226553404	NA	0.945033541	-0.736965594	0	0	0	0.220622279	1.025789939	-1.490140082
Steroidobacter_denitrificans	1.703503173	0.250312329	1.389110245	0.699120209	0.129352998	0.591885821	0.625384417	0.782531948	2.716965951	9.930737338	10.51897765	8.520944699
Paenibacillus_sp._HB172198	1.705079723	-0.740838322	0.663548051	0.766099483	1.987226937	2.142590656	0	11.35434857	0	0.079364871	-0.445250429	-0.072118655

Sandaracinobacter_sp._M6	1.707104441	1.009771557	0.062631446	-2.369903274	0.860436882	-2.911415518	1.820920474	1.369931751	1.272414901	-0.952923614	-0.645533539	-0.740900522
Azoarcus_communis	1.711113034	1.395070258	0.443022405	-3.920316542	-3.201052949	-3.065169521	0.177591222	1.841541993	1.227964821	1.242625812	0.814553172	1.57994501
Gloeobacter_violaceus	1.723408274	-2.716978315	-3.013788207	4.427680519	1.128217712	-0.43486972	0	0	0	-0.621707282	-0.086182911	-2.351637128
Arcanobacterium_haemolyticum	1.731008539	0.875128033	0.431616241	-0.654755797	1.01356245	0.958530461	0.211676311	0.36278196	0.210978712	0.904303271	0.409642353	0.383471933
Dermacoccus_sp._PAMC28757	1.735156984	-0.474296381	0.852189995	NA	NA	NA	1.426527797	0.687165659	0.554587679	0.086491622	-8.186525318	-8.186525318
Francisella_philomiragia	1.738208761	0.905784658	NA	-2.349474422	-2.169925001	-1.689256528	-0.053312027	1.001880294	1.314564567	0.92942584	0.971741054	-1.018051046
Achromobacter_spanius	1.739411594	-2.00546286	-0.92149976	-1.468963381	1.287036883	1.680360351	-0.483898247	-1.222388423	0.145119434	-0.928716162	0.385504959	-0.033082382
Pantoea_dispersa	1.750480401	0.43710238	1.765534746	2.021523551	-0.036792031	-0.439460973	-0.202980925	1.088785421	0.527819676	0.123302748	0.541153341	0.55402191
Paenibacillus_sp._B01	1.753591425	-0.367501103	-0.016346142	2.12375866	1.61811219	1.300286264	-10.04984855	-1.442518236	-10.04984855	0.43887933	0.329334463	0.65769075
Paenibacillus_sp._IHB_B_3084	1.7693123	-0.753509581	-0.346055253	0.638307998	0.084356126	1.00870049	0.150029462	0.751240874	0.846402595	2.848713352	2.636916146	2.38345487
Xanthomonas_cucurbitae	1.776225025	1.571076974	0.610710922	NA	NA	NA	0	0	8.451211112	8.920850045	9.199672345	0
Leclercia_sp._119287	1.786169592	0.861949579	0.356012363	NA	NA	NA	1.272597205	3.148889992	1.846647135	1.018125045	0.825765933	-7.890768904
Cytobacillus_oceanisediminis	1.788550587	-0.148738273	0.626563405	0.836777106	0.226643097	-0.140095336	-0.164035732	-2.221828935	-10.60396009	2.607702451	2.551755379	2.846233495
Helicobacter_pylori	1.804892219	-0.568896245	0.380000128	-5.110019478	-3.520017478	-2.599195336	1.341299261	1.229979369	1.405894658	-1.191244473	-0.855041912	-0.825673372
Oenococcus_sicerae	1.816463514	NA	2.25352732	NA	2.06030051	-0.725283789	-8.637284195	3.077352	1.054141792	2.229900103	1.7060421	-0.129220711
Paenibacillus_rhizovicinus	1.819589164	-0.805153239	-0.566294673	1.020385395	-0.145323688	-0.986458009	-0.488534955	0.145552053	-0.824823458	0.193124062	1.125649789	0.388684195
Gramella_salexigens	1.826463745	1.099535674	-2.900036925	NA	NA	-0.835337335	-0.20525431	-0.605207274	-1.042026512	-0.149658534	-2.210700279	0.288639407
Mesorhizobium_ciceri	1.841554495	NA	0.10122205	-1.495331547	3.648068502	3.438499876	-0.028110525	0.769409189	2.953695589	1.329822397	0.966039013	0.5894878
Mycolicibacterium_fallax	1.853095467	-1.321928095	0.579009728	NA	NA	-2.051865369	0.034928517	-0.126920213	-1.623449821	-8.64625988	-0.463036402	1.111963334
Paraburkholderia_aromaticivorans	1.870162562	1.593537236	1.725524996	2.784828725	1.286940795	1.16567374	1.298559745	1.211461093	-0.511022467	1.045164731	-0.890375509	0.741708243
Gramella_sp._MAR_2010_147	1.873050087	2.168972952	1.503255933	-1.804647678	0.341093965	1.572989859	-1.307592533	-0.296665563	-1.448666161	-0.620134096	-0.266145722	0.871321359
Streptomyces_olivoreticuli	1.889423334	-1.950961798	0.836005127	5.557484245	3.834301494	-0.286757317	-8.10567284	0.004753048	1.699337408	1.517492349	2.413009391	2.039892727
Lysobacter_gummosus	1.898523462	-1.436126894	0.626085391	NA	1.469009068	0.48133987	-2.137047707	-1.15612172	1.006333936	2.45795925	0.004048859	1.553556513
Cronobacter_condimenti	1.910077867	1.34112	-2.966211687	0.475930916	NA	NA	-0.611688738	-9.488205685	-1.007322605	0.97663811	1.580265898	0.318118925
Candidatus_Bipolaricaulis_anaerobius	1.923053338	0.166690537	2.914772163	1.227462658	0.609293505	-0.875805101	-2.695357564	-0.491974082	-1.484312398	1.500600398	1.055146182	0.295903074
Lactococcus_piscium	1.92366517	-1.429222108	2.59036862	-5.108524457	0.08243709	0.496195415	-0.640457613	1.330320267	0.952839908	0.239025667	1.331383719	1.376760637
Bifidobacterium_thermophilum	1.925129714	0.107383535	0.803488065	-1.06120907	2.49782486	1.959038899	0	0	9.157116995	1.43840735	1.623311863	1.278573752
Ralstonia_pickettii	1.93497484	-0.298279904	0.372209596	2.014847764	1.405053411	2.547444156	-9.855435379	-1.228065653	-1.012799039	1.577326098	1.406448234	1.368768349
Pseudomonas_mendocina	1.942590383	-0.568371536	-1.704617444	5.486584989	2.281050549	2.048782373	0	0	6.15711649	-1.40797268	0.830619437	0.318447526
Bdellovibrio_sp._NC01	1.945152703	-0.670538132	-1.781281035	NA	NA	NA	0	0	0	0	0	6.544320001
Pseudomonas_oryzihabitans	1.960690298	0.547948098	1.261673097	2.769052271	1.245384064	2.35023214	0	0	0	9.86727874	0	9.917868806
Micromonospora_inositola	1.969288997	-0.365347164	-1.102653177	NA	0.045658717	NA	-10.20011392	-0.773467571	-3.04299945	1.983556424	0.167901268	-8.048034513
Devosia_sp._MC521	1.970927076	-0.877329271	-2.376354591	0.306590772	1.243557031	2.183495641	0.286489526	0.561022602	0.155961487	0	0	8.398029648
Clostridium_scatologenes	1.971169485	0.382059444	1.962992612	0.92174185	0.985484938	0.569543454	-1.119001539	1.353607859	1.753506203	0.061763909	0.367444641	0.427424973
Paenibacillus_cellulosilyticus	1.977929933	-1.67171441	-0.197493261	1.489589335	1.509270391	2.71694445	-0.259573371	0.559163927	0.682242541	0.164408846	-0.176171466	0.137631446
Paenibacillus_terrae	1.979583288	0.17670763	1.89390682	-1.619004074	1.362386826	0.885044926	-0.608517229	1.921436331	1.21379496	1.540943343	0.093415543	0.48981687
Streptomyces_noursei	1.996160855	NA	0.120776147	NA	NA	NA	0	0	0	0	0	0
Schaalia_sp._JY-X169	1.996314953	1.600303047	2.139223179	2.977537203	1.650110502	1.238512087	0.21457817	0.725413528	-0.12261334	0.981742388	-0.901458404	-0.398796858
Pseudomonas_sp._StFLB209	2.004469517	NA	0.340228912	6.528696769	-2.916457186	-1.196932436	0	0	9.929258409	2.774892951	1.592433005	-1.197146985
Herbiconiux_sp._SALV-R1	2.029692103	-1.021268617	0.384951773	NA	NA	NA	-0.840277664	0.840154782	0.276165296	0	10.18652284	0
Corynebacterium_sp._2039	2.038855936	-0.853479603	-1.723212439	NA	NA	NA	-1.509419451	-0.678246414	-1.573549035	8.45395786	8.22753412	9.412216274
Paenibacillus_sp._JDR-2	2.041248783	0.100842488	1.572803684	1.429116162	-0.12696426	0.372695781	-11.1883663	-0.24105812	-0.30004162	-0.40675155	0.722569242	0.305108429
Nitrospira_moscoviensis	2.055204234	-0.600497988	0.550885452	NA	-3.01638186	-0.955352426	0	8.607330314	0	0.529764688	0.192571425	0.171355673
Micromonospora_zamorensis	2.055534677	0.027005879	1.735496989	NA	NA	NA	0	0	8.904155902	-9.247927513	-9.247927513	-9.247927513
Actinoalloteichus_hymeniacidonis	2.059734665	NA	-2.589174752	NA	-1.012600037	1.113062664	-1.498034349	-0.120078439	2.083914145	0.37449156	-0.504695141	-1.010931162
Paenibacillus_sp._FSL_P4-0081	2.069422686	-1.162849512	1.600702071	0.166606476	-1.141566446	0.848659697	-0.38212744	1.208327334	0.703000727	1.215469102	-0.053279384	-0.384157677
Arthrobacter_sp._PAMC25564	2.072488627	-0.213519894	2.066072087	-2.41063232	-1.935294311	0.114458725	-1.289776521	-0.927719472	-2.016290827	-1.271399258	-1.429597036	-0.483127626
Haematobacter_massiliensis	2.081801731	-2.628895846	1.796922236	NA	NA	NA	-0.664240906	1.277643968	0.295355126	-0.626956589	-0.708348137	-7.930737338
Streptococcus_sp._DAT741	2.090720508	0.46557408	2.005504286	NA	NA	NA	0.255549223	0.445081421	1.717403492	1.553125682	0.004289499	1.347463187
Halorhodospira_halophila	2.099473258	-0.887478461	1.695462042	1.516906356	0.792675027	1.918539827	-0.493133184	0.370899694	-0.42868072	0.594725311	1.502912545	-0.834903483
Vibrio_sp._EJY3	2.124247427	NA	-0.976441711	NA	NA	NA	-0.973407266	2.098772677	0.961149699	0	5.472487771	9.306820443
Desulfobacter_hydrogenophilus	2.130812033	-0.881934196	-0.531379124	1.634958468	0.216840802	0.544200056	-1.459106161	0.583647067	0.592977084	-0.122513164	1.047352881	1.395408693
Pantoea_alhagi	2.133071277	NA	-1.634482637	-2.818049523	-0.603730402	-0.47819952	-8.624046047	0.754151253	-8.624046047	0.412885096	0.410158984	1.178597141
Collimonas_pratensis	2.144229387	1.144839277	1.2472177	NA	-1.102646049	2.202229126	0.054480621	0.730036533	1.344098678	-2.615443112	0.649949027	-0.195567543
Sphingomonas_sp._LK11	2.152447205	NA	0.394549284	4.03796785	1.619178216	0.160059648	-0.304405668	-0.652966008	0.195769066	1.195090096	-1.387020487	-8.202941691
Citrobacter_freundii_complex_sp._CFNIH2	2.155109004	-0.853479603	1.423408161	NA	NA	NA	0.608412746	0.922398871	-0.381648047	0	8.996239023	0
Rhodococcus_opacus	2.161958174	NA	NA	NA	NA	NA	0	0	0	0	0	0
Ottowia_oryzae	2.186998515	2.372909468	2.355755597	-0.756442969	2.588826618	1.41814341	-9.076815597	-0.449445872	2.620547312	-0.790781942	-0.923495864	0.457809142
Candidatus_Coxiella_mudrowiae	2.202214539	-1.08246216	-3.921997488	NA	NA	NA	-1.049037152	0.246188699	0.799794264	-8.750428971	-8.750428971	-8.750428971
Paenibacillus_albus	2.216552768	-1.208110077	1.417933897	1.126377286	0.225952232	1.306360316	-9.760342386	0.007540794	0.180304498	1.021733522	-0.457685574	0.177372128
Rathayibacter_toxicus	2.238059363	-1.883186335	0.631987836	-0.537864446	0.808389071	0.629592299	0.480632448	1.284995193	0.276091627	0.350403503	-2.027120138	0.391940955
Brachyspira_hyodysenteriae	2.241623978	1.64189348	1.927360482	0.355239973	-2.359297854	1.28844122	-9.751239421	-0.12387152	-0.133854607	-0.06124413	0.610192629	0.648436753
Pseudomonas_viridiflava	2.242177073	-2.921644362	NA	2.897856784	NA	0.800390974	-11.10685734	-1.680210991	-1.041010411	0.47912045	-0.625367521	0.061575235
Pseudomonas_mediterranea	2.247246015	-0.187107682	1.780601096	NA	NA	NA	0	0	0	-8.044394119	1.155278225	-8.044394119
Chryseobacterium_aureum	2.250908632	-1.312508943	-1.540912057	2.430553836	-0.872972287	-0.957694966	8.409390936	0	0	-1.410658511	1.006017484	0.661811128
Paenibacillus_physcomitrellae	2.257511124	-2.37845849	-0.696162083	0.567565253	1.231287623	0.501958166	0.494926963	0.837058988	0.440818897	0.367295188	0.210146506	-0.302311633
Sphingomonas_sp._HDW15C	2.257720606	-3.226698639	1.189554828	NA	NA	-1.054065157	-0.191404919	-0.498845437	0.470891009	-2.669859063	-10.00375682	-0.177208332
Dickeya_solani	2.262630911	1.557264512	3.355897944	-1.574173705	-0.322730484	0.458884584	0.159142023	0.613752459	0.196771229	-0.074645251	-0.138898015	-0.389783232
Paenibacillus_lautus	2.264087303	-0.179140493	0.459923978	2.288519053	-2.775364808	-1.564771783	-0.468414645	-0.024500298	0.273267948	0.024879969	0.274182418	-0.431376564
Thalassococcus_sp._S3	2.264161071	-0.221791424	0.862947248	2.76523616	1.418839069	3.222528364	-8.624046047	-8.624046047	-0.318604416	-1.034618197	-1.777090267	-1.895867375
Pseudomonas_brassicacearum	2.29031399	0.44161954	2.304050604	1.629077117	0.775959726	NA	0.177620594	-0.386470339	-0.359433715	0.833387204	0.371968777	-8.087462841
Geobacillus_sp._Y4.1MC1	2.291069456	0.498574293	1.115642598	-1.921671639	1.680778288	1.638645659	0.373975695	-0.709564287	0.072964344	-8.048034513	-8.048034513	-8.048034513
Vibrio_mimicus	2.298476456	0.703018262	2.492995864	NA	NA	NA	0	0	0	-0.325297376	-0.376725717	-7.745596618
Xylella_taiwanensis	2.30225061	-1.173482821	1.684498174	NA	1.043455659	1.326255874	-1.391004277	0.933430424	-0.772152532	1.656258787	-3.304494598	-8.776982369
Jeongeupia_sp._USM3	2.304212069	NA	0.554347133	NA	NA	NA	-0.183203376	1.228692515	0.813204244	9.320423752	7.222389201	0
Blautia_sp._YL58	2.316091647	1.74654441	1.299943233	NA	NA	NA	0.073438327	0.870300265	-0.3777838	0	6.815921204	7.380824669
Nocardioides_sp._CF8	2.324342648	-0.238593547	0.463161499	NA	NA	1.91553321	-0.774008505	-3.339696205	-2.277231344	8.868307104	7.369230901	0
Bacteroides_ovatus	2.327882088	2.34342769	1.383036536	-0.456465551	0.229100586	-0.070754988	-0.192469423	-0.446010268	0.006121818	-0.391058341	-0.612866957	-0.479683226
Acinetobacter_ursingii	2.333711469	-1.258461676	1.53117514	NA	NA	NA	0.881318848	-0.569538227	0.850245743	-8.971543554	-1.602312653	0.849699482
Beutenbergia_cavernae	2.339486466	NA	1.02087873	3.938599455	2.237578825	2.721746207	-0.797361418	-1.930441759	-0.733193032	0.612124314	1.461156299	2.40760321
Thermosulfuriphilus_ammonigenes	2.343025249	-2.382440445	2.479356624	2.392652142	NA	0.242449413	1.95275157	1.460994323	2.874390267	-8.048034513	3.36298384	-1.503714512
Providencia_sneebia	2.347951215	NA	-2.432334323	1.691706587	NA	NA	-1.520990912	0.855399515	2.380720978	7.303780748	5.472487771	0
Sphingobacterium_multivorum	2.366690463	-0.83232745	-1.906328032	NA	NA	NA	-0.684966362	-2.083746562	-2.259905175	-10.00936677	-10.00936677	-10.00936677
Bradyrhizobium_sp._CCBAU_53338	2.37559676	-0.334601925	1.081760972	NA	NA	NA	0.307439528	-0.045745204	0.304152895	-7.741466986	1.17142235	-7.741466986
Rhodococcus_sp._S2-17	2.384202188	-0.336464188	NA	NA	NA	NA	-0.322705969	1.633871721	1.345370077	0	0	8.741466986
Massilia_armeniaca	2.390766971	-0.291516782	0.425844254	NA	NA	NA	0	0	0	7.894817763	6.470320477	0
Paenibacillus_stellifer	2.393145844	0.021385837	0.760994358	2.102654421	1.79421485	2.047648007	-0.578839283	-0.678723195	0.384025406	-0.273494453	0.283496727	-0.68338082
Aeromonas_salmonicida	2.399839668	1.783881113	0.904161966	-1.235593878	0.106268145	0.473888567	0	0	9.140944773	0.186072424	-0.039250816	-0.059069353
Halotalea_alkalilenta	2.409781963	-0.094972148	0.793037619	3.533551971	1.853658481	1.943317654	0.780367458	0.846497893	-0.173123984	0.820775438	2.433458696	3.27022851
Streptococcus_halotolerans	2.418483229	-1.572420222	-4.295826552	-0.989010975	-0.415300166	0.356340779	-10.76185038	-1.113084764	-10.76185038	0.768142328	-0.857785018	0.71655855
Gynuella_sunshinyii	2.443726718	1.593457295	-0.678071905	2.781697562	1.500024203	3.052698826	-0.72981396	0.323624673	-0.369540551	-0.638312199	0.398630551	0.387911289
Bacteroides_sp._M10	2.489877548	2.542718632	1.594492599	-0.283664262	0.114237631	-0.204334453	-1.512804671	-0.941511324	-0.567193677	-0.333856681	-0.608922064	-0.508299551
Variovorax_sp._PAMC26660	2.492320366	0.077781121	2.428903385	0.91934042	0.570409588	-0.321544858	-1.440052853	-1.19874358	-0.912406744	1.432960063	0.003929269	-1.251538111
Bacteroides_caecimuris	2.495807795	2.533160529	1.615763405	0.287830855	0.369053049	-0.067847741	-1.588342096	-0.288178471	-0.634068719	-0.274983556	-0.600698303	-0.487434197
Bacillus_ciccensis	2.497841305	1.100121302	2.689448995	-4.793586016	-2.242266359	-0.522421035	0.264838414	1.889913	0.570598062	1.559404736	1.509884521	1.234538609
Bacteroides_xylanisolvens	2.500907134	2.573992796	1.575798623	-0.64823674	0.004473477	-0.299143382	-0.773159934	-0.424775741	-0.447958577	-0.171963403	-0.547784045	-0.49621253
Halomonas_chromatireducens	2.513745702	-1.540933859	-0.840077243	-0.904011089	-0.645360826	-2.16442139	0.38029601	0.370695586	0.320055506	0.654616783	0.552879492	2.321176167
Dehalogenimonas_sp._GP	2.516159094	-0.809973361	-0.397419181	0.096994244	0.216941007	0.845207292	0.034610354	0.54027663	0.937548633	-7.930737338	1.184740747	1.181266819
Paenibacillus_riograndensis	2.53074385	-1.128385226	0.404838004	-0.039801008	-0.509733582	-0.212143355	9.550746785	8.382131157	8.451211112	0.57825219	-0.042542586	0.064505317
Vibrio_natriegens	2.541965095	-1.767890167	2.593176321	-2.570966371	NA	-0.732531077	0.322242642	-1.570652606	1.190549572	10.72280753	0	6.930737338
Xanthomonas_translucens	2.551810215	-1.55720945	-0.69838422	-0.095615572	-2.093504826	-2.592494103	0	0	0	-0.677439986	-1.153435608	-1.089573931
Terribacillus_goriensis	2.591626231	-0.435814069	-1.080489918	-2.598316128	-0.195715732	0.132755209	1.479566037	1.296854106	0.477275526	-1.551985178	-0.68923121	-1.466926433
Bacteroides_caccae	2.606269137	2.652528752	1.726854792	-1.483617254	0.329413318	0.209122598	-0.884467476	0.024621653	-1.329214647	-0.31814512	-0.591973766	-0.472947145
Sphingorhabdus_lacus	2.608967964	2.406540323	3.341641948	NA	NA	4.191378111	0.787938836	0.86168592	-0.300517941	0	0	9.626926402
Thermanaeromonas_toyohensis	2.612435748	-1.919749192	-1.123637617	1.816395901	1.685080493	1.675448558	-0.300168201	0.367681229	0.377655606	-0.514505021	0.030949184	1.936850939
Chryseobacterium_sp._NBC_122	2.616598422	2.773996325	2.199005735	1.016998659	-0.453182096	0.207775788	0.241943782	-0.210584785	0.206734279	0.538436463	0.026498107	1.879910764
Parageobacillus_toebii	2.620064643	0.391035509	1.679106466	NA	NA	NA	-0.775804343	0.11814619	-0.350160895	0	0	10.71424552
Candidatus_Nanopelagicus_abundans	2.621799269	NA	1.214553605	NA	NA	-1.98767325	0	0	0	0.427041376	-0.04717477	-2.374044643
Enterococcus_sp._CR-Ec1	2.622041128	0.8142585	1.079885709	NA	NA	NA	-0.985465357	-0.301529337	-9.488205685	-1.988291068	-10.44224893	-10.44224893
Paenibacillus_lentus	2.622642768	-1.748362329	0.204182474	0.365011341	0.862353727	0.853883161	0.41629122	0.05750477	1.416960409	0.093208511	-0.06718338	0.277939618
Bacteroides_sp._HF-5141	2.631154046	2.680103459	1.762397993	0.079922452	0.475205904	0.150015435	-0.691571875	-0.093778908	0.047561087	-0.308617413	-0.61446344	-0.498995232

Dialister_pneumosintes	2.638349713	0.072149786	2.05158245	2.686573099	0.230910203	2.23409145	0.686335968	1.976200318	1.658631376	0.112761628	-0.541871127	-1.229796251
Pseudomonas_sp._NC02	2.641330315	NA	2.923174134	NA	NA	-0.210579855	0	0	0	0.828462401	0.461157513	0.764675808
Nitrospira_sp._KM1	2.642062908	-0.162534041	1.297318331	NA	0.100565996	0.77835328	-0.873574488	1.071255053	2.970424879	1.159231466	1.630821529	-8.818048457
Flavobacterium_psychrophilum	2.651363726	2.83302421	1.628661044	-0.13637633	0.067626666	1.191982441	1.14217346	1.259439383	0.548365903	-0.620311787	-0.458990285	-1.270983436
Rodentibacter_pneumotropicus	2.706756745	1.976818778	0.3238633	11.78754432	1.769556379	1.804907736	-0.509014274	0.270010342	1.729942094	1.062734948	1.927956319	2.127656447
Enterococcus_faecalis	2.708741896	-1.728559069	0.535520829	0.080638912	-0.81345049	-0.659458308	-2.343528785	-0.145054694	1.782640968	0.407718221	0.319432578	-0.139132358
Nitrincola_sp._KXZD1103	2.723113806	-0.975452392	-3.604449	NA	NA	NA	9.161703808	9.3781973	0	7.303780748	0	0
Serinicoccus_hydrothermalis	2.725876621	-0.216518861	0.168149377	NA	NA	NA	-1.375213331	0.743555459	-2.013526138	8.878562894	5.472487771	0
Streptosporangium_sp._'caverna'	2.750218001	2.249455522	4.007544704	NA	NA	NA	-0.708027598	0.678223053	1.429934892	8.442943496	9.869953123	8.996239023
Mycoplasmopsis_gallinacea	2.756074417	0.451548221	-1.476028129	-2.768499753	-3.337479639	-4.225504006	-0.919837793	0.16111001	-0.716989069	0.040845042	-0.040360079	-0.458565681
Streptomyces_sp._fd1-xmd	2.756600085	1.143418171	1.481063911	NA	NA	NA	0	0	0	0	0	0
Blattabacterium_sp._(Blattella_germanica)	2.78783597	2.584871659	1.113673298	-1.492947409	1.036619636	1.692790238	-0.333741514	-1.156169847	-1.006310458	-0.219960375	-0.793452564	0.318083147
Candidatus_Hamiltonella_defensa	2.830696104	0.315096061	-2.26895363	-4.771466055	NA	NA	-4.733518548	-1.689899584	-0.898731699	-2.199389963	-0.932801993	-1.39357904
Dialister_hominis	2.830825132	-0.716970964	0.705639351	-1.935014197	-0.082134536	-0.266827158	-0.501888084	0.766375655	0.331268778	-0.586827615	0.197270717	-1.661472795
Bacteroides_sp._CBA7301	2.847730375	2.84170788	1.863700328	-1.762469411	0.444796796	0.314313022	-0.190712983	0.348710634	0.44924526	-0.40895635	-0.752216591	-0.588070316
Labrenzia_sp._THAF82	2.891085729	3.291260692	2.971617067	1.257937539	0.897392181	1.908156356	8.166278594	0	0	-0.553359794	-0.554919782	0.585673288
Streptococcus_marmotae	2.895529272	-1.678145872	-1.222269151	2.889378195	0.295683402	2.099757505	-9.760342386	-3.077670777	2.5367831	-0.509465097	0.123747595	0.025384529
Bacillus_sp._FJAT-42376	2.923432986	-1.370293305	0.717345229	1.307692728	-0.280053287	-0.317308046	0	0	0	0.044240994	0.184365137	-0.04787715
Fusobacterium_periodonticum	2.964466861	-0.611838207	1.933116322	3.633665692	0.680778288	0.228570996	-2.24660059	-2.108716522	-0.459155202	0	0	0
Neisseria_sp._oral_taxon_014	3.038855936	1.535194919	-0.025090981	NA	NA	NA	-1.640457613	0.372983627	-0.204358499	10.16741815	9.79982071	6.930737338
Pusillimonas_sp._T7-7	3.038855936	1.75033544	-0.728697978	3.61076197	3.256769831	3.177440834	-8.04736934	-8.04736934	-8.04736934	-0.78889384	0.546688759	-0.003620288
Arcobacter_cibarius	3.070058093	0.978982219	3.404754526	-1.401046189	-2.311295797	0.024188259	0.344157542	0.395314164	0.942074587	-0.076915151	1.286010923	1.237717129
Paenibacillus_sp._CAA11	3.07527405	-2.097553845	-0.000237324	0.667723696	-0.808701348	0.111476911	-0.667424661	-9.076815597	0.24818397	-0.208364472	0.014434303	-0.10797064
Hydrogenovibrio_thermophilus	3.075438135	NA	NA	NA	NA	NA	-1.65106616	-1.560645102	0.083822177	0	0	0
Stenotrophomonas_sp._SAU14A_NAIMI4_5	3.157781818	1.018576324	0.190550674	NA	NA	NA	-1.564619506	-1.726277692	-0.738160693	-9.049848549	-9.049848549	-9.049848549
Francisella_sp._Scap27	3.161463423	NA	-0.160464672	-5.244212454	NA	-1.273609754	-10.94331334	-10.94331334	-3.786198869	0.266900571	0.276953686	0.6304367
Chromobacterium_paludis	3.190222416	0.601010442	0.36579398	NA	NA	NA	-0.279021568	0.673114805	1.239952998	0	7.222389201	10.4101015
Viridibacillus_sp._JNUCC-6	3.339185258	0.57053543	2.244523591	-2.344828497	-1.823219334	-0.429555485	-1.114514951	-1.070043807	2.03858618	-0.555161723	-0.037130231	-0.628824197
Sphingopyxis_macrogoltabida	3.340524899	-0.605721061	3.541553851	1.423078096	0.768400815	0.812962855	0	0	7.157114468	-9.046215465	-9.046215465	-9.046215465
Achromobacter_insolitus	3.357031896	NA	0.842458723	3.123871658	NA	-0.438442104	-0.375695098	0.022094054	-0.191039519	-1.04258569	-2.081251627	-1.742279477
Corynebacterium_simulans	3.391922543	1.229481846	1.589653299	0.058245141	1.601082166	3.989492836	-0.046443067	-0.217373905	0.806836649	0.767506559	0.57179974	0.391554488
Erythrobacter_litoralis	3.420170016	0.749450566	0.180470364	NA	1.184443467	1.089517686	0.315755929	1.609221124	2.489412374	1.386740343	1.769562892	1.091132009
Enterococcus_casseliflavus	3.460995092	0.302216018	0.417469017	7.25298082	-0.098282353	0.124978817	0.154628153	0.546599676	-0.015990049	0.600901853	-0.199094109	-0.098709861
Monaibacterium_sp._ALG8	3.500353981	0.041171308	1.027089807	1.998873862	1.693356436	1.743435089	-2.060914055	-0.610314774	0.083013623	0.921816963	1.538195866	-0.417407356
Oceanisphaera_avium	3.531768561	NA	NA	-2.891238648	-3.867896464	NA	1.754401179	0.305437982	1.605310607	1.151955258	1.08152363	1.717994529
Pseudomonas_sp._09C_129	3.573068822	-3.160715525	NA	NA	NA	NA	0.104394539	0.297394306	0.643294085	0	7.902875524	7.539158811
Streptomyces_sp._CMB-StM0423	3.582539838	NA	1.258589746	NA	NA	NA	-9.638930002	-9.638930002	1.499981716	0	0	0
Empedobacter_stercoris	3.610526786	3.761098735	2.798054104	-1.255930536	0.738192898	-0.872015554	-1.860650608	-0.976611738	-0.436840553	-1.779255287	-0.376827836	0.972246401
Bdellovibrio_exovorus	3.630435674	-2.263034406	0.084316286	NA	-3.114990396	-3.578646601	-0.437399174	-1.711534953	0.213073364	-1.334212276	1.17693645	-3.115477217
Kosakonia_radicincitans	3.640754452	1.830680029	2.566283948	NA	NA	-1.32394444	0.431517558	-0.378112711	0.05176662	-0.83711505	-0.806381845	-2.786842549
Rhizobium_oryzihabitans	3.837827274	NA	0.665580961	0.961122631	0.442478027	-0.164607284	0	0	7.157114468	0	9.040015679	9.306820443
Paenibacillus_lutimineralis	3.883135153	-1.770794174	0.492853124	-0.313809624	0.326563938	0.356689061	0	0	0	0.186609776	0.209657243	0.099704636
Enterobacter_hormaechei	4.009120378	8.643075883	5.002766204	0.195664322	2.411759505	2.802741984	-0.747711453	0.544689659	-0.897777802	-8.745953257	-8.745953257	0.154913551
Rathayibacter_tanaceti	4.203644077	2.263034406	3.689396974	NA	NA	NA	0.540570528	-9.714245518	-9.714245518	0	0	0
Staphylococcus_sp._AntiMn-1	4.335180502	6.715895213	NA	NA	NA	NA	0.113222716	-0.473398401	-0.100288288	-3.074293898	1.15728016	1.831270433
Pragia_sp._CF-458	4.340464046	1.0489096	NA	NA	NA	NA	0.611708368	0.177177032	0.586667043	8.759332297	9.214319121	0
Candidatus_Planktophila_dulcis	4.457923312	1.229481846	-1.723212439	NA	NA	NA	0.442825885	0.43104713	0.19183333	0	0	0
Oceanimonas_sp._GK1	4.50779464	2.360218949	2.809721937	2.227400335	-0.015692283	1.069506505	-2.062129062	0.729596354	-0.798864458	0.378308948	0.124652417	0.684821163
Akkermansia_muciniphila	4.577059087	2.276669013	0.30854399	5.065779713	5.983761545	5.192369168	0.394054555	0.245637473	0.650105962	-1.305756303	-1.247010279	-0.897599158
Acinetobacter_sp._NEB149	4.72134163	-2.63493096	NA	NA	NA	NA	1.029904991	1.066324759	1.474648297	0	0	0
Enterococcus_sp._FDAARGOS_553	7.759592221	5.417532717	5.410825248	NA	NA	NA	0	0	0	0.696986878	-7.745956618	-7.745956618
Enterococcus_gallinarum	10.28696525	8.262493535	7.893730541	11.02495462	-1.190688881	-0.035045944	0	8.382131157	6.15711649	-0.051482981	-0.542110842	-1.014858775
Enterococcus_sp._M190262	11.15198782	9.220118701	8.726976954	13.0730321	1.401024303	1.579099133	-0.458203795	1.164461348	1.449140325	-0.578540748	-0.627240173	-0.711262528
Lactobacillus_sp._wkB8	NA	-8.982277463	NA	-0.687529153	-1.332269618	-1.170525249	0.00922713	0.159441923	0.750073314	-1.301074967	-0.775417355	-0.299568266
Seonamhaeicola_sp._S2-3	NA	-8.633971402	-3.415186821	-5.928072891	-7.626016596	-2.782783622	-0.745710329	0.792818101	0.085811724	-0.459793609	0.322286817	-0.659490996
Candidatus_Protochlamydia_naegleriophila	NA	-8.250997456	NA	NA	NA	-3.599884867	1.891448949	2.21630896	3.280106743	-0.479094973	-0.564850888	-0.253430878
Lactobacillus_heilongjiangensis	NA	-8.012064557	-6.739265234	-0.327151577	-0.226622953	-1.034443992	-9.222246695	-9.222246695	-0.916805065	-0.486881479	-0.664817594	-0.692386062
Microbulbifer_sp._A4B17	NA	-7.090637089	-0.540314065	-3.614077887	-0.545311139	0.056038851	-2.130930078	-11.33772224	-5.18060575	1.236031069	2.033361454	1.310842533
Hydrogenophaga_sp._PBC	NA	-7.04466648	-2.587006461	-2.7473572	-1.861869081	2.079012608	0.609519764	2.047259568	0.915572493	-0.777153682	-0.777972232	-0.018743834
Mycoplasma_wenyonii	NA	-6.790574563	NA	NA	NA	NA	-7.757316664	-7.757316664	1.106587725	-0.994008529	-0.104821661	1.285746827
Streptomyces_sp._3214.6	NA	-6.607599183	2.694957893	3.749611747	4.490678845	3.923167813	-0.231232656	0.294702624	1.054535705	0.355544237	0.960848507	0.142693837
Bosea_sp._ANAM02	NA	-6.136248772	1.53718099	2.066495412	2.981837672	2.46156036	9.188925064	8.382131157	11.79175304	0.483098706	0.82555203	-0.727334925
Vibrio_tubiashii	NA	-6.094362579	-2.176918205	-0.592307351	-1.660149997	0.03562391	-1.304854582	-9.714245518	-3.557129028	9.370687407	6.815921204	10.05165759
Flavobacterium_crocinum	NA	-6.090170279	-4.761901104	1.285468157	0.755363655	1.443209504	0.69950533	0.035948995	0.434375066	0.922344747	1.206396291	1.920472837
Lysobacter_sp._H21R4	NA	-6.079402548	-2.729154557	NA	1.204509488	1.052857116	-0.862379238	-0.212024005	-1.373593028	0.503684914	-0.076492364	0.598888928
Synechococcus_sp._SynAce01	NA	-5.88447313	-0.844129089	-0.933025858	-0.825375523	-1.093058055	0.371966353	1.622849987	-0.600202196	-0.266465311	-0.610896277	-0.155433999
Arachidicoccus_ginsenosidivorans	NA	-5.838009906	-4.628384781	-1.263697105	1.803682584	1.196547784	-0.179393147	-0.087222602	-1.138473342	1.034571619	0.749131264	1.680931559
Galbibacter_sp._BG1	NA	-5.815051729	-1.899711943	1.729038678	0.827911805	0.450420377	0	0	0	0.922956104	-1.760046146	1.192645078
Paraburkholderia_sp._SOS3	NA	-5.781223514	2.071802585	3.834436766	5.155466949	4.74659051	0.033360702	0.107126367	0.746179648	-0.226779634	-0.442058665	-1.168670718
Sphingobium_sp._RAC03	NA	-5.780193428	-0.659408361	0.304763796	-3.018708527	-0.835327245	-0.423856683	-0.395619312	0.788805683	0.778863343	-0.193821477	-3.639383154
Spiroplasma_diminutum	NA	-5.432017547	NA	NA	NA	NA	-2.552596645	-2.571453774	-1.204673894	-0.288227458	-0.595569468	-8.967707557
Winogradskyella_helgolandensis	NA	-5.402766372	-1.740747854	-3.389391063	-1.520513793	-0.864495131	1.196834429	2.555641321	2.080019748	0.842993297	0.424477477	-1.437115862
Spiroplasma_apis	NA	-5.331429963	-1.753901314	NA	-2.417747984	NA	0.604319583	1.56661734	2.298736873	0.43318074	1.055350596	1.080108954
Legionella_endosymbiont_of_Polyplax_serrata	NA	-5.331343522	-9.910820483	NA	-8.929761595	NA	1.891448949	0.850013649	3.048427208	-0.142149252	-0.066288361	-0.76668725
Azoarcus_sp._CIB	NA	-5.323516092	-0.296072829	2.19727996	1.650127547	0.880947328	-0.054885168	-0.238722751	0.385688382	-1.914029758	-1.650001604	-2.703607512
Tenacibaculum_jejuense	NA	-5.30031728	-5.085206615	-4.652375703	-0.642925491	-0.628579714	-0.058553831	1.180533699	0.981586543	1.069839675	0.757696804	0.848227747
Mycolicibacterium_fortuitum	NA	-5.297087967	-1.714245518	NA	NA	-3.086093411	9.822499133	0	11.86917354	1.53297809	0.327487479	0.542568107
Lysobacter_sp._H21R20	NA	-5.181312342	-0.914640752	-1.046214555	0.696375588	3.106704021	-1.847301234	0.992660157	-0.120658781	-0.336129012	-0.26730688	0.162424143
Halarcobacter_ebronensis	NA	-5.083922484	-3.247100653	NA	NA	NA	-0.118876165	-0.138823823	0.507955698	-0.449500826	0.917874132	0.707575687
Sphingobium_japonicum	NA	-5.051780963	-1.620574527	NA	NA	NA	-0.305883107	0.109659527	0.803414571	-0.173556086	1.108764485	-0.07210008
Lactobacillus_backii	NA	-5.041309377	-0.84116153	-0.534014989	1.408321166	0.600032976	-9.177646265	-9.177646265	-1.432509796	-0.990813677	-1.986925748	-0.034732919
Methylococcus_sp._IM1	NA	-4.984893108	-2.80032133	NA	1.165250042	1.407317152	-1.122156018	0.116035758	0.374992748	-0.118011522	-0.670362828	0.607891795
Pelagibaca_abyssi	NA	-4.977179039	NA	NA	1.281969595	0.859678712	1.449250174	0.21051259	0.041953594	-1.832981028	-10.28693889	-1.63828291
Micromonospora_carbonacea	NA	-4.897325732	0.895256759	NA	NA	NA	-1.039372331	-0.431226143	-0.735097557	-1.808194426	-2.088049686	0.491781352
Desulfurella_acetivorans	NA	-4.893866588	-3.594748376	NA	NA	-3.541419231	-0.60393491	0.009876014	1.528536863	0.306655491	0.579724715	0.060868535
Sphingopyxis_sp._LPB0140	NA	-4.878003634	-5.549734012	NA	-2.306661338	NA	-1.568122812	0.58341818	-1.110355359	0.379752335	0.460382489	0.821538229
Vibrio_harveyi	NA	-4.869518245	-0.197647679	NA	-2.500188426	-0.761776728	-8.04736934	0.559960973	-0.302232871	-0.148976682	1.439897775	1.014125781
Candidatus_Pelagibacter_sp._IMCC9063	NA	-4.863292136	-1.148975281	-2.135605832	-0.492540377	1.322827313	-1.898125413	-1.916982542	1.249245254	0.467062774	-1.222314568	0.053820792
Martelella_sp._AD-3	NA	-4.823604637	-5.314195451	NA	-5.875454902	NA	-2.30563583	-10.71502677	0.193230737	-0.261695701	-0.377536034	0.121655033
Candidatus_Methylospira_mobilis	NA	-4.793013521	-0.137324968	1.818877766	-1.462809858	-1.34765066	-1.868263351	-2.865301015	-0.515850093	0.274111941	0.809964892	0.770076526
Campylobacter_lanienae	NA	-4.776150378	0.609493507	NA	-2.642092144	-1.225219388	-1.296597991	-10.17311494	-0.237815793	-0.177443631	-1.322392498	-10.0018726
Zymobacter_palmae	NA	-4.766712938	-6.42567602	-0.158977243	1.217414329	0.524469147	-0.341839985	-0.744658415	-0.763230478	-0.024668545	0.887974162	0.463748491
Dickeya_chrysanthemi	NA	-4.667807116	-4.452696452	NA	NA	1.69614669	0.684810015	0.050567902	-0.249756474	-0.32644975	-0.778031094	-1.655598043
Marinomonas_mediterranea	NA	-4.652726543	-0.141931883	NA	NA	NA	0	0	0	-1.555260799	-3.211576964	-2.619678315
Ancylobacter_pratisalsi	NA	-4.610423258	1.93911972	NA	NA	NA	0.227651022	0.60089331	-0.08480811	-8.044394119	1.273922722	2.415037499
Streptomyces_ambofaciens	NA	-4.50380823	NA	NA	NA	0.185662405	0.023610177	0.896374221	2.856825574	8.366322214	7.59792591	7.63420844
Pseudomonas_sp._S34	NA	-4.485346568	3.323799642	3.991675445	4.916143235	4.247476477	-0.723858257	0.260174597	0.038722398	0.718885079	0.91303601	-1.283229384
Yangia_sp._CCB-MM3	NA	-4.477660212	1.055266349	NA	0.686779837	-1.50113091	10.82900121	6.682671608	11.1883663	0.567884521	0.362966058	0.367813185
Achromobacter_denitrificans	NA	-4.430206547	0.712551241	-0.800223128	-0.452512205	1.189707614	-0.148659834	0.242501772	-0.1007176	1.188323613	1.31833806	2.007886911
Tetragenococcus_osmophilus	NA	-4.425254037	NA	NA	NA	1.502399906	-8.076815597	-8.076815597	0.080303925	0.736026161	0.011089623	0.941373567
Olivibacter_sp._LS-1	NA	-4.385735542	-0.639254153	NA	NA	NA	9.690633595	9.426646347	10.42378824	-0.450961761	-0.534868333	0.445255261
Permianibacter_aggregans	NA	-4.290884915	-5.047458794	NA	NA	0.738525265	-0.852775894	-0.805476587	-0.318273638	0.644106296	1.619054045	1.844731968
Starkeya_novella	NA	-4.207540738	-0.537891412	3.720569488	0.523054259	-1.015089336	-3.867959986	-1.623986314	1.072572594	-1.42326555	-0.038863731	-1.096435261
Sphingosinicella_sp._BN140058	NA	-4.188661564	0.375375815	NA	NA	-1.074898394	-10.17311494	-10.17311494	-10.17311494	0.261662437	0.161880159	1.225488825
Streptomyces_cattleya	NA	-4.181880135	-1.170800689	0.008067281	1.28643901	0.671314539	-1.057659335	-1.160701813	-1.006744057	1.706994203	1.837220818	-1.197146985
Erwinia_billingiae	NA	-4.158937712	1.612566301	NA	NA	NA	0	10.40552443	9.624047875	2.171827303	1.607683282	2.928480306
Marivivens_sp._JLT3646	NA	-4.092288809	-6.563179543	NA	NA	NA	-2.051503157	-0.501919979	-0.771394302	1.108626116	1.388935259	-0.18033844
Acinetobacter_haemolyticus	NA	-4.08649314	-1.884794144	2.851599705	1.065937757	1.609441121	0	0	0	-1.134689743	-0.458551723	0.687790084
Corynebacterium_striatum	NA	-4.071146847	-1.144589218	2.30860961	0.916734845	2.075486377	-0.293533263	0.289693346	0.052617836	1.777881428	4.438631879	2.472884324
Variovorax_sp._38R	NA	-4.067132251	0.772543264	NA	NA	-1.527603121	-1.950979896	-3.917066879	-1.35031202	-0.589466668	1.42777971	0.582621368
Hydrogenobacter_thermophilus	NA	-4.038404169	-4.228289164	-2.369727067	-1.359567201	NA	-9.222246695	-9.222246695	-0.067428586	2.135402777	-8.186525318	3.676371577

Bacillus_luti	NA	-4.032126779	-1.61238962	NA	1.209631399	1.158903595	-1.728503429	-0.580930709	0.335892247	0.994836909	0.787885058	1.016029736
Minicystis_rosea	NA	-4.029107774	-0.799561352	0.419424499	-0.275452761	-1.488757309	2.473493504	-9.076815597	0.374395515	-0.345010147	-1.470804785	-0.246577143
Schaalia_odontolytica	NA	-3.995498608	0.681238412	0.564810242	-0.491625703	2.418671494	-0.018693221	0.54771287	-2.072792797	0.477829679	0.102361718	0.343265678
Winogradskyella_sp._PC-19	NA	-3.963669923	-5.95542049	NA	-2.460694863	-2.171890526	0	0	0	-0.218570976	-0.472487275	-0.258606178
Sphingobacterium_psychroaquaticum	NA	-3.956439624	-1.301013043	0.255168561	-0.470524587	-0.680119734	0	8.627369725	7.745136469	1.456033593	1.633655331	0.792571872
Mesorhizobium_sp._M7A.F.Ce.TU.012.03.2.1	NA	-3.948650242	-2.399616216	1.204970431	-0.964584316	0.585531499	-9.760342386	0.274580349	-2.603227918	0.531549953	2.51513093	1.969339447
Maribacter_sp._HTCC2170	NA	-3.923531402	-0.114157312	NA	-1.853241867	1.85862924	-0.257171706	-1.44306972	-0.254066137	0.914251772	-0.398799108	1.679764222
Shewanella_sp._SNU_WT4	NA	-3.917444374	0.285294366	NA	0.686760852	1.158558073	-2.711703032	-0.681025121	0.724993377	0.342279602	0.996053804	2.288431183
Streptococcus_ferus	NA	-3.910093028	-0.075685186	-0.270657168	-0.348417067	0.39956853	-0.693832542	-0.08114921	0.064339955	0.160975474	-1.167658833	-1.047966805
Xylophilus_rhododendri	NA	-3.906890596	0.269727424	-0.59873535	-0.778097209	0.536171062	2.191250354	3.088339946	1.76349466	-0.394018866	0.503643613	0.073288099
Mesorhizobium_loti	NA	-3.894009304	0.45923556	1.106441698	0.692088102	-0.640132748	-1.212642582	-0.717092079	-1.292479748	-2.212996704	1.174240729	0.657676684
Xanthomonas_sacchari	NA	-3.892914785	-0.36597451	-2.522219602	-3.129534847	-0.71927997	8.129283017	0	9.819597937	-0.581257991	-1.016015357	-1.926218686
Brevundimonas_naejangsanensis	NA	-3.877301605	-0.942293451	0.004592137	0.399468974	2.435172261	-9.340255147	0.548765048	1.529269297	0.370078785	-0.030652451	0.852913382
Corynebacterium_doosanense	NA	-3.840784854	-4.62567419	0.195130341	2.761627648	0.483055923	0.944015362	1.704875959	-0.473489778	-1.52770799	0.388511948	1.886103818
Streptomyces_actuosus	NA	-3.821809095	-0.929824706	NA	NA	NA	0.974676611	-1.701693544	1.201201154	-0.454363307	-0.342105281	-1.508000519
Glaciecola_nitratireducens	NA	-3.816178772	-2.162091911	-2.71539006	1.697356188	3.076790173	1.765674062	0.997586965	-2.926966383	0.278310541	0.748887391	0.428071381
Limnobacter_sp._SAORIC-580	NA	-3.785398455	-1.251099858	2.980032047	2.353937822	2.665372631	10.45681641	10.38409299	10.67547194	-0.410511799	0.718435311	0.528491543
Diaphorobacter_aerolatus	NA	-3.783091987	0.297250121	-0.619629633	1.563308135	1.14917942	-0.072405598	-0.033466897	-0.290059782	-10.31288296	-1.116484923	-1.664226977
Lactobacillus_futsaii	NA	-3.759962627	-3.263732436	-2.240419371	-1.916507281	-1.385179406	-0.161982515	-0.114244936	0.777953257	-1.044357226	-0.744264402	-0.673827988
Bordetella_genomosp._8	NA	-3.755122871	0.88363995	NA	1.723047092	NA	-0.301135024	-0.812228526	-0.991232004	-1.141983214	1.193467553	0.510652417
Haemophilus_parahaemolyticus	NA	-3.755122871	1.494309104	NA	NA	NA	0	0	0	0	0	6.930737338
Campylobacter_fetus	NA	-3.744759104	-1.932831452	-4.63625223	-4.867388285	-0.975583041	-1.41624801	-0.655756898	0.032082784	0.275294023	0.57428403	0.211653281
Sphingobium_amiense	NA	-3.73622456	-0.16852126	NA	NA	3.025959227	0.188026988	1.844919133	2.458127686	-1.658328354	-0.943972755	0.599139552
Hymenobacter_qilianensis	NA	-3.734271485	-1.921798803	0.592438246	1.170811252	0.179804037	0.942966637	1.421137196	1.62384551	-0.237150523	-0.218825806	0.524065804
Pantoea_rwandensis	NA	-3.717736916	3.678907874	4.619180895	5.701230684	5.008077079	0.313062823	-0.249436344	-0.833794662	-0.65108341	0.294639937	0.378912465
Pragia_fontium	NA	-3.716966729	-0.908704166	NA	NA	NA	-0.240765382	0.075638956	0.320885201	-1.904270592	0.720379342	-9.324929833
Streptomyces_sp._WAC_01438	NA	-3.716966729	0.908983442	2.823623809	-1.303246674	1.742935963	-0.068597277	1.800158255	1.860367769	10.52094273	9.535275377	10.45943162
Brachybacterium_faecium	NA	-3.699623483	-4.95419631	NA	NA	NA	0.207632997	0.03917357	0.176218088	-9.055282436	1.620083642	2.066684861
Winogradskyella_forsetii	NA	-3.694242237	-1.49836573	-3.982371366	-1.957987207	-0.888269681	1.847997914	-9.635636507	1.677813434	-0.241122959	-0.107519829	-0.169076687
Variovorax_sp._PAMC28562	NA	-3.651327439	-0.078777722	NA	NA	NA	-0.619992877	-1.052909109	-1.154875961	1.449310242	-0.913042479	-1.276759192
Mycoplasma_hyopneumoniae	NA	-3.62406232	1.539241765	NA	1.277307475	-0.18200735	0	0	9.819597937	1.611040157	0.8576932	-0.007623525
Halobacteriovorax_sp._BALOs_7	NA	-3.606049771	-1.250528423	NA	NA	-0.295777573	0	6.682671608	0	0.151218953	1.538272708	1.116079683
Corynebacterium_terpenotabidum	NA	-3.593053088	-1.150618354	1.06026703	-0.553813802	-0.764580898	-0.048371517	-0.078397486	-0.023683027	-0.842601743	-1.736964125	-3.48671878
Arcobacter_cloacae	NA	-3.548859676	-4.388395004	NA	-3.670420665	-4.671543822	0.651524697	0.617431499	1.057903008	0.055135199	0.749447113	0.232890603
Streptomyces_sp._S4.7	NA	-3.530855095	0.849973769	NA	2.253102288	1.586892528	0	0	0	1.279643717	0.534801438	-0.008395921
Streptomyces_lydicus	NA	-3.524589883	-1.151546472	2.610536117	-1.48707468	1.44074594	0.202022042	0.028228285	-0.106930639	1.537686795	2.305177199	2.329561357
Candidatus_Midichloria_mitochondrii	NA	-3.509400078	-1.596167955	NA	NA	NA	1.160187276	1.034514306	4.173509037	-1.460719092	-1.276548872	-1.02167955
Thermodesulfovibrio_yellowstonii	NA	-3.49418464	-3.839044956	NA	-4.802767653	-1.543820731	0.138226107	0.148230214	0.93746525	0.533322508	1.462576578	0.811641838
Protaetiibacter_sp._SSC-01	NA	-3.481221545	2.237556478	1.07979377	1.961567688	2.029115386	0	0	0	-10.26893889	-0.486038988	0.525775918
Helicobacter_sp._MIT_01-6242	NA	-3.43021664	-3.316447765	-4.498658274	-2.026262685	NA	-0.537862967	-0.081402633	0.256823392	-1.037780764	-0.313545183	0.189967668
Arcobacter_cryaerophilus	NA	-3.416485288	-2.454139884	NA	-2.873483719	-1.442720968	0.6484147	0.990122565	0.790666051	-0.277185506	0.011012463	-0.114629606
Halorhodospira_halochloris	NA	-3.405657641	-0.341617282	NA	NA	-3.070523501	-0.28161207	-0.171229821	-0.258086038	-0.226649038	0.112173315	0.111113403
Tistrella_mobilis	NA	-3.38848364	-0.476444342	1.721188425	-1.610289459	-0.865134467	-1.303241481	-1.705785625	1.028190831	-0.244926974	-0.241711955	-0.329565974
Lysinimonas_sp._KACC_19322	NA	-3.383896255	-1.538127449	-2.60948003	-1.331178867	NA	-1.053570978	0.816564789	0.952381667	-1.39246548	0.312014708	-0.439562915
Mycoplasma_bovis	NA	-3.366375855	0.218881645	-5.547670311	-1.297273774	-0.411695464	10.00332114	0	0	0.144803411	0.483793005	1.526166862
Kineobactrum_sp._M2	NA	-3.346470279	0.587046819	2.618594504	3.847908292	3.101143089	0.02810768	-0.557436655	0.521300766	0.078172104	0.128721775	-1.937795626
Synechococcus_sp._RSCCF101	NA	-3.341147601	-0.691414845	-0.151944825	0.517873277	1.136502	-1.104076307	-1.305936672	0.569318361	-1.245314201	0.081553456	0.94102469
Nitrosomonas_sp._AL212	NA	-3.339660975	-1.814819467	NA	-0.021423579	1.539903688	-1.504658575	0.521522311	1.35617164	0.015935702	0.979047146	0.093225686
Salinisphaera_sp._LB1	NA	-3.322635125	-3.12870881	1.787058934	0.083034136	0.93092302	-0.424362229	0.171690362	0.115776655	-1.060235814	0.287708212	0.652683267
Sphingomonas_sp._LM7	NA	-3.308389041	-1.626359123	-2.581292453	-0.631643648	1.179447776	-0.396601447	-0.083586145	0.017351931	0.217070689	0.114271553	0.056742512
Campylobacter_hepaticus	NA	-3.299309792	2.22981705	NA	-1.627370199	-0.793710752	-3.161154596	0.007021012	-0.632659957	-0.347076968	0.492842619	1.408184597
Nocardioides_euryhalodurans	NA	-3.292998224	-0.278485394	2.93681001	1.710921775	2.109373666	0.478764661	1.362762055	0.214850265	1.695208272	0.794165395	2.515724422
Streptococcus_vestibularis	NA	-3.285517455	NA	2.110945312	NA	NA	-0.335182736	0.3760146	-0.917251171	2.034938104	-1.617142364	-8.087462841
Isosphaera_pallida	NA	-3.25804717	-0.781816584	0.524048121	-2.178337241	-2.641993446	-0.353863267	-0.095116585	1.028935168	-0.995041501	-0.976361906	0.472463418
Corynebacterium_frankenforstense	NA	-3.252842776	-1.715539583	3.730149933	0.325793034	0.767177979	-12.39922985	1.257988097	-0.598439128	9.271463808	10.91787179	10.306825
Campylobacter_volucris	NA	-3.251273541	-0.915935735	NA	-1.296684795	0.119868014	-0.453326516	-0.532115767	0.309938927	-0.608052711	-0.712763498	-0.55841592
Leucobacter_denitrificans	NA	-3.24961389	-3.401616984	NA	NA	1.800290053	0	0	10.2067946	9.707359132	9.212698215	9.940558798
Halomonas_sp._HG01	NA	-3.238198745	-1.766853805	-0.875367154	0.950223339	-1.515727893	-0.668399543	-0.480154878	-0.644550132	-1.614710891	-0.060119956	1.671328944
Mycoplasma_meleagridis	NA	-3.201011995	-1.970922767	NA	NA	NA	0.384061849	0.585056791	1.160464672	-3.049366077	-2.125612705	-10.35314683
Alicycliphilus_denitrificans	NA	-3.175415014	-5.014950341	NA	-1.517315173	-1.16294838	-0.872313094	1.303196012	0.41634973	-0.988024557	0.067631324	0.310894509
Streptomyces_vietnamensis	NA	-3.160715525	-1.9510904	NA	NA	NA	0	0	9.104480817	8.442943496	8.215935782	0
Labrenzia_alexandrii	NA	-3.147769256	-0.564574609	0.146937769	0.351546911	-0.804886274	-1.412179015	-0.468034331	0.592381311	-0.03878851	1.412344684	0.906887974
Chryseobacterium_sp._G0186	NA	-3.114071767	NA	-1.492265826	1.210077812	0.85056235	-1.02915536	-0.949317763	0.23176003	-1.186897205	0.637329302	0.613882756
Buttiauxella_sp._3AFRM03	NA	-3.098171613	-1.002314673	-3.182620074	0.845644811	1.313494435	-0.723496528	-0.857515232	-0.047710634	0.170832284	0.264083193	-0.096773187
Streptomyces_sp._M56	NA	-3.071269825	-1.279856447	2.802858631	2.250481355	0.784732013	0	0	0	0.322409595	0.427327958	-1.451625735
Casimicrobium_huifangae	NA	-3.045679371	-2.817237905	2.760568665	1.002025309	2.729226404	0.669969911	1.041109168	1.36237723	-0.260931737	0.181103103	0.518640881
Mycoplasma_anserisalpingitidis	NA	-3.00921853	-0.476997491	0.573663845	-0.679240141	-0.4830425	-11.06706456	-2.45973425	-1.035900983	0.339678105	0.391920467	0.622380456
Friedmanniella_luteola	NA	-2.997437486	0.544320516	1.181720283	-0.75501943	-2.085880649	0	0	11.57674414	0.660229681	1.518169931	1.039420863
Saccharothrix_espanaensis	NA	-2.991988186	-3.87791642	-0.481379994	0.17531826	0.492933362	-0.706488183	-0.482176853	0.159954459	1.457823396	0.125188824	0.772781183
Lactobacillus_zhachilii	NA	-2.95642665	-2.887191017	-1.083780657	-0.940083774	-0.500257892	-0.043119135	-2.794945732	-11.17707689	-0.973806095	-0.667605397	-0.657267207
Candidatus_Nanopelagicus_hibericus	NA	-2.949168697	-3.299665944	-2.234838684	0.930811662	2.69659821	0	0	0	1.85472277	2.593923368	-0.810729649
Nonlabens_dokdonensis	NA	-2.938543797	-0.555215157	-0.667833113	-0.149844755	2.533728813	-0.347594242	-1.214077173	-2.02450493	-0.293598861	1.178530088	2.91653061
Wenzhouxiangella_marina	NA	-2.928398413	-2.148179621	NA	NA	NA	0	10.10089883	9.477205768	9.451211112	8.033423002	10.81057163
Candidatus_Pelagibacter_ubique	NA	-2.919545896	-3.704435232	-1.076815597	NA	0.079688889	0	0	0	0.797167214	-0.922538637	-0.294104246
Alloactinosynnema_sp._L-07	NA	-2.917444374	-1.644645051	NA	NA	NA	-10.50724956	1.281542167	1.552155741	7.129283017	0	0
Acinetobacter_schindleri	NA	-2.917444374	-0.700965577	1.652866565	-0.612883929	1.29802868	-0.080475375	-0.283058653	0.535832226	-1.107383535	0.40242745	0.730101053
Pseudoalteromonas_phenolica	NA	-2.917444374	NA	NA	NA	NA	-1.108326609	-0.128224169	0.238024162	0	0	0
Sphingorhabdus_sp._YGSMI21	NA	-2.913476108	-1.801266604	NA	NA	0.557591529	-0.784923975	0.477252971	0.227548752	-0.694497453	0.465193353	-1.188761643
Paraburkholderia_phenoliruptrix	NA	-2.890986443	-1.152003093	1.665040119	-0.144209347	1.371500851	-0.368473101	-0.400059501	-1.002899381	1.51579327	2.076205935	1.96734796
Arcobacter_peruensis	NA	-2.874489345	-4.149140435	NA	NA	-1.425428245	-0.54381402	-0.109350859	-0.150052801	-0.463196245	0.105308372	-0.118288941
Halioglobus_maricola	NA	-2.872689784	-1.150973959	-0.639597757	-2.391830108	-0.591802246	0.417278349	-0.07035866	0.789684907	1.292195708	0.41435037	0.478929814
Vibrio_furnissii	NA	-2.858539257	1.102084981	-2.026472211	NA	1.193628398	-1.584420615	-0.036084723	-1.260091876	0.451862667	0.303042048	0.883040858
Sulfitobacter_sp._THAF37	NA	-2.844107762	-1.571308439	NA	NA	NA	0	0	8.30544603	0	0	0
Streptomyces_sp._TLI_053	NA	-2.844107762	0.261189291	NA	-0.539928747	-0.380272081	0.779171345	1.181584288	-0.038009253	8.45395786	0	0
Corynebacterium_sp._23H37-10	NA	-2.844107762	NA	NA	-1.66828371	-1.755219347	-1.31430653	0.465907588	-1.584606933	-8.902878538	1.511172407	0.093360485
Halomonas_huangheensis	NA	-2.844107762	NA	NA	NA	NA	-0.493783894	1.372838162	0.961638806	8.45395786	9.100838995	6.544320001
Streptococcus_sp._oral_taxon_061	NA	-2.822047095	-1.35785657	NA	-0.944160363	-0.983975825	0.15757377	0.41218234	-1.926347878	-0.315410163	-2.664945106	-2.122022493
Achromobacter_pestifer	NA	-2.815347713	0.021137525	2.747625736	0.460149001	0.727109398	-0.673969577	-0.259345873	-0.601236199	1.9116726	1.755128112	0.955246361
Oenococcus_oeni	NA	-2.80700437	-1.316678744	NA	1.063533712	0.033897478	0	0	7.157114468	2.398868506	-0.893734747	-7.709655951
Streptomyces_hygroscopicus	NA	-2.804320862	-1.459937916	0.063242434	-0.639132216	-0.517249111	-0.025811957	-0.404485064	-0.298189977	-0.573537035	0.280903866	-0.12691724
Tenacibaculum_dicentrarchi	NA	-2.78642497	0.610053482	NA	-1.63592019	-0.381498914	8.502740328	0	7.745136469	-0.697296587	-0.634323795	-0.510102709
Kocuria_rosea	NA	-2.780781826	0.652707107	0.032604665	-1.692987897	NA	1.182601111	-1.383247462	1.525535111	-0.678587356	-1.644018936	-9.54689446
Flavobacterium_columnare	NA	-2.749303929	0.293426337	-3.630990339	-2.337576306	-0.2012865	-0.308787226	0.217675042	1.026504415	0.189055744	0.173122584	0.154824576
Nostoc_sp._NIES-3756	NA	-2.739530072	0.031331395	-0.750437377	2.00228383	0.827844778	-1.76671127	-0.544949501	1.968787305	0.020087957	0.596308048	2.259372462
Methylibium_petroleiphilum	NA	-2.734311141	0.059339979	NA	NA	1.962506918	-1.746876958	-11.61780262	-1.615114159	-0.57709274	-0.92994482	1.383845205
Pseudomonas_protegens	NA	-2.720965115	-1.359967456	3.489787677	0.59216827	0.007719093	10.12693673	11.25427869	12.20734688	8.235214866	7.29523629	8.520944699
Marinobacter_sp._NP-4(2019)	NA	-2.714548128	1.991502823	NA	NA	NA	0	0	0	8.27301694	0	0
Calothrix_sp._336/3	NA	-2.688421742	-4.646601567	-2.163373667	0.85402938	-0.142515062	-1.366691586	-0.193118266	2.487492483	0.19923503	2.050366128	1.653449924
Shewanella_marisflavi	NA	-2.66422998	1.29100015	NA	NA	NA	0	0	0	0.398549376	-0.44646821	-1.500074118
Micromonospora_sp._WMMA2032	NA	-2.63493096	2.922967059	NA	NA	NA	0.115044113	0.12630002	-0.892617002	1.482502357	1.995831663	0.0880861
Bacillus_pseudomycoides	NA	-2.623190554	0.134859887	NA	-1.502228749	1.047095415	0	0	0	-1.048330067	0.173021935	-0.627750114
Caulobacter_henricii	NA	-2.605886793	-0.246341207	3.424335244	2.179108324	-1.605000254	-1.016344051	-2.785114334	-0.786078626	1.142606757	0.254084548	-0.504285574
Corynebacterium_kroppenstedtii	NA	-2.604006119	-0.480117128	NA	0.935631867	0.520405743	0.171736252	0.372072987	0.583154288	-0.196784258	0.101464547	1.888555627
Aerococcus_sanguinicola	NA	-2.582677752	-1.26408537	0.039940013	-0.832843572	2.295228813	-0.068761229	-0.159225789	0.123910101	1.22767644	2.683306936	1.791231786
Aquitalea_sp._THG-DN7.12	NA	-2.574593527	-0.733291273	NA	-1.759583897	-0.519160442	-0.404166866	1.559375195	0.397354331	0.427718413	0.052199324	0.933051366
Gluconacetobacter_diazotrophicus	NA	-2.565382132	0.073373186	NA	-0.231005552	0.549794882	0.239232752	-0.526858307	-0.892147726	1.32029597	0.073374199	0.983233659
Staphylococcus_lutrae	NA	-2.561194335	1.045990904	0.816986541	-1.049536013	0.113349662	0	9.39192664	6.15711649	-0.792643178	-0.959823764	0.254023991
Myroides_sp._CJ210	NA	-2.552392035	-1.256577301	NA	-1.465508101	0.187388076	9.746667148	8.627369725	0	-0.123614706	-0.8527408	0.017432165
Rhizobium_flavum	NA	-2.519060564	NA	-1.09530696	-1.356934545	NA	0	0	0	-8.809501266	-8.809501266	-1.175292825
Synechococcus_sp._LTW-R	NA	-2.515652277	-0.211168824	-0.069406007	0.419402692	2.833452359	0.256922052	1.957117943	1.850186998	1.086284268	1.235333473	1.779220763
Flavobacterium_arcticum	NA	-2.498438608	-2.223957676	NA	NA	NA	0.428117502	0.469947206	0.671547262	1.007481705	0.284179106	-1.182030684

Arcobacter_defluvii	NA	-2.49386719	-2.105990696	NA	NA	-4.468873914	-0.822006746	0.848049655	1.185178173	0.532195608	0.338834916	0.153011104
Arcobacter_nitrofigilis	NA	-2.491140478	NA	0.524266569	NA	3.614378989	-0.258910376	-0.714310576	-0.192440717	8.366322214	5.472487771	8.658211483
Citrobacter_sp._LY-1	NA	-2.476625298	-2.351843673	NA	NA	NA	-0.551275366	-0.036662531	-0.274560862	-8.087462841	-8.087462841	-0.706638172
Acidibrevibacterium_fodinaquatile	NA	-2.441316824	0.631385897	-0.227960782	-0.771595467	-0.925149679	-0.212677465	-0.317836839	-0.341259358	-0.064719486	0.764568878	1.964911068
Azoarcus_sp._KH32C	NA	-2.430862466	-5.383556752	-0.217403712	2.292805284	3.457543274	-0.379889224	0.392858491	-0.093182171	1.181997283	-0.958500534	-0.748771341
Brevundimonas_vancanneytii	NA	-2.409013712	-0.221386708	NA	NA	NA	-0.268327976	0.132280706	0.424068846	8.89683794	7.65343985	10.306825
Pantoea_sp._201603H	NA	-2.402374086	-2.200937334	NA	-2.627907146	-1.362765078	-0.044021396	0.775880003	0.511856302	-7.807354922	-0.512118632	2.730513807
Salinivibrio_kushneri	NA	-2.400595351	0.82468174	0.819467172	-0.082740431	-0.795284553	0	0	0	0.457279948	0.014902552	1.73932578
Microbacterium_sp._4R-513	NA	-2.39634227	-1.054819768	NA	NA	NA	0.220851815	-0.235281034	-0.501217959	7.129283017	7.222389201	9.038918989
Sphingopyxis_sp._OPL5	NA	-2.39523435	1.103927443	0.535037275	1.628115704	1.111549859	-0.815857766	0.606618798	0.493252191	1.408430311	2.603664305	2.209534013
Avibacterium_volantium	NA	-2.376854305	-1.886650338	5.591448806	-0.336182079	1.205504981	2.822856652	1.866335397	3.222925986	9.64385619	10.1581931	0
Acidovorax_sp._JMULE5	NA	-2.361389478	0.276293303	-0.277663708	NA	-0.070175294	-1.307483662	1.056658169	0.48359374	1.057225611	0.798563457	2.022093771
Rothia_nasimurium	NA	-2.338919346	0.818042491	-0.245510713	1.336900602	-0.982901192	0	0	0	0.307422363	0.49728423	1.760862337
Bartonella_australis	NA	-2.327405413	-4.458856267	NA	-0.794025338	NA	-0.775265593	0.283414915	-0.600291825	-0.234464538	-0.038114396	-1.086858284
Candidatus_Enterovibrio_luxaltus	NA	-2.321928095	-0.32764122	-0.039052777	NA	NA	-0.367823073	0.315936546	0.993442826	0	0	9.112004156
Thermodesulfobium_narugense	NA	-2.313590212	-1.343235673	NA	1.877413395	3.200811378	-0.897414737	-0.443431	0.455080604	0.60869952	-8.202941691	-8.202941691
Sphingomonas_indica	NA	-2.308960474	-0.218968573	NA	-3.222392421	-0.074000581	-9.661778098	-9.661778098	1.018897887	2.455196503	2.513409905	3.121091718
Agrobacterium_sp._RAC06	NA	-2.304138781	-3.143674109	NA	NA	NA	0.28901281	1.013014986	0.991350375	8.62935662	0	0
Urechidicola_croceus	NA	-2.280177479	-3.530027895	NA	-2.220804382	-1.654753204	0.139584418	0.16380518	0.828168255	-0.092812212	0.363536941	0.560555879
Pseudomonas_saudiphocaensis	NA	-2.270986529	-1.355615224	1.808706393	-1.397566363	-0.080634002	2.502774434	1.136661803	1.203198384	10.14465824	11.16138386	9.306820443
Staphylococcus_piscifermentans	NA	-2.265683984	NA	NA	-0.447624794	-2.016509087	0	0	0	1.318151775	0.201248778	-10.19393187
Fluoribacter_dumoffii	NA	-2.261265313	0.973951149	-1.631132682	-0.068331831	1.390152026	-0.192547301	0.230265997	0.304459398	2.606981488	3.405585208	1.921699848
Mycolicibacterium_hassiacum	NA	-2.25958121	-1.878343233	2.927850214	0.222392421	-1.938013113	1.117824322	1.062495575	0.782095564	-2.11180619	0.814039318	1.268541572
Leadbetterella_byssophila	NA	-2.258546873	0.519822427	-1.885262657	-2.140297941	1.281858519	-8.637284195	-8.637284195	1.261385207	0.267589697	0.127514497	0.69907169
Rickettsia_australis	NA	-2.256458829	-1.671923098	NA	NA	NA	0.051467605	1.041318804	-8.552496194	-2.435489334	-10.70850627	-10.70850627
Nitrospirillum_amazonense	NA	-2.254633257	-0.801075859	-0.500386783	2.929744062	2.256614736	-2.696967559	-1.825458386	-0.460569566	0.891173638	0.68804925	-0.429249668
Saprospira_grandis	NA	-2.254611827	0.120632083	-3.919630615	0.777232467	1.274344125	-0.803809845	-0.236022621	0.860516621	1.004257808	0.852449412	1.276766008
Caldicellulosiruptor_hydrothermalis	NA	-2.239465935	-2.427538283	0.193964013	2.69664529	2.160510971	-7.757316664	0.353109224	3.037391611	-0.352085594	0.108430218	1.501605102
Massilia_sp._YMA4	NA	-2.213610057	0.024542001	NA	-0.760002602	0.326544052	0	6.682671608	0	0.943761468	0.196701185	0.40391982
Hyphomicrobium_nitrativorans	NA	-2.205174196	-0.32960748	-1.164102841	-2.619694439	2.131125164	-0.108641549	-0.278033634	-0.190319882	0.788176069	1.851105549	-0.825084128
Komagataeibacter_nataicola	NA	-2.204091605	-2.198092864	-2.092628243	NA	0.110152587	0.310858555	-0.139023941	0.245508657	-0.534544594	-8.900866808	1.908097367
Methyloceanibacter_sp._wino2	NA	-2.192866787	-0.285976191	-2.442269245	0.91556881	1.334605071	-9.222246695	-9.222246695	0.740912383	-1.061108376	-0.483256265	-1.624946537
Capnocytophaga_leadbetteri	NA	-2.186878135	2.623264605	5.064481372	6.318181918	5.462413758	1.967201045	-1.566345542	3.168322879	0.168066359	0.381934708	1.867745058
Erythrobacter_sp._HKB08	NA	-2.177771085	-4.806768182	NA	NA	NA	0.321573813	-0.130295223	1.088324225	1.052698458	1.406964199	-0.26303183
Leptospira_weilii	NA	-2.170191698	1.283554846	NA	-0.78827489	0.015476595	0	7.68267863	10.51981314	-0.373602323	0.497878669	-0.325428953
Paracoccus_kondratievae	NA	-2.166649869	-0.833246455	2.859732162	-0.933086667	0.291112511	-0.391830514	1.093415752	2.13838999	9.016808288	0	0
Arcobacter_canalis	NA	-2.162528273	-1.755847762	NA	-3.013737946	-1.539806758	-1.411242353	-2.537981834	-2.516411315	-0.941810754	-1.318252437	-1.511768324
Bordetella_trematum	NA	-2.15251193	-1.04016997	4.724103443	0.934510918	2.309926997	-1.868815202	-0.219936565	0.043621195	0.127681476	2.746406354	1.935036398
Delftia_acidovorans	NA	-2.135298309	-1.271018958	-0.476123834	-1.086775596	-0.286158567	0.272649132	0.516076086	0.705424652	0.404637707	0.233148177	-0.172584174
Streptococcus_sp._LPB0220	NA	-2.113047316	0.457933159	NA	NA	NA	0.605425419	0.746364093	0.512695771	1.277845425	-1.121643985	-0.885088738
Grimontia_hollisae	NA	-2.096585979	-1.159832583	NA	-1.666483931	1.572302929	0.318662786	0.353108803	1.013224806	-2.51222038	-1.703805302	0.708850173
Algibacter_alginicilyticus	NA	-2.081498415	-2.602358248	-0.189072348	0.61356942	1.374505577	0.283806573	1.234689037	2.138227232	-1.362569598	-0.622525914	0.044622906
Microbacterium_sp._1S1	NA	-2.070726052	-4.028905876	NA	-0.048079716	0.093216872	0	0	0	-2.157435612	-0.086900378	-0.480140162
Amycolatopsis_mediterranei	NA	-2.066624732	0.174967579	NA	NA	NA	-0.504632103	-0.075046672	0.085867946	-0.943727682	0.835928173	1.721474946
Taylorella_asinigenitalis	NA	-2.060542579	-0.526388156	1.934836334	2.325492782	2.120188272	-0.879031457	-0.04207256	-0.571783582	-0.944910901	-0.009511415	0.024298596
Thiomonas_arsenitoxydans	NA	-2.05622779	0.84225345	NA	NA	NA	-1.250822414	-1.281219156	-0.011978164	9.571121208	7.369230901	9.430452552
Empedobacter_falsenii	NA	-2.054707706	-0.043173385	NA	-2.212725882	-0.72547093	-3.220091008	-0.05323256	0.811152683	-0.950586103	0.138363601	-0.007967962
Haloactinobacterium_sp._HY164	NA	-2.031609607	-1.313022542	2.47914457	0.265173317	1.010662983	0.705090853	1.551977185	1.666940673	2.277641969	3.343568899	3.147952785
Synechococcus_sp._PCC_7502	NA	-2.026242903	-0.842188301	NA	0.097031806	0.392597276	0	0	0	1.300111957	2.2895323	0.945405819
Tolumonas_auensis	NA	-2.026082051	1.476569735	0.516085696	-0.519109953	1.961292116	-0.478693045	0.268933117	0.175228959	1.350004165	0.872906332	0.287672598
Calothrix_brevissima	NA	-2.021156972	-2.844477278	NA	NA	NA	9.561202011	0	9.154818109	0.928412128	2.151341928	1.736437747
Sulfuriferula_nivalis	NA	-2.018285353	-0.306790446	-3.357838055	-0.635905276	0.707375554	0	0	0	-1.098028719	0.239942278	-2.494207784
Mycolicibacterium_parafortuitum	NA	-2.013958458	-1.771101934	NA	NA	NA	0	6.682671608	0	8.366322214	8.979187942	0
Mycoplasma_hyosynoviae	NA	-2.012383724	2.467257182	NA	-1.495678138	0.470808839	-0.447240403	0.840737989	0.567587511	-0.416420656	0.402150962	0.683919925
Limnohabitans_sp._63ED37-2	NA	-2.009691423	1.902845736	2.481300611	-1.855943084	0.773328313	-1.267091226	-0.198577956	-0.750461681	1.246925336	1.630757044	2.261085537
Psychrobacter_sp._P11F6	NA	-2.00604482	-1.210710616	NA	-0.293980627	-1.482028978	0	0	0	0.134106889	1.800752187	-1.235628248
Pseudomonas_mosselii	NA	-2.004413265	-0.26744767	NA	0.031609607	-2.728871912	2.073168903	1.399589119	1.583619457	-0.710496364	-8.044394119	-8.044394119
Nocardioides_sp._MC1495	NA	-2.001105937	0.044628603	2.488446071	1.726246706	-2.32394444	-0.595765906	-0.429809501	1.399865899	8.45395786	9.568588898	10.11807156
Corynebacterium_ammoniagenes	NA	-1.99223461	1.197711189	-1.573647187	3.642189122	4.517953484	-2.377545108	-1.047105676	0.065140485	3.277756021	5.481309116	5.165527168
Sulfitobacter_sp._SK012	NA	-1.990576746	-4.861293729	NA	-0.037474705	-0.597346225	-0.170205569	-2.089688031	-0.063238124	-1.801454321	-8.930737338	0.021517591
Helicobacter_cetorum	NA	-1.981317821	-4.077930469	NA	-3.532103069	-3.020895438	0.063202367	-0.836913731	0.14970785	-1.111445273	-0.720560078	-1.149106574
Edwardsiella_ictaluri	NA	-1.979801191	1.054215245	-0.932706887	1.150329148	2.314825096	-0.17085408	-0.37136153	-0.192970401	-0.82498341	0.249071753	-9.636624621
Vibrio_sp._THAF190c	NA	-1.972332251	-0.489939483	NA	NA	NA	0	0	0	0.355480655	-8.087462841	-8.087462841
Synechococcus_sp._PCC_73109	NA	-1.958472655	0.508742803	NA	-1.625734172	1.661770093	-1.977924065	0.940605396	1.011928067	2.280839342	1.33134683	1.351782021
Lysobacter_lycopersici	NA	-1.949626477	-4.902320763	NA	NA	NA	-0.522976614	-0.631772488	0.990647624	0.610209924	3.219020108	1
Mycoplasma_sp._Mirounga_ES2806-NAS	NA	-1.948710771	0.655569907	1.038252369	1.499622392	-2	-1.095751911	-0.626015885	-0.18562544	-2.545547145	-5.128364937	-2.151903971
Sphingobium_sp._Cam5-1	NA	-1.941491595	0.491467813	NA	NA	NA	-2.537473426	-0.664684599	-0.464140129	0	9.214319121	0
Sulfurospirillum_multivorans	NA	-1.938323103	-0.728697978	NA	NA	NA	0.244615558	0.45437457	0.212075401	0	8.291552911	10.74090227
Diaminobutyricimonas_sp._LJ205	NA	-1.938323103	NA	NA	NA	NA	-2.181167879	0.966706084	0.835537848	0	0	0
Methylophilus_sp._TWE2	NA	-1.925161698	-3.298379021	NA	-0.985680329	0.292120788	0	0	0	0	8.215935782	9.423466822
Sphingobium_herbicidovorans	NA	-1.921062863	-2.927440433	NA	NA	NA	0	0	8.624046047	1.584963626	-8.739216921	-1.808479583
Sedimentisphaera_salicampi	NA	-1.918903113	-1.305276854	0.986048555	0.080988841	0.528765995	-0.772749567	0.104682126	1.351395267	-0.884202732	2.242008873	0.900070836
Terasakiella_sp._SH-1	NA	-1.906680205	-0.165189089	NA	-3.640984818	-1.189629082	0	0	8.904155902	1.740844476	0.227085984	0.839782997
Nocardioides_sp._WS12	NA	-1.905475495	-1.939283919	3.184250348	-1.953924129	-0.760751847	0	0	9.822499133	1.285922642	0.597662259	0.964686653
Nocardia_nova	NA	-1.894009304	NA	0.327281329	1.340048625	0.490780062	-9.488205685	-9.488205685	1.000909706	0.404995647	-9.046215465	-9.046215465
Methylovulum_psychrotolerans	NA	-1.891106738	0.298019512	-3.261750951	0.930722302	-0.332745194	-0.142946309	-1.121365872	-0.227062457	-0.631371397	-1.630525231	-0.285553484
Staphylococcus_stepanovicii	NA	-1.890112807	-3.591151555	4.861293729	3.084517871	3.154021454	0.860157469	0.87481535	0.608299224	-0.334724317	-0.117763682	0.890368413
Capnocytophaga_gingivalis	NA	-1.888709075	-0.076684162	-0.936049921	1.717701488	1.516926749	10.16056477	11.94830152	9.97102616	0.70871791	0.615682564	1.14406923
Chryseobacterium_piperi	NA	-1.886537064	-2.184345278	NA	NA	-2.703977949	0	0	6.15711649	0.758584518	0.829980684	0.727513173
Arthrobacter_sp._NEB_688	NA	-1.870445663	-2.811678255	NA	1.596839243	0.140840048	0.284515876	0.492724433	0.297820694	1.425436526	2.092364594	1.52841196
Shewanella_psychrophila	NA	-1.866817026	-2.894515149	NA	NA	NA	-0.399436628	-0.209964177	-0.094717254	0	0	0
Paracoccus_pantotrophus	NA	-1.853095467	-1.915603364	NA	NA	NA	-1.029846532	-0.781188298	0.833400495	1.380694124	2.084606207	-7.890768904
Capnocytophaga_ochracea	NA	-1.849591926	-2.9486717	NA	NA	NA	-1.644309014	-1.949714127	-1.957443703	0.690891773	1.43486485	1.41374526
endosymbiont_of_Sipalinus_gigas	NA	-1.833028316	0.442434734	NA	NA	NA	0.001829432	0.283484069	-0.072781174	-8.910891527	0.162358348	0.45979588
Streptomyces_sp._JB150	NA	-1.828416538	-1.227745656	0.058245141	NA	2.42613909	-0.423213177	0.874150153	3.605052856	1.275365491	1.074407049	1.578587895
Alkalihalobacillus_lehensis	NA	-1.824953529	-0.485797462	NA	0.167290071	0.543387142	-0.474438165	-0.398737498	0.284639044	0.722033858	1.056259083	1.114926062
Fluviicola_taffensis	NA	-1.821956581	-2.511088255	-0.640379615	2.094310598	1.794415866	0.553529745	-1.44385609	0.358019562	2.545870171	1.948069048	3.428181085
Serratia_rubidaea	NA	-1.817895816	-0.763170304	1.869125999	1.643233482	1.016073105	0.226066035	0.95765067	0.798588978	-1.010536386	0.058932071	-0.040664982
Pseudomonas_frederiksbergensis	NA	-1.814734452	-1.02118533	NA	NA	NA	0	0	0	-10.20130304	-0.871881158	-0.472248272
Microbacterium_sp._TPU_3598	NA	-1.810079754	-1.571262367	-0.6476714	-0.034516532	1.682019342	-0.382398292	-0.718789265	0.897398499	0	0	0
Macrococcus_canis	NA	-1.809280584	-1.709332653	2.028509961	0.43166019	-0.477276248	0.413578627	1.000291598	1.14537372	1.048925202	1.07315579	0.573241016
Vagococcus_sp._CF-49	NA	-1.808477642	-1.501565408	-0.116607153	NA	-0.411984456	-0.405082839	0.369497119	0.192900745	1.508036896	3.325549136	2.323378036
Serinicoccus_profundi	NA	-1.767890167	-0.40487488	NA	NA	NA	2.178000449	-8.076815597	-0.331679128	9.367779474	0	10.05165759
Brevibacterium_sp._'Marine'	NA	-1.767656675	0.118901249	NA	-1.582346553	1.415495633	-2.638603473	0.859483235	1.826141799	0.248645291	0.126795109	1.034295135
Haemophilus_haemolyticus	NA	-1.758578199	-0.586596303	3.07384033	-1.35486244	0.801151667	-0.41459095	-0.011262194	-0.195051435	1.331771676	0.06742167	1.101562614
Thermoanaerobacter_italicus	NA	-1.745427173	1.257384467	NA	2.494699051	1.671187954	-0.142595172	-1.345709056	0.370053011	0.305667651	0.182197193	0.116902948
Microbacterium_amylolyticum	NA	-1.745349531	2.07919344	NA	0.981303906	1.99612491	1.815869914	3.714327234	3.000267013	0.382847461	0.061149087	-0.488553207
Lactobacillus_sp._NFFJ11	NA	-1.742283311	-0.580193257	-2.295685192	NA	0.688751604	1.455372976	-8.04736934	-8.04736934	0.245043178	-0.046104159	1.183268612
Fervidobacterium_pennivorans	NA	-1.73141676	-2.205809537	NA	NA	NA	0.055549473	0.507118601	0.258715507	1.025401265	1.534784817	-2.323861363
Spirosoma_sp._BT328	NA	-1.722872668	0.667526177	NA	1.242437501	1.766409372	-0.730368908	-0.203373553	-0.907165926	0.638261631	-0.627096154	1.220297747
Lactobacillus_sp._CBA3606	NA	-1.712508012	0.370470358	NA	0.806881053	2.323254101	0.320105007	0.949316862	0.753239097	-0.238881715	-8.681825211	-0.160880512
Azospirillum_ramasamyi	NA	-1.703468682	0.084612228	NA	NA	NA	10.2623063	9.380166652	9.311646154	0	9.41644499	10.31137369
Deinococcus_sp._S14-83	NA	-1.693428389	-0.612398184	NA	0.353441838	0.567657477	9.248359308	0	10.44228004	0.534148167	0.925588584	2.887525271
Corynebacterium_sp._zg-320	NA	-1.690739284	-3.048512578	0.878057913	NA	-0.419435963	-2.769386385	-1.51850275	-3.741554933	-8.992465383	0.959791002	1.398423666
Magnetospirillum_sp._ME-1	NA	-1.685126663	-1.337961276	3.408238924	0.667661071	-0.152476031	8.502740328	9.889020195	10.5533701	-10.5666915	-5.094203729	-1.259871057
Listeria_grayi	NA	-1.665764834	-2.572343462	-0.328986688	-0.482847305	0.926575788	1.4854222	-8.799135887	0.357981108	1.88703862	1.460627303	1.854987408
Mannheimia_haemolytica	NA	-1.662417312	-2.436805314	4.303387295	-1.786319088	-0.82695076	-10.09131637	-0.860313649	-1.22741198	0.919109209	0.908360687	1.445314095
Celeribacter_manganoxidans	NA	-1.661204729	-0.688794049	1.485059308	0.229827661	0.021157475	-10.17311494	-10.17311494	-10.17311494	1.138866935	0.902309234	1.493803237
Acidovorax_sp._KKS102	NA	-1.655006314	-0.548375691	-1.278674897	-0.102002011	-0.087266836	1.240414418	1.816005304	1.230570292	0.679978598	-3.008027591	0.473486781
Marinobacter_similis	NA	-1.647246779	-0.746024933	2.747210533	2.864337157	0.713910358	-0.619066981	-0.316154255	-0.439068848	0.651254179	0.673634397	1.172099214
Thiomicrospira_aerophila	NA	-1.63493096	3.039113499	NA	NA	NA	0	0	0	-0.782282449	2.103883308	0.33621712

Pseudoalteromonas_rubra	NA	-1.630724676	-0.262519709	NA	0.129575327	0.61533615	0	8.559465045	0	-0.416496249	0.554993503	2.101044682
Microbacterium_oryzae	NA	-1.629704628	1.258589746	NA	NA	NA	0	0	0	0	0	0
Sphingobium_yanoikuyae	NA	-1.619157006	-0.025958443	1.987013159	1.233901591	2.132915486	0	8.627369725	8.71892325	-0.936648082	0.270004115	-0.722910126
Tetrasphaera_sp._HKS02	NA	-1.618158925	-0.333966941	3.890588783	1.357156583	3.174720004	0	0	8.548995253	-0.388830739	1.070652156	1.801757608
Alkalihalobacillus_pseudofirmus	NA	-1.612863477	1.892985927	-0.367109435	-1.44422167	-0.414129264	0.630232669	2.215366451	2.231701798	-0.500536528	0.438262986	0.996648155
Bacillus_sp._THAF10	NA	-1.612432104	2.324241961	-3.424128107	-0.983296682	-1.366087171	-0.319850311	-0.088247953	-0.072166298	0.205876855	0.456192541	1.002522082
Pseudomonas_sp._C27(2019)	NA	-1.592418306	-0.243240798	NA	NA	NA	0.284226244	-1.132077509	-0.154930249	0.969284476	-0.831878822	0.850698082
Sulfurimonas_paralvinellae	NA	-1.559017304	0.024868424	NA	-0.116042901	0.976869472	-0.26504028	-10.49276451	1.780448298	-0.257828581	0.143948961	-0.033222126
Corynebacterium_sanguinis	NA	-1.544320516	0.326285962	1.007421711	-0.41917723	-2.442581834	0.192824936	-1.384958706	-1.165356985	0.382248036	1.323550217	1.532697918
Rhodococcus_coprophilus	NA	-1.530855095	-3.102163534	NA	NA	NA	0	0	9.154818109	1.156289403	0.853490361	2.380166182
Salinibacterium_sp._ZJ450	NA	-1.52348499	-0.78051331	NA	NA	NA	-0.640062375	-0.418720632	-0.412177902	-7.741466986	0.474468795	-0.810729649
Streptococcus_sp._oral_taxon_431	NA	-1.510140927	-1.619100091	-1.657894023	NA	-1.291766124	0	8.382131157	0	-0.391477261	1.577639106	1.367604233
Salegentibacter_sp._T436	NA	-1.505617545	NA	-0.324261924	0.729233957	2.100136671	0.432979172	-0.073911368	1.585464668	0.550563239	1.599895764	0.660117272
Chryseobacterium_sp._G0162	NA	-1.49441762	1.152153806	NA	NA	-0.741311449	-0.851171693	0.169803079	-0.319331914	0.661368949	0.569178329	1.877751886
Geminocystis_sp._NIES-3709	NA	-1.472046288	0.958693433	-1.045590964	0.25492879	0.09489026	-0.051677211	0.736515291	1.051347971	-0.181796739	-0.171142995	0.429926015
Corynebacterium_cystitidis	NA	-1.46456577	1.089842508	NA	NA	NA	10.17990909	0	8.742080338	-0.126239997	-0.721880195	-9.531381461
Pirellula_staleyi	NA	-1.463856517	-1.690977828	-0.431805713	NA	3.660533162	-9.37029072	-9.37029072	-3.21317423	-1.784455504	-0.833875062	-0.630981834
Leeuwenhoekiella_sp._ZYFB001	NA	-1.462485963	0.153415549	NA	-0.205748665	1.220652138	-1.376884676	-0.606019239	-0.427982666	0.443828037	1.493499044	1.274319458
Sinorhizobium_sp._RAC02	NA	-1.461473685	-1.659895056	NA	NA	NA	0.20770311	0.340579553	-0.38806991	1.089311225	2.571869575	3.071777102
Xanthomonas_arboricola	NA	-1.444364171	-2.144533409	2.201633861	1.947141553	1.982297998	0	9.695386649	10.41228403	-1.086493756	-0.733096693	-2.159626946
Leucobacter_triazinivorans	NA	-1.439802019	0.58964658	4.129188256	0.374679762	1.998340776	2.502934484	2.489891447	3.030348417	0.269478503	-1.067659339	-8.721099189
Methylocella_tundrae	NA	-1.437524509	-2.559444137	-0.992289158	-1.267680771	2.301841681	-1.577281884	-1.352270249	-0.428962056	0.477541364	1.176536152	0.899136598
Synechococcus_sp._KORDI-52	NA	-1.432012733	-0.486082581	NA	0.515549189	-0.244772199	0.375345621	0.286682111	0.924341652	-0.371557165	-0.222014989	1.12296135
Dietzia_psychralcaliphila	NA	-1.428499759	1.989540228	NA	NA	NA	0.455767385	1.675698914	0.771004154	-0.035919574	-7.930737338	-1.386417337
Mycolicibacterium_vanbaalenii	NA	-1.426922468	-2.293926364	NA	NA	NA	9.143268406	11.38065586	8.598897393	0	6.470320477	0
Flammeovirga_sp._MY04	NA	-1.416973223	-1.586123624	-1.286746762	-2.364202902	-1.098000585	-0.132144374	-0.26976608	-0.516776627	0.899522536	1.849194661	1.589747563
Psychroflexus_torquis	NA	-1.416491096	-0.761508669	2.014222617	-0.345570135	1.896790703	0	8.559465045	0	1.769521859	1.421055409	0.427434218
Corynebacterium_stationis	NA	-1.403058512	4.756640053	0.410450813	10.17069566	10.48361007	-0.227099894	0.788031063	0.528106001	10.51821338	14.26063474	13.52146478
Capnocytophaga_cynodegmi	NA	-1.401747882	-0.079716037	-2.055686576	1.988341795	1.269419625	0	0	0	-1.086212239	0.060533793	0.874684431
Hymenobacter_baengnokdamensis	NA	-1.39777204	0.635643022	-0.20787277	0.158095158	1.440022872	-0.318512891	-0.466594866	-1.150758638	0.497847223	0.548511049	0.796100603
Shewanella_donghaensis	NA	-1.393749713	-1.009123526	NA	2.052543864	2.835216397	-9.462410248	0.421065473	0.286545686	1.530341084	3.289871996	0.416186261
Bradyrhizobium_japonicum	NA	-1.389959802	0.054804437	NA	NA	3.136015309	-0.375478778	2.394485449	1.143282944	-0.385891154	2.025535092	0.636374853
Ornithobacterium_rhinotracheale	NA	-1.387329324	-1.89987479	NA	0.949722329	1.235015916	10.00332114	10.7351752	9.069511529	-0.2767617	-0.469947313	1.107834678
Brevundimonas_mediterranea	NA	-1.384212373	-1.146744631	NA	NA	NA	-0.416394163	-0.839946578	-0.583557254	7.129283017	10.06123617	9.832890014
Pseudarthrobacter_sp._NBSH8	NA	-1.368777974	-0.768892105	NA	NA	-0.95429639	-3.10248452	-1.221131858	-2.981178801	-0.473457166	1.545791903	-7.807354922
Ornithinimicrobium_sp._H23M54	NA	-1.355653217	0.590817386	NA	1.048334961	0.901603806	-0.008869183	-0.454539469	-2.71376683	-0.275347962	-10.44777547	-1.140955028
Mycoplasma_crocodyli	NA	-1.350450178	NA	NA	-0.44633363	0.69805628	0.422104926	0.399834193	0.219327067	0	5.472487771	9.540451616
Streptomyces_qinzhouensis	NA	-1.349942471	-1.39665897	-2.625661816	NA	-1.39709082	1.304534982	1.399716114	1.264302129	-2.857011741	-1.591170456	-1.865452591
Synechococcus_sp._KORDI-100	NA	-1.349942471	-0.133624564	NA	-2.349504339	-0.69455432	0.301185907	1.03463541	2.096779653	-2.610931696	-2.433665028	-1.373379455
Candidatus_Methylopumilus_rimovensis	NA	-1.347350692	-0.607982607	3.122349025	-0.006648376	1.14352103	0	0	9.61571239	2.112651276	2.6027095	1.003237571
Mycoplasma_genitalium	NA	-1.334346452	-0.052057821	-2.609540523	-1.131569501	0.649770796	0.301465974	0.220482904	0.50156293	-0.575506954	-0.79696462	0.412663615
Actinomyces_sp._ZJ750	NA	-1.333126301	1.383650855	0.717225854	NA	-1.448815713	-1.482589319	0.062039694	1.063279831	3.304823942	1.889748381	1.352514847
Pseudomonas_sp._MSPm1	NA	-1.328982852	0.088583895	1.706886267	-1.250245345	0.052158591	-0.235415723	-11.09791361	-2.378990359	-0.403312644	-1.579851623	-1.371567507
Aurantimicrobium_sp._MWH-Mo1	NA	-1.31956108	0.230693686	2.509122559	2.234717099	0.668469065	-8.076815597	2.985351997	3.131638259	1.554458881	0.279990343	2.071983115
Cellulomonas_shaoxiangyii	NA	-1.31482995	2.136128257	3.550540336	0.873188998	0.080408084	0.318760744	2.400910592	1.579092404	0.545636693	2.317091249	1.126079748
Ewingella_americana	NA	-1.299623159	2.171437895	1.298411692	2.249657808	2.113438305	0.837365123	-0.407077483	1.659589116	-0.808940729	-1.908547913	0.488301897
Dermabacter_vaginalis	NA	-1.280107919	-0.116230573	-2.199416933	-1.609101198	1.549904249	-0.641353021	0.555498544	-0.726126426	0.25093303	0.259734124	1.075546023
Photorhabdus_laumondii	NA	-1.275368818	-0.687355146	NA	0.09726355	-0.482643193	-8.637284195	-8.637284195	3.194957094	9.926789659	8.215935782	7.539158811
Altererythrobacter_marensis	NA	-1.264149062	-1.013593727	4.12199823	2.833936733	3.201179254	8.166278594	6.682671608	12.55293081	-0.784411397	0.366980541	0.639771732
Octadecabacter_antarcticus	NA	-1.246025715	-1.327678906	NA	NA	NA	0.677959937	1.2227946	1.044017495	-0.035593163	0.631502496	-1.650463244
Roseomonas_gilardii	NA	-1.237245724	1.610751182	1.62658159	3.39933785	4.002197556	-1.30539662	-9.471675214	1.117039421	-0.7843641	-1.132446709	-0.757836402
Microcystis_aeruginosa	NA	-1.236243493	-2.52464134	NA	NA	NA	-0.254603867	-0.228764017	0.383004925	0.024213615	-1.589444044	-8.884680334
Virgibacillus_dokdonensis	NA	-1.226718836	-0.141176063	-0.014355293	-0.481972707	0.462367465	-0.209601346	-0.049447948	0.049884272	-2.493438	-2.731460078	-1.335141053
Ochrobactrum_quorumnocens	NA	-1.209603561	-0.396662725	2.312285092	1.448028396	1.236812661	-2.359002419	-0.842828895	0.90609755	-0.578881418	-0.893362211	-0.683054364
Flavobacterium_sp._MDT1-60	NA	-1.204639612	0.275384567	-2.19237954	-4.791245879	-1.988941973	0	9.384097309	7.157114468	0.194340565	0.230280692	0.12644781
Euhalothece_natronophila	NA	-1.20421535	-0.572395867	NA	NA	NA	0.093245427	0.16257832	0.071808276	9.202939243	10.92807417	9.0750325
Staphylococcus_caprae	NA	-1.183864192	-0.809668788	-0.682300619	1.143887141	-0.968673666	-0.002013188	-0.244105657	0.251693151	8.725649145	0	8.412218392
Alteromonas_australica	NA	-1.178438397	0.01529373	-0.60823228	NA	-1.127841043	-0.712402629	-0.289559585	0.053965588	0.250886086	-1.396408699	-2.119111212
Halothiobacillus_neapolitanus	NA	-1.173482821	3.519756309	-0.634867407	-0.42043364	0.6617496	10.25481605	10.35434857	6.15711649	-0.402783809	-0.718971791	0.34559177
Neptunomonas_concharum	NA	-1.172280422	3.981942024	NA	-3.084021864	-0.425320978	-0.932882111	-0.13786598	2.293584985	-0.504974509	0.215328739	0.961736327
Exiguobacterium_sp._AT1b	NA	-1.166641305	-0.548359687	NA	1.195115808	1.160566249	0.020377323	-0.611197818	0.851077023	0.238711042	1.27192765	0.864675588
Candidatus_Accumulibacter_phosphatis	NA	-1.154123145	0.919965834	NA	NA	NA	0	0	0	-1.482020247	1.348146687	-1.435093333
Chloroflexus_aurantiacus	NA	-1.153189813	-1.174558739	0.665695263	NA	0.066785526	-2.366443652	-0.790501709	-1.845467564	-0.457653377	-0.166314569	-0.052426281
Porphyrobacter_sp._YT40	NA	-1.14980888	-1.161678253	3.695860391	3.427646346	2.670623099	0.417957522	0.864702721	-0.725880146	0.380525208	-0.232883981	0.500581177
Moraxella_bovis	NA	-1.14839184	-2.920513879	NA	NA	NA	0.587267351	1.806360547	0.948371031	-8.087462841	0.128472941	0.654004145
Bartonella_krasnovii	NA	-1.147320938	-0.965331448	NA	NA	NA	0	0	0	0	9.537864847	0
Coraliomargarita_akajimensis	NA	-1.133496014	0.295139509	-1.968215614	1.69098818	1.326957027	-3.024096286	0.08905343	0.377244047	-1.012062267	0.405516976	0.898053159
Nocardioides_sp._HY056	NA	-1.122549266	-3.171825607	NA	NA	NA	-9.372270887	-0.99013973	-9.372270887	0.251016169	0.607845208	-0.013117132
Polaribacter_sp._L3A8	NA	-1.120506639	-1.418297828	NA	-1.906890596	-2.335635514	-0.723365125	-0.046150999	-0.398612766	0.022742591	0.251680474	-0.362816929
Paracoccus_yeei	NA	-1.114111178	0.761918967	NA	-1.225488335	0.148287199	0.383555746	-9.177646265	1.381816868	0	0	7.539158811
Nitrosococcus_wardiae	NA	-1.112621139	1.510233149	0.09946966	NA	-0.491280761	-11.63357198	-11.63357198	-0.925583099	2.411759719	-0.351805501	1.54432384
Massilia_plicata	NA	-1.10773024	1.356673337	NA	NA	-1.158609688	0	0	0	-2.014451156	0.343554202	1.735525776
Cupriavidus_oxalaticus	NA	-1.10578258	-0.119605634	1.904902956	2.160995989	2.5993462	0	0	9.561202011	0.582266205	0.107267769	0.354042628
Flavobacterium_commune	NA	-1.103171958	-2.162002439	NA	NA	NA	-0.728189161	0.586797373	0.894406224	1.863684244	2.407621445	1.612003828
Marinobacter_salarius	NA	-1.097451858	-0.337419177	NA	NA	1.612038301	-9.482717218	-1.37229133	1.198363033	-0.586988156	3.14677805	3.600748526
Pedobacter_sp._eg	NA	-1.087241042	-0.384840734	-1.178470668	0.590023607	1.900190179	0.413890285	0.278774711	0.854959754	0.624799083	0.869334644	0.356775779
Thermocrinis_ruber	NA	-1.08532734	NA	NA	NA	NA	0	0	6.15711649	0	6.815921204	0
Antarcticibacterium_sp._PAMC_28998	NA	-1.073492501	-0.57415366	2.685392807	0.756069184	1.849586982	1.353755004	2.259508761	1.802876821	-0.515166956	-0.298154447	-0.045674111
Maribacter_sp._T28	NA	-1.071261375	-2.363451244	NA	2.409156577	2.233931614	1.4324932	2.205391248	0.307917854	1.539899026	0.221853664	0.227227911
Sphingobacterium_thalpophilum	NA	-1.060737633	0.188043541	-3.085806207	-0.008611094	0.652602083	1.211975731	-1.873012683	0.969079433	0.25308557	-0.006136503	0.29281002
Thiomicrospira_cyclica	NA	-1.058563892	-1.796511246	1.395704205	1.364782819	0.745915918	0.231561528	-0.680685254	1.227543181	0.200581981	0.87307969	0.4833216
Micromonospora_rifamycinica	NA	-1.058005603	-0.106915204	NA	1.386387295	5.060189911	0	9.426646347	8.863904389	-9.665335917	-0.630812042	1.771375625
Streptomyces_xiamenensis	NA	-1.051377359	1.222692826	3.350740043	1.858621387	0.895702039	-2.706222505	-0.044725167	-1.789505194	-8.202941691	2.329735664	0.318003008
Halomonas_hydrothermalis	NA	-1.049724038	0.193087736	0.543847592	0.11807632	0.962459019	-0.377057946	0.078084155	0.439897376	-2.186475311	-0.785614818	-0.225125986
Lysobacter_enzymogenes	NA	-1.042572716	0.553935605	NA	NA	1.697271317	0.538078589	-0.149417164	-1.708188992	3.124561892	1.445497128	2.792551626
Paraglaciecola_mesophila	NA	-1.030876542	-2.994821827	NA	NA	NA	0.869130746	1.012289271	2.179001783	0	0	0
Bacillus_dafuensis	NA	-1.026204401	-2.207892852	-1.779455046	-3.360932173	-1.707025634	0	9.426646347	10.37177664	0.342724105	0.993326296	0.22646033
Sphingosinithalassobacter_sp._zrk23	NA	-1.026060666	-0.288585151	NA	1.341862022	2.043973885	0	8.382131157	6.15711649	-0.738828983	-0.877435953	0.792665783
Campylobacter_corcagiensis	NA	-1.019790366	-1.206753298	NA	1.520767298	NA	-0.042363132	-0.427480319	0.328126716	-10.5635109	-0.330694279	-1.372039549
Tuwongella_immobilis	NA	-1.010812762	-0.288129737	NA	1.266599461	1.213484186	0.001834662	-0.398089591	0.119718051	-0.984621899	0.074015431	0.016717929
Mesoplasma_lactucae	NA	-1.003531691	0.569595673	NA	NA	-1.072095145	0	0	9.854019031	8.6794801	8.6794801	8.520944699
Hydrogenophaga_sp._PBL-H3	NA	-0.998230907	-0.992232167	4.673683909	2.809799133	3.201913029	-1.713353323	-1.215266614	-0.23867254	-0.33667484	0.82997136	-0.623257074
Filimonas_lacunae	NA	-0.996076405	0.406299238	0.723967596	2.366111605	2.394925143	0.956806951	1.460701981	1.445559538	0.61083704	0.082282754	0.632291363
Anabaena_sp._90	NA	-0.991132762	-1.928848484	-2.420525125	-0.135122906	1.525703619	0.873936482	0.255184953	-0.047542697	0.638413336	2.923978315	2.056452429
Mycolicibacterium_moriokaense	NA	-0.99073685	-0.257070666	2.239321492	-0.950881039	-2.075308521	0.616172895	1.870835719	-0.265982329	-0.437320222	0.597594687	-0.332520693
Micromonospora_echinofusca	NA	-0.965234582	1.685670621	NA	NA	NA	-0.442907316	1.559789987	-0.109762685	-8.087462841	1.019317305	-0.54830403
Ehrlichia_ruminantium	NA	-0.960588927	1.212439062	-5.06045721	-0.259843139	0.253846205	-1.152018674	-0.164431655	0.636520906	0.007193953	0.590206948	0.95771353
Paracoccus_sp._SC2-6	NA	-0.942434712	-2.838326309	NA	0.55366637	NA	0.402739082	0.791503871	1.742893033	0.27229372	-0.254695625	-1.569365646
Beijerinckia_indica	NA	-0.940698212	0.230880863	-0.556170234	1.431225652	1.114796219	0.58946987	0.816359589	0.888947415	1.452047221	0.222970223	0.4160485
Micromonospora_chokoriensis	NA	-0.938323103	NA	NA	NA	NA	0.446508891	-0.135975098	1.275344174	-7.700439718	0.515496064	-7.700439718
Komagataeibacter_xylinus	NA	-0.934346761	-4.228636175	-1.427082841	1.632690272	1.312738923	2.652958074	2.472179656	0.404067483	-0.902313791	2.259974606	2.038802208
Sanguibacter_keddieii	NA	-0.931889504	-1.95070169	0.870877798	-0.012058417	1.87462506	-1.219938331	0.12275936	0.583674152	-2.418893483	-0.424921582	-1.806709514
Mycoplasma_bovirhinis	NA	-0.93021167	1.034700322	-6.836473777	-2.829070403	-1.390701863	-1.095850134	-12.12576538	-0.394060271	0.055565788	0.453800123	0.42430551
Sphingobium_chlorophenolicum	NA	-0.93021167	1.913325589	NA	NA	NA	0.384289208	-0.047229722	0.565160525	0	0	0
Formosa_sp._Hel1_33_131	NA	-0.93021167	NA	0.597901556	-2.515079648	6.773280988	0.253600133	-0.951741706	0.90361669	0.490959881	0.072591172	-0.777368279
Mycoplasma_mycoides	NA	-0.93021167	NA	NA	NA	-2.313944899	-8.076815597	1.307281711	1.484386414	-0.427774315	0.319463437	0.178738646
Brevibacterium_siliguriense	NA	-0.927131548	-0.453540987	1.671674134	0.980990474	1.164714145	0.24728692	1.276453975	1.744195723	-0.222179144	1.594132183	0.315631033
Aquimarina_sp._AD10	NA	-0.924601232	-0.585428864	NA	NA	-0.457320225	0.484849138	1.127103552	-0.658049729	1.793323308	1.711777168	1.547524417
Arsenophonus_nasoniae	NA	-0.921152525	-2.026762713	NA	0.759062185	2.128620166	0.324557219	-0.195398314	0.402498099	-0.427188107	1.956591495	-8.727920455
Pseudomonas_azotoformans	NA	-0.911015109	-0.779963	NA	2.806582808	1.187231582	0.058801837	-0.087746544	-0.283498756	8.389450655	10.95758818	10.36632221
Actinomyces_sp._2129	NA	-0.908956437	0.333598484	-3.289855389	-0.089000251	2.234929425	0.763271533	-8.552496194	1.071551681	-0.210477326	-0.099726162	0.501236599

Labrenzia_sp._VG12	NA	-0.901352412	-0.610941567	1.200480957	-1.297522782	-2.941378972	-0.869530559	-9.372270887	2.612407461	-0.703605525	-0.368781908	1.987286668
Sedimentisphaera_cyanobacteriorum	NA	-0.899327302	2.465451098	-1.186204578	NA	0.26333086	-1.577431637	0.043442471	-0.643213907	0.947169088	1.235862247	0.465997662
Streptomyces_davaonensis	NA	-0.893413092	2.181513268	NA	-1.201633861	-0.900543229	0	0	0	-0.163169438	0.564414094	1.194709013
Pseudomonas_xanthomarina	NA	-0.876396566	0.538640933	NA	NA	0.717381198	0	11.24295971	10.3948485	-1.309508836	-1.543974089	-1.688366232
Owenweeksia_hongkongensis	NA	-0.870641604	-0.508248177	NA	0.164936907	0.495672278	-1.7326008	-1.502796924	-0.674070705	0.353095407	0.49816039	0.513825783
Campylobacter_rectus	NA	-0.870606478	-1.504112831	NA	NA	NA	-0.476429563	-0.291437012	0.736441264	0	0	0
Streptomyces_phaeolivaceus	NA	-0.862850035	-0.925357931	NA	0.021247734	1.783304309	0	0	6.15711649	10.09714987	0	10.15059775
Enterobacter_sp._JUb54	NA	-0.853479603	2.0707841	NA	NA	1.699192252	-0.919443854	-0.84597236	1.603186376	-0.442175869	1.607190208	1.841507765
Arcobacter_ellisii	NA	-0.853479603	NA	NA	NA	-2.302154422	0.509239491	0.459377081	1.94769792	0.950615922	1.047082511	-0.9371194
Kocuria_flava	NA	-0.815250892	-0.280326063	2.042513427	-0.645542566	2.711803648	-0.125621364	0.080251487	1.824582189	-0.470109662	1.334841033	0.850698082
Dolichospermum_compactum	NA	-0.814734452	NA	NA	NA	NA	0.503288398	0.52277887	0.489132026	0	6.815921204	0
Rhodobacter_capsulatus	NA	-0.790176252	0.946892388	0.288251361	-2.899400266	-0.307387932	-0.407799879	0.775845638	-1.016178612	-0.854529937	0.603529704	0.744068725
Thermoanaerobacter_siderophilus	NA	-0.771761321	1.986265889	NA	NA	0.427440581	9.292949655	0	11.36482956	0.969243708	1.412094299	1.188806304
Pseudolabrys_taiwanensis	NA	-0.768384169	-0.272056194	NA	-1.766916804	-0.554211176	8.876516947	8.566400396	9.875121155	0.526545814	0.86150328	1.637429921
Mycoplasma_mobile	NA	-0.765897886	-1.897240426	NA	NA	NA	-9.076815597	-9.076815597	-1.919701129	9.764871591	7.222389201	0
Halobacteriovorax_marinus	NA	-0.76366645	-1.138091539	NA	NA	NA	0.314133034	0.492209044	0.673696156	10.23601419	9.866248096	8.89683794
Cellulomonas_fimi	NA	-0.75802721	NA	NA	NA	NA	0	0	0	7.303780748	0	0
Brachybacterium_vulturis	NA	-0.747563068	-0.692542388	1.063862062	1.435964983	0.050519694	8.166278594	0	8.548995253	0.357017657	0.031881466	0.511403215
Mycolicibacterium_madagascariense	NA	-0.74139784	1.463449151	2.106638757	-0.44633363	2.551027454	8.502740328	0	10.63811005	-0.320807745	0.161408537	2.652995708
Leptospira_interrogans	NA	-0.74139784	2.882913051	NA	NA	-0.555127271	-4.366705739	-2.887158758	-3.322760279	-0.443179051	0.706986715	1.293786543
Streptomyces_rimosus	NA	-0.731865474	0.992989853	NA	0.334287337	0.736774623	-8.076815597	-8.076815597	-1.919699107	0.050353029	-0.359602166	0.805388709
Serratia_sp._FDAARGOS_506	NA	-0.727315424	0.105912023	NA	NA	NA	0	9.426646347	8.157119522	0	0	9.423466822
Sphingomonas_sp._HDW15B	NA	-0.719793457	0.690497969	NA	NA	NA	-1.575643265	-0.415400257	-0.356175409	1.272460606	1.078856488	1.13529247
Lysobacter_sp._CW239	NA	-0.708507567	-0.458263242	1.173323794	-0.318208209	-0.326487687	-9.334172707	-0.95204155	-0.00917314	-2.594092944	1.188016027	0.130452872
Mannheimia_sp._ZY190616	NA	-0.707562652	-2.463656204	-0.501477515	-0.321928095	2.553935605	-0.397482308	0.198896418	0.178415078	-2.170956497	-0.026144727	-1.929377743
Chryseobacterium_antarcticum	NA	-0.693896872	-3.5334322	NA	NA	-1.258068153	9.129283017	0	0	-0.565597176	-1.353463668	-10.11634396
Lysobacter_oculi	NA	-0.693896872	-1.631612594	3.112496721	1.807354922	1.930621963	1.089286287	2.255837424	0.694723111	8.366322214	8.033423002	6.544320001
Microbacterium_sp._Y-01	NA	-0.693896872	-1.300233024	NA	3.204333011	2.30671882	0.086912259	-0.811325351	-0.531972246	2.388677029	3.03456031	2.765746678
Planococcus_kocurii	NA	-0.687560551	-2.860053083	NA	NA	NA	-8.091313724	0.475086672	0.389569356	0	9.353146825	0
Simplicispira_suum	NA	-0.671841323	-2.356475274	NA	NA	NA	0.666421675	0.646005745	0.680760975	-2.657187003	0.327206217	0.797813442
Solimonas_sp._K1W22B-7	NA	-0.671655973	-1.601474212	1.82825718	0.922202169	1.697034606	-2.497112256	-0.607198074	-1.32323861	1.024982357	2.799109935	2.698985144
Mycoplasma_hominis	NA	-0.659466026	0.27641888	-3.33671246	-1.655934172	-0.484079144	-0.265722017	0.346687566	0.195182278	-0.067127963	-0.075262954	-0.751519649
Pseudoalteromonas_luteoviolacea	NA	-0.647698256	4.52044454	-1.698616983	-2.27880573	0.091986575	0	6.682671608	0	0.572970752	0.635022065	1.148338551
Methylibium_sp._Pch-M	NA	-0.647698256	NA	NA	-0.890952471	NA	0	0	7.157114468	8.442943496	7.996236198	0
Nautilia_profundicola	NA	-0.646670421	0.251654435	1.230414217	-2.309534238	-3.294917142	0	0	0	0.073640986	1.545453749	-0.403089499
Paraburkholderia_megapolitana	NA	-0.617593505	0.604344214	NA	NA	NA	-0.295014752	-0.412194347	1.111539824	9.99905574	8.297682077	7.539158811
Intrasporangium_calvum	NA	-0.615287038	0.416191194	-0.408368906	NA	-1.009529763	-1.532868935	-0.875386397	-0.751019543	1.949074408	-0.395706825	-8.849664685
Spiribacter_salinus	NA	-0.615287038	1.860128514	NA	1.882733948	1.765376997	0	0	8.904155902	-0.813708875	-9.493188974	1.413198093
Synechococcus_sp._CC9902	NA	-0.613662813	0.342186934	NA	1.553946541	1.558308628	0	10.47352312	0	-0.476288536	-1.624078448	-0.611385333
Lactobacillus_hilgardii	NA	-0.594105913	-2.365549316	1.465927273	-0.450939546	-0.171055265	0	0	8.451211112	0.900058562	1.344168673	1.50916715
Sulfurimonas_gotlandica	NA	-0.593786351	3.663983244	-3.805139084	-1.110573093	0.036493537	0	0	6.15711649	-0.52672057	0.552935861	0.598334047
Acidiphilium_cryptum	NA	-0.587317922	1.550828371	-1.208670186	-1.750819603	0.306047828	-0.14690576	0.153044988	0.301048413	2.636966353	2.978914706	1.724026538
Candidatus_Methylopumilus_planktonicus	NA	-0.57905311	-2.330837815	1.884241857	1.647985103	0.793030447	0	0	0	-1.028617458	0.272130415	1.104938882
Coxiella_endosymbiont_of_Amblyomma_sculptum	NA	-0.522601039	0.1631325	NA	1.9644704	-0.044579898	-9.177646265	-1.067220377	1.287552787	0.10709105	0.587747627	0.887901012
Aquicella_siphonis	NA	-0.51277168	1.183414752	NA	NA	NA	1.295455485	-3.705345677	-1.645936947	-1.035919574	-8.930737338	-0.518518946
Altererythrobacter_ishigakiensis	NA	-0.51277168	2.467439197	-1.767080925	-0.225338953	-1.573966296	-0.307807032	-0.472600427	0.4267486	-0.23307889	-1.037988484	1.619264303
Aquitalea_sp._USM4	NA	-0.512450001	-0.831728517	1.672705395	-0.562287063	1.178470111	-0.094059701	0.080185106	-0.078875729	0.915607813	0.171541662	0.494764692
Herminiimonas_arsenicoxydans	NA	-0.507483748	-0.854828546	NA	0.357156583	-0.106499621	-0.105297541	-0.197928507	0.232348033	-9.994353437	-2.699117147	-9.994353437
Candidatus_Pelagibacter_sp._HIMB1321	NA	-0.49683159	0.866183696	NA	NA	-1.86016767	0.844901073	0.805091026	0.945385377	-0.284404881	-1.559631711	-0.60361298
Janibacter_indicus	NA	-0.443635694	1.820879158	NA	1.95615246	2.315792222	-0.063747326	-0.276695108	0.467974775	1.213752426	2.633655681	0.06319378
Thalassolituus_oleivorans	NA	-0.434552318	-0.253647356	1.042320938	-0.142442198	0.698820683	2.913339585	2.665515512	2.156388073	-0.960462616	-0.972681587	-0.227582924
Xylanimonas_allomyrinae	NA	-0.430994376	0.076585594	0.955778386	1.608889685	0.085729874	0.734179212	-9.880696238	1.205804928	-1.882910635	-4.138239303	-3.069400969
Colwellia_sp._MT41	NA	-0.410892672	-1.83066809	NA	-1.67421025	NA	-0.745107449	1.028953859	0.752581058	9.919856983	9.723375961	9.661778098
Altererythrobacter_sp._B11	NA	-0.370837695	NA	NA	NA	NA	-0.937420022	-0.636241567	0.645257836	8.27301694	0	7.539158811
Saccharopolyspora_spinosa	NA	-0.359730128	NA	NA	NA	NA	-1.138901444	0.953247964	0.989283565	0	0	0
Lacunisphaera_limnophila	NA	-0.352665849	0.140058286	-2.516442122	1.416171038	1.135756008	0.577645509	1.123347351	0.5884626	0.014580096	0.41413018	-0.38920289
Dermabacter_jinjuensis	NA	-0.341595128	-0.926557629	NA	-1.231403663	0.423546357	0.149463285	1.410886052	1.817271652	7.129283017	0	6.544320001
Citrobacter_sp._SNU_WT2	NA	-0.340575255	1.524615535	NA	-1.911463325	0.389339345	0.069333807	-0.664848081	1.822977038	-0.014164229	-0.67559492	-0.892701533
Alcanivorax_pacificus	NA	-0.334026237	0.765678163	NA	1.296981738	1.102738106	0.310380291	-0.446251527	0.128841231	-0.078894854	0.890502817	1.483046116
Microbacterium_protaetiae	NA	-0.332762836	-0.416527117	NA	-2	-1.556765609	-2.486736667	0.161154059	-1.502911417	1.503192306	1.35389015	0.110009918
Thermomicrobium_roseum	NA	-0.330751945	-0.487680598	1.9286714	0.142916268	-1.396742871	0.700248505	0.439291123	-0.148419655	0.542460445	-1.500932394	0.934150934
Bacillus_sp._M4U3P1	NA	-0.327491704	-0.301188422	-0.105462095	-0.46664962	0.923296898	-0.091763014	0.256061707	-0.101845787	-0.151099339	1.261692838	2.237116496
Bathymodiolus_septemdierum_thioautotrophic_gill_symbiont	NA	-0.316607763	-3.273018494	NA	NA	NA	0.371966353	-7.757316664	2.353101393	-0.793969653	-1.259242827	-9.247927513
Corynebacterium_aurimucosum	NA	-0.316607763	-0.264456481	-1.089846617	1.888628389	-2.889977213	-8.637284195	-8.637284195	-8.637284195	-0.963324516	0.238225212	0.676065722
Halomonas_sp._HL-93	NA	-0.316607763	2.269829132	-1.273625051	1.948138882	1.320836109	0.387894569	-0.635842769	-1.228663307	0.433901211	-0.175249294	-9.569855608
Dichelobacter_nodosus	NA	-0.313416595	NA	-1.879187667	NA	-2.5477596	0.42742393	0.396310083	0.696943048	0.529953288	1.772366713	1.740054163
Mannheimia_sp._USDA-ARS-USMARC-1261	NA	-0.299118212	0.460998346	NA	NA	NA	-0.687229928	-0.535350431	-0.087210754	1.22718864	2.065272323	1.344875579
Advenella_kashmirensis	NA	-0.293358943	NA	NA	0.993942489	2.429821651	-0.619991794	0.517225144	0.30724326	1.143224548	-0.27571064	0.918886224
Sphingomonas_wittichii	NA	-0.291516782	0.137832193	NA	NA	NA	0	0	8.548995253	-1.514267709	-8.818048457	-8.818048457
Mycoplasma_pulmonis	NA	-0.291112511	-1.241172257	NA	1.490156979	3.371158259	-0.159687981	0.120332446	-1.311462103	-9.742590705	-1.189026684	-0.466466299
Lysobacter_sp._HDW10	NA	-0.284064894	2.104593947	NA	NA	1.450301848	1.266356564	3.547643674	0.78534489	0.344413831	0.867142522	1.101967424
Microbulbifer_sp._GL-2	NA	-0.28144343	2.386440031	NA	NA	NA	-0.878620597	-0.311730502	-0.419236055	2.294785159	0.25027958	1.560863826
Mycoplasma_phocirhinis	NA	-0.281166765	NA	NA	-0.653609848	0.716440197	0.333334288	-0.019492295	-0.646642489	0.710688857	1.836549826	-0.161155448
Marinobacter_sp._LQ44	NA	-0.276749689	2.479615444	1.502146695	-0.107069346	NA	-10.75807327	-0.004555646	-1.207326488	-1.241362614	-0.600052976	-0.300964668
Mycoplasma_sp._NEAQ87857	NA	-0.276493621	1.20312631	-2.352288144	-1.820518582	-0.232215155	-1.161585039	0.461230361	-0.523697474	-0.156484264	-0.027792317	0.779168707
Synechococcus_sp._MEDNS5	NA	-0.271858256	-1.833990049	-3.780293026	-1.304955016	-1.191426071	0	0	0	8.45395786	5.472487771	7.539158811
Pantoea_sp._CCBC3-3-1	NA	-0.270016006	-0.264456481	NA	3.266094206	2.382394108	-0.46739464	1.45530723	2.16609278	0.068171317	0.763643887	0.446381772
Synechococcus_sp._PCC_7003	NA	-0.267644226	2.338385038	-3.644538961	-0.185495424	-4.467989569	-0.866435612	-0.721453069	-0.454461688	0.472428583	-0.581735098	-0.36460385
Pelistega_sp._NLN63	NA	-0.265079332	-0.340696378	6.111082427	0.224493944	2.956884808	-0.140021186	-0.064225862	0.093998946	-0.540648939	2.008283439	0.937275706
Pseudoalteromonas_agarivorans	NA	-0.259341813	-1.27715309	3.115477217	2.068587085	2.669774205	-3.384290388	-2.647826086	-1.361880752	-1.234188849	0.364590682	1.029075854
Gallionella_capsiferriformans	NA	-0.258185006	-2.271804615	-0.949891152	0.939512017	0.743847695	0.661769587	0.336540055	0.39436761	-0.692398735	0.106306781	-1.290451466
Singulisphaera_acidiphila	NA	-0.244168935	0.086440976	-0.030057234	0.476902755	1.445805458	0.450382254	-1.061158757	1.899509834	-1.655962955	-0.304570425	0.765968568
Polaribacter_reichenbachii	NA	-0.230258267	2.258123281	NA	-0.452301551	0.829324293	0	0	6.15711649	-0.578341174	-1.167026761	-1.225999088
Methylocystis_bryophila	NA	-0.213992226	0.594597939	NA	-0.957738776	0.354158954	-0.681714286	-10.3402507	0.546695675	-0.968319735	-1.127470179	0.81040561
Pseudomonas_otitidis	NA	-0.210217707	-1.542793046	0.058245141	NA	1.906530327	9.029913869	9.627367901	10.04241178	-7.741466986	-0.925545783	1.731562912
Nocardia_brasiliensis	NA	-0.210217707	0.21801929	3.635030236	0.843227662	1.715088174	-8.552496194	-8.552496194	-1.395381726	-0.773299203	-1.420206702	0.118176341
Streptococcus_uberis	NA	-0.208586622	-2.786695451	NA	-3.215889476	-0.164710752	0.480522394	-2.014630669	1.22178011	-0.463584264	0.010695235	-0.222766444
[Arcobacter]_porcinus	NA	-0.189866048	-1.634482637	NA	-1.587371007	1.450984196	0.459386584	0.282034706	0.403735374	1.302004063	0.726208941	2.272663622
Vibrio_sp._ZWAL4003	NA	-0.18508064	-0.42932345	NA	0.275365208	2.387887898	-0.644854595	0.448129205	-2.901632363	-10.63843591	0.780823808	-0.711646255
Streptococcus_lutetiensis	NA	-0.172272098	NA	-0.926926998	-1.657865404	0.731546288	-8.624046047	1.071340602	2.962962577	0.238958448	0.367880571	0.220148167
Pseudomonas_yamanorum	NA	-0.160464672	-1.111790825	NA	1.164179693	2.220387731	-0.109336598	-0.637552141	-1.167275626	-0.245150949	1.045472044	0.047446221
Streptomyces_sp._SirexAA-E	NA	-0.160464672	1.018859027	1.035189428	NA	NA	0.737104769	-0.530971162	0.637184604	0	0	7.380824669
Leminorella_richardii	NA	-0.157797952	1.743224585	NA	NA	NA	0.051446682	-8.10567284	1.857486238	10.35461304	10.0835724	10.36632221
Campylobacter_armoricus	NA	-0.157297393	-2.19745452	NA	NA	NA	-1.252225897	-0.848194721	-1.106160806	9.366322214	5.472487771	7.539158811
Oceanobacillus_kimchii	NA	-0.15465657	1.433607293	NA	0.799185247	1.068171503	-0.227671071	0.131201038	0.6744304	1.519031886	2.263481507	1.622114619
Myxococcus_hansupus	NA	-0.131705679	-0.15307933	NA	0.759972159	1.999325686	8.876516947	0	0	-1.414339489	1.195295961	0.29256491
Chlamydia_gallinacea	NA	-0.129807347	-2.027093661	NA	NA	NA	0.060605754	2.24867573	1.816168097	0	0	0
Methylotenera_mobilis	NA	-0.122436076	-2.507557997	2.691214018	1.81976429	2.266765559	0.218221871	0.244411073	0.383692547	1.158779934	2.210627882	2.562703651
Phyllobacterium_sp._628	NA	-0.122147109	-2.337987946	NA	NA	NA	-11.04364935	-11.04364935	-2.243043846	-1.355827493	-0.746720927	-0.5767084
Streptomyces_sp._SCUT-3	NA	-0.097884653	-1.862034	NA	1.349158546	1.535091424	0	0	0	2.525425521	2.355606955	0.890290279
Microbulbifer_sp._YPW1	NA	-0.095446785	-2.102268051	0.713295549	0.126792531	1.232096685	1.976472596	-0.382515681	-1.784866258	1.426700417	-9.266786541	0.411519572
Sphingomonas_koreensis	NA	-0.077979213	0.1302413	NA	NA	NA	10.62985747	9.252665432	12.55620187	0.749296396	0.263378355	0.423272298
Microbacterium_sp._ST-M6	NA	-0.073336612	-2.702333709	NA	NA	NA	0	0	12.78061358	-7.807354922	1.166104247	-1.263034921
Mesoplasma_chauliocola	NA	-0.073336612	-2.589174752	NA	NA	NA	-1.20618359	-0.008689406	-2.149571498	-7.709655951	1.756589809	-7.709655951
Sphingomonas_lacunae	NA	-0.073336612	-0.700965577	NA	NA	NA	-0.471068556	-1.267989748	0.347697149	0	10.32942637	0
Pseudomonas_sp._UW4	NA	-0.069741071	3.967127149	0.736756276	0.741056147	2.218275245	0.263204432	0.12546061	0.412239594	1.638751777	2.288495113	1.036062623
Mumia_sp._ZJ1417	NA	-0.046592694	0.382971997	3.572081209	NA	-0.596550475	-0.768255329	-9.177646265	-0.726435153	0.884522783	-0.098048908	1.390559223
Sinimarinibacterium_sp._NLF-5-8	NA	-0.044947406	0.529322758	NA	NA	1.161780215	0.862063259	1.227049057	0.751001976	-1.89104775	-2.130641688	-10.16406469
Leptospira_kmetyi	NA	-0.039345636	1.140311661	NA	-2.453624923	0.075310798	-1.733989503	-1.681598274	1.061445503	-0.080085361	0.784367874	0.379969322
Halomonas_sp._Y2R2	NA	-0.026938545	0.553055934	0.454323282	-0.905208806	-2.317447672	-1.450031391	0.436402214	-0.510361464	0.586526868	0.073489019	0.126253562
Planococcus_sp._MB-3u-03	NA	-0.017921908	-0.502747737	0.6559609	-1.745168125	-0.044728407	-0.524481103	0.837504541	0.641207848	-0.027986496	1.211425203	0.395152905

Actinobacillus_indolicus	NA	-0.010344336	1.395496368	6.366513765	-1.81576234	0.182785526	-0.342584857	-0.704264769	-0.712384105	-0.629193223	-0.636169042	-0.277406034
Luteimonas_yindakuii	NA	0.002839952	0.66109357	NA	NA	0.813481025	0	0	8.157119522	1.002230988	-2.280108493	2.354169783
Janibacter_limosus	NA	0.007089425	-0.040723208	2.908522472	1.158867819	-0.361237797	-0.14113302	0.237661069	0.033451916	0	10.87344411	10.33390446
Rhodovulum_sulfidophilum	NA	0.007089425	1.534336428	0.814360528	-0.980891177	-0.657276706	1.272595019	0.836501668	2.309751234	-0.33481277	0.674672439	-2.97012947
Corynebacterium_vitaeruminis	NA	0.007122516	1.072038959	2.963536715	3.106624567	3.288757446	-1.314126238	-0.656858775	-1.194521901	-0.626415045	-0.154394876	0.161668723
Pseudomonas_rhodesiae	NA	0.019310754	0.261328084	NA	NA	NA	0	0	0	0	0	0
Staphylococcus_pettenkoferi	NA	0.028178533	-0.165662975	NA	-2.980291318	-0.70234092	0	0	0	1.926908835	2.408429603	2.878823142
Methylovorus_glucosotrophus	NA	0.032180406	2.349859028	NA	NA	NA	-0.868838784	-3.641193484	-4.006482903	-2.159291219	-0.022056397	0.734823987
Jonesia_denitrificans	NA	0.034031629	-1.481806511	3.499324346	0.155081665	0.989367024	-0.557888051	-0.202979409	0.05277273	1.13504996	0.406923159	2.858445856
Chryseobacterium_haifense	NA	0.0415335	-2.919992352	NA	NA	0.646949388	9.564669681	10.35434857	8.624046047	-8.750428971	-1.934507767	-8.750428971
Lentilitoribacter_sp._Alg239-R112	NA	0.045524739	2.216759495	NA	NA	-2.852235364	-0.851584153	0.634487534	0.393782986	0.500795131	1.04312099	0.239751972
Pseudomonas_syringae_group_genomosp._3	NA	0.0489096	1.662965013	NA	NA	NA	-0.504927762	-2.429454811	-0.289419673	-8.048034513	-8.048034513	-8.048034513
Staphylococcus_schweitzeri	NA	0.0489096	NA	NA	NA	-1.913075056	2.103606842	2.106036645	0.602321915	1.009418902	-0.667583046	0.04124034
Entomoplasma_luminosum	NA	0.067227255	NA	1.29339319	0.742946571	2.63888167	-0.313288968	-1.381050579	1.003590847	0.039698399	0.141738208	-0.018644692
Sphingomonas_sp._AAP5	NA	0.075773472	1.000534233	-2.616614951	-1.073777926	0.257382312	-13.06935746	-0.761846393	-0.547087349	-0.980745443	-0.628106745	-1.692729238
Pasteurella_skyensis	NA	0.092399242	2.164867345	2.04929075	3.761648522	2.695559332	1.744034667	2.366256547	3.000478028	-1.543426855	-0.623906334	-1.288037403
Gemella_morbillorum	NA	0.107148355	1.949978051	-2.683161727	-0.212841792	-0.244624445	8.157119522	0	0	0.003975515	0.572021549	0.579819159
Pseudomonas_versuta	NA	0.129559634	-2.386278506	NA	NA	NA	0	0	6.15711649	0	0	0
Shewanella_loihica	NA	0.129559634	-0.915387772	-0.122606985	3.632167204	3.132081206	-1.06847303	-0.602223001	0.064645471	1.507384103	1.37603937	0.387422619
Streptomyces_sp._ZFG47	NA	0.136861517	5.000073545	1.003012519	2.643534483	2.97152433	-0.168968809	-0.169123994	-0.207197505	1.963179546	1.910548568	1.526383713
Arcobacter_halophilus	NA	0.143124942	NA	NA	NA	-3.189186904	-0.206605174	0.490213362	0.470103733	-0.445963754	-1.674782444	-2.720586134
Synechococcus_sp._PROS-9-1	NA	0.149513541	-0.402197154	NA	0.336063616	0.95491211	-0.018556781	1.032499126	-0.573333704	-0.491289154	-0.515766993	-1.306004272
Acholeplasma_oculi	NA	0.152673958	1.019115315	-0.20755148	1.22351245	1.877778774	0.406794891	1.273951803	1.16150301	0.457369338	0.787887656	0.272820848
Piscirickettsia_salmonis	NA	0.159304664	0.617752436	NA	NA	NA	8.409390936	8.110425888	9.540215237	1.255614927	-0.503189265	0.895558075
Streptomyces_sp._DSM_40868	NA	0.164386818	-1.723212439	NA	2.37601561	1.470685529	0	0	0	-7.741466986	-7.741466986	1.210787943
Nostoc_piscinale	NA	0.164386818	-1.723212439	NA	NA	NA	0	0	7.157114468	0	0	6.544320001
Bremerella_volcania	NA	0.164386818	1.615048227	1.580226803	1.006066001	-1.144816553	-0.58793256	-0.05859733	-0.980331835	-0.121019889	0.001202246	-3.106127351
Thermosipho_africanus	NA	0.166133422	-1.819360934	NA	-1.388807439	1.556738568	0.430504348	0.404141043	0.516290729	0.520574809	0.639711643	-0.246482851
Methylovorus_sp._MP688	NA	0.169730686	-3.646591157	NA	NA	NA	0	8.110425888	0	0.289506617	0.902372345	0.444129102
Streptomyces_griseochromogenes	NA	0.171930137	-1.633941294	0.447681856	0.451354395	1.107094306	0	9.426646347	6.15711649	0.546143252	0.405430975	2.379493382
Chitinibacter_fontanus	NA	0.178414724	0.809099127	1.220271778	-0.03128267	-0.406791805	0.139539576	-0.019850988	0.520192084	-1.35366846	0.131580272	0.035445733
endosymbiont_of_Acanthamoeba_sp._UWC8	NA	0.190368391	-0.092227292	NA	-1.427637536	-0.057587492	-1.120418918	-1.032960244	-0.75868612	2.967039288	-0.925545783	0.797691825
Campylobacter_insulaenigrae	NA	0.202018631	2.084857287	NA	0.104133873	-3.493362981	-0.356823547	0.321247437	1.125124465	0.813262928	-0.013334083	0.286177486
Mucilaginibacter_mallensis	NA	0.246946935	1.522003869	0.405653473	2.159243268	1.619466589	-2.486736667	-2.961177559	0.352426622	-0.458144931	0.888768951	0.580286419
Actinomadura_sp._WMMB_499	NA	0.248591483	-2.118284018	-1.906890596	NA	NA	0	8.627369725	9.793234257	1.310568946	2.684356727	0.654004145
Synechococcus_sp._PCC_11901	NA	0.250360576	NA	NA	NA	1.455027231	-10.28455809	-0.401082367	0.5263027	8.033423002	0	0
Methylophaga_frappieri	NA	0.250360576	NA	NA	NA	NA	0.489179237	1.526433568	0.148725867	-9.247927513	-9.247927513	-9.247927513
Mycobacterium_dioxanotrophicus	NA	0.280487293	0.969580604	1.298081353	NA	-0.356216584	-0.056182634	-0.376610131	0.523692169	0	10.50514661	6.544320001
Coprothermobacter_proteolyticus	NA	0.295143831	1.925445069	-2.140217179	0.234513665	1.478146601	0.706055165	0.760051262	1.116502109	-0.197591467	0.285727288	-2.293569522
Aquabacterium_olei	NA	0.29789159	0.855095051	2.938063237	2.824283361	2.749549547	0.016623419	1.208881129	2.015842356	1.586351711	1.152839906	0.416079012
Cupriavidus_pauculus	NA	0.305541906	0.187350304	-2.649942532	3.678714154	3.772224819	0	11.76298407	11.79434275	-0.539674162	1.221728073	2.254149551
Actinomadura_verrucosospora	NA	0.307221596	0.036123446	2.238646321	3.336447564	-0.605000254	-0.46787729	0.586738252	1.08917631	1.711716154	0.912880808	-1.140578619
Methylovirgula_ligni	NA	0.314873337	2.369517553	NA	NA	NA	-0.631802755	-0.373638978	0.528048853	0	9.568588898	9.994353437
Mycoplasma_arthritidis	NA	0.319717068	-1.274123584	NA	0.156429159	-0.529619467	0	0	0	0	0	0
Arthrobacter_sp._U41	NA	0.323697188	-0.96761786	NA	0.328948523	0.292063454	0.131684976	0.17782536	0.739561646	-0.057242301	1.817231501	1.318625263
Komagataeibacter_saccharivorans	NA	0.323697188	4.151366795	NA	-1.347138658	-1.183392231	-0.307833255	-0.354715238	-2.846158636	1.341458115	-0.171496914	1.375090537
Phycicoccus_endophyticus	NA	0.348684553	2.018535951	NA	NA	NA	1.114206799	1.125028963	1.189638523	9.437404619	10.14940418	8.398029648
Gilliamella_apicola	NA	0.402575815	1.48181708	-1.778584683	2.150806049	-0.149346545	0	0	0	-1.425504498	-0.36784393	0.601095041
Fusobacterium_sp._oral_taxon_203	NA	0.434390926	NA	1.902376837	2.782408565	2.948387257	0	0	7.745136469	1.799233488	0.172959062	0.435392781
Serratia_grimesii	NA	0.453256822	1.302431919	NA	NA	-3.837373602	0.954172468	0.694505867	1.036992633	-0.564821608	1.678604331	1.484529534
Ephemeroptericola_cinctiostellae	NA	0.454604584	-0.63155206	NA	0.665723513	0.19018384	-0.690714114	1.041405239	0.293505289	0.866014036	1.330063383	-0.431198513
Brevundimonas_diminuta	NA	0.459172909	NA	NA	NA	NA	-0.236507456	0.774968038	1.117871798	0	0	0
Sulfitobacter_sp._JL08	NA	0.470183418	-0.900619513	NA	0.866646964	1.039673308	0.05246742	-1.394143989	0.642107653	2.420367616	2.471360903	1.791493051
Francisella_sp._FSC1006	NA	0.516689769	0.048595766	3.260767247	1.637594003	2.791957817	-9.671461455	-1.988782825	-9.671461455	0.98838837	1.740022214	0.994208428
Amylolactobacillus_amylophilus	NA	0.518550585	2.644210182	-0.021721203	-1.768642516	0.61652138	-0.149172861	-0.048826882	-0.837161822	1.542624427	1.195640838	1.941343088
Bosea_sp._PAMC_26642	NA	0.53287399	-1.425305835	NA	NA	NA	0	0	7.745136469	0	7.811644421	0
Lacinutrix_sp._Bg11-31	NA	0.570735076	-0.761840263	NA	NA	1.242043509	-1.390409646	0.324511161	0.209269336	1.960190025	2.683599296	2.483102013
Nordella_sp._HKS_07	NA	0.62568524	1.305230469	NA	3.482128174	3.852208511	0.93971214	0.302614579	1.3304619	0.01272761	1.010430339	0.906567904
Wolbachia_endosymbiont_of_Folsomia_candida	NA	0.635599089	-1.203006767	NA	2.594834791	3.740203968	-10.20011392	0.20541051	1.686481952	1.934788105	2.903973112	1.327746147
Variovorax_sp._PBL-H6	NA	0.638140433	2.596935142	NA	NA	NA	-0.154539179	2.19121947	0.77286286	0	0	0
Hylemonella_gracilis	NA	0.645516687	0.703971305	NA	NA	NA	-0.329767009	-0.079016188	-0.071911451	8.033423002	10.29768437	10.23521247
Erythrobacter_sp._HL-111	NA	0.648670989	2.670385851	NA	NA	1.490615834	-10.6190553	0.531732825	1.675462067	-0.545031518	1.620743395	1.013786042
Salmonella_bongori	NA	0.668273555	0.122059372	3.71212408	0.513851645	-0.386468347	8.246199041	0	6.15711649	1.356206169	2.219860416	2.768548804
Bordetella_holmesii	NA	0.683740655	-1.274439169	-0.560539027	1.328948523	0.09859703	-0.430822356	-0.451214306	0.763600543	8.27301694	7.902875524	10.36048499
Lysinibacillus_sp._2017	NA	0.683936116	-0.132325609	1.360240199	1.251299296	2.80564354	-5.577109037	-2.597267698	-3.476749005	2.149711195	3.567811987	2.799742792
Campylobacter_sp._RM16704	NA	0.729074809	0.878041621	NA	0.55442256	-0.101993988	9.564669681	0	6.15711649	-9.483815777	-1.267879995	-9.483815777
Haemophilus_influenzae	NA	0.760501109	0.681330895	12.9761442	3.777360182	2.401616984	0.721620988	-10.51265159	-0.583393179	0.277482276	-0.216346057	0.466630816
Campylobacter_subantarcticus	NA	0.770102409	NA	NA	-1.447288436	-1.632643478	0.293421116	0.306259592	-0.226021115	-0.08865762	0.886862345	-1.189162431
Streptomyces_sp._SCSIO_3032	NA	0.777311063	-0.258416641	NA	NA	NA	-9.751239421	-0.498573988	1.726427789	0.404235705	0.97832148	-2.581524793
Chlamydia_pneumoniae	NA	0.7880132	NA	NA	NA	NA	-0.116078867	-1.67164278	0.39458148	8.442943496	7.222389201	8.412218392
Lysobacter_alkalisoli	NA	0.819169688	-3.280309006	0.817225166	1.122317649	-1.030022483	0.514571294	0.338456697	1.544936632	0.892522816	2.586776537	2.511658043
Citrobacter_amalonaticus	NA	0.877943277	-0.645756464	1.087328768	-0.004565494	0.378686931	0.514048899	1.042653899	1.392274647	-8.824427375	-3.351939603	-0.695144358
Thermoanaerobacter_ethanolicus	NA	0.903937278	-1.51583814	1.552811977	0.554617038	0.798432377	-0.121476381	0.556896536	0.014802053	-0.570967837	1.846251646	0.831642989
Salinivibrio_costicola	NA	0.905784658	2.49516583	NA	NA	NA	-10.91438513	-0.479183693	-0.863298574	1.007987639	0.664362705	0.413580239
Streptomyces_aquilus	NA	0.916284264	3.386310998	NA	NA	NA	0.186378477	-0.324456994	0.896856354	0.701476509	-0.372236085	-0.810729649
Rhodoluna_limnophila	NA	0.926398477	0.998102965	NA	NA	NA	-1.035069407	-2.041072541	-0.295708878	0.435584877	-2.458249566	-7.930737338
Winkia_sp._C64	NA	0.955052594	-2.51583814	-0.954348888	NA	NA	-0.17269154	0.458020023	1.733574817	9.785725451	9.833944757	6.544320001
Methyloligella_sp._GL2	NA	0.958548753	0.807610923	1.449590664	-2.206028975	-3.464010153	-0.623995024	0.453283109	-0.186646401	0.685163203	1.826790563	-1.933871088
Sphingopyxis_sp._PAMC25046	NA	0.972099425	-0.972752589	-1.155616608	2.962988216	3.358080053	0	0	0	0.018526163	-0.475833789	1.994126913
Streptomyces_dengpaensis	NA	1.005320332	-0.532450664	0.353947445	NA	-0.835906219	0	6.682671608	0	1.674044896	-3.237171625	0.736044549
Colwellia_psychrerythraea	NA	1.016311264	-0.700965577	NA	-0.204358499	0.733047308	0	0	0	0.727314296	0.13876748	0.333874183
Microbacterium_lemovicicum	NA	1.068557334	3.185806187	-2.383384298	-2.217025912	-0.427809726	-2.955277418	-0.758577649	-0.302724271	1.381532493	1.491835234	1.524635634
Pseudohalocynthiibacter_aestuariivivens	NA	1.0808444	0.923151921	3.244359541	-1.695889651	1.073609907	-0.336669522	0.151206306	-0.392738557	-1.077900933	-0.183740636	0.388244549
Citrobacter_braakii	NA	1.084450322	2.98133449	NA	NA	0.856520979	0.592679048	0.530508852	0.790150185	8.366322214	0	8.412218392
Neochlamydia_sp._S13	NA	1.097571487	1.087116414	NA	NA	0.668469065	-8.552496194	1.870335982	-8.552496194	-1.338673056	-0.235770341	0.261183794
Legionella_jordanis	NA	1.102793549	-1.426461821	NA	NA	NA	0	0	0	7.333897756	8.660591394	9.564783984
Microbacterium_esteraromaticum	NA	1.102861554	1.688106964	NA	3.075426519	3.176590442	-0.492166496	0.843780085	0.797820688	0.010690568	1.495711882	0.02688914
Pirellula_sp._SH-Sr6A	NA	1.113158957	1.341721146	0.281935942	3.490998155	3.702295477	-0.468995065	1.254653657	0.693126312	0.841316532	1.382434035	-0.241766782
Flavobacterium_kingsejongi	NA	1.122595591	0.184893664	1.514972387	1.238087404	-2.127952731	8.166278594	11.88763569	9.466123378	-0.591211241	-0.055242805	-0.845942127
Cellulosimicrobium_sp._JZ28	NA	1.132211998	0.68221596	NA	4.706764725	4.214865895	8.166278594	0	0	1.042018294	1.735813974	0.78813378
Candidatus_Hodgkinia_cicadicola	NA	1.162859098	1.973800139	3.056921832	-0.039235163	NA	-1.423045531	0.088872133	-0.443127338	8.033423002	11.00683798	7.63420844
Acinetobacter_junii	NA	1.182803793	0.370143299	NA	NA	NA	-0.660946376	-1.081554037	-0.180230086	1.721602941	0.440873992	-0.352605891
Pandoraea_oxalativorans	NA	1.198533594	1.408508881	0.054259973	3.346443632	3.644464529	-1.604981554	0.619569779	-0.385057217	-0.638828427	1.23467862	1.172533194
Rhodococcus_sp._SGAir0479	NA	1.209080677	1.586215384	-3.625287874	3.588615857	3.68541781	0.187134722	0.359632067	0.913603174	-0.243274809	0.936195613	-0.416911007
Sphingomonas_sp._C8-2	NA	1.210987658	-1.274439169	1.496937753	NA	1.62797457	0.899445622	1.183878966	1.462353641	-7.890768904	0.051745601	2.107356065
Leisingera_aquaemixtae	NA	1.229481846	-1.610053482	NA	NA	NA	0.961827244	1.497409765	1.466267101	0	0	0
Microbulbifer_sp._THAF38	NA	1.243140165	1.521578806	NA	3.300415903	3.680826899	8.246199041	8.110425888	0	-0.513978517	0.398520904	0.190856295
Silvanigrella_aquatica	NA	1.245122402	-0.770201252	NA	3.551247607	4.075567088	-0.032758948	0.161556538	0.503736556	0.558784745	1.461721602	0.691673619
Acidithiobacillus_ferrivorans	NA	1.246347737	1.745791491	-2.093910171	2.687050033	2.967756392	2.932120164	5.014032232	4.746233969	0.305345025	1.015571422	0.02715785
Ruania_sp._HY168	NA	1.248591483	0.429684275	NA	2.837120317	2.727336202	0	0	0	-1.573948137	0.584962501	0.633275308
Ehrlichia_sp._HF	NA	1.248591483	0.432803617	NA	NA	NA	9.029913869	8.110425888	8.944644719	0	0	0
Spiroplasma_sabaudiense	NA	1.248591483	1.08315133	NA	1.821204901	1.420824491	8.409390936	11.22114603	10.51891866	-1.135013591	-0.283463015	-0.919157452
Winogradskyella_sp._PG-2	NA	1.261394047	0.20049294	NA	1.979021121	1.870587425	1.296926568	-8.04736934	1.648017308	0.963929631	-0.203096939	0.732774763
Bosea_sp._F3-2	NA	1.313104245	3.189824559	2.640436413	0.091423028	2.430543739	0	0	0	-0.191854546	0.993116114	0.414587158
Thermocrinis_albus	NA	1.325794678	1.174186525	NA	1.80073672	-1.941224328	-0.306297153	2.216887121	0.759149959	1.920873912	2.458401727	-1.346448903
Sulfurospirillum_halorespirans	NA	1.361836816	0.122228259	NA	NA	NA	0.16828318	0.095154336	-0.038538051	-7.700439718	-7.700439718	-7.700439718
Candidatus_Fukatsuia_symbiotica	NA	1.36579398	2.025195315	NA	NA	NA	0.591779592	0.604368422	-0.24820265	-9.06608919	-9.06608919	-0.476127257
Pseudomonas_citronellolis	NA	1.382049087	-1.835770604	NA	NA	3.047134835	0.566808864	-0.381936089	1.248110243	8.033423002	10.31665489	10.5533125
Sphingomonas_sp._XS-10	NA	1.391296548	1.455728013	NA	3.04622631	3.525047285	0	0	7.157114468	-0.581411608	-0.048341753	0.266212767
Vibrio_owensii	NA	1.393122723	-0.678555301	NA	NA	NA	0.853899407	1.292145529	0.165355933	0	8.372138089	8.189824559
Auritidibacter_sp._NML130574	NA	1.411872137	-1.781850435	1.22787293	2.331439533	1.105399668	-0.78664906	-0.460590478	-0.846257137	-1.480207566	4.093840187	4.636023891

Cellulomonas_sp._JZ18	NA	1.419645054	NA	NA	NA	NA	0	6.682671608	7.157114468	8.27301694	0	7.539158811
Mycobacterium_branderi	NA	1.423235665	-0.610858925	NA	0.652980924	1.307930944	-0.725790568	0.115693751	-0.014011946	2.069580755	2.31511683	1.734885026
Nocardioides_sp._dk884	NA	1.451487729	0.702672417	NA	NA	NA	0.222395986	-3.492703286	0.382787488	0.409963444	0.886213465	-7.890768904
Oceanicoccus_sagamiensis	NA	1.467221806	0.984912856	-1.51121004	2.75392235	2.871562307	-0.166142889	0.538253744	-1.643297562	0.80692863	-1.169050376	0.54041508
Candidatus_Xiphinematobacter_sp._Idaho_Grape	NA	1.525202314	0.957527988	NA	3.990760332	4.27730939	10.17990909	0	10.45121111	0.270105939	0.17736956	1.360096456
Thermus_parvatiensis	NA	1.557264512	1.75093737	NA	NA	NA	1.286676595	1.190503095	1.12073366	7.333897756	5.472487771	10.56478779
Mycobacterium_sp._DL592	NA	1.559717546	1.766647452	NA	3.12180879	3.149487023	0.149848162	0.285586162	0.642037009	-1.029949008	0.851911293	-0.231774411
Pseudomonas_salegens	NA	1.571076974	1.706803624	NA	-1.096019213	-0.86094621	0	8.559465045	10.70209475	8.45395786	0	0
Legionella_longbeachae	NA	1.571076974	4.399288976	NA	NA	NA	8.876516947	8.382131157	11.69617643	0.150151372	-1.501133819	0.993821425
Geminocystis_herdmanii	NA	1.584962501	4.571497777	0.846328638	1.541291691	2.574707946	-0.152083976	0.333133306	0.64383872	0.322583152	0.316549718	0.860171423
Microbacterium_schleiferi	NA	1.586363856	0.057715498	NA	NA	NA	0	0	0	-7.709655951	0.10198847	-0.17049714
Enterobacter_sp._638	NA	1.598153952	1.736028819	NA	2.926038563	3.428351807	-8.799135887	0.849629726	-8.799135887	-0.576007711	0.892801595	0.229956023
Thiomonas_sp._X19	NA	1.624181327	1.860891269	-2.357902088	3.002404529	3.155363947	-0.200034819	-2.016099053	1.051332204	-0.640441057	0.649522184	0.279098842
Dokdonia_donghaensis	NA	1.633872101	NA	NA	NA	NA	8.246199041	0	0	0	0	0
Nostoc_sp._C052	NA	1.655884287	0.807354922	NA	-1.238203724	NA	-9.72047685	-9.72047685	-9.72047685	-0.645338315	-0.246892368	-10.5552314
Rhizobium_sp._WL3	NA	1.679385819	2.474647242	-2.095378048	3.913352846	4.38210361	0.580303087	0.836197102	0.063122286	-0.984109944	0.284858137	-0.948858089
Halomonas_alkaliphila	NA	1.679395141	2.857250414	NA	NA	NA	0.355120969	-0.219901101	0.067608684	0.956815246	1.998106995	1.749202918
Yersinia_frederiksenii	NA	1.704526676	-1.425305835	NA	NA	NA	0.405867823	-8.624046047	0.967221959	-9.247927513	1.516944077	1.076253033
Altererythrobacter_sp._TH136	NA	1.714296519	1.700192533	NA	3.320519626	3.759198254	-0.661195156	2.158376525	2.095232011	-0.122270213	0.762300994	0.048192299
Methylomicrobium_alcaliphilum	NA	1.731747885	1.461797081	-0.544408402	3.00342339	3.712798481	0	0	0	-1.484506425	1.411957674	-1.089946433
Methylobacillus_flagellatus	NA	1.792121983	1.600333358	NA	0.032237649	0.189844555	0.298560277	0.774850678	0.673801421	-0.431012193	-0.668500288	0.112371439
Qipengyuania_sediminis	NA	1.91398002	2.785875195	-0.913911023	NA	NA	0	0	0	0.612310308	-0.975173758	0.02293075
Hoyosella_subflava	NA	1.915714446	2.132062047	NA	NA	1.588986757	-1.906495063	0.316881181	1.553293937	9.252665432	0	9.993405473
Massilia_putida	NA	1.923480766	3.626960849	NA	NA	-0.187549152	0	8.559465045	6.15711649	-0.858575379	-0.175733275	2.052007039
Pandoraea_pulmonicola	NA	1.927550066	2.14908267	NA	3.030427643	3.028833644	-11.1883663	-2.56099658	-11.1883663	-0.023036838	0.983289468	-0.138976413
Brachybacterium_sp._SGAir0954	NA	1.938730675	-0.848298757	0.058245141	NA	3.555852912	0	0	8.904155902	0.489099909	3.514121939	2.335260157
Pseudomonas_sp._CMR5c	NA	1.969552384	-0.770177373	NA	4.330439595	3.749714087	0.512650076	-8.091313724	-8.091313724	0.043412262	0.09302271	1.07530227
Lysobacter_capsici	NA	1.983090221	2.150660241	NA	3.062149603	3.331673876	0.411426604	2.562346455	3.437408975	-0.299247547	1.335470735	-1.551072133
Methylococcus_capsulatus	NA	2.037495283	2.304434143	0.031466186	3.467022105	3.841685087	-9.069511529	1.760932374	-0.588628449	-0.140340708	0.49143089	0.644788668
Enterobacter_sp._RHBSTW-00175	NA	2.084888898	NA	NA	NA	NA	0.013556458	2.095850978	-1.692231858	0.188171465	-1.934507767	-1.21127016
Halothiobacillus_sp._LS2	NA	2.132663421	1.085973223	NA	4.88544421	4.31517976	-0.700040829	-1.111038601	1.667342154	1.523386217	2.374019364	3.107415277
Aquitalea_magnusonii	NA	2.172872285	NA	NA	NA	NA	-0.324020855	-0.484049916	-0.115949154	-0.503574174	-7.807354922	-7.807354922
Cronobacter_malonaticus	NA	2.22839343	-0.550926177	1.419359105	NA	3.817486826	0.392629675	0.742882457	0.966679065	1.78006026	0.891204798	2.736148888
Staphylococcus_fleurettii	NA	2.259526668	2.653172584	NA	NA	NA	0.05387771	-9.222246695	0.338955316	8.442943496	7.29523629	10.60053552
Citrobacter_koseri	NA	2.272684576	NA	NA	2.13774911	1.346571833	-0.186734308	-0.486442097	-0.079666863	1.586677255	-0.321933891	3.141012654
Thioclava_nitratireducens	NA	2.301277865	0.656825756	NA	3.538529532	4.287158599	1.807634821	2.340211495	1.492331523	0.755860384	0.112347352	1.536899012
Pseudomonas_sp._DTU12.3	NA	2.326128083	3.59787339	NA	-2.173163376	-2.836398581	8.157119522	8.607330314	0	0.297553464	-0.764931263	-8.992465383
Streptococcus_parasanguinis	NA	2.490573854	1.661698017	-3.906544584	-0.827040615	-0.229851812	-8.799135887	1.080168598	0.652075224	0.31285221	0.460289485	-1.697904285
Pediococcus_claussenii	NA	2.602816166	-0.139162748	2.887049683	NA	NA	0.046326441	-0.474254589	0.426540306	0.682232449	-4.33379278	-0.606608207
Enterococcus_sp._FDAARGOS_375	NA	2.75161237	2.959856654	6.985398289	NA	-1.421682342	-0.457767453	-8.624046047	3.072130388	-0.704871964	-1.011848974	-0.127950813
Gordonia_sp._YC-JH1	NA	2.868944496	3.168825452	NA	3.603156238	3.53221709	5.101653509	1.279504132	0.830925535	0.204165923	1.347619738	0.950407026
Spiroplasma_culicicola	NA	2.898735783	1.449840471	NA	NA	-1.563473078	0.827655837	-10.25158892	0.6512007	11.34466763	9.568588898	9.412216274
Pseudomonas_sp._02C_26	NA	2.939266598	1.620336831	NA	NA	NA	0.685378666	0.893113496	0.95934747	-0.44560576	-0.656098804	-8.727920455
Saccharopolyspora_erythraea	NA	3.004598517	1.660875015	1.635440946	5.352321811	5.120695521	0	0	0	-0.228025527	0.111590115	-0.050696843
Izhakiella_sp._KSNA2	NA	3.126949746	3.824972653	1.450201092	1.762807629	1.885808798	0.582753142	0.752798213	0.691801863	-0.919519953	0.143550776	0.783574089
Phreatobacter_sp._NMCR1094	NA	3.190077658	2.283031314	NA	0.292466632	NA	0	0	9.695386649	9.722388878	9.432681779	9.997179481
Hymenobacter_sp._HDW8	NA	3.334661585	1.874469118	NA	1.894042825	2.729971906	-0.074595952	0.109347332	0.276231497	-1.401883786	-0.862429167	-0.882970981
Enterobacter_cloacae_complex_sp.	NA	3.375170116	0.136188096	NA	NA	NA	-0.508001178	-8.637284195	3.05889224	0	0	0
Enterobacter_chengduensis	NA	3.39185874	1.74464974	NA	-0.697104006	-0.594240314	-0.454162421	0.862156265	0.851249587	0	8.979187942	9.956133631
Acidiphilium_multivorum	NA	3.496166768	1.656488256	-0.250788145	1.255129208	2.133556339	0.088620328	0.217818005	0.154548445	-10.36777511	0.029546563	-0.315207056
Flavobacterium_sp._LPB0248	NA	3.504971266	2.401141273	-3.534990812	NA	-3.938346506	0.043238258	0.496596325	-0.076940537	0.596234074	1.358141235	1.204684529
secondary_endosymbiont_of_Ctenarytaina_eucalypti	NA	3.676232192	3.999040442	NA	NA	NA	-0.785283075	-0.038738684	-0.047706941	1.727315424	2.207511136	-8.076815597
Enterobacter_bugandensis	NA	4.847576825	0.783525122	NA	-0.512884823	1.990359751	0.032982252	-0.420481715	-0.164745683	3.105269274	1.823898991	1.721648932
Aeromonas_sp._ASNIH3	NA	5.497002957	2.514158546	NA	NA	NA	-1.593964861	-0.737907585	-0.258554204	-0.72941987	-0.690130724	-0.328278044
Leclercia_adecarboxylata	NA	6.447179594	3.813033589	0.349631772	-1.741209919	-2.351395004	-1.4213249	-0.329176503	-1.553914777	-0.987394371	0.023897651	-0.236412591
Kluyvera_genomosp._3	NA	6.453932323	3.045613344	-2.453209744	NA	NA	0.387701844	0.632847866	0.716624538	-1.25570176	-0.05197458	-1.348201789
Enterobacter_roggenkampii	NA	6.818675697	3.212161225	NA	1.514787207	-0.442408471	-0.973818883	0.094668	0.366470909	-1.543974089	-9.438791853	1.38138711
Enterobacter_kobei	NA	7.131650031	2.923378718	NA	NA	NA	3.016304814	3.060060197	1.642105941	7.129283017	0	0
Yersinia_entomophaga	NA	7.177771085	3.889079319	NA	NA	NA	-8.799135887	0.62751046	0.512510266	-1.17024945	0.549657464	-9.536571664
Lactobacillus_farciminis	NA	NA	-8.074845663	0.564218797	-0.364341802	-1.674739507	8.876516947	0	6.15711649	-1.220664931	-0.714424762	-0.163794432
Persephonella_marina	NA	NA	-5.871013559	NA	NA	-2.728428484	0.916058547	0.080164221	0.313656174	-0.093954156	1.656263434	1.538749504
Methylophaga_nitratireducenticrescens	NA	NA	-5.193633428	-2.386581053	-2.553691039	0.235470766	-0.273782511	0.059995813	0.451825756	0.386800933	1.918995051	1.860776107
Synechococcus_sp._WH_8109	NA	NA	-5.154373998	2.208494838	0.594927781	0.089012776	0.256018593	0.221721385	0.513382791	1.278120127	1.454042371	0.650370173
Caulobacter_sp._Ji-3-8	NA	NA	-5.152240359	NA	0.359172445	-2.071827349	-3.471793493	0.113983231	-0.596149061	-0.597292791	-0.221646702	-0.35721442
Shewanella_sp._YLB-08	NA	NA	-5.120578948	-5.00404865	NA	NA	1.076676715	1.336668568	1.233440313	1.175864264	1.04471748	0.359243032
Woeseia_oceani	NA	NA	-4.981852653	NA	-1.289633946	-1.460434635	-0.167365812	-0.89980795	-0.53765593	-0.192645078	-0.86507364	0.45169597
Dysgonomonas_sp._HDW5A	NA	NA	-4.954604486	NA	NA	NA	0	0	9.280348569	-0.906815789	-1.167626969	0.073726129
Flavobacterium_sp._M31R6	NA	NA	-4.880181785	NA	NA	1.580263164	-1.802694462	-3.622763181	2.130472428	0	7.59792591	7.930737338
Lysobacter_sp._TY2-98	NA	NA	-4.768108977	NA	NA	NA	11.36632221	10.40552443	9.069511529	1.446254599	2.881239016	-0.613585755
Pseudomonas_alkylphenolica	NA	NA	-4.742863721	NA	-4.612305313	NA	0	8.627369725	9.921840937	-0.545752479	-0.394807529	0.152681237
Elizabethkingia_sp._JS20170427COW	NA	NA	-4.710364932	-2.045648086	-2.876693114	-2.587946696	-13.01414778	-1.170795915	-0.731690601	-0.357111304	0.310417304	0.069229473
Candidatus_Sulfurimonas_baltica	NA	NA	-4.68182404	NA	-2.736965594	-2.638112207	0.52126534	-0.408195719	-0.105201802	0.186223419	0.839717538	0.353596084
Vibrio_sp._HDW18	NA	NA	-4.652939295	NA	NA	NA	-2.026489905	-0.413285185	-1.675304851	0.072739974	-0.740846902	-0.542949748
Ciceribacter_thiooxidans	NA	NA	-4.59820371	NA	NA	NA	0.354766824	1.352009357	-0.402898552	-0.102810218	0.529130524	0.172224044
Weissella_ceti	NA	NA	-4.548226028	1.924252814	NA	0.18932472	-1.590611076	-2.437478759	-1.63820999	-0.38669568	1.697795659	3.561517078
Sediminicola_sp._YIK13	NA	NA	-4.460240985	NA	-0.374065718	0.628210749	0.262537948	1.619840074	1.44823716	-0.895420746	1.871089124	3.06642342
Francisella_orientalis	NA	NA	-4.44014028	NA	NA	NA	-0.030680638	-0.81877963	1.105634626	0	0	9.412216274
Caballeronia_insecticola	NA	NA	-4.43506707	NA	3.489088736	1.265733555	-8.799135887	-0.191805574	0.638899079	0.851137594	-2.61497507	-8.087462841
Edwardsiella_hoshinae	NA	NA	-4.374346629	NA	-0.75085248	-3.049321602	-0.574643657	0.354411893	0.656049295	0.409902311	-0.351413144	1.448450654
Synechococcus_sp._PROS-7-1	NA	NA	-4.282153636	NA	NA	NA	-8.637284195	0.789362153	-8.637284195	-10.1581931	-10.1581931	-10.1581931
Methylomicrobium_buryatense	NA	NA	-4.199132181	NA	NA	NA	0.390978347	-2.956258394	1.39411438	-8.087462841	-8.087462841	-8.087462841
Aureitalea_sp._RR4-38	NA	NA	-4.155250291	NA	0.424034014	1.575805144	0.259770205	5.984870678	4.720345627	-0.883485991	2.693161241	2.849120064
Candidatus_Fokinia_solitaria	NA	NA	-4.137215876	-0.085630072	NA	1.35767128	-0.557984666	-0.213979135	0.051551571	2.167631187	1.843694193	2.648795644
Limnohabitans_sp._103DPR2	NA	NA	-4.105227684	0.620442567	NA	-0.674537855	0.696476131	0.554089863	-0.209621764	0.54189484	-8.824427375	2.461362054
Elizabethkingia_miricola	NA	NA	-4.05145497	0.312169128	2.265733555	2.973157199	0	0	0	0.940499937	1.668705375	0.367824272
Streptococcus_pseudopneumoniae	NA	NA	-3.990477226	0.058245141	NA	NA	-8.04736934	-8.04736934	-1.89025285	-2.856090589	-10.15987134	-10.15987134
Pseudomonas_lalkuanensis	NA	NA	-3.929503834	NA	NA	NA	-2.15324255	-2.103667062	-1.90037956	9.033423002	0	8.761551232
Spiroplasma_gladiatoris	NA	NA	-3.904150433	1.680713398	-0.496929516	0.516070613	2.530514717	2.843671249	2.203537612	-1.604800693	-11.36413466	0.437844436
Aggregatibacter_segnis	NA	NA	-3.809161679	NA	0.963267449	NA	0.182249828	1.866261312	2.388046667	-0.637874587	1.584693719	1.625035054
Acinetobacter_tandoii	NA	NA	-3.691779931	NA	-1.165564606	NA	0.073937643	-0.097642224	0.372684492	0.00913253	0.98259081	-3.078894491
Methylobacterium_sp._C1	NA	NA	-3.567981323	NA	NA	NA	0.434406194	1.373573788	1.650213916	0	0	0
Iodobacter_fluviatilis	NA	NA	-3.540012207	NA	NA	NA	-10.76185038	-0.234911062	-2.019770039	0	7.59792591	6.544320001
Spiroplasma_floricola	NA	NA	-3.5360529	NA	NA	NA	-0.033320395	-8.637284195	-0.013238148	0	0	10.1015424
Paenisporosarcina_sp._K2R23-3	NA	NA	-3.518929578	NA	-1.652208371	-5.465368915	-0.758037075	0.269900359	0.126674296	0.328065161	0.63200715	0.150919506
Vibrio_tritonius	NA	NA	-3.501856065	NA	1.08039207	NA	0	0	9.561202011	9.290018847	9.537864847	0
Nocardioides_sp._dk3136	NA	NA	-3.474310795	NA	NA	NA	-9.177646265	-9.177646265	0.562901891	0	0	9.878561362
Rhodococcus_sp._WMMA185	NA	NA	-3.452512205	0.211951355	-0.378118036	0.642201949	-0.376633243	1.724232195	0.04773188	0.595640197	1.701589177	2.177762746
Candidatus_Ruthia_magnifica	NA	NA	-3.447787721	2.419503687	0.219699318	2.1725733	9.870925659	9.426646347	12.61988753	0.185559311	-0.238287743	1.522041844
Streptomyces_clavuligerus	NA	NA	-3.42685325	NA	1.031326334	-1.102051271	0	0	0	7.303780748	0	10.306825
Mycoplasma_citelli	NA	NA	-3.419820296	NA	NA	NA	0.984053059	-1.124436423	-0.117596583	0	7.369230901	7.539158811
Brevundimonas_sp._DS20	NA	NA	-3.388697107	NA	0.275365208	NA	0.315662832	0.250226761	1.098076211	0.624855228	-0.925545783	-7.741466986
Pseudomonas_guangdongensis	NA	NA	-3.386278506	NA	-1.099816873	NA	0.09133639	1.340666213	0.075813724	-0.140015166	-0.013273024	-9.50779464
Providencia_vermicola	NA	NA	-3.379832168	NA	NA	NA	-1.202059247	0.629621509	0.760735383	-0.302719834	1.680632922	1.554116703
Guyparkeria_halophila	NA	NA	-3.305408506	NA	1.507871974	1.175388284	0.25183864	0.321483483	0.162364856	-0.522278368	-1.251691316	-0.103269271
Acinetobacter_lactucae	NA	NA	-3.303549566	NA	NA	NA	-0.584962501	1.249568143	2.918295359	9.960001932	9.432681779	0
Algoriphagus_machipongonensis	NA	NA	-3.29799004	-1.438312642	-0.012921227	-2.932346176	0.120554254	-0.01023677	-0.142925868	-0.033738414	-0.470981708	-0.012718504
Idiomarina_loihiensis	NA	NA	-3.279058833	2.485153097	0.389230388	1.518839382	-0.947401746	-0.207724501	-0.304208025	0.387960135	1.257488033	1.836341489
Paraburkholderia_terricola	NA	NA	-3.278692818	NA	0.982297998	1.683559531	-0.459841035	-0.690985515	0.575604913	2.872796558	-7.709655951	0.702562441
Streptomyces_scabiei	NA	NA	-3.275634443	NA	-0.583256179	NA	-8.637284195	0.746813114	-0.480164672	-9.594945967	-9.594945967	-9.594945967
Colwellia_sp._20A7	NA	NA	-3.258670561	-3.457348304	-3.280533455	NA	0	0	0	-1.338279337	0.520224313	-0.307126002
Sphingobium_sp._PAMC28499	NA	NA	-3.168411949	NA	NA	-0.523791703	0	0	6.15711649	8.45395786	10.1438041	10.03617361

Shewanella_baltica	NA	NA	-3.11391332	NA	-0.566715358	0.182730986	1.205453174	1.207109646	1.399549136	-0.032146887	-1.174589915	-0.541763465
Spiribacter_sp._2438	NA	NA	-3.055638877	NA	NA	-2.602309744	9.161703808	0	9.624047875	-1.312362124	-0.348577803	-8.64625988
Rhodobacter_sp._LPB0142	NA	NA	-3.024476092	-1.417235055	NA	2.656188113	0.37256782	0.167352309	0.525241134	0.766241405	0.642417132	2.028195442
Actinobacillus_pleuropneumoniae	NA	NA	-3.009123671	3.120855394	-4.122587214	-5.113953465	0.498298242	-0.31522341	-0.046250785	0.018470121	-0.302046942	0.049623594
Rhodanobacter_glycinis	NA	NA	-2.981187499	NA	1.077118677	0.244554985	0.03077187	1.512181534	1.581769251	0.818069635	-1.77444249	-0.244526522
Methyloceanibacter_caenitepidi	NA	NA	-2.94560486	0.205010343	0.445225535	0.44204954	-0.269655006	1.318146831	1.565954533	-3.467531026	-1.504762569	0.372335007
Rhizobium_sp._007	NA	NA	-2.94560486	NA	-0.506959989	-0.970616193	-0.347922394	-8.75731333	-0.600193808	-9.403721476	-0.885394857	1.257459828
Marivirga_tractuosa	NA	NA	-2.939535359	-1.417235055	0.569508848	-0.091792476	0.285185704	-2.267383966	-0.970482391	-1.942016816	-4.835851259	-1.07312177
Bacillus_albus	NA	NA	-2.920513879	NA	NA	NA	-7.757316664	1.850013649	0.693894448	0	0	0
Xanthomonas_phaseoli	NA	NA	-2.920513879	NA	NA	NA	-0.147732396	-0.795515108	-0.273490363	-0.760038085	-10.72678865	-10.72678865
Ochrobactrum_anthropi	NA	NA	-2.920513879	NA	NA	NA	-0.073396138	0.801405414	0.607596609	-0.867586445	-9.860015828	-0.181745715
Bradyrhizobium_zhanjiangense	NA	NA	-2.897687242	NA	-1.165370317	2.185929207	-0.694808687	-0.517615064	0.463517417	-0.167736857	0.46799033	0.728168646
Apibacter_raozihei	NA	NA	-2.867396049	-0.200278036	-0.961755347	NA	-0.016407218	1.371475376	0.394253261	1.092478713	1.792988323	2.028374261
Burkholderia_sp._CCGE1003	NA	NA	-2.864631977	1.339646201	NA	-0.026655632	0	0	0	8.129283017	0	10.488513
Bacillus_tequilensis	NA	NA	-2.855589701	2.202180785	-1.00841224	-0.569839226	1.137687947	0.295456718	0.94358147	-0.211212241	-0.337153448	-3.349365223
Nitrosomonas_sp._Is79A3	NA	NA	-2.832445903	NA	-0.567989422	NA	-10.54549545	-10.54549545	-0.68864511	7.894817763	9.828664899	8.539158811
Roseivivax_sp._THAF30	NA	NA	-2.832445903	NA	NA	NA	-2.059083288	0.780692785	0.672367641	0	6.815921204	0
Acinetobacter_defluvii	NA	NA	-2.807354922	2.475300456	NA	NA	0.491998822	1.580564479	3.679512089	0	0	0
Mycobacterium_canettii	NA	NA	-2.771402193	NA	0.206984517	-0.408115532	-0.265903804	-1.716374726	0.326237788	-2.76499753	-0.432153657	-3.524458277
Pseudomonas_taetrolens	NA	NA	-2.711987939	-0.868403117	NA	-2.149303944	-8.941980727	2.904935632	2.125695632	-2.366541962	-0.015107476	0.775293713
Nonlabens_sp._MB-3u-79	NA	NA	-2.702333709	-1.046214555	-1.155443625	2.843289408	-1.440836505	-1.843380649	0.994907854	0.701775814	-0.146092484	-1.843464544
Sphingobium_sp._KCTC_72723	NA	NA	-2.702333709	NA	NA	NA	-0.427975107	0.55743162	-1.549068891	7.303780748	0	7.63420844
Mycoplasma_dispar	NA	NA	-2.702333709	NA	NA	NA	1.505510944	1.782096243	2.737505357	0	7.902875524	7.539158811
Melaminivora_sp._SC2-9	NA	NA	-2.637820948	-0.229356856	-1.996379684	-2.476753284	-0.768309828	-0.415837852	1.516509068	-1.494959964	1.844872003	2.067510475
Dyella_thiooxydans	NA	NA	-2.621420503	NA	NA	NA	-1.272079639	-2.642447826	-0.664430186	-8.809501266	-0.581967146	-8.809501266
Pseudomonas_sp._14181154	NA	NA	-2.589174752	NA	NA	NA	0.246389145	0.083453656	0.528298685	0	6.815921204	0
Amycolatopsis_albispora	NA	NA	-2.573466862	NA	NA	NA	-0.354457032	-0.119328572	1.102817682	9.536571664	9.539158811	6.544320001
Sulfurimonas_sp._CVO	NA	NA	-2.51583814	-0.126263773	NA	NA	1.774364998	1.997566741	-0.331679128	-1.789347	0.354754226	1.31817912
Pseudomonas_multiresinivorans	NA	NA	-2.51583814	NA	-2.298966946	-1.154577037	10.93262078	11.74742936	14.75854595	0.662963553	-7.700439718	1.606380725
Francisella_noatunensis	NA	NA	-2.51583814	NA	NA	NA	0	0	0	0	7.369230901	0
Candidatus_Rhodoluna_planktonica	NA	NA	-2.499437464	NA	NA	NA	0.594000699	0.919891651	0.422492731	0	0	0
Gordonia_rubripertincta	NA	NA	-2.46546133	-0.619629633	-0.008082346	1.353812156	9.029913869	10.40552443	8.157119522	-0.06144933	-3.25646477	0.491572135
Edwardsiella_tarda	NA	NA	-2.42685325	NA	NA	NA	-12.27294756	-4.162521675	-1.153649753	0.465662018	-7.807354922	-7.807354922
Lactobacillus_nenjiangensis	NA	NA	-2.422647033	-1.158356874	-0.279592521	-1.025941903	-2.746444683	-1.625270489	-1.342905425	-2.078035187	-0.355369455	-0.236459798
Mycobacterium_paraseoulense	NA	NA	-2.419820296	0.363690619	NA	NA	0	0	0	0	9.218522068	0
Mycolicibacter_sinensis	NA	NA	-2.419820296	NA	NA	NA	9.029913869	0	0	-1.23102703	-1.472404944	0.094937384
Granulicella_sp._WH15	NA	NA	-2.336994706	0.713893608	-2.226905078	-1.082515168	1.10935585	-9.471675214	-9.471675214	0.502659746	1.576078882	1.502969129
Streptomyces_sp._endophyte_N2	NA	NA	-2.306661338	NA	0.357156583	NA	-0.695781686	1.109105831	0.739897179	0	8.655827639	0
Mycolicibacterium_aubagnense	NA	NA	-2.306661338	NA	NA	-0.236733444	1.961311851	2.648901317	1.262265817	9.271463028	10.42206477	10.74371523
Microbacterium_sp._1.5R	NA	NA	-2.306661338	NA	NA	NA	-2.990199898	0.370709121	-1.294009865	-7.544323092	-0.321933891	-7.544323092
Nonlabens_sp._Ci31	NA	NA	-2.271596419	NA	NA	2.348645433	-9.624047875	-9.624047875	-2.466933407	0.898158475	0.833603053	-0.175508574
Capnocytophaga_sp._H4358	NA	NA	-2.250177092	NA	NA	NA	0	0	0	-0.518497929	0.646096773	-0.124840378
Moraxella_cuniculi	NA	NA	-2.23426337	NA	NA	-0.996178289	-0.480164672	1.011481419	-8.637284195	-0.124289584	1.315744016	0.853600282
Bradyrhizobium_guangzhouense	NA	NA	-2.205555527	NA	1.722794694	3.466139552	0.63810116	0.323231058	-0.381973122	-0.915624817	-0.558291386	-0.637053577
Corynebacterium_epidermidicanis	NA	NA	-2.187627003	NA	NA	NA	0	8.382131157	10.2623063	-8.809501266	-0.130021166	-0.288556567
Pseudomonas_sp._CC6-YY-74	NA	NA	-2.163173959	-0.812775198	1.269460675	NA	0	0	0	0.811529865	2.104704131	-0.161788093
Mesorhizobium_sp._AA22	NA	NA	-2.156951724	NA	NA	NA	-0.072172052	0.383106704	1.591960071	-7.930737338	-7.930737338	-7.930737338
Pseudogulbenkiania_sp._NH8B	NA	NA	-2.144084997	1.823843045	-0.103093493	0.545741191	-0.433017624	-0.201563815	0.526036206	-0.720381668	-1.196224834	0.276180109
Pseudomonas_sp._NS1(2017)	NA	NA	-2.120885843	NA	-1.73345111	1.097775216	-9.848338129	-9.848338129	0.584965631	0.811959847	1.012609683	1.360071836
Sanguibacter_sp._HDW7	NA	NA	-2.120154652	-0.817015295	-3.057374263	0.892460979	-0.172600896	0.43241274	-0.126766645	2.359256065	1.686345188	2.176662458
Mycolicibacterium_chubuense	NA	NA	-2.11753632	0.355774724	1.590855069	0.978185639	1.560295146	-8.552496194	3.661215604	9.74819285	10.80842528	9.968666793
Thermomonas_brevis	NA	NA	-2.108321592	-4.185324912	NA	-3.370947206	0.022805024	-0.358263726	0.345575604	-0.085002436	-0.402343141	0.148857509
Candidatus_Thioglobus_sp._NP1	NA	NA	-2.10773024	NA	NA	1.128945273	0.460165992	-0.54376148	-2.768776146	2.055182915	2.805148067	1.173838936
Stenotrophomonas_sp._G4	NA	NA	-2.04524368	NA	2.51473899	3.963899259	-0.149013818	0.6222264	-0.755217521	0.486422851	-0.483344463	-0.478500493
Deefgea_sp._D17	NA	NA	-2.027093661	NA	0.655735575	NA	-0.642016365	-8.799135887	-0.493694257	-0.409002615	0.777230126	-1.347095785
Mycolicibacterium_rhodesiae	NA	NA	-1.982722009	NA	NA	NA	0	8.263377193	7.157114468	-0.091497956	-0.67272434	-0.215601549
Bifidobacterium_indicum	NA	NA	-1.948918152	0.799274588	0.346041936	0.574479276	0.567498101	0.968993333	0.116047361	0.785261151	0.644620747	-0.409688845
Pseudomonas_sp._DY-1	NA	NA	-1.931730724	NA	NA	NA	-10.30750822	-10.30750822	1.386492652	0	0	6.544320001
Corynebacterium_mycetoides	NA	NA	-1.9040167	NA	0.736965594	2.947092734	0	8.110425888	0	-0.364352403	0.833381926	0.928129826
Pseudomonas_sp._ATCC_43928	NA	NA	-1.887916202	NA	NA	NA	-1.523506969	0.040074912	-1.12150813	0	8.215935782	9.306820443
Massilia_sp._WG5	NA	NA	-1.840059087	NA	NA	NA	0.357711646	-1.722931788	0.616784561	-9.018664771	1.637161079	-1.47950596
Sphingobium_sp._TKS	NA	NA	-1.835101816	NA	NA	NA	-0.818611099	0.771140729	1.206344119	0.405923347	-8.048034513	-8.048034513
Streptomyces_albulus	NA	NA	-1.832943751	2.426804556	NA	1.348870575	0.494838492	1.090289377	2.356141434	-0.735558104	-2.944717727	0.332035284
Geminocystis_sp._NIES-3708	NA	NA	-1.814593748	-1.259166588	-2.279589306	-0.839790153	1.891448949	-1.074645056	-0.600202196	1.005704001	-1.672298166	-0.253786471
Mycobacterium_grossiae	NA	NA	-1.814176188	NA	NA	-0.297717541	0	0	0	0	9.432681779	0
Burkholderia_oklahomensis	NA	NA	-1.800565756	NA	NA	NA	0.959062146	0.500060519	0.35241267	0.203283598	-9.247927513	0.31685647
Pseudomonas_sp._ACM7	NA	NA	-1.790179778	NA	NA	NA	-0.072979493	-2.788071744	2.271521162	7.894817763	5.472487771	0
Bradyrhizobium_sp._CCBAU_53340	NA	NA	-1.735615388	0.836899198	NA	-0.40183196	0.782705631	-0.239405229	-0.326410713	1.05011056	0.291393899	0.72521104
Arthrobacter_sp._24S4-2	NA	NA	-1.723212439	NA	NA	NA	0.224760049	-0.177619864	0.026620093	0	0	0
Corynebacterium_sp._zg-917	NA	NA	-1.669367516	NA	-0.767921339	0.16547621	-0.310130707	0.27743548	0.89502558	2.259386629	2.273018494	1.883383843
Leucobacter_sp._HDW9C	NA	NA	-1.666239542	NA	NA	NA	0.256892074	0.453127562	0.617597446	1.088488073	-0.576265166	-1.218372574
Xanthomonas_vasicola	NA	NA	-1.644645051	NA	NA	NA	-1.085246857	-1.202816245	-0.960342617	0	0	9.410805357
Pseudomonas_lurida	NA	NA	-1.643306487	NA	NA	NA	0.257348286	0.260711701	0.707760425	-8.700439718	-8.700439718	-8.700439718
Rhodococcus_pyridinivorans	NA	NA	-1.639557476	NA	NA	NA	-1.014152592	-0.853451962	-0.24531162	-8.076815597	0.042125476	-8.076815597
Arthrobacter_agilis	NA	NA	-1.63076619	4.46922837	2.84467327	-0.602137645	-0.386692047	-0.380736031	-1.621754183	0.383978151	0.818002166	1.042125476
Pontivivens_sp._MT2928	NA	NA	-1.624639115	NA	NA	-1.820590749	0.01126848	0.098931363	1.290115413	0.004048859	-0.178358837	1.884018156
Streptomyces_sp._ADI95-16	NA	NA	-1.592230501	NA	NA	NA	0	0	0	0.339348471	-0.268355749	-1.851438136
Nakamurella_sp._PAMC28650	NA	NA	-1.589174752	NA	-0.008082346	NA	10.35133681	9.913704737	9.466123378	-7.741466986	-2.268979215	2.586456108
Nakamurella_panacisegetis	NA	NA	-1.589174752	NA	NA	NA	1.171543711	2.442997542	0.404067483	0	0	0
Bradyrhizobium_amphicarpaeae	NA	NA	-1.588906987	NA	-0.420922696	1.588247754	-2.507719679	-1.218303281	-0.261628677	0.574236094	2.982528864	2.455861757
Candidatus_Nitrosoglobus_terrae	NA	NA	-1.535825722	NA	-3.470526255	-2.881648704	1.506233235	1.499336127	-0.040500945	-0.182306594	-0.68212902	0.153382955
Nocardia_farcinica	NA	NA	-1.491290532	NA	-0.737245702	NA	0	8.382131157	8.451211112	-2.082609919	-1.403854713	-1.380062451
Cellulophaga_lytica	NA	NA	-1.462045421	NA	NA	-0.71804392	-1.524341806	-0.585901779	-0.366058958	-0.138337919	0.481094234	0.908104375
Bordetella_pseudohinzii	NA	NA	-1.432704537	-1.033539111	-1.756531762	-0.584676734	-0.719299876	0.237923654	0.386877287	-1.595838816	0.19475578	1.948657094
Micromonospora_echinospora	NA	NA	-1.425305835	4.139837131	1.177962321	2.111441704	1.081694889	0.496732389	-1.726538745	2.472852134	0.254769211	-7.741466986
Enterobacteriaceae_endosymbiont_of_Donacia_crassipes	NA	NA	-1.418452164	NA	NA	NA	-0.941752139	0.168412164	0.797237283	1.960128852	3.809601965	0.77160128
Endozoicomonas_montiporae	NA	NA	-1.391358503	0.584363499	1.734987945	1.67717331	-0.551348305	-1.572744193	-3.04299945	-1.173596188	0.992991746	0.672841409
Streptomyces_albireticuli	NA	NA	-1.386278506	NA	NA	NA	-1.303179957	-3.041199281	-0.756609485	0	0	9.306820443
Thiobacillus_denitrificans	NA	NA	-1.367025137	NA	0.263232456	0.053655312	0.624716912	1.226465441	1.627031439	-0.307924921	-1.66644277	-0.579761145
Mitsuaria_sp._7	NA	NA	-1.362131637	-0.655718335	0.879160586	-0.740828234	-8.799135887	0.592790753	-0.642016365	0.459177468	0.332102275	-0.372210762
Vibrio_qinghaiensis	NA	NA	-1.353078261	-1.68222263	-1.276809549	-2.244345154	-10.13861739	-1.511247666	1.07316142	-8.076815597	-8.076815597	1.879318033
Acetobacter_senegalensis	NA	NA	-1.33965835	-1.680778288	2.11324705	2.218247934	0.79049811	-0.230896009	1.120575228	-0.662965635	1.235488563	-0.44904473
Dinoroseobacter_shibae	NA	NA	-1.336464188	0.149853026	NA	1.860830453	0	0	0	0.365132593	0.734655433	-0.259191572
Gluconobacter_albidus	NA	NA	-1.326586963	0.410049785	-0.544761442	-0.063621201	0	0	7.745136469	0.340363312	0.871852389	0.451359532
Bartonella_birtlesii	NA	NA	-1.325247604	-0.250875893	0.986008816	0.955405046	0.010467069	-0.548333076	1.178781441	-1.0099727	0.897674457	-0.595320368
Thioploca_ingrica	NA	NA	-1.315753298	NA	NA	0.996603024	-1.116045476	-0.742772961	-0.63309115	-1.055949181	1.045876107	1.53732508
Streptomyces_lincolnensis	NA	NA	-1.31024629	-0.217238955	-2.434278141	0.506178662	2.321925604	-0.61818122	1.013524017	1.173204635	0.537385261	2.409207096
Sporosarcina_sp._P37	NA	NA	-1.292921282	NA	0.806222064	NA	-1.135048856	-9.739012655	-1.140115263	2.102361718	2.894582406	1.08579704
Mycobacterium_noviomagense	NA	NA	-1.29152535	-0.249440006	-1.567989422	2.255843351	0.17039256	1.232421991	1.851774253	8.442943496	7.222389201	11.23282262
Tsukamurella_tyrosinosolvens	NA	NA	-1.290794063	NA	NA	NA	-1.112234884	-0.602500332	0.108847746	0	8.857980995	8.324932083
Acinetobacter_sp._ACNIH1	NA	NA	-1.278535499	NA	NA	NA	0.607881948	0.435051415	0.15107116	0	7.222389201	10.86573327
cyanobacterium_endosymbiont_of_Epithemia_turgida	NA	NA	-1.262094845	NA	-0.786744642	-0.309747151	0.254066252	1.371617152	1.837192809	1.821185376	3.44052316	1.006069036
Jatrophihabitans_sp._GAS493	NA	NA	-1.25919234	NA	NA	0.355228104	1.074741466	0.702310524	0.665627774	0.25927983	-1.252386045	0.0695454
Mameliella_alba	NA	NA	-1.247441138	NA	-2.245451437	0.485535755	-8.624046047	0.003323679	-1.466931578	1.426126678	0.825169185	-2.380492503
Methylomicrobium_sp._wino1	NA	NA	-1.238966646	NA	NA	NA	-1.822678355	-0.072293584	1.549596162	10.73018989	7.29523629	10.15987134
Pseudodesulfovibrio_profundus	NA	NA	-1.2263452	-4.223689226	-2.631823865	-2.739003954	-0.812855759	-9.222246695	-0.152735167	0.876588651	0.597582037	0.186772192
Ralstonia_insidiosa	NA	NA	-1.223350704	2.863977936	2.103960572	2.31472361	0.743182717	-1.930441759	-2.189882864	-0.272054879	-0.389818561	0.879527993
Janthinobacterium_sp._1_2014MBL_MicDiv	NA	NA	-1.211264971	NA	NA	NA	1.155083679	1.727538558	2.600977157	8.318917839	0	0
Streptococcus_sp._1643	NA	NA	-1.195664322	NA	NA	NA	0.571063578	0.19804776	0.757622826	-0.501986434	-1.159569606	0.616846441
Tenacibaculum_todarodis	NA	NA	-1.193654073	-1.173507933	2.653137867	2.551813157	-2.027183191	-0.099021135	1.058433395	2.234958026	1.565136753	2.060366984
Klebsiella_sp._LTGPAF-6F	NA	NA	-1.18377784	NA	NA	NA	0.378934644	-0.076075166	0.288967999	2.214666644	-7.741466986	-7.741466986

Psychrobacter_urativorans	NA	NA	-1.174789893	NA	NA	NA	0	0	6.15711649	7.894817763	0	8.902878538
Entomomonas_moraniae	NA	NA	-1.168409088	NA	-2.12855968	-1.984169771	-0.206247617	0.148536273	0.067500677	0.293405944	0.546327378	-1.87608211
Lactobacillus_gastricus	NA	NA	-1.165536045	1.534061602	1.834036965	NA	0.316133795	-0.311023567	0.018531822	-1.029073824	-0.693261561	1.243564864
Candidatus_Rickettsiella_viridis	NA	NA	-1.156611463	2.273991158	0.416856787	0.600353808	0	0	0	1.581811424	1.103399481	2.283671865
Crenobacter_cavernae	NA	NA	-1.130973554	NA	-1.259409077	-1.822808859	-0.184877257	0.734938726	1.112829681	1.23135639	0.790942023	0.223140405
Mycolicibacterium_boenickei	NA	NA	-1.118284018	NA	NA	NA	8.129283017	9.889020195	0	-7.709655951	-7.709655951	-7.709655951
Devriesea_agamarum	NA	NA	-1.118109273	2.70265123	0.255479213	-1.877898392	0.432218026	1.183605659	0.586487874	1.304528329	0.600953668	0.977062051
Spiribacter_roseus	NA	NA	-1.116231962	-2.832272554	1.682080679	0.607836967	-9.177646265	-9.177646265	-3.020529775	-1.381349841	2.051707296	-0.286304185
Streptomyces_prasinus	NA	NA	-1.064114324	NA	2.027093661	4.393537874	0	10.04488586	7.745136469	-0.387189824	-0.012073125	-1.416644459
Geobacillus_genomosp._3	NA	NA	-1.057838011	-1.045978877	NA	2.208621432	-0.223522617	-0.183465045	-0.169920318	1.896330364	3.517911793	2.883888435
Vibrio_nigripulchritudo	NA	NA	-1.035919574	NA	NA	NA	9.806477123	6.682671608	11.80721	9	7.811644421	9.559696379
Gordonia_sp._KTR9	NA	NA	-1.028061083	1.152003093	NA	-0.063725598	4.140234486	-8.091313724	-0.934199256	0.952627721	1.811980398	-0.256560464
Coxiella_burnetii	NA	NA	-0.997864256	NA	NA	NA	-1.12292983	-1.19216164	0.170949719	-0.733742014	0.141898452	0.52432337
Mycobacterium_haemophilum	NA	NA	-0.993415347	0.129924928	1.063201852	2.451031326	-8.799135887	-8.799135887	1.287968888	0.734162936	1.406175808	-0.145081498
Polaribacter_sp._Hel1_33_78	NA	NA	-0.962581527	NA	NA	-0.124305663	-1.026132118	-1.97366852	-3.771802218	1.248460303	0.708617363	-0.582749679
Capnocytophaga_sp._oral_taxon_323	NA	NA	-0.947791522	NA	NA	-1.223446253	9.029913869	0	10.23970592	2.378247588	2.20522648	1.322840976
Brenneria_rubrifaciens	NA	NA	-0.93037869	-1.17342794	-1.151774439	-0.949047514	-9.638930002	-0.011562101	-1.014883956	0.708463323	0.84316188	0.027422732
Paracoccus_jeotgali	NA	NA	-0.917143068	NA	NA	NA	0.309043495	0.038387655	1.260242545	1.273300342	0.538822321	1.893048879
Vibrio_breoganii	NA	NA	-0.915387772	NA	0.111031312	NA	-0.903365622	0.510928661	-0.687272281	9.979182225	0	0
Aeromicrobium_erythreum	NA	NA	-0.915387772	NA	NA	NA	-1.142190996	0.374669258	0.613636545	7.129283017	6.815921204	8.866249642
Burkholderia_stabilis	NA	NA	-0.912537159	NA	NA	NA	9.029913869	9.627367901	10.43330376	8.366322214	7.058890082	8.324932083
Paracoccus_aminovorans	NA	NA	-0.911562035	-0.23346129	-0.907023987	0	-0.25967407	-0.224105757	-0.10853653	-1.186354944	-0.738292793	-0.998233033
Isoptericola_dokdonensis	NA	NA	-0.906983844	0.350845919	NA	-0.606682068	-0.359868533	0.286541655	-0.022656177	0.457833517	0.037622195	0.215291292
Mycobacterium_sp._JS623	NA	NA	-0.899695094	NA	NA	-3.181588015	-8.091313724	-8.091313724	-0.934199256	0.585472874	-0.385965644	0.712189581
Buttiauxella_agrestis	NA	NA	-0.879318518	NA	NA	NA	9.661778098	8.566400396	8.548995253	0	8.912889336	0
Halomonas_sp._NBT06E8	NA	NA	-0.851569831	NA	NA	NA	0.407878431	1.197277775	1.080456551	0.63447975	1.6392895	1.517851869
Bradyrhizobium_betae	NA	NA	-0.836286085	3.149975412	0.357156583	1.346571833	0	0	0	1.709744125	-7.741466986	0.797691825
Pseudomonas_lini	NA	NA	-0.816553859	NA	NA	NA	0.774100615	1.239246839	-0.645569345	0	0	0
Oligella_urethralis	NA	NA	-0.802154913	4.442966885	3.48909213	3.884883072	-0.26598169	1.608413401	0.818019949	1.883534944	1.571791211	2.322095307
Burkholderia_sp._THE68	NA	NA	-0.779963	1.101879614	2.76605212	2.670353992	0.883646901	-8.552496194	2.146842008	0.766361142	1.580270632	1.575572544
Salinimonas_sp._G2-b	NA	NA	-0.705095079	NA	NA	NA	9.386058628	10.03742341	8.742080338	-0.536484326	-0.583893738	-2.265181265
Acinetobacter_baylyi	NA	NA	-0.704082232	4.014686297	0.675936161	4.313652302	0.132117962	-0.185895721	-0.117747718	-1.059781806	-0.959060723	1.377285159
Maribacter_dokdonensis	NA	NA	-0.700965577	NA	NA	NA	-0.753541261	0.333210181	1.604499271	0	0	0
Thalassotalea_sp._HSM_43	NA	NA	-0.687355146	NA	NA	NA	-0.694756961	-1.965437322	1.076896539	-0.545741191	1.108465187	1.441767865
Pseudomonas_poae	NA	NA	-0.687355146	NA	NA	NA	0	0	0	7.333897756	7.29523629	8.539158811
Flavobacterium_sp._P2-65	NA	NA	-0.671912924	-1.881412318	0.967247287	1.802257054	-1.104381491	-0.079402579	0.653888041	0.176522693	0.83456779	0.117763685
Paraburkholderia_sprentiae	NA	NA	-0.648443104	1.321408486	-0.442131981	1.514339372	-0.897143934	-0.264544615	0.908938492	-0.590069563	0.439527343	1.267326042
Rothia_terrae	NA	NA	-0.622842384	NA	NA	NA	0	0	7.157114468	0.461580085	-8.930737338	0.704677885
Sphingomonas_sp._HMP6	NA	NA	-0.622582156	NA	NA	NA	0.011321576	0.278934734	0.264921162	-8.759332297	2.320599093	-1.378507628
Azoarcus_sp._DD4	NA	NA	-0.521537121	NA	0.374679762	-0.81067528	-0.602465739	-0.579937376	0.33446268	-1.705095079	1.067793046	0.043943348
Variibacter_gotjawalensis	NA	NA	-0.508827665	NA	-1.237039197	0.452958774	-0.115876743	-0.25095123	0.470260238	-0.009559131	1.363031469	1.598591458
Novosphingobium_sp._ABRDHK2	NA	NA	-0.475268948	NA	NA	NA	1.938731386	1.664540777	1.831383843	8.27301694	7.811644421	0
Mycoplasma_penetrans	NA	NA	-0.472878194	1.319605537	-1.463843369	-0.19738017	0.253281938	-0.4695851	0.479548396	-1.398107278	-0.642072645	-1.445173854
Streptomyces_sp._HM190	NA	NA	-0.458353237	NA	NA	NA	10.77013528	0	9.477205768	1.375866075	1.053939841	1.79255792
Corynebacterium_aquilae	NA	NA	-0.431224991	2.251310409	1.75511813	1.024697237	-0.786494195	-0.304053736	-0.746923529	-2.890307711	-2.650359827	-0.91630292
Bordetella_genomosp._13	NA	NA	-0.406261846	2.575110979	0.565376034	-0.004370151	-1.423685389	-0.252443095	-0.031583415	0.877433123	1.135274658	0.484133266
Mesorhizobium_huakuii	NA	NA	-0.404841732	NA	0.536256493	0.414545364	0.622018893	0.159821693	0.420392701	0.620365597	2.774986117	2.033216084
Methylomonas_sp._LW13	NA	NA	-0.36882907	NA	NA	NA	-0.81961441	0.044380899	-0.423122458	0.938013113	1.472852134	1.565353457
Synechococcus_sp._WH_8101	NA	NA	-0.368410349	NA	NA	NA	-0.434187659	-3.126568908	-0.674056	-8.969625392	1.193609151	1.181809811
Synechocystis_sp._CACIAM_05	NA	NA	-0.340255374	NA	NA	NA	10.31371068	0	9.280348569	-7.807354922	-7.807354922	-1.263034921
Mesorhizobium_sp._DCY119	NA	NA	-0.315204739	NA	NA	NA	-10.82900121	-0.81421355	0.155025549	8.366322214	6.815921204	6.930737338
Tomitella_sp._HY188	NA	NA	-0.306961209	-0.463306708	NA	NA	1.951214764	-2.394143989	0.950592833	-1.645504043	0.028268802	0.487307786
Acinetobacter_indicus	NA	NA	-0.284682969	-0.175030143	-2.139345878	-0.966633888	-8.799135887	-0.688709999	3.415681047	1.561279821	1.053245916	1.188806575
Actinomyces_naeslundii	NA	NA	-0.282638964	NA	NA	NA	8.502740328	9.426646347	10.19565518	0	0	0
Elizabethkingia_ursingii	NA	NA	-0.268981786	NA	NA	NA	-1.071948891	-1.218717549	-0.669762481	0.768169053	-0.030155249	-0.270721947
Ichthyobacterium_seriolicida	NA	NA	-0.249901693	NA	1.76402749	1.370610033	0.116378817	-0.271208004	-0.316241387	-0.089739462	0.122177904	1.841304461
Pseudomonas_sp._NP-1	NA	NA	-0.237539278	NA	NA	NA	-9.739012655	-9.739012655	0.117837682	0.719734557	0.957663619	1.806413522
Novosphingobium_ginsenosidimutans	NA	NA	-0.227146469	-2.537965021	-1.234940884	-0.740399416	1.287904352	2.594217591	2.878973343	8.562242424	5.472487771	7.380824669
Synechococcus_sp._A15-44	NA	NA	-0.17596277	NA	NA	NA	0.678483533	2.219569255	0.104796144	9.706207623	9.154818109	8.324932083
Actinomyces_sp._Z16	NA	NA	-0.173451513	2.822314383	2.301348997	3.333030548	-0.269399919	-0.534696551	-0.359050177	-0.995027552	-3.703217843	0.077623389
Lacinutrix_venerupis	NA	NA	-0.131931695	NA	2.64694014	2.432739501	0	0	0	-0.919883456	0.489440392	0.811943733
Citromicrobium_sp._JL477	NA	NA	-0.129423692	0.452660661	NA	0.014403065	-9.999492713	0.817930689	0.546871246	1.997196338	2.595066964	2.06031012
Roseivivax_sp._THAF40	NA	NA	-0.122304097	NA	NA	NA	0	0	0	-8.932707872	-8.932707872	-8.932707872
Acinetobacter_sp._WCHAc010052	NA	NA	-0.119575234	NA	-0.820552224	NA	1.109747655	1.601396273	-8.04736934	3.762359577	2.391886453	2.503047127
Mycolicibacterium_doricum	NA	NA	-0.073462162	NA	NA	NA	-0.310738214	-0.575115359	0.069688728	-7.745956618	-0.376725717	-7.745956618
Edaphobacter_sp._12200R-103	NA	NA	-0.065339098	NA	0.484976759	-0.148507344	-0.34401721	0.060060901	-1.20473161	0.101744798	0.433967871	0.20882702
Arcobacter_marinus	NA	NA	-0.042870118	NA	-1.385536153	-1.150851216	-0.289766302	-0.361902534	0.638097466	0.094428513	0.198884505	-0.159973462
Flavobacterium_sp._HYN0086	NA	NA	-0.041629624	-1.230910203	0.658432192	0.795018262	0.037969293	3.287869671	1.604072925	0.375904925	1.266695342	0.070408184
Microbacterium_sp._A18JL200	NA	NA	-0.040583351	NA	1.5075905	0.374218595	-7.757316664	-7.757316664	-7.757316664	-7.741466986	2.195170953	1.254772037
Wolbachia_endosymbiont_of_Ctenocephalides_felis_wCfeT	NA	NA	-0.038219322	NA	NA	NA	-0.648619899	-1.087416559	-1.298053796	-0.852862583	0.48651137	1.125021862
Micromonospora_terminaliae	NA	NA	-0.011769222	-0.539928747	0.738372415	1.880852735	-8.04736934	-0.758685571	1.057111477	9.271463028	9.415308204	6.544320001
Streptococcus_sp._116-D4	NA	NA	-0.010742381	1.040434864	-1.178337241	-0.461763436	0.00917046	-4.057876547	0.49208862	-2.069906906	-0.223593916	-1.060471326
Altererythrobacter_amylolyticus	NA	NA	-0.008200965	NA	NA	-0.322239322	-2.167854978	-1.710407627	-1.370057966	0.638370842	-0.13526667	2.126118943
Pistricoccus_aurantiacus	NA	NA	-0.004432246	NA	NA	NA	10.4275957	11.97232648	9.157116995	0.003783815	1.057616999	0.275994033
Candidatus_Planktophila_limnetica	NA	NA	0.048595766	NA	NA	NA	0.184239123	1.39242036	1.258523691	-1.466808248	-1.338673393	0.061400545
Sinomonas_atrocyanea	NA	NA	0.050015538	NA	0.22026612	-1.149251433	0.081913677	3.232450144	2.830190881	0.913389425	2.319653204	1.989171619
Sulfitobacter_pontiacus	NA	NA	0.055330706	NA	NA	-1.669851398	2.569799097	-8.10567284	1.510039549	2.221627845	2.396291984	1.743811234
Plautia_stali_symbiont	NA	NA	0.057715498	NA	NA	NA	0.580784331	1.198363215	0.115679953	0	0	8.539158811
Mycolicibacterium_gilvum	NA	NA	0.094106899	-0.277109591	1.212856396	1.687180306	0	0	0	1.514501331	-1.752277376	1.091361114
Lentzea_guizhouensis	NA	NA	0.102759574	NA	NA	NA	0	0	0	0	6.815921204	6.930737338
Isoptericola_variabilis	NA	NA	0.106436936	NA	1.443476685	NA	-0.646996434	-0.063127712	0.003561976	-0.320807745	-0.519077785	2.932725282
Rhodococcus_sp._H-CA8f	NA	NA	0.113787455	NA	NA	0.311487674	-1.254814292	0.202913191	-0.785199916	-0.596644306	-0.676211563	1.396893098
Sphingorhabdus_sp._JK6	NA	NA	0.116473397	NA	NA	NA	-2.632414123	-1.362887298	-2.484092015	9.424865417	10.14974712	10.43045255
Rhizobium_phaseoli	NA	NA	0.137748193	NA	NA	NA	0	0	0	8.129283017	8.758446432	10.43809528
[Mycobacterium]_stephanolepidis	NA	NA	0.139032207	NA	NA	NA	0.311356088	0.810523288	-0.584133727	0	0	6.930737338
Mycobacterium_sp._MS1601	NA	NA	0.140032627	2.464291189	0.38827059	-2.314873337	0.566553747	-0.758688371	0.061873934	1.54191496	2.524685314	3.003006923
Ketogulonicigenium_robustum	NA	NA	0.146535181	-0.494359952	-1.485426827	-2.193008615	-10.49276451	-10.49276451	-0.045309292	0.397582325	-3.874763741	-9.347251513
Candidatus_Nitrotoga_sp._AM1P	NA	NA	0.148075588	NA	NA	NA	-0.507048021	-1.748635348	0.159854549	3.363595263	-7.709655951	1.478519931
Azospirillum_oryzae	NA	NA	0.155418549	NA	1.643207642	3.451550711	-1.149050158	-1.449488418	0.283488137	1.038809564	0.623770521	1.685428372
Pseudonocardia_sp._AL041005-10	NA	NA	0.157885617	0.186943585	NA	0.857497923	-10.91642439	-1.532327085	-1.761606285	1.833568759	2.04660726	-8.803055861
Mycoplasma_iowae	NA	NA	0.169925001	1.087390664	-0.795349831	0.060139773	-0.345729749	-0.840115539	-0.771035584	-1.931992569	-2.641558408	-3.392899958
Streptococcus_periodonticum	NA	NA	0.170870721	NA	1.143887141	-0.725283789	8.409390936	0	0	0.924986975	0.180150161	1.105684719
Herbaspirillum_robiniae	NA	NA	0.173331603	NA	NA	NA	-0.891667114	-0.42046945	-0.177435509	0	0	0
Streptococcus_milleri	NA	NA	0.209198495	0.653187274	0.75753297	-1.80110519	0	0	0	-0.004319838	-0.289199346	-0.346406154
Flavobacterium_branchiophilum	NA	NA	0.21810684	NA	NA	NA	0.546567318	0.449308639	1.39994206	2.513155624	1.37553867	0.603772739
Brevibacterium_sp._PAMC23299	NA	NA	0.256359316	1.477321778	-0.576500922	0.098403704	8.409390936	0	0	-8.930737338	-3.458249566	-1.296528897
Vibrio_taketomensis	NA	NA	0.261749726	NA	NA	NA	-1.511681441	-3.198022395	-1.457082106	1.803714528	1.48594341	1.630271599
Streptomyces_sp._GS7	NA	NA	0.282115147	1.078855421	NA	NA	-2.453831854	-1.564645542	0.241314091	0	0	7.63420844
Dokdonia_sp._Dokd-P16	NA	NA	0.294037242	NA	NA	NA	0.373674467	0.512185272	0.299371042	0.360310412	-1.469264743	0.182448585
Microbacterium_sp._L-031	NA	NA	0.313903924	NA	NA	NA	-11.54021524	0.266994764	-0.086898298	-0.425305459	-8.776982369	-2.232662368
Acinetobacter_sp._10FS3-1	NA	NA	0.313951374	NA	NA	-2.429384269	1.01482744	0.372900202	0.684313353	-2.167659601	-0.651338766	-1.110514994
Tolypothrix_sp._PCC_7910	NA	NA	0.321928095	NA	NA	NA	0.007771015	-0.947714969	-1.4051204	-2.361728031	-5.332183756	-1.165632957
Comamonas_koreensis	NA	NA	0.334577767	-1.175480245	NA	NA	-10.30543479	-0.337027187	0.243560463	-0.310409435	0.115676007	-0.928927978
Corynebacterium_sp._LMM-1652	NA	NA	0.340228912	NA	-0.881428587	-0.975694093	0.540675684	0.689703282	0.744093033	-2.314916198	-1.741462346	-2.5553962
Rhodococcus_triatomae	NA	NA	0.343346914	NA	NA	-0.7825752	-9.222246695	1.72506149	-1.477110226	11.25108588	9.692324318	9.700439718
Proteus_sp._GOKU	NA	NA	0.344473207	NA	-2.211153239	-0.724031118	0	8.110425888	0	-1.12911357	-0.204066971	-0.758409483
Rhodococcus_sp._WAY2	NA	NA	0.369098122	NA	NA	NA	-2.979835972	-3.820201799	-3.377603245	0.221735947	2.162532441	1.675762983
Pseudoalteromonas_sp._A25	NA	NA	0.387067681	NA	-2.261437621	0.50372157	10.71658799	12.49025924	11.41372312	-0.952650893	-0.585675377	-0.195400323
Mycoplasma_neurolyticum	NA	NA	0.400138623	NA	NA	NA	0.603044016	0.043458815	-0.095825178	3.078711976	2.939771425	2.458205358
Variovorax_sp._SRS16	NA	NA	0.410074663	-0.975151038	NA	-0.368669975	-0.97320669	-1.528125717	-0.447242311	-0.93548209	-2.08556619	-10.98441846
Actinotalea_sp._JY-7876	NA	NA	0.44516144	2.564174028	-0.10596293	-0.267996389	0	0	0	-7.807354922	-7.807354922	-0.426530253

Acidovorax_sp._RAC01	NA	NA	0.456567233	NA	NA	NA	-0.418816424	-0.228350271	-0.236329573	0.668702101	-2.570659131	-0.241922092
Streptomyces_nigra	NA	NA	0.467778961	-1.28077077	0.276373786	-1.154926837	-9.222246695	-9.222246695	-0.207464616	-1.944146765	0.918323459	1.627568442
Yersinia_ruckeri	NA	NA	0.469991601	-2.519450758	-0.379354431	0.934580873	1.556107876	1.64175914	0.947889172	-0.243699995	-0.491354157	-0.707696668
Lentibacillus_sp._CBA3610	NA	NA	0.472320415	-4.497316379	-2.815949394	-0.002338874	-1.055968101	-0.66278165	1.013109401	-0.615460283	-0.266200599	-0.262171059
Streptomyces_alboniger	NA	NA	0.474318437	NA	NA	NA	0.868878775	0.711651977	-0.139142854	0	0	6.930737338
Nitrobacter_hamburgensis	NA	NA	0.482180356	NA	NA	-0.677210852	9.029913869	0	6.15711649	1.136472097	-0.123258335	2.286046351
Lactobacillus_hordei	NA	NA	0.502795491	0.83353174	NA	-1.732687038	-0.054262533	0.970767304	0.223512428	-0.221362072	-4.392730491	-1.344273563
Silicimonas_algicola	NA	NA	0.507091591	4.062778867	2.13774911	0.893500379	-0.000325476	0.167731087	0.802676239	2.355536798	-0.321933891	2.429130337
Candidatus_Profftella_armatura	NA	NA	0.56116493	NA	NA	0.507493688	0.30129765	0.292994966	0.381819168	8.366322214	9.092051475	8.513068266
Sphingomonas_sediminicola	NA	NA	0.573112783	NA	NA	NA	0	0	0	0	9.914485295	8.996239023
Mycoplasma_canis	NA	NA	0.584727438	NA	-2.799659131	-1.198506868	8.166278594	11.50454591	11.50815626	-0.231252519	0.381064184	0.548573419
Sphingomonas_taxi	NA	NA	0.638525777	0.674857581	NA	NA	0	0	0	-8.076815597	-8.076815597	-8.076815597
Xylanimicrobium_sp._FW10M-9	NA	NA	0.639024235	2.077679364	0.136573053	NA	-1.486085732	1.580279352	2.147001575	1.652146522	1.004445905	1.561522263
Streptomyces_spongiicola	NA	NA	0.646713552	-2.184083064	NA	-2.63076619	0	0	0	-0.405875203	-0.893734747	-1.16533595
Nocardia_sp._WCH-YHL-001	NA	NA	0.647467443	NA	NA	NA	-9.838345161	2.377573032	2.060841801	1.249184371	0.589162489	0.904364721
Novosphingobium_sp._ES2-1	NA	NA	0.687810908	NA	1.77909107	1.503547669	8.6039638	0	10.3198753	9.027905997	0	9.403721476
Acinetobacter_lanii	NA	NA	0.71357179	NA	NA	NA	0.321697364	0.942676818	0.755469097	0.553550658	1.880306869	-0.238387599
Pseudomonas_sp._OIL-1	NA	NA	0.746024933	1.506066277	NA	0.063927327	0.846647135	2.753536495	-1.600200174	-2.186561479	-0.616213959	0.628489362
Nocardioides_sp._HDW12A	NA	NA	0.750085354	NA	NA	NA	8.6039638	0	0	8.982993575	6.470320477	9.560969008
endosymbiont_of_Plagiopylea_sp.	NA	NA	0.782289585	NA	NA	NA	-0.421700802	-0.205443562	0.23210076	8.033423002	0	0
Sphingomonas_hengshuiensis	NA	NA	0.815623225	NA	NA	NA	-0.288983575	-0.275573309	-0.100574271	0	7.222389201	7.63420844
Helicobacter_apodemus	NA	NA	0.823699354	-8.274567836	-6.709492134	-6.908404301	0.327200395	0.657166819	0.64964937	-1.271392145	-0.94471052	-1.161661978
Pseudoalteromonas_sp._R3	NA	NA	0.853275842	-1.106915204	-1.974464908	-0.034506708	0.411426604	-8.091313724	1.996387779	-0.953776702	-2.031514906	0.4778676
Pseudomonas_brenneri	NA	NA	0.853758882	NA	NA	NA	0	0	0	-1.158829543	-1.062611219	-9.431486483
Pseudomonas_sp._URMO17WK12:I11	NA	NA	0.862637358	4.30496862	1.86713514	0.89984836	10.0877015	0	9.052320788	-1.226684521	0.217121387	0.395622575
Streptomyces_pristinaespiralis	NA	NA	0.864344901	NA	NA	NA	0.794311369	0.654894623	0.550575536	-0.123663851	-7.544323092	1.352514847
Winogradskyella_schleiferi	NA	NA	0.873880863	-3.826502764	-2.805892484	-2.811017516	-0.30852445	-2.336443935	-0.975256777	-0.42897669	0.706828046	0.333905322
Weissella_soli	NA	NA	0.911778725	-3.069022492	0.009734821	-0.221632908	0.48682835	1.61089954	-1.078655678	-2.075928642	-1.128672506	-0.589959923
Capnocytophaga_sp._FDAARGOS_737	NA	NA	0.918205909	NA	NA	NA	0	9.426646347	0	-1.508342774	1.26257666	-9.781359714
Methylobacterium_sp._DM1	NA	NA	0.99045378	NA	NA	NA	0.440182802	0.581157804	0.396896581	7.303780748	7.59792591	0
Mesorhizobium_sp._M4B.F.Ca.ET.058.02.1.1	NA	NA	1.036836768	NA	NA	NA	1.045725523	0.465561765	-0.574049582	0	5.472487771	8.952254929
Glycocaulis_alkaliphilus	NA	NA	1.076825339	NA	-0.158181668	-2.276413436	0.596244267	0.06487324	0.833832547	0.035544915	-0.418312631	1.231480476
Candidatus_Kinetoplastibacterium_sorsogonicusi	NA	NA	1.094215342	NA	NA	NA	-0.032387824	-0.051389556	0.040142999	-8.087462841	-8.087462841	-8.087462841
Ketobacter_alkanivorans	NA	NA	1.127333575	NA	-1.279624625	-3.025172606	0.271858125	-2.041394452	1.182935727	-0.902138975	-0.210655092	-3.683355917
Geobacillus_thermodenitrificans	NA	NA	1.148784587	NA	-0.069086738	0.59057313	-0.873763971	0.199008522	-0.30299624	-0.178174888	1.30697559	-0.065205772
Chromobacterium_haemolyticum	NA	NA	1.182765347	NA	NA	NA	1.053349401	1.526705047	2.669711639	8.45395786	0	0
Thermotoga_profunda	NA	NA	1.218000652	NA	NA	NA	0	0	9.819597937	7.129283017	0	0
Marinobacter_sp._es.042	NA	NA	1.235480587	NA	NA	NA	-0.36591156	-0.01762813	1.15053853	0	0	0
Chryseobacterium_arthrosphaerae	NA	NA	1.265429533	NA	NA	-1.892305803	0.378847938	1.502609063	2.311225981	-0.499414662	-3.621551222	-2.341446079
Nocardia_sp._CS682	NA	NA	1.270558882	2.51735969	2.604071324	1.647698256	0	0	0	3.134775703	0.528385586	0.141790045
Weissella_viridescens	NA	NA	1.276067302	NA	-2.209357086	-1.136425564	-1.989294591	0.565242055	-1.45800645	2.332654501	0.502499707	0.760459297
Helicobacter_heilmannii	NA	NA	1.361466927	NA	NA	NA	-1.240043825	-0.869902628	-1.298402097	-1.192017177	-0.577993564	-0.684076538
Pseudomonas_nitroreducens	NA	NA	1.362570079	NA	NA	NA	0	0	8.30544163	0.489099909	1.371756398	2.276919944
Bradyrhizobium_paxllaeri	NA	NA	1.396575257	NA	NA	NA	0.195856357	0.008813874	0.459082281	-0.627573908	-2.767787787	-0.649036221
Rhodoferax_antarcticus	NA	NA	1.443707838	NA	-1.937498582	2.20259119	0	0	0	0.40511801	0.921543563	0.348410905
Porphyrobacter_neustonensis	NA	NA	1.498691049	NA	NA	NA	0.715715368	0.298407766	0.019082183	0.30394921	-0.3720265	-1.038928832
Borrelia_hermsii	NA	NA	1.504581401	NA	NA	NA	-8.10567284	-8.10567284	-8.10567284	0	5.472487771	0
Oblitimonas_alkaliphila	NA	NA	1.507913045	-2.931890571	-0.230098115	0.951050648	2.963936358	1.599036262	1.858395725	1.99684234	1.861442438	2.6466632
Thalassobius_gelatinovorus	NA	NA	1.514547593	NA	NA	NA	0	0	8.548995253	0.736765891	-0.937285677	0.71290539
Psychrobacter_arcticus	NA	NA	1.520496494	1.193964013	NA	1.543314803	0	9.3781973	0	2.787963568	1.458205358	-7.741466986
Pseudomonas_thivervalensis	NA	NA	1.525572172	NA	1.13589584	1.118702636	0	0	0	-10.66829112	-1.140814118	-10.66829112
Deinococcus_actinosclerus	NA	NA	1.53888663	0.243783927	0.53671215	0.890794803	-2.601607896	1.485047945	0.042974297	-0.9173499	0.222031432	-0.423767191
Neisseria_wadsworthii	NA	NA	1.540794917	-1.617907961	-1.579960086	0.874423562	0	10.44275693	9.691425987	0.103390804	0.556846045	0.699775554
Rhizobium_lusitanum	NA	NA	1.555127271	4.463571974	5.224491323	4.847346627	-2.636342889	-2.655200018	0.206700571	-1.050245478	-2.197952684	-0.954030163
Sphingomonas_sp._AE3	NA	NA	1.593432359	NA	NA	-1.470016369	-0.745563489	-0.792265843	-0.333261149	0.577197633	-0.742699291	-1.873460392
Francisella_sp._LA112445	NA	NA	1.641330315	-2.130502024	0.528638894	1.896716951	0.287781339	-0.306407174	0.453337976	7.333897756	0	6.544320001
Campylobacter_canadensis	NA	NA	1.801018965	1.190235007	0.07912865	1.348229056	1.525461995	2.088317518	1.768674454	-0.072149786	-1.873354016	-0.063364523
Neisseria_animalis	NA	NA	1.865590967	NA	NA	NA	0	0	0	-0.078080015	0.271988856	-9.442943496
Streptomyces_gilvosporeus	NA	NA	1.963672993	NA	NA	NA	1.166336141	0.701264226	-0.645335119	-9.415038204	-0.131178921	-0.780833394
Micromonospora_auratinigra	NA	NA	1.970684433	NA	NA	NA	0.706346948	1.993582744	1.903941817	1.626782676	1.580948847	-0.453254401
Campylobacter_curvus	NA	NA	2.018589026	NA	NA	-4.911730235	-1.393551698	-2.990893235	-0.820751857	-0.986855791	-1.087241965	-1.50458824
Candidatus_Liberibacter_africanus	NA	NA	2.030762254	NA	1.472633801	2.533628672	0	0	0	-0.801454321	3.567648178	-1
Marinobacter_adhaerens	NA	NA	2.057591422	NA	NA	NA	0.957975247	1.189692988	0.023478549	0.544321988	-0.438124021	2.884388597
Lysobacter_maris	NA	NA	2.068809158	NA	NA	NA	-0.82336941	0.489837721	0.994758165	9.855907186	8.225607527	10.31816994
Litorilituus_sediminis	NA	NA	2.081239848	NA	-0.35419857	-0.785436383	0.663965095	0.747107516	0.938284579	0.83344185	1.438856612	0.986830538
Burkholderia_anthina	NA	NA	2.082504504	3.985236647	4.470722359	4.066030729	-0.695518243	0.290284545	-0.686505175	-0.124786386	0.628668164	-0.979765516
Georhizobium_profundi	NA	NA	2.092353421	NA	0.275365208	NA	0	0	0	0	6.470320477	0
Myxococcus_stipitatus	NA	NA	2.152223	3.255249861	1.063528414	0.241581296	0	0	0	2.143929793	3.367152618	1.499465521
Nocardioides_mesophilus	NA	NA	2.152667804	NA	NA	NA	0.799055277	1.252225883	-0.535481039	-1.378363799	-1.954485892	-1.560908287
Nostoc_sp._PCC_7107	NA	NA	2.173295786	-0.877626001	-0.698076581	0.360817108	10.37325669	0	9.819597937	2.619612137	0.087462841	1.870951191
Erwinia_tasmaniensis	NA	NA	2.196397213	0.296856073	2.549406131	3.687916245	-0.029601869	-1.277805758	-0.713854121	-0.117098079	-10.0962803	0.503632538
Noviherbaspirillum_sp._UKPF54	NA	NA	2.256653485	3.60039591	4.181384618	3.874569869	1.198604943	-0.527916784	0.181203786	-0.081347244	1.163524362	-0.272068059
Pajaroellobacter_abortibovis	NA	NA	2.364677742	NA	1.031326334	1.920342743	-3.002018628	-2.658995042	-3.187057891	0.409563741	0.509169901	-8.044394119
Aquifex_aeolicus	NA	NA	2.4335824	-3.537717781	-3.479614827	-4.036380436	0.321016105	0.965827563	0.847802548	1.392085799	1.351344022	1.279121984
Blastomonas_fulva	NA	NA	2.526688839	3.426412289	4.024610976	3.349016149	-11.32397797	-2.716647656	-4.166863502	0.540196911	1.585871043	-0.230849678
Pseudomonas_marincola	NA	NA	2.592379972	-0.560539027	0.738372415	0.495445583	-8.624046047	0.003323679	0.656302523	0.999477334	0.376019259	0.573408797
Idiomarina_andamanensis	NA	NA	2.632125451	NA	NA	-0.114054328	0	0	0	0.093071952	-0.158076898	0.223413046
Mycoplasma_sp._Phocoena_C-264-GEN	NA	NA	3.059507471	NA	NA	-2.102051271	9.129283017	8.627369725	8.451211112	-1.545741191	-0.282477064	1.533244493
Vagococcus_sp._MN-17	NA	NA	3.093142264	NA	NA	NA	0.517499461	-3.047103762	1.658241915	0	11.21997316	0
Actinomyces_tangfeifanii	NA	NA	3.147006409	0.009257968	0.977378203	0.295685192	-2.235830968	-3.023459864	-0.614791584	-10.50250366	-0.964638808	-0.037621009
Corynebacterium_casei	NA	NA	3.227992371	NA	NA	NA	1.972776766	1.8108137	0.76553335	13.17169754	16.7686108	16.23149684
Marinomonas_sp._MWYL1	NA	NA	3.240252269	-3.925187657	-1.379182554	-0.980364037	9.029913869	0	0	-0.574838964	0.190204619	0.794410824
Chlamydia_sp._H15-1957-10C	NA	NA	3.403001628	NA	-1.154198609	-0.648590944	-0.521221272	0.523969877	-0.206132734	-0.389227865	0.69709441	0.945397341
Austwickia_chelonae	NA	NA	3.508647855	-1.039668615	0.362990752	0.485246502	0.037969293	-8.091313724	-8.091313724	1.161507958	1.241430505	0.554162601
Mesoplasma_florum	NA	NA	3.552192167	-1.468292226	-0.836215992	0.141116174	-1.53966339	-0.37368861	-0.814872358	-0.095389777	0.117197415	1.074752218
Halomonas_sp._PGE1	NA	NA	3.68311562	1.591672177	1.083302647	1.120899433	8.246199041	0	0	1.373763457	-8.963858445	-2.033121107
Tamlana_sp._UJ94	NA	NA	3.854448439	-2.210106048	-2.076000159	-2.007613184	-8.799135887	0.828232014	-1.053999418	-0.104469267	-0.939072696	0.986678622
Rickettsia_canadensis	NA	NA	3.930390058	NA	-0.861535308	-0.256858509	0.763271533	0.074873531	-1.395381726	0.240647046	0.721651768	1.328172036
Mycoplasma_struthionis	NA	NA	4.412240008	-3.814014335	-0.909530237	-0.361305098	0.863419255	-0.886144072	-1.361631693	-0.145952881	0.382948072	1.043553968
Flaviflexus_salsibiostraticola	NA	NA	4.49717102	NA	NA	NA	-0.657667547	0.306144997	0.063403705	-0.859696126	-0.530322256	0.889654747
Dyella_japonica	NA	NA	5.085580383	1.882896657	1.309065505	0.919412556	-3.538490495	0.160753911	-0.155252067	-0.923829516	-0.752673379	-0.635633845
Delftia_tsuruhatensis	NA	NA	5.188631196	-3.893344064	-0.763885952	-0.288095865	0.261610979	0.911577075	-0.256847343	-0.057505256	0.447593457	0.618691032
Helicobacter_canis	NA	NA	NA	-7.683659934	NA	NA	-0.051971612	-0.51749056	-0.592213847	-1.419267394	-1.171124163	-1.284357373
Mycoplasma_anseris	NA	NA	NA	-7.504873913	-7.763739741	-1.716121571	10.02990835	9.648765614	0	0.541996303	0.960608396	0.998096135
Legionella_sainthelensi	NA	NA	NA	-6.260833498	NA	-0.034675885	-2.847230032	-1.432091449	-3.658541483	0.856139407	0.654810871	0.016079755
Sulfurimonas_autotrophica	NA	NA	NA	-5.574095521	-7.931252114	NA	1.964980993	-0.171766162	-2.642019397	-0.018404264	0.256858208	-0.414198778
Paraglaciecola_psychrophila	NA	NA	NA	-5.536770243	NA	-5.611220192	0.844583862	-0.052797626	1.241915033	0.257405659	-0.284846464	-1.195766348
Solibacillus_sp._R5-41	NA	NA	NA	-4.363547184	0.469651461	-0.546485541	0.711641333	1.725240339	1.877045548	1.931863613	1.624128148	0.358788507
Bacillus_wiedmannii	NA	NA	NA	-4.032966772	NA	-2.235401167	-8.04736934	3.003098346	2.995657943	0.253771981	1.783899802	0.55298773
Polaribacter_sp._ALD11	NA	NA	NA	-4.015892585	-4.431446717	-0.98333337	0.801570824	0.730707839	-0.507684544	-0.922327505	0.541678456	-0.602412928
Chitinophaga_sp._1303	NA	NA	NA	-3.955165538	NA	NA	-2.424253145	-0.526131262	-0.766729895	0.201551361	1.4353702	0.78755302
Yersinia_hibernica	NA	NA	NA	-3.944375885	-3.390709515	-2.401294266	0	10.95261022	11.40117285	0	0	0
Staphylococcus_chromogenes	NA	NA	NA	-3.785743375	-5.528835072	-2.249216259	-1.354867138	-0.3610564	-0.386575341	-0.010521153	0.153749872	0.02977332
Thiosulfatimonas_sediminis	NA	NA	NA	-3.403006656	-4.395756917	0.689896902	-0.312746582	0.038042595	0.78211227	-0.182403414	0.845011679	0.915818444
Roseomonas_mucosa	NA	NA	NA	-3.397001502	NA	-2.104938047	0.2138964	0.204399652	-1.065127173	-0.223208304	0.054693082	-0.951466813
Campylobacter_sp._RM6137	NA	NA	NA	-3.196557148	0.942744625	NA	-1.002103259	-0.443058244	0.55925327	-1.023728483	0.954576445	-1.087133917
Pseudoalteromonas_aliena	NA	NA	NA	-3.17529209	-1.874986807	-1.123964828	-0.252732286	-0.342882492	-0.102007857	0	0	0
Parashewanella_tropica	NA	NA	NA	-3.111534752	NA	NA	1.361299657	0.291388341	1.500060837	2.262777493	2.08625102	2.175394942
Capnocytophaga_sp._oral_taxon_902	NA	NA	NA	-3.03818897	0.960262243	1.016488123	-9.953931598	1.768098888	0.939233394	0.64859265	3.523227366	2.280388316
Candidatus_Thioglobus_singularis	NA	NA	NA	-3.033906415	-2.295534	NA	0	0	0	0	0	8.412218392
Shewanella_oneidensis	NA	NA	NA	-2.855552215	1.85079639	2.195756216	8.166278594	0	10.81159144	1.702918094	1.307891679	1.550540845

Segniliparus_rotundus	NA	NA	NA	-2.676544808	-0.50829056	0.166329294	0.33321268	0.419250102	1.02568034	0.053936049	1.265700429	0.506243078
Lactobacillus_oligofermentans	NA	NA	NA	-2.611532221	NA	-1.220492311	-0.081857191	1.20096582	0.700272143	1.047462621	1.544862781	2.23991252
Sphingomonas_sp._KC8	NA	NA	NA	-2.267670816	2.676503955	-1.095683348	0	9.648765614	10.08649453	-1.827268272	-9.247927513	-0.613722704
Thermoanaerobacter_wiegelii	NA	NA	NA	-2.200207568	-1.637271374	-1.355858439	0.407389054	0.489775707	0.930891338	0.994848211	1.393899706	1.183494265
Dokdonia_sp._PRO95	NA	NA	NA	-2.199208937	NA	-1.109198771	-0.401594444	0.59703839	0.748534852	-0.407569231	-0.446230696	2.505665402
Kangiella_koreensis	NA	NA	NA	-2.160801208	NA	NA	-0.295116713	1.852240814	1.526237204	-9.485159115	-4.012671343	-9.485159115
Kitasatospora_setae	NA	NA	NA	-2.145501528	0.624300649	-0.906890596	0	0	0	1.431860184	1.039084619	-8.186525318
Pantoea_sp._PSNIH1	NA	NA	NA	-1.963207969	NA	-2.127952731	0.579277841	0	0.667699685	9.347251513	9.803054246	7.380824669
Flavobacterium_sangjuense	NA	NA	NA	-1.947109788	NA	-5.091019199	0.355870607	-1.295238279	-0.667408015	0.211595629	0.979176638	0.083536826
Citrobacter_sp._NMI7904_11	NA	NA	NA	-1.928932129	-2.286342811	-2.970220338	0	0	7.745136469	-2.310695811	-1.26539591	-10.58371275
Streptomyces_sp._Z022	NA	NA	NA	-1.857767468	2.057970889	1.916934769	0.664002864	0.684592884	0.297899166	0.028999801	1.876270395	0.476766174
Polaribacter_sp._KT_15	NA	NA	NA	-1.705056346	NA	0.757105065	1.599319871	0.853330995	1.679574772	-9.117209319	0.61524136	1.471505317
Bacillus_sp._JAS24-2	NA	NA	NA	-1.682604508	NA	-5.597028776	0	0	0	-0.92123731	1.237423985	0.282611773
Alteromonas_macleodii	NA	NA	NA	-1.674565962	1.152266335	-2.352077706	0.854869564	1.066425369	0.757079228	1.843342311	1.460953325	-3.336284413
Thauera_sp._K11	NA	NA	NA	-1.662418641	NA	NA	-0.339482303	0.282675202	0.118067387	0.299441155	-1.76818849	-0.003788497
Mesorhizobium_sp._NZP2298	NA	NA	NA	-1.603866458	-1.082562595	NA	-1.047639216	-0.330713993	-1.408716505	0	6.470320477	0
Pontimonas_salivibrio	NA	NA	NA	-1.544693498	NA	-2.374043342	-0.121382216	-0.067166787	0.518748327	0.278859373	0.454280421	1.038381953
Mycolicibacter_minnesotensis	NA	NA	NA	-1.479129589	-1.740757173	0.621109667	0	0	0	-9.14125651	-1.107833509	1.084348607
Pectobacterium_atrosepticum	NA	NA	NA	-1.477248683	NA	NA	0.405785635	0.302641448	2.001496018	9.027905997	8.91607949	0
Streptomyces_globisporus	NA	NA	NA	-1.436748497	0.654225163	-0.456084982	-8.552496194	2.055675446	0.759149959	-0.093500812	-0.003742547	-0.361172527
Spiroplasma_taiwanense	NA	NA	NA	-1.421526836	NA	-0.961665268	9.248359308	9.426646347	8.944644719	0.901169973	-2.304466582	0.177467869
Limnobaculum_parvum	NA	NA	NA	-1.221463747	-0.592413492	NA	0.499133515	0.57559599	1.614726841	7.303780748	0	7.539158811
Bosea_sp._AS-1	NA	NA	NA	-1.213883874	NA	NA	0	10.22005178	9.819597937	1.734987945	2.408848322	0.212002135
Staphylococcus_argenteus	NA	NA	NA	-1.198607756	-0.560557188	1.983497369	-1.723302362	-0.191665985	0.551892352	7.333897756	8.926791141	8.587466258
Streptomyces_sp._769	NA	NA	NA	-1.175692754	-0.90560359	-1.018906864	0.143800028	-1.01311329	-0.678543638	10.16741815	9.927382426	10.74258734
Mesotoga_prima	NA	NA	NA	-1.1716319	NA	-2.57718515	-1.049733723	0.142998447	0.604436194	7.129283017	8.225607527	0
Lactobacillus_paraplantarum	NA	NA	NA	-1.164057398	-0.285103679	-0.609048119	-1.174542864	0.137251217	1.496509356	0.787197154	-0.556002908	-3.444364686
Erythrobacter_seohaensis	NA	NA	NA	-1.160464672	4.199531464	4.063923988	-3.334981927	-0.512317324	0.193459904	0.883686098	0.666482582	0.419713986
Acidithiobacillus_caldus	NA	NA	NA	-1.152437575	1.580175995	-0.555933452	0	0	7.157114468	1.610601824	-0.743615301	1.16438854
Mycoplasma_hyorhinis	NA	NA	NA	-1.049784833	NA	3.835050594	0.623164684	0.251205105	0.418306808	1.037585954	1.281125553	0.753827683
Pseudothermotoga_hypogea	NA	NA	NA	-1.04722201	0.687926097	0.07462825	-0.508001178	-8.637284195	2.824195253	-0.067427848	-0.494213497	-1.103746085
Providencia_sp._WCHPHu000369	NA	NA	NA	-1.021005126	-3.698520329	NA	-1.622566565	-0.763288038	-0.282832105	1.703750026	1.620813518	1.21157026
Methylosinus_trichosporium	NA	NA	NA	-1	2.310739538	4.035925918	0	0	0	0.138731754	0.880564249	0.340328645
Nocardioides_sp._KUDC_5002	NA	NA	NA	-0.865484333	-0.883186335	-0.410538113	-0.511983078	1.454195308	1.670585759	8.874468093	9.541226355	8.89683794
Sphingobium_sp._SCG-1	NA	NA	NA	-0.830857159	NA	-0.426814667	-0.15556538	0.742973585	0.995000491	-7.807354922	-7.807354922	-7.807354922
Raoultella_planticola	NA	NA	NA	-0.819369107	1.78828598	0.152202485	-8.076815597	-8.076815597	-8.076815597	-0.431002422	0.307756693	0.670973615
Synechococcus_sp._PCC_7336	NA	NA	NA	-0.813458983	-2.456556769	-2.150298787	0.218949353	0.328294618	0.830362693	0.719983373	1.177612767	0.12297434
Paraglaciecola_sp._L3A3	NA	NA	NA	-0.808120887	NA	NA	-0.416353209	-0.433347629	0.761745624	0	0	0
Vibrio_europaeus	NA	NA	NA	-0.805054584	NA	NA	8.157119522	9.384097309	7.157114468	0.046805694	-4.186914158	-0.556114121
Arthrobacter_sp._Rue61a	NA	NA	NA	-0.745245072	-0.44633363	NA	-1.219157627	0.550367836	0.347855234	0	9.214319121	8.520944699
Mycoplasma_bovoculi	NA	NA	NA	-0.745245072	0.07539307	NA	-0.150666532	-0.307826229	-0.256833985	0	8.215935782	7.380824669
Methylorubrum_extorquens	NA	NA	NA	-0.745245072	2.07979377	NA	-0.602378065	1.230895419	0.042932377	2.557546445	1.153968626	-1.104332885
Chlamydia_pecorum	NA	NA	NA	-0.729210754	NA	-1.374927286	-0.134543867	-8.637284195	-8.637284195	2.271871908	2.173625459	-2.220167983
Pseudomonas_palleroniana	NA	NA	NA	-0.687456494	NA	-1.693262116	0.849903489	1.249109668	0.508625418	0	0	8.125842211
Bradyrhizobium_sp._CCGE-LA001	NA	NA	NA	-0.603866458	NA	0.032079971	0.391671044	-0.063998833	-0.168729608	8.442943496	7.996236198	8.741466986
Neisseria_flavescens	NA	NA	NA	-0.588819976	0.155081665	0.466593144	0	9.627367901	0	0	0	0
Pulveribacter_suum	NA	NA	NA	-0.560539027	NA	0.704224193	0	0	10.91472147	0.708001242	-7.745956618	-7.745956618
Pseudomonas_balearica	NA	NA	NA	-0.560539027	NA	1.393322435	0.145454112	-0.532716473	-0.341114534	8.366322214	0	0
Nitrobacter_winogradskyi	NA	NA	NA	-0.560539027	NA	NA	1.532498086	0.495352102	-0.27643025	0	0	0
Lelliottia_sp._WB101	NA	NA	NA	-0.539928747	0.078743115	-0.295775807	-0.710905245	0.438804051	0.945878447	-1.074382196	-1.640388423	-2.705887283
Sulfitobacter_sp._D7	NA	NA	NA	-0.498041554	-2.088928888	-0.930528024	-9.222246695	-9.222246695	-1.065127173	-1.782810831	-1.39034986	-1.558207557
Bosea_vaviloviae	NA	NA	NA	-0.492844299	-1.406256614	-0.741502375	-0.730797008	-0.607356023	-0.331308099	0	8.148390145	8.996239023
Raoultella_ornithinolytica	NA	NA	NA	-0.476132947	-2.732627662	-1.353859765	0	0	6.15711649	-7.741466986	2.954344182	-7.741466986
Microbacterium_sp._WY121	NA	NA	NA	-0.431805713	NA	3.220329955	-1.153005712	-0.732653274	-0.828660501	-1.076725791	-0.679990643	-0.842260537
Curvibacter_sp._AEP1-3	NA	NA	NA	-0.431805713	NA	NA	0	10.40262839	0	-7.700439718	0.597242359	2.730012834
Candidatus_Chazhemtobacterium_aquaticus	NA	NA	NA	-0.415701876	-1.419028359	-1.643420358	0	0	0	0.684396697	0.451794879	0.001103108
Thauera_humireducens	NA	NA	NA	-0.412353415	1.109575346	0.57823036	-1.07900295	-0.410770922	-0.242817772	-0.308123927	-1.387020487	2.329092734
Staphylococcus_capitis	NA	NA	NA	-0.405894087	0.07953274	0.662509767	-8.552496194	1.837479243	1.294418598	0.347520067	1.134248851	0.945505424
Pseudomonas_sp._BIGb0427	NA	NA	NA	-0.391982263	NA	NA	1.702319852	2.928844125	3.104009114	8.442943496	9.238404739	9.042570472
Paraphotobacterium_marinum	NA	NA	NA	-0.369167608	NA	-2.872352177	-0.658438443	-0.424719681	-0.186670134	-0.249064449	-1.305773933	0.309920269
Duganella_sp._GN2-R2	NA	NA	NA	-0.285165754	NA	-1.447803255	-9.791755477	1.042280961	0.121949261	0.155467468	-0.275574301	-0.639743786
Methylobacterium_aquaticum	NA	NA	NA	-0.279223644	0.55366637	1.826684865	-0.25700315	0.207212537	-0.059405985	3.075516637	-2.268979215	-7.741466986
Nocardioides_sp._JS614	NA	NA	NA	-0.279223644	NA	0.797591953	-0.709098786	-9.739012655	0.371405402	0	7.65343985	0
Pseudarthrobacter_phenanthrenivorans	NA	NA	NA	-0.279223644	NA	1.400256456	1.067391135	0.778691528	0.392116973	-0.651795564	-8.924812504	2.541095659
Blattabacterium_sp._(Cryptocercus_kyebangensis)	NA	NA	NA	-0.278600663	NA	NA	0	0	0	0	0	0
Marinomonas_sp._SM1966	NA	NA	NA	-0.263460671	-0.741233926	0.122508786	0	9.3781973	7.157114468	-9.489179631	-0.679678365	-1.95002082
Pseudomonas_sp._IzPS59	NA	NA	NA	-0.263460671	NA	NA	-0.379883394	-0.195791568	0.660736427	9.99905574	9.067883368	8.634204809
Mycobacterium_heidelbergense	NA	NA	NA	-0.242074787	-1.326073773	1.312652952	-8.10565284	0.460727555	1.634875315	0	10.50051328	9.821243036
Lactobacillus_alimentarius	NA	NA	NA	-0.228685136	-0.608904222	0.662315026	9.147899389	8.559465045	8.965784285	0.082753498	0.359514817	0.763262315
Staphylococcus_schleiferi	NA	NA	NA	-0.213333082	NA	-1.55763487	8.6039638	10.05970948	10.63067666	-0.811915629	0.442121616	-0.12891948
Terricaulis_silvestris	NA	NA	NA	-0.207488414	0.377474086	0.356565097	-9.177646265	-0.55027654	1.479270139	0.153350777	-7.741466986	-1.197146985
Hoeflea_phototrophica	NA	NA	NA	-0.202333237	NA	-0.804153782	0.065825552	1.074453815	-0.643722256	-0.245832095	0.110990436	-1.777354328
Rickettsia_felis	NA	NA	NA	-0.157919828	NA	0.891721548	-1.603108027	-0.120621007	-2.024250152	0	7.222389201	8.520944699
Serratia_symbiotica	NA	NA	NA	-0.11146043	0.422393087	1.297044099	-2.195076605	-0.006039827	-1.464648088	1.683398431	-2.268979215	2.183345517
Curtobacterium_sp._BH-2-1-1	NA	NA	NA	-0.047195168	NA	NA	0.14497802	0.263546142	0.634580433	0.078789482	-8.818048457	-1.183840017
Rhodococcus_jostii	NA	NA	NA	-0.045166316	-1.356934545	-2.197358232	0	0	0	-8.044394119	2.168308958	2.916092975
Halarcobacter_bivalviorum	NA	NA	NA	-0.039052777	NA	NA	0	0	0	9.978238539	9.46216073	11.40158887
Pseudoalteromonas_atlantica	NA	NA	NA	-0.020137646	NA	NA	0	0	0	-9.055282436	-3.582794664	-9.055282436
Oleispira_antarctica	NA	NA	NA	0	1.024423474	-0.353050612	0.33813689	-1.354719046	-1.509496928	-9.875492884	-0.204247286	-1.685668325
Mycobacterium_florentinum	NA	NA	NA	0	NA	1.292063454	-0.44588475	-2.367176941	-0.10520383	0	0	0
Streptomyces_chromofuscus	NA	NA	NA	0.032625147	0.311994926	-0.033380038	0	0	9.154818109	-0.972467311	-0.195234723	-0.587385994
Legionella_spiritensis	NA	NA	NA	0.036674447	NA	-2.345898657	8.502740328	6.682671608	10.12693673	1.372090651	1.2916643	-0.020178417
Stenotrophomonas_sp._WZN-1	NA	NA	NA	0.183827764	-0.985159509	-1.243140687	-12.14065406	-2.369765559	-3.835212425	0.631523139	-1.716204841	1.561667531
Spongiibacter_sp._IMCC21906	NA	NA	NA	0.296856073	NA	-0.483267104	-0.423213177	-0.442070306	1.353027702	9.14974712	9.33687918	8.952254929
Micromonospora_krabiensis	NA	NA	NA	0.301691469	-0.909974546	-0.164484879	0.405867823	0.336483039	0.218590293	-0.326003165	0.333900737	-0.212822234
Sphingomonas_sp._PAMC26645	NA	NA	NA	0.303038201	-0.762605415	-1.086088977	-0.129674992	0.255226775	1.102329488	1.502754724	-0.602033343	1.608813782
Candidatus_Carsonella_ruddii	NA	NA	NA	0.402627646	NA	-1.033015057	0	0	0	-0.452166292	-1.12870533	-2.560154693
Weissella_jogaejeotgali	NA	NA	NA	0.442931505	-1.771807343	1.085067715	0.613190788	1.376776304	1.722750692	-1.258300802	1.371997586	-1.675278956
Borrelia_sp._A-FGy1	NA	NA	NA	0.449202114	NA	NA	-1.22531808	-0.440753158	-0.054881115	2.456474018	3.317248006	-8.886711698
Candidatus_Cytomitobacter_primus	NA	NA	NA	0.452011095	-1.900391958	-0.906483572	0	0	0	0.955782828	1.513861084	0.040076008
Ferriphaselus_amnicola	NA	NA	NA	0.482378566	-1.174451478	-0.56673084	-0.457792683	-0.236864724	-0.096727683	1.002799855	1.78906432	0.985930757
Bosea_sp._Tri-49	NA	NA	NA	0.490554136	1.972778531	-0.730239731	0.327634193	-0.18856979	2.364676638	-7.807354922	-0.584965721	1.499465521
Arthrobacter_sp._KBS0702	NA	NA	NA	0.520715577	0.86710573	-1.390875449	0	0	0	7.129283017	9.788990698	0
Acidihalobacter_ferrooxydans	NA	NA	NA	0.546051404	NA	NA	-1.24739684	-0.574284067	-0.084892338	8.641448488	8.033423002	0
Pseudoalteromonas_paragorgicola	NA	NA	NA	0.57392845	NA	NA	0.807622478	1.320053809	0.528353449	0.438542767	-1.005134792	-9.552029252
Aliarcobacter_faecis	NA	NA	NA	0.590355759	0.228170142	-1.521537121	0.026112613	1.64025119	-0.916805065	-0.516006033	-0.811997829	0.043391819
Vibrio_chagasii	NA	NA	NA	0.59487797	NA	NA	-0.397686663	0.267437278	-0.232173491	0	0	9.020516091
Leptolyngbya_sp._NIES-3755	NA	NA	NA	0.594885993	-1.970431978	-2.433310898	0	0	0	0.054537284	-0.065504045	-0.221587121
Mycoplasma_maculosum	NA	NA	NA	0.610538747	0.685384111	0.552541023	1.091986723	1.051760942	0.607039218	-2.126530203	0.848634049	-0.897557634
Hydrogenophaga_crassostreae	NA	NA	NA	0.616085018	0.909584705	-2.736965594	0	0	6.15711649	0.77160761	0.9622644	1.259967823
Mycoplasma_alkalescens	NA	NA	NA	0.645335119	0.022431046	-0.073784269	-9.681078494	-9.681078494	-2.523964026	-1.118883456	0.926368479	-0.589463883
Leuconostoc_lactis	NA	NA	NA	0.803857478	NA	NA	-0.833358028	0.922750274	-1.573648179	-1.844482807	-1.881714907	0.12222489
Parageobacillus_thermoglucosidasius	NA	NA	NA	0.81521704	NA	-2.260199105	0.453759265	0.000683101	1.018546464	1.004286288	0.995700939	1.25334101
Rhodococcus_sp._PBTS_2	NA	NA	NA	0.844417016	NA	0.490780062	0.953098272	0.530514717	-8.076815597	0	0	0
Allokutzneria_albata	NA	NA	NA	0.864944015	2.035026319	2.249758474	-0.177843098	0.085522505	0.834274777	1.298081353	0.421820377	1.219860416
Rathayibacter_festucae	NA	NA	NA	0.870347897	-0.877424452	-1.94024056	0	0	0	-0.400197846	-1.254670099	-3.881944754
Mycoplasma_sp._(ex_Biomphalaria_glabrata)	NA	NA	NA	0.880368211	-0.047508926	2.249120289	0.064858816	0.540011863	0.665245586	-1.141947411	-0.598543183	-0.787610187
Salinimonas_lutimaris	NA	NA	NA	0.914236477	-0.111361109	-1.44670972	0	0	0	0.478792188	-1.243848233	-2.7447051
Sphingomonas_melonis	NA	NA	NA	0.93047087	NA	-3.399505754	0.301665277	-0.051072838	0.651050005	-0.24015524	-0.187291173	1.077011712
Bdellovibrio_sp._ZAP7	NA	NA	NA	0.942093874	NA	1.703531366	0	0	10.2919074	0.321928095	-8.044394119	-8.044394119
Burkholderia_vietnamiensis	NA	NA	NA	0.974082626	NA	-1.236733444	0.006378768	1.366044744	0.604454802	7.129283017	0	7.380824669

Luteibacter_pinisoli	NA	NA	NA	1.050140644	2.952151706	2.929698639	-2.748813719	-1.454961315	-1.020928966	-0.686070797	-2.216945822	-1.925282287
Methylobacterium_mesophilicum	NA	NA	NA	1.146649859	0.736965594	NA	-0.282233615	0.291413179	-0.997804335	0	0	0
Planococcus_halocryophilus	NA	NA	NA	1.159409142	NA	-1.749144503	-0.141111326	-0.132945339	-0.238830758	2.510323253	2.515949167	-7.709655951
Geobacillus_sp._GHH01	NA	NA	NA	1.186061517	NA	NA	0.09884121	2.923211811	1.906196132	-8.076815597	-0.265171176	-1.146078259
Cronobacter_turicensis	NA	NA	NA	1.193964013	0.982297998	-0.725283789	-0.359734507	-0.933825865	-0.876087706	-0.847996907	-0.313157885	-2.983157005
Thauera_hydrothermalis	NA	NA	NA	1.203308061	NA	NA	-0.765715548	0.143901816	0.285670503	7.420659242	9.400309502	0
Thalassospira_xiamenensis	NA	NA	NA	1.206450877	2.687467139	2.632543565	1.287221416	0.503916331	0.214036434	-1.925612465	-0.488716209	0.531484525
Labrenzia_sp._CP4	NA	NA	NA	1.220087796	NA	-0.529619467	-0.809493167	-1.579836728	0.092254565	-2.654786931	-0.924391908	0.653917164
Streptococcus_constellatus	NA	NA	NA	1.236399279	3.194732835	0.181606806	1.788237985	2.589392373	0.428274741	0	0	7.63420844
Synechococcus_sp._A18-40	NA	NA	NA	1.283888734	0.982463519	0.590315735	-0.816099254	1.504779479	2.153676428	-9.018664771	-0.304419254	-9.018664771
Arthrobacter_sp._UKPF54-2	NA	NA	NA	1.288656923	NA	-0.185189273	0.557105602	0.429231111	0.117858862	1.085860737	-1.129994545	2.430689234
Nocardia_seriolae	NA	NA	NA	1.326041652	-1.235888264	-2.076311951	0.648678963	0.859423143	-2.065132227	0	7.369230901	8.741466986
Micromonospora_siamensis	NA	NA	NA	1.337328188	NA	NA	-9.222246695	-9.222246695	2.049111278	0	0	0
Brevibacterium_sp._CS2	NA	NA	NA	1.441291005	0.783662971	0.343737666	1.08474666	0.518009436	1.333303684	-0.652425084	-0.05030257	-0.97305854
Vespertiliibacter_pulmonis	NA	NA	NA	1.522582337	-1.831260938	-1.148451114	-0.063410333	-0.565135448	0.141691371	1.240231443	0.762674529	2.150890248
Mycoplasma_canadense	NA	NA	NA	1.526143856	0.357156583	1.572299265	0	8.110425888	11.29294851	-1.851654916	-2.571571875	-1.723722997
Bacillus_paranthracis	NA	NA	NA	1.596920965	NA	NA	-0.423213177	-8.552496194	-8.552496194	0	0	11.3593817
Mycoplasma_sp._Mirounga_ES2806-GEN	NA	NA	NA	1.617752436	2.179108324	2.694560028	0.842168012	0.254283965	0.582106976	-0.970413389	-0.693164409	0.390407731
Rathayibacter_sp._VKM_Ac-2804	NA	NA	NA	1.646639397	NA	NA	-9.552494273	0.771483697	0.398790442	7.420659242	0	0
Geobacillus_thermoleovorans	NA	NA	NA	1.652601218	1.963967269	0.832957507	0.177555553	0.379421352	-0.303612288	0.662963553	-0.641549636	1.504131426
Ureaplasma_urealyticum	NA	NA	NA	1.678319096	-0.548367041	-0.103381088	-0.210577015	-0.765069812	-0.529443851	-0.900808304	-1.193216866	-1.843186273
Vibrio_sp._HBUAS61001	NA	NA	NA	1.69540642	-1.390632187	-0.730118653	0.767764841	-0.280758959	-1.050222826	0	5.472487771	0
Pseudomonas_sp._S-6-2	NA	NA	NA	1.720888699	-0.327649299	1.791864458	8.409390936	9.39192664	7.157114468	2.820310497	-2.571906348	0.903973112
Actinomyces_pacaensis	NA	NA	NA	1.760784214	-0.298109475	0.91248355	-2.016202266	-2.292782849	-0.536082843	7.303780748	7.712410067	8.89683794
Cronobacter_sp._JZ38	NA	NA	NA	1.788658828	NA	NA	-0.25305395	-0.215289249	-0.767767262	0.256418178	-1.370604114	-8.186525318
Pseudomonas_sp._S150	NA	NA	NA	1.812748181	0.601082166	NA	8.129283017	0	6.15711649	0	9.975375111	6.544320001
Pseudomonas_cichorii	NA	NA	NA	1.812748181	NA	0.270220946	-0.930378479	0.217689588	0.677721854	-0.465910543	0.370614293	0.877571171
Eikenella_exigua	NA	NA	NA	1.843308186	NA	-2.295455884	0.386399094	-1.122999017	-0.0898868	0.099863894	-9.194756854	1.958795177
Legionella_geestiana	NA	NA	NA	1.854802084	-1.972561642	-0.06508351	8.157119522	0	0	1.594644451	1.930980991	1.176388058
Brevundimonas_sp._'scallop'	NA	NA	NA	1.854883292	NA	-0.936530086	0	0	0	-8.759332297	1.455800235	0.900069632
Epidermidibacterium_keratini	NA	NA	NA	1.856455874	NA	-1.097241128	-0.472248282	-1.735972944	0.000990826	-0.600214585	-0.565780407	0.444387339
Shewanella_sp._Arc9-LZ	NA	NA	NA	1.882969326	-0.450454408	0.061995593	-0.647983586	1.463931325	1.334574527	0.414041696	0.266350779	-9.916875182
Mycolicibacterium_alvei	NA	NA	NA	1.897856784	1.078855421	NA	8.129283017	0	9.921840937	2.004470678	-2.273468846	-0.206797806
Macrococcus_caseolyticus	NA	NA	NA	1.937093248	4.283921772	4.986350253	1.451207294	2.748750658	3.442016505	8.129283017	11.38499954	11.66474504
Ureaplasma_parvum	NA	NA	NA	1.938828037	0.188123147	-1.211083061	0.264245417	1.302592138	1.619886908	-0.557448763	-3.616290209	-4.07302478
Pseudoalteromonas_lipolytica	NA	NA	NA	1.942495798	-0.249294349	0.958568903	-7.757316664	-1.074645056	-7.757316664	-0.352529961	-1.003332755	0.189019027
Actinomyces_sp._oral_taxon_169	NA	NA	NA	2.071161029	0.357156583	0.717381198	-1.796763731	-0.253260527	0.692528215	0.812018867	1.016258265	-8.048034513
Sphingobium_sp._MI1205	NA	NA	NA	2.200056091	0.177962321	-0.529619467	0	0	0	-2.118644496	-1.128986441	-0.289859161
Mycoplasma_parvum	NA	NA	NA	2.289210305	-0.37003875	-2.452207292	-2.108341553	-0.735470932	0.057511605	-1.145130763	-3.630186152	-3.78471137
Litoreibacter_sp._LN3S51	NA	NA	NA	2.456443762	-0.883186335	0.316122473	1.248543346	-1.271707431	-0.076693969	1.109361339	-0.029895881	0.884870544
Exiguobacterium_mexicanum	NA	NA	NA	2.473435757	-0.44633363	1.570440283	-0.423213177	0.557920784	0.843329236	-0.106579539	-4.308871942	-9.781359714
Acinetobacter_sp._WCHA55	NA	NA	NA	2.476214837	NA	NA	-3.387372937	1.644138229	0.850747445	0	5.472487771	0
Yersinia_rohdei	NA	NA	NA	2.47942952	NA	-1.97139308	-8.552496194	-0.170365038	1.025961415	8.35167691	8.979187942	7.63420844
Streptomyces_sp._NEAU-S7GS2	NA	NA	NA	2.542002933	3.490050854	NA	-2.205751871	-1.65562247	-0.895028106	0	7.222389201	0
Mycoplasma_ovis	NA	NA	NA	2.584000383	1.191281889	-1.045596866	0.532925743	0.709353626	0.291809683	-0.905601558	-4.061716987	-3.908771082
Klebsiella_quasipneumoniae	NA	NA	NA	2.587891832	2.145478641	1.181606806	-2.529404524	0.331407344	0.197557445	1.18476507	0.307268377	0.745182948
Nakamurella_multipartita	NA	NA	NA	2.625804595	0.357156583	0.942119084	0	0	0	1.705254494	1.364310043	0.995510369
Geobacillus_thermocatenulatus	NA	NA	NA	2.744161096	NA	-0.348563221	-8.941980727	2.230559293	-1.196844258	-7.930737338	-7.930737338	1.021517591
Methylobacterium_currus	NA	NA	NA	2.746838653	0.275365208	2.411356411	9.955253221	0	10.46427005	-8.202941691	1.323230827	-8.202941691
Geobacillus_sp._WCH70	NA	NA	NA	2.798801369	-0.006648376	NA	-0.473004644	-1.756549942	-1.114805811	0.327321354	-1.838306731	0.246124511
Aminobacterium_colombiense	NA	NA	NA	2.925440244	0.416485262	NA	-0.019801434	0.442331261	0.780531407	0	10.76298823	8.587466258
Blautia_coccoides	NA	NA	NA	2.943905438	NA	NA	-11.18385865	-11.18385865	-1.132772095	-9.06608919	-9.06608919	-0.545144492
Mycolicibacterium_psychrotolerans	NA	NA	NA	2.957991503	-1.304955016	NA	0	0	0	10.33464201	9.505150581	8.89683794
Streptomyces_griseoviridis	NA	NA	NA	2.973364873	2.525091045	-1.102051271	0	0	0	0	8.776982369	0
Citrobacter_sp._RHB25-C09	NA	NA	NA	2.991684732	2.08375135	1.260267972	-1.197888319	-3.056901131	0.004377232	-1.215900948	0.26838444	-2.642013106
Vibrio_astriarenae	NA	NA	NA	3.121698422	NA	NA	-0.183751836	-10.80280714	-1.858162418	0	7.222389201	8.520944699
Leclercia_sp._1106151	NA	NA	NA	3.159985833	0.922452238	1.60423888	-9.95921229	0.567727027	-0.67886372	-0.700962055	1.193387004	0.072195026
Marinomonas_primoryensis	NA	NA	NA	3.413081784	2.11897175	3.259476586	0	0	0	0.660514791	0.771325746	1.141047429
Pseudomonas_rhizosphaerae	NA	NA	NA	3.431541272	2.789685646	2.017005293	-1.686576872	-0.886401726	-1.814597791	8.45395786	7.058890082	10.12670447
Enterobacteriaceae_endosymbiont_of_Donacia_cincticornis	NA	NA	NA	3.435159095	NA	1.177708808	-9.222246695	-9.222246695	-9.222246695	-1.237578825	-1.36149062	-1.153962006
Litoricola_lipolytica	NA	NA	NA	3.477610628	NA	0.848340856	-1.285319057	-0.603875195	-0.741417706	1.619318062	-0.155543451	-0.54830403
Halomonas_sp._THAF12	NA	NA	NA	3.487465972	-0.31836148	1.046383865	-1.699032476	1.188241535	1.869859902	0	9.319823382	9.707359132
Mycobacterium_leprae	NA	NA	NA	3.53921691	NA	0.800390974	0.063078884	-2.663640444	1.032871343	2.365050452	1.809670814	0.630175795
Candidatus_Planktophila_lacus	NA	NA	NA	3.669386937	NA	1.482327589	1.330448727	-0.1296955	2.61852624	8.920850045	9.727920455	0
Microbacterium_sp._NY27	NA	NA	NA	3.693995375	NA	1.06950451	1.888338227	3.411793595	4.316052642	-9.857980995	-9.857980995	-9.857980995
Sporosarcina_psychrophila	NA	NA	NA	3.705414743	-0.894891836	1.120452242	-0.07189706	-1.75192209	-1.012223236	-0.173331603	2.270201693	1
Nocardioides_sp._zg-579	NA	NA	NA	3.827286201	0.357156583	NA	0	0	6.15711649	8.89683794	9.543805176	8.412218392
Jiangella_sp._DSM_45060	NA	NA	NA	3.865522959	1.873419503	0.856520979	-0.791880862	-0.147880973	-0.234722358	0.563188956	-0.668379703	-0.256560464
Tabrizicola_piscis	NA	NA	NA	3.9253475	0.89403707	0.090010166	-0.584962501	-9.714245518	-3.557129028	-0.206231809	1.617574461	0.731212761
Ensifer_sojae	NA	NA	NA	4.086013209	NA	0.181606806	1.177115549	2.205362721	2.321981859	-2.2327824	-2.837520438	-9.653441641
Novosphingobium_sp._P6W	NA	NA	NA	4.187051389	-0.401616984	NA	-3.26523098	-0.228300136	-1.124293646	0	8.912889336	6.930737338
Pandoraea_fibrosis	NA	NA	NA	4.316145742	1.591360272	0.30723421	-7.757316664	-7.757316664	-7.757316664	-0.612183969	2.404044896	2.071244611
Brevibacterium_luteolum	NA	NA	NA	4.338243821	-0.44633363	3.01654529	8.876516947	10.60901248	10.77688535	-0.85746452	-1.84968642	1.255706129
Rhizorhabdus_dicambivorans	NA	NA	NA	4.419453009	0.663061737	0.969352606	1.237778985	1.42112943	1.841376869	9.367779474	7.902875524	8.189824559
Methylobacterium_sp._17Sr1-1	NA	NA	NA	4.483364361	0.994217647	1.593911457	-9.372270887	0.137684196	-0.653347637	0	8.297682077	0
Mesorhizobium_sp._N3	NA	NA	NA	4.698888933	0.177962321	0.993942489	0.122049213	1.144075519	-0.358342306	2.146841388	-1.337034445	2.559699383
Cellvibrio_japonicus	NA	NA	NA	4.941064097	2.251052383	NA	0	8.627369725	10.39971585	9.978238539	9.665335917	11.17575537
Agrobacterium_larrymoorei	NA	NA	NA	4.989139007	2.24610401	NA	-0.616922901	1.860448772	2.384636129	-3.234728316	-0.666091199	0.357316112
Glaesserella_sp._15-184	NA	NA	NA	6.441193854	NA	1.019097825	8.876516947	11.0109756	9.929258409	-2.125276524	1.524462617	3.013886224
Myroides_odoratus	NA	NA	NA	NA	-4.773250775	-5.836066883	0.741386966	-1.875240198	2.912622144	1.325488822	-0.277613368	-3.381891659
Vibrio_tapetis	NA	NA	NA	NA	-4.525578045	-2.304994646	0	0	0	-0.092938933	0.229306887	0.477898527
Photorhabdus_thracensis	NA	NA	NA	NA	-3.961525852	-2.403325747	0.064823886	0.604682694	1.141080473	0.989804233	0.059706246	1.417013078
Caldicellulosiruptor_bescii	NA	NA	NA	NA	-3.832741992	-0.575519587	-1.191717843	0.773257307	1.375783122	10.53915881	8.297682077	11.04939253
Wolbachia_endosymbiont_of_Cimex_lectularius	NA	NA	NA	NA	-2.922553976	NA	-11.02299885	-1.644801554	-0.784916607	-0.233861249	1.933020668	0.008885783
Wenyingzhuangia_fucanilytica	NA	NA	NA	NA	-2.897297047	0.118957366	0	0	8.451211112	0.512206158	1.613583823	1.660472687
Klebsiella_huaxiensis	NA	NA	NA	NA	-2.646216565	-2.10987277	0.855025889	0.186179793	0.741076903	1.751829132	2.662691228	1.671615115
Streptomyces_violaceoruber	NA	NA	NA	NA	-1.995986937	NA	1.436533988	0.449271822	1.323408139	-0.595584371	-0.468843943	-0.604988422
Halomonas_aestuarii	NA	NA	NA	NA	-1.852941496	0.097589827	-0.908172422	0.488709756	-0.711785289	-8.886711698	0.435216397	1.223549742
Sulfurospirillum_deleyianum	NA	NA	NA	NA	-1.719366221	-1.775880474	2.455932428	1.499827311	3.229981994	-0.571733774	0.193068681	-0.169023125
Mycolicibacterium_phlei	NA	NA	NA	NA	-1.66828371	NA	0	10.35434857	10.36632221	0	8.945639889	9.125844794
Nostoc_sp._'Lobaria_pulmonaria_(5183)_cyanobiont'	NA	NA	NA	NA	-1.592175991	-1.080136085	0.148244429	-0.004555063	0.120977213	-2.2472049	-0.168660937	1.635207579
Arcobacter_lekithochrous	NA	NA	NA	NA	-1.56602481	-0.798629522	-0.67881319	0.418754014	0.133814454	-1.663688143	-0.042020218	0.813760299
Leptospirillum_ferriphilum	NA	NA	NA	NA	-1.49683159	NA	-9.222246695	-9.222246695	-9.222246695	0	8.22753412	9.947392937
Trichormus_azollae	NA	NA	NA	NA	-1.449695314	-0.844220216	0.597042974	-0.754167895	-0.342144722	-0.063526075	-0.24772869	0.743012688
Pseudomonas_sp._DG56-2	NA	NA	NA	NA	-1.44633363	-0.699279218	-0.29639556	-2.116464279	0.678069881	-8.087462841	1.450402006	2.340897331
Orrella_dioscoreae	NA	NA	NA	NA	-1.41187022	NA	1.213881977	2.00174729	3.573856595	-8.048034513	-2.575546742	-8.048034513
Campylobacter_sputorum	NA	NA	NA	NA	-1.39908277	-0.615480827	0.436596307	0.423052698	-0.254181155	-0.457494861	0.314993452	0.925676955
Klebsiella_quasivariicola	NA	NA	NA	NA	-1.305448161	NA	-0.419830047	0.646096159	-0.011395837	-7.745956618	-7.745956618	1.889458605
Brevundimonas_sp._SGAir0440	NA	NA	NA	NA	-1.299053629	-0.212544635	0.22678618	1.535078571	1.631748144	-1.464239492	1.143121963	1.430034357
Hoeflea_sp._IMCC20628	NA	NA	NA	NA	-1.261627585	-0.359807979	-8.909620116	-0.799194228	2.855251475	0	0	0
Formosa_sediminum	NA	NA	NA	NA	-1.232225292	0.650591169	3.436723464	2.358155088	1.858154556	-1.157995697	0.079402779	0.298672743
Spiroplasma_alleghenense	NA	NA	NA	NA	-1.171724995	-2.070029598	-10.13861739	-2.028191503	-3.981500901	0	0	9.518324652
Enterobacter_sichuanensis	NA	NA	NA	NA	-1.109624491	0.568579233	-1.216002391	0.548281614	-0.007895757	0.523220522	-0.277297488	2.036013229
Weeksella_virosa	NA	NA	NA	NA	-1.089617141	1.037856018	0.636924723	0.703510625	1.013953925	-2.423307692	-0.526701777	0.076263318
Ruegeria_sp._TM1040	NA	NA	NA	NA	-1.054319641	-0.277433014	0.076318117	0.589538688	0.845646505	1.677777176	-9.273019272	-0.86080088
Methylomonas_denitrificans	NA	NA	NA	NA	-0.908576972	NA	0	10.40359793	9.691425987	0.864580833	-0.853428487	-8.849664685
Pseudomonas_sp._gcc21	NA	NA	NA	NA	-0.874844869	0.896635141	-0.383699685	0.122303614	-2.237913556	0.628218768	0.371968777	-2.677587047
Wolbachia_endosymbiont_of_Chrysomya_megacephala	NA	NA	NA	NA	-0.870706893	-1.684372048	-0.64312635	-0.209136381	-0.074822139	-0.694807969	-1.475193537	-0.239197543
Gemmata_sp._SH-PL17	NA	NA	NA	NA	-0.769463504	NA	-1.062605445	0.119806831	0.273310988	-7.544323092	-7.544323092	0.976621606
Acidovorax_sp._T1	NA	NA	NA	NA	-0.759446173	0.247202203	-0.462667222	-0.24415959	-0.23625626	0	0	0
Mesoplasma_tabanidae	NA	NA	NA	NA	-0.723397718	-0.104775659	0.418508903	1.793282166	2.406816992	0.380944248	0.853445084	1.549181221

Moritella_yayanosii	NA	NA	NA	NA	-0.683831174	-2.394114726	-0.537896594	0.340288447	0.012905365	-11.06204614	-1.286161006	-0.246662842
Francisella_uliginis	NA	NA	NA	NA	-0.567989422	1.086960598	-11.04053631	-0.763353546	-3.295399845	2.02179916	-7.700439718	-7.700439718
Waddlia_chondrophila	NA	NA	NA	NA	-0.553691039	-1.110456648	1.334865608	0.762298246	-0.250377082	-1.387201433	-8.721099189	2.84875642
Rhizobium_sp._NXC24	NA	NA	NA	NA	-0.418395801	-0.910439944	-0.238734434	0.475557733	0.692470622	-0.915586102	0.45596742	1.009736758
Mesorhizobium_oceanicum	NA	NA	NA	NA	-0.401616984	0.793030447	-0.010044446	1.336836909	0.256732459	-0.183446141	-9.651051691	0.305081939
Wolbachia_endosymbiont_of_Ctenocephalides_felis_wCfeJ	NA	NA	NA	NA	-0.388452714	3.3321086	0.219620833	-1.179847472	-0.031884764	0.230871415	0.451794879	0.187949306
Streptomyces_rubrolavendulae	NA	NA	NA	NA	-0.345597001	NA	-9.222246695	1.028264913	2.034174312	-8.186525318	-8.186525318	-8.186525318
Anabaenopsis_circularis	NA	NA	NA	NA	-0.272655324	1.301737935	0.193087464	0.432734444	3.060312502	2.269526274	3.969120223	0.877810307
Ketogulonicigenium_vulgare	NA	NA	NA	NA	-0.261627585	NA	-1.4551103	-0.445079182	-2.56878913	0	0	0
Bradyrhizobium_vignae	NA	NA	NA	NA	-0.223941208	-0.153379711	1.351071551	-1.067220377	1.236563279	9.448461189	5.472487771	0
Sulfurospirillum_sp._JPD-1	NA	NA	NA	NA	-0.202408047	0.661334664	0.924241029	0.004753048	-8.10567284	-8.725649145	-0.509713363	-8.725649145
Synechococcus_sp._RS9909	NA	NA	NA	NA	-0.202408047	1.588055096	0	0	11.99210278	8.81164121	7.996236198	6.930737338
Candidatus_Endolissoclinum_faulkneri	NA	NA	NA	NA	-0.202408047	NA	-0.264857512	-0.146790699	0.088384352	8.86005338	9.214319121	0
Leucobacter_sp._HDW9A	NA	NA	NA	NA	-0.13289427	NA	-8.552496194	-8.552496194	-8.552496194	0	7.222389201	0
Blattabacterium_sp._DPU	NA	NA	NA	NA	-0.105522865	1.390139397	-0.99154732	-1.912832489	0.229881795	0.410268657	-0.024977007	0.608971245
Streptomyces_globosus	NA	NA	NA	NA	-0.039235163	1.158147834	-10.77463883	-10.77463883	-1.829994111	8.366322214	0	9.129283017
Marinomonas_sp._FW-1	NA	NA	NA	NA	-0.009984089	1.534336428	9.248359308	8.627369725	0	-0.311944006	2.661556432	2.117533441
Novosphingobium_sp._THN1	NA	NA	NA	NA	0.011630437	1.337764643	0	0	0	2.065364727	2.539683648	1.344448356
Xanthomonas_hyacinthi	NA	NA	NA	NA	0.011681805	NA	0	0	0	0	0	7.63420844
Pseudomonas_sp._7SR1	NA	NA	NA	NA	0.021247734	NA	-0.301925961	-0.630227894	0.721813757	0.898620403	-7.544323092	-7.544323092
Yersinia_kristensenii	NA	NA	NA	NA	0.06808899	-1.117266052	-10.77463883	-10.77463883	-10.77463883	0	8.803055861	0
Cellulophaga_algicola	NA	NA	NA	NA	0.39378495	2.416304778	8.166278594	11.69221881	8.624046047	0.760506687	-0.049984107	0.842029736
Streptomyces_sp._Tu_2975	NA	NA	NA	NA	0.460071253	NA	9.248359308	0	0	-7.930737338	-0.218327271	0.481481054
Mycolicibacterium_anyangense	NA	NA	NA	NA	0.555133146	NA	-10.00842862	-0.624331314	-10.00842862	7.420659242	0	9.873444113
Rhizobium_indicum	NA	NA	NA	NA	0.601082166	0.650110502	8.129283017	9.426646347	0	0	0	0
Macrococcus_sp._IME1552	NA	NA	NA	NA	0.65790937	-2.21081607	0.019797466	-0.575945333	-0.52592228	-9.744833837	1.681079637	1.207419635
Mycolicibacter_hiberniae	NA	NA	NA	NA	0.673158009	1.262411346	0	0	0	-8.202941691	0.607845208	2.115228252
Glutamicibacter_arilaitensis	NA	NA	NA	NA	1.061550256	0.451406161	0.476845153	-0.378607876	-0.265548154	-8.087462841	0.46610118	2.165994673
Tenacibaculum_sp._DSM_106434	NA	NA	NA	NA	1.145273893	2.571568684	0.500972567	1.161901043	1.30133502	0.979459222	-1.681510989	-0.698830465
Bacillus_mojavensis	NA	NA	NA	NA	1.1514259	-0.973317957	-10.14210706	-0.750180417	0.168505724	2.55544922	-7.741466986	-7.741466986
Mycoplasma_conjunctivae	NA	NA	NA	NA	1.213281371	1.955300193	-0.067621511	0.366337849	1.63373753	0	6.470320477	7.539158811
Spiroplasma_chinense	NA	NA	NA	NA	1.258629227	-0.353050612	1.892322049	1.288606464	2.566545034	-0.296273425	-0.836341397	0.33581558
Methylobacterium_terrae	NA	NA	NA	NA	1.356975042	-0.276101309	-1.444229182	-1.956232754	-0.602560361	10.34503926	0	9.918863237
Marinobacter_sp._BSs20148	NA	NA	NA	NA	1.386387295	NA	-0.146481151	-1.2435074	-0.219570032	9.979182225	0	0
Acinetobacter_cumulans	NA	NA	NA	NA	1.433159518	1.073730389	2.33046609	-8.10567284	4.418601956	-1.631538719	-4.378219468	-0.302526885
Pseudomonas_sp._M30-35	NA	NA	NA	NA	1.628577276	NA	0.33014713	-0.688709999	-2.642019397	9.915879379	10.8790701	7.539158811
Arcobacter_trophiarum	NA	NA	NA	NA	1.852427451	0.946089718	-0.392383526	-0.574996919	0.280072015	-1.300159721	-2.967556679	-0.358139918
Pseudomonas_sp._SK	NA	NA	NA	NA	2.574978412	-1.187442763	8.129283017	9.426646347	7.745136469	0.186036324	-0.850979852	-9.276124405
Paenibacillus_peoriae	NA	NA	NA	NA	2.921628936	1.256108348	-1.443550273	0.055139068	0.252150681	0.288151149	1.617787339	1.183159412
Maize_bushy_stunt_phytoplasma	NA	NA	NA	NA	2.933572638	2.123445483	-2.251576531	-2.550541579	-4.503850977	1.567684509	1.529524258	-0.426530253
Pseudomonas_synxantha	NA	NA	NA	NA	2.958870124	1.793877418	8.129283017	0	7.745136469	9.582456272	8.215935782	8.520944699
Acidovorax_citrulli	NA	NA	NA	NA	3.332694526	0.270220946	-2.015995415	-10.17311494	-0.362250938	-9.661778098	-9.661778098	-2.122619287
Enterobacteriaceae_endosymbiont_of_Plateumaris_pusilla	NA	NA	NA	NA	3.856357094	3.677432271	9.029913869	8.110425888	8.157119522	1.799089731	1.691048565	0.827925264
Helicobacter_cholecystus	NA	NA	NA	NA	NA	-4.694079781	-11.34176898	0.399546313	-0.486332044	-0.976008832	-0.355084438	-0.526344215
Mesoplasma_coleopterae	NA	NA	NA	NA	NA	-4.034843327	0.07083003	0.579214794	1.245022537	-0.478662889	1.349837236	0.094144806
Aliivibrio_fischeri	NA	NA	NA	NA	NA	-2.998738351	-1.363608003	-0.263121507	-0.194286961	-0.319321435	-0.68114838	-0.467517285
Candidatus_Sulfurimonas_marisnigri	NA	NA	NA	NA	NA	-2.980968739	0.467776285	0.105263189	0.1535762	2.818314909	2.944231406	1.887308226
Sphingomonas_sp._FARSPH	NA	NA	NA	NA	NA	-2.97656925	-0.609246158	-0.154602891	1.051395445	10.11981006	9.772590054	11.59152235
Helicobacter_acinonychis	NA	NA	NA	NA	NA	-2.860667579	0.838058271	0.025835822	0.714075293	-1.372389296	-1.081448893	-0.814472975
Providencia_rustigianii	NA	NA	NA	NA	NA	-2.827982883	-1.284779136	-0.668123655	-0.641679895	-0.721380209	-0.757600359	0.20681241
Pseudoalteromonas_arctica	NA	NA	NA	NA	NA	-2.820590749	-8.04736934	0.334761816	-8.04736934	-8.963858845	-1.741469244	-1.424699634
Vibrio_scophthalmi	NA	NA	NA	NA	NA	-2.805878516	0	8.110425888	8.944644719	-9.117209319	-0.692064765	1.945734007
Mycoplasma_pullorum	NA	NA	NA	NA	NA	-2.480252781	0.063533621	-0.213191249	-0.751611669	9.806280552	8.033423002	11.33352996
Polynucleobacter_duraquae	NA	NA	NA	NA	NA	-2.405564747	-0.728664192	-0.093056067	-0.245795508	-9.132713066	-2.316791862	-0.09196581
Fischerella_sp._NIES-4106	NA	NA	NA	NA	NA	-2.363323038	-0.843841264	-0.330459812	-0.264647257	1.554725845	0.723744785	1.21252456
Micromonospora_sp._WMMC415	NA	NA	NA	NA	NA	-2.145245727	-0.719506368	-9.222246695	2.306476003	0.777853642	-9.321928095	-9.321928095
Algibacter_sp._L1A34	NA	NA	NA	NA	NA	-1.9749002	-0.099793828	-0.123676428	-0.211687727	-0.686635831	0.080885308	0.588571101
Janthinobacterium_sp._B9-8	NA	NA	NA	NA	NA	-1.932335612	-9.342276942	-2.659605334	0.218925069	-10.20049405	-2.297618531	-1.661335243
Citrobacter_farmeri	NA	NA	NA	NA	NA	-1.859375128	0.32389449	-1.752017756	-0.72913442	0.938013113	-1.271146509	-0.810729649
Mycolicibacterium_thermoresistibile	NA	NA	NA	NA	NA	-1.840219556	0.611075114	-8.637284195	0.916955452	0.753518142	1.339575961	0.680380623
Arcobacter_aquimarinus	NA	NA	NA	NA	NA	-1.830438535	0.671448165	1.342034058	0.617825812	8.282314694	7.29523629	0
Enterobacter_soli	NA	NA	NA	NA	NA	-1.796523827	-0.046515506	-0.599874745	-0.509214668	9.571121208	8.739216921	0
Formosa_sp._Hel3_A1_48	NA	NA	NA	NA	NA	-1.193501305	-0.781941647	0.178285343	0.490515069	-0.292746028	0.951847859	0.108744261
Vitreoscilla_sp._C1	NA	NA	NA	NA	NA	-1.117266052	0.101970389	0.741426774	-1.177053184	1.618906863	2.169922761	3.635948584
Mycoplasma_capricolum	NA	NA	NA	NA	NA	-0.9685626	-0.0614025	-2.40864476	-9.091316369	10.33985	9.846117173	9.969631147
Luteibacter_rhizovicinus	NA	NA	NA	NA	NA	-0.909989834	0.698304921	-0.363379008	-0.696054408	8.86005338	7.222389201	0
Brevundimonas_sp._MF30-B	NA	NA	NA	NA	NA	-0.900824595	0.168005535	-0.425636546	-0.505328925	0.504696747	0.532178858	-0.28165618
Sphingomonas_insulae	NA	NA	NA	NA	NA	-0.81067528	0.171603216	0.676885519	0.373700077	0	0	7.539158811
Xanthomonas_sp._SS	NA	NA	NA	NA	NA	-0.794116666	9.029913869	8.382131157	11.5287227	0	5.472487771	8.412218392
Shewanella_frigidimarina	NA	NA	NA	NA	NA	-0.772641326	-1.8346979	0.167812839	0.344396778	1.871485259	2.063254523	0.476550579
Micromonospora_sagamiensis	NA	NA	NA	NA	NA	-0.725283789	-1.371256369	-10.757315	-3.012178528	0	0	0
Methylomonas_sp._DH-1	NA	NA	NA	NA	NA	-0.725283789	-0.267692012	-0.321455596	0.28405795	8.27301694	0	0
Sphingomonas_sp._sand1-3	NA	NA	NA	NA	NA	-0.605327849	-1.829929273	-0.331844388	-2.802097821	0.914806729	0.756713819	0.72661761
Lysinibacillus_varians	NA	NA	NA	NA	NA	-0.432028216	-0.884241885	-9.488205685	1.569048262	1.465910543	-0.585675097	1.841633126
Mycoplasma_anatis	NA	NA	NA	NA	NA	-0.383949821	1.701375603	0.720621516	2.201632403	0	0	0
Enterobacter_sp._RHBSTW-00994	NA	NA	NA	NA	NA	-0.370542485	0.431150785	0.626102657	0.022674048	-1.041378238	-0.41151664	-1.933570508
Sporosarcina_sp.	NA	NA	NA	NA	NA	-0.360655929	-8.10567284	-8.10567284	-8.10567284	0	0	0
Streptomyces_peucetius	NA	NA	NA	NA	NA	-0.353050612	-0.015942457	1.227878164	-0.553600218	0	0	0
Paraburkholderia_dokdonella	NA	NA	NA	NA	NA	-0.348563221	0	0	0	-8.759332297	-8.759332297	-8.759332297
Halioglobus_japonicus	NA	NA	NA	NA	NA	-0.340284353	0.193575415	-0.438846458	-0.044223282	2.613557653	0.128472941	2.502497221
Enterobacter_sp._R4-368	NA	NA	NA	NA	NA	-0.290566941	0.985423361	1.936844476	2.928419524	0.99068189	1.34134362	1.44550278
Janibacter_sp._YB324	NA	NA	NA	NA	NA	-0.275035503	-0.382852592	-0.285094953	-0.128645899	0	0	0
Rickettsia_akari	NA	NA	NA	NA	NA	-0.250374834	-1.006347866	-0.091940938	0.300073925	0	0	0
Acinetobacter_pittii	NA	NA	NA	NA	NA	-0.226415815	0.513083358	1.414479409	1.252346302	0.742164071	0.6327816	0.577794073
Kordia_sp._SMS9	NA	NA	NA	NA	NA	-0.173202996	-0.205449427	-0.904404143	-0.665334443	0.301987814	2.125240236	-8.087462841
Legionella_sp._PC997	NA	NA	NA	NA	NA	-0.168681835	-0.17400535	0.961097371	1.828258707	0	7.222389201	0
Rickettsia_prowazekii	NA	NA	NA	NA	NA	-0.153379711	0	0	0	0	0	10.27301461
Serratia_ficaria	NA	NA	NA	NA	NA	-0.14974712	-0.579470653	-0.187210723	-0.177405538	1.476192999	-0.175092191	3.174492302
Streptomyces_parvulus	NA	NA	NA	NA	NA	-0.122719158	-1.194521996	-2.541757234	-2.479299396	-8.709659397	0.812594524	1.66175789
Microcoleus_sp._PCC_7113	NA	NA	NA	NA	NA	-0.106499621	0.541775871	0.414117216	0.286180746	0	9.33687918	8.952254929
Aminobacter_sp._MSH1	NA	NA	NA	NA	NA	-0.070175294	0.172413062	0.041873387	-0.369613436	0	0	0
Pseudarthrobacter_sp._NIBRBAC000502770	NA	NA	NA	NA	NA	0.031326334	1.579573215	2.868074671	2.634140219	-7.807354922	0.420179198	-7.807354922
Borreliella_valaisiana	NA	NA	NA	NA	NA	0.09105198	-8.637284195	-1.954612586	0.307360524	2.267020886	0.359445817	0.019695801
Halothece_sp._PCC_7418	NA	NA	NA	NA	NA	0.151521312	0.73031037	-0.251368147	-0.321290574	0.694819784	1.565867489	1.663965147
Deinococcus_soli_Cha_et_al._2016	NA	NA	NA	NA	NA	0.226990656	-0.408996723	-1.064951617	1.442845404	0	0	8.544319228
Pseudomonas_antarctica	NA	NA	NA	NA	NA	0.235128409	0.071912254	-0.365382784	-0.369385015	8.45395786	6.470320477	6.930737338
Luteimonas_sp._JM171	NA	NA	NA	NA	NA	0.323008361	0.331176172	-0.220833728	-0.395687331	-8.971543554	0.742701964	0.788898638
Bartonella_quintana	NA	NA	NA	NA	NA	0.365552662	-1.020526743	-9.177646265	3.13451346	0.195573617	0.785088643	0.583003158
Pseudonocardia_sp._HH130629-09	NA	NA	NA	NA	NA	0.401616984	-8.637284195	-8.637284195	-8.637284195	-0.612183969	2.497737701	-7.741466986
Mycobacterium_kubicae	NA	NA	NA	NA	NA	0.41657614	0.58222207	-1.031599688	2.45298466	0	0	0
Acinetobacter_sp._TGL-Y2	NA	NA	NA	NA	NA	0.535493824	0.140817728	0.406657986	0.644575761	-9.433236982	0.104627865	0.941074399
Blochmannia_endosymbiont_of_Camponotus_nipponensis	NA	NA	NA	NA	NA	0.552541023	-0.413656787	-0.237965794	0.145926905	2.790836544	1.833045982	-0.54334808
Corynebacterium_kutscheri	NA	NA	NA	NA	NA	0.564174028	0	0	0	-7.700439718	-2.227951947	-0.161280907
Streptomyces_sp._jing01	NA	NA	NA	NA	NA	0.69805628	0	0	0	-0.334038873	0.004377344	-1.846245032
Rheinheimera_sp._D18	NA	NA	NA	NA	NA	0.69805628	0	0	0	8.363403271	0	10.64024494
Novosphingobium_pentaromativorans	NA	NA	NA	NA	NA	0.980634675	0	0	8.624046047	9.239997035	10.26600174	8.412218392
Sphingobacterium_sp._21	NA	NA	NA	NA	NA	1.011101591	10.39777088	6.682671608	10.79766153	-0.783682093	-1.271541637	0.864792088
Pseudomonas_pelagia	NA	NA	NA	NA	NA	1.107357409	0	0	0	8.366322214	0	9.306820443
Acinetobacter_seifertii	NA	NA	NA	NA	NA	1.154866101	0.525140417	0.77677715	1.231682822	1.229639555	0.484640115	-0.287595047
Polaribacter_sp._MED152	NA	NA	NA	NA	NA	1.20861639	-2.266359026	-0.990650688	-0.818817298	11.26795708	9.636624621	7.380824669
Rhodovulum_sp._MB263	NA	NA	NA	NA	NA	1.323334916	0	9.627367901	0	-0.547634196	-8.990577692	-8.990577692

Aquicella_lusitana	NA	NA	NA	NA	NA	1.338928835	0.669209637	1.937898986	0.839947608	-0.82269618	1.178231037	0.726888108
Acinetobacter_sp._WCHA45	NA	NA	NA	NA	NA	1.342933953	-0.427875103	1.067319698	0.721553958	-0.569948942	1.700335203	1.704949232
Paraburkholderia_sp._DHF22	NA	NA	NA	NA	NA	1.417062335	-0.175075451	-0.136682705	1.078683652	-2.154757826	-10.18818083	0.317630726
Mesorhizobium_terrae	NA	NA	NA	NA	NA	1.529791347	-1.091923809	-2.735777493	-0.367160818	0.881090759	0.966752742	1.723543033
Corynebacterium_resistens	NA	NA	NA	NA	NA	1.795239847	-0.83967456	-0.285884824	-2.341091447	0	9.650572508	9.220782177
Psychroserpens_sp._NJDZ02	NA	NA	NA	NA	NA	1.795461243	-1.389437466	-0.045219747	-1.011590461	0	0	0
Candidatus_Ruthia_endofausta	NA	NA	NA	NA	NA	1.906890596	0	10.95261022	11.69023811	0.479861246	-2.213628931	0.082306519
Rickettsiales_endosymbiont_of_Stachyamoeba_lipophora	NA	NA	NA	NA	NA	1.999288436	0	6.682671608	6.15711649	-0.586947818	0.687219922	1.580987139
Candidatus_Nucleicultrix_amoebiphila	NA	NA	NA	NA	NA	2.129790811	10.00842862	8.110425888	8.904155902	1.385156418	1.159622487	1.821428839
Pseudoalteromonas_spongiae	NA	NA	NA	NA	NA	2.130167292	0.926578931	1.281110396	1.438621983	0.977875083	0.202948609	-2.674926845
Janibacter_melonis	NA	NA	NA	NA	NA	2.610845135	-0.138266755	-1.279113175	0.794146426	1.133439676	1.701058918	0.151798528
Neisseria_polysaccharea	NA	NA	NA	NA	NA	2.684305029	-0.924088562	-0.20679051	0.725453519	0.142105999	2.442855872	1.888110321
Ehrlichia_chaffeensis	NA	NA	NA	NA	NA	2.691289402	-0.419751137	1.067601787	-0.76318615	0.825113016	-0.656683685	-0.039335571
Candidatus_Sneabacter_namystus	NA	NA	NA	NA	NA	2.702516435	0.329326081	0.146469057	0.477801302	10.46964182	9.132713066	9.554588852
Streptococcus_sp._HSISM1	NA	NA	NA	NA	NA	2.754105342	-0.046099373	-0.634621743	-0.639737965	9.662965606	7.369230901	9.423466822
Mycoplasma_phocidae	NA	NA	NA	NA	NA	2.80514186	0.231822607	1.218716029	-0.173761309	0.392783067	-0.784386169	-0.50285295
Polaribacter_sp._L12M9	NA	NA	NA	NA	NA	3.081986617	0	10.02990835	12.13876776	-7.930737338	3.257276541	1.021517591
Legionella_sp._PC1000	NA	NA	NA	NA	NA	3.196694584	-1.245559964	0.759424089	-0.234927063	-0.230426862	1.116381732	0.714413752
Candidatus_Pantoea_carbekii	NA	NA	NA	NA	NA	3.328070499	9.248359308	0	6.15711649	0.483690131	0.277109697	-2.701045414
Azospirillum_sp._TSA2s	NA	NA	NA	NA	NA	3.926155647	0	0	6.15711649	1.300391059	-0.749745803	-1.432956831
Xanthomonas_vesicatoria	NA	NA	NA	NA	NA	NA	-13.71580116	-3.750016873	-2.877455997	2.050073041	0.16879219	1.73725818
Streptomyces_lavendulae	NA	NA	NA	NA	NA	NA	-11.13862379	-2.511254065	-1.319025853	-0.014611512	-1.23211331	0.364183878
Alteromonas_naphthalenivorans	NA	NA	NA	NA	NA	NA	-11.11929781	0.196983722	-3.962183342	-8.700439718	-0.107982681	2.247438258
Mycoplasma_suis	NA	NA	NA	NA	NA	NA	-10.84975693	-0.666456526	-0.616568693	-1.668443086	-3.306479742	-10.12240095
Enterobacteriaceae_endosymbiont_of_Plateumaris_rustica	NA	NA	NA	NA	NA	NA	-10.78953364	-10.78953364	-2.308650565	0	0	0
Pseudomonas_extremorientalis	NA	NA	NA	NA	NA	NA	-10.74437136	0.563142548	0.100405007	-7.741466986	-7.741466986	-7.741466986
Pseudarthrobacter_sp._NIBRBAC000502771	NA	NA	NA	NA	NA	NA	-10.60059127	-10.60059127	-10.60059127	0	0	0
Bacillus_sp._N3536	NA	NA	NA	NA	NA	NA	-10.39679741	-1.144131981	-0.443526353	0.933523482	-7.745956618	-7.745956618
Azospirillum_sp._TSH58	NA	NA	NA	NA	NA	NA	-10.34429591	-10.34429591	0.314236932	0	0	0
Bartonella_schoenbuchensis	NA	NA	NA	NA	NA	NA	-10.33417271	0.676802894	0.365165496	0	0	8.412218392
Micromonospora_sp._B006	NA	NA	NA	NA	NA	NA	-10.30750822	-10.30750822	-1.826625138	0	0	0
Pseudomonas_libanensis	NA	NA	NA	NA	NA	NA	-10.21784867	-10.21784867	-10.21784867	0	0	0
Microbacterium_sp._Nx66	NA	NA	NA	NA	NA	NA	-10.2067946	-1.647329558	-1.049677608	8.45395786	0	0
Mycobacterium_sp._DL440	NA	NA	NA	NA	NA	NA	-10.17311494	-3.490443329	-10.17311494	8.86005338	0	0
Stenotrophomonas_acidaminiphila	NA	NA	NA	NA	NA	NA	-10.17311494	-0.146965012	-2.427978469	-0.129410951	-0.400633991	1.959355407
Sphingobium_fuliginis	NA	NA	NA	NA	NA	NA	-10.16627608	-0.113952578	-1.542230036	-7.741466986	-0.372236085	-7.741466986
Rathayibacter_sp._VKM_Ac-2762	NA	NA	NA	NA	NA	NA	-10.11988785	-0.206183118	0.349937085	7.420659242	0	0
Stenotrophomonas_sp._ZAC14A_NAIMI4_1	NA	NA	NA	NA	NA	NA	-10.09970285	0.302925539	0.526416828	-8.048034513	-8.048034513	-8.048034513
Vibrio_sp._Scap24	NA	NA	NA	NA	NA	NA	-10.00077468	-3.318103069	-1.519891597	0	0	0
Pseudomonas_sp._LG1E9	NA	NA	NA	NA	NA	NA	-9.95921229	-0.575114981	-2.802097821	8.366322214	0	0
Streptomyces_viridosporus	NA	NA	NA	NA	NA	NA	-9.936637939	0.513635116	1.651650383	0	0	0
Photorhabdus_asymbiotica	NA	NA	NA	NA	NA	NA	-9.932617823	-9.932617823	-9.932617823	8.45395786	7.902875524	0
Aeromicrobium_sp._MF47	NA	NA	NA	NA	NA	NA	-9.851180595	0.893190766	-0.906535876	8.21916852	0	8.520944699
Stenotrophomonas_sp._NA06056	NA	NA	NA	NA	NA	NA	-9.851180595	2.053318556	1.885529969	0	0	0
Mycobacterium_simiae	NA	NA	NA	NA	NA	NA	-9.800605508	0.113099229	-9.800605508	0	0	0
Micromonospora_craniellae	NA	NA	NA	NA	NA	NA	-9.714245518	-9.714245518	0.24891356	0	0	8.847580444
Tardiphaga_sp._vice278	NA	NA	NA	NA	NA	NA	-9.681078494	-9.681078494	-9.681078494	-8.981090586	-8.981090586	-8.981090586
Mycobacterium_tuberculosis	NA	NA	NA	NA	NA	NA	-9.64385619	-9.64385619	-1.16297311	0	5.472487771	0
Gemella_sp._oral_taxon_928	NA	NA	NA	NA	NA	NA	-9.638930002	-9.638930002	0.180667935	7.894817763	8.912889336	8.952254929
Pseudoalteromonas_sp._1_2015MBL_MicDiv	NA	NA	NA	NA	NA	NA	-9.638930002	-9.638930002	0.2597394	0	0	0
Cardinium_endosymbiont_of_Sogatella_furcifera	NA	NA	NA	NA	NA	NA	-9.624047875	-9.624047875	-2.466933407	0	0	0
Ornithinimicrobium_sp._AMA3305	NA	NA	NA	NA	NA	NA	-9.552494273	-9.552494273	-9.552494273	0	0	0
Thermotoga_sp._RQ7	NA	NA	NA	NA	NA	NA	-9.552494273	0.630806136	0.001745374	0	0	8.741466986
Sulfitobacter_sp._SK025	NA	NA	NA	NA	NA	NA	-9.445579808	1.039887272	-0.6449743	0	0	0
Nitrosospira_lacus	NA	NA	NA	NA	NA	NA	-9.384097309	1.02142712	-9.384097309	9.366322214	5.472487771	10.25895463
Aeromonas_sp._ASNIH4	NA	NA	NA	NA	NA	NA	-9.36433386	0.609291764	-2.207219392	-7.741466986	-0.519077785	-7.741466986
Rickettsia_endosymbiont_of_Bemisia_tabaci	NA	NA	NA	NA	NA	NA	-9.321928095	0.424739053	2.694121046	9.956133631	8.225607527	0
Enterobacter_sp._ODB01	NA	NA	NA	NA	NA	NA	-9.222246695	1.120028024	2.540363731	0	0	0
Bradyrhizobium_symbiodeficiens	NA	NA	NA	NA	NA	NA	-9.222246695	1.727047477	2.13010025	-0.974910574	-2.025538312	-9.247927513
Curtobacterium_sp._SGAir0471	NA	NA	NA	NA	NA	NA	-9.222246695	1.772750153	1.691802018	-0.291707555	-0.964136117	-0.552316878
Sphingobium_sp._RSMS	NA	NA	NA	NA	NA	NA	-9.209009766	-1.098583878	1.940045049	0	0	8.996239023
Enterobacter_ludwigii	NA	NA	NA	NA	NA	NA	-9.177646265	-9.177646265	-9.177646265	0	0	0
Cellulosimicrobium_cellulans	NA	NA	NA	NA	NA	NA	-9.177646265	-9.177646265	-9.177646265	0.531549953	1.035515383	-0.107258546
Sphingobium_sp._EP60837	NA	NA	NA	NA	NA	NA	-9.177646265	-9.177646265	-2.020531797	9.194756854	8.398029648	10.78299821
Mycoplasmopsis_columbina	NA	NA	NA	NA	NA	NA	-9.177646265	0.206451044	1.158559005	0.572577222	2.302375297	0.820504981
Chlamydia_sp._S15-834C	NA	NA	NA	NA	NA	NA	-9.177646265	1.048982677	-9.177646265	0	7.222389201	0
Paenibacillus_sp._Izh-N1	NA	NA	NA	NA	NA	NA	-9.177646265	1.224982125	0.90945851	0.53638293	0.543123478	0.530239475
Candidatus_Annandia_adelgestsuga	NA	NA	NA	NA	NA	NA	-9.177646265	2.176702306	0.400811344	0	0	0
Enterococcus_sp._DA9	NA	NA	NA	NA	NA	NA	-9.093718617	1.641456587	0.047226156	8.45395786	0	0
Rhodococcus_sp._YL-1	NA	NA	NA	NA	NA	NA	-9.084082873	-0.456713147	1.482313717	7.894817763	8.354615244	0
Bifidobacterium_coryneforme	NA	NA	NA	NA	NA	NA	-9.076815597	-9.076815597	-9.076815597	0.531549953	-7.741466986	-7.741466986
Kordiimonas_pumila	NA	NA	NA	NA	NA	NA	-9.062167594	-9.062167594	-9.062167594	0	0	0
Pseudomonas_sp._SGAir0191	NA	NA	NA	NA	NA	NA	-9.062167594	-9.062167594	-0.610956482	0	0	0
Mesorhizobium_sp._M3A.F.Ca.ET.080.04.2.1	NA	NA	NA	NA	NA	NA	-9.062167594	0.321929714	-9.062167594	8.714245518	7.712410067	7.539158811
Mycobacterium_pseudoshottsii	NA	NA	NA	NA	NA	NA	-9.049848549	-9.049848549	0.844693378	1.026975854	-0.482022847	0.324997222
Spiroplasma_sp._TIUS-1	NA	NA	NA	NA	NA	NA	-8.941980727	-0.314611001	0.369665427	-9.288480843	0.56638754	-1.538055222
Mycolicibacterium_insubricum	NA	NA	NA	NA	NA	NA	-8.933959107	-0.306589382	-1.188822638	0	8.225607527	9.518324652
Citrobacter_sp._RHBSTW-00881	NA	NA	NA	NA	NA	NA	-8.799135887	-0.688709999	0.355682222	0	0	0
Nitrosococcus_oceani	NA	NA	NA	NA	NA	NA	-8.799135887	0.584961421	-0.080212637	0	0	0
Stenotrophomonas_sp._ESTM1D_MKCIP4_1	NA	NA	NA	NA	NA	NA	-8.799135887	0.592790753	0.043500452	0	9.330916878	7.63420844
Sphingobium_cloacae	NA	NA	NA	NA	NA	NA	-8.799135887	0.828232014	1.710817216	0	5.472487771	0
Pseudonocardia_sp._EC080610-09	NA	NA	NA	NA	NA	NA	-8.75731333	2.039610449	-0.600193808	0.185554099	-0.71823194	-8.087462841
Mycoplasma_arginini	NA	NA	NA	NA	NA	NA	-8.637284195	-8.637284195	-8.637284195	-1.603109847	1.580949438	-8.906890596
Chlamydia_trachomatis	NA	NA	NA	NA	NA	NA	-8.637284195	-8.637284195	-8.637284195	0	0	0
Chlamydia_felis	NA	NA	NA	NA	NA	NA	-8.637284195	-0.255153038	1.682591109	0	7.222389201	0
Streptomyces_sp._MOE7	NA	NA	NA	NA	NA	NA	-8.637284195	0.275060287	0.949726305	0	10.33687472	0
Leifsonia_sp._PS1209	NA	NA	NA	NA	NA	NA	-8.637284195	0.990083707	1.569510409	8.716534837	0	11.46284476
Mesorhizobium_sp._M1B.F.Ca.ET.045.04.1.1	NA	NA	NA	NA	NA	NA	-8.624046047	-0.513620159	0.176559462	0	0	8.928764108
Neisseria_weaveri	NA	NA	NA	NA	NA	NA	-8.552496194	-8.552496194	-2.395379704	0	0	0
Legionella_cherrii	NA	NA	NA	NA	NA	NA	-8.552496194	0.21689208	2.835031138	-0.008486466	0.670290124	-0.755416446
Mycobacterium_conspicuum	NA	NA	NA	NA	NA	NA	-8.10567284	-8.10567284	-8.10567284	0.563360989	1.206423539	0.688373698
Pandoraea_faecigallinarum	NA	NA	NA	NA	NA	NA	-8.10567284	0.004753048	0.736963499	0	0	0
Haemophilus_pittmaniae	NA	NA	NA	NA	NA	NA	-8.10567284	0.004753048	1.290152589	0	0	8.520944699
Rhodococcus_sp._2G	NA	NA	NA	NA	NA	NA	-8.10567284	1.004750437	0.61325041	0	8.996239023	0
Myroides_sp._A21	NA	NA	NA	NA	NA	NA	-8.10567284	1.320973507	0.345538271	0	0	0
Streptomyces_sp._QHH-9511	NA	NA	NA	NA	NA	NA	-8.091313724	-8.091313724	-8.091313724	-7.890768904	0.105467294	-7.890768904
Synechococcus_sp._RS9907	NA	NA	NA	NA	NA	NA	-8.091313724	-8.091313724	-8.091313724	0	0	0
Chryseobacterium_carnis	NA	NA	NA	NA	NA	NA	-8.091313724	-8.091313724	-8.091313724	8.920850045	8.912889336	0
Leptospira_borgpetersenii	NA	NA	NA	NA	NA	NA	-8.091313724	-8.091313724	0.214127906	0	5.472487771	8.520944699
Yersinia_aleksiciae	NA	NA	NA	NA	NA	NA	-8.091313724	-8.091313724	1.063504385	0	9.415038204	0
Streptomyces_sp._C8S0	NA	NA	NA	NA	NA	NA	-8.091313724	-8.091313724	2.663724051	-7.741466986	-0.519077785	0.909584705
Pseudomonas_sp._THAF7b	NA	NA	NA	NA	NA	NA	-8.091313724	-0.408635094	0.065805798	0	0	0
Micromonospora_narathiwatensis	NA	NA	NA	NA	NA	NA	-8.076815597	-8.076815597	-8.076815597	0	0	0
Synechococcus_sp._ROS8604	NA	NA	NA	NA	NA	NA	-8.076815597	-8.076815597	-8.076815597	0	0	0
Mycolicibacter_terrae	NA	NA	NA	NA	NA	NA	-8.076815597	0.033610291	-1.919699107	0	0	0
Entomoplasma_melaleucae	NA	NA	NA	NA	NA	NA	-8.04736934	-8.04736934	-8.04736934	0	0	9.110266661
Enterobacteriaceae_endosymbiont_of_Plateumaris_braccata	NA	NA	NA	NA	NA	NA	-8.04736934	-8.04736934	1.568343049	0	0	0
Bartonella_grahamii	NA	NA	NA	NA	NA	NA	-8.04736934	-8.04736934	3.049345814	0	8.225607527	0
Rhizobium_gallicum	NA	NA	NA	NA	NA	NA	-8.04736934	-1.364697732	-8.04736934	0	0	0
Sphingopyxis_lindanitolerans	NA	NA	NA	NA	NA	NA	-8.04736934	2.687035453	2.840612793	0	7.222389201	0
Sphingopyxis_alaskensis	NA	NA	NA	NA	NA	NA	-7.757316664	-7.757316664	2.057193705	2.368567673	1.92528898	-1.201636617

Streptococcus_sp._HSISB1	NA	NA	NA	NA	NA	NA	-7.757316664	-1.074645056	-1.600200174	-1.073658674	-8.202941691	1.475364422
Pseudomonas_sp._BIOMIG1BAC	NA	NA	NA	NA	NA	NA	-7.757316664	2.983998629	-0.600202196	0	0	0
Pectobacterium_punjabense	NA	NA	NA	NA	NA	NA	-5.640086024	-2.004342062	-2.135022304	0	0	0
Methylomonas_koyamae	NA	NA	NA	NA	NA	NA	-3.492365963	-0.378371371	-1.19848486	0	0	0
Enterobacter_sp._E20	NA	NA	NA	NA	NA	NA	-3.177712817	0.849261089	-0.567983179	0	0	0
Edwardsiella_piscicida	NA	NA	NA	NA	NA	NA	-3.135793983	-1.781614862	-3.02476308	-1.884455155	0.66682801	0.747426903
Ensifer_mexicanus	NA	NA	NA	NA	NA	NA	-3.122137357	-2.997507959	0.807711192	7.303780748	6.815921204	7.544323092
Aurantimicrobium_minutum	NA	NA	NA	NA	NA	NA	-3.120478175	-11.6232185	-1.10697677	8.81164121	10.26569708	0
Tateyamaria_omphalii	NA	NA	NA	NA	NA	NA	-3.0991064	0.937942109	-3.188185919	0	8.60239081	8.634204809
Streptomyces_sp._WAC_06738	NA	NA	NA	NA	NA	NA	-3.008758343	0.148620355	-11.13804136	0	0	0
Rickettsia_bellii	NA	NA	NA	NA	NA	NA	-2.920308548	-0.599051732	-0.852006135	0.823645055	1.620054763	0.601281042
Ruegeria_sp._AD91A	NA	NA	NA	NA	NA	NA	-2.911376305	-1.160991534	-0.220300163	0	9.537864847	0
Methylobacterium_sp._XJLW	NA	NA	NA	NA	NA	NA	-2.824790481	-0.035854855	0.412632984	0	0	0
Aeromonas_sp._ASNIH5	NA	NA	NA	NA	NA	NA	-2.823570003	-0.963698671	-1.88536778	-7.709655951	0.517878169	-7.709655951
Geobacillus_zalihae	NA	NA	NA	NA	NA	NA	-2.801280408	-0.548893246	-0.786883103	0	0	0
Enterobacteriaceae_endosymbiont_of_Donacia_marginata	NA	NA	NA	NA	NA	NA	-2.705158623	0.207747761	-3.462762482	0.602664502	0.22086648	-8.076815597
Candidatus_Mikella_endobia	NA	NA	NA	NA	NA	NA	-2.626196828	-1.338680754	-3.085628224	-0.25264745	-0.04813869	-0.424215921
Micromonospora_sp._L5	NA	NA	NA	NA	NA	NA	-2.620435598	1.535894559	-0.053542181	0	6.815921204	0
Limnospira_fusiformis	NA	NA	NA	NA	NA	NA	-2.613605706	-1.049953195	-0.999084541	0	0	0
Apibacter_sp._B3706	NA	NA	NA	NA	NA	NA	-2.604888325	0.183497517	-1.591196905	0	7.222389201	0
cyanobacterium_endosymbiont_of_Rhopalodia_gibberula	NA	NA	NA	NA	NA	NA	-2.575732318	1.452375986	-0.040110889	0.753518142	-7.700439718	0.820504981
Methylobacterium_sp._AMS5	NA	NA	NA	NA	NA	NA	-2.525191908	-0.26254399	1.471290459	-7.709655951	-7.709655951	-7.709655951
Acinetobacter_oleivorans	NA	NA	NA	NA	NA	NA	-2.47934197	-2.029131179	1.019230217	-1.455551549	1.802270696	1.009955135
Actinobacillus_suis	NA	NA	NA	NA	NA	NA	-2.456017576	2.142736821	1.521857623	0	6.815921204	7.539158811
Paraburkholderia_phymatum	NA	NA	NA	NA	NA	NA	-2.435680621	-2.571453774	-10.68187966	-1.099123327	-9.372140267	-9.372140267
Borreliella_spielmanii	NA	NA	NA	NA	NA	NA	-2.430926656	0.240422683	-0.478497726	1.238159737	0.46651446	0.882295239
Shewanella_decolorationis	NA	NA	NA	NA	NA	NA	-2.405738874	-1.095997936	-0.343878241	0	0	0
endosymbiont_of_Pachyrhynchus_infernalis	NA	NA	NA	NA	NA	NA	-2.362813436	0.448241469	0.640960173	-0.310776199	-0.791105956	-1.761142716
Bibersteinia_trehalosi	NA	NA	NA	NA	NA	NA	-2.350659744	-0.87282812	-5.070060201	0.879563872	1.96795106	0.275637457
Synechococcus_sp._A15-62	NA	NA	NA	NA	NA	NA	-2.34581524	-0.112354264	1.835909794	0	5.472487771	9.655829428
Nostoc_sp._ATCC_53789	NA	NA	NA	NA	NA	NA	-2.334013163	-1.68531153	-0.321428119	-9.247927513	-9.247927513	-2.317190176
Candidatus_Riesia_pediculischaeffi	NA	NA	NA	NA	NA	NA	-2.324992969	-0.503570494	-2.195750913	0	0	0
Stenotrophomonas_sp._Pemsol	NA	NA	NA	NA	NA	NA	-2.184714094	0.118289451	0.292685156	0	0	0
Stenotrophomonas_sp._YAU14D1_LEIMI4_1	NA	NA	NA	NA	NA	NA	-2.125231191	-2.483685162	-1.571202337	-0.586988156	1.98021222	-7.890768904
Sphingopyxis_sp._MG	NA	NA	NA	NA	NA	NA	-2.0944694	-0.11645683	0.581016321	0	0	0
Kerstersia_gyiorum	NA	NA	NA	NA	NA	NA	-2.081897528	-2.303730171	-1.943780989	0	0	8.952254929
Psychrobacter_sp._G	NA	NA	NA	NA	NA	NA	-2.076068719	1.118154087	-0.908188675	0	0	0
Vibrio_aquimaris	NA	NA	NA	NA	NA	NA	-2.061359438	-10.47075037	-0.046962133	-1.678590021	-1.89592599	0.425360943
Mycobacterium_marinum	NA	NA	NA	NA	NA	NA	-2.041427817	0.178097729	0.649522447	-2.762242676	-3.812870415	-11.03525962
Xanthomonas_euroxanthea	NA	NA	NA	NA	NA	NA	-2.021791295	0.230752016	1.182294907	0	5.472487771	0
Pseudoalteromonas_arabiensis	NA	NA	NA	NA	NA	NA	-2.013162538	-3.178861055	-1.023777378	0	0	0
Candidatus_Moranella_endobia	NA	NA	NA	NA	NA	NA	-1.974702054	-3.701421382	-0.657409905	7.894817763	0	7.63420844
Candidatus_Nesciobacter_abundans	NA	NA	NA	NA	NA	NA	-1.935971814	1.250658094	0.140782542	-7.745956618	-1.27563614	0.004469003
Candidatus_Wolbachia_massiliensis	NA	NA	NA	NA	NA	NA	-1.92203375	1.218318604	1.577439009	1.366411237	1.493470728	-8.044394119
Acinetobacter_venetianus	NA	NA	NA	NA	NA	NA	-1.906275559	-10.06339508	0.583739512	-1.220925482	-2.199384985	-1.375087189
Arthrobacter_sp._PGP41	NA	NA	NA	NA	NA	NA	-1.898117281	-10.30750822	0.57318496	2.062284278	1.278859373	3.238966646
[Bacillus]_caldolyticus	NA	NA	NA	NA	NA	NA	-1.890679831	-0.61172416	-0.952060562	0	0	0
Janthinobacterium_svalbardensis	NA	NA	NA	NA	NA	NA	-1.884798629	-1.013510292	-0.895240143	-9.247927513	-9.247927513	-9.247927513
Altererythrobacter_epoxidivorans	NA	NA	NA	NA	NA	NA	-1.877399844	1.525976368	-0.472514691	0	8.41672576	9.372140267
Amycolatopsis_sp._BJA-103	NA	NA	NA	NA	NA	NA	-1.862499385	-0.583507454	0.087457195	0	9.33687918	0
Legionella_clemsonensis	NA	NA	NA	NA	NA	NA	-1.8346979	-1.247226956	-1.156624615	0	0	0
Mesorhizobium_sp._NZP2234	NA	NA	NA	NA	NA	NA	-1.833092358	-0.281094173	0.395295159	0	0	0
Azotobacter_chroococcum	NA	NA	NA	NA	NA	NA	-1.740221406	-0.22815782	0.917319146	1.975196609	2.438081102	1.653732312
Campylobacter_sp._RM8964	NA	NA	NA	NA	NA	NA	-1.697048762	-0.424932569	-1.06140802	0.026550433	0.735112486	1.614867312
Leclercia_sp._J807	NA	NA	NA	NA	NA	NA	-1.693994082	-1.992517334	0.581626109	0	6.815921204	0
Stenotrophomonas_indicatrix	NA	NA	NA	NA	NA	NA	-1.678242419	-0.703911428	-1.106017033	0	0	0
Chlamydia_caviae	NA	NA	NA	NA	NA	NA	-1.671322491	-0.151839895	1.340339265	0	0	0
Cellvibrio_sp._KY-GH-1	NA	NA	NA	NA	NA	NA	-1.671322491	1.347871074	0.535599761	-7.544323092	-7.544323092	2.490021948
Vibrio_rumoiensis	NA	NA	NA	NA	NA	NA	-1.64994457	1.016673101	-0.204916365	0	9.803054246	0
Sphingobium_sp._LF-16	NA	NA	NA	NA	NA	NA	-1.635588574	1.58947698	-9.764871591	8.033423002	7.996236198	8.412218392
Mycobacterium_marseillense	NA	NA	NA	NA	NA	NA	-1.613884874	-0.632544325	-0.54477992	0	0	0
Candidatus_Planktophila_sulfonica	NA	NA	NA	NA	NA	NA	-1.61320414	-0.539708676	-0.374023331	0	0	0
Actinomyces_gaoshouyii	NA	NA	NA	NA	NA	NA	-1.608898529	-1.439282053	-1.1472136	0	9.523561956	0
Streptomyces_sp._TN58	NA	NA	NA	NA	NA	NA	-1.591193833	-9.72047685	0.062372333	0	6.815921204	0
Janthinobacterium_agaricidamnosum	NA	NA	NA	NA	NA	NA	-1.57271458	1.02929658	0.818405205	0.830988447	-3.377176913	1.315242242
Kosakonia_oryzae	NA	NA	NA	NA	NA	NA	-1.552878366	-1.12633439	-1.435662268	-7.807354922	-7.807354922	-7.807354922
Actinosynnema_pretiosum	NA	NA	NA	NA	NA	NA	-1.543110979	0.518060335	0.675903722	-7.745956618	-7.745956618	-7.745956618
Klebsiella_sp._P1CD1	NA	NA	NA	NA	NA	NA	-1.518388426	0.087759052	0.102271868	0	0	0
Burkholderia_diffusa	NA	NA	NA	NA	NA	NA	-1.510749659	1.769970359	0.862008175	0	0	0
Ehrlichia_muris	NA	NA	NA	NA	NA	NA	-1.50196111	-0.241593356	-2.511125235	8.442943496	7.222389201	10.08214904
Pseudomonas_sp._TUM18999	NA	NA	NA	NA	NA	NA	-1.500372384	-2.288363974	-0.753203886	0	0	0
Streptomyces_sp._P3	NA	NA	NA	NA	NA	NA	-1.484398354	-0.251505091	0.436574708	-0.721140627	-9.087462841	-0.675244449
Synechocystis_sp._PCC_6803	NA	NA	NA	NA	NA	NA	-1.483917096	-10.61320011	0.809626809	-7.700439718	-7.700439718	-7.700439718
Synechococcus_sp._Minos11	NA	NA	NA	NA	NA	NA	-1.439103219	-0.09905882	-0.08837858	0	0	6.930737338
Enterobacter_sp._LU1	NA	NA	NA	NA	NA	NA	-1.429877496	-0.32528751	1.159300144	0	0	0
Candidatus_Filomicrobium_marinum	NA	NA	NA	NA	NA	NA	-1.425684296	-0.749441288	-0.740871622	-7.807354922	-7.807354922	-7.807354922
Lactococcus_sp._TMW21615	NA	NA	NA	NA	NA	NA	-1.424910183	-0.443525465	0.667209345	0	0	0
Mycolicibacterium_chitae	NA	NA	NA	NA	NA	NA	-1.406957981	-0.379246507	-0.885831064	0	0	0
Mycoplasma_sp._C264-NAS	NA	NA	NA	NA	NA	NA	-1.395316827	0.215458482	-1.553394754	10.28693889	0	8.741466986
Lelliottia_nimipressuralis	NA	NA	NA	NA	NA	NA	-1.38507994	-0.765534337	-0.093010052	0	0	0
Bacillus_sp._Lzh-5	NA	NA	NA	NA	NA	NA	-1.373349807	-1.618036353	0.749891008	7.333897756	0	0
Methylacidiphilum_kamchatkense	NA	NA	NA	NA	NA	NA	-1.352411871	-0.265696283	0.717571837	-8.202941691	-1.144051608	2.009761387
Gordonia_iterans	NA	NA	NA	NA	NA	NA	-1.325808722	-0.479919464	0.789891379	7.129283017	0	0
Staphylococcus_sp._MI_10-1553	NA	NA	NA	NA	NA	NA	-1.315832736	-1.01748445	-2.928218891	9.339850003	0	0
Delftia_sp._Cs1-4	NA	NA	NA	NA	NA	NA	-1.30802604	0.003004695	-1.312762392	-9.087462841	-9.087462841	-9.087462841
Citrobacter_sp._CRE-46	NA	NA	NA	NA	NA	NA	-1.304095569	-0.532122803	0.100571679	0	0	0
Pseudomonas_psychrophila	NA	NA	NA	NA	NA	NA	-1.291437739	0.933499036	1.131506837	0	8.979187942	0
Microbacterium_oxydans	NA	NA	NA	NA	NA	NA	-1.259940589	-9.863904389	-1.558462759	8.89683794	0	0
Pseudomonas_sp._CCOS_191	NA	NA	NA	NA	NA	NA	-1.248326487	-0.851594009	0.984600315	-7.890768904	-7.890768904	-7.890768904
Rhizobium_selenitireducens	NA	NA	NA	NA	NA	NA	-1.23235096	-0.762295172	0.492417626	0	9.214319121	0
Bradyrhizobium_guangdongense	NA	NA	NA	NA	NA	NA	-1.208417944	0.101045598	0.773819881	0	0	0
Salinivibrio_sp._YCSC6	NA	NA	NA	NA	NA	NA	-1.190161202	-0.145519522	-0.077503244	0	0	0
Entomoplasma_freundtii	NA	NA	NA	NA	NA	NA	-1.18656785	-1.511659887	0.332218194	0	0	0
Weissella_cryptocerci	NA	NA	NA	NA	NA	NA	-1.167685663	-0.127978765	0.664064129	-9.125844794	1.882114526	-9.125844794
Marinobacter_psychrophilus	NA	NA	NA	NA	NA	NA	-1.154259437	-0.877181423	-1.190373236	0	0	8.996239023
Neorickettsia_helminthoeca	NA	NA	NA	NA	NA	NA	-1.154013736	-0.749787912	-4.448110379	0	0	0
Pseudomonas_sp._LPH1	NA	NA	NA	NA	NA	NA	-1.153391403	-0.00249992	-0.74955978	-7.807354922	-7.807354922	-7.807354922
Kluyvera_cryocrescens	NA	NA	NA	NA	NA	NA	-1.153391403	1.908733984	0.198910341	0	0	0
Serratia_ureilytica	NA	NA	NA	NA	NA	NA	-1.151663056	0.332107758	-0.47832174	0	0	8.741466986
Phytobacter_diazotrophicus	NA	NA	NA	NA	NA	NA	-1.14863724	-0.269107901	0.132385864	0	0	0
Hypericibacter_adhaerens	NA	NA	NA	NA	NA	NA	-1.143103337	-0.591965187	0.06489054	1.732914298	1.474471221	-7.741466986
Sphingorhabdus_sp._SMR4y	NA	NA	NA	NA	NA	NA	-1.099755959	0.064017623	-0.83979633	7.303780748	8.979187942	0
Borrelia_duttonii	NA	NA	NA	NA	NA	NA	-1.099255006	-0.942437477	0.994606741	10.46964182	8.225607527	10.306825
Acetobacter_ascendens	NA	NA	NA	NA	NA	NA	-1.097810533	1.280951108	-0.60764612	-1.446648222	0.245810052	-0.338210579
Burkholderia_sp._BDU6	NA	NA	NA	NA	NA	NA	-1.086675357	-0.317726217	-0.894976382	0	0	0
Altererythrobacter_mangrovi	NA	NA	NA	NA	NA	NA	-1.084941127	-2.405285111	-1.17423443	8.86005338	0	0
Sphingomonas_daechungensis	NA	NA	NA	NA	NA	NA	-1.076952533	-1.293809435	-0.12991018	0	0	0
Mesorhizobium_sp._INR15	NA	NA	NA	NA	NA	NA	-1.070564037	0.132326034	-1.292957784	0	5.472487771	0
Cycloclasticus_sp._P1	NA	NA	NA	NA	NA	NA	-1.070188561	0.071677633	0.197108627	0	0	0
Citrobacter_youngae	NA	NA	NA	NA	NA	NA	-1.05978902	0.376292634	0.574814602	0	0	0
Sodalis_endosymbiont_of_Henestaris_halophilus	NA	NA	NA	NA	NA	NA	-1.051204157	0.369471714	1.065002608	-1.371906497	2.277467799	0.151900574

Rickettsia_asiatica	NA	NA	NA	NA	NA	NA	-1.042433562	-0.206564801	0.220399827	0	5.472487771	0
Streptococcus_gwangjuense	NA	NA	NA	NA	NA	NA	-1.040784254	1.392963142	1.357447613	0	0	0
Stenotrophomonas_sp._169	NA	NA	NA	NA	NA	NA	-1.034623092	-0.361489576	-0.694432858	7.129283017	9.215938208	0
Escherichia_sp._E4742	NA	NA	NA	NA	NA	NA	-1.028295146	0.392603008	1.642174041	0	0	7.539158811
Pseudomonas_veronii	NA	NA	NA	NA	NA	NA	-1.01993468	-0.669681898	0.356643478	8.442943496	0	0
Micromonospora_aurantiaca	NA	NA	NA	NA	NA	NA	-1.018537882	-0.093888189	0.433450753	0	0	0
Burkholderia_sp._DHOD12	NA	NA	NA	NA	NA	NA	-1.004674736	-11.13395775	-0.329681502	8.27301694	0	0
Bacillus_sp._IHB_B_7164	NA	NA	NA	NA	NA	NA	-0.995545068	0.648901794	0.93854012	0	0	0
Geobacillus_sp._JS12	NA	NA	NA	NA	NA	NA	-0.990897038	0.253364406	-0.224000835	0	0	0
Microbacterium_sp._PM5	NA	NA	NA	NA	NA	NA	-0.985094873	0.227650386	-0.038536499	7.303780748	5.472487771	0
Chromobacterium_sp._IIBBL_112-1	NA	NA	NA	NA	NA	NA	-0.973407266	-3.320649527	0.955891154	0	0	0
Pectobacterium_wasabiae	NA	NA	NA	NA	NA	NA	-0.965457223	-0.149532813	-0.77188402	0.163380524	-8.202941691	0.749313238
Streptomyces_asterosporus	NA	NA	NA	NA	NA	NA	-0.956824355	-0.195964718	-0.800906212	0	0	0
Shigella_dysenteriae	NA	NA	NA	NA	NA	NA	-0.954862727	-0.455449448	-0.041536231	0	0	0
Shigella_flexneri	NA	NA	NA	NA	NA	NA	-0.951914107	-0.406078621	0.223373667	0	0	0
Kosakonia_sp._CCTCC_M2018092	NA	NA	NA	NA	NA	NA	-0.943456408	-2.189935813	-0.30753912	-10.65999589	-10.65999589	-10.65999589
Klebsiella_sp._PO552	NA	NA	NA	NA	NA	NA	-0.935222536	-0.809694132	-1.496514832	0	0	0
Methylobacterium_phyllosphaerae	NA	NA	NA	NA	NA	NA	-0.914732314	-1.562515026	1.905825883	0	7.59792591	0
Pseudomonas_sp._ADAK18	NA	NA	NA	NA	NA	NA	-0.912392006	1.894959306	-2.912395039	0	9.54432116	8.658211483
Pseudomonas_sp._LTJR-52	NA	NA	NA	NA	NA	NA	-0.907886272	-0.186321711	-0.595164089	8.86005338	8.215935782	0
Fervidobacterium_islandicum	NA	NA	NA	NA	NA	NA	-0.906434392	-0.406030552	-0.841816598	0	0	7.63420844
Candidatus_Arsenophonus_lipoptenae	NA	NA	NA	NA	NA	NA	-0.903028584	1.104040216	0.568150688	0	5.472487771	8.658211483
Massilia_albidiflava	NA	NA	NA	NA	NA	NA	-0.902552172	0.352295143	0.47630211	-0.034858734	2.296678445	0.18135894
Azospirillum_humicireducens	NA	NA	NA	NA	NA	NA	-0.901449892	0.229565874	-0.307209667	0	0	0
Candidatus_Vesicomyosocius_okutanii	NA	NA	NA	NA	NA	NA	-0.89891588	-2.019527039	0.762337333	0	0	8.761551232
Pseudomonas_sp._SCB32	NA	NA	NA	NA	NA	NA	-0.884241885	-9.488205685	-0.331088689	0	0	0
Rhizobium_sp._CIAT894	NA	NA	NA	NA	NA	NA	-0.884241885	-0.108039033	3.30077156	0	8.776982369	7.539158811
Corynebacterium_testudinoris	NA	NA	NA	NA	NA	NA	-0.883115672	-0.532855205	-0.734247145	7.333897756	0	0
Wolbachia_endosymbiont_of_Cruorifilaria_tuberocauda	NA	NA	NA	NA	NA	NA	-0.870290219	-0.826353856	-0.438019589	-8.087462841	-8.087462841	-8.087462841
Thalassotalea_crassostreae	NA	NA	NA	NA	NA	NA	-0.859605825	-0.863756225	-1.286082772	-2.940975523	-10.24475627	0.143982182
Thermomonas_sp._XSG	NA	NA	NA	NA	NA	NA	-0.832649632	-0.623330738	-0.413941806	-0.386337744	-0.599531044	-0.626815165
Pseudarthrobacter_sulfonivorans	NA	NA	NA	NA	NA	NA	-0.832594214	-0.094208423	1.195341644	0	0	9.736965031
Actinobacillus_equuli	NA	NA	NA	NA	NA	NA	-0.828312241	-1.501705303	-0.002567428	-7.700439718	-7.700439718	-7.700439718
Salinibacterium_sp._UTAS2018	NA	NA	NA	NA	NA	NA	-0.828082557	-0.511774683	0.31755148	-9.247927513	-9.247927513	-2.703607512
Burkholderia_pyrrocinia	NA	NA	NA	NA	NA	NA	-0.826539945	-0.362731548	0.075971232	0	0	7.539158811
Streptococcus_sp._HSISS2	NA	NA	NA	NA	NA	NA	-0.81991345	-0.184273979	0.744614655	0	8.912889336	0
Enterobacter_sp._HK169	NA	NA	NA	NA	NA	NA	-0.802449264	-1.361796977	0.805683301	0	0	0
Paraburkholderia_sp._Msb3	NA	NA	NA	NA	NA	NA	-0.801313903	1.805264566	2.221378762	0	0	0
Exiguobacterium_sp._U13-1	NA	NA	NA	NA	NA	NA	-0.800033662	-1.464833147	-0.778831312	0	6.815921204	8.658211483
Acinetobacter_piscicola	NA	NA	NA	NA	NA	NA	-0.795208243	1.57434372	2.397067177	0	0	0
Mesorhizobium_sp._WSM1497	NA	NA	NA	NA	NA	NA	-0.78634807	-0.482183158	-0.267757758	0	0	0
Candidatus_Purcelliella_pentastirinorum	NA	NA	NA	NA	NA	NA	-0.786036791	0.31516159	0.23081842	-7.890768904	1.054870985	0.870782328
Mycolicibacterium_monacense	NA	NA	NA	NA	NA	NA	-0.785294935	1.265651673	1.78839426	7.333897756	0	0
Polaromonas_sp._JS666	NA	NA	NA	NA	NA	NA	-0.782207972	0.601391918	0.849962941	8.45395786	9.214319121	0
Serratia_quinivorans	NA	NA	NA	NA	NA	NA	-0.761777316	-1.172562963	0.717802621	0	0	0
Sulfitobacter_guttiformis	NA	NA	NA	NA	NA	NA	-0.75787024	-10.95128471	-10.95128471	0	0	0
Blattabacterium_clevelandi	NA	NA	NA	NA	NA	NA	-0.75222841	-1.88220409	0.667632136	0	0	0
Kocuria_sp._KD4	NA	NA	NA	NA	NA	NA	-0.739026495	-0.406028609	-0.985282694	0	0	0
Enterobacter_cloacae_complex_sp._FDA-CDC-AR_0132	NA	NA	NA	NA	NA	NA	-0.738268356	-0.02525719	-0.642537805	0	0	0
Paenarthrobacter_aurescens	NA	NA	NA	NA	NA	NA	-0.725647076	-4.415242	0.515710125	0	0	8.520944699
Streptomyces_roseochromogenus	NA	NA	NA	NA	NA	NA	-0.719506368	-9.222246695	0.228964416	0	8.776982369	0
Acinetobacter_towneri	NA	NA	NA	NA	NA	NA	-0.70751936	-0.379453571	0.936389188	0	0	0
Nitrosococcus_halophilus	NA	NA	NA	NA	NA	NA	-0.706600085	-10.02236781	-0.556244435	-2.55838485	-9.687667867	-2.306843198
Agrobacterium_sp._MA01	NA	NA	NA	NA	NA	NA	-0.70213612	-0.434699599	0.76769085	0	0	0
Micromonospora_coriariae	NA	NA	NA	NA	NA	NA	-0.677446832	0.303392969	0.098704916	0	0	0
Polaromonas_sp.	NA	NA	NA	NA	NA	NA	-0.674180453	-0.268236046	0.647227867	0	0	8.736966721
Enterobacter_sp._RHBSTW-00975	NA	NA	NA	NA	NA	NA	-0.665169319	0.208912862	-9.953931598	0	0	0
Agrobacterium_sp._33MFTa1.1	NA	NA	NA	NA	NA	NA	-0.659523441	0.103707818	1.136321471	0	0	0
Rhizobium_sp._S41	NA	NA	NA	NA	NA	NA	-0.646033867	0.611110708	0.718482898	0	0	0
Mycoplasma_ovipneumoniae	NA	NA	NA	NA	NA	NA	-0.645451714	-0.075922816	0.361985332	0	0	0
Nostoc_sp._'Peltigera_membranacea_cyanobiont'_N6	NA	NA	NA	NA	NA	NA	-0.642498071	0.025617498	-0.033612388	0	0	0
Shewanella_sp._MR-7	NA	NA	NA	NA	NA	NA	-0.639193579	0.245143798	0.132384939	-7.807354922	2.218711987	1.71096973
Enterobacteriaceae_endosymbiont_of_Plateumaris_sericea	NA	NA	NA	NA	NA	NA	-0.637998807	0.088115111	-1.155885261	8.442943496	9.606096097	10.3479867
Mesoplasma_entomophilum	NA	NA	NA	NA	NA	NA	-0.635427336	0.160774314	0.344818681	-9.247927513	0.070389328	-9.247927513
Burkholderia_sp._PAMC_26561	NA	NA	NA	NA	NA	NA	-0.627640808	-1.055163915	-0.744453569	-1.399519721	0.612502816	-8.820178962
Marinomonas_posidonica	NA	NA	NA	NA	NA	NA	-0.62542956	-1.939880661	-1.850456881	-8.044394119	-8.044394119	-8.044394119
Enterobacter_sp._CRENT-193	NA	NA	NA	NA	NA	NA	-0.623626418	-0.117421919	0.414407827	0	0	0
Enterobacteriaceae_endosymbiont_of_Donacia_tomentosa	NA	NA	NA	NA	NA	NA	-0.618282896	1.297566444	-1.477110226	0	0	0
Synechococcus_sp._PCC_7002	NA	NA	NA	NA	NA	NA	-0.607267814	0.0718158	0.079369788	0	0	0
Borrelia_turcica	NA	NA	NA	NA	NA	NA	-0.597633804	0.024176498	0.191769938	-7.544323092	1.669996028	-7.544323092
Streptomyces_avermitilis	NA	NA	NA	NA	NA	NA	-0.59740686	-0.441616844	-0.259893327	-7.807354922	-7.807354922	2.790073827
Neisseria_cinerea	NA	NA	NA	NA	NA	NA	-0.596469369	-3.187739808	1.256509485	0	7.996236198	6.544320001
Alteromonas_sp._MB-3u-76	NA	NA	NA	NA	NA	NA	-0.596179926	-2.115411501	-1.422224001	-0.56910855	-1.27423836	0.881697064
Mycoplasma_columbinasale	NA	NA	NA	NA	NA	NA	-0.595828153	-0.609566416	-1.756802408	-8.044394119	-2.571906348	-8.044394119
Blattabacterium_sp._(Blaberus_giganteus)	NA	NA	NA	NA	NA	NA	-0.59493223	0.431379544	0.3461799	0	0	0
Streptococcus_sp._CNU_G3	NA	NA	NA	NA	NA	NA	-0.594068236	0.372593272	0.049159131	0	0	0
Anaplasma_platys	NA	NA	NA	NA	NA	NA	-0.589398042	-0.713197285	-0.269656244	0	0	7.539158811
Stenotrophomonas_sp._SAU14A_NAIMI4_8	NA	NA	NA	NA	NA	NA	-0.586198243	0.993708244	1.54605266	0	0	0
Sphingomonas_sp._HKS19	NA	NA	NA	NA	NA	NA	-0.582896409	-0.46073905	-2.171231573	-8.709659397	-1.893738193	-1.778922059
Polaribacter_sp._SA4-12	NA	NA	NA	NA	NA	NA	-0.579701548	-0.660916806	-0.060118131	8.868307104	8.979187942	0
Orbus_sp._IPMB12	NA	NA	NA	NA	NA	NA	-0.574381547	-1.040864146	-0.467733859	-0.706319807	0.355866226	0.081719972
Nostoc_sp._TCL240-02	NA	NA	NA	NA	NA	NA	-0.558167629	-0.848915755	-0.920737709	8.960001932	5.472487771	0
Acinetobacter_sp._NEB_394	NA	NA	NA	NA	NA	NA	-0.552936846	0.453529545	-8.799135887	0	0	0
Actinosynnema_mirum	NA	NA	NA	NA	NA	NA	-0.53062618	0.116141662	-0.297529807	0	7.369230901	0
Candidatus_Paracaedibacter_acanthamoebae	NA	NA	NA	NA	NA	NA	-0.513534994	1.301068786	0.823108869	0	0	0
Mesorhizobium_sp._Pch-S	NA	NA	NA	NA	NA	NA	-0.50635349	-0.243709867	2.399599049	0	0	0
Acinetobacter_sp._C16S1	NA	NA	NA	NA	NA	NA	-0.504234577	0.630213464	-0.519490776	0	0	0
Shewanella_putrefaciens	NA	NA	NA	NA	NA	NA	-0.493074276	0.751552994	-1.867540687	-1.435436172	0.4751022	-8.739216921
Aureimonas_sp._LMG_31693	NA	NA	NA	NA	NA	NA	-0.489857597	-0.606096675	0.2328222	0.789523692	-0.98055249	0.650888701
Sphingopyxis_sp._FD7	NA	NA	NA	NA	NA	NA	-0.480164672	-8.637284195	0.878056896	0	0	8.129283017
Chryseobacterium_sp._SNU_WT5	NA	NA	NA	NA	NA	NA	-0.456884177	-0.656492238	0.305893341	0	0	8.520944699
Acinetobacter_shaoyimingii	NA	NA	NA	NA	NA	NA	-0.45681301	-0.650526878	-0.434783997	0	0	7.380824669
Enterobacter_sp._18A13	NA	NA	NA	NA	NA	NA	-0.455144752	-0.781258702	1.085043455	0	0	0
Burkholderia_plantarii	NA	NA	NA	NA	NA	NA	-0.454985626	2.371745871	2.798080462	0.635588574	-0.584965721	-7.807354922
Citrobacter_sp._RHB35-C21	NA	NA	NA	NA	NA	NA	-0.45229785	1.872754035	2.148786253	0	6.815921204	0
Otariodibacter_oris	NA	NA	NA	NA	NA	NA	-0.447868833	0.448106637	0.613971031	0	0	7.539158811
Acinetobacter_wanghuae	NA	NA	NA	NA	NA	NA	-0.430071805	0.310546006	0.86893093	0	5.472487771	10.1514352
Mesoplasma_syrphidae	NA	NA	NA	NA	NA	NA	-0.428517747	2.003037872	0.300863181	10.59308294	8.809501266	7.539158811
Pseudomonas_sp._SWI7	NA	NA	NA	NA	NA	NA	-0.425793604	0.684985713	-0.750054206	0	0	0
Pseudomonas_cremoricolorata	NA	NA	NA	NA	NA	NA	-0.423453262	0.573020427	0.486794618	0	9.569855608	0
Pseudoalteromonas_sp._THAF3	NA	NA	NA	NA	NA	NA	-0.423213177	-8.552496194	-8.552496194	0	0	0
Candidatus_Kinetoplastibacterium_oncopeltii	NA	NA	NA	NA	NA	NA	-0.418975583	-0.217441992	-0.189008303	-0.314354336	-0.065315943	-0.876152827
Mycolicibacterium_phocaicum	NA	NA	NA	NA	NA	NA	-0.417705058	-0.740964192	1.085819535	0	0	0
Candidatus_Kinetoplastibacterium_crithidii	NA	NA	NA	NA	NA	NA	-0.417039021	-0.22660782	-0.163144724	0	0	0
Candidatus_Liberibacter_solanacearum	NA	NA	NA	NA	NA	NA	-0.414295965	1.032967479	1.328583351	-8.048034513	0.728947856	3.351777446
Paracoccus_mutanolyticus	NA	NA	NA	NA	NA	NA	-0.412717577	-0.874485173	-0.006076599	0	0	0
Anoxybacillus_sp._PDR2	NA	NA	NA	NA	NA	NA	-0.412513098	-0.280417813	0.45320513	0	0	0
Paraburkholderia_sp._PGU16	NA	NA	NA	NA	NA	NA	-0.4082568	-0.303323562	-1.116993139	0	0	9.495855027
Cronobacter_dublinensis	NA	NA	NA	NA	NA	NA	-0.408150961	0.700618827	0.742475267	0	8.912889336	8.412218392
Parasphingopyxis_sp._CP4	NA	NA	NA	NA	NA	NA	-0.399064449	0.918168418	1.309613398	0	0	0
Bacillus_sp._PAMC28748	NA	NA	NA	NA	NA	NA	-0.395859825	0.53577796	-0.043935722	-8.048034513	-8.048034513	-0.508875702

Proteus_cibarius	NA	NA	NA	NA	NA	NA	-0.394097136	-1.428989047	-1.407101511	0	0	0
Microbacterium_chocolatum	NA	NA	NA	NA	NA	NA	-0.391085154	2.939884963	-0.156401115	8.982993575	7.222389201	0
Vibrio_jasicida	NA	NA	NA	NA	NA	NA	-0.387198025	0.184037493	0.53609164	8.969625392	0	9.438791853
Veillonella_sp._T1-7	NA	NA	NA	NA	NA	NA	-0.386144101	0.108023963	-0.572246235	9.699282671	8.225607527	8.398029648
Aliivibrio_salmonicida	NA	NA	NA	NA	NA	NA	-0.384748725	-1.249406502	0.144454943	0	8.776982369	0
Klebsiella_grimontii	NA	NA	NA	NA	NA	NA	-0.381864172	-0.629117505	-0.542841242	0	0	0
Mycoavidus_sp._B2-EB	NA	NA	NA	NA	NA	NA	-0.371784117	0.437830386	1.228915745	0	0	7.380824669
Azospira_sp._I09	NA	NA	NA	NA	NA	NA	-0.371415812	1.436679612	0.337677503	-8.924812504	-0.121756642	0.542793046
Rhodoluna_lacicola	NA	NA	NA	NA	NA	NA	-0.371062068	0.53743209	-0.248044538	0	0	0
Corynebacterium_sp._L2-79-05	NA	NA	NA	NA	NA	NA	-0.370565136	0.04803905	-0.069487543	0	8.776982369	7.63420844
Alteromonas_sp._BL110	NA	NA	NA	NA	NA	NA	-0.368585662	-0.037381309	0.062673843	0	0	0
Amycolatopsis_sp._YIM_10	NA	NA	NA	NA	NA	NA	-0.365259415	1.174538931	1.096467566	-9.431846483	-9.431846483	-9.431846483
Candidatus_Portiera_aleyrodidarum	NA	NA	NA	NA	NA	NA	-0.354891108	0.177396935	0.348406383	-2.214282598	-9.548180354	0.563829017
Bartonella_clarridgeiae	NA	NA	NA	NA	NA	NA	-0.352151491	0.869654795	0.829571668	0	0	6.544320001
Stanieria_sp._NIES-3757	NA	NA	NA	NA	NA	NA	-0.351779054	0.344698327	-0.038068041	9.252665432	8.425144554	0
Paracoccus_sp._BM15	NA	NA	NA	NA	NA	NA	-0.350225149	0.297761299	0.172142856	8.6794801	7.222389201	0
Acinetobacter_sp._MYb10	NA	NA	NA	NA	NA	NA	-0.3476579	0.376965663	0.368437391	0.27759617	-2.318759596	-0.746753692
Brevundimonas_sp._M20	NA	NA	NA	NA	NA	NA	-0.339448812	0.794465551	-0.538072499	0	0	0
Polynucleobacter_sp._LimPoW16	NA	NA	NA	NA	NA	NA	-0.33259557	0.418023012	0.223101096	-1.288855458	-1.55936504	0.363353039
Klebsiella_sp._WP8-S18-ESBL-06	NA	NA	NA	NA	NA	NA	-0.331705105	-0.784501634	-1.415228238	0	0	0
Luteimonas_sp._YGD11-2	NA	NA	NA	NA	NA	NA	-0.325658835	0.61849517	-0.787237628	0	0	0
Exiguobacterium_acetylicum	NA	NA	NA	NA	NA	NA	-0.316556379	-0.206275689	1.829660136	0	0	0
Serratia_sp._3ACOL1	NA	NA	NA	NA	NA	NA	-0.309077235	1.157868836	0.767702262	0	0	0
Clostridium_sporogenes	NA	NA	NA	NA	NA	NA	-0.30682832	-0.415498141	-0.089958357	0.215937399	-0.224481165	-0.135579961
Agrobacterium_sp._H13-3	NA	NA	NA	NA	NA	NA	-0.306050459	0.586549642	1.427332972	0	0	0
Blattabacterium_punctulatus	NA	NA	NA	NA	NA	NA	-0.305907006	0.030790266	-0.068709296	0	0	0
Burkholderia_latens	NA	NA	NA	NA	NA	NA	-0.277754612	-1.047322486	0.537452257	0	0	0
Erwinia_pyrifoliae	NA	NA	NA	NA	NA	NA	-0.275866351	-0.00966235	0.985339041	0	0	0
Pasteurella_stomatis	NA	NA	NA	NA	NA	NA	-0.269041772	0.467049287	-0.603030442	0	0	9.572383803
Xanthomonas_axonopodis	NA	NA	NA	NA	NA	NA	-0.265843926	0.811918044	-10.00332114	0	6.470320477	8.952254929
Exiguobacterium_sibiricum	NA	NA	NA	NA	NA	NA	-0.262407806	0.130039557	0.964095517	-1.680218249	-3.337013494	0.497319177
Stenotrophomonas_sp._ZAC14D2_NAIMI4_7	NA	NA	NA	NA	NA	NA	-0.245822397	-0.25810969	-0.316175179	0	0	8.952254929
Mannheimia_sp._16CN0041	NA	NA	NA	NA	NA	NA	-0.231200418	0.415699236	0.998878326	0	8.488109258	9.060695932
Enterobacteriaceae_endosymbiont_of_Donacia_piscatrix	NA	NA	NA	NA	NA	NA	-0.229153275	1.464185387	2.895896309	0	0	10.83289001
Legionella_waltersii	NA	NA	NA	NA	NA	NA	-0.22881869	-0.023807469	2.752620156	-10.58871464	-3.366325435	-2.176496244
Gordonia_insulae	NA	NA	NA	NA	NA	NA	-0.223081657	0.705589269	1.571650348	-0.597211925	-0.714299015	-9.693486957
Brevundimonas_sp._GW460-12-10-14-LB2	NA	NA	NA	NA	NA	NA	-0.214331529	1.257965377	0.118973563	0	0	0
Enterobacter_sp._RHB15-C17	NA	NA	NA	NA	NA	NA	-0.213025448	0.140899787	0.27692429	-7.890768904	-7.890768904	-7.890768904
Marinitoga_piezophila	NA	NA	NA	NA	NA	NA	-0.205979395	0.065972032	-0.967124801	0	0	0
Metakosakonia_sp._MRY16-398	NA	NA	NA	NA	NA	NA	-0.201368384	-1.129790564	0.15227307	0	0	0
Lysobacter_sp._H23M41	NA	NA	NA	NA	NA	NA	-0.199992058	-0.156325206	0.11547742	-0.413579099	0.791606528	-0.189516111
Mycoplasmopsis_columboralis	NA	NA	NA	NA	NA	NA	-0.195172088	0.828232014	0.043500452	0	8.215935782	0
Acinetobacter_sp._NCu2D-2	NA	NA	NA	NA	NA	NA	-0.192064465	0.642016078	0.639449088	-0.95037776	-11.40158887	-11.40158887
Bordetella_parapertussis	NA	NA	NA	NA	NA	NA	-0.185744542	-0.211110337	0.345926028	0	0	0
Shigella_boydii	NA	NA	NA	NA	NA	NA	-0.185468139	0.148924157	0.699973585	-7.700439718	-7.700439718	-7.700439718
Nostoc_sp._NIES-2111	NA	NA	NA	NA	NA	NA	-0.181825539	-2.974867302	-2.015781661	0	7.369230901	0
Pseudarthrobacter_sp._NIBRBAC000502772	NA	NA	NA	NA	NA	NA	-0.179808751	2.476393843	-0.033334912	-8.930737338	-0.0278588	0.259087221
Phaeobacter_piscinae	NA	NA	NA	NA	NA	NA	-0.177486728	-3.185448715	1.477689619	-7.709655951	-7.709655951	-7.709655951
Campylobacter_sp._RM16192	NA	NA	NA	NA	NA	NA	-0.176293452	1.390189145	-0.183341764	-1.115689085	-2.47631247	-3.140445184
Candidatus_Ishikawaella_capsulata	NA	NA	NA	NA	NA	NA	-0.17516904	-0.242887017	-0.230389742	0	0	0
Mycoplasma_haemofelis	NA	NA	NA	NA	NA	NA	-0.171592802	-0.302118349	0.458319403	0	0	0
Corynebacterium_singulare	NA	NA	NA	NA	NA	NA	-0.17143483	-0.289330658	0.248565788	-0.899160943	0.709947646	-8.202941691
Idiomarina_piscisalsi	NA	NA	NA	NA	NA	NA	-0.162563057	-0.748883536	-0.748883536	0	6.815921204	7.63420844
Paraburkholderia_xenovorans	NA	NA	NA	NA	NA	NA	-0.161657283	-1.146524671	-0.400364711	0	10.06877828	7.544323092
Leuconostoc_kimchii	NA	NA	NA	NA	NA	NA	-0.161599563	0.515326411	0.296526811	-9.247927513	-9.247927513	-9.247927513
Sphingorhabdus_sp._M41	NA	NA	NA	NA	NA	NA	-0.158709284	0.148147442	0.531009823	7.129283017	5.472487771	0
Amycolatopsis_keratiniphila	NA	NA	NA	NA	NA	NA	-0.157436875	-0.46531891	0.7515349	0	0	0
Hartmannibacter_diazotrophicus	NA	NA	NA	NA	NA	NA	-0.156573233	-0.022756535	0.394027513	0	9.338364242	11.11243951
Vibrio_rotiferianus	NA	NA	NA	NA	NA	NA	-0.155594777	-0.316556082	-0.171467827	0	9.358431429	0
Klebsiella_sp._WP4-W18-ESBL-05	NA	NA	NA	NA	NA	NA	-0.150245456	-0.050438439	-0.700618377	0	0	0
Rhizobium_favelukesii	NA	NA	NA	NA	NA	NA	-0.149058768	-0.692228136	-0.611814806	0	10.73301532	0
Campylobacter_mucosalis	NA	NA	NA	NA	NA	NA	-0.148494157	0.092347799	1.014235343	-0.232835105	-0.319306971	-0.2201133
Lactobacillus_sp._JM1	NA	NA	NA	NA	NA	NA	-0.144146668	-0.300448232	-0.708765591	0	0	0
Sulfurimonas_sp._1-1N	NA	NA	NA	NA	NA	NA	-0.143105258	2.674680497	0.924709574	-0.881605299	-9.247927513	-0.726982815
Pseudomonas_extremaustralis	NA	NA	NA	NA	NA	NA	-0.137989117	0.082180797	-0.321681878	-9.184875343	-9.184875343	-9.184875343
Variovorax_sp._PAMC_28711	NA	NA	NA	NA	NA	NA	-0.133628257	1.200632837	1.189122214	0	0	0
Rhodoferax_sp._BAB1	NA	NA	NA	NA	NA	NA	-0.133421668	-0.511519997	-0.777209467	0	0	7.63420844
Pseudomonas_glycinae	NA	NA	NA	NA	NA	NA	-0.121755761	0.405572303	-0.663029417	0	0	0
Aeromicrobium_choanae	NA	NA	NA	NA	NA	NA	-0.11593146	-0.622582006	-1.037989474	0	0	0
Agromyces_aureus	NA	NA	NA	NA	NA	NA	-0.11570207	1.116127921	0.475373509	-7.741466986	2.329995376	-7.741466986
Pseudomonas_denitrificans_(nom._rej.)	NA	NA	NA	NA	NA	NA	-0.110562195	0.654514231	0.746899088	9.020516091	6.815921204	0
Acinetobacter_equi	NA	NA	NA	NA	NA	NA	-0.109088424	0.164814079	0.064527754	0	0	8.658211483
Bacillus_sp._ABP14	NA	NA	NA	NA	NA	NA	-0.106868369	-1.707862879	-1.922217026	0	0	8.520944699
Pseudomonas_amygdali	NA	NA	NA	NA	NA	NA	-0.103814033	0.528253414	0.340137264	0	0	0
Agrobacterium_sp._CGMCC_11546	NA	NA	NA	NA	NA	NA	-0.098779068	1.049772262	0.603656237	0	0	0
Herminiimonas_arsenitoxidans	NA	NA	NA	NA	NA	NA	-0.097708242	0.1423551	-0.109322722	0	0	9.306820443
Prochlorococcus_sp._MIT_0604	NA	NA	NA	NA	NA	NA	-0.090795053	-0.09366962	-0.127479896	0	0	8.952254929
Mesorhizobium_sp._NZP2077	NA	NA	NA	NA	NA	NA	-0.089939567	-0.122814929	0.674928934	-7.700439718	1.636439462	0.425402493
Shewanella_maritima	NA	NA	NA	NA	NA	NA	-0.079913324	-1.531977998	0.431090465	0	0	0
Sphingomonas_sp._Cra20	NA	NA	NA	NA	NA	NA	-0.078655403	-0.105350292	0.51860584	0	0	0
Pseudomonas_simiae	NA	NA	NA	NA	NA	NA	-0.075483746	-0.18694953	0.288700132	0	0	0
Colwellia_beringensis	NA	NA	NA	NA	NA	NA	-0.069434482	0.329767694	0.423365157	8.27301694	6.815921204	8.950313848
Brucella_melitensis	NA	NA	NA	NA	NA	NA	-0.061422608	0.134034918	1.939417523	7.303780748	0	0
Pseudoalteromonas_nigrifaciens	NA	NA	NA	NA	NA	NA	-0.061226307	0.063363378	1.659594262	0	0	8.520944699
Kocuria_rhizophila	NA	NA	NA	NA	NA	NA	-0.05953692	0.119392316	0.619554166	0	0	0
Cylindrospermopsis_curvispora	NA	NA	NA	NA	NA	NA	-0.057810225	-0.153561085	-0.740374234	0	0	0
Aeromonas_sp._Ne-1	NA	NA	NA	NA	NA	NA	-0.056504389	-0.834060729	-1.068311231	0	0	0
Candidatus_Nasuia_deltocephalinicola	NA	NA	NA	NA	NA	NA	-0.054849295	-1.533033765	-4.28280787	-8.904884456	-8.904884456	-0.246672974
Fischerella_sp._NIES-3754	NA	NA	NA	NA	NA	NA	-0.053158823	0.791507388	0.762922772	0.102275772	-2.025538312	-0.027145336
Burkholderia_sp._IDO3	NA	NA	NA	NA	NA	NA	-0.050815772	0.161362836	-0.494138266	0	0	0
Citrobacter_sp._RHBSTW-00524	NA	NA	NA	NA	NA	NA	-0.049755867	2.206334957	0.81382602	0	0	0
Acinetobacter_sp._SWBY1	NA	NA	NA	NA	NA	NA	-0.049572386	1.401138607	1.572515357	8.366322214	0	0
Rickettsia_amblyommatis	NA	NA	NA	NA	NA	NA	-0.046301505	0.590656071	1.330030725	0	0	0
Citrobacter_sp._LUTT5	NA	NA	NA	NA	NA	NA	-0.03603561	0.518603253	-0.204900015	0	0	0
Acinetobacter_sp._TTH0-4	NA	NA	NA	NA	NA	NA	-0.034713175	1.934546995	1.458317939	0.074000581	-2.189037431	0.155793963
Candidatus_Kinetoplastibacterium_galatii	NA	NA	NA	NA	NA	NA	-0.033994882	-0.225436703	-0.214392895	0	0	0
Streptococcus_sp._FDAARGOS_521	NA	NA	NA	NA	NA	NA	-0.03316761	0.529604422	-0.062011206	0	0	0
Mesorhizobium_sp._M1E.F.Ca.ET.045.02.1.1	NA	NA	NA	NA	NA	NA	-0.024044954	2.238485142	3.046096415	-0.407569231	-2.268979215	-0.360642317
Mesorhizobium_erdmanii	NA	NA	NA	NA	NA	NA	-0.011295174	-0.304223446	-0.619117792	0	0	8.741466986
Mesorhizobium_sp._M2A.F.Ca.ET.043.02.1.1	NA	NA	NA	NA	NA	NA	0	0	0	-10.24475627	-3.774435794	-2.70559746
Lactococcus_sp._TMW21612	NA	NA	NA	NA	NA	NA	0	0	0	-10.21674586	-1.002426737	-2.672422766
Sulfurospirillum_barnesii	NA	NA	NA	NA	NA	NA	0	0	0	-9.536571664	-9.536571664	-1.902363224
Bartonella_sp._1-1C	NA	NA	NA	NA	NA	NA	0	0	0	-9.087462841	-9.087462841	-9.087462841
Marinomonas_arctica	NA	NA	NA	NA	NA	NA	0	0	0	-9.018664771	-9.018664771	-9.018664771
Marinobacterium_sp._LSUCC0821	NA	NA	NA	NA	NA	NA	0	0	0	-8.186525318	-8.186525318	-8.186525318
Microterricola_viridarii	NA	NA	NA	NA	NA	NA	0	0	0	-8.048034513	-8.048034513	2.240450944
Methylotenera_versatilis	NA	NA	NA	NA	NA	NA	0	0	0	-8.044394119	2.396475048	0.476550579
Bradyrhizobium_sp._WSM471	NA	NA	NA	NA	NA	NA	0	0	0	-7.930737338	-7.930737338	-7.930737338
Microbacterium_sp._CGR1	NA	NA	NA	NA	NA	NA	0	0	0	-7.745956618	-7.745956618	-7.745956618
Bradyrhizobium_genosp._L	NA	NA	NA	NA	NA	NA	0	0	0	-7.741466986	-7.741466986	-7.741466986
Vibrio_cidicii	NA	NA	NA	NA	NA	NA	0	0	0	-7.741466986	-0.925545783	1.550088227

Pseudomonas_moraviensis	NA	NA	NA	NA	NA	NA	0	0	0	-7.709655951	-7.709655951	-7.709655951
Streptococcus_sp._NPS_308	NA	NA	NA	NA	NA	NA	0	0	0	-7.709655951	1.79549463	-7.709655951
Bordetella_avium	NA	NA	NA	NA	NA	NA	0	0	0	-7.544323092	-7.544323092	-7.544323092
Enterococcus_sp._HSIEG1	NA	NA	NA	NA	NA	NA	0	0	0	-1.914029758	0.134335524	1.195015982
Mycolicibacterium_pulveris	NA	NA	NA	NA	NA	NA	0	0	0	-0.986521564	-0.449574959	-0.848577585
Pseudomonas_sp._R4-39-08	NA	NA	NA	NA	NA	NA	0	0	0	-0.323139177	0.73477991	-9.632995197
Bacillus_sp._24	NA	NA	NA	NA	NA	NA	0	0	0	0	0	0
Bathymodiolus_thermophilus_thioautotrophic_gill_symbiont	NA	NA	NA	NA	NA	NA	0	0	0	0	0	0
Candidatus_Methylopumilus_turicensis	NA	NA	NA	NA	NA	NA	0	0	0	0	0	0
Citrobacter_sp._RHBSTW-00887	NA	NA	NA	NA	NA	NA	0	0	0	0	0	0
Corynebacterium_humireducens	NA	NA	NA	NA	NA	NA	0	0	0	0	0	0
Francisella_salina	NA	NA	NA	NA	NA	NA	0	0	0	0	0	0
Halomonas_sp._AM6	NA	NA	NA	NA	NA	NA	0	0	0	0	0	0
Leclercia_sp._W6	NA	NA	NA	NA	NA	NA	0	0	0	0	0	0
Microbacterium_sp._str._'China'	NA	NA	NA	NA	NA	NA	0	0	0	0	0	0
Pseudomonas_sp._IzPS23	NA	NA	NA	NA	NA	NA	0	0	0	0	0	0
Pseudomonas_sp._S35	NA	NA	NA	NA	NA	NA	0	0	0	0	0	0
Serratia_sp._FGI94	NA	NA	NA	NA	NA	NA	0	0	0	0	0	0
Streptomyces_aureoverticillatus	NA	NA	NA	NA	NA	NA	0	0	0	0	0	0
Streptomyces_leeuwenhoekii	NA	NA	NA	NA	NA	NA	0	0	0	0	0	0
Streptomyces_sp._QMT-12	NA	NA	NA	NA	NA	NA	0	0	0	0	0	0
Xanthomonas_sp._SI	NA	NA	NA	NA	NA	NA	0	0	0	0	0	0
Yersinia_intermedia	NA	NA	NA	NA	NA	NA	0	0	0	0	0	0
Yersinia_similis	NA	NA	NA	NA	NA	NA	0	0	0	0	0	7.63420844
Rhizobium_acidisoli	NA	NA	NA	NA	NA	NA	0	0	0	0	0	8.952254929
Hafnia_sp._CBA7124	NA	NA	NA	NA	NA	NA	0	0	0	0	0	9.306820443
Streptomyces_nodosus	NA	NA	NA	NA	NA	NA	0	0	0	0	0	9.625708843
Corynebacterium_renale	NA	NA	NA	NA	NA	NA	0	0	0	0	6.470320477	0
Plantactinospora_sp._BC1	NA	NA	NA	NA	NA	NA	0	0	0	0	6.815921204	0
Bradyrhizobium_guangxiense	NA	NA	NA	NA	NA	NA	0	0	0	0	7.222389201	0
Pseudomonas_sp._K2W31S-8	NA	NA	NA	NA	NA	NA	0	0	0	0	7.222389201	0
Psychrobacter_sp._P11G3	NA	NA	NA	NA	NA	NA	0	0	0	0	7.59792591	0
Sphingobium_baderi	NA	NA	NA	NA	NA	NA	0	0	0	0	7.902875524	0
Rathayibacter_sp._VKM_Ac-2801	NA	NA	NA	NA	NA	NA	0	0	0	0	8.912889336	8.412218392
Blochmannia_endosymbiont_of_Camponotus_(Colobopsis)_obliquus	NA	NA	NA	NA	NA	NA	0	0	0	0	10.52916698	0
Mycolicibacterium_arabiense	NA	NA	NA	NA	NA	NA	0	0	0	0	10.66711154	0
Mesorhizobium_sp._M7D.F.Ca.US.005.01.1.1	NA	NA	NA	NA	NA	NA	0	0	0	0.872125177	0.420179198	2.906890596
Pseudomonas_orientalis	NA	NA	NA	NA	NA	NA	0	0	0	2.876095202	2.575216119	0.379885594
Sphingobacterium_sp._G1-14	NA	NA	NA	NA	NA	NA	0	0	0	7.129283017	5.472487771	0
Rathayibacter_tritici	NA	NA	NA	NA	NA	NA	0	0	0	7.129283017	9.33687918	0
Pseudomonas_sp._FDAARGOS_380	NA	NA	NA	NA	NA	NA	0	0	0	7.303780748	7.369230901	0
Burkholderia_sp._YI23	NA	NA	NA	NA	NA	NA	0	0	0	7.333897756	0	8.520944699
Cellulophaga_baltica	NA	NA	NA	NA	NA	NA	0	0	0	7.420659242	8.902878538	11.50581155
Streptomyces_sp._Go-475	NA	NA	NA	NA	NA	NA	0	0	0	8.27301694	0	0
Streptococcus_ruminantium	NA	NA	NA	NA	NA	NA	0	0	0	8.45395786	0	6.544320001
Oenococcus_sp._UCMA_16435	NA	NA	NA	NA	NA	NA	0	0	0	8.45395786	7.222389201	0
Pseudomonas_sp._R32	NA	NA	NA	NA	NA	NA	0	0	0	9.254242075	0	0
Bacillus_haynesii	NA	NA	NA	NA	NA	NA	0	0	0	9.320423752	8.225607527	8.952254929
Rickettsia_africae	NA	NA	NA	NA	NA	NA	0	0	0	9.451211112	0	0
Enterobacteriaceae_endosymbiont_of_Donacia_vulgaris	NA	NA	NA	NA	NA	NA	0	0	0	9.797661526	11.10939321	8.986789195
Psychromonas_ingrahamii	NA	NA	NA	NA	NA	NA	0	0	0	9.838152884	0	8.17326238
Pseudomonas_sp._MPC6	NA	NA	NA	NA	NA	NA	0	0	0	10.04074178	5.472487771	0
Streptomyces_rectiverticillatus	NA	NA	NA	NA	NA	NA	0	0	6.15711649	-7.741466986	-2.268979215	-7.741466986
Streptomyces_vinaceus	NA	NA	NA	NA	NA	NA	0	0	6.15711649	-7.709655951	-7.709655951	-7.709655951
Cardinium_endosymbiont_of_Encarsia_pergandiella	NA	NA	NA	NA	NA	NA	0	0	6.15711649	-1.803424855	-8.932707872	-8.932707872
Burkholderia_sp._JP2-270	NA	NA	NA	NA	NA	NA	0	0	6.15711649	-1.757428681	0.431605144	0.269793631
Aminobacter_sp._SR38	NA	NA	NA	NA	NA	NA	0	0	6.15711649	0	0	0
Blochmannia_endosymbiont_of_Polyrhachis_(Hedomyrma)_turneri	NA	NA	NA	NA	NA	NA	0	0	6.15711649	0	0	0
Mycobacterium_stomatepiae	NA	NA	NA	NA	NA	NA	0	0	6.15711649	0	0	0
Pseudomonas_pohangensis	NA	NA	NA	NA	NA	NA	0	0	6.15711649	0	0	0
Pseudomonas_sp._KUIN-1	NA	NA	NA	NA	NA	NA	0	0	6.15711649	0	0	0
Rhizobium_sp._Khangiran2	NA	NA	NA	NA	NA	NA	0	0	6.15711649	0	0	9.306820443
Sphingobium_indicum	NA	NA	NA	NA	NA	NA	0	0	6.15711649	0	6.815921204	0
Candidatus_Phytoplasma_ziziphi	NA	NA	NA	NA	NA	NA	0	0	6.15711649	0.734825613	-8.076815597	-8.076815597
Mycolicibacterium_aichiense	NA	NA	NA	NA	NA	NA	0	0	6.15711649	1.755935354	1.12685628	-1.54314284
Sphingobium_hydrophobicum	NA	NA	NA	NA	NA	NA	0	0	6.15711649	8.27301694	8.807354922	7.63420844
Cellvibrio_sp._KY-YJ-3	NA	NA	NA	NA	NA	NA	0	0	6.15711649	9.333899991	0	9.403721476
Synechococcus_sp._CC9605	NA	NA	NA	NA	NA	NA	0	0	7.157114468	0	0	0
Mycobacterium_gallinarum	NA	NA	NA	NA	NA	NA	0	0	7.157114468	0	7.29523629	7.63420844
Calothrix_sp._NIES-2100	NA	NA	NA	NA	NA	NA	0	0	7.157114468	1.169528354	0.139120185	-1.532495596
Haemophilus_sp._oral_taxon_036	NA	NA	NA	NA	NA	NA	0	0	7.157114468	8.45395786	0	0
Bacillus_aryabhattai	NA	NA	NA	NA	NA	NA	0	0	7.745136469	0	0	0
Pseudomonas_sp._R11-23-07	NA	NA	NA	NA	NA	NA	0	0	8.157119522	-9.247927513	0.556203508	0.351985329
Sphingomonas_sp._MM-1	NA	NA	NA	NA	NA	NA	0	0	8.157119522	0	0	0
Streptomyces_sp._Mg1	NA	NA	NA	NA	NA	NA	0	0	8.451211112	0	8.215935782	8.952254929
Pantoea_sp._MT58	NA	NA	NA	NA	NA	NA	0	0	8.451211112	7.333897756	0	0
Vibrio_panuliri	NA	NA	NA	NA	NA	NA	0	0	8.451211112	9.969631147	5.472487771	8.412218392
Brucella_ovis	NA	NA	NA	NA	NA	NA	0	0	8.48088308	1.025397745	0.923093054	0.259087221
Rothia_amarae	NA	NA	NA	NA	NA	NA	0	0	8.598897393	0	7.222389201	0
Streptomyces_sp._CNQ-509	NA	NA	NA	NA	NA	NA	0	0	8.71892325	0	0	7.63420844
Pseudomonas_sp._CFSAN084952	NA	NA	NA	NA	NA	NA	0	0	8.863904389	0	0	0
Sporosarcina_sp._resist	NA	NA	NA	NA	NA	NA	0	0	8.904155902	0	9.214319121	0
Pantoea_sp._SM3640	NA	NA	NA	NA	NA	NA	0	0	9.014782079	0	0	0
Pseudomonas_oleovorans	NA	NA	NA	NA	NA	NA	0	0	9.157116995	-9.247927513	-1.952691223	-9.247927513
Rhodococcus_imtechensis	NA	NA	NA	NA	NA	NA	0	0	9.561202011	0	0	0
Spiroplasma_melliferum	NA	NA	NA	NA	NA	NA	0	0	9.561202011	0	0	0
Synechococcus_sp._PCC_7117	NA	NA	NA	NA	NA	NA	0	0	9.561202011	0	0	0
Mycobacteroides_saopaulense	NA	NA	NA	NA	NA	NA	0	0	9.591268006	1.709744125	-0.519077785	-7.741466986
Pseudomonas_sp._Leaf58	NA	NA	NA	NA	NA	NA	0	0	9.845490051	0	0	7.539158811
Microbacterium_aurum	NA	NA	NA	NA	NA	NA	0	0	10.03116358	-8.186525318	-8.186525318	-8.186525318
Mycobacterium_cookii	NA	NA	NA	NA	NA	NA	0	0	10.11041806	-7.807354922	-7.807354922	-7.807354922
Plantibacter_sp._PA-3-X8	NA	NA	NA	NA	NA	NA	0	0	10.14210706	0.419627066	1.755230778	-7.709655951
Pseudomonas_sp._TKP	NA	NA	NA	NA	NA	NA	0	0	10.45027306	0	0	0
Pseudothermotoga_thermarum	NA	NA	NA	NA	NA	NA	0	0	10.79876339	0	0	0
Streptomyces_malaysiensis	NA	NA	NA	NA	NA	NA	0	0	11.2903357	7.894817763	0	0
Pseudomonas_sp._R84	NA	NA	NA	NA	NA	NA	0	6.682671608	0	-10.74539702	-10.74539702	-10.74539702
Psychrobacter_sp._PRwf-1	NA	NA	NA	NA	NA	NA	0	6.682671608	6.15711649	-8.087462841	-8.087462841	-8.087462841
Legionella_adelaidensis	NA	NA	NA	NA	NA	NA	0	6.682671608	6.15711649	0	9.214319121	0
Wigglesworthia_glossinidia	NA	NA	NA	NA	NA	NA	0	6.682671608	9.691425987	-9.586214923	-0.249335743	-9.586214923
Mycobacteroides_salmoniphilum	NA	NA	NA	NA	NA	NA	0	6.682671608	9.819597937	0	0	8.952254929
Streptomyces_coeruleorubidus	NA	NA	NA	NA	NA	NA	0	7.68267863	7.157114468	7.420659242	7.902875524	0
Actinomyces_viscosus	NA	NA	NA	NA	NA	NA	0	7.68267863	8.863904389	9.410805357	6.470320477	9.423466822
Cupriavidus_neocaledonicus	NA	NA	NA	NA	NA	NA	0	8.110425888	7.745136469	0	5.472487771	0
Pseudomonas_sp._S09G_359	NA	NA	NA	NA	NA	NA	0	8.110425888	8.71892325	0	0	0
Pseudomonas_sediminis	NA	NA	NA	NA	NA	NA	0	8.110425888	10.9720033	-0.56828376	-1.788838054	1.092060553
Brevibacillus_sp._7WMA2	NA	NA	NA	NA	NA	NA	0	8.382131157	6.15711649	0	0	0
Candidatus_Nardonella_dryophthoridicola	NA	NA	NA	NA	NA	NA	0	8.382131157	8.800605508	8.366322214	8.033423002	9.0750325
Rhodococcus_sp._008	NA	NA	NA	NA	NA	NA	0	8.382131157	9.791755477	0	0	7.63420844
Pseudomonas_sp._R5-89-07	NA	NA	NA	NA	NA	NA	0	8.559465045	0	-9.060695932	-9.060695932	-9.060695932
Chromobacterium_sp._Rain0013	NA	NA	NA	NA	NA	NA	0	8.559465045	0	0	9.33687918	0
Psychrobacter_sp._DAB_AL43B	NA	NA	NA	NA	NA	NA	0	8.627369725	0	1.114096762	2.337961435	-7.745956618

Planococcus_donghaensis	NA	NA	NA	NA	NA	NA	0	8.627369725	6.15711649	-7.709655951	0.84390807	0.811288748
Acinetobacter_larvae	NA	NA	NA	NA	NA	NA	0	9.380166652	11.84084872	9.360480604	7.222389201	9.306820443
Spiroplasma_turonicum	NA	NA	NA	NA	NA	NA	0	9.384097309	0	-8.044394119	-0.44646821	1.480474687
Streptomyces_fagopyri	NA	NA	NA	NA	NA	NA	0	9.384097309	0	0	0	0
Streptomyces_finlayi	NA	NA	NA	NA	NA	NA	0	9.426646347	8.624046047	-9.201307944	-9.201307944	-1.567099503
Bacillus_pacificus	NA	NA	NA	NA	NA	NA	0	9.426646347	8.624046047	-7.807354922	-2.334867151	-1.263034921
Vibrio_diabolicus	NA	NA	NA	NA	NA	NA	0	9.509955083	7.157114468	-7.709655951	-7.709655951	-7.709655951
Streptomyces_sp._INR7	NA	NA	NA	NA	NA	NA	0	9.766378171	0	0	0	0
Pseudoalteromonas_issachenkonii	NA	NA	NA	NA	NA	NA	0	9.822499133	0	0	9.214319121	9.730186495
Sphingopyxis_sp._EG6	NA	NA	NA	NA	NA	NA	0	9.965784285	9.324999567	-1.394022699	0.581935566	0.220446777
Borreliella_afzelii	NA	NA	NA	NA	NA	NA	0	10.05479422	9.311646154	-8.723377667	0.316638012	-8.723377667
Bartonella_bovis	NA	NA	NA	NA	NA	NA	0	10.05724718	10.8166957	0	0	0
Mycobacterium_ulcerans	NA	NA	NA	NA	NA	NA	0	10.05724718	11.09251931	0	0	0
Hydrogenophaga_sp._RAC07	NA	NA	NA	NA	NA	NA	0	10.17877688	9.894541928	0	0	0
Providencia_stuartii	NA	NA	NA	NA	NA	NA	0	10.19004687	7.745136469	3.004589567	3.080423696	-0.17049714
Kitasatospora_aureofaciens	NA	NA	NA	NA	NA	NA	0	10.35434857	0	0	0	0
Leuconostoc_carnosum	NA	NA	NA	NA	NA	NA	0	10.46334045	0	0	5.472487771	0
Thermomonas_carbonis	NA	NA	NA	NA	NA	NA	0	10.48728736	0	-0.472881574	-0.936328264	1.990518947
Candidatus_Thioglobus_autotrophicus	NA	NA	NA	NA	NA	NA	0	10.65202931	6.15711649	0.594887354	0.409691935	0.891441129
Idiomarina_sp._X4	NA	NA	NA	NA	NA	NA	0	10.96381366	9.770888497	0	0	7.539158811
Micromonospora_coxensis	NA	NA	NA	NA	NA	NA	0	11.94929782	10.6290287	0	0	8.539158811
Corynebacterium_deserti	NA	NA	NA	NA	NA	NA	0	12.14500549	10.8876395	-7.741466986	-1.271146509	-7.741466986
Halomonas_sp._GT	NA	NA	NA	NA	NA	NA	0.00177331	0.22261648	0.413428859	0	0	9.631782751
Helicobacter_felis	NA	NA	NA	NA	NA	NA	0.003475068	0.075431774	0.372877353	0.359899861	-1.06962745	0.830551489
Psychrobacter_sp._P11G5	NA	NA	NA	NA	NA	NA	0.004796216	0.289510457	0.317122238	0	0	0
Sulfurimonas_sp._B2	NA	NA	NA	NA	NA	NA	0.007142914	-1.76825487	0.237622957	0.696986878	2.207269329	2.336192424
Streptococcus_sp._CNU_G2	NA	NA	NA	NA	NA	NA	0.007167689	-0.02077786	0.371115813	0	0	0
Thermotoga_sp._Cell2	NA	NA	NA	NA	NA	NA	0.012782428	-0.350135188	0.563992257	7.303780748	0	6.544320001
Halomonas_sp._JS92-SW72	NA	NA	NA	NA	NA	NA	0.013187709	-0.208709428	-0.637665033	-0.38669568	0.135159583	-7.807354922
Citrobacter_sp._RHBSTW-00986	NA	NA	NA	NA	NA	NA	0.016410183	0.513416259	0.567061517	0	0	8.520944699
Catenovulum_sp._CCB-QB4	NA	NA	NA	NA	NA	NA	0.01737499	0.215742378	1.465823526	8.81164121	10.59469783	10.49718321
Ancylobacter_sp._TS-1	NA	NA	NA	NA	NA	NA	0.019865689	-0.25351719	-0.427947746	1.073449322	-0.941930351	-0.175088078
Erythrobacter_gangjinensis	NA	NA	NA	NA	NA	NA	0.02293378	0.241012447	-0.009014924	9.982038114	0	6.544320001
Rhodococcus_sp._B7740	NA	NA	NA	NA	NA	NA	0.02803912	-0.286126259	-0.489723007	0	0	0
Citrobacter_sp._CFNIH10	NA	NA	NA	NA	NA	NA	0.034148556	1.655215073	1.183875206	1.124782655	2.142195065	2.804144293
Rhodococcus_sp._BH4	NA	NA	NA	NA	NA	NA	0.043395142	0.554284518	0.721525825	0	0	0
Cycloclasticus_sp._PY97N	NA	NA	NA	NA	NA	NA	0.045184772	-0.278881685	0.458901716	0	0	0
Bordetella_pertussis	NA	NA	NA	NA	NA	NA	0.046314099	1.199183802	1.612072475	0	7.222389201	8.520944699
Microbacterium_sp._10M-3C3	NA	NA	NA	NA	NA	NA	0.047543002	0.076738912	-0.247927017	0	0	0
Gordonia_polyisoprenivorans	NA	NA	NA	NA	NA	NA	0.050258602	-0.20920954	0.192035856	0	0	0
Mycoplasma_salivarium	NA	NA	NA	NA	NA	NA	0.055403061	-0.892810809	-0.679440539	9.628141111	8.803055861	0
Amycolatopsis_japonica	NA	NA	NA	NA	NA	NA	0.066682082	0.356270927	0.559090461	0	0	0
Leuconostoc_sp._C2	NA	NA	NA	NA	NA	NA	0.070938799	0.138115354	0.76028635	10.0018726	0	0
Pseudomonas_sp._B10	NA	NA	NA	NA	NA	NA	0.077442006	-0.06114748	0.751911151	0	0	0
Burkholderia_ubonensis	NA	NA	NA	NA	NA	NA	0.08017642	0.171436044	-0.300529606	-9.194756854	-0.967222734	-9.194756854
Streptomyces_sp._CdTB01	NA	NA	NA	NA	NA	NA	0.081355788	0.682192868	1.867207656	-0.6668036	-8.087462841	0.809375099
Aeromonas_sp._WP2-W18-CRE-05	NA	NA	NA	NA	NA	NA	0.08222241	0.450432827	1.115156042	10.99482719	8.225607527	9.306820443
Altererythrobacter_namhicola	NA	NA	NA	NA	NA	NA	0.088951389	-0.227871058	-0.826562354	0	0	0
Aeromonas_sp._CU5	NA	NA	NA	NA	NA	NA	0.092459947	-1.10470527	0.29661075	0	0	0
Pseudomonas_trivialis	NA	NA	NA	NA	NA	NA	0.093815276	0.217646998	0.22345783	-7.890768904	-0.668379703	-0.351610093
Obesumbacterium_proteus	NA	NA	NA	NA	NA	NA	0.103520821	-0.47679604	0.884179993	-0.556871148	-7.890768904	-7.890768904
Burkholderia_sp._MSMB0266	NA	NA	NA	NA	NA	NA	0.11011082	0.224743226	0.28294146	0	0	0
Paraburkholderia_terrae	NA	NA	NA	NA	NA	NA	0.115460604	-0.099333079	1.055846613	-1.306490181	-9.201307944	0.754825687
Swingsia_samuiensis	NA	NA	NA	NA	NA	NA	0.116198655	0.856379457	0.681705195	-0.456933667	-8.910891527	0.395928916
Agromyces_protaetiae	NA	NA	NA	NA	NA	NA	0.125260867	1.044087927	1.532391397	0	0	0
Shewanella_sp._MR-4	NA	NA	NA	NA	NA	NA	0.127688067	-0.304387561	0.70432176	9.020516091	7.29523629	0
Salinibacterium_sp._dk2585	NA	NA	NA	NA	NA	NA	0.129452698	-0.333638667	0.249396458	0	0	0
Pseudoalteromonas_piscicida	NA	NA	NA	NA	NA	NA	0.133093185	-0.559055988	0.03381093	1.875462864	-0.331984052	1.486987341
Ferrimonas_sp._S7	NA	NA	NA	NA	NA	NA	0.133801102	0.37668762	0.589090212	0	0	6.544320001
Dietzia_sp._oral_taxon_368	NA	NA	NA	NA	NA	NA	0.142325685	-0.236748112	0.342112335	7.333897756	0	0
Pseudomonas_umsongensis	NA	NA	NA	NA	NA	NA	0.14426422	-0.686765704	0.342318495	0	0	0
Kangiella_profundi	NA	NA	NA	NA	NA	NA	0.15960913	0.298238171	-0.484124627	8.45395786	0	7.63420844
Acidovorax_ebreus	NA	NA	NA	NA	NA	NA	0.163834116	1.08863207	0.579980963	0	0	0
Lactobacillus_parabuchneri	NA	NA	NA	NA	NA	NA	0.166012571	0.438378316	0.238840576	0.465662018	-7.807354922	2.893084796
Dechlorosoma_suillum	NA	NA	NA	NA	NA	NA	0.169383444	1.34983075	-1.919699107	0	0	0
Synechococcus_sp._KORDI-49	NA	NA	NA	NA	NA	NA	0.170139761	-0.621937804	0.655688615	7.303780748	8.225607527	0
Agromyces_sp._HY052	NA	NA	NA	NA	NA	NA	0.174928886	1.125703692	-1.333834572	7.129283017	7.369230901	8.996239023
Colwellia_sp._Arc7-D	NA	NA	NA	NA	NA	NA	0.176416152	0.184803895	0.308755181	0	0	0
Mycoplasma_flocculare	NA	NA	NA	NA	NA	NA	0.177288847	0.748120548	1.47066879	0	0	0
Bacillus_sp._BS98	NA	NA	NA	NA	NA	NA	0.190713303	-0.490075416	-0.19596153	0	0	0
Vibrio_cincinnatiensis	NA	NA	NA	NA	NA	NA	0.192336582	1.614206143	0.879871585	-0.316232577	0.972171477	0.60328137
Prochlorococcus_sp._MIT_0801	NA	NA	NA	NA	NA	NA	0.204253983	-0.358345736	-0.273468659	0	9.214319121	0
Rhodococcus_sp._M8	NA	NA	NA	NA	NA	NA	0.214505309	0.424365516	1.790261259	0	0	0
Borrelia_mayonii	NA	NA	NA	NA	NA	NA	0.216240565	1.128405095	-0.686691867	0.203752993	-0.74017114	-3.908950633
Shewanella_sediminis	NA	NA	NA	NA	NA	NA	0.222113026	1.134088552	-0.090451201	9.296152225	9.447909749	7.544323092
Aminobacter_aminovorans	NA	NA	NA	NA	NA	NA	0.23340461	0.796475857	0.722713582	0	8.553564021	0
Shewanella_sp._FDAARGOS_354	NA	NA	NA	NA	NA	NA	0.233993964	-0.281207019	-0.904102079	3.27520977	0.138144686	0.908776182
Stenotrophomonas_sp._MYb57	NA	NA	NA	NA	NA	NA	0.237055116	1.149934086	-0.261037009	0	0	0
Hydrogenophaga_pseudoflava	NA	NA	NA	NA	NA	NA	0.254144817	0.500392961	-0.627091333	0	8.225607527	0
Gluconobacter_thailandicus	NA	NA	NA	NA	NA	NA	0.254431453	-0.517958094	-0.061970347	0	0	0
Caulobacter_segnis	NA	NA	NA	NA	NA	NA	0.260365975	-0.334650413	-0.078076338	8.908893949	0	7.539158811
Chlamydia_avium	NA	NA	NA	NA	NA	NA	0.268580065	1.578365058	2.230738717	0	7.29523629	0
Candidatus_Pseudomonas_adelgestsugas	NA	NA	NA	NA	NA	NA	0.273680737	0.632116016	-0.693823111	-1.255057841	-9.288480843	-0.131975514
Bartonella_ancashensis	NA	NA	NA	NA	NA	NA	0.282211371	0.651298413	0.537492695	-8.076815597	-8.076815597	-8.076815597
Borrelia_crocidurae	NA	NA	NA	NA	NA	NA	0.287595242	0.697606879	-0.523374368	0	0	0
Flavobacterium_indicum	NA	NA	NA	NA	NA	NA	0.295424137	0.660556625	1.063124686	7.333897756	0	0
Erythrobacter_sp._THAF29	NA	NA	NA	NA	NA	NA	0.297238068	1.4450792	1.643242861	0	0	0
Burkholderia_sp._KBS0801	NA	NA	NA	NA	NA	NA	0.304075032	0.64129628	1.120792553	0	0	0
Escherichia_fergusonii	NA	NA	NA	NA	NA	NA	0.306640757	0.464331194	3.226902988	8.86005338	7.222389201	0
Exiguobacterium_sp._ZWU0009	NA	NA	NA	NA	NA	NA	0.311401829	-0.044173383	0.431141301	0	0	0
Pseudomonas_sp._FGI182	NA	NA	NA	NA	NA	NA	0.312214759	0.708098129	1.025725919	0	0	0
Novosphingobium_sp._PP1Y	NA	NA	NA	NA	NA	NA	0.31410077	0.159406942	1.297315848	0	0	0
Glutamicibacter_nicotianae	NA	NA	NA	NA	NA	NA	0.316821299	-0.434746359	0.167006964	7.129283017	0	0
Cobetia_marina	NA	NA	NA	NA	NA	NA	0.318077212	0.468151321	-0.934199256	0	5.472487771	0
Xenorhabdus_hominickii	NA	NA	NA	NA	NA	NA	0.324404743	2.251837145	0.54296919	0.915028644	-0.431254045	-0.448624507
Pseudomonas_sp._B11D7D	NA	NA	NA	NA	NA	NA	0.346913551	-1.018937599	-1.133937793	8.27301694	0	8.634204809
Rhodococcus_aetherivorans	NA	NA	NA	NA	NA	NA	0.350260467	0.139791213	-0.356839596	0	0	6.930737338
Neorickettsia_sennetsu	NA	NA	NA	NA	NA	NA	0.351227835	1.212594046	0.894254875	0	0	0
Porphyrobacter_sp._LM_6	NA	NA	NA	NA	NA	NA	0.360053625	0.495227333	0.49210916	0.966738072	-2.400044242	2.314510623
Diaphorobacter_polyhydroxybutyrativorans	NA	NA	NA	NA	NA	NA	0.360877208	1.005675927	0.520938825	0	0	6.544320001
Pseudomonas_sp._31-12	NA	NA	NA	NA	NA	NA	0.369093022	-0.123490614	0.457884389	0	0	0
Pantoea_eucalypti	NA	NA	NA	NA	NA	NA	0.371647852	0.736560444	-0.327939387	0	0	0
Aurantimicrobium_sp._MWH-Uga1	NA	NA	NA	NA	NA	NA	0.380004923	0.033607681	1.672903018	8.442943496	0	0
Nostoc_sphaeroides	NA	NA	NA	NA	NA	NA	0.388460634	0.460854518	0.139742734	0	8.809501266	8.996239023
Dolichospermum_flos-aquae	NA	NA	NA	NA	NA	NA	0.389447937	0.403398148	1.02718619	-9.324929833	-1.099322306	-9.324929833
Cobetia_pacifica	NA	NA	NA	NA	NA	NA	0.390061192	1.284472956	1.718030425	7.333897756	0	6.544320001
Sulfurimonas_sp._GYSZ_1	NA	NA	NA	NA	NA	NA	0.395845777	0.246491441	0.602681444	9.448461189	6.470320477	0
Mycobacterium_kansasii	NA	NA	NA	NA	NA	NA	0.398757213	0.147050145	0.241954375	0	0	0
Shewanella_woodyi	NA	NA	NA	NA	NA	NA	0.40896193	1.620880636	-7.757316664	-0.339033564	-9.247927513	-9.247927513
Delftia_sp._HK171	NA	NA	NA	NA	NA	NA	0.411426604	-8.091313724	-8.091313724	-7.709655951	1.94139574	-0.075447511
Bacillus_sp._MD-5	NA	NA	NA	NA	NA	NA	0.414076023	0.242078136	1.138594374	0	8.809501266	8.634204809

Bacillus_sp._Cs-700	NA	NA	NA	NA	NA	NA	0.418004292	-2.689599278	2.484578673	-8.977279923	-2.16135872	-8.977279923
Liberibacter_crescens	NA	NA	NA	NA	NA	NA	0.425552729	-0.096445708	0.059002282	-8.044394119	-0.822004918	-8.044394119
Acinetobacter_chinensis	NA	NA	NA	NA	NA	NA	0.425924731	1.34983075	2.423667804	0	0	0
Clavibacter_sp._DM1	NA	NA	NA	NA	NA	NA	0.4288155	-0.401673138	-0.076047465	0	0	0
Idiomarina_sp._OT37-5b	NA	NA	NA	NA	NA	NA	0.438809361	1.745568902	2.450543785	0	0	0
Pseudomonas_mandelii	NA	NA	NA	NA	NA	NA	0.441740293	0.481926504	0.764303965	0	7.222389201	0
Burkholderia_sp._RPE67	NA	NA	NA	NA	NA	NA	0.445507684	1.45227618	0.702684813	0	0	0
Citrobacter_rodentium	NA	NA	NA	NA	NA	NA	0.454174492	0.116453983	-0.305810419	0.969284476	-0.668379703	-7.890768904
Streptomyces_sp._Tu6071	NA	NA	NA	NA	NA	NA	0.455370987	1.332797311	2.139871371	-8.924812504	-0.708876722	-8.924812504
Mycoplasma_cloacale	NA	NA	NA	NA	NA	NA	0.455575446	-0.986600761	0.259019104	0.017209281	-0.321929718	2.358958104
Leifsonia_shinshuensis	NA	NA	NA	NA	NA	NA	0.458439195	-0.344166003	0.889604937	-7.745956618	-7.745956618	1.560863826
Mycobacterium_paraintracellulare	NA	NA	NA	NA	NA	NA	0.460593287	-1.055305394	2.145243119	0	0	0
Enterobacteriaceae_endosymbiont_of_Donacia_clavipes	NA	NA	NA	NA	NA	NA	0.462690264	0.43399398	0.20676376	0	7.369230901	0
Enterobacteriaceae_endosymbiont_of_Macroplea_mutica	NA	NA	NA	NA	NA	NA	0.469344151	-0.085181743	-0.199369892	0	0	0
Synechococcus_sp._SYN20	NA	NA	NA	NA	NA	NA	0.471291115	0.096727007	-0.004772266	0	0	10.21270308
Salmonella_sp._SJTUF14178	NA	NA	NA	NA	NA	NA	0.473992418	1.03495016	1.096130738	0	0	0
Wolbachia_pipientis	NA	NA	NA	NA	NA	NA	0.477417675	-8.552496194	0.602321915	8.45395786	0	0
Bradyrhizobium_diazoefficiens	NA	NA	NA	NA	NA	NA	0.479337963	0.465734217	0.408878093	8.81164121	0	0
Sphingomonas_lutea	NA	NA	NA	NA	NA	NA	0.480866426	1.032350648	0.709212677	0	0	6.930737338
Pseudoalteromonas_sp._13-15	NA	NA	NA	NA	NA	NA	0.486183432	0.105260975	-0.310109202	-9.14125651	-9.14125651	-9.14125651
Bradyrhizobium_ottawaense	NA	NA	NA	NA	NA	NA	0.489433068	-0.516544589	-0.767908395	0	0	0
Microbacterium_sp._No._7	NA	NA	NA	NA	NA	NA	0.491107398	1.00851249	0.258639498	0.238160717	-8.700439718	2.07379552
Arsenophonus_endosymbiont_of_Aleurodicus_dispersus	NA	NA	NA	NA	NA	NA	0.499223741	-0.054386847	0.220521966	0	7.222389201	0
Bradyrhizobium_sp._1(2017)	NA	NA	NA	NA	NA	NA	0.499245806	-0.164993723	-0.249973993	0	0	0
Kushneria_sp._YCWA18	NA	NA	NA	NA	NA	NA	0.502305139	-2.016486465	-1.486739194	8.033423002	10.59681404	6.930737338
Spiroplasma_tabanidicola	NA	NA	NA	NA	NA	NA	0.502959731	-0.257618454	0.789970995	0	0	9.423466822
Fervidobacterium_nodosum	NA	NA	NA	NA	NA	NA	0.504072976	0.486181351	0.576102951	0.14873522	1.334923156	-8.202941691
Agrobacterium_fabrum	NA	NA	NA	NA	NA	NA	0.506917878	0.563438568	0.468747183	7.129283017	0	0
Bradyrhizobium_sp._323S2	NA	NA	NA	NA	NA	NA	0.511579908	0.180179647	0.099022542	0	0	6.544320001
Streptomyces_atratus	NA	NA	NA	NA	NA	NA	0.513232733	0.614473844	0.368216399	0	7.996236198	0
Erythrobacter_atlanticus	NA	NA	NA	NA	NA	NA	0.514637854	0.323362891	0.525123685	0	0	0
Arthrobacter_sp._ZXY-2	NA	NA	NA	NA	NA	NA	0.52219321	1.779278057	2.328883639	0	0	0
Candidatus_Pelagibacter_giovannonii	NA	NA	NA	NA	NA	NA	0.525711404	0.514208145	1.597733927	-8.186525318	-8.186525318	-8.186525318
Sphingomonas_sp._YZ-8	NA	NA	NA	NA	NA	NA	0.527148203	0.033610291	3.48611843	0	5.472487771	0
Legionella_fallonii	NA	NA	NA	NA	NA	NA	0.540134301	0.263464742	0.009507351	-0.111430576	0.425950418	-0.677700737
Arthrobacter_sp._AQ5-05	NA	NA	NA	NA	NA	NA	0.540260149	0.995788629	1.067869419	0	0	0
Mycoplasma_gallopavonis	NA	NA	NA	NA	NA	NA	0.551369749	-1.500740626	1.658829002	-7.890768904	-2.418281133	-7.890768904
Klebsiella_sp._WP3-S18-ESBL-05	NA	NA	NA	NA	NA	NA	0.562557641	-0.911956829	0.591330933	0	0	0
Actinobacillus_lignieresii	NA	NA	NA	NA	NA	NA	0.567393582	2.403420758	1.955661986	-7.741466986	-7.741466986	-7.741466986
Variovorax_sp._PBL-E5	NA	NA	NA	NA	NA	NA	0.569507968	1.166259301	-2.480167705	0	0	0
Alcanivorax_sp._NBRC_101098	NA	NA	NA	NA	NA	NA	0.572279644	0.706530002	0.605673704	0	0	0
Asaia_bogorensis	NA	NA	NA	NA	NA	NA	0.573870655	3.078701798	2.070346987	1.636465795	1.472852134	2.539303784
Pseudomonas_fragi	NA	NA	NA	NA	NA	NA	0.576786823	0.359848287	0.462285885	-8.202941691	1.971983992	-1.65862169
Streptomyces_albidoflavus	NA	NA	NA	NA	NA	NA	0.57904711	-0.488066029	-0.472300293	0	0	0
Streptococcus_sp._I-G2	NA	NA	NA	NA	NA	NA	0.593042068	0.410383414	0.430659587	0	0	0
Planococcus_maritimus	NA	NA	NA	NA	NA	NA	0.607583687	0.359856244	0.896214091	0	8.22753412	7.63420844
Thermoanaerobacter_mathranii	NA	NA	NA	NA	NA	NA	0.611924147	-9.760342386	1.319509025	0	0	7.539158811
Paraoerskovia_marina	NA	NA	NA	NA	NA	NA	0.613362799	1.204843071	2.101824138	9.410805357	8.834154981	10.42836017
Marinitoga_sp._1137	NA	NA	NA	NA	NA	NA	0.618051679	0.859693025	1.359278724	8.442943496	0	0
Serinicoccus_marinus	NA	NA	NA	NA	NA	NA	0.618345583	-0.001573136	2.290487976	0	0	0
Labrenzia_aggregata	NA	NA	NA	NA	NA	NA	0.62151105	-0.157182028	0.425966678	0	0	0
Mannheimia_sp._BNO311	NA	NA	NA	NA	NA	NA	0.631481422	1.12912635	0.776534841	0	0	9.423466822
Pseudoalteromonas_marina	NA	NA	NA	NA	NA	NA	0.633599951	0.540038426	0.218001529	0	0	0
Tomitella_sp._HY172	NA	NA	NA	NA	NA	NA	0.641203557	0.19477955	-0.673661661	-7.807354922	0.969627447	-7.807354922
Bacillus_sp._SYJ	NA	NA	NA	NA	NA	NA	0.645853753	0.613028124	0.63321395	0	6.815921204	9.412216274
Streptococcus_sp._FDAARGOS_192	NA	NA	NA	NA	NA	NA	0.646565799	2.282908688	0.729608927	0	7.222389201	7.63420844
Synechococcus_sp._PCC_8807	NA	NA	NA	NA	NA	NA	0.64860935	1.965671464	2.274005968	0	8.372138089	7.380824669
Vibrio_sp._dhg	NA	NA	NA	NA	NA	NA	0.655143758	0.817520322	-0.318273374	0	5.472487771	6.930737338
Pseudomonas_lundensis	NA	NA	NA	NA	NA	NA	0.658307592	0.084560527	-0.475248657	-8.890771943	-0.081270678	0.10546708
Serratia_proteamaculans	NA	NA	NA	NA	NA	NA	0.663549625	1.87429363	1.831793444	0	0	0
Burkholderia_sp._MSMB0856	NA	NA	NA	NA	NA	NA	0.672113797	0.206451044	-0.233001546	0	0	10.07859116
Bartonella_tribocorum	NA	NA	NA	NA	NA	NA	0.690960039	0.869144812	2.082556468	0	0	0
Plantibacter_sp._M259	NA	NA	NA	NA	NA	NA	0.694750646	-0.024500866	-0.303945502	8.35167691	0	0
Acetobacter_ghanensis	NA	NA	NA	NA	NA	NA	0.700583191	-0.855347493	1.614597288	0	0	0
Acinetobacter_sp._LoGeW2-3	NA	NA	NA	NA	NA	NA	0.702594631	1.191002283	1.232617933	8.442943496	8.225607527	8.658211483
Synechococcus_sp._A15-127	NA	NA	NA	NA	NA	NA	0.70285789	1.843024372	1.434249146	7.129283017	0	0
Mycobacterium_seoulense	NA	NA	NA	NA	NA	NA	0.702970953	2.522126768	1.920878188	0.485992853	-8.924812504	-8.924812504
Myroides_odoratimimus	NA	NA	NA	NA	NA	NA	0.704618973	-0.163568939	-1.092524479	0	0	0
Streptomyces_alboflavus	NA	NA	NA	NA	NA	NA	0.71746884	-0.134057739	-0.181207246	-9.973453429	-9.973453429	-9.973453429
Micrococcus_sp._KBS0714	NA	NA	NA	NA	NA	NA	0.719479853	0.844573051	0.9888598	0	0	0
Mesorhizobium_australicum	NA	NA	NA	NA	NA	NA	0.727934014	0.054642819	1.393993339	0	0	0
Brevibacterium_sp._JNUCC-42	NA	NA	NA	NA	NA	NA	0.730925922	0.899214134	1.213616697	-0.089169336	-2.50098676	1.511040334
Wolbachia_endosymbiont_of_Dipetalonema_caudispina	NA	NA	NA	NA	NA	NA	0.736741622	1.231733537	0.952571727	0	0	0
Mycobacterium_shigaense	NA	NA	NA	NA	NA	NA	0.737635356	-0.410263803	1.574719064	0	0	0
Dickeya_fangzhongdai	NA	NA	NA	NA	NA	NA	0.744108893	0.927330649	0.237828319	7.129283017	0	10.94982671
Rhizobium_sp._NXC14	NA	NA	NA	NA	NA	NA	0.749746321	0.503502155	0.491999865	0	9.214319121	8.125842211
Pseudomonas_sp._DTU12.1	NA	NA	NA	NA	NA	NA	0.752667329	-0.881243689	0.263568382	0	0	0
Pseudomonas_sp._TCU-HL1	NA	NA	NA	NA	NA	NA	0.755786482	-1.394136967	0.74278234	0	0	0
Edwardsiella_sp._EA181011	NA	NA	NA	NA	NA	NA	0.757076341	1.04543454	2.678545964	-7.745956618	-7.745956618	-7.745956618
Cnuibacter_physcomitrellae	NA	NA	NA	NA	NA	NA	0.77052186	0.584527582	0.432436229	0	8.912889336	6.930737338
Streptomyces_qaidamensis	NA	NA	NA	NA	NA	NA	0.770844106	-8.10567284	2.28723485	-9.247927513	-9.247927513	-9.247927513
Rhizobium_sp._Y9	NA	NA	NA	NA	NA	NA	0.777451278	0.173075206	-1.825262274	0	0	0
[Haemophilus]_ducreyi	NA	NA	NA	NA	NA	NA	0.778581665	0.850188523	-0.195607569	-0.349786471	-9.723375961	1.379472415
Simkania_negevensis	NA	NA	NA	NA	NA	NA	0.77884382	0.14732031	1.768696132	7.894817763	0	0
Hafnia_alvei	NA	NA	NA	NA	NA	NA	0.789900784	1.442997542	-2.919699107	0	7.59792591	0
Pseudoalteromonas_sp._DL-6	NA	NA	NA	NA	NA	NA	0.792708847	2.286525248	1.208794688	0	0	0
Vibrio_alfacsensis	NA	NA	NA	NA	NA	NA	0.796599741	0.968777806	-1.251610256	0	9.33687918	9.306820443
Pseudomonas_sp._S1-A32-2	NA	NA	NA	NA	NA	NA	0.799826583	0.807290671	1.400021594	0	0	7.539158811
Streptomyces_luteoverticillatus	NA	NA	NA	NA	NA	NA	0.812529597	1.49646961	0.840461063	-8.202941691	-8.202941691	-8.202941691
Synechococcus_sp._WH_8020	NA	NA	NA	NA	NA	NA	0.827568257	0.749542536	0.311138515	0	7.222389201	0
Parasphingopyxis_algicola	NA	NA	NA	NA	NA	NA	0.838619125	0.304157324	-0.579663493	8.366322214	0	0
Acinetobacter_bereziniae	NA	NA	NA	NA	NA	NA	0.844233687	1.865664848	1.205474632	0	7.222389201	10.22560512
Mycoplasma_tullyi	NA	NA	NA	NA	NA	NA	0.868209897	2.052083246	0.882008779	0.159241194	-9.372140267	-1.832981455
Synechococcus_sp._A18-46.1	NA	NA	NA	NA	NA	NA	0.874403702	0.174842563	1.591022113	0	0	0
Polynucleobacter_wuianus	NA	NA	NA	NA	NA	NA	0.879066252	0.240546935	0.017122653	0	0	0
Thermus_sp._WG	NA	NA	NA	NA	NA	NA	0.899590687	-1.309778646	1.893816457	0	0	0
Mycolicibacterium_mageritense	NA	NA	NA	NA	NA	NA	0.901940305	2.097473072	1.628101002	0.356408513	-1.358426368	0.714437203
Streptomyces_fungicidicus	NA	NA	NA	NA	NA	NA	0.903149733	1.34965379	0.177937181	7.420659242	0	0
Chromobacterium_rhizoryzae	NA	NA	NA	NA	NA	NA	0.913547583	-8.799135887	-8.799135887	7.303780748	0	0
Aquitalea_denitrificans	NA	NA	NA	NA	NA	NA	0.918797485	1.629339357	1.294884428	0	9.214319121	0
Pseudomonas_sp._R2A2	NA	NA	NA	NA	NA	NA	0.924241029	-8.10567284	-1.94855635	0	0	0
Actinobacillus_delphinicola	NA	NA	NA	NA	NA	NA	0.925361029	2.002019706	0.762602066	0	0	0
Gordonia_phthalatica	NA	NA	NA	NA	NA	NA	0.931619749	0.083123747	-0.109951149	0	9.33687918	0
Lelliottia_jeotgali	NA	NA	NA	NA	NA	NA	0.938600145	-8.091313724	3.505258091	0	0	7.63420844
Pseudoalteromonas_piratica	NA	NA	NA	NA	NA	NA	0.946347884	-0.200271783	0.598705231	0	7.222389201	8.520944699
Ruegeria_sp._PR1b	NA	NA	NA	NA	NA	NA	0.953098272	-8.076815597	2.484386414	0	0	0
Microbacterium_sp._PAMC_28756	NA	NA	NA	NA	NA	NA	0.953098272	4.190290011	2.090602549	0	0	0
Methylophilus_medardicus	NA	NA	NA	NA	NA	NA	0.959352297	-0.169634825	-0.111629621	0	0	0
Candidatus_Liberibacter_americanus	NA	NA	NA	NA	NA	NA	0.975244849	-2.580703237	1.150834698	0	0	0
Streptomyces_coelicolor	NA	NA	NA	NA	NA	NA	0.98368153	0.946285699	-0.291119265	0	8.912889336	0
Acidithiobacillus_ferrooxidans	NA	NA	NA	NA	NA	NA	0.990880591	0.598523085	1.616047797	7.129283017	8.91607949	0

Streptomyces_sp._SYP-A7193	NA	NA	NA	NA	NA	NA	1.009904489	-1.181896441	0.244370467	0	0	0
Polynucleobacter_paneuropaeus	NA	NA	NA	NA	NA	NA	1.011482813	1.052022073	1.50872246	0	0	0
Kocuria_indica	NA	NA	NA	NA	NA	NA	1.029286812	-0.79020003	-0.658288992	0	0	7.539158811
Hydrogenobaculum_sp._HO	NA	NA	NA	NA	NA	NA	1.05413253	1.068765104	-0.222555633	0	0	0
Streptomyces_autolyticus	NA	NA	NA	NA	NA	NA	1.055164484	1.845016211	1.946636856	0	0	0
Borrelia_maritima	NA	NA	NA	NA	NA	NA	1.056780811	-0.427282111	0.347705199	0	0	0
Nocardia_asteroides	NA	NA	NA	NA	NA	NA	1.071789771	3.490934813	3.687586026	8.366322214	0	7.63420844
Legionella_lansingensis	NA	NA	NA	NA	NA	NA	1.076595033	-0.029636245	1.228858193	-7.709655951	-7.709655951	-7.709655951
Rhizobium_sp._Kim5	NA	NA	NA	NA	NA	NA	1.077169781	-1.067220377	1.095298383	8.990577692	0	0
Pseudomonas_luteola	NA	NA	NA	NA	NA	NA	1.096269419	0.827670457	1.99124133	0	0	0
Pseudomonas_sp._NIBRBAC000502773	NA	NA	NA	NA	NA	NA	1.106040215	0.678506525	-1.395381726	0	0	0
Paraburkholderia_atlantica	NA	NA	NA	NA	NA	NA	1.108959786	1.154175726	0.1898645	-7.890768904	-7.890768904	-7.890768904
Mycolicibacterium_celeriflavum	NA	NA	NA	NA	NA	NA	1.109659204	1.547462605	1.878529296	0	0	0
Kribbella_qitaiheensis	NA	NA	NA	NA	NA	NA	1.111943291	1.235430317	0.999536137	-8.087462841	-8.087462841	-0.453254401
Burkholderia_metallica	NA	NA	NA	NA	NA	NA	1.117410088	1.404417407	2.206913607	7.129283017	0	0
Mycolicibacterium_vaccae	NA	NA	NA	NA	NA	NA	1.133481936	-8.799135887	1.270381012	0	7.222389201	0
Apibacter_sp._B2966	NA	NA	NA	NA	NA	NA	1.135688866	1.336329989	1.070607279	9.632995197	5.472487771	0
Curtobacterium_sp._csp3	NA	NA	NA	NA	NA	NA	1.138893673	0.370273165	2.843706826	-7.700439718	1.51549849	-7.700439718
Rhodococcus_sp._AQ5-07	NA	NA	NA	NA	NA	NA	1.145474625	1.126160724	1.576857709	-7.741466986	-7.741466986	-7.741466986
Brucella_sp._2280	NA	NA	NA	NA	NA	NA	1.171131114	-1.97003192	1.034110936	0	0	0
Pseudomonas_granadensis	NA	NA	NA	NA	NA	NA	1.176431286	1.041111475	2.162699142	0	0	0
Streptococcus_koreensis	NA	NA	NA	NA	NA	NA	1.233641464	0.746813114	2.260009181	8.27301694	8.215935782	0
Gryllotalpicola_protaetiae	NA	NA	NA	NA	NA	NA	1.249723561	1.07951237	2.158406544	0.321928095	-8.044394119	1.262426324
Candidatus_Borrelia_tachyglossi	NA	NA	NA	NA	NA	NA	1.272597205	-7.757316664	0.984763674	-8.186525318	1.027793803	1.225690956
Enterobacteriaceae_endosymbiont_of_Donacia_cinerea	NA	NA	NA	NA	NA	NA	1.290976532	1.109120114	-1.053999418	0	0	6.544320001
Enterobacter_sp._RHBSTW-00593	NA	NA	NA	NA	NA	NA	1.295937196	1.588931848	0.810111293	0	0	0
Streptomyces_tendae	NA	NA	NA	NA	NA	NA	1.306759378	0.122380818	-0.856488075	0	9.981667168	0
Salipiger_pacificus	NA	NA	NA	NA	NA	NA	1.319656725	0.429684049	1.194130379	8.366322214	0	0
Pseudomonas_sp._MRSN12121	NA	NA	NA	NA	NA	NA	1.354235448	-1.464597073	2.057313425	0.635588574	-7.807354922	0.850856561
Spiroplasma_sp._BIUS-1	NA	NA	NA	NA	NA	NA	1.368282258	2.39483909	1.737548442	8.27301694	0	0
Mycoplasma_agalactiae	NA	NA	NA	NA	NA	NA	1.433046416	0.249000082	-0.872204635	7.333897756	5.472487771	10.52094273
Rhizobium_sp._NIBRBAC000502774	NA	NA	NA	NA	NA	NA	1.444402444	2.13934172	0.888617733	0	0	0
Lactococcus_petauri	NA	NA	NA	NA	NA	NA	1.448827423	0.840026805	-1.404423743	0	0	0
Octadecabacter_temperatus	NA	NA	NA	NA	NA	NA	1.464528668	1.36568991	1.89561618	0	0	0
Nonlabens_sediminis	NA	NA	NA	NA	NA	NA	1.464691098	1.846479808	4.004385982	0	0	0
Pseudoalteromonas_shioyasakiensis	NA	NA	NA	NA	NA	NA	1.470182712	0.626653954	0.818257589	-7.700439718	-7.700439718	-7.700439718
Bradyrhizobium_genosp._B	NA	NA	NA	NA	NA	NA	1.536391488	-0.31775935	0.564887708	0	0	0
Dokdonia_sp._4H-3-7-5	NA	NA	NA	NA	NA	NA	1.543092773	3.208547844	1.051444155	0.525558915	0.882801086	2.152465291
Borrelia_miyamotoi	NA	NA	NA	NA	NA	NA	1.559108993	1.096269419	0.987719043	0.620365597	0.47965091	-7.745956618
Borreliella_garinii	NA	NA	NA	NA	NA	NA	1.569174127	2.403208365	0.908851267	0	0	0
Verrucosispora_maris	NA	NA	NA	NA	NA	NA	1.589542302	0.90879571	2.322155327	0	7.996236198	0
Deinococcus_swuensis	NA	NA	NA	NA	NA	NA	1.625271625	0.93859198	-0.934196847	-0.534544594	-1.678477607	0.604283773
Alteromonas_sp._76-1	NA	NA	NA	NA	NA	NA	1.659851714	0.310581341	0.815615354	0	7.369230901	0
Francisella_hispaniensis	NA	NA	NA	NA	NA	NA	1.67570281	0.489190957	0.779554469	0.726203886	0.989639434	0.468666831
Leclercia_sp._LSNIH1	NA	NA	NA	NA	NA	NA	1.680460348	-0.779911656	2.414407895	0	0	0
Stenotrophomonas_sp._YAU14A_MKIMI4_1	NA	NA	NA	NA	NA	NA	1.720278465	1.199512515	1.678784523	0	0	0
Aeromonas_dhakensis	NA	NA	NA	NA	NA	NA	1.815576872	2.298661713	1.728284213	0	7.29523629	0
Sporosarcina_sp._P33	NA	NA	NA	NA	NA	NA	1.841898889	-2.805534076	2.663733656	0	0	0
Aster_yellows_witches'-broom_phytoplasma	NA	NA	NA	NA	NA	NA	1.918672595	3.016597541	3.621207884	0	10.36632221	0
Streptomyces_sp._CB09001	NA	NA	NA	NA	NA	NA	1.930337848	-1.394143989	0.723789911	0	0	0
Altererythrobacter_dongtanensis	NA	NA	NA	NA	NA	NA	2.005050538	1.291684862	1.582490944	1.089483018	-7.807354922	-7.807354922
Streptococcus_sp._NCTC_11567	NA	NA	NA	NA	NA	NA	2.040023218	0.119317169	0.436949887	0	0	0
Neisseria_sicca	NA	NA	NA	NA	NA	NA	2.088317518	2.703797985	-0.919701129	0	0	0
Microcella_alkaliphila	NA	NA	NA	NA	NA	NA	2.141352738	-1.074645056	2.749932899	0	0	0
Aliivibrio_wodanis	NA	NA	NA	NA	NA	NA	2.158001834	3.356960209	2.321441771	2.500909247	2.355682887	2.018975205
Leclercia_sp._LSNIH3	NA	NA	NA	NA	NA	NA	2.295845065	0.63642887	4.905718149	0	0	0
Kitasatospora_albolonga	NA	NA	NA	NA	NA	NA	2.325416728	-8.552496194	0.071549852	-0.473457166	-0.584965721	-7.807354922
Burkholderia_stagnalis	NA	NA	NA	NA	NA	NA	2.459433061	1.797706471	1.374809653	0.679794704	0.813906005	2.061833519
Vibrio_azureus	NA	NA	NA	NA	NA	NA	2.482329189	2.631027274	0.44683136	8.442943496	0	0
Arthrospira_platensis	NA	NA	NA	NA	NA	NA	2.514250071	3.223056411	3.310420567	-8.202941691	-8.202941691	-8.202941691
Nocardia_terpenica	NA	NA	NA	NA	NA	NA	2.769387784	3.201953263	4.685981665	10.33613831	9.353146825	0
Bradyrhizobium_sp._ORS_285	NA	NA	NA	NA	NA	NA	8.129283017	0	11.5287227	0.487150477	1.442685365	0.870622662
Streptomyces_hawaiiensis	NA	NA	NA	NA	NA	NA	8.129283017	6.682671608	7.745136469	0	0	0
Allofrancisella_guangzhouensis	NA	NA	NA	NA	NA	NA	8.129283017	7.68267863	12.72416422	0	0	0
Enterobacter_cloacae_complex_sp._ECNIH7	NA	NA	NA	NA	NA	NA	8.129283017	10.35434857	11.36880654	0	0	0
Phaeobacter_porticola	NA	NA	NA	NA	NA	NA	8.157119522	0	11.03242461	1.47701057	1.732015062	-0.256560464
Chlamydia_psittaci	NA	NA	NA	NA	NA	NA	8.166278594	8.110425888	9.30543935	0	0	0
Bartonella_elizabethae	NA	NA	NA	NA	NA	NA	8.246199041	0	0	0	0	0
Leclercia_sp._W17	NA	NA	NA	NA	NA	NA	8.246199041	9.380166652	0	0	0	0
Streptomyces_sampsonii	NA	NA	NA	NA	NA	NA	8.246199041	9.39192664	0	0	0	0
Oecophyllibacter_saccharovorans	NA	NA	NA	NA	NA	NA	8.409390936	0	0	-8.044394119	-8.044394119	-8.044394119
Raoultella_electrica	NA	NA	NA	NA	NA	NA	8.502740328	0	7.157114468	0	0	0
Streptomyces_olivaceus	NA	NA	NA	NA	NA	NA	8.502740328	0	9.39582543	0	0	0
Borrelia_turicatae	NA	NA	NA	NA	NA	NA	8.502740328	9.426646347	0	0	0	0
Streptomyces_collinus	NA	NA	NA	NA	NA	NA	8.6039638	0	0	0	0	0
Streptomyces_sp._3211	NA	NA	NA	NA	NA	NA	8.6039638	8.110425888	9.61571239	0	8.215935782	0
Streptomyces_ferrugineus	NA	NA	NA	NA	NA	NA	8.876516947	0	0	0	0	0
Massilia_sp._Se16.2.3	NA	NA	NA	NA	NA	NA	8.876516947	0	9.672264444	9.271463028	7.369230901	0
Mycobacterium_lepraemurium	NA	NA	NA	NA	NA	NA	8.876516947	9.426646347	0	0.712490874	-2.268979215	-1.197146985
Chitinimonas_arctica	NA	NA	NA	NA	NA	NA	8.876516947	10.50724956	10.88382698	-7.741466986	-1.271146509	-7.741466986
Streptomyces_sp._RLB1-33	NA	NA	NA	NA	NA	NA	9.029913869	6.682671608	8.904155902	0	0	0
Vibrio_spartinae	NA	NA	NA	NA	NA	NA	9.029913869	8.110425888	10.93496609	0.509938549	-8.900866808	-8.900866808
Neisseria_canis	NA	NA	NA	NA	NA	NA	9.029913869	10.40359793	9.731319031	8.27301694	9.214319121	0
Sodalis_praecaptivus	NA	NA	NA	NA	NA	NA	9.129283017	8.110425888	6.15711649	7.333897756	6.815921204	7.63420844
Pseudoalteromonas_espejiana	NA	NA	NA	NA	NA	NA	9.129283017	8.6794801	0	0.278859373	0.310566807	-0.54830403
Candidatus_Liberibacter_asiaticus	NA	NA	NA	NA	NA	NA	9.129283017	10.95261022	9.965784285	-0.958854017	-0.830680029	-0.640457613
Gordonia_sp._JH63	NA	NA	NA	NA	NA	NA	9.315767728	0	0	0	0	8.741466986
Methyloversatilis_sp._RAC08	NA	NA	NA	NA	NA	NA	9.315767728	11.41660755	10.77163802	7.129283017	8.912889336	0
Polynucleobacter_sp._MWH-UH21B	NA	NA	NA	NA	NA	NA	9.397770881	9.426646347	9.873724011	8.366322214	0	0
Pseudomonas_sp._ADPe	NA	NA	NA	NA	NA	NA	9.436143096	8.769388274	9.526943227	0	0	0
Mycolicibacterium_aurum	NA	NA	NA	NA	NA	NA	9.509955083	0	6.15711649	-7.930737338	-0.708348137	-1
Actinomyces_sp._zg-325	NA	NA	NA	NA	NA	NA	9.648765614	0	8.904155902	0	8.776982369	0
Moritella_viscosa	NA	NA	NA	NA	NA	NA	9.648765614	9.426646347	7.745136469	-0.793969653	-0.268739571	-1.867102844
Pseudoalteromonas_carrageenovora	NA	NA	NA	NA	NA	NA	9.661778098	10.51444778	8.965784285	0	0	0
Streptomyces_exfoliatus	NA	NA	NA	NA	NA	NA	9.732861042	10.87442045	9.965784285	7.303780748	0	6.544320001
Mycoplasma_synoviae	NA	NA	NA	NA	NA	NA	9.752760297	0	7.157114468	-0.486315358	-8.759332297	0.428843584
Mycoplasma_sp._Pen4	NA	NA	NA	NA	NA	NA	9.794711524	10.51085316	11.31112956	-7.741466986	-7.741466986	-1.197146985
Plantactinospora_sp._BB1	NA	NA	NA	NA	NA	NA	9.870925659	9.822499133	8.451211112	0	0	0
Citrobacter_sp._RHB35-C17	NA	NA	NA	NA	NA	NA	9.870925659	11.95459256	12.04022807	0	0	0
Pseudactinotalea_sp._HY158	NA	NA	NA	NA	NA	NA	10.00715344	0	11.27929137	0.921170151	-8.818048457	-0.692206246
Exiguobacterium_sp._MH3	NA	NA	NA	NA	NA	NA	10.31371068	0	6.15711649	0	0	0
Enterobacter_cloacae_complex_sp._FDA-CDC-AR_0164	NA	NA	NA	NA	NA	NA	10.52160044	10.46334045	0	0	0	0
Burkholderia_sp._BDU8	NA	NA	NA	NA	NA	NA	10.76336178	9.973625624	12.83242328	-7.741466986	-7.741466986	-7.741466986
Xanthomonas_albilineans	NA	NA	NA	NA	NA	NA	10.93797696	8.110425888	0	0	0	0
