# Supplementary material for: Host circadian behaviors exert only weak selective pressure on the gut microbiome under stable conditions but are critical for recovery from antibiotic treatment
Source: PLoS Biol. 2022 Nov 9;20(11):e3001865. doi: 10.1371/journal.pbio.3001865 (PMC9645659; doi:10.1371/journal.pbio.3001865)
Supplement: S6 Table — All groups distribute both groups and single mice among the individual cages. (PDF) [file pbio.3001865.s014.pdf]

Cage allocations by mouse group:

WT(UT)	Cage#2	3 mice	Total: 11
	Cage#4	4 mice	
	Cage#5	3 mice	
	Cage#18	1 mouse	
Per(UT)	Cage#8	4 mice	Total: 15
	Cage#10	3 mice	
	Cage#12	2 mice	
	Cage#13	2 mice	
	Cage#14	1 mouse	
	Cage#15	2 mice	
	Cage#19	1 mouse	
WT(T)	Cage#1	4 mice	Total: 11
	Cage#3	4 mice	
	Cage#6	1 mouse	
	Cage#16	1 mouse	
	Cage#17	1 mouse	
Per(T)	Cage#7	4 mice	Total:13
	Cage#9	4 mice	
	Cage#11	4 mice	
	Cage#20	1 mouse	

S6 Table. Cage allocation of mice for each group. All groups distribute both groups and single mice among the individual cages.
