# Supplementary material for: Host circadian behaviors exert only weak selective pressure on the gut microbiome under stable conditions but are critical for recovery from antibiotic treatment
Source: PLoS Biol. 2022 Nov 9;20(11):e3001865. doi: 10.1371/journal.pbio.3001865 (PMC9645659; doi:10.1371/journal.pbio.3001865)
Supplement: S7 Table — Each value here is the mean relative abundance of the chosen species from all the biological replicates at a given time point for the WT(T), Per(T), WT(UT), or Per(UT) samples, which were used to derive the volcano plots in Fig 4B. (PDF) [file pbio.3001865.s015.pdf]

S7 Table. Relative bacterial abundances at the species level (total sequencing reads for each species are divided by total sequencing reads per sample). Each value here is the mean relative abundance of the chosen species from all the biological replicates at a given time point for the WT(T), Per(T), WT(UT) or Per(UT) samples, which were used to derive the volcano plots in Fig 4B.

S7 Table

Relative bacterial abundances(mean value for each species at each time point) as species that were used to derive the volcano plots in Fig 4B.

	WT(T) Relative Abundance				Per(T) Relative Abundance				WT(UT) Relative Abundance				Per(UT) Relative Abundance			
Species	-14	11	154	238	-14	11	154	238	-14	11	154	238	-14	11	154	238
Candidatus_Babela_massiliensis	2.90364E-07	1.16364E-08	0	1.58545E-07	2.01538E-07	5.90308E-07	3.31692E-07	6.16077E-07	2.11273E-07	9.72727E-08	5.39909E-07	7.12364E-07	4.25733E-07	3.56733E-07	4.54333E-07	4.86267E-07
Candidatus_Bipolaricaulis_anaerobius	5.86364E-08	2.22364E-07	6.58182E-08	4.42182E-07	2.21615E-07	5.18923E-07	3.38077E-07	1.20769E-07	2.39545E-07	1.86636E-07	3.39818E-07	4.64636E-07	9.54667E-08	2.70133E-07	1.98373E-07	1.172E-07
Thermobaculum_terrenum	4.02545E-07	0.00000065	1.06909E-07	1.18609E-07	8.98077E-07	7.13077E-08	5.06231E-07	4.83846E-07	5.04273E-07	2.10091E-07	4.93818E-07	8.93364E-07	2.93733E-07	1.53133E-07	1.77867E-07	3.636E-07
Candidatus_Vampirococcus_archaeovorus	2.27182E-07	9.09091E-08	3.80364E-08	5.46636E-07	2.10154E-07	4.68846E-07	2.22538E-07	2.28615E-07	1.61364E-07	0.000000469	1.26727E-07	7.85455E-07	6.92E-08	2.15933E-07	1.06933E-07	1.048E-07
Coprothermobacter_proteolyticus	1.24545E-07	0	1.52818E-07	4.73091E-07	1.14615E-07	0.000000026	1.34846E-07	3.19308E-07	2.28818E-07	1.80273E-07	2.38545E-07	9.92727E-08	9.74E-08	8.49333E-08	1.18733E-07	1.98667E-08
Caldithrix_abyssi	6.08545E-07	7.13773E-07	5.55945E-07	7.32364E-07	3.27846E-07	8.93615E-07	6.07231E-07	0.000000411	0.000000775	6.80909E-07	5.53182E-07	7.33364E-07	4.22733E-07	4.536E-07	3.88667E-07	4.754E-07
Dictyoglomus_thermophilum	3.11818E-08	1.00955E-07	6.17727E-08	9.54545E-09	1.19769E-07	3.17385E-07	2.64154E-07	1.10385E-07	3.75455E-08	5.90909E-08	6.68182E-08	0.00000015	1.03533E-07	1.59467E-07	8.784E-08	2.15933E-07
Dictyoglomus_turgidum	4.64545E-07	0.00000013	3.24636E-07	1.34727E-07	5.19154E-07	0	2.63538E-07	3.84154E-07	6.61818E-08	3.11182E-07	1.34364E-07	1.41136E-07	4.81467E-07	1.446E-07	0.000000426	4.27267E-07
Caldisericum_exile	5.09909E-07	1.77273E-08	5.34545E-08	7.96091E-08	9.88769E-07	6.10769E-08	8.59231E-08	4.31154E-07	1.26727E-07	7.02727E-08	0.000000048	2.13727E-07	6.33467E-07	7.43933E-07	8.27773E-07	1.08107E-06
Desulfurispirillum_indicum	2.15764E-06	6.63455E-07	2.13209E-06	2.13282E-06	8.32538E-07	1.71054E-06	2.26346E-06	1.64654E-06	1.38018E-06	1.69382E-06	2.18164E-06	2.88382E-06	9.41667E-07	1.37493E-06	1.24893E-06	1.11853E-06
Thermodesulfovibrio_yellowstonii	4.64455E-07	0	4.12182E-08	3.24545E-08	8.69538E-07	0	3.11538E-08	2.98231E-07	1.15455E-07	2.14636E-07	0.000000163	3.28091E-07	4.15867E-07	6.01867E-07	1.14613E-06	7.29933E-07
Leptospirillum_ferriphilum	1.46364E-07	0	0	0	8.79231E-08	0	3.11538E-08	0	0	0	0	9.25455E-08	0	0	2.99733E-08	9.87333E-08
Leptospirillum_ferrooxidans	0.000001175	7.95455E-08	7.40909E-08	7.16727E-07	5.44538E-07	7.29231E-08	1.13077E-07	5.23923E-07	4.52818E-07	3.36455E-07	1.81509E-06	5.57818E-07	2.284E-07	2.77667E-07	7.67267E-07	3.406E-07
Nitrospira_japonica	1.23818E-07	8.77273E-08	2.13182E-07	4.56636E-07	2.13538E-07	5.29077E-07	2.73846E-08	8.87692E-08	2.09455E-07	2.28727E-07	1.58818E-07	2.69273E-07	2.85067E-07	0.000000253	2.832E-07	1.186E-07
Nitrospira_sp._KM1	1.90273E-07	1.18773E-06	0.00000017	4.67636E-07	1.36385E-07	0	1.46231E-07	2.33923E-07	9.19091E-08	7.51818E-08	2.43091E-07	6.36909E-07	4.51333E-08	1.008E-07	1.39773E-07	0
Candidatus_Nitrospira_inopinata	4.06727E-06	6.85909E-07	5.80727E-06	4.60636E-06	0.00000062	3.74077E-07	8.14385E-06	0.000008158	2.29627E-06	1.79327E-06	3.68727E-06	3.14727E-06	6.706E-07	1.0908E-06	1.8312E-06	1.37713E-06
Nitrospira_defluvii	2.71364E-07	3.60545E-07	1.40273E-07	3.14818E-07	3.15385E-08	0	1.21923E-07	0.000000042	1.17636E-07	0.000000103	2.76364E-08	1.90182E-07	2.14E-08	6.93333E-08	0	7.50667E-08
Nitrospira_moscoviensis	1.76545E-07	7.33727E-07	1.16436E-07	2.58636E-07	2.52077E-07	0	3.11538E-08	0.00000013	4.19909E-07	0.000000282	2.04091E-07	3.03636E-07	3.06067E-07	4.41867E-07	3.49773E-07	3.44667E-07
Caldimicrobium_thiodismutans	9.92727E-08	2.11273E-07	4.12182E-08	2.70973E-07	6.30769E-08	0.000000199	3.33077E-08	1.78846E-07	2.39455E-07	2.99727E-07	7.10909E-08	1.44455E-07	0	0	1.33107E-07	0
Thermosulfuriphilus_ammonigenes	1.28364E-07	6.51273E-07	2.46182E-08	7.15818E-07	1.42077E-07	7.46077E-07	0	1.68077E-07	3.94545E-08	9.11818E-08	1.02182E-07	1.06273E-07	2.64667E-08	0	2.72307E-07	9.33333E-09
Thermodesulfobacterium_sp._TA1	1.13545E-07	6.70909E-08	0	3.24545E-08	0	1.48538E-07	2.63077E-08	3.57692E-08	0	6.37273E-08	0	2.14545E-08	8.38667E-08	3.15333E-08	2.97333E-08	7.95333E-08
Thermodesulfobacterium_geofontis	0.000001092	3.94091E-07	7.69218E-07	3.05545E-07	5.26231E-07	2.52308E-08	4.73846E-07	3.16308E-07	4.22909E-07	4.94273E-07	3.65182E-07	6.49455E-07	2.73467E-07	2.77733E-07	1.89067E-07	1.664E-07
Thermodesulfobacterium_commune	2.03182E-07	0.000000065	4.94545E-08	0.000000054	8.30769E-08	0	8.33077E-08	5.38154E-07	7.50909E-08	0	1.16273E-07	7.51818E-08	8.24667E-08	6.38667E-08	2.98667E-08	5.70667E-08
Thermodesulfatator_indicus	3.84818E-07	3.13182E-07	3.01436E-07	5.59455E-07	2.66462E-07	0	0.000000235	3.77462E-07	0.000000378	3.81364E-07	2.96545E-07	5.58818E-07	1.62933E-07	5.276E-07	1.068E-07	3.65867E-07
Thermosulfurimonas_marina	1.03591E-06	2.92091E-07	3.00545E-07	8.24818E-07	8.55538E-07	4.70385E-07	1.35085E-06	5.70077E-07	7.83818E-07	5.31273E-07	6.72818E-07	1.63727E-06	5.65933E-07	1.33207E-06	6.92467E-07	6.236E-07
Desulfurobacterium_thermolithotrophum	1.78091E-07	1.68182E-08	0	7.76364E-08	7.48462E-08	6.27692E-08	1.51077E-07	1.01385E-07	0	2.87273E-08	3.78909E-07	7.07273E-08	1.938E-07	4.98E-08	2.152E-07	0.000000111
Thermovibrio_ammonificans	6.42455E-07	3.65545E-07	2.87309E-07	1.93127E-06	4.25538E-07	5.66154E-07	8.95077E-07	1.43662E-06	4.09182E-07	0.000000133	6.93273E-07	1.49545E-06	6.92667E-08	4.398E-07	8.686E-07	9.04933E-07
Thermosulfidibacter_takaii	4.75727E-07	3.81273E-07	1.26091E-07	0.000000431	3.27923E-07	0	6.18692E-07	0.000000053	2.76364E-07	2.07636E-07	2.27545E-07	3.78364E-07	4.50667E-08	7.02667E-08	4.60733E-07	4.56067E-07
Sulfurihydrogenibium_sp._YO3AOP1	7.34909E-07	1.09868E-06	5.11727E-07	4.70909E-07	6.08154E-07	1.42769E-07	7.61077E-07	1.23077E-07	2.63455E-07	1.49636E-07	7.47636E-07	1.17227E-06	1.45733E-07	2.566E-07	3.564E-07	5.21333E-07
Sulfurihydrogenibium_azorense	5.67182E-07	3.99773E-07	2.60545E-07	5.43273E-07	0.000000573	4.55385E-07	4.53615E-07	4.96846E-07	2.65727E-07	5.22727E-08	2.45818E-07	9.48636E-07	6.40733E-07	1.03847E-06	1.10773E-06	5.746E-07
Persephonella_marina	3.03273E-07	0	0	5.18182E-09	7.64692E-07	0	0	1.15385E-07	5.79091E-08	0.000000047	0	0	4.018E-07	3.76467E-07	1.26647E-06	1.1674E-06
Hydrogenobaculum_sp._HO	0	2.34545E-08	0	0	0	0	0	0	0	0	0	0	0	0	0	0
Hydrogenobaculum_sp._Y04AAS1	3.62727E-08	0.000000065	0	4.79091E-09	6.30769E-08	0	0	0	5.97273E-08	0	0	3.16364E-08	5.08667E-08	8.58E-08	0.00000009	0
Aquifex_aeolicus	3.11818E-08	0	0	1.68455E-07	5.86923E-07	5.05385E-08	5.26154E-08	3.57692E-08	7.50909E-08	9.49091E-08	3.95455E-08	1.04364E-06	4.01333E-08	1.05333E-07	1.024E-07	9.74E-08
Hydrogenobacter_sp._T-8	0.000000061	5.41818E-08	7.40909E-08	4.79091E-09	0	0	0	0	0	0	0	5.50455E-08	0	0	0	0
Hydrogenobacter_thermophilus	5.57182E-07	0	3.39091E-08	2.97273E-08	3.37538E-07	6.53077E-08	1.31538E-07	0	3.20364E-07	1.96182E-07	2.46273E-07	6.13091E-07	2.91333E-08	0.000000128	0	3.72467E-07
Thermocrinis_ruber	9.97273E-08	0	0.000000047	0	0	0	0	0	0	0	0	9.03636E-08	0	0	1.12667E-08	0
Thermocrinis_albus	8.53636E-08	0	2.13982E-07	1.92636E-07	1.12846E-07	0	3.93154E-07	2.93846E-08	0	0.000000116	1.06727E-07	9.31818E-08	2.37333E-08	8.98667E-08	1.3044E-07	9.33333E-09
Thermocrinis_minervae	1.28873E-06	0.00000026	5.23218E-07	7.42909E-07	1.91231E-07	0.000000026	6.64462E-07	5.56923E-07	1.94545E-07	7.80909E-07	4.76273E-07	5.82636E-07	3.60333E-07	5.04333E-07	1.08973E-06	4.12667E-07
Elusimicrobium_minutum	2.30436E-06	0.000000459	3.80636E-07	0.000000483	2.48231E-07	5.10769E-07	0	2.40308E-07	3.66727E-07	2.60727E-07	6.58182E-07	6.37545E-07	1.79333E-07	3.032E-07	2.21533E-07	3.49667E-07
Endomicrobium_proavitum	3.77545E-07	1.62682E-07	3.77391E-07	1.65182E-07	6.17692E-08	2.34615E-07	1.09923E-07	1.09692E-07	3.21727E-07	1.83182E-07	2.65818E-07	3.08455E-07	2.72E-08	1.322E-07	1.33333E-08	1.276E-07
Candidatus_Endomicrobium_trichonymphae	1.55845E-06	4.54545E-07	6.92364E-07	6.64636E-07	0.000000398	8.41538E-08	6.32692E-07	1.16838E-06	4.46273E-07	5.84818E-07	1.08709E-06	1.46818E-06	6.752E-07	8.552E-07	1.5612E-06	8.94067E-07
Athalassotoga_saccharophila	1.57264E-06	1.40455E-07	1.403E-07	1.33218E-06	1.21692E-07	5.15231E-07	5.30923E-07	0.000000838	5.20182E-07	5.49909E-07	5.85818E-07	4.22364E-07	4.76733E-07	4.98133E-07	2.85933E-07	4.15733E-07
Kosmotoga_pacifica	2.08182E-07	1.49545E-07	4.90909E-08	1.99636E-07	0.000000105	0	0.00000013	1.90769E-08	0	1.40545E-07	1.33636E-07	1.63545E-07	1.33333E-07	0.000000014	1.62107E-07	7.26E-08
Kosmotoga_olearia	3.40273E-07	8.18182E-08	2.47455E-07	1.30655E-06	2.43231E-07	6.27692E-08	6.84615E-08	1.42154E-07	7.95091E-07	9.99455E-07	9.07818E-07	0.000001307	2.56333E-07	2.58067E-07	4.8624E-07	6.46133E-07
Mesotoga_prima	0	1.37273E-08	6.71818E-08	2.65455E-07	1.13846E-07	5.05385E-08	0	1.90769E-08	1.86636E-07	6.69091E-08	0	2.00909E-07	0	0.000000014	2.99333E-08	0
Mesotoga_infera	9.68636E-07	0.000000106	1.14909E-07	1.71873E-06	4.51846E-07	8.52231E-07	8.49615E-07	2.73077E-06	7.07273E-07	6.54727E-07	1.86936E-06	0.000001607	7.54333E-07	6.15533E-07	1.54847E-06	1.2544E-06
Petrotoga_mobilis	0.000001033	1.70091E-07	6.19582E-07	1.16909E-06	3.55692E-07	6.76923E-08	8.86154E-07	9.61154E-07	5.14091E-07	9.71909E-07	1.59045E-06	1.26136E-06	3.90333E-07	3.18267E-07	1.56133E-07	1.02313E-06
Marinitoga_piezophila	0	0	0	0	0	0	0	0	0	0	0	7.13636E-09	0	0	0	0
Marinitoga_sp._1137	0	0	0	0	0	0	0	1.46923E-08	0	0	0	0	0	3.48E-08	0	0
Tepiditoga_spiralis	0.000001899	7.68727E-07	3.92436E-07	1.33309E-06	9.49923E-07	0	1.42646E-06	1.32523E-06	0.000001671	9.82818E-07	1.84627E-06	2.03691E-06	1.02093E-06	1.07947E-06	9.07467E-07	0.000000973
Defluviitoga_tunisiensis	1.78345E-06	4.25091E-07	1.85455E-07	1.92455E-07	1.50577E-06	1.60431E-06	1.57623E-06	9.73077E-07	1.23027E-06	1.11964E-06	8.46818E-07	2.00327E-06	2.0034E-06	1.99247E-06	2.15707E-06	2.19247E-06
Fervidobacterium_islandicum	0	0	0	0	0	0	0	0	0	0	0	0	0	0	0	1.98667E-08
Fervidobacterium_nodosum	0	0	5.34545E-08	4.88182E-08	0	0	2.63077E-08	2.46923E-08	0.000000087	8.32727E-08	2.44091E-07	1.42727E-08	2.94667E-08	3.26667E-08	7.43333E-08	0
Fervidobacterium_pennivorans	1.57273E-07	0	4.73636E-08	3.40909E-08	1.17077E-07	0	0	0	0	9.28182E-08	4.41727E-07	1.87909E-07	9.94667E-08	2.02467E-07	2.882E-07	1.98667E-08
Thermosipho_melanesiensis	5.27273E-08	1.21273E-07	4.65818E-08	1.78818E-07	6.09538E-07	1.03077E-07	2.63077E-08	7.72308E-08	1.02364E-07	3.11091E-07	3.35909E-07	9.86909E-07	3.15467E-07	3.40133E-07	5.162E-07	2.844E-07
Thermosipho_africanus	2.30909E-07	0	2.59091E-07	6.54273E-08	0.000000225	0	8.59231E-08	6.61923E-07	0.000000134	4.69091E-07	6.78545E-07	0.000000377	3.03933E-07	0.000000436	4.73533E-07	2.562E-07
Pseudothermotoga_thermarum	0	0	0	5.18182E-09	0	0	0	0	0	0	0	0	0	0	0	0
Pseudothermotoga_hypogea	0	0	0	3.24545E-08	8.83846E-08	4.27692E-08	1.42385E-07	9.30769E-08	3.75455E-08	8.85455E-08	1.00545E-07	1.34545E-07	2.61333E-07	2.494E-07	1.85533E-07	1.216E-07
Thermotoga_sp._RQ7	0	0	0	0	0	1.01692E-07	0	0	0	0	0	0	0	0	0	4.28E-08
Thermotoga_profunda	2.56364E-08	0	0	5.96364E-08	0	3.15385E-08	0	0.000000011	0	1.27636E-07	6.88182E-08	7.55455E-08	0	0.000000014	0	0
Thermotoga_sp._Cell2	0	1.77273E-08	5.43636E-08	0	0	9.72308E-08	0	0	0	3.03636E-08	0	0	0	1.58E-08	0	9.33333E-09
Thermotoga_caldifontis	5.82727E-08	4.05455E-08	5.78909E-08	1.29091E-07	0	0	0	7.72308E-08	0	7.25455E-08	1.02727E-08	0.00000007	2.91333E-08	0	4.44E-09	0
Chloracidobacterium_thermophilum	0.000000657	1.36909E-07	1.74727E-07	4.37182E-07	6.17692E-08	4.53538E-07	2.46462E-07	3.44462E-07	2.43909E-07	0.000000075	0.000000087	2.29273E-07	4.27333E-08	8.12667E-08	2.35667E-07	4.44667E-07
Luteitalea_pratensis	3.41645E-06	8.42545E-07	2.76468E-06	2.97818E-06	3.93231E-07	5.88308E-07	1.71846E-06	3.26592E-06	9.41818E-07	1.27273E-06	2.44036E-06	2.95618E-06	8.644E-07	1.11967E-06	7.272E-07	8.20733E-07
Paludibaculum_fermentans	1.01482E-05	2.2745E-06	5.55918E-06	6.52427E-06	9.18154E-07	3.52231E-07	5.90154E-06	7.26692E-06	3.54145E-06	4.71818E-06	4.29636E-06	6.40545E-06	1.3338E-06	1.1648E-06	1.5418E-06	1.58293E-06
Candidatus_Solibacter_usitatus	0.000001389	2.17273E-07	1.06718E-06	1.40345E-06	5.97769E-07	1.42631E-06	1.21908E-06	1.32662E-06	8.08909E-07	0.000000635	1.07255E-06	1.20491E-06	1.00193E-06	1.27727E-06	1.76033E-06	1.04147E-06
Candidatus_Koribacter_versatilis	5.85273E-07	3.17955E-07	0.000000276	5.18818E-07	7.18538E-07	2.95385E-08	1.90077E-07	2.47692E-08	2.62091E-07	2.96182E-07	2.97909E-07	3.24636E-07	7.88533E-07	6.792E-07	7.74067E-07	8.67933E-07
Edaphobacter_sp._12200R-103	3.05909E-07	0	0	2.92364E-07	1.76154E-07	0	2.46538E-07	1.58923E-07	1.14273E-07	1.29455E-07	1.32182E-07	4.72636E-07	1.94333E-07	2.08533E-07	2.62533E-07	2.246E-07
Edaphobacter_sp._4G125	9.63636E-08	1.53455E-07	1.63636E-08	1.68455E-07	9.75385E-08	9.29231E-08	8.37692E-08	1.90615E-07	9.28182E-08	1.60545E-07	7.90909E-08	2.01818E-07	7.15333E-08	1.30667E-07	1.07773E-07	4.84133E-07
Acidisarcina_polymorpha	4.89818E-07	1.48727E-07	1.23364E-07	1.83727E-07	5.47769E-07	9.09231E-07	3.14615E-07	4.58154E-07	7.15091E-07	5.49545E-07	9.38909E-07	6.70364E-07	6.48733E-07	7.36467E-07	8.49467E-07	5.96267E-07
Granulicella_sp._5B5	2.14364E-07	0.000000065	4.12727E-09	0	2.45462E-07	0	0.000000087	1.96385E-07	1.74636E-07	1.57364E-07	1.68636E-07	1.07455E-07	2.17667E-07	2.22733E-07	7.81333E-08	1.076E-07
Granulicella_mallensis	5.41455E-07	2.09364E-07	6.09818E-07	5.86364E-07	2.61615E-07	0	8.81538E-08	0.00000117	4.24727E-07	6.27273E-07	2.25727E-07	6.39909E-07	3.00533E-07	2.44133E-07	4.50733E-07	0.000000472
Granulicella_sp._WH15	2.05636E-07	0	0	4.07E-08	1.23154E-07	0.000000202	2.63077E-08	5.81538E-08	1.07091E-07	1.04545E-07	4.09364E-07	2.94273E-07	1.086E-07	1.53867E-07	3.238E-07	3.078E-07
Granulicella_tundricola	0.00000113	5.29182E-07	2.12618E-07	1.04573E-06	6.15385E-07	7.97231E-07	1.63454E-06	1.60462E-07	9.15273E-07	7.27273E-07	5.31364E-07	3.43182E-07	2.50467E-07	0.000000324	2.456E-07	8.91333E-08
Acidobacterium_sp._4Y35	5.04364E-07	1.86636E-07	0.000000373	1.12545E-07	2.59615E-07	1.79231E-08	1.13077E-07	0.000000087	4.88364E-07	6.58909E-07	5.54636E-07	5.24455E-07	2.714E-07	5.43933E-07	4.354E-07	1.49733E-07
Acidobacterium_capsulatum	3.37345E-06	4.54409E-07	7.73455E-07	2.02536E-06	1.78331E-06	8.93846E-07	9.70077E-07	1.50238E-06	2.63582E-06	2.09727E-06	2.73591E-06	3.30273E-06	1.44387E-06	0.000001673	1.2348E-06	1.84687E-06
Terriglobus_albidus	1.03127E-06	0.00000013	1.31545E-07	7.31636E-07	3.88462E-07	0	3.31231E-07	1.37792E-06	7.64818E-07	5.75818E-07	1.32555E-06	0.000000889	5.78067E-07	5.50933E-07	6.98667E-07	4.01733E-07
Terriglobus_saanensis	4.80273E-07	0.000000065	3.52818E-07	2.12455E-07	1.93846E-07	1.40769E-07	2.20769E-07	2.65308E-07	7.31273E-07	5.93909E-07	9.43545E-07	9.50091E-07	4.474E-07	4.40333E-07	6.01667E-07	4.27133E-07
Terriglobus_roseus	3.22545E-07	3.06545E-07	4.48982E-07	2.46536E-06	9.78846E-07	1.59531E-06	1.39215E-06	3.01569E-06	3.27091E-07	5.86091E-07	1.29409E-06	2.57336E-06	5.18733E-07	9.044E-07	1.34467E-06	1.3104E-06
Aminobacterium_colombiense	0	0	0	9.97273E-09	5.74615E-08	4.36538E-07	7.66923E-08	0	8.61818E-08	0	7.90909E-08	7.85455E-08	0	0	1.73773E-07	3.84667E-08
Thermovirga_lienii	5.25364E-07	5.93091E-07	2.88091E-08	1.32273E-07	2.53846E-07	9.46154E-09	1.77846E-07	4.42846E-07	2.50636E-07	2.94273E-07	1.56364E-07	5.86909E-07	1.63467E-07	2.194E-07	7.42E-08	3.084E-07
Jonquetella_anthropi	1.87209E-06	5.33909E-07	4.34455E-07	1.42645E-06	1.70046E-06	3.89954E-06	6.82077E-07	1.05938E-06	1.22418E-06	1.68209E-06	9.64364E-07	0.000002268	9.60333E-07	0.000001707	1.46413E-06	1.36493E-06
Thermanaerovibrio_velox	0.000000809	5.41818E-08	4.53455E-08	4.13182E-07	1.58615E-07	1.12346E-06	7.12538E-07	2.25154E-07	4.62909E-07	5.09455E-07	0.000000513	8.86545E-07	4.57133E-07	3.43733E-07	4.01067E-07	0.00000033
Thermanaerovibrio_acidaminovorans	4.89636E-06	0.000003812	1.60409E-06	3.19173E-06	1.85954E-06	9.71362E-06	2.86308E-06	5.35577E-06	3.84364E-06	2.06345E-06	2.82655E-06	5.26909E-06	1.4476E-06	2.1896E-06	1.99653E-06	1.95267E-06
Acetomicrobium_mobile	3.19273E-07	6.14364E-07	6.72636E-08	4.48727E-07	3.02308E-07	1.53354E-06	6.05385E-07	7.29769E-07	2.48636E-07	2.32182E-07	5.14727E-07	3.77818E-07	1.68533E-07	5.36667E-07	5.33667E-07	3.45867E-07
Aminomonas_paucivorans	6.28364E-06	4.14727E-06	1.84516E-06	5.06773E-06	2.52208E-06	0.000005041	2.98438E-06	3.36185E-06	5.09136E-06	4.19636E-06	4.73318E-06	7.40455E-06	2.71353E-06	3.30133E-06	3.3496E-06	3.15133E-06
Cloacibacillus_porcorum	0.000029	2.14435E-05	5.81918E-06	2.16308E-05	2.21677E-05	3.46108E-05	1.92538E-05	2.14823E-05	0.000031783	2.43109E-05	2.69355E-05	0.0000301	0.000015762	2.49493E-05	0.000018102	1.72867E-05
Turneriella_parva	2.68182E-07	2.32727E-08	9.85455E-08	9.70909E-08	1.30615E-07	1.71923E-06	3.06923E-07	2.05923E-07	1.81545E-07	6.69091E-08	7.90909E-08	9.64545E-08	1.27267E-07	3.50667E-08	2.30267E-07	9.12E-08
Leptospira_borgpetersenii	0	0	0	0	0	0	0	0	0	0	0	0	0	0	4.44E-09	3.67333E-08
Leptospira_biflexa	1.24545E-06	2.21636E-07	2.22727E-07	9.63636E-08	5.24769E-07	0	5.32154E-07	3.86769E-07	2.39727E-07	0.000000028	1.39091E-07	2.83273E-07	2.72E-08	3.50667E-08	0	9.93333E-08
Leptospira_interrogans	2.96364E-08	0	1.77273E-08	2.18609E-07	2.31923E-07	0	0	1.57846E-07	1.06545E-07	5.22727E-08	4.98182E-08	1.08727E-07	4.48667E-08	0.000000033	7.324E-08	0.00000011
Leptospira_weilii	9.83636E-08	0	2.18545E-08	2.39455E-07	2.28231E-07	0	1.32154E-07	2.30692E-07	5.15909E-07	1.77455E-07	3.98182E-07	2.93273E-07	5.08733E-07	3.92667E-07	7.184E-07	0.000000406
Leptospira_mayottensis	4.50364E-07	3.32136E-07	1.93464E-07	2.95182E-07	6.03846E-08	0	3.29615E-07	2.48615E-07	6.81273E-07	2.95273E-07	6.77182E-07	1.53545E-07	6.64667E-08	0	1.12667E-08	8.13333E-08
Leptospira_kmetyi	1.99364E-07	0	0.000000194	4.39455E-07	8.29692E-07	0	1.51462E-07	8.74154E-07	1.23945E-06	3.43636E-07	5.32909E-07	8.26364E-07	6.08667E-07	5.758E-07	1.04833E-06	7.92067E-07
Leptospira_tipperaryensis	5.79273E-07	0.000000065	7.40909E-08	1.95455E-07	1.56938E-06	6.77385E-06	1.70985E-06	0.000002316	9.08455E-07	5.84818E-07	1.01555E-06	1.15373E-06	1.37407E-06	8.49333E-07	1.47353E-06	2.19293E-06
Leptospira_santarosai	1.04855E-06	4.50818E-07	2.74218E-07	0.000000731	3.54154E-07	1.12231E-07	7.39462E-07	1.47877E-06	5.25727E-07	5.52182E-07	7.85455E-07	1.24609E-06	4.97733E-07	2.94667E-07	3.63133E-07	3.72267E-07
Brachyspira_hampsonii	4.35818E-07	3.33818E-07	1.21091E-07	1.11818E-07	3.06154E-07	0	1.57692E-07	0.000000052	0.000001641	1.47909E-07	1.77591E-06	1.08555E-06	2.002E-07	3.14867E-07	7.138E-07	5.70933E-07
Brachyspira_hyodysenteriae	1.06455E-07	5.03455E-07	3.32218E-07	4.04909E-07	2.22615E-07	2.84769E-07	4.33846E-08	5.43769E-07	1.69364E-07	2.99182E-07	1.95909E-07	9.35909E-07	2.406E-07	2.306E-07	3.67267E-07	3.77133E-07
Brachyspira_intermedia	9.09545E-07	1.77273E-08	7.83455E-08	4.40909E-08	2.17231E-06	2.95385E-08	1.94692E-07	0.000000276	0	1.33273E-07	3.04364E-07	8.26818E-08	1.1408E-06	1.50573E-06	1.15947E-06	1.14367E-06
Brachyspira_pilosicoli	1.84136E-06	1.24409E-07	7.20909E-08	3.80727E-07	4.11115E-06	0.000000189	5.35869E-06	7.54769E-06	7.45182E-07	7.53818E-07	1.72255E-06	3.01364E-06	3.75867E-06	4.35373E-06	6.59067E-06	5.70733E-06
Brachyspira_murdochii	2.35009E-06	2.50455E-07	1.10673E-07	2.03364E-08	7.90123E-06	4.13846E-08	1.84692E-07	2.97615E-07	8.26364E-08	4.91818E-07	2.25545E-07	1.75636E-07	5.0598E-06	5.04853E-06	4.8846E-06	2.9248E-06
Borreliella_garinii	0	0	0	0	0	0	0	0	0	0	0	7.13636E-09	0	0	0	0
Borreliella_spielmanii	0	0	0	0	1.39692E-07	0	0	0	7.89091E-08	0	0	1.42727E-08	4.68E-08	1.104E-07	6.46667E-08	8.62667E-08
Borreliella_afzelii	0	0	0	0	0	0	2.73846E-08	1.90769E-08	0	0	0	0	4.22667E-08	0	5.264E-08	0
Candidatus_Borrelia_tachyglossi	0	0	0	0	0	0	1.05385E-07	0	2.64545E-08	2.85455E-08	0	0.000000035	2.91333E-08	0	5.94E-08	6.81333E-08
Borreliella_burgdorferi	9.61818E-08	8.84545E-08	0	1.26091E-07	7.72308E-08	0	5.26154E-08	2.40385E-07	2.64545E-08	0	2.46545E-07	1.91273E-07	5.35333E-08	1.16467E-07	1.60867E-07	8.19333E-08
Borreliella_valaisiana	0	0	0	0	2.36154E-07	0	0	2.51538E-07	0	0	0	0	5.82E-08	2.80133E-07	7.46667E-08	0.000000059
Borrelia_mayonii	3.62727E-08	0	0	0	1.33846E-07	0	0	0	0	0	0	0	1.402E-07	1.61467E-07	8.39333E-08	9.33333E-09
Borrelia_maritima	3.11818E-08	0	0	0	0	0	0	0	0	0	0	9.03636E-08	0	0	0	0
Borrelia_duttonii	0	0	0	1.91818E-08	3.58462E-08	0	0	0	0	0	0	0	0	1.418E-07	2.99333E-08	1.26667E-07
Borrelia_hermsii	2.96364E-08	0	0	8.40909E-08	0	0	0	1.46923E-08	0	5.22727E-08	0	1.20909E-07	0	0	4.44E-09	0
Borrelia_crocidurae	0	1.04545E-08	0	0	2.75385E-08	0	0	0	0	0	6.88182E-08	0	0	0	0	0
Borrelia_turcica	0	0	0	5.18182E-09	0	0	2.73846E-08	0	6.64545E-08	0	1.02727E-08	3.16364E-08	1.86667E-08	0	5.94E-08	0
Borrelia_turicatae	0	0	3.70909E-08	0	0	0	0	0	0	0	0	0	0	0	0	0
Borrelia_coriaceae	0.00000014	1.64545E-07	1.63636E-08	1.22636E-07	0	0	0	2.46923E-08	3.94727E-07	1.26273E-07	1.02182E-07	1.01636E-07	2.14667E-08	0	0	3.84667E-08
Borrelia_miyamotoi	0	2.70909E-08	5.32182E-08	0	3.15385E-08	0	0	0	0	0	3.33636E-08	0	2.14667E-08	0.000000033	2.99333E-08	0
Borrelia_sp._A-FGy1	0	0	0	0	5.93846E-08	8.10769E-08	0	0	0	0	0	7.13636E-09	4.73333E-08	2.598E-07	4.718E-07	0
Salinispira_pacifica	4.34909E-07	6.73227E-07	3.08182E-07	8.15091E-07	1.33923E-07	1.37692E-07	6.96923E-08	4.58308E-07	9.20909E-08	3.18364E-07	0.000000899	5.71727E-07	8.52E-08	1.15067E-07	9.23067E-08	1.826E-07
Thiospirochaeta_perfilievii	1.82773E-06	8.43545E-07	5.10636E-07	4.10545E-07	9.03308E-07	4.25154E-07	9.28462E-07	8.16231E-07	3.66727E-07	5.30636E-07	0.000001207	7.70182E-07	6.81533E-07	8.088E-07	1.29213E-06	7.88333E-07
Oceanispirochaeta_crateris	9.32818E-07	7.68364E-07	2.90364E-07	5.66727E-07	2.94923E-07	0.000000495	4.87154E-07	8.83231E-07	3.98364E-07	9.22727E-08	6.40273E-07	7.94818E-07	2.078E-07	3.21067E-07	5.72267E-07	9.54E-08
Sediminispirochaeta_smaragdinae	1.97409E-06	1.89818E-06	3.47818E-07	3.46509E-06	4.19869E-06	1.61702E-05	1.75162E-06	2.21523E-06	3.25891E-06	3.27982E-06	3.63973E-06	5.48109E-06	3.65807E-06	6.65267E-06	3.10133E-06	1.76687E-06
Sphaerochaeta_pleomorpha	1.46373E-06	2.81509E-06	5.81909E-07	1.43818E-06	5.70231E-07	1.70046E-06	0.000000442	9.99077E-07	1.22564E-06	8.03455E-07	1.10109E-06	2.54018E-06	4.72867E-07	1.00873E-06	6.26067E-07	7.15067E-07
Sphaerochaeta_coccoides	1.39736E-06	2.35345E-06	5.364E-07	1.87273E-06	2.33262E-06	2.53008E-06	2.23731E-06	1.60785E-06	1.85973E-06	7.02545E-07	0.00000116	2.07009E-06	2.11393E-06	2.4858E-06	2.51613E-06	1.4814E-06
Sphaerochaeta_globosa	0.00000348	1.16482E-06	1.67516E-06	3.06882E-06	1.74438E-06	0.000009459	3.41492E-06	4.01685E-06	2.34036E-06	2.21618E-06	2.17555E-06	3.35273E-06	1.91973E-06	1.72173E-06	1.64207E-06	0.000001833
Spirochaeta_thermophila	3.43818E-06	3.51636E-07	3.35364E-06	5.20909E-06	1.12085E-06	5.74308E-07	5.82446E-06	6.99323E-06	3.74918E-06	1.46582E-06	2.91909E-06	2.86791E-06	1.38527E-06	9.49667E-07	1.80647E-06	1.29553E-06
Spirochaeta_africana	0.000000899	1.16364E-08	7.82182E-08	4.00727E-07	7.58692E-07	2.03692E-07	2.05546E-06	2.41838E-06	1.23345E-06	1.23173E-06	1.04145E-06	1.46855E-06	4.294E-07	1.38873E-06	6.686E-07	6.174E-07
Treponema_putidum	2.91545E-07	4.88545E-07	5.03182E-08	5.52455E-07	3.38077E-07	2.02923E-07	1.22462E-07	5.57846E-07	0.000000202	2.51727E-07	2.36273E-07	5.64545E-07	2.614E-07	3.576E-07	1.35933E-07	4.008E-07
Treponema_socranskii	1.45355E-06	0.000000162	7.87273E-07	1.41555E-06	7.30385E-07	2.87231E-07	6.43462E-07	1.20423E-06	1.72818E-06	1.69782E-06	1.00745E-06	1.92309E-06	6.62933E-07	0.000000861	1.06607E-06	7.95267E-07
Treponema_caldarium	0.000000184	8.20909E-08	0.000000258	5.79909E-07	0.000001632	8.08154E-07	3.06769E-07	7.62615E-07	7.74909E-07	3.58455E-07	5.10364E-07	8.35636E-07	1.15433E-06	1.28047E-06	1.2764E-06	1.3552E-06
Treponema_phagedenis	2.38591E-06	1.73668E-06	7.82727E-07	3.62655E-06	4.38885E-06	2.92785E-06	0.000005937	3.05808E-06	0.000004	4.00509E-06	3.03545E-06	4.34609E-06	2.97973E-06	3.87553E-06	0.00000355	3.3464E-06
Treponema_pedis	1.39873E-06	5.76182E-07	1.50518E-06	9.75727E-07	1.83185E-06	2.70769E-07	6.36769E-07	3.75769E-07	1.65409E-06	2.22382E-06	2.34836E-06	1.90518E-06	1.92133E-06	3.52747E-06	2.07327E-06	1.64893E-06

Treponema_brennaborense	2.80245E-06	1.31045E-06	3.50773E-06	6.57727E-06	0.000001677	1.49523E-06	9.67877E-06	1.08111E-05	0.000003917	2.58464E-06	3.35382E-06	3.62018E-06	8.06933E-07	1.66953E-06	2.42673E-06	2.13793E-06
Treponema_azotonutricium	5.38909E-06	8.05727E-07	2.58132E-06	3.48591E-06	1.71369E-06	0.000002381	4.12385E-06	4.35569E-06	3.21718E-06	3.07182E-06	3.29909E-06	4.42455E-06	1.82773E-06	2.79907E-06	2.1886E-06	2.0372E-06
Treponema_sp._OMZ_804	1.04791E-06	0.000000993	2.02764E-07	1.21427E-06	8.94846E-07	1.09938E-06	0.000000738	8.85385E-07	1.06755E-06	0.000001176	1.19591E-06	1.89536E-06	4.92533E-07	5.33667E-07	8.632E-07	1.44747E-06
Treponema_sp._Marseille-Q4132	1.12836E-06	2.20545E-07	1.83036E-07	4.83909E-07	5.50769E-07	1.22154E-07	2.79308E-07	5.47615E-07	1.02755E-06	0.000000888	6.04636E-07	7.11909E-07	7.68467E-07	1.2442E-06	7.87533E-07	1.06073E-06
Treponema_sp._OMZ_838	9.84818E-07	6.46182E-07	2.53182E-07	6.10909E-07	4.13308E-07	1.03308E-06	6.90846E-07	0.000000582	6.75182E-07	4.19818E-07	1.37627E-06	1.47082E-06	6.452E-07	7.538E-07	8.94133E-07	5.64133E-07
Treponema_denticola	3.74891E-06	3.64827E-06	9.91727E-07	3.21427E-06	1.67377E-06	6.51962E-06	2.05423E-06	2.02823E-06	6.16355E-06	7.40364E-06	3.78145E-06	5.00273E-06	1.86067E-06	2.06927E-06	2.51593E-06	1.62733E-06
Treponema_primitia	3.88291E-06	0.000004769	1.36373E-06	4.14909E-06	1.63808E-06	4.24531E-06	2.07515E-06	2.19308E-06	3.58073E-06	2.95636E-06	3.68173E-06	5.55909E-06	1.65887E-06	3.8504E-06	3.07287E-06	0.000001744
Treponema_succinifaciens	5.69182E-06	2.23473E-06	1.6565E-06	6.26182E-06	1.18392E-05	2.08615E-05	5.53577E-06	0.0000084	4.09818E-06	3.57936E-06	6.77091E-06	9.07545E-06	9.45067E-06	1.05587E-05	0.000010894	8.16467E-06
Kiritimatiella_glycovorans	1.86055E-06	2.34818E-07	9.43436E-07	2.38282E-06	6.46692E-07	1.10369E-06	7.81231E-07	1.83985E-06	1.57236E-06	8.71364E-07	2.44091E-06	2.06582E-06	7.42067E-07	7.22467E-07	8.18867E-07	9.15933E-07
Waddlia_chondrophila	0	0	0	2.96273E-07	7.72308E-08	0	5.26154E-08	3.57692E-08	6.68182E-08	9.36364E-08	2.04091E-07	3.16364E-08	4.22E-08	1.61333E-08	0	0.000000304
Simkania_negevensis	0	2.70909E-08	0	4.79091E-09	0	0	0	0	0	0	3.95455E-08	4.21364E-08	0	2.38E-08	0	0
Parachlamydia_acanthamoebae	7.83636E-08	5.41818E-08	0	4.79091E-09	2.04769E-07	0	0	7.63077E-07	0	9.56364E-08	0	0	1.51667E-07	1.13133E-07	3.454E-07	4.85333E-07
Neochlamydia_sp._S13	3.11818E-08	0	6.67273E-08	6.62455E-08	2.75385E-08	0	0	4.37692E-08	0.000000117	1.04273E-07	1.69909E-07	3.74545E-08	4.33333E-08	1.71333E-08	3.68E-08	5.19333E-08
Candidatus_Protochlamydia_amoebophila	5.97273E-08	0.00000013	6.98182E-08	3.94545E-08	6.23077E-08	0	0	0	2.60273E-07	0.000000034	1.34364E-07	1.83545E-07	1.066E-07	1.61333E-08	4.44E-09	1.22E-08
Candidatus_Protochlamydia_naegleriophila	1.25736E-06	0	4.12727E-09	0	1.48762E-06	0	0	1.22692E-07	1.73091E-06	1.78636E-06	8.19091E-07	3.13364E-06	2.82593E-06	2.0274E-06	1.9104E-06	2.37067E-06
Chlamydia_psittaci	0	0	0	0	0	0	0	1.46923E-08	0	0	0	1.42727E-08	0	0	0	0
Chlamydia_trachomatis	0	0	0	0	0	0	0	0	3.98182E-08	0	0	0.000000035	0	0	0	0
Chlamydia_felis	0	0	0	0	0	0	0	0	0	0	0	0	0	0	1.49333E-08	0
Chlamydia_sp._S15-834C	0	0	0	0	0	0	0	0	0	0	0	0	0	0	1.49333E-08	0
Chlamydia_caviae	0	0	2.96364E-08	0	0	0	0	0	0	0	0	0	0	0	0	0
Chlamydia_avium	0	0.000000065	0	1.22727E-07	0	0	0	0	1.55636E-07	1.38909E-07	3.33636E-08	0	0	0	1.57067E-08	0
Chlamydia_suis	3.62727E-08	2.70909E-08	2.64818E-08	4.20909E-08	0	6.30769E-08	8.92308E-08	0	8.51818E-08	3.12364E-07	1.73364E-07	2.73273E-07	9.78E-08	9.24E-08	4.44E-09	0
Chlamydia_gallinacea	7.81818E-08	0	7.14545E-08	1.91818E-08	0	0	0	0	0	0	3.95455E-08	3.16364E-08	0	0	0	0
Chlamydia_pneumoniae	4.29091E-08	0	7.40909E-08	0	0	0	0	0	4.45455E-08	2.85455E-08	0	3.16364E-08	0	3.48E-08	1.49333E-08	3.40667E-08
Chlamydia_sp._2742-308	3.12545E-07	0.000000065	4.85364E-07	6.83636E-08	0.000001231	0	0.00000013	1.50308E-07	1.06545E-07	2.62818E-07	1.41182E-07	9.03636E-08	4.03667E-07	4.328E-07	7.72507E-07	6.72933E-07
Chlamydia_sp._H15-1957-10C	1.11818E-07	0	0	1.18282E-06	9.94846E-07	0	0.000000447	6.34615E-07	0	1.69455E-07	0.000000535	5.67682E-07	1.44573E-06	1.10387E-06	2.34387E-06	2.78407E-06
Chlamydia_pecorum	0	0	6.33636E-08	1.70091E-07	1.85538E-07	1.11923E-07	0	7.15385E-08	0.000000027	3.03636E-08	6.88182E-08	7.13636E-09	1.58733E-07	7.666E-07	7.16133E-07	3.40667E-08
Candidatus_Kuenenia_stuttgartiensis	0.00000025	0.000000565	9.98545E-08	2.95636E-07	3.58154E-07	4.03846E-07	2.74538E-07	2.30923E-07	4.39091E-07	3.55182E-07	5.65545E-07	6.78273E-07	3.79933E-07	7.49067E-07	8.07533E-07	6.70667E-07
Anaerohalosphaera_lusitana	8.50545E-07	1.24636E-07	5.15791E-07	2.08155E-06	4.80846E-07	1.92308E-07	3.20923E-07	2.48692E-07	7.14091E-07	0.000000899	1.52155E-06	1.07818E-06	4.128E-07	6.61867E-07	0.000000584	7.686E-07
Limihaloglobus_sulfuriphilus	1.26255E-06	1.68182E-08	2.72309E-07	0.000001146	0.000000231	2.75231E-07	9.27615E-07	1.07969E-06	0.000000779	2.18636E-07	6.41091E-07	1.02455E-06	0.000000244	1.97333E-07	3.91533E-07	4.77067E-07
Sedimentisphaera_salicampi	3.28909E-07	0	8.69818E-08	1.33091E-07	8.79231E-08	1.74154E-07	0.000000093	1.26846E-07	2.22636E-07	5.19182E-07	9.71818E-07	2.54545E-07	1.14067E-07	6.18E-08	5.396E-07	2.12867E-07
Sedimentisphaera_cyanobacteriorum	6.91818E-08	0	3.70909E-08	3.82091E-07	3.11923E-07	1.37077E-07	0	3.74385E-07	7.19182E-07	1.64909E-07	1.77636E-07	3.38273E-07	2.45667E-07	4.73667E-07	5.786E-07	3.39333E-07
Phycisphaera_mikurensis	2.16627E-06	8.43455E-07	5.44545E-07	8.02545E-07	2.75854E-06	9.75077E-07	3.73846E-07	7.45385E-07	4.50027E-06	3.42455E-06	1.67136E-06	1.69691E-06	2.4802E-06	4.35687E-06	2.64533E-06	2.09193E-06
Isosphaera_pallida	7.08818E-07	0	7.40909E-08	4.12273E-07	1.19077E-07	1.71231E-07	2.63077E-08	1.90769E-08	3.14727E-07	9.72727E-08	7.90909E-08	0.000000471	1.93733E-07	9.72E-08	9.84667E-08	2.688E-07
Singulisphaera_acidiphila	5.81545E-07	0	0.000000491	6.17455E-07	2.46231E-07	2.41154E-07	3.42692E-07	6.70769E-07	5.58909E-07	4.57455E-07	4.61727E-07	5.21273E-07	3.40133E-07	1.07933E-07	2.754E-07	5.784E-07
Paludisphaera_borealis	4.93364E-07	6.58182E-08	6.33636E-08	3.02727E-07	2.13231E-07	0.000000294	1.01462E-07	1.74462E-07	5.26273E-07	1.16364E-07	4.65636E-07	7.11364E-07	2.186E-07	1.18133E-07	1.984E-07	2.07667E-07
Aquisphaera_giovannonii	1.09664E-05	2.79173E-06	1.11247E-05	7.81864E-06	4.28462E-07	1.18415E-06	1.25269E-06	4.79931E-06	4.04727E-06	6.71727E-06	7.54836E-06	9.38636E-06	4.16533E-07	7.352E-07	6.97133E-07	5.01133E-07
Thermogutta_terrifontis	9.76364E-08	1.38864E-07	0	1.93636E-08	2.88462E-08	0	2.63077E-08	1.24846E-07	5.97273E-08	1.55273E-07	3.95455E-08	6.41364E-08	0	3.26667E-08	0	6.33333E-08
Mariniblastus_fucicola	1.91909E-07	0.00000013	1.10909E-07	7.98182E-08	3.34615E-08	0	0.00000008	2.47692E-08	2.39091E-07	0	6.88182E-08	0	0	0	0	3.67333E-08
Rhodopirellula_baltica	2.84636E-07	9.75455E-08	5.34545E-08	1.50909E-07	1.53077E-07	5.47692E-08	2.19308E-07	4.64615E-07	0.00000036	1.60818E-07	3.29818E-07	2.82455E-07	0	3.09333E-08	2.38E-08	1.20267E-07
Pirellula_staleyi	5.49182E-07	0	1.99091E-07	1.70091E-07	2.88462E-08	2.13846E-08	0	3.64769E-07	1.84455E-07	6.42727E-08	3.33727E-07	9.60636E-07	1.12533E-07	3.26667E-08	6.31333E-08	7.26667E-08
Pirellula_sp._SH-Sr6A	4.79091E-07	0	1.03636E-06	1.21427E-06	2.71231E-07	3.29769E-07	3.04954E-06	3.53054E-06	8.27727E-07	3.39727E-07	5.20909E-07	5.30545E-07	1.71533E-07	3.07333E-07	4.472E-07	1.45067E-07
Roseimaritima_ulvae	2.64845E-06	2.80727E-07	2.0433E-06	2.86864E-06	3.46308E-07	7.66154E-08	1.38723E-06	1.16015E-06	1.60909E-06	9.06364E-07	1.12855E-06	2.00527E-06	4.57067E-07	3.85667E-07	4.56533E-07	7.27133E-07
Lacipirellula_parvula	2.13264E-06	0.00000033	9.33455E-07	1.56845E-06	7.39846E-07	1.17677E-05	2.74415E-06	3.65892E-06	2.01218E-06	9.59091E-07	1.70618E-06	3.47091E-06	6.308E-07	1.09933E-06	1.02127E-06	1.7024E-06
Bythopirellula_goksoyri	6.02455E-07	0.000000065	1.39636E-07	1.48818E-07	0	3.21538E-07	5.54615E-08	4.40923E-07	8.15455E-08	1.58455E-07	0.000000239	3.05273E-07	8.54667E-08	1.26667E-07	5.09067E-08	1.64867E-07
Tuwongella_immobilis	9.52091E-07	0	4.72491E-07	7.79727E-07	2.92769E-07	0	7.04385E-07	6.78923E-07	9.03818E-07	5.88091E-07	4.35636E-07	4.05909E-07	3.546E-07	1.792E-07	3.73267E-07	3.58733E-07
Frigoriglobus_tundricola	1.22909E-06	1.53273E-07	3.54945E-07	1.44891E-06	1.88462E-07	6.13846E-08	3.15462E-07	8.74462E-07	9.14273E-07	7.27455E-07	8.65545E-07	8.69818E-07	7.99333E-07	8.556E-07	8.24933E-07	1.4454E-06
Limnoglobus_roseus	9.48727E-07	2.17818E-07	1.52309E-07	1.49927E-06	8.00692E-07	7.29231E-08	0.000000431	6.55846E-07	2.99455E-07	4.15364E-07	0.000000488	8.67273E-07	0.000000536	5.748E-07	8.518E-07	1.30753E-06
Gemmata_sp._SH-PL17	1.04364E-07	0	0	0	0.000000099	0	5.80769E-08	0	5.97273E-08	9.92727E-08	0	1.90227E-07	1.86667E-08	0	0	3.67333E-08
Gemmata_massiliana	4.07273E-07	4.37636E-07	3.764E-07	0.000000767	9.69231E-08	0	4.92846E-07	5.96231E-07	7.86273E-07	4.32091E-07	3.80909E-07	5.98682E-07	3.362E-07	1.434E-07	2.444E-07	2.85333E-07
Gemmata_obscuriglobus	3.70818E-07	3.41818E-07	4.464E-07	2.07573E-06	3.28231E-07	1.19831E-06	2.02315E-06	3.28962E-06	5.20182E-07	2.71636E-07	1.93191E-06	1.31909E-06	3.69267E-07	6.626E-07	1.23013E-06	1.14533E-06
Bremerella_volcania	1.58182E-08	0	1.77273E-08	4.84545E-08	5.47692E-08	1.63769E-07	0.00000011	2.47692E-08	0	0.000000047	2.76364E-08	3.87727E-08	1.60733E-07	1.478E-07	1.60867E-07	1.86667E-08
Planctopirus_limnophila	8.96364E-08	9.36364E-09	0	0	0	0	1.69846E-07	7.28462E-08	2.64545E-08	8.62727E-08	0	1.58455E-07	0.000000027	3.09333E-08	0.000000041	1.98667E-08
Planctopirus_ephydatiae	2.87818E-07	0.000000065	1.84727E-07	8.53636E-08	0	0	0	0	0	1.02909E-07	0	2.48545E-07	0	0.000000014	0	6.33333E-08
Gimesia_maris	4.57636E-07	2.41818E-08	1.58818E-07	4.14545E-07	6.33846E-08	7.09231E-08	2.26154E-07	1.73231E-07	1.66182E-07	1.46364E-07	3.89455E-07	0.000000292	4.23333E-08	1.62133E-07	9.29733E-08	0.000000083
Gimesia_benthica	1.46273E-07	1.44227E-07	7.80727E-08	7.27273E-08	1.18385E-07	0	1.28923E-07	4.07692E-08	6.56545E-07	1.38291E-06	1.06827E-06	1.04145E-06	1.40667E-07	3.728E-07	2.566E-07	0.00000063
Fuerstia_marisgermanicae	3.75091E-07	0.000000044	3.36455E-07	2.93182E-07	3.25846E-07	5.19231E-08	7.26462E-07	4.30538E-07	3.40455E-07	0.000000418	4.02909E-07	5.74273E-07	2.07267E-07	3.55733E-07	6.984E-08	0.000000288
Crateriforma_conspicua	1.03518E-06	0.000000065	1.87091E-07	2.25091E-07	1.55315E-06	2.95385E-08	1.88846E-07	0.000000158	1.46255E-06	1.26764E-06	8.71727E-07	1.41427E-06	1.91933E-06	1.19307E-06	1.5694E-06	0.000001929
Rubinisphaera_brasiliensis	1.98364E-06	2.93727E-07	1.64091E-06	1.50727E-06	7.17692E-08	1.52692E-07	1.79538E-06	1.96408E-06	6.13545E-07	8.52182E-07	1.25718E-06	1.31164E-06	6.99333E-08	4.13533E-07	1.24333E-07	4.12067E-07
Alienimonas_californiensis	1.02455E-06	1.04473E-06	1.94909E-07	1.00945E-06	7.58231E-07	5.55231E-07	9.58769E-07	1.57038E-06	1.54209E-06	1.23845E-06	9.78818E-07	2.01564E-06	4.75333E-07	8.06333E-07	4.74667E-07	6.706E-07
Planctomyces_sp._SH-PL14	7.75091E-07	3.24545E-07	3.69818E-07	3.24273E-07	6.10846E-07	5.86615E-07	4.02923E-07	4.42692E-07	3.95909E-07	4.91273E-07	6.94364E-07	2.64091E-07	0.000000417	7.88133E-07	6.14933E-07	9.46533E-07
Planctomyces_sp._SH-PL62	1.35436E-06	3.24545E-07	3.70909E-08	1.45764E-06	3.12231E-07	2.13077E-07	2.06385E-07	8.08231E-07	6.44818E-07	2.80273E-07	9.97636E-07	1.09255E-06	4.70733E-07	6.80133E-07	8.272E-07	0.000001238
Candidatus_Xiphinematobacter_sp._Idaho_Grape	1.59818E-07	0	0.00000046	3.10364E-07	0.000000061	0	9.69769E-07	1.18285E-06	2.35545E-07	1.32909E-07	2.28909E-07	2.68273E-07	1.44733E-07	1.74533E-07	1.63667E-07	3.71533E-07
Methylacidiphilum_kamchatkense	0	7.04545E-08	0	5.18182E-09	0	9.57692E-08	0	0.000000154	0	0	0	6.32727E-08	2.94667E-08	0	1.33333E-08	1.18667E-07
Methylacidiphilum_fumariolicum	2.44545E-07	9.54545E-09	0	4.79091E-09	3.15385E-08	0	6.13846E-08	1.15385E-07	1.38364E-07	2.04636E-07	1.02727E-08	1.22182E-07	9.12667E-08	1.16267E-07	2.01333E-07	1.00133E-07
Methylacidiphilum_infernorum	1.80691E-06	4.66182E-07	0.000000074	4.44182E-07	5.52923E-07	7.76923E-08	9.87923E-07	9.59231E-08	1.46227E-06	0.000004179	1.79618E-06	0.000001978	0.000000278	3.482E-07	2.062E-07	2.966E-07
Coraliomargarita_akajimensis	1.02882E-06	0	4.68945E-07	1.26236E-06	2.55538E-07	6.53077E-08	8.25077E-07	6.41077E-07	1.02336E-06	0.000000664	1.15945E-06	6.21273E-07	3.52267E-07	1.74667E-07	4.666E-07	6.56467E-07
Nibricoccus_aquaticus	1.30282E-06	1.71364E-07	5.66545E-07	1.32464E-06	1.93308E-07	1.20277E-06	3.13538E-07	8.76308E-07	1.43236E-06	0.000000996	1.42582E-06	1.54536E-06	6.17667E-07	7.45067E-07	9.22467E-07	8.90067E-07
Ereboglobus_luteus	1.97027E-06	6.75E-08	1.52509E-06	2.04364E-06	3.03923E-07	2.23538E-07	2.26969E-06	1.25792E-06	8.27182E-07	1.34618E-06	1.88855E-06	1.94382E-06	5.926E-07	7.74267E-07	6.80667E-07	1.25527E-06
Lacunisphaera_limnophila	1.11909E-06	0	8.764E-07	1.23318E-06	4.15923E-07	7.26923E-08	0.00000111	9.13923E-07	1.15582E-06	7.52545E-07	9.28364E-07	8.52545E-07	6.62867E-07	6.696E-07	8.83267E-07	5.06133E-07
Opitutus_terrae	3.75273E-06	7.29273E-07	2.37071E-06	4.06809E-06	1.00246E-06	2.29615E-06	6.27877E-06	0.000004193	2.94618E-06	2.85764E-06	5.04455E-06	4.20091E-06	1.11173E-06	1.52953E-06	0.000001228	1.0126E-06
Opitutus_sp._GAS368	2.36827E-06	1.41636E-07	1.59436E-06	1.42509E-06	9.39538E-07	0.000000406	2.69469E-06	1.72538E-06	8.38818E-07	8.05455E-07	1.90127E-06	1.65636E-06	6.848E-07	1.53153E-06	1.12873E-06	1.14493E-06
Verrucomicrobium_sp._GAS474	1.15436E-06	1.94545E-07	1.19664E-07	1.66773E-06	3.40769E-07	1.06692E-07	4.26846E-07	4.82308E-07	0.000000789	2.61364E-07	4.73091E-07	6.59545E-07	2.29667E-07	4.396E-07	2.91467E-07	6.766E-07
Verrucomicrobium_spinosum	2.35545E-07	4.58818E-07	1.56273E-07	6.60909E-07	2.61923E-07	1.31531E-06	6.23846E-07	6.53538E-07	4.42727E-07	4.80636E-07	4.22727E-07	6.89909E-07	2.616E-07	3.34933E-07	7.85067E-07	0.000000351
Roseimicrobium_sp._ORNL1	1.53636E-06	1.04709E-06	2.50877E-06	2.99764E-06	8.38769E-07	9.41769E-07	5.63492E-06	4.31031E-06	1.49691E-06	9.60545E-07	1.11982E-06	1.12491E-06	7.91267E-07	7.11667E-07	1.09093E-06	1.07227E-06
Luteolibacter_sp._G-1-1-1	1.03073E-06	1.31273E-07	1.15036E-07	5.25091E-07	1.29308E-07	4.48846E-07	1.60462E-07	1.74846E-07	3.27727E-07	2.57455E-07	1.75909E-07	6.03545E-07	6.42E-08	8.51333E-08	7.98667E-08	4.38267E-07
Akkermansia_glycaniphila	0.000003841	0.000000651	0.000003035	3.28364E-06	0.000001073	6.60769E-07	3.10692E-06	2.81392E-06	2.16818E-06	1.67109E-06	2.20164E-06	1.89927E-06	8.254E-07	1.9686E-06	1.5942E-06	1.93127E-06
Akkermansia_muciniphila	0.014766292	0.352456524	0.071551704	0.018287628	0.00013168	0.00441029	0.008333055	0.004814753	0.001234557	0.000732939	0.000334897	0.001961049	0.000225793	9.13353E-05	9.51312E-05	0.000121201
Candidatus_Chazhemtobacterium_aquaticus	3.62727E-08	0	0	0	2.22769E-07	0.000000167	8.33077E-08	7.13077E-08	0	0	6.68182E-08	3.59455E-07	8.72E-08	1.40133E-07	1.19267E-07	8.72667E-08
Candidatus_Nanosynbacter_lyticus	3.04279E-05	1.81818E-06	1.23182E-05	2.46118E-05	1.96092E-05	8.84615E-08	2.56015E-05	2.17408E-05	1.86409E-05	2.32864E-05	3.64091E-05	3.35491E-05	1.65313E-05	0.000025762	2.40427E-05	1.73213E-05
Candidatus_Saccharimonas_aalborgensis	3.46855E-05	1.56364E-06	1.45636E-05	2.57245E-05	2.25585E-05	1.83769E-07	2.48369E-05	2.75969E-05	2.17373E-05	2.55545E-05	3.77545E-05	3.58727E-05	0.000018148	0.000027716	0.00002894	2.00273E-05
Flexistipes_sinusarabici	1.07227E-06	9.29091E-08	2.07636E-07	6.58909E-07	1.24769E-06	2.68769E-07	3.06231E-07	5.93231E-07	1.06764E-06	9.97727E-07	1.17836E-06	9.89727E-07	9.874E-07	1.2042E-06	1.3746E-06	0.000001227
Geovibrio_thiophilus	0.000003919	7.30182E-07	1.27091E-06	3.91973E-06	4.29815E-06	8.05538E-07	2.60331E-06	1.80485E-06	2.18082E-06	2.77145E-06	2.99145E-06	2.97673E-06	2.67547E-06	4.50933E-06	4.18367E-06	3.42307E-06
Deferribacter_desulfuricans	5.43909E-06	1.44727E-07	4.10636E-07	6.83636E-08	7.65369E-06	2.61538E-07	0.000002451	1.82085E-06	2.28473E-06	4.21773E-06	4.16227E-06	3.44518E-06	4.6408E-06	4.74347E-06	6.65867E-06	6.2954E-06
Denitrovibrio_acetiphilus	6.27145E-06	9.21364E-07	0.000000463	1.12064E-06	1.10178E-05	2.06554E-06	0.000002112	7.81077E-07	3.02627E-06	1.41645E-06	3.66545E-06	2.65473E-06	7.42947E-06	7.9762E-06	7.83987E-06	6.2472E-06
Calditerrivibrio_nitroreducens	0.00000951	7.66818E-08	2.66636E-07	1.40818E-07	2.30877E-05	0.000000285	1.39454E-06	1.29238E-06	0.000001742	2.65582E-06	3.30155E-06	1.33709E-06	0.00001609	1.43623E-05	1.70769E-05	1.15647E-05
Sneathia_amnii	3.78182E-07	3.36773E-07	4.63091E-07	1.96273E-07	1.26385E-07	2.95385E-08	0.000000714	0.000000323	4.30636E-07	5.45545E-07	9.19727E-07	7.93545E-07	0.000000237	3.682E-07	5.78133E-07	3.784E-07
Sebaldella_termitidis	2.76173E-06	2.24436E-06	1.27557E-06	2.37118E-06	0.000002118	2.15154E-06	0.000002511	1.64985E-06	2.36927E-06	2.08345E-06	2.62691E-06	3.84882E-06	1.33233E-06	1.46913E-06	1.92913E-06	1.53413E-06
Streptobacillus_moniliformis	9.77455E-07	1.91818E-08	3.34582E-07	1.11636E-06	3.07846E-06	4.48769E-07	1.54685E-06	2.67731E-06	5.29127E-06	2.73982E-06	3.51564E-06	0.00000241	3.5412E-06	2.49073E-06	3.3174E-06	2.06487E-06
Pseudoleptotrichia_goodfellowii	2.02736E-06	1.86864E-07	1.85036E-07	3.04818E-07	1.44585E-06	5.31692E-07	2.75615E-07	1.63538E-07	1.79245E-06	1.60755E-06	1.91473E-06	1.80227E-06	2.83833E-06	2.17573E-06	2.26373E-06	1.5864E-06
Leptotrichia_hongkongensis	1.67818E-07	9.99091E-08	1.15182E-07	2.45518E-07	0	2.95385E-08	2.08154E-07	4.69462E-07	1.22455E-07	2.02818E-07	1.27182E-07	3.21818E-07	6.37333E-08	1.25067E-07	1.712E-07	2.516E-07
Leptotrichia_buccalis	5.92727E-08	9.36364E-08	1.97818E-07	2.137E-07	5.23769E-07	0	7.45385E-08	2.33769E-07	0.000000198	2.64636E-07	6.86273E-07	0.00000105	1.99867E-07	1.26067E-07	2.942E-07	5.71667E-07
Leptotrichia_sp._oral_taxon_498	1.26455E-06	1.72236E-06	6.18364E-07	0.000000419	9.67077E-07	9.67154E-07	1.69308E-06	9.31538E-07	7.39545E-07	1.13818E-06	8.26182E-07	9.89727E-07	1.0158E-06	8.762E-07	1.34787E-06	5.724E-07
Leptotrichia_hofstadii	2.17909E-07	6.34182E-07	1.97636E-07	0.000000137	8.36154E-08	9.46154E-09	0	2.46923E-08	5.73091E-07	4.20455E-07	4.19091E-07	2.62909E-07	0.000000112	2.472E-07	8.23333E-08	1.93133E-07
Leptotrichia_shahii	9.02727E-07	6.49091E-07	1.27273E-07	4.78727E-07	1.14977E-06	3.83077E-08	7.03385E-07	0.000000503	3.95727E-07	3.88091E-07	6.74545E-07	3.06582E-06	6.424E-07	6.912E-07	1.4128E-06	1.05433E-06
Leptotrichia_wadei	4.00818E-07	6.14273E-07	7.97091E-08	5.16455E-07	3.35692E-07	1.09846E-07	5.96846E-07	6.78769E-07	5.11091E-07	5.07545E-07	0.000001291	6.10636E-07	3.03667E-07	6.28867E-07	3.81933E-07	4.69867E-07
Leptotrichia_trevisanii	1.57945E-06	2.08273E-07	1.76455E-07	1.07018E-06	4.92662E-06	1.88077E-07	1.53854E-06	0.000002378	7.22464E-06	3.11727E-06	4.39491E-06	2.94945E-06	4.78453E-06	3.49453E-06	3.92673E-06	2.38547E-06
Leptotrichia_sp._oral_taxon_847	1.51909E-07	1.04545E-08	7.39455E-08	4.20909E-08	3.99231E-07	0	4.33846E-08	1.92154E-07	1.11364E-07	1.48091E-07	2.42909E-07	1.44636E-07	1.85467E-07	7.558E-07	3.164E-07	3.69333E-07
Leptotrichia_sp._oral_taxon_212	2.32536E-06	0.000000108	1.83173E-06	5.80418E-06	2.62962E-06	2.24846E-07	9.80846E-07	1.59877E-06	7.69364E-06	7.63364E-06	6.23091E-06	5.00909E-06	3.7914E-06	3.6434E-06	4.44933E-06	0.000003464
Ilyobacter_polytropus	2.04227E-06	1.13109E-06	9.18182E-07	2.16173E-06	4.40385E-07	2.69154E-07	0.000000814	1.05192E-06	9.58636E-07	9.48273E-07	1.28209E-06	1.28609E-06	0.000000714	7.61933E-07	1.0566E-06	1.62593E-06
Fusobacterium_periodonticum	2.70909E-08	2.11455E-07	1.77273E-08	1.03455E-07	3.58462E-08	4.44923E-07	5.74615E-08	0.000000042	5.97273E-08	2.87273E-08	3.33636E-08	1.19091E-07	0	0	0	0
Fusobacterium_sp._oral_taxon_203	8.81818E-08	0	1.19164E-07	0	6.73077E-08	2.51615E-07	4.63077E-07	5.19538E-07	2.14273E-07	4.84909E-07	3.70545E-07	7.32182E-07	0.00000019	6.61267E-07	2.142E-07	2.56933E-07
Fusobacterium_necrophorum	1.07964E-06	5.42545E-07	6.14727E-07	5.71182E-07	1.26338E-06	5.64308E-07	0.000001602	8.31923E-07	1.04336E-06	1.77455E-06	1.50991E-06	2.56209E-06	6.50733E-07	1.1678E-06	1.76367E-06	9.464E-07
Fusobacterium_pseudoperiodonticum	3.16636E-07	0.000000128	4.12727E-09	1.51545E-07	1.59769E-07	5.91538E-08	1.74538E-07	4.82385E-07	3.46182E-07	4.24273E-07	3.36273E-07	1.26091E-07	0.000000316	4.61933E-07	3.682E-07	4.802E-07
Fusobacterium_gonidiaformans	5.80909E-07	1.02318E-07	3.53364E-07	5.09818E-07	0.000000537	1.83385E-07	5.01923E-07	1.32754E-06	4.23182E-07	8.64091E-07	8.86182E-07	1.17609E-06	0.000000542	8.10533E-07	1.04067E-06	1.03987E-06
Fusobacterium_mortiferum	9.90545E-07	7.92045E-07	4.23636E-07	9.55273E-07	1.35608E-06	3.61385E-07	1.01238E-06	1.95462E-06	4.09091E-07	3.95273E-07	1.08045E-06	1.21673E-06	1.83273E-06	2.10693E-06	1.97627E-06	3.17347E-06
Fusobacterium_hwasookii	1.39127E-06	5.84545E-07	2.96364E-07	2.26455E-07	1.13877E-06	2.95385E-08	1.33077E-07	1.43308E-07	3.28545E-07	1.83545E-07	1.43182E-07	1.24245E-06	4.268E-07	1.1236E-06	9.81667E-07	4.638E-07
Fusobacterium_varium	2.33936E-06	8.16273E-07	1.27436E-06	2.08191E-06	0.000000745	1.51923E-07	7.62231E-07	1.65138E-06	2.28736E-06	3.22036E-06	2.08591E-06	2.51755E-06	8.416E-07	1.3682E-06	1.38387E-06	9.604E-07
Fusobacterium_sp._NSJ-57	1.18236E-06	2.08182E-08	4.94545E-08	8.34636E-07	3.02315E-06	0	9.71462E-07	1.82223E-06	4.15809E-06	1.92664E-06	2.51918E-06	2.21873E-06	0.000002526	2.03873E-06	2.75773E-06	2.69567E-06
Fusobacterium_ulcerans	2.79291E-06	4.15091E-07	8.76727E-07	1.06773E-06	2.26723E-06	3.89231E-07	1.26023E-06	1.75623E-06	1.37827E-06	1.45855E-06	2.88882E-06	1.75291E-06	2.02273E-06	0.000002398	3.53647E-06	3.14073E-06
Fusobacterium_nucleatum	4.40091E-06	2.27409E-06	3.37145E-06	1.01055E-05	1.41662E-05	1.16562E-06	8.16538E-06	1.18585E-05	3.27182E-06	0.00000629	8.76182E-06	1.00836E-05	1.74147E-05	0.000017176	0.000022996	0.000027474
Hydrogenophilus_thermoluteolus	8.61364E-07	9.36364E-09	5.64436E-07	6.69909E-07	3.51077E-07	3.85692E-06	1.11685E-06	1.10954E-06	3.61909E-07	7.09545E-07	4.45545E-07	0.000000483	6.756E-07	4.976E-07	1.04833E-06	7.05133E-07
Mariprofundus_aestuarium	7.50545E-07	2.70909E-08	1.63636E-08	1.86818E-07	1.39077E-07	5.28462E-08	5.36923E-08	0	2.27636E-07	0.000000115	2.66545E-07	2.21636E-07	2.14E-08	4.67333E-08	4.82E-08	1.03067E-07
Mariprofundus_ferrinatatus	1.05809E-06	0.000000065	1.09782E-06	1.00709E-06	3.94308E-07	3.72538E-07	3.15385E-07	0.000000217	1.18127E-06	2.65364E-07	1.11782E-06	7.07273E-07	4.35867E-07	6.57067E-07	4.48533E-07	5.10667E-07
Silvanigrella_aquatica	4.13818E-07	0	9.80909E-07	2.42636E-07	1.14615E-07	0	1.34362E-06	1.93246E-06	1.60818E-07	9.11818E-08	2.74455E-07	3.71091E-07	9.14667E-08	1.34733E-07	2.51933E-07	1.47733E-07
Bacteriovorax_stolpii	2.95755E-06	0.00000013	1.23082E-06	2.44091E-06	7.32308E-07	1.77462E-06	6.01769E-07	8.07692E-07	1.90436E-06	7.10182E-07	2.02536E-06	1.40073E-06	5.45667E-07	5.42667E-07	9.09667E-07	1.1138E-06
Halobacteriovorax_sp._BALOs_7	9.02182E-07	0	7.40909E-08	3.79182E-07	3.76385E-07	0	0	3.06615E-07	3.32545E-07	2.17273E-07	1.65082E-06	4.92727E-07	2.582E-07	2.86733E-07	7.49933E-07	5.59667E-07
Halobacteriovorax_marinus	2.23091E-07	0	1.314E-07	1.01364E-07	0	0	0	1.12308E-07	5.48182E-08	0.000000081	0.000000169	7.69091E-08	0	1.206E-07	9.33333E-08	4.76667E-08
Bdellovibrio_sp._ZAP7	2.96364E-08	0	0	0	7.72308E-08	1.48385E-07	0	2.51538E-07	0	6.08182E-08	0	7.13636E-09	2.64E-08	0.000000033	0	0
Bdellovibrio_sp._NC01	5.20909E-08	2.00591E-07	3.27273E-08	1.51545E-08	0	0	4.33846E-08	5.48462E-08	0	0.000000103	2.76364E-08	4.79091E-08	0	0	0	9.33333E-09
Bdellovibrio_exovorus	7.85455E-08	9.72727E-07	1.63636E-08	8.32727E-08	2.27923E-07	0	2.63077E-08	1.90769E-08	1.46636E-07	1.03182E-07	4.36364E-08	1.63818E-07	1.612E-07	6.39333E-08	3.64467E-07	1.86E-08
Bdellovibrio_bacteriovorus	8.21091E-07	0.000000418	1.93773E-07	3.18273E-07	1.79154E-07	1.30862E-06	8.87154E-07	8.14077E-07	1.04927E-06	0.000000581	6.00364E-07	8.41545E-07	2.50667E-07	2.11867E-07	5.788E-07	8.342E-07
Bdellovibrio_sp._KM01	2.12727E-07	8.18182E-08	3.70909E-08	2.39091E-08	4.02462E-07	7.44308E-07	2.43692E-07	1.16531E-06	9.43091E-07	4.21364E-07	5.40455E-07	1.80568E-06	1.1058E-06	6.342E-07	3.54133E-07	1.4046E-06
Acidithiobacillus_ferrooxidans	5.57273E-08	0	0	0	0	3.15385E-08	0	0	9.77273E-08	7.55455E-08	2.64545E-07	1.235E-07	0	0.000000014	4.83067E-08	0
Acidithiobacillus_ferrivorans	6.25182E-07	0	1.48318E-06	2.09673E-06	6.46923E-07	1.51538E-07	4.16615E-06	0.000005061	1.01736E-06	6.11636E-07	9.02727E-07	7.79636E-07	4.94667E-07	6.11267E-07	1.00007E-06	5.04067E-07
Acidithiobacillus_caldus	0	2.84545E-08	1.66364E-08	9.54545E-09	8.51538E-08	3.83077E-08	2.54615E-07	5.79231E-08	0	5.22727E-08	4.98182E-08	9.75E-08	9.66667E-08	2.952E-07	5.77333E-08	2.16667E-07
Acidithiobacillus_thiooxidans	4.21636E-07	6.61636E-07	3.81091E-07	7.18909E-07	0.000000176	0	4.65692E-07	2.30077E-07	2.49091E-07	0.000000334	2.79545E-07	4.23818E-07	3.296E-07	3.34067E-07	5.808E-07	2.49267E-07
Candidatus_Nasuia_deltocephalinicola	5.41818E-08	0	0	0	0	2.52308E-08	0	0	0	0	0	0	4.79333E-08	0	0	4.04E-08
Candidatus_Profftella_armatura	2.96364E-08	0	0	4.37273E-08	0.000000077	0	0	1.09462E-07	0.000000089	7.02727E-08	0	0.000000035	0	0.000000033	5.45733E-08	3.65333E-08
Candidatus_Accumulibacter_phosphatis	8.25455E-08	0	3.70909E-08	1.56182E-07	0	0.00000016	2.65846E-07	9.03846E-08	1.63636E-07	2.12545E-07	6.68182E-08	0.000000233	4.50667E-08	1.61333E-08	1.14733E-07	1.66667E-08
Casimicrobium_huifangae	3.06273E-07	0	3.70909E-08	4.34545E-08	3.28538E-07	2.22638E-06	0.000000658	2.17854E-06	5.40909E-07	2.48364E-07	2.36727E-07	6.08955E-07	5.762E-07	4.80867E-07	6.53267E-07	8.25467E-07
Candidatus_Kinetoplastibacterium_galatii	0	8.63182E-08	0	0	0	1.91077E-07	0	0	0	0	0	0	0	0	0	0
Candidatus_Kinetoplastibacterium_crithidii	0	0	0	0	0	2.52308E-08	0	0	0	0	0	7.13636E-09	0	0	0	0
Candidatus_Kinetoplastibacterium_sorsogonicusi	2.96364E-08	0	0	6.32727E-08	0	0	4.33846E-08	0	0	0	0	0	2.72E-08	0	0	0
Candidatus_Kinetoplastibacterium_oncopeltii	0	0	0	0	1.26462E-07	0	0	0	0	0	0	0	1.33133E-07	1.07067E-07	1.2724E-07	7.25333E-08
Candidatus_Kinetoplastibacterium_desouzaii	6.06364E-07	0.000000065	1.10909E-07	2.71455E-07	1.39438E-06	8.57769E-06	2.33108E-06	2.34846E-06	1.06409E-06	1.01409E-06	1.33736E-06	0.000001427	1.89787E-06	1.8342E-06	1.80133E-06	0.000002598
Dechloromonas_aromatica	0.000000153	2.30818E-07	1.63636E-08	1.44091E-07	1.26308E-07	3.25923E-07	3.27231E-07	4.01385E-07	2.46455E-07	1.81636E-07	2.92636E-07	4.05909E-07	1.572E-07	9.90667E-08	2.30467E-07	0.000000404
Dechloromonas_sp._HYN0024	3.91273E-07	0.000000065	1.08027E-06	0.000001043	2.83769E-07	0	2.78462E-06	3.17623E-06	4.73909E-07	2.70727E-07	4.31636E-07	2.41182E-07	2.25867E-07	2.25333E-07	2.60333E-07	3.34467E-07

Aromatoleum_aromaticum	9.27364E-07	0.00000013	0.00000024	1.07182E-06	5.85385E-08	5.96923E-08	0.000000214	3.81538E-07	4.81727E-07	3.97364E-07	2.33091E-07	6.99182E-07	4.46667E-08	1.89267E-07	1.30333E-07	1.802E-07
Oryzomicrobium_terrae	3.00727E-06	0.000000644	1.93509E-06	1.58109E-06	6.14846E-07	1.02462E-07	1.26285E-06	0.000001915	1.11791E-06	7.50818E-07	0.000001369	0.000002216	4.748E-07	6.33067E-07	7.41333E-07	5.07133E-07
Dechlorosoma_suillum	0	0	0	0	0	0	0	5.81538E-08	5.79091E-08	0	3.33636E-08	1.23091E-07	0	0	0	0
Azospira_sp._I09	6.08182E-08	0	0	0	0	4.33846E-08	0.000000057	5.22308E-08	0	1.17273E-07	1.61727E-07	0.000000035	4.86E-08	0	4.46667E-08	7.08E-08
Thauera_chlorobenzoica	3.69091E-08	2.70909E-08	0	4.79091E-09	0	0	0	8.62308E-08	0	0	0	6.42727E-08	0	0	0	0
Thauera_hydrothermalis	0	0	0	3.55182E-08	2.59231E-08	5.96923E-08	0	0	0	0	0	7.13636E-09	0	1.71333E-08	6.75733E-08	0
Thauera_sp._K11	0	0	8.75455E-09	5.98182E-08	6.76923E-08	2.13846E-08	0	0	5.34545E-08	0	6.88182E-08	7.89091E-08	5.08667E-08	6.26E-08	1.49333E-08	5.07333E-08
Thauera_humireducens	0	6.35455E-08	3.39091E-08	4.94545E-08	5.50769E-08	4.13846E-08	1.18846E-07	8.22308E-08	3.75455E-08	3.03636E-08	1.74545E-07	6.35455E-08	2.94667E-08	2.38E-08	1.12667E-08	1.48067E-07
Thauera_sp._MZ1T	4.09455E-06	9.83182E-07	3.68344E-06	0.000003103	5.58231E-07	1.41769E-07	3.53038E-06	3.03415E-06	0.000001	1.61264E-06	2.08782E-06	2.52227E-06	0.000000363	5.73533E-07	5.20307E-07	3.89067E-07
Thauera_aromatica	1.71091E-07	0.000000065	0	0.000000155	8.63923E-07	3.96154E-08	0	4.19231E-07	7.87818E-07	4.97182E-07	7.60273E-07	3.60909E-07	7.20133E-07	0.000000909	0.000000803	8.70267E-07
Azoarcus_pumilus	8.95455E-08	2.34545E-08	0	9.97273E-09	0.000000205	1.18231E-07	0	1.90769E-08	2.16364E-08	0	2.76364E-08	1.77636E-07	2.24E-08	1.26067E-07	4.44E-09	1.58933E-07
Azoarcus_sp._DD4	1.94182E-07	0	0	1.35273E-07	3.34615E-08	0	4.33846E-08	1.90769E-08	1.11364E-07	0	6.65455E-08	4.70909E-07	7.76E-08	2.38E-08	1.62667E-07	0.00000008
Azoarcus_sp._KH32C	0.0000002	0	3.70909E-08	4.79091E-09	9.62308E-08	8.27692E-08	4.71538E-07	1.05715E-06	7.56364E-08	0	1.22091E-07	1.44091E-07	8.72667E-08	0.000000198	4.49067E-08	5.19333E-08
Azoarcus_sp._M9-3-2	1.14636E-07	3.28182E-08	4.12727E-09	3.87273E-08	1.16846E-07	2.25615E-07	6.44615E-08	1.10385E-07	2.79455E-07	2.38545E-07	2.25636E-07	7.71364E-08	6.65333E-08	8.33333E-08	4.04667E-08	7.84667E-08
Azoarcus_sp._DN11	1.80909E-07	0.000000121	4.12727E-09	1.47273E-08	1.25769E-07	9.59462E-07	6.06923E-08	7.26846E-07	1.16182E-07	0	2.96727E-07	0.000000268	9.18E-08	1.08933E-07	4.81467E-07	2.532E-07
Azoarcus_sp._CIB	1.65273E-07	0	4.12727E-09	1.34609E-07	3.88462E-08	1.78154E-07	1.21923E-07	7.15385E-08	0.000000067	9.11818E-08	1.08364E-07	1.02364E-07	6.08E-08	1.61333E-08	1.93733E-08	9.33333E-09
Azoarcus_communis	5.34909E-07	1.75136E-06	1.40682E-06	7.27182E-07	0.000000573	3.78462E-08	6.23077E-08	6.84615E-08	6.47273E-07	2.83364E-07	1.10627E-06	1.67973E-06	2.49533E-07	5.90467E-07	4.38867E-07	0.000000746
Azoarcus_olearius	4.80364E-06	2.07064E-06	5.49512E-06	4.68455E-06	7.92692E-07	0.000000086	7.56485E-06	7.65431E-06	2.31464E-06	2.81245E-06	2.81018E-06	3.64464E-06	1.25627E-06	8.524E-07	1.6412E-06	1.09113E-06
Thiobacillus_denitrificans	1.13727E-07	0	0	4.40909E-08	9.33846E-08	0	1.12077E-07	9.69231E-08	0.000000054	2.08818E-07	1.02727E-08	1.04364E-07	9.22667E-08	7.45333E-08	2.90667E-08	6.17333E-08
Sulfuritortus_calidifontis	7.19545E-07	4.00909E-08	2.18545E-08	2.58455E-07	2.80231E-07	3.12462E-07	8.26923E-07	4.59538E-07	6.75455E-07	0.00000076	4.38909E-07	1.06409E-06	1.49267E-07	1.67467E-07	4.44933E-07	1.684E-07
Methylophilus_medardicus	0	0	0	4.68818E-08	0	0	0	2.47692E-08	0	0	0	0	0	0	0	0
Methylophilus_sp._TWE2	9.39091E-08	0	2.47273E-08	9.54545E-09	6.16923E-08	0	3.11538E-08	7.55385E-08	4.45455E-08	0	0	1.08773E-07	0	0	2.97333E-08	6.86667E-08
Methylovorus_glucosotrophus	3.62727E-08	0	3.70909E-08	1.84909E-07	0	7.66154E-08	2.63077E-08	1.71462E-07	2.15182E-07	5.22727E-08	2.43455E-07	3.37545E-07	7.20667E-08	1.61333E-08	7.09733E-08	1.19933E-07
Methylovorus_sp._MP688	0.00000006	0	6.74909E-08	4.79091E-09	4.75385E-08	0	0	0	1.19091E-07	3.89091E-08	7.68182E-08	8.16364E-08	0.000000027	0.000000033	5.04667E-08	3.67333E-08
Candidatus_Methylopumilus_turicensis	0	0	0	0	0	0.000000026	0	0	0	0	0	0	0	0	0	0
Candidatus_Methylopumilus_rimovensis	8.32727E-08	0	3.27273E-08	5.46364E-08	3.34615E-08	2.91385E-07	3.33077E-08	7.39231E-08	0	0	0	7.13636E-09	4.45333E-08	1.926E-07	2.70507E-07	8.92667E-08
Candidatus_Methylopumilus_planktonicus	2.43636E-07	0	1.63091E-07	4.84273E-08	1.23462E-07	4.55769E-07	3.86923E-07	2.13923E-07	2.75455E-08	1.81818E-07	5.52727E-08	1.90273E-07	1.49333E-07	7.32E-08	1.80333E-07	3.212E-07
Candidatus_Methylopumilus_universalis	5.70636E-07	7.79273E-07	3.24273E-07	2.64545E-07	9.83846E-08	1.00908E-06	3.85769E-07	4.20923E-07	3.02909E-07	0.000000109	6.10545E-07	5.58455E-07	1.404E-07	2.46067E-07	0.000000264	2.66867E-07
Methylobacillus_flagellatus	1.41455E-07	0	4.89891E-07	4.28909E-07	2.28077E-07	0	2.33231E-07	2.60154E-07	1.71636E-07	1.24727E-07	2.41364E-07	8.42727E-08	2.09867E-07	1.55667E-07	1.3204E-07	2.26867E-07
Methylotenera_versatilis	0	0	0	0	0	0	0	0	6.50909E-08	0.000000068	1.02727E-08	1.33182E-07	2.64E-08	0	0.000000139	3.67333E-08
Methylotenera_mobilis	1.38545E-07	0	1.27273E-07	2.43636E-08	1.37385E-07	8.87308E-07	0.000000485	6.61154E-07	2.28455E-07	5.70909E-08	1.10909E-07	1.82318E-07	8.85333E-08	1.97667E-07	4.098E-07	5.23067E-07
Candidatus_Nitrotoga_sp._AM1P	2.77545E-07	0	0	3.07545E-07	6.76923E-08	0	0	0	1.18545E-07	9.97273E-08	4.10727E-07	8.29182E-07	2.09333E-08	2.15467E-07	0	5.83333E-08
Ferriphaselus_amnicola	0	0	3.70909E-08	1.00273E-07	4.11308E-07	5.74615E-07	1.82231E-07	2.77692E-07	1.71182E-07	2.74273E-07	2.15727E-07	7.33182E-07	1.202E-07	2.40867E-07	4.154E-07	2.38067E-07
Gallionella_capsiferriformans	0.000000324	0	2.70909E-07	6.70909E-08	0.000000074	3.83077E-08	1.41923E-07	1.23923E-07	2.04545E-07	3.46909E-07	0	0.00000007	2.26667E-07	1.40267E-07	0.000000244	9.26667E-08
Sideroxydans_lithotrophicus	1.58809E-06	0.00000013	3.04545E-06	2.79273E-06	1.49431E-06	1.29808E-06	9.10238E-06	8.48977E-06	2.03645E-06	1.80027E-06	2.16082E-06	3.63091E-06	1.0418E-06	1.36347E-06	2.52653E-06	1.50933E-06
Sulfuricella_denitrificans	1.91727E-07	2.34545E-08	3.70909E-08	8.43636E-08	3.58462E-08	2.79385E-07	8.48462E-08	5.66923E-08	1.08364E-07	6.69091E-08	0.000000093	2.48955E-07	0	1.18667E-07	1.07867E-07	1.35667E-07
Sulfuriferula_sp._AH1	4.00636E-07	5.74545E-08	3.70909E-08	0.00000025	6.00769E-08	4.45615E-07	8.87692E-08	5.84923E-07	1.73364E-07	0.000000081	4.21273E-07	1.13773E-07	2.91333E-08	2.38E-08	1.38107E-07	3.20267E-07
Sulfuriferula_nivalis	2.16545E-07	0	5.34545E-08	1.75064E-07	0.000000194	1.89231E-08	1.24846E-07	3.16769E-07	1.74818E-07	8.02727E-08	1.05545E-07	2.73364E-07	1.73533E-07	8.10667E-08	2.04933E-07	3.08E-08
Sulfuriferula_plumbiphila	2.97909E-07	2.47273E-07	5.93364E-08	1.34064E-07	3.63538E-07	6.47923E-07	0.000000596	8.12154E-07	2.48182E-07	0.0000001	4.92364E-07	4.93182E-07	2.48933E-07	2.66267E-07	5.22267E-07	3.10933E-07
Sulfurimicrobium_lacus	2.18364E-07	0.000000065	7.40909E-08	1.93364E-07	3.27231E-07	6.76077E-07	3.40769E-07	1.68769E-07	3.66909E-07	6.72636E-07	4.14182E-07	1.10536E-06	3.75733E-07	6.812E-07	3.8064E-07	7.30067E-07
Methyloversatilis_sp._RAC08	0	0	0	3.99091E-08	3.58462E-08	0	0	0	0	0.000000047	6.88182E-08	0	0	0.000000014	4.82E-08	0
Sterolibacterium_denitrificans	1.45727E-07	1.68727E-07	2.44182E-07	6.38182E-08	1.74538E-07	2.52308E-08	0	2.47692E-08	0	0	6.88182E-08	0	1.44133E-07	1.69533E-07	8.26E-08	1.44933E-07
Sulfuritalea_hydrogenivorans	3.35345E-06	1.05009E-06	8.20909E-07	2.25545E-07	1.01908E-06	1.23385E-07	2.04408E-06	1.46923E-06	4.78909E-07	5.90091E-07	9.81091E-07	2.04855E-06	7.00667E-08	1.75467E-07	1.3084E-07	1.35467E-07
Denitratisoma_oestradiolicum	6.75455E-07	1.95136E-07	2.70727E-07	6.18273E-07	1.87385E-07	1.65385E-07	2.50846E-07	1.86923E-07	3.52818E-07	2.02636E-07	1.27818E-07	2.05636E-07	1.33667E-07	1.99067E-07	1.71733E-07	8.16E-08
Denitratisoma_sp._DHT3	1.73418E-06	2.30591E-07	7.45855E-07	4.11909E-07	2.73131E-06	0	1.12031E-06	2.20438E-06	4.61973E-06	2.74573E-06	9.85727E-07	2.13818E-06	3.97467E-06	1.60987E-06	2.54893E-06	4.42133E-06
Nitrosospira_briensis	1.29455E-07	8.76818E-08	2.08182E-07	2.06482E-06	2.67846E-07	4.66769E-07	1.15854E-06	3.06569E-06	9.49091E-08	1.77545E-07	1.51545E-06	1.51727E-06	3.188E-07	3.42733E-07	5.57267E-07	4.168E-07
Nitrosospira_lacus	0	0	4.12727E-09	1.93636E-08	0	0	0	0	1.73636E-07	5.73636E-08	0	1.42727E-08	0	0.000000066	4.44E-09	1.22533E-07
Nitrosospira_multiformis	3.51636E-07	7.87727E-07	1.09285E-06	0.000000189	2.07923E-07	7.76154E-08	6.47462E-07	1.17723E-06	4.16909E-07	1.31727E-07	3.45091E-07	8.08364E-07	5.88667E-08	2.29133E-07	2.734E-07	4.57867E-07
Nitrosomonas_sp._Is79A3	3.69091E-08	0	0	5.18182E-09	0.000000039	0	2.63077E-08	0	0	8.26364E-08	6.88182E-08	1.37364E-07	0	2.38E-08	9.09333E-08	3.72E-08
Nitrosomonas_europaea	3.12455E-07	2.34545E-08	7.03182E-07	6.28727E-07	8.74615E-08	7.40846E-07	1.15031E-06	8.47231E-07	2.28545E-07	2.88909E-07	8.93636E-08	8.02727E-08	1.57867E-07	1.644E-07	2.11733E-07	1.41533E-07
Nitrosomonas_eutropha	2.94818E-07	0.000000603	1.45273E-07	1.51364E-07	0.000000077	1.75385E-07	3.10462E-07	9.89231E-08	0.000000196	0.000000163	5.97636E-07	3.45545E-07	7.48667E-08	0.00000007	1.50533E-07	1.22E-08
Nitrosomonas_sp._AL212	2.25482E-06	0	2.22727E-07	6.40909E-07	4.64462E-07	0	4.57615E-07	1.35054E-06	1.28191E-06	1.48045E-06	1.24136E-06	6.41909E-07	6.242E-07	6.31133E-07	1.2304E-06	6.65867E-07
Nitrosomonas_communis	8.30909E-07	1.93182E-08	1.90191E-07	4.51182E-07	6.69308E-07	4.63769E-06	1.42754E-06	2.48823E-06	8.37818E-07	4.48182E-07	1.06955E-06	1.16555E-06	7.842E-07	7.05333E-07	1.09267E-06	2.09033E-06
Nitrosomonas_stercoris	1.39145E-06	1.63545E-07	6.28182E-08	4.00909E-07	2.29792E-06	7.88608E-06	2.48138E-06	4.57154E-06	2.93545E-06	2.26545E-06	1.53709E-06	2.31027E-06	3.45067E-06	0.000002138	2.85913E-06	4.9444E-06
Nitrosomonas_ureae	0.000001139	9.21818E-08	1.31545E-07	1.17227E-06	4.19815E-06	2.03422E-05	4.77262E-06	7.29538E-06	2.73318E-06	1.70736E-06	3.65364E-06	2.99882E-06	4.7192E-06	4.43267E-06	6.83867E-06	6.93753E-06
Pseudogulbenkiania_sp._NH8B	2.71636E-07	0	0	6.14545E-08	3.34615E-08	1.18462E-07	3.11538E-08	4.88462E-08	0	0	0	9.85455E-08	4.61333E-08	0.000000028	2.01333E-08	5.58667E-08
Deefgea_sp._D17	7.81818E-08	0	0	1.91818E-08	2.75385E-08	0	4.33846E-08	0	8.67273E-08	0	3.77273E-08	9.71818E-08	4.24E-08	3.19333E-08	7.26667E-08	1.66667E-08
Microvirgula_aerodenitrificans	2.28909E-07	1.68182E-08	3.70909E-08	1.16873E-06	3.86308E-07	0	0.000000333	1.73385E-07	4.79636E-07	9.48727E-07	0.00000022	4.42091E-07	1.854E-07	8.56E-08	4.83307E-07	6.86E-08
Jeongeupia_sp._USM3	2.30909E-08	1.14045E-07	0	3.39091E-08	9.09231E-08	0	0	0	0	0.000000047	3.33636E-08	3.31818E-07	0	6.39333E-08	1.49333E-08	0
Aquitalea_denitrificans	0	0	0	0	0	0	0	0	0	0	0	0	0	0	5.94E-08	0
Aquitalea_sp._USM4	5.29091E-08	0	3.70909E-08	2.97273E-08	3.88462E-08	1.23846E-07	2.63077E-08	8.79231E-08	0.000000091	1.08273E-07	1.33364E-07	1.47636E-07	2.64E-08	4.98E-08	2.97333E-08	3.72E-08
Aquitalea_sp._THG-DN7.12	4.15909E-07	0	6.98182E-08	2.50182E-07	3.46154E-07	0	1.02231E-07	2.41538E-07	6.01182E-07	5.07455E-07	1.52727E-07	0.000000672	3.058E-07	4.11333E-07	3.17067E-07	5.83867E-07
Aquitalea_magnusonii	2.96364E-08	0	1.33636E-07	0	0	0	0	0	0	0.000000034	0	0	2.24E-08	1.58E-08	0	0
Chitinibacter_fontanus	1.18091E-07	0	1.33636E-07	2.06909E-07	4.33923E-07	0.000001011	4.24615E-07	3.27308E-07	1.78545E-07	1.41636E-07	2.06545E-07	9.27273E-08	3.002E-07	1.17467E-07	3.28867E-07	3.07667E-07
Chitinibacter_sp._2T18	9.45455E-08	0.000000065	0	4.02727E-08	6.03846E-08	0	0	4.27615E-07	0	1.33091E-07	0	2.14545E-08	1.68533E-07	0.000000102	3.89267E-07	2.91267E-07
Vogesella_sp._LIG4	4.94182E-07	1.02082E-06	9.04364E-08	6.80636E-07	5.47692E-08	2.25477E-06	1.41538E-07	3.14231E-07	8.20909E-08	0.000000317	4.06091E-07	3.18455E-07	7.46667E-08	1.07667E-07	1.28733E-07	3.024E-07
Laribacter_hongkongensis	6.80364E-07	0.000000065	1.064E-07	4.44545E-07	1.11615E-06	2.16923E-08	3.57923E-07	5.88692E-07	3.00364E-07	0.000000401	2.27455E-07	2.25545E-07	9.436E-07	1.01233E-06	8.26467E-07	9.17067E-07
Aquaspirillum_sp._LM1	0.000001058	9.78636E-07	1.02818E-07	2.27364E-07	5.42615E-07	1.57692E-07	3.08231E-07	1.73538E-07	1.04545E-06	6.17727E-07	2.24182E-07	9.27545E-07	6.824E-07	7.192E-07	4.29533E-07	0.00000034
Chromobacterium_rhizoryzae	0	0	0	0	0	0	0	2.46923E-08	0	0	0	0	0	1.58E-08	0	0
Chromobacterium_sp._IIBBL_112-1	0	0	0	3.24545E-08	0	0	0	0	0	0	0	0	0	0	0	0
Chromobacterium_sp._Rain0013	2.70909E-08	0	0	0	0	0	0	0	0	0	0	3.87727E-08	0	0	6.46667E-08	0
Chromobacterium_paludis	2.70909E-08	2.47273E-07	4.10909E-08	3.49091E-08	0	0	3.11538E-08	0	0	0.000000028	3.95455E-08	1.75727E-07	0	0	1.49333E-08	1.36067E-07
Chromobacterium_haemolyticum	1.58182E-08	0	0	3.59091E-08	0	0	0	0	5.79091E-08	7.55455E-08	1.99727E-07	1.22136E-07	0	3.50667E-08	0	0
Chromobacterium_sp._ATCC_53434	2.23091E-07	1.62545E-07	5.75818E-08	7.28182E-08	0	9.19231E-08	0	7.10769E-08	0	3.89091E-08	2.76364E-08	1.95909E-07	0	2.38E-08	4.83067E-08	3.72E-08
Chromobacterium_vaccinii	1.93091E-07	5.09273E-07	1.52273E-07	5.70091E-07	2.88462E-08	1.36308E-07	1.36538E-07	1.60769E-07	4.45455E-08	0	2.76364E-08	4.75364E-07	1.28333E-07	8.56E-08	5.94E-08	1.86667E-08
Chromobacterium_violaceum	4.92727E-07	4.87773E-07	1.92182E-07	2.13455E-07	9.22308E-08	7.21538E-08	4.22692E-07	2.39846E-07	5.34545E-08	0.000000383	3.89273E-07	6.87545E-07	4.58E-08	1.78267E-07	4.46667E-08	2.79333E-08
Iodobacter_fluviatilis	5.57273E-08	0	0	4.79091E-09	0	8.43846E-08	0.000000057	4.64615E-08	3.98182E-08	0	0	9.54545E-08	0	0	1.93733E-08	9.33333E-09
Iodobacter_sp._H11R3	8.94909E-07	4.54545E-07	8.63091E-08	8.15727E-07	3.06231E-07	0.000000136	8.72923E-07	3.89462E-07	5.85091E-07	2.18455E-07	4.65727E-07	1.99273E-07	2.33333E-07	6.22267E-07	3.36533E-07	4.08667E-07
Alysiella_filiformis	1.04736E-06	0.000000065	2.59091E-07	1.34364E-07	4.89846E-07	5.05385E-08	3.71538E-07	3.23462E-07	2.75455E-08	3.62727E-08	4.36364E-08	4.45273E-07	2.72E-08	9.93333E-08	1.94267E-07	9.53333E-08
Snodgrassella_alvi	1.17636E-07	1.18636E-07	2.96364E-08	8.88818E-08	0.000000169	2.37538E-07	0	1.90769E-08	2.21818E-07	2.66273E-07	1.94818E-07	5.21727E-07	1.61267E-07	0.00000007	2.572E-07	1.10067E-07
Simonsiella_muelleri	9.77273E-08	1.03636E-08	4.12727E-09	1.99091E-08	0	0	2.63077E-08	1.90769E-08	1.25909E-07	7.55455E-08	2.68818E-07	7.85455E-08	2.14E-08	0.000000033	5.96667E-08	0
Kingella_oralis	4.73455E-07	8.77273E-08	2.57727E-07	2.67455E-07	0	0	5.19385E-07	5.65846E-07	4.03273E-07	3.71545E-07	3.43818E-07	2.19727E-07	1.86667E-08	1.58E-08	7.51067E-08	5.90667E-08
Kingella_kingae	1.21818E-07	2.34545E-08	4.53455E-08	2.47364E-07	2.23769E-07	8.65077E-07	4.55769E-07	4.39077E-07	0.000000027	0.000000156	1.93818E-07	1.24364E-07	2.53067E-07	9.73333E-08	3.82133E-07	6.61867E-07
Crenobacter_cavernae	9.21818E-08	0	0	4.20909E-08	2.99615E-07	0	1.25154E-07	8.46923E-08	5.97273E-08	0	0	0	2.39933E-07	5.63333E-07	4.15133E-07	2.80067E-07
Conchiformibius_steedae	2.57455E-07	1.16364E-08	1.27273E-07	3.36818E-07	4.15462E-07	1.78462E-07	3.67385E-07	2.62923E-07	3.78727E-07	8.02727E-08	1.58545E-07	2.14364E-07	3.312E-07	3.19333E-07	1.74467E-07	3.59533E-07
Chitinolyticbacter_meiyuanensis	1.40455E-06	1.94545E-07	6.72545E-07	1.55618E-06	4.89615E-07	0	3.22615E-07	9.14385E-07	1.31709E-06	6.27273E-07	1.38418E-06	1.50991E-06	6.90933E-07	1.2344E-06	5.326E-07	7.41333E-07
Eikenella_exigua	0	0	0	1.93636E-08	0.000000054	1.93769E-07	0	0.000000011	2.16364E-08	8.02727E-08	1.02727E-08	1.42727E-08	5.86E-08	6.28E-08	0	2.278E-07
Eikenella_corrodens	2.52109E-06	0.000000292	2.23673E-07	2.54355E-06	4.67231E-07	1.00854E-06	1.83954E-06	2.05569E-06	1.48645E-06	1.33773E-06	1.87255E-06	2.32255E-06	0.000000639	8.42067E-07	1.07827E-06	8.77267E-07
Vitreoscilla_sp._C1	0	0	0	1.47273E-08	4.13846E-08	0	0	1.90769E-08	9.95455E-08	1.09545E-07	1.02182E-07	2.47364E-07	2.14667E-08	6.59333E-08	9.66E-08	2.66867E-07
Vitreoscilla_filiformis	1.89455E-06	9.45273E-07	2.83545E-07	9.77273E-07	0.000000524	3.42615E-06	9.07385E-07	1.01654E-06	6.16545E-07	9.43091E-07	1.60109E-06	0.000002035	4.57867E-07	5.58267E-07	5.79267E-07	6.89267E-07
Neisseria_sicca	0	0	0	0	0	0	3.33077E-08	0	0	0	0	3.94545E-08	0	0	0	0
Neisseria_cinerea	0	0	0	5.16364E-08	0	0	0	0	5.79091E-08	0	0	0	0	0	2.55333E-08	9.33333E-09
Neisseria_weaveri	3.11818E-08	0	0	0	0	0	0	0	0	0	0	0	0	0	0	0
Neisseria_flavescens	0	0.000000065	0	9.67273E-08	5.76154E-08	3.83077E-08	6.41538E-08	7.96154E-08	0	0	1.02727E-08	3.64818E-07	0	0	0	0
Neisseria_animalis	2.96364E-08	0	0	0.000000108	0	8.42769E-07	5.54615E-08	6.55385E-08	2.17273E-07	1.91545E-07	2.76364E-08	5.37273E-08	6.96E-08	6.59333E-08	8.404E-08	0
Neisseria_canis	0	0	0.000000046	0	2.05385E-08	0	0	0	0	0	0	0	0	3.09333E-08	5.94E-08	0
Neisseria_polysaccharea	0	8.65E-08	0.000000046	4.82727E-08	1.02154E-07	0	0	6.56615E-07	0	0	0	1.42727E-08	1.32667E-07	1.464E-07	7.21333E-07	4.91067E-07
Neisseria_subflava	2.82818E-07	1.93182E-08	1.54818E-07	2.52818E-07	2.09385E-07	0	2.50692E-07	1.36308E-07	8.00636E-07	8.32727E-08	6.83455E-07	3.67273E-07	7.22E-08	1.69467E-07	3.09933E-07	1.156E-07
Neisseria_mucosa	0.000000206	2.65455E-08	6.33636E-08	1.43636E-08	1.00769E-07	6.52692E-07	1.84308E-07	4.88154E-07	8.19091E-08	0.0000002	4.94636E-07	6.02545E-07	2.172E-07	1.16667E-07	8.97333E-08	3.834E-07
Neisseria_meningitidis	2.61636E-07	0.000000065	0	5.18182E-09	2.48692E-07	2.95385E-08	1.25846E-07	1.33769E-07	6.52636E-07	4.89364E-07	0.000000142	2.10455E-07	5.07267E-07	2.254E-07	2.62333E-07	7.716E-07
Neisseria_sp._oral_taxon_014	1.58182E-08	0.00000013	4.58455E-08	1.55455E-08	7.05385E-08	0	0	0	0	3.89091E-08	0	1.27818E-07	0	0.000000115	8.91333E-08	1.22E-08
Neisseria_animaloris	6.82091E-07	9.20909E-08	5.12855E-07	0.000000836	2.88462E-08	5.54077E-07	5.83769E-07	8.37385E-07	9.13182E-07	2.68364E-07	6.25909E-07	3.57455E-06	1.00507E-06	9.96E-08	1.37867E-07	2.54867E-07
Neisseria_zoodegmatis	4.54545E-08	0.000000065	4.12727E-09	2.43636E-08	0.000000065	1.44615E-07	0	4.66923E-08	0	0.000000047	6.68182E-08	3.57273E-08	0	0.000000075	0	4.02E-08
Neisseria_shayeganii	3.50455E-07	0.000000022	2.46182E-08	1.92273E-07	2.88462E-08	2.67077E-07	1.10385E-07	1.90769E-08	3.90727E-07	1.41818E-07	1.15727E-07	3.17091E-07	0	3.35333E-08	2.5424E-07	3.97333E-08
Neisseria_dentiae	5.16364E-07	0.000000168	4.49818E-07	6.93818E-07	3.41769E-07	0	8.33692E-07	5.52692E-07	6.11364E-07	8.00273E-07	8.41455E-07	6.73545E-07	3.72933E-07	1.99733E-07	2.53733E-07	5.11533E-07
Neisseria_elongata	2.04245E-06	7.91636E-07	0.000001025	2.64845E-06	3.17923E-07	6.82615E-07	1.67562E-06	0.00000092	8.29091E-07	1.15564E-06	1.87809E-06	1.98973E-06	1.936E-07	4.412E-07	3.686E-07	6.15867E-07
Neisseria_wadsworthii	5.42727E-08	0	0	1.57909E-07	2.21462E-07	7.21538E-08	7.40769E-08	0.000000406	1.04273E-07	1.29636E-07	1.16455E-07	2.56273E-07	7.80667E-08	8.38667E-08	1.1484E-07	1.268E-07
Neisseria_musculi	1.07736E-06	7.66364E-08	2.67345E-07	1.35173E-06	5.68769E-07	1.58638E-06	5.48769E-07	9.30615E-07	6.44818E-07	0.000000686	1.13745E-06	1.19036E-06	6.17333E-07	4.86467E-07	1.02627E-06	1.24667E-06
Neisseria_gonorrhoeae	4.29091E-08	5.41818E-08	0	9.00909E-08	2.01308E-07	2.31462E-07	0.000000149	8.05846E-07	1.45364E-07	3.33636E-07	1.00818E-07	1.64818E-07	1.30533E-07	2.46067E-07	1.1224E-07	1.198E-07
Neisseria_chenwenguii	2.48273E-07	4.86545E-07	0.000000107	4.80727E-07	2.43615E-07	6.91077E-07	3.82385E-07	4.77385E-07	5.69818E-07	0.000000387	3.16364E-07	0.000000301	2.688E-07	3.60067E-07	2.64867E-07	3.95467E-07
Neisseria_brasiliensis	5.41909E-07	1.48455E-07	0	9.54545E-09	6.69615E-07	0	0	1.79846E-07	0	1.32636E-07	0	9.03636E-08	7.63467E-07	5.73733E-07	9.11933E-07	1.11573E-06
Neisseria_zalophi	0.000001166	4.79182E-07	0.000000477	1.02936E-06	9.41692E-07	8.30769E-07	1.76931E-06	6.13462E-07	2.56173E-06	1.75618E-06	8.23091E-07	1.21991E-06	1.1432E-06	6.57133E-07	1.10967E-06	1.24407E-06
Neisseria_bacilliformis	9.21545E-07	0.000000065	1.63636E-08	5.60091E-07	7.12462E-07	0	3.58308E-07	1.32238E-06	6.41818E-07	0.00000068	0.000000988	1.20955E-06	7.654E-07	1.0258E-06	9.66467E-07	6.90867E-07
Neisseria_sp._10022	0.000000061	1.90727E-07	5.34545E-08	6.14273E-08	2.88462E-08	1.23531E-06	2.58154E-07	1.68846E-07	8.80909E-08	2.40909E-07	1.02727E-08	4.56909E-07	2.72E-08	4.88E-08	1.12667E-08	9.37333E-08
Neisseria_sp._KEM232	1.34773E-06	2.44909E-07	9.87091E-08	3.32545E-07	1.71162E-06	8.16923E-08	3.19846E-07	1.80692E-07	2.62909E-07	1.00791E-06	8.33455E-07	9.35636E-07	1.26093E-06	1.5406E-06	2.01347E-06	1.35687E-06
Neisseria_sp._ZJ785	1.02109E-06	5.51409E-07	3.19182E-07	1.84618E-06	7.74615E-07	1.40869E-06	2.17823E-06	2.43638E-06	1.31555E-06	5.92182E-07	1.16745E-06	1.14927E-06	5.35933E-07	5.47933E-07	5.79307E-07	3.30667E-07
Mitsuaria_sp._7	2.56364E-08	0	0	9.97273E-09	2.87692E-07	1.82615E-07	5.29154E-07	1.72154E-07	1.36364E-07	0.000000187	6.88182E-08	1.63182E-07	1.35733E-07	1.866E-07	1.70867E-07	1.04867E-07
Rhizobacter_gummiphilus	8.54545E-08	6.08182E-08	0	9.37E-08	6.00769E-08	1.35385E-07	8.66154E-08	1.39308E-07	7.62727E-08	0	8.86364E-08	1.42727E-08	3.74E-08	1.15467E-07	6.46667E-08	1.22E-08
Sphaerotilus_natans	1.59727E-07	1.38273E-07	0	3.74636E-07	7.84769E-07	2.95385E-08	0	0	8.41909E-07	2.82455E-07	6.31273E-07	6.57727E-07	7.62067E-07	7.32267E-07	9.54333E-07	9.73267E-07
Leptothrix_cholodnii	2.41545E-07	4.84636E-07	5.34545E-08	1.71091E-07	1.28231E-07	9.46154E-09	4.33846E-08	3.16538E-07	2.48727E-07	1.28091E-07	3.34636E-07	3.92455E-07	0	1.638E-07	3.27733E-07	1.86E-08
Inhella_inkyongensis	1.79873E-06	4.57182E-07	6.27309E-07	4.23909E-07	3.63923E-07	0	8.82077E-07	2.65846E-06	1.22436E-06	6.08091E-07	1.00191E-06	1.46018E-06	8.662E-07	6.27533E-07	8.34067E-07	1.79867E-06
Xylophilus_rhododendri	6.19091E-08	0	4.12727E-09	7.46364E-08	6.47692E-08	4.27692E-08	3.77692E-08	9.39231E-08	2.64545E-08	1.20818E-07	0.000000225	8.98182E-08	7.42E-08	5.64667E-08	1.052E-07	7.80667E-08
Paucibacter_sp._KCTC_42545	8.48364E-07	1.20436E-06	2.68982E-07	4.02091E-07	1.41923E-07	1.30769E-07	9.15385E-08	2.67769E-07	3.69364E-07	3.97273E-07	3.49909E-07	8.26909E-07	2.26867E-07	4.88E-08	2.41133E-07	1.84133E-07
Roseateles_depolymerans	1.75818E-07	2.41091E-07	1.54555E-07	1.15455E-07	1.60538E-07	0	0.000000119	8.66923E-08	1.46364E-07	2.76182E-07	1.54727E-07	1.81909E-07	1.81867E-07	6.49333E-08	5.93333E-08	3.65333E-08
Aquabacterium_olei	7.37273E-08	0	9.06364E-08	1.33364E-07	0.000000054	4.13846E-07	3.82462E-07	3.63154E-07	1.99818E-07	1.21727E-07	2.75364E-07	5.87091E-07	6.92E-08	2.078E-07	1.53867E-07	9.23333E-08
Methylibium_sp._Pch-M	2.56364E-08	0	1.63636E-08	0	8.91538E-08	0	4.80769E-08	0	2.16364E-08	0	0	6.41364E-08	0	3.48E-08	2.55333E-08	0
Methylibium_petroleiphilum	2.46818E-07	0	3.70909E-08	2.57182E-07	7.94615E-08	0	0	3.09692E-07	7.56364E-08	1.13636E-07	2.64545E-07	1.45955E-07	1.21933E-07	8.17333E-08	0.000000064	3.182E-07
Rubrivivax_gelatinosus	3.96091E-07	2.37455E-07	4.16636E-08	1.17818E-07	6.39692E-07	4.81615E-07	1.03623E-06	8.10462E-07	3.97182E-07	1.40327E-06	1.02655E-06	9.59455E-07	6.77133E-07	1.41667E-06	1.57253E-06	1.22527E-06
Thiomonas_arsenitoxydans	7.37273E-08	0	1.77273E-08	1.32182E-07	0	0	1.18077E-07	0	0	0	1.16455E-07	1.02364E-07	0	7.60667E-08	1.65333E-08	0.000000069
Thiomonas_sp._X19	0.000000607	0	1.87118E-06	2.20482E-06	6.34077E-07	1.23692E-07	5.08108E-06	5.64938E-06	6.74091E-07	7.23364E-07	6.21182E-07	4.60455E-07	0.000000387	2.48267E-07	6.07067E-07	4.696E-07
Thiomonas_intermedia	8.19545E-07	8.18182E-08	2.24818E-07	5.44818E-07	2.81846E-07	5.19231E-06	1.58092E-06	1.90023E-06	9.39364E-07	4.26455E-07	1.70536E-06	1.00686E-06	5.844E-07	6.564E-07	9.43067E-07	6.11467E-07
Sutterella_faecalis	4.98691E-06	4.00455E-07	2.59091E-07	1.45291E-06	1.13215E-05	5.71748E-05	1.58731E-05	2.20362E-05	1.08741E-05	7.13818E-06	1.14291E-05	1.14891E-05	1.44772E-05	1.02867E-05	0.000017406	2.13767E-05
Sutterella_megalosphaeroides	1.01609E-05	1.30709E-06	9.95091E-07	2.57845E-06	2.28054E-05	8.66597E-05	2.39562E-05	4.19231E-05	2.92864E-05	1.60636E-05	1.77618E-05	2.13727E-05	3.14233E-05	2.09647E-05	3.01973E-05	4.25228E-05
Kerstersia_gyiorum	0	0	0	0	0	0	0	0	0	0	0	0	0	0	0	4.95333E-08
Paenalcaligenes_hominis	2.70909E-08	2.34545E-08	0	0	2.59231E-08	0	0	0	1.64273E-07	3.03636E-08	2.76364E-08	0	7.18667E-08	0	2.09733E-08	3.84667E-08
Orrella_dioscoreae	3.11818E-08	0	0	0	0.00000007	0	2.63077E-08	0	0.000000081	5.22727E-08	6.55455E-08	0.00000005	2.64667E-08	0	4.44E-09	0
Basilea_psittacipulmonis	1.16636E-07	3.17091E-07	1.24182E-07	2.88364E-07	1.05154E-07	0	0	4.93846E-08	0.00000016	5.18455E-07	1.97455E-07	1.17682E-07	4.38E-08	0	0	3.31867E-07
Oligella_urethralis	0.000000107	0	0	6.13636E-08	5.45385E-08	1.18623E-06	6.12385E-07	8.05692E-07	3.77364E-07	1.15091E-07	9.60909E-08	3.08636E-07	1.17333E-07	4.32933E-07	3.488E-07	5.86733E-07

Pigmentiphaga_sp._H8	6.08182E-08	0.000000065	0	1.18182E-07	0	1.06923E-07	0	0.000000022	5.97273E-08	0.000000081	1.37727E-07	1.28091E-07	0	3.09333E-08	0	1.39067E-07
Pigmentiphaga_aceris	3.01455E-07	1.53364E-07	2.18545E-08	1.30636E-07	0	6.27692E-08	7.92308E-08	8.28462E-08	7.46364E-08	1.27273E-07	0	1.84364E-07	4.74667E-08	4.20667E-08	0	1.86E-08
Castellaniella_defragrans	2.08727E-07	9.36364E-09	0.000000172	5.01182E-07	4.13846E-08	2.37385E-07	0	8.44615E-08	5.79091E-08	0	2.76364E-08	1.41364E-07	2.64E-08	4.70667E-08	6.16667E-08	1.45267E-07
Algicoccus_marinus	2.14818E-06	2.08182E-08	4.27182E-07	3.07273E-08	4.29462E-07	3.26923E-07	1.38346E-06	1.48538E-06	2.09255E-06	0.000003362	2.31273E-06	0.000001572	2.298E-07	1.11133E-07	1.79933E-07	6.708E-07
Pelistega_sp._NLN63	1.28364E-07	0	1.06818E-07	1.01364E-07	9.04615E-08	6.25292E-06	1.05692E-07	7.02385E-07	1.77909E-07	6.74545E-07	1.09627E-06	8.82909E-07	2.084E-07	1.43267E-07	8.384E-07	3.99067E-07
Taylorella_equigenitalis	0.000001846	1.98182E-08	1.43545E-07	0	3.07615E-07	3.81077E-07	1.13131E-06	1.35977E-06	1.51436E-06	3.69591E-06	2.14745E-06	1.75655E-06	4.81067E-07	0.000000096	3.95307E-07	0.00000082
Taylorella_asinigenitalis	7.22909E-07	0	1.733E-07	5.01909E-07	0.000000199	7.60846E-07	9.97462E-07	8.65154E-07	1.05873E-06	6.14909E-07	4.95273E-07	5.40455E-07	6.594E-07	3.42533E-07	6.55067E-07	6.706E-07
Advenella_kashmirensis	4.54545E-08	0	3.70909E-08	0	2.75385E-08	0	5.48462E-08	1.48385E-07	6.49091E-08	8.88182E-08	3.33636E-08	1.97545E-07	6.70667E-08	1.48133E-07	5.54E-08	1.268E-07
Advenella_mimigardefordensis	4.82727E-07	5.52182E-07	2.13364E-07	4.69727E-07	7.80154E-07	8.37385E-07	8.18538E-07	7.09846E-07	9.20818E-07	4.04455E-07	1.02436E-06	3.21273E-07	3.192E-07	2.96667E-07	4.608E-07	5.06133E-07
Pusillimonas_sp._T7-7	1.58182E-08	0.00000013	5.32182E-08	9.54545E-09	0.000000054	6.59692E-07	5.16154E-07	4.88538E-07	8.65455E-08	1.91455E-07	1.88909E-07	2.58182E-07	1.33267E-07	7.71333E-08	1.94667E-07	1.32933E-07
Pusillimonas_sp._DMV24BSW_D	1.49173E-06	0.00000013	4.45182E-07	7.59909E-07	3.58923E-07	1.07908E-06	5.08923E-07	4.22385E-07	1.13773E-06	3.98636E-07	8.62636E-07	6.09091E-07	4.45733E-07	3.99267E-07	4.51467E-07	8.57467E-07
Pusillimonas_sp._ye3	1.41755E-06	4.335E-07	5.34545E-08	4.93182E-07	5.27308E-07	5.24769E-07	3.28923E-07	0.000000857	9.17182E-07	9.35182E-07	7.52182E-07	6.53182E-07	7.94933E-07	0.000000727	5.28333E-07	6.22467E-07
Bordetella_parapertussis	0	2.05E-08	0	0	0	0	0	0	0	0	0	0	0	0	0	0
Bordetella_pertussis	2.98182E-08	0	0	0	0	0	0	0	0	0	0	3.94545E-08	0	0	1.49333E-08	3.67333E-08
Bordetella_holmesii	2.30909E-08	0	3.70909E-08	9.54545E-09	3.15385E-08	2.13846E-08	7.92308E-08	3.37692E-08	0	0	3.95455E-08	8.87273E-08	0	3.09333E-08	2.39333E-08	1.31467E-07
Bordetella_bronchiseptica	1.59909E-07	0.000000065	1.63636E-08	2.90636E-07	2.70154E-07	2.03077E-07	1.28462E-07	1.16077E-07	2.19182E-07	1.86182E-07	0	4.21364E-08	2.83067E-07	3.24333E-07	0.000000134	4.78E-08
Bordetella_avium	0	0	2.96364E-08	3.24545E-08	0	2.13846E-08	3.11538E-08	0	2.16364E-08	0	0	0	1.86667E-08	0	0	0
Bordetella_pseudohinzii	1.54364E-07	0	0	5.71818E-08	1.94154E-07	9.48462E-08	5.74615E-08	1.29462E-07	1.25273E-07	0.00000013	1.82818E-07	6.01818E-08	1.42067E-07	0.000000047	1.626E-07	5.484E-07
Bordetella_bronchialis	2.29091E-07	3.35455E-08	1.63636E-08	5.42727E-08	1.48308E-07	0	6.96923E-08	4.07692E-08	1.35273E-07	7.25455E-08	3.33636E-08	2.29455E-07	1.02667E-07	8.58E-08	9.53067E-08	9.24E-08
Bordetella_hinzii	1.16545E-07	4.39091E-08	0	1.13636E-07	7.96923E-08	0	0	3.48538E-07	0.000000257	3.50636E-07	4.23636E-07	6.17364E-07	0.000000053	0	1.93733E-08	2.48933E-07
Bordetella_genomosp._13	2.43364E-07	0	0	1.83636E-07	2.03462E-07	1.21246E-06	3.01077E-07	2.02846E-07	1.98091E-07	5.22727E-08	4.60909E-07	5.91182E-07	0.000000106	1.94733E-07	2.3284E-07	1.48267E-07
Bordetella_petrii	1.53091E-07	1.11545E-07	4.33818E-07	1.54364E-07	9.32308E-08	1.54692E-07	2.20462E-07	6.28462E-08	0	1.14455E-07	3.95182E-07	1.00364E-07	1.32133E-07	1.108E-07	1.6444E-07	1.61733E-07
Bordetella_sp._N	3.21909E-07	2.83636E-07	9.18182E-08	1.38818E-07	2.40308E-07	5.47692E-08	2.70769E-07	2.16692E-07	3.50455E-07	1.16545E-07	0.000000287	2.25818E-07	4.58E-08	1.866E-07	1.434E-07	4.05533E-07
Bordetella_sp._H567	2.11818E-07	0.000000022	3.82182E-08	9.97273E-09	2.05385E-08	1.39846E-07	6.66154E-08	0	8.54545E-08	0	2.24909E-07	1.42727E-08	0	4.70667E-08	0.000000078	0.000000168
Bordetella_genomosp._8	5.57273E-08	0	4.12727E-09	1.02818E-07	5.20769E-08	0	1.71923E-07	0	0	0.000000116	0	0.00000014	1.03133E-07	4.67333E-08	2.35867E-07	1.46933E-07
Bordetella_flabilis	9.49091E-08	8.39091E-08	1.54091E-07	1.54818E-07	8.79231E-08	4.27462E-07	1.59615E-07	3.40077E-07	0.000000159	2.85455E-08	2.52636E-07	2.06909E-07	4.38E-08	1.48533E-07	1.1164E-07	1.83533E-07
Bordetella_genomosp._9	4.67273E-07	1.58455E-07	2.76073E-07	1.19818E-07	3.02154E-07	0	1.22692E-07	2.59692E-07	6.93636E-07	2.74364E-07	3.80182E-07	1.15718E-06	3.904E-07	1.802E-07	1.04573E-07	1.71067E-07
Bordetella_trematum	6.58818E-07	0	1.48182E-07	3.20364E-07	0.000000039	1.03077E-06	7.45385E-08	1.93385E-07	0.000000091	3.55818E-07	3.07273E-08	8.18091E-07	4.46667E-08	4.88E-08	2.99733E-07	1.708E-07
Alcaligenes_faecalis	1.83591E-06	4.71636E-07	2.09027E-06	3.09818E-06	6.00308E-07	4.76923E-08	3.72308E-06	4.98185E-06	1.73191E-06	0.000001401	1.76927E-06	1.65136E-06	9.26933E-07	1.1276E-06	1.49227E-06	1.61273E-06
Alcaligenes_aquatilis	8.00727E-07	3.24545E-07	1.63636E-08	5.47E-08	8.55692E-07	2.95385E-08	2.94462E-07	1.07538E-07	8.31545E-07	8.97545E-07	0.000000708	1.69818E-06	1.41927E-06	1.32067E-06	1.35827E-06	1.8694E-06
Achromobacter_pestifer	1.15182E-07	0	1.63636E-08	1.16882E-07	1.26538E-07	8.49846E-07	1.74077E-07	2.09462E-07	5.45455E-08	4.63364E-07	2.76364E-08	2.31818E-07	2.94667E-08	1.10867E-07	9.94667E-08	5.71333E-08
Achromobacter_sp._B7	2.27909E-07	1.84818E-07	1.63636E-08	5.63636E-08	2.43385E-07	4.13846E-08	6.06923E-08	0.000000347	0.000000027	0	2.13818E-07	2.36636E-07	1.83067E-07	9.58E-08	0	0.000000101
Achromobacter_sp._AONIH1	4.12727E-07	0.000000065	1.56182E-07	3.02364E-07	7.02308E-08	3.83077E-08	3.11538E-08	1.29462E-07	3.15455E-07	3.62727E-08	0	1.46455E-07	4.27333E-08	0	1.12667E-08	1.86E-08
Achromobacter_insolitus	3.69091E-08	3.78182E-07	0	6.61818E-08	3.34615E-08	2.91692E-07	0	2.46923E-08	0	3.62727E-08	0.000000039	1.73273E-07	6.64667E-08	3.22667E-08	1.57067E-08	1.98667E-08
Achromobacter_xylosoxidans	1.42509E-06	4.90273E-07	2.52455E-07	1.51009E-06	2.13769E-07	4.97308E-07	2.57615E-07	1.31177E-06	6.85273E-07	4.12182E-07	0.000000979	1.05755E-06	5.27867E-07	2.14533E-07	8.38867E-07	6.41867E-07
Achromobacter_sp._MFA1_R4	2.70909E-08	4.11818E-08	0	3.37273E-08	6.73846E-08	0	0	0	0.000000054	7.02727E-08	5.52727E-08	1.72182E-07	0	8.39333E-08	1.57067E-08	6.33333E-08
Achromobacter_spanius	2.35727E-07	7.87091E-07	5.87091E-08	1.24455E-07	8.17692E-08	2.95385E-08	1.99538E-07	2.62077E-07	1.29636E-07	2.87818E-07	1.05636E-07	6.72545E-07	1.382E-07	7.26E-08	1.80533E-07	1.35067E-07
Achromobacter_denitrificans	7.99636E-07	0	3.70909E-08	1.31036E-06	0.00000026	1.49308E-07	0.00000019	5.93077E-07	5.37364E-07	4.48636E-07	3.03818E-07	4.15636E-07	2.522E-07	5.74733E-07	6.28933E-07	1.01433E-06
Herminiimonas_arsenicoxydans	5.27273E-08	0	3.70909E-08	2.91545E-08	2.05385E-08	0	2.63077E-08	1.90769E-08	0.000000084	0	0	3.94545E-08	0.000000102	0	1.57067E-08	0
Herminiimonas_arsenitoxidans	0	5.03636E-08	0.000000046	4.79091E-09	0	0	0	1.28538E-07	0	0	0	0	0	0	0	6.33333E-08
Duganella_sp._GN2-R2	0	0	0	0	8.79231E-08	7.21538E-08	0	3.22308E-08	0	0	0	1.04636E-07	8.32E-08	9.26667E-08	6.87333E-08	5.34E-08
Duganella_sp._AF9R3	0.000000188	1.39091E-07	1.17455E-07	1.34818E-07	3.65385E-07	5.36154E-07	1.27154E-07	1.59231E-07	1.72545E-07	2.13545E-07	3.33727E-07	1.00327E-06	7.72667E-08	1.10933E-07	2.55333E-08	2.54333E-07
Noviherbaspirillum_sp._UKPF54	0.000000418	0	0	1.99755E-06	1.75231E-07	2.12538E-06	3.17931E-06	2.57023E-06	5.69182E-07	4.51545E-07	9.75818E-07	6.02273E-07	3.24667E-07	3.06867E-07	7.27267E-07	2.68867E-07
Undibacterium_sp._YM2	5.20909E-08	0.00000013	5.34545E-08	2.34364E-07	4.64615E-08	0	0	0.000000011	1.23818E-07	0	0	1.38591E-07	2.94667E-08	4.91333E-08	0.000000072	0.000000274
Undibacterium_sp._KW1	4.73636E-08	0.000000065	0	1.41273E-07	0	1.79231E-08	4.33846E-08	3.56923E-08	1.15455E-07	1.03727E-07	5.27273E-07	1.97273E-07	0	4.64667E-08	0	3.97333E-08
Undibacterium_parvum	2.70909E-08	0.000000065	0	6.80636E-08	1.64615E-07	0	2.29154E-07	7.13077E-08	3.98182E-08	5.90909E-08	8.93636E-08	7.13636E-09	1.58667E-07	1.48333E-07	5.40333E-07	1.03133E-07
Collimonas_fungivorans	2.59909E-07	0.000000065	0	2.54791E-07	4.80769E-08	5.73769E-07	5.96154E-08	3.77308E-07	1.73909E-07	1.95273E-07	4.09182E-07	2.00545E-07	4.86E-08	1.40933E-07	7.54E-08	2.20333E-07
Collimonas_pratensis	3.89091E-08	0.000000172	8.60364E-08	9.23636E-08	1.37769E-07	0	6.41538E-08	0.000000634	8.86364E-08	0	1.82545E-07	9.64545E-08	9.88667E-08	1.61333E-08	1.55133E-07	8.63333E-08
Collimonas_arenae	3.78455E-07	1.80364E-07	1.53073E-07	2.63364E-07	6.75154E-07	0	2.32615E-07	4.26615E-07	0.000000386	3.10818E-07	5.71182E-07	5.29727E-07	6.376E-07	3.83333E-07	9.198E-07	1.17593E-06
Herbaspirillum_robiniae	2.56364E-08	0	0	2.89091E-08	0	0	7.10769E-08	6.16154E-08	0	0	2.47545E-07	6.35455E-08	0	0	0	0
Herbaspirillum_frisingense	1.83636E-07	1.40909E-07	7.07273E-08	9.35455E-08	6.73846E-08	1.64923E-07	3.11538E-08	0	0	5.67273E-08	3.77273E-08	0.00000005	2.94667E-08	0	1.026E-07	2.314E-07
Herbaspirillum_seropedicae	5.27909E-07	7.66364E-08	2.56636E-07	6.80818E-07	3.36846E-07	5.49123E-06	5.29538E-07	2.64154E-07	0.000000351	1.74818E-07	8.27091E-07	9.49091E-07	2.746E-07	2.33667E-07	8.90067E-07	0.00000052
Herbaspirillum_huttiense	1.70545E-07	1.22682E-07	2.63218E-07	4.77364E-07	8.06154E-08	1.57385E-07	9.15385E-08	8.01538E-08	2.24455E-07	0.000000128	1.27182E-07	2.05364E-07	2.862E-07	6.72E-08	8.12667E-08	3.53333E-08
Herbaspirillum_sp._meg3	5.63818E-07	2.70909E-08	2.01545E-07	1.89364E-07	6.39923E-07	0	2.16077E-07	0.000000442	1.14073E-06	6.31182E-07	2.76364E-07	2.86818E-07	7.69933E-07	4.12933E-07	4.99133E-07	8.08733E-07
Herbaspirillum_hiltneri	2.91545E-07	2.49636E-07	5.34545E-08	3.10909E-08	1.16923E-07	2.10462E-07	0	9.66923E-08	1.87545E-07	5.22727E-08	3.64091E-07	2.23455E-07	2.37333E-08	4.88E-08	2.38307E-07	1.86667E-08
Herbaspirillum_rubrisubalbicans	2.49882E-06	0.00000013	1.80909E-07	0.000000085	5.02308E-08	0	7.92308E-08	1.46923E-08	3.08091E-07	1.19727E-07	2.56727E-07	2.69273E-07	6.24667E-08	1.496E-07	5.84667E-07	8.68E-08
Janthinobacterium_svalbardensis	0	0	0	0	0	0	0	0	0	0	0	0	6.08E-08	0	0	0
Janthinobacterium_lividum	2.29545E-07	0.00000013	0	3.40909E-08	6.93077E-08	0	6.13846E-08	0	0	0	0	1.18182E-07	9.19333E-08	5.89333E-08	1.49333E-08	4.34E-08
Janthinobacterium_sp._LM6	6.23636E-08	1.68182E-08	0	0	0	2.13846E-08	0	0	0	2.85455E-08	0	0	6.08E-08	0	1.12667E-08	6.81333E-08
Janthinobacterium_sp._1_2014MBL_MicDiv	2.30909E-08	0	0	9.97273E-09	0	0	0	0	3.75455E-08	0	4.36364E-08	2.67909E-07	0	3.19333E-08	0	0
Janthinobacterium_sp._Marseille	1.79091E-07	8.84545E-08	7.38182E-08	1.17727E-07	9.75385E-08	0	2.41846E-07	1.97923E-07	6.59091E-08	3.22455E-07	1.52455E-07	0.000000185	1.14667E-07	0.000000033	1.79333E-07	2.22667E-07
Janthinobacterium_sp._SNU_WT3	2.95455E-07	2.36364E-07	1.21091E-07	1.69455E-07	3.05615E-07	1.03331E-06	3.37231E-07	0.000000419	0.000000249	1.88091E-07	5.35545E-07	5.50364E-07	2.542E-07	1.94067E-07	4.85133E-07	5.06667E-07
Janthinobacterium_sp._17J80-10	3.95182E-07	2.70909E-08	7.40909E-08	1.08727E-07	8.98462E-08	0	7.45385E-08	1.01923E-07	1.86455E-07	5.22727E-08	7.85455E-08	8.36364E-08	8.45333E-08	0	4.44E-09	4.78E-08
Janthinobacterium_sp._B9-8	1.15091E-07	0	0	0	0.000000165	0	0	4.32308E-08	2.16364E-08	8.02727E-08	0.000000039	0.000000179	1.17667E-07	0	2.39333E-08	3.72E-08
Janthinobacterium_agaricidamnosum	0.00000006	0	0	0	0	2.13846E-08	0	0	0	0	1.02727E-08	7.13636E-09	4.61333E-08	8.20667E-08	4.44E-09	1.148E-07
Janthinobacterium_sp._HH102	2.43818E-07	0.00000005	1.65455E-08	4.85727E-07	2.40154E-07	2.47846E-07	0.000000903	8.07077E-07	3.57545E-07	5.19909E-07	4.29182E-07	3.59909E-07	2.88133E-07	7.48933E-07	3.834E-07	1.384E-07
Oxalobacter_formigenes	1.68064E-06	7.63364E-07	4.22218E-07	1.06782E-06	2.64269E-06	1.24408E-06	1.83215E-06	1.26031E-06	1.69145E-06	1.37864E-06	1.27109E-06	3.65482E-06	6.37067E-07	1.08393E-06	1.04167E-06	1.32987E-06
Massilia_sp._Se16.2.3	0	0	0	0	0	0	0	0	0	3.03636E-08	2.76364E-08	0	0	6.18E-08	1.65333E-08	0
Massilia_umbonata	2.39364E-07	1.45091E-07	0	1.02455E-07	0.000000153	0	2.73846E-08	2.47692E-08	2.16364E-08	9.54545E-08	1.02727E-08	1.45545E-07	7.16E-08	3.22667E-08	1.198E-07	1.00733E-07
Massilia_plicata	6.19091E-08	0	2.87273E-08	1.58545E-07	8.79231E-08	0	0	3.93846E-08	5.91818E-08	8.08182E-08	2.47545E-07	1.42727E-08	1.204E-07	2.98E-08	1.52773E-07	4.00933E-07
Massilia_sp._WG5	5.27273E-08	0	0	1.47273E-08	0	4.27692E-08	0	6.91538E-08	4.91818E-08	1.93545E-07	3.77273E-08	1.42727E-08	5.18667E-08	0	1.61333E-07	1.86E-08
Massilia_flava	9.06636E-07	3.21182E-07	1.26364E-07	5.16818E-07	2.26923E-08	0	2.63077E-08	4.37692E-08	5.80091E-07	1.90182E-07	2.13909E-07	2.61909E-07	0	1.18467E-07	1.66533E-07	1.44867E-07
Massilia_armeniaca	3.62727E-08	1.90227E-07	2.96364E-08	4.87273E-08	0	5.29231E-08	0	0	0	0	0.000000061	5.17273E-08	0	2.38E-08	8.86667E-09	0
Massilia_albidiflava	0	0	1.63636E-08	1.98909E-07	0	0.00000031	0	2.47692E-08	5.79091E-08	1.13636E-07	2.48636E-07	5.64545E-08	5.02667E-08	4.90667E-08	2.46973E-07	0.000000057
Massilia_putida	3.15455E-08	0	1.19664E-07	3.89727E-07	1.58385E-07	0	0	1.39077E-07	2.58818E-07	5.90909E-08	0	7.13636E-09	9.12667E-08	5.03333E-08	8.08E-08	3.78467E-07
Massilia_violaceinigra	4.80364E-07	3.18636E-07	0.00000015	1.79545E-07	1.98923E-07	7.71538E-08	3.00538E-07	1.67769E-07	2.38455E-07	2.64545E-07	2.32455E-07	0.000000635	9.59333E-08	6.48667E-08	1.39867E-07	1.27333E-07
Massilia_lutea	2.86182E-07	2.95273E-07	3.00182E-07	6.92727E-08	0.000000147	1.71462E-07	2.79462E-07	2.46923E-08	1.53545E-07	0	3.33636E-08	5.23636E-07	0	0.000000042	1.41333E-07	1.98667E-08
Massilia_oculi	3.98727E-07	2.87273E-08	8.31818E-09	0.000000147	1.54231E-07	2.87385E-07	1.55462E-07	2.30308E-07	1.89545E-07	2.14455E-07	4.47273E-07	9.05455E-08	8.28E-08	2.27267E-07	2.11733E-07	3.98533E-07
Massilia_sp._NR_4-1	4.42545E-07	0.00000026	9.90909E-08	4.17182E-07	5.96692E-07	5.88692E-06	0.000001784	3.42385E-06	1.01864E-06	6.01273E-07	0.000001289	1.18645E-06	1.52507E-06	9.84533E-07	1.54373E-06	2.50187E-06
Massilia_sp._LPB0304	7.55727E-07	1.94545E-07	3.70909E-08	1.65909E-07	1.36077E-06	8.17769E-06	1.59031E-06	3.48231E-06	1.41891E-06	7.71727E-07	1.14318E-06	1.06109E-06	1.49907E-06	9.10267E-07	2.61487E-06	2.37753E-06
Massilia_sp._YMA4	1.72209E-06	0	3.71273E-07	1.75164E-06	4.22462E-07	0	2.49462E-07	5.29769E-07	8.15909E-07	5.68455E-07	7.90273E-07	2.27364E-06	5.536E-07	1.06487E-06	6.34467E-07	7.32467E-07
Pulveribacter_suum	2.96364E-08	0	0	0	3.15385E-08	2.13846E-08	0	5.13846E-08	2.75455E-08	0	5.52727E-08	4.21364E-08	2.14667E-08	3.50667E-08	0	0
Limnohabitans_sp._103DPR2	8.24545E-08	0	0	4.79091E-09	0.000000138	2.12154E-07	0	8.64615E-08	1.84091E-07	8.63636E-08	2.76364E-08	1.42727E-07	4.53333E-08	0.000000066	0	2.49667E-07
Limnohabitans_sp._63ED37-2	1.49364E-07	0	3.70909E-08	5.58545E-07	9.52308E-08	5.31769E-07	2.63077E-08	1.62769E-07	5.46364E-08	1.61636E-07	1.06364E-07	1.39091E-07	5.55333E-08	1.318E-07	1.71973E-07	2.662E-07
Hylemonella_gracilis	4.73636E-08	0	7.40909E-08	7.71545E-08	0	2.95385E-08	3.11538E-08	1.10385E-07	9.72727E-08	1.79182E-07	6.55455E-08	1.75364E-07	0	2.62E-08	1.25867E-07	1.20533E-07
Curvibacter_sp._AEP1-3	2.30909E-08	0	0	0	2.88462E-08	2.13846E-08	0	0	5.34545E-08	3.89091E-08	1.19818E-07	1.45955E-07	2.08E-08	0	3.14667E-08	0.000000138
Verminephrobacter_eiseniae	6.42091E-07	7.38636E-08	1.36273E-06	7.27273E-07	5.74077E-07	3.15846E-07	1.25023E-06	2.09023E-06	4.70909E-07	3.57455E-07	5.66818E-07	4.00909E-07	4.91467E-07	6.91067E-07	8.848E-07	1.06013E-06
Melaminivora_sp._SC2-9	2.98182E-08	0	0	4.79091E-09	1.22462E-07	1.04462E-07	3.06923E-08	0.000000022	1.60091E-07	0.000000128	1.74455E-07	2.65909E-07	4.45333E-08	1.58E-08	1.59973E-07	1.86667E-07
Simplicispira_suum	3.95727E-07	0	2.484E-07	7.72727E-08	0	4.73385E-07	1.31385E-07	3.66077E-07	3.32545E-07	1.57636E-07	1.35636E-07	3.03909E-07	9.96667E-08	1.58E-08	1.2504E-07	1.73267E-07
Diaphorobacter_polyhydroxybutyrativorans	0	0	0	0	0	0	3.33077E-08	2.47692E-08	0	0	0	0	0	0	0	9.33333E-09
Diaphorobacter_sp._HDW4B	1.78273E-07	9.27273E-08	1.72727E-07	3.03091E-07	0	0	0	1.04615E-07	2.00091E-07	7.29091E-08	7.90909E-08	4.59091E-08	1.29467E-07	0	2.83733E-08	7.39333E-08
Diaphorobacter_sp._HDW4A	3.45636E-07	3.99273E-07	2.81609E-07	1.87182E-07	1.08538E-07	0.000000026	3.12231E-07	2.02154E-07	4.14909E-07	3.62727E-08	2.45909E-07	4.00818E-07	1.542E-07	1.60267E-07	4.80133E-07	1.51267E-07
Diaphorobacter_ruginosibacter	1.99455E-07	1.04727E-07	2.88545E-08	2.67364E-07	6.46923E-08	0	2.63077E-08	1.85462E-07	1.56636E-07	0.000000056	3.33636E-08	1.26909E-07	2.09333E-08	0.000000014	6.46667E-08	3.08E-08
Diaphorobacter_aerolatus	5.68182E-08	0	4.12727E-09	6.98182E-08	2.75385E-08	1.79231E-08	8.13846E-08	6.10769E-08	1.51091E-07	7.02727E-08	0.000000143	2.37727E-07	1.272E-07	0	5.86667E-08	4.01333E-08
Ramlibacter_tataouinensis	6.41455E-07	0.000000491	6.31273E-07	6.93545E-07	1.43462E-07	4.46308E-07	1.21154E-06	9.87615E-07	2.53364E-07	2.88455E-07	3.25091E-07	4.77273E-07	2.04067E-07	2.17267E-07	2.25467E-07	4.124E-07
Kinneretia_sp._DAIF2	1.05273E-07	0.00000013	3.40909E-08	1.29427E-07	0	0	8.69231E-08	0.000000011	5.97273E-08	2.87273E-08	3.77273E-08	1.20545E-07	2.14E-08	5.19333E-08	8.484E-08	5.19333E-08
Candidatus_Symbiobacter_mobilis	8.70636E-07	0.000000065	2.42364E-06	2.00055E-06	9.76692E-07	2.76846E-07	7.64308E-06	0.0000067	1.13355E-06	1.09764E-06	0.000000975	0.000001983	9.85067E-07	6.486E-07	1.38687E-06	1.3178E-06
Serpentinomonas_raichei	1.09909E-07	2.70909E-08	0	9.97273E-09	7.02308E-08	3.11538E-08	1.95385E-07	1.02538E-07	1.44636E-07	1.84364E-07	0.000000195	3.11909E-07	5.58667E-08	5.81333E-08	4.46667E-08	9.26E-08
Serpentinomonas_mccroryi	2.24491E-06	7.10545E-07	2.75936E-06	2.33155E-06	9.82308E-08	1.51254E-06	0.000000615	4.78154E-07	8.44636E-07	7.20636E-07	1.27073E-06	1.51209E-06	1.556E-07	2.51267E-07	2.42933E-07	3.48533E-07
Ottowia_oryzae	7.12727E-08	3.24545E-07	3.69182E-07	3.64818E-07	6.69231E-08	3.96154E-08	4.02615E-07	1.78846E-07	1.24091E-07	0	2.13636E-07	2.74455E-07	1.45667E-07	8.42E-08	7.68E-08	2.00067E-07
Ottowia_sp._oral_taxon_894	2.17573E-06	1.16364E-08	2.43909E-07	6.32091E-07	5.90769E-07	1.22692E-07	9.48462E-07	2.20085E-06	1.84645E-06	2.01555E-06	1.62864E-06	1.79627E-06	5.98667E-07	9.84533E-07	6.19067E-07	6.786E-07
Schlegelella_thermodepolymerans	4.68209E-06	9.40818E-07	5.00014E-06	2.74682E-06	8.51846E-07	6.15462E-07	7.41231E-06	6.71938E-06	1.32818E-06	2.80136E-06	3.35455E-06	3.12291E-06	8.69733E-07	2.40427E-06	1.48373E-06	1.35467E-06
Polaromonas_sp.	0	0	0	0	3.15385E-08	0	0	0	0	0	0	7.13636E-09	0	0	0	4.26667E-08
Polaromonas_sp._JS666	0	7.40909E-08	0	0	0	0	0	0	0	0	0	0	0	3.50667E-08	5.94E-08	0
Polaromonas_naphthalenivorans	3.36091E-07	1.57273E-07	1.62636E-07	5.11545E-07	8.55385E-08	8.30769E-08	1.24077E-07	2.00923E-07	0.000000131	0.000000249	3.18364E-07	3.55091E-07	2.706E-07	5.68E-08	3.24773E-07	1.84733E-07
Polaromonas_vacuolata	1.63636E-07	0.000000047	0	1.93636E-08	5.63692E-07	0	0.000000293	1.12385E-07	7.90091E-07	2.66182E-07	3.79091E-08	5.27545E-07	8.57933E-07	4.04933E-07	4.87733E-07	9.276E-07
Rhodoferax_sp._BAB1	3.11818E-08	0	0	0	0	0.000000133	0	3.22308E-08	0	0	0	0	0	0	0	1.98667E-08
Rhodoferax_saidenbachensis	6.74545E-08	5.41818E-08	0	2.95455E-08	0	0	0	1.04615E-07	1.72091E-07	3.89091E-08	2.05455E-08	1.43455E-07	1.098E-07	2.62E-08	9.45333E-08	1.19133E-07
Rhodoferax_antarcticus	5.71818E-08	0	0	1.55545E-07	1.00769E-07	0	2.63077E-08	4.63846E-07	1.08545E-07	1.58182E-07	1.35636E-07	7.55455E-08	7.05333E-08	9.34E-08	1.336E-07	8.98E-08
Rhodoferax_ferrireducens	1.39564E-06	0.000000065	1.85455E-07	1.11964E-06	2.59077E-07	7.33077E-08	1.95846E-07	7.47385E-07	2.95636E-07	6.52818E-07	1.12364E-06	5.91364E-07	2.62733E-07	1.61933E-07	1.12333E-07	3.144E-07
Rhodoferax_koreense	0.00000006	2.70909E-08	1.83764E-07	1.88545E-07	3.58462E-08	2.53385E-07	1.41077E-07	5.81538E-08	3.98182E-08	0	2.13091E-07	2.76273E-07	0.000000134	3.19333E-08	0.000000238	2.018E-07
Rhodoferax_sediminis	6.92818E-07	1.48909E-07	1.25182E-07	5.42091E-07	2.89077E-07	6.10462E-07	4.47538E-07	1.00731E-06	8.40818E-07	3.31727E-07	6.27091E-07	9.71455E-07	2.14667E-07	3.98267E-07	3.916E-07	6.58867E-07
Alicycliphilus_denitrificans	3.35091E-07	0	3.70909E-08	1.03636E-08	4.91923E-07	0	1.71846E-07	2.19692E-07	7.59273E-07	0.00000057	6.43818E-07	8.40091E-07	1.1918E-06	6.00867E-07	0.000001249	1.4784E-06
Comamonas_sp._NLF-7-7	3.69091E-08	0.000000065	0	0	3.58462E-08	0	0	0	0	0	0	0	0	1.61333E-08	1.65333E-08	1.22E-08
Comamonas_aquatica	2.96364E-08	1.16364E-08	1.63636E-08	0	2.05385E-08	0	9.03077E-08	5.22308E-08	0	0	0	6.21818E-08	2.91333E-08	3.78E-08	5.52667E-08	7.25333E-08
Comamonas_serinivorans	2.33909E-07	2.22318E-07	0	4.82727E-08	1.76846E-07	3.01615E-07	3.06923E-08	5.03846E-08	5.97273E-08	0.000000081	0	9.14545E-08	7.82667E-08	3.15333E-08	8.86667E-09	1.85467E-07
Comamonas_thiooxydans	2.98182E-08	1.16364E-08	1.06909E-07	5.42727E-08	0	3.83077E-08	0	9.72308E-08	0	0	6.66364E-08	3.66818E-07	0	0	1.442E-07	0.000000318
Comamonas_koreensis	1.42455E-07	0	0	1.79636E-07	3.00231E-07	1.32923E-07	0	0	3.81182E-07	1.71727E-07	6.58727E-07	5.96545E-07	2.93067E-07	2.36333E-07	3.17533E-07	1.53933E-07
Comamonas_kerstersii	2.25455E-07	3.98364E-07	1.01364E-07	1.50545E-07	1.45077E-07	9.08462E-08	4.55077E-07	4.90692E-07	1.53273E-07	1.37091E-07	3.73636E-07	3.80727E-07	2.14E-08	1.99333E-07	4.86667E-07	4.986E-07
Comamonas_piscis	3.22636E-07	5.41364E-07	2.31764E-07	1.65545E-07	1.05154E-07	8.30769E-08	1.57692E-07	1.10538E-07	2.65545E-07	1.78727E-07	8.02727E-08	0.000000335	2.09333E-08	0	2.09733E-08	6.33333E-08
Comamonas_testosteroni	0.000001454	2.03818E-07	6.62091E-08	4.52727E-07	8.30462E-07	4.05231E-07	1.31154E-07	3.60692E-07	6.84455E-07	7.77273E-07	6.76636E-07	1.20973E-06	4.39933E-07	5.32667E-07	4.21867E-07	7.65133E-07
Hydrogenophaga_sp._RAC07	0	0	0	4.79091E-09	0	7.21538E-08	0	0	8.91818E-08	0	0	0	0	0	0	0
Hydrogenophaga_pseudoflava	0	0	0	3.24545E-08	0	2.13846E-08	0	0	0	0	0	0.000000035	0	0	2.99333E-08	0
Hydrogenophaga_crassostreae	0	2.32727E-08	0	2.55245E-07	1.64615E-07	2.52308E-07	3.09231E-07	2.46923E-08	1.19091E-07	1.19182E-07	2.76364E-08	1.81364E-07	1.09733E-07	1.87333E-07	2.138E-07	2.628E-07
Hydrogenophaga_sp._BA0156	5.57273E-08	1.58727E-07	6.33636E-08	3.47273E-08	0	1.86462E-07	0	0	5.79091E-08	0	0	4.92727E-08	0	0	1.12667E-08	0
Hydrogenophaga_sp._PAMC20947	2.40364E-07	1.27182E-07	1.03727E-07	4.28182E-08	2.09846E-07	2.61923E-07	1.79846E-07	3.96154E-07	0.000000484	0.000000274	0.000000391	2.44727E-07	7.29333E-08	1.54067E-07	7.07067E-08	1.324E-07
Hydrogenophaga_sp._PBL-H3	7.40909E-08	0	3.70909E-08	3.72455E-08	2.59231E-08	6.61615E-07	1.81769E-07	2.38538E-07	1.75364E-07	1.25636E-07	1.04545E-07	2.15636E-07	2.39267E-07	1.89467E-07	4.25333E-07	1.55333E-07
Hydrogenophaga_sp._NH-16	2.13936E-06	1.16427E-06	8.20636E-07	1.14273E-06	1.46031E-06	0.000011141	2.02162E-06	1.46454E-06	1.11609E-06	2.09773E-06	4.71909E-06	3.54564E-06	3.0278E-06	4.26593E-06	3.61093E-06	5.28447E-06
Hydrogenophaga_sp._PBC	1.15582E-06	0	8.75455E-09	1.92364E-07	2.57231E-07	3.83077E-08	7.07692E-08	1.08685E-06	5.86364E-07	4.84636E-07	7.27091E-07	4.63273E-07	6.04267E-07	3.526E-07	3.524E-07	5.96467E-07
Hydrogenophaga_sp._BPS33	9.50364E-07	0.00000013	1.10909E-07	6.55909E-07	0.000000583	6.10769E-08	0	4.80846E-07	6.52636E-07	6.28545E-07	1.27909E-06	0.000000762	2.756E-07	4.58133E-07	1.03287E-06	4.44133E-07
Acidovorax_sp._RAC01	0.00000006	0	0	8.23364E-08	0	0	0	0	1.50727E-07	0	1.06364E-07	7.13636E-09	6.69333E-08	1.064E-07	1.12667E-08	5.66E-08
Acidovorax_citrulli	0	0	3.70909E-08	0	2.05385E-08	0	2.06923E-07	2.47692E-08	0	0	0	7.13636E-09	0.000000081	0	0	1.86E-08
Acidovorax_ebreus	0	0	0	2.43636E-08	2.75385E-08	0	0	0	0	0	3.33636E-08	0.000000035	0	0	0	0
Acidovorax_cattleyae	2.70909E-08	7.69091E-08	0	4.79091E-09	4.93846E-08	0	0	0	0	1.22818E-07	6.66364E-08	6.35455E-08	2.14667E-08	1.01133E-07	1.12667E-08	0
Acidovorax_sp._JMULE5	3.80727E-07	0	7.40909E-08	4.61091E-07	2.59231E-08	2.13846E-08	0	2.46923E-08	1.74636E-07	5.22727E-08	1.49091E-07	3.29818E-07	4.86E-08	1.01133E-07	8.45333E-08	1.974E-07
Acidovorax_sp._T1	0	0	0	0	5.63846E-08	0	3.33077E-08	6.69231E-08	2.16364E-08	0	0.000000078	0.000000035	0	0	0	0
Acidovorax_sp._KKS102	2.12545E-07	0	6.74909E-08	1.45336E-07	1.76923E-07	7.29231E-08	1.64846E-07	1.66538E-07	2.81545E-07	1.03727E-07	1.51455E-07	1.42273E-07	1.20133E-07	1.92467E-07	1.49333E-08	1.668E-07
Acidovorax_sp._16-35-5	2.08636E-07	0.000000065	5.56182E-08	2.07364E-07	2.41462E-07	2.84923E-07	1.61538E-07	3.69231E-07	3.48182E-07	1.02636E-07	7.70909E-08	5.24273E-07	5.01333E-08	3.55533E-07	2.54267E-07	3.52667E-07

Acidovorax_sp._HDW3	6.41727E-07	1.10864E-06	2.12727E-07	4.02273E-07	3.15385E-08	2.38308E-07	1.83615E-07	2.17615E-07	1.60636E-07	3.98455E-07	4.58545E-07	2.57727E-07	0	0	4.46667E-08	1.86E-08
Acidovorax_monticola	1.37727E-07	1.96273E-07	8.35636E-08	2.06364E-07	3.58462E-08	0	0.000000215	8.51538E-08	0.00000013	0	0	1.61636E-07	4.27333E-08	0	1.49333E-08	0
Acidovorax_sp._1608163	2.95364E-07	1.92364E-07	6.99455E-08	3.58273E-07	1.43231E-07	7.82308E-07	6.33385E-07	6.29154E-07	8.88182E-07	5.43364E-07	7.06909E-07	4.72182E-07	2.52667E-07	2.324E-07	3.98133E-07	4.86267E-07
Acidovorax_carolinensis	6.72364E-07	0.000000437	3.29636E-07	4.67818E-07	1.09308E-07	0	7.92308E-08	0.000000098	3.05636E-07	1.37818E-07	1.11909E-07	0.000000372	7.08667E-08	8.03333E-08	7.29333E-08	1.836E-07
Acidovorax_avenae	3.46364E-07	8.82727E-08	1.63636E-08	6.92727E-08	3.66923E-07	4.30769E-08	8.69231E-08	0.000000756	3.43273E-07	1.18909E-07	2.51364E-07	1.69636E-07	3.66333E-07	4.91933E-07	5.20933E-07	5.186E-07
Variovorax_sp._PBL-E5	0	0	0	4.79091E-09	0	0	0	0	0	0	0	0	0	0	0	0
Variovorax_sp._SRS16	9.92727E-08	0	0	1.31909E-07	2.21385E-07	1.12615E-07	0	1.71462E-07	2.44727E-07	1.32545E-07	6.84545E-08	4.50182E-07	2.026E-07	1.05933E-07	4.77333E-08	0
Variovorax_sp._RA8	1.41545E-07	1.17273E-07	0	2.39727E-08	3.15385E-08	0	2.60077E-07	1.40231E-07	1.78636E-07	1.57273E-07	0	4.92727E-08	6.11333E-08	9.82667E-08	0	0
Variovorax_sp._PAMC_28711	0	0	0	0	0	0	0	0	5.97273E-08	0.000000047	1.02182E-07	8.26818E-08	0	0	0	0
Variovorax_sp._PAMC28562	0.00000011	0	8.75455E-09	1.04155E-07	0	0	6.40769E-08	0	8.91818E-08	1.54909E-07	1.74545E-07	3.74545E-08	4.50667E-08	1.23067E-07	2.39333E-08	1.86E-08
Variovorax_sp._38R	6.91818E-08	0	4.12727E-09	1.18182E-07	0.00000011	0	0	3.81538E-08	2.07727E-07	1.04545E-07	4.92727E-08	1.41273E-07	9.58E-08	6.36667E-08	2.57733E-07	1.43467E-07
Variovorax_sp._PBL-H6	1.58182E-08	0	2.46182E-08	9.57E-08	0	3.15385E-08	9.61538E-08	3.56923E-08	0.000000177	0	3.95455E-08	0.000000107	0	0	0	0
Variovorax_sp._HW608	2.42909E-07	2.70909E-08	9.20909E-08	4.68273E-07	2.98385E-07	6.66077E-07	4.01846E-07	1.19331E-06	1.14273E-07	3.43909E-07	2.14545E-07	5.71455E-07	5.06333E-07	3.33333E-07	5.804E-07	8.258E-07
Variovorax_sp._PAMC26660	3.11818E-08	1.75455E-07	3.29091E-08	1.67909E-07	2.17154E-07	4.10692E-07	3.22462E-07	1.73769E-07	3.38273E-07	3.23727E-07	2.28636E-07	3.73182E-07	7.33333E-08	0.000000198	7.35333E-08	3.08E-08
Variovorax_sp._PBS-H4	4.28273E-07	9.85636E-07	2.04255E-07	3.35909E-07	9.06154E-08	0	6.23077E-08	3.68615E-07	5.91818E-08	1.98636E-07	3.68818E-07	8.97455E-07	5.18667E-08	4.91333E-08	1.27133E-07	6.76667E-08
Variovorax_sp._PMC12	2.93091E-07	4.25636E-07	5.75818E-08	6.26364E-08	3.15385E-08	0	1.05385E-07	1.28538E-07	1.41909E-07	0.000000034	0	1.37364E-07	9.04667E-08	1.69667E-07	2.5364E-07	1.01267E-07
Variovorax_sp._WDL1	6.82364E-07	1.61818E-07	4.01909E-07	4.06727E-07	2.74462E-07	1.77077E-07	3.59923E-07	1.65231E-07	2.38182E-07	4.49364E-07	3.01545E-07	8.89273E-07	4.078E-07	8.49333E-08	9.24E-08	2.22733E-07
Variovorax_paradoxus	0.000001629	3.28182E-08	2.38727E-07	2.86564E-06	3.64846E-07	1.85046E-06	2.32954E-06	2.87062E-06	9.50545E-07	1.58736E-06	0.000002432	1.92855E-06	8.662E-07	1.01667E-06	9.01067E-07	1.1756E-06
Delftia_sp._Cs1-4	0	0	0	0	0	0	0	0	0	0	0	0	5.44E-08	0	0	0
Delftia_sp._HK171	0	0	0	0	0	3.83077E-08	1.33077E-07	1.46923E-08	0	1.27273E-07	0	7.13636E-09	2.09333E-08	0	8.04E-08	1.98667E-08
Delftia_acidovorans	1.48573E-06	0	3.38182E-07	6.15636E-07	7.06308E-07	5.07769E-07	3.32538E-07	5.79231E-07	0.00000085	8.62091E-07	5.56455E-07	1.53327E-06	5.52267E-07	7.31067E-07	6.49133E-07	0.00000049
Delftia_tsuruhatensis	5.57273E-08	0	0	2.03236E-06	2.69077E-06	1.81077E-07	1.58462E-06	2.20369E-06	2.83636E-07	5.09455E-07	9.48455E-07	8.32182E-07	3.80467E-06	0.000003656	5.18867E-06	0.000005842
Ephemeroptericola_cinctiostellae	1.83091E-07	0	2.50909E-07	1.18182E-07	6.76923E-08	0	1.07385E-07	7.72308E-08	2.95636E-07	1.81273E-07	1.13273E-07	1.64455E-07	5.18667E-08	9.45333E-08	1.304E-07	3.84667E-08
Caballeronia_insecticola	1.12091E-07	0	0	5.18182E-09	2.05385E-08	0	2.30615E-07	4.93846E-08	5.79091E-08	0.000000116	2.76364E-08	7.13636E-09	2.72E-08	4.90667E-08	4.44E-09	0
Chitinimonas_arctica	0	0	0	6.16091E-08	0	0	0	4.37692E-08	8.20909E-08	0	0.000000039	8.98182E-08	2.14E-08	0	8.86667E-09	0
Limnobacter_sp._SAORIC-580	5.69091E-08	0	4.12727E-09	2.39091E-08	1.04231E-07	8.22385E-07	5.32846E-07	6.61231E-07	3.10636E-07	1.50273E-07	0.000000247	0.000000117	2.194E-07	1.65067E-07	0.000000361	3.16467E-07
Mycetohabitans_rhizoxinica	3.89091E-08	0.000000065	1.77273E-08	1.50909E-07	2.88462E-08	0.000000239	2.34308E-07	1.17231E-07	0	8.88182E-08	1.45909E-07	2.54091E-07	6.08E-08	3.48E-08	0	1.98667E-08
Mycoavidus_cysteinexigens	4.54545E-08	1.17273E-07	3.70909E-08	0	2.05385E-08	4.68077E-07	2.68462E-07	1.25692E-07	0	0.000000056	3.95455E-08	0.000000035	4.74667E-08	1.58E-08	0	9.33333E-09
Mycoavidus_sp._B2-EB	0	0	0	0	0.000000039	0	0	0	9.96364E-08	1.81273E-07	5.39091E-08	3.87727E-08	0	0	0	1.66667E-08
Lautropia_mirabilis	3.19273E-07	1.98273E-07	4.37273E-08	1.097E-07	2.89538E-07	7.61462E-07	3.10154E-07	8.32692E-07	6.11091E-07	5.52091E-07	2.58091E-07	5.24818E-07	7.766E-07	5.06933E-07	7.734E-07	8.08067E-07
Cupriavidus_neocaledonicus	0	0	0	0	0	0	0	0	0	0	0	0	0	0	4.44E-09	0
Cupriavidus_basilensis	4.87273E-08	1.04545E-07	8.61818E-08	5.21909E-07	3.58462E-08	0	2.63077E-08	1.90769E-08	0.000000303	5.22727E-08	1.85909E-07	1.88727E-07	0	5.12E-08	6.44E-08	7.99333E-08
Cupriavidus_campinensis	3.45545E-07	2.34545E-08	6.67273E-08	7.18182E-08	3.58462E-08	0	0	9.27692E-08	3.94545E-08	0.000000034	7.29091E-08	3.56636E-07	1.364E-07	2.98E-08	1.25573E-07	1.06733E-07
Cupriavidus_necator	8.33364E-07	2.34545E-08	0	2.61636E-07	0.000000542	6.76923E-08	2.63077E-08	9.66154E-08	0.000000431	0.000000397	3.98091E-07	1.67727E-07	0.000000494	3.18667E-07	1.844E-07	3.272E-07
Cupriavidus_pauculus	2.54364E-07	0	3.14364E-07	2.89636E-07	5.93846E-08	9.46154E-09	7.60462E-07	8.11385E-07	1.34545E-07	8.02727E-08	2.74545E-07	2.26682E-07	1.27533E-07	8.77333E-08	2.9744E-07	6.084E-07
Cupriavidus_nantongensis	6.24545E-08	1.04545E-08	2.18545E-08	5.74545E-08	0	0	0	1.90769E-08	0	0	3.33636E-08	7.13636E-09	0	0	4.44E-09	1.86E-08
Cupriavidus_gilardii	7.04455E-07	4.43636E-07	3.04855E-07	1.02455E-07	7.24615E-08	0	1.31538E-07	9.31538E-08	2.38545E-07	0.000000047	2.15727E-07	8.20727E-07	2.64667E-08	1.61333E-08	0	0
Cupriavidus_pinatubonensis	1.02273E-07	2.32455E-07	3.70909E-08	1.08545E-07	7.45385E-07	0	2.48692E-07	1.52615E-07	7.42091E-07	4.13364E-07	1.94091E-07	3.01727E-07	5.54667E-07	1.92467E-07	2.766E-07	7.656E-07
Cupriavidus_taiwanensis	4.46091E-07	0.000000239	4.53455E-08	5.97182E-07	2.41923E-07	1.17369E-06	1.40769E-07	4.04846E-07	5.57727E-07	0.000000342	7.22727E-07	9.50727E-07	2.90533E-07	3.30067E-07	2.5064E-07	3.412E-07
Cupriavidus_metallidurans	2.98818E-07	2.84045E-07	1.76027E-07	2.45091E-07	2.73846E-07	9.68462E-08	2.31385E-07	5.09077E-07	8.13636E-08	0.000000241	7.24091E-07	8.02182E-07	3.226E-07	2.34067E-07	3.822E-07	2.84667E-07
Cupriavidus_oxalaticus	4.71909E-07	0	2.19273E-07	4.34364E-07	1.67615E-07	6.27692E-07	7.49615E-07	1.01577E-06	3.61364E-07	0.00000052	1.09182E-06	0.000000926	1.546E-07	2.31467E-07	1.66533E-07	1.976E-07
Ralstonia_mannitolilytica	6.08182E-08	8.86364E-09	0	0	3.58462E-08	9.33077E-08	2.11154E-07	0	0.000000027	5.22727E-08	0.000000039	0	8.21333E-08	0	0	0
Ralstonia_insidiosa	4.56364E-08	0	0	1.95455E-08	6.17692E-08	4.49692E-07	2.65538E-07	3.07308E-07	0.000000091	0.000000047	1.65545E-07	2.30364E-07	1.77667E-07	1.47133E-07	1.356E-07	3.26867E-07
Ralstonia_pickettii	1.73273E-07	6.62545E-07	1.40909E-07	2.24273E-07	3.34615E-08	1.35231E-07	8.86154E-08	1.95615E-07	8.43636E-08	6.74545E-08	2.61818E-07	2.14364E-07	0.00000008	2.38733E-07	2.12067E-07	2.066E-07
Ralstonia_solanacearum	1.82627E-06	0.00000037	2.66512E-06	0.000003805	1.23885E-06	2.75846E-07	9.39385E-06	7.15015E-06	1.75782E-06	1.58527E-06	1.08791E-06	1.15027E-06	0.000000696	5.98733E-07	1.1322E-06	2.51733E-07
Paraburkholderia_atlantica	0	0	0	0	0	0	0	0	0	0	0	0	2.37333E-08	0	0	0
Paraburkholderia_sp._Msb3	0	0	0	5.18182E-09	0	0	0	0	9.77273E-08	0	0	0	0	0	0	0
Paraburkholderia_dokdonella	0	0	0	4.40909E-08	3.15385E-08	0	0	2.47692E-08	0	2.85455E-08	0	2.09455E-07	4.33333E-08	0	0	0
Paraburkholderia_terrae	0	0	0	9.40909E-08	0	0	0	0	0	0	0	8.29091E-08	5.88667E-08	2.38E-08	0	9.93333E-08
Paraburkholderia_sp._DHF22	0	0	0	0	2.05385E-08	0	0	5.48462E-08	9.96364E-08	5.22727E-08	0	3.74545E-08	1.16667E-07	2.62E-08	0	1.454E-07
Paraburkholderia_phymatum	0	0	2.87273E-08	0	0	3.46923E-08	0	0	0	0	0	0	6.62667E-08	3.09333E-08	0	0
Paraburkholderia_phytofirmans	6.23636E-08	1.16364E-08	0	1.56182E-07	0	0.000000696	0.000000341	2.30154E-07	2.64545E-08	5.22727E-08	0	7.64545E-08	0	0	4.44E-09	1.98667E-08
Paraburkholderia_terricola	9.26364E-08	0	0	9.54545E-09	3.15385E-08	0	6.23077E-08	1.01308E-07	1.14545E-07	2.87273E-08	1.02727E-08	0	2.09333E-08	1.53333E-07	0	3.40667E-08
Paraburkholderia_xenovorans	0	0.000000109	3.70909E-08	7.64273E-08	0	1.23846E-07	0	3.00769E-08	0	8.62727E-08	2.05455E-08	1.30791E-06	0	0	1.074E-07	1.86667E-08
Paraburkholderia_sp._PGU19	3.62727E-08	0.000000065	2.47273E-08	1.72273E-07	3.34615E-08	0	0	0	3.98182E-08	0	0	3.64636E-07	0	0	0	0
Paraburkholderia_caffeinilytica	1.21636E-07	0.00000013	1.63636E-08	0	7.16923E-08	0	0	7.40769E-08	8.20909E-08	1.02636E-07	2.76364E-08	6.41364E-08	0	1.11467E-07	8.86667E-09	0
Paraburkholderia_aromaticivorans	0.000000061	0.000000223	1.84091E-07	2.01727E-07	0.000000061	4.20385E-07	1.48846E-07	1.36846E-07	9.77273E-08	8.59091E-08	1.30909E-07	3.22364E-07	6.08E-08	1.25467E-07	3.28E-08	1.01667E-07
Paraburkholderia_fungorum	1.57909E-07	0.000000047	0	0	1.48846E-07	0	0	1.04615E-07	3.94545E-08	1.95455E-07	3.33636E-08	9.51818E-08	1.14467E-07	1.17533E-07	1.33773E-07	1.26533E-07
Paraburkholderia_sp._7Q-K02	1.09455E-07	9.61364E-08	3.70909E-08	3.00636E-07	4.13846E-08	0	1.41923E-07	2.02692E-07	1.26455E-07	5.22727E-08	1.75909E-07	4.92909E-07	1.86667E-08	6.80667E-08	2.09733E-08	3.20667E-08
Paraburkholderia_phenoliruptrix	1.92727E-07	0	2.59818E-08	8.67273E-08	6.46923E-08	2.05154E-07	5.85385E-08	1.67385E-07	7.50909E-08	0	6.88182E-08	0	4.32E-08	1.23533E-07	1.82173E-07	1.68933E-07
Paraburkholderia_tropica	4.53364E-07	4.00909E-08	1.19164E-07	1.15545E-07	6.46923E-08	1.06923E-07	2.61462E-07	1.67308E-07	2.62091E-07	5.67364E-07	5.71636E-07	5.97818E-07	0	0	8.92E-08	0
Paraburkholderia_hospita	1.02264E-06	2.15182E-07	1.37273E-07	0.00000056	1.15769E-07	1.59615E-07	4.76615E-07	3.62385E-07	4.05273E-07	6.85727E-07	6.59818E-07	3.13818E-07	9.96667E-08	0.00000007	1.518E-07	0.000000215
Paraburkholderia_sp._PGU16	0	0	0.000000054	5.94545E-08	0	0	0	1.87462E-07	0	0	2.05455E-08	4.65909E-08	0	0	0	7.22E-08
Paraburkholderia_sprentiae	2.93545E-07	0	0	1.87273E-07	2.13615E-07	5.33846E-07	1.57231E-07	6.10231E-07	2.20182E-07	9.72727E-08	3.59727E-07	2.88636E-07	1.79133E-07	0.000000119	2.42933E-07	4.312E-07
Paraburkholderia_caledonica	3.50636E-07	2.34545E-08	9.04545E-08	1.67364E-07	1.08538E-07	1.79231E-08	3.06923E-08	2.46923E-08	9.34545E-08	8.26364E-08	1.02727E-08	2.60273E-07	6.99333E-08	5.06E-08	1.46107E-07	1.39733E-07
Paraburkholderia_caribensis	1.22818E-07	0.000000065	3.64364E-07	7.67273E-08	0	0	3.36769E-07	5.92077E-07	2.25091E-07	2.99818E-07	4.50273E-07	4.97909E-07	6.61333E-08	2.30133E-07	1.39067E-07	2.82333E-07
Paraburkholderia_graminis	8.34727E-07	0.000000065	7.40909E-08	2.41273E-07	1.60923E-07	0	2.35462E-07	1.09723E-06	6.05909E-07	5.27727E-07	4.82091E-07	4.43455E-07	6.332E-07	4.28267E-07	3.42373E-07	0.000000406
Paraburkholderia_megapolitana	5.69091E-08	0	3.70909E-08	8.65182E-08	0	0	1.96615E-07	1.90769E-08	0	0	2.76364E-08	3.57273E-08	0	1.02333E-07	3.14667E-08	1.86E-08
Paraburkholderia_sp._7MH5	1.53382E-06	3.40364E-07	1.38391E-06	1.10255E-06	1.37385E-07	9.56154E-08	6.13077E-07	1.10792E-06	6.20636E-07	4.21545E-07	9.10364E-07	6.38182E-07	2.14E-08	2.67467E-07	2.3464E-07	1.852E-07
Paraburkholderia_sp._SOS3	4.81455E-07	0	8.75455E-09	2.02409E-06	0.000000156	2.22538E-06	0.00000556	4.18785E-06	7.39818E-07	7.73818E-07	0.000001125	1.63164E-06	4.024E-07	3.43867E-07	2.962E-07	0.000000179
Pandoraea_faecigallinarum	2.70909E-08	0	0	0	0	0	0	0	0	0	0	3.16364E-08	0	0	0	0
Pandoraea_fibrosis	0	0	0	1.68727E-07	2.88462E-08	5.74615E-07	8.69231E-08	3.56923E-08	3.75455E-08	0.000000344	3.07273E-08	8.00909E-07	2.14E-08	0.000000014	1.13267E-07	8.99333E-08
Pandoraea_sputorum	3.89727E-07	1.03636E-06	0	2.95091E-07	0	0	6.40769E-08	1.16277E-06	9.01818E-07	1.91455E-07	1.10909E-07	5.88909E-07	0	0	8.03733E-08	1.794E-07
Pandoraea_vervacti	2.48727E-07	8.12727E-08	4.10909E-08	9.03636E-08	2.30769E-07	3.87615E-07	0.000000243	3.04385E-07	2.29909E-07	1.60818E-07	2.64727E-07	3.35773E-07	2.39733E-07	2.126E-07	3.09467E-07	4.89467E-07
Pandoraea_thiooxydans	1.55091E-07	2.71818E-08	6.33636E-08	7.56364E-08	5.42308E-08	5.91538E-08	1.06615E-07	0.000000011	1.09364E-07	2.98636E-07	1.26545E-07	1.12591E-07	7.43333E-08	3.09333E-08	1.49733E-07	0.000000241
Pandoraea_norimbergensis	7.55455E-08	0.000000065	1.45036E-07	3.43364E-08	3.86923E-07	0	2.24692E-07	1.59615E-07	4.97455E-07	4.38182E-07	4.12727E-07	3.51273E-07	5.40933E-07	3.146E-07	6.16667E-07	0.00000099
Pandoraea_oxalativorans	7.75182E-07	0	1.77909E-06	2.05782E-06	4.59615E-07	4.77231E-07	4.67492E-06	5.74762E-06	1.14109E-06	1.21845E-06	1.08709E-06	7.74818E-07	5.96667E-07	3.832E-07	1.40413E-06	1.34493E-06
Pandoraea_sp._XY-2	7.88091E-07	1.34091E-07	9.88182E-08	9.92182E-07	7.04538E-07	4.13846E-08	6.23077E-08	3.15077E-07	1.01673E-06	5.53273E-07	8.56455E-07	0.000000618	8.804E-07	8.32467E-07	9.47067E-07	1.31287E-06
Pandoraea_pulmonicola	7.56364E-07	0	2.87727E-06	3.35482E-06	7.92154E-07	0	6.47231E-06	6.46538E-06	1.34273E-06	1.44955E-06	1.29482E-06	9.13364E-07	0.000000686	6.75133E-07	1.3562E-06	0.000000623
Pandoraea_pnomenusa	1.41273E-07	3.26227E-07	9.04545E-08	1.27818E-07	1.51769E-07	2.22385E-07	2.44615E-07	0	0.000000388	3.52364E-07	1.45182E-07	3.77364E-07	0	1.784E-07	4.46667E-08	0
Pandoraea_apista	1.05709E-06	7.66364E-08	7.40909E-08	3.27273E-07	1.15677E-06	6.67492E-06	1.97615E-06	0.000001864	1.05718E-06	1.61436E-06	1.06955E-06	1.34273E-06	1.7946E-06	1.13347E-06	2.41733E-06	2.24053E-06
Polynucleobacter_sp._MWH-UH21B	0	0	0	0	0	0	0	0.000000042	0	0	0	0	0	0.000000033	0	0
Polynucleobacter_paneuropaeus	0	0	0	0	0	0	0	0	0	0	0	1.42727E-08	0	0	0	0
Polynucleobacter_wuianus	0	0	0	0	0	0	0	0	5.97273E-08	0.000000034	0	5.37273E-08	0	0	0	0
Polynucleobacter_sp._LimPoW16	4.60909E-08	0	0	0	0	0	3.06923E-08	1.23692E-07	0.000000077	3.03636E-08	0	7.13636E-09	8.19333E-08	3.35333E-08	2.78E-08	1.054E-07
Polynucleobacter_duraquae	0	0	0	5.18182E-09	7.78462E-08	0	0	1.46923E-08	0	3.03636E-08	0	3.74545E-08	5.61333E-08	0	1.12667E-08	5.26667E-08
Polynucleobacter_acidiphobus	5.95455E-07	6.91636E-07	4.12182E-08	7.46091E-08	2.22769E-07	4.13846E-08	2.10769E-07	5.81538E-08	1.92091E-07	0.000000109	1.09818E-07	5.84455E-07	6.66E-08	5.71333E-08	2.008E-07	1.36133E-07
Polynucleobacter_difficilis	2.23909E-07	0.00000013	2.87273E-08	2.55182E-08	2.46538E-07	4.99385E-07	1.75231E-07	0.000000628	2.58909E-07	0.000000081	1.54364E-07	2.39727E-07	3.00867E-07	3.996E-07	7.06867E-07	8.98733E-07
Polynucleobacter_asymbioticus	7.66727E-07	3.24545E-07	1.28764E-07	2.74618E-06	5.29231E-07	3.43538E-07	7.16308E-07	2.18531E-06	3.13727E-07	5.21455E-07	8.43818E-07	1.58627E-06	4.85867E-07	8.64933E-07	1.11213E-06	1.2262E-06
Polynucleobacter_necessarius	0.000009	1.06364E-06	1.57413E-06	6.61545E-06	2.67285E-06	1.26385E-07	0.000000988	3.37208E-06	5.04091E-06	3.62818E-06	3.82364E-06	6.14036E-06	4.85067E-06	4.85133E-06	2.84913E-06	0.000003652
Burkholderia_metallica	2.30909E-08	0	0	0	0	0	0	0	0	0.000000056	0	0	0	0.000000014	0	0
Burkholderia_pyrrocinia	0	0	0	0	0	0	0	0	0	0	0	0	0	0	0	1.86E-08
Burkholderia_sp._BDU8	0	0	0	0	0	0	0	6.05385E-08	0	0	0	0	2.14E-08	0	0	0
Burkholderia_sp._BDU6	0	0	0	0	0	0	0	0	2.72727E-08	0	0	0	0	0	0	0
Burkholderia_sp._MSMB0266	0	0	0	0	0	0	0	0.000000042	0	0	0	1.47364E-07	0	0	0	0
Burkholderia_sp._RPE67	0	0	0	0	0	0	0	0	0	0	0	7.13636E-09	0	0	0	0
Burkholderia_sp._DHOD12	0	0	0	5.18182E-09	0	0	0	0	0	0.000000028	0	0	0	3.09333E-08	0	0
Burkholderia_vietnamiensis	0	0	0	1.93636E-08	2.59231E-08	5.09231E-08	0	0.000000011	0	0	0	0	0	0.000000014	0	1.66667E-08
Burkholderia_sp._MSMB0856	2.98182E-08	0	0	0	0	0	0	0	0	0	0	0	0	0	0	1.08133E-07
Burkholderia_sp._PAMC_26561	0	0	0	3.55182E-08	0	1.00677E-06	2.40385E-07	3.29231E-08	3.75455E-08	0	0	0	4.52E-08	1.71333E-08	6.91067E-08	0
Burkholderia_stagnalis	0	0.000000047	0	0	4.46154E-08	0	0	0	0	0	0	0	2.09333E-08	3.35333E-08	3.68E-08	8.74E-08
Burkholderia_stabilis	1.19273E-07	0	0	6.33636E-08	0	0	0	7.69231E-08	0	0	1.33636E-07	7.13636E-09	0	0.000000033	1.33333E-08	3.20667E-08
Burkholderia_sp._IDO3	5.57273E-08	0	0	0	0	0	0	0	0	7.57273E-08	1.30909E-07	3.94545E-08	0	0	0	0
Burkholderia_sp._KBS0801	0	0	0	0	0	0	0	0	0	5.73636E-08	1.30909E-07	2.26364E-07	0	0	0	0
Burkholderia_oklahomensis	6.74545E-08	0	0	1.93636E-08	0	0	0	0	0	0.000000034	0	0	6.08E-08	0.00000007	0	7.57333E-08
Burkholderia_sp._PAMC_28687	3.11818E-08	0.00000002	0	0	0	0	4.33846E-08	0	8.43636E-08	0	0	7.13636E-09	8.72E-08	0	0	5.32E-08
Burkholderia_contaminans	4.87273E-08	1.37273E-08	0	1.51545E-08	5.47692E-08	0	3.11538E-08	8.01538E-08	1.02636E-07	5.22727E-08	1.02727E-08	1.99091E-07	0.000000082	4.02E-08	4.20667E-08	0.000000118
Burkholderia_sp._YI23	1.12091E-07	0	0	0	5.20769E-08	0	0	0	0.000000027	0	2.76364E-08	7.13636E-09	0	1.61333E-08	0	3.67333E-08
Burkholderia_sp._Bp7605	2.70909E-08	3.66364E-08	0	0.000000066	1.71385E-07	6.58385E-07	1.72615E-07	2.29308E-07	7.15455E-08	0	1.14727E-07	1.46273E-07	7.16667E-08	2.06667E-07	3.35267E-07	3.75733E-07
Burkholderia_lata	5.69091E-08	1.04545E-08	0	9.54545E-09	3.15385E-08	0	3.06923E-08	0.000000011	0.000000191	5.67273E-08	2.23727E-07	0.000000116	4.32E-08	6.18E-08	2.55333E-08	1.98667E-08
Burkholderia_glumae	2.15273E-07	1.78227E-07	3.70909E-08	8.30909E-08	3.58462E-08	6.01769E-07	2.83154E-07	4.16615E-07	2.72727E-08	2.99727E-07	2.74818E-07	2.18455E-07	2.388E-07	9.72667E-08	3.75133E-07	0.000000326
Burkholderia_latens	1.58182E-08	0	0	0	0	0	0	0	0	0	0	0	0	0	0	0
Burkholderia_diffusa	1.58182E-08	0	0	0	0	0	0	0	0	0	3.77273E-08	0	0	0	0	0
Burkholderia_ambifaria	2.10091E-07	2.14545E-07	3.70909E-08	7.27273E-08	0.000000094	0	0	1.29615E-07	3.98182E-08	8.26364E-08	1.14727E-07	2.53364E-07	1.41067E-07	0.000000014	1.5824E-07	4.62667E-08
Burkholderia_ubonensis	4.56364E-08	0	0	0	0	1.42154E-07	3.06923E-08	0.000000011	0	2.85455E-08	6.68182E-08	3.74545E-08	5.86E-08	0	2.99733E-08	0
Burkholderia_cenocepacia	1.88909E-06	0.00000013	0	0	0	0	0	3.22308E-08	4.45455E-08	8.39091E-08	0	0	2.72E-08	0	0	0
Burkholderia_sp._JP2-270	2.56364E-08	0	0	0	9.34615E-08	0	0	0	0.000000084	5.83636E-08	3.33636E-08	6.01818E-08	4.73333E-08	0.000000014	6.384E-08	5.70667E-08
Burkholderia_sp._CCGE1003	7.26364E-08	0	0	9.97273E-09	1.68077E-07	4.25385E-07	0	0.000000165	1.12727E-07	0.000000075	1.44091E-07	3.95636E-07	0	0.000000028	0	1.43667E-07
Burkholderia_thailandensis	0.000000111	8.49091E-08	1.63636E-08	1.57727E-07	1.26462E-07	5.96923E-08	1.95846E-07	0.000000121	2.55909E-07	2.31455E-07	1.80182E-07	3.01727E-07	2.09333E-08	6.49333E-08	6.73733E-08	1.77733E-07
Burkholderia_pseudomallei	3.57455E-07	2.72727E-08	5.74545E-08	0.000000908	3.40462E-07	1.60815E-06	7.41846E-07	1.18623E-06	3.77727E-07	2.69091E-07	2.19455E-07	0.000000373	5.30733E-07	3.93733E-07	3.932E-07	8.48667E-07
Burkholderia_sp._THE68	5.57273E-08	0	0	3.24545E-08	3.15385E-08	6.76923E-08	2.14538E-07	2.00769E-07	7.62727E-08	3.89091E-08	8.26364E-08	2.43727E-07	4.79333E-08	8.15333E-08	1.43333E-07	1.42867E-07
Burkholderia_cepacia	0.000000518	8.34091E-08	2.51182E-08	0.000000465	0.000000105	4.13846E-08	1.24538E-07	1.79077E-07	2.41909E-07	9.36364E-08	2.81364E-07	2.77545E-07	1.40867E-07	8.43333E-08	5.06E-08	3.318E-07
Burkholderia_seminalis	1.58818E-07	4.65455E-08	1.77118E-07	1.63727E-07	6.33846E-08	1.25415E-06	0.000000217	2.50538E-07	0.000000224	3.40818E-07	8.93636E-08	2.68545E-07	0.000000141	9.35333E-08	2.7884E-07	2.50667E-07
Burkholderia_mallei	2.92545E-07	3.01818E-07	0	1.80818E-07	2.11462E-07	1.58154E-07	1.25692E-07	0.000000123	0.000000134	3.28909E-07	1.02727E-08	4.28182E-08	0.000000271	1.566E-07	5.97333E-08	0.000000874
Burkholderia_plantarii	0	0	0	8.40909E-08	0	0	0	2.46923E-08	0	0.000000028	0	8.29091E-08	2.24E-08	3.48E-08	1.49333E-08	0
Burkholderia_sp._KK1	3.43091E-07	0.000000065	7.82182E-08	2.10364E-07	3.88462E-08	1.63615E-07	0	6.45385E-08	4.31091E-07	2.23909E-07	3.33455E-07	1.22136E-07	3.59333E-07	2.33467E-07	2.358E-07	4.314E-07
Burkholderia_anthina	2.15091E-07	0	0	0.000000911	0.000000085	1.34615E-06	1.88469E-06	1.42369E-06	4.14091E-07	3.41273E-07	6.60273E-07	9.78045E-07	1.416E-07	1.29867E-07	2.18933E-07	7.18E-08
Burkholderia_multivorans	0.000000335	1.02455E-07	7.80727E-08	2.98909E-07	1.11154E-07	2.94462E-07	9.16769E-07	5.19769E-07	2.09182E-07	0.000000272	5.92727E-07	1.03818E-06	2.37333E-08	1.58733E-07	1.49507E-07	1.77733E-07
Burkholderia_gladioli	1.84436E-06	1.39545E-07	1.24182E-07	2.62636E-07	3.32415E-06	6.06462E-07	1.62231E-07	1.51308E-07	1.95618E-06	1.65873E-06	8.61727E-07	2.00391E-06	2.28667E-06	2.19467E-06	1.68033E-06	2.30633E-06
Kordiimonas_pumila	0	0	0	3.24545E-08	2.05385E-08	0	0	0	2.64545E-08	0	0	0	0	0	0	0
Candidatus_Puniceispirillum_marinum	2.30909E-08	4.75909E-08	0	5.18182E-09	2.05385E-08	0	0	5.72308E-08	1.12727E-07	6.48182E-08	0.000000039	9.31818E-08	4.51333E-08	0	3.52E-08	2.29333E-07
Parvularcula_bermudensis	4.77455E-07	5.73273E-07	1.70727E-07	6.22455E-07	1.15231E-07	0	0.000000134	2.53538E-07	2.57818E-07	0.000000028	7.90909E-08	1.04364E-07	2.188E-07	1.15867E-07	2.05507E-07	1.302E-07
Polymorphum_gilvum	4.02455E-07	4.09818E-07	1.83364E-07	4.88455E-07	1.26615E-07	7.21538E-08	1.28077E-07	9.53077E-07	5.75909E-07	1.53091E-07	6.53273E-07	3.48818E-07	1.606E-07	3.48867E-07	2.42267E-07	0.000000689
Micavibrio_aeruginosavorus	2.46618E-06	7.65682E-07	2.16236E-06	0.00000159	0.000000255	2.05462E-07	5.24077E-07	5.83231E-07	9.57364E-07	9.16182E-07	1.74527E-06	1.33291E-06	1.118E-07	1.69267E-07	2.01867E-07	3.09733E-07
Phreatobacter_stygius	5.36727E-07	1.06091E-07	2.59091E-07	6.67273E-08	7.48462E-08	0.000000026	2.30769E-07	0.000000011	5.29636E-07	7.67364E-07	8.26545E-07	8.87545E-07	0.000000286	8.60667E-08	1.76667E-07	3.32333E-07
Phreatobacter_sp._NMCR1094	3.69091E-08	0	3.36855E-07	1.79636E-07	6.46923E-08	0	7.92308E-08	0	1.14909E-07	2.85455E-08	0.000000078	1.14545E-07	0	8.44667E-08	6.91067E-08	1.022E-07
Phreatobacter_cathodiphilus	8.28182E-08	1.16364E-08	0	5.16364E-08	2.75385E-08	0.000000167	2.21231E-07	1.50615E-07	0	0	0	1.04091E-07	0	1.00867E-07	0	9.33333E-09
Magnetococcus_marinus	1.51273E-07	1.22273E-07	8.22636E-08	4.82727E-08	2.25231E-07	1.86154E-07	5.96154E-08	2.97692E-08	2.64545E-08	0.000000034	1.06727E-07	1.81727E-07	1.44133E-07	4.694E-07	4.74867E-07	2.23333E-07

Candidatus_Paracaedibacter_acanthamoebae	0	0	0	0	0	0	0	0	0	0	6.88182E-08	0	0	0	0	0
Candidatus_Nesciobacter_abundans	0	0	0	0	0	2.13846E-08	0	0	0	0	0	0	2.14667E-08	0	8.86667E-09	2.15333E-08
endosymbiont_of_Acanthamoeba_sp._UWC8	1.58545E-07	0	1.80909E-07	1.48727E-07	7.07692E-08	0	2.63077E-08	0.000000068	1.14545E-07	1.19182E-07	1.02727E-08	8.36364E-08	2.14E-08	1.67333E-07	1.12667E-08	3.72E-08
Candidatus_Cytomitobacter_primus	0	0	1.63636E-08	1.43636E-08	1.02231E-07	1.39846E-07	2.73846E-08	5.45385E-08	1.17636E-07	0	6.68182E-08	1.26818E-07	6.62667E-08	1.28533E-07	1.8924E-07	6.81333E-08
Candidatus_Cytomitobacter_indipagum	1.70255E-06	0.00000026	7.83364E-07	1.57455E-06	7.72308E-08	0	1.61923E-06	1.23954E-06	6.31182E-07	1.03427E-06	7.94818E-07	6.93091E-07	3.00333E-07	3.628E-07	4.21667E-07	3.43267E-07
Candidatus_Nucleicultrix_amoebiphila	0	0	0	0	1.56077E-07	0	0	6.83077E-07	3.98182E-08	0	0	0	1.46733E-07	3.83267E-07	3.278E-07	5.186E-07
Emcibacter_sp._MEBiC09520	3.45636E-07	3.68182E-08	1.15036E-07	7.56455E-07	5.37769E-07	2.38592E-06	6.30615E-07	8.12615E-07	0.000000212	4.77455E-07	3.02727E-07	3.97909E-07	2.582E-07	4.82133E-07	8.43667E-07	4.846E-07
Emcibacter_congregatus	1.73655E-06	2.00636E-06	4.78545E-07	1.59327E-06	5.28154E-07	2.76354E-06	7.49615E-07	1.72208E-06	1.56755E-06	9.25091E-07	1.11709E-06	1.80209E-06	0.000000707	1.13247E-06	1.42413E-06	1.13613E-06
Candidatus_Fonsibacter_ubiquis	3.99727E-07	8.43182E-08	1.53427E-07	1.87091E-07	4.75385E-08	0	0.000000282	3.07615E-07	1.12636E-07	3.43636E-07	4.81273E-07	1.58364E-07	2.64667E-08	1.684E-07	1.41733E-07	2.59067E-07
Candidatus_Pelagibacter_giovannonii	0	0	3.70909E-08	4.68818E-08	2.05385E-08	0	0	0	0	0.000000116	0	4.28182E-08	2.91333E-08	0	0	0
Candidatus_Pelagibacter_sp._HIMB1321	2.30909E-08	0	1.63636E-08	4.20909E-08	2.85692E-07	0	0	7.86923E-08	5.50909E-08	9.11818E-08	6.65455E-08	1.68909E-07	7.52667E-08	6.18E-08	2.55333E-08	4.95333E-08
Candidatus_Pelagibacter_sp._RS40	1.03364E-07	1.05273E-07	4.90909E-08	8.23636E-08	0	0	1.13231E-07	0	9.12727E-08	1.09727E-07	9.44545E-08	3.94545E-08	2.91333E-08	0.000000033	1.682E-07	0
Candidatus_Pelagibacter_sp._RS39	2.05909E-07	0.000000065	0	4.50909E-08	1.08538E-07	0	1.78462E-07	3.22308E-08	0	0	1.02727E-08	8.45727E-07	2.08E-08	1.70933E-07	0.000000268	1.50067E-07
Candidatus_Pelagibacter_sp._FZCC0015	1.33818E-07	0.00000013	4.81818E-07	1.68091E-07	2.88462E-08	0	9.88462E-08	2.20385E-07	0	0	4.52909E-07	1.89455E-07	4.23333E-08	9.45333E-08	8.12E-08	0
Candidatus_Pelagibacter_ubique	6.24545E-08	0	8.25455E-09	4.79091E-09	6.23077E-08	2.95385E-08	0	6.58462E-08	0	0	3.33636E-08	1.22818E-07	1.58333E-07	2.75133E-07	8.35333E-08	1.29133E-07
Candidatus_Pelagibacter_sp._IMCC9063	2.54818E-07	0	8.75455E-09	1.14909E-07	2.22077E-07	5.05385E-08	1.57846E-07	5.55538E-07	1.83909E-07	3.53818E-07	5.99545E-07	1.32455E-07	4.262E-07	5.89133E-07	1.82667E-07	4.424E-07
Terricaulis_silvestris	0	0	0	9.97273E-09	4.93846E-08	4.27692E-08	6.41538E-08	6.32308E-08	0	0	7.85455E-08	2.14545E-08	2.14E-08	2.38E-08	0	9.33333E-09
Caulobacter_segnis	0	0	0	0	0	0	0	0	0	2.85455E-08	3.01364E-07	7.13636E-09	0	4.80667E-08	0	1.86E-08
Caulobacter_sp._FWC26	1.04909E-07	1.40182E-07	1.63636E-08	4.02727E-08	2.75385E-08	1.54308E-07	1.95385E-07	0.000000042	7.18182E-08	0.000000047	0	3.57273E-08	1.53133E-07	0.000000041	7.43333E-08	6.94E-08
Caulobacter_rhizosphaerae	2.22545E-07	6.79091E-08	3.70909E-08	9.21818E-08	3.15385E-08	7.29231E-08	0	0	5.79091E-08	0	2.54545E-07	2.49727E-07	0	5.13333E-08	7.87733E-08	8.19333E-08
Caulobacter_sp._Ji-3-8	1.84273E-07	0	0	5.18182E-09	6.17692E-08	0	7.92308E-08	1.46923E-08	0.000000114	8.59091E-08	1.85273E-07	1.90727E-07	1.33133E-07	0.000000088	1.14173E-07	1.03933E-07
Caulobacter_sp._K31	1.40727E-07	6.73636E-08	0	1.91636E-07	1.32077E-07	3.11923E-07	1.94077E-07	4.58154E-07	3.98182E-08	5.22727E-08	6.66364E-08	7.78182E-08	6.95333E-08	1.04533E-07	1.12667E-07	1.12867E-07
Caulobacter_flavus	7.26455E-07	4.11818E-08	0.000000763	7.78909E-07	3.51923E-07	0	0.000000683	4.79769E-07	6.96182E-07	6.63364E-07	7.68091E-07	3.74636E-07	4.06733E-07	3.708E-07	2.844E-07	4.132E-07
Caulobacter_vibrioides	3.35818E-07	9.38636E-07	3.34818E-08	3.27455E-07	3.15385E-08	0	0	8.23077E-08	0.000000064	1.61273E-07	8.29091E-08	1.32182E-07	9.07333E-08	1.672E-07	1.44307E-07	1.742E-07
Caulobacter_henricii	2.75273E-07	0	4.52182E-08	2.32064E-07	3.34615E-08	3.59231E-07	1.51538E-07	0.000000011	1.59727E-07	3.62727E-08	3.31818E-07	2.21636E-07	7.18667E-08	1.58667E-07	8.57067E-08	5.06667E-08
Caulobacter_mirabilis	3.84182E-07	1.94545E-07	4.14455E-07	2.81182E-07	1.08769E-07	8.89077E-07	0	2.84615E-07	3.60727E-07	1.04273E-07	2.30636E-07	0.000000782	1.16333E-07	1.012E-07	1.53133E-07	9.30667E-08
Asticcacaulis_excentricus	1.05727E-06	2.11364E-07	1.88518E-07	7.28273E-07	7.85769E-07	3.33231E-07	2.25308E-07	6.14538E-07	4.12727E-07	5.02455E-07	9.71273E-07	1.81691E-06	1.40467E-07	5.208E-07	5.422E-07	7.26533E-07
Phenylobacterium_sp._HYN0004	1.06736E-06	1.41291E-06	2.20545E-07	7.49545E-07	2.02077E-07	9.05385E-08	1.50846E-07	7.99385E-07	4.05909E-07	1.79545E-07	3.33727E-07	3.14545E-07	1.926E-07	3.526E-07	3.624E-07	1.65733E-07
Phenylobacterium_zucineum	3.79455E-07	0.000000065	3.93636E-07	4.42727E-08	4.72231E-07	1.93292E-06	5.66154E-07	3.67462E-07	2.16364E-08	1.85909E-07	1.54818E-07	4.50091E-07	3.50667E-07	3.13467E-07	3.634E-07	4.06267E-07
Brevundimonas_sp._GW460-12-10-14-LB2	0	0	0	0	0	2.13846E-08	1.10769E-07	2.97692E-08	3.98182E-08	0	0	0	0	0	0	0
Brevundimonas_sp._SGAir0440	0	0	0	3.72455E-08	6.73846E-08	0	2.73846E-08	5.81538E-08	4.45455E-08	8.02727E-08	1.30909E-07	2.85455E-08	9.01333E-08	3.26667E-08	1.99067E-07	2.42867E-07
Brevundimonas_sp._M20	2.98182E-08	0	0	0	0	3.01538E-07	0	0	2.01182E-07	5.22727E-08	3.79091E-08	1.47455E-07	0	0	0	0
Brevundimonas_vesicularis	3.11818E-08	9.54545E-09	0	9.97273E-09	0	0	0	0	3.98182E-08	0	3.77273E-08	4.59091E-08	0	3.09333E-08	0	0
Brevundimonas_mediterranea	9.68182E-08	0	3.70909E-08	4.37273E-08	0	3.83077E-08	8.92308E-08	0	2.75455E-08	1.38545E-07	0	5.89091E-07	0	0.000000014	1.0684E-07	9.12E-08
Brevundimonas_vancanneytii	8.69091E-08	0	1.63636E-08	7.45455E-08	0	0	3.77692E-08	5.22308E-08	2.75455E-08	0.000000081	1.70455E-07	0.00000005	0	4.76667E-08	2.01333E-08	1.26667E-07
Brevundimonas_diminuta	4.60909E-08	0	6.33636E-08	0	0	3.15385E-08	2.63077E-08	0.000000022	0	0	0	0	0	0	0	0
Brevundimonas_sp._'scallop'	0	0	0	3.24545E-08	8.03846E-08	2.90769E-07	0	0.000000042	5.79091E-08	0	1.19818E-07	0	4.33333E-08	0	1.18867E-07	8.08667E-08
Brevundimonas_sp._DS20	5.42727E-08	0	0	5.18182E-09	3.58462E-08	0	4.33846E-08	0	5.91818E-08	0	0.000000078	5.64545E-08	2.14E-08	0.000000033	1.12667E-08	0
Brevundimonas_sp._LM2	0.000000154	2.70909E-08	2.18545E-08	1.75791E-07	0	9.81538E-08	4.33846E-08	5.81538E-08	1.11909E-07	8.08182E-08	1.27636E-07	3.82364E-07	1.132E-07	5.53333E-08	8.72E-08	7.08E-08
Brevundimonas_subvibrioides	1.08182E-07	0.00000013	6.14545E-08	1.80818E-07	9.32308E-08	0	3.06923E-08	0	1.17636E-07	5.22727E-08	1.07818E-07	1.04636E-07	8.14E-08	6.74667E-08	0.000000041	6.52E-08
Brevundimonas_naejangsanensis	1.14945E-06	0	7.82182E-08	5.98182E-07	3.61923E-07	3.63077E-07	4.77385E-07	1.95738E-06	8.22091E-07	5.72818E-07	1.18209E-06	7.12636E-07	4.88333E-07	6.31133E-07	4.78067E-07	0.000000882
Brevundimonas_sp._MF30-B	0	0	0	0	2.05385E-08	0	0	0.000000011	0	0	0	0	4.88667E-08	6.93333E-08	7.06667E-08	4.02E-08
Rickettsiales_endosymbiont_of_Stachyamoeba_lipophor	0	0	1.77273E-08	2.43636E-08	0.000000039	0	0	1.55923E-07	0	0	1.30909E-07	0	4.84667E-08	3.22667E-08	7.804E-08	0.000000145
Candidatus_Deianiraea_vastatrix	6.11818E-08	4.02727E-08	4.12727E-09	1.18064E-07	8.55385E-08	0	2.63077E-08	3.22308E-08	0	6.37273E-08	1.21818E-07	5.50455E-08	0	6.86E-08	8.16667E-08	4.90667E-08
Candidatus_Fokinia_solitaria	9.11818E-08	0	0	5.18182E-09	2.26923E-08	2.13846E-08	0	5.81538E-08	8.91818E-08	5.22727E-08	0	4.59091E-08	1.86667E-08	8.38667E-08	0.000000067	1.17067E-07
Candidatus_Midichloria_mitochondrii	0.000000047	0	4.12727E-09	1.55455E-08	0	0	7.54615E-08	4.93846E-08	2.72727E-08	5.83636E-08	0	6.09091E-08	2.81667E-07	1.02333E-07	1.16267E-07	1.38733E-07
Candidatus_Sneabacter_namystus	0	0	0	0	2.05385E-08	0	0	1.33692E-07	0	0	0	0	0	1.418E-07	5.61333E-08	7.52E-08
Candidatus_Phycorickettsia_trachydisci	8.01636E-07	0.000000065	0	5.06727E-07	3.29769E-07	0	3.77692E-08	3.51615E-07	5.40182E-07	0	1.29191E-06	3.56773E-07	2.61533E-07	4.59133E-07	5.372E-07	3.24133E-07
Orientia_tsutsugamushi	6.84182E-07	2.39727E-07	4.12727E-09	3.84909E-07	1.39992E-06	8.92308E-08	1.38692E-07	2.08769E-07	1.44545E-07	1.04545E-07	3.96364E-07	6.48727E-07	6.33267E-07	8.86733E-07	1.08777E-06	5.53667E-07
Rickettsia_africae	0	0	0	0	0	0	0	0	0	0	0	0	0	0.00000007	0	0
Rickettsia_prowazekii	0	0	0	0	3.58462E-08	0	0	3.22308E-08	0	9.40909E-08	0	0	0	0	0	1.23733E-07
Rickettsia_akari	0	0	0	0	2.26923E-08	0	0	1.90769E-08	0	0	0	0	0	0	0	0
Rickettsia_amblyommatis	2.96364E-08	0	0	0	0	0	0	0	0	0	0	7.13636E-09	0	0	0	0
Rickettsia_endosymbiont_of_Bemisia_tabaci	0	0	0	0	0	0	0	0	0	0	0	0	0	9.93333E-08	2.99333E-08	0
Rickettsia_asiatica	0	0	0	0	0	0	0	0	0	0	0	0	0	0	4.44E-09	0
Rickettsia_felis	0	0	0	0	5.63846E-08	5.05385E-08	0	1.04615E-07	9.85455E-08	0	9.41818E-08	7.13636E-09	0	0	1.49333E-08	3.67333E-08
Rickettsia_typhi	1.04873E-06	0.000000065	2.22727E-07	4.01273E-07	5.51615E-07	0	0	5.55385E-07	6.94273E-07	3.15273E-07	1.34427E-06	1.37827E-06	2.87933E-07	1.91133E-07	8.20733E-07	4.576E-07
Rickettsia_australis	9.79091E-08	0	2.04909E-08	3.07273E-08	0	1.28308E-07	1.00077E-07	6.32308E-08	0	0.000000034	0	0	1.67333E-07	3.09333E-08	0	0
Rickettsia_canadensis	5.57273E-08	0	0	8.49636E-07	8.49923E-07	0	4.67769E-07	7.11308E-07	1.17636E-07	1.98273E-07	6.88182E-08	3.06364E-07	0.000000913	1.07873E-06	1.5056E-06	2.2924E-06
Rickettsia_bellii	4.38818E-07	0	0	0	5.87538E-07	0	0	0	0.000000027	0	0	7.13636E-09	3.204E-07	5.67067E-07	9.84867E-07	4.86067E-07
Neorickettsia_helminthoeca	5.12727E-08	0	0	0	0	0	0	0	0	0	0	0	0	0	0	0
Neorickettsia_sennetsu	0	0	0	5.18182E-09	0	0	3.11538E-08	0	0	0	0	0	0	0	0	0
Anaplasma_platys	0	0	0	0	0	0	0	0	0	0	0	0	0	0	0	1.86E-08
Anaplasma_marginale	1.19091E-07	2.05E-08	2.62727E-08	1.25182E-07	0	0	0	0	0	0	0	0	0	0	0	0
Anaplasma_phagocytophilum	1.41091E-07	2.29636E-07	2.59818E-08	3.55182E-08	5.21538E-08	0	0	0	8.67273E-08	1.68273E-07	1.98091E-07	1.04091E-07	4.48667E-08	4.76667E-08	4.44E-09	2.25733E-07
Anaplasma_ovis	6.65455E-08	3.49091E-08	6.99309E-07	2.61455E-07	0	0	0	7.63077E-08	0	0.000000028	0	2.95455E-07	6.94E-08	9.42667E-08	8.784E-08	0.000000026
Ehrlichia_canis	3.11818E-08	9.54545E-09	0	2.39091E-08	0	0	0	4.69231E-08	0	0	0	0	0	0	0	5.52E-08
Ehrlichia_sp._HF	3.11818E-08	0	7.40909E-08	4.20909E-08	0	0	0	0	7.97273E-08	0	0	9.03636E-08	0	0	0	0
Ehrlichia_muris	0	0	5.93636E-08	3.07273E-08	0	0	0	0	0	2.85455E-08	0	0	0	3.48E-08	1.49333E-08	1.084E-07
Ehrlichia_chaffeensis	0	0	0	0	6.73846E-08	0	0	4.35231E-07	3.94545E-08	0	0	6.41364E-08	0.000000233	4.128E-07	1.478E-07	2.26733E-07
Ehrlichia_ruminantium	8.76455E-07	0	4.50364E-07	0.000002031	1.27831E-06	3.83077E-08	1.06762E-06	1.52423E-06	2.54273E-07	3.98182E-07	6.13182E-07	9.82273E-07	1.8544E-06	1.86367E-06	2.79173E-06	3.60167E-06
Wolbachia_endosymbiont_of_Dipetalonema_caudispina	0	0	0	0	0	7.21538E-08	0	0	0	0	0	0	0	0	0	0
Wolbachia_pipientis	2.98182E-08	0	0	0	0	0	7.54615E-08	2.46923E-08	0	0	0	0	0	3.50667E-08	0	0
Candidatus_Wolbachia_massiliensis	0	0	0	0	0	0	0	0	0	0	0	2.14545E-08	2.64E-08	6.80667E-08	7.43333E-08	0
Wolbachia_endosymbiont_of_Cimex_lectularius	2.56364E-08	0	0	0	1.99462E-07	0	2.63077E-08	0	1.17636E-07	0	1.35636E-07	3.78636E-07	1.40333E-07	1.19333E-07	5.35867E-07	1.412E-07
Wolbachia_endosymbiont_of_Cruorifilaria_tuberocauda	0	0	0	2.43636E-08	0	0	0	0.000000042	5.97273E-08	0	2.05455E-08	1.42727E-08	2.72E-08	0	0	0
Wolbachia_endosymbiont_of_Chrysomya_megacephala	0	0	3.70909E-08	7.96091E-08	3.14231E-07	0	1.71846E-07	9.77692E-08	0	5.22727E-08	6.88182E-08	0	2.70533E-07	1.67133E-07	9.73067E-08	2.292E-07
Wolbachia_endosymbiont_of_Folsomia_candida	1.43364E-07	0	2.22727E-07	6.22727E-08	2.05385E-08	0	1.24077E-07	2.74462E-07	0	0	8.29091E-08	1.36273E-07	2.64E-08	1.00933E-07	1.976E-07	6.62667E-08
Wolbachia_endosymbiont_of_Ctenocephalides_felis_wC	1.11273E-07	0	0	1.08364E-07	0	0	6.13846E-08	1.41385E-07	1.76182E-07	7.25455E-08	6.66364E-08	5.27545E-07	6.33333E-08	3.50667E-08	8.87333E-08	1.38133E-07
Wolbachia_endosymbiont_of_Litomosoides_sigmodontis	3.11818E-08	9.54545E-09	3.70909E-08	0	3.15385E-08	0	0	1.10385E-07	1.02636E-07	8.53636E-08	1.80182E-07	0	7.13333E-08	0	1.93733E-08	0
Wolbachia_endosymbiont_of_Dirofilaria_(Dirofilaria)_imr	1.25818E-07	0.000000065	4.12727E-08	1.47545E-07	0	4.28385E-07	2.52769E-07	3.47538E-07	0	1.96182E-07	2.76364E-08	0	4.68E-08	1.71333E-08	5.75333E-08	6.33333E-08
Wolbachia_endosymbiont_of_Ctenocephalides_felis_wC	0	0	0	0	3.58462E-08	0	2.73846E-08	0.000000361	3.75455E-08	5.22727E-08	0	0.000000057	8.72E-08	1.02333E-07	1.19267E-07	9.93333E-08
Candidatus_Endolissoclinum_faulkneri	2.96364E-08	0	0	0	3.58462E-08	0	3.11538E-08	0	2.72727E-08	0.000000034	3.77273E-08	1.42727E-08	0	4.64667E-08	5.94E-08	0
Nitrospirillum_amazonense	0.000000495	0	1.03727E-07	2.84091E-07	3.84769E-07	0.000000272	2.93185E-06	1.83869E-06	4.84091E-07	0.000000318	2.67273E-07	5.61455E-07	4.64733E-07	8.61933E-07	7.48733E-07	3.45133E-07
Defluviicoccus_vanus	4.23909E-07	4.06818E-07	1.14545E-07	5.12909E-07	7.72308E-08	0	1.40769E-07	2.64154E-07	0	7.77273E-08	3.16727E-07	4.28182E-08	2.032E-07	1.336E-07	2.04733E-07	1.078E-07
Magnetospira_sp._QH-2	8.99273E-07	6.91591E-07	2.33273E-07	7.21455E-07	6.84077E-07	1.51385E-07	1.14462E-06	5.21462E-07	0.000000873	4.94636E-07	9.00273E-07	6.51091E-07	3.80933E-07	7.882E-07	4.65533E-07	4.19133E-07
Thalassospira_xiamenensis	0	2.34545E-08	9.51818E-08	4.33364E-08	0.000000039	0.00000009	2.51231E-07	2.41846E-07	2.75455E-08	0	9.64545E-08	0	9.95333E-08	2.62E-08	7.09333E-08	1.43867E-07
Thalassospira_marina	2.39182E-07	1.55136E-07	0.000000225	2.94545E-07	0	0	2.63077E-08	1.90769E-08	1.29364E-07	0	3.64727E-07	1.54045E-07	2.37333E-08	2.62E-08	3.41733E-08	0.000000102
Thalassospira_indica	1.78445E-06	7.32727E-07	6.24355E-07	1.61591E-06	3.31769E-07	4.82077E-07	7.10385E-07	2.85692E-07	8.66727E-07	4.01273E-07	1.07318E-06	5.89909E-07	3.59467E-07	0.000000367	1.65133E-07	4.83333E-07
Niveispirillum_cyanobacteriorum	2.03273E-07	3.97091E-07	2.46455E-07	4.16273E-07	1.38615E-07	1.56154E-07	6.06923E-08	1.35154E-07	2.59909E-07	3.08545E-07	6.60273E-07	1.01764E-06	1.12667E-07	0.000000047	4.102E-07	8.85333E-08
Hypericibacter_adhaerens	0	1.03636E-08	4.12727E-09	1.65273E-07	3.58462E-08	0	0	0	2.64545E-08	7.25455E-08	0	0	2.14E-08	7.11333E-08	5.94667E-08	0
Hypericibacter_terrae	1.03827E-06	8.86364E-09	3.70909E-08	7.05909E-07	3.39923E-07	0	7.92308E-08	3.81538E-08	3.08182E-07	2.95273E-07	4.36273E-07	5.35818E-07	1.09067E-07	2.08667E-07	2.56267E-07	1.336E-07
Pararhodospirillum_photometricum	1.36818E-07	3.23182E-08	4.50909E-08	1.63818E-07	0	2.16923E-08	0	0.000000042	2.72727E-08	0	3.79091E-08	3.57273E-08	1.04133E-07	6.57333E-08	0	9.50667E-08
Skermanella_pratensis	3.55727E-06	1.02864E-06	2.88421E-06	2.91264E-06	2.90308E-07	0.000000196	2.79392E-06	2.83177E-06	1.53691E-06	1.62045E-06	1.95755E-06	2.56327E-06	1.09667E-07	5.28933E-07	5.05907E-07	4.086E-07
Tistrella_mobilis	9.05364E-07	0	8.64545E-08	6.50727E-07	3.71308E-07	1.22423E-06	1.21615E-07	2.03846E-07	4.21727E-07	2.84909E-07	4.84364E-07	7.58091E-07	6.72467E-07	5.67467E-07	5.68733E-07	5.35133E-07
Indioceanicola_profundi	1.07091E-06	8.21727E-07	1.67164E-07	9.37364E-07	2.45154E-07	3.52615E-07	1.83385E-07	9.45769E-07	4.24364E-07	4.26273E-07	6.93818E-07	6.14091E-07	4.97867E-07	3.43733E-07	5.29933E-07	8.33933E-07
Haematospirillum_jordaniae	4.21418E-06	3.24545E-07	4.81818E-07	1.93736E-06	2.97885E-06	2.11846E-07	1.40462E-07	1.85115E-06	3.26891E-06	2.24818E-06	9.94727E-06	2.51364E-06	1.5088E-06	0.000001878	3.20067E-06	1.80193E-06
Rhodospirillum_centenum	3.37182E-07	8.71818E-07	2.81891E-07	6.22455E-07	2.04692E-07	0	3.87154E-07	0	0.00000012	1.96636E-07	4.36818E-07	5.03273E-07	9.56E-08	2.26733E-07	7.76667E-08	1.512E-07
Rhodospirillum_rubrum	3.53636E-07	1.21364E-07	1.41436E-07	5.17636E-07	3.09538E-07	1.08308E-07	2.05231E-07	3.12846E-07	6.87818E-07	5.60455E-07	0.000000577	1.36618E-06	2.202E-07	5.19333E-07	2.97933E-07	3.47667E-07
Ferrovibrio_terrae	4.04273E-06	1.76364E-06	1.03073E-06	0.00000509	1.03754E-06	7.82462E-07	4.24662E-06	4.72885E-06	1.60427E-06	1.72664E-06	4.79682E-06	4.24182E-06	6.46133E-07	1.4944E-06	1.47427E-06	9.602E-07
Magnetospirillum_magneticum	7.71455E-07	3.24545E-07	1.78364E-07	6.90182E-07	8.17692E-08	4.45231E-07	3.43308E-07	1.26615E-07	2.74182E-07	8.85455E-08	2.58091E-07	3.63091E-07	2.45333E-07	2.66467E-07	3.308E-07	2.82133E-07
Magnetospirillum_sp._ME-1	1.19273E-07	0	3.70909E-08	4.71818E-08	5.21538E-08	5.53692E-07	8.28462E-08	4.69231E-08	2.04273E-07	2.86727E-07	5.88455E-07	2.26455E-07	1.51667E-07	0	4.44E-09	6.33333E-08
Magnetospirillum_sp._XM-1	2.46364E-07	3.72818E-07	5.04545E-08	2.53273E-07	3.15385E-08	1.06769E-07	3.11538E-08	1.97538E-07	2.71909E-07	2.24909E-07	2.83455E-07	3.71909E-07	5.88667E-08	1.96267E-07	4.49067E-08	6.93333E-08
Magnetospirillum_gryphiswaldense	4.97273E-07	2.44545E-07	1.974E-07	1.36645E-06	4.31538E-07	1.50154E-06	4.23292E-06	2.23523E-06	0.000000554	1.11282E-06	0.000001146	1.08109E-06	9.54667E-08	4.73733E-07	4.25467E-07	1.12867E-07
Azospirillum_sp._TSH58	0	0	0	1.91818E-08	0	2.13846E-08	0	0	0	0	0	0	0	0	0	0
Azospirillum_humicireducens	2.98182E-08	0	0	0	0	0	0	0	0	0	0	7.13636E-09	0	0	0	0
Azospirillum_baldaniorum	0.000000092	1.03636E-08	3.70909E-08	9.54545E-09	3.58462E-08	2.16923E-08	0	0	6.50909E-08	6.37273E-08	6.88182E-08	1.42727E-08	0	1.58E-08	0	0
Azospirillum_thiophilum	3.89091E-08	4.02727E-08	0	1.91818E-08	2.05385E-08	3.20846E-07	3.11538E-08	5.22308E-08	0.000000027	5.22727E-08	3.33636E-08	0.00000005	6.64667E-08	1.58E-08	2.28467E-07	1.86E-08
Azospirillum_sp._TSA2s	0	0	4.12727E-09	4.79091E-09	1.86923E-08	0	0	2.84154E-07	0	7.55455E-08	0	0.000000057	5.04E-08	1.24133E-07	2.99733E-08	1.86667E-08
Azospirillum_sp._TSH100	4.45455E-07	7.54818E-07	2.56218E-07	1.55991E-06	2.34846E-07	2.13938E-06	4.62331E-06	2.73577E-06	6.54909E-07	7.50909E-07	1.61745E-06	1.75427E-06	4.70533E-07	5.87733E-07	6.692E-07	8.88867E-07
Azospirillum_brasilense	1.20391E-06	2.43818E-07	1.10891E-07	7.90545E-07	7.15154E-07	5.73538E-07	7.57769E-07	1.17908E-06	6.21818E-07	5.68273E-07	8.43273E-07	1.77427E-06	7.376E-07	2.42533E-07	6.52467E-07	6.76333E-07
Azospirillum_ramasamyi	2.70909E-08	0	8.31818E-09	2.87273E-08	0	0	6.41538E-08	3.83077E-07	0	1.22636E-07	1.72455E-07	2.14545E-08	0	0	6.83333E-08	1.27067E-07
Azospirillum_lipoferum	1.31255E-06	4.05182E-07	1.60332E-06	1.79173E-06	1.07538E-07	3.37385E-07	8.37692E-08	0.000000294	5.74909E-07	6.60818E-07	8.70727E-07	8.20091E-07	4.01333E-08	8.61333E-08	2.97333E-08	1.40467E-07
Azospirillum_oryzae	7.05727E-07	0	0	0.000000786	2.05385E-08	0	6.41538E-08	2.24692E-07	2.61909E-07	2.29455E-07	2.49273E-07	3.16727E-07	1.516E-07	3.11467E-07	2.336E-07	4.876E-07
Azospirillum_thermophilum	3.21636E-07	2.80545E-07	3.68545E-08	1.77545E-07	8.79231E-08	4.03077E-08	2.40231E-07	1.49538E-07	2.36545E-07	9.17273E-08	2.06909E-07	1.32655E-06	1.47667E-07	3.72733E-07	2.82373E-07	1.30133E-07
Oecophyllibacter_saccharovorans	0	1.03636E-08	0	1.91818E-08	0	0	5.36923E-08	0	2.33364E-07	0	3.95455E-08	0	2.64E-08	0	0	0
Acidibrevibacterium_fodinaquatile	2.01455E-07	0	3.70909E-08	3.12064E-07	3.39462E-07	2.89846E-07	1.98846E-07	1.78769E-07	7.15455E-08	1.16273E-07	3.95455E-08	2.19091E-07	1.35267E-07	1.29333E-07	2.298E-07	5.28067E-07
Bombella_sp._KACC_21507	2.98182E-08	1.04545E-08	0	3.85455E-08	2.26923E-08	2.52308E-08	0	0	4.86364E-08	6.69091E-08	3.33636E-08	7.13636E-09	2.64667E-08	0.000000014	2.01333E-08	8.36E-08
Roseococcus_sp._NIBR12	2.19545E-07	4.51818E-08	1.00455E-07	2.60609E-07	9.33077E-08	2.71231E-07	1.48308E-07	2.41462E-07	3.64818E-07	2.50909E-07	3.08636E-07	8.20636E-07	5.09333E-08	4.90667E-08	1.31667E-07	0
Neokomagataea_tanensis	1.52545E-07	4.67091E-07	1.17855E-07	4.68818E-08	6.23077E-08	0	3.33077E-08	2.12308E-07	0	6.37273E-08	0	2.78182E-07	2.14E-08	0	0	0
Asaia_bogorensis	0	0	2.07636E-08	2.07273E-07	0	0	3.77692E-08	0	2.72727E-08	0.00000015	9.48182E-08	7.07273E-08	2.14E-08	6.65333E-08	5.94E-08	1.244E-07
Swingsia_sp._F3b2	2.70909E-08	5.41818E-08	0	7.59727E-08	0.000000124	7.21538E-08	0	6.03846E-08	1.53273E-07	3.89091E-08	2.94182E-07	3.09091E-07	4.33333E-08	1.33933E-07	8.86667E-09	5.70667E-08
Swingsia_samuiensis	0	0	7.29455E-08	6.12727E-08	0	0	3.11538E-08	3.81538E-08	0	0	0	7.55455E-08	4.81333E-08	3.50667E-08	0	6.33333E-08
Neoasaia_chiangmaiensis	2.58818E-07	3.55909E-07	5.75818E-08	0.000000078	2.88462E-08	1.91769E-07	0	1.57615E-07	5.79091E-08	6.37273E-08	5.52727E-08	3.17091E-07	2.24E-08	8.95333E-08	9.35733E-08	2.10067E-07
Granulibacter_bethesdensis	7.81455E-07	2.00545E-06	2.21864E-06	1.49227E-06	2.57615E-07	4.42692E-07	7.08769E-07	1.07985E-06	1.85482E-06	0.00000097	1.05818E-06	6.57364E-07	6.232E-07	8.37267E-07	6.35133E-07	0.000000555
Kozakia_baliensis	0.00000041	1.96227E-07	5.70727E-07	0.000000283	9.46154E-08	5.29231E-08	1.67769E-07	1.48385E-07	1.26455E-07	1.78727E-07	1.88455E-07	3.87273E-07	1.64933E-07	2.882E-07	2.06707E-07	3.364E-07
Gluconacetobacter_diazotrophicus	2.19545E-07	0	3.70909E-08	0.000000231	3.76923E-07	0	3.21154E-07	5.51769E-07	4.96091E-07	1.43545E-07	3.23182E-07	5.18182E-07	1.41333E-07	3.52933E-07	1.48707E-07	2.794E-07
Parasaccharibacter_apium	1.05455E-07	3.12727E-08	7.81818E-08	0.00000021	2.26538E-07	0	0.000000192	3.84769E-07	2.41636E-07	3.68182E-07	3.10091E-07	4.49727E-07	1.25133E-07	2.936E-07	3.16267E-07	1.21533E-07
Roseomonas_gilardii	1.36091E-07	0	5.77273E-08	4.15636E-07	3.15385E-08	9.73846E-08	3.32769E-07	5.05385E-07	3.05727E-07	2.27818E-07	3.44364E-07	3.31273E-07	3.40333E-07	1.976E-07	1.5524E-07	2.01267E-07
Roseomonas_mucosa	7.81455E-07	0	0	0	3.32231E-07	3.15385E-08	0	7.72308E-08	3.14273E-07	1.16727E-06	6.88182E-08	5.61636E-07	9.04133E-07	7.74533E-07	9.39067E-07	4.67533E-07
Komagataeibacter_xylinus	8.98182E-08	0	0.000000047	4.79091E-09	6.78462E-08	2.52308E-08	2.10385E-07	1.68538E-07	0	2.43182E-07	1.56182E-07	1.53545E-07	0.000000118	6.31333E-08	5.652E-07	4.84867E-07
Komagataeibacter_saccharivorans	2.96364E-08	0	3.70909E-08	5.26636E-07	2.44231E-07	0	0.000000096	1.07538E-07	3.96455E-07	4.29364E-07	4.32727E-07	2.13545E-07	1.03733E-07	2.62867E-07	9.21067E-08	2.69067E-07
Komagataeibacter_medellinensis	1.59655E-06	0.000000065	5.32818E-07	0.000000494	0.000000191	3.21154E-07	3.63923E-07	3.07385E-07	7.50636E-07	4.21273E-07	0.000001423	2.52182E-07	5.38933E-07	5.04467E-07	3.36133E-07	3.28733E-07
Komagataeibacter_nataicola	1.70909E-07	0	3.70909E-08	3.72455E-08	1.07615E-07	2.52308E-08	0	1.16154E-07	8.23636E-08	3.03636E-08	2.11727E-07	9.25455E-08	4.78E-08	0.000000033	0	1.794E-07
Komagataeibacter_oboediens	3.48909E-07	2.63818E-07	5.01273E-08	1.10636E-07	0.000000039	1.43831E-06	1.51385E-07	1.00846E-07	2.41636E-07	2.28909E-07	2.31818E-07	2.54727E-07	1.81333E-07	1.57467E-07	2.33133E-07	1.358E-07
Komagataeibacter_europaeus	1.89818E-07	4.07909E-07	4.26636E-07	0.000000137	3.34615E-08	1.79231E-08	1.00077E-07	5.04615E-08	3.53727E-07	0.000000116	2.09455E-07	8.30273E-07	6.66667E-08	1.58E-08	0	0
Komagataeibacter_hansenii	6.88818E-07	1.12364E-07	0.000000469	1.04536E-06	6.63615E-07	1.51538E-07	1.01146E-06	9.29538E-07	8.50182E-07	7.60091E-07	1.21518E-06	1.04418E-06	7.55467E-07	7.978E-07	0.000000486	9.23267E-07
Acidiphilium_multivorum	2.56364E-08	0	2.89273E-07	8.08182E-08	7.03846E-08	5.91538E-08	0.000000168	3.08846E-07	2.45636E-07	5.70909E-08	6.97273E-08	1.62909E-07	1.32133E-07	0	1.34867E-07	1.062E-07

Acidiphilium_cryptum	5.57273E-08	0	3.70909E-08	1.63273E-07	8.85385E-08	3.83077E-08	2.63077E-08	1.09462E-07	1.53545E-07	7.51818E-08	2.87636E-07	3.77727E-07	2.24E-08	1.39333E-07	1.766E-07	0.000000074
Stella_humosa	2.28364E-07	5.48182E-07	9.04545E-08	2.46545E-07	3.15385E-08	3.76615E-07	2.67846E-07	1.55154E-07	9.72727E-08	9.92727E-08	2.11182E-07	2.16636E-07	5.86E-08	4.05667E-07	2.50533E-07	5.10667E-08
Acetobacter_ghanensis	0	0	0	3.07273E-08	0	0	0	5.22308E-08	0	0	0	0	0	0	0	0
Acetobacter_tropicalis	2.54545E-07	2.68864E-07	0.000000046	2.29818E-07	0	0	3.11538E-08	4.93846E-08	0	0	0	0	0	0	0	0
Acetobacter_ascendens	0	0	8.21818E-08	6.44545E-08	6.93077E-08	0	0	0	8.87273E-08	1.41091E-07	5.52545E-07	1.16364E-07	4.30667E-08	1.58E-08	5.10667E-08	3.40667E-08
Acetobacter_orientalis	5.42727E-08	7.54545E-08	0	3.85273E-07	6.17692E-08	0	2.63077E-08	7.42308E-08	5.97273E-08	7.02727E-08	6.66364E-08	1.17909E-07	4.17333E-08	9.03333E-08	0	3.67333E-08
Acetobacter_senegalensis	1.43273E-07	0	0	5.66091E-08	5.74615E-08	1.79231E-08	2.48615E-07	2.67385E-07	2.97727E-07	2.87818E-07	5.13455E-07	3.22091E-07	7.72667E-08	4.88E-08	1.81933E-07	5.66E-08
Acetobacter_sp._KACC_21233	3.23364E-07	1.59455E-07	0.000000135	8.73636E-08	3.58462E-08	1.24954E-06	1.64231E-07	2.44615E-07	1.48364E-07	6.37273E-08	2.17273E-07	1.88818E-07	7.90667E-08	7.42667E-08	9.92E-08	2.906E-07
Acetobacter_aceti	4.01182E-07	1.37545E-07	3.03573E-07	4.30182E-07	2.24769E-07	2.20231E-07	1.12923E-07	6.93846E-07	3.24182E-07	5.32455E-07	5.32909E-07	4.09182E-07	0.000000457	0.000000298	7.65667E-07	6.62333E-07
Acetobacter_persici	4.05273E-07	1.94545E-07	3.27273E-08	0.000000327	4.84923E-07	4.33846E-08	6.96923E-08	2.46923E-08	1.26727E-07	0.000000034	0	1.88545E-07	1.83133E-07	9.87333E-08	1.28267E-07	3.72E-08
Acetobacter_pasteurianus	7.00909E-07	3.35455E-08	6.98182E-08	9.06364E-08	1.35531E-06	0	1.16769E-07	6.69923E-07	0.000001182	4.83455E-07	6.21364E-07	2.92182E-07	9.926E-07	1.22633E-06	9.51333E-07	9.666E-07
Gluconobacter_thailandicus	8.69091E-08	0	0	0	0	0	3.33077E-08	2.47692E-08	0	3.62727E-08	0	0	0	0	0	0
Gluconobacter_albidus	1.69182E-07	0	0	6.74545E-08	1.78308E-07	2.36923E-07	1.22231E-07	1.70615E-07	7.02727E-08	5.72727E-08	2.53727E-07	2.02409E-07	9.02E-08	1.142E-07	1.65067E-07	1.23333E-07
Gluconobacter_oxydans	3.51091E-06	1.98273E-07	5.14636E-07	1.03027E-06	2.52746E-06	2.25692E-07	2.32877E-06	3.10762E-06	3.98882E-06	4.45545E-06	4.90482E-06	2.85173E-06	2.81547E-06	2.93973E-06	2.29733E-06	0.000003354
Qipengyuania_sediminis	3.69091E-08	0	1.39091E-07	2.54545E-07	4.75385E-08	2.52308E-08	0	0	6.68182E-08	0.000000028	7.69091E-08	3.87727E-08	4.99333E-08	7.63333E-08	2.54E-08	5.07333E-08
Croceicoccus_naphthovorans	0.000000064	9.54545E-09	0	1.49455E-07	1.06385E-07	0	0	7.15385E-08	2.36545E-07	3.62727E-08	2.73182E-07	2.14545E-08	2.64667E-08	1.78467E-07	1.65333E-08	1.87067E-07
Croceicoccus_marinus	2.29364E-07	1.68182E-08	0	1.83636E-07	0.000000273	0.000000459	6.81692E-07	9.41538E-08	1.97636E-07	2.72364E-07	0.000000267	2.28091E-07	4.234E-07	1.19533E-07	2.68667E-07	3.70533E-07
Erythrobacter_gangjinensis	0	0	0	0	0	0	0	0.000000042	0	0	0	0	0	1.01133E-07	0	9.33333E-09
Erythrobacter_atlanticus	0	0	0	5.16364E-08	0	0	0	1.90769E-08	0	0	0	7.55455E-08	0	0	0	0
Erythrobacter_sp._THAF29	0	0	0	4.79091E-09	0	0	3.33077E-08	0.000000042	0	0	0	0	0	0	0	0
Erythrobacter_sp._HKB08	1.34091E-07	0	2.96364E-08	4.79091E-09	0	0.000000648	3.33077E-08	0	0	0	0	1.42727E-08	2.24E-08	4.64667E-08	5.94E-08	1.86667E-08
Erythrobacter_sp._KY5	1.11818E-07	0.000000065	0	4.32455E-08	0	0	0	1.01615E-07	1.12727E-07	2.56182E-07	7.74545E-08	5.70909E-08	0	0	0	0
Erythrobacter_mangrovi	2.11545E-07	3.68273E-07	0.000000047	5.26364E-07	1.14923E-07	0	1.09923E-07	1.43308E-07	8.87273E-08	1.78909E-07	1.46818E-07	1.50636E-07	1.05933E-07	2.05333E-07	1.61333E-07	1.58667E-07
Erythrobacter_sp._HL-111	3.15455E-08	0	4.94545E-08	2.00818E-07	2.59231E-08	0	0	7.28462E-08	0.000000054	9.33636E-08	1.46818E-07	7.13636E-09	4.51333E-08	3.09333E-08	1.388E-07	9.11333E-08
Erythrobacter_aureus	1.04364E-06	0.00000013	2.94764E-07	7.29818E-07	0.000000151	0.000000913	1.93615E-07	3.35692E-07	0.000000491	3.68364E-07	4.87182E-07	4.28909E-07	2.362E-07	0.000000384	3.45467E-07	1.86133E-07
Erythrobacter_flavus	1.40818E-07	3.10909E-08	2.04136E-07	7.27273E-08	0	0	0	0	1.46091E-07	2.40909E-07	0	9.75E-08	0	3.50667E-08	0	0
Erythrobacter_seohaensis	0	0	0	0	9.64615E-08	4.31538E-08	1.77231E-06	1.61331E-06	2.75455E-08	0	2.76364E-08	6.73636E-08	2.156E-07	3.978E-07	3.422E-07	2.884E-07
Erythrobacter_litoralis	7.57273E-08	8.10636E-07	1.27309E-07	8.58182E-08	2.34923E-07	0	5.33923E-07	4.99923E-07	2.06818E-07	2.94364E-07	2.84364E-07	7.97818E-07	1.094E-07	2.86067E-07	0.000000373	2.33067E-07
Altererythrobacter_dongtanensis	0	0	0	0	0	0	0	0.000000011	2.75455E-08	0	0	0	2.24E-08	4.76667E-08	0	0
Altererythrobacter_namhicola	0	0	0	0	0	0	0	0	0	3.62727E-08	6.88182E-08	0	0	0	0	0
Altererythrobacter_epoxidivorans	1.22455E-07	0	0	0	9.92308E-08	0	0	0	6.45455E-08	1.34273E-07	0.000000048	8.21364E-08	0	0	3.41733E-08	6.62667E-08
Altererythrobacter_mangrovi	0	0	0	4.79091E-09	0	6.30769E-08	1.41385E-07	0	3.75455E-08	1.10636E-07	8.02727E-08	7.44545E-08	0	4.64667E-08	0	0
Altererythrobacter_atlanticus	5.01636E-07	3.88364E-07	7.57273E-08	1.37909E-07	4.75385E-08	1.79231E-08	0.000000087	6.95462E-07	0.000000134	6.25455E-08	6.97273E-08	0.000000267	9.83333E-08	1.71333E-08	4.344E-08	8.67333E-08
Altererythrobacter_ishigakiensis	3.11818E-08	0	2.18545E-08	1.72455E-07	1.00538E-07	2.95385E-08	0.000000086	3.37692E-08	8.54545E-08	9.92727E-08	1.86818E-07	5.30909E-08	6.92667E-08	5.89333E-08	3.37333E-08	2.128E-07
Altererythrobacter_sp._B11	5.86364E-08	0	4.53455E-08	0	0	0	0	0	0	0	1.06364E-07	6.35455E-08	0	3.09333E-08	0	1.86E-08
Altererythrobacter_sp._BO-6	2.29545E-07	6.92727E-08	4.66364E-08	0.000000444	1.03846E-07	1.39846E-07	1.98462E-07	0.000000171	4.11727E-07	2.69455E-07	4.46727E-07	5.04273E-07	2.28933E-07	7.29333E-08	2.268E-07	1.12867E-07
Altererythrobacter_sp._TH136	4.89818E-07	0	1.60727E-06	1.59164E-06	3.94154E-07	0	3.93769E-06	0.000005337	9.12545E-07	6.31364E-07	7.11182E-07	4.32182E-07	4.55333E-07	4.18333E-07	7.72333E-07	4.708E-07
Altererythrobacter_sp._ZODW24	5.52727E-08	1.16364E-08	0	2.39091E-08	6.69231E-08	5.19231E-08	0	1.35154E-07	4.45455E-08	1.32182E-07	2.35636E-07	8.36364E-08	0	3.48E-08	0	0
Altererythrobacter_amylolyticus	8.82091E-07	0	0	8.77091E-07	8.91538E-08	0	0	7.13077E-08	2.94909E-07	1.79455E-07	1.45364E-07	4.52409E-07	1.974E-07	3.07267E-07	1.79733E-07	8.61733E-07
Altererythrobacter_marensis	1.15355E-06	0	4.80273E-07	5.71364E-07	2.91923E-07	5.08292E-06	2.08146E-06	2.68485E-06	7.69273E-07	5.08364E-07	7.82273E-07	8.78636E-07	5.68733E-07	3.302E-07	7.33467E-07	8.86133E-07
Porphyrobacter_neustonensis	1.58182E-08	0	0	4.47E-08	6.52923E-07	0	0	0	0	0.000000047	3.79091E-08	9.39091E-08	6.83667E-07	0.000000844	5.28267E-07	3.32733E-07
Porphyrobacter_sp._LM_6	7.57273E-08	0	0	0	0	0	1.10769E-07	5.98462E-08	1.13545E-07	7.55455E-08	2.50909E-07	9.25E-08	4.68E-08	9.14667E-08	8.86667E-09	2.328E-07
Porphyrobacter_sp._YT40	3.05273E-07	0	1.37582E-07	1.36455E-07	2.05385E-08	2.66154E-07	0.000000221	1.30769E-07	8.13636E-08	3.12727E-07	1.02182E-07	5.56273E-07	2.71467E-07	3.534E-07	0.000000231	3.84067E-07
Porphyrobacter_sp._CACIAM_03H1	3.56273E-07	7.77273E-08	0	9.54545E-08	4.13846E-08	7.36154E-08	2.63077E-08	5.22308E-08	0.000000176	8.32727E-08	4.59545E-07	2.31545E-07	1.43533E-07	1.02867E-07	3.31467E-07	4.27133E-07
Parasphingopyxis_sp._CP4	5.57273E-08	0	0	0	0	0	0	0	0	0	0	0	0	0	0	0
Parasphingopyxis_algicola	0	3.87273E-08	1.63636E-08	4.02727E-08	0	0	0	0	5.37273E-08	0	6.88182E-08	0	0	0.000000033	0	0
Tardibacter_chloracetimidivorans	3.40818E-07	0.00000013	1.89273E-07	3.21636E-07	0	5.86846E-07	1.63154E-07	2.17462E-07	3.23182E-07	0.00000038	2.04091E-07	6.55909E-07	2.24E-08	4.49333E-08	7.44E-08	9.33333E-09
Rhizorhabdus_dicambivorans	1.14455E-07	0	0	0	6.03846E-08	1.29215E-06	9.56154E-08	1.18231E-07	3.75455E-08	1.44727E-07	0	3.73727E-07	0	6.60667E-08	2.39333E-08	2.92E-08
Sandaracinobacter_sp._M6	9.36364E-08	3.05727E-07	1.88545E-07	9.77909E-08	1.10538E-07	2.13846E-08	2.00692E-07	1.46923E-08	0	0	2.76364E-08	4.28182E-08	1.46733E-07	7.58E-08	9.38E-08	8.78E-08
Novosphingopyxis_sp._pb63	1.25355E-06	0.000000508	4.32482E-07	9.61636E-07	0.000000798	0	1.28077E-07	6.44923E-07	9.83727E-07	4.90545E-07	2.27655E-06	7.34636E-07	0.000000117	3.714E-07	3.56333E-07	7.74533E-07
Sphingorhabdus_sp._M41	0	0	0	3.59091E-08	3.15385E-08	0	0	0	0	0	0	4.79091E-08	0	0.000000014	4.44E-09	0
Sphingorhabdus_lacus	2.70909E-08	1.65273E-07	1.43636E-07	2.74636E-07	2.05385E-08	0	0	3.75231E-07	8.54545E-08	3.89091E-08	0	3.87727E-08	0	0	0	7.90667E-08
Sphingorhabdus_sp._SMR4y	0	8.86364E-09	0	0	0	6.53077E-08	3.11538E-08	5.81538E-08	0	0	6.68182E-08	7.13636E-09	0	1.58E-08	5.04667E-08	0
Sphingorhabdus_sp._JK6	1.11364E-07	0	0	1.20727E-07	0	0	0	0	8.54545E-08	0	1.32727E-07	7.13636E-09	0	6.87333E-08	1.136E-07	0.000000138
Sphingorhabdus_sp._YGSMI21	2.79455E-07	0	3.70909E-08	8.01818E-08	2.59231E-08	0	0	3.81538E-08	0.000000124	0	2.45091E-07	2.215E-07	4.24E-08	2.62E-08	5.85333E-08	1.86E-08
Blastomonas_fulva	1.89455E-07	0	0	1.09173E-06	1.74923E-07	1.88062E-06	2.84692E-06	1.78238E-06	0.000000436	3.39818E-07	8.28182E-07	3.41545E-07	1.76733E-07	0.000000257	5.30533E-07	1.506E-07
Blastomonas_sp._RAC04	1.00936E-06	2.12318E-07	8.09091E-07	1.52255E-06	4.70692E-07	1.20423E-06	5.03615E-07	1.30962E-06	9.40545E-07	5.45455E-07	1.17018E-06	1.10373E-06	2.96467E-07	5.76667E-07	6.82067E-07	6.838E-07
Citromicrobium_sp._JL477	5.82727E-08	0	0	5.32727E-08	0.000000115	1.57385E-07	0	1.16154E-07	2.40182E-07	2.76818E-07	0.000000388	0.000000138	5.15333E-08	2.05733E-07	3.11373E-07	2.14933E-07
Novosphingobium_sp._PP1Y	0	0	0	0	5.45385E-08	0	0	0	1.15455E-07	0.000000028	0	8.98182E-08	0	0	0	0
Novosphingobium_sp._THN1	0	0	0	0.000000095	1.04538E-07	0	1.05385E-07	2.64231E-07	0.00000014	1.09545E-07	1.97727E-07	1.62909E-07	2.37333E-08	9.93333E-08	0.000000138	6.02667E-08
Novosphingobium_sp._ABRDHK2	9.26364E-08	0	0	6.66364E-08	0	4.35231E-07	2.63077E-08	0	5.79091E-08	0	1.35364E-07	1.08773E-07	0	3.09333E-08	2.24667E-08	0
Novosphingobium_ginsenosidimutans	5.57273E-08	0	0	4.76091E-08	0.000000151	0.000000026	6.41538E-08	9.03846E-08	8.20909E-08	2.56364E-07	7.56364E-08	9.75E-08	0	3.78E-08	4.44E-09	1.66667E-08
Novosphingobium_sp._P6W	0	1.86091E-07	1.80727E-07	2.97518E-07	4.11538E-08	7.49615E-07	3.11538E-08	0	8.61818E-08	3.62727E-08	0	1.73182E-07	0	0	4.82E-08	1.22E-08
Novosphingobium_resinovorum	3.89091E-08	9.85455E-08	0	2.47273E-08	3.15385E-08	9.00769E-08	3.11538E-08	0.000000077	0	0.000000028	2.05455E-08	7.64545E-08	2.24E-08	6.60667E-08	1.10133E-07	0.000000028
Novosphingobium_sp._Gsoil_351	6.90182E-07	0.000000065	0.000000397	9.54545E-09	0	0	5.96154E-08	1.87769E-07	6.62727E-08	2.85455E-08	0	3.17727E-07	6.70667E-08	1.71333E-08	1.24333E-07	0
Novosphingobium_pentaromativorans	0	0	1.63636E-08	1.43636E-08	2.88462E-08	0	0	5.69231E-08	0	9.40909E-08	0.000000039	7.13636E-09	0	6.04667E-08	1.23133E-07	3.40667E-08
Novosphingobium_sp._ES2-1	3.69091E-08	0	0	5.94545E-08	1.86923E-08	0	6.41538E-08	0.000000053	2.16364E-08	2.85455E-08	7.67273E-08	0.000000075	0	5.22E-08	0	6.77333E-08
Novosphingobium_tardaugens	2.18364E-07	1.64545E-07	8.25455E-09	0.000000076	0.000000141	8.16154E-08	3.11538E-08	9.42308E-08	3.63364E-07	2.43273E-07	0.000000227	4.74182E-07	7.94667E-08	1.89333E-07	1.30573E-07	1.57467E-07
Novosphingobium_aromaticivorans	0.00000011	0.000000125	8.25455E-09	3.40727E-07	6.69231E-08	0	2.63077E-08	4.93846E-08	2.33364E-07	0	1.97455E-07	1.89545E-07	0	1.14267E-07	5.04667E-08	8.92667E-08
Zymomonas_mobilis	6.42545E-07	2.90091E-07	2.43364E-07	6.03909E-07	4.10615E-07	6.74923E-07	4.52308E-07	0.000000258	5.32091E-07	4.18455E-07	4.57364E-07	8.41818E-07	4.78867E-07	4.98667E-07	7.76067E-07	2.91133E-07
Sphingosinicella_microcystinivorans	3.82945E-06	0.000000935	3.17664E-06	2.99918E-06	3.43462E-07	1.74846E-07	3.00462E-06	3.65054E-06	1.25336E-06	1.75655E-06	2.11464E-06	1.80836E-06	2.32467E-07	6.97067E-07	4.15133E-07	4.80333E-07
Sphingosinicella_sp._BN140058	6.76364E-07	0	3.70909E-08	8.77364E-07	1.62692E-07	0	0	7.72308E-08	0.000000384	3.00909E-07	4.87636E-07	0.000000374	3.50333E-07	0.00000042	3.91933E-07	8.192E-07
Sphingosinithalassobacter_sp._zrk23	8.37909E-07	0	4.11455E-07	0.000000686	3.36154E-07	0	8.52077E-07	1.38623E-06	3.40364E-07	1.95455E-07	4.42091E-07	4.92682E-07	0.000000503	3.014E-07	2.738E-07	8.71333E-07
Sphingosinithalassobacter_sp._CS137	2.59545E-06	3.24545E-07	5.92636E-07	2.12618E-06	0.000000566	2.95385E-08	8.78077E-07	1.09592E-06	2.24718E-06	1.20164E-06	1.88182E-06	0.000001363	1.04373E-06	9.84733E-07	5.32133E-07	1.38833E-06
Sphingopyxis_sp._MG	0	0	0	0	0	0	0	0	2.75455E-08	0	0	0	0	0	0	0
Sphingopyxis_sp._FD7	0	5.81818E-08	1.14545E-07	2.43636E-07	0	0	0	0	2.16364E-08	0	0	1.22182E-07	0	0	0	0.000000028
Sphingopyxis_lindanitolerans	0	0	0	3.07273E-08	0	0	1.15538E-07	7.13077E-08	1.73636E-07	0	2.76364E-08	3.87727E-08	0	0	1.49333E-08	0
Sphingopyxis_fribergensis	7.31818E-08	1.94545E-07	0	3.02727E-08	0.000000065	0	0	0	2.25455E-07	2.85455E-08	9.48182E-08	8.56364E-08	2.44E-08	0	0	5.07333E-08
Sphingopyxis_sp._EG6	0	8.12727E-08	9.04545E-08	7.45455E-08	0	0	2.63077E-08	4.64615E-08	0	0.000000034	1.32182E-07	9.03636E-08	4.24E-08	1.61333E-08	6.34667E-08	4.94E-08
Sphingopyxis_sp._PAMC25046	5.19727E-07	0	1.01955E-06	2.64818E-07	0.000000164	7.36154E-08	1.27877E-06	1.68162E-06	3.60273E-07	3.32455E-07	0.000000234	1.75455E-07	1.59933E-07	0.000000162	0.000000115	6.37133E-07
Sphingopyxis_sp._113P3	7.62455E-07	5.45455E-08	7.33182E-07	1.29518E-06	6.25923E-07	7.57692E-08	6.00385E-07	9.77769E-07	1.11227E-06	1.18336E-06	6.42364E-07	3.54182E-07	7.922E-07	3.838E-07	5.58133E-07	9.17533E-07
Sphingopyxis_macrogoltabida	3.11818E-08	3.15864E-07	2.04909E-08	3.63091E-07	7.05385E-08	1.89154E-07	1.20154E-07	1.23923E-07	3.75455E-08	0.000000028	2.76364E-08	9.59091E-08	5.28667E-08	0	0	0
Sphingopyxis_alaskensis	0	0	8.75455E-09	7.40636E-08	2.05385E-08	0	0	0	0	0.000000028	2.06636E-07	4.21364E-08	2.14667E-08	1.10867E-07	8.15333E-08	9.33333E-09
Sphingopyxis_sp._OPL5	2.38545E-07	0	4.53455E-08	5.12727E-07	1.54538E-07	2.23923E-07	4.77692E-07	3.33923E-07	7.18182E-08	2.57455E-07	1.02727E-08	4.55091E-07	6.38667E-08	1.69533E-07	3.882E-07	2.954E-07
Sphingopyxis_sp._QXT-31	1.24155E-06	1.41227E-07	1.76909E-07	7.52545E-07	6.73846E-08	0	0	1.74615E-07	2.97364E-07	1.34727E-07	3.85545E-07	0.000000455	2.22933E-07	1.014E-07	2.42667E-07	5.68533E-07
Sphingopyxis_sp._LPB0140	2.42727E-07	0	8.25455E-09	5.18182E-09	1.30154E-07	0	2.63077E-08	0	2.26273E-07	2.15273E-07	6.71818E-08	1.12182E-07	8.96667E-08	1.16667E-07	1.23373E-07	1.58467E-07
Sphingobium_sp._KCTC_72723	3.11818E-08	0	0	4.79091E-09	0	2.52308E-08	0	0	3.98182E-08	2.85455E-08	0	7.31818E-08	0	1.58E-08	0	1.98667E-08
Sphingobium_sp._RSMS	0	0	0	3.72455E-08	0	0	0	0	0	0	0	0	0	0	0	5.10667E-08
Sphingobium_sp._CAP-1	7.83636E-08	1.04545E-08	0	8.47909E-08	8.36154E-08	4.27692E-08	4.33846E-08	6.45385E-08	1.03455E-07	2.03818E-07	0	6.93636E-08	8.32E-08	0	0	2.92E-08
Sphingobium_fuliginis	0	0	0	0	0	0	0	1.46923E-08	0	0	0	0	2.14E-08	0	1.65333E-08	0
Sphingobium_sp._TKS	1.67273E-07	0	0	4.68818E-08	0	0	5.26154E-08	0.000000086	0.000000177	2.85455E-08	6.88182E-08	3.16364E-08	2.64667E-08	3.50667E-08	0	0
Sphingobium_cloacae	0	0	0	0	3.15385E-08	0	0	0	0.000000027	3.89091E-08	2.76364E-08	3.02545E-07	0	0	4.44E-09	0
Sphingobium_baderi	0	0	1.63636E-08	6.00636E-08	0	0.000000508	6.89231E-08	7.88462E-08	9.95455E-08	0.000000028	7.90909E-08	1.42727E-08	0	0	2.39333E-08	0
Sphingobium_indicum	0	0	1.63636E-08	4.72727E-08	0	0	0	9.36154E-08	4.80909E-08	1.06455E-07	0	4.79091E-08	0	0	1.12667E-08	0
Sphingobium_chlorophenolicum	3.11818E-08	0	1.63636E-08	1.17455E-07	0	0	0	0	0	0	0	1.42727E-08	0	0	0	0
Sphingobium_sp._SCG-1	0	0	5.34545E-08	0	1.26154E-07	7.09231E-08	0	9.38462E-08	0	0	0	1.88045E-07	2.24E-08	0	0	0
Sphingobium_sp._PAMC28499	3.46545E-07	0	0	3.85455E-08	6.03846E-08	0	0	0.000000042	1.12182E-07	6.37273E-08	1.70818E-07	1.57909E-07	0	3.50667E-08	1.13133E-07	0.000000105
Sphingobium_sp._MI1205	1.11818E-07	0	0	0	2.75385E-08	1.26538E-07	3.11538E-08	1.90769E-08	1.21909E-07	2.85455E-08	1.12455E-07	1.82364E-07	6.08E-08	0.000000014	2.78E-08	4.97333E-08
Sphingobium_amiense	1.12064E-06	0	8.40909E-08	9.97091E-07	2.75385E-08	0	0	2.24308E-07	2.18909E-07	2.00091E-07	2.61273E-07	0.0000047	2.00333E-07	6.34667E-08	1.04133E-07	3.03467E-07
Sphingobium_sp._LF-16	0	0	0	1.43636E-08	1.86923E-08	0	0	0	8.51818E-08	2.87273E-08	1.27636E-07	3.94545E-08	0	2.62E-08	2.55333E-08	3.40667E-08
Sphingobium_sp._RAC03	8.99273E-07	0	1.63636E-08	5.69364E-07	4.65692E-07	5.75231E-07	5.74615E-08	0.000000261	1.63909E-07	4.82455E-07	4.19909E-07	3.69455E-07	2.68333E-07	4.604E-07	2.346E-07	2.15333E-08
Sphingobium_sp._SYK-6	0.000000283	0.000000281	7.40909E-08	1.50727E-07	2.34385E-07	2.83846E-08	2.63077E-08	1.88769E-07	2.09818E-07	3.62273E-07	3.81273E-07	1.70364E-07	1.568E-07	1.76467E-07	1.87467E-07	8.51333E-08
Sphingobium_sp._Cam5-1	6.80909E-08	0	1.77273E-08	9.57273E-08	4.13846E-08	0	0	0	0	0	0	1.42727E-08	0	0	5.94E-08	0
Sphingobium_japonicum	2.75909E-07	0	8.31818E-09	8.97273E-08	0	2.52308E-08	8.92308E-08	1.78308E-07	2.64545E-08	0	1.70364E-07	1.895E-07	1.094E-07	0.000000097	2.35933E-07	1.04067E-07
Sphingobium_hydrophobicum	0	0	9.37091E-08	3.88727E-07	0	0	0	2.19077E-07	2.16364E-08	0	0	9.00455E-08	0	3.09333E-08	4.48E-08	1.98667E-08
Sphingobium_sp._YG1	4.73545E-07	0.000000065	1.48182E-07	1.02818E-06	2.62154E-07	7.66154E-08	1.06615E-07	7.40769E-08	9.62545E-07	8.62273E-07	1.06664E-06	1.39082E-06	0.000000241	3.896E-07	4.18933E-07	4.456E-07
Sphingobium_sp._EP60837	3.69091E-08	0	0	0	0	1.79231E-08	1.05692E-07	2.47692E-08	0	0	0.0000001	6.41364E-08	0	5.86E-08	3.37333E-08	1.762E-07
Sphingobium_herbicidovorans	1.09273E-07	0	2.88545E-08	1.43636E-08	0	0	3.33077E-08	2.47692E-08	0	0	0	3.74545E-08	4.27333E-08	1.282E-07	0	1.22E-08
Sphingobium_yanoikuyae	1.16745E-06	0	3.80036E-07	1.14664E-06	8.36923E-08	3.31769E-07	1.96846E-07	3.67077E-07	1.17427E-06	5.47909E-07	1.22755E-06	6.56091E-07	6.40067E-07	3.344E-07	7.718E-07	3.878E-07
Sphingomonas_lutea	0	0	0	2.15818E-07	0	0	0	2.46923E-08	0	0	0	4.79091E-08	0	0	0	1.22E-08
Sphingomonas_sp._FARSPH	0	0	0	0	2.53692E-07	0	0	3.22308E-08	0	3.89091E-08	0	1.27909E-07	0	1.11267E-07	8.74667E-08	3.086E-07
Sphingomonas_melonis	1.11818E-07	0	0	0	1.16077E-07	2.21231E-07	0	0.000000011	0	5.72727E-08	0	7.89091E-08	7.17333E-08	6.07333E-08	0.000000063	1.51333E-07
Sphingomonas_sediminicola	2.96364E-08	0	0	4.40909E-08	0	1.52462E-07	1.33077E-07	8.58462E-08	0	1.24727E-07	0	3.94545E-08	0	0	9.65067E-08	5.10667E-08
Sphingomonas_hengshuiensis	3.11818E-08	0	0	5.48818E-08	0	0	2.63077E-08	0	1.15455E-07	0	0	0	0	0	1.49333E-08	1.98667E-08
Sphingomonas_sp._MM-1	5.57273E-08	0	0	0	0	0	0	0	0	0	6.68182E-08	0	0	0	0	0
Sphingomonas_sp._AE3	9.26364E-08	0	0	2.79545E-07	1.44692E-07	0	0	5.22308E-08	5.97273E-08	2.21727E-07	1.45909E-07	3.53818E-07	9.52667E-08	1.42133E-07	5.69333E-08	0.000000026
Sphingomonas_sp._NIC1	2.03091E-07	8.84545E-08	0	5.16364E-08	0	0	0	0	2.75455E-08	0	2.76364E-08	3.57273E-08	7.18667E-08	3.42E-08	0	1.98667E-08
Sphingomonas_sp._YZ-8	0	0.00000013	0	4.79091E-09	0	5.91538E-08	0	0	1.55273E-07	0.000000081	4.44182E-07	1.46727E-07	0	0	4.44E-09	0
Sphingomonas_sp._C33	5.27273E-08	1.37273E-08	3.70909E-08	9.44545E-08	0	4.33077E-07	1.20385E-07	9.11538E-08	0	5.22727E-08	0.000000136	8.42727E-08	1.684E-07	9.08E-08	1.012E-07	2.15333E-08
Sphingomonas_sp._Cra20	0	0	1.63636E-08	3.89091E-08	0	0	0	0	1.64273E-07	0.000000028	2.76364E-08	7.12727E-08	0	0	0	0
Sphingomonas_wittichii	3.62727E-08	0	2.96364E-08	3.99091E-08	0	0	0	0	0	0	0	0	4.51333E-08	1.58E-08	0	0
Sphingomonas_lacunae	3.11818E-08	0	2.96364E-08	1.91818E-08	0	0	0	4.41538E-08	5.79091E-08	0	0	7.13636E-09	0	0	1.28667E-07	0
Sphingomonas_sp._HMP9	7.83636E-08	1.16364E-08	2.96364E-08	1.55455E-08	3.76538E-07	5.58462E-07	5.26154E-08	0.000000086	1.38636E-07	0.000000128	0.000000369	1.65909E-07	0.000000141	1.27733E-07	4.82E-08	1.202E-07
Sphingomonas_rhizophila	1.58182E-08	3.87273E-08	7.40909E-08	3.55182E-08	0	6.79769E-07	3.33077E-08	1.10154E-07	1.12727E-07	0	2.76364E-08	7.13636E-09	7.46667E-08	1.61333E-08	0.000000064	2.92E-08
Sphingomonas_sp._MK52	1.76909E-07	0.000000065	3.70909E-08	1.55455E-08	0	0	0	0	0	6.08182E-08	6.88182E-08	4.92727E-08	0	0	0	1.86E-08
Sphingomonas_taxi	2.56364E-08	0	0	3.99091E-08	2.59231E-08	4.13846E-08	0	0	0	0	3.33636E-08	7.13636E-09	0.000000027	0	0	0
Sphingomonas_sp._C8-2	2.30909E-08	0	5.34545E-08	9.54545E-09	5.76154E-08	1.62615E-07	0	1.78077E-07	6.61818E-08	3.89091E-08	7.29091E-08	1.87409E-07	2.37333E-08	0	2.46E-08	1.02267E-07
Sphingomonas_sp._HMP6	3.69091E-08	0	0	2.39727E-08	0	0	0	0	0	1.22364E-07	7.23636E-08	3.74545E-08	4.33333E-08	0	2.16467E-07	1.66667E-08
Sphingomonas_sp._KC8	0	1.16364E-08	1.77273E-08	4.79091E-09	8.63077E-08	1.79231E-08	5.51769E-07	4.03846E-08	4.45455E-08	0.000000115	2.34273E-07	1.48545E-07	6.08E-08	1.71333E-08	0	3.97333E-08
Sphingomonas_sp._HKS19	0	4.23136E-07	1.77273E-08	0	0	4.27692E-08	0	0.000000042	0	0	0	7.55455E-08	4.18667E-08	0	1.12667E-08	1.22E-08
Sphingomonas_daechungensis	0	0	4.12727E-09	4.79091E-09	0	0	0	0	0	0	0	0	0	0	0	0
Sphingomonas_paucimobilis	1.98091E-07	0.000000087	4.52182E-08	3.04273E-07	5.34615E-08	2.80846E-07	0.000000096	1.44154E-07	1.40818E-07	1.25091E-07	4.43091E-07	9.96364E-08	3.96E-08	1.00133E-07	1.3204E-07	1.22E-08
Sphingomonas_insulae	0	3.66364E-08	8.25455E-09	3.76364E-08	3.34615E-08	0	0	1.90769E-08	0	0	3.33636E-08	7.13636E-09	0	0	0	1.86E-08
Sphingomonas_sanxanigenens	1.75509E-06	2.48182E-07	1.22027E-06	1.17355E-06	2.85462E-07	1.79231E-08	1.09946E-06	9.36692E-07	3.47727E-07	5.28636E-07	0.00000103	1.71091E-06	9.06E-08	1.57733E-07	2.008E-07	9.56E-08
Sphingomonas_sp._PAMC26645	0	3.49091E-08	5.04E-08	1.06064E-07	1.51385E-07	1.86769E-07	8.92308E-08	7.13077E-08	3.98182E-08	0.000000047	1.66455E-07	7.89091E-08	4.53333E-08	1.28467E-07	2.98667E-08	1.38267E-07
Sphingomonas_sp._HDW15B	1.72091E-07	0	1.04491E-07	2.77727E-07	0	0	1.40462E-07	2.46923E-08	3.98182E-08	3.89091E-08	0	3.94545E-08	2.37333E-08	5.73333E-08	5.01333E-08	5.21333E-08
Sphingomonas_sp._AP4-R1	2.56364E-08	1.04545E-08	1.22491E-07	3.35455E-08	0	0	6.41538E-08	0.000000011	1.15455E-07	0.000000093	6.88182E-08	4.92727E-08	0	0.000000033	1.57067E-08	8.19333E-08
Sphingomonas_sp._IC081	8.25455E-08	1.16364E-08	1.13673E-07	5.90909E-08	7.24615E-08	0	4.80769E-08	1.90769E-08	0	0.000000034	2.38727E-07	1.46727E-07	5.59333E-08	0	6.19733E-08	9.33333E-09

Sphingomonas_sp._AAP5	1.22727E-07	0	1.29345E-07	2.45545E-07	1.31154E-07	2.13846E-08	6.23077E-08	1.56769E-07	1.32091E-07	1.67273E-07	4.36364E-08	3.39909E-07	2.084E-07	1.056E-07	1.3484E-07	6.44667E-08
Sphingomonas_sp._HDW15C	7.72727E-08	3.69545E-07	8.25455E-09	1.76245E-07	6.69231E-08	0	0	3.22308E-08	1.30182E-07	3.62727E-08	0	1.67636E-07	1.02667E-07	1.61333E-08	0	9.08E-08
Sphingomonas_sp._LM7	1.63909E-07	0	1.65455E-08	5.30909E-08	0.000000151	2.52308E-08	9.74615E-08	0.000000342	1.78091E-07	6.66364E-08	1.57182E-07	1.87455E-07	1.396E-07	1.62267E-07	1.51107E-07	1.452E-07
Sphingomonas_sp._HDW15A	1.41109E-06	7.62273E-08	1.68464E-06	1.88627E-06	4.96154E-07	0	3.65269E-06	5.64754E-06	1.34127E-06	9.08273E-07	4.93455E-07	1.31491E-06	3.79067E-07	0.000000132	6.06933E-07	3.70267E-07
Sphingomonas_sp._XS-10	8.44545E-07	0	2.21536E-06	2.31655E-06	6.07154E-07	0	5.01538E-06	6.98946E-06	0.000001071	6.45545E-07	9.30182E-07	5.71091E-07	7.708E-07	5.15133E-07	7.454E-07	0.000000927
Sphingomonas_koreensis	6.25455E-08	0	5.92545E-08	6.84545E-08	0	1.75977E-06	3.45462E-07	3.41077E-07	0.000000406	2.02909E-07	4.36364E-08	1.93455E-07	1.398E-07	0.000000235	1.678E-07	1.87467E-07
Sphingomonas_panacis	8.23636E-08	6.65455E-08	3.70909E-08	4.88818E-08	3.34615E-08	0	7.54615E-08	5.74615E-08	3.75455E-08	0	1.02727E-08	7.71364E-08	4.58E-08	2.746E-07	5.264E-08	0.000000112
Sphingomonas_indica	5.73909E-07	0	1.15818E-07	4.93091E-07	2.90769E-07	0	3.11538E-08	2.76231E-07	8.64909E-07	2.70455E-07	5.20727E-07	4.05545E-07	7.23333E-08	3.96667E-07	0.000000413	6.29333E-07
Sphingomonas_alpina	0.000000718	5.05455E-08	1.40255E-06	5.58727E-07	1.95615E-07	6.41538E-08	1.09115E-06	0.00000068	7.03455E-07	4.08273E-07	9.79273E-07	8.23636E-07	1.916E-07	4.404E-07	2.606E-07	5.51267E-07
Sphingomonas_sp._LMO-1	1.00873E-06	0.00000013	4.61727E-07	8.57091E-07	2.33538E-07	5.05846E-07	1.10385E-07	7.17846E-07	5.70364E-07	8.08273E-07	4.77091E-07	9.86182E-07	1.78533E-07	3.488E-07	4.52267E-07	3.38467E-07
Sphingomonas_sp._CL5.1	1.03036E-06	6.49909E-07	7.53091E-08	5.20636E-08	0.000001754	0	2.63077E-08	5.13077E-08	1.21909E-07	2.16364E-07	0	1.91455E-07	1.5226E-06	1.8432E-06	2.72247E-06	1.86113E-06
Sphingomonas_sp._LK11	3.69091E-08	1.64091E-07	0	4.85182E-08	2.88462E-08	4.73846E-07	8.86154E-08	3.22308E-08	4.91818E-08	0	3.95455E-08	6.35455E-08	2.94667E-08	6.74667E-08	1.12667E-08	0
Sphingomonas_sp._NBWT7	1.48755E-06	3.50636E-07	0.000001795	1.61527E-06	6.29308E-07	1.19692E-07	1.41423E-06	1.84246E-06	0.000000693	7.80727E-07	1.10691E-06	1.24036E-06	1.27733E-07	3.93133E-07	4.45467E-07	2.59533E-07
Sphingomonas_sp._sand1-3	0	0	0	0	8.58846E-07	0	0	5.64538E-07	2.02727E-07	0.000000034	3.79091E-08	3.57273E-08	3.918E-07	7.38667E-07	0.000000662	6.48333E-07
Glycocaulis_alkaliphilus	9.97273E-08	0	0	2.10364E-07	0.00000012	0	1.07538E-07	2.47692E-08	1.10909E-07	1.13091E-07	7.53636E-08	1.59182E-07	5.88E-08	6.02667E-08	0.000000044	1.38067E-07
Hirschia_baltica	1.66227E-06	1.94545E-07	1.10909E-07	6.89909E-07	7.48231E-07	5.91538E-08	7.45385E-08	7.61538E-07	7.12636E-07	7.16909E-07	2.16309E-06	1.72118E-06	4.25067E-07	3.868E-07	5.724E-07	6.45867E-07
Hyphomonas_sp._CACIAM_19H1	0.000000123	1.16364E-08	1.30545E-07	1.09182E-07	3.24769E-07	0.000001239	8.86154E-08	1.00923E-07	1.42636E-07	1.33364E-07	3.37273E-07	2.45909E-07	1.836E-07	1.36733E-07	3.424E-07	5.51733E-07
Hyphomonas_neptunium	2.86455E-07	1.29364E-07	3.19764E-07	4.00909E-07	5.76154E-08	0	8.11538E-08	1.76308E-07	0.000000187	0.000000152	3.88909E-07	6.01818E-08	7.67333E-08	1.05267E-07	2.59333E-07	1.93133E-07
Hyphomonas_sp._Mor2	7.94727E-07	8.18182E-08	1.06636E-07	7.27545E-07	2.17308E-07	1.03285E-06	4.38692E-07	1.09769E-07	8.04364E-07	2.06545E-07	9.35727E-07	4.35727E-07	2.99467E-07	2.38133E-07	3.71333E-07	4.686E-07
Hyphobacterium_sp._CCMP332	1.85691E-06	1.66455E-07	5.434E-07	1.09509E-06	4.86538E-07	0	1.61192E-06	9.87077E-07	1.14309E-06	1.37055E-06	1.40664E-06	9.37636E-07	5.97333E-07	6.42667E-07	5.528E-07	8.78467E-07
Maricaulis_maris	1.46364E-07	0.000000062	1.40545E-07	7.30909E-08	1.29538E-07	0	1.89308E-07	2.44692E-07	3.98182E-08	3.03636E-08	3.05545E-07	3.57273E-08	2.10533E-07	2.508E-07	1.97267E-07	3.028E-07
Tabrizicola_piscis	0	0	0	2.39091E-08	3.58462E-08	5.44615E-07	6.66154E-08	3.81538E-08	0	0	0	1.17909E-07	6.70667E-08	5.81333E-08	2.058E-07	1.11333E-07
Litoreibacter_sp._LN3S51	0	0	0	0	5.74615E-08	3.15385E-07	3.11538E-08	7.15385E-08	0	0	0	1.42727E-08	8.12667E-08	1.75333E-07	7.96E-08	1.50067E-07
Tateyamaria_omphalii	2.56364E-08	0	0	0	0	0	5.74615E-08	0	0	0	0	3.74545E-08	0	0	3.88667E-08	3.97333E-08
Roseivivax_sp._THAF40	3.69091E-08	0	0	3.39091E-08	0	0	0	0	3.94545E-08	0.000000028	0	3.16364E-08	4.88667E-08	0	0	0
Roseivivax_sp._THAF30	3.69091E-08	0	0	5.18182E-09	0	0	0	0	0	0	0	0	0	0	1.12667E-08	0
Pontivivens_sp._MT2928	7.37273E-08	0	0	2.39091E-08	6.73846E-08	0	0	1.90769E-08	5.42727E-08	6.76364E-08	6.55455E-08	3.25364E-07	2.37333E-08	2.38E-08	2.09733E-08	8.76E-08
Stappia_indica	1.41091E-07	2.34545E-08	0	1.47273E-08	5.20769E-08	0	0	0	3.98182E-08	9.36364E-08	6.68182E-08	1.90727E-07	0	0	0	0
Stappia_sp._ES.058	6.74545E-08	0.000000065	0	1.36091E-07	4.06077E-07	1.64462E-07	6.55692E-07	6.55385E-08	3.94545E-08	0	8.29091E-08	3.07636E-07	1.46133E-07	1.91467E-07	2.01707E-07	1.36533E-07
Ketogulonicigenium_vulgare	3.11818E-08	0	0	0	3.15385E-08	0	2.63077E-08	0	2.72727E-08	5.22727E-08	0	3.09682E-07	0	0	0	0
Ketogulonicigenium_robustum	5.52727E-08	0	0	6.11818E-08	1.74462E-07	1.23846E-07	6.23077E-08	3.81538E-08	0.000000268	3.96364E-07	6.98727E-07	2.84818E-07	6.51333E-08	8.58E-08	4.44E-09	0
Sagittula_sp._P11	2.56364E-08	7.66364E-08	0	4.79091E-09	3.15385E-08	0	5.20923E-07	2.59462E-07	0	0	1.16818E-07	2.02182E-07	4.00667E-08	2.62E-08	0	0
Yoonia_vestfoldensis	1.54545E-07	2.76818E-07	0	5.81909E-07	2.05385E-08	0	5.54615E-08	0	1.02636E-07	1.61182E-07	2.61636E-07	0.000000588	4.88667E-08	0.000000081	5.23067E-08	1.286E-07
Pseudovibrio_sp._FO-BEG1	1.19727E-07	2.34545E-08	1.63636E-08	1.10909E-07	1.04231E-07	0	0.000000216	3.00769E-08	5.48182E-08	0.000000056	1.56364E-07	1.64091E-07	4.78E-08	3.09333E-08	1.12667E-08	0
Maribius_sp._THAF1	2.98182E-08	0.000000047	3.27273E-08	9.69091E-08	2.26923E-08	0	2.63077E-08	3.67692E-07	0	0	0	1.04636E-07	0	0	1.49333E-08	0
Roseicitreum_antarcticum	2.43727E-07	2.34545E-08	0	5.36364E-08	5.63846E-08	5.96615E-07	9.74615E-08	1.34692E-07	1.42455E-07	1.99273E-07	2.76364E-08	8.56364E-08	2.14E-08	1.43533E-07	1.044E-07	0.000000128
Brevirhabdus_pacifica	2.33091E-07	1.01182E-07	3.70909E-08	9.96364E-08	5.50769E-08	0	0	0	0	2.87273E-08	2.76364E-08	6.32727E-08	1.86667E-08	0	5.94667E-08	1.22E-08
Leisingera_methylohalidivorans	1.98455E-07	1.16364E-08	0	2.90909E-08	0	0	5.74615E-08	0.000000011	0	0.000000047	0	8.15909E-08	2.91333E-08	6.12667E-08	1.056E-07	7.56E-08
Leisingera_aquaemixtae	1.58182E-08	0	3.70909E-08	5.18182E-09	0	0	0	0	0	0	0	0	0	0	0	0
Pseudohalocynthiibacter_aestuariivivens	3.15455E-08	0	6.67273E-08	5.98182E-08	8.52308E-08	8.07692E-07	2.63077E-08	1.79385E-07	1.41909E-07	1.34909E-07	2.05455E-08	6.85182E-07	1.01467E-07	4.80667E-08	8.93333E-08	1.328E-07
Thioclava_nitratireducens	1.22818E-07	0	6.05364E-07	1.93636E-07	6.63846E-08	0	7.71385E-07	1.29608E-06	1.45364E-07	2.87273E-08	1.28273E-07	1.82545E-07	1.13267E-07	1.91267E-07	1.2244E-07	3.28667E-07
Halocynthiibacter_arcticus	1.54727E-07	1.47727E-07	0	1.55455E-08	3.15385E-08	6.41538E-08	5.74615E-08	0.000000088	1.60818E-07	1.19545E-07	0.000000337	2.65182E-07	5.58667E-08	1.48133E-07	2.99733E-08	2.566E-07
Roseibacterium_elongatum	1.15273E-07	8.91818E-08	8.70909E-08	0.000000052	1.25692E-07	7.29231E-08	1.60462E-07	1.46231E-07	1.68273E-07	0.000000246	3.33636E-08	5.70909E-08	6.51333E-08	1.606E-07	5.81067E-08	9.93333E-08
Silicimonas_algicola	3.69091E-08	0	0	5.24545E-08	2.05385E-08	3.43231E-07	9.03846E-08	3.81538E-08	1.75091E-07	2.11364E-07	1.33182E-07	1.78091E-07	1.86667E-08	9.55333E-08	1.49333E-08	1.00533E-07
Thalassococcus_sp._S3	7.27273E-08	3.49364E-07	6.23636E-08	1.32273E-07	8.74615E-08	5.94615E-07	2.33846E-07	8.16385E-07	8.16364E-08	1.08273E-07	1.14636E-07	1.55636E-07	1.434E-07	0.00000007	4.184E-08	3.85333E-08
Defluviimonas_alba	2.96182E-07	2.34545E-08	1.06636E-07	1.59818E-07	5.63846E-08	0	8.87692E-08	5.72308E-08	1.11636E-07	8.59091E-08	0.000000175	7.01455E-07	1.36067E-07	1.08533E-07	0	1.44533E-07
Antarctobacter_heliothermus	1.57545E-06	3.83909E-07	8.33545E-07	8.55455E-07	2.17154E-07	1.16646E-06	2.98154E-07	2.66154E-07	8.82545E-07	4.90727E-07	1.15064E-06	1.45227E-06	2.91467E-07	3.66867E-07	5.30067E-07	1.93933E-07
Epibacterium_mobile	2.87455E-07	1.67545E-07	3.87091E-07	9.53636E-08	5.63846E-08	2.16923E-08	5.36923E-08	2.15769E-07	4.76818E-07	1.67091E-07	2.48273E-07	3.21818E-07	2.09333E-08	2.56667E-07	0.000000041	2.81267E-07
Profundibacter_amoris	3.49273E-07	6.48045E-07	5.89455E-08	4.93909E-07	1.75923E-07	5.86154E-07	2.42385E-07	4.93846E-07	4.37636E-07	0.000000271	4.73727E-07	5.62545E-07	1.81933E-07	1.71933E-07	6.89333E-08	2.19667E-07
Pelagibaca_abyssi	0.00000013	0	4.12727E-09	0	3.15385E-08	0	7.66923E-08	5.72308E-08	0	6.74545E-08	6.88182E-08	9.38182E-08	1.24933E-07	3.50667E-08	0	4.01333E-08
Haematobacter_massiliensis	1.35182E-07	5.72273E-07	2.18545E-08	4.69727E-07	0	0.000000136	0	0.000000068	0.000000133	2.71455E-07	7.69091E-08	8.42727E-08	2.44E-08	1.58E-08	1.49333E-08	0
Marinovum_algicola	6.69818E-07	3.60909E-07	7.82182E-08	4.13727E-07	1.39692E-07	4.69231E-08	1.14923E-07	1.78615E-07	2.47909E-07	1.22545E-07	2.96727E-07	5.68455E-07	7.15333E-08	1.12267E-07	2.75573E-07	1.09267E-07
Confluentimicrobium_sp._EMB200-NS6	7.71818E-08	5.41818E-08	4.12182E-08	1.89636E-07	0	3.93692E-07	3.33077E-08	0.000000073	1.32182E-07	0	1.73273E-07	0.000000107	0	0.000000033	4.93067E-08	5.07333E-08
Boseongicola_sp._CCM32	2.14909E-07	4.25955E-07	6.99455E-08	2.80636E-07	2.12308E-07	3.68615E-07	5.93846E-07	2.06846E-07	3.26636E-07	1.29909E-07	1.76818E-07	3.92909E-07	4.16867E-07	3.80067E-07	1.96573E-07	2.30733E-07
Pseudopuniceibacterium_sp._HQ09	1.87609E-06	4.87409E-07	9.25073E-07	1.73727E-06	2.28308E-07	2.42692E-07	2.00262E-06	9.38692E-07	7.70364E-07	1.15182E-06	1.37536E-06	8.04818E-07	2.256E-07	5.198E-07	3.696E-07	2.39533E-07
Salipiger_pacificus	0	0.00000013	0	4.79091E-09	3.15385E-08	0	0	0	0	0	0	0	0	0.000000033	0	0
Salipiger_profundus	7.57455E-07	0.000000112	1.07036E-07	3.28273E-07	8.79231E-08	0	1.39538E-07	3.98231E-07	4.08273E-07	3.86273E-07	1.41182E-07	5.50455E-07	1.004E-07	7.87333E-08	3.4644E-07	3.59933E-07
Yangia_sp._CCB-MM3	1.83909E-07	0	8.25455E-09	3.82182E-07	0.000000054	0	8.69231E-08	1.90769E-08	0	1.81909E-07	1.02727E-08	2.33364E-07	1.07667E-07	1.596E-07	1.38467E-07	1.38933E-07
Oceanicola_sp._D3	2.19909E-07	0.000000047	6.58182E-08	7.61818E-08	2.15538E-07	0	1.51538E-07	8.13846E-08	1.14909E-07	3.17091E-07	2.26182E-07	6.93636E-08	1.13267E-07	3.42E-08	1.21933E-07	1.514E-07
Roseobacter_denitrificans	8.66364E-08	1.16364E-08	0	9.65455E-08	1.43615E-07	1.50808E-06	1.72769E-07	6.14615E-08	1.71545E-07	2.85091E-07	8.29091E-08	5.48455E-07	1.74667E-07	8.64667E-08	2.2124E-07	2.88667E-08
Roseobacter_litoralis	7.37273E-08	5.41818E-08	0	9.20909E-08	7.56154E-08	0	0	0	0	6.69091E-08	1.05091E-07	4.28182E-08	2.24E-08	0.000000078	4.38667E-08	7.09333E-08
Roseobacter_ponti	6.34909E-07	2.525E-07	0.000000178	1.78545E-07	2.20154E-07	0	1.09154E-07	1.58077E-07	2.58364E-07	1.06545E-07	2.86182E-07	2.22909E-07	1.19067E-07	9.26667E-08	0.000000217	5.10667E-08
Ruegeria_sp._PR1b	0	0	0	1.91818E-08	0	0	0	0	0	0	0	0	0	0	0	0
Ruegeria_sp._AD91A	0	0	0	2.23909E-07	0	0	7.43846E-08	0.000000042	6.68182E-08	0	0	4.21364E-08	0	0	7.43333E-08	0
Ruegeria_sp._THAF33	4.37636E-07	1.42636E-07	2.15091E-07	1.47909E-07	0	0	2.08923E-07	9.84615E-08	1.63909E-07	6.37273E-08	1.28273E-07	8.74545E-07	7.22667E-08	0.000000082	5.93333E-08	1.86533E-07
Ruegeria_pomeroyi	2.64455E-07	1.16364E-08	0	1.33091E-07	0.000000093	6.53077E-08	8.86154E-08	2.05231E-07	1.86909E-07	0	7.10909E-08	7.71364E-08	7.17333E-08	1.15133E-07	2.81533E-07	1.49933E-07
Ruegeria_sp._TM1040	0	0	0	5.27273E-08	1.33231E-07	0	6.41538E-08	1.09923E-07	1.20091E-07	1.18545E-07	1.75545E-07	2.85455E-08	6.18667E-08	1.97933E-07	0	3.40667E-08
Rhodobaca_barguzinensis	3.15455E-07	2.34545E-08	6.67273E-08	1.35064E-07	2.14923E-07	1.94538E-07	0	1.74462E-07	3.33273E-07	3.02818E-07	4.20636E-07	5.47091E-07	0.000000053	2.16267E-07	2.72867E-07	2.20933E-07
Roseovarius_indicus	3.85727E-07	0.000000065	3.70909E-08	4.88818E-08	2.06308E-07	8.73077E-08	3.80769E-07	4.76769E-07	3.75455E-08	2.59455E-07	3.95455E-08	0.000000066	0	1.474E-07	7.82667E-08	1.746E-07
Roseovarius_sp._MME-070	6.74545E-08	1.16364E-08	3.70909E-08	4.79091E-09	6.73846E-08	0	6.41538E-08	2.46923E-08	0.000000027	7.29091E-08	6.66364E-08	1.40364E-07	2.91333E-08	1.234E-07	0	1.86E-08
Roseovarius_sp._THAF9	0.000000047	3.35455E-08	4.93636E-08	7.81818E-08	0	0	0	9.03846E-08	0	2.87273E-08	0.000000039	0	2.37333E-08	4.91333E-08	4.44E-09	0
Monaibacterium_sp._ALG8	1.58273E-07	1.79109E-06	1.62855E-07	3.22545E-07	1.23231E-07	4.92538E-07	3.98538E-07	4.12615E-07	1.11909E-07	0.000000164	3.95455E-08	3.53364E-07	1.35867E-07	2.574E-07	3.946E-07	1.01733E-07
Octadecabacter_temperatus	0	0	0	0	3.34615E-08	0	0	0	0	0	0	0	0	0	0	0
Octadecabacter_arcticus	3.01455E-07	3.10364E-07	7.40909E-08	1.14273E-07	2.74923E-07	1.79231E-08	2.63077E-08	1.78769E-07	3.98182E-08	0.000000028	3.95455E-08	5.47818E-07	2.91333E-08	1.284E-07	4.83067E-08	1.50733E-07
Octadecabacter_sp._SW4	2.30909E-08	1.16364E-08	3.32727E-08	2.82545E-07	2.75385E-08	1.10985E-06	1.53308E-07	1.43308E-07	9.23636E-08	3.03636E-08	1.41909E-07	7.07273E-08	1.13933E-07	6.04667E-08	1.44333E-07	2.34067E-07
Octadecabacter_antarcticus	1.08545E-07	0	4.57636E-08	4.32455E-08	0	0	1.13077E-07	0.000000022	2.16364E-08	0	0	0	7.66E-08	7.47333E-08	1.18667E-07	2.44E-08
Celeribacter_indicus	3.74818E-07	0.000000182	4.10818E-07	1.76091E-07	6.65385E-08	3.83077E-08	3.22385E-07	1.88538E-07	3.85455E-07	2.14636E-07	4.53182E-07	1.72273E-07	1.014E-07	9.78E-08	2.49867E-07	0
Celeribacter_marinus	0.000000134	2.70909E-08	4.58455E-08	1.04136E-06	1.02308E-07	8.37923E-07	1.90192E-06	1.64138E-06	0.000000315	6.36364E-07	4.56364E-07	6.76545E-07	1.056E-07	1.65867E-07	1.39307E-07	0.000000097
Celeribacter_ethanolicus	4.05182E-07	0.000000065	7.20909E-08	6.99636E-07	7.80615E-07	1.42077E-07	5.24846E-07	1.25654E-06	2.96364E-07	3.17636E-07	6.79455E-07	9.31818E-07	9.63333E-08	1.17333E-07	3.31667E-07	3.324E-07
Celeribacter_baekdonensis	1.52909E-07	5.05455E-08	4.11182E-07	8.64273E-07	9.09231E-08	0.000000295	2.06454E-06	1.36131E-06	1.88273E-07	4.01636E-07	1.89273E-07	0.000000227	8.32E-08	3.492E-07	3.79267E-07	1.56533E-07
Celeribacter_manganoxidans	9.18364E-07	0	2.90364E-07	5.69727E-07	0.000000151	4.22692E-07	1.77077E-07	1.53231E-07	5.13818E-07	8.59091E-08	1.09036E-06	4.62091E-07	1.41533E-07	3.11667E-07	2.64533E-07	3.986E-07
Planktomarina_temperata	1.66727E-07	0.000000065	9.59455E-08	1.53273E-07	1.99231E-07	4.60846E-07	1.65846E-07	2.88462E-07	3.18273E-07	2.62182E-07	2.00455E-07	1.47636E-07	4.21533E-07	3.904E-07	4.04333E-07	2.91133E-07
Rhodobacter_sp._LPB0142	1.94545E-07	0	0	2.39091E-08	6.73846E-08	2.52308E-08	0	4.24769E-07	2.33364E-07	0.000000056	4.00909E-07	3.71909E-07	4.81333E-08	8.18667E-08	7.51333E-08	1.96333E-07
Rhodobacter_sp._CZR27	1.77545E-07	1.16364E-08	0	1.45273E-07	0	0	2.63077E-08	0	1.04273E-07	1.39182E-07	8.26364E-08	0	4.74E-08	0	4.20667E-08	1.40667E-07
Rhodobacter_capsulatus	2.20091E-07	0	1.27273E-07	4.24273E-07	8.20154E-07	1.00154E-06	1.09923E-07	6.62769E-07	4.16182E-07	2.15182E-07	2.94091E-07	2.02727E-07	2.53867E-07	1.404E-07	3.85733E-07	4.252E-07
Rhodobacter_blasticus	3.27182E-07	8.84545E-08	2.22309E-07	2.08909E-07	9.49231E-08	2.16923E-08	2.04385E-07	6.57692E-08	2.15273E-07	1.40818E-07	2.40727E-07	4.00455E-07	2.94667E-08	5.47333E-08	1.606E-07	5.10667E-08
Rhodobacter_sphaeroides	2.65209E-06	5.11364E-07	2.85923E-06	2.84045E-06	2.52538E-07	1.33462E-07	2.75615E-07	1.06415E-06	1.14282E-06	1.37818E-06	2.08209E-06	1.67255E-06	2.93933E-07	4.71467E-07	4.60133E-07	7.064E-07
Thalassobius_gelatinovorus	2.56364E-08	0	0	7.32455E-08	9.09231E-08	0	0	0	0	6.27273E-08	0	2.63727E-07	9.75333E-08	1.62533E-07	5.09333E-08	1.59867E-07
Dinoroseobacter_shibae	3.62727E-08	0	0	1.43636E-08	1.03308E-07	1.14615E-07	0	3.75231E-07	2.75455E-08	0	3.79091E-08	0.000000184	0.000000075	9.66E-08	1.248E-07	6.26667E-08
Pseudorhodobacter_sp._S12M18	2.62909E-07	0.000000022	1.63636E-08	7.28182E-08	1.50231E-07	4.11769E-06	7.05308E-07	7.90308E-07	0.00000049	8.39909E-07	5.52455E-07	1.38273E-07	3.024E-07	5.72267E-07	6.254E-07	3.43733E-07
Mameliella_alba	2.35909E-07	0	0	9.93636E-08	1.45538E-07	0	3.06923E-08	2.03769E-07	2.19727E-07	1.44727E-07	5.15727E-07	2.26364E-07	4.86E-08	1.306E-07	8.61067E-08	9.33333E-09
Marivivens_sp._JLT3646	0.00000049	0	2.87273E-08	5.18182E-09	8.12154E-07	0	0	0	0	0	0	0	2.468E-07	5.322E-07	6.46333E-07	2.178E-07
Jannaschia_sp._CCS1	1.32582E-06	2.17273E-07	1.85209E-07	2.53727E-07	3.91385E-07	0	9.21385E-07	9.31231E-07	0.000000709	0.000000273	4.65818E-07	0.000000527	2.50533E-07	4.14933E-07	2.25933E-07	1.76867E-07
Paraoceanicella_profunda	1.91082E-06	4.45273E-07	6.26818E-07	1.94355E-06	3.90308E-07	4.73846E-08	6.65769E-07	1.48777E-06	1.09236E-06	1.02291E-06	4.72636E-07	8.67727E-07	7.28067E-07	6.022E-07	0.000000863	9.538E-07
Parasedimentitalea_marina	2.48091E-07	2.06182E-07	0	4.45455E-07	3.53615E-07	2.52308E-08	1.65231E-07	0.000000461	3.67273E-07	1.74273E-07	2.74455E-07	9.38182E-08	1.17333E-07	3.12067E-07	5.63467E-07	1.65267E-07
Rhodovulum_sulfidophilum	3.69091E-08	0	3.70909E-08	1.06909E-07	5.19231E-08	9.13077E-08	2.63077E-08	3.29231E-08	5.79091E-08	0	0	1.42727E-08	9.56E-08	7.58E-08	1.526E-07	1.22E-08
Rhodovulum_sp._MB263	0	3.23182E-08	7.82182E-08	0.000000052	3.15385E-08	0	0	7.89231E-08	3.75455E-08	0	0	7.13636E-09	5.08667E-08	3.48E-08	0	0
Rhodovulum_sp._P5	1.71818E-07	1.16364E-08	4.12727E-09	1.31064E-07	2.56846E-07	8.66154E-08	1.44462E-07	3.42077E-07	0.00000016	1.54273E-07	1.93727E-07	0.00000014	1.02267E-07	1.38067E-07	1.59773E-07	1.668E-07
Paracoccus_mutanolyticus	0	0	0	0	0	0	0	0	0	0	0	7.13636E-09	0	0	0	0
Paracoccus_sp._BM15	0	0	0	1.03636E-08	0	0	0	0	6.61818E-08	0	0	7.13636E-09	0	0.000000041	1.49333E-08	0
Paracoccus_pantotrophus	0.000000134	0	3.70909E-08	3.55182E-08	3.58462E-08	0	0	0	0	0	0	0	2.37333E-08	6.18E-08	1.00667E-07	0
Paracoccus_aminovorans	1.58182E-07	0	0	8.40909E-08	0.000000104	8.84615E-08	5.54615E-08	0.000000104	2.11091E-07	0	0	4.45909E-08	2.17867E-07	9.57333E-08	1.306E-07	1.09067E-07
Paracoccus_sanguinis	2.88727E-07	1.24227E-07	4.90909E-08	1.62064E-07	1.17692E-07	8.84615E-08	0	5.81538E-08	1.25818E-07	2.87273E-08	1.44455E-07	1.73455E-07	5.28667E-08	1.00333E-07	2.05267E-07	9.54E-08
Paracoccus_sp._SC2-6	1.02727E-07	0	5.34545E-08	1.43636E-08	3.58462E-08	0	5.26154E-08	0	0	0.000000034	2.76364E-08	7.44545E-08	1.104E-07	1.33333E-07	9.25333E-08	3.72E-08
Paracoccus_contaminans	0.00000006	1.37273E-08	0.000000047	8.75455E-08	0	6.26923E-07	2.40231E-07	0	5.79091E-08	5.22727E-08	0	2.14545E-08	2.14E-08	4.64667E-08	0	7.09333E-08
Paracoccus_jeotgali	3.69091E-08	0	0	1.95455E-08	0	9.03846E-07	2.38154E-07	3.57846E-07	3.54727E-07	0.00000016	2.94364E-07	3.88818E-07	1.066E-07	2.57667E-07	1.54867E-07	3.95933E-07
Paracoccus_zhejiangensis	3.86545E-07	1.04727E-07	9.60909E-08	4.20909E-07	0	2.67308E-07	3.67538E-07	2.97231E-07	0.000000027	2.24909E-07	2.44455E-07	4.06364E-07	7.30667E-08	7.88E-08	5.10667E-08	1.23267E-07
Paracoccus_aminophilus	2.99273E-07	4.24091E-08	2.22727E-07	1.34927E-06	2.10077E-07	0	1.05385E-07	1.09462E-07	7.16727E-07	1.02245E-06	0.000000692	1.15055E-06	0.000000295	1.40933E-07	5.224E-07	3.79867E-07
Paracoccus_sp._AK26	9.55455E-08	2.20909E-08	4.12182E-08	3.45182E-08	6.23077E-08	6.76923E-08	8.11538E-08	1.71077E-07	5.48182E-08	5.22727E-08	0	2.14545E-08	2.14E-08	0	0	5.88667E-08
Paracoccus_denitrificans	0.000000125	3.96727E-07	1.33309E-07	2.89364E-07	5.63846E-08	0	0	9.03846E-08	1.10455E-07	1.26273E-07	1.49091E-07	9.21364E-07	1.36533E-07	1.37267E-07	1.49333E-08	8.92667E-08
Paracoccus_kondratievae	0.000000092	0	2.04909E-08	5.16364E-08	5.02308E-08	3.64615E-07	2.63077E-08	6.14615E-08	1.61273E-07	0.000000047	3.95455E-08	7.13636E-09	0	5.18E-08	0	0
Paracoccus_liaowanqingii	1.24182E-07	3.53545E-07	6.99455E-08	0.000000081	1.24154E-07	5.65077E-07	9.38462E-08	0	0.000000084	2.85455E-08	1.12455E-07	3.38364E-07	7.58667E-08	1.62467E-07	1.778E-07	1.32733E-07
Paracoccus_yeei	2.99091E-07	0	1.38173E-07	5.07182E-07	7.17692E-08	0	3.06923E-08	7.95385E-08	0	1.37727E-07	4.01818E-07	5.70909E-08	0	0	0	1.86E-08
Paracoccus_sp._Arc7-R13	7.79636E-07	5.97273E-08	2.16273E-07	5.85727E-07	9.62308E-08	1.49223E-06	2.77385E-07	2.71538E-07	4.09455E-07	2.95818E-07	0.000000977	0.000000662	1.388E-07	3.48E-08	2.72333E-07	8.19333E-08
Gemmobacter_sp._HYN0069	1.62145E-06	1.53455E-07	3.43182E-07	0.000000717	4.70615E-07	2.95385E-08	1.68538E-07	5.24154E-07	7.37545E-07	6.02364E-07	2.05727E-06	1.37582E-06	2.86467E-07	6.088E-07	5.33333E-07	5.23933E-07
Phaeobacter_piscinae	0	0	0	0	0	0	0	0	0	0	0	7.13636E-09	2.09333E-08	0	0	0
Phaeobacter_porticola	0	0	0	4.79091E-09	0	0	0	0	0	3.62727E-08	3.77273E-08	7.13636E-09	2.37333E-08	6.60667E-08	7.884E-08	1.98667E-08
Phaeobacter_inhibens	1.21018E-06	3.79091E-08	5.24545E-07	8.36182E-07	2.39769E-07	5.96154E-07	4.40154E-07	8.65462E-07	0.000000629	3.37091E-07	9.51636E-07	4.01727E-07	2.95133E-07	4.25067E-07	4.35467E-07	3.39333E-07
Phaeobacter_gallaeciensis	1.97827E-06	6.60636E-07	1.03018E-06	0.000000883	4.02615E-07	1.16564E-05	2.86315E-06	3.28577E-06	1.04873E-06	7.56636E-07	1.37191E-06	1.96309E-06	6.99267E-07	5.96067E-07	4.96667E-07	9.44133E-07
Labrenzia_aggregata	0	0	0	0	0	0	5.36923E-08	5.22308E-08	0	9.77273E-08	0	7.07273E-08	0	0	0	0
Labrenzia_sp._CP4	5.93636E-08	0	0	0	2.75385E-08	6.41538E-08	0	1.90769E-08	6.67273E-08	1.58545E-07	2.76364E-08	6.32727E-08	1.016E-07	1.61333E-08	5.35333E-08	1.59867E-07
Labrenzia_sp._VG12	1.47727E-07	0	7.90909E-08	9.67273E-08	1.46538E-07	3.36769E-07	5.96154E-08	1.90769E-08	1.82545E-07	0.000000028	4.98182E-08	2.91273E-07	5.32E-08	3.26667E-08	4.12E-08	2.10933E-07
Labrenzia_alexandrii	2.30273E-07	0	2.59818E-08	1.557E-07	4.82846E-07	5.34615E-07	6.16077E-07	2.76385E-07	7.00182E-07	1.11182E-07	2.92909E-07	3.66455E-07	1.83467E-07	1.786E-07	4.88333E-07	0.000000344
Labrenzia_sp._PHM005	9.77273E-08	4.11818E-08	4.12727E-09	4.79091E-09	8.33846E-08	4.27692E-08	2.58154E-07	1.42769E-07	0.00000038	1.25182E-07	2.48455E-07	4.56091E-07	5.08667E-08	8.98667E-08	1.23667E-07	2.79133E-07
Labrenzia_sp._THAF82	1.91909E-07	1.42364E-06	1.87873E-06	1.50536E-06	2.45385E-07	5.86846E-07	4.57077E-07	0.000000921	7.81727E-07	3.20727E-07	5.14364E-07	4.67591E-07	3.612E-07	2.46133E-07	2.45867E-07	5.42067E-07
Pannonibacter_phragmitetus	1.57909E-06	5.38591E-07	1.60391E-07	1.20082E-06	1.60138E-06	1.23369E-06	3.53769E-06	2.25385E-06	2.28609E-06	2.07464E-06	2.18655E-06	0.00000244	3.1954E-06	2.83567E-06	2.00793E-06	3.2244E-06
Sulfitobacter_sp._SK025	0	0	0	0	0	0	3.11538E-08	0	0	0	0	0	0	0	0	0
Sulfitobacter_guttiformis	0	8.86364E-09	0	0	0	0	0	0	2.75455E-08	5.22727E-08	2.76364E-08	0	0	0	0	0
Sulfitobacter_sp._D7	0	6.21818E-08	0	1.04727E-07	1.32538E-07	9.38462E-08	3.11538E-08	6.95385E-08	2.72727E-08	1.95727E-07	3.95455E-08	6.35455E-08	2.778E-07	8.07333E-08	1.05973E-07	9.43333E-08
Sulfitobacter_sp._AM1-D1	5.41818E-08	2.72727E-08	1.10909E-07	3.02727E-08	6.19231E-08	0.000000026	4.33846E-08	0	3.75455E-08	0	6.65455E-08	1.00364E-07	2.09333E-08	0	3.28E-08	9.33333E-09
Sulfitobacter_sp._SK011	4.09182E-07	0.000000065	1.06818E-07	1.65727E-07	1.37923E-07	0	0	2.10154E-07	1.12727E-07	1.22182E-07	2.04091E-07	1.80818E-07	2.08933E-07	0.000000103	5.98867E-07	3.04467E-07
Sulfitobacter_pontiacus	0.000000286	0	0	2.97182E-07	0.00000007	0	0	0.000000022	1.18545E-07	3.54636E-07	3.25818E-07	1.17273E-07	4.48667E-08	2.09267E-07	2.362E-07	1.50267E-07
Sulfitobacter_sp._BSw21498	3.47636E-07	1.55273E-07	2.87273E-08	3.33909E-07	3.58462E-08	7.00154E-07	2.41923E-07	2.36231E-07	4.45455E-08	0.000000056	1.14636E-07	3.17273E-07	5.28667E-08	1.05333E-07	2.20533E-07	2.05667E-07
Sulfitobacter_sp._SK012	2.77455E-07	0	6.98182E-08	9.54545E-09	0.000000054	0	5.26154E-08	3.56923E-08	3.75455E-08	1.36636E-07	2.06727E-07	9.85455E-08	4.88E-08	0.000000014	0	4.95333E-08
Sulfitobacter_sp._THAF37	2.96364E-08	0	4.12727E-09	9.97273E-09	0	1.98462E-07	6.23077E-08	0	0	0	1.06545E-07	7.13636E-09	0	0	0	0
Sulfitobacter_pseudonitzschiae	0.000000244	0.000000065	3.70909E-08	2.02245E-07	1.21692E-07	0	2.55077E-07	1.90923E-07	2.34545E-07	8.32727E-08	8.93636E-08	4.28182E-08	1.124E-07	1.49333E-07	1.718E-07	1.99467E-07
Sulfitobacter_sp._JL08	2.81727E-07	0	3.90273E-07	1.50909E-07	4.13846E-08	0	7.54615E-08	8.50769E-08	1.73636E-07	2.76545E-07	4.40182E-07	2.66455E-07	0.000000027	1.44533E-07	1.49733E-07	9.34667E-08
Hartmannibacter_diazotrophicus	0	8.13636E-08	2.00909E-07	2.68182E-07	0	2.52308E-08	2.63077E-08	0	2.75455E-08	3.62727E-08	9.64545E-08	6.41364E-08	0	0	6.47333E-08	2.214E-07

Pseudorhodoplanes_sinuspersici	3.78273E-07	3.81727E-07	2.03909E-07	3.76364E-08	0	2.52308E-08	1.80923E-07	1.20923E-07	1.34364E-07	0.000000047	1.37273E-07	1.52545E-07	0	6.18E-08	4.44E-09	0
Lichenihabitans_psoromatis	1.95818E-07	1.08182E-07	1.16818E-07	2.29273E-07	3.15385E-08	8.52308E-08	0	0	5.07273E-07	7.73636E-08	9.99091E-08	1.47455E-07	6.08E-08	3.15333E-08	0	7.25333E-08
Chelatococcus_sp._CO-6	2.54273E-07	1.94545E-07	0	1.47273E-08	6.46923E-08	0.00000029	0	1.23077E-07	1.78273E-07	9.11818E-08	1.53091E-07	4.87364E-07	2.14667E-08	6.18E-08	1.33333E-08	0
Chelatococcus_daeguensis	0.000000226	4.65455E-08	9.03091E-08	3.42818E-07	1.04231E-07	6.27692E-08	0	5.22308E-08	8.72727E-08	0.000000034	3.22182E-07	2.65273E-07	2.14E-08	1.47867E-07	8.93733E-08	3.602E-07
Candidatus_Hodgkinia_cicadicola	2.56364E-08	0	5.74E-08	1.007E-07	3.15385E-08	2.62462E-07	3.06923E-08	0	4.45455E-08	0.000000028	6.65455E-08	3.16364E-08	0	2.62E-08	2.05773E-07	1.98667E-08
Cohaesibacter_sp._ES.047	7.82182E-07	6.08727E-07	1.52518E-07	7.73273E-07	1.38308E-07	1.67385E-06	2.40269E-06	1.63346E-06	5.66091E-07	7.33455E-07	8.07364E-07	6.96636E-07	2.62333E-07	4.868E-07	6.22067E-07	2.61667E-07
Breoghania_sp._L-A4	9.68636E-07	2.90727E-07	1.83909E-06	2.56845E-06	5.16077E-07	2.95385E-08	0.000006478	5.89862E-06	0.000001403	8.97364E-07	8.85818E-07	5.80727E-07	6.116E-07	3.17267E-07	6.18667E-07	6.824E-07
Azorhizobium_caulinodans	6.70909E-08	5.05455E-08	0	0.000000042	0	0	0	5.47692E-08	5.79091E-08	0	6.66364E-08	7.13636E-09	6.64667E-08	0	0	4.04E-08
Starkeya_novella	6.85273E-07	0	3.70909E-08	0.000000472	6.65385E-08	8.77154E-07	9.56154E-08	3.29231E-08	0.000000173	7.29091E-08	4.92727E-08	2.36727E-07	1.68067E-07	6.26667E-08	1.636E-07	7.86E-08
Starkeya_sp._ORNL1	5.41818E-08	0.00000013	1.28818E-08	1.04973E-07	2.05385E-08	1.06538E-07	3.77692E-08	3.93846E-08	8.67273E-08	0	1.02727E-08	5.03273E-07	2.378E-07	4.72667E-08	1.408E-07	4.37733E-07
Xanthobacter_autotrophicus	3.63727E-07	1.16364E-08	4.12727E-09	1.45364E-07	0.000000929	3.11538E-08	2.66692E-07	1.07077E-07	1.24882E-06	5.54909E-07	3.95091E-07	7.73364E-07	3.35533E-07	2.77133E-07	1.45867E-07	3.58733E-07
Ancylobacter_sp._TS-1	0	0	0	1.43636E-08	0	0	3.77692E-08	4.32308E-08	5.79091E-08	1.47727E-07	2.76364E-08	1.36455E-07	7.46667E-08	1.57133E-07	3.88667E-08	6.61333E-08
Ancylobacter_pratisalsi	1.00818E-07	0	4.12727E-09	3.86609E-07	0	4.48154E-07	0	3.57692E-08	5.97273E-08	0	1.97727E-07	2.61455E-07	2.64E-08	0	6.384E-08	1.408E-07
Labrys_sp._KNU-23	0.000000089	2.14818E-07	1.23636E-08	1.19636E-07	1.43538E-07	1.13538E-07	2.63077E-08	1.46923E-08	1.01545E-07	0.000000081	0	5.64545E-08	0	6.44667E-08	2.09733E-08	4.28E-08
Pseudolabrys_sp._FHR47	5.42727E-08	1.70909E-07	2.04909E-08	3.47273E-08	8.55385E-08	1.89231E-08	0	6.10769E-08	0	6.92727E-08	4.36364E-08	7.37727E-08	2.09333E-08	0.00000007	0	1.98667E-08
Pseudolabrys_taiwanensis	3.17909E-07	0	1.86636E-07	2.63273E-07	1.28538E-07	0	3.77692E-08	8.75385E-08	2.75545E-07	3.88273E-07	3.11091E-07	2.76182E-07	5.04E-08	7.26E-08	9.15733E-08	1.568E-07
Parvibaculum_lavamentivorans	4.30636E-07	2.30909E-08	4.61527E-07	0.000000071	8.18077E-07	1.28692E-06	5.26154E-08	9.63077E-08	1.06827E-06	2.88091E-07	6.99091E-07	5.52182E-07	4.84667E-08	3.71333E-07	2.36133E-07	3.64933E-07
Pyruvatibacter_mobilis	9.31273E-07	5.38364E-07	1.65127E-07	5.72455E-07	4.46308E-07	0.000000241	3.82154E-07	2.66615E-07	5.32909E-07	5.49182E-07	7.82545E-07	8.78727E-07	3.40067E-07	4.09067E-07	5.01667E-07	4.246E-07
Candidatus_Phaeomarinobacter_ectocarpi	2.01364E-07	0.000000065	7.40909E-08	1.33455E-07	4.54615E-08	5.91538E-08	1.13231E-07	1.00692E-07	1.35909E-07	3.51545E-07	6.32636E-07	2.12455E-07	4.61333E-08	6.93333E-08	5.17333E-08	9.33333E-09
Methyloligella_sp._GL2	7.31818E-08	0	1.42218E-07	1.28091E-07	1.21385E-07	3.31538E-07	2.63077E-08	0.000000011	2.18545E-07	2.20909E-07	4.12455E-07	2.93818E-07	7.10667E-08	1.14267E-07	2.52107E-07	1.86E-08
Methyloceanibacter_caenitepidi	3.69091E-08	0	0	4.79091E-09	1.02769E-07	1.18462E-07	1.39923E-07	1.39615E-07	5.79091E-08	0.000000034	0	0.000000035	1.54867E-07	0.000000014	5.45733E-08	2.00467E-07
Methyloceanibacter_sp._wino2	5.07091E-07	0	1.10909E-07	4.15909E-07	1.02846E-07	1.89231E-08	0.000000194	2.59385E-07	1.68091E-07	0.000000191	0	3.83818E-07	1.41467E-07	6.78E-08	1.012E-07	4.58667E-08
Nordella_sp._HKS_07	8.72182E-07	0	1.34573E-06	2.15536E-06	3.75308E-07	0	4.19385E-06	5.42023E-06	0.000001014	7.22727E-07	1.12164E-06	5.72455E-07	4.364E-07	4.40267E-07	8.79133E-07	8.18067E-07
Ochrobactrum_pituitosum	1.25909E-07	0.00000026	0	7.02455E-08	2.16769E-07	4.30308E-07	1.88385E-07	2.11615E-07	2.60273E-07	1.17909E-07	0	1.13545E-07	5.01333E-08	1.71333E-08	2.39333E-08	5.10667E-08
Ochrobactrum_sp._MT180101	5.42727E-08	7.36364E-08	2.59091E-07	0.00000022	0	0	0	0	2.75455E-08	0	2.76364E-08	4.59091E-08	0	0.000000014	0	4.76667E-08
Ochrobactrum_pseudogrignonense	6.19091E-08	1.06955E-07	8.25455E-09	0	5.02308E-08	1.27077E-07	3.49077E-07	5.81538E-08	0.000000087	1.14273E-07	0	0	1.666E-07	0	1.39933E-07	0.00000008
Ochrobactrum_quorumnocens	8.84182E-07	0	3.82309E-07	6.71636E-07	1.03923E-07	5.16154E-07	2.83538E-07	2.44923E-07	0.000000502	5.81182E-07	5.17273E-07	0.000000817	3.94533E-07	2.64133E-07	2.124E-07	2.45733E-07
Ochrobactrum_anthropi	3.62727E-08	0	0	4.79091E-09	0	0	0	3.57692E-08	0	6.37273E-08	0	7.13636E-09	9.29333E-08	5.09333E-08	0	8.19333E-08
Brucella_melitensis	0	0	0	0	0	0	0	0	0	0	0	0	0	1.58E-08	0	0
Brucella_inopinata	3.62727E-08	0.000000065	0	0	0	0	0	0	0	0	0	0	0	0	0	0
Brucella_ovis	0	0	0	3.07273E-08	0	0	0	0	5.97273E-08	0	3.95455E-08	0	2.44E-08	4.96667E-08	4.62667E-08	2.92E-08
Brucella_sp._2280	0	0	1.75455E-08	0	0	0	0	0	0	0	0	0	0	0	0	0
Brucella_intermedia	4.44182E-07	1.81273E-07	2.03982E-07	2.95273E-07	1.80154E-07	3.31538E-07	3.33077E-08	0.00000013	6.01455E-07	1.16636E-07	3.47727E-07	1.67364E-07	5.01467E-07	2.68467E-07	3.70173E-07	3.20867E-07
Aureimonas_altamirensis	5.39182E-07	8.12727E-08	2.95455E-07	3.86909E-07	0	0	1.19615E-07	1.21692E-07	2.24909E-07	1.64091E-07	3.77273E-08	6.58364E-07	8.39333E-08	6.56667E-08	8.26E-08	2.42467E-07
Aureimonas_sp._AU20	3.66727E-07	0.000000071	4.12727E-09	9.44545E-08	0.000000039	1.27231E-07	6.44615E-08	3.57692E-08	1.90727E-07	1.46364E-07	3.49182E-07	2.22818E-07	0	7.53333E-08	0	3.67333E-08
Aureimonas_sp._LMG_31693	5.57273E-08	0	0	0	0	3.15385E-08	0	0	5.97273E-08	2.87273E-08	0	0	2.94667E-08	5.09333E-08	1.49333E-08	4.62667E-08
Martelella_endophytica	1.64691E-06	1.87636E-07	8.97091E-07	0.000001449	4.10769E-07	1.89154E-07	1.34215E-06	1.00346E-06	2.56218E-06	1.15218E-06	1.54191E-06	1.44264E-06	3.67867E-07	2.338E-07	0.000000525	5.28667E-07
Martelella_mediterranea	0.000000199	2.34545E-08	4.92182E-08	1.42364E-07	0.000000117	1.67615E-07	1.24769E-07	3.37692E-08	0	1.09636E-07	1.17545E-07	2.18455E-07	1.47267E-07	1.30867E-07	1.9084E-07	2.754E-07
Martelella_sp._AD-3	1.16718E-06	0	4.12182E-08	2.93364E-08	1.80185E-06	0	3.06923E-08	0	2.25273E-06	1.69145E-06	9.20727E-07	1.61945E-06	2.3224E-06	1.93713E-06	1.78767E-06	2.52673E-06
Methylovirgula_ligni	2.98182E-08	0	3.70909E-08	1.54091E-07	0	0	5.54615E-08	0	1.34364E-07	0.000000028	2.69455E-07	1.80182E-07	0	0	7.59333E-08	0.000000102
Beijerinckia_indica	3.05909E-07	0	1.59373E-07	0.000000359	8.77692E-08	5.96923E-08	2.36692E-07	1.90077E-07	7.15455E-08	0.000000103	1.02182E-07	2.16545E-07	4.74667E-08	1.29867E-07	5.54E-08	6.33333E-08
Methylocella_tundrae	4.12636E-07	0	1.52345E-07	0.00000007	1.43538E-07	7.21538E-08	5.96154E-08	7.07769E-07	3.67182E-07	6.37273E-08	2.18727E-07	7.37727E-08	3.75667E-07	5.23067E-07	8.49133E-07	7.006E-07
Methylocella_silvestris	2.08536E-06	0.000000177	8.45909E-07	1.06964E-06	6.55692E-07	1.17872E-05	2.49462E-06	0.000004423	1.60282E-06	9.65364E-07	1.85155E-06	1.39145E-06	0.000000846	1.0192E-06	0.000000517	1.6444E-06
Methylorubrum_extorquens	0	2.84545E-08	1.75455E-08	3.55182E-08	3.58462E-08	2.13846E-08	1.51538E-07	0	2.75455E-08	0	3.79091E-08	8.55455E-08	4.01333E-08	2.36267E-07	8.93067E-08	1.86667E-08
Methylorubrum_populi	0.00000113	1.53455E-07	1.81909E-06	2.60491E-06	3.44154E-07	1.73846E-07	4.44323E-06	0.000005371	5.65091E-07	0.000000745	6.25727E-07	6.68273E-07	5.518E-07	4.87933E-07	9.64667E-07	9.284E-07
Microvirga_sp._17_mud_1-3	9.88182E-08	9.38182E-08	0.000000093	1.83636E-07	3.88462E-08	2.13846E-08	0	3.81538E-08	5.45455E-08	5.22727E-08	1.02727E-08	0	9.88667E-08	9.78667E-08	0.000000039	9.74E-08
Microvirga_ossetica	8.69091E-08	1.16364E-08	0	1.32818E-07	0.000000077	1.41769E-07	4.83538E-07	1.57154E-07	0	2.87273E-08	2.90545E-07	2.91273E-07	9.62667E-08	1.664E-07	1.5844E-07	0.000000131
Microvirga_thermotolerans	3.39455E-07	0.000000642	7.80727E-08	3.54545E-07	1.54692E-07	1.39062E-06	6.13846E-08	3.26462E-07	2.65545E-07	9.11818E-08	0.000000262	6.89455E-07	5.36667E-08	1.15933E-07	2.60373E-07	1.70467E-07
Methylobacterium_sp._XJLW	0	0	0	0	0	3.15385E-08	0	0	0	0	0	0	0	0	0	0
Methylobacterium_sp._AMS5	0	0	0	0	0	4.13846E-08	0	0	0	0	0	0	2.09333E-08	0	0	0
Methylobacterium_phyllosphaerae	0	0	0	0	0	3.15385E-08	0	0	0	0	0	0	0	0	1.93733E-08	0
Methylobacterium_currus	0	0	0	4.40909E-08	3.58462E-08	2.40615E-07	4.33846E-08	1.90692E-07	5.79091E-08	0	0	2.45136E-07	2.94667E-08	0	7.37333E-08	0
Methylobacterium_sp._C1	5.68182E-08	0	0	4.79091E-09	0	0	0	0	0	0	0	0	0	0	0	0
Methylobacterium_sp._DM1	2.96364E-08	0	0	5.88818E-08	0	0	9.88462E-08	0	0	3.89091E-08	2.76364E-08	7.13636E-09	0	1.58E-08	1.93733E-08	0
Methylobacterium_sp._17Sr1-1	0	0	0	3.72455E-08	2.88462E-08	6.45231E-07	5.74615E-08	8.70769E-08	0	9.11818E-08	3.95455E-08	7.13636E-09	0	0	3.14667E-08	0
Methylobacterium_mesophilicum	0	0	3.70909E-08	0	1.86923E-08	4.13846E-08	3.11538E-08	0	0	8.02727E-08	6.88182E-08	2.14545E-08	0	0	0	0
Methylobacterium_sp._4-46	2.84364E-07	3.64364E-07	1.63636E-08	1.55455E-08	1.42462E-07	8.30769E-08	5.26154E-08	1.06308E-07	3.74545E-07	2.60727E-07	1.73727E-07	2.08909E-07	1.83733E-07	1.65933E-07	1.9624E-07	2.87933E-07
Methylobacterium_radiotolerans	3.15455E-08	2.70909E-08	3.70909E-08	1.91818E-08	0	0	6.96923E-08	0.000000011	0	0	8.29091E-08	3.33364E-07	7.93333E-08	5.70667E-08	4.83067E-08	6.19333E-08
Methylobacterium_aquaticum	0	0	0	3.99091E-08	3.58462E-08	2.95385E-08	5.26154E-08	1.27154E-07	3.98182E-08	2.85455E-08	8.02727E-08	0	2.14E-08	1.804E-07	4.44E-09	0
Methylobacterium_brachiatum	1.48818E-07	1.84455E-07	3.28545E-08	2.87727E-07	2.19769E-07	4.13846E-08	1.19154E-07	5.97385E-07	0.000000054	6.25455E-08	7.90909E-08	7.75455E-08	6.66667E-08	8.33333E-08	9.29067E-08	1.35933E-07
Methylobacterium_durans	2.26364E-07	8.18182E-08	8.25455E-09	7.09091E-08	8.79231E-08	5.69846E-07	2.38154E-07	0.000000227	1.99818E-07	2.31182E-07	3.95455E-08	9.85455E-08	5.08667E-08	1.44733E-07	1.642E-07	2.424E-07
Methylobacterium_sp._WL1	1.73818E-07	1.37409E-07	1.70091E-08	7.65182E-08	0	2.73846E-07	1.17077E-07	7.28462E-08	1.26727E-07	0.000000156	3.93273E-07	4.12182E-07	3.96E-08	0.000000014	1.12667E-08	3.84667E-08
Methylobacterium_sp._17Sr1-43	3.11818E-08	1.68182E-08	1.33636E-07	4.79091E-09	0	7.29231E-08	4.33846E-08	0	0	5.22727E-08	0	7.13636E-09	0	0	0	0
Methylobacterium_terrae	0	0	0	3.35455E-08	7.50769E-08	0	1.92308E-07	0.000000062	4.32727E-08	0.000000028	0	0	0	1.30067E-07	0	9.68E-08
Methylobacterium_nodulans	3.69727E-07	2.42818E-07	0.000000066	1.09091E-07	1.08231E-07	8.41538E-08	1.19308E-07	4.89462E-07	6.37364E-07	0.000000439	8.06909E-07	6.43091E-07	0.000000412	5.02467E-07	4.50667E-07	2.93267E-07
Candidatus_Filomicrobium_marinum	0	0	0	0	0	0	0	0	0	2.16091E-07	0	0	2.24E-08	0	0	0
Youhaiella_tibetensis	2.79455E-07	1.16364E-08	6.17091E-08	2.49909E-07	7.05385E-08	4.36154E-07	1.36077E-07	8.85385E-08	1.82636E-07	2.74455E-07	2.41091E-07	2.53455E-07	1.01067E-07	1.63933E-07	2.03067E-07	0.000000182
Rhodomicrobium_vannielii	2.98182E-08	1.16364E-08	9.44545E-08	8.22727E-08	8.86923E-08	1.14077E-07	1.48923E-07	3.22231E-07	1.02455E-07	1.72182E-07	0.00000024	1.69909E-07	6.38667E-08	8.29333E-08	3.00067E-07	0.000000105
Pelagibacterium_halotolerans	3.01636E-07	4.61818E-08	1.09636E-07	3.82273E-07	5.18231E-07	2.28077E-07	3.19923E-07	3.12846E-07	5.02545E-07	9.24273E-07	5.93727E-07	5.08545E-07	2.23733E-07	2.65933E-07	2.60133E-07	2.03067E-07
Maritalea_myrionectae	5.46636E-07	1.16364E-08	1.60182E-07	2.29364E-07	1.78231E-07	1.04462E-07	2.65846E-07	2.00538E-07	2.02182E-07	2.38364E-07	2.14818E-07	1.52909E-07	9.39333E-08	1.77933E-07	2.41773E-07	3.75867E-07
Hyphomicrobium_sp._MC1	2.98182E-08	1.16364E-08	0	9.49091E-08	2.05385E-08	3.72154E-07	0	0	2.31545E-07	2.24727E-07	3.33636E-08	0	7.79333E-08	0.000000014	2.14573E-07	3.20667E-08
Hyphomicrobium_nitrativorans	1.68245E-06	0	3.64855E-07	1.33882E-06	1.61692E-07	7.21538E-08	2.63077E-08	7.08308E-07	4.62364E-07	6.06545E-07	1.02445E-06	7.83273E-07	2.524E-07	4.35867E-07	9.106E-07	1.42467E-07
Hyphomicrobium_denitrificans	1.15127E-06	2.11364E-07	3.13945E-07	4.26273E-07	3.79308E-07	2.74738E-06	1.67769E-06	2.10538E-06	4.99273E-07	1.35827E-06	1.63618E-06	1.62427E-06	8.97467E-07	8.08867E-07	0.00000081	9.93133E-07
Devosia_sp._S02	0.00000013	6.36364E-08	2.87273E-08	1.55791E-07	4.13846E-08	3.30869E-06	1.27462E-07	7.40769E-08	2.72727E-08	0.000000028	2.66364E-07	8.29091E-08	1.14867E-07	2.09667E-07	1.22067E-07	1.87867E-07
Devosia_ginsengisoli	7.12636E-07	1.11864E-07	7.47273E-08	1.82636E-07	1.47231E-07	1.59038E-06	5.48462E-08	9.73077E-08	1.58364E-07	6.42727E-08	1.26636E-07	3.57091E-07	0	0.000000133	0.000000039	5.26667E-08
Devosia_sp._1566	2.47727E-07	2.74545E-08	9.94545E-08	0.000000413	0.000000125	0.000000286	0.000000057	1.23923E-07	9.43636E-08	2.08273E-07	2.25909E-07	0.000000138	7.24E-08	3.42E-08	4.64667E-08	6.52667E-08
Devosia_sp._MC521	4.73909E-07	1.85782E-06	0.000000258	9.12727E-08	3.34615E-08	4.13846E-08	7.92308E-08	0.000000152	2.33364E-07	2.08273E-07	1.87636E-07	8.53636E-08	0	0	0	3.37333E-08
Devosia_sp._I507	4.41182E-07	3.96727E-07	1.564E-07	5.03273E-07	0.000000241	8.97385E-07	1.41292E-06	1.14123E-06	1.00455E-06	3.20273E-07	0.00000113	7.90545E-07	1.576E-07	0.000000033	2.21533E-07	2.324E-07
Devosia_sp._A16	0.000000832	1.82773E-07	4.95364E-08	6.05818E-07	1.34462E-07	1.39692E-07	0	1.67615E-07	3.77273E-07	4.82818E-07	1.53727E-07	2.37545E-07	3.33133E-07	2.32733E-07	5.78533E-07	4.31133E-07
Devosia_sp._D6-9	2.14909E-07	0.000000033	8.31818E-09	8.75455E-08	1.99692E-07	2.26615E-07	2.50462E-07	5.72308E-08	5.46364E-08	1.54909E-07	5.10636E-07	2.92364E-07	1.386E-07	2.45533E-07	2.94933E-07	0.000000173
Blastochloris_tepida	1.26145E-06	1.94545E-07	0	3.00909E-07	0.000000254	1.41923E-07	6.39077E-07	1.62215E-06	4.30182E-07	8.31636E-07	0.000000438	1.10927E-06	5.402E-07	7.602E-07	9.28933E-07	1.07467E-06
Blastochloris_viridis	5.92727E-08	0.000000065	0	0.000000319	9.65385E-08	0	0	5.22308E-08	2.16364E-08	8.02727E-08	1.00545E-07	2.10182E-07	2.44E-08	1.79733E-07	7.45067E-08	1.00067E-07
Rhodoplanes_sp._Z2-YC6860	2.20818E-07	0.000000065	2.07309E-07	3.62545E-07	2.14462E-07	6.06154E-07	6.57231E-07	3.47692E-07	1.87818E-07	3.73182E-07	4.36273E-07	7.42909E-07	7.50667E-08	2.336E-07	3.21307E-07	2.44333E-07
Methylosinus_sp._C49	3.46727E-07	0.00000013	1.52818E-07	3.15909E-07	0	0.000000026	0	2.55846E-07	7.97273E-08	1.19364E-07	0.000000039	4.36545E-07	0	0.000000041	0	0
Methylosinus_trichosporium	1.73818E-07	0	0	0	3.58462E-08	1.79231E-08	1.77846E-07	0.000000588	2.20818E-07	6.66364E-08	1.39909E-07	7.64091E-07	9.24667E-08	1.018E-07	1.7024E-07	1.17067E-07
Methylocystis_sp._SC2	1.03818E-07	1.91818E-08	0	2.50182E-07	1.97615E-07	2.78308E-07	6.13615E-07	0.000000111	2.00636E-07	1.23909E-07	1.85455E-07	2.19091E-07	2.688E-07	2.22733E-07	1.49867E-07	4.22067E-07
Methylocystis_bryophila	1.58273E-07	0	1.36455E-07	0.000000239	6.46923E-08	0	3.33077E-08	8.26923E-08	4.34545E-07	1.10727E-07	2.94182E-07	2.54818E-07	1.26133E-07	6.44667E-08	5.77333E-08	2.212E-07
Methylocystis_heyeri	5.92727E-07	4.13182E-07	1.37218E-07	1.33409E-06	1.28154E-07	0.00000157	2.97062E-06	2.19538E-06	2.46091E-07	0.000000243	8.67091E-07	7.14364E-07	2.06667E-07	1.62267E-07	2.83467E-07	2.72067E-07
Methylocystis_parvus	2.68364E-07	2.56818E-08	0	1.39518E-06	6.93077E-08	1.80469E-06	2.26008E-06	2.01615E-06	8.05182E-07	3.98455E-07	4.95545E-07	6.23455E-07	2.72533E-07	2.834E-07	3.0104E-07	1.10467E-07
Methylocystis_rosea	1.73927E-06	6.80818E-07	4.88591E-07	8.45182E-07	5.51769E-07	3.25077E-07	1.52308E-06	5.06385E-07	9.02455E-07	8.25091E-07	7.30455E-07	1.01082E-06	2.71667E-07	6.778E-07	2.64667E-07	3.98667E-07
Terasakiella_sp._SH-1	8.57091E-07	0	2.28591E-07	7.64364E-07	6.56385E-07	0	5.26154E-08	2.87769E-07	6.57727E-07	5.19818E-07	8.00091E-07	7.13818E-07	4.88067E-07	1.63127E-06	5.71267E-07	8.73533E-07
Hoeflea_sp._IMCC20628	0	0	0	0	6.30769E-08	0	2.63077E-08	4.91538E-08	0	0.000000047	0	7.13636E-09	0	0	0	0
Hoeflea_phototrophica	6.80909E-08	0	0	0	9.52308E-08	8.27692E-08	0	5.45385E-08	2.72727E-08	0	6.71818E-08	1.14545E-07	1.634E-07	1.378E-07	1.76467E-07	4.76667E-08
Aminobacter_sp._SR38	0	0	0	0	0	0	0	0.000000011	0	0	0	0	0	0	0	0
Aminobacter_aminovorans	6.24545E-08	0	0	0	0	0	0	0	0	0	0	4.79091E-08	0	0	3.75733E-08	0
Aminobacter_sp._MSH1	5.27273E-08	0	0	0	2.59231E-08	0	0	2.46923E-08	0	2.87273E-08	0	3.74545E-08	0	0	0	0
Phyllobacterium_sp._628	1.23182E-07	0	1.13182E-07	2.43636E-08	0	2.13846E-08	0	3.81538E-08	0.000000071	0.000000081	1.26545E-07	0.000000154	0.000000122	4.76667E-08	7.27067E-08	8.18E-08
Phyllobacterium_zundukense	1.56091E-07	2.70909E-08	1.07855E-07	1.15909E-07	1.28846E-07	0	1.19231E-07	2.46462E-07	2.15727E-07	1.38636E-07	2.11091E-07	1.90909E-07	4.58E-08	4.49333E-08	3.43733E-08	9.15333E-08
Salaquimonas_pukyongi	7.72727E-08	2.34545E-08	1.63636E-08	2.43636E-08	2.53538E-07	2.33692E-07	1.31538E-07	0	0	1.75727E-07	2.41455E-07	1.72455E-07	1.594E-07	1.40133E-07	8.86667E-09	3.84667E-08
Roseitalea_porphyridii	1.45182E-07	0.000000065	8.64545E-08	2.29727E-07	1.54846E-07	0	3.77692E-08	2.11385E-07	4.01273E-07	2.03818E-07	2.58545E-07	2.33182E-07	1.446E-07	3.36067E-07	2.53667E-07	2.43333E-07
Oricola_sp._MEBiC13590	7.00909E-08	5.63636E-08	3.70909E-08	2.99455E-07	2.06308E-07	7.89231E-08	2.63077E-08	1.83385E-07	2.40545E-07	1.03727E-07	3.42545E-07	3.82455E-07	8.74667E-08	2.688E-07	0.000000246	4.73133E-07
Chelativorans_sp._BNC1	4.16091E-07	2.06182E-07	2.50182E-07	7.40636E-07	2.48615E-07	0	0	2.96692E-07	2.58727E-07	2.60909E-07	3.90909E-07	1.02418E-06	2.24867E-07	3.80333E-07	3.70267E-07	5.832E-07
Lentilitoribacter_sp._Alg239-R112	3.23273E-07	0	3.33636E-07	1.50273E-06	3.56615E-07	0	0	4.93846E-08	1.28145E-06	9.13091E-07	7.53364E-07	0.000001429	2.65867E-07	3.762E-07	5.47867E-07	3.13933E-07
Nitratireductor_sp._SY7	6.74545E-08	0.000000198	1.63636E-08	0.000000054	0	0	4.33846E-08	0	1.75182E-07	0.000000028	0.000000039	7.13636E-09	2.14E-08	3.48E-08	2.69067E-08	9.33333E-09
Nitratireductor_sp._OM-1	1.02555E-05	2.36909E-06	1.33768E-05	8.04127E-06	1.80623E-06	7.72462E-07	0.00001388	0.000011374	3.70455E-06	5.92682E-06	8.64636E-06	7.63864E-06	1.20447E-06	3.1914E-06	2.57173E-06	1.62693E-06
Mesorhizobium_sp._Pch-S	0	0	0	0	0	9.46154E-09	0	0	0	0	0	0	0	0	0	0
Mesorhizobium_sp._NZP2234	3.11818E-08	0	0	0	0	0	0	0	0	0	0	0	0	0	0	0
Mesorhizobium_sp._M1B.F.Ca.ET.045.04.1.1	0	9.54545E-09	0	0	0	0	0	0	0	0	0	4.79091E-08	0	0	0	4.87333E-08
Mesorhizobium_sp._M2A.F.Ca.ET.043.02.1.1	0	0	0	0	0.000000039	0	0	0	0	0	0	0	1.21333E-07	0	8.86667E-09	1.86E-08
Mesorhizobium_sp._NZP2298	0	0	0	0	0.000000065	2.13846E-08	3.06923E-08	0	3.75455E-08	0	0	0	0	0	8.86667E-09	0
Mesorhizobium_oceanicum	0	0	0	1.91818E-08	4.11538E-08	0	3.11538E-08	7.13077E-08	1.26727E-07	0	0	3.57273E-08	8.04E-08	7.08E-08	0	9.93333E-08
Mesorhizobium_sp._AA22	8.55455E-08	0	0	1.91818E-08	0	0	0	0.000000011	5.97273E-08	3.62727E-08	0	2.95455E-07	2.44E-08	0	0	0
Mesorhizobium_sp._NZP2077	0	0	0	1.65273E-07	0	0	0	2.97692E-08	0	0	0	0.000000035	2.08E-08	0	6.46667E-08	2.79333E-08
Mesorhizobium_sp._M7D.F.Ca.US.005.01.1.1	0	1.77273E-08	0	0	2.26923E-08	0	0	0	0	3.89091E-08	0	0	2.24E-08	0.000000041	2.99733E-08	0.000000168
Mesorhizobium_huakuii	2.56364E-08	0	0	1.93636E-08	5.63846E-08	0	8.17692E-08	7.51538E-08	1.73091E-07	6.69091E-08	0	2.14545E-08	2.14667E-08	0.000000033	1.46933E-07	8.78667E-08
Mesorhizobium_erdmanii	0	0	0	0	2.05385E-08	0	0	0	0	0	6.88182E-08	4.65909E-08	0	0	0	4.28E-08
Mesorhizobium_sp._M1E.F.Ca.ET.045.02.1.1	0	0	0	5.68182E-08	0	0.000000098	0	0	0	0	1.63273E-07	1.37909E-07	2.14E-08	1.61333E-08	4.44E-09	1.66667E-08
Mesorhizobium_sp._INR15	0	0	0	0	0	1.89231E-07	0	0	0	0	3.95455E-08	0	0	0	4.44E-09	0
Mesorhizobium_sp._N3	0	0	0	2.98091E-07	2.75385E-08	7.15231E-07	3.11538E-08	5.48462E-08	4.45455E-08	0	6.71818E-08	2.85455E-08	2.24E-08	9.92E-08	8.86667E-09	1.32067E-07
Mesorhizobium_loti	2.43273E-07	0	1.63636E-08	3.34455E-07	5.02308E-08	1.08154E-07	8.11538E-08	3.22308E-08	2.11273E-07	2.24182E-07	0.000000201	4.12455E-07	7.48E-08	1.61333E-08	1.688E-07	0.000000118
Mesorhizobium_australicum	0	0	5.74545E-08	4.88818E-08	0	0	0	0	0.000000027	0	0	0	0	0	0	0
Mesorhizobium_sp._M6A.T.Cr.TU.016.01.1.1	5.94545E-08	7.04545E-08	6.18182E-08	3.24545E-08	2.05385E-08	0	0	6.04615E-08	1.51455E-07	1.32364E-07	2.66273E-07	2.14545E-08	1.60867E-07	6.56E-08	2.55333E-08	5.24E-08
Mesorhizobium_sp._M4B.F.Ca.ET.058.02.1.1	1.58182E-08	0	0	3.24545E-08	0	0	3.33077E-08	0	0	0	0	5.30909E-08	0	0	4.44E-09	4.95333E-08
Mesorhizobium_sp._M3A.F.Ca.ET.080.04.2.1	8.39091E-08	0	0	0	0	0	8.92308E-08	0	8.43636E-08	5.72727E-08	2.73182E-07	4.79091E-08	0	0.000000042	2.09733E-08	1.86E-08
Mesorhizobium_sp._WSM1497	0	0	6.33636E-08	2.41545E-08	0	0	0	3.66923E-07	0	0	0	6.41364E-08	0	0	0	0
Mesorhizobium_sp._8	1.62455E-07	9.54545E-09	0	2.81727E-07	3.34615E-08	0	6.96923E-08	4.37692E-08	1.72182E-07	0.000000028	4.99273E-07	1.65909E-07	4.88E-08	7.42667E-08	1.25867E-07	1.04933E-07
Mesorhizobium_sp._DCY119	6.08182E-08	0	0	4.88818E-08	3.34615E-08	0	0	0	2.64545E-08	0	7.85455E-08	5.50455E-08	0	0.000000033	1.12667E-08	1.22E-08
Mesorhizobium_opportunistum	4.87273E-08	3.45455E-08	2.11936E-07	7.09364E-07	7.63077E-08	1.50692E-06	1.29723E-06	1.15723E-06	3.28364E-07	4.05273E-07	3.02818E-07	7.62455E-07	1.70133E-07	0.000000033	2.99733E-08	5.06667E-08
Mesorhizobium_japonicum	3.62909E-07	6.59636E-07	1.55945E-07	0.000000355	0	0	3.11538E-08	1.46923E-08	5.97273E-08	0	0	2.47182E-07	0	0	0	5.10667E-08
Mesorhizobium_amorphae	1.36636E-07	2.39818E-07	2.04909E-08	6.78182E-08	4.86154E-08	0	6.41538E-08	0	3.94545E-08	5.22727E-08	4.98182E-08	4.59091E-08	4.99333E-08	7.62667E-08	4.94667E-08	0.000000104
Mesorhizobium_sp._M7A.F.Ce.TU.012.03.2.1	1.27455E-07	0	8.25455E-09	2.41545E-08	0.000000065	1.49846E-07	3.33077E-08	9.75385E-08	2.72727E-08	0.000000034	0	2.65909E-07	2.14E-08	3.09333E-08	1.22333E-07	8.38E-08
Mesorhizobium_sp._NBSH29	1.70091E-07	0.000000178	2.06727E-07	2.96882E-07	0	9.46154E-09	3.77692E-08	1.46538E-07	1.02091E-07	5.22727E-08	1.79273E-07	0	2.37333E-08	3.32667E-08	1.37733E-07	0.00000009
Mesorhizobium_ciceri	3.62727E-08	0.00000013	0	3.89091E-08	2.13615E-07	7.57692E-08	0.000002678	2.31592E-06	3.07273E-07	0	0	3.03455E-07	2.16333E-07	5.438E-07	4.226E-07	3.11267E-07
Mesorhizobium_terrae	0	0	0	4.20909E-08	6.49231E-08	0	0	1.87462E-07	3.98182E-08	8.08182E-08	1.19091E-07	5.70909E-08	1.33133E-07	2.452E-07	2.602E-07	4.39667E-07
Mesorhizobium_sp._M9A.F.Ca.ET.002.03.1.2	1.25973E-06	9.80909E-07	2.114E-07	9.31818E-07	1.01338E-06	1.11254E-05	2.80338E-06	3.39846E-07	8.36727E-07	9.00909E-07	7.87091E-07	1.04545E-06	1.2742E-06	9.186E-07	1.3688E-06	1.17393E-06
Variibacter_gotjawalensis	8.13636E-08	0	0	5.71818E-08	1.62462E-07	0	6.89231E-08	2.22385E-07	1.34091E-07	0.000000028	9.31818E-08	8.26818E-08	1.31133E-07	1.30267E-07	3.37307E-07	3.97133E-07
Tardiphaga_sp._vice278	0	2.70909E-08	1.63636E-08	0	1.01923E-07	0	0	0	0.000000027	0	0	2.85455E-08	5.05333E-08	0	0	0
Tardiphaga_robiniae	8.84545E-08	1.39364E-07	0	5.14545E-08	3.58462E-08	0	0	2.47692E-08	0	0	0	3.94545E-08	7.13333E-08	0	4.44E-09	4.89333E-08

Nitrobacter_winogradskyi	0	0	0	0	3.15385E-08	2.13846E-08	0	0	0	0	0	1.42727E-08	0	0	0	0
Nitrobacter_hamburgensis	0.000000104	0	0	1.45273E-07	5.15385E-08	0	0	3.22308E-08	0.000000124	1.24545E-07	1.04545E-07	1.87545E-07	4.23333E-08	9.30667E-08	3.88667E-08	2.06467E-07
Oligotropha_carboxidovorans	3.25218E-06	4.51182E-07	2.97323E-06	0.000001815	0	4.13846E-08	0	3.20769E-07	1.11509E-06	9.93273E-07	2.22564E-06	1.31655E-06	2.64E-08	3.48E-08	1.08973E-07	1.22E-08
Bosea_sp._Tri-49	0	9.62727E-08	0	3.24545E-08	4.93846E-08	6.93846E-08	1.93846E-07	2.97692E-08	2.64545E-08	0	7.90909E-08	1.68455E-07	2.24E-08	0	1.49333E-08	6.33333E-08
Bosea_sp._PAMC_26642	2.56364E-08	0	3.70909E-08	9.54545E-09	0	1.55385E-07	0	0	0.000000089	0	9.44545E-08	1.42727E-08	0	0	2.24667E-08	0
Bosea_sp._F3-2	2.98182E-08	0	7.40909E-08	2.72091E-07	4.93846E-08	3.07923E-07	5.26154E-08	2.66231E-07	0	1.56364E-07	0.000000041	7.13636E-09	1.04933E-07	9.18667E-08	2.08867E-07	1.39867E-07
Bosea_sp._AS-1	0	0	4.12727E-09	0	1.47385E-07	6.35385E-08	0	0	0	0	0	1.50182E-07	4.38E-08	1.458E-07	2.326E-07	5.07333E-08
Bosea_sp._ANAM02	5.85091E-07	0	8.31818E-09	1.69809E-06	4.68846E-07	1.96385E-06	3.70385E-06	2.58246E-06	4.05909E-07	4.38636E-07	0.000000366	5.40364E-07	1.89067E-07	2.64267E-07	3.35067E-07	1.142E-07
Bosea_sp._RAC05	2.64236E-06	7.55545E-07	3.08591E-06	2.03473E-06	1.75462E-07	2.53615E-07	1.60315E-06	0.000002604	7.56455E-07	1.20355E-06	1.55664E-06	1.94491E-06	0.000000354	6.82667E-07	4.922E-07	3.41533E-07
Bosea_vaviloviae	0	0	7.06727E-08	2.22818E-07	0.000000147	1.04462E-07	5.54615E-08	8.79231E-08	2.16364E-08	5.22727E-08	0.000000039	1.50909E-07	0	0	2.83733E-08	5.10667E-08
Rhodopseudomonas_palustris	3.34909E-06	3.54818E-07	1.16272E-06	2.81045E-06	1.11208E-06	1.34946E-05	8.47185E-06	7.84846E-06	2.22436E-06	2.55409E-06	3.08973E-06	3.70818E-06	1.57927E-06	1.3618E-06	2.0638E-06	1.58287E-06
Bradyrhizobium_genosp._L	0	0	0	0	0	0	0	0	0	0	0	0	2.14E-08	0	0	0
Bradyrhizobium_guangdongense	5.57273E-08	0	0	0	0	0	0	0	0	0	0	0	0	0	0	0
Bradyrhizobium_sp._323S2	0	0	0	0	0	0	0	0	0	0	0	0	0	0	0	9.33333E-09
Bradyrhizobium_diazoefficiens	0	0	0	5.18182E-09	0	0	0	0	0.000000215	0.000000047	0	0	0	4.49333E-08	0	0
Bradyrhizobium_genosp._B	0	0	0	1.03636E-08	0	2.13846E-08	0	0	1.15455E-07	0	0	0	0	0	0	0
Bradyrhizobium_guangxiense	0	0	0	0	0	0	0	0	0	0	0	7.13636E-09	0	0	1.49333E-08	0
Bradyrhizobium_betae	1.52364E-07	0	0	8.53364E-08	2.05385E-08	1.82308E-07	2.63077E-08	5.22308E-08	2.63455E-07	1.27273E-07	0	0.000000105	2.14E-08	0.00000007	0	3.72E-08
Bradyrhizobium_sp._1(2017)	0	0	0	0	0	2.13846E-08	0	0	0	2.87273E-08	3.30909E-07	3.94545E-08	0	0	0	0
Bradyrhizobium_paxllaeri	5.27273E-08	0	0	1.38818E-07	0	1.55769E-07	4.33846E-08	3.22308E-08	1.19091E-07	9.24545E-08	3.93636E-07	1.32182E-07	7.67333E-08	4.96667E-08	1.12667E-08	4.89333E-08
Bradyrhizobium_ottawaense	0	0	0	0	0	0	0	0	0	0	1.02727E-08	1.42727E-08	0	0	0	0
Bradyrhizobium_sp._WSM471	0	0	0	1.93636E-08	0	0	0	0	4.45455E-08	0	1.02727E-08	0	2.44E-08	0	0	0
Bradyrhizobium_lablabi	5.27273E-08	0.000000062	0	5.49882E-07	2.88846E-07	1.30646E-06	2.03846E-07	3.37692E-08	1.60273E-07	1.27273E-07	1.73273E-07	4.38727E-07	2.02867E-07	1.97133E-07	3.50667E-07	2.03333E-07
Bradyrhizobium_sp._CCBAU_51765	2.09182E-07	2.34545E-08	0	9.89091E-08	6.15385E-08	4.27692E-08	2.63077E-08	8.19231E-08	2.75455E-08	9.24545E-08	8.75455E-08	8.26818E-08	0.000000075	0	1.352E-07	9.08E-08
Bradyrhizobium_sp._ORS_278	0.000000061	1.04545E-08	0	3.39091E-08	0	1.63077E-07	2.22308E-07	3.00769E-08	0	0	3.95455E-08	1.35091E-07	8.52667E-08	0.000000033	4.93333E-08	4.89333E-08
Bradyrhizobium_arachidis	1.08455E-07	0.00000013	1.63636E-08	1.42791E-07	0	0	1.07538E-07	7.60769E-08	0.000000027	3.62727E-08	6.88182E-08	1.44864E-07	0	1.17067E-07	1.57067E-08	1.41933E-07
Bradyrhizobium_sp._SK17	2.44727E-07	9.36364E-09	1.02636E-07	9.12727E-08	0	7.66154E-08	0	0	0	0	0	7.13636E-09	0	0	0	0
Bradyrhizobium_sp._CCBAU_53338	3.11818E-08	1.61818E-07	2.47273E-08	0.000000066	0	0	0	0	2.64545E-08	2.85455E-08	1.30909E-07	7.44545E-08	2.14E-08	0	4.82E-08	0
Bradyrhizobium_canariense	0.000000286	0.000000065	3.70909E-08	0.000000203	3.58462E-08	4.48923E-07	0	0	0.000000099	0.000000028	0	1.42727E-08	0	0	1.12667E-08	9.33333E-09
Bradyrhizobium_sp._CCBAU_53340	9.70909E-08	0	0	2.91545E-08	4.46154E-08	7.96923E-08	0	3.37692E-08	5.79091E-08	0	0	0	5.09333E-08	1.05467E-07	6.23333E-08	8.42E-08
Bradyrhizobium_sp._CCBAU_51753	6.84545E-08	9.54545E-09	0	1.03636E-08	0	0.000000026	0	0	0	0.000000116	2.05455E-08	2.14545E-08	0	0	7.44E-08	9.33333E-09
Bradyrhizobium_sp._ORS_285	0	0	0	2.41545E-08	3.34615E-08	0	0	0	8.91818E-08	0.000000028	0.000000227	1.29273E-07	4.78E-08	0.000000067	1.29933E-07	8.74E-08
Bradyrhizobium_symbiodeficiens	0	0	0	2.03364E-08	0	2.63615E-07	0	0	5.48182E-08	6.69091E-08	7.12727E-08	3.20909E-07	6.08E-08	3.09333E-08	1.49333E-08	0
Bradyrhizobium_sp._CCBAU_051011	2.55182E-07	1.05273E-07	3.70909E-08	1.36818E-07	0	2.13846E-08	2.11615E-07	4.39231E-08	2.09727E-07	7.29091E-08	1.39727E-07	1.62727E-07	1.86667E-08	0	4.44E-09	0
Bradyrhizobium_amphicarpaeae	1.33182E-07	0	0	4.42727E-08	1.57462E-07	0	1.17615E-07	4.73462E-07	5.34545E-08	0	0.000000039	3.95455E-07	0.000000027	4.02E-08	2.134E-07	1.48133E-07
Bradyrhizobium_icense	1.90273E-07	1.78273E-07	0	2.83818E-07	1.45154E-07	1.08715E-06	1.60462E-07	8.29231E-08	7.46364E-08	9.30909E-08	3.94182E-07	0.000000223	6.73333E-08	3.50667E-08	1.55933E-07	7.54667E-08
Bradyrhizobium_guangzhouense	7.43455E-07	0	0	1.61182E-07	1.12538E-07	0	3.71462E-07	1.24369E-06	4.65091E-07	0.000000464	5.58545E-07	0.000000431	4.85933E-07	2.576E-07	0.00000033	3.12467E-07
Bradyrhizobium_sp._BTAi1	2.78364E-07	2.34545E-08	1.27273E-07	1.84818E-07	1.35462E-07	7.09231E-08	1.75385E-07	4.16308E-07	1.33273E-07	1.32545E-07	7.69091E-08	1.24818E-07	1.602E-07	2.44667E-07	5.33533E-07	0.000000763
Bradyrhizobium_sp._6(2017)	6.46909E-07	5.27727E-08	3.85909E-07	6.42636E-07	3.11462E-07	1.09123E-06	3.02154E-07	7.53846E-07	4.87636E-07	5.60818E-07	3.44636E-07	6.61545E-07	4.756E-07	2.79333E-07	2.38667E-07	2.442E-07
Bradyrhizobium_erythrophlei	3.28182E-07	3.46682E-07	1.48391E-07	8.47545E-07	1.13223E-06	2.98077E-07	3.50692E-07	5.96692E-07	4.81364E-07	0.000000396	6.73727E-07	8.17273E-07	1.856E-07	3.88533E-07	4.642E-07	3.35067E-07
Bradyrhizobium_oligotrophicum	7.39273E-07	3.25545E-07	1.04664E-07	2.83636E-07	9.43846E-08	8.71538E-08	3.78538E-07	1.90769E-08	5.02727E-07	4.98273E-07	4.07273E-07	8.09364E-07	4.51333E-08	0.000000128	0.000000039	3.87133E-07
Bradyrhizobium_japonicum	6.80909E-08	0	2.59818E-08	7.07273E-08	0.000000039	0	0	3.42846E-07	0	1.45636E-07	6.53636E-08	3.25364E-07	0.000000098	0.000000075	0.000000399	1.52333E-07
Bradyrhizobium_vignae	0	0	0	4.88182E-08	3.58462E-08	0	3.06923E-08	3.22308E-08	0	0	0	7.13636E-09	0	6.98667E-08	4.44E-09	0
Bradyrhizobium_zhanjiangense	2.20182E-07	0	0	2.95455E-08	9.73077E-08	0	4.33846E-08	4.42769E-07	2.14818E-07	0.000000276	3.00182E-07	0.000000152	1.974E-07	1.75733E-07	2.7304E-07	0.000000327
Bradyrhizobium_cosmicum	0.000000415	0.000000095	4.10909E-08	1.48818E-07	5.98077E-07	9.46154E-09	1.06615E-07	8.51538E-08	5.07455E-07	0.00000035	1.01191E-06	4.59727E-07	3.878E-07	2.91267E-07	3.94533E-07	2.69933E-07
Bradyrhizobium_sp._CCBAU_53421	8.37091E-07	7.45455E-08	1.10909E-07	3.86364E-07	3.71538E-07	3.15385E-08	2.30231E-07	1.75308E-06	4.03182E-07	4.74455E-07	7.62636E-07	4.64273E-07	6.13667E-07	4.05067E-07	2.82133E-07	3.174E-07
Bradyrhizobium_sp._CCGE-LA001	0	0	0	0	0.000000065	4.27692E-08	0	6.64615E-08	0	2.87273E-08	0	0	0	3.48E-08	2.55333E-08	4.28E-08
Bartonella_sp._1-1C	0	0	0	0	0	0	0	0	0	0	0	0	5.44E-08	0	0	0
Bartonella_ancashensis	0	0	0	0	0	8.30769E-08	0	0	0	0	0	0	0.000000027	0	0	0
Bartonella_bovis	0	0	0	0	3.58462E-08	0	0	0	0	0	0	0	0	0	0	0
Bartonella_schoenbuchensis	0	0	0	0	0	3.58923E-07	0	0	0	0	0	0	0	0	0	3.40667E-08
Bartonella_clarridgeiae	0	6.97273E-08	0	2.43636E-07	0	0	0	0	0	0	0	7.84545E-08	0	0	0	9.33333E-09
Bartonella_elizabethae	0	0	0	3.07273E-08	0	0	0	0	0	0	0	0	0	0	0	0
Bartonella_grahamii	0	0	0	0	0	0	0	0	0	0	0	0	0	0	2.99333E-08	0
Bartonella_quintana	0	0	0	0	1.19769E-07	0	0	1.54308E-07	1.33636E-07	0.000000034	1.02727E-08	8.47273E-08	9.82667E-08	1.12533E-07	1.69333E-07	1.472E-07
Bartonella_vinsonii	1.56909E-07	1.68182E-08	0	3.40909E-08	0	0	0	0	1.38909E-07	1.33727E-07	0.000000048	7.13636E-09	0	0	0	0
Bartonella_tribocorum	3.11818E-08	0	0	0	0	0	0	0	0	0	3.33636E-08	4.45909E-08	0	0	0	0
Bartonella_kosoyi	1.19145E-06	0.00000013	3.70909E-07	8.81818E-08	4.11615E-07	0	3.68462E-07	1.42769E-07	3.75455E-08	8.39091E-08	7.69091E-08	6.775E-07	0	0.000000033	0	0
Bartonella_birtlesii	0.000000089	0	0	3.55182E-08	1.03615E-07	8.70769E-08	2.05231E-07	2.00923E-07	0.000000118	8.85455E-08	2.41455E-07	2.96364E-07	1.258E-07	6.24667E-08	2.34373E-07	8.32667E-08
Bartonella_krasnovii	2.57909E-07	0	1.16436E-07	1.32091E-07	0	0	0	0	5.00091E-07	0.000000081	1.97727E-07	5.89091E-08	0	0	7.43333E-08	0
Bartonella_australis	2.27909E-07	0	4.54091E-08	1.03636E-08	1.86462E-07	0	1.07538E-07	0	6.14545E-08	1.20818E-07	1.02727E-08	1.36455E-07	1.18667E-07	1.00867E-07	1.15573E-07	5.58667E-08
Bartonella_henselae	0.000000662	7.66364E-08	0.000000277	0.000000294	1.45085E-06	0	2.06923E-07	8.59231E-08	4.84909E-07	1.32818E-07	6.65455E-08	3.39136E-07	8.23067E-07	5.306E-07	1.29827E-06	8.15333E-07
Bartonella_bacilliformis	1.15273E-07	4.39091E-08	7.40909E-08	4.79091E-09	6.33538E-07	4.31538E-08	0	0	0	0.000000056	1.98182E-07	0.0000001	4.558E-07	2.62133E-07	7.0384E-07	2.354E-07
Bartonella_apis	0.000001272	9.80182E-07	3.99545E-07	1.77391E-06	8.32154E-07	1.42323E-06	1.48792E-06	8.85923E-07	1.06255E-06	1.16445E-06	9.34091E-07	9.79273E-07	4.42267E-07	1.30893E-06	7.798E-07	9.90267E-07
Bartonella_sp._HY038	2.40727E-07	0.00000026	7.91818E-08	4.24091E-07	2.97846E-07	0	2.94615E-07	2.02077E-07	3.21545E-07	3.41727E-07	2.49273E-07	4.28182E-08	7.46E-08	7.904E-07	5.40333E-07	2.35133E-07
Georhizobium_profundi	3.11818E-08	0	0	1.32973E-07	3.58462E-08	0	4.33846E-08	0	6.49091E-08	0	1.02727E-08	7.55455E-08	0	0	8.86667E-09	0
Ciceribacter_thiooxidans	7.86091E-07	0	0	3.24545E-08	1.29862E-06	0	0	0	0	9.36364E-08	1.06727E-07	1.58182E-07	1.25613E-06	1.16973E-06	1.81267E-06	1.4154E-06
Shinella_sp._HZN7	2.96364E-08	1.68182E-08	0	3.99091E-08	1.26769E-07	1.16308E-07	7.54615E-08	1.71385E-07	5.91818E-08	0	2.76364E-08	2.27091E-07	1.95133E-07	1.84267E-07	2.2224E-07	0.000000131
Candidatus_Liberibacter_solanacearum	1.38727E-07	0	0	0	0	0	0	8.62308E-08	2.72727E-08	0	0	0	2.64667E-08	0	4.38667E-08	2.702E-07
Candidatus_Liberibacter_americanus	0	0	1.63636E-08	4.79091E-09	0	0	0	2.46923E-08	4.45455E-08	0	0	1.42727E-08	0	0	0	0
Candidatus_Liberibacter_africanus	1.58182E-08	0	0	6.46364E-08	2.05385E-08	0	0.000000057	1.18923E-07	2.64545E-08	1.34909E-07	3.33636E-08	1.09727E-07	2.44E-08	0.000000014	2.89307E-07	1.22E-08
Liberibacter_crescens	0	0	0	0	0	0	0	0	5.97273E-08	0.000000065	0.000000132	4.65909E-08	2.64E-08	0	1.49333E-08	0
Candidatus_Liberibacter_asiaticus	0	1.87273E-08	2.04909E-08	0	0	0	0	2.97692E-08	1.26545E-07	0	1.00182E-07	1.49818E-07	0.000000159	8.18E-08	8.94E-08	0.000000102
Ensifer_mexicanus	2.30909E-08	0	0	0	0	0	0	6.41538E-08	0	0.000000034	2.06364E-07	0	0	1.58E-08	1.12667E-08	1.86667E-08
Ensifer_alkalisoli	2.26909E-07	3.28182E-08	1.63636E-08	1.61545E-07	1.05154E-07	0.000000026	4.14308E-07	6.23077E-08	2.47545E-07	2.41636E-07	5.42636E-07	5.56455E-07	2.24E-08	3.99333E-08	0.000000202	6.33333E-08
Ensifer_sojae	0	0	1.63636E-08	3.07273E-08	3.15385E-08	5.35615E-07	0	3.57692E-08	4.91818E-08	0	2.30818E-07	2.14545E-08	8.05333E-08	1.71333E-08	1.12667E-08	0
Ensifer_adhaerens	1.93182E-06	4.17273E-07	2.60036E-07	1.72291E-06	8.09692E-07	5.55077E-07	5.68077E-07	8.40615E-07	0.000002399	3.10891E-06	0.000001467	2.52345E-06	0.000000626	1.10433E-06	4.88933E-07	1.5666E-06
Sinorhizobium_americanum	8.56364E-08	0.000000065	0	3.85455E-08	0	0	0.00000008	0	5.50909E-08	1.04545E-07	2.76364E-08	1.06273E-07	0	0	4.44E-09	1.39067E-07
Sinorhizobium_sp._RAC02	5.33455E-07	0	1.93709E-07	1.68818E-07	0	1.55308E-07	1.35923E-07	5.77538E-07	2.38545E-07	3.32909E-07	4.87909E-07	0.00000039	2.14E-08	4.55333E-08	1.2724E-07	1.79933E-07
Sinorhizobium_medicae	2.20364E-07	1.02455E-07	5.77273E-08	3.32818E-07	5.76154E-08	6.41538E-08	0	1.40846E-07	0	1.58455E-07	0.000000061	6.02273E-07	2.14E-08	0.000000014	6.46667E-08	5.10667E-08
Sinorhizobium_fredii	0.000002304	7.46636E-07	1.43136E-06	1.78336E-06	1.17208E-06	2.71154E-07	3.55838E-06	4.97254E-06	1.68491E-06	1.89773E-06	2.69364E-06	2.18309E-06	1.1148E-06	1.5284E-06	1.2052E-06	1.14207E-06
Sinorhizobium_meliloti	4.74273E-07	2.89364E-07	4.79273E-07	4.32182E-07	6.34231E-07	6.53077E-08	7.66923E-08	0.000000228	1.00709E-06	7.26545E-07	4.18182E-07	1.28155E-06	4.82733E-07	1.0954E-06	6.03133E-07	8.90667E-07
Neorhizobium_sp._NCHU2750	1.40091E-07	3.825E-07	0.000000605	2.81091E-07	1.19231E-07	1.35385E-07	7.28154E-07	9.19769E-07	2.51273E-07	8.59091E-08	2.44091E-07	1.80091E-07	4.72667E-08	1.33133E-07	0.000000159	2.636E-07
Neorhizobium_galegae	2.80818E-07	0.000000065	4.12727E-09	3.31091E-07	3.15385E-08	1.05538E-07	0.000000331	0.000000099	1.96273E-07	0.000000047	3.25455E-07	1.12818E-07	4.30667E-08	0	9.87733E-08	4.34E-08
Neorhizobium_sp._SOG26	1.03209E-06	2.70909E-08	4.12727E-09	0	2.92769E-07	1.73692E-07	0	9.03846E-08	0.00000016	5.39818E-07	1.97727E-07	4.86818E-07	7.57733E-07	5.54467E-07	7.48333E-07	8.39333E-07
Agrobacterium_sp._H13-3	0	0	0	0	0	0	0	0	0.000000054	0	0	0	0	0	0	0
Agrobacterium_sp._CGMCC_11546	0	2.34545E-08	0	0	0	0	0	0	0	0	0	0	0	0	0	0
Agrobacterium_sp._33MFTa1.1	2.70909E-08	0	0	0	0	0	0	0	0	0	0	3.94545E-08	0	0	0	0
Agrobacterium_sp._MA01	0	9.54545E-09	0	0	0	0	0	0	0	0	6.88182E-08	0	0	0	0	0
Agrobacterium_fabrum	0	0	0	0	0	0	1.36538E-07	0	0	0	3.95455E-08	1.42727E-08	0	0.000000014	0	0
Agrobacterium_sp._RAC06	1.83182E-07	0	3.70909E-08	2.07273E-08	0	0	0	0	0	0	0	3.68045E-07	0	3.96E-08	0	0
Agrobacterium_larrymoorei	0	4.48182E-08	0	8.53364E-08	2.88462E-08	9.16154E-07	1.36846E-07	0	0	0.000000034	9.05455E-08	0	1.48733E-07	1.58E-08	9.37333E-08	1.90533E-07
Agrobacterium_rhizogenes	5.57273E-08	2.70909E-08	3.70909E-08	3.72455E-08	3.15385E-08	2.13462E-07	0.000000191	9.54615E-08	0	3.03636E-08	1.72636E-07	1.79091E-07	5.58667E-08	0	6.17333E-08	1.17267E-07
Agrobacterium_sp._T29	6.74545E-08	0.000000047	4.53455E-08	4.32727E-08	0	1.57077E-07	5.96154E-08	2.70154E-07	7.05455E-08	1.04545E-07	1.38091E-07	7.64545E-08	4.38667E-08	9.68667E-08	1.12667E-08	0
Agrobacterium_vitis	1.49827E-06	1.35091E-07	2.75636E-07	1.15764E-06	5.79923E-07	0.000000543	1.00538E-06	1.56123E-06	1.29855E-06	1.56427E-06	1.16282E-06	1.76727E-06	0.000000745	1.07073E-06	7.12333E-07	9.486E-07
Agrobacterium_tumefaciens	4.85727E-07	4.36909E-07	8.28527E-07	1.74691E-06	8.08385E-07	8.53769E-07	1.65262E-06	2.10092E-06	7.15273E-07	9.11636E-07	1.61282E-06	1.87782E-06	6.61467E-07	7.02933E-07	0.000001325	7.17533E-07
Rhizobium_sp._NIBRBAC000502774	0	0	0	0	0	1.23846E-07	0	0	0	0	0	0	0	0	0	0
Rhizobium_sp._Y9	0	0	0	0	1.86923E-08	0	0	0	0	0	0	0	0	0	0	0
Rhizobium_acidisoli	0	0	0	0	0	0	0	0	0	3.89091E-08	0	0	0	0	0	4.95333E-08
Rhizobium_sp._S41	0	0	0	5.18182E-09	0	0	0	0	0	0	0	0	0	0	0	0
Rhizobium_selenitireducens	0	0	0	0	0	0	0	0	0	0	0	0	0	0	5.94E-08	0
Rhizobium_favelukesii	0	0.000000065	0	1.52364E-07	0	4.13846E-08	2.63077E-08	0.000000022	1.36364E-07	0	0	7.13636E-09	0	0	1.702E-07	0
Rhizobium_sp._CIAT894	0	0.000000065	0	6.79727E-08	0	0	0	0	5.79091E-08	0	1.33636E-07	2.98091E-07	0	0	4.38667E-08	1.86E-08
Rhizobium_indicum	0.000000114	0	0	0	2.05385E-08	0	3.11538E-08	3.22308E-08	0	0	0	0	0	0	0	0
Rhizobium_sp._007	3.69091E-08	0	0	4.79091E-09	3.73846E-08	0	2.63077E-08	1.90769E-08	0	2.77727E-07	2.02455E-07	4.64273E-07	6.77333E-08	0	3.66667E-08	1.61933E-07
Rhizobium_sp._Khangiran2	0	1.68182E-08	0	0	0	2.13846E-08	0	0	0	0	2.06364E-07	7.13636E-09	0	0	0	6.33333E-08
Rhizobium_gallicum	6.23636E-08	0	0	0	0	0	0	0	0	0	7.90909E-08	0	0	0	0	0
Rhizobium_sp._NXC14	0	0	0	4.79091E-09	0	2.95385E-08	0	0	0	0	1.51455E-07	7.13636E-09	0	0	5.94E-08	2.79333E-08
Rhizobium_pseudoryzae	3.69091E-08	2.34545E-08	3.70909E-08	3.07273E-08	7.69231E-08	1.35385E-07	1.16615E-07	0.000000011	3.75455E-08	6.37273E-08	3.95455E-08	1.42727E-08	8.60667E-08	1.58E-08	7.59333E-08	9.33333E-09
Rhizobium_tropici	1.19273E-07	9.54545E-09	3.27273E-08	3.18818E-07	6.76923E-08	7.09231E-08	6.51538E-08	2.08154E-07	1.42909E-07	0	1.73909E-07	0.000000187	2.08E-08	0.000000014	7.09733E-08	4.97333E-08
Rhizobium_oryzihabitans	1.58182E-08	2.26182E-07	0	2.50909E-08	5.20769E-08	1.01385E-07	7.07692E-08	4.64615E-08	3.75455E-08	3.89091E-08	7.72727E-08	0	0	0	5.264E-08	6.33333E-08
Rhizobium_sp._IE4771	7.85455E-08	0.000000065	0	0	0	0	0	4.93846E-08	0	5.22727E-08	0	7.12727E-08	0	0	4.44E-09	0
Rhizobium_etli	0.000000158	4.33091E-07	2.44091E-07	4.77455E-07	4.80769E-08	9.48462E-08	1.74308E-07	0.000000121	2.70182E-07	2.52818E-07	1.50364E-07	4.78182E-07	6.26667E-08	1.36733E-07	1.3064E-07	1.216E-07
Rhizobium_daejeonense	7.03545E-07	4.04545E-07	2.51818E-07	4.83636E-07	3.15385E-08	5.29231E-08	5.26154E-08	3.28462E-07	4.17545E-07	5.62273E-07	1.70818E-07	3.09818E-07	2.37333E-08	5.12E-08	1.886E-07	9.33333E-09
Rhizobium_sp._ADMK78	0.000000273	0.000000373	0	1.96273E-07	1.80769E-07	0	4.80769E-08	1.47308E-07	2.23636E-07	2.66727E-07	1.84818E-07	1.32636E-07	0.000000129	0.000000033	1.958E-07	2.36533E-07
Rhizobium_phaseoli	4.87273E-08	0	0	5.36091E-08	0	0	1.18077E-07	0.000000125	2.94545E-07	3.89091E-08	1.94455E-07	1.63182E-07	0	0.000000028	4.33067E-08	1.38733E-07
Rhizobium_sp._Kim5	0	1.68182E-08	0	0	0	0	0	0	0	0	0.000000039	0.000000035	0	5.08667E-08	0	0
Rhizobium_flavum	5.01818E-08	0	8.75455E-09	0	6.73846E-08	3.15385E-08	2.63077E-08	0	4.89091E-08	6.37273E-08	0	3.57636E-07	4.48667E-08	0	0	1.98667E-08
Rhizobium_pusense	0.00000007	5.94091E-08	2.52727E-07	1.58273E-07	7.47692E-08	1.89231E-07	4.06846E-07	1.54308E-07	0.000000064	0	8.59091E-08	4.14182E-07	0.000000129	3.20933E-07	3.37133E-07	1.562E-07
Rhizobium_rhizoryzae	3.69091E-08	0.000000065	4.72727E-07	9.54545E-09	2.26923E-08	4.23846E-07	1.28077E-07	8.94615E-08	8.72727E-08	2.87273E-08	8.02727E-08	2.14545E-08	1.02067E-07	5.22E-08	1.1084E-07	0.00000008
Rhizobium_hidalgonense	0.000000129	1.48364E-07	9.62182E-08	4.79091E-09	0	0	2.63077E-08	3.57692E-08	4.32727E-08	1.04545E-07	7.72727E-08	2.14545E-07	0	0	0	0
Rhizobium_sp._ACO-34A	4.77636E-07	1.16364E-08	1.3784E-06	2.09455E-07	0	1.79231E-08	1.15746E-06	8.68615E-07	1.04727E-07	7.51818E-08	2.86182E-07	4.43273E-07	1.79267E-07	3.09933E-07	3.33333E-07	5.482E-07
Rhizobium_sp._NXC24	0	0	2.89091E-08	0.000000235	1.21308E-07	0	9.07692E-08	6.45385E-08	0	7.25455E-08	0	0.00000019	9.84667E-08	5.22E-08	1.35067E-07	1.98267E-07
Rhizobium_sp._WL3	2.30727E-07	0	0.000000739	1.28245E-06	9.13846E-08	2.13846E-08	1.37692E-06	1.90554E-06	3.09455E-07	1.67455E-07	2.22273E-07	2.33091E-07	1.866E-07	9.43333E-08	2.27333E-07	9.66667E-08
Rhizobium_sp._11515TR	0.000000092	1.09773E-07	1.93891E-07	4.99091E-08	0	0	0	1.29462E-07	0	0	1.02727E-08	7.13636E-09	0	1.61333E-08	0	0
Rhizobium_lusitanum	2.38364E-07	0	0	7.00455E-07	4.62308E-08	0.00000102	1.72846E-06	1.33085E-06	4.42273E-07	2.14727E-07	3.83273E-07	5.15273E-07	7.20667E-08	3.48E-08	1.57067E-08	3.72E-08
Rhizobium_grahamii	3.02364E-06	5.84818E-07	3.31845E-06	2.16055E-06	5.02077E-07	1.74846E-07	3.46846E-06	3.29577E-06	8.07364E-07	1.59836E-06	2.11382E-06	1.73973E-06	4.12867E-07	0.000000615	3.63067E-07	8.55267E-07
Rhizobium_leguminosarum	8.85909E-07	6.71545E-07	0.000000367	1.28045E-06	6.31308E-07	1.63385E-07	8.09077E-07	1.14869E-06	6.13091E-07	5.93727E-07	9.23273E-07	1.54136E-06	1.1846E-06	1.35087E-06	1.5376E-06	1.7876E-06
Rhizobium_jaguaris	9.27091E-07	2.95909E-08	8.25591E-07	1.15982E-06	1.18438E-06	4.27692E-08	1.02892E-06	2.48231E-06	3.51909E-07	1.75182E-07	3.30182E-07	4.33273E-07	8.53733E-07	5.10067E-07	1.36373E-06	6.38667E-07
Bathymodiolus_thermophilus_thioautotrophic_gill_symbi	0	0	0	0	0	0	0	0	0	0	0	9.03636E-08	0	0	0	0
endosymbiont_of_Plagiopylea_sp.	2.56364E-08	0	0	4.40909E-08	2.59231E-08	0	0	0	3.94545E-08	0	0	0	0	2.62E-08	0	0
Candidatus_Vesicomyosocius_okutanii	0	0	0	0	0	0	1.17769E-07	0	0	3.62727E-08	3.77273E-08	3.16364E-08	0	0	0	4.34E-08
Bathymodiolus_septemdierum_thioautotrophic_gill_symb	9.22727E-08	0	7.40909E-08	9.54545E-09	5.63846E-08	0	0	0	8.04545E-08	2.28545E-07	2.27455E-07	7.82273E-08	6.08E-08	3.50667E-08	2.54E-08	0
Salinisphaera_sp._LB1	3.71091E-07	0	3.70909E-08	4.24273E-08	2.15538E-07	7.43846E-07	2.28308E-07	4.10923E-07	1.71364E-07	3.95182E-07	3.95636E-07	4.52091E-07	3.16267E-07	1.51667E-07	3.86067E-07	4.972E-07
Pseudohongiella_spirulinae	1.12182E-07	3.94091E-08	2.58273E-07	8.68182E-08	0	0	1.01923E-07	9.31538E-08	5.37273E-08	2.85455E-08	1.99818E-07	7.13636E-09	4.51333E-08	0	4.44E-09	3.40667E-08
endosymbiont_of_unidentified_scaly_snail_isolate_Monj	4.34909E-07	9.85455E-08	1.09636E-07	3.50182E-07	3.61231E-07	1.97385E-07	0.000000206	3.54154E-07	5.17091E-07	2.60182E-07	6.16364E-07	4.42727E-07	1.628E-07	3.67933E-07	5.32507E-07	3.16067E-07
Immundisolibacter_cernigliae	4.78818E-07	2.29636E-07	9.13455E-08	3.92364E-07	0.000000287	4.13846E-08	4.54692E-07	3.16077E-07	4.61364E-07	6.88545E-07	1.00509E-06	6.93727E-07	1.73533E-07	4.01533E-07	0.000000429	2.428E-07
endosymbiont_of_Pachyrhynchus_infernalis	0	0	0	6.14545E-08	0	0	0	4.96846E-07	0	8.39091E-08	0	7.07273E-08	7.47533E-07	6.02667E-07	0.000000432	2.20533E-07

Candidatus_Nardonella_dryophthoridicola	0	0	0	0	0	0	0	0	0	0	0	1.42727E-08	0	0.000000033	2.62E-08	5.39333E-08
endosymbiont_of_Sipalinus_gigas	2.96364E-08	0	8.31818E-09	4.02727E-08	0	0	9.38462E-08	1.33615E-07	7.56364E-08	0.000000081	5.73727E-07	2.835E-07	4.81333E-08	0	5.38667E-08	6.62E-08
Sinimarinibacterium_sp._NLF-5-8	5.92727E-08	0	5.74545E-08	8.55455E-08	2.88462E-08	0	0	6.45385E-08	6.50909E-08	0	4.99909E-07	0.000000085	1.14733E-07	3.09333E-08	2.62E-08	0
Solimonas_sp._K1W22B-7	1.91727E-07	0	1.20364E-07	6.31818E-08	1.35538E-07	4.81308E-07	2.56846E-07	4.39462E-07	0.000000187	3.42909E-07	2.70545E-07	6.75727E-07	4.58E-08	9.32E-08	3.18773E-07	2.974E-07
Steroidobacter_denitrificans	1.05273E-07	3.42864E-07	1.25218E-07	2.75727E-07	1.81231E-07	2.94231E-07	1.98231E-07	2.73154E-07	1.68727E-07	1.52818E-07	3.64818E-07	5.98818E-07	0	9.76E-08	1.46733E-07	3.67333E-08
Dichelobacter_nodosus	4.60909E-08	0	3.70909E-08	0	1.88462E-07	5.12308E-08	0	3.22308E-08	4.45455E-08	0	6.66364E-08	3.57273E-08	5.28667E-08	7.63333E-08	1.806E-07	1.766E-07
Cardiobacterium_hominis	1.61091E-07	2.12182E-07	4.12727E-09	2.28364E-07	2.73692E-07	1.07369E-06	1.26385E-07	3.83538E-07	2.26455E-07	2.20818E-07	6.06091E-07	5.39636E-07	7.73333E-08	9.76E-08	2.52467E-07	0.000000292
Sulfurifustis_variabilis	9.40909E-08	1.68182E-08	0	3.06636E-07	1.04231E-07	1.08092E-06	1.49469E-06	1.67592E-06	3.03182E-07	1.22545E-07	4.56364E-07	5.74909E-07	5.44E-08	0.000000014	3.52E-08	1.80733E-07
Sulfuricaulis_limicola	1.96509E-06	4.34455E-07	5.72636E-07	1.49391E-06	2.34077E-07	2.52308E-08	6.83538E-07	1.00885E-06	6.08091E-07	9.30091E-07	6.98455E-07	1.14455E-06	1.69333E-07	4.31867E-07	2.31933E-07	0.000000575
Acidiferrobacter_sp._SPIII_3	1.96636E-07	0.000000065	1.63636E-08	2.78818E-07	8.17692E-08	1.20615E-07	3.96154E-07	1.56462E-07	6.14182E-07	3.42636E-07	2.82273E-07	4.57636E-07	1.414E-07	1.45733E-07	1.87667E-07	5.76333E-07
Orbus_sp._IPMB12	0	0	0	3.07273E-08	0.000000039	0	0	0	0	0	1.30909E-07	7.12727E-08	1.08667E-07	6.66E-08	1.39067E-07	0.000000115
Gilliamella_apicola	3.11818E-08	0	4.12182E-08	8.70909E-08	6.49231E-08	1.89231E-08	2.88308E-07	5.85385E-08	8.19091E-08	1.68636E-07	7.90909E-08	8.42727E-08	0.000000166	6.18E-08	1.2864E-07	2.518E-07
Frischella_perrara	2.73909E-07	4.64455E-07	1.22273E-07	5.01182E-07	3.72308E-07	0	3.42538E-07	3.27846E-07	2.68455E-07	2.15273E-07	1.94273E-07	1.20909E-07	2.386E-07	7.288E-07	0.000000219	7.13267E-07
Candidatus_Reidiella_endopervernicosa	3.35245E-06	6.62909E-07	3.36755E-06	2.41327E-06	2.20077E-07	2.95769E-07	3.17846E-06	3.18554E-06	1.11464E-06	0.000001375	3.13382E-06	1.58364E-06	2.23467E-07	7.90867E-07	0.000000644	6.19667E-07
Sedimenticola_thiotaurini	1.94818E-06	2.84545E-08	7.764E-07	1.17673E-06	2.44154E-07	1.56077E-06	5.34308E-07	0.000000548	1.45927E-06	7.54364E-07	1.10127E-06	1.70364E-06	3.75133E-07	3.824E-07	5.206E-07	6.276E-07
Candidatus_Ruthia_magnifica	1.08818E-07	0	0	9.97273E-09	8.83846E-08	4.72846E-07	1.02923E-07	3.98462E-07	5.45455E-08	1.94636E-07	0.000000087	3.16364E-08	1.156E-07	1.31467E-07	0.000000098	0.000000332
Candidatus_Ruthia_endofausta	0	0	0	0	6.70769E-08	0	0	2.51538E-07	2.39273E-07	0.000000068	0	1.10091E-07	6.92667E-08	9.66E-08	1.49333E-08	7.33333E-08
Thiolapillus_brandeum	4.14091E-07	6.73636E-08	1.83436E-07	5.36455E-07	0.000000216	1.36385E-07	0.000000189	2.84154E-07	1.57727E-07	3.06182E-07	5.30364E-07	8.10455E-07	3.39133E-07	2.196E-07	4.23933E-07	3.92267E-07
Candidatus_Thioglobus_singularis	0	0	0	5.18182E-09	2.58308E-07	3.15385E-08	5.26154E-08	0	3.98182E-08	0.000000028	0	4.79727E-07	0	0	0	3.40667E-08
Candidatus_Thioglobus_autotrophicus	0	0	2.04909E-08	0	0	0	0	8.38462E-08	1.19091E-07	5.22727E-08	3.58273E-07	1.00364E-07	4.50667E-08	6.80667E-08	5.98667E-08	8.36E-08
Candidatus_Thioglobus_sp._NP1	6.19091E-08	0	0	1.43636E-08	1.59615E-07	0	0	3.49077E-07	1.48818E-07	5.22727E-08	0.000000093	0.00000005	8.72E-08	3.624E-07	6.09467E-07	1.96733E-07
Gallaecimonas_mangrovi	1.28464E-05	5.72545E-07	4.92909E-06	1.25855E-05	2.69731E-06	3.46923E-08	1.39385E-05	9.43538E-06	5.35909E-06	1.05107E-05	5.67545E-06	6.07555E-06	2.56853E-06	3.70647E-06	2.50367E-06	2.50007E-06
Methylocaldum_marinum	1.53418E-06	2.03909E-07	1.11909E-06	6.77364E-07	3.81462E-07	1.13769E-05	2.83085E-06	3.76538E-06	1.15036E-06	9.73182E-07	1.25373E-06	1.04682E-06	7.46667E-07	6.016E-07	8.32067E-07	1.08047E-06
Methylovulum_psychrotolerans	4.52455E-07	0	1.21982E-07	5.56273E-07	0.000000242	2.52308E-08	4.61308E-07	1.92154E-07	2.19727E-07	2.47273E-07	5.16273E-07	7.93091E-07	3.538E-07	2.284E-07	1.14267E-07	2.90267E-07
Methylomicrobium_buryatense	0.000000088	0	0	4.79091E-09	0	0	6.13846E-08	0	0	0	6.88182E-08	0.000000035	2.72E-08	0	0	0
Methylomicrobium_album	3.76091E-06	0.00000057	1.2678E-06	4.12564E-06	4.05077E-07	4.86462E-06	1.78131E-06	0.000000793	8.36364E-07	6.46364E-07	1.13291E-06	1.96036E-06	9.932E-07	4.13733E-07	7.71467E-07	7.78267E-07
Methylomicrobium_alcaliphilum	1.25909E-07	0	4.18182E-07	3.46818E-07	1.05231E-07	7.21538E-08	8.43846E-07	1.37977E-06	3.69636E-07	0	2.74273E-07	0.000000163	1.25733E-07	4.49333E-08	3.34573E-07	5.90667E-08
Methylomicrobium_sp._wino1	2.33455E-07	0	0	9.89091E-08	0	8.96154E-07	3.33077E-08	1.51538E-07	1.09636E-07	9.11818E-08	3.33636E-08	1.37455E-07	0	1.69867E-07	1.57067E-08	1.144E-07
Methylococcus_sp._IM1	1.69273E-06	0	5.34545E-08	0.000000243	6.28769E-07	0	1.41015E-06	1.66777E-06	8.60818E-07	9.88364E-07	9.34818E-07	6.55545E-07	9.20933E-07	8.486E-07	5.78667E-07	1.40353E-06
Methylococcus_capsulatus	4.90273E-07	0	2.01273E-06	2.42182E-06	3.97692E-07	4.06462E-07	4.39769E-06	5.70177E-06	1.06664E-06	3.70091E-07	0.000000715	7.65273E-07	4.54533E-07	4.124E-07	0.000000639	7.10667E-07
Candidatus_Methylospira_mobilis	1.02827E-06	0	3.70909E-08	9.34909E-07	0.000000148	5.22154E-07	5.36923E-08	5.81538E-08	3.41364E-07	4.24273E-07	4.00727E-07	8.93455E-07	2.998E-07	3.62533E-07	5.256E-07	5.11267E-07
Methylomonas_koyamae	2.70909E-08	0	0	0	0	0	0	0	2.75455E-08	0	0	0	0	0	0	0
Methylomonas_sp._LW13	0.000000108	0	0	8.36364E-08	0	0	4.80769E-08	0.000000042	0.000000053	3.89091E-08	1.02727E-08	4.92727E-08	2.14E-08	0.000000041	5.94E-08	6.33333E-08
Methylomonas_denitrificans	0	0	0	9.54273E-08	4.93846E-08	0	2.63077E-08	0	0.000000154	2.76818E-07	2.15909E-07	2.11818E-07	4.61333E-08	0.000000084	2.55333E-08	0
Methylomonas_sp._DH-1	0	0	5.09091E-08	2.04909E-07	6.30769E-08	0	0	3.81538E-08	0	0	0	1.10545E-07	0	3.09333E-08	0	0
Methylomonas_sp._GJ1	1.74191E-06	3.08364E-07	1.72336E-06	1.04445E-06	0.000000176	0.000000026	1.10754E-06	1.16685E-06	9.30636E-07	8.95091E-07	7.16818E-07	1.33791E-06	1.65933E-07	1.72067E-07	1.41267E-07	5.994E-07
Methylomonas_methanica	5.36455E-07	1.37273E-08	2.88545E-08	5.18182E-09	0.000000271	0	3.38077E-07	2.03692E-07	4.12364E-07	0.000000461	1.10473E-06	8.49455E-07	4.438E-07	1.03107E-06	1.03753E-06	6.61133E-07
Spongiibacter_sp._IMCC21906	0	0	1.10909E-07	0	2.05385E-08	2.52308E-08	0	1.46923E-08	2.72727E-08	0	0	4.28182E-08	0	5.68E-08	6.46667E-08	4.95333E-08
Oceanicoccus_sagamiensis	1.39818E-07	0	3.86582E-07	2.76727E-07	1.78923E-07	6.27692E-08	1.20692E-06	1.30946E-06	0.000000247	0.000000162	9.35636E-07	5.72909E-07	1.92933E-07	3.37533E-07	8.58E-08	2.806E-07
Zhongshania_aliphaticivorans	7.26364E-08	1.16364E-08	5.52545E-08	0	0	0	0	0	0	0	6.66364E-08	5.37273E-08	2.24E-08	0	4.44E-09	0
Congregibacter_litoralis	1.11091E-07	7.87273E-08	8.21818E-08	9.09091E-08	1.19769E-07	0.000000419	0	0.000000062	8.54545E-08	1.06273E-07	4.69182E-07	1.75182E-07	4.38E-08	0.000000014	8.76E-08	0
Kineobactrum_sp._M2	3.77273E-07	0	3.70909E-08	5.66727E-07	1.39154E-07	8.54615E-07	2.00369E-06	1.19408E-06	0.000000379	2.31545E-07	5.13727E-07	7.30091E-07	1.99733E-07	2.10933E-07	2.18373E-07	5.21333E-08
Halioglobus_japonicus	0	0	3.27273E-08	1.43636E-08	2.55538E-07	0	0	2.01846E-07	5.34545E-08	0	0	0	2.72E-08	1.66467E-07	2.97333E-08	1.54133E-07
Halioglobus_pacificus	1.42455E-07	2.12818E-07	4.12727E-09	1.67545E-07	9.30769E-08	3.68462E-07	1.75538E-07	2.95769E-07	2.33091E-07	4.14091E-07	1.06545E-07	6.01818E-08	0.000000077	4.84667E-08	0.000000098	1.86667E-08
Halioglobus_maricola	2.40636E-07	0	3.28545E-08	1.08364E-07	2.27692E-07	1.46154E-07	4.33846E-08	1.51077E-07	5.63455E-07	2.74909E-07	2.74091E-07	5.66364E-07	6.99333E-08	1.71267E-07	9.32E-08	9.74667E-08
Microbulbifer_hydrolyticus	1.45727E-07	3.49091E-08	3.27273E-08	0.000000325	9.11538E-08	0.000001779	2.24385E-07	3.76385E-07	2.66182E-07	0.00000009	5.04091E-07	4.54636E-07	2.09333E-08	1.14333E-07	3.01733E-07	1.71867E-07
Microbulbifer_sp._YPW1	8.23636E-08	0	7.70909E-08	1.91818E-08	8.79231E-08	1.44154E-07	0.000000096	2.06538E-07	0	9.05455E-08	0	3.74091E-07	6.16E-08	1.656E-07	0	8.19333E-08
Microbulbifer_agarilyticus	4.01727E-07	5.23364E-07	1.85036E-07	8.81818E-08	4.11538E-08	1.45231E-07	9.74615E-08	3.36692E-07	3.75455E-08	1.10727E-07	0	0.000000475	2.08E-08	7.53333E-08	8.48E-08	3.20667E-08
Microbulbifer_sp._SH-1	6.56636E-07	5.22727E-08	3.78127E-07	1.03627E-06	4.95615E-07	5.19231E-08	9.39077E-07	1.27785E-06	8.49545E-07	8.70364E-07	0.000000778	2.75727E-07	6.94333E-07	6.148E-07	6.04133E-07	1.2306E-06
Microbulbifer_sp._GL-2	9.26364E-08	0	7.62182E-08	4.84364E-07	0	0	5.54615E-08	0	8.72727E-08	0	7.29091E-08	1.26909E-07	2.14667E-08	1.05333E-07	2.55333E-08	6.33333E-08
Microbulbifer_sp._A4B17	5.62545E-07	0	4.12727E-09	3.86818E-07	2.19462E-07	1.79231E-08	1.50385E-07	2.28154E-07	4.11455E-07	3.31727E-07	2.76182E-07	4.31818E-07	1.13267E-07	2.668E-07	4.63667E-07	0.000000281
Microbulbifer_sp._THAF38	7.42364E-07	0	1.75727E-06	2.13136E-06	4.97385E-07	0	4.90023E-06	6.37869E-06	1.09982E-06	6.91909E-07	5.78091E-07	4.14909E-07	5.78333E-07	0.000000405	7.62333E-07	6.60133E-07
Microbulbifer_sp._ALW1	5.80182E-07	3.87273E-08	1.07345E-07	4.18455E-07	1.16385E-07	4.90308E-07	5.36923E-08	3.00769E-08	0.000000371	1.32545E-07	9.05455E-08	7.64545E-08	1.55067E-07	1.746E-07	1.57067E-08	2.29267E-07
Microbulbifer_thermotolerans	0.000000343	8.86364E-09	1.40545E-07	5.35182E-07	1.05846E-07	0	0	9.15462E-07	4.25182E-07	4.70636E-07	4.15818E-07	7.67818E-07	2.384E-07	3.44467E-07	6.672E-07	3.60533E-07
Microbulbifer_aggregans	1.58055E-06	1.53273E-07	6.34636E-07	1.22027E-06	1.30031E-06	1.03692E-07	1.13946E-06	1.33685E-06	1.99818E-06	1.37455E-06	1.17509E-06	2.76564E-06	2.39373E-06	2.59687E-06	1.89973E-06	3.03873E-06
Simiduia_agarivorans	3.88364E-07	1.98545E-07	1.35636E-07	4.40091E-07	4.04615E-07	9.89769E-07	2.63077E-08	1.06077E-07	1.69455E-07	1.46364E-07	2.49455E-07	2.93909E-07	8.32E-08	0.000000066	7.74667E-08	3.84667E-08
Cellvibrio_sp._KY-GH-1	0	0	0	4.79091E-09	0	0	1.05385E-07	1.90769E-08	0	0	1.35364E-07	0	1.86667E-08	0	0	1.04867E-07
Cellvibrio_sp._PSBB023	6.43727E-07	4.37545E-07	2.14618E-07	4.49364E-07	0.000000379	8.93846E-08	5.40462E-07	2.85692E-07	2.36545E-07	5.65455E-08	2.40273E-07	0.000000586	1.01067E-07	1.39333E-07	4.03733E-07	4.796E-07
Cellvibrio_sp._KY-YJ-3	0	2.56818E-08	1.23636E-08	2.43636E-08	5.93846E-08	0	0	0	0	0	1.37273E-07	4.79091E-08	0	6.45333E-08	0	6.77333E-08
Cellvibrio_sp._PSBB006	8.14727E-07	8.69182E-07	0.000000232	2.78791E-07	0.000000078	0	2.78692E-07	7.10154E-07	2.77636E-07	4.04364E-07	4.14455E-07	1.29545E-07	0	2.30733E-07	2.01133E-07	1.17333E-07
Cellvibrio_japonicus	0	0.000000047	0	4.79091E-09	2.05385E-08	6.30923E-07	9.77692E-08	0	5.97273E-08	0	2.04091E-07	1.92955E-07	0	1.00867E-07	8.12E-08	2.31333E-07
Agarilytica_rhodophyticola	2.48364E-07	2.34545E-08	1.77273E-08	3.10636E-07	3.83385E-07	0	1.79231E-07	2.91615E-07	1.92636E-07	2.02636E-07	1.49727E-07	3.21455E-07	1.34667E-07	1.66333E-07	2.64333E-07	3.25267E-07
Teredinibacter_sp._Bs08	2.58545E-07	0.000000065	1.23636E-08	5.06636E-07	1.84308E-07	3.02769E-07	1.24077E-07	0.000000477	2.40545E-07	0.000000172	1.03091E-07	0.000000266	8.02667E-08	3.42667E-07	4.008E-07	2.89267E-07
Teredinibacter_turnerae	3.79091E-07	2.08182E-08	1.48182E-07	1.84982E-06	3.45077E-07	0	9.15385E-08	3.36923E-07	2.01618E-06	1.41909E-06	1.93718E-06	2.16591E-06	8.42133E-07	8.196E-07	8.10133E-07	8.82067E-07
Saccharophagus_degradans	2.00273E-07	0.000000065	0	9.07364E-07	1.27662E-06	5.05385E-08	2.26638E-06	3.40546E-06	2.75909E-07	1.64545E-07	0.000000478	2.92818E-07	1.61987E-06	2.0658E-06	1.90413E-06	2.5186E-06
Guyparkeria_halophila	4.73636E-08	0	0	4.79091E-09	3.15385E-08	0	8.96923E-08	7.12308E-08	0	3.62727E-08	0	7.89091E-08	6.08E-08	4.23333E-08	2.55333E-08	5.66E-08
Woeseia_oceani	3.01636E-07	0	0	9.54545E-09	1.93692E-07	0	7.92308E-08	7.03846E-08	2.31909E-07	3.86455E-07	5.44636E-07	4.48818E-07	2.72E-08	2.38E-08	1.49333E-08	3.72E-08
Sulfurivermis_fontis	3.87636E-07	0.000000995	2.57818E-07	4.95182E-07	1.83385E-07	5.64077E-07	7.22692E-07	6.56615E-07	5.99636E-07	7.77818E-07	7.31455E-07	0.000001138	1.956E-07	3.658E-07	2.688E-07	4.544E-07
Thiohalobacter_thiocyanaticus	3.22364E-07	0.00000013	4.12727E-09	3.37364E-07	2.88308E-07	5.66462E-07	3.63615E-07	3.10308E-07	0.000000307	0	6.71818E-08	5.30182E-07	3.24133E-07	2.64333E-07	1.86933E-07	3.194E-07
Wenzhouxiangella_marina	3.50182E-07	0	0.000000046	0.000000079	0	5.19692E-07	4.80769E-08	0	2.82455E-07	0.000000075	0.00000035	3.46182E-07	0	0.00000007	2.62E-08	1.796E-07
Wenzhouxiangella_sp._AB-CW3	1.99464E-06	1.94545E-07	4.12727E-09	8.64364E-07	3.93846E-07	3.58462E-08	2.37538E-07	1.02154E-06	8.94455E-07	0.000000533	1.10909E-06	0.000000993	3.92667E-07	5.56667E-07	0.000000783	8.98933E-07
Halothiobacillus_sp._LS2	1.29545E-07	0	5.68091E-07	0.000000275	2.88462E-08	0	8.52615E-07	5.74231E-07	0.000000207	1.87182E-07	2.49455E-07	9.38182E-08	4.78667E-08	1.376E-07	2.48133E-07	4.12533E-07
Halothiobacillus_neapolitanus	3.69091E-08	0	1.63636E-08	4.23336E-07	1.92308E-07	1.23846E-07	1.43692E-07	3.04231E-07	5.79091E-08	3.03636E-08	6.68182E-08	1.66273E-07	2.70933E-07	2.04933E-07	1.646E-07	3.44267E-07
Sulfuriflexus_mobilis	1.01182E-07	9.54545E-09	0	1.06273E-07	3.58462E-08	0	7.45385E-08	4.38462E-08	3.98182E-08	6.37273E-08	1.85455E-07	4.28182E-08	5.18667E-08	2.38E-08	3.31333E-08	3.84667E-08
Granulosicoccus_antarcticus	1.62636E-06	0.00000013	1.08818E-06	1.34109E-06	5.55385E-07	0.000000039	5.36231E-07	1.70438E-06	1.53109E-06	6.63455E-07	1.23291E-06	1.64909E-06	9.12533E-07	6.542E-07	5.988E-07	1.09787E-06
Spiribacter_roseus	8.69091E-08	0	0	4.00909E-08	6.73846E-08	9.46154E-09	2.16231E-07	1.02692E-07	0.000000148	8.08182E-08	2.78455E-07	7.78182E-08	0.00000019	7.29333E-08	7.87733E-07	1.558E-07
Spiribacter_sp._2438	0.000000161	0	0	1.93636E-08	1.15846E-07	0	0	1.90769E-08	3.75455E-08	0	1.34182E-07	9.20909E-08	4.00667E-08	1.61333E-08	3.14667E-08	0
Spiribacter_salinus	5.68182E-08	0	3.70909E-08	2.06273E-07	4.13846E-08	0	1.52615E-07	1.40692E-07	1.81636E-07	8.85455E-08	7.85455E-08	9.03636E-08	7.20667E-08	0.000000041	0	1.91933E-07
Spiribacter_curvatus	5.27273E-07	4.42455E-07	1.61036E-07	2.87727E-07	2.47538E-07	1.23846E-07	0.000000293	1.62308E-07	2.67455E-07	0.000000081	2.34182E-07	3.92091E-07	0.000000099	1.662E-07	2.178E-07	1.83133E-07
Alkalilimnicola_ehrlichii	0.000000474	2.09318E-07	2.164E-07	5.02727E-07	0.000000262	1.04769E-07	2.32538E-07	3.57385E-07	5.15182E-07	3.82182E-07	3.76818E-07	8.74636E-07	3.53867E-07	0.000000338	2.31733E-07	2.154E-07
Ectothiorhodospira_haloalkaliphila	9.46364E-08	1.00955E-07	2.94727E-07	9.66091E-08	9.52308E-08	0	2.86692E-07	1.71077E-07	2.96818E-07	6.25455E-08	6.88182E-08	2.15909E-07	1.564E-07	2.38E-08	1.24707E-07	1.46533E-07
Ectothiorhodospira_sp._BSL-9	1.12464E-06	9.09273E-07	1.65455E-06	1.01836E-06	1.80308E-06	5.93692E-07	3.92769E-07	2.56769E-07	1.87991E-06	1.75782E-06	1.64927E-06	1.88273E-06	1.5776E-06	1.85313E-06	1.84753E-06	0.000001704
Halorhodospira_halophila	1.40091E-07	6.00364E-07	7.57273E-08	4.53727E-07	8.79231E-08	2.51615E-07	1.52308E-07	3.32385E-07	0.000000106	4.00091E-07	1.22727E-07	0.000000191	6.87333E-08	1.038E-07	1.948E-07	3.85333E-08
Halorhodospira_halochloris	1.17536E-06	0	1.10909E-07	9.27545E-07	8.27154E-07	0	0	9.84615E-08	1.14673E-06	0.000001665	9.60636E-07	1.51927E-06	1.1716E-06	1.00127E-06	1.26633E-06	1.2654E-06
Acidihalobacter_ferrooxydans	0	0	0	1.91818E-08	7.32308E-08	1.06923E-07	0	0	2.72727E-08	0.000000028	0	0	0	3.99333E-08	2.62E-08	0
Acidihalobacter_prosperus	1.02818E-07	0.000000065	2.87273E-08	0.000000052	4.93846E-08	2.13846E-08	0	3.81538E-08	2.64545E-08	1.87182E-07	9.64545E-08	0.000000247	0	6.36E-08	1.49333E-08	6.94E-08
Acidihalobacter_aeolianus	1.60236E-06	0.000000177	4.32182E-07	0.000000574	7.33769E-07	0	0.000000171	3.34385E-07	2.38673E-06	1.26055E-06	1.23109E-06	2.01773E-06	1.65507E-06	1.4812E-06	1.8434E-06	2.5578E-06
Thioalkalivibrio_sp._K90mix	1.41091E-07	2.01636E-07	9.64545E-08	3.15455E-07	2.45923E-07	1.82308E-07	2.63077E-08	2.38308E-07	0.000000129	5.22727E-08	3.95455E-08	2.63091E-07	1.00133E-07	6.87333E-08	1.72067E-07	3.60333E-07
Thioalkalivibrio_nitratireducens	2.72545E-07	3.95091E-07	1.91036E-07	3.43364E-08	1.78077E-07	2.19846E-07	1.13231E-07	0.000000022	8.54545E-08	9.40909E-08	4.98182E-08	1.87909E-07	2.64E-08	3.09333E-08	1.78E-08	8.96E-08
Thioalkalivibrio_paradoxus	1.13545E-07	1.01182E-07	3.68545E-08	2.36091E-07	2.05385E-08	0	4.33846E-08	0	1.68909E-07	0.000000062	0	8.98182E-08	5.34667E-08	0.000000028	1.57067E-08	4.01333E-08
Thioalkalivibrio_sulfidiphilus	1.84009E-06	0.000000432	3.57818E-07	1.11327E-06	4.58846E-07	6.88077E-07	9.33769E-07	5.79692E-07	1.29809E-06	9.31545E-07	1.56409E-06	1.46573E-06	8.96333E-07	1.1604E-06	0.000000663	6.09467E-07
Thioalkalivibrio_versutus	6.98364E-07	3.60455E-08	9.93909E-08	4.76273E-07	2.78538E-07	0.000000026	5.27231E-07	3.27231E-07	3.91818E-07	6.11091E-07	4.48455E-07	2.45636E-07	3.37067E-07	4.37733E-07	4.1084E-07	5.25733E-07
Marichromatium_purpuratum	6.91545E-07	5.85182E-07	2.69709E-07	0.000000892	6.03846E-08	2.02462E-07	1.88077E-07	1.14538E-07	3.19364E-07	2.30909E-07	4.79636E-07	6.79455E-07	1.584E-07	2.06667E-07	6.55333E-08	3.67333E-08
Thiocystis_violascens	0.00000462	5.56818E-07	4.88468E-06	6.40882E-06	1.52592E-06	1.34069E-06	1.01015E-05	1.04932E-05	0.000004157	5.55164E-06	5.69636E-06	3.29745E-06	1.5194E-06	1.54433E-06	2.46267E-06	0.00000366
Thermochromatium_tepidum	5.45455E-07	5.96182E-07	4.12727E-09	1.59845E-06	3.30769E-07	0.000001869	2.58385E-06	2.05738E-06	3.99455E-07	6.32909E-07	6.70455E-07	7.65455E-07	1.144E-07	0.000000273	4.46733E-07	3.592E-07
Allochromatium_vinosum	1.19509E-06	1.53273E-07	6.96909E-07	8.28273E-07	4.17308E-07	5.25308E-07	0.000000734	3.31077E-06	9.88727E-07	6.00545E-07	0.000001166	1.29282E-06	7.96067E-07	7.61267E-07	6.65667E-07	8.10067E-07
Rheinheimera_sp._D18	0	0	0	0	3.58462E-08	0	0	5.81538E-08	0	0	0	0	0	3.29333E-08	0	1.596E-07
Rheinheimera_sp._LHK132	5.13545E-07	1.53455E-07	3.08727E-07	7.36273E-07	1.12869E-06	0	1.61923E-07	4.47231E-07	8.97182E-07	4.37818E-07	0.000000218	7.30909E-07	7.658E-07	1.0228E-06	1.26033E-06	1.31407E-06
Nitrosococcus_oceani	3.11818E-08	0	0	0	0	0	0	0	0	0	0	0	0	0	0	0
Nitrosococcus_halophilus	0	1.58636E-07	0	4.79091E-09	0	0	0	0	3.98182E-08	0	3.36091E-07	8.26818E-08	8.24667E-08	0.000000014	0	1.66667E-08
Nitrosococcus_watsonii	6.94636E-07	2.17273E-07	1.81036E-07	1.40364E-07	2.88462E-08	0	1.13231E-07	1.95769E-07	2.07182E-07	3.17636E-07	5.30909E-07	4.78318E-07	0	6.80667E-08	4.44E-09	5.19333E-08
Nitrosococcus_wardiae	1.45273E-07	0	6.71818E-08	4.13818E-07	2.24154E-07	2.40154E-07	0	1.59462E-07	1.73727E-07	1.29091E-07	3.76091E-07	4.60909E-07	4.96E-08	2.63933E-07	3.88667E-08	1.44667E-07
Thioflavicoccus_mobilis	4.04818E-07	0.00000013	4.95364E-08	3.21455E-07	1.60308E-07	5.47692E-08	3.33077E-08	1.73923E-07	3.00091E-07	0.000000096	9.16364E-08	1.44727E-07	0.0000002	2.658E-07	0.000000084	5.17933E-07
Candidatus_Nitrosoglobus_terrae	5.77273E-07	0	0	1.99091E-07	1.25515E-06	0	1.13231E-07	1.70308E-07	4.22909E-07	2.45182E-07	3.42545E-07	0.000000252	0.000001021	8.998E-07	6.36333E-07	1.13553E-06
Candidatus_Thiodictyon_syntrophicum	1.80864E-06	4.73364E-07	4.15982E-07	0.000000698	9.94077E-07	5.35769E-07	2.46615E-07	4.97538E-07	2.00409E-06	1.77955E-06	1.63991E-06	2.38091E-06	2.23687E-06	1.59007E-06	1.37907E-06	1.85613E-06
Succinivibrio_dextrinosolvens	1.33336E-05	0.000002567	4.15377E-06	5.37718E-06	4.97154E-06	3.99027E-05	1.72731E-05	1.47177E-05	1.16545E-05	1.30964E-05	1.24491E-05	1.13473E-05	6.30933E-06	0.000004852	5.85267E-06	7.47867E-06
Tolumonas_auensis	1.86727E-07	0	4.58455E-08	5.19636E-07	0.000000154	2.20231E-07	1.07462E-07	5.99692E-07	4.24636E-07	4.35818E-07	7.11364E-07	5.54727E-07	1.96067E-07	4.998E-07	3.59067E-07	2.39333E-07
Oceanimonas_sp._GK1	1.58182E-08	3.59864E-07	8.12182E-08	1.10909E-07	1.13769E-07	5.32769E-07	1.12538E-07	2.38769E-07	6.45455E-08	1.47727E-07	1.06727E-07	0.000000138	1.10067E-07	1.43067E-07	0.00000012	1.76933E-07
Zobellella_denitrificans	7.19727E-07	2.04909E-07	3.91636E-07	9.76091E-07	0.000000437	4.84385E-07	2.65154E-07	3.07923E-07	9.26182E-07	1.08945E-06	0.000000977	1.00909E-06	2.56133E-07	5.172E-07	3.44267E-07	5.81933E-07
Oceanisphaera_profunda	2.83182E-07	2.46364E-07	5.46727E-07	2.09636E-07	5.90769E-08	0	1.18754E-06	1.09308E-06	3.79455E-07	5.65455E-08	0.000000123	1.49591E-07	4.23333E-08	1.37933E-07	1.05733E-07	2.79067E-07
Oceanisphaera_avium	5.12727E-08	0.000000593	0	0	4.54846E-07	6.13077E-08	3.11538E-08	0	5.92727E-07	2.08273E-07	5.93364E-07	6.13636E-07	3.016E-07	6.702E-07	6.38267E-07	9.922E-07
Aeromonas_sp._CU5	0	0	0	0	0	0	0	2.93846E-08	0	0	0	0	0	0	0	0
Aeromonas_sp._Ne-1	0	7.45455E-08	0	4.79091E-09	0	0	0	0	0	0	0	1.25364E-07	0	0	0	0
Aeromonas_sp._ASNIH4	0	0	0	9.50909E-08	0	0	0	1.90769E-08	0	0	0	0	2.14E-08	0	1.49333E-08	0
Aeromonas_dhakensis	3.62727E-08	0	0	0	2.75385E-08	0	0	0	0	0	0	0	0	0	1.57067E-08	0
Aeromonas_sp._ASNIH5	0	0	0	5.18182E-09	0	0	0	0.000000022	0	0	3.95455E-08	0	2.09333E-08	0	2.99733E-08	0
Aeromonas_sp._ASNIH3	1.58182E-08	0	7.14364E-07	9.03636E-08	0	0	1.18723E-06	8.20692E-07	2.16364E-08	0.000000028	0	1.10545E-07	1.44133E-07	8.69333E-08	8.93333E-08	1.148E-07
Aeromonas_sp._CA23	3.36364E-07	0.000000065	3.27273E-08	2.377E-07	1.63923E-07	4.13846E-08	2.63077E-08	3.15462E-07	5.04364E-07	3.72455E-07	0.000000154	5.84636E-07	0	0.000000098	1.44373E-07	1.66667E-08
Aeromonas_caviae	2.57182E-07	6.04182E-07	7.11818E-08	0.00000047	3.58462E-08	9.38462E-08	6.16385E-07	2.69769E-07	1.29091E-07	0	2.06364E-07	1.66273E-07	8.79333E-08	2.69267E-07	2.31533E-07	2.456E-07
Aeromonas_simiae	0.000000273	6.09091E-08	3.00182E-07	4.54091E-07	1.95385E-07	0.000000239	0.000000145	1.27154E-07	3.38273E-07	0.000000445	3.26636E-07	4.24455E-07	1.00267E-07	6.49333E-08	1.61267E-07	1.25067E-07
Aeromonas_schubertii	1.57991E-06	9.58364E-07	7.03673E-07	0.000000967	2.11769E-07	8.56154E-08	1.00285E-06	1.28277E-06	8.49182E-07	5.80727E-07	7.09909E-07	1.35427E-06	3.03133E-07	5.02333E-07	3.50533E-07	4.67533E-07
Aeromonas_sp._WP2-W18-CRE-05	6.80909E-08	0	0	0	0	0	0	3.57692E-08	3.75455E-08	2.67091E-07	0.000000039	1.42727E-08	0	2.04067E-07	2.99333E-08	6.33333E-08
Aeromonas_salmonicida	2.27727E-07	1.20182E-06	7.84182E-07	4.26182E-07	2.08308E-07	8.84615E-08	2.24231E-07	2.89308E-07	3.36818E-07	3.89091E-08	1.14818E-07	5.31182E-07	0.000000231	2.628E-07	2.248E-07	2.21733E-07
Aeromonas_media	5.44818E-07	1.41636E-07	8.25455E-09	6.07636E-07	3.27154E-07	5.16385E-07	5.31615E-07	2.08769E-07	2.64727E-07	4.27364E-07	4.83545E-07	1.12127E-06	2.666E-07	2.686E-07	6.95333E-08	2.17533E-07
Aeromonas_hydrophila	1.62955E-06	6.16455E-07	4.47127E-07	5.10364E-07	5.74462E-07	8.00846E-07	1.00192E-06	6.63846E-07	1.37518E-06	1.46164E-06	1.65982E-06	1.79745E-06	9.792E-07	1.06953E-06	7.83867E-07	4.95267E-07
Aeromonas_veronii	3.97091E-06	1.80309E-06	8.16982E-07	2.68082E-06	1.41277E-06	2.78562E-06	2.17431E-06	2.88531E-06	2.23809E-06	3.36764E-06	3.94118E-06	4.14455E-06	1.36413E-06	1.14533E-06	1.42267E-06	2.3844E-06
Aeromonas_encheleia	3.06082E-06	0.00000013	7.57273E-07	1.09109E-06	7.52385E-07	7.43077E-06	2.12569E-06	3.49954E-06	1.62527E-06	8.01364E-07	2.67091E-06	1.29136E-06	6.642E-07	1.52333E-06	9.27867E-07	1.81187E-06
Candidatus_Rickettsiella_viridis	7.91818E-08	0	0	3.55182E-08	1.21923E-07	5.89692E-07	1.62769E-07	1.84846E-07	1.88273E-07	0	2.69455E-07	2.09455E-07	7.13333E-08	2.13533E-07	1.53267E-07	3.47333E-07
Candidatus_Coxiella_mudrowiae	7.85455E-08	3.61455E-07	3.70909E-08	5.18182E-09	0	0	0	0	0	5.22727E-08	2.76364E-08	0	4.30667E-08	0	0	0
Coxiella_burnetii	6.13636E-08	0	0	3.07273E-08	0	6.56615E-07	1.33846E-07	4.82923E-07	9.91818E-08	6.25455E-08	1.14636E-07	0	9.93333E-08	5.97333E-08	1.096E-07	1.42867E-07
Coxiella_endosymbiont_of_Amblyomma_sculptum	7.41909E-07	0	5.16455E-07	8.30727E-07	2.42692E-07	0	9.47154E-07	2.35308E-07	3.84455E-07	5.18364E-07	5.93364E-07	3.90545E-07	0.000000276	2.97267E-07	4.148E-07	5.10733E-07
Aquicella_lusitana	0	0	9.64545E-08	1.39909E-07	0.000000061	0	0	1.54308E-07	2.72727E-08	1.46727E-07	1.38636E-07	2.51909E-07	2.758E-07	1.55933E-07	6.24133E-07	4.56467E-07
Aquicella_siphonis	3.11818E-08	0	2.18545E-08	7.08182E-08	0	0	0	0	0	0.000000047	1.56182E-07	1.75455E-07	4.88E-08	2.38E-08	0	3.40667E-08
Tatlockia_micdadei	3.11818E-08	0.000000065	0	0	0	0	0	0	0	0	0	3.74545E-08	0	0	0	0
Fluoribacter_dumoffii	1.77818E-07	0	3.70909E-08	3.49273E-07	7.81538E-08	2.52308E-08	7.45385E-08	2.04846E-07	0.000000212	6.42727E-08	0.0000001	2.92091E-07	2.24E-08	1.36467E-07	2.37373E-07	8.48667E-08
Legionella_waltersii	0	0	0	0	0	0	0	0	0	0	0	5.38182E-07	0.000000154	0	1.49333E-08	3.40667E-08
Legionella_sp._PC997	0	4.63182E-08	0	0	3.34615E-08	0	0	2.97692E-08	0	0	0	0	0	0	1.49333E-08	0
Legionella_fallonii	0	0	0	0	8.23077E-08	0	0	0	0	0	0	0	1.04933E-07	9.71333E-08	1.40973E-07	6.56E-08

Legionella_adelaidensis	0	0	0	0	0	0	0	0	0	0	2.76364E-08	4.21364E-08	0	0	5.94E-08	0
Legionella_sp._PC1000	0	0	0	4.79091E-09	1.01769E-07	0	0	9.33077E-07	0.000000027	0	2.76364E-08	4.21364E-08	0.000000196	1.67067E-07	4.24933E-07	3.216E-07
Legionella_clemsonensis	0	0	3.70909E-08	0	0	0	0	0	0	0	6.65455E-08	1.42727E-08	0	0	0	0
Legionella_anisa	0.000000092	7.66364E-08	3.70909E-08	0	3.34615E-08	0	0	0	0	6.08182E-08	3.95455E-08	0	2.08E-08	5.68E-08	7.59333E-08	8.19333E-08
Legionella_geestiana	0	0	1.10909E-07	4.06364E-08	1.48231E-07	5.36154E-07	3.77692E-08	1.41692E-07	0	2.74273E-07	4.81818E-08	1.16136E-06	2.64E-08	7.97333E-08	1.00667E-07	5.96667E-08
Legionella_lansingensis	0	0.000000065	1.14545E-07	0	0	0	0	0	1.19091E-07	0.000000047	0	1.36955E-07	2.09333E-08	0	0	0
Legionella_oakridgensis	3.26273E-07	3.82364E-07	1.61182E-07	1.61273E-07	1.07692E-07	2.95385E-08	2.44615E-07	4.66923E-08	1.53636E-07	0.000000028	6.78455E-07	4.41182E-07	0	9.78E-08	4.062E-07	3.87067E-07
Legionella_hackeliae	4.74636E-07	4.66636E-07	4.99455E-07	2.83364E-07	2.02462E-07	0	0.000000096	2.30846E-07	1.21909E-07	1.95727E-07	2.32273E-07	2.99273E-07	4.27333E-08	1.756E-07	1.912E-07	8.74E-08
Legionella_jordanis	1.20636E-07	0	2.59091E-07	4.48818E-08	0	0	1.42385E-07	2.43077E-07	1.50364E-07	3.62727E-08	1.06182E-07	8.80909E-08	0	1.61333E-08	4.04667E-08	7.57333E-08
Legionella_spiritensis	0	0	4.12727E-09	9.54545E-09	7.46923E-08	7.66154E-08	0	1.46923E-08	0	0.000000047	0	1.42727E-08	9.12E-08	2.36067E-07	2.23267E-07	8.99333E-08
Legionella_cherrii	0	0	0	0	0	0	0	0	0	5.22727E-08	0	0	6.82E-08	6.78E-08	1.08533E-07	4.04E-08
Legionella_israelensis	3.34182E-07	0.000000065	4.67273E-08	1.85636E-07	3.70154E-07	1.67538E-07	5.26154E-08	7.68462E-08	2.59545E-07	3.16273E-07	5.39091E-08	1.32455E-07	3.42267E-07	3.90267E-07	2.24307E-07	3.95733E-07
Legionella_longbeachae	1.58182E-08	0	0.000000047	3.33791E-07	0	6.53077E-08	0	2.81615E-07	0.000000054	0.000000047	1.33455E-07	1.12045E-07	4.68E-08	5.19333E-08	1.65333E-08	9.32E-08
Legionella_pneumophila	1.09282E-06	1.80091E-07	2.86727E-07	4.70182E-07	1.06377E-06	8.95385E-07	4.13462E-07	1.00592E-06	5.16455E-07	3.74364E-07	3.62818E-07	3.53545E-07	6.266E-07	8.46067E-07	1.32073E-06	7.572E-07
Legionella_sp._TUM19329	4.60182E-07	2.41818E-08	4.12727E-09	4.35455E-08	7.26692E-07	4.94308E-07	1.12308E-07	5.72923E-07	2.75364E-07	3.03636E-08	0.000000125	1.11227E-07	6.246E-07	5.45067E-07	1.29431E-06	1.1326E-06
Legionella_sainthelensi	0	0	2.18545E-08	7.97273E-08	7.25538E-07	9.46154E-09	0	7.08308E-07	1.68091E-07	0.000000034	0	1.92182E-07	4.69933E-07	8.50667E-07	7.39867E-07	4.752E-07
Legionella_endosymbiont_of_Polyplax_serrata	4.61182E-06	0	1.14545E-07	4.79091E-09	1.84189E-05	0	3.77692E-08	0	0	0	0	0	1.29487E-05	1.17337E-05	1.23672E-05	7.6108E-06
Candidatus_Enterovibrio_luxaltus	1.23636E-07	0	2.47273E-08	9.85182E-08	2.59231E-08	2.52308E-08	0	0	2.16364E-08	7.51818E-08	0	7.13636E-09	0	0	0	5.53333E-08
Grimontia_hollisae	1.58636E-07	0	3.70909E-08	0.000000071	1.52615E-07	0	4.80769E-08	4.53846E-07	0.000000622	4.11455E-07	1.43545E-07	2.32091E-07	6.652E-07	1.166E-07	2.042E-07	1.08727E-06
Paraphotobacterium_marinum	0	1.16364E-08	5.73455E-08	3.24545E-08	1.39692E-07	1.08154E-07	0	1.90769E-08	0	2.87273E-08	9.64545E-08	1.53909E-07	0.000000074	6.22667E-08	2.99333E-08	9.17333E-08
Salinivibrio_sp._YCSC6	0	0	0	1.93636E-08	0	0	0	0	0	0	0	0	0	0	0	0
Salinivibrio_costicola	1.58182E-08	0	2.96364E-08	8.91818E-08	0	0	2.73846E-08	5.98462E-08	7.05455E-08	0	9.44545E-08	8.60455E-08	6.60667E-08	1.32867E-07	1.04707E-07	0.000000088
Salinivibrio_kushneri	1.09636E-07	0	2.07636E-08	1.94182E-07	9.38462E-08	1.65615E-07	8.86154E-08	5.40769E-08	1.86455E-07	5.22727E-08	2.09455E-07	2.36727E-07	4.88E-08	0.000000067	4.93067E-08	1.62933E-07
Aliivibrio_wodanis	0	0	0	1.50909E-07	1.38077E-07	0	0	0	0	0	0	0	2.14E-08	1.21133E-07	1.09533E-07	8.67333E-08
Aliivibrio_salmonicida	0	0	0	0	0	0	2.63077E-08	0	0	0	0	0	0	0	4.38667E-08	0
Aliivibrio_fischeri	0	1.37273E-08	4.12727E-09	0	8.79231E-08	0	0	0.000000011	0	0	0	7.13636E-09	1.014E-07	8.12667E-08	6.324E-08	7.33333E-08
Photobacterium_profundum	2.48545E-07	7.74091E-07	1.04764E-07	1.46255E-06	8.76923E-08	1.47923E-06	3.08046E-06	1.90015E-06	3.14273E-07	2.53818E-07	4.19545E-07	1.01127E-06	1.07867E-07	4.80667E-08	2.86933E-07	4.06667E-07
Photobacterium_gaetbulicola	7.15091E-07	5.01818E-08	1.58818E-07	1.29364E-07	1.24538E-07	1.30215E-06	8.13846E-08	1.56769E-07	2.31364E-07	1.84364E-07	5.44091E-07	6.67455E-07	9.66667E-08	1.336E-07	5.72E-08	2.416E-07
Photobacterium_damselae	8.40636E-06	7.26182E-07	1.86517E-06	8.68364E-06	0.000002921	4.94462E-07	1.20831E-06	3.64077E-06	5.76364E-06	4.88591E-06	5.99727E-06	5.83764E-06	4.49047E-06	5.23307E-06	3.7542E-06	4.95267E-06
Vibrio_sp._Scap24	0	0	0	0	3.34615E-08	0	0	0	0	0	0	0	0	0	0	0
Vibrio_diabolicus	0	0	0	0	0	0	0	0	0	0	0	0.000000035	2.09333E-08	0	0	0
Vibrio_astriarenae	0	0	0	0	2.05385E-08	1.78769E-07	0	0	0	0	0	7.13636E-09	0	0	1.49333E-08	3.67333E-08
Vibrio_scophthalmi	0	0	0	0	2.25385E-07	0	0	3.22308E-08	0.000000084	0.000000034	0	7.13636E-09	5.55333E-08	0	3.43733E-08	2.13933E-07
Vibrio_sp._dhg	0	0	0	0	0	0	0	0	0	0	0	0	0	0	4.44E-09	1.22E-08
Vibrio_sp._EJY3	2.98182E-08	0.00000013	0	1.51545E-08	0	0	0	0	0	0	0	4.28182E-08	0	0	4.44E-09	6.33333E-08
Vibrio_rotiferianus	2.98182E-08	0	0	0	0	0	0	0	0	0	0	0	0	0	6.564E-08	0
Vibrio_rumoiensis	0	0	0	0	0	5.10615E-07	9.77692E-08	0	0	0	0	7.55455E-08	0	0	8.93333E-08	0
Vibrio_tritonius	5.42727E-08	0	0	4.79091E-09	8.79231E-08	0	1.85923E-07	0	1.21909E-07	0	0	6.09091E-08	0	6.26E-08	7.43333E-08	0
Vibrio_navarrensis	6.08182E-08	2.11818E-08	0	4.02727E-08	0.000000108	0	2.63077E-08	0	2.72727E-08	2.87273E-08	0	2.14545E-08	2.64E-08	9.44667E-08	1.2264E-07	0
Vibrio_chagasii	2.30909E-08	0	0	0	3.34615E-08	5.05385E-08	0	0	1.84727E-07	1.68091E-07	1.99727E-07	1.25318E-07	0	0	0	5.19333E-08
Vibrio_crassostreae	2.27545E-07	3.68182E-08	0	7.06364E-08	7.72308E-08	3.83077E-08	2.73846E-08	0	0	8.95455E-08	1.02727E-08	3.58364E-07	2.08E-08	0	0	5.70667E-08
Vibrio_nigripulchritudo	6.65455E-08	0	0	3.24545E-08	0	1.79231E-08	6.23077E-08	3.93846E-08	1.02455E-07	0	1.02727E-08	3.57273E-08	0	5.12E-08	2.24667E-08	7.54667E-08
Vibrio_ponticus	6.25455E-08	9.54545E-09	0	3.24545E-08	1.68769E-07	0	0	6.32308E-08	0	5.22727E-08	2.76364E-08	1.95773E-07	1.73133E-07	4.69333E-08	1.13573E-07	1.88533E-07
Vibrio_qinghaiensis	0.000000133	0	0	5.20636E-08	1.39231E-07	4.33846E-08	5.74615E-08	2.93846E-08	3.62909E-07	1.27273E-07	3.95455E-08	1.32182E-07	0.000000027	0	0	9.93333E-08
Vibrio_sp._HBUAS61001	0	0	0	0	1.56308E-07	5.06231E-07	5.96154E-08	9.42308E-08	8.91818E-08	0.000000028	1.45636E-07	3.63818E-07	0	0	4.44E-09	0
Vibrio_alginolyticus	1.64182E-07	2.05545E-07	0	0	7.81538E-08	6.76923E-08	0	7.69231E-08	8.43636E-08	0	3.95455E-08	1.42727E-08	0	0	0	4.95333E-08
Vibrio_europaeus	0	0	0	5.16364E-08	7.23077E-08	4.13846E-08	0	0	2.16273E-07	0.000000028	2.95636E-07	4.28182E-08	8.08667E-08	8.35333E-08	4.44E-09	0.000000055
Vibrio_cidicii	0	8.86364E-09	1.63636E-08	0	2.05385E-08	0	0	0	0	0	0	0	2.14E-08	0	1.12667E-08	6.26667E-08
Vibrio_natriegens	5.57273E-08	3.24545E-07	1.63636E-08	3.36273E-07	1.49923E-07	2.52308E-08	0	9.02308E-08	2.74818E-07	6.74545E-08	2.06364E-07	8.60455E-08	0	0.000000169	0	1.22E-08
Vibrio_cholerae	1.77364E-07	2.09727E-07	4.10909E-08	2.58091E-07	1.00846E-07	4.13846E-08	2.63077E-08	9.03846E-08	6.14545E-08	3.89091E-08	2.03182E-07	1.27727E-07	1.258E-07	8.84667E-08	1.28667E-07	9.86E-08
Vibrio_sp._Sal10	2.56364E-08	1.68182E-08	1.28182E-07	2.87273E-08	2.75385E-08	0	0	0	2.03364E-07	2.87273E-08	1.04273E-07	5.50455E-08	1.802E-07	1.58E-08	3.14667E-08	0
Vibrio_metoecus	4.29091E-08	9.54545E-09	2.33636E-07	6.24545E-08	0	0	1.50769E-07	4.37692E-08	4.86364E-08	0	3.77273E-08	0	2.24E-08	0	9.364E-08	4.086E-07
Vibrio_mimicus	5.86364E-08	2.88455E-07	9.54545E-08	3.30091E-07	0	4.13846E-08	3.77692E-08	4.93846E-08	3.45455E-07	5.88182E-07	2.84364E-07	1.66818E-07	2.14667E-08	1.71333E-08	1.65333E-08	0
Vibrio_taketomensis	6.80909E-08	0	0	8.16364E-08	3.58462E-08	0	0	0	6.45455E-08	6.92727E-08	1.07909E-07	2.85455E-08	2.64667E-08	9.24E-08	7.41333E-08	8.19333E-08
Vibrio_spartinae	0	0	0	0	1.12923E-07	0	0	0	1.20182E-07	3.89091E-08	1.12455E-07	7.13636E-09	4.78E-08	6.80667E-08	0	0
Vibrio_casei	2.96364E-08	0.000000065	0	0	0	0	3.33077E-08	1.03692E-07	1.80545E-07	0	1.04364E-07	4.65909E-08	0	1.61333E-08	0	0.000000168
Vibrio_sp._ZWAL4003	1.26091E-07	0	1.10909E-07	9.36364E-08	3.58462E-08	0	4.33846E-08	1.87615E-07	1.13182E-07	5.22727E-08	5.39091E-08	0.000000465	1.594E-07	0	2.73867E-07	9.73333E-08
Vibrio_harveyi	1.08427E-06	0	3.70909E-08	9.45455E-07	3.03769E-07	0	5.36923E-08	1.79154E-07	8.17364E-07	2.60455E-07	1.65373E-06	7.09136E-07	2.54133E-07	2.292E-07	6.89467E-07	5.13267E-07
Vibrio_breoganii	2.70909E-08	0	0	1.43636E-08	2.88462E-08	0	3.11538E-08	0	2.64545E-08	0	0.000000039	2.14545E-08	0	1.00933E-07	0	0
Vibrio_atlanticus	9.57455E-07	0.00000013	5.26455E-07	6.67182E-07	3.40692E-07	5.22692E-07	6.40769E-08	0.000000121	7.21364E-07	2.40545E-07	7.03545E-07	3.01182E-07	3.014E-07	1.61867E-07	2.40067E-07	1.51867E-07
Vibrio_anguillarum	3.11818E-08	1.04545E-08	6.66364E-08	8.49091E-08	4.62308E-08	0	0	3.22308E-08	0	0	7.29091E-08	1.42727E-08	1.86667E-08	0	0	0
Vibrio_owensii	5.42727E-08	0	1.42545E-07	3.39091E-08	0	0	0	3.57692E-08	0	0	0	1.12591E-07	0	0	3.31333E-08	2.92E-08
Vibrio_aphrogenes	1.04909E-07	1.68182E-08	4.12727E-09	1.03636E-08	8.02308E-08	0	2.73846E-08	0	6.59091E-08	0	1.00545E-07	1.42727E-08	1.562E-07	2.27133E-07	7.11333E-08	1.86E-08
Vibrio_tubiashii	0.000000282	0	4.12727E-09	6.23636E-08	9.84615E-08	6.53077E-08	3.11538E-08	1.00923E-07	1.83727E-07	4.63455E-07	3.11636E-07	1.54455E-07	0	6.62E-08	1.12667E-08	1.06133E-07
Vibrio_alfacsensis	0	0.000000112	3.42727E-08	1.62182E-07	0	1.35385E-07	1.98615E-07	1.19615E-07	0	8.02727E-08	1.56182E-07	9.59091E-08	0	0	6.46667E-08	6.33333E-08
Vibrio_jasicida	0	0	4.12727E-09	9.54545E-09	0	7.66154E-08	5.96154E-08	0	0	0	2.76364E-08	4.92727E-08	0	5.01333E-08	0	6.94E-08
Vibrio_vulnificus	8.30545E-07	8.49091E-08	1.95027E-07	0.000001032	4.39154E-07	4.37923E-07	8.45923E-07	1.22838E-06	5.94091E-07	9.23818E-07	9.67455E-07	1.19973E-06	5.88333E-07	6.166E-07	3.716E-07	8.08267E-07
Vibrio_mediterranei	2.58564E-06	7.29091E-08	2.33091E-07	0.000000341	3.58538E-07	1.21662E-06	1.45823E-06	1.88138E-06	1.74455E-06	2.74727E-06	3.07455E-06	1.39918E-06	8.04933E-07	5.03933E-07	4.384E-07	1.43393E-06
Vibrio_gazogenes	2.35182E-07	1.46636E-07	6.45455E-07	3.70364E-07	3.95154E-07	3.68615E-07	2.28692E-07	7.51538E-08	4.99727E-07	4.48636E-07	4.01273E-07	4.43727E-07	2.83067E-07	6.872E-07	7.326E-07	3.18267E-07
Vibrio_tapetis	0	1.37273E-08	0	5.98818E-08	6.05923E-07	0	2.63077E-08	1.22615E-07	1.02091E-07	1.25182E-07	3.79091E-08	1.99455E-07	4.13533E-07	3.87733E-07	4.84773E-07	5.75933E-07
Vibrio_aquimaris	0	0	0	0	0	0	0	0	0	2.85455E-08	6.68182E-08	1.42727E-08	1.114E-07	3.48E-08	2.99333E-08	1.496E-07
Vibrio_metschnikovii	2.56364E-08	2.34545E-08	4.09818E-08	7.82091E-07	0	0	0	1.46923E-08	1.02636E-07	5.22727E-08	4.39636E-07	1.99818E-07	0	1.47667E-07	1.57067E-08	5.38667E-08
Vibrio_fluvialis	2.67364E-07	8.86364E-09	8.63091E-08	2.98636E-07	1.34846E-07	2.95385E-08	1.52077E-07	2.49385E-07	3.17545E-07	0.000000213	7.25273E-07	6.02909E-07	2.64E-08	0.000000119	3.37133E-07	4.04E-08
Vibrio_panuliri	0	0	0	4.79091E-09	0	1.26846E-07	0	5.48462E-08	0	0	0	0	0	1.00267E-07	4.44E-09	3.40667E-08
Vibrio_furnissii	1.23364E-07	0	1.70091E-08	2.64818E-07	2.11538E-07	5.19231E-08	0	4.83846E-07	1.12727E-07	0	3.95455E-08	2.37182E-07	2.67533E-07	3.65933E-07	3.30067E-07	4.934E-07
Vibrio_cincinnatiensis	0	0	0	0	0.000000065	0	0	0	8.20909E-08	1.02636E-07	2.76364E-08	1.87364E-07	2.99067E-07	2.402E-07	5.86707E-07	4.54333E-07
Vibrio_sp._THAF190c	1.45545E-07	0	3.70909E-08	1.03636E-07	3.15385E-08	0	0	0	0.000000054	9.22727E-08	2.06364E-07	3.74545E-08	2.72E-08	3.48E-08	0	0
Vibrio_sp._HDW18	1.30364E-07	0	0	5.18182E-09	0.000000054	0	0	0	4.86364E-08	0	9.99091E-08	0	9.92667E-08	1.044E-07	5.94E-08	6.81333E-08
Vibrio_sp._SM1977	3.59636E-07	0.000000065	1.23182E-07	1.12636E-07	6.04615E-07	0	0	0	0	8.85455E-08	3.95455E-08	8.98182E-08	3.17533E-07	3.39667E-07	6.784E-07	4.59667E-07
Vibrio_azureus	0	0	0.000000089	0	0	0	0	0	0	0	0	0	0	3.48E-08	0	0
Vibrio_parahaemolyticus	7.40909E-08	7.74545E-08	2.50218E-07	2.05518E-07	2.08154E-07	9.73846E-08	0	1.44846E-07	2.61545E-07	1.67273E-07	3.56818E-07	0.000000035	2.23333E-07	3.03133E-07	2.238E-07	1.15733E-07
Vibrio_campbellii	9.97909E-07	0.000000065	0.000000507	9.95273E-07	4.09769E-07	5.91538E-08	5.80769E-08	4.41692E-07	9.20727E-07	0.000000704	0.000001046	1.33509E-06	5.266E-07	5.03867E-07	5.43533E-07	8.63067E-07
Vibrio_cyclitrophicus	0.000000564	3.24545E-07	2.59818E-08	3.94273E-07	1.55385E-07	7.21538E-08	2.38692E-07	0.000000121	1.08545E-07	1.96182E-07	2.65818E-07	1.21727E-07	1.28867E-07	5.92133E-07	5.03507E-07	2.43133E-07
Vibrio_coralliilyticus	7.74545E-07	6.52727E-07	3.69409E-07	1.63273E-07	3.98615E-07	1.11438E-06	3.69154E-07	3.87769E-07	4.51091E-07	1.32545E-07	6.54727E-07	8.00182E-07	3.82333E-07	9.67267E-07	9.94533E-07	7.274E-07
Thioploca_ingrica	1.11273E-07	0	0	4.47E-08	2.45308E-07	0	0	4.89462E-07	7.73636E-08	0	0	9.03636E-08	1.27933E-07	6.15333E-08	2.64133E-07	3.71333E-07
Beggiatoa_leptomitoformis	2.20082E-06	0.000000065	2.32455E-07	0.000001148	1.02446E-06	1.25846E-07	1.31154E-07	1.64385E-06	1.31373E-06	9.20273E-07	3.23718E-06	2.32427E-06	8.426E-07	8.56867E-07	1.0356E-06	1.2064E-06
Thiosulfatimonas_sediminis	0	0	0	1.69818E-07	6.46077E-07	6.10769E-08	3.06923E-08	1.04223E-06	0.000000027	0.000000028	1.02727E-08	4.21364E-08	7.39267E-07	6.51467E-07	1.32793E-06	1.39473E-06
Methylophaga_frappieri	3.11818E-08	0	3.70909E-08	0	0	2.13846E-08	0	0	0	0	0	1.42727E-08	6.08E-08	0	0	0
Methylophaga_nitratireducenticrescens	3.79273E-07	0	0	1.03636E-08	1.54462E-07	2.95385E-08	2.63077E-08	1.81846E-07	4.22545E-07	0.000000062	3.36091E-07	4.65909E-08	7.82667E-08	1.02333E-07	2.95973E-07	2.84267E-07
Hydrogenovibrio_thermophilus	2.96364E-08	2.49818E-07	0	0	0	0	0	0	0	0	0	0	0	0	0	0
Hydrogenovibrio_marinus	2.12091E-07	1.04545E-08	0	2.43636E-08	3.32846E-07	6.39385E-07	2.03308E-07	9.79308E-07	9.37273E-08	7.73636E-08	1.73909E-07	1.12545E-07	2.16733E-07	5.45733E-07	4.68667E-07	4.43133E-07
Hydrogenovibrio_crunogenus	2.87909E-07	1.05273E-07	1.89582E-07	1.35545E-07	5.63846E-08	7.17077E-07	0.000000349	5.30077E-07	3.15818E-07	1.48273E-07	3.07273E-07	6.90909E-08	2.014E-07	3.778E-07	3.094E-07	2.558E-07
Piscirickettsia_salmonis	3.69091E-08	0	4.12182E-08	5.66364E-08	3.85462E-07	0	0	0	6.53636E-07	2.12273E-07	3.85091E-07	5.37273E-07	1.852E-07	4.422E-07	1.30667E-07	3.44533E-07
Thiomicrospira_sp._S5	7.68182E-08	1.37273E-08	0	6.64545E-08	1.57615E-07	6.05846E-07	4.66846E-07	6.50231E-07	2.87727E-07	1.31182E-07	1.97909E-07	0.000000289	1.95467E-07	0.000000231	6.32E-08	4.15667E-07
Thiomicrospira_aerophila	2.56364E-08	0	8.25455E-09	2.10727E-07	0	0	1.25154E-07	3.23692E-07	3.75455E-08	6.37273E-08	2.04091E-07	2.69727E-07	2.94667E-08	1.71333E-08	1.26667E-07	3.72E-08
Thiomicrospira_cyclica	4.63909E-07	0	2.22727E-07	1.33545E-07	2.86769E-07	7.54538E-07	7.38538E-07	4.80923E-07	2.07545E-07	1.47455E-07	8.77273E-08	6.26273E-07	3.45933E-07	3.97533E-07	6.336E-07	4.836E-07
Cycloclasticus_sp._PY97N	0	0.00000039	0	0	0	0	0	0	0	0	1.35636E-07	7.89091E-08	0	0	0	0
Cycloclasticus_sp._P1	0	0	0.000000025	0	0	0	0	0	0	0	0	0	0	0	0	0
Thiosulfativibrio_zosterae	3.84091E-07	0.000000065	1.12182E-07	7.32727E-07	1.01377E-06	7.13385E-07	4.18154E-07	1.24423E-06	4.23636E-07	2.20545E-07	4.76091E-07	7.36182E-07	8.868E-07	1.08727E-06	1.2808E-06	2.78007E-06
Thiomicrorhabdus_aquaedulcis	2.53182E-07	2.71818E-08	4.12727E-09	1.80064E-07	4.11538E-08	2.52308E-08	6.41538E-08	5.81538E-08	5.97273E-08	2.43182E-07	2.76364E-08	1.42909E-07	0	0	0	0
Thiomicrorhabdus_indica	9.23455E-07	6.18636E-07	4.57091E-07	0.000000751	4.09846E-07	0.000000345	2.98077E-07	2.47769E-07	7.35364E-07	1.89455E-07	7.51818E-07	9.27636E-07	3.66467E-07	2.90533E-07	3.77733E-07	1.93333E-07
Thiomicrorhabdus_sp._G1	4.04909E-07	9.86364E-08	2.78218E-07	4.63909E-07	2.73846E-07	6.44692E-07	3.84385E-07	3.86923E-07	8.67273E-08	1.53727E-07	2.70091E-07	2.71182E-07	2.81867E-07	2.136E-07	2.65133E-07	3.068E-07
Thiomicrorhabdus_sp._G2	0.000001779	0.000000065	4.25073E-07	1.59191E-06	4.82231E-07	0	0.000000233	4.07923E-07	8.23182E-07	8.70636E-07	1.23745E-06	2.09445E-06	4.07867E-07	5.71667E-07	5.994E-07	1.3646E-06
Allofrancisella_guangzhouensis	0	0	0	0	0	0	0	0	0	0	0	7.13636E-09	0	0	0	0
Allofrancisella_frigidaquae	2.81818E-07	4.49091E-07	6.20091E-07	2.36245E-07	8.33154E-07	0	1.82615E-07	1.13154E-07	7.08273E-07	8.63182E-07	4.59909E-07	1.46818E-06	5.46733E-07	9.294E-07	7.96267E-07	7.53867E-07
Francisella_salina	0	0	0	0	0	0	0	0	0	0	0	7.13636E-09	0	0	0	0
Francisella_orientalis	0.000000104	0	0	4.79091E-09	3.34615E-08	0	0	0	5.97273E-08	0	0	0.000000057	0	0	0	6.81333E-08
Francisella_noatunensis	2.96364E-08	0	0	5.18182E-09	0	0	6.41538E-08	3.22308E-08	5.79091E-08	0.000000034	1.30909E-07	8.29091E-08	0	0	1.65333E-08	0
Francisella_sp._LA112445	3.11818E-08	0	0	9.72727E-08	1.13846E-07	0.000000026	1.64231E-07	4.23923E-07	1.42727E-07	0.000000081	8.93636E-08	4.45909E-08	0	1.61333E-08	0	9.33333E-09
Francisella_halioticida	4.33636E-07	3.33818E-07	1.71182E-07	2.24818E-07	1.07692E-07	2.95385E-08	1.53462E-07	4.39231E-08	3.98182E-08	0.00000014	0.000000283	1.67091E-07	2.14E-08	3.32667E-08	3.042E-07	3.484E-07
Francisella_uliginis	4.56364E-08	0	0	0	0.000000039	0	2.63077E-08	8.28462E-08	5.97273E-08	0	1.97455E-07	6.93636E-08	2.08E-08	8.44667E-08	0	0
Francisella_philomiragia	1.58182E-08	5.27727E-08	2.96364E-08	0	1.95231E-07	3.83077E-08	4.33846E-08	6.05385E-08	0	7.29091E-08	0	7.13636E-09	1.34067E-07	2.55333E-07	2.62933E-07	6.62E-08
Francisella_tularensis	3.71364E-07	0.000000065	2.04909E-08	4.88545E-07	2.62462E-07	0	8.82615E-07	8.35846E-07	1.40518E-06	4.67182E-07	0.000001254	5.935E-07	5.63267E-07	1.70267E-07	5.22933E-07	4.36467E-07
Francisella_marina	2.98636E-07	1.94545E-07	7.40909E-08	1.60273E-07	2.53692E-07	0	1.18077E-07	5.04846E-07	3.25364E-07	3.45455E-07	6.68182E-08	1.46727E-07	0.000000395	4.128E-07	3.2924E-07	3.99333E-07
Francisella_persica	2.35555E-06	9.36364E-09	1.63273E-07	1.62273E-07	4.41231E-07	1.89923E-07	1.58608E-06	1.64969E-06	1.57645E-06	3.27345E-06	3.30682E-06	1.35109E-06	7.08667E-07	2.67467E-07	3.554E-07	7.13867E-07
Francisella_hispaniensis	0	1.15364E-07	0	1.43636E-08	0	0	2.25231E-07	2.46923E-08	5.45455E-08	0	1.02727E-08	7.13636E-09	7.17333E-08	1.18667E-07	1.4244E-07	9.92667E-08
Francisella_frigiditurris	0.000000089	0.00000013	1.63636E-08	2.87273E-08	1.72154E-07	0	6.23077E-08	2.46923E-08	0.000000217	2.02091E-07	4.16636E-07	1.92818E-07	2.01867E-07	7.48467E-07	6.456E-07	2.05267E-07
Francisella_sp._FSC1006	5.57273E-08	0	7.97273E-08	5.76364E-08	7.24615E-08	6.94538E-07	2.25462E-07	5.01846E-07	1.20818E-07	1.44091E-07	1.70909E-07	7.82273E-08	1.414E-07	2.80533E-07	4.72333E-07	2.81667E-07
Francisella_sp._Scap27	3.62727E-08	3.24545E-07	0	3.24545E-08	2.56569E-06	6.76923E-08	0	1.06123E-06	9.31818E-07	6.72727E-07	2.06818E-07	3.80727E-07	1.36207E-06	1.63887E-06	1.65033E-06	2.10853E-06
Ahniella_affigens	1.01545E-07	1.35455E-07	0	5.18182E-09	0	8.66923E-08	0	0	0	0.000000028	5.52727E-08	0.000000057	0	0	0	0
Frateuria_aurantia	0.00000006	9.23636E-08	0	2.43636E-08	3.34615E-08	4.76923E-08	5.74615E-08	2.93846E-08	4.45455E-08	3.03636E-08	0.000000061	0	2.24E-08	5.22E-08	1.12667E-08	1.902E-07
Dokdonella_koreensis	0.000001145	1.67545E-07	3.27273E-08	2.007E-07	3.69231E-07	4.30154E-07	7.13077E-07	1.64146E-06	4.53545E-07	4.37091E-07	9.08273E-07	1.12809E-06	8.27733E-07	6.41267E-07	5.99467E-07	5.23067E-07
Dyella_japonica	3.69091E-08	0	0	1.25327E-06	4.52385E-07	1.66846E-06	1.12092E-06	8.55615E-07	0	0	0	3.05455E-07	5.58267E-07	2.94267E-07	3.31333E-07	3.59333E-07
Dyella_thiooxydans	1.48636E-07	0	0	2.41545E-08	0	3.98462E-07	6.23077E-08	2.51538E-07	4.86364E-08	0	9.96364E-08	0.000000035	4.48667E-08	0	2.99733E-08	0
Dyella_sp._M7H15-1	0.000000103	1.37273E-08	2.01818E-07	7.15636E-07	3.64538E-07	1.09315E-06	6.45077E-07	0.000001103	3.27636E-07	5.48636E-07	5.53364E-07	8.71364E-07	3.89667E-07	2.454E-07	2.85333E-07	0.00000061
Dyella_sp._G9	5.49818E-07	1.16364E-08	6.33636E-08	2.39545E-07	0.000000267	9.46154E-09	2.31385E-07	5.43769E-07	4.30818E-07	4.63455E-07	4.64273E-07	7.57091E-07	3.75267E-07	2.592E-07	4.26133E-07	0.000000287
Luteibacter_rhizovicinus	0	0	0	0	3.58462E-08	0	0	1.90769E-08	0	0	0	6.01818E-08	0	4.64667E-08	1.49333E-08	0
Luteibacter_pinisoli	0	2.96818E-08	5.81636E-08	2.74364E-07	3.15385E-08	6.53077E-08	2.44077E-07	2.40308E-07	0	9.92727E-08	0	1.41273E-07	1.88133E-07	1.16933E-07	4.04667E-08	4.95333E-08
Rhodanobacter_glycinis	2.56273E-07	0	0	3.24545E-08	1.05769E-07	0	2.23154E-07	1.25308E-07	3.80727E-07	2.09636E-07	1.35455E-07	1.25545E-07	9.70667E-08	1.71133E-07	2.83733E-08	8.19333E-08
Rhodanobacter_denitrificans	3.05064E-06	0.000000784	2.48818E-06	1.79982E-06	1.63077E-07	1.71385E-07	1.83923E-06	2.63115E-06	6.30545E-07	7.82636E-07	1.40336E-06	1.75282E-06	2.378E-07	4.61333E-07	2.664E-07	0.000000268
Aerosticca_soli	1.40636E-06	1.45091E-07	3.70909E-08	1.54727E-07	1.36954E-06	0	1.40615E-07	2.11769E-07	0.00000101	1.55582E-06	7.92455E-07	1.01591E-06	2.11913E-06	1.56227E-06	1.43027E-06	1.9924E-06
Pseudolysobacter_antarcticus	0.000001104	2.34545E-08	5.80364E-07	8.40545E-07	8.43846E-08	4.07769E-07	3.93692E-07	3.06615E-07	0.00000061	2.12182E-07	6.27182E-07	2.80364E-07	2.19133E-07	1.756E-07	1.95867E-07	1.84867E-07
Arenimonas_daejeonensis	4.70909E-07	9.86364E-08	1.46064E-07	1.52909E-06	2.18308E-07	2.27838E-06	4.54031E-06	2.82769E-06	2.40818E-07	5.70273E-07	5.60273E-07	9.00455E-07	4.06333E-07	3.68733E-07	3.702E-07	3.298E-07
Luteimonas_sp._YGD11-2	0	0	0	5.18182E-09	0	2.95385E-08	0	0	0	0	0	0	0	0	0	0
Luteimonas_sp._MC1825	1.74273E-07	3.23273E-07	2.17673E-07	3.63273E-07	0	0	2.73846E-08	0	6.62727E-08	0	7.29091E-08	4.21364E-08	0	0	5.88E-08	0
Luteimonas_yindakuii	3.69091E-08	0	3.69818E-08	5.83636E-08	7.23077E-08	0	0	1.27077E-07	8.78182E-08	9.22727E-08	0	7.13636E-09	4.30667E-08	8.62667E-08	8.86667E-09	2.202E-07
Luteimonas_sp._JM171	0	0	8.31818E-09	0	2.05385E-08	0	0	2.56923E-08	0	0	0	8.80909E-08	5.02E-08	0	0.000000084	8.67333E-08
Luteimonas_chenhongjianii	5.25455E-07	1.21273E-07	6.69182E-07	6.51545E-07	8.53077E-08	8.30769E-08	0	1.28538E-07	1.55909E-07	1.70182E-07	4.89364E-07	1.92182E-07	1.15467E-07	1.634E-07	2.00133E-07	3.38733E-07

Luteimonas_granuli	4.24182E-07	2.80682E-07	6.17091E-08	1.70091E-07	0.000000039	3.16769E-07	1.32769E-07	2.50385E-07	9.85455E-08	5.22727E-08	3.33636E-08	3.69273E-07	4.88667E-08	6.59333E-08	4.44E-09	1.22133E-07
Lysobacter_sp._H23M41	0	0	0	5.16364E-08	0	0	4.33846E-08	0	0	0	0	0	4.38667E-08	3.29333E-08	7.59333E-08	3.84667E-08
Lysobacter_oculi	0.00000006	0	3.70909E-08	1.93636E-08	3.15385E-08	2.72769E-07	1.10385E-07	1.20231E-07	2.16364E-08	3.62727E-08	0	9.75E-08	0	0.000000033	2.62E-08	9.33333E-09
Lysobacter_maris	1.58182E-08	0	0	6.63636E-08	3.15385E-08	0	0	0	0	0	3.95455E-08	8.87273E-08	0	9.26667E-08	2.99333E-08	1.27667E-07
Lysobacter_lycopersici	1.43273E-07	0	3.70909E-08	4.79091E-09	2.05385E-08	0	0	0	4.45455E-08	0	0.000000117	2.85455E-08	2.14E-08	3.26667E-08	1.99267E-07	4.28E-08
Lysobacter_alkalisoli	1.86364E-07	0	3.28818E-07	1.91818E-08	1.00846E-07	1.77692E-07	2.19538E-07	4.93846E-08	8.51818E-08	2.85455E-08	3.33636E-08	6.32727E-08	2.64667E-08	4.91333E-08	0.000000159	1.50933E-07
Lysobacter_sp._TY2-98	1.30545E-07	0	0	4.79091E-09	0	0	0	0.000000042	0	0	1.35455E-07	8.26818E-08	1.86667E-08	5.08667E-08	1.37533E-07	1.22E-08
Lysobacter_sp._H21R20	1.07536E-06	0	2.96364E-08	5.70455E-07	0.000000061	2.95385E-08	9.88462E-08	5.25462E-07	6.60727E-07	4.28727E-07	9.04636E-07	5.80545E-07	2.842E-07	2.25133E-07	2.36133E-07	3.18067E-07
Lysobacter_sp._H23M47	3.89818E-07	4.69909E-07	4.73636E-08	2.42455E-07	0	2.13846E-08	0	7.40769E-08	7.97273E-08	1.04545E-07	1.02727E-08	2.09545E-07	0	0	0	0
Lysobacter_antibioticus	9.04545E-08	0.000000065	1.06945E-07	5.50909E-08	3.58462E-08	2.62615E-07	0	6.36308E-07	5.37273E-08	0	1.82091E-07	3.57273E-08	0	0.00000018	2.78E-08	0.000000083
Lysobacter_sp._H21R4	2.79091E-07	0	4.12727E-09	4.20909E-08	1.52538E-07	0	3.51538E-07	3.16462E-07	2.74364E-07	1.90182E-07	1.37273E-07	1.68727E-07	3.25333E-07	4.61267E-07	3.08533E-07	4.92733E-07
Lysobacter_sp._II4	5.12727E-08	0.000000065	4.12727E-09	3.47273E-08	0.000000054	0	2.63077E-08	0	1.37636E-07	3.89091E-08	1.30909E-07	4.02909E-07	0	7.76E-08	0	5.88667E-08
Lysobacter_capsici	4.93455E-07	0	1.95082E-06	2.19109E-06	5.10692E-07	0	4.26538E-06	5.14154E-06	9.21545E-07	4.23364E-07	9.98727E-07	1.19509E-06	4.712E-07	3.82933E-07	1.18911E-06	1.608E-07
Lysobacter_soli	0.000003823	7.23727E-07	3.66136E-06	0.000001935	4.36692E-07	2.58846E-07	3.33931E-06	4.01346E-06	1.45445E-06	1.34236E-06	3.15273E-06	2.02209E-06	3.186E-07	1.14687E-06	8.39467E-07	7.77133E-07
Lysobacter_sp._CW239	0.000000108	0	6.60909E-08	7.86091E-08	1.34077E-07	3.02385E-07	1.07538E-07	1.06923E-07	2.29455E-07	9.11818E-08	1.45909E-07	2.57091E-07	8.45333E-08	0.000000014	1.926E-07	9.25333E-08
Lysobacter_sp._HDW10	2.72636E-07	0	2.23909E-07	1.17255E-06	1.80692E-07	0	0	4.93769E-07	5.95455E-07	0.000000812	7.43636E-07	1.58727E-06	5.37267E-07	6.82133E-07	0.00000098	0.000001088
Lysobacter_gummosus	1.66636E-07	6.21273E-07	6.15818E-08	2.57182E-07	3.88462E-08	0	1.07538E-07	5.42308E-08	3.98182E-08	9.36364E-08	1.35545E-07	2.85182E-07	2.37333E-08	1.304E-07	2.38E-08	6.96667E-08
Lysobacter_enzymogenes	2.56364E-08	0	1.24455E-08	3.76364E-08	8.36154E-08	0	0	2.71154E-07	1.29636E-07	8.08182E-08	0	1.89364E-07	1.86667E-08	1.628E-07	5.084E-08	1.29333E-07
Thermomonas_carbonis	0	0	0	0	2.05385E-08	0	0	0	5.46364E-08	5.22727E-08	1.38273E-07	1.18909E-07	4.58E-08	0.000000033	2.39333E-08	0.000000182
Thermomonas_sp._XSG	0	0	0	5.16364E-08	3.15385E-08	0	0	0	0.000000064	0.000000034	0.000000061	9.85455E-08	8.62667E-08	0.000000066	5.69333E-08	5.58667E-08
Thermomonas_sp._HDW16	3.23364E-07	2.32727E-08	0	3.65273E-07	0.00000027	1.79923E-07	5.85385E-08	3.57692E-08	4.23818E-07	6.37818E-07	4.35091E-07	1.95636E-07	1.56133E-07	0.000000014	1.03573E-07	1.45333E-07
Thermomonas_sp._SY21	4.98182E-07	5.18091E-07	2.03164E-07	4.54273E-07	0	0	0	8.28462E-08	1.72091E-07	0	8.02727E-08	4.61364E-07	2.72E-08	0	0	0
Thermomonas_brevis	9.81818E-07	0	0	2.277E-07	1.18815E-06	6.53077E-08	0	1.14846E-07	1.36109E-06	1.07509E-06	9.12364E-07	8.70636E-07	2.0216E-06	1.90593E-06	1.5296E-06	2.24133E-06
Xylella_taiwanensis	3.69091E-08	1.82045E-07	1.63636E-08	1.18636E-07	7.16923E-08	0	1.47769E-07	1.79769E-07	1.37091E-07	5.22727E-08	2.61818E-07	8.02727E-08	4.38667E-08	1.38267E-07	4.44E-09	0
Xylella_fastidiosa	1.62127E-06	1.03373E-06	1.86909E-07	7.77182E-07	0.000001543	9.14923E-07	8.03615E-07	7.85923E-07	0.000001045	1.38718E-06	1.60073E-06	2.81636E-06	6.10333E-07	1.28127E-06	1.1404E-06	8.69333E-07
Pseudoxanthomonas_mexicana	1.76791E-06	2.56636E-06	1.14855E-06	2.39809E-06	0.000000777	3.50692E-07	6.18769E-07	1.35862E-06	1.49845E-06	1.73427E-06	1.71955E-06	2.58582E-06	9.11333E-07	6.97867E-07	6.79867E-07	1.25127E-06
Pseudoxanthomonas_spadix	0.000001301	0.000000065	8.68891E-07	4.34364E-07	3.06538E-07	4.49231E-07	5.84154E-07	1.68054E-06	0.000000693	5.22364E-07	1.18655E-06	3.42636E-07	3.82067E-07	4.24467E-07	0.000000996	7.16667E-07
Pseudoxanthomonas_suwonensis	1.39055E-06	7.45455E-08	2.21694E-06	4.13182E-06	1.27654E-06	1.77462E-06	9.60769E-06	7.21231E-06	2.16364E-06	1.30982E-06	1.31427E-06	1.22336E-06	1.0832E-06	1.40387E-06	1.97067E-06	8.29533E-07
Stenotrophomonas_sp._ZAC14A_NAIMI4_1	0	0	0	0	0	0	0	0	0	0	0	0	2.64667E-08	0	0	0
Stenotrophomonas_sp._ZAC14D2_NAIMI4_7	0	0	0	0	0	1.44615E-07	0	0	0	0	0	0	0	0	0	4.95333E-08
Stenotrophomonas_sp._SAU14A_NAIMI4_8	0	0	0	0	0	0	0	0	2.64545E-08	0	0	7.13636E-09	0	0	0	0
Stenotrophomonas_sp._169	0	4.79091E-08	0	4.79091E-09	0	6.44615E-08	2.21538E-07	0	0	0	0	0	0	0.000000014	5.94667E-08	0
Stenotrophomonas_sp._NA06056	0	0	0	0	0	0	3.06923E-08	0	0	0.000000034	0	0	0	0	0	0
Stenotrophomonas_acidaminiphila	0	0	0	0	0	1.21385E-07	0	0	0	0	0	0	4.74E-08	4.33333E-08	3.59067E-08	1.84333E-07
Stenotrophomonas_sp._YAU14D1_LEIMI4_1	0	0	0	0	0	0	0	0	0	0	0	0	2.37333E-08	1.58E-08	9.364E-08	0
Stenotrophomonas_sp._ESTM1D_MKCIP4_1	0	0	0	0	0	4.33846E-08	0	0	0	0	0	9.03636E-08	0	0	6.44E-08	1.98667E-08
Stenotrophomonas_sp._YAU14A_MKIMI4_1	0	0	0	0	0	0	2.63077E-08	0	0	0	0	0	0	0	0	0
Stenotrophomonas_sp._G4	1.73727E-07	0	0	4.20909E-08	3.34615E-08	0	1.91231E-07	5.22154E-07	4.45455E-08	0	9.41818E-08	0.00000007	8.31333E-08	1.16467E-07	5.94667E-08	5.96667E-08
Stenotrophomonas_indicatrix	0	0	0	0	0	8.30769E-08	0	0	0	0	3.33636E-08	0	0	0	0	0
Stenotrophomonas_sp._Pemsol	0	0	0	0	0	0	0	0	0	0	3.33636E-08	0	0	0	0	0
Stenotrophomonas_sp._WZN-1	0	0	5.34545E-08	0	5.20769E-08	5.91538E-08	2.63077E-08	0.000000022	2.75455E-08	5.83636E-08	1.46909E-07	1.42182E-07	2.91333E-08	4.51333E-08	8.86667E-09	0.000000086
Stenotrophomonas_sp._MYb57	0	0	0	0	0	4.13846E-08	0	0	0	0	0.0000001	0	0	0	0	0
Stenotrophomonas_sp._364	1.06636E-07	1.94545E-07	5.94455E-07	7.26364E-08	1.86923E-08	2.95385E-08	4.15385E-07	7.40769E-08	2.72727E-08	5.70909E-08	7.23636E-08	2.285E-07	2.72E-08	0.000000014	6.46667E-08	0
Stenotrophomonas_sp._SAU14A_NAIMI4_5	0.000000047	4.19455E-07	9.52182E-08	5.36364E-08	0	1.35092E-06	0.000000057	0	1.88909E-07	2.87273E-08	0	1.58364E-07	0.000000053	0	0	0
Stenotrophomonas_maltophilia	2.08091E-07	0.000000256	2.45309E-07	3.46273E-07	3.64385E-07	1.77669E-06	5.32923E-07	6.59385E-07	1.64273E-07	7.73636E-08	0.000000383	2.98091E-07	2.87267E-07	3.398E-07	3.46933E-07	5.22867E-07
Stenotrophomonas_sp._LM091	3.62727E-08	9.54545E-09	8.31818E-09	0	0	4.97846E-07	4.80769E-08	3.57692E-08	7.50909E-08	0	1.02727E-08	0	0	1.39333E-07	5.24E-08	4.04E-08
Stenotrophomonas_rhizophila	6.60909E-08	1.00727E-07	0	9.97273E-09	0.000000061	2.03769E-07	1.92462E-07	0.000000011	9.23636E-08	0	1.71545E-07	4.92727E-08	0	2.20133E-07	2.27573E-07	1.32533E-07
Stenotrophomonas_sp._ZAC14D2_NAIMI4_6	8.12636E-06	7.14545E-07	0.00000167	7.74364E-06	2.51485E-06	0	5.94462E-07	3.31385E-06	5.33273E-06	0.00000426	3.81273E-06	4.92091E-06	4.63867E-06	5.0164E-06	3.04873E-06	3.14333E-06
Xanthomonas_sp._SI	0	2.70909E-08	0	0	0	0	0	0	0	0	0	0	0	0	0	0
Xanthomonas_albilineans	0	0	0	0	0	0	0	0	0	0	0	0.000000035	0	0	0	0
Xanthomonas_euroxanthea	0	0	0	0	0	6.30769E-08	0	0	0	0	0	0	0	0	4.44E-09	0
Xanthomonas_axonopodis	0	0	0	0	0	0	0	0	0	0	0	1.21045E-07	0	0	8.86667E-09	4.95333E-08
Xanthomonas_hyacinthi	0	0.000000135	2.87273E-08	5.18182E-09	1.13538E-07	0	1.14462E-07	0	0	0.000000028	0	9.03636E-08	0	0	0	1.98667E-08
Xanthomonas_vasicola	3.11818E-08	0	0	9.97273E-09	0	0	3.33077E-08	3.93846E-08	2.16364E-08	0	0	0	0	0	0	6.80667E-08
Xanthomonas_sp._SS	0	0	0	1.16091E-07	5.21538E-08	0	0	3.00769E-08	1.10909E-07	5.22727E-08	4.81818E-08	0.000000057	0	0	4.44E-09	3.40667E-08
Xanthomonas_cassavae	1.01182E-07	0.000000177	6.67273E-08	2.93273E-07	4.11538E-08	0	2.63077E-08	9.03846E-08	1.36727E-07	6.37273E-08	2.76364E-08	0.000000068	2.44E-08	6.36E-08	0	7.70667E-08
Xanthomonas_cucurbitae	1.58182E-08	5.41818E-08	0.000000047	2.41545E-08	0	0	0	0	2.75455E-08	0	0	3.87727E-08	0	4.84667E-08	5.88E-08	0
Xanthomonas_arboricola	3.46364E-07	0	1.27273E-07	7.83364E-08	3.15385E-08	1.45077E-07	1.21615E-07	1.24615E-07	2.12273E-07	5.22727E-08	2.49091E-07	4.81727E-07	7.44667E-08	3.50667E-08	4.48E-08	1.66667E-08
Xanthomonas_fragariae	2.96364E-08	8.86364E-09	0	3.39091E-08	0	0	5.36923E-08	1.57385E-07	5.79091E-08	5.22727E-08	3.33636E-08	4.79091E-08	2.94667E-08	0	0	0
Xanthomonas_sp._GW	1.09545E-07	1.77273E-08	2.96364E-08	0	0	0	0	0	0	5.22727E-08	3.33636E-08	2.95455E-07	2.09333E-08	0	0	0
Xanthomonas_sacchari	0.000000551	0	3.70909E-08	4.27545E-07	3.30538E-07	5.75385E-08	3.77692E-08	2.00769E-07	4.10636E-07	5.20818E-07	0.000000189	6.88273E-07	4.40867E-07	2.94667E-07	0.000000218	0.000000116
Xanthomonas_vesicatoria	1.41273E-07	0	0	0	2.44846E-07	0	0	0	2.11273E-07	2.64545E-07	1.00627E-06	3.07818E-07	6.60667E-08	2.736E-07	7.42667E-08	2.20267E-07
Xanthomonas_citri	1.25973E-06	0.000000065	0	5.07909E-07	4.00923E-07	0	9.15385E-08	2.55846E-07	0.000000113	6.40455E-07	2.48455E-07	2.60182E-07	3.662E-07	0.000000124	4.42467E-07	0.000000102
Xanthomonas_translucens	5.57909E-07	3.27141E-06	1.89582E-07	3.43818E-07	5.61385E-07	5.25385E-07	1.31538E-07	9.30769E-08	1.34545E-06	0	0.0000001	1.83091E-07	3.35533E-07	2.098E-07	1.5084E-07	1.57667E-07
Xanthomonas_campestris	8.22545E-07	4.89864E-07	2.38818E-07	0.00000057	4.86692E-07	9.20769E-07	4.10385E-07	2.19154E-07	6.34364E-07	5.55727E-07	4.02364E-07	1.35064E-06	2.55733E-07	1.822E-07	9.56667E-08	7.82667E-08
Xanthomonas_phaseoli	3.62727E-08	0	0	4.79091E-09	0	0	6.66154E-08	0	0	0	0	0	1.69467E-07	1.00067E-07	0	0
Xanthomonas_oryzae	1.11218E-06	1.04545E-08	3.43455E-07	7.64545E-07	2.77077E-07	1.22385E-07	7.92615E-07	1.33485E-06	5.26909E-07	4.65636E-07	0.00000082	5.33636E-07	4.502E-07	4.42867E-07	5.54333E-07	1.03433E-06
Xanthomonas_hortorum	9.06091E-07	7.43636E-08	4.32673E-07	1.49227E-06	7.22923E-07	1.16615E-07	2.05462E-07	2.55154E-07	5.21364E-07	6.54091E-07	8.22182E-07	8.17909E-07	2.854E-07	5.146E-07	2.64933E-07	7.226E-07
Xanthomonas_euvesicatoria	1.82582E-06	0.000000977	8.55391E-07	0.000002406	1.74415E-06	8.13231E-07	1.48869E-06	2.06231E-06	1.58518E-06	1.37445E-06	2.81091E-06	3.51218E-06	1.40247E-06	0.000001434	2.27553E-06	2.42767E-06
Moritella_viscosa	0	0	0	8.08182E-08	0	0	6.23077E-08	1.90769E-08	0.000000027	0	6.68182E-08	7.55455E-08	6.08E-08	3.50667E-08	5.04667E-08	1.66667E-08
Moritella_marina	8.25455E-08	2.70909E-08	1.63636E-08	8.91818E-08	2.59231E-08	0	2.63077E-08	5.81538E-08	0.000000054	0	0	1.42727E-08	1.64933E-07	0.000000033	5.88E-08	1.35333E-07
Moritella_yayanosii	0.00000006	0	0	0	7.72308E-08	0	4.80769E-08	1.46923E-08	2.37909E-07	0.000000034	4.92727E-08	3.16364E-08	2.138E-07	0	8.76667E-08	1.802E-07
Ferrimonas_sp._S7	0	1.68182E-08	3.70909E-08	0	3.88462E-08	0	0	0	5.79091E-08	0	0	0	0	0	0	9.33333E-09
Ferrimonas_balearica	1.54636E-07	2.24818E-07	2.81636E-07	6.74545E-08	1.65154E-07	4.49462E-07	4.01385E-07	9.32308E-08	1.88818E-07	1.34091E-07	0.000000489	3.67727E-07	1.196E-07	1.05867E-07	8.84667E-08	3.67733E-07
Idiomarina_piscisalsi	3.62727E-08	0	0	0	0	0	0	0	3.94545E-08	0	3.95455E-08	0	0	0	1.12667E-08	1.98667E-08
Idiomarina_andamanensis	2.96364E-08	0	0	1.83727E-07	2.01385E-07	0	0	1.86077E-07	1.02636E-07	2.80182E-07	1.83727E-07	3.87727E-08	2.96133E-07	3.15867E-07	2.654E-07	3.45733E-07
Idiomarina_sp._X4	0	0	1.77273E-08	0	0	0	0	0	0	0	0	0	0	0	0	1.86E-08
Idiomarina_sp._OT37-5b	0	0	4.12727E-09	3.24545E-08	0	0	0	0	0	3.89091E-08	0	0	0	0	0	0
Idiomarina_loihiensis	2.32091E-07	0	0	2.39091E-08	2.89615E-07	1.62154E-06	3.79308E-07	8.29923E-07	2.22182E-07	1.64909E-07	2.75545E-07	4.05773E-07	1.694E-07	2.21667E-07	0.000000405	6.04933E-07
Psychromonas_ingrahamii	0	0	0	0	0	0	6.41538E-08	0	0	0.000000056	8.02727E-08	1.42727E-08	0	9.15333E-08	0	2.88667E-08
Psychromonas_sp._CNPT3	1.99409E-06	9.27273E-08	1.27545E-07	1.08909E-07	0.000000473	1.42462E-07	2.02954E-06	2.35292E-06	1.81836E-06	2.87718E-06	1.67536E-06	1.45309E-06	6.80733E-07	4.73933E-07	8.02133E-07	1.05527E-06
Pseudoalteromonas_marina	0	8.77273E-08	0	0	0	0	0	0	0	0	0	0	0	0	0	0
Pseudoalteromonas_shioyasakiensis	0	0	0	0	0	0	0	0	0	0	0	0	2.08E-08	0	0	0
Pseudoalteromonas_arabiensis	0	0	0	0	0	0	0	0	0	0	0	3.16364E-08	0	0	0	0
Pseudoalteromonas_sp._DL-6	0	0	0	1.43636E-08	0	0	0	0	0	0	0	0	0	0	0	0
Pseudoalteromonas_sp._13-15	0	1.94545E-07	0	2.34364E-07	0	0	0	0	3.98182E-08	0	0	9.64545E-08	5.64667E-08	0	0	0
Pseudoalteromonas_atlantica	0	0	0	5.18182E-09	3.88462E-08	3.83077E-08	0	0	0	0	0	0	5.32E-08	0	4.44E-09	0
Pseudoalteromonas_paragorgicola	0	0	0	0	1.07692E-07	1.60308E-07	0	0	0	0	0	0	7.50667E-08	1.01733E-07	3.74E-08	0
Pseudoalteromonas_sp._1_2015MBL_MicDiv	0	0	0	0	0	0	0	0	0	6.08182E-08	1.02727E-08	0	0	0	0	0
Pseudoalteromonas_spongiae	0	0	0	6.14545E-08	3.58462E-08	0	0	1.56923E-07	0	0	2.76364E-08	2.00864E-07	3.176E-07	6.25533E-07	3.65573E-07	4.97333E-08
Pseudoalteromonas_sp._THAF3	0	0	0	4.88818E-08	0	0	0	0	0	0	3.77273E-08	1.66591E-07	0	0	0	0
Pseudoalteromonas_piscicida	0	0	0	1.63518E-07	0	0	0	0	0	1.11818E-07	2.44273E-07	4.72909E-07	2.64E-08	9.68667E-08	2.09733E-08	0.000000074
Pseudoalteromonas_piratica	2.70909E-08	0	0	0	0	2.95385E-08	0	0	0.000000087	0	6.71818E-08	0	0	0	1.49333E-08	3.67333E-08
Pseudoalteromonas_issachenkonii	0	0	0	0	0	0	6.66154E-08	7.42308E-08	0	5.22727E-08	7.90909E-08	1.05455E-07	0	0	5.94E-08	8.49333E-08
Pseudoalteromonas_sp._A25	1.90455E-07	0	0	2.49064E-07	4.16769E-07	0	8.69231E-08	5.90923E-07	8.16727E-07	2.53727E-07	4.41818E-07	2.32182E-07	5.39467E-07	2.78733E-07	3.59467E-07	4.71133E-07
Pseudoalteromonas_aliena	0	0	0	0	1.42938E-06	1.58231E-07	3.89692E-07	6.55846E-07	0	1.95455E-07	3.43818E-07	2.77182E-06	0	0	0	0
Pseudoalteromonas_carrageenovora	0	2.34545E-08	1.63636E-08	0	0	0	7.54615E-08	0.000000022	7.73636E-08	0	5.52727E-08	8.26818E-08	0	0	0	0
Pseudoalteromonas_espejiana	0	0	2.47273E-08	0	0	2.95385E-08	1.06615E-07	2.47692E-08	0	0	0	0	2.72E-08	0.000000033	3.37333E-08	1.86E-08
Pseudoalteromonas_arctica	1.58182E-08	0	0	0	1.34769E-07	0	0	1.90769E-08	0	0	0	5.70909E-08	4.99333E-08	0	1.49333E-08	1.86E-08
Pseudoalteromonas_tunicata	1.58455E-07	0.00000013	0	6.52455E-08	1.69846E-07	0	3.77692E-08	3.56923E-08	0.000000064	6.69091E-08	9.96364E-08	4.28182E-08	0	9.98667E-08	8.86667E-09	9.33333E-09
Pseudoalteromonas_agarivorans	1.77364E-07	0	1.48182E-07	7.31818E-08	2.05385E-08	0.000000178	8.61538E-08	1.30692E-07	1.06364E-07	1.09091E-07	5.52727E-08	1.81909E-07	1.228E-07	5.22E-08	1.58107E-07	2.506E-07
Pseudoalteromonas_sp._R3	7.99091E-08	0	0	1.44364E-07	2.92923E-07	0.000000136	7.45385E-08	0.000000286	0.000000404	3.53636E-07	5.52545E-07	0.000000167	2.314E-07	1.19467E-07	5.66E-08	3.22267E-07
Pseudoalteromonas_rubra	2.03818E-07	0	6.58182E-08	1.69909E-07	1.30154E-07	0	1.42385E-07	1.99385E-07	2.34091E-07	9.56364E-08	4.47091E-07	5.12864E-07	6.59333E-08	4.94E-08	9.68667E-08	2.82867E-07
Pseudoalteromonas_lipolytica	0	1.16364E-08	2.62727E-08	5.18182E-09	9.64615E-08	3.70769E-07	8.11538E-08	1.87462E-07	9.93636E-08	1.79091E-07	2.36182E-07	1.41364E-07	2.89067E-07	2.264E-07	1.442E-07	3.29533E-07
Pseudoalteromonas_phenolica	3.11818E-08	0	4.12727E-09	0	0	0	0	1.46923E-08	0	0	0	7.13636E-09	0	0	0	0
Pseudoalteromonas_nigrifaciens	0	0	4.12727E-09	5.18182E-09	0	0	2.73846E-08	0	0	0	0	0	0	0	0	3.67333E-08
Pseudoalteromonas_ulvae	1.68282E-06	1.57182E-07	5.08273E-07	1.15645E-06	1.11154E-07	0	1.09723E-06	0.000001279	4.58182E-07	1.18882E-06	9.66636E-07	3.77727E-07	8.80667E-08	3.11067E-07	5.71267E-07	5.76733E-07
Pseudoalteromonas_luteoviolacea	2.56364E-08	0	1.63636E-08	5.88364E-07	0.000000791	2.43692E-07	0.000000163	8.43077E-07	1.02455E-07	2.11727E-07	6.87727E-07	6.48955E-07	1.03613E-06	1.54133E-06	1.60907E-06	2.29667E-06
Litorilituus_sediminis	1.11818E-07	0	0	4.73182E-07	2.96462E-07	0	2.31923E-07	0.000000172	0	0	1.99727E-07	2.33727E-07	2.568E-07	4.576E-07	6.962E-07	5.08933E-07
Thalassotalea_crassostreae	5.92727E-08	0	0	0	0	2.16923E-08	0	0	9.82727E-08	0.000000028	0	0	1.21333E-07	1.58E-08	0	1.34067E-07
Thalassotalea_sp._LPB0316	2.62364E-07	8.84545E-08	9.94545E-08	6.48182E-08	1.57692E-07	2.63385E-07	1.24615E-07	1.26154E-07	2.66545E-07	3.79818E-07	7.40727E-07	3.89182E-07	2.64E-08	1.02867E-07	1.21933E-07	6.62667E-08
Thalassotalea_sp._HSM_43	3.11818E-08	0	0	1.93636E-08	1.18923E-07	0	0	0	7.15455E-08	0	0	0	5.08E-08	3.48E-08	1.09533E-07	0.000000138
Thalassotalea_sp._PS06	5.92636E-07	0.000000065	0	5.58182E-07	2.37038E-06	9.48462E-08	5.49154E-07	1.65731E-06	3.22973E-06	1.49573E-06	1.88645E-06	1.07055E-06	2.39127E-06	1.42827E-06	1.99627E-06	9.46867E-07
Colwellia_sp._Arc7-D	0.000000143	0	0	0	0	0	0	0	0	3.03636E-08	0	0.000000035	0	0	0	0
Colwellia_beringensis	0	0	0	4.79091E-09	0	0	0	9.46154E-08	0	1.09364E-07	7.90909E-08	3.16364E-08	0	3.09333E-08	1.12667E-08	4.94667E-08
Colwellia_sp._PAMC_20917	2.25273E-07	7.54545E-08	6.18182E-08	3.84545E-08	1.95538E-07	0	3.11538E-08	2.18385E-07	0.000000054	8.56364E-08	2.83727E-07	4.67818E-07	3.102E-07	8.20667E-08	3.6024E-07	0.000000114
Colwellia_psychrerythraea	3.11818E-08	0	6.30727E-08	1.91818E-08	1.30462E-07	0	1.13231E-07	2.16846E-07	1.02455E-07	1.11273E-07	6.71818E-08	1.17818E-07	1.45933E-07	2.416E-07	1.60667E-07	1.83933E-07
Colwellia_sp._MT41	1.47455E-07	0	1.10909E-07	4.14545E-08	1.20538E-07	0	3.77692E-08	0	5.07636E-07	8.88182E-08	2.35455E-07	0.000000426	0	9.68667E-08	8.45333E-08	0.000000081
Colwellia_sp._20A7	2.94091E-07	0	0	3.07273E-08	7.45231E-07	6.78462E-08	7.66923E-08	0	8.51818E-08	0	6.68182E-08	8.67273E-08	5.55933E-07	2.19867E-07	7.97307E-07	4.49333E-07
Colwellia_sp._Arc7-635	1.77818E-06	1.68182E-08	7.50818E-07	5.59182E-07	1.05846E-06	0.000000329	2.91769E-07	1.26631E-06	7.85909E-07	6.76364E-08	8.78545E-07	7.05727E-07	7.40867E-07	4.43933E-07	6.55733E-07	7.41067E-07
Colwellia_sp._PAMC_21821	1.54364E-07	1.04545E-08	4.12182E-08	6.94545E-08	1.31454E-06	2.52308E-08	1.51538E-07	1.64846E-07	5.81818E-07	2.40909E-07	9.31818E-08	3.67091E-07	1.0468E-06	1.2446E-06	1.47377E-06	1.3348E-06
Parashewanella_spongiae	2.56364E-08	1.37273E-08	1.63636E-08	6.79727E-08	0.000000154	0	0	0.000000022	0	0	0	8.15909E-08	1.86667E-08	4.09333E-08	1.57067E-08	0
Parashewanella_tropica	0	0	0	0	2.99846E-07	3.46923E-08	0	0	8.91818E-08	0.000000062	1.59364E-07	1.39091E-07	4.68E-08	2.246E-07	1.98733E-07	2.114E-07
Shewanella_maritima	0	2.70909E-08	0	0	0	0	0	0	0	0	0	0	0	0	0	0
Shewanella_polaris	5.57273E-08	0.000000065	0	3.07273E-08	2.75385E-08	0	0	0	0	0	0	0	4.18667E-08	0	0	0
Shewanella_sp._FDAARGOS_354	0	0	0	0	0	0	0	0	0	0	0	3.94545E-08	2.72E-08	2.63333E-07	2.99333E-08	5.10667E-08
Shewanella_sp._YLB-08	1.80273E-07	0	0	5.18182E-09	6.86231E-07	2.13846E-08	0	0	5.79091E-08	0	0	1.42727E-08	1.70467E-07	3.85133E-07	3.51667E-07	2.18667E-07
Shewanella_sp._MR-7	0	0	0	0	3.15385E-08	0	0	0	0	0	0	7.13636E-09	2.24E-08	0	1.04267E-07	7.33333E-08
Shewanella_halifaxensis	3.69091E-08	5.94091E-08	0	0	0	3.15385E-08	3.77692E-08	0	0	0.000000116	6.68182E-08	0	2.14E-08	0	0	0
Shewanella_sp._YLB-06	1.11818E-07	1.41636E-07	0	0	9.76154E-08	1.89231E-08	0	7.40769E-08	1.17636E-07	1.03545E-07	1.64909E-07	2.10909E-07	5.44E-08	1.95467E-07	1.992E-07	0.000000212
Shewanella_chilikensis	7.88182E-08	0.000000065	0	6.60636E-08	7.61538E-08	0	0	0	1.67636E-07	1.04273E-07	3.95455E-08	0.000000035	4.17333E-08	0	1.3824E-07	4.04E-08
Shewanella_baltica	1.67636E-07	0	0	1.93636E-08	0.000000139	0	9.38462E-08	1.57769E-07	3.74818E-07	0	1.16818E-07	2.39909E-07	8.77333E-08	8.58E-08	3.88667E-08	6.02667E-08
Shewanella_putrefaciens	0	0	0	0	1.43538E-07	0	0	0	4.45455E-08	0	7.90909E-08	1.45818E-07	4.27333E-08	1.58E-08	5.94E-08	0
Shewanella_decolorationis	7.15455E-08	0	0	0	0	0	0	3.22308E-08	0	0	3.95455E-08	2.14545E-08	0	0	0	0
Shewanella_donghaensis	3.89364E-07	0	1.48182E-07	1.93455E-07	2.59231E-08	0	1.07538E-07	0.000000185	0	1.34909E-07	1.02727E-08	0.000000178	2.09333E-08	6.04667E-08	2.04733E-07	2.79333E-08
Shewanella_aestuarii	3.34909E-07	2.26864E-07	1.72909E-07	1.02182E-07	3.29769E-07	3.83077E-08	1.70846E-07	3.60846E-07	3.34455E-07	2.49545E-07	6.03364E-07	3.61455E-07	2.42267E-07	1.44467E-07	2.962E-07	1.67733E-07
Shewanella_sediminis	0	0	1.12545E-07	0.000000109	0.000000039	0	0	0	1.14909E-07	0	1.06182E-07	3.94545E-08	0	6.28667E-08	6.984E-08	1.86667E-08
Shewanella_piezotolerans	0.000000281	9.61364E-08	1.35636E-07	0	3.34615E-08	0	0	1.42692E-07	0.000000064	7.29091E-08	2.29909E-07	3.51682E-07	1.268E-07	0	0	1.86E-08

Shewanella_woodyi	6.80909E-08	0	0	0	0	0	1.47769E-07	0.000000113	1.15455E-07	8.58182E-08	1.69909E-07	3.94545E-08	6.08E-08	4.80667E-08	0	0
Shewanella_psychrophila	4.06273E-07	0	1.11391E-07	5.46364E-08	0	0	3.23846E-07	3.31385E-07	1.77818E-07	2.32091E-07	1.72091E-07	2.63091E-07	0	0	0	0
Shewanella_sp._SNU_WT4	6.23636E-08	0	4.12727E-09	0.000000076	4.13846E-08	0	6.66154E-08	9.23846E-08	1.65727E-07	2.85455E-08	1.04545E-07	1.20136E-07	2.44E-08	3.09333E-08	4.86667E-08	1.192E-07
Shewanella_benthica	7.37273E-08	1.98182E-08	4.12727E-09	0	3.15385E-08	0	6.41538E-08	0	1.15455E-07	0	1.02727E-08	7.64545E-08	0	0	9.25333E-08	6.75333E-08
Shewanella_violacea	2.96727E-07	1.03636E-08	3.65982E-07	3.58909E-07	1.27077E-07	1.62538E-06	8.71385E-07	1.00323E-06	3.79455E-07	0.000000221	0.00000015	2.72409E-07	5.58667E-08	1.12133E-07	2.776E-07	3.43733E-07
Shewanella_marisflavi	1.03727E-07	0	1.63636E-08	2.53818E-07	0	1.39846E-07	1.27308E-07	3.08769E-07	2.64545E-08	6.37273E-08	0.000000061	2.30455E-07	2.64E-08	3.48E-08	1.93733E-08	9.33333E-09
Shewanella_frigidimarina	0	0	0	0	8.92308E-08	0	0	5.22308E-08	0	0	0	7.12727E-08	2.64E-08	9.66E-08	1.10333E-07	3.67333E-08
Shewanella_oneidensis	0	0	0	0	1.82615E-07	2.52308E-08	6.58692E-07	8.36615E-07	0	0	5.52727E-08	8.98182E-08	1.58333E-07	5.15467E-07	0.000000392	4.638E-07
Shewanella_amazonensis	1.85564E-06	1.68182E-08	6.64545E-07	1.77227E-06	9.77692E-08	0	1.58569E-06	1.12369E-06	0.000000445	9.85273E-07	0.000001004	1.04591E-06	2.48533E-07	2.57867E-07	2.12333E-07	4.89533E-07
Shewanella_khirikhana	1.13955E-06	2.41545E-07	1.94636E-07	1.03427E-06	4.53692E-07	0	2.63077E-08	6.23077E-07	8.00364E-07	0.000000312	1.96164E-06	1.24545E-06	4.688E-07	2.94933E-07	9.35467E-07	0.000000461
Shewanella_algae	1.16182E-07	0.000000065	4.43E-08	8.81818E-08	1.19462E-07	1.74692E-07	1.09154E-07	4.38769E-07	2.23364E-07	1.96545E-07	3.95455E-08	7.62273E-08	2.66933E-07	3.442E-07	4.234E-07	1.75467E-07
Shewanella_sp._Arc9-LZ	0	0	0	0	1.78692E-07	6.59077E-07	1.30769E-07	1.86538E-07	7.46364E-08	0.000000081	5.82727E-08	7.95455E-08	9.66667E-08	1.288E-07	1.16267E-07	0
Shewanella_sp._Scap07	8.04727E-07	1.94545E-07	7.40909E-08	1.68545E-07	1.17038E-06	5.35385E-06	1.25954E-06	1.49708E-06	9.53091E-07	0.000000771	1.02836E-06	1.66336E-06	1.02553E-06	9.81667E-07	1.60147E-06	1.47413E-06
Shewanella_sp._MR-4	0	0	0	4.79091E-09	0	3.11154E-07	0	0.000000042	0	0	0	7.13636E-09	0	5.19333E-08	1.57067E-08	0
Shewanella_pealeana	2.70909E-08	6.00909E-08	0	9.27909E-08	2.07815E-06	0	0	4.65538E-07	2.40091E-07	5.56182E-07	6.55455E-08	0.0000047	9.48133E-07	1.57933E-06	8.43267E-07	1.3198E-06
Shewanella_loihica	2.70909E-08	0	2.96364E-08	1.43636E-08	1.38223E-06	1.26962E-06	1.71385E-05	0.000012118	1.24818E-07	2.85455E-08	1.47545E-07	6.35455E-08	1.6376E-06	4.6556E-06	4.25047E-06	2.14207E-06
Saliniradius_amylolyticus	1.47273E-07	2.13273E-07	3.82182E-08	1.32727E-07	0	0	1.28462E-07	0	0	3.03636E-08	0	6.21818E-08	0	0.000000033	7.26667E-08	0
Hydrocarboniclastica_marina	9.57727E-07	8.90909E-08	8.82182E-08	2.62182E-07	5.17846E-07	6.88308E-07	7.07923E-07	1.63238E-06	7.32545E-07	1.11827E-06	1.83573E-06	1.12582E-06	9.24467E-07	6.31533E-07	7.356E-07	7.05333E-07
Glaciecola_nitratireducens	1.04364E-06	0	7.40909E-08	2.33182E-07	0.000000194	2.95385E-08	6.29154E-07	1.63685E-06	6.37636E-07	6.63182E-07	7.42818E-07	5.98909E-07	4.88467E-07	5.924E-07	8.20867E-07	6.572E-07
Glaciecola_amylolytica	2.02545E-07	8.13636E-08	4.26036E-07	8.58091E-07	2.23846E-07	0	9.03846E-08	2.97692E-08	2.04455E-07	2.59727E-07	0.000000379	2.34455E-07	4.72E-08	1.29867E-07	2.50533E-07	1.55867E-07
Paraglaciecola_sp._L3A3	0	0	0	0	7.24615E-08	4.13846E-08	0	0	0	0	6.71818E-08	2.85455E-08	0	0	0	0
Paraglaciecola_mesophila	4.55091E-07	0	2.22727E-07	5.70909E-08	0	0	0	1.73077E-07	0	0	3.77273E-08	5.30909E-08	0	0	0	0
Paraglaciecola_sp._L1A13	4.65091E-07	3.95182E-07	1.37545E-07	3.52273E-07	2.58538E-07	7.29231E-08	0	9.42308E-08	5.34545E-07	3.28182E-07	6.49273E-07	3.05273E-07	0.00000015	1.58E-08	1.47973E-07	0
Paraglaciecola_psychrophila	1.66818E-07	0	0	0	0.000001207	0.000000026	0	2.46923E-08	5.79091E-08	0.000000047	0	7.13636E-09	6.478E-07	7.74333E-07	5.31733E-07	2.828E-07
Agarivorans_gilvus	8.24545E-08	1.04545E-08	0	0	1.19308E-07	0	0	0	0	6.25455E-08	0	3.31818E-07	2.086E-07	9.73333E-08	1.29733E-07	8.74667E-08
Lacimicrobium_alkaliphilum	1.74464E-06	2.69773E-07	8.41545E-07	9.74273E-07	3.54231E-07	7.11077E-07	2.74615E-07	7.04923E-07	9.85727E-07	7.86545E-07	1.51336E-06	5.71182E-07	5.26867E-07	3.882E-07	2.34867E-07	4.55733E-07
Salinimonas_sp._G2-b	5.29091E-08	0	0	3.24545E-08	0	0.000000026	6.23077E-08	0	1.61364E-07	9.24545E-08	6.53636E-08	4.79091E-08	4.48667E-08	3.09333E-08	2.99333E-08	9.33333E-09
Salinimonas_lutimaris	0	0.000000213	1.63636E-08	0	6.73077E-08	1.26846E-07	6.23077E-08	2.46923E-08	1.80545E-07	0	1.67636E-07	4.92727E-08	1.24667E-07	1.73733E-07	5.264E-08	1.86E-08
Salinimonas_sp._KX18D6	4.77182E-07	1.16364E-08	0	3.22155E-07	1.01615E-06	0	0	2.02154E-07	2.06091E-07	0	1.70455E-07	9.45E-08	0.000000517	4.45667E-07	8.24733E-07	4.58533E-07
Salinimonas_sediminis	5.57273E-08	8.18182E-08	4.12727E-09	2.23636E-07	2.85846E-07	5.70769E-07	3.43615E-07	5.34385E-07	3.38909E-07	1.52545E-07	4.26636E-07	2.43273E-07	3.046E-07	0.000000486	2.95467E-07	0.000000339
Catenovulum_sp._CCB-QB4	0	0	8.25455E-09	3.07273E-08	0	2.52308E-08	1.38692E-07	8.22308E-08	0	0	0	7.13636E-09	0	4.49333E-08	1.5464E-07	1.44533E-07
Catenovulum_sediminis	0.000000484	7.50091E-07	8.94E-08	0.000000431	0.000000262	6.53077E-08	2.90462E-07	1.34462E-07	2.06273E-07	1.62909E-07	1.57455E-07	7.51818E-08	1.22733E-07	4.20467E-07	3.3624E-07	2.28467E-07
Marinobacter_adhaerens	3.62727E-08	0	0	0.000000151	0	0	0	1.68385E-07	9.31818E-08	3.89091E-08	0	3.16364E-08	2.24E-08	3.26667E-08	1.65333E-08	1.654E-07
Marinobacter_sp._BSs20148	0	0	0	4.20909E-08	2.05385E-08	0	5.36923E-08	0	0	0	0	0	0	1.00933E-07	0	0
Marinobacter_salinus	1.58364E-07	3.49091E-08	3.27273E-08	1.91818E-08	0	0	0.00000023	1.19923E-07	1.15455E-07	8.88182E-08	1.06727E-07	4.21364E-08	4.01333E-08	1.61333E-08	1.282E-07	0
Marinobacter_hydrocarbonoclasticus	9.71818E-08	1.85273E-07	2.96364E-08	9.00909E-08	1.86923E-08	6.89846E-07	2.63077E-08	5.22308E-08	1.01636E-07	1.34909E-07	7.53636E-08	8.42727E-08	4.72667E-08	0	1.12667E-08	2.79333E-08
Marinobacter_psychrophilus	2.57091E-07	0	0	0	0	0	0	0	0	0	0	0	0	0	0	5.10667E-08
Marinobacter_sp._es.042	1.58182E-08	0	0	3.72455E-08	0	0	0	1.01923E-07	2.16364E-08	0	0	0	0	0	0	0
Marinobacter_sp._LQ44	5.57273E-08	0	0.000000046	3.10818E-07	1.10769E-07	3.13769E-07	1.02846E-07	0	1.13182E-07	1.62909E-07	1.04545E-07	3.57636E-07	0.000000148	6.26E-08	9.764E-08	1.20133E-07
Marinobacter_similis	1.16182E-07	0	3.70909E-08	6.92727E-08	1.00923E-07	6.77615E-07	7.34923E-07	1.65538E-07	2.26818E-07	2.85455E-08	2.93273E-07	9.25E-08	1.276E-07	2.004E-07	2.03533E-07	2.87533E-07
Marinobacter_sp._CP1	0.00000011	6.38182E-08	0	5.29091E-08	3.15385E-08	0.000000203	1.58308E-07	0.000000042	8.51818E-08	9.92727E-08	7.23636E-08	6.01818E-08	2.14667E-08	1.71333E-08	1.93733E-08	1.236E-07
Marinobacter_sp._NP-4(2019)	2.70909E-08	0	4.12727E-09	1.07727E-07	0	0	0	0	3.98182E-08	0.000000047	1.16636E-07	1.42727E-08	0	3.09333E-08	0	0
Marinobacter_fonticola	1.99091E-07	0.000000188	6.76818E-08	2.41273E-07	0	1.26923E-07	3.77692E-08	8.92308E-08	0.00000016	2.85455E-08	1.78909E-07	5.64545E-08	4.78E-08	3.26667E-08	3.86E-08	2.92E-08
Marinobacter_salarius	1.61727E-07	0	7.55818E-08	0.000000128	1.63769E-07	0	0	5.00615E-07	5.91818E-08	3.03455E-07	3.02455E-07	1.59818E-07	2.37333E-08	1.58E-08	2.102E-07	2.87933E-07
Marinobacter_sp._LV10R510-11A	0.000002823	6.65727E-07	2.95645E-06	2.22509E-06	2.86077E-07	1.68538E-07	2.38615E-06	2.89954E-06	1.25018E-06	1.39982E-06	1.32464E-06	2.09927E-06	5.23667E-07	9.12067E-07	6.58267E-07	4.65733E-07
Marinobacter_sp._JH2	2.83718E-06	5.08909E-07	2.67064E-06	1.47409E-06	0.000000089	0	0	2.46923E-07	4.87545E-07	4.90182E-07	1.44445E-06	1.09555E-06	0	2.882E-07	4.44E-09	1.22E-08
Marinobacter_sp._Arc7-DN-1	1.26027E-06	5.03636E-08	8.30909E-08	1.34773E-06	5.69231E-07	0	3.33077E-08	0.00000049	7.27182E-07	7.51545E-07	7.66909E-07	6.12364E-07	2.72133E-07	8.86533E-07	4.78667E-07	6.29733E-07
Alteromonas_sp._BL110	0	0	0	0	1.10538E-07	0	0	0	0	0	0	2.14545E-08	0	0	0	0
Alteromonas_sp._76-1	0	0	0	0	0	0	0	0	0	0	0	0	0	0	1.65333E-08	0
Alteromonas_sp._MB-3u-76	0	0	0	0	0	0	1.15077E-07	8.01538E-08	0	0	0	0	7.24E-08	4.88E-08	2.99333E-08	1.334E-07
Alteromonas_sp._RKMC-009	1.16182E-07	6.07727E-08	2.17273E-07	3.95182E-08	9.05385E-08	3.06154E-07	0	2.11308E-07	1.46909E-07	1.84364E-07	1.28909E-07	7.56636E-07	0	0.000000014	1.45533E-07	1.34267E-07
Alteromonas_naphthalenivorans	0	0	0	1.05364E-07	0	1.08077E-07	2.04769E-07	0	0	0	1.19273E-07	9.03636E-08	4.16E-08	0	3.86E-08	1.97533E-07
Alteromonas_australica	2.04727E-07	0	9.04545E-08	2.06909E-07	4.80769E-08	3.15385E-08	0	0.000000022	1.54909E-07	2.87273E-08	0.000000183	1.11182E-07	0.000000053	6.30667E-08	2.01333E-08	1.22E-08
Alteromonas_macleodii	0	2.74909E-07	3.29818E-08	2.89091E-08	1.26462E-07	3.96154E-08	2.81077E-07	2.47692E-08	3.98182E-08	0	1.02727E-08	4.92727E-08	9.42667E-08	3.38267E-07	2.59507E-07	9.33333E-09
Alteromonas_mediterranea	1.92545E-07	0.000000065	2.03491E-07	4.22364E-07	4.07308E-07	8.22308E-08	0.000000197	1.39231E-07	0	5.83636E-08	3.33636E-08	3.54545E-07	2.446E-07	3.188E-07	2.04867E-07	5.18733E-07
Endozoicomonas_montiporae	5.12727E-08	0	0	1.95455E-08	6.17692E-08	9.26154E-08	2.05615E-07	1.97538E-07	0.000000227	1.63545E-07	6.71818E-08	1.04818E-07	8.52667E-08	3.78E-08	1.69707E-07	1.35933E-07
Litoricola_lipolytica	0	0	0	4.72727E-08	3.58462E-08	3.99308E-07	0	6.45385E-08	1.15455E-07	0.000000142	1.97545E-07	1.84182E-07	5.44E-08	1.67133E-07	4.884E-08	3.72E-08
Kangiella_profundi	0	2.34545E-08	0	0	0	0	0	0	3.94545E-08	0	0	0	0	3.50667E-08	0	1.98667E-08
Kangiella_koreensis	0	0	0	1.93636E-08	0.000000097	2.16923E-08	0	0	3.98182E-08	0	7.90909E-08	1.18182E-07	7.16667E-08	0	4.44E-09	0
Kangiella_sediminilitoris	1.01345E-06	0.000000065	0	4.03727E-07	0.000000236	0	5.58462E-07	1.01162E-06	2.36727E-07	4.61091E-07	6.68364E-07	6.54091E-07	4.08067E-07	0.000000342	5.812E-07	4.76133E-07
Oleiphilus_messinensis	2.42364E-07	2.26545E-07	3.02455E-07	1.65091E-07	5.03231E-07	2.83846E-08	0	6.51538E-08	4.95818E-07	4.32909E-07	1.13682E-06	1.19745E-06	3.35267E-07	3.748E-07	6.21467E-07	2.22067E-07
Reinekea_forsetii	1.26455E-07	1.15727E-07	8.48909E-08	2.05818E-07	1.04538E-07	0	2.56846E-07	0.000000185	0.000000141	1.28545E-07	9.89091E-08	8.22727E-08	1.86667E-08	3.50667E-08	8.48E-08	8.48E-08
Gynuella_sunshinyii	1.84727E-07	0.000001005	5.57455E-07	1.15455E-07	1.13462E-07	7.80231E-07	3.20923E-07	9.41462E-07	2.63818E-07	2.41818E-07	2.78909E-07	9.34455E-07	2.80067E-07	1.79933E-07	3.692E-07	3.66467E-07
Saccharospirillum_mangrovi	5.27273E-08	7.40909E-08	4.12182E-08	4.35455E-08	2.80462E-07	2.95385E-08	0	0	1.89909E-07	1.32273E-07	2.14545E-07	3.25636E-07	5.01667E-07	5.036E-07	3.93467E-07	7.65333E-07
Hahella_sp._KA22	3.69091E-08	1.68182E-08	0	2.62909E-07	5.76154E-08	1.80462E-07	8.69231E-08	0.000000104	0.000000054	8.62727E-08	2.44545E-07	9.14545E-08	2.91333E-08	4.64667E-08	1.16467E-07	1.90933E-07
Hahella_chejuensis	9.14091E-07	0.000000419	1.98345E-07	7.56636E-07	0.000000459	2.67538E-07	2.42385E-07	4.79846E-07	6.50182E-07	5.62818E-07	4.95545E-07	1.13709E-06	2.79733E-07	4.474E-07	2.38467E-07	4.42867E-07
Spartinivicinus_ruber	3.23727E-07	1.68182E-08	1.37545E-07	1.67273E-07	1.23846E-07	2.20692E-07	2.09231E-07	1.93692E-07	1.44273E-07	1.40818E-07	3.32091E-07	4.30818E-07	1.39467E-07	3.21533E-07	2.21067E-07	2.70267E-07
Bermanella_marisrubri	2.56364E-08	1.16364E-08	0	1.16273E-07	7.16923E-08	4.35385E-07	0	0.000000011	1.04364E-07	0.000000028	2.76364E-08	2.48091E-07	1.634E-07	2.88467E-07	4.184E-08	9.60667E-08
Oleispira_antarctica	3.11818E-08	0	0	0	3.15385E-08	3.15385E-08	6.41538E-08	2.46923E-08	3.94545E-08	0	6.65455E-08	0	9.39333E-08	0	8.15333E-08	2.92E-08
Nitrincola_sp._KXZD1103	5.82727E-08	3.84773E-07	2.96364E-08	4.79091E-09	0	2.32615E-07	6.23077E-08	0	0	0.000000034	0	0	0	1.58E-08	0	0
Bacterioplanes_sanyensis	1.93273E-07	4.65455E-08	1.72727E-07	2.78727E-07	2.08308E-07	7.11846E-07	8.87692E-08	3.40846E-07	1.18818E-07	2.80727E-07	4.18364E-07	2.78818E-07	1.95067E-07	1.58267E-07	3.64133E-07	5.90133E-07
Thalassolituus_oleivorans	8.77364E-07	0	6.49182E-07	7.35909E-07	5.06462E-07	1.04308E-06	4.58846E-07	8.22077E-07	8.91273E-07	6.94636E-07	5.77182E-07	1.22327E-06	5.23067E-07	2.688E-07	2.66533E-07	4.46733E-07
Marinobacterium_sp._LSUCC0821	0	0	0	0	0	0	0	0	0	0	0	0	2.91333E-08	0	0	0
Marinobacterium_aestuarii	4.68909E-07	9.85455E-08	2.20818E-07	5.63182E-07	5.54769E-07	1.44385E-07	8.34154E-07	4.34692E-07	1.37555E-06	0.000000222	1.61273E-07	3.82818E-07	4.276E-07	4.806E-07	0.000000456	3.28467E-07
Neptunomonas_phycophila	3.62727E-08	2.34545E-08	1.63636E-08	9.97273E-09	2.26923E-08	0	0	1.05769E-07	0.000000027	2.87273E-08	2.02091E-07	3.57273E-08	0	0	2.55333E-08	8.32E-08
Neptunomonas_concharum	5.57273E-08	0	2.47273E-08	8.80545E-07	6.28154E-07	0	7.40769E-08	4.67769E-07	2.64545E-08	2.22182E-07	1.37273E-07	1.01364E-06	1.2226E-06	8.61533E-07	1.4194E-06	2.3812E-06
Marinomonas_arctica	0	9.36364E-09	0	0	0	2.16923E-08	0	0	0	0	0	0	5.18667E-08	0	0	0
Marinomonas_posidonica	0	0	0	4.82727E-08	0	0	0	0	0	0	5.52727E-08	7.13636E-09	2.64E-08	0	0	0
Marinomonas_primoryensis	0	1.16364E-08	3.70909E-08	0.000000052	7.80769E-08	8.31692E-07	3.39154E-07	7.47692E-07	1.84273E-07	0.000000259	3.70727E-07	2.20455E-07	1.40533E-07	2.22133E-07	2.39867E-07	3.09933E-07
Marinomonas_sp._SM1966	0	0	0	1.43636E-08	5.20769E-08	4.33846E-08	3.11538E-08	5.66923E-08	0.000000027	5.22727E-08	4.92909E-07	0.000000115	7.18667E-08	0	4.48667E-08	1.86E-08
Marinomonas_mediterranea	1.03818E-07	0	4.12727E-09	9.40909E-08	0	0.000000284	2.63077E-08	1.24923E-07	2.97818E-07	0	3.58364E-07	2.80409E-07	1.79467E-07	6.10667E-08	1.93733E-08	2.92E-08
Marinomonas_sp._FW-1	0	0	0	1.81364E-07	8.92308E-08	0	8.86154E-08	2.58462E-07	1.18182E-07	0	3.77273E-08	5.70909E-08	4.32E-08	3.48E-08	2.73333E-07	1.87467E-07
Marinomonas_sp._MWYL1	1.11818E-07	0	0	1.05664E-06	1.25738E-06	8.27692E-08	4.83385E-07	6.37308E-07	8.72727E-08	3.23091E-07	9.28818E-07	1.73836E-06	1.77867E-06	1.19413E-06	2.02933E-06	3.08487E-06
Ketobacter_alkanivorans	1.21182E-07	0	0	2.64727E-07	2.62385E-07	0	1.08077E-07	3.22308E-08	1.02455E-07	3.62727E-08	9.42727E-08	4.34364E-07	1.56733E-07	8.38667E-08	1.3544E-07	1.22E-08
Alcanivorax_sp._NBRC_101098	0	0	0	7.45455E-08	0	0	0	0	0	0	0	0	0	0	0	0
Alcanivorax_xenomutans	1.01455E-07	2.34545E-08	7.40909E-08	0	2.88462E-08	3.44923E-07	6.44615E-08	2.16385E-07	7.73636E-08	3.89091E-08	2.05455E-08	9.07273E-08	4.88667E-08	1.01733E-07	3.58E-08	2.05933E-07
Alcanivorax_dieselolei	1.02727E-07	1.41636E-07	0	2.90909E-08	3.88462E-08	0	0	0	1.71636E-07	1.13636E-07	2.31455E-07	2.18182E-07	0	0	0	0
Alcanivorax_pacificus	9.14455E-07	0	7.25455E-07	1.55473E-06	2.58462E-07	0	6.35077E-07	5.55077E-07	0.000001229	9.75182E-07	5.51455E-07	6.11818E-07	3.95867E-07	3.748E-07	7.33867E-07	1.1066E-06
Alcanivorax_sp._N3-2A	5.04345E-05	1.70571E-05	2.87345E-06	2.37018E-05	2.96038E-05	1.13108E-05	1.37708E-05	1.22685E-05	3.19955E-05	1.96664E-05	2.36718E-05	5.44737E-05	2.60492E-05	7.71493E-06	0.000010582	2.03227E-05
Candidatus_Portiera_aleyrodidarum	0	0	0	4.79091E-09	0	0	0	2.49231E-07	0	0	0	0	7.48667E-08	1.61333E-08	0	1.10667E-07
Pistricoccus_aurantiacus	2.96364E-08	0	0	2.95455E-08	0	0	0	0	0	0	1.16818E-07	2.14545E-08	1.268E-07	1.27133E-07	2.63933E-07	1.53533E-07
Chromohalobacter_salexigens	7.40909E-08	8.18182E-08	7.17273E-08	4.02091E-07	7.23077E-08	0.000000136	5.74615E-08	2.21077E-07	1.15636E-07	8.70909E-08	5.52727E-08	3.14364E-07	5.88667E-08	0	1.65333E-08	1.17533E-07
Salinicola_tamaricis	2.35364E-07	0.000000065	4.12727E-09	1.91818E-08	1.13385E-07	1.23392E-06	2.63077E-08	1.17615E-07	2.39364E-07	5.22727E-08	2.38364E-07	8.67273E-08	6.62667E-08	1.46067E-07	1.26973E-07	6.14E-08
Halotalea_alkalilenta	1.57818E-07	8.38636E-07	1.47764E-07	2.73455E-07	6.73846E-08	7.80308E-07	2.43538E-07	2.59154E-07	1.30727E-07	1.33091E-07	1.14727E-07	1.14118E-06	2.14E-08	3.78E-08	1.156E-07	2.06467E-07
Cobetia_pacifica	0	0	0	0	0	0	0	0	0	3.03636E-08	0	0	0	1.61333E-08	0	9.33333E-09
Cobetia_marina	0	0	0	0	2.05385E-08	0	0	0	0	0	0	0	0	0	4.44E-09	0
Cobetia_sp._L2A1	1.36636E-07	1.53273E-07	4.12727E-09	4.32455E-08	1.86923E-08	0	6.41538E-08	3.22308E-08	2.75455E-08	8.02727E-08	2.54636E-07	5.70909E-08	6.75333E-08	4.49333E-08	0	0
Candidatus_Carsonella_ruddii	8.24545E-08	0	0	0	0.000000119	1.57308E-07	0	5.81538E-08	0	0	0	7.13636E-09	2.92133E-07	2.13533E-07	1.336E-07	4.95333E-08
Zymobacter_palmae	4.45455E-07	0	1.63636E-08	5.18182E-09	1.75462E-07	1.57154E-07	0.000000408	2.52385E-07	0.000000262	2.06727E-07	1.56364E-07	1.54364E-07	1.49467E-07	1.46933E-07	2.766E-07	2.06133E-07
Kushneria_sp._YCWA18	0	2.34545E-08	0	1.12091E-07	0	8.10769E-08	2.73846E-08	3.06308E-07	4.45455E-08	1.24727E-07	0	5.70909E-08	0	2.62E-08	1.54867E-07	1.22E-08
Kushneria_marisflavi	2.29955E-06	2.30182E-07	6.10591E-07	2.07609E-06	3.65154E-07	2.33077E-07	1.80608E-06	1.61054E-06	7.10091E-07	1.34364E-06	0.000001284	1.35755E-06	3.326E-07	4.69867E-07	4.66867E-07	3.40133E-07
Kushneria_konosiri	1.11109E-06	1.08141E-06	4.85909E-07	1.11064E-06	7.64769E-07	0.000000856	2.09069E-06	5.84308E-07	1.07327E-06	7.23727E-07	9.34273E-07	1.28155E-06	3.60933E-07	5.92067E-07	4.206E-07	4.59333E-07
Halomonas_sp._THAF12	0	0	0	3.24545E-08	3.88462E-08	4.35692E-07	3.11538E-08	8.02308E-08	0	2.87273E-08	0	0	0	0	6.39067E-08	8.36E-08
Halomonas_sp._AM6	8.28182E-08	0	0	0	3.15385E-08	0	0	0	4.45455E-08	1.71636E-07	5.29273E-07	3.94545E-08	0	0	0	0
Halomonas_sp._JS92-SW72	0	0	0	3.24545E-08	0	6.27692E-08	0	2.47692E-08	5.79091E-08	5.65455E-08	1.02727E-08	9.54545E-08	2.24E-08	1.71333E-08	2.46E-08	0
Halomonas_sp._GT	0	0	0	9.55455E-08	6.89231E-08	0	0	0	0	0	7.70909E-08	7.13636E-09	0	0	0	7.93333E-08
Halomonas_socia	3.11818E-08	2.70909E-08	0	5.18182E-09	0	0	8.69231E-08	0	3.75455E-08	6.42727E-08	3.77273E-08	3.57273E-08	4.52E-08	0	5.94E-08	3.08E-08
Halomonas_sp._SH5A2	2.79545E-07	0.000000065	3.27273E-08	1.78818E-07	0.000000039	5.91538E-08	5.96154E-08	0.000000042	1.73636E-07	0	0.000000048	0.000000097	0	0	2.62E-08	4.76667E-08
Halomonas_sp._HL-93	3.69091E-08	0	2.96364E-08	0.000000178	0.000000061	2.52308E-08	2.35385E-07	1.52385E-07	2.22091E-07	8.95455E-08	0	0	0.000000076	1.02667E-07	6.73067E-08	0
Halomonas_sp._THAF5a	1.87727E-07	4.42909E-07	0.000000046	1.01727E-07	0	1.30769E-07	7.45846E-07	2.46923E-08	6.62727E-08	3.62727E-08	0	4.05273E-07	0	3.09333E-08	3.43733E-08	6.33333E-08
Halomonas_sp._PA16-9	1.04818E-07	1.16364E-08	3.70909E-08	1.99364E-07	0	0.00000028	0	9.63077E-08	7.64545E-08	2.87273E-08	5.52727E-08	1.15273E-07	4.74E-08	4.49333E-08	1.74933E-07	6.33333E-08
Halomonas_subglaciescola	3.69091E-08	6.55455E-08	7.66364E-08	1.93636E-08	1.23308E-07	2.52308E-08	0	0	0.000000064	2.85455E-08	2.76364E-08	3.57273E-08	4.00667E-08	0.000000047	2.38E-08	8.94667E-08
Halomonas_sp._BC-M4-5	1.49364E-07	0.000000044	1.20818E-07	1.03545E-07	0	2.16923E-08	8.11538E-08	7.87692E-08	0.00000016	2.26636E-07	3.95455E-08	5.70909E-08	0.000000193	0	7.62E-08	3.67333E-08
Halomonas_sp._1513	2.55273E-07	3.61182E-07	5.34545E-08	2.93273E-07	0	1.25923E-07	8.23077E-08	3.81538E-08	5.96182E-07	5.22727E-08	1.88273E-07	2.49364E-07	2.64E-08	0	1.586E-07	1.07533E-07
Halomonas_sp._PGE1	1.58182E-08	0	0	2.03182E-07	0.000000077	2.32077E-07	1.63154E-07	1.67462E-07	2.29909E-07	1.31182E-07	7.69091E-08	2.45091E-07	4.99333E-08	1.294E-07	0	1.22E-08
Halomonas_sp._HG01	5.17273E-07	0	5.48182E-08	0.000000152	1.23615E-07	6.73846E-08	2.38846E-07	4.32308E-08	2.46091E-07	5.12909E-07	0.000000205	7.69636E-07	9.47333E-08	3.09333E-08	9.08667E-08	3.01733E-07
Halomonas_huangheensis	2.96364E-08	0	4.12727E-09	0	0	0	2.63077E-08	0	4.80909E-08	0	2.76364E-08	1.01636E-07	0	3.50667E-08	5.49067E-08	9.33333E-09
Halomonas_beimenensis	7.55727E-07	1.29973E-06	1.47582E-07	9.95909E-07	3.18231E-07	9.09769E-07	1.98615E-07	1.65385E-07	2.96273E-07	4.68818E-07	6.07364E-07	1.75364E-06	2.29667E-07	1.84067E-07	3.24467E-07	2.59267E-07
Halomonas_meridiana	4.73364E-07	2.34545E-08	9.26091E-08	1.43482E-06	2.29385E-07	1.62692E-06	0.00000244	2.13015E-06	0.000000319	6.65909E-07	9.64182E-07	5.23545E-07	1.17133E-07	9.75333E-08	1.598E-07	3.38533E-07
Halomonas_chromatireducens	5.35091E-07	3.05591E-06	1.83891E-07	2.98909E-07	1.10692E-07	5.91538E-08	7.07692E-08	2.46923E-08	2.53364E-07	2.96545E-07	8.15455E-08	1.62727E-07	1.02867E-07	1.61933E-07	1.50907E-07	5.14067E-07
Halomonas_sp._Y2R2	5.20909E-08	0	5.11273E-08	7.64273E-08	1.48385E-07	2.03308E-07	7.92308E-08	2.97692E-08	0	1.32545E-07	1.30909E-07	1.83091E-07	1.42867E-07	2.14533E-07	1.50333E-07	1.55933E-07
Halomonas_aestuarii	0	0	1.63273E-07	3.41636E-07	9.89231E-08	0	2.73846E-08	1.05846E-07	3.75455E-08	5.22727E-08	0	1.11455E-07	4.73333E-08	0	0.000000064	1.10533E-07
Halomonas_hydrothermalis	1.11091E-07	0	5.36636E-08	0.000000127	1.54231E-07	2.24846E-07	1.67385E-07	3.00538E-07	1.01227E-06	3.99273E-07	1.85636E-07	4.90182E-07	0.00000064	1.406E-07	3.71267E-07	5.47533E-07
Halomonas_sp._GFAJ-1	1.02909E-07	1.97273E-07	8.70909E-07	0.000000501	5.23923E-07	0	0	0	0.000000864	6.86727E-07	1.01809E-06	8.72636E-07	4.91933E-07	8.83267E-07	4.702E-07	7.902E-07
Halomonas_titanicae	5.57273E-08	0.000000065	0	7.45455E-08	2.75154E-07	2.47538E-07	1.56962E-06	2.29254E-06	1.17636E-07	0	0	9.31818E-08	2.65533E-07	4.074E-07	2.41333E-07	2.37333E-07
Halomonas_alkaliphila	5.57273E-08	0	1.78491E-07	4.03818E-07	5.93846E-08	0	0	0	7.15455E-08	1.54636E-07	1.68455E-07	1.43045E-07	5.08667E-08	9.87333E-08	2.032E-07	0.000000171
Halomonas_elongata	1.12645E-06	2.15455E-07	7.57182E-07	8.57545E-07	0.000000164	3.75154E-07	6.02538E-07	2.79385E-07	1.45655E-06	0.000000258	9.56182E-07	5.82091E-07	4.358E-07	3.58467E-07	3.79867E-07	2.30933E-07
Halomonas_sp._NBT06E8	6.17455E-07	0	0	3.42182E-07	7.81538E-08	0	0	0	2.56455E-07	2.07182E-07	1.84818E-07	3.44727E-07	1.34933E-07	2.09467E-07	4.20333E-07	3.864E-07
Halomonas_sp._YLGW01	5.89818E-07	1.56364E-07	8.64545E-08	5.47545E-07	2.78077E-07	4.05308E-07	1.74077E-07	3.24308E-07	3.17727E-07	3.03636E-08	9.93091E-07	0.000000573	5.38667E-08	2.878E-07	2.51133E-07	2.62067E-07
Halomonas_sp._KO116	5.61727E-06	0.00000026	7.40909E-08	4.89909E-06	1.35985E-06	0	4.33846E-08	2.06692E-06	2.06791E-06	3.18145E-06	1.85445E-06	2.77818E-06	3.2784E-06	2.5148E-06	2.72747E-06	4.38207E-06
Halomonas_sp._N3-2A	0.000214082	7.60369E-05	8.13573E-06	8.84718E-05	0.000113221	4.49825E-05	5.84975E-05	4.75972E-05	0.000133732	0.000116088	7.69367E-05	0.000190581	0.000121413	3.83576E-05	4.46737E-05	7.35251E-05
Moraxella_bovis	3.62727E-08	0	1.63636E-08	4.79091E-09	0	0	0	0.000000011	0	0	0	9.03636E-08	2.72E-08	0	2.97333E-08	4.28E-08
Moraxella_cuniculi	1.14636E-07	0	0	2.43636E-08	0.000000116	0	0	5.81538E-08	5.97273E-08	0.000000047	3.33636E-08	0.000000035	1.12267E-07	0.000000103	2.79467E-07	2.02867E-07
Moraxella_osloensis	2.81673E-06	1.05364E-07	2.60545E-07	3.05818E-07	6.41385E-07	2.97385E-07	1.33992E-06	1.37131E-06	1.69973E-06	3.62327E-06	3.52364E-06	2.58891E-06	7.836E-07	5.29933E-07	7.49533E-07	0.000001797
Moraxella_catarrhalis	6.74091E-07	1.68182E-08	0	1.43636E-08	7.86538E-07	6.15231E-07	2.36077E-07	3.21231E-07	0.000000324	2.37636E-07	5.80273E-07	4.80182E-07	5.368E-07	0.000000647	8.904E-07	9.15067E-07
Moraxella_bovoculi	1.98882E-06	0.000000065	0.000000978	1.98236E-06	9.61077E-07	0	1.40969E-06	1.12631E-06	5.51091E-07	8.48818E-07	6.55909E-07	7.36455E-07	7.24333E-07	8.564E-07	1.03693E-06	9.36733E-07
Psychrobacter_sp._PRwf-1	3.62727E-08	0	0	0	0	0	0	0	0	0	0	0	2.72E-08	0	0	0
Psychrobacter_sp._P11G3	0	0	0	0	3.58462E-08	0	0	0	0	0	0	0	0	0	1.93733E-08	0

Psychrobacter_sp._DAB_AL43B	0	0	0	0	0	0	0	0	0	0	0	0.000000057	2.14667E-08	4.64667E-08	1.08533E-07	0
Psychrobacter_sp._G	0	5.03636E-08	0	2.39091E-08	0	0	0	1.81846E-07	0	0	6.65455E-08	0	0	0	0	0
Psychrobacter_sp._P11G5	1.58182E-08	0	0	0	0	0	0	2.46923E-08	0	0	0	7.55455E-08	0	0	0	0
Psychrobacter_urativorans	1.28273E-07	0	0	5.68182E-08	0	0	0	7.13846E-08	0	0	0	0	0	2.38E-08	0	4.78667E-08
Psychrobacter_sp._P11F6	1.04364E-07	0	2.59818E-08	4.50909E-08	7.17692E-08	0	5.85385E-08	2.56923E-08	0.000000342	0	4.36364E-08	3.24909E-07	4.38E-08	4.80667E-08	1.526E-07	1.86E-08
Psychrobacter_arcticus	2.30909E-08	0	0	6.62455E-08	3.15385E-08	7.21538E-08	0	9.19231E-08	1.04545E-07	1.33273E-07	1.15182E-07	8.16364E-08	2.14E-08	1.478E-07	5.88E-08	0
Acinetobacter_towneri	0	0	0	0	0	2.07231E-07	0	0	0	0	0	0	0	0	0	0
Acinetobacter_sp._NEB_394	0	0	0	0	0	7.21538E-08	0	0	0	0	0	0	0	0	0	0
Acinetobacter_piscicola	2.56364E-08	0	0	0	0	0	0	0	0	0	0	0	0	0	0	0
Acinetobacter_shaoyimingii	0	0	0	0	0	3.83077E-08	0	0	0	0	0	0	0	0	0	1.66667E-08
Acinetobacter_sp._SWBY1	0	0	0	0	0	0	0	4.94615E-08	0	0	0	0	0	0.000000033	0	0
Acinetobacter_bereziniae	0	0	0	0	0	0	0	3.57692E-08	0	0	0	0	0	0	1.49333E-08	1.19733E-07
Acinetobacter_sp._ACNIH2	9.93636E-08	7.87273E-08	0	6.61545E-08	5.47692E-08	2.33769E-07	0	5.81538E-08	7.18182E-08	8.62727E-08	0	0.000000035	2.37333E-08	0	4.82E-08	0
Acinetobacter_oleivorans	0	0	0	0	1.36538E-07	0	0	0	0	0	0	0	4.33333E-08	1.58E-08	1.51133E-07	8.72667E-08
Acinetobacter_sp._TGL-Y2	0	8.86364E-09	0	0	2.05385E-08	0	0	2.97692E-08	4.45455E-08	0	0	1.42727E-08	6.91333E-08	0	7.43333E-08	1.32733E-07
Acinetobacter_sp._WCHA45	0	1.04545E-08	0	0	9.23077E-08	0	0	2.34154E-07	0	0	0	1.42727E-08	1.17867E-07	7.94E-08	3.8304E-07	3.84267E-07
Acinetobacter_chinensis	0	0	0	2.85182E-07	0	0	2.63077E-08	1.90769E-08	0	0	0	2.14545E-08	0	0	0	0
Acinetobacter_defluvii	3.62727E-08	0	0	5.18182E-09	2.59231E-08	1.44154E-07	0	0	0	0	6.88182E-08	0	0	0	0	0
Acinetobacter_sp._10FS3-1	3.62727E-08	0	0	4.50909E-08	1.73615E-07	0	0	3.22308E-08	1.15455E-07	2.49273E-07	6.88182E-08	6.42727E-08	1.48267E-07	0.000000033	9.44E-08	6.86667E-08
Acinetobacter_sp._NCu2D-2	3.11818E-08	0	0	0	0	0	0	0	0	0	6.88182E-08	3.94545E-08	2.70533E-07	0.00000014	0	0
Acinetobacter_sp._MYb10	0	5.58636E-07	0	0	0	4.27692E-08	0	0	2.64545E-08	3.89091E-08	2.70909E-07	7.89091E-08	3.94333E-07	0.000000478	7.904E-08	0.000000235
Acinetobacter_lactucae	1.41818E-07	0	0	1.43636E-08	0	0	0	0	0.000000027	1.21636E-07	6.88182E-08	5.64545E-08	0	9.96E-08	6.91067E-08	0
Acinetobacter_equi	0	0	0	0	0	7.96923E-08	0	0	0	0	3.95455E-08	0	0	0	0	4.04E-08
Acinetobacter_ursingii	6.87273E-08	3.46455E-07	2.87273E-08	1.98636E-07	0	0	0	2.46923E-08	4.43091E-07	4.11455E-07	1.35636E-07	1.16909E-07	5.02E-08	0	1.65333E-08	9.04667E-08
Acinetobacter_baylyi	3.15455E-08	0	0	1.93636E-08	0.000000039	6.30385E-07	6.23077E-08	7.75538E-07	9.72727E-08	0.000000034	0	1.92136E-07	9.03333E-08	4.33333E-08	4.64667E-08	2.34667E-07
Acinetobacter_lanii	2.83727E-07	0	0	4.65273E-07	7.53077E-08	0	0	0	1.96636E-07	2.60455E-07	8.86364E-08	5.17273E-08	4.33333E-08	6.36E-08	1.59533E-07	3.67333E-08
Acinetobacter_sp._WCHA55	0	0	0	4.79091E-09	3.88462E-08	2.16154E-07	0	0	0.000000054	1.79545E-07	3.33636E-08	0	0	0	4.44E-09	0
Acinetobacter_sp._TTH0-4	0	0	0	0	0	4.13846E-08	6.41538E-08	7.55385E-08	0	0	1.00182E-07	5.50455E-08	6.08E-08	0.000000064	1.33333E-08	6.77333E-08
Acinetobacter_wanghuae	0	0	0	1.24455E-07	0	0	0	0	1.01636E-07	0.000000116	7.23636E-08	8.87273E-08	0	0	4.44E-09	1.13733E-07
Acinetobacter_soli	2.96364E-08	2.70909E-08	0	1.84909E-07	0	0	0	0	2.11182E-07	6.37273E-08	1.41182E-07	2.66545E-07	0	0	0	0
Acinetobacter_sp._WCHAc010052	5.07455E-07	0	0	4.67091E-07	4.64615E-08	0	2.63077E-08	0	4.40818E-07	4.31273E-07	1.11364E-07	2.76091E-07	2.09333E-08	2.84067E-07	1.09867E-07	1.18667E-07
Acinetobacter_sp._ACNIH1	2.38182E-07	0	0	9.81818E-08	2.59231E-08	0	0	0	0	3.62727E-08	1.48273E-07	5.89091E-08	0	0	1.49333E-08	1.866E-07
Acinetobacter_nosocomialis	0.000000508	4.01182E-07	1.40545E-07	2.96273E-07	3.77154E-07	9.36923E-08	4.19231E-07	2.91692E-07	6.70909E-08	2.85455E-08	5.75545E-07	5.22545E-07	0.000000087	2.53467E-07	3.098E-07	0.000000576
Acinetobacter_haemolyticus	1.48727E-07	0	8.75455E-09	4.02727E-08	1.09385E-07	7.89538E-07	0.000000229	3.33769E-07	6.45455E-08	3.89091E-08	2.82273E-07	2.74318E-07	2.73733E-07	1.24667E-07	1.992E-07	4.40933E-07
Acinetobacter_gyllenbergii	1.65873E-06	2.03818E-07	4.77545E-07	4.43273E-07	2.86923E-07	0	7.48462E-07	7.65385E-07	4.90636E-07	4.06091E-07	4.38818E-07	8.64273E-07	0	3.42E-08	1.99507E-07	1.416E-07
Acinetobacter_guillouiae	0.000001303	4.53409E-07	2.78909E-07	8.40364E-07	3.59692E-07	0	4.33846E-08	6.34615E-07	5.68909E-07	6.83545E-07	2.10845E-06	8.44909E-07	2.658E-07	1.56933E-07	5.06933E-07	2.99933E-07
Acinetobacter_schindleri	3.11818E-08	0	4.12727E-09	1.91818E-08	2.10692E-07	6.62538E-07	1.37769E-07	5.18077E-07	5.48182E-08	1.98455E-07	3.01636E-07	9.92727E-08	3.792E-07	0.000000176	5.012E-07	0.000000629
Acinetobacter_venetianus	0	0	8.25455E-09	0	0	0	0	0	9.34545E-08	1.05545E-07	0	1.22136E-07	1.96267E-07	8.42E-08	4.27333E-08	7.56667E-08
Acinetobacter_junii	3.62727E-08	0	8.23455E-08	4.68818E-08	0	0	5.43077E-07	1.72846E-07	0	0	6.88182E-08	1.325E-07	6.73333E-08	2.22067E-07	9.14E-08	5.27333E-08
Acinetobacter_sp._Marseille-Q1620	3.62727E-08	5.41818E-08	2.04909E-08	2.47273E-08	8.36154E-08	3.07231E-07	0	5.74615E-08	0.000000113	0	6.71818E-08	0.000000127	4.27333E-08	8.52E-08	1.12667E-08	6.33333E-08
Acinetobacter_sp._NEB149	2.56364E-08	6.76273E-07	4.12727E-09	0	0	0	0	0	3.75455E-08	0.000000028	0	0	0	0	0	0
Acinetobacter_sp._C16S1	0	0	8.25455E-09	6.65455E-08	0	0	0	0	0	0	0	0	0	0	0	0
Acinetobacter_johnsonii	8.17273E-08	1.04545E-08	1.94509E-07	1.73909E-07	1.86923E-08	1.36923E-06	1.07538E-07	0	0	0	6.65455E-08	0	1.86667E-08	6.41333E-08	1.28507E-07	9.33333E-09
Acinetobacter_cumulans	0	0	0.000000025	0	2.05385E-08	0	5.54615E-08	4.32308E-08	2.64545E-08	9.23636E-08	0	0	9.23333E-08	2.98E-08	4.44E-09	7.48667E-08
Acinetobacter_radioresistens	3.28273E-06	4.92045E-07	1.32209E-06	2.33782E-06	1.24469E-06	2.95385E-08	1.80808E-06	1.53738E-06	1.01645E-06	1.53873E-06	1.25409E-06	9.71545E-07	5.56733E-07	0.000000731	8.51467E-07	8.49667E-07
Acinetobacter_larvae	3.11818E-08	0	0	0	3.15385E-08	0	0	0	0.000000027	0.000000028	1.30909E-07	3.94545E-08	0	6.57333E-08	1.49333E-08	6.33333E-08
Acinetobacter_lwoffii	4.87545E-07	9.39273E-07	2.18182E-07	5.82545E-07	7.78462E-08	0	0	0	8.98273E-07	4.02455E-07	6.51909E-07	0.000000267	8.22667E-08	0.000000087	1.51667E-07	6.11333E-08
Acinetobacter_sp._LoGeW2-3	0	0	2.96364E-08	0	0	0	0	0	0	0	0	7.13636E-09	0	3.48E-08	2.99333E-08	4.04E-08
Acinetobacter_sp._WCHAc010034	3.85909E-07	0.00000013	2.94891E-07	2.95636E-07	1.58462E-07	0	1.75538E-07	1.12154E-07	0.000000302	3.32727E-07	2.36182E-07	0.000000245	3.244E-07	3.98133E-07	4.23707E-07	2.94533E-07
Acinetobacter_tandoii	6.19091E-08	0	0	4.79091E-09	1.39769E-07	0	6.23077E-08	0	0.000000054	0	6.68182E-08	9.79091E-08	1.67867E-07	1.68933E-07	3.31707E-07	1.98667E-08
Acinetobacter_indicus	5.12727E-08	0	0	4.20909E-08	5.06308E-07	4.48462E-07	1.14923E-07	2.59077E-07	3.13091E-07	0.000000034	1.38182E-07	0.000000472	2.42933E-07	7.16933E-07	5.04133E-07	5.538E-07
Acinetobacter_wuhouensis	5.41818E-08	0.00000013	1.30091E-07	9.98364E-07	7.96308E-07	0	0.000000201	6.20385E-07	2.64545E-08	2.01909E-07	8.54818E-07	0.00000071	7.306E-07	1.00173E-06	1.06373E-06	1.6852E-06
Acinetobacter_calcoaceticus	3.48727E-07	0.000000501	6.67273E-08	2.68818E-07	2.96154E-07	4.27692E-08	4.40923E-07	1.04423E-06	1.78727E-07	2.22455E-07	5.45273E-07	1.00364E-06	1.352E-07	1.66667E-07	4.06667E-07	0.000001054
Acinetobacter_pittii	0	0	0	0	5.38615E-07	0	0	4.60385E-07	0.000000229	1.77455E-07	7.12727E-08	1.14091E-07	3.13867E-07	0.000000525	4.86667E-07	4.68467E-07
Acinetobacter_seifertii	0	0	3.70909E-08	3.07273E-08	1.70385E-07	0	0	3.79385E-07	0	0	0	0.000000176	1.05867E-07	2.48267E-07	1.48133E-07	8.67333E-08
Acinetobacter_baumannii	3.64982E-06	3.42873E-06	2.88645E-06	2.95136E-06	3.46585E-06	3.54447E-05	7.25462E-06	2.66454E-06	3.45455E-06	4.85273E-06	1.44798E-05	4.68236E-06	0.000006894	1.02307E-05	0.000008109	1.09647E-05
Oblitimonas_alkaliphila	1.09545E-07	0	0	3.11545E-07	1.92538E-07	2.52308E-08	1.64154E-07	3.72231E-07	1.46909E-07	2.29455E-07	1.48091E-07	4.28727E-07	5.34E-08	2.13133E-07	1.9404E-07	3.344E-07
Entomomonas_moraniae	1.08091E-07	0	0	4.80909E-08	2.30077E-07	0	5.26154E-08	5.81538E-08	0	1.11727E-07	0.000000048	0.000000784	3.58267E-07	4.39067E-07	5.232E-07	9.76E-08
Permianibacter_aggregans	1.71364E-07	0	8.75455E-09	5.18182E-09	2.84462E-07	0	0	4.74615E-07	8.78182E-08	3.03636E-08	0	0	1.67267E-07	2.614E-07	5.138E-07	6.008E-07
Azotobacter_chroococcum	0	0	5.35818E-08	9.54545E-09	0	0	1.46462E-07	6.05385E-08	0	0	0	1.42727E-08	2.64E-08	1.038E-07	1.43067E-07	8.30667E-08
Azotobacter_salinestris	1.74464E-06	6.46818E-07	1.85818E-06	2.23118E-06	0	0	6.08231E-07	0.000001178	7.96636E-07	7.46364E-07	1.28445E-06	1.43882E-06	2.64667E-08	1.13333E-07	1.67867E-07	7.46667E-08
Azotobacter_vinelandii	2.65182E-07	6.38182E-08	2.87273E-08	5.16364E-08	1.65615E-07	1.03354E-06	4.31231E-07	6.34231E-07	2.95818E-07	0.000000034	1.58909E-07	3.04727E-07	2.34667E-07	1.39733E-07	2.04467E-07	4.18333E-07
Pseudomonas_sp._IzPS23	0	0	0	0	0	6.76923E-08	0	0	0	0	0	0	0	0	0	0
Pseudomonas_sp._B10	0	1.68182E-08	0	0	0	0	0	0	0	0	0	0	0	0	0	0
Pseudomonas_sp._SWI7	0	6.70909E-08	0	0	0	0	0	0	0	0	0	0	0	0	0	0
Pseudomonas_sp._SCB32	0	7.40909E-08	0	0	0	0	0	0	0	2.87273E-08	0	0	0	0	0	0
Pseudomonas_moraviensis	0	0	0	0	0	0	0	0	0	0	0	0	2.09333E-08	0	0	0
Pseudomonas_sp._LPH1	0	0	0	0	0	0	0	0	0	0	0	0	2.24E-08	0	0	0
Pseudomonas_umsongensis	5.57273E-08	0	0	0	0	0	0	0	0	0	0	0	0	0	0	0
Pseudomonas_granadensis	3.11818E-08	0	0	0	0	0	0	0	0	0	0	0	0	0	0	0
Pseudomonas_sp._Leaf58	0	8.86364E-09	0	0	0	0	0	0	0	6.08182E-08	0	0	0	0	0	1.86E-08
Pseudomonas_sp._ADPe	0	0	0	0	0	0	0	0	3.75455E-08	0	0	0	0	0	0	0
Pseudomonas_sp._DTU12.1	0	0	0	0	0	0	0	3.81538E-08	0	0	0	0	0	0	0	0
Pseudomonas_extremaustralis	0	0	0	0	0	0	0	5.81538E-08	0	0	0	0	5.82E-08	0	0	0
Pseudomonas_pelagia	0	0	0	0	3.58462E-08	0	0	7.72308E-08	0	1.08818E-07	0	5.30909E-08	0	0.000000033	0	6.33333E-08
Pseudomonas_sp._SGAir0191	5.67273E-08	0	0	0	0	0	0	0	5.79091E-08	0	0	1.42727E-08	0	0	0	0
Pseudomonas_sp._B11D7D	0	0	0	0	0	0	0	0	0	0	0	7.13636E-09	0	3.09333E-08	0	3.97333E-08
Pseudomonas_sp._R84	0	0	0	0	0	0	0	0	0	0	0	2.79955E-07	1.71667E-07	0	0	0
Pseudomonas_sp._TCU-HL1	0	1.68182E-08	0	0	0	0	0	0	0	0	0	7.13636E-09	0	0	0	0
Pseudomonas_libanensis	0	0	0	0	0	0	0	0	0	0	0	7.13636E-09	0	0	0	0
Pseudomonas_extremorientalis	0	9.54545E-09	0	0	0	0	0	0	0	0	0	7.13636E-09	2.14E-08	0	0	0
Pseudomonas_sp._R2A2	0	0	0	5.18182E-09	0	0	0	0.000000042	0.000000027	0	0	0	0	0	0	0
Pseudomonas_sp._NIBRBAC000502773	0	3.24545E-07	0	1.03636E-08	0	0	0	0	0	0	0	0	0	0	0	0
Pseudomonas_glycinae	0	0	0	4.79091E-09	0	0	0	0	0	0	0	0	0	0	0	0
Pseudomonas_pohangensis	0	0	0	4.79091E-09	0	0	0	0	0	0.000000028	0	2.14545E-08	0	0	0	0
Pseudomonas_sp._R32	0	0	0	4.79091E-09	0	0	0	0	0	0	0	0	0	6.10667E-08	0	0
Pseudomonas_amygdali	0	1.68182E-08	0	5.29091E-08	0	0	0	1.15385E-07	0	0	0	0	0	0	0	0
Pseudomonas_sp._31-12	2.96364E-08	0	0	0	0	0	0	0	0	0	0	0.000000035	0	0	0	0
Pseudomonas_sp._TKP	0	0	0	3.99091E-08	0	0	0	0	0	0	0	0	0	0	0	0
Pseudomonas_palleroniana	0	0	0	1.16091E-07	6.16923E-08	3.83077E-08	0	1.90769E-08	0	0	0	1.39909E-07	0	0	0	2.79333E-08
Pseudomonas_lundensis	0	0	0	4.40909E-08	0	0	0	0	0	0	0	0	4.74667E-08	0	4.48667E-08	5.10667E-08
Pseudomonas_trivialis	0	0.000000065	0	9.54545E-09	5.76154E-08	0	0	0	0	0	0	1.42727E-08	2.37333E-08	0	1.49333E-08	1.86E-08
Pseudomonas_sp._ATCC_43928	3.69091E-08	0	0	9.97273E-09	0	0	0	0	0	0	0	1.78136E-07	0	0	2.97333E-08	6.33333E-08
Pseudomonas_sp._R11-23-07	0	0.000000065	0	4.88182E-08	3.88462E-08	0	0	0	0	0	0	7.13636E-09	6.08E-08	0	8.94E-08	7.76E-08
Pseudomonas_psychrophila	0	0	0	2.43636E-08	0	0	0	0	0	0	0	0	0	0	5.04667E-08	0
Pseudomonas_fragi	0	0	0	5.50909E-08	0	0	0	0	0	0	0	0.000000092	2.94667E-08	0	1.156E-07	9.33333E-09
Pseudomonas_koreensis	2.30909E-08	1.68182E-08	0	2.13636E-07	1.41846E-07	0	5.54615E-08	2.97692E-08	9.21818E-08	0	0	1.38273E-07	6.08E-08	1.20933E-07	0.000000493	2.232E-07
Pseudomonas_cremoricolorata	0	0	0	0	3.15385E-08	0	0	0	0	0	0	0	0	0	0.000000076	0
Pseudomonas_sp._FDAARGOS_380	0	0	0	1.43636E-08	0	0	0	0	0	0	0	7.13636E-09	0	1.58E-08	1.65333E-08	0
Pseudomonas_sp._S150	0	0	0	0	2.05385E-08	7.21538E-08	3.11538E-08	0	0.000000084	1.22182E-07	0	0	0	0	1.00667E-07	9.33333E-09
Pseudomonas_taetrolens	1.59636E-07	0	0	2.43636E-08	1.09538E-07	0.00000006	0	2.46923E-08	6.50909E-08	5.70909E-08	0	3.16364E-08	8.32E-08	1.61333E-08	8.23333E-08	1.424E-07
Pseudomonas_oleovorans	0	0	0	0	0	6.68154E-07	4.80769E-08	1.90769E-08	0	0	0	9.75E-08	6.08E-08	0	1.57067E-08	0
Pseudomonas_sp._R4-39-08	0	0	0	0	3.34615E-08	0	0	0	0	0	0	0	7.94E-08	6.34667E-08	1.32133E-07	0
Pseudomonas_lurida	2.98182E-08	0	0	9.54545E-09	0	8.69231E-07	0	0	0	0	0	0	4.16E-08	0	0	0
Pseudomonas_sp._BIOMIG1BAC	2.98182E-08	0	0	0	0	0	0	0	0	0	0	0	0	0	0	0
Pseudomonas_mucidolens	1.16909E-07	6.58636E-08	0	0.000000136	3.15385E-08	0	0	2.47692E-08	5.34545E-08	0	0	0.000000035	0	0	1.19573E-07	1.86E-08
Pseudomonas_cichorii	0	0	0	0	2.05385E-08	7.21538E-08	0	2.47692E-08	0	0	0	0	0.000000093	6.73333E-08	1.2024E-07	1.70867E-07
Pseudomonas_sp._Os17	5.57273E-08	3.58182E-08	0	0	0	0	1.45462E-07	2.97692E-08	0	3.62727E-08	0	1.42727E-08	0	0	0	0
Pseudomonas_sp._DG56-2	5.12727E-08	0	0	0	7.16923E-08	0	2.63077E-08	4.41538E-08	0	0	0	0	2.72E-08	0	7.43333E-08	1.378E-07
Pseudomonas_sp._SK	0	0	0	0	3.34615E-08	0	1.99385E-07	1.46923E-08	0	0	0	0	0.000000062	7.05333E-08	3.43733E-08	0
Pseudomonas_sp._LAB-08	0.000000089	1.89227E-07	2.87273E-08	4.50909E-08	0	0	2.63077E-08	7.77692E-08	2.64545E-08	0	0	0	2.24E-08	0	0	1.00067E-07
Pseudomonas_sp._LG1E9	0	0	2.87273E-08	9.54545E-09	0	0	0	1.46923E-08	0	5.73636E-08	0	0	0	0.000000033	0	0
Pseudomonas_litoralis	1.28273E-07	4.51818E-08	0	1.55791E-07	0	0	5.26154E-08	0	2.75455E-08	5.22727E-08	0	7.13636E-09	0	5.12E-08	5.94E-08	6.33333E-08
Pseudomonas_sp._14181154	3.11818E-08	0	0	5.18182E-09	0	0	0	0	3.98182E-08	0	0	0	0	0	1.12667E-08	0
Pseudomonas_sp._K2W31S-8	3.11818E-08	0	0	0	0	0	0	0	0	0	0	1.93045E-07	0	0	1.49333E-08	0
Pseudomonas_poae	3.11818E-08	0	0	1.93636E-08	0	0	3.33077E-08	0	4.45455E-08	6.37273E-08	0	0.00000007	0	1.61333E-08	1.57067E-08	3.72E-08
Pseudomonas_migulae	1.50545E-07	2.11818E-08	0	3.24545E-08	2.59231E-08	0	2.63077E-08	0.000000068	0	0.000000028	0	7.13636E-09	4.94E-08	0	0	3.40667E-08
Pseudomonas_antarctica	0	1.94545E-07	0	2.11455E-07	3.34615E-08	0	0	3.93846E-08	1.24182E-07	5.22727E-08	6.88182E-08	2.45727E-07	0	3.50667E-08	8.86667E-09	1.22E-08
Pseudomonas_sabulinigri	8.23636E-08	7.38636E-08	0	1.58909E-07	0	0	6.44615E-08	5.66923E-08	2.20545E-07	0	6.88182E-08	1.07182E-07	1.486E-07	9.79333E-08	2.0364E-07	9.15333E-08
Pseudomonas_multiresinivorans	2.96364E-08	0	0	5.18182E-09	1.29462E-07	0	2.63077E-08	5.81538E-08	0	0.000000028	6.88182E-08	2.14545E-08	2.08E-08	3.29333E-08	0	6.33333E-08
Pseudomonas_mandelii	0	0	0	0	0	0	0	0	0	0	1.02727E-08	0	0	0	1.49333E-08	0
Pseudomonas_sp._MPC6	0	0	0	3.85455E-08	0	3.34769E-07	0	0	3.94545E-08	1.04545E-07	1.02727E-08	1.48636E-07	0	1.05333E-07	4.44E-09	0
Pseudomonas_sp._DY-1	1.23818E-07	0	0	3.24545E-08	3.88462E-08	0	0	0	0	0	1.02727E-08	7.13636E-09	0	0	0	9.33333E-09
Pseudomonas_sp._TUM18999	0	0	0	0	3.34615E-08	0	0	0	2.64545E-08	0	1.02727E-08	0	0	0	0	0
Pseudomonas_sp._StFLB209	2.56364E-08	1.02864E-07	0	3.24545E-08	2.85154E-07	2.63037E-05	3.77692E-08	1.24385E-07	8.61818E-08	5.22727E-08	2.05455E-08	3.87727E-08	2.14E-08	1.46467E-07	6.45333E-08	9.33333E-09
Pseudomonas_thivervalensis	6.23636E-08	0	0	1.79545E-07	5.02308E-08	0	1.10385E-07	1.09077E-07	1.74091E-07	0.000000028	2.76364E-08	2.00909E-07	1.62733E-07	0	7.38E-08	0
Pseudomonas_sp._S-6-2	1.22455E-07	0	0	0	8.74615E-08	2.88308E-07	6.96923E-08	3.02846E-07	1.11273E-07	0	9.64545E-08	1.41818E-07	2.64E-08	1.86467E-07	4.44E-09	4.94E-08
Pseudomonas_sp._R5-89-07	0	9.54545E-09	0	1.93636E-08	0	0	0	0	0	0	2.76364E-08	2.14545E-08	5.34E-08	0	0	0
Pseudomonas_sp._CC6-YY-74	1.45364E-07	0	0	3.24545E-08	1.07692E-07	6.13077E-08	2.59615E-07	0	0	0	2.76364E-08	5.37273E-08	4.27333E-08	0.000000075	1.838E-07	3.82E-08
Pseudomonas_sp._CCOS_191	0	0	0	0	0	0	0	0	0	0	6.65455E-08	0	2.37333E-08	0	0	0
Pseudomonas_simiae	2.70909E-08	0	0	0	0	0	0	0	0	0	8.56364E-08	0	0	0	0	0
Pseudomonas_luteola	0	0	0	0	0	0	0	0	0	0	3.77273E-08	0	0	0	0	0
Pseudomonas_sp._FGI182	0	0	0	0	0	0	0	0	0	0	3.77273E-08	0	0	0	0	0
Pseudomonas_guangdongensis	5.41818E-08	0	0	5.18182E-09	5.63846E-08	0	2.63077E-08	0	3.75455E-08	8.08182E-08	6.00909E-08	1.42727E-08	7.28E-08	6.60667E-08	7.21333E-08	0
Candidatus_Pseudomonas_adelgestsugas	0	0	0	9.54545E-09	0	0	0	0	7.56364E-08	2.85455E-08	3.95455E-08	3.94545E-08	6.25333E-08	2.62E-08	0	5.70667E-08
Pseudomonas_nitroreducens	3.62727E-08	0	0	9.32727E-08	0	0	3.33077E-08	5.66923E-08	0.000000054	3.89091E-08	1.52273E-07	6.66364E-08	1.86667E-08	2.62E-08	4.83067E-08	9.04667E-08
Pseudomonas_sp._CFSAN084952	0	0	0	0	0	0	0	0	0	0	3.95455E-08	0	0	0	0	0
Pseudomonas_sp._NP-1	5.52727E-08	0	0	4.68818E-08	2.04923E-07	0	0	0	9.26364E-08	0	3.95455E-08	4.59091E-08	4.38E-08	7.21333E-08	8.50667E-08	1.532E-07

Pseudomonas_sp._gcc21	0	9.54545E-09	2.87273E-08	2.41545E-08	9.84615E-08	0	5.36923E-08	1.83308E-07	1.90545E-07	2.83818E-07	2.17273E-07	1.41182E-07	0.000000119	1.83933E-07	0.000000154	1.86E-08
Pseudomonas_mosselii	1.48818E-07	0	3.70909E-08	1.23636E-07	7.29231E-08	0	7.45385E-08	0.000000011	7.18182E-08	3.89091E-08	0	5.70909E-08	2.64E-08	1.61333E-08	0	0
Pseudomonas_alcaligenes	7.37273E-08	2.34545E-08	3.70909E-08	1.44364E-07	6.19231E-08	0	1.64923E-07	2.39615E-07	3.75455E-08	1.22182E-07	2.85909E-07	3.22818E-07	4.88E-08	0.000000014	8.27333E-08	0
Pseudomonas_balearica	0	0.00000039	3.27273E-08	2.34636E-07	3.15385E-08	2.13846E-08	0	8.28462E-08	4.45455E-08	3.62727E-08	1.02727E-08	7.12727E-08	0	0.000000033	0	0
Pseudomonas_fuscovaginae	1.24545E-07	2.70909E-08	6.98182E-08	0	0	0	0	0	4.45455E-08	0.000000056	2.76364E-08	7.13636E-09	0	0	4.44E-09	0
Pseudomonas_versuta	2.70909E-08	0	2.96364E-08	5.18182E-09	0	0	0	0	0	0	0	9.83636E-08	0	0	0	0
Pseudomonas_xinjiangensis	8.68182E-08	0.000000177	1.56545E-07	1.80455E-07	0	6.76923E-08	1.04077E-07	1.88769E-07	4.32727E-08	0.000000034	0	2.85455E-08	0	0.000000014	0	0
Pseudomonas_azotoformans	5.57273E-08	0	2.96364E-08	3.24545E-08	2.05385E-08	0	1.43692E-07	4.67692E-08	2.72727E-08	3.89091E-08	0	0	0	3.35333E-08	1.98867E-07	0.000000132
Pseudomonas_lini	4.29091E-08	0	0	2.43636E-08	0	0	0	0	0	0	0	2.85455E-08	0	0	0	0
Pseudomonas_otitidis	4.29091E-08	0	3.70909E-08	1.47273E-08	2.05385E-08	2.13846E-08	0	0.000000077	2.72727E-08	0	2.05455E-08	2.85455E-08	2.14E-08	0	1.12667E-08	7.10667E-08
Pseudomonas_lalkuanensis	2.92273E-07	0	0	1.91818E-08	0	0	0	7.28462E-08	2.16364E-08	3.89091E-08	1.45909E-07	7.13636E-09	0	5.24E-08	0	4.34E-08
Pseudomonas_syringae_group_genomosp._3	1.58182E-08	0	1.63636E-08	5.00909E-08	0	0	7.92308E-08	0	0	0	0	0	2.64667E-08	0	0	0
Pseudomonas_sp._ACM7	1.06273E-07	0	0	3.07273E-08	0	0	5.26154E-08	0	0	5.67273E-08	6.88182E-08	7.13636E-09	0	2.38E-08	4.44E-09	0
Pseudomonas_sp._IzPS59	6.85455E-08	0	0	0	5.20769E-08	4.33846E-08	0	0	7.95455E-08	3.03636E-08	7.70909E-08	3.94545E-08	0	1.02333E-07	5.36667E-08	3.97333E-08
Pseudomonas_sp._S35	1.58182E-08	0	0	0	0	0	0	0	0	0	0	0	0	0	0	0
Pseudomonas_sp._URMO17WK12:I11	9.30909E-08	0	0	1.69273E-07	5.79231E-08	1.14492E-06	2.11308E-07	1.08077E-07	5.42727E-08	9.40909E-08	8.77273E-08	1.17818E-07	1.646E-07	7.03333E-08	1.91333E-07	2.16533E-07
Pseudomonas_sp._IzPS32d	3.15455E-08	0.000000047	0	0	3.58462E-08	1.13538E-07	3.11538E-08	2.46923E-08	9.44545E-08	0	9.44545E-08	6.41364E-08	0	0	4.44E-09	0
Pseudomonas_sp._ABC1	1.32727E-07	1.46818E-07	3.70909E-08	2.97273E-08	1.10462E-07	5.91538E-08	9.48462E-08	4.93846E-08	3.75455E-08	2.87273E-08	2.76364E-08	7.13636E-09	0	0	4.64667E-08	3.72E-08
Pseudomonas_protegens	2.44545E-07	0	3.70909E-08	9.52727E-08	7.16923E-08	8.05385E-07	1.08077E-07	7.20769E-08	4.91818E-08	0	3.68364E-07	2.14636E-07	0	3.01333E-08	1.57067E-08	3.67333E-08
Pseudomonas_salegens	1.58182E-08	0	0.000000047	5.16364E-08	5.85385E-08	0	2.73846E-08	3.22308E-08	0	0	2.42364E-07	1.34636E-07	0	3.50667E-08	0	0
Pseudomonas_sp._02C_26	2.56364E-08	0	1.96636E-07	7.88182E-08	0	0	0	0	1.93273E-07	0	6.68182E-08	0.000000057	4.24E-08	3.11333E-08	2.69067E-08	0
Pseudomonas_rhizosphaerae	0	3.23182E-08	0	2.06609E-07	9.56923E-08	1.03246E-06	6.61692E-07	3.87308E-07	0.000000106	5.22727E-08	6.66364E-08	1.35727E-07	0	3.50667E-08	1.33333E-08	1.118E-07
Pseudomonas_sp._ADAK18	0	0	6.55455E-08	0	0	0	0	0	2.64545E-08	3.62727E-08	3.33636E-08	0	0	0	7.46667E-08	4.04E-08
Pseudomonas_sp._S1-A32-2	2.70909E-08	0	0	0	0	0	0	0	7.50909E-08	0	1.28182E-07	0.000000099	0	0	0	1.86E-08
Pseudomonas_sp._THAF7b	0	0	0	0	0	0	2.63077E-08	0	0	0	3.33636E-08	0	0	0	0	0
Pseudomonas_yamanorum	4.14545E-08	0	3.70909E-08	1.91818E-08	9.89231E-08	0	2.21692E-07	0.000000461	1.07273E-07	9.92727E-08	0.000000132	1.70727E-07	1.03667E-07	8.74667E-08	2.13973E-07	1.07133E-07
Pseudomonas_sihuiensis	9.63091E-07	1.04545E-08	0.0000005	1.14782E-06	9.67692E-08	2.92308E-07	8.65077E-07	1.17877E-06	7.17455E-07	6.65091E-07	4.61182E-07	3.19455E-07	3.616E-07	1.88333E-07	3.55733E-07	1.556E-07
Pseudomonas_xanthomarina	6.80909E-08	0	3.70909E-08	9.89091E-08	2.05385E-08	0	0	3.37692E-08	1.49727E-07	6.37273E-08	8.82727E-08	2.10636E-07	6.94E-08	0.000000028	2.38E-08	2.15333E-08
Pseudomonas_mendocina	4.63727E-07	1.78255E-06	3.12727E-07	1.42273E-07	7.26923E-08	3.25923E-06	3.53308E-07	3.00769E-07	2.91909E-07	5.08182E-07	5.10818E-07	7.70364E-07	1.992E-07	7.50667E-08	3.54267E-07	2.484E-07
Pseudomonas_sp._OIL-1	2.30909E-08	0	0	3.87273E-08	1.42615E-07	4.05077E-07	0	1.49077E-07	4.53455E-07	3.19273E-07	1.14818E-07	1.02591E-07	4.21533E-07	9.26E-08	0.000000275	6.51667E-07
Pseudomonas_sp._LTJR-52	2.70909E-08	0	0	0	0	0	6.44615E-08	0	2.75455E-08	5.22727E-08	4.92727E-08	0	0	4.64667E-08	2.97333E-08	0
Pseudomonas_sp._11K1	3.11818E-08	1.03636E-08	0	9.03636E-08	0	0	0	0	4.32727E-08	0	1.77909E-07	2.85455E-08	0	1.61333E-08	0	0
Pseudomonas_sp._MSPm1	0.000001025	0	0.000000408	1.08991E-06	8.98462E-08	2.93308E-07	3.77692E-08	9.31538E-08	6.35091E-07	1.43545E-07	5.37364E-07	4.80909E-07	3.56733E-07	2.69733E-07	1.19333E-07	1.37867E-07
Pseudomonas_sp._CMR5c	1.13182E-07	0	4.43273E-07	6.63636E-08	0.000000039	0	7.84615E-07	5.24615E-07	1.20364E-07	2.85455E-08	2.61273E-07	6.41364E-08	1.33133E-07	1.372E-07	0.000000142	2.80533E-07
Pseudomonas_brenneri	4.29091E-08	0	0	7.75455E-08	1.28231E-07	0	0	0	0	2.85455E-08	0.000000039	0	6.90667E-08	3.09333E-08	3.30667E-08	0
Pseudomonas_synxantha	0	0	8.75455E-09	0	0.000000039	0	3.03231E-07	1.35231E-07	0	0.000000028	6.88182E-08	0	0	7.66667E-08	2.97333E-08	3.67333E-08
Pseudomonas_sp._C27(2019)	5.29091E-08	0	1.75455E-08	4.47E-08	0	0	1.14923E-07	3.57692E-08	1.28636E-07	6.27273E-08	9.41818E-08	7.13636E-09	2.37333E-08	4.64667E-08	1.33333E-08	4.28E-08
Pseudomonas_sp._KUIN-1	0	0	5.25455E-08	0	0	0	0	0	0	0	0	0	0	0	0	0
Pseudomonas_frederiksbergensis	6.23636E-08	0	1.77273E-08	3.07273E-08	2.26923E-08	0	0	0	9.95455E-08	0	6.68182E-08	1.42727E-08	1.17733E-07	0	6.43333E-08	8.48667E-08
Pseudomonas_mediterranea	9.11818E-08	4.32909E-07	8.00909E-08	3.13273E-07	0	0	3.33077E-08	0	0	0	2.76364E-08	4.21364E-08	2.64E-08	0	5.88E-08	0
Pseudomonas_brassicacearum	3.69091E-08	1.80545E-07	5.01273E-08	1.82273E-07	5.61538E-08	1.73692E-07	9.61538E-08	0	6.14545E-08	1.03545E-07	3.33636E-08	7.37727E-08	2.72E-08	4.84667E-08	3.52E-08	0
Pseudomonas_silesiensis	2.48727E-07	4.66818E-07	1.21309E-07	1.47273E-08	0	0	9.53077E-08	9.89231E-08	0	1.56364E-07	3.77273E-08	4.63545E-07	6.08E-08	0	1.39933E-07	1.66667E-08
Pseudomonas_graminis	2.30909E-08	1.37273E-08	8.25455E-09	2.48973E-07	1.59692E-07	2.05077E-07	5.34846E-07	6.05385E-08	7.62727E-08	0.000000195	2.76364E-08	3.17455E-07	6.64667E-08	1.73067E-07	3.92133E-07	3.82E-08
Pseudomonas_knackmussii	4.94273E-07	0.000000749	9.49091E-08	7.43545E-07	0	0	0	4.93846E-08	8.54545E-08	0	0	9.27273E-08	0	4.64667E-08	1.28667E-07	0
Pseudomonas_furukawaii	1.76809E-06	1.49182E-07	5.83309E-07	1.01809E-06	3.97308E-07	1.14308E-05	2.61085E-06	4.16769E-06	1.52918E-06	0.000000815	1.34391E-06	1.01227E-06	0.000000992	6.19667E-07	8.13733E-07	9.65667E-07
Pseudomonas_sp._09C_129	3.69091E-08	4.39273E-07	4.12727E-09	0	0	0.000000065	0	0	0	0	0	0	0	0	2.39333E-08	1.86E-08
Pseudomonas_sp._ATCC_13867	2.09545E-07	1.68182E-08	8.25455E-09	0	0	4.49538E-07	3.06923E-08	0.000000042	3.98182E-08	0.000000028	0	0.000000282	2.72E-08	2.38E-08	1.49333E-08	1.13667E-07
Pseudomonas_sp._DTU12.3	3.11818E-08	0	1.56364E-07	3.77545E-07	3.08769E-07	0	6.84615E-08	4.32308E-08	1.36636E-07	1.37091E-07	0.000000167	1.70227E-07	5.09333E-08	6.26E-08	2.99733E-08	0
Pseudomonas_asturiensis	0.000000088	1.68182E-08	4.12727E-09	4.79091E-09	0	0	6.41538E-08	0	0	0	0	4.65909E-08	2.14667E-08	0	1.12667E-08	0
Pseudomonas_viridiflava	6.25455E-08	2.95909E-07	8.25455E-09	0	2.05385E-08	1.53077E-07	0	3.57692E-08	8.80909E-08	3.89091E-08	1.34364E-07	4.59091E-08	4.58667E-08	6.39333E-08	2.97333E-08	4.78667E-08
Pseudomonas_cedrina	1.96364E-07	0.000000065	4.12727E-09	7.97273E-08	0.000000039	1.89231E-07	9.29231E-08	4.02615E-07	1.26727E-07	0	0	3.31318E-07	2.14E-08	3.50667E-08	5.084E-08	1.86E-08
Pseudomonas_rhodesiae	2.56364E-08	0	2.59818E-08	3.07273E-08	0	6.53077E-08	0	0	0	0	0	7.13636E-09	0	0	0	0
Pseudomonas_citronellolis	5.12727E-08	0	1.33636E-07	1.43636E-08	5.76154E-08	0	0	4.76231E-07	7.61818E-08	0.000000028	0	1.55364E-07	0	2.62E-08	1.27533E-07	1.50267E-07
Pseudomonas_sp._13159349	6.31636E-07	1.38818E-07	1.33636E-07	0.000000079	1.25615E-07	9.46154E-09	0	5.96154E-08	2.35364E-07	3.22636E-07	0.000000048	2.72364E-07	1.142E-07	0.000000014	4.884E-08	8.53333E-08
Pseudomonas_oryzihabitans	1.12455E-07	4.37727E-07	1.64409E-07	2.69636E-07	5.74615E-08	3.91692E-07	1.36231E-07	0.000000293	0.000000054	9.11818E-08	2.05455E-08	9.03636E-08	0	9.34E-08	0	9.67333E-08
Pseudomonas_sp._S09G_359	0	0	1.24455E-08	0	0	0	0	0	0	0	0	0	0	0	0	0
Pseudomonas_resinovorans	1.06909E-07	1.57727E-07	0.000000533	1.54518E-07	3.03615E-07	0	0	0	3.44909E-07	3.81182E-07	2.14273E-07	4.37273E-07	2.71667E-07	6.17267E-07	3.978E-07	4.014E-07
Pseudomonas_denitrificans_(nom._rej.)	1.18091E-07	0	0	0	0	0	3.06923E-08	0.000000011	7.50909E-08	3.62727E-08	3.33636E-08	2.85455E-08	0	5.19333E-08	1.12667E-08	0
Pseudomonas_alcaliphila	8.09091E-08	2.08182E-08	2.18545E-08	9.28818E-08	6.73846E-08	2.77385E-07	2.94462E-07	5.81538E-08	2.16364E-08	0	1.71182E-07	1.42727E-08	7.43333E-08	8.34667E-08	1.282E-07	1.04667E-07
Pseudomonas_sediminis	1.03818E-07	0	0	0	0	0	0	0	0.000000067	0	0	1.45955E-07	5.16E-08	3.48E-08	1.49333E-08	0.00000011
Pseudomonas_saudiphocaensis	0.000000258	0	5.34545E-08	1.00818E-07	8.20769E-08	2.87538E-07	3.11538E-08	7.76154E-08	0.000000111	7.02727E-08	1.19273E-07	0.000000244	0	1.132E-07	2.2904E-07	6.33333E-08
Pseudomonas_agarici	2.96909E-07	5.47273E-08	2.96364E-08	2.53273E-07	1.14154E-07	1.02462E-07	1.33846E-07	0	1.39455E-07	1.77364E-07	6.88182E-08	6.35455E-08	6.41333E-08	5.06E-08	7.61067E-08	2.00067E-07
Pseudomonas_parafulva	2.77273E-07	8.18182E-08	1.65455E-08	4.63818E-07	2.09385E-07	0	3.54615E-07	1.28538E-07	4.71182E-07	1.66545E-07	9.64545E-08	2.79545E-07	3.42667E-07	4.10667E-07	3.46467E-07	0.000000285
Pseudomonas_syringae	0.00000223	7.61364E-07	7.53118E-07	9.95455E-07	6.63231E-07	1.87385E-06	3.85538E-07	4.69923E-07	1.08882E-06	6.00364E-07	1.11873E-06	1.64973E-06	3.31533E-07	5.69867E-07	0.000000837	9.17467E-07
Pseudomonas_coronafaciens	3.35155E-06	8.38636E-07	3.06045E-06	1.84236E-06	3.21769E-07	5.86769E-07	2.55646E-06	3.41077E-06	1.20027E-06	1.49027E-06	1.96082E-06	2.44373E-06	2.59467E-07	3.82733E-07	5.66467E-07	0.000000514
Pseudomonas_marincola	5.29091E-08	0	0	3.19091E-07	3.15385E-08	2.13846E-08	5.26154E-08	4.44615E-08	0	0.000000034	6.71818E-08	1.42727E-08	9.19333E-08	1.838E-07	1.19307E-07	1.368E-07
Pseudomonas_prosekii	6.37727E-07	0.000000112	7.81818E-08	3.19273E-07	3.17692E-07	0	2.63077E-08	1.01923E-07	6.58182E-07	4.63818E-07	1.22182E-07	5.38545E-07	3.906E-07	3.58933E-07	2.73933E-07	4.844E-07
Pseudomonas_aeruginosa	5.51364E-07	1.07427E-06	6.33545E-07	6.51091E-07	8.96692E-07	1.45262E-06	1.01108E-06	1.22646E-06	6.52818E-07	6.88818E-07	6.25727E-07	1.09936E-06	6.28467E-07	7.60333E-07	7.04533E-07	8.24867E-07
Pseudomonas_oryzae	5.56909E-07	1.98727E-07	2.06364E-07	2.66636E-07	5.34462E-07	5.29769E-07	5.57077E-07	3.47462E-07	4.69455E-07	1.61091E-07	6.72273E-07	0.00000075	6.82E-08	3.698E-07	2.38733E-07	4.19867E-07
Pseudomonas_psychrotolerans	2.69182E-07	0.000000384	1.12545E-07	4.39091E-08	1.61462E-07	1.00262E-06	8.33077E-08	3.02538E-07	5.77818E-07	7.28455E-07	8.92091E-07	8.63818E-07	3.36933E-07	9.13067E-07	6.57533E-07	2.96933E-07
Pseudomonas_chlororaphis	0.000001372	3.83364E-07	3.67636E-07	1.48564E-06	6.43308E-07	4.61308E-07	8.20385E-07	1.08723E-06	6.90182E-07	9.85364E-07	8.30818E-07	6.97545E-07	7.088E-07	1.06967E-06	9.31733E-07	1.5358E-06
Pseudomonas_alkylphenolica	0.000000869	0	0	3.24545E-08	1.17592E-06	0	4.80769E-08	0	1.53382E-06	1.86782E-06	6.99818E-07	1.37764E-06	2.05673E-06	1.40893E-06	1.56433E-06	2.28633E-06
Pseudomonas_entomophila	1.33473E-06	5.785E-07	2.38864E-07	3.83636E-07	1.19146E-06	5.07231E-07	2.96385E-07	6.52846E-07	1.10818E-06	8.51636E-07	9.67545E-07	1.75191E-06	1.62067E-06	1.0974E-06	1.05947E-06	1.58647E-06
Pseudomonas_stutzeri	0.00000339	1.58936E-06	8.88664E-07	2.72309E-06	1.46877E-06	3.96492E-06	4.34308E-06	4.87892E-06	3.36855E-06	1.37591E-06	2.08518E-06	5.11091E-06	0.000000902	1.5146E-06	1.37787E-06	1.08433E-06
Pseudomonas_monteilii	2.64182E-07	9.54545E-09	1.65455E-08	1.15282E-06	1.63154E-07	1.38938E-06	2.55192E-06	1.79162E-06	5.04636E-07	3.05545E-07	6.04364E-07	5.18364E-07	2.94067E-07	2.374E-07	1.45667E-07	3.446E-07
Pseudomonas_fulva	1.33091E-07	3.75909E-08	0	4.79091E-09	3.89538E-07	6.08692E-07	6.57538E-07	2.07077E-07	1.04545E-07	0.000000047	1.04364E-07	6.21818E-08	5.458E-07	5.60067E-07	8.30067E-07	0.000000313
Pseudomonas_putida	1.44736E-06	5.70455E-07	1.13945E-07	4.44182E-07	1.82854E-06	9.17231E-07	0.000000329	7.12154E-07	1.56827E-06	1.67364E-06	1.82364E-06	0.000001831	1.99767E-06	1.68687E-06	2.57907E-06	2.82067E-06
Pseudomonas_sp._7SR1	0	0	6.12727E-08	1.71455E-07	2.59231E-08	0	2.63077E-08	0	1.54818E-07	2.21273E-07	2.76364E-08	1.815E-07	1.86667E-08	3.48E-08	0	0
Pseudomonas_sp._MRSN12121	0	0	0	0.000000042	0	0	0	0	0	0	0	7.13636E-09	2.24E-08	3.48E-08	0	4.04E-08
Pseudomonas_sp._M30-35	0	0	0	0	7.76923E-08	0	2.40231E-07	0	0.000000064	0.000000034	0	0	0	9.66E-08	1.88333E-07	1.86E-08
Pseudomonas_sp._SNU_WT1	0.000000061	8.05455E-08	4.90909E-08	2.25455E-07	3.34615E-08	8.05231E-07	0.000000139	2.82923E-07	3.75455E-08	3.89091E-08	2.76364E-08	0.000000057	1.26933E-07	3.48E-08	1.95707E-07	8.51333E-08
Pseudomonas_sp._BIGb0427	0.00000006	0	0	0	4.13846E-08	3.15385E-08	0	0	0	9.11818E-08	6.71818E-08	1.80182E-07	0	3.48E-08	6.04E-08	5.27333E-08
Pseudomonas_sp._NS1(2017)	8.09818E-07	0	0	1.86182E-07	3.15385E-07	0	9.48462E-08	0.000000675	3.27636E-07	4.52091E-07	1.76727E-07	4.37227E-07	2.354E-07	4.13267E-07	4.74933E-07	6.04267E-07
Pseudomonas_sp._S34	1.96091E-07	0	8.75455E-09	1.96345E-06	2.07231E-07	3.29662E-06	6.25692E-06	3.93615E-06	6.73273E-07	9.39455E-07	1.64064E-06	8.15273E-07	1.93733E-07	3.18867E-07	3.648E-07	7.96E-08
Pseudomonas_sp._UW4	2.02273E-07	0	1.92727E-07	3.16345E-06	7.42308E-07	0.000001237	1.24069E-06	3.45423E-06	4.54364E-07	5.15545E-07	1.65655E-06	2.37836E-06	3.41067E-07	1.06207E-06	1.66627E-06	6.994E-07
Pseudomonas_sp._HLS-6	1.93545E-07	0.00000013	7.81818E-08	5.41909E-07	3.28769E-07	6.33846E-07	1.05692E-07	4.32308E-08	2.82091E-07	3.18455E-07	6.15545E-07	1.11909E-07	1.43467E-07	6.01933E-07	7.17667E-07	2.06667E-07
Pseudomonas_sp._NC02	3.11818E-08	1.94545E-07	0	2.36518E-07	2.46385E-07	0	0	2.12923E-07	2.77909E-07	4.65182E-07	7.29091E-08	6.09091E-08	1.71867E-07	3.052E-07	2.366E-07	0.000000292
Pseudomonas_veronii	0	0	0	4.79091E-09	0	0	0	3.22308E-08	2.72727E-08	0	0	0	0	3.48E-08	0	0
Pseudomonas_orientalis	0	0	0	2.99091E-08	0	0	1.18846E-07	2.51538E-07	2.08091E-07	1.54909E-07	3.34545E-07	1.23727E-07	2.14667E-08	1.576E-07	1.27933E-07	2.79333E-08
Pseudomonas_fluorescens	8.89636E-07	4.66636E-07	2.97282E-07	8.83909E-07	4.61538E-07	1.14523E-06	8.61692E-07	1.20792E-06	7.23818E-07	4.01091E-07	1.26782E-06	1.31136E-06	8.046E-07	4.692E-07	6.58467E-07	0.000001013
Pseudomonas_corrugata	5.61727E-07	0.000000065	3.07364E-07	7.26727E-07	3.04154E-07	9.46154E-08	4.16846E-07	5.97462E-07	8.61091E-07	0.00000066	9.37273E-07	1.82545E-07	3.21467E-07	3.278E-07	3.90667E-07	3.36667E-07
Pseudomonas_tolaasii	4.33117E-05	1.09424E-05	1.18027E-05	2.84464E-05	8.08077E-06	1.44189E-05	0.000100134	2.14692E-05	1.47518E-05	9.08636E-06	1.74264E-05	0.00002229	0.000029583	0.00000876	0.000012024	1.50053E-05
Limnobaculum_parvum	7.26364E-08	0	0	0	1.19462E-07	5.12308E-08	7.92308E-08	0	7.95455E-08	0.000000056	6.71818E-08	4.19727E-07	0	1.58E-08	0	1.86E-08
Leminorella_richardii	9.82727E-08	0	8.80909E-08	0.000000329	0	0	3.11538E-08	1.18385E-07	2.98909E-07	5.72727E-08	2.74545E-07	1.81909E-07	0	1.30933E-07	1.08507E-07	0.000000132
Pragia_sp._CF-458	1.58182E-08	3.20455E-07	3.27273E-08	0	0	8.84615E-08	3.77692E-08	1.36692E-07	0	0	3.95455E-08	0.000000097	0	4.33333E-08	5.94E-08	0
Pragia_fontium	5.42727E-08	0	4.12727E-09	2.89091E-08	0	3.09231E-07	1.17077E-07	7.15385E-08	7.62727E-08	1.13091E-07	1.41182E-07	3.38273E-07	6.41333E-08	1.71333E-08	1.05667E-07	0
Plesiomonas_shigelloides	3.69091E-07	1.76182E-07	1.47455E-07	3.28727E-07	1.82692E-07	1.27792E-06	5.02231E-07	1.85769E-07	2.00636E-07	3.11273E-07	2.91727E-07	2.33364E-07	2.23733E-07	2.90467E-07	3.44733E-07	4.146E-07
Obesumbacterium_proteus	0	0	8.75455E-09	0	0	2.44608E-06	0	0	0	0.000000047	6.68182E-08	0	2.37333E-08	1.61333E-08	0	0
Hafnia_sp._CBA7124	0	0	0	0	0	0	0	0	0	0	0	0	0	0	0	6.33333E-08
Hafnia_paralvei	6.65455E-08	0.000000065	1.63636E-08	5.18182E-09	8.98462E-08	3.54615E-08	0	9.62308E-08	3.14909E-07	2.28455E-07	1.73545E-07	0	0	0.000000047	7.46667E-08	0
Hafnia_alvei	0	0	9.38909E-07	8.71818E-08	0	0	0	0	0	1.44727E-07	1.08364E-07	1.76182E-07	0	0	1.93733E-08	0
Edwardsiella_sp._EA181011	0	0	0	0	0	0	0	0	0	0	0	0	2.14667E-08	0	0	0
Edwardsiella_tarda	5.57273E-08	0	0	1.03636E-08	0	0	0	0	0	0	0	0	2.24E-08	3.09333E-08	0	0
Edwardsiella_hoshinae	9.93636E-08	0	0	4.79091E-09	1.57923E-07	0	9.38462E-08	1.90769E-08	1.55818E-07	0.000000109	1.60545E-07	1.44545E-07	2.15867E-07	2.868E-07	1.692E-07	5.89133E-07
Edwardsiella_ictaluri	1.04455E-07	0	2.64818E-08	2.16909E-07	5.63846E-08	2.95385E-08	1.25154E-07	2.80538E-07	0	0	0	2.14545E-08	7.96E-08	4.49333E-08	9.46E-08	0
Edwardsiella_piscicida	0	1.16364E-08	4.29091E-08	6.42727E-08	0	1.89231E-08	3.77692E-08	0	3.98182E-08	2.85455E-08	1.33636E-07	3.94545E-08	1.19133E-07	3.22667E-08	1.89133E-07	0.0000002
Lonsdalea_britannica	1.81091E-07	2.00864E-07	1.45818E-07	1.73909E-07	8.79231E-08	8.00385E-07	6.23077E-08	2.47692E-08	1.28636E-07	0	6.88182E-08	4.79091E-08	4.58667E-08	6.12667E-08	2.81133E-07	1.02733E-07
Brenneria_goodwinii	1.12455E-07	0.000000065	0	1.73273E-07	3.92308E-08	3.11846E-07	5.26154E-08	8.46154E-08	2.52818E-07	1.56636E-07	1.37091E-07	3.94273E-07	1.118E-07	0.00000007	2.40707E-07	8.06667E-08
Brenneria_rubrifaciens	4.79909E-07	0	0	2.51818E-07	5.39231E-07	2.39077E-07	2.42692E-07	2.79308E-07	0.000000875	1.06736E-06	4.15182E-07	9.60455E-07	2.50133E-07	4.08733E-07	4.48733E-07	2.54933E-07
Sodalis_praecaptivus	0	3.35455E-08	0	0	0	0	0	0	0	0	0	0	0	1.61333E-08	1.12667E-08	1.98667E-08
Candidatus_Sodalis_pierantonius	3.11818E-08	2.34545E-08	1.70091E-08	4.79091E-09	2.05385E-08	1.02462E-07	3.11538E-08	0.000000115	1.44636E-07	8.08182E-08	1.18636E-07	2.85455E-08	0	1.424E-07	0.000000048	8.19333E-08
Sodalis_endosymbiont_of_Henestaris_halophilus	0	0	0	1.91818E-08	1.21923E-07	0	0	0	0	0	0	7.75455E-08	9.00667E-08	3.48E-08	4.36667E-07	1.00067E-07
Sodalis_glossinidius	5.48818E-07	1.03273E-07	2.59727E-07	3.58727E-07	2.83769E-07	7.99077E-07	4.97231E-07	5.19923E-07	6.17091E-07	6.97545E-07	9.70545E-07	1.08409E-06	2.54533E-07	4.84333E-07	6.63067E-07	4.09067E-07
Pectobacterium_wasabiae	0	0	0	0	0	0	0	0	0	0	0	0	2.94667E-08	0.000000033	0	4.95333E-08
Pectobacterium_punjabense	0	0	0	5.18182E-09	0	2.95385E-08	0	0	0	0	0	7.13636E-09	0	0	0	0
Pectobacterium_atrosepticum	0	4.39091E-08	0	2.87273E-08	5.95385E-08	2.13846E-08	0	0	0	0	0	0.000000113	0	5.22E-08	4.83067E-08	0
Pectobacterium_odoriferum	1.53636E-07	0.000000177	3.27273E-08	1.85818E-07	1.03692E-07	0	5.26154E-08	1.90769E-08	4.45455E-08	2.08273E-07	4.92727E-08	0.00000005	0.000000027	4.94E-08	7.45067E-08	4.34E-08
Pectobacterium_versatile	2.21727E-07	2.81455E-07	6.62091E-08	3.40273E-07	4.93846E-08	0	3.77692E-08	7.40769E-08	2.14364E-07	3.73636E-07	3.55727E-07	1.50773E-07	0	1.71333E-08	0	8.31333E-08
Pectobacterium_brasiliense	9.27273E-08	0.00000013	2.62727E-08	0.00000012	8.85385E-08	6.43154E-07	2.63077E-08	0	1.04545E-07	1.56364E-07	2.39909E-07	1.13273E-07	1.62333E-07	4.51333E-08	0.000000182	1.03067E-07
Pectobacterium_carotovorum	2.56364E-08	2.34545E-08	1.11545E-07	8.31545E-08	6.76923E-08	2.58308E-07	3.11538E-08	1.17154E-07	2.75455E-08	0.000000047	7.70909E-08	1.48909E-07	6.44667E-08	0.000000033	5.54E-08	0
Pectobacterium_polaris	8.24545E-08	9.80909E-08	9.97273E-08	4.77091E-07	1.07154E-07	0	0	4.10769E-08	2.72727E-08	1.49545E-07	1.97909E-07	1.79909E-07	6.90667E-08	1.14133E-07	8.47333E-08	1.86E-08
Pectobacterium_parmentieri	2.55727E-07	3.59545E-08	3.70909E-08	5.79455E-07	6.67154E-07	2.52308E-08	2.75923E-07	6.97538E-07	1.20655E-06	4.39727E-07	1.18482E-06	1.15082E-06	1.1116E-06	6.84133E-07	0.000000657	8.27733E-07
Dickeya_fangzhongdai	0	0	0	0	0	0	0	0	0	7.57273E-08	1.61273E-07	7.13636E-09	0	0.000000014	0	1.978E-07
Dickeya_dianthicola	8.69091E-08	7.66364E-08	1.63636E-08	7.79091E-08	9.84615E-08	2.95385E-08	1.19308E-07	1.84923E-07	2.64545E-08	2.86455E-07	1.35636E-07	9.59091E-08	2.14E-08	3.50667E-08	6.41067E-08	8.85333E-08
Dickeya_sp._NCPPB_3274	0.000000151	1.16364E-08	2.96364E-08	2.03882E-07	1.05231E-07	1.29269E-06	3.98538E-07	2.93846E-08	4.45455E-08	5.65455E-08	3.77273E-08	1.36273E-07	7.69333E-08	1.16867E-07	1.836E-07	1.716E-07
Dickeya_solani	2.70909E-08	0.00000013	7.97273E-08	2.77364E-07	2.76692E-07	9.29231E-08	2.21231E-07	3.80308E-07	2.90273E-07	1.68818E-07	3.28455E-07	4.07818E-07	3.38467E-07	3.214E-07	3.074E-07	2.58333E-07
Dickeya_chrysanthemi	1.04909E-07	0	4.12727E-09	4.79091E-09	3.58462E-08	0	0	1.16154E-07	1.02636E-07	1.50636E-07	3.77273E-08	1.31545E-07	5.86E-08	4.67333E-08	3.41733E-08	1.86E-08
Dickeya_paradisiaca	2.13091E-07	4.57727E-07	6.14545E-08	0.000000367	0.000000039	3.46923E-08	0	3.11077E-07	2.42364E-07	4.64182E-07	1.63091E-07	5.59909E-07	2.72E-08	1.04667E-07	1.81933E-07	1.86667E-08
Dickeya_poaceiphila	1.12091E-07	1.93182E-08	6.62182E-08	1.89545E-07	8.33846E-08	0	0	0	5.34545E-08	6.37273E-08	2.76364E-08	0.0000001	0	0	4.44E-09	3.08E-08
Dickeya_zeae	1.32982E-06	8.55091E-07	6.75955E-07	1.37945E-06	7.21615E-07	1.03785E-06	1.02477E-06	1.51762E-06	8.54818E-07	1.28964E-06	1.62082E-06	1.91455E-06	2.22667E-07	3.52533E-07	4.69867E-07	6.93067E-07
Dickeya_dadantii	1.95909E-07	3.04364E-07	6.23636E-08	6.64545E-08	0	3.15385E-08	0	9.76154E-08	4.45455E-08	1.04545E-07	7.69091E-08	1.21455E-07	0	3.48E-08	5.86667E-08	1.22E-08
Photorhabdus_asymbiotica	0	0	1.10909E-07	4.79091E-09	0	0	0	0	0	0	0.000000048	0	0	3.50667E-08	2.39333E-08	0
Photorhabdus_laumondii	3.11818E-08	0	1.28818E-08	1.93636E-08	7.16923E-08	0	7.66923E-08	5.13077E-08	1.13182E-07	1.43545E-07	0	1.71955E-07	0	9.73333E-08	2.97333E-08	1.86E-08
Photorhabdus_thracensis	0	1.18273E-07	0	1.91818E-08	4.78154E-07	0	3.06923E-08	9.03846E-08	3.94545E-08	0	9.41818E-08	1.35091E-07	1.278E-07	2.538E-07	1.332E-07	3.41267E-07
Xenorhabdus_bovienii	3.89182E-07	0.00000013	8.21818E-08	2.07273E-08	3.79385E-07	8.53846E-08	6.89231E-08	4.93846E-08	3.79818E-07	4.19636E-07	0.00000019	4.19455E-07	4.672E-07	2.93333E-07	2.94667E-07	1.14267E-07
Xenorhabdus_poinarii	4.23909E-07	4.40364E-07	6.22091E-08	0.000000324	2.46385E-07	0	1.83846E-07	3.56923E-08	1.58909E-07	5.67273E-08	4.75182E-07	7.03818E-07	3.17733E-07	5.92067E-07	2.82067E-07	4.826E-07
Xenorhabdus_doucetiae	3.09636E-07	2.32636E-07	1.06945E-07	1.19091E-07	1.00615E-07	8.92308E-08	7.66923E-08	3.19692E-07	4.27909E-07	8.02727E-08	1.45727E-07	4.91818E-07	4.28667E-08	1.228E-07	2.90667E-07	4.85533E-07
Xenorhabdus_nematophila	4.68364E-07	4.39091E-08	4.12182E-08	0.00000044	4.69538E-07	1.03038E-06	4.10769E-07	5.15154E-07	7.70909E-07	4.23636E-07	5.62545E-07	1.25627E-06	1.68667E-07	4.22867E-07	3.74333E-07	7.33467E-07

Xenorhabdus_hominickii	0	0	0	7.31818E-08	1.02308E-07	0	0	0	0	0	2.76364E-08	3.87727E-08	6.76E-08	1.27467E-07	5.01333E-08	4.95333E-08
Arsenophonus_endosymbiont_of_Aleurodicus_dispersus	0	0	0	4.79091E-09	2.26923E-08	0	0	0	0	0	0	0.000000035	0	0	1.49333E-08	0
Candidatus_Arsenophonus_lipoptenae	0	0	0	0	0	0	0	0	0	0	0	7.13636E-09	0	0	4.44E-09	4.04E-08
Arsenophonus_endosymbiont_of_Aphis_craccivora	2.12091E-07	5.25773E-07	6.58182E-08	2.40636E-07	0	0	0	2.46923E-08	1.77091E-07	3.44273E-07	2.50455E-07	2.26091E-07	8.56E-08	0	5.084E-08	6.92667E-08
Arsenophonus_nasoniae	3.51182E-07	0	1.85455E-07	8.61818E-08	7.17692E-08	0	1.21462E-07	3.13846E-07	1.76455E-07	1.28727E-07	9.13273E-07	1.22727E-06	4.24E-08	3.15333E-08	1.64573E-07	0
Morganella_morganii	6.95909E-07	7.45455E-07	4.65255E-07	5.33182E-07	2.83308E-07	2.09308E-07	5.24846E-07	8.71077E-07	0.000000459	0.000000599	6.88545E-07	0.000001026	0.000000373	4.24267E-07	4.916E-07	0.0000006
Proteus_columbae	2.96364E-08	1.68182E-08	0	1.93636E-08	2.88462E-08	0	0	0	0	0	0	0	0	0	0	0
Proteus_cibarius	0	0	1.75455E-07	0	0	0	0	0	0	0	0	0	0	0	0	0
Proteus_mirabilis	9.29818E-07	2.08182E-08	2.41027E-07	1.93091E-07	7.17692E-08	5.36308E-07	5.26154E-08	2.28769E-07	5.36182E-07	3.40727E-07	7.98545E-07	7.47727E-07	1.48067E-07	1.972E-07	6.12667E-08	1.266E-07
Proteus_sp._GOKU	7.67455E-07	0	0	9.74427E-07	2.59662E-06	0	5.60769E-07	0.000001572	5.46555E-06	2.21545E-06	3.60255E-06	1.12709E-06	3.0862E-06	0.000001411	2.67913E-06	1.8244E-06
Proteus_vulgaris	1.27655E-06	4.00455E-07	5.59545E-07	0.000000966	7.19077E-07	0	1.28023E-06	1.18977E-06	1.41873E-06	1.30391E-06	1.87927E-06	0.000000896	8.846E-07	1.34647E-06	9.84467E-07	9.76667E-07
Providencia_stuartii	0	0	0	0	0	0	0	0	1.19091E-07	0	0	0	2.09333E-08	0.000000168	1.77067E-07	1.86E-08
Providencia_vermicola	9.93636E-08	0	0	9.54545E-09	0	1.00908E-06	4.80769E-08	2.56923E-08	7.18182E-08	1.22818E-07	1.69545E-07	4.59091E-08	6.09333E-08	4.94E-08	1.95333E-07	1.78933E-07
Providencia_rettgeri	2.98182E-08	9.54545E-09	3.70909E-08	1.05545E-07	2.05538E-07	0	0	2.14692E-07	1.19636E-07	1.27455E-07	0.000000303	1.26091E-07	1.29333E-07	2.20733E-07	3.278E-07	1.10933E-07
Providencia_heimbachae	1.63364E-07	0.000000258	1.22364E-07	1.43727E-07	0	2.52308E-08	7.40769E-08	1.77462E-07	8.49091E-08	1.91909E-07	1.74727E-07	3.80182E-07	0	1.61333E-08	4.82E-08	1.23733E-07
Providencia_sneebia	8.39091E-08	4.27182E-07	0	1.55455E-08	6.17692E-08	1.99538E-07	0	0	5.79091E-08	0	3.42727E-07	4.35136E-07	0	1.58E-08	4.44E-09	0
Providencia_rustigianii	4.56364E-08	0	0	0	1.35462E-07	0	0	1.90769E-08	1.09182E-07	9.40909E-08	0.000000039	0	5.32E-08	3.22667E-08	3.14667E-08	6.14E-08
Providencia_sp._WCHPHu000369	0	0.0000001	4.12727E-09	0	4.04462E-07	1.99308E-07	3.11538E-08	0	0.00000059	1.32636E-07	1.37273E-07	1.58045E-07	1.09533E-07	3.568E-07	3.36867E-07	2.53667E-07
Providencia_alcalifaciens	1.37982E-06	9.94818E-07	0.000000496	8.02818E-07	1.10408E-06	0.000001661	1.77992E-06	4.44308E-07	1.30782E-06	1.21591E-06	1.17791E-06	1.10891E-06	3.63267E-07	6.89067E-07	6.094E-07	6.12533E-07
Candidatus_Fukatsuia_symbiotica	2.70909E-08	0	6.98182E-08	1.10273E-07	0	0	0	1.90769E-08	0	0.000000163	6.88182E-08	2.14545E-08	5.36E-08	0	0	3.85333E-08
Rouxiella_badensis	6.13636E-08	1.68182E-08	0	4.79091E-09	0	6.53077E-08	0	0	0	3.03636E-08	0	7.13636E-09	0	3.50667E-08	1.12667E-08	4.76667E-08
Nissabacter_sp._SGAir0207	1.03355E-06	2.48955E-07	3.89091E-07	0.000000868	1.31692E-07	4.46923E-07	2.44077E-07	2.85231E-07	4.40727E-07	8.59091E-08	2.45727E-07	8.25909E-07	2.17067E-07	1.12667E-07	4.73133E-07	1.884E-07
Gibbsiella_quercinecans	0.000000397	0.000000075	4.12727E-09	2.82364E-07	3.58462E-08	0	2.63077E-08	5.22308E-08	1.40273E-07	3.03636E-08	2.79091E-07	4.59091E-08	3.74E-08	1.48067E-07	1.0664E-07	1.032E-07
Chania_multitudinisentens	1.69273E-07	6.80909E-08	1.85036E-07	4.97427E-07	2.13692E-07	0	0.000000148	1.14923E-07	8.20909E-08	1.73091E-07	1.21818E-07	2.06182E-07	4.81333E-08	8.48E-08	1.17133E-07	2.78733E-07
Yersinia_intermedia	0	0	0	0	3.58462E-08	0	0	0	0	0	0	0	0	0	0	0
Yersinia_similis	0	0	0	0	0	0	0	0	0	0	0	0	0	0	0	1.98667E-08
Yersinia_kristensenii	0	0	0	4.68818E-08	4.13846E-08	0	4.33846E-08	1.90769E-08	1.75182E-07	0	0	0	0	0	4.46667E-08	0
Yersinia_aleksiciae	0	0	0	3.24545E-08	0	0	0	0	2.72727E-08	0	0	0.000000057	0	0	6.82667E-08	0
Yersinia_hibernica	0	0	0	0	2.75923E-07	1.79231E-08	2.63077E-08	5.22308E-08	0	0	1.98182E-07	2.70455E-07	0	0	0	0
Yersinia_mollaretii	1.34091E-07	0.000000065	3.70909E-08	9.83636E-08	8.35385E-08	0	3.41769E-07	1.62231E-07	7.73636E-08	0.000000056	1.06545E-07	0.000000068	0	9.38E-08	2.022E-07	3.84667E-08
Yersinia_canariae	3.69091E-08	2.74545E-08	2.87273E-08	1.91818E-08	0	0	0	0	2.75455E-08	0.000000116	3.33636E-08	1.42727E-08	0	0.000000033	4.44E-09	1.86E-08
Yersinia_ruckeri	1.16545E-07	0	0	1.61427E-07	2.89769E-07	5.05385E-08	2.22769E-07	5.53846E-07	5.50909E-08	0.000000162	1.71909E-07	1.06273E-07	7.80667E-08	6.59333E-08	5.55333E-08	4.78E-08
Yersinia_rohdei	0	0	3.70909E-08	1.53455E-07	5.76154E-08	3.21308E-07	0	1.46923E-08	3.75455E-08	0	3.33636E-08	7.64545E-08	0	3.26667E-08	5.04667E-08	1.98667E-08
Yersinia_entomophaga	2.96364E-08	0	4.29091E-06	4.39091E-07	1.99077E-07	0	0	0	4.45455E-08	0	6.88182E-08	6.35455E-08	7.42667E-08	0.000000033	1.08707E-07	0
Yersinia_enterocolitica	5.56909E-07	5.63182E-07	1.57517E-05	1.50809E-06	0.000000334	8.87692E-08	4.15692E-07	1.44462E-07	1.66182E-07	2.05455E-07	4.26545E-07	4.65455E-07	0.000000142	2.542E-07	0.000000568	2.42667E-07
Yersinia_pseudotuberculosis	8.32727E-08	0.000000065	4.52727E-08	6.71818E-08	1.06462E-07	1.09462E-07	1.51538E-07	0	4.86364E-08	3.03636E-08	1.58273E-07	1.23455E-07	2.37333E-08	6.52667E-08	8.86667E-09	5.82667E-08
Yersinia_pestis	1.09545E-07	8.39091E-08	2.35636E-07	1.85909E-07	1.02769E-07	2.93769E-07	8.96923E-08	0.000000111	1.01182E-07	1.04545E-07	0.000000078	2.97091E-07	1.17667E-07	0.000000067	5.35333E-08	8.93333E-08
Yersinia_frederiksenii	2.56364E-08	0	8.35545E-08	9.54545E-09	0	2.52308E-08	3.33077E-08	3.22308E-08	3.94545E-08	5.22727E-08	0	7.71364E-08	6.08E-08	0	0.000000174	1.282E-07
Yersinia_aldovae	1.61091E-07	3.81545E-07	1.04455E-07	3.76364E-08	2.53385E-07	9.46154E-09	8.76923E-08	2.69231E-07	7.20909E-08	2.82909E-07	0	1.11455E-07	1.384E-07	4.26533E-07	9.84667E-08	3.48933E-07
Ewingella_americana	1.92545E-07	0	7.82182E-08	8.67364E-07	2.59769E-07	6.38923E-07	1.23538E-06	1.12408E-06	5.80364E-07	5.20364E-07	4.20364E-07	9.77091E-07	3.484E-07	1.98867E-07	9.28E-08	4.88733E-07
Rahnella_sp._ERMR1:05	2.16273E-07	6.13273E-07	8.61818E-08	4.11273E-07	0.000000109	6.75231E-07	8.37692E-08	1.86846E-07	1.19091E-07	1.38182E-07	1.59818E-07	4.11909E-07	1.30867E-07	0	6.564E-08	2.39533E-07
Rahnella_aquatilis	4.05091E-07	9.73182E-08	1.35673E-07	1.83909E-07	1.47462E-07	0	5.96154E-08	1.90077E-07	1.39182E-07	1.90091E-07	7.67273E-08	3.69273E-07	4.028E-07	1.078E-07	8.42667E-08	8.11333E-08
Rahnella_sp._Y9602	1.86273E-07	0.00000013	9.89091E-08	2.63364E-07	0.0000001	0	6.23077E-08	2.01538E-07	2.59364E-07	2.19273E-07	1.47091E-07	2.43273E-07	2.72333E-07	4.25933E-07	2.264E-07	3.15667E-07
Serratia_ureilytica	0	1.16364E-08	0	0	0	0	0	0	0	0	0	0	0	0	0	4.28E-08
Serratia_proteamaculans	0	0	0	0	0	0	0	0	0	0	0	3.94545E-08	0	0	0	0
Serratia_quinivorans	0	0	0	0	5.74615E-08	0	0	0	0	0.000000056	2.76364E-08	7.13636E-09	0	0	0	0
Serratia_symbiotica	0	4.65091E-07	3.70909E-08	1.11455E-07	4.13846E-08	3.83077E-08	5.54615E-08	1.01692E-07	7.50909E-08	2.18364E-07	1.22455E-07	1.30182E-07	2.14E-08	6.87333E-08	4.44E-09	9.72E-08
Serratia_grimesii	2.70909E-08	0	3.70909E-08	6.68182E-08	2.72692E-07	0	0	1.90769E-08	0	0	0	0	1.23267E-07	8.33333E-08	3.946E-07	3.44933E-07
Serratia_sp._FDAARGOS_506	2.70909E-08	0	1.63636E-08	2.91545E-08	0	0	0	0	2.72727E-08	0	2.76364E-08	3.94545E-08	0	0	0	6.86667E-08
Serratia_rubidaea	4.93273E-07	0	1.39909E-07	2.90636E-07	9.26923E-08	3.38615E-07	2.89538E-07	1.87462E-07	1.20182E-07	1.96818E-07	3.97364E-07	4.41455E-07	1.00733E-07	0.00000005	1.04933E-07	9.79333E-08
Serratia_sp._KUDC3025	1.88364E-07	1.57318E-07	3.70909E-08	0.000000162	0	0	0	0	2.64545E-08	0	7.29091E-08	8.29091E-08	0	0	0	0
Serratia_sp._FGI94	0	0	8.75455E-09	1.91818E-08	0	0	0	0	0	0	0	0	0	0	0	0
Serratia_odorifera	6.03727E-07	1.98636E-07	2.47727E-07	4.11455E-07	0	0.000000363	2.63077E-08	2.49231E-07	1.08545E-07	1.77091E-07	1.00182E-07	9.57727E-08	2.14E-08	0.000000074	1.49867E-07	1.64333E-07
Serratia_sp._3ACOL1	0	0	4.12727E-09	0	0	0	0	0	0	0	0	0	0	0	0	0
Serratia_sp._MYb239	2.46091E-07	4.72273E-07	9.64545E-08	1.48727E-07	3.93538E-07	0	2.63077E-08	8.19231E-08	3.20545E-07	0.000000034	2.61091E-07	6.85727E-07	8.00667E-08	1.81067E-07	0	2.57533E-07
Serratia_plymuthica	5.02182E-07	6.46364E-07	2.64364E-07	5.65364E-07	9.92308E-08	6.53077E-08	4.33846E-08	1.74462E-07	3.72818E-07	1.79909E-07	4.81636E-07	0.00000078	6.94E-08	0.000000102	9.94667E-08	1.15533E-07
Serratia_ficaria	0	0	2.18545E-08	9.54545E-09	1.09231E-07	0	0	9.84615E-08	0	6.37273E-08	2.76364E-08	0	1.86667E-08	5.19333E-08	1.65333E-08	1.68533E-07
Serratia_marcescens	0.000000877	3.09136E-07	3.99373E-06	6.96909E-07	5.40462E-07	1.59408E-06	6.16846E-07	7.19231E-07	1.70482E-06	9.38909E-07	1.05236E-06	2.20427E-06	5.32533E-07	4.17933E-07	0.000000604	4.14667E-07
Serratia_sp._ATCC_39006	9.60545E-07	3.30455E-07	2.43218E-07	5.24182E-07	0.000000647	1.53385E-07	1.29877E-06	5.17923E-07	1.17264E-06	1.72636E-07	4.62182E-07	6.64909E-07	0.000000339	4.83467E-07	1.37933E-07	3.84533E-07
Serratia_fonticola	6.05273E-07	1.39227E-06	2.17973E-07	4.08364E-07	2.63615E-07	1.07669E-06	2.96077E-07	5.30231E-07	5.58909E-07	6.45455E-07	7.07727E-07	4.27091E-07	1.61067E-07	4.34667E-07	4.17667E-07	0.00000006
Serratia_liquefaciens	2.30473E-06	4.66091E-07	1.11182E-07	3.60273E-07	3.78077E-07	0	0	0.00000164	1.14545E-06	1.22964E-06	2.16391E-06	7.64591E-07	3.67533E-07	7.302E-07	7.212E-07	7.18267E-07
Wigglesworthia_glossinidia	3.62727E-08	0	0	0	2.05385E-08	0	0	0	0	0	0	0	7.68667E-08	0	6.46667E-08	0
Izhakiella_sp._KSNA2	3.11818E-08	0	2.724E-07	4.41909E-07	6.46923E-08	1.76769E-07	2.19538E-07	2.39077E-07	6.50909E-08	2.08636E-07	3.79091E-08	0.000000347	2.266E-07	1.198E-07	2.50307E-07	3.90067E-07
Tatumella_citrea	1.22273E-07	1.03636E-08	5.57545E-08	9.54545E-09	3.34615E-08	4.13846E-08	0	2.56923E-08	3.94545E-08	0	0	3.16364E-08	0	0	0	0
Tatumella_sp._TA1	3.11818E-08	2.56818E-08	5.36182E-07	2.12818E-07	0	1.55385E-07	5.54615E-08	5.45385E-08	0.000000027	0.000000068	3.33636E-08	1.19273E-07	5.15333E-08	6.36E-08	1.01333E-07	1.682E-07
Tatumella_ptyseos	5.20909E-08	7.38636E-08	2.59818E-08	3.61182E-07	4.77462E-07	0.000000026	4.33846E-08	2.13308E-07	2.16364E-08	0.000000163	1.37273E-07	9.64545E-08	4.624E-07	0.000000285	8.88667E-07	8.678E-07
Mixta_intestinalis	3.90818E-07	4.15409E-07	1.03727E-07	1.68182E-07	2.84846E-07	0	3.11538E-08	1.05308E-07	5.13818E-07	6.88455E-07	6.77909E-07	5.98182E-07	9.36E-08	1.61333E-08	1.01973E-07	6.52E-08
Mixta_calida	1.03109E-06	2.82091E-07	6.70273E-07	1.48955E-06	2.33846E-07	9.46154E-09	6.85692E-07	1.14377E-06	1.02936E-06	6.81091E-07	6.74636E-07	3.04591E-07	3.68333E-07	2.82867E-07	3.562E-07	6.44533E-07
Mixta_gaviniae	1.06664E-06	8.31091E-07	1.97818E-07	5.25455E-07	2.98923E-07	0	4.31077E-07	0.00000111	4.94364E-07	4.71727E-07	6.37273E-07	1.53409E-06	3.42067E-07	0.00000049	4.80133E-07	3.65533E-07
Mixta_theicola	3.33273E-06	5.28455E-07	1.62327E-06	1.43927E-06	1.38438E-06	4.39231E-08	1.57477E-06	2.89846E-06	2.73336E-06	1.90909E-06	1.48573E-06	0.000002911	2.6546E-06	1.52853E-06	1.84293E-06	2.52727E-06
Buchnera_aphidicola	8.67727E-06	1.46009E-06	1.27636E-06	3.65709E-06	0.00000689	3.26546E-06	2.63869E-06	6.23962E-06	4.75818E-06	7.44364E-06	1.59864E-05	8.04364E-06	5.26333E-06	5.07893E-06	7.74867E-06	5.9958E-06
Pantoea_sp._MT58	0	0	0	0	0	0	0	0	0	0	0	0	0	1.61333E-08	0	0
Pantoea_eucalypti	0	0	0	0	0	8.84615E-08	0	0	0	0	0	0	0	0	0	0
Pantoea_sp._SO10	7.88182E-08	9.54545E-09	0	1.32273E-07	0	5.55769E-07	0	8.13846E-08	2.75455E-08	5.67818E-07	1.76727E-07	1.30455E-07	1.044E-07	1.36067E-07	1.42933E-07	1.408E-07
Candidatus_Pantoea_carbekii	0	0	1.77273E-08	5.78182E-08	2.88462E-08	0	0	2.89692E-07	0	0	0	1.42727E-08	7.93333E-08	1.10933E-07	9.61333E-08	1.22E-08
Pantoea_ananatis	3.35818E-07	1.00527E-06	2.76436E-07	5.52545E-07	3.71308E-07	5.79538E-07	2.96385E-07	8.42077E-07	0.000000763	5.62727E-07	5.96636E-07	9.33455E-07	8.412E-07	4.904E-07	5.79133E-07	7.786E-07
Pantoea_sp._CCBC3-3-1	3.69091E-08	0	3.06091E-08	3.07273E-08	6.03846E-08	0	5.80923E-07	3.14846E-07	0	5.22727E-08	0	0.000000035	1.19867E-07	1.25667E-07	2.03507E-07	1.63333E-07
Pantoea_sp._201603H	4.89455E-07	0	9.25818E-08	1.06455E-07	1.62615E-07	0	2.63077E-08	6.32308E-08	5.97273E-08	1.09545E-07	6.68182E-08	1.86636E-07	2.24E-08	0	1.57067E-08	1.48667E-07
Pantoea_sp._PSNIH1	0	2.61818E-08	2.16727E-08	3.04091E-07	8.33846E-08	2.13846E-08	0	1.90769E-08	0	0	0	1.42727E-08	0	6.51333E-08	8.93333E-08	1.66667E-08
Pantoea_sp._SM3640	0	0	8.25455E-09	0	0	0	0	0	0	0	0	0	0	0	0	0
Pantoea_stewartii	1.74891E-06	5.86727E-07	2.89514E-06	1.46845E-06	0.000000115	2.13846E-08	2.00077E-07	4.44462E-07	4.58364E-07	5.72091E-07	1.07618E-06	9.07091E-07	2.14E-08	9.68667E-08	3.964E-08	4.78E-08
Pantoea_dispersa	7.72727E-08	0.00000026	1.04618E-07	2.62727E-07	1.49692E-07	6.07769E-07	1.45923E-07	1.10385E-07	3.16909E-07	1.35545E-07	1.35636E-07	3.67818E-07	0.000000133	1.44867E-07	1.93533E-07	1.95267E-07
Pantoea_agglomerans	5.04455E-07	5.78364E-07	3.12273E-07	3.54364E-07	6.30385E-07	0.000003646	3.13154E-07	1.13692E-07	5.51091E-07	0.000000304	9.35091E-07	3.74182E-07	7.66E-08	7.766E-07	2.796E-07	7.31067E-07
Pantoea_rwandensis	1.15182E-07	0	8.75455E-09	1.47518E-06	8.09231E-08	1.98877E-06	4.21031E-06	2.60408E-06	5.29455E-07	3.48182E-07	7.04364E-07	0.000000563	2.21733E-07	1.412E-07	2.71973E-07	2.88333E-07
Pantoea_alhagi	2.96364E-08	0.00000013	0	9.54545E-09	2.08308E-07	2.95385E-08	1.37077E-07	1.49538E-07	0	0	0	3.87727E-08	2.12467E-07	2.82867E-07	2.82333E-07	4.80933E-07
Pantoea_vagans	1.22027E-06	1.18927E-06	1.23364E-07	9.96273E-07	5.91538E-07	1.34323E-06	1.83085E-06	4.93385E-07	1.11482E-06	5.76364E-07	5.87273E-07	1.15009E-06	1.91867E-07	5.82933E-07	4.77467E-07	3.25933E-07
Pantoea_sp._At-9b	4.31636E-07	4.78227E-07	1.234E-07	2.41727E-07	2.84154E-07	4.61308E-07	2.73923E-07	5.84615E-08	5.52364E-07	3.35182E-07	4.03364E-07	4.11636E-07	3.59867E-07	5.23067E-07	2.04533E-07	4.26067E-07
Erwinia_pyrifoliae	0	0	0	0	0	0	0	0	7.50909E-08	8.02727E-08	0	2.14545E-08	0	0	0	0
Erwinia_tasmaniensis	5.12727E-08	0	0	0.000000235	2.05385E-08	2.52308E-08	1.20231E-07	2.64692E-07	0.000000071	1.53182E-07	0	0	1.09467E-07	1.00933E-07	0	1.552E-07
Erwinia_persicina	9.96364E-08	0.000000065	1.72182E-07	3.35091E-07	5.45385E-08	2.95385E-08	1.08077E-07	9.19231E-08	3.98182E-08	5.22727E-08	8.31818E-08	2.38909E-07	0	1.16133E-07	1.12667E-08	1.66667E-08
Erwinia_amylovora	0.000000314	5.55455E-08	5.48182E-08	2.39091E-08	1.15462E-07	2.13846E-08	3.16769E-07	0	1.35636E-07	5.03818E-07	0.000000528	1.26455E-07	5.44E-08	7.02667E-08	2.24667E-08	0
Erwinia_billingiae	7.37273E-08	0	4.12727E-09	2.25455E-07	0.00000017	0	0	0	5.79091E-08	1.60545E-07	2.44636E-07	7.78182E-08	2.24E-08	1.00933E-07	6.82667E-08	1.70533E-07
Erwinia_gerundensis	5.57273E-08	5.99091E-08	8.21364E-08	0	9.82308E-08	1.56615E-07	2.63077E-08	4.37692E-08	2.36727E-07	9.56364E-08	1.92727E-07	8.67273E-08	9.78E-08	3.35333E-08	2.08733E-07	1.592E-07
Erwinia_sp._J780	3.06364E-07	0.00000013	2.06364E-08	2.80273E-07	3.34923E-07	0	2.42923E-07	1.90385E-07	2.04727E-07	3.43091E-07	2.10545E-07	1.23091E-07	2.04067E-07	4.09533E-07	4.38133E-07	3.02867E-07
Candidatus_Erwinia_haradaeae	0.00000264	0.000000508	1.37357E-06	4.32718E-06	1.78285E-06	0	0.000003381	2.13308E-06	1.23664E-06	2.25918E-06	2.73045E-06	0.000002348	0.000002038	2.1546E-06	2.48827E-06	0.000003054
Enterobacteriaceae_endosymbiont_of_Macroplea_mutic	0	0	0	0	0	0	0	0	0	0	0	3.16364E-08	0	0	0	0
Candidatus_Ishikawaella_capsulata	0	0	0	3.07273E-08	0	0	0	0	5.42727E-08	0	0	0	0	0	0	0
Enterobacteriaceae_endosymbiont_of_Donacia_piscatrix	0	0	0	1.43636E-08	0	0	0	2.93846E-08	0	0	0	0	0	0	0	1.824E-07
Candidatus_Annandia_adelgestsuga	0	0	0	4.79091E-09	0	0	0	0	0	0	0	9.75E-08	0	0	0	0
Enterobacteriaceae_endosymbiont_of_Donacia_margina	0	0	0	0	0	0	0	0	0	0	0	0	0.000000027	0.000000041	3.14667E-08	0
Enterobacteriaceae_endosymbiont_of_Donacia_clavipes	0	0	0	0	2.59231E-08	0	0	0	0	0.000000034	0	0	0	0	1.65333E-08	0
Candidatus_Moranella_endobia	0	0	0	0	0	1.53077E-07	3.11538E-08	0	0	0	0	0	0	2.38E-08	0	1.98667E-08
Enterobacteriaceae_endosymbiont_of_Donacia_tomento	0	0	0	0	0	0	0	0	3.34273E-07	2.85455E-08	2.68182E-07	0	0	0	0	0
Enterobacteriaceae_endosymbiont_of_Plateumaris_brac	0	0	0	5.18182E-09	0	0	0	0	0	1.93182E-07	1.37273E-07	1.90364E-07	0	0	0	0
Enterobacteriaceae_endosymbiont_of_Donacia_cinerea	2.98182E-08	0	0	0	0	0	0	0	0	0.000000047	2.06364E-07	9.75E-08	0	0	0	9.33333E-09
Plautia_stali_symbiont	3.11818E-08	0	0	3.24545E-08	0	1.04923E-07	5.85385E-08	3.93846E-08	0	0	1.02727E-08	0	0	0	0	3.72E-08
Enterobacteriaceae_endosymbiont_of_Donacia_crassipe	5.12727E-08	0	0	1.91818E-08	0	0	0	5.72308E-08	5.97273E-08	6.08182E-08	1.86182E-07	3.16364E-08	2.14E-08	8.32667E-08	3.00067E-07	3.65333E-08
Enterobacteriaceae_endosymbiont_of_Donacia_vulgaris	0	0	1.63636E-08	0	0	0	0.000000255	1.11077E-07	0	0	6.65455E-08	1.80909E-07	0	0.000000089	2.20933E-07	5.07333E-08
Enterobacteriaceae_endosymbiont_of_Plateumaris_rust	0	0	2.47273E-08	4.79091E-09	0	0	0	0	0	0	0	2.14545E-08	0	0	0	0
Pseudescherichia_vulneris	1.67091E-07	2.70909E-08	0	2.13609E-07	7.77692E-08	9.10769E-08	6.44615E-08	8.89231E-08	6.50909E-08	2.73909E-07	1.71727E-07	1.95091E-07	6.92E-08	0.000000079	2.09933E-07	3.02067E-07
Candidatus_Hoaglandella_endobia	1.02727E-07	2.34545E-08	0	5.96364E-08	7.72308E-08	2.95385E-08	1.42385E-07	2.46923E-08	2.64545E-08	0	0	5.30909E-08	0	0	7.02E-08	1.86667E-08
Candidatus_Riesia_pediculischaeffi	0	0	0	0	0	0	0	0	0	3.62727E-08	0	1.42727E-08	0	0	0	0
Candidatus_Riesia_pediculicola	1.55545E-07	3.99091E-07	1.49818E-07	2.69091E-07	0.000000039	0	0	3.57692E-08	3.86636E-07	0.000000393	2.70909E-07	7.89091E-08	8.56E-08	2.38E-08	0	7.82667E-08
secondary_endosymbiont_of_Ctenarytaina_eucalypti	2.56364E-08	0	3.27727E-07	4.09909E-07	3.88462E-08	0	0	0	1.60182E-07	8.26364E-08	1.16818E-07	1.55636E-07	0.000000027	8.94E-08	1.24707E-07	0
Metakosakonia_sp._MRY16-398	0	0	2.04545E-06	7.97273E-08	0	0	0	0	0	0	0	0	0	0	0	0
Shigella_flexneri	0	0	2.62727E-08	0	0	2.28154E-07	0	3.22308E-08	0	0	0	7.13636E-09	0	0	0	0
Shigella_boydii	0	0	7.88182E-08	2.39727E-08	0	0	0	4.64615E-08	0.000000087	0.000000028	2.61818E-07	0	2.08E-08	0	0	0
Shigella_dysenteriae	0	1.04545E-08	2.00909E-07	1.47273E-08	0	2.13846E-08	0	0	0	0	0	0	0	0	0	0
Atlantibacter_hermannii	2.28091E-07	1.65455E-07	1.86673E-07	1.41636E-07	4.11538E-08	4.01615E-07	2.56923E-07	1.25923E-07	1.02455E-07	1.02909E-07	2.64091E-07	1.55773E-07	7.17333E-08	4.64667E-08	2.55333E-08	1.25133E-07
Shimwellia_blattae	2.11545E-07	9.03636E-08	8.74909E-08	1.80091E-07	1.07538E-07	2.17692E-07	1.89231E-07	1.98385E-07	1.78909E-07	1.33364E-07	0.000000136	3.09091E-07	1.37067E-07	2.38E-08	1.022E-07	0
Kluyvera_cryocrescens	0	0	8.75455E-09	0	0	0	0	0	0	0	0	0	0	0	0	0
Kluyvera_genomosp._3	6.19091E-08	0	5.42727E-06	5.11182E-07	1.42385E-07	0.000000026	0	0	9.73636E-08	1.84909E-07	0.00000039	1.65182E-07	3.768E-07	1.578E-07	3.63467E-07	0.000000148
Kluyvera_intermedia	1.01818E-07	1.49091E-07	1.75816E-06	3.61636E-07	2.88462E-08	1.64169E-06	4.50769E-07	0.000000257	2.15636E-07	1.04364E-07	1.02727E-08	8.55455E-08	6.89333E-08	8.24E-08	1.0284E-07	1.41933E-07
Enterobacteriaceae_endosymbiont_of_Donacia_cinctico	0	1.15545E-07	4.12727E-09	0	2.05385E-08	2.22154E-07	0	4.64615E-08	0	8.95455E-08	0.000000082	7.13636E-09	1.528E-07	6.48E-08	5.94667E-08	6.86667E-08
Candidatus_Purcelliella_pentastirinorum	0	0	8.25455E-09	1.47273E-08	0	0	0	0	0	0	2.76364E-08	0	2.37333E-08	0	4.93067E-08	4.34E-08
Phytobacter_diazotrophicus	5.57273E-08	0	0	0	0	0	0	0	0	0	2.76364E-08	0	0	0	0	0
Phytobacter_ursingii	1.02664E-06	1.93091E-07	1.94209E-07	1.06455E-06	7.69154E-07	3.83077E-08	1.40769E-07	5.71308E-07	1.00664E-06	3.63727E-07	2.70845E-06	1.56682E-06	5.44333E-07	3.39533E-07	4.436E-07	6.19333E-07
Yokenella_regensburgei	2.59364E-07	4.18182E-07	1.60909E-07	3.26909E-07	4.16385E-07	6.76923E-08	5.67692E-07	3.68923E-07	3.77182E-07	3.11636E-07	8.18818E-07	8.22455E-07	1.352E-07	3.918E-07	2.712E-07	0.000000255
[Enterobacter]_lignolyticus	1.08273E-06	9.85455E-08	1.94055E-06	1.89518E-06	6.95538E-07	0	3.44538E-06	6.04815E-06	1.49482E-06	1.14336E-06	1.14809E-06	4.85727E-07	6.794E-07	5.82067E-07	9.40133E-07	1.09913E-06
Pluralibacter_gergoviae	1.99727E-07	2.70909E-08	4.10649E-06	4.46609E-07	0.00000032	9.46154E-09	2.30077E-07	2.34231E-07	1.40182E-07	2.45364E-07	0.00000035	2.02182E-07	7.94667E-08	1.378E-07	1.524E-07	1.23467E-07
Lelliottia_sp._WB101	0	0	8.75455E-09	1.91818E-08	3.15385E-08	2.16923E-08	3.33077E-08	2.56923E-08	0	0	0	1.82591E-07	7.96E-08	3.78E-08	2.55333E-08	1.22E-08
Lelliottia_nimipressuralis	0	0	4.63636E-07	0	0	0	0	1.90769E-08	0	0	0	0	0	0	0	0
Lelliottia_jeotgali	0	0	9.37636E-08	3.99091E-08	0	7.27692E-07	0	0	0	0	1.02727E-08	0	0	0	0	1.98667E-08
Lelliottia_amnigena	1.37364E-07	6.70909E-08	4.26364E-08	9.30909E-08	1.11692E-07	4.54154E-07	1.22615E-07	2.49846E-07	1.98364E-07	2.54182E-07	6.00909E-08	3.04182E-07	0.00000009	2.022E-07	2.26573E-07	1.32533E-07
Buttiauxella_agrestis	2.70909E-08	0	0	1.47273E-08	0	0	0	0	3.94545E-08	0	0	1.42727E-08	0	0	4.82E-08	0
Buttiauxella_sp._3AFRM03	1.30427E-06	0	1.52309E-07	6.51091E-07	4.30231E-07	4.73846E-08	7.73154E-07	1.06931E-06	1.20964E-06	6.70545E-07	1.36073E-06	9.69455E-07	4.12067E-07	4.63867E-07	4.9484E-07	3.85333E-07
Raoultella_ornithinolytica	0	1.37273E-08	9.16364E-08	1.03636E-08	2.21385E-07	1.59154E-07	3.33077E-08	8.66154E-08	2.16373E-06	2.74273E-07	1.53182E-07	2.08182E-07	2.14E-08	0	1.65867E-07	0
Raoultella_electrica	0	0	8.22727E-07	0	0	0	0	0	0	0	0	0	0	0	0	0

Raoultella_terrigena	0.000001653	2.92318E-07	2.32455E-07	1.34673E-06	5.41462E-07	0	2.63077E-08	9.44615E-07	9.27818E-07	7.14636E-07	3.06555E-06	9.58364E-07	2.368E-07	5.04467E-07	0.000000595	7.31933E-07
Raoultella_planticola	0	3.68273E-07	1.78636E-07	0.000000638	1.11308E-07	6.30769E-08	3.84462E-07	1.23692E-07	1.62909E-07	2.85091E-07	7.85455E-08	2.50364E-07	1.59533E-07	1.18333E-07	1.97467E-07	0.000000254
Leclercia_sp._1106151	0.000000073	0	0	0	0.000000061	5.45231E-07	1.15615E-07	1.85462E-07	1.73182E-07	0	1.72636E-07	0.000000075	1.14333E-07	7.03333E-08	2.61467E-07	1.202E-07
Leclercia_sp._LSNIH3	0	0	8.75455E-09	0	0	0	0	0	0	0	0	0	0	0	0	0
Leclercia_sp._W6	0	0	5.25455E-08	0	0	0	0	0	0	0	0	0	0	0	0	0
Leclercia_sp._W17	0	0	1.75455E-07	3.99091E-08	0	0	0	0	0	0	0	0	0	0	0	0
Leclercia_sp._LSNIH1	0	0	2.00909E-07	7.97273E-08	0	0	0	0	0	0	0	0	0	0	0	0
Leclercia_adecarboxylata	2.96364E-08	0	2.58595E-06	4.16545E-07	2.96769E-07	3.78154E-07	8.87692E-08	5.81538E-08	6.73636E-08	5.22727E-08	6.65455E-08	1.49545E-07	2.35533E-07	1.188E-07	2.39467E-07	1.99933E-07
Leclercia_sp._119287	3.11818E-08	1.07545E-07	5.66727E-08	3.99091E-08	0	0	2.73846E-08	4.93846E-08	3.94545E-08	1.14273E-07	3.95455E-08	7.13636E-09	2.37333E-08	4.80667E-08	4.20667E-08	0
Leclercia_sp._J807	0	0	2.16727E-08	1.91818E-08	0	0	0	0	0	0	0	0	0	0	1.12667E-08	0
Leclercia_sp._29361	5.03364E-07	1.37273E-08	5.25455E-08	4.68545E-07	0	1.37462E-07	0	8.46154E-08	4.35818E-07	3.07182E-07	4.13727E-07	4.62545E-07	2.09333E-08	3.11667E-07	1.8484E-07	3.20667E-08
Cronobacter_dublinensis	0	0	0	0	0	0	0	0	0	0	0	0	0	0	4.82E-08	3.40667E-08
Cronobacter_turicensis	0	0	3.70909E-08	3.24545E-08	3.15385E-08	7.21538E-08	6.23077E-08	1.90769E-08	0	5.22727E-08	2.76364E-08	2.85455E-08	7.38E-08	0.000000041	5.94E-08	9.33333E-09
Cronobacter_universalis	2.07727E-07	1.68182E-08	1.63636E-08	4.79091E-09	1.29692E-07	0	0	1.90769E-08	0.000000177	0	0	3.57273E-08	0.000000099	4.49333E-08	8.72667E-08	2.39267E-07
Cronobacter_condimenti	1.15091E-07	4.32545E-07	0	1.47273E-08	1.64154E-07	2.28308E-07	0	0	5.97273E-08	3.89091E-08	1.46818E-07	2.14545E-08	1.162E-07	2.28667E-07	3.47467E-07	1.44867E-07
Cronobacter_sp._JZ38	0	0	0	4.50909E-08	3.58462E-08	1.23846E-07	0	0	2.64545E-08	0	0	7.84545E-08	2.91333E-08	3.48E-08	1.12667E-08	0
Cronobacter_malonaticus	1.02818E-07	0	4.81818E-07	7.01818E-08	2.88462E-08	7.71538E-08	0	4.06692E-07	1.02364E-07	0.000000047	6.88182E-08	1.12182E-07	4.72667E-08	1.62333E-07	8.76667E-08	3.14933E-07
Cronobacter_sakazakii	2.48109E-06	2.89909E-07	9.78309E-07	1.37836E-06	5.76923E-07	4.68385E-07	1.03108E-06	4.34769E-07	2.60682E-06	3.60909E-06	2.45736E-06	2.27036E-06	4.75667E-07	8.25333E-07	6.22533E-07	7.87267E-07
Scandinavium_goeteborgense	2.98982E-06	3.51636E-07	9.78027E-06	1.02318E-05	3.56085E-06	1.12769E-07	0.000030564	2.69341E-05	6.10036E-06	7.17455E-06	0.00000502	3.25982E-06	2.96327E-06	2.2854E-06	0.000004924	2.9434E-06
Cedecea_lapagei	0.000000155	9.55455E-08	4.67636E-08	2.58909E-07	1.00538E-07	0.00000036	0.000000301	1.61615E-07	0.000000482	4.04455E-07	4.15182E-07	2.83091E-07	7.32667E-08	6.38E-08	1.04307E-07	2.344E-07
Cedecea_neteri	0.000000574	0.00000025	1.57136E-07	2.84818E-07	5.97385E-07	1.79231E-08	5.46846E-07	7.37538E-07	8.25091E-07	0.000000643	4.31909E-07	4.49545E-07	0.000000793	6.68533E-07	1.05767E-06	7.51067E-07
Cedecea_sp._FDAARGOS_727	8.33727E-07	0.000000065	4.28455E-07	4.12364E-07	3.46538E-07	1.20385E-07	2.45538E-07	2.09462E-07	5.19545E-07	2.99636E-07	6.55727E-07	5.57273E-07	3.77667E-07	2.712E-07	0.000000616	3.00267E-07
Enterobacteriaceae_endosymbiont_of_Plateumaris_seri	0	0	1.63636E-08	0	0	0	0	1.01385E-07	0	0	0	0	0	3.48E-08	7.79333E-08	1.30333E-07
Enterobacteriaceae_endosymbiont_of_Plateumaris_pus	0	0	8.31818E-09	0	1.08462E-07	0	1.57092E-06	1.38769E-06	3.98182E-08	0.000000047	2.76364E-08	2.14545E-08	1.98333E-07	6.902E-07	6.404E-07	3.52067E-07
Kosakonia_oryzae	0	0	0	0	3.15385E-08	0	0	0	2.75455E-08	9.40909E-08	3.77273E-08	0	2.24E-08	0	0	0
Kosakonia_sp._CCTCC_M2018092	0	1.16364E-08	0	0	0	0	2.06923E-07	5.22308E-08	0	0	3.95455E-08	0	1.618E-07	0	0	0
Kosakonia_cowanii	0.000000184	1.65955E-07	7.30545E-07	1.08155E-07	0.000000039	6.27692E-08	0	2.89462E-07	0	0.000000028	2.18273E-07	5.37273E-08	7.44347E-06	0.000000014	4.44E-09	0
Kosakonia_sacchari	3.96364E-07	4.65091E-07	9.64818E-08	3.61727E-07	0	0	3.77692E-08	3.41692E-07	7.15455E-08	0.000000163	1.34364E-07	5.87273E-07	1.86667E-08	0	1.2004E-07	7.26667E-08
Kosakonia_arachidis	1.62909E-07	9.68182E-08	7.83273E-07	2.72455E-07	0.00000072	7.21538E-08	0	9.69231E-08	4.87818E-07	6.55182E-07	4.56273E-07	5.08182E-07	5.33733E-07	5.722E-07	4.73467E-07	6.25333E-07
Kosakonia_radicincitans	2.70909E-08	3.37909E-07	9.63636E-08	1.60455E-07	2.75385E-08	0	0	0.000000011	1.62182E-07	1.83818E-07	0	7.44545E-08	4.702E-07	2.632E-07	2.68867E-07	6.81333E-08
Kosakonia_sp._SMBL-WEM22	2.06727E-06	3.58091E-07	3.02636E-07	6.66182E-06	8.55462E-07	1.01869E-05	1.62479E-05	1.17277E-05	2.71809E-06	4.66273E-06	0.0000049	2.37345E-06	1.17647E-06	1.20127E-06	8.64667E-07	7.234E-07
Escherichia_sp._E4742	0	0	0	0	0	0	0	4.93846E-08	0	0	0	0	0	0	0	1.86E-08
Escherichia_fergusonii	0	0.00000026	2.51182E-08	2.93364E-08	0	0	0	2.46923E-08	0.000000177	0.000000047	0	1.00364E-07	0	4.64667E-08	1.49333E-08	0
Escherichia_albertii	1.15091E-07	8.72727E-08	1.89545E-07	8.23364E-08	0.000000039	1.78538E-07	3.33077E-08	2.46923E-08	4.45455E-08	1.04545E-07	1.18636E-07	2.67727E-07	6.08E-08	1.58E-08	6.31067E-08	1.17267E-07
Escherichia_marmotae	2.58545E-07	2.34545E-08	4.39664E-07	0.000000336	1.36385E-07	2.95385E-08	7.71538E-07	1.18554E-06	1.96182E-07	9.36364E-08	1.90182E-07	2.88727E-07	5.09333E-08	1.60467E-07	0.000000299	1.62667E-07
Escherichia_coli	2.51555E-06	6.10455E-07	3.83231E-05	6.21282E-06	1.09231E-06	8.53462E-07	2.54969E-06	2.26915E-06	1.97545E-06	1.39209E-06	2.88282E-06	1.58009E-06	1.3532E-06	1.46707E-06	1.49387E-06	2.1424E-06
Citrobacter_sp._RHBSTW-00881	0	1.68182E-08	0	0	0	0	0	0	0	0	0	0	0	0	0	0
Citrobacter_sp._RHBSTW-00986	0	0	0	0	2.05385E-08	0	0	0	0	0	0	0	0	0	0	3.67333E-08
Citrobacter_sp._FDAARGOS_156	3.11818E-08	2.41818E-08	0	0	0	0	0	0	0	0	0	0	0	2.38E-08	0	1.22E-08
Citrobacter_sp._CFNIH10	4.87273E-08	0	0	0	0	0	0	0	0	1.61364E-07	6.68182E-08	0	2.14E-08	4.66667E-08	9.44667E-08	1.49467E-07
Citrobacter_sp._NMI7904_11	3.62727E-08	0	0	0	2.80308E-07	7.36154E-08	5.74615E-08	3.57692E-08	0.000000084	0.000000056	1.99727E-07	0.000000635	1.53467E-07	3.09333E-08	6.384E-08	0
Citrobacter_braakii	2.56364E-08	0	5.43636E-08	2.02455E-07	2.88462E-08	0	0	5.22308E-08	0	0	3.33636E-08	3.94545E-08	0	0.000000033	0	3.40667E-08
Citrobacter_amalonaticus	5.82727E-08	0	1.07091E-07	3.72455E-08	9.73846E-08	2.06923E-07	9.70769E-08	1.26615E-07	0.000000027	0	6.68182E-08	1.52273E-07	4.53333E-08	0	4.44E-09	0.000000028
Citrobacter_koseri	2.70909E-08	0	1.30909E-07	0	2.05385E-08	0	9.03846E-08	5.22308E-08	6.48182E-08	0.000000068	1.02727E-08	6.32727E-08	1.86667E-08	5.60667E-08	1.49333E-08	1.64667E-07
Citrobacter_rodentium	0	0	3.06364E-07	4.79091E-09	0	0	0	0	3.98182E-08	0	0	0	2.37333E-08	4.64667E-08	1.49333E-08	0
Citrobacter_sp._RHB35-C17	0	0	8.75455E-09	0	0	0	0	0	0	0	0	0	0	0	0	0
Citrobacter_sp._RHB35-C21	0	0	8.75455E-09	0	0	0	0	0	0	0	0	0	0	0	1.12667E-08	0
Citrobacter_sp._LY-1	4.87273E-08	0	8.75455E-09	9.54545E-09	0	0	0	0	0	0	0	7.13636E-09	2.72E-08	0	0	1.66667E-08
Citrobacter_sp._RHB25-C09	0	3.87273E-08	8.75455E-09	3.46182E-07	3.34615E-08	2.66154E-07	1.41846E-07	8.01538E-08	1.45182E-07	0	8.02727E-08	0	1.82267E-07	7.84667E-08	2.19533E-07	2.92E-08
Citrobacter_sp._CRE-46	0	0	2.00909E-07	0	0	0	0	0	0	0	0	0	0	0	0	0
Citrobacter_sp._RHBSTW-00524	0	0	0.00000021	0	0	0	0	0	0	0	0	0	0	0	0	0
Citrobacter_sp._RHBSTW-00887	0	0	2.27273E-07	7.97273E-08	0	0	0	0	0	0	0	0	0	0	0	0
Citrobacter_sp._LUTT5	0	0	3.06364E-07	1.59091E-07	0	0	0	0	0	0	0	0	0	0	0	0
Citrobacter_freundii_complex_sp._CFNIH2	1.58182E-08	7.04545E-08	8.75455E-09	4.24273E-08	3.15385E-08	0	0	0	1.02455E-07	0	6.68182E-08	1.94455E-07	0	0	5.10667E-08	0
Citrobacter_portucalensis	6.36964E-06	6.05364E-07	5.10909E-07	4.75545E-07	0	0	0	0	7.12818E-07	2.78273E-07	1.53255E-06	9.27273E-07	0	0	0	0
Citrobacter_youngae	0	0	9.72727E-07	0	0	0	0	0	0	0	0	0	0	0	0	0
Citrobacter_werkmanii	0.000000292	1.57091E-07	2.96691E-06	3.63882E-07	6.30769E-08	0	5.96154E-08	3.93846E-08	0	0	3.33636E-08	3.94545E-08	0	3.50667E-08	4.48667E-08	0
Citrobacter_farmeri	0	9.36364E-09	2.46182E-08	3.40909E-08	1.19462E-07	0	0	3.29231E-08	0	3.89091E-08	2.11636E-07	9.85455E-08	2.14E-08	0.000000041	8.86667E-09	1.22E-08
Citrobacter_sp._SNU_WT2	1.89364E-07	0	1.49545E-07	5.44818E-07	1.15462E-07	0	3.06923E-08	1.51231E-07	4.39273E-07	2.35727E-07	1.49545E-07	1.43545E-07	3.54867E-07	3.514E-07	2.22173E-07	1.91133E-07
Citrobacter_freundii	4.74636E-07	2.51545E-07	9.70618E-06	1.14855E-06	6.74231E-07	0.000000098	7.92308E-08	4.87308E-07	5.78455E-07	1.87455E-07	4.51273E-07	4.85909E-07	3.86267E-07	3.85533E-07	7.38467E-07	9.53933E-07
Salmonella_sp._SJTUF14178	0	0	1.75455E-08	0	0	0	0	0	0	0	0	0	0	0	0	0
Salmonella_bongori	1.54455E-07	0	2.45455E-07	1.68091E-07	1.07692E-07	1.41138E-06	1.53769E-07	8.23846E-08	0	2.87273E-08	0	1.79727E-07	2.44E-08	6.24667E-08	1.13667E-07	1.66267E-07
Salmonella_enterica	3.74364E-06	7.01182E-07	8.3089E-05	7.63073E-06	6.86462E-06	2.35546E-06	1.03508E-06	1.87046E-06	9.12182E-06	6.92636E-06	7.25364E-06	7.44545E-06	6.55933E-06	0.00000517	0.000006682	7.22667E-06
Enterobacter_soli	0	0	2.62727E-08	0	1.02077E-07	0	0	2.93846E-08	0	0	0	0	0	7.60667E-08	4.27333E-08	0
Enterobacter_sp._HK169	0	0	8.75455E-09	0	0	0	0	0	0	0	0	0	0	0	0	0
Enterobacter_sp._JUb54	1.58182E-08	0	8.75455E-09	6.64545E-08	2.05385E-08	0	0	6.66923E-08	2.72727E-08	0	6.88182E-08	1.42727E-08	2.14667E-08	1.58E-08	6.54E-08	7.69333E-08
Enterobacter_sp._RHB15-C17	0	2.41818E-08	8.75455E-09	0	0	0	0	0	0	0	2.05455E-08	7.13636E-09	2.37333E-08	0	0	0
Enterobacter_sp._RHBSTW-00975	0	0	8.75455E-09	0	0	0	0	0	0	0	0	0	0	0	0	0
Enterobacter_sp._CRENT-193	0	0	8.75455E-09	0	0	0	0	0	0	0	0	0	0	0	0	0
Enterobacter_sp._E20	0	0	1.49091E-07	0	0	0	0	0	0	0	0	0	0	0	0	0
Enterobacter_sp._RHBSTW-00994	0	0	1.83636E-07	9.19091E-08	7.77692E-08	0	0	6.01538E-08	5.42727E-08	1.84545E-07	1.08364E-07	7.13636E-09	6.36667E-08	3.09333E-08	4.78667E-08	1.66667E-08
Enterobacter_sp._RHBSTW-00593	0	0	1.92727E-07	0	0	0	0	0	0	0	0	0	0	0	0	0
Enterobacter_sp._LU1	0	0	5.07273E-07	0	0	0	0	0	0	0	0	0	0	0	0	0
Enterobacter_sp._RHBSTW-00175	0.000000132	0	0.00000056	0	0	0	0	0	1.02636E-07	0	2.76364E-08	2.85455E-08	4.30667E-08	4.90667E-08	1.12667E-08	1.86E-08
Enterobacter_sp._18A13	0	0	6.12727E-07	4.79091E-09	0	0	0	0	0	0	3.95455E-08	7.13636E-09	0	0	0	0
Enterobacter_sp._ODB01	0	0	5.29091E-06	6.38182E-07	0	0	0	0	0	0	0	0	0	0	0	0
Enterobacter_ludwigii	0	0	1.83636E-07	0	0	0	0	0	0	0	0	0	0	0	0	0
Enterobacter_sichuanensis	0	0	4.57818E-07	2.23245E-07	1.13538E-07	0	5.26154E-08	1.68385E-07	7.97273E-08	2.85455E-08	6.88182E-08	1.42727E-08	2.44E-08	3.50667E-08	2.01333E-08	1.00067E-07
Enterobacter_cloacae_complex_sp._FDA-CDC-AR_013	0	0	8.75455E-09	0	0	0	0	0	0	0	0	0	0	0	0	0
Enterobacter_cloacae_complex_sp._FDA-CDC-AR_016	0	0	8.75455E-09	0	0	0	0	0	0	0	0	0	0	0	0	0
Enterobacter_cloacae_complex_sp._ECNIH7	0	0	0.00000007	0	0	0	0	0	0	0	0	0	0	0	0	0
Enterobacter_cloacae_complex_sp.	7.25455E-08	0	7.52727E-07	7.97273E-08	0	0	0	0	0	0	1.37273E-07	7.13636E-09	0	0	0	0
Enterobacter_kobei	1.58182E-08	0	2.21818E-06	0.00000012	0	0	0	5.81538E-08	0	0	0	0	0	0.000000014	0	0
Enterobacter_roggenkampii	2.98182E-08	0	3.36595E-06	2.76336E-07	2.59231E-08	0	7.40769E-08	1.90769E-08	9.23636E-08	1.16636E-07	4.98182E-08	1.11818E-07	6.94E-08	2.38E-08	0	1.808E-07
Enterobacter_cancerogenus	4.11455E-07	2.99409E-07	8.624E-07	3.37727E-07	1.64692E-07	8.59846E-07	1.26485E-06	5.27923E-07	5.65727E-07	6.39818E-07	7.35909E-07	4.58364E-07	6.31667E-07	5.704E-07	4.71333E-07	2.99267E-07
Enterobacter_cloacae	1.86482E-06	2.53636E-07	0.006090756	0.000491604	1.72077E-06	6.01846E-07	3.52077E-07	5.21769E-07	1.97118E-06	1.93936E-06	1.48318E-06	1.72382E-06	7.326E-07	1.1064E-06	7.134E-07	1.07527E-06
Enterobacter_hormaechei	6.80909E-08	1.09636E-06	2.72216E-05	2.18309E-06	2.75385E-08	3.15385E-08	1.46538E-07	1.92154E-07	7.56182E-07	0	7.90909E-08	8.08455E-07	4.29333E-08	0	0	4.78E-08
Enterobacter_chengduensis	6.80909E-08	0	7.14727E-07	2.28182E-07	0.000000054	0	3.33077E-08	3.57692E-08	0.000000237	0.000000034	0.000000162	1.37364E-07	0	0	5.04667E-08	9.93333E-08
Enterobacter_sp._SA187	8.53636E-08	7.41818E-08	3.91273E-08	1.93636E-08	9.84615E-08	0	9.74615E-08	1.46923E-08	1.82364E-07	0	1.46818E-07	1.25545E-07	5.07333E-08	1.30533E-07	9.25333E-08	2.32533E-07
Enterobacter_oligotrophicus	1.66373E-06	5.95455E-07	1.83118E-06	1.32527E-06	2.23231E-07	1.18538E-07	1.34354E-06	1.76746E-06	3.76727E-07	7.57909E-07	1.12355E-06	7.40727E-07	7.50667E-08	4.41133E-07	2.02533E-07	3.77533E-07
Enterobacter_bugandensis	1.47455E-07	0	4.24545E-06	2.53818E-07	7.50769E-08	0	5.26154E-08	2.98308E-07	2.49545E-07	1.36182E-07	2.86455E-07	2.02273E-07	2.14667E-08	1.84733E-07	0.000000076	7.08E-08
Enterobacter_asburiae	1.30182E-07	0.000000069	1.10909E-06	1.12182E-07	1.04231E-07	3.83077E-08	0	1.48538E-07	4.80909E-08	9.78182E-08	2.17273E-07	1.45409E-07	2.20267E-07	3.46867E-07	1.14333E-07	1.86667E-08
Enterobacter_sp._N18-03635	9.77455E-07	1.12486E-06	1.84082E-06	1.13064E-06	3.20615E-07	0.000000275	1.20231E-06	1.02162E-06	4.86727E-07	6.66545E-07	1.21127E-06	9.50909E-07	1.91533E-07	4.88533E-07	6.40533E-07	1.326E-07
Enterobacter_sp._R4-368	0	0	0	0	1.97769E-07	0	0	1.61692E-07	3.94545E-08	1.05727E-07	0	2.14545E-08	9.84E-08	1.95533E-07	2.49333E-07	0.000000268
Enterobacter_sp._638	7.42273E-07	0	2.24727E-06	2.47264E-06	5.96846E-07	0	4.53615E-06	6.42538E-06	9.90182E-07	9.21545E-07	1.03082E-06	1.05564E-06	4.71067E-07	0.000000316	8.74667E-07	5.52467E-07
Enterobacter_sp._E76	6.72091E-07	2.21636E-07	7.40909E-08	8.11909E-07	2.46385E-07	0	0	8.31538E-08	2.66818E-07	4.06636E-07	3.60727E-07	2.96273E-07	2.54467E-07	3.758E-07	3.94133E-07	7.87467E-07
Candidatus_Hamiltonella_defensa	2.30909E-08	1.64273E-07	2.87273E-08	4.79091E-09	5.92462E-07	2.16923E-08	0	0	0	0	2.76364E-08	6.41364E-08	7.35467E-07	1.60133E-07	3.85267E-07	2.79933E-07
Candidatus_Tachikawaea_gelatinosa	2.46455E-07	0.000000065	3.87091E-07	5.90455E-06	8.80923E-07	2.56231E-06	4.65738E-06	8.56692E-06	9.02455E-07	5.88455E-07	0.000003277	4.71273E-06	1.0254E-06	1.74407E-06	2.8902E-06	0.000002049
Blochmannia_endosymbiont_of_Polyrhachis_(Hedomyrr	0	0	0	0	0	1.02462E-07	3.33077E-08	7.28462E-08	0	0	0	0	0	0	0	0
Blochmannia_endosymbiont_of_Camponotus_(Colobops	0	0	0	0	3.88462E-08	0	0	0	0	6.27273E-08	0	9.03636E-08	0	0	1.47773E-07	0
Candidatus_Blochmannia_vafer	5.42727E-08	0.000000065	0	0	0	0	0	0	0	0.000000075	0	7.13636E-09	0	0.000000033	1.49333E-08	3.33333E-08
Blochmannia_endosymbiont_of_Colobopsis_nipponica	2.56364E-08	0.000000065	7.05727E-08	2.87273E-08	4.86154E-08	0	0	2.93846E-08	0	0	1.60909E-07	7.13636E-09	0	0	3.14667E-08	0
Blochmannia_endosymbiont_of_Camponotus_nipponen	0	0	8.25455E-09	0	7.96154E-08	0	0	1.16769E-07	0	0	0	3.57273E-08	5.35333E-08	3.70467E-07	1.90733E-07	3.67333E-08
Candidatus_Doolittlea_endobia	1.23482E-06	0.00000026	3.79664E-07	1.80264E-06	2.15862E-06	2.95385E-08	1.37192E-06	1.75462E-06	4.02727E-07	3.62636E-07	8.74818E-07	1.43641E-06	2.26433E-06	1.96013E-06	3.27913E-06	5.31533E-06
Candidatus_Mikella_endobia	1.48655E-06	0	0	0	6.28308E-06	0	0	0	0	0	0	7.75455E-08	3.56313E-06	2.99073E-06	3.4462E-06	2.6554E-06
Klebsiella_sp._P1CD1	0	0	0	0	0	0	0	0	5.97273E-08	0	0	0	0	0	0	0
Klebsiella_huaxiensis	0	0	0	3.87273E-08	1.64692E-07	0	2.63077E-08	3.81538E-08	2.72727E-08	0	0	3.16364E-08	1.86667E-08	6.28667E-08	1.182E-07	5.94667E-08
Klebsiella_sp._WP3-S18-ESBL-05	0	0	0	0	0	0	0	0	0.00000535	2.87273E-08	6.88182E-08	0	0	0	0	0
Klebsiella_sp._LTGPAF-6F	7.37273E-08	0	0	3.24545E-08	0	0.000000026	0	0	0	0	2.05455E-08	1.46818E-07	2.14E-08	9.93333E-08	0	0
Klebsiella_sp._WP4-W18-ESBL-05	0	0	8.75455E-09	0	0	0	0	0	0	0	0	0	0	0	0	0
Klebsiella_sp._PO552	0	0	5.25455E-08	0	0	0	0	0	2.98182E-07	0	0	0	0	0	0	0
Klebsiella_sp._WP8-S18-ESBL-06	0	0	1.13636E-07	3.55182E-08	0	0	0	0	0	0	0	0	0	0	0	0
Klebsiella_quasivariicola	0	0	2.89091E-07	3.16727E-07	0.000000077	0	3.11538E-08	0	3.83455E-07	2.85455E-08	6.71818E-08	1.90364E-07	2.14667E-08	0	0	7.95333E-08
Klebsiella_grimontii	0	0	6.09091E-06	5.98182E-07	0	0	0	0	9.77273E-08	0	0	0	0	0	0	0
Klebsiella_michiganensis	0.000000424	0.000000287	5.97091E-07	6.57336E-07	1.11462E-07	4.60692E-07	5.08615E-07	6.63538E-07	4.57809E-06	2.21909E-07	6.18818E-07	4.57545E-07	1.54267E-07	1.66267E-07	1.27573E-07	1.11267E-07
Klebsiella_oxytoca	0.000000509	7.22364E-07	7.60273E-07	2.31091E-07	6.73077E-08	1.80846E-07	3.10615E-07	1.08923E-07	4.25182E-07	1.27818E-07	1.32545E-07	8.87364E-07	9.28E-08	2.28067E-07	1.64307E-07	1.392E-07
Klebsiella_quasipneumoniae	0	2.34545E-08	3.51663E-05	2.87091E-06	3.15385E-08	1.89615E-07	1.39538E-07	7.15385E-08	0.000000694	1.53182E-07	6.71818E-08	3.16364E-08	0.000000091	2.06867E-07	1.126E-07	1.52533E-07
Klebsiella_variicola	1.96455E-07	0.00000013	1.364E-07	2.94273E-07	2.64154E-07	0	2.84077E-07	1.70538E-07	0.000001601	1.55636E-07	3.16364E-07	0.000000329	0.000000222	1.408E-07	2.11267E-07	1.672E-07
Klebsiella_pneumoniae	7.05364E-07	7.27364E-07	1.67599E-05	1.66155E-06	5.30923E-07	1.64615E-07	5.74538E-07	9.48385E-07	0.000452572	4.78218E-06	0.000012584	1.35614E-05	4.37133E-07	8.86333E-07	6.452E-07	1.3634E-06
Klebsiella_aerogenes	1.64827E-06	2.91909E-07	2.61951E-05	3.81364E-06	6.34692E-07	6.84892E-06	5.83323E-06	8.39923E-06	2.05436E-06	1.28382E-06	2.49873E-06	1.47891E-06	1.08873E-06	5.10667E-07	1.40447E-06	1.6584E-06
Otariodibacter_oris	0	0	0	0	0	2.95385E-08	3.33077E-08	0	0	0	0	0	0	0	0	1.86E-08
Frederiksenia_canicola	1.11818E-07	7.54545E-08	0	0	0	4.69385E-07	3.06923E-08	3.81538E-08	0	6.25455E-08	0	0	6.42E-08	6.10667E-08	4.96667E-08	3.74667E-07
Bibersteinia_trehalosi	0	0	0	0	0	1.21831E-06	3.33077E-08	9.03846E-08	0	0	0	0	2.91333E-08	5.36E-08	1.13973E-07	3.52667E-08
Vespertiliibacter_pulmonis	0	8.86364E-09	0	6.31818E-08	1.87231E-07	5.37923E-07	5.26154E-08	8.44615E-08	5.97273E-08	0	0	0	2.37333E-08	5.60667E-08	4.02667E-08	1.054E-07
Bisgaardia_hudsonensis	3.69091E-08	5.05455E-08	1.53818E-07	4.68182E-08	2.75385E-08	1.41769E-07	0	2.46923E-08	3.94545E-08	1.04545E-07	0.000000061	0.000000066	1.47867E-07	2.268E-07	2.33667E-07	3.52667E-08
Gallibacterium_anatis	2.44364E-07	2.51545E-07	0.000000046	1.77364E-07	5.44615E-07	0.000000999	2.90923E-07	5.57077E-07	4.62545E-07	4.31364E-07	1.09582E-06	9.55909E-07	4.09533E-07	3.622E-07	7.72333E-07	0.000000842
Glaesserella_parasuis	4.73364E-07	1.03636E-08	2.47273E-08	9.54545E-09	2.05385E-08	7.15405E-05	3.65154E-07	2.19769E-07	2.28636E-07	5.72727E-08	0	0	1.358E-07	3.34067E-07	8.11333E-08	4.95333E-08
Glaesserella_sp._15-184	0	0	3.70909E-08	8.07273E-08	1.32769E-07	1.15369E-05	0	2.69077E-07	0.000000053	0.000000163	1.35455E-07	3.05273E-07	6.89333E-08	1.58E-08	1.98307E-07	5.568E-07
Avibacterium_paragallinarum	3.26091E-07	0.00000013	0	8.21818E-08	3.51923E-07	3.91608E-06	1.03777E-06	1.95592E-06	0.000000614	2.70909E-07	9.10727E-07	1.04082E-06	5.69933E-07	6.72933E-07	1.28587E-06	1.70087E-06
Avibacterium_volantium	2.84727E-07	0	5.48182E-08	0.000000077	5.47692E-08	2.64077E-06	4.33846E-08	1.26308E-07	2.75455E-08	0.000000028	1.25182E-07	1.61818E-07	0	0.00000008	1.14267E-07	0
Histophilus_somni	1.52036E-06	4.29091E-08	2.55273E-07	6.44545E-08	3.06769E-07	3.69642E-05	7.49769E-07	8.70538E-07	1.67618E-06	1.52209E-06	2.75264E-06	1.27582E-06	0.000000525	1.88667E-07	3.47467E-07	0.000000944
[Mannheimia]_succiniciproducens	3.69091E-08	1.03636E-08	0.000000047	5.18182E-09	7.78462E-08	4.71169E-06	0	3.00769E-08	0	0	3.77273E-08	8.80909E-08	0	0	5.04667E-08	3.33333E-08
Basfia_succiniciproducens	1.28818E-07	2.34545E-08	0	2.95455E-08	1.28231E-07	7.95385E-07	6.04462E-07	4.59769E-07	1.16182E-07	3.03636E-08	2.76455E-07	1.28091E-07	0.00000012	7.78E-08	8.59333E-08	3.092E-07
Haemophilus_sp._oral_taxon_036	0	0	0	0	0	0	0	0	0	0	0	0	0	3.50667E-08	0	0
Haemophilus_pittmaniae	2.30909E-08	0	0	0	0	8.54231E-07	0	0.000000011	0	0	7.90909E-08	0	0	0	0	3.67333E-08
Haemophilus_parahaemolyticus	5.57273E-08	0	4.12727E-09	0.000000157	0	5.87077E-07	3.33077E-08	1.03385E-07	1.06364E-07	6.37273E-08	8.02727E-08	5.37273E-08	0	0	0	1.22E-08
[Haemophilus]_ducreyi	0	0	0	0	0	7.21538E-08	8.11538E-08	6.79231E-08	0	0	0	4.59091E-08	8.45333E-08	6.63333E-08	0	2.19933E-07
Haemophilus_influenzae	5.52727E-08	0	9.36364E-08	8.86364E-08	1.86923E-08	0.000150592	2.56308E-07	9.87692E-08	7.20909E-08	1.63636E-07	2.76364E-08	2.07545E-07	1.87667E-07	2.27467E-07	1.61533E-07	2.59333E-07

Haemophilus_parainfluenzae	4.27273E-07	1.06182E-07	8.28455E-08	1.34791E-07	2.43154E-07	5.17923E-06	8.48462E-08	1.35538E-07	1.24636E-07	1.32545E-07	1.69909E-07	7.44545E-08	2.64E-08	2.97133E-07	8.68667E-08	1.86E-08
Haemophilus_haemolyticus	1.36545E-06	0	4.03545E-07	9.09273E-07	4.32846E-07	3.64462E-06	1.69231E-07	7.54231E-07	5.53182E-07	5.30818E-07	6.00909E-07	8.49909E-07	2.84267E-07	7.15533E-07	2.97867E-07	0.00000061
Actinobacillus_lignieresii	0	0	0	0	0	0	0	0	0	0	0	0	2.14E-08	0	0	0
Actinobacillus_delphinicola	0	0	0	0	0	0	0	0	0	0	0	2.14545E-08	0	0	0	0
Actinobacillus_equuli	0	0	0	2.89091E-08	0	1.31308E-07	3.06923E-08	3.22308E-08	0	0	0	0	2.08E-08	0	0	0
Actinobacillus_suis	0	0	0	4.79091E-09	0	0	0	0	0	0	0	0	0	0	1.12667E-08	1.86E-08
Actinobacillus_porcitonsillarum	2.17364E-07	7.66364E-08	3.70909E-08	3.60727E-07	1.23846E-07	5.45255E-05	2.63077E-08	1.39538E-07	3.53364E-07	2.19455E-07	2.43818E-07	3.32636E-07	2.24E-08	2.45067E-07	1.83573E-07	1.94133E-07
Actinobacillus_indolicus	3.94455E-07	0	3.91636E-07	1.03773E-06	4.03769E-07	3.33123E-05	1.14692E-07	4.58308E-07	3.90182E-07	2.37818E-07	5.35818E-07	1.58227E-07	4.06267E-07	2.62667E-07	2.614E-07	3.352E-07
Actinobacillus_succinogenes	2.24091E-07	0.00000013	8.25455E-09	8.88818E-08	1.11154E-07	0.00000091	9.74615E-08	4.32308E-08	1.76545E-07	8.85455E-08	2.76818E-07	3.38364E-07	0.000000119	1.13733E-07	1.118E-07	8.50667E-08
Actinobacillus_pleuropneumoniae	1.70164E-06	0	0	2.11364E-07	1.11615E-06	9.70946E-06	6.40769E-08	3.22308E-08	0.000001269	1.40182E-06	8.58909E-07	1.53418E-06	1.83627E-06	1.85993E-06	1.4894E-06	1.90053E-06
Rodentibacter_sp._DSM_111151	6.71364E-07	6.67909E-07	2.58727E-07	2.19273E-07	1.07692E-07	0.00000898	3.42769E-07	1.61231E-07	1.46364E-07	1.98545E-07	5.67727E-07	5.21636E-07	0	9.58667E-08	2.18067E-07	4.65667E-07
Rodentibacter_pneumotropicus	2.70909E-08	1.76864E-07	1.06636E-07	3.39091E-08	0.000000201	0.000710625	6.85308E-07	7.02308E-07	2.75455E-08	0.000000028	9.87273E-08	7.12727E-08	0.000000285	5.95333E-07	1.08447E-06	1.24547E-06
Rodentibacter_heylii	1.20818E-06	7.87273E-08	1.10909E-07	2.07273E-08	5.97515E-06	0.020548911	1.34923E-05	1.74142E-05	6.13364E-07	5.46545E-07	6.74545E-07	1.21009E-06	0.000010972	0.000020586	2.70328E-05	3.38023E-05
Mannheimia_sp._BNO311	0	0	0	0	0	0	0	0	0	0	0	0	0	0	0	6.86667E-08
Mannheimia_sp._16CN0041	0	0	0	9.97273E-09	0	2.52308E-08	0	0	0	0	0	0	0	0	3.59067E-08	5.34E-08
Mannheimia_sp._USDA-ARS-USMARC-1261	4.56364E-08	0	3.70909E-08	6.28182E-08	0	1.38615E-06	2.72615E-07	3.19385E-07	2.25455E-07	0.000000062	1.50455E-07	2.39545E-07	9.14667E-08	2.14133E-07	3.828E-07	2.32333E-07
Mannheimia_ovis	3.10727E-07	9.31818E-07	1.09682E-06	7.97727E-07	1.25077E-07	4.73077E-08	1.57385E-07	2.82154E-07	3.27545E-07	1.52545E-07	3.47818E-07	2.88818E-07	1.51267E-07	7.38E-08	1.29107E-07	2.19267E-07
Mannheimia_sp._ZY190616	2.48727E-07	0	1.52309E-07	4.50909E-08	5.42308E-08	3.83077E-08	4.33846E-08	3.18462E-07	0	6.37273E-08	2.73364E-07	1.74818E-07	2.33867E-07	5.19333E-08	2.29667E-07	6.14E-08
Mannheimia_varigena	3.11818E-08	2.11818E-08	0	1.99727E-07	1.41077E-07	0.000010702	2.06385E-07	7.03846E-08	1.39455E-07	0.00000009	1.07636E-07	2.01091E-07	1.272E-07	2.75267E-07	1.892E-07	6.44667E-08
Mannheimia_granulomatis	1.39891E-06	2.81091E-06	2.11318E-07	0.000000683	1.25385E-06	7.90077E-07	1.07077E-06	7.02846E-07	3.09091E-07	1.95455E-07	1.37982E-06	1.32073E-06	9.92E-08	1.08053E-06	1.53927E-06	0.000001076
Mannheimia_haemolytica	7.10273E-07	0	2.24382E-07	1.31182E-07	1.06054E-06	2.09399E-05	3.07462E-07	5.97846E-07	8.26364E-08	2.00182E-07	3.48182E-07	1.80727E-07	0.000000475	8.982E-07	8.91533E-07	1.29353E-06
Aggregatibacter_segnis	7.26364E-08	0	0	5.18182E-09	2.75385E-08	0	5.36923E-08	0	2.39727E-07	0.000000047	1.30909E-07	7.13636E-09	4.81333E-08	3.09333E-08	1.44373E-07	1.48467E-07
Aggregatibacter_actinomycetemcomitans	0.000003145	0.000000382	3.08273E-07	2.98182E-07	9.48769E-07	1.18892E-05	1.47146E-06	1.15323E-06	2.39391E-06	3.87727E-06	3.57027E-06	2.62345E-06	6.10067E-07	5.18133E-07	6.612E-07	1.56487E-06
Aggregatibacter_aphrophilus	7.70455E-07	1.10455E-07	1.52582E-07	3.30182E-07	6.55077E-07	4.15077E-06	0.00000034	2.95846E-07	2.77909E-07	1.59909E-07	7.29091E-08	1.67182E-07	8.32133E-07	7.712E-07	9.59467E-07	9.218E-07
Pasteurella_stomatis	0	0	0	0	0	0	3.06923E-08	0	0	0	0	0	0	0	0	7.61333E-08
Pasteurella_skyensis	2.30909E-08	0	2.46182E-08	1.03545E-07	3.15385E-08	1.30538E-07	4.27769E-07	2.04308E-07	0	9.36364E-08	9.05455E-08	0.000000035	1.164E-07	3.99333E-08	7.55333E-08	4.76667E-08
Pasteurella_multocida	0.000828971	0.000281371	3.11058E-05	0.000344845	0.000435605	0.000197697	0.000226691	0.000183837	0.000514564	0.000425717	0.000299325	0.000725746	0.000454618	0.000139547	0.000168673	0.000299105
Bradymonas_sediminis	4.51455E-07	7.66364E-08	3.32727E-07	2.16909E-07	2.34846E-07	1.22846E-07	2.14385E-07	6.74308E-07	2.40091E-07	0.000000243	2.78818E-07	6.63182E-07	2.35867E-07	2.04533E-07	2.898E-07	9.37333E-08
Persicimonas_caeni	0.000001481	6.69818E-07	8.49391E-07	3.13891E-06	5.50538E-07	1.59585E-06	3.71915E-06	2.64377E-06	1.35618E-06	1.14773E-06	1.42918E-06	0.000001694	7.82467E-07	6.22067E-07	7.63067E-07	1.09633E-06
Desulfarculus_baarsii	1.34218E-06	3.47273E-07	1.70364E-07	7.61727E-07	6.93308E-07	1.20631E-06	7.23846E-07	8.05385E-07	1.30227E-06	9.48182E-07	1.10509E-06	1.21782E-06	0.000000704	6.61533E-07	6.87867E-07	1.02147E-06
Candidatus_Desulfofervidus_auxilii	3.00818E-07	3.82636E-07	1.06818E-07	4.66636E-07	5.45462E-07	3.31538E-07	1.00231E-06	2.65385E-07	5.21182E-07	6.06455E-07	2.00455E-07	6.19909E-07	0.000000331	5.282E-07	3.19733E-07	8.67333E-08
Desulfomonile_tiedjei	1.16282E-06	2.31545E-07	5.43636E-07	1.95782E-06	1.00831E-06	1.15423E-06	3.04231E-06	5.62023E-06	0.000001024	6.18636E-07	2.40091E-06	3.25727E-06	6.01333E-07	0.000001424	1.74407E-06	2.15853E-06
Desulfobacca_acetoxidans	5.06273E-07	3.73682E-07	1.49636E-07	2.62182E-07	9.75385E-08	1.14615E-07	0.00000015	2.22077E-07	0.000000281	2.43636E-07	3.61364E-07	2.94636E-07	2.20067E-07	2.79667E-07	2.02933E-07	2.798E-07
Syntrophus_aciditrophicus	1.16736E-06	4.51727E-07	4.69091E-07	1.27145E-06	9.89923E-07	5.01538E-07	2.28108E-06	4.60877E-06	9.61182E-07	7.77455E-07	1.44336E-06	2.58509E-06	7.83333E-07	1.0876E-06	1.67847E-06	1.1152E-06
Syntrophobacter_fumaroxidans	4.65636E-07	8.84545E-08	3.61364E-07	7.44545E-07	2.14931E-06	4.85077E-07	0.00000018	3.63308E-06	5.15636E-07	5.69818E-07	5.20818E-07	6.64545E-07	2.02873E-06	3.49733E-06	4.98667E-06	0.000005494
Desulfoglaeba_alkanexedens	2.68164E-06	2.05455E-07	3.15636E-07	6.64364E-07	1.95208E-06	7.88538E-07	1.12769E-06	3.08462E-06	2.81855E-06	2.83627E-06	3.27655E-06	3.85218E-06	1.5596E-06	2.00607E-06	1.82707E-06	0.000002586
Hippea_maritima	2.39191E-06	0.000000075	2.83273E-07	1.04045E-06	1.37392E-06	2.91077E-07	0.000001447	2.09862E-06	2.38873E-06	3.34364E-06	4.84382E-06	0.000001866	1.75267E-06	1.23167E-06	1.70333E-06	3.7506E-06
Desulfurella_acetivorans	1.10273E-06	0	3.70909E-08	9.12727E-08	2.92962E-06	0	0	2.51615E-07	7.02727E-08	2.87273E-08	0.000000588	3.00182E-07	1.52227E-06	1.8828E-06	2.27513E-06	1.58787E-06
Desulfurivibrio_alkaliphilus	1.34018E-06	1.06764E-06	1.81982E-07	1.30055E-06	1.26554E-06	5.71238E-06	8.00077E-07	8.78154E-07	0.000001207	1.40682E-06	1.02573E-06	1.32418E-06	5.76733E-07	8.632E-07	1.11993E-06	1.03933E-06
Desulfocapsa_sulfexigens	1.34873E-06	8.005E-07	3.95636E-07	1.14618E-06	3.05308E-07	2.43385E-07	5.68385E-07	4.39231E-07	6.64273E-07	1.28936E-06	1.82836E-06	1.80927E-06	2.29867E-07	4.54867E-07	7.112E-07	5.00067E-07
Desulfotalea_psychrophila	8.35455E-07	1.68045E-07	3.23545E-07	5.76545E-07	5.12308E-07	1.60615E-07	8.31154E-07	5.00308E-07	9.14364E-07	1.00664E-06	3.71727E-07	1.09555E-06	7.48667E-07	6.854E-07	8.01533E-07	1.01313E-06
Desulfopila_sp._IMCC35005	4.75273E-07	3.52727E-07	7.17273E-08	9.97273E-08	1.44769E-06	2.93846E-07	3.33077E-08	2.11177E-06	0.000000485	3.07727E-07	1.94636E-07	3.47182E-07	1.5456E-06	1.8082E-06	4.05867E-06	3.19833E-06
Desulfopila_sp._IMCC35004	1.39955E-06	1.00918E-06	7.71727E-07	1.08645E-06	0.000001509	1.37462E-07	6.56538E-07	0.000004477	8.71455E-07	7.63545E-07	1.10973E-06	1.18982E-06	1.44293E-06	2.15507E-06	3.59787E-06	0.000004192
Desulfobulbus_propionicus	1.26355E-06	2.46273E-07	3.92855E-07	1.45773E-06	0.000000482	1.67769E-06	2.35738E-06	1.82423E-06	9.62364E-07	6.50091E-07	1.91482E-06	1.89882E-06	6.786E-07	7.244E-07	7.426E-07	5.63533E-07
Desulfobulbus_oralis	2.03882E-06	5.17091E-07	5.20091E-07	2.08309E-06	2.12785E-06	5.04615E-07	1.12631E-06	2.10985E-06	2.65191E-06	3.21809E-06	2.31909E-06	0.00000342	1.45453E-06	3.12487E-06	3.76047E-06	2.47507E-06
Desulfobacterium_autotrophicum	8.97818E-07	7.10045E-07	2.01636E-07	1.11882E-06	4.66538E-07	7.23077E-08	8.61462E-07	7.16692E-07	0.000000241	4.42091E-07	1.27745E-06	7.29182E-07	2.35933E-07	3.14133E-07	1.28467E-07	4.50267E-07
Desulfobacula_toluolica	3.79364E-07	6.19818E-07	1.25618E-07	7.44273E-07	5.20385E-07	4.13769E-07	4.43692E-07	3.43077E-07	1.83909E-07	3.33364E-07	3.33636E-08	3.59364E-07	2.87133E-07	4.99133E-07	9.60733E-07	8.65267E-07
Desulfobacter_hydrogenophilus	8.76909E-07	3.84055E-06	4.75845E-07	6.06727E-07	5.26462E-07	1.63508E-06	6.11846E-07	7.67692E-07	4.08091E-07	5.87273E-07	6.99182E-07	9.58364E-07	3.996E-07	3.67067E-07	8.25867E-07	1.0512E-06
Desulfobacter_postgatei	5.69182E-07	4.02455E-07	2.18909E-07	4.01909E-07	3.49615E-07	0.000000199	0	3.88846E-07	3.50273E-07	6.59727E-07	8.85727E-07	3.25727E-07	2.93867E-07	2.41667E-07	2.55333E-07	4.03667E-07
Desulfatibacillum_aliphaticivorans	3.14818E-06	0.000002107	1.13664E-06	1.88609E-06	2.40892E-06	1.06662E-06	0.000001593	1.27023E-06	1.34245E-06	1.69318E-06	0.000001838	2.82636E-06	1.75173E-06	1.86067E-06	2.07627E-06	1.3828E-06
Desulfococcus_multivorans	2.41682E-06	0.000001198	1.09909E-06	1.62573E-06	9.94538E-07	1.23292E-06	0.000000756	1.55523E-06	0.00000399	2.90709E-06	2.54182E-06	2.95182E-06	2.02893E-06	2.08947E-06	2.02127E-06	0.000002952
Desulfococcus_oleovorans	3.40691E-06	1.27036E-06	3.45273E-07	1.63409E-06	1.14185E-06	1.16346E-06	6.59077E-07	1.69185E-06	2.84936E-06	1.72291E-06	3.08727E-06	2.82182E-06	9.622E-07	1.8546E-06	1.9474E-06	1.82547E-06
Desulfosarcina_widdelii	4.89273E-06	0.000001757	1.71364E-06	4.39627E-06	1.19169E-06	1.21746E-06	2.06654E-06	9.52769E-07	3.54227E-06	2.27018E-06	4.80664E-06	3.86545E-06	1.26133E-06	1.3668E-06	1.83967E-06	1.0576E-06
Desulfosarcina_ovata	4.49273E-06	3.29755E-06	3.44727E-06	0.00000446	1.23808E-06	3.34338E-06	2.65323E-06	2.67308E-06	2.17882E-06	2.00973E-06	2.73264E-06	0.00000471	1.1684E-06	1.1442E-06	1.88153E-06	1.29827E-06
Desulfosarcina_alkanivorans	2.45773E-06	1.92209E-06	9.04845E-07	2.79736E-06	1.54292E-06	5.03154E-06	2.79469E-06	2.80123E-06	2.13718E-06	1.93027E-06	2.37636E-06	2.63309E-06	1.3384E-06	2.8136E-06	2.04093E-06	2.2618E-06
Vulgatibacter_incomptus	6.91636E-07	1.43727E-07	5.75818E-08	7.88636E-07	0.000000289	1.82523E-06	2.74692E-07	5.04769E-07	4.86636E-07	3.11636E-07	2.23455E-07	4.59545E-07	0.000000199	1.378E-07	0.000000095	3.24667E-07
Haliangium_ochraceum	1.16991E-06	4.93273E-07	3.37545E-07	5.09545E-07	3.31846E-07	6.98231E-07	3.99615E-07	0.000000519	9.55455E-07	8.28636E-07	5.10455E-07	6.02455E-07	3.94333E-07	5.768E-07	2.546E-07	4.164E-07
Labilithrix_luteola	4.10727E-07	6.57273E-08	1.58182E-07	5.23091E-07	2.23462E-07	2.65923E-07	3.84308E-07	4.57154E-07	4.22273E-07	2.15727E-07	5.43091E-07	3.86818E-07	3.396E-07	1.16933E-07	2.93867E-07	0.000000297
Sandaracinus_amylolyticus	8.50727E-07	1.16364E-08	0.000000197	3.53909E-07	0.000000209	3.90154E-07	4.89769E-07	6.28385E-07	9.14182E-07	6.93091E-07	7.90364E-07	8.56091E-07	1.61133E-07	4.838E-07	2.41733E-07	2.03867E-07
Minicystis_rosea	9.02091E-07	0	5.52545E-08	5.18273E-07	3.53615E-07	4.72923E-07	2.92154E-07	0.000000126	6.16909E-07	2.90818E-07	1.16455E-07	2.73364E-07	4.13733E-07	3.25733E-07	1.49267E-07	3.48733E-07
Pajaroellobacter_abortibovis	3.62727E-08	0	0	1.86818E-07	3.15385E-08	0	6.44615E-08	1.19385E-07	2.64545E-08	5.22727E-08	4.92727E-08	1.47455E-07	2.64E-08	3.50667E-08	3.75733E-08	0
Chondromyces_crocatus	1.65891E-06	6.09045E-07	1.13045E-06	1.61273E-06	5.20692E-07	1.11146E-05	3.06223E-06	3.10538E-06	9.50091E-07	6.73818E-07	1.29909E-06	1.51436E-06	1.00673E-06	8.866E-07	1.0768E-06	1.12653E-06
Sorangium_cellulosum	4.70545E-06	5.84182E-07	7.83591E-07	0.000003212	3.52308E-06	2.64877E-06	2.43754E-06	5.38846E-06	4.33518E-06	4.16709E-06	3.56273E-06	5.53182E-06	0.000004468	4.2516E-06	5.26333E-06	0.00000497
Myxococcus_hansupus	2.70909E-08	0	2.47273E-08	2.43636E-08	4.11538E-08	0	6.96923E-08	1.64538E-07	2.64545E-08	0	1.02727E-08	3.505E-07	4.56667E-08	1.71333E-08	1.04573E-07	5.59333E-08
Myxococcus_stipitatus	2.98182E-08	0	0	1.32545E-07	1.04231E-07	9.95231E-07	2.17846E-07	1.23231E-07	1.47091E-07	1.61909E-07	7.03909E-07	6.04545E-07	2.24E-08	0.000000099	2.31133E-07	6.33333E-08
Myxococcus_xanthus	0.000001675	1.94545E-07	4.164E-07	4.34545E-07	2.66538E-07	4.05462E-07	0.000000446	3.94615E-07	9.25455E-07	2.66364E-07	7.76455E-07	1.49564E-06	3.85667E-07	2.096E-07	2.95133E-07	1.94067E-07
Corallococcus_coralloides	0.000003657	4.83364E-07	2.35482E-06	2.48136E-06	6.01231E-07	4.95077E-07	2.43438E-06	2.03608E-06	1.50109E-06	1.00791E-06	1.91909E-06	2.36536E-06	5.93533E-07	0.000001017	6.9504E-07	7.83133E-07
Corallococcus_macrosporus	2.14909E-07	8.27273E-08	4.53455E-08	3.08091E-07	2.58538E-07	3.90692E-07	1.77846E-07	1.32462E-07	2.28727E-07	2.03182E-07	1.76818E-07	2.00091E-07	1.378E-07	3.48E-08	2.45733E-07	1.988E-07
Anaeromyxobacter_dehalogenans	6.90636E-07	5.58727E-07	9.87091E-08	7.41455E-07	5.71231E-07	5.40385E-07	0.000000413	8.50462E-07	8.25909E-07	5.92545E-07	6.14636E-07	9.44273E-07	5.328E-07	5.35333E-07	6.53107E-07	1.00353E-06
Anaeromyxobacter_sp._K	4.75364E-07	0.00000013	7.40909E-08	4.17545E-07	0	0	3.77692E-08	4.93846E-08	1.30818E-07	0	7.68182E-08	0.000000035	4.52E-08	8.49333E-08	9.68667E-08	1.22E-08
Anaeromyxobacter_sp._Fw109-5	1.51318E-06	1.45745E-06	0.000000366	1.04518E-06	6.64077E-07	4.33846E-08	4.61385E-07	8.59769E-07	7.40273E-07	7.34636E-07	0.000000645	9.71727E-07	4.20733E-07	4.898E-07	3.53067E-07	5.42933E-07
Archangium_gephyra	3.54273E-07	0.000000065	2.96364E-08	3.63091E-07	4.77154E-07	2.74923E-07	3.03923E-07	2.29154E-07	2.65727E-07	1.86727E-07	3.06727E-07	8.81455E-07	3.008E-07	5.46467E-07	4.50333E-07	8.67867E-07
Stigmatella_aurantiaca	7.19636E-07	2.81364E-07	1.48182E-07	7.25636E-07	1.20538E-07	1.99923E-07	4.66538E-07	0.000000499	5.74273E-07	7.36909E-07	5.62091E-07	6.99636E-07	2.542E-07	1.70733E-07	2.25707E-07	1.484E-07
Melittangium_boletus	7.10455E-07	1.68182E-08	1.52309E-07	3.87727E-07	1.75615E-07	7.52462E-07	1.94538E-07	6.66308E-07	8.88091E-07	4.32091E-07	6.58636E-07	5.33545E-07	0.000000187	3.73267E-07	3.88533E-07	1.81867E-07
Cystobacter_fuscus	1.55282E-06	4.02364E-07	2.66636E-07	9.57091E-07	1.02908E-06	9.57538E-07	9.89923E-07	8.51231E-07	1.37527E-06	1.13591E-06	1.36609E-06	2.72273E-06	1.0702E-06	1.27813E-06	9.10867E-07	7.43867E-07
Desulfuromonas_soudanensis	0.000000939	1.96255E-06	3.54182E-07	1.28827E-06	4.37462E-07	8.98846E-07	3.29692E-07	8.66462E-07	0.000001153	9.00182E-07	2.70582E-06	2.72773E-06	4.34933E-07	4.096E-07	6.732E-07	9.214E-07
Desulfuromonas_sp._DDH964	0.000002499	6.39909E-07	1.14591E-06	1.72209E-06	8.66077E-07	7.94538E-07	1.14854E-06	1.78554E-06	2.12982E-06	8.42273E-07	3.19545E-06	0.000002137	1.67093E-06	1.15427E-06	1.30753E-06	1.28633E-06
Pelobacter_carbinolicus	1.09591E-06	4.35818E-07	4.93636E-08	1.25291E-06	3.36154E-07	6.56154E-07	6.74846E-07	5.73462E-07	7.22273E-07	5.58273E-07	1.10291E-06	1.07545E-06	2.676E-07	5.79667E-07	1.00413E-06	1.15773E-06
Pelobacter_sp._SFB93	5.82636E-07	4.30273E-07	2.37036E-07	7.29727E-07	5.09154E-07	9.61385E-07	5.80308E-07	3.58154E-07	5.21727E-07	5.32545E-07	2.29455E-07	6.81182E-07	3.35067E-07	5.21467E-07	7.32533E-07	4.45133E-07
Pelobacter_acetylenicus	1.18518E-05	1.43436E-06	5.05373E-06	9.29436E-06	4.79077E-06	7.80923E-06	4.97377E-06	0.00000346	0.00001076	6.72273E-06	1.01064E-05	6.23727E-06	4.93067E-06	6.45667E-06	0.000005086	5.49733E-06
Pelobacter_propionicus	5.61182E-06	7.86727E-07	1.24355E-05	1.29064E-05	4.19746E-06	3.58462E-08	3.72383E-05	3.45318E-05	7.33182E-06	6.81427E-06	6.90364E-06	5.70173E-06	3.62913E-06	3.62293E-06	7.46053E-06	3.6192E-06
Geoalkalibacter_subterraneus	1.45227E-06	1.77573E-06	4.97182E-07	8.14091E-07	1.10754E-06	1.41546E-06	6.64692E-07	5.00846E-07	2.06355E-06	2.23691E-06	1.70991E-06	1.65736E-06	1.4028E-06	1.22013E-06	1.40313E-06	1.3204E-06
Geobacter_anodireducens	6.52455E-07	0.000000144	7.39455E-08	1.64273E-07	1.94692E-07	4.26308E-07	5.14615E-07	3.55615E-07	5.19273E-07	4.04636E-07	6.33364E-07	5.11182E-07	2.76533E-07	0.000000307	5.07267E-07	2.024E-07
Geobacter_daltonii	1.07009E-06	2.93727E-07	3.86345E-07	1.27936E-06	6.63692E-07	0.000000544	5.43077E-07	1.08777E-06	9.33273E-07	7.30273E-07	8.73727E-07	1.90436E-06	5.96467E-07	7.70533E-07	1.07587E-06	7.56467E-07
Geobacter_lovleyi	1.22373E-06	8.77727E-07	4.95545E-07	1.01418E-06	6.86692E-07	9.07846E-07	5.75538E-07	4.65462E-07	8.91364E-07	0.000001335	0.000001502	1.43982E-06	4.61133E-07	6.18867E-07	7.06667E-07	5.35733E-07
Geobacter_pickeringii	4.66545E-07	9.88182E-08	4.66618E-07	2.00364E-07	8.75538E-07	2.42385E-07	1.05662E-06	4.39615E-07	7.09182E-07	6.43091E-07	8.36182E-07	7.25818E-07	1.1108E-06	1.46713E-06	1.03633E-06	7.88467E-07
Geobacter_bemidjiensis	6.79545E-07	1.01364E-07	1.56182E-07	1.03855E-06	3.42692E-07	1.71385E-06	5.12615E-07	5.61923E-07	5.44727E-07	0.000000699	5.08818E-07	7.17818E-07	2.43667E-07	5.46733E-07	5.10133E-07	7.68533E-07
Geobacter_sulfurreducens	0.000000643	0.000000968	5.438E-07	4.29909E-07	1.91462E-07	9.29538E-07	4.75154E-07	7.41923E-07	0.000000822	6.64455E-07	7.55455E-07	2.35427E-06	6.79467E-07	6.99733E-07	0.000000568	1.89867E-07
Geobacter_metallireducens	6.83364E-07	4.37455E-07	1.05839E-06	1.36282E-06	9.53308E-07	3.12923E-07	0.000001037	0.000002163	4.91364E-07	6.51909E-07	1.12673E-06	1.03909E-06	8.66467E-07	9.628E-07	1.65933E-06	1.79087E-06
Geobacter_bremensis	3.33091E-06	9.66545E-07	1.3061E-06	3.40091E-06	9.45308E-07	3.13538E-06	3.84154E-06	3.53031E-06	2.94091E-06	1.87809E-06	0.000002815	2.94818E-06	1.12333E-06	9.40067E-07	1.04173E-06	1.16633E-06
Geobacter_uraniireducens	1.26536E-06	1.23418E-06	0.000001011	1.15127E-06	9.76692E-07	1.04223E-06	1.64646E-06	1.53223E-06	1.00536E-06	6.10182E-07	1.87236E-06	2.30836E-06	7.25333E-07	0.000001382	1.55167E-06	1.44247E-06
Geobacter_sp._M18	9.11727E-07	0.000000065	3.59455E-07	6.27273E-07	2.76385E-07	8.24308E-07	2.45692E-07	3.26154E-07	7.32091E-07	5.61455E-07	6.00818E-07	6.69636E-07	4.07133E-07	2.80933E-07	5.884E-07	1.81867E-07
Geobacter_sp._DSM_9736	2.74755E-06	7.66364E-08	1.30364E-06	1.71364E-06	8.95615E-07	7.84931E-06	2.18792E-06	3.22385E-06	1.89245E-06	1.63364E-06	2.06527E-06	1.16736E-06	1.21613E-06	9.836E-07	1.6768E-06	2.16553E-06
Geobacter_sp._FeAm09	1.16882E-06	1.78136E-06	4.35727E-07	0.000001656	9.58077E-07	1.41815E-06	1.37415E-06	0.000001098	1.21764E-06	1.10636E-06	5.01727E-07	2.11145E-06	5.50933E-07	1.00893E-06	1.0304E-06	6.34667E-07
Geobacter_sp._M21	8.66273E-07	8.18909E-07	2.39636E-07	1.44182E-06	3.66692E-07	1.84292E-06	1.07231E-06	5.92308E-07	5.16727E-07	3.25636E-07	9.40545E-07	7.47818E-07	5.73867E-07	5.47667E-07	1.11747E-06	6.872E-07
Desulfohalobium_retbaense	1.67827E-06	2.54173E-06	7.42091E-07	2.73527E-06	1.24838E-06	2.82708E-06	2.12223E-06	3.26585E-06	2.00518E-06	1.93264E-06	3.47073E-06	3.40264E-06	1.65887E-06	2.26407E-06	2.51493E-06	2.60473E-06
Desulfomicrobium_baculatum	1.88355E-06	9.65818E-07	5.03818E-07	1.35736E-06	8.09923E-07	1.01692E-06	5.50308E-07	9.20692E-07	1.81227E-06	0.000001898	2.31991E-06	2.67009E-06	7.94733E-07	1.6476E-06	0.000001882	8.26467E-07
Desulfomicrobium_orale	3.19818E-06	4.96273E-07	0.000000441	1.23382E-06	0.000002062	2.17569E-06	6.25231E-07	1.33631E-06	0.000004117	2.29382E-06	3.27418E-06	0.000003427	1.8152E-06	3.4836E-06	2.3458E-06	1.8126E-06
Desulfolutivibrio_sulfoxidireducens	1.97991E-06	1.34245E-06	4.31636E-07	1.36227E-06	1.02992E-06	2.50808E-06	7.10615E-07	1.15615E-06	1.40973E-06	1.10264E-06	1.29782E-06	3.02773E-06	4.73067E-07	8.59867E-07	9.256E-07	0.000000825
Pseudodesulfovibrio_piezophilus	2.24818E-07	7.28864E-07	3.43E-08	5.26091E-07	4.11538E-08	0	7.92308E-08	2.46923E-08	1.41909E-07	2.49818E-07	2.05818E-07	2.50727E-07	2.24733E-07	2.61867E-07	4.57573E-07	0.00000008
Pseudodesulfovibrio_indicus	1.27027E-06	1.93727E-06	6.93636E-07	1.12645E-06	3.35692E-07	1.19092E-06	3.96385E-07	1.15638E-06	4.30727E-07	6.05636E-07	6.61727E-07	1.03282E-06	4.53933E-07	5.16467E-07	3.66933E-07	5.604E-07
Pseudodesulfovibrio_profundus	3.21182E-07	0	0	1.37273E-07	3.99538E-07	2.13846E-08	6.44615E-08	5.98462E-08	5.87727E-07	6.97182E-07	6.22727E-07	8.47636E-07	4.452E-07	8.174E-07	6.73667E-07	5.06733E-07
Pseudodesulfovibrio_aespoeensis	2.39336E-06	0.00000057	8.69636E-07	8.49364E-07	5.35615E-07	8.96923E-07	1.82162E-06	1.50977E-06	2.15636E-06	2.31818E-06	0.00000265	0.000003368	1.1164E-06	1.92533E-06	1.78733E-06	1.18733E-06
Pseudodesulfovibrio_sp._zrk46	1.24309E-06	2.86273E-07	4.98091E-07	1.00264E-06	4.08462E-07	0.000000785	5.16231E-07	4.12308E-07	8.57909E-07	6.95909E-07	5.54273E-07	1.41491E-06	5.682E-07	1.22173E-06	1.20047E-06	9.22733E-07
Pseudodesulfovibrio_sp._SRB007	1.27591E-06	3.23182E-08	3.26209E-07	2.24636E-06	7.14769E-07	2.96592E-06	4.84762E-06	3.74362E-06	1.58345E-06	2.04509E-06	1.45355E-06	2.17491E-06	8.58867E-07	1.42667E-06	1.72013E-06	1.23827E-06
Lawsonia_intracellularis	2.58236E-06	8.06636E-07	1.24545E-07	1.07718E-06	4.91777E-06	7.20308E-07	4.94462E-07	7.64254E-06	5.08727E-06	6.85564E-06	6.57927E-06	6.94909E-06	4.08613E-06	8.4384E-06	1.32407E-05	8.83933E-06
Desulfocurvibacter_africanus	8.55909E-06	1.03723E-06	8.99818E-07	1.40282E-06	2.30369E-05	8.10538E-07	8.56308E-07	1.87238E-06	0.000002425	2.32527E-06	0.000003143	2.68545E-06	1.51847E-05	1.35273E-05	0.000016748	0.000012404
Desulfovibrio_magneticus	2.50436E-06	0.000002777	0.000000626	1.71282E-06	1.46092E-06	4.58846E-07	6.66462E-07	2.57185E-06	0.000001515	1.22309E-06	1.76173E-06	3.08618E-06	7.96933E-07	1.17547E-06	1.3596E-06	5.20467E-07
Desulfovibrio_carbinoliphilus	1.75627E-06	9.55273E-07	5.36891E-07	1.78091E-06	9.21615E-07	2.00654E-06	0.000001237	1.08592E-06	1.61309E-06	8.01273E-07	1.76055E-06	1.75145E-06	1.16113E-06	1.4268E-06	1.2916E-06	1.4744E-06
Desulfovibrio_hydrothermalis	1.88355E-06	0.000000065	5.69636E-07	9.60364E-07	5.84231E-07	1.36308E-07	8.81538E-08	6.63308E-07	6.99091E-07	7.40091E-07	9.55273E-07	1.51436E-06	6.64667E-07	7.46267E-07	7.08067E-07	1.07967E-06
Desulfovibrio_carbinolicus	1.14445E-06	0.00000026	4.04582E-07	1.09009E-06	6.15154E-07	1.44031E-06	0.00000008	5.85231E-07	7.25364E-07	1.26436E-06	6.27273E-07	1.94845E-06	5.29533E-07	7.994E-07	7.97533E-07	7.41933E-07
Desulfovibrio_ferrophilus	4.57364E-06	1.31991E-06	2.77455E-06	5.42455E-06	2.08669E-06	1.09069E-06	3.11838E-06	5.53108E-06	2.41145E-06	3.24455E-06	0.00000334	3.84091E-06	1.38193E-06	2.08953E-06	2.00587E-06	1.8546E-06
Desulfovibrio_salexigens	2.35536E-06	3.00455E-07	3.15727E-07	1.49955E-06	2.75785E-06	2.66154E-07	9.50308E-07	4.87346E-06	1.38582E-06	1.66318E-06	1.51173E-06	1.84418E-06	1.88367E-06	3.72007E-06	8.29867E-06	6.49933E-06
Desulfovibrio_gigas	1.99345E-06	4.41909E-07	4.39455E-07	1.10127E-06	1.56938E-06	8.95154E-07	8.89231E-07	0.00000113	2.13473E-06	0.000003118	2.91473E-06	5.63336E-06	2.24813E-06	2.78393E-06	2.85667E-06	0.000002154
Desulfovibrio_alaskensis	3.65545E-06	3.24545E-07	5.12436E-07	2.78345E-06	0.000002481	1.34308E-07	1.16823E-06	2.60269E-06	3.11245E-06	4.86755E-06	4.14391E-06	4.08909E-06	3.04527E-06	3.78693E-06	4.97067E-06	4.46933E-06
Desulfovibrio_sulfodismutans	4.49091E-06	1.05936E-06	2.94195E-06	3.20318E-06	1.80446E-06	1.27954E-06	3.17692E-06	3.44154E-06	1.99182E-06	2.81636E-06	2.91545E-06	4.27364E-06	0.000001517	2.47553E-06	3.15347E-06	1.8338E-06
Desulfovibrio_marinus	1.39382E-05	2.68809E-06	9.85955E-06	1.42418E-05	7.78231E-06	1.22446E-05	1.83839E-05	2.30392E-05	1.32382E-05	1.20964E-05	1.18555E-05	1.13155E-05	9.95933E-06	9.05067E-06	9.38667E-06	8.45267E-06
Candidatus_Desulfovibrio_trichonymphae	8.92636E-06	7.24091E-07	4.53545E-07	4.82882E-06	7.00146E-06	7.35231E-07	1.06546E-06	5.89508E-06	4.83273E-06	8.24409E-06	7.24691E-06	6.52182E-06	5.97227E-06	9.26067E-06	1.15513E-05	8.75667E-06
Desulfovibrio_desulfuricans	1.47373E-05	4.25864E-06	1.44927E-06	9.72636E-06	8.98892E-06	3.21923E-06	2.67415E-06	6.94669E-06	1.04682E-05	1.80818E-05	1.35455E-05	1.56273E-05	1.06013E-05	1.74407E-05	2.19486E-05	1.49873E-05
Desulfovibrio_sp._G11	1.89091E-07	5.84545E-07	0	2.59091E-08	5.62846E-07	0	0	0.000000011	9.91818E-08	0	3.37818E-07	1.90182E-07	1.30047E-06	2.47987E-06	3.78833E-06	3.0792E-06
Desulfovibrio_sp._IOR2	3.77418E-06	0.000002215	7.60636E-07	2.72773E-06	3.10185E-06	1.38154E-06	1.47654E-06	1.97569E-06	4.06091E-06	4.31818E-06	4.72273E-06	4.52009E-06	1.7582E-06	3.65073E-06	3.00693E-06	2.80667E-06
Desulfovibrio_sp._86	1.37527E-05	1.27827E-06	2.89964E-06	8.11709E-06	9.07231E-06	9.75231E-07	7.89846E-06	9.33923E-06	9.04364E-06	1.46864E-05	9.27182E-06	1.22209E-05	1.09793E-05	1.97367E-05	1.88047E-05	1.63253E-05
Desulfovibrio_vulgaris	1.92836E-05	2.07145E-06	5.75275E-06	9.19455E-06	1.98892E-05	1.58554E-06	7.93308E-06	0.00001908	0.0000203	3.93306E-05	3.28521E-05	4.16545E-05	2.09807E-05	3.91529E-05	4.57285E-05	3.45267E-05
Desulfovibrio_piger	3.69317E-05	5.96964E-06	4.65109E-06	1.83999E-05	3.64292E-05	3.81823E-06	8.60231E-06	3.50377E-05	2.65764E-05	5.37019E-05	3.44152E-05	4.27066E-05	3.67377E-05	5.81616E-05	7.63491E-05	5.82202E-05
Desulfovibrio_fairfieldensis	3.49305E-05	3.91091E-06	2.00927E-06	1.89591E-05	5.15335E-05	1.63785E-06	6.50615E-06	1.19493E-05	2.34245E-05	4.81436E-05	3.28004E-05	3.61082E-05	0.000122924	0.000300948	0.000343959	0.000269151
Nitratiruptor_labii	1.73909E-07	1.93909E-07	1.23273E-07	6.78182E-08	2.87846E-07	2.67769E-07	4.92308E-07	3.52615E-07	6.14545E-08	1.98545E-07	3.72727E-07	2.38091E-07	4.32133E-07	8.23333E-08	2.624E-07	3.174E-07
Nitratiruptor_sp._SB155-2	7.37273E-08	1.78273E-07	6.26182E-08	4.87273E-08	1.03354E-06	8.84615E-08	0	3.81538E-08	5.37273E-08	1.48273E-07	1.38455E-07	1.99909E-07	8.17133E-07	8.58533E-07	8.73867E-07	9.12933E-07
Cetia_pacifica	2.96364E-08	1.77273E-08	4.12727E-09	0	1.35538E-07	2.95385E-08	0	2.12769E-07	0.000000129	0	0	9.59091E-08	5.58667E-08	0.000000306	1.75667E-07	0.000000097
Caminibacter_mediatlanticus	2.54182E-07	2.41818E-08	3.70909E-08	3.76364E-08	7.68077E-07	2.21846E-07	9.14615E-08	1.47385E-07	1.60455E-07	1.17273E-07	5.87636E-07	7.11545E-07	7.84067E-07	1.0812E-06	1.2694E-06	9.82333E-07
Nautilia_profundicola	1.22455E-07	0	7.82182E-08	1.45791E-07	1.87231E-07	4.39308E-07	3.77692E-08	1.90769E-08	1.96182E-07	3.89091E-08	0.000000061	0.000000157	1.06933E-07	1.12533E-07	3.12133E-07	8.08667E-08
Nautilia_sp._PV-1	6.83727E-07	5.15455E-08	0	0	2.58623E-06	5.91538E-08	3.33077E-08	2.06615E-06	8.43636E-08	0	0	0	1.75447E-06	1.55987E-06	3.57313E-06	2.71073E-06
Nitratifractor_salsuginis	2.94027E-06	1.15009E-06	3.82391E-06	3.51091E-06	2.99692E-07	4.83154E-07	0.000002625	3.03431E-06	1.11245E-06	1.57009E-06	2.71655E-06	2.52091E-06	0.000000744	1.0122E-06	9.968E-07	1.02093E-06
Sulfurovum_lithotrophicum	0.000000088	2.26364E-07	7.97273E-08	0	3.15385E-08	2.52308E-08	1.48769E-07	1.00154E-07	1.24727E-07	0	9.44545E-08	1.79636E-07	4.86667E-08	5.73333E-08	1.81067E-07	0.000000059
Sulfurovum_sp._NBC37-1	8.62636E-07	4.48818E-07	2.28636E-07	6.78545E-07	2.55846E-07	6.38385E-07	1.32085E-06	3.48154E-07	6.01545E-07	4.34909E-07	0.000000295	5.06455E-07	2.378E-07	3.64067E-07	2.94867E-07	6.68733E-07
Sulfuricurvum_kujiense	2.47636E-07	2.88727E-07	1.56273E-07	6.44364E-07	2.86846E-07	5.00769E-07	0.00000123	2.79154E-07	3.12818E-07	5.09182E-07	1.12227E-06	8.45364E-07	5.65267E-07	5.91733E-07	3.85333E-07	7.356E-07
Sulfurimonas_sp._1-1N	0	0	0	0	6.47692E-08	0	0	0	7.89091E-08	0	0	0	6.08E-08	0.000000033	0	3.67333E-08
Sulfurimonas_sp._CVO	2.96364E-08	0	0	5.18182E-09	2.75385E-08	2.52308E-08	0	0	0	0	0	0	8.22667E-08	2.38E-08	1.052E-07	2.05133E-07
Candidatus_Sulfurimonas_baltica	0.000000133	0	0	5.18182E-09	2.89231E-07	0	4.33846E-08	4.64615E-08	1.75455E-07	2.00545E-07	6.71818E-08	7.82273E-08	9.19333E-08	1.046E-07	1.64533E-07	1.17467E-07

Sulfurimonas_sp._S2-6	9.44636E-07	8.58182E-08	2.09182E-07	0.00000013	4.43077E-07	1.30769E-07	4.52846E-07	1.21069E-06	8.27273E-07	1.54782E-06	1.25391E-06	1.47464E-06	5.64067E-07	1.322E-07	9.98667E-08	5.51867E-07
Candidatus_Sulfurimonas_marisnigri	2.98182E-08	0	0	0	1.50615E-07	0	0	1.90769E-08	4.45455E-08	0	1.02727E-08	1.42727E-08	2.37333E-08	1.674E-07	1.82667E-07	8.78E-08
Sulfurimonas_paralvinellae	1.07709E-06	0	3.65545E-07	1.09582E-06	3.43385E-07	0	3.16846E-07	6.75846E-07	1.34691E-06	8.11455E-07	1.57109E-06	1.45491E-06	0.000000697	5.82933E-07	7.70133E-07	6.81133E-07
Sulfurimonas_gotlandica	1.11818E-07	0	7.40909E-08	1.41736E-06	1.20108E-06	8.59231E-08	5.56231E-07	1.23185E-06	0.000000081	3.36091E-07	0.000000574	1.12827E-06	0.000001835	1.27373E-06	2.69207E-06	2.77813E-06
Sulfurimonas_denitrificans	6.98182E-07	6.50909E-07	2.30091E-07	2.32545E-07	1.55223E-06	1.52692E-07	1.65715E-06	8.83308E-07	2.15727E-07	0.000000047	5.72727E-07	7.55091E-07	2.4554E-06	1.63173E-06	1.91467E-06	1.66273E-06
Sulfurimonas_sp._B2	0	0	3.27273E-08	9.54545E-09	4.93846E-08	0	0	0	0	0	0	1.42727E-08	2.14667E-08	3.48E-08	9.91333E-08	1.084E-07
Sulfurimonas_sp._GYSZ_1	6.80909E-08	0	0	0	3.58462E-08	0	0	0	0	0.000000034	6.71818E-08	0	0	6.98667E-08	8.86667E-09	0
Sulfurimonas_sp._NW10	1.10336E-06	3.19045E-07	1.43727E-07	1.08909E-07	0.000001576	9.45385E-08	1.90923E-07	2.11308E-07	1.53636E-07	8.62727E-08	4.65182E-07	1.80182E-07	1.28173E-06	1.16333E-06	1.72827E-06	1.1758E-06
Sulfurimonas_autotrophica	2.05245E-06	0	0	0	6.68423E-06	1.40308E-07	2.73846E-08	0	0	0	1.10909E-07	8.71909E-07	3.2558E-06	3.21453E-06	3.89027E-06	2.44327E-06
Aliarcobacter_faecis	0	0	0	0	5.47692E-08	8.24615E-08	6.41538E-08	1.90769E-08	0	0	0	0	3.166E-07	2.214E-07	1.80333E-07	3.26267E-07
[Arcobacter]_porcinus	2.96364E-08	0	2.59818E-08	9.54545E-09	9.22308E-08	0	3.06923E-08	2.52154E-07	0	3.89091E-08	3.79091E-08	0	9.33333E-08	2.30133E-07	1.544E-07	0.000000451
Sulfurospirillum_barnesii	0	0	0	4.79091E-09	0	1.23846E-07	0	1.90769E-08	0	0	0	0	7.42667E-08	0	0	1.98667E-08
Sulfurospirillum_halorespirans	2.98182E-08	0	7.66364E-08	3.24545E-08	0	0	1.77846E-07	2.47692E-08	0	0	0	7.13636E-09	2.08E-08	0	0	0
Sulfurospirillum_sp._JPD-1	0	1.87273E-08	1.77273E-08	1.43636E-08	3.58462E-08	0	3.11538E-08	5.66923E-08	0	0	0	0	4.23333E-08	0	2.97333E-08	0
Sulfurospirillum_multivorans	1.58182E-08	0	4.12727E-09	9.54545E-09	0	0	2.63077E-08	1.34308E-07	0	0	0.000000103	1.42727E-08	0	0	3.13333E-08	1.71133E-07
Sulfurospirillum_deleyianum	0	2.70909E-08	1.874E-07	1.82455E-07	0.000000211	0	6.40769E-08	6.16154E-08	0	5.67273E-08	2.67455E-07	7.44545E-08	2.53267E-07	1.704E-07	2.89533E-07	2.25267E-07
Sulfurospirillum_cavolei	5.82364E-07	5.17273E-08	7.99091E-08	3.38091E-07	2.05769E-07	6.98462E-08	4.89231E-07	9.05385E-08	0.000000511	1.88455E-07	4.77091E-07	4.80636E-07	2.22933E-07	2.13267E-07	3.70333E-07	2.72933E-07
Sulfurospirillum_sp._UCH001	1.32564E-06	5.75909E-07	2.93909E-07	3.22455E-07	7.36385E-07	5.91538E-08	3.35385E-07	9.98462E-08	0.000000344	3.13273E-07	0.000000193	0.000000659	7.75467E-07	6.48267E-07	7.51933E-07	4.77933E-07
Halarcobacter_ebronensis	0.000000555	0	1.63636E-08	5.84545E-08	0.000000289	0	0	0	1.96273E-07	1.39455E-07	1.30909E-07	4.21364E-08	4.19867E-07	3.07467E-07	7.93267E-07	6.85667E-07
Halarcobacter_bivalviorum	0	0	2.04909E-08	4.79091E-09	2.59231E-08	2.52308E-08	0	0	2.56909E-07	2.07545E-07	2.00909E-07	3.57273E-08	0	1.00867E-07	7.05333E-08	2.70533E-07
Pseudoarcobacter_acticola	8.25364E-07	8.98136E-07	3.08182E-07	1.27009E-06	7.11923E-07	6.62308E-07	1.47192E-06	4.84462E-07	9.96091E-07	1.02318E-06	8.53727E-07	1.51545E-06	2.32267E-07	4.83667E-07	2.13667E-07	5.11067E-07
Arcobacter_molluscorum	2.04455E-07	0.000000065	1.63091E-07	7.58182E-08	5.27077E-07	1.67538E-07	0	1.18615E-07	1.08273E-07	2.43818E-07	2.76364E-08	2.21727E-07	9.41333E-08	1.236E-07	1.69067E-07	2.29533E-07
Arcobacter_mytili	3.84273E-07	0.000000361	1.52309E-07	2.07273E-08	2.88462E-08	0	6.44615E-08	3.83615E-07	1.45818E-07	0	2.39727E-07	3.10409E-07	8.22667E-08	3.09333E-08	1.49333E-08	2.20267E-07
Arcobacter_canalis	2.23727E-07	0	4.99727E-08	6.62455E-08	2.51615E-07	0	3.11538E-08	8.65385E-08	4.18091E-07	4.69727E-07	3.11273E-07	2.94545E-07	0.000000392	2.04067E-07	1.572E-07	1.37467E-07
Arcobacter_pacificus	0.000000409	4.13273E-07	6.75282E-07	4.21273E-07	2.18154E-07	0	9.79692E-07	1.99077E-07	3.90364E-07	1.81818E-07	6.89545E-07	6.67364E-07	0	2.842E-07	0.000000219	4.59133E-07
Arcobacter_marinus	1.80091E-07	0	0	1.74818E-07	3.95923E-07	0	1.51538E-07	1.78308E-07	0	0	0	3.57273E-08	5.056E-07	5.398E-07	5.80333E-07	4.52533E-07
Arcobacter_halophilus	2.96364E-08	0	3.27273E-08	0	0.000000174	0	0	1.90769E-08	0	0	7.23636E-08	8.55455E-08	2.86067E-07	0.00000021	8.96E-08	4.34E-08
Arcobacter_aquimarinus	0	0	0	4.40909E-08	6.78462E-08	0	0	1.90769E-08	0	0	0	1.42727E-08	0	3.11333E-08	1.57067E-08	0
Arcobacter_butzleri	0.000000061	0.000000065	0	2.39091E-08	1.65538E-07	0	0	2.51538E-07	7.15455E-08	5.65455E-08	1.24091E-07	4.92727E-08	1.514E-07	9.70667E-08	1.41773E-07	6.12667E-08
Arcobacter_nitrofigilis	0.000000092	0	1.63636E-08	0	2.05385E-08	2.95385E-08	0	2.51538E-07	0	7.77273E-08	2.76364E-08	7.89091E-08	0	0.000000033	4.44E-09	4.04E-08
Arcobacter_lanthieri	4.24727E-06	1.44418E-06	1.12936E-06	4.21818E-07	0.00000152	0.000000026	1.45338E-06	1.53908E-06	5.71455E-07	5.47636E-07	6.27091E-07	2.19182E-06	7.36667E-08	3.43733E-07	3.62067E-07	1.52733E-07
Arcobacter_thereius	1.44727E-07	1.00909E-07	8.25455E-09	0	7.24615E-08	0	3.77692E-08	9.84615E-08	0	2.15727E-07	7.90909E-08	1.62545E-07	4.01333E-08	2.38E-08	3.064E-08	1.86667E-08
Arcobacter_skirrowii	5.67182E-07	2.53636E-07	0.000000067	2.04273E-07	4.05615E-07	0	1.29538E-07	7.66692E-07	7.51727E-07	4.64182E-07	9.19273E-07	4.67091E-07	6.95067E-07	3.53867E-07	7.78733E-07	5.11933E-07
Arcobacter_ellisii	1.58182E-08	0	8.75455E-09	0	7.24615E-08	0	0	1.46923E-08	8.10909E-08	6.27273E-08	2.76364E-08	0.000000057	7.03333E-08	1.35933E-07	1.45333E-07	3.67333E-08
Arcobacter_anaerophilus	3.69091E-08	1.68182E-08	0	2.41545E-08	1.82769E-07	0	3.11538E-08	3.27385E-07	4.86364E-08	1.90909E-07	1.70636E-07	8.26818E-08	4.27333E-08	0.00000021	5.49867E-07	7.54333E-07
Arcobacter_trophiarum	0	0	0	0	1.11154E-07	0	4.01385E-07	2.14154E-07	0	0	1.04545E-07	1.80909E-07	2.10467E-07	8.54667E-08	2.69067E-08	1.642E-07
Arcobacter_suis	8.69091E-08	2.70909E-08	1.10909E-07	4.88182E-08	0	0	3.81385E-07	2.36154E-07	2.11545E-07	2.85091E-07	3.78364E-07	4.65909E-08	4.23333E-08	1.354E-07	1.35667E-07	0.000000068
Arcobacter_peruensis	4.32273E-07	0	5.89455E-08	2.43636E-08	4.14462E-07	0	0	1.54308E-07	1.61909E-07	7.57273E-08	2.25091E-07	1.04091E-07	5.564E-07	4.036E-07	5.98533E-07	5.126E-07
Arcobacter_cloacae	4.34091E-07	0	3.70909E-08	2.07273E-08	1.25854E-06	0	9.88462E-08	4.93846E-08	9.40909E-08	9.92727E-08	2.76364E-08	2.00455E-07	5.64733E-07	5.86733E-07	9.494E-07	6.63667E-07
Arcobacter_lekithochrous	0	0	0	5.13727E-07	1.24585E-06	0	4.20769E-07	7.16231E-07	9.77273E-08	1.22182E-07	6.79545E-07	7.25909E-07	1.47007E-06	0.000000464	1.42787E-06	2.58407E-06
Arcobacter_venerupis	4.61455E-07	1.16364E-08	1.15036E-07	1.09455E-07	1.04362E-06	7.37154E-07	5.40615E-07	8.22308E-07	2.15273E-07	7.55455E-08	7.34182E-07	3.91273E-07	5.52733E-07	6.494E-07	9.54733E-07	5.314E-07
Arcobacter_cibarius	0.000000066	5.54273E-07	1.30091E-07	0.000000699	8.73538E-07	3.30769E-07	0.000000176	8.88308E-07	3.23636E-07	3.78727E-07	9.37909E-07	1.00291E-06	9.284E-07	8.802E-07	2.26393E-06	2.1894E-06
Arcobacter_defluvii	6.10909E-07	0	1.08455E-07	1.41909E-07	1.21454E-06	0	0	5.48462E-08	7.89091E-08	1.09545E-07	1.06364E-07	2.62364E-07	9.34267E-07	1.35107E-06	1.1816E-06	1.0388E-06
Arcobacter_cryaerophilus	1.31118E-06	0	1.228E-07	2.39273E-07	3.67769E-06	0	5.01846E-07	1.35292E-06	0.0000016	1.32845E-06	1.75364E-06	2.14673E-06	3.52367E-06	2.90773E-06	3.55067E-06	3.25453E-06
Campylobacter_canadensis	2.96364E-08	0	0	1.03273E-07	2.59231E-08	5.91538E-08	2.73846E-08	0.000000066	0	0	0	0	1.148E-07	1.092E-07	3.13333E-08	1.09867E-07
Campylobacter_sp._RM16192	0	0	0.000000046	4.94545E-08	1.08308E-07	0	0	0	3.75455E-08	0	3.77273E-08	0	2.29267E-07	1.058E-07	4.12E-08	0.000000026
Campylobacter_rectus	6.78182E-08	0	3.70909E-08	2.39091E-08	0	0	2.63077E-08	4.93846E-08	0	0	0	0	0	0	0	0
Campylobacter_blaseri	3.20091E-07	0.000000065	0	1.51545E-08	1.19154E-07	8.80769E-07	0	5.74615E-08	4.45455E-08	0	2.76364E-08	0.000000057	0.000000048	4.64667E-08	1.49333E-08	7.33333E-08
Campylobacter_sp._CCUG_57310	2.99636E-07	2.34545E-07	2.13727E-07	7.68182E-08	0.000000039	0	0	1.90769E-08	0.000000463	3.05364E-07	4.11364E-07	1.73227E-07	4.28667E-08	0	2.83733E-08	2.084E-07
Campylobacter_armoricus	2.06818E-07	0	1.85455E-07	4.50909E-08	0	0	1.28462E-07	1.21615E-07	3.75455E-08	3.62727E-08	1.73273E-07	0.000000066	0	0.000000066	4.44E-09	1.86E-08
Campylobacter_showae	4.82727E-07	2.30818E-07	1.06818E-07	0.000000359	2.61308E-07	2.16923E-08	1.34154E-07	2.32154E-07	3.71727E-07	4.59455E-07	6.18364E-07	8.98273E-07	9.15333E-08	2.02667E-07	3.544E-07	2.61467E-07
Campylobacter_lanienae	1.13091E-07	0	4.12727E-09	1.72545E-07	2.35769E-07	0	3.77692E-08	1.00846E-07	0.000000098	6.37273E-08	0	1.68273E-07	1.02533E-07	9.06667E-08	0.000000041	0
Campylobacter_subantarcticus	6.74545E-08	0	1.15036E-07	0	1.18308E-07	0	4.33846E-08	3.81538E-08	3.75455E-08	0	3.33636E-08	0	9.06E-08	8.52E-08	1.67533E-07	3.97333E-08
Campylobacter_hyointestinalis	3.27909E-07	6.10909E-08	1.73491E-07	8.06091E-08	1.83385E-07	1.26823E-06	2.09769E-07	4.91385E-07	3.71273E-07	1.59727E-07	5.33455E-07	1.97182E-07	3.00133E-07	5.51467E-07	1.69667E-07	4.124E-07
Campylobacter_corcagiensis	6.67273E-08	0	3.29091E-08	2.89091E-08	2.59231E-08	0	7.43846E-08	0	3.75455E-08	5.22727E-08	1.67182E-07	1.21727E-07	1.51333E-07	0	1.20333E-07	5.84667E-08
Campylobacter_sp._RM6137	0	0	5.14909E-08	1.26455E-07	2.31308E-07	2.52308E-08	4.44615E-07	0	8.91818E-08	1.45909E-07	7.29091E-08	8.09091E-08	8.17333E-08	4.02E-08	1.584E-07	3.84667E-08
Campylobacter_sp._CGEMS	5.47364E-07	9.99636E-07	3.55909E-07	7.97364E-07	7.35538E-07	2.47392E-06	6.30769E-07	7.31154E-07	5.77182E-07	6.30727E-07	0.000000467	0.000001128	5.51867E-07	5.87733E-07	8.43667E-07	5.44733E-07
Campylobacter_sp._RM16704	0.00000006	0	9.94545E-08	1.10273E-07	0.000000142	0	2.08538E-07	1.32308E-07	1.03818E-07	1.38545E-07	1.61091E-07	1.06364E-07	7.16E-08	0	2.97333E-08	0
Campylobacter_gracilis	2.43027E-06	1.17773E-07	1.16776E-06	2.33482E-06	5.45923E-07	0	1.52208E-06	1.51062E-06	1.06764E-06	1.43718E-06	8.08182E-07	1.01282E-06	3.242E-07	6.954E-07	3.32733E-07	4.94067E-07
Campylobacter_volucris	2.35455E-07	0	2.47273E-08	1.24791E-07	4.27077E-07	0	1.73846E-07	4.64077E-07	8.92455E-07	3.01091E-07	6.66636E-07	5.97364E-07	7.054E-07	4.628E-07	4.304E-07	0.000000479
Campylobacter_concisus	2.46182E-07	2.34545E-08	5.37273E-08	6.96364E-08	1.48769E-07	3.35692E-07	1.51538E-07	0	3.43455E-07	3.60364E-07	2.16636E-07	1.21518E-06	1.462E-07	3.518E-07	4.60267E-07	3.27933E-07
Campylobacter_hepaticus	1.61091E-07	0	1.63636E-08	7.55636E-07	9.61308E-07	0	3.11154E-07	5.54538E-07	0	1.04273E-07	2.85727E-07	6.49682E-07	9.72133E-07	7.64267E-07	0.000001368	2.58007E-06
Campylobacter_ureolyticus	2.22691E-06	0.00000078	1.42958E-06	3.51273E-07	3.32385E-07	4.13846E-08	3.33077E-08	0.000000167	7.93818E-07	2.20182E-07	8.17545E-07	1.90827E-06	2.59333E-07	2.57867E-07	2.09867E-07	1.404E-07
Campylobacter_avium	7.15455E-08	2.34545E-08	6.67273E-08	2.95455E-08	3.71538E-07	0	3.06923E-08	2.46923E-08	2.71636E-07	5.22727E-08	1.31455E-07	2.84182E-07	9.96933E-07	6.034E-07	6.312E-07	4.00467E-07
Campylobacter_peloridis	0.000000784	8.41818E-08	2.03818E-07	1.85791E-06	4.07769E-07	5.18769E-07	1.83146E-06	4.05315E-06	1.62182E-07	0.000000185	1.67727E-06	2.24009E-06	0.000000385	4.90067E-07	1.37047E-06	6.014E-07
Campylobacter_mucosalis	0	9.36364E-09	0	0	2.18846E-07	0	0	0	0	0	0	0	5.13067E-07	4.366E-07	4.112E-07	4.40467E-07
Campylobacter_hominis	2.04091E-07	0.00000013	3.27273E-08	9.93636E-08	1.19831E-06	3.94308E-07	0	0	0	0	0	0.000000057	2.0196E-06	8.45733E-07	9.12533E-07	7.64333E-07
Campylobacter_iguaniorum	1.13827E-06	1.17273E-06	3.67545E-07	9.87273E-07	8.50538E-07	0	6.64692E-07	8.70923E-07	1.07136E-06	4.83091E-07	7.19818E-07	6.06364E-07	9.48333E-07	1.01967E-06	9.89133E-07	8.19733E-07
Campylobacter_geochelonis	2.03727E-07	0.00000013	4.58455E-08	1.54636E-07	5.25308E-07	5.09231E-08	1.14923E-07	6.79231E-08	9.93636E-08	8.62727E-08	2.28545E-07	5.70909E-08	3.09333E-07	5.928E-07	5.97133E-07	2.03333E-07
Campylobacter_sputorum	0	9.36364E-09	0	4.57091E-07	8.32385E-07	0	3.15615E-07	5.43308E-07	0.000000054	0	4.62455E-07	3.21545E-07	1.31173E-06	9.55267E-07	1.6318E-06	2.49173E-06
Campylobacter_insulaenigrae	2.96364E-08	0	3.40909E-08	1.25727E-07	1.65462E-07	0	1.77846E-07	1.46923E-08	0	0	2.74727E-07	0.000000075	2.39133E-07	4.202E-07	2.36933E-07	2.916E-07
Campylobacter_cuniculorum	1.18882E-06	3.98364E-07	5.54364E-07	8.87545E-07	0.000001349	1.99846E-07	5.15769E-07	3.79846E-07	6.96455E-07	3.34091E-07	0.000000904	1.03736E-06	1.76813E-06	1.04647E-06	1.32373E-06	1.23147E-06
Campylobacter_curvus	3.15455E-08	0	0	1.27818E-07	5.74231E-07	0	0	1.90769E-08	8.43636E-08	8.60909E-08	5.41909E-07	2.14545E-08	1.6826E-06	0.000000849	7.91933E-07	0.000000593
Campylobacter_ornithocola	1.29455E-07	3.24545E-07	2.18545E-08	3.64364E-07	1.55308E-07	5.87692E-07	3.06462E-07	1.35231E-07	7.15455E-08	9.40909E-08	9.31818E-08	0.000000103	4.78667E-08	4.94533E-07	2.94467E-07	0.000000182
Campylobacter_coli	1.35964E-06	0.000000103	1.47909E-07	0.000000099	1.84677E-06	8.25692E-07	6.00538E-07	7.09462E-07	6.11909E-07	6.18909E-07	8.73455E-07	9.06545E-07	1.30013E-06	1.50827E-06	1.53647E-06	1.59387E-06
Campylobacter_helveticus	2.86727E-07	5.42727E-07	5.83636E-08	1.08273E-07	2.49523E-06	0.000000026	7.82308E-07	4.07769E-07	1.24273E-07	3.27727E-07	0.000000165	2.91682E-07	4.66733E-06	0.000001797	2.6022E-06	1.89593E-06
Campylobacter_jejuni	1.02345E-06	2.6975E-06	1.29358E-06	1.91209E-06	0.000002264	1.02015E-06	2.90562E-06	5.06623E-06	0.000000717	8.83182E-07	8.56545E-07	1.91018E-06	0.00000411	2.45267E-06	2.18447E-06	1.87007E-06
Campylobacter_fetus	2.19364E-07	0	1.63636E-08	5.74545E-08	1.26638E-06	5.09231E-08	4.33846E-08	0.000000644	1.02364E-07	0	1.80455E-07	1.49545E-07	1.4374E-06	1.7396E-06	2.1402E-06	1.66453E-06
Campylobacter_lari	1.47991E-06	0.000000904	7.82182E-08	6.88909E-07	2.62169E-06	0	4.51923E-07	7.86538E-07	7.03455E-07	0.000000367	9.20545E-07	1.03727E-07	2.16547E-06	2.13907E-06	2.1684E-06	1.8128E-06
Campylobacter_sp._RM8964	0	0	0	0	5.10615E-07	0	0	0	0	3.62727E-08	0	0	2.90733E-07	2.96133E-07	4.83933E-07	8.90467E-07
Campylobacter_sp._RM6914	1.33773E-06	0.000000065	3.18455E-07	1.19873E-06	1.02869E-06	0	8.17692E-08	3.91769E-07	1.01936E-06	7.08091E-07	0.000001033	1.01318E-06	2.1234E-06	1.56427E-06	9.336E-07	1.33913E-06
Campylobacter_upsaliensis	2.93818E-07	5.89909E-07	3.25455E-07	8.81364E-07	0.000002383	9.72308E-08	0.000000311	1.26077E-07	0.000000459	7.27273E-07	7.37727E-07	5.64273E-07	4.51367E-06	2.18867E-06	2.4498E-06	2.30813E-06
Campylobacter_pinnipediorum	3.44691E-06	5.93409E-07	4.48673E-07	2.97909E-07	7.15738E-06	0	6.91077E-07	4.25231E-07	6.36091E-07	9.96545E-07	4.43364E-07	1.26909E-06	5.2822E-06	4.77727E-06	5.86167E-06	2.7548E-06
Wolinella_succinogenes	1.73264E-06	1.94545E-07	0	2.51818E-07	2.03154E-07	2.13846E-08	5.41462E-07	1.54408E-06	3.66091E-07	5.36455E-07	7.64818E-07	1.27705E-06	4.04467E-07	2.92533E-07	6.392E-07	7.55667E-07
Helicobacter_felis	0	0	0	0	7.50769E-08	0	0	0	0	0	0	0	1.00467E-07	1.28933E-07	4.78667E-08	1.78667E-07
Helicobacter_heilmannii	2.70909E-08	0	0	6.96091E-08	4.60769E-07	0	0	0	3.75455E-08	7.57273E-08	3.33636E-08	0.00000007	5.71333E-07	2.50067E-07	3.82733E-07	3.556E-07
Helicobacter_sp._MIT_01-6242	1.91091E-07	0	1.77273E-08	1.91818E-08	4.90385E-07	2.16923E-08	1.20385E-07	0	2.68273E-07	3.55727E-07	5.82727E-08	2.98818E-07	0.000001287	6.26867E-07	1.0356E-06	1.46813E-06
Helicobacter_mustelae	3.00182E-07	2.02091E-07	0	1.60545E-07	5.25615E-07	1.01692E-07	3.20385E-07	0.000000022	1.02455E-07	1.11818E-07	2.99727E-07	4.63182E-07	1.05127E-06	3.87867E-07	1.0264E-06	1.1828E-06
Helicobacter_bizzozeronii	2.36909E-07	1.37273E-08	0	0	6.92308E-07	0	0	0	0	5.22727E-08	1.02727E-08	5.30909E-08	0.0000007	8.644E-07	7.94533E-07	6.91333E-07
Helicobacter_canadensis	0.000002372	1.94545E-07	4.07273E-07	5.08727E-07	1.52115E-06	0	1.41385E-06	9.40538E-07	5.57909E-07	2.12818E-07	6.98636E-07	0.000000645	2.53387E-06	2.04287E-06	1.80153E-06	2.26727E-06
Helicobacter_acinonychis	3.11818E-08	0	0	0	8.26923E-07	0	0	1.13846E-07	2.64545E-08	5.70909E-08	8.02727E-08	0	2.13747E-06	8.256E-07	1.01007E-06	1.2154E-06
Helicobacter_cetorum	8.09091E-08	0	2.04909E-08	4.79091E-09	1.37485E-06	0	1.18846E-07	1.69385E-07	0.000000053	0.000000034	1.33273E-07	1.02591E-07	2.84007E-06	1.31447E-06	1.72353E-06	1.2806E-06
Helicobacter_suis	3.62455E-07	0.00000039	8.63636E-08	0.000000258	2.26923E-07	0	0.000000458	8.19231E-08	2.11182E-07	2.68818E-07	1.41273E-07	2.13182E-07	3.27533E-07	5.31733E-07	3.436E-07	8.00267E-07
Helicobacter_pullorum	4.33182E-07	7.66364E-08	7.82182E-08	9.09727E-08	2.55515E-06	0	1.16846E-07	3.08923E-07	1.46455E-07	0.00000015	8.15455E-08	4.53273E-07	5.21667E-06	2.13553E-06	2.95887E-06	2.7248E-06
Helicobacter_cholecystus	0	0	0	0	1.42769E-06	0	0	5.51538E-08	0	0	0	0	3.22093E-06	1.63747E-06	2.5182E-06	2.23633E-06
Helicobacter_canis	0	0	0	0	8.50846E-06	4.13846E-08	0	0	0	0	0	0	0.000019128	0.000007152	8.49427E-06	7.85307E-06
Helicobacter_pylori	9.82273E-07	3.43209E-06	6.62182E-07	1.27827E-06	6.83277E-06	1.97846E-07	5.95615E-07	1.12762E-06	9.54727E-07	8.05636E-07	7.74182E-07	1.63091E-06	1.30753E-05	0.000005726	7.22867E-06	7.37733E-06
Helicobacter_himalayensis	3.12091E-06	4.27464E-06	1.25255E-06	2.74436E-06	1.45608E-05	4.46523E-06	2.58923E-06	2.83246E-06	3.92018E-06	5.14055E-06	2.75636E-06	3.74109E-06	0.00002712	0.000013674	0.000013518	1.31767E-05
Helicobacter_apodemus	2.96364E-08	0	0	5.24545E-08	0.00002494	8.05385E-08	2.38308E-07	2.07615E-07	7.89091E-08	0	7.90909E-08	0	4.38855E-05	0.00001818	0.0000228	1.96167E-05
Helicobacter_bilis	3.11818E-08	4.11818E-08	3.70909E-08	5.18182E-09	0.00002566	0	0	2.38462E-07	4.45455E-08	1.56818E-07	2.20273E-07	1.42727E-08	5.43207E-05	2.41773E-05	0.00002536	2.17973E-05
Helicobacter_cinaedi	0.000000863	6.67773E-07	0.00000031	7.33273E-07	0.000219094	7.63077E-07	0.000001283	1.74231E-07	4.42091E-07	7.21909E-07	5.64182E-07	6.18727E-07	0.000423371	0.000167034	0.000194204	0.000182664
Helicobacter_hepaticus	3.10209E-05	1.16364E-08	6.67273E-08	8.58245E-07	0.001013103	2.03692E-06	9.61231E-07	0.000001859	0.000003692	1.99991E-06	3.75555E-06	1.46936E-06	0.001001966	0.000580767	0.000636764	0.000545327
Helicobacter_typhlonius	0.000101653	1.72591E-06	2.28818E-07	3.58273E-07	0.201117441	0.000305039	0.000000223	1.84408E-06	0.000100534	6.15273E-06	0.000000473	9.35455E-07	0.087792867	0.090228814	0.088097927	0.07271049
Fimbriimonas_ginsengisoli	2.62809E-06	0.000000206	4.58718E-07	1.10991E-06	1.30823E-06	8.88462E-08	8.68615E-07	2.64762E-06	1.56418E-06	1.15064E-06	2.91482E-06	2.65636E-06	1.3398E-06	1.04987E-06	1.36333E-06	1.2134E-06
Chthonomonas_calidirosea	7.21545E-07	7.43636E-08	7.82182E-08	3.22636E-07	3.12538E-07	2.32077E-06	2.35462E-06	1.98115E-06	0.000000237	4.02545E-07	3.98455E-07	6.85727E-07	5.07867E-07	8.93467E-07	6.62933E-07	3.73533E-07
Truepera_radiovictrix	5.46273E-07	2.20227E-07	2.09127E-07	7.33364E-07	7.32308E-08	9.81538E-08	0	2.56923E-08	8.49091E-08	1.21545E-07	1.02727E-08	0.000000268	1.582E-07	2.404E-07	2.586E-07	1.824E-07
Deinococcus_soli_Cha_et_al._2016	0	0	0	0	2.75385E-08	0	0	3.22308E-08	0	0	0	0	0	0	0	3.73333E-08
Deinococcus_swuensis	0	0	0	0	0	0	0	2.46923E-08	3.75455E-08	0.000000028	5.52727E-08	6.73636E-08	4.78E-08	0.000000033	1.49333E-08	7.26667E-08
Deinococcus_actinosclerus	4.14545E-08	0	0	1.20455E-07	1.04462E-07	1.23692E-07	1.51538E-07	1.93692E-07	1.57273E-07	0	2.27364E-07	5.02364E-07	1.142E-07	6.04667E-08	1.332E-07	8.51333E-08
Deinococcus_perariditoris	6.89091E-07	2.26864E-07	9.01818E-08	2.07364E-07	8.30769E-08	3.89308E-07	3.11538E-08	3.60692E-07	2.62182E-07	1.28545E-07	3.44182E-07	8.18364E-07	5.66667E-08	1.15733E-07	3.61533E-07	3.08E-08
Deinococcus_sp._S14-83	2.79909E-07	0	8.65455E-08	1.83091E-07	9.89231E-08	0	1.26385E-07	1.46615E-07	1.29091E-07	0.000000316	0.000000366	3.99364E-07	0.000000061	8.83333E-08	1.15867E-07	4.514E-07
Deinococcus_sp._NW-56	0.000000282	3.34091E-07	7.40909E-08	5.05909E-07	0.00000026	1.41538E-07	1.45154E-07	3.60462E-07	5.04091E-07	1.46909E-07	6.29364E-07	1.05873E-06	5.01333E-08	3.50667E-08	0.000000291	1.69133E-07
Deinococcus_metallilatus	7.83636E-08	3.49091E-08	4.12182E-08	2.07091E-07	0.000000151	4.94538E-07	1.92308E-07	3.40846E-07	0.000000248	2.34545E-07	2.03818E-07	3.59545E-07	1.28133E-07	2.52333E-07	4.4464E-07	3.47733E-07
Deinococcus_puniceus	4.68455E-07	7.40455E-07	5.32182E-08	7.45182E-07	2.85538E-07	4.81923E-07	5.92846E-07	0.000000149	3.35091E-07	1.17909E-07	1.18182E-07	4.26273E-07	0.000000299	1.23067E-07	1.85933E-07	1.97867E-07
Deinococcus_gobiensis	6.99273E-07	0.000001009	5.32182E-08	2.34545E-07	1.36385E-07	1.60615E-07	2.92846E-07	1.62385E-07	3.39818E-07	2.77091E-07	3.45364E-07	3.92636E-07	1.874E-07	2.12333E-07	1.54467E-07	2.95933E-07
Deinococcus_ficus	5.29636E-07	1.42636E-07	1.02818E-07	8.13636E-08	2.82385E-07	2.94154E-07	5.84538E-07	4.50692E-07	2.64636E-07	3.25182E-07	7.20545E-07	8.26636E-07	1.144E-07	3.16667E-07	9.80667E-08	2.95467E-07
Deinococcus_proteolyticus	1.28891E-06	7.55273E-07	3.36455E-07	8.28545E-07	3.60308E-07	1.46762E-06	1.03254E-06	6.71615E-07	2.63636E-07	3.73727E-07	2.81455E-07	9.67273E-07	2.766E-07	1.746E-07	5.48667E-07	0.00000052
Deinococcus_irradiatisoli	5.56091E-07	9.71273E-07	8.20909E-08	3.93545E-07	1.52846E-07	1.07462E-07	7.14154E-07	1.60615E-07	1.43182E-07	1.77091E-07	1.97545E-07	1.34055E-06	1.86933E-07	5.57333E-08	7.95333E-08	1.338E-07
Deinococcus_wulumuqiensis	1.48718E-06	1.00527E-06	2.31727E-07	1.60782E-06	9.08538E-07	3.15769E-06	1.60223E-06	8.12154E-07	7.52727E-07	6.27545E-07	8.23727E-07	1.46845E-06	7.13267E-07	6.84067E-07	7.09733E-07	5.66667E-07
Deinococcus_radiodurans	2.70727E-07	1.47455E-07	4.53455E-08	1.18882E-07	1.35077E-07	5.14846E-07	1.78462E-07	2.50231E-07	1.90545E-07	4.17182E-07	3.18909E-07	8.76364E-07	1.86667E-08	1.35467E-07	1.81067E-07	8.78667E-08
Deinococcus_deserti	0.000000125	0.000000065	0.000000047	1.74273E-07	1.25308E-07	5.55385E-08	2.60923E-07	3.32692E-07	4.84818E-07	1.97545E-07	1.06182E-07	3.67909E-07	1.66933E-07	2.52067E-07	2.22373E-07	9.60667E-08
Deinococcus_maricopensis	1.65455E-06	3.70636E-07	1.79264E-06	9.66636E-07	5.29231E-07	1.18523E-06	1.33085E-06	1.61354E-06	9.03091E-07	9.48455E-07	1.01809E-06	1.18018E-06	3.58467E-07	1.08767E-06	6.07133E-07	6.06733E-07
Deinococcus_geothermalis	1.39527E-06	1.66318E-07	6.18636E-07	1.12527E-06	1.82846E-07	1.07308E-07	1.74769E-06	0.000001167	7.03182E-07	1.14527E-06	9.31455E-07	7.09818E-07	3.682E-07	5.35467E-07	0.000000654	4.41067E-07
Deinococcus_sp._D7000	0.000000351	0.000000065	0.000000025	1.02064E-07	2.21077E-07	0	2.63077E-08	1.71308E-07	1.76727E-07	1.41909E-07	1.53182E-07	6.28182E-07	0	1.09267E-07	1.49333E-08	9.45333E-08
Deinococcus_sp._AJ005	1.59691E-06	9.51364E-07	3.41867E-06	2.68027E-06	4.76077E-07	3.77154E-07	1.50662E-06	1.03754E-06	1.16936E-06	7.51636E-07	2.19773E-06	2.41836E-06	7.402E-07	7.846E-07	8.22467E-07	1.27113E-06
Marinithermus_hydrothermalis	3.03364E-07	1.01091E-07	3.28545E-08	2.05455E-07	2.97538E-07	0.00000029	0.000000528	2.57385E-07	4.85091E-07	6.25455E-08	2.86727E-07	3.93273E-07	1.50667E-07	3.68333E-07	4.35867E-07	3.298E-07
Meiothermus_taiwanensis	0.000000301	5.81636E-07	3.92273E-07	4.36727E-07	5.42308E-08	2.96692E-07	2.74846E-07	7.79231E-08	0.000000027	8.62727E-08	1.05636E-07	2.35182E-07	2.24E-08	8.98667E-08	8.244E-08	2.134E-07
Meiothermus_ruber	8.53727E-07	7.66545E-07	1.78273E-07	4.42727E-07	1.03846E-07	2.32923E-07	2.41538E-07	1.86231E-07	1.63545E-07	4.67091E-07	9.31455E-07	8.95455E-07	1.032E-07	1.63333E-07	2.38333E-07	8.01333E-08
Meiothermus_silvanus	1.69255E-06	1.88855E-06	4.52727E-07	1.53882E-06	2.90923E-07	1.20985E-06	2.88077E-07	2.22538E-07	1.21191E-06	6.32818E-07	7.79727E-07	1.89545E-06	2.158E-07	2.43267E-07	5.98467E-07	0.000000168
Oceanithermus_profundus	3.21091E-07	1.10273E-07	2.12945E-07	0.000000198	2.28846E-07	7.15385E-07	4.06385E-07	2.28615E-07	4.53545E-07	2.57273E-07	0.000000364	2.18364E-07	2.93333E-07	0.000000267	1.15733E-07	2.49267E-07
Thermus_sp._WG	0	0	0	0	0	0	4.33846E-08	0	0	0	0	0	0	0	0	0
Thermus_scotoductus	3.80818E-07	1.14818E-07	2.13182E-07	2.68636E-07	1.23308E-07	1.39846E-07	4.22692E-07	2.02154E-07	2.94909E-07	2.56727E-07	7.29091E-08	6.11182E-07	2.37333E-08	2.33067E-07	1.24973E-07	2.03667E-07
Thermus_parvatiensis	2.70909E-08	0	7.97273E-08	9.11818E-08	0	6.53077E-08	1.28923E-07	1.99769E-07	0	0	0.000000039	7.13636E-09	0	1.61333E-08	4.44E-09	1.51467E-07
Thermus_thermophilus	6.73273E-07	8.83091E-07	4.18855E-07	4.48545E-07	2.01538E-07	2.81769E-07	3.85231E-07	3.68462E-07	4.62727E-07	3.72545E-07	4.75091E-07	7.95727E-07	1.74867E-07	3.08533E-07	5.2664E-07	2.25133E-07
Thermus_aquaticus	3.71818E-07	1.64045E-07	2.07727E-07	2.16364E-07	4.59538E-07	4.22154E-07	6.05538E-07	1.48615E-07	1.11027E-06	4.66545E-07	4.56818E-07	1.10109E-06	2.954E-07	0.000000692	3.95933E-07	2.90267E-07
Thermus_caldilimi	5.46182E-07	0.000000209	1.33455E-07	5.83455E-07	2.20462E-07	2.46669E-06	0.000000793	4.03846E-07	5.38636E-07	5.32545E-07	1.39364E-06	7.07909E-07	1.944E-07	2.948E-07	5.39573E-07	4.65933E-07
Thermus_oshimai	1.01236E-06	7.66364E-08	5.89455E-08	2.96545E-07	5.17077E-07	1.99415E-06	2.62308E-07	9.98692E-07	1.85027E-06	1.11055E-06	6.89909E-07	1.39291E-06	5.58733E-07	9.274E-07	5.65867E-07	4.00533E-07
Thermus_sp._CCB_US3_UF1	8.47364E-07	5.71636E-07	1.60273E-07	6.15364E-07	1.27308E-07	1.16231E-07	4.57231E-07	2.11692E-07	1.19982E-06	5.53545E-07	0.000000717	7.73636E-07	2.52133E-07	5.35733E-07	1.33067E-07	2.58733E-07

Thermus_brockianus	5.61727E-07	5.99773E-07	3.88727E-07	0.000000571	1.69692E-07	3.56923E-07	2.86846E-07	5.62692E-07	5.98182E-07	3.59091E-07	5.69727E-07	1.31182E-06	3.84333E-07	4.59667E-07	7.35133E-07	1.114E-07
Candidatus_Promineofilum_breve	1.28055E-06	7.49636E-07	1.85309E-06	2.35845E-06	9.08538E-07	3.76338E-06	3.83231E-06	3.73454E-06	2.07191E-06	1.59673E-06	1.56555E-06	2.76273E-06	7.90133E-07	4.80133E-07	1.09847E-06	7.892E-07
Ktedonosporobacter_rubrisoli	1.94364E-07	7.53636E-08	4.51727E-08	1.79364E-07	3.58462E-08	1.53769E-07	1.10385E-07	1.90769E-08	5.31182E-07	2.02182E-07	6.68182E-08	3.00727E-07	2.14E-08	2.59667E-07	1.57733E-07	3.454E-07
Thermomicrobium_roseum	9.31818E-08	0	7.40909E-08	6.64545E-08	5.02308E-08	1.91231E-07	5.54615E-08	1.90769E-08	1.31273E-07	8.76364E-08	6.55455E-08	4.28182E-08	1.554E-07	2.26333E-07	5.49067E-08	2.96933E-07
Sphaerobacter_thermophilus	2.17455E-07	2.85091E-07	0	2.47091E-07	1.53538E-07	3.21923E-07	1.47338E-06	2.08077E-06	0.000000106	1.97727E-07	2.40818E-07	2.92091E-07	4.292E-07	7.642E-07	6.91067E-07	4.73067E-07
Roseiflexus_sp._RS-1	1.58182E-07	0.000000071	3.78909E-08	4.38545E-07	8.39231E-08	1.45846E-07	5.06846E-07	1.06154E-07	2.86636E-07	3.13545E-07	2.27455E-07	3.40727E-07	0.000000097	2.722E-07	3.638E-07	4.56933E-07
Roseiflexus_castenholzii	3.54545E-07	7.66364E-08	9.15273E-08	2.17636E-07	2.16231E-07	3.73231E-07	3.21154E-07	3.77538E-07	3.12818E-07	1.65455E-07	3.70636E-07	0.000000724	2.12667E-07	3.07667E-07	3.214E-07	1.57133E-07
Chloroflexus_aurantiacus	1.84273E-07	0	8.28545E-08	8.16364E-08	1.68846E-07	2.67846E-07	0	1.76846E-07	1.74364E-07	2.87273E-08	4.88091E-07	1.79636E-07	8.78E-08	6.39333E-08	7.824E-08	8.46667E-08
Chloroflexus_aggregans	2.42182E-07	4.13455E-07	3.70909E-08	4.95455E-07	2.38154E-07	5.55385E-08	2.25385E-07	1.72385E-07	1.94727E-07	1.99455E-07	2.83182E-07	3.72955E-07	0.000000295	2.246E-07	1.90107E-07	1.25067E-07
Caldilinea_aerophila	8.38818E-06	3.15282E-06	1.03878E-05	8.41091E-06	0.000000482	3.29231E-07	3.03815E-06	5.37077E-06	3.78455E-06	5.70727E-06	5.46655E-06	6.54445E-06	8.638E-07	1.06847E-06	1.07533E-06	0.000001263
Dehalogenimonas_sp._GP	1.52909E-07	8.74727E-07	8.72182E-08	1.16091E-07	1.19462E-07	1.27769E-07	1.38846E-07	2.14615E-07	2.23182E-07	2.78909E-07	2.03909E-07	4.74545E-07	2.44E-08	0	5.54667E-08	5.53333E-08
Dehalogenimonas_lykanthroporepellens	6.08182E-08	1.93182E-07	3.68545E-08	1.96818E-07	3.15385E-08	0.00000104	3.86231E-07	6.89231E-08	2.82545E-07	0.000000028	1.98091E-07	3.23091E-07	4.88E-08	2.38067E-07	2.15867E-07	2.05267E-07
Dehalogenimonas_formicexedens	1.37236E-06	1.21545E-07	3.29118E-06	3.04882E-06	1.27015E-06	1.63762E-06	6.62169E-06	0.000006721	1.61955E-06	8.10182E-07	2.60991E-06	2.23218E-06	1.25587E-06	1.31853E-06	1.27667E-06	2.8256E-06
Dehalococcoides_mccartyi	0.00000619	7.99673E-06	2.57794E-06	1.64158E-05	0.00000484	8.12831E-06	7.46538E-06	9.13615E-06	6.66909E-06	4.62264E-06	9.15364E-06	0.000025785	8.54467E-06	1.02073E-05	1.03807E-05	9.33467E-06
Tepidiforma_bonchosmolovskayae	2.05873E-06	9.28682E-07	3.87864E-07	1.08773E-06	6.31462E-07	2.99692E-07	4.75615E-07	7.33615E-07	2.59982E-06	1.23064E-06	1.95564E-06	2.19709E-06	8.26267E-07	9.702E-07	0.000000591	6.638E-07
Brevefilum_fermentans	1.08909E-06	6.24545E-07	1.654E-07	1.02827E-06	4.63385E-07	1.14423E-06	4.40308E-07	1.33292E-06	5.97455E-07	4.42364E-07	8.00273E-07	4.92727E-07	3.236E-07	3.88867E-07	1.12407E-06	9.41333E-07
Pelolinea_submarina	4.53609E-06	2.93273E-06	1.09559E-06	3.05155E-06	2.60346E-06	6.95638E-06	3.97338E-06	3.95385E-06	3.35727E-06	3.08636E-06	3.20455E-06	0.00000481	3.08167E-06	0.00000344	0.000003274	0.00000321
Anaerolinea_thermophila	0.000001329	4.33909E-07	2.05864E-07	1.71236E-06	1.48915E-06	4.03231E-07	1.30315E-06	1.15808E-06	1.67627E-06	1.72773E-06	0.000002216	0.00000263	1.81193E-06	1.76093E-06	2.05787E-06	3.15433E-06
Anaerolinea_sp._'rifampicinis'	0.00000344	0.000000437	6.71818E-07	2.59027E-06	2.57638E-06	4.03077E-08	2.77885E-06	4.03308E-06	2.23273E-06	0.000003026	5.01091E-06	4.11036E-06	2.50433E-06	3.24447E-06	3.05533E-06	2.62327E-06
cyanobacterium_endosymbiont_of_Rhopalodia_gibberul	0	0	1.63636E-08	1.03636E-08	0	0	0	0.000000011	2.64545E-08	0	0	0	2.08E-08	3.50667E-08	0	3.67333E-08
cyanobacterium_endosymbiont_of_Epithemia_turgida	2.51182E-07	0	0	1.04727E-07	7.48462E-08	0	4.33846E-08	6.03846E-08	1.55091E-07	1.24818E-07	5.95455E-08	3.10955E-07	2.37333E-08	8.38667E-08	2.57667E-07	4.76667E-08
Chroococcidiopsis_thermalis	3.20727E-07	5.88182E-08	3.40909E-08	1.28909E-07	9.96923E-08	0	1.82231E-07	2.46923E-08	1.78364E-07	1.45364E-07	3.59182E-07	7.78636E-07	2.032E-07	1.024E-07	1.74667E-07	0.00000018
Gloeobacter_violaceus	5.73455E-07	1.89364E-06	8.72182E-08	0.000000071	1.22077E-07	2.62723E-06	2.66846E-07	9.03077E-08	2.11909E-07	2.33636E-07	2.80909E-07	3.31273E-07	2.028E-07	1.318E-07	1.9104E-07	3.97333E-08
Gloeobacter_kilaueensis	3.14818E-07	0.00000075	1.73091E-07	0.000000686	1.92769E-07	9.06385E-07	4.64154E-07	2.72462E-07	0.000000435	4.85091E-07	4.87545E-07	1.48755E-06	2.01867E-07	2.19667E-07	1.5904E-07	1.75467E-07
Stanieria_cyanosphaera	2.37364E-07	1.04727E-07	1.11164E-07	6.24545E-08	1.74692E-07	2.13846E-08	4.33846E-08	4.12154E-07	1.44091E-07	2.85727E-07	3.24818E-07	3.07091E-07	4.38E-08	1.552E-07	1.86333E-07	1.87333E-07
Stanieria_sp._NIES-3757	0	2.34545E-08	0	6.12727E-08	0	0	0	8.89231E-08	4.45455E-08	0	6.71818E-08	4.59091E-08	0	0.000000061	3.43733E-08	0
Pleurocapsa_minor	5.81091E-07	1.53455E-07	1.63636E-08	4.64545E-07	1.29308E-06	6.17538E-07	7.07692E-08	2.38077E-07	7.87636E-07	7.54909E-07	0.000000823	2.48836E-06	3.756E-07	1.12553E-06	1.1712E-06	8.81133E-07
Gloeomargarita_lithophora	3.74545E-06	3.06545E-07	1.46727E-06	3.64482E-06	4.11554E-06	7.53769E-07	1.65008E-06	3.67431E-06	5.56891E-06	3.21655E-06	5.02636E-06	4.73273E-06	3.80013E-06	3.60113E-06	0.000005804	2.54587E-06
Crinalium_epipsammum	5.54545E-08	2.34545E-08	1.85455E-07	0.000000064	0.000000099	1.41308E-07	6.41538E-08	1.45769E-07	4.45455E-08	0.000000109	9.60909E-08	2.14545E-08	0	1.21133E-07	2.18467E-07	7.68667E-08
Cyanothece_sp._PCC_7425	0.000000519	2.03909E-07	1.63909E-07	7.08182E-07	0.000000152	4.90769E-07	1.14923E-07	1.87692E-07	2.63455E-07	1.89909E-07	7.90909E-08	2.63636E-07	1.01467E-07	1.71733E-07	1.672E-07	2.64867E-07
Geitlerinema_sp._PCC_7407	4.57273E-07	0.000000147	2.09636E-07	5.52818E-07	3.01923E-07	5.18769E-07	7.28923E-07	5.67385E-07	0.000000214	4.09455E-07	5.09545E-07	8.87636E-07	1.73133E-07	2.554E-07	2.23467E-07	5.012E-07
Oscillatoria_acuminata	3.00427E-06	1.29091E-07	2.13545E-07	1.25636E-07	3.65231E-07	5.98077E-07	1.27623E-06	1.90192E-06	1.98964E-06	4.89418E-06	4.60745E-06	1.39618E-06	5.76133E-07	5.27733E-07	5.67773E-07	1.34987E-06
Oscillatoria_nigro-viridis	9.78182E-07	4.04136E-07	1.88636E-07	4.99909E-07	1.76692E-07	3.36692E-07	6.05769E-07	6.05692E-07	6.60545E-07	1.42473E-06	1.47009E-06	7.53455E-07	3.62733E-07	4.03867E-07	6.94533E-07	9.704E-07
Moorea_producens	1.45818E-06	8.99091E-07	3.45727E-07	1.50764E-06	2.02762E-06	8.49231E-08	1.82077E-07	1.08769E-06	0.000001906	7.86727E-07	1.27382E-06	1.86627E-06	1.89533E-06	1.9968E-06	2.44053E-06	1.67573E-06
Limnospira_fusiformis	0	0	0	0	0	0	0	0	4.45455E-08	0	0	0	0	0	0	0
Arthrospira_platensis	0	0	0	4.79091E-09	0	0	0	0	0	5.22727E-08	7.90909E-08	1.38273E-07	2.94667E-08	0	0	0
Microcoleus_sp._PCC_7113	0	0	0	0	2.05385E-08	0	0	1.90769E-08	4.89091E-08	0	3.95455E-08	2.14545E-08	0	0	6.46667E-08	4.95333E-08
Planktothrix_agardhii	1.55545E-07	0.000000065	1.64127E-07	2.66155E-07	0.000000116	3.93077E-08	2.26077E-07	2.14692E-07	2.72727E-08	1.72727E-07	9.89091E-08	0.000000101	7.10667E-08	1.398E-07	9.62667E-08	1.50933E-07
Oxynema_sp._AP17	3.84727E-07	1.36182E-07	9.88636E-07	1.77709E-06	4.21692E-07	0.000000106	0	2.93615E-07	7.69545E-07	5.96091E-07	5.83182E-07	0.000000384	2.564E-07	2.00467E-07	6.67733E-07	4.528E-07
Trichodesmium_erythraeum	5.40636E-07	4.47545E-07	3.25036E-07	9.82545E-07	3.23923E-07	5.01385E-07	2.95615E-07	7.08231E-07	1.29045E-06	8.86455E-07	7.85636E-07	8.91364E-07	5.18267E-07	4.45267E-07	6.26067E-07	8.90267E-07
Microcystis_aeruginosa	3.34273E-07	0	1.41891E-07	5.80909E-08	0	0	0	1.13846E-07	1.23182E-07	0	3.96364E-07	2.85455E-08	4.72667E-08	4.80667E-08	1.57067E-08	0
Halothece_sp._PCC_7418	0	0	0	0	1.38231E-07	0	0	1.53538E-07	0	0	0	0	9.63333E-08	1.55933E-07	2.852E-07	3.05267E-07
Crocosphaera_subtropica	3.28455E-07	8.58182E-08	0	2.45455E-08	4.74692E-07	0	1.94923E-07	1.37077E-07	5.50909E-08	0.000000047	0.000000152	4.10045E-07	1.016E-07	2.314E-07	7.00667E-08	2.266E-07
Euhalothece_natronophila	0.000000239	0	1.03727E-07	1.60727E-07	0	0	7.40769E-08	1.55077E-07	1.72364E-07	1.72818E-07	1.27636E-07	2.44818E-07	0	5.89333E-08	1.9484E-07	5.39333E-08
Candidatus_Atelocyanobacterium_thalassa	8.94545E-08	7.38636E-08	8.25455E-09	1.03455E-07	2.75385E-08	0	8.11538E-08	1.15615E-07	6.62727E-08	0	0	2.14545E-08	8.17333E-08	1.70133E-07	4.82E-08	3.97333E-08
Rippkaea_orientalis	1.40545E-07	1.94545E-07	0	4.79091E-09	2.55769E-07	2.13846E-08	0	7.69231E-08	2.64545E-08	2.85455E-08	6.68182E-08	1.11273E-07	1.092E-07	1.166E-07	0.000000166	1.33933E-07
Gloeothece_citriformis	5.77636E-07	1.04545E-08	2.01909E-07	1.16545E-07	1.72538E-07	0	1.33846E-07	2.88308E-07	4.29455E-07	2.91727E-07	3.73091E-07	4.45455E-07	2.426E-07	2.19867E-07	3.558E-07	1.70267E-07
Gloeothece_verrucosa	3.78182E-07	0.00000013	9.47273E-08	0.000000163	3.61538E-07	5.16769E-07	1.36308E-07	1.36692E-07	4.84455E-07	4.26364E-07	2.02273E-07	1.92636E-07	1.73333E-07	5.94267E-07	4.414E-07	4.58667E-07
Cyanobacterium_aponinum	3.55273E-06	1.80727E-07	1.11782E-06	1.42982E-06	2.22746E-06	1.34692E-07	7.51615E-07	1.74785E-06	0.000001989	0.000002021	2.90045E-06	1.79009E-06	1.57707E-06	0.000002437	2.99333E-06	9.79133E-07
Gloeocapsa_sp._PCC_7428	9.05909E-07	1.98091E-07	2.09545E-07	1.00873E-06	0.000000077	0	2.78769E-07	5.54769E-07	4.16455E-07	3.48909E-07	1.11318E-06	0.000001207	2.846E-07	5.252E-07	2.68533E-07	4.11667E-07
Chondrocystis_sp._NIES-4102	5.84727E-07	8.18182E-08	3.14818E-07	5.33364E-07	0.000000275	3.76154E-07	1.59615E-07	0.000000666	5.22818E-07	4.56636E-07	5.94818E-07	1.29018E-06	4.72E-08	2.968E-07	2.42933E-07	2.55267E-07
Geminocystis_herdmanii	5.54545E-08	0	1.66364E-07	1.31855E-06	2.58846E-07	4.65385E-07	7.53385E-07	1.54208E-06	5.45455E-08	1.35545E-07	7.27636E-07	7.12273E-07	2.93733E-07	3.67333E-07	3.658E-07	5.332E-07
Geminocystis_sp._NIES-3708	1.81636E-07	0	0	5.16364E-08	1.83385E-07	7.66154E-08	3.77692E-08	1.02462E-07	7.50909E-08	0.000000034	4.98182E-08	7.85455E-08	0.000000143	2.87133E-07	4.48667E-08	1.19933E-07
Geminocystis_sp._NIES-3709	1.33664E-06	0	4.81818E-07	2.59782E-06	2.75969E-06	1.33692E-06	3.29308E-06	2.94731E-06	6.37727E-07	8.31818E-07	1.55655E-06	1.12436E-06	3.09133E-06	2.72533E-06	2.74553E-06	4.16453E-06
Cylindrospermopsis_curvispora	0	1.16364E-08	0	0	0	0	0	0	0	0	0	0	0	0	0	0
Sphaerospermopsis_kisseleviana	3.11818E-08	8.84545E-08	0	4.79091E-09	0	0	0	0	0	3.03636E-08	0	4.79091E-08	2.24E-08	1.58E-08	0	1.86E-08
Anabaenopsis_circularis	0	0	3.70909E-08	3.85455E-08	1.78231E-07	0	1.47538E-07	4.39385E-07	1.23727E-07	2.87273E-08	3.59636E-07	7.90455E-07	2.09333E-08	1.00933E-07	3.2784E-07	3.84667E-08
Dolichospermum_flos-aquae	0	0	0	9.54545E-09	0	0	0	0	2.10636E-07	0	1.24091E-07	2.14545E-08	6.41333E-08	0	2.99333E-08	0
Dolichospermum_compactum	3.11818E-08	0	1.77273E-08	0	0	0	0	0	0	0	0	7.13636E-09	0	0	1.12667E-08	0
Nodularia_spumigena	8.54455E-07	2.63636E-08	8.34273E-07	1.20482E-06	4.03769E-07	3.11231E-07	2.19877E-06	3.26923E-06	3.66636E-07	4.89273E-07	3.81545E-07	0.000000979	3.56733E-07	1.79667E-07	6.222E-07	2.93867E-07
Fischerella_sp._NIES-3754	2.98182E-08	0	0	0	0	0	0	0	0	3.03636E-08	0	0	6.08E-08	6.52667E-08	1.49333E-08	5.96667E-08
Fischerella_sp._NIES-4106	0	0	0	0	9.05615E-07	0	0	0.000000176	1.18182E-07	2.85455E-08	0	2.14545E-08	0.000000285	8.37267E-07	4.70667E-07	6.60467E-07
Tolypothrix_sp._PCC_7910	5.52727E-08	0	0	6.90909E-08	9.64615E-08	0	0	0	1.13727E-07	0	1.91182E-07	7.13636E-09	1.78867E-07	3.48E-08	4.44E-09	7.97333E-08
Microchaete_diplosiphon	3.11818E-08	6.07273E-08	0.000000146	4.79091E-09	2.05385E-08	0	2.37308E-07	0.000000119	0	0	2.45909E-07	8.29091E-08	6.08E-08	3.50667E-08	1.57067E-08	4.76667E-08
Rivularia_sp._PCC_7116	6.82727E-07	4.95091E-07	6.98182E-08	3.29364E-07	2.26846E-07	0.000000472	2.04538E-07	4.86462E-07	5.03727E-07	1.53545E-07	4.29727E-07	3.07364E-07	3.30133E-07	2.286E-07	4.988E-07	4.07867E-07
Scytonema_sp._NIES-4073	5.42727E-08	1.16364E-08	9.01818E-08	1.43727E-07	0	0	2.63077E-08	0	2.16364E-08	2.87273E-08	6.65455E-08	0	0	4.67333E-08	2.38E-08	4.62667E-08
Scytonema_sp._HK-05	0.000000452	6.44818E-07	1.70618E-07	6.71455E-07	0.00000038	2.05846E-07	1.06115E-06	6.35923E-07	3.53636E-07	1.71273E-07	2.57273E-07	6.77091E-07	1.67467E-07	3.142E-07	1.234E-07	9.23333E-08
Cylindrospermum_stagnale	1.88636E-07	2.19091E-07	2.15773E-07	1.11091E-07	1.78077E-07	6.76923E-08	1.41538E-07	4.35846E-07	2.23545E-07	2.69818E-07	0.000000405	7.36636E-07	2.304E-07	2.26267E-07	8.18667E-08	2.918E-07
Anabaena_sp._WA102	3.62727E-08	0.000000047	3.70909E-08	2.70427E-07	3.15385E-08	0	0	0	0	0	7.72727E-08	1.45409E-07	2.64E-08	0	7.43333E-08	4.97333E-08
Anabaena_sp._90	7.37273E-08	0	3.70909E-08	1.93636E-08	1.35077E-07	2.52308E-08	0.000000123	3.88923E-07	1.29727E-07	0.0000001	6.82091E-07	2.86545E-07	4.88667E-08	7.60667E-08	3.70867E-07	2.03267E-07
Anabaena_cylindrica	3.88545E-07	0.00000024	1.10909E-07	2.32909E-07	7.02308E-08	2.95385E-08	5.25385E-07	1.29692E-07	3.75455E-08	1.78364E-07	9.64545E-08	3.18091E-07	2.24E-08	6.74667E-08	1.50307E-07	1.86E-08
Trichormus_azollae	0	3.99091E-08	2.95182E-08	4.85182E-08	1.76077E-07	0	6.44615E-08	9.80769E-08	5.79091E-08	0.000000047	3.95455E-08	8.36364E-08	1.238E-07	1.18467E-07	1.04267E-07	2.072E-07
Nostoc_sp._'Peltigera_membranacea_cyanobiont'_N6	0	0	0	4.79091E-09	7.02308E-08	0	0	0	0	0	0	0	0	0	0	0
Nostoc_flagelliforme	3.62727E-08	4.24091E-08	0	9.54545E-09	6.69231E-08	9.45385E-08	0	3.93846E-08	0	0	0	0	4.46667E-08	1.15533E-07	0	0
Nostoc_sp._NIES-2111	0	0	0	4.79091E-09	0	0	0	0	2.75455E-08	0	0	7.13636E-09	0	0	1.65333E-08	0
Nostoc_sp._TCL240-02	0	0	0	0	0	0	0	0	2.75455E-08	0	0	3.94545E-08	0	4.98E-08	4.44E-09	0
Nostoc_sp._ATCC_53789	0	0.000000065	0	2.43636E-08	0	0	6.23077E-08	0	0	0.000000047	0	0	6.08E-08	0	0	1.22E-08
Nostoc_sp._'Lobaria_pulmonaria_(5183)_cyanobiont'	0	0	0	4.30909E-08	9.25385E-08	0	3.06923E-08	4.37692E-08	0.000000054	0.000000034	0	6.41364E-08	6.64667E-08	0.000000014	5.91333E-08	2.06467E-07
Nostoc_sphaeroides	0	0	0	4.79091E-09	0	0	0	0	0	0	2.61818E-07	0	0	0	4.48667E-08	5.10667E-08
Nostoc_edaphicum	1.91182E-07	0.000000065	3.70909E-08	2.59091E-08	3.95615E-07	0.000000231	0	0.000000096	9.77273E-08	8.47273E-08	4.05091E-07	8.01273E-07	0	0	0	0
Nostoc_sp._C052	6.25455E-08	0	1.97091E-07	1.09455E-07	1.29385E-07	0	5.48462E-08	0	8.26364E-08	1.67455E-07	3.95455E-08	6.95727E-07	1.50467E-07	9.62E-08	1.268E-07	0
Nostoc_carneum	4.29091E-08	8.86364E-09	0	1.25182E-07	1.70538E-07	0	0	2.51538E-07	0	0.000000075	3.33636E-08	0.000000035	8.32E-08	6.39333E-08	9.204E-08	1.074E-07
Nostoc_sp._TCL26-01	2.80909E-07	5.36364E-08	1.15182E-07	2.61364E-07	3.17769E-07	3.58462E-08	1.22615E-07	4.13846E-07	2.54091E-07	3.50182E-07	1.21909E-07	4.30818E-07	1.796E-07	2.46867E-07	5.48773E-07	0.000000296
Nostoc_sp._NIES-3756	9.89636E-07	0	1.48182E-07	1.01136E-06	1.21385E-07	7.21538E-08	4.86308E-07	2.15462E-07	5.06182E-07	4.76636E-07	5.05636E-07	4.42273E-07	1.37933E-07	1.39867E-07	2.08533E-07	6.604E-07
Nostoc_linckia	1.40727E-07	1.77273E-08	0	1.29091E-07	1.85846E-07	0	6.23077E-08	3.37692E-07	0.000000027	4.05818E-07	4.15273E-07	0.0000005	5.64667E-08	1.572E-07	1.946E-07	2.374E-07
Nostoc_sp._NIES-4103	0.000000847	1.16364E-08	2.66273E-07	1.34182E-07	1.23462E-07	5.01077E-07	3.17462E-07	3.17846E-07	2.79727E-07	2.14636E-07	6.46364E-07	4.55364E-07	1.254E-07	9.40667E-08	0.000000127	8.62667E-08
Nostoc_piscinale	1.58182E-08	0	1.77273E-08	4.79091E-09	0	0	0	0	0.000000106	0	0.000000039	0	0	0	0	9.33333E-09
Nostoc_punctiforme	3.69455E-07	7.20455E-07	2.77909E-07	7.60182E-07	0.000000126	0	6.06462E-07	3.46462E-07	2.41636E-07	4.74909E-07	6.28091E-07	1.71827E-06	2.32867E-07	0.00000034	4.43733E-07	3.04133E-07
Nostoc_sp._PCC_7524	4.14545E-08	1.37273E-08	2.64818E-07	6.96545E-07	0.000000104	1.76769E-07	6.41538E-08	7.42308E-08	2.03455E-07	0	1.70455E-07	1.33182E-07	0	4.49333E-08	0	5.10667E-08
Nostoc_sp._CENA543	5.41727E-07	0.000000065	1.65364E-06	1.58327E-06	4.95385E-07	0	4.47923E-06	4.95762E-06	9.61182E-07	6.04273E-07	4.32636E-07	4.70909E-07	3.87733E-07	3.56133E-07	5.65533E-07	3.98467E-07
Nostoc_sp._PCC_7107	3.62727E-08	0	0	1.63609E-07	7.03846E-08	3.83077E-08	4.33846E-08	9.03846E-08	0	0	0	7.13636E-09	2.24E-08	1.37667E-07	2.38E-08	8.19333E-08
Calothrix_sp._NIES-2100	3.69091E-08	0	0	0	5.76154E-08	0	0	0	0	0	0	2.14545E-08	0.000000027	6.07333E-08	2.97333E-08	9.33333E-09
Calothrix_sp._NIES-3974	3.11818E-08	6.84545E-08	0	3.59091E-08	1.10692E-07	4.33846E-08	0	5.22308E-08	3.29091E-07	1.43455E-07	3.95455E-08	3.30091E-07	4.78667E-08	2.62E-08	1.08707E-07	0
Calothrix_parasitica	5.10636E-07	1.57909E-07	9.14545E-08	2.14727E-07	2.62846E-07	1.02462E-07	1.70385E-07	3.37692E-08	4.89818E-07	1.98364E-07	3.38727E-07	5.25182E-07	1.43333E-07	1.32067E-07	2.282E-07	1.262E-07
Calothrix_parietina	3.42455E-07	4.50727E-07	1.314E-07	3.24545E-07	3.34615E-08	1.10154E-07	7.92308E-08	2.46923E-08	0.000000054	1.29636E-07	0	0.000000085	0	6.78E-08	3.43733E-08	0
Calothrix_brevissima	1.86091E-07	0	4.58455E-08	2.59091E-08	0	2.95385E-08	1.48538E-07	9.58462E-08	7.59091E-08	7.77273E-08	2.39091E-07	1.72455E-07	5.44E-08	1.03533E-07	2.41667E-07	1.81267E-07
Calothrix_sp._336/3	2.39091E-07	0	3.70909E-08	9.54545E-09	1.77462E-07	3.96154E-08	3.20769E-07	1.60769E-07	3.31636E-07	3.23455E-07	4.75091E-07	2.87727E-07	8.91333E-08	1.02333E-07	3.692E-07	2.804E-07
Calothrix_sp._PCC_7507	2.94636E-07	2.70909E-08	0.000000035	6.50455E-07	8.07462E-07	2.56154E-07	3.01077E-07	9.82615E-07	0	9.36364E-08	3.96909E-07	4.21182E-07	1.13773E-06	1.06507E-06	9.27133E-07	5.05333E-07
Calothrix_sp._NIES-2098	0.000000747	2.38227E-07	1.26182E-07	4.49727E-07	2.20385E-07	4.13846E-08	7.96538E-07	1.31538E-07	4.63727E-07	3.16455E-07	4.52091E-07	3.83364E-07	1.55267E-07	6.86867E-07	3.85867E-07	9.41333E-08
Halomicronema_hongdechloris	1.90636E-07	0.000000044	3.70909E-08	2.20091E-07	2.88462E-08	2.25062E-06	6.07385E-07	6.13846E-07	0.00000014	1.01273E-07	2.35636E-07	8.47273E-08	1.158E-07	1.376E-07	8.084E-08	7.39333E-08
Thermoleptolyngbya_sp._PKUAC-SCTA183	5.72909E-07	7.13636E-08	4.52364E-07	0.000000227	4.25615E-07	4.09077E-07	5.47692E-07	3.98692E-07	4.08364E-07	7.61818E-07	1.64727E-07	1.51755E-06	2.72867E-07	3.37133E-07	2.68933E-07	1.40867E-07
Pseudanabaena_sp._PCC_7367	1.15964E-06	0.000001178	6.01273E-07	6.10091E-07	8.16923E-07	4.21538E-07	1.17815E-06	9.68769E-07	4.92455E-07	4.14909E-07	8.89364E-07	1.94764E-06	4.86E-08	1.81067E-07	1.05653E-06	1.12807E-06
Pseudanabaena_sp._ABRG5-3	2.95609E-06	8.98636E-07	5.89182E-07	8.96727E-07	0.000000598	2.61538E-07	1.62308E-06	0.000001992	4.40655E-06	6.95455E-06	4.67255E-06	3.16982E-06	6.47667E-07	3.99533E-07	4.88067E-07	1.41807E-06
Synechocystis_sp._PCC_6803	0	0	0	0	0	0	0	0	0	0	0	0	2.08E-08	0	0	0
Synechocystis_sp._CACIAM_05	3.69091E-08	0	0	2.91545E-08	0	4.39231E-08	0	7.77692E-08	5.79091E-08	0	3.95455E-08	1.61455E-07	2.24E-08	0	0	9.33333E-09
Synechocystis_sp._PCC_6714	5.52636E-07	1.47764E-06	2.58091E-07	5.44909E-07	2.75385E-08	0.000000026	0.000000093	8.96923E-08	2.53182E-07	3.32364E-07	4.11091E-07	5.54545E-07	7.52667E-08	1.35067E-07	2.81133E-07	3.67333E-08
Leptolyngbya_boryana	1.45727E-07	1.01182E-07	0.000000047	1.03636E-08	0	2.52308E-08	0	0	5.97273E-08	0	3.77273E-08	1.34818E-07	2.44E-08	4.09333E-08	2.09733E-08	9.25333E-08
Leptolyngbya_sp._O-77	5.44727E-07	9.02727E-07	1.48673E-07	2.35091E-07	0.000000061	7.21538E-08	3.81615E-07	3.48462E-07	1.08545E-07	6.37273E-08	8.13636E-08	3.10545E-07	0.000000088	0	1.41933E-07	1.98667E-08
Leptolyngbya_sp._PCC_7376	2.77182E-07	6.97273E-08	0.000000485	1.25545E-07	2.94769E-07	9.93846E-08	7.92308E-08	9.50769E-08	5.65091E-07	0.000000277	3.45818E-07	0.000000257	2.52867E-07	4.39933E-07	4.07467E-07	2.938E-07
Leptolyngbya_sp._NIES-3755	0	0	0	0	2.52615E-07	3.81538E-07	6.44615E-08	4.67692E-08	8.20909E-08	0	1.02727E-08	2.61636E-07	2.388E-07	0.000000248	2.282E-07	2.048E-07
Chamaesiphon_minutus	4.68727E-07	0.000000335	1.08727E-07	4.03636E-07	3.15769E-07	0	2.91077E-07	2.46385E-07	0.00000022	2.62364E-07	5.69273E-07	7.86091E-07	2.19133E-07	5.15267E-07	3.64667E-07	0.00000021
Acaryochloris_marina	2.37036E-06	6.86182E-07	1.20327E-06	1.14655E-06	1.98238E-06	0.000000354	1.17823E-06	1.04338E-06	1.21436E-06	1.90755E-06	0.000001863	2.52818E-06	1.38107E-06	1.73173E-06	2.43953E-06	1.58913E-06
Prochlorococcus_sp._MIT_0604	2.96364E-08	0	0	0	0	0	0	0	0	0	0	0	0	0	0	4.95333E-08
Prochlorococcus_sp._MIT_0801	0	0	0	0	0	0	0	1.30385E-07	0	0	0	0	0	0	5.94E-08	0
Prochlorococcus_marinus	1.83755E-06	8.63727E-07	6.90182E-07	6.99091E-07	1.21469E-06	4.71154E-07	1.23231E-06	0.000001327	4.93364E-07	7.63636E-07	2.29118E-06	2.31727E-06	0.000000862	1.38053E-06	0.000002137	1.8106E-06
Thermosynechococcus_sp._NK55a	5.68182E-08	6.07273E-08	0	8.27909E-08	0	0	0	0	1.15455E-07	0	1.02727E-08	7.13636E-09	4.48667E-08	0	1.49333E-08	0
Thermosynechococcus_elongatus	4.62455E-07	9.54545E-09	3.70909E-08	1.17182E-07	8.79231E-08	0.00000029	4.24231E-07	2.39615E-07	1.13727E-07	0	0	7.85455E-08	5.18667E-08	1.71333E-08	1.02133E-07	5.26667E-08
Thermosynechococcus_sp._CL-1	2.61273E-07	1.27182E-07	0	6.94545E-08	1.29154E-07	1.85154E-07	1.26231E-07	1.59846E-07	1.07091E-07	3.22818E-07	2.84182E-07	0.000000585	6.41333E-08	3.67933E-07	9.46667E-08	2.64267E-07
Cyanobium_sp._NIES-981	1.27445E-06	1.55391E-06	6.28182E-08	0.000001217	3.58462E-08	1.25077E-07	1.55538E-07	3.56923E-08	4.85545E-07	3.00818E-07	9.99364E-07	5.95091E-07	1.516E-07	1.65733E-07	3.384E-07	2.10467E-07
Cyanobium_sp._NS01	3.52909E-07	2.06182E-07	9.05455E-08	6.56636E-07	2.10077E-07	6.53077E-08	4.63154E-07	2.31231E-07	2.11909E-07	1.80273E-07	0.000000134	4.64545E-07	6.67333E-08	1.06467E-07	3.51733E-07	1.31467E-07
Cyanobium_gracile	1.76873E-06	7.77182E-07	3.31455E-07	1.02064E-06	7.94231E-07	2.01692E-07	3.81692E-07	7.70769E-07	2.93391E-06	7.80455E-07	1.74673E-06	1.51445E-06	2.24867E-07	5.65533E-07	7.03267E-07	5.46533E-07
Dactylococcopsis_salina	5.17273E-06	4.02609E-06	7.54182E-07	4.56691E-06	4.24815E-06	3.53708E-06	7.18577E-06	3.69846E-06	0.000007014	6.87155E-06	2.89273E-06	4.27636E-06	2.43747E-06	3.53193E-06	2.45487E-06	1.35947E-06
Synechococcus_sp._CC9605	0	0	0	0	0	0	0	2.46923E-08	0	0	0	0	0	0	0	0
Synechococcus_sp._PCC_7117	0	0	0	0	0	0	0	0	8.20909E-08	0	0	0	0	0	0	0
Synechococcus_sp._WH_8020	2.96364E-08	0	0	0	0	1.01692E-07	0	2.46923E-08	0	0	0	4.79091E-08	0	0	1.49333E-08	0
Synechococcus_sp._KORDI-49	2.96364E-08	0	0	0	0	0	0	0	0	0	0	7.13636E-09	0	1.58E-08	2.99333E-08	0
Synechococcus_sp._PROS-7-1	1.00818E-07	0	0	5.18182E-09	0	1.31308E-07	4.33846E-08	1.46923E-08	0	0	0	5.64545E-08	1.14267E-07	0	0	0
Synechococcus_sp._NOUM97013	3.62727E-08	0.000000065	0	4.79091E-09	4.93846E-08	4.27692E-08	4.33846E-08	0	0.000000084	0.000000056	0	7.13636E-09	1.104E-07	0	2.24667E-08	9.33333E-09
Synechococcus_sp._PCC_8807	0	0	0	2.43636E-08	0	0	0	0	0	2.85455E-08	0	7.55455E-08	0	0	3.31333E-08	1.66667E-08
Synechococcus_sp._A15-60	3.62727E-08	1.16364E-08	0	1.93636E-08	0	0	2.73846E-08	0	0	0	0	0	0	3.50667E-08	0	0
Synechococcus_sp._A15-127	0	0	0	0	0	0	0	0	3.98182E-08	0	6.88182E-08	0	0	0.000000014	0	0
Synechococcus_sp._A15-44	0.000000157	0	0	1.38973E-07	1.15769E-07	0	0	0	0	0	2.04091E-07	4.65909E-08	0	8.35333E-08	0.000000057	3.20667E-08
Synechococcus_sp._WH_8101	3.11818E-08	0	0	2.41545E-08	0.000000065	0	0	0	3.75455E-08	7.77273E-08	3.95455E-08	3.94545E-08	5.01333E-08	0	1.14667E-07	1.13733E-07
Synechococcus_sp._PCC_11901	3.11818E-08	0	3.70909E-08	0	0.000000039	0	0	1.06923E-07	4.45455E-08	0	7.90909E-08	2.14545E-08	0	2.62E-08	0	0
Synechococcus_sp._KORDI-100	9.45455E-08	0	3.70909E-08	8.61818E-08	1.34077E-07	0	2.63077E-08	8.28462E-08	0	1.27273E-07	0	6.21818E-08	1.04667E-07	1.71333E-08	1.93733E-08	4.04E-08
Synechococcus_sp._MEDNS5	8.94545E-08	0	7.40909E-08	2.50909E-08	0.00000013	9.46154E-09	5.26154E-08	5.69231E-08	1.56636E-07	0.000000056	0	2.74545E-07	0	3.50667E-08	4.44E-09	1.86E-08
Synechococcus_sp._RCC307	0.000000107	1.33864E-07	1.63636E-08	3.37273E-08	0	0	0	0	0	0	1.68636E-07	7.55455E-08	0	0	0	0
Synechococcus_sp._WH_7803	3.69091E-08	2.34545E-08	5.34545E-08	1.05455E-07	0	0	2.04154E-07	7.72308E-08	7.50909E-08	7.55455E-08	2.76364E-08	7.12727E-08	1.06533E-07	1.58E-08	0	8.06667E-08

Synechococcus_sp._RS9909	0	2.57636E-07	8.19091E-08	5.18182E-09	3.58462E-08	0	3.11538E-08	1.07769E-07	1.55273E-07	0	1.37273E-07	0	0	4.49333E-08	2.55333E-08	1.22E-08
Synechococcus_elongatus	2.70909E-08	3.35455E-08	9.51818E-08	1.91818E-08	0	0	0	0	0	0	0	0	0	0	0	0
Synechococcus_sp._PCC_73109	1.15182E-07	0	2.96364E-08	1.63882E-07	1.71154E-07	0	5.54615E-08	5.41538E-07	2.60182E-07	3.03636E-08	4.98455E-07	2.85455E-08	4.27333E-08	2.07667E-07	1.07533E-07	1.09067E-07
Synechococcus_sp._A18-46.1	0	0	2.96364E-08	0	0	0	0	0	0	0	0	4.07364E-07	0	0	0	0
Synechococcus_sp._RS9907	1.58182E-08	0	0	0	0	0	0	0	0	0	0	0	0	0	0	0
Synechococcus_sp._SYN20	0	0	1.29182E-07	2.72455E-07	0	0	0	3.57692E-08	0.000000027	5.73636E-08	3.95455E-08	3.16364E-08	0	0	0	1.18667E-07
Synechococcus_sp._WH_8109	1.84545E-07	0	0	5.18182E-09	1.22077E-07	5.64231E-07	1.84385E-07	1.29846E-07	2.25818E-07	6.29727E-07	5.54818E-07	3.14364E-07	1.538E-07	0.000000373	4.21373E-07	2.414E-07
Synechococcus_sp._HK01-R	1.38545E-07	1.53455E-07	0	8.34545E-08	4.62308E-08	0	5.54615E-08	2.82077E-07	1.53727E-07	2.72818E-07	2.58455E-07	1.90727E-07	4.61333E-08	7.60667E-08	1.674E-07	3.40667E-08
Synechococcus_sp._BIOS-E4-1	2.26182E-07	4.29636E-07	1.40545E-07	1.12273E-07	1.24846E-07	0	2.50846E-07	1.83154E-07	2.97818E-07	1.38545E-07	1.20455E-07	4.41909E-07	1.86667E-08	3.42E-08	2.38E-08	2.92E-08
Synechococcus_sp._PCC_7502	6.35455E-07	0	0.000000156	3.54455E-07	2.45462E-07	0	2.62538E-07	3.22231E-07	4.09182E-07	3.62727E-08	5.33091E-07	6.36727E-07	8.70667E-08	2.144E-07	4.25667E-07	1.67667E-07
Synechococcus_sp._PROS-9-1	1.89818E-07	0	2.10545E-07	1.43636E-07	0.0000001	0	1.26231E-07	1.93846E-07	0.000000081	0	3.68182E-07	8.54091E-07	6.90667E-08	4.91333E-08	4.83067E-08	2.79333E-08
Synechococcus_sp._BMK-MC-1	9.92727E-08	9.20909E-08	0	9.54545E-09	6.30769E-08	3.54308E-07	0	5.72308E-08	2.16364E-08	0.000000028	6.66364E-08	1.42727E-08	0	0.000000033	0	2.79333E-08
Synechococcus_sp._A15-24	7.64727E-07	0.00000013	0	4.14545E-08	0	2.13846E-08	0	7.72308E-08	2.13455E-07	2.10727E-07	4.36636E-07	1.43455E-07	1.782E-07	0	1.57733E-07	1.226E-07
Synechococcus_sp._CC9902	0.00000045	0	2.94091E-07	5.70455E-07	1.47077E-07	0	4.31846E-07	4.33154E-07	6.82455E-07	4.21091E-07	0.000000491	4.30727E-07	3.83867E-07	2.75933E-07	1.24533E-07	2.51267E-07
Synechococcus_sp._PCC_7002	0	0	8.75455E-09	0	0	0	4.80769E-08	0	0	0	3.77273E-08	0	0	0	0	0
Synechococcus_sp._A15-62	0	0	0.00000035	5.50909E-08	0	0	0	1.90769E-08	0.000000027	0	0	0	0	0	4.44E-09	8.06667E-08
Synechococcus_sp._A18-40	0	2.70909E-08	1.77273E-08	7.86364E-08	4.13846E-08	1.00769E-07	8.17692E-08	6.23077E-08	2.72727E-08	0	0	0	5.18667E-08	0	0.000000042	0
Synechococcus_sp._TAK9802	3.18364E-07	3.79636E-07	4.12727E-09	1.12182E-07	6.30769E-08	0	2.76923E-07	7.63846E-08	1.89545E-07	1.13091E-07	8.93636E-08	7.07273E-08	8.04E-08	1.058E-07	2.97333E-08	8.19333E-08
Synechococcus_sp._MIT_S9220	5.99273E-07	8.91818E-08	4.12727E-09	6.59545E-07	6.19231E-08	2.21846E-07	5.36923E-08	5.22308E-08	3.29455E-07	3.18273E-07	2.55727E-07	2.16818E-07	4.48667E-08	0.000000233	2.20773E-07	1.94933E-07
Synechococcus_sp._Minos11	0	0	4.12727E-09	0	0	0	2.63077E-08	0	0	0	0	3.94545E-08	0	0	0	1.22E-08
Synechococcus_sp._SynAce01	2.43818E-07	0	4.12727E-09	1.35818E-07	2.88154E-07	1.50923E-07	1.62615E-07	1.35077E-07	2.72727E-07	6.69091E-08	2.54636E-07	0.000000568	8.42E-08	0.00000007	5.51333E-08	7.56E-08
Synechococcus_sp._ROS8604	0	0	2.18545E-08	3.24545E-08	0	0	0	0	0	3.62727E-08	0	2.14545E-08	0	0	0	0
Synechococcus_sp._KORDI-52	3.60091E-07	0	1.33455E-07	2.57091E-07	1.93231E-07	0	2.76231E-07	1.63077E-07	2.22455E-07	0.00000038	2.37727E-07	3.62636E-07	4.24867E-07	3.284E-07	3.64267E-07	9.25333E-07
Synechococcus_lividus	0.000000226	3.95455E-08	1.93364E-07	1.31364E-07	6.81538E-08	5.97231E-07	1.28615E-07	2.37692E-07	5.50909E-08	8.63636E-08	2.15182E-07	2.66455E-07	6.46E-08	5.22E-08	4.124E-08	1.98667E-08
Synechococcus_sp._JA-2-3B'a(2-13)	8.17273E-08	1.02545E-07	6.74909E-08	1.66182E-07	9.68462E-08	2.45615E-07	0.000000057	2.96769E-07	2.41364E-07	1.78636E-07	2.76364E-08	1.51273E-07	8.62E-08	1.16467E-07	1.72933E-07	5.58667E-08
Synechococcus_sp._PCC_7003	2.96364E-08	0	2.46182E-08	1.49882E-07	3.25154E-07	0.000000026	2.85923E-07	1.46923E-08	2.70091E-07	2.30364E-07	0.000000268	0.000000125	1.48667E-07	2.06267E-07	9.93333E-08	1.15467E-07
Synechococcus_sp._PCC_6312	3.42636E-07	7.40909E-08	4.12727E-09	9.09091E-08	1.61154E-07	7.78154E-07	1.42385E-07	3.69538E-07	3.00909E-07	2.06091E-07	2.77545E-07	7.59364E-07	1.358E-07	1.48133E-07	3.686E-07	2.96267E-07
Synechococcus_sp._MVIR-18-1	7.30818E-07	1.94545E-07	7.40909E-08	7.92545E-07	6.33154E-07	0	3.77692E-08	3.36923E-07	6.37182E-07	4.01364E-07	2.00909E-06	8.16818E-07	1.66133E-07	4.944E-07	7.36667E-07	2.50933E-07
Synechococcus_sp._RSCCF101	0.000000632	0	6.23636E-08	3.91364E-07	2.11615E-07	1.90462E-07	0.000000303	4.65231E-07	1.82182E-07	2.57818E-07	4.07636E-07	0.000000449	2.946E-07	1.24267E-07	3.11733E-07	5.656E-07
Synechococcus_sp._A15-28	1.51818E-07	3.87273E-08	3.70909E-08	1.60818E-07	0.000000243	0	1.99846E-07	3.88385E-07	2.11091E-07	1.73455E-07	3.69909E-07	2.08455E-07	4.23333E-08	0.000000244	2.75933E-07	1.77267E-07
Synechococcus_sp._BIOS-U3-1	1.82909E-07	1.32364E-07	1.63636E-08	1.41182E-07	1.75538E-07	2.37538E-07	7.02846E-07	1.02308E-07	3.30364E-07	0.000000458	3.53273E-07	3.29273E-07	1.40533E-07	4.496E-07	2.13867E-07	8.76E-08
Synechococcus_sp._PCC_7336	0	0	4.12182E-08	4.79091E-09	5.78462E-07	3.29154E-07	1.05385E-07	1.30308E-07	1.56727E-07	2.30818E-07	5.54727E-07	2.29182E-06	3.042E-07	5.01067E-07	6.88107E-07	3.31267E-07
Synechococcus_sp._PROS-U-1	5.27273E-08	9.20909E-08	7.40909E-08	3.82727E-08	3.77615E-07	0	5.36923E-08	4.64615E-08	1.48636E-07	8.08182E-08	1.44364E-07	0.0000001	1.60067E-07	2.76333E-07	2.23067E-07	5.39333E-08
Synechococcus_sp._NIES-970	7.94273E-07	8.25864E-07	1.80909E-07	8.00182E-07	1.34062E-06	7.94308E-07	2.40792E-06	1.07631E-06	5.93818E-07	8.42182E-07	5.08182E-07	5.41545E-07	0.000000795	5.08333E-07	8.56467E-07	1.0134E-06
Synechococcus_sp._LTW-R	1.11164E-06	0	1.94391E-07	9.60273E-07	1.52308E-07	1.45154E-07	2.03692E-07	1.08562E-06	3.82818E-07	0.000000224	2.85182E-07	2.80636E-07	3.02333E-07	6.41933E-07	7.118E-07	1.03773E-06
Synechococcus_sp._CB0101	1.22809E-06	3.24545E-07	5.01545E-07	0.000001002	0.000000306	9.26154E-08	0.000000057	1.16746E-06	5.86818E-07	5.92818E-07	1.00764E-06	9.77727E-07	6.15867E-07	6.474E-07	2.896E-07	4.10067E-07
Synechococcus_sp._JA-3-3Ab	1.13855E-06	0.000000145	5.50455E-07	1.23645E-06	5.23923E-07	7.87692E-07	0.000001382	1.15438E-06	9.78455E-07	1.16845E-06	0.000001141	1.39664E-06	5.14067E-07	1.1624E-06	6.81267E-07	7.05333E-07
Candidatus_Izimaplasma_sp._HR1	0.00001358	2.20909E-06	2.69153E-06	1.29045E-05	1.58508E-05	6.35308E-07	7.72192E-06	1.15697E-05	5.71155E-06	5.93136E-06	9.47364E-06	0.0000145	1.65887E-05	0.000015798	0.000021514	0.000024968
Entomoplasma_freundtii	0	0	0	0	0	0	0	0	0	5.22727E-08	0	0	0	0	0	0
Entomoplasma_melaleucae	0	0	0	3.84545E-08	0	0	0	1.94077E-07	0	0	5.52727E-08	0	0	0	0	5.52667E-08
Entomoplasma_somnilux	2.98182E-08	9.54545E-09	3.70909E-08	0	8.98462E-08	2.31538E-07	3.33077E-08	5.66923E-08	2.16364E-08	0	0	1.04636E-07	2.09333E-08	3.09333E-08	1.49333E-08	2.51533E-07
Entomoplasma_luminosum	1.58182E-08	0	1.65727E-08	0	2.59231E-08	6.35385E-08	4.33846E-08	1.61462E-07	0.000000133	5.22727E-08	4.92727E-08	7.78182E-08	1.19467E-07	1.228E-07	1.318E-07	1.17933E-07
Mesoplasma_entomophilum	0	0	0	0	8.02308E-08	0	0	0	0	0	0	0	6.08E-08	0	6.384E-08	0
Mesoplasma_coleopterae	0	0	0	4.79091E-09	3.12692E-07	0	0	1.90769E-08	0.000000084	0	0	9.98182E-08	1.10733E-07	7.94667E-08	2.8224E-07	1.182E-07
Mesoplasma_syrphidae	0	0	3.70909E-08	0	1.01923E-07	0	0	0	1.17636E-07	0	6.88182E-08	1.42727E-08	0	1.54467E-07	4.48667E-08	1.86E-08
Mesoplasma_chauliocola	3.11818E-08	0	2.96364E-08	5.18182E-09	0	0	2.34308E-07	1.80154E-07	0	0	3.95455E-07	1.58364E-07	2.09333E-08	0	7.07333E-08	0
Mesoplasma_lactucae	7.43636E-08	0	3.70909E-08	1.10364E-07	5.20769E-08	0	0	2.47692E-08	0.000000071	1.50273E-07	1.00182E-07	2.14545E-08	0	0.000000041	0.000000041	3.67333E-08
Mesoplasma_tabanidae	0	0	4.12727E-09	6.40455E-07	5.18308E-07	0	3.13923E-07	0.000000482	5.79091E-08	1.79455E-07	4.12727E-07	1.18182E-07	7.35733E-07	9.58067E-07	1.32933E-06	2.15313E-06
Mesoplasma_florum	5.12727E-08	0	0	6.01455E-07	7.22385E-07	2.61077E-07	4.04615E-07	7.96615E-07	1.48909E-07	1.09545E-07	0.000000439	0.000000138	1.34947E-06	1.26313E-06	1.46367E-06	2.84247E-06
Spiroplasma_sp._BIUS-1	0	0	0	0	0	0	0	0	0	0	0	0	0	3.09333E-08	0	0
Spiroplasma_sp._TIUS-1	0	0	0	0	0	2.95385E-08	0	0	0	0	0	0	6.25333E-08	0	9.26E-08	2.15333E-08
Spiroplasma_cantharicola	1.45273E-07	1.04545E-08	0	0	6.46923E-08	7.29231E-08	0	0	0	0	0	2.85455E-08	3.96E-08	3.09333E-08	3.37333E-08	1.98667E-08
Spiroplasma_gladiatoris	7.17273E-08	0	0	4.79091E-09	1.25923E-07	4.03692E-07	8.92308E-08	1.80077E-07	5.50909E-08	0	0	0	2.636E-07	8.66667E-08	0	3.57067E-07
Spiroplasma_floricola	1.10727E-07	0	0	9.54545E-09	0	0	0	6.32308E-08	4.32727E-08	3.62727E-08	2.66545E-07	3.94545E-08	0	0	0	1.09867E-07
Spiroplasma_melliferum	0	0	0	0	0	0	0	0	0	0	6.71818E-08	7.13636E-09	0	0	0	0
Spiroplasma_eriocheiris	7.30455E-07	0.000000344	9.04545E-08	3.18973E-07	4.26692E-07	2.84769E-07	7.26231E-07	1.76462E-07	6.71727E-07	4.81909E-07	2.59636E-07	1.79909E-07	4.164E-07	2.16467E-07	1.12933E-07	1.58933E-07
Spiroplasma_monobiae	2.46455E-07	4.23545E-07	2.09545E-07	2.08455E-07	0.000000191	3.46923E-08	2.87692E-07	5.30462E-07	1.59091E-07	0.000000075	1.41509E-06	1.12136E-06	2.37533E-07	2.90733E-07	3.54267E-07	4.794E-07
Spiroplasma_poulsonii	0.000000144	9.54545E-09	1.63636E-08	9.54545E-09	1.35538E-07	0	0	5.66923E-08	5.42727E-08	0	0	1.69727E-07	1.86667E-08	4.49333E-08	5.70667E-08	3.40667E-08
Spiroplasma_tabanidicola	4.54545E-08	0	0	0	0	0	0	1.04615E-07	0	0	3.95455E-08	0	0	0	0	6.86667E-08
Spiroplasma_alleghenense	0	2.40909E-08	0	3.24545E-08	6.16923E-08	0	2.73846E-08	1.46923E-08	0	3.03636E-08	3.33636E-08	7.13636E-09	0	0	0	7.33333E-08
Spiroplasma_sabaudiense	3.11818E-08	0	7.40909E-08	6.60636E-08	7.17692E-08	0	2.53615E-07	1.92154E-07	0.000000157	6.37273E-08	4.39545E-07	1.35909E-07	2.31333E-07	1.05333E-07	1.90067E-07	1.22333E-07
Spiroplasma_turonicum	0	0	7.82182E-08	9.13636E-08	0	0	0	0	3.75455E-08	1.61364E-07	1.61636E-07	0.000000057	2.64E-08	0	1.93733E-08	7.36667E-08
Spiroplasma_diminutum	1.78182E-07	0	4.12727E-09	0	0	3.30923E-07	0	0	0	1.07818E-07	2.67182E-07	4.45909E-08	5.00667E-08	0.000000041	3.31333E-08	0
Spiroplasma_syrphidicola	3.85636E-07	0.000000065	1.85455E-07	2.40791E-07	0	0	0	3.81538E-08	1.20182E-07	0.000000056	4.36364E-08	3.20273E-07	2.08E-08	4.88E-08	4.44E-09	9.33333E-09
Spiroplasma_chinense	0	0	0	4.79091E-09	3.15385E-08	0	7.54615E-08	2.46923E-08	0	0	3.77273E-08	7.13636E-09	4.27333E-08	3.48E-08	2.39333E-08	5.39333E-08
Spiroplasma_taiwanense	0	0	4.23455E-08	4.68818E-08	1.25692E-07	4.69231E-08	0	6.45385E-08	0	3.89091E-08	1.06727E-07	1.58545E-07	4.38E-08	8.18E-08	8.86667E-09	4.95333E-08
Spiroplasma_chrysopicola	5.27273E-08	2.74545E-08	0	0	8.61538E-08	0	0	0.000000042	9.73636E-08	0.000000034	0	0	0	6.74667E-08	2.09733E-08	0
Spiroplasma_helicoides	0.000000541	0.000000193	2.05636E-07	2.02364E-07	9.10769E-08	1.30769E-07	2.67615E-07	0.000000914	2.75091E-07	0.000000134	0.000000476	1.68909E-07	2.56533E-07	2.70733E-07	7.54333E-07	1.07273E-06
Spiroplasma_corruscae	4.39455E-07	1.37273E-08	5.00273E-07	4.28182E-08	5.40231E-07	0	6.58462E-07	2.26538E-07	9.95455E-08	4.50636E-07	4.03182E-07	7.64545E-08	3.23067E-07	5.398E-07	5.02867E-07	6.87533E-07
Spiroplasma_clarkii	2.96364E-08	2.34545E-08	3.70909E-08	1.91818E-08	2.88462E-08	3.83077E-08	0	8.62308E-08	5.97273E-08	0.000000062	0	7.55455E-08	1.17933E-07	1.86667E-07	1.948E-07	1.646E-07
Spiroplasma_culicicola	3.11818E-08	0	2.32545E-07	8.51818E-08	5.63846E-08	0	0	1.90769E-08	3.98182E-08	7.25455E-08	0.000000111	1.72727E-07	0	2.60067E-07	7.59333E-08	6.81333E-08
Spiroplasma_litorale	3.69091E-08	2.74545E-08	6.98182E-08	7.43636E-08	2.37538E-07	8.30769E-08	1.54692E-07	3.42923E-07	5.50909E-08	5.22727E-08	1.35636E-07	6.93636E-08	3.74067E-07	4.75933E-07	2.54933E-07	3.00133E-07
Spiroplasma_apis	1.66182E-07	0	4.12727E-09	4.92727E-08	4.64462E-07	0	8.69231E-08	0	0	0	0.000000039	0	3.50267E-07	4.72933E-07	7.27933E-07	7.40533E-07
Candidatus_Hepatoplasma_crinochetorum	2.56364E-08	0.000000065	0	1.247E-07	4.68154E-07	1.81308E-07	1.31154E-07	1.55769E-07	0.000000107	9.36364E-08	7.85455E-08	1.07455E-07	3.58867E-07	0.000000214	6.81333E-08	5.26667E-08
Ureaplasma_parvum	0	0	4.12182E-08	4.32727E-08	4.85462E-07	1.86123E-06	5.53077E-07	2.09692E-07	8.43636E-08	1.16273E-07	1.71727E-07	1.08182E-07	9.27933E-07	6.30533E-07	7.56667E-08	5.51333E-08
Ureaplasma_urealyticum	6.65455E-08	0	0	0	7.57538E-07	2.42454E-06	0.000000518	7.05154E-07	2.47273E-07	5.22727E-08	0.000000141	3.95364E-07	1.35627E-06	7.264E-07	5.93133E-07	0.000000378
Mycoplasmopsis_columbina	0	0	0	4.40909E-08	5.90769E-08	0	0	0	7.89091E-08	0	0	1.42727E-08	2.08E-08	3.09333E-08	1.026E-07	3.67333E-08
Mycoplasmopsis_glycophila	7.17273E-07	0.00000013	2.96364E-07	1.03636E-07	3.26923E-07	0	2.13846E-07	7.13077E-08	0.000000054	0	8.71818E-08	1.96136E-07	0	0	0	0.000000102
Mycoplasmopsis_columboralis	0	0	3.55455E-08	0	0	0	0	5.22308E-08	0	0	3.33636E-08	0	0	0	2.97333E-08	0
Mycoplasmopsis_californica	5.92273E-07	1.72964E-06	2.31182E-07	1.26055E-06	1.35715E-06	1.04462E-07	1.87769E-07	2.32462E-07	1.49273E-07	0.000000412	5.36818E-07	2.39564E-06	8.04267E-07	1.10853E-06	1.21953E-06	1.14193E-06
Mycoplasmopsis_gallinacea	4.86818E-07	3.28873E-06	6.65727E-07	0.000000175	1.01162E-06	1.48462E-07	1.00077E-07	5.40769E-08	1.41909E-07	5.22727E-08	3.95455E-08	2.82818E-07	7.08067E-07	7.284E-07	6.88533E-07	5.15267E-07
Mycoplasma_ovipneumoniae	0	0.000000065	0	0	0	0	0	0	0	3.89091E-08	0	0	0	0	0	0
Mycoplasma_sp._Pen4	0	0	0	0	0	0	0	0	0	0	0	0	2.14E-08	0	0	9.33333E-09
Mycoplasma_sp._C264-NAS	0	0	0	4.79091E-09	0	0	0	0	0	0	0	9.05455E-08	0	1.24933E-07	0	4.28E-08
Mycoplasma_haemofelis	0	0	0	1.91818E-08	0	0	0	0	0	6.37273E-08	0	0	0	0	0	0
Mycoplasma_salivarium	2.70909E-08	0	0	0	0	0	0	2.46923E-08	0	0	0	5.50455E-08	0	7.91333E-08	4.46667E-08	0
Mycoplasma_bovoculi	0	0	0	0	3.58462E-08	2.13846E-08	3.77692E-08	0	0	0	0	0	0	0	2.97333E-08	1.66667E-08
Mycoplasma_citelli	5.12727E-08	0	0	4.79091E-09	0	0	0	0	0.000000027	0	0	0	0	0	1.65333E-08	1.86E-08
Mycoplasma_columbinasale	0	0	0	2.41545E-08	0	0	0	0	0	3.03636E-08	0	0	2.64E-08	0	4.44E-09	0
Mycoplasma_synoviae	0	3.54545E-08	0	0	0	0	3.11538E-08	0	0	0	0	7.13636E-09	4.33333E-08	3.09333E-08	0	5.83333E-08
Mycoplasma_tullyi	0	0	0	9.54545E-09	0	4.58462E-07	2.11769E-07	1.46923E-08	9.75455E-08	3.62727E-08	0	7.13636E-09	6.62667E-08	0.000000074	0	1.86E-08
Mycoplasma_alkalescens	0	0	0	0	0.000000054	8.44615E-08	5.48462E-08	5.13077E-08	1.21545E-07	0	0	0	2.35133E-07	1.08267E-07	4.46867E-07	1.56267E-07
Mycoplasma_sp._Mirounga_ES2806-GEN	0	0	0	0	3.34615E-08	1.02692E-07	1.51538E-07	2.16615E-07	0	0	0	7.13636E-09	1.31933E-07	6.73333E-08	8.16E-08	1.72933E-07
Mycoplasma_suis	0	0	0	3.99091E-08	0	2.13846E-08	2.63077E-08	0	3.94545E-08	0	0	3.74545E-08	1.11467E-07	3.50667E-08	1.12667E-08	0
Mycoplasma_ovis	0	0	0	0	1.73077E-07	1.03777E-06	3.95231E-07	8.38462E-08	0	0	0	0	5.11667E-07	2.73133E-07	3.064E-08	3.40667E-08
Mycoplasma_parvum	0	0	0	0	2.29846E-07	1.12346E-06	1.77846E-07	0.000000042	0	0	0	0	6.82667E-07	3.08667E-07	5.51333E-08	4.95333E-08
Mycoplasma_iowae	2.98182E-08	0	0	3.35455E-08	1.80892E-06	3.84377E-06	1.04231E-06	1.88592E-06	3.75455E-08	6.37273E-08	0	7.89091E-08	0.000003972	1.04093E-06	6.36533E-07	3.78133E-07
Mycoplasma_dispar	3.11818E-08	0	0	4.79091E-09	0	0	0	0	0	0	0	0	0	0	2.39333E-08	1.86E-08
Mycoplasma_gallopavonis	3.11818E-08	0	0	0	0	0	0	0	0	0	0	0	2.37333E-08	0	4.44E-09	0
Mycoplasma_arginini	0	0	0	0	0	0	2.73846E-08	1.90769E-08	5.79091E-08	0	3.95455E-08	0	0.000000048	1.58E-08	1.436E-07	0
Mycoplasma_conjunctivae	0	2.65455E-08	3.70909E-08	2.04273E-07	2.26923E-08	0	5.26154E-08	0.000000088	7.18182E-08	0	0	1.43727E-07	0	0	8.86667E-09	1.86E-08
Mycoplasma_sp._1654_15	5.54545E-08	0.000000065	3.70909E-08	5.00909E-08	8.53846E-08	1.33231E-07	3.33077E-08	1.55538E-07	1.17636E-07	2.85455E-08	5.52727E-08	8.26818E-08	1.97667E-07	3.09333E-08	1.56467E-07	5.39333E-08
Mycoplasma_cynos	2.01636E-07	2.57091E-07	1.63636E-08	9.54545E-09	2.77154E-07	4.27692E-07	0	0	5.97273E-08	9.36364E-08	1.08364E-07	1.42727E-08	3.092E-07	3.64133E-07	2.09133E-07	1.778E-07
Mycoplasma_haemocanis	7.37273E-08	2.70909E-08	1.63636E-08	9.54545E-09	0	0	0	0	0	5.22727E-08	0	0.000000035	0	0	0	0
Mycoplasma_pulmonis	7.26364E-08	0	5.93636E-08	3.07273E-08	3.34615E-08	0	0.000000094	3.46231E-07	8.67273E-08	6.08182E-08	2.37636E-07	2.76091E-07	8.56667E-08	0	3.75733E-08	0.000000062
Mycoplasma_nasistruthionis	5.20909E-08	8.50909E-08	0	9.54545E-09	0	0	4.33846E-08	2.07231E-07	0.000000104	6.08182E-08	1.02727E-08	2.14545E-08	2.64E-08	0	0	0
Mycoplasma_anatis	0.000000047	0	0	0	3.88462E-08	0	0	2.97692E-08	6.68182E-08	0	1.35636E-07	0	0	0	0	0
Mycoplasma_flocculare	1.58182E-08	0	0	0	0	0	0	0	0	0	0	7.13636E-09	0	0	0	0
Mycoplasma_phocirhinis	1.02182E-07	0	8.40909E-08	0	4.13846E-08	0	2.63077E-08	0.000000068	8.54545E-08	5.22727E-08	0	1.10545E-07	0.000000082	1.342E-07	2.92867E-07	7.33333E-08
Mycoplasma_sp._(ex_Biomphalaria_glabrata)	0	0	0	4.80909E-08	1.09231E-07	2.01077E-07	1.05692E-07	5.19308E-07	5.48182E-08	0	1.69636E-07	0	2.38333E-07	0.000000108	1.574E-07	1.38067E-07
Mycoplasma_putrefaciens	1.14391E-06	1.94545E-07	3.33636E-07	1.18091E-07	6.03308E-07	0	3.71538E-07	5.81538E-07	2.55818E-07	1.69273E-07	1.89909E-07	6.75045E-07	0	4.91333E-08	4.44E-09	1.98667E-08
Mycoplasma_cloacale	0	0	0	4.79091E-09	0	0	0	1.35231E-07	0	0	3.33636E-08	0.000000035	6.66667E-08	6.74667E-08	5.33333E-08	0.000000342
Mycoplasma_phocicerebrale	3.62727E-08	0.000000065	0	0	0	0	0	5.88462E-08	6.62727E-08	0	3.33636E-08	0	5.44E-08	4.64667E-08	0	0
Mycoplasma_sp._Mirounga_ES2806-NAS	1.43182E-07	0	3.70909E-08	2.25545E-07	1.31692E-07	2.70462E-07	3.72385E-07	3.29231E-08	1.35545E-07	3.62727E-08	4.36364E-08	1.14636E-07	5.22333E-07	8.94667E-08	1.49333E-08	1.17533E-07
Mycoplasma_yeatsii	1.04364E-07	0.000000065	7.40909E-08	5.18182E-09	0	0	4.33846E-08	8.94615E-08	5.97273E-08	0	3.69909E-07	7.89091E-08	4.96E-08	0	1.18667E-07	6.86667E-08
Mycoplasma_bovigenitalium	1.28645E-06	0.00000013	5.92727E-07	1.28273E-07	0.000000615	0	8.24385E-07	0.00000051	1.22091E-07	6.66364E-08	2.56545E-07	9.56909E-07	2.91333E-08	1.514E-07	0	2.79333E-08
Mycoplasma_arthritidis	5.93636E-08	0	7.40909E-08	2.45455E-08	2.75385E-08	0	3.06923E-08	1.90769E-08	0	0	0.000000039	0	0	0	0	0
Mycoplasma_felis	2.07727E-07	1.49182E-07	0	1.15182E-07	0	0	4.33846E-08	0	0.000000027	1.84091E-07	2.48455E-07	0.000000695	0	0	0	4.76667E-08
Mycoplasma_canadense	0	0	4.12727E-09	5.18182E-09	2.05385E-08	5.91538E-08	2.63077E-08	6.10769E-08	0	0	0.000000039	4.79091E-08	1.68667E-07	4.67333E-08	2.83733E-08	5.10667E-08
Mycoplasma_wenyonii	4.56909E-07	0	4.12727E-09	0	0	0	3.68769E-07	5.92615E-07	5.45182E-07	4.28455E-07	0.000000235	0.000000134	2.08333E-07	1.046E-07	1.93733E-07	5.07933E-07
Mycoplasma_maculosum	0	0	2.18545E-08	0	9.46154E-08	1.44462E-07	1.52154E-07	1.38769E-07	0	0	3.77273E-08	5.37273E-08	2.18333E-07	0.00000005	3.93173E-07	1.172E-07
Mycoplasma_crocodyli	5.57273E-08	0	2.18545E-08	0	3.58462E-08	0	2.63077E-08	5.81538E-08	0	0	6.68182E-08	0	0	0	4.44E-09	7.44667E-08
Mycoplasma_gallisepticum	2.48636E-07	2.34545E-08	1.06945E-07	2.03364E-08	1.86923E-08	9.06923E-08	3.77692E-08	9.31538E-08	0.000000027	1.04545E-07	3.33636E-08	1.83545E-07	0	5.08667E-08	1.49333E-08	0
Mycoplasma_meleagridis	7.59091E-08	0	8.25455E-09	1.93636E-08	0	1.86769E-07	6.66154E-08	0	0	0	1.42182E-07	0	1.308E-07	1.58E-08	2.99733E-08	0
Mycoplasma_agalactiae	0	0	1.23636E-08	0	0	0	0	7.35385E-08	0	0	0	0	0	1.61333E-08	4.44E-09	1.46933E-07
Mycoplasma_pullorum	3.11818E-08	0	0	0	1.79846E-07	0	0	3.22308E-08	4.45455E-08	0	0	1.42727E-08	0	8.95333E-08	2.62E-08	2.58067E-07
Mycoplasma_anserisalpingitidis	1.42727E-07	0	1.77273E-08	1.02545E-07	4.37154E-07	6.50615E-07	0.000000273	3.12769E-07	1.55273E-07	0.000000028	7.56364E-08	1.31273E-07	2.75733E-07	3.48933E-07	3.618E-07	4.24467E-07
Mycoplasma_penetrans	2.35182E-07	0	0	1.69455E-07	1.03292E-06	2.57815E-06	3.74462E-07	9.00846E-07	0.000001053	1.71818E-07	5.07909E-07	2.64455E-07	1.94873E-06	7.394E-07	1.24873E-06	7.15667E-07
Mycoplasma_phocidae	0	0	0	0	3.58462E-08	0	0	2.50538E-07	0	0.000000034	2.76364E-08	1.13636E-07	2.254E-07	2.95933E-07	1.30867E-07	1.59067E-07
Mycoplasma_orale	3.62727E-08	1.37273E-08	5.93636E-08	2.03364E-08	2.05385E-08	0	0	5.81538E-08	1.15636E-07	1.34909E-07	1.02727E-08	1.50773E-07	6.51333E-08	2.38733E-07	5.98667E-08	1.15933E-07
Mycoplasma_neurolyticum	5.97273E-08	0	0	7.88182E-08	0	0	0	8.44615E-08	5.79091E-08	0	0	8.55455E-08	2.14E-08	1.808E-07	1.642E-07	1.176E-07
Mycoplasma_mobile	1.08364E-07	0	6.37273E-08	2.90909E-08	0	0	0	0	6.59091E-08	0	0.000000922	1.48273E-07	0	0.000000087	1.49333E-08	0
Mycoplasma_genitalium	1.25691E-06	0	4.98455E-07	1.21236E-06	4.55385E-07	7.46154E-08	2.07846E-07	7.14462E-07	8.01636E-07	4.94182E-07	4.67818E-07	8.98727E-07	6.388E-07	4.28667E-07	3.67667E-07	8.50333E-07
Mycoplasma_hyorhinis	0	0	0	7.61818E-07	1.22462E-07	5.91538E-08	0	1.74769E-06	0	0.000000047	5.93364E-07	1.42727E-08	6.69333E-08	1.374E-07	1.62667E-07	1.12867E-07
Mycoplasma_edwardii	6.23818E-07	1.94545E-07	1.48182E-07	5.18182E-08	5.32462E-07	0	1.86385E-07	6.84923E-07	9.15455E-08	0	4.75727E-07	3.81955E-07	2.24E-08	1.898E-07	1.39333E-07	0.00000019
Mycoplasma_bovis	7.64091E-07	0	7.40909E-08	8.89273E-07	1.21615E-06	0.000000026	4.94846E-07	9.14231E-07	4.07818E-07	3.11364E-07	5.49273E-07	1.03555E-06	1.02993E-06	1.13867E-06	1.44027E-06	2.9664E-06
Mycoplasma_struthionis	5.57273E-08	0	0	1.18655E-06	9.52077E-07	6.76923E-08	5.06846E-07	7.41154E-07	0	1.79545E-07	0.000000535	6.80864E-07	1.2638E-06	1.1422E-06	0.000001648	2.60507E-06
Mycoplasma_hyosynoviae	1.38273E-07	0	3.42727E-08	7.64636E-07	6.51846E-07	0	2.31154E-07	9.03385E-07	2.85273E-07	4.96364E-07	6.79545E-07	1.96818E-07	1.49227E-06	1.11813E-06	0.000001972	2.39733E-06

Mycoplasma_hyopneumoniae	0.000000204	0	1.65455E-08	5.92909E-07	0.000000196	0	4.75077E-07	1.72769E-07	1.02091E-07	8.85455E-08	2.07909E-07	6.93636E-08	1.01133E-07	3.08933E-07	1.83267E-07	1.006E-07
Mycoplasma_hominis	1.23545E-07	0	7.82182E-08	1.49636E-07	1.81077E-07	1.79231E-08	5.74615E-08	1.29462E-07	1.49455E-07	2.08273E-07	3.84364E-07	1.73455E-07	3.12133E-07	2.97933E-07	2.96267E-07	1.854E-07
Mycoplasma_capricolum	2.98182E-08	0	0	0	1.95538E-07	0	0	9.99231E-08	3.75455E-08	0	0	1.42727E-08	0	1.296E-07	9.204E-08	1.00267E-07
Mycoplasma_mycoides	3.11818E-08	0	1.63636E-08	0	2.62008E-06	0	0	5.26923E-07	2.60909E-07	1.00909E-07	6.64182E-07	4.88727E-07	1.62047E-06	1.20467E-06	2.02213E-06	1.8342E-06
Mycoplasma_canis	0.000000372	0	0	5.57909E-07	1.26562E-06	0	1.81769E-07	5.51462E-07	4.45455E-08	9.36364E-08	5.00818E-07	5.73955E-07	1.47733E-06	1.25853E-06	1.92393E-06	2.1608E-06
Mycoplasma_sp._Phocoena_C-264-GEN	2.56364E-08	0	0	2.13727E-07	6.30769E-08	0	0	1.46923E-08	9.73636E-08	8.56364E-08	1.47545E-07	1.95909E-07	1.016E-07	3.48E-08	8.35333E-08	2.94067E-07
Mycoplasma_sp._NEAQ87857	5.38455E-07	0	4.44545E-07	1.23973E-06	1.56254E-06	0.000000306	4.42385E-07	1.33023E-06	0.000000397	0.000000359	1.46218E-06	0.000000761	1.7018E-06	1.52687E-06	1.66933E-06	2.92053E-06
Mycoplasma_bovirhinis	3.11818E-08	0	1.63636E-08	6.38818E-08	2.88346E-06	2.52308E-08	4.05769E-07	1.09969E-06	6.77909E-07	1.04545E-07	1.91273E-07	4.92636E-07	1.56367E-06	1.62507E-06	2.14167E-06	2.09833E-06
Mycoplasma_anseris	0	0	0	0	3.28335E-05	1.80769E-07	1.51077E-07	9.99338E-06	0.000010173	0.000005069	2.56609E-06	9.55682E-06	1.61891E-05	2.35711E-05	3.15061E-05	3.23355E-05
Maize_bushy_stunt_phytoplasma	0	0	0	0	2.88462E-08	0	2.20385E-07	1.25692E-07	0	0	0	0	2.24E-08	6.64E-08	6.46667E-08	1.66667E-08
Candidatus_Phytoplasma_ziziphi	0	0	0	2.43636E-08	0	0	2.63077E-08	1.90769E-08	0	0	0	0	0.000000027	4.49333E-08	0	0
Aster_yellows_witches'-broom_phytoplasma	0	0	0	1.91818E-08	2.26923E-08	0	0	0	0	0	1.00182E-07	0.000000057	0	0	0.000000132	0
Candidatus_Phytoplasma_mali	1.29509E-06	1.94545E-07	2.96364E-07	7.22455E-08	3.38462E-07	0	0.000000347	2.44692E-07	1.20182E-07	0.000000028	8.26364E-08	4.67818E-07	2.72E-08	1.214E-07	1.77933E-07	5.66E-08
Candidatus_Phytoplasma_australiense	6.31636E-06	4.10818E-07	9.88845E-07	2.71182E-06	5.13154E-06	2.95385E-08	3.34354E-06	4.42808E-06	3.18809E-06	0.000004791	4.47182E-06	3.49314E-06	0.000005832	5.69133E-06	8.50933E-06	9.70333E-06
Acholeplasma_hippikon	2.45564E-06	0.00000039	1.29725E-06	1.21682E-06	5.40462E-07	5.63385E-07	6.53462E-07	8.95769E-07	1.12664E-06	4.89818E-07	2.05409E-06	1.96509E-06	4.42333E-07	3.772E-07	1.13587E-06	6.25533E-07
Acholeplasma_axanthum	1.03787E-05	2.62418E-06	2.32736E-06	1.16073E-06	4.39277E-06	1.51538E-07	0.000004999	0.000003903	1.48309E-06	0.000002214	2.52955E-06	5.02136E-06	4.056E-07	8.438E-07	6.422E-07	8.58267E-07
Acholeplasma_oculi	2.65818E-07	0	2.95491E-07	5.38727E-07	2.40538E-07	2.08308E-07	5.61692E-07	0.000000884	2.88818E-07	4.29909E-07	2.44009E-06	0.000001213	5.68133E-07	7.80067E-07	9.80907E-07	6.864E-07
Acholeplasma_laidlawii	1.82545E-07	1.68182E-08	1.25855E-07	9.29545E-07	8.25385E-07	0	8.76462E-07	1.22962E-06	9.15455E-08	0.000000523	8.54818E-07	9.08182E-07	9.758E-07	8.89333E-07	1.49033E-06	2.38187E-06
Acholeplasma_brassicae	6.85455E-06	2.13559E-06	1.82518E-06	1.00159E-05	0.00001592	1.23462E-07	7.54192E-06	1.25785E-05	1.66982E-06	2.39809E-06	5.73173E-06	8.38945E-06	1.55173E-05	1.52791E-05	0.000023078	0.000028384
Acholeplasma_palmae	2.64564E-06	4.00455E-07	8.48091E-07	1.35527E-05	2.07282E-05	8.43846E-08	1.03766E-05	1.54277E-05	9.70273E-07	3.66836E-06	0.000007852	1.19395E-05	0.000023392	2.33103E-05	3.46074E-05	3.89975E-05
Conexibacter_woesei	6.41818E-07	0.000001347	1.38727E-07	5.75091E-07	2.58308E-07	7.11538E-07	2.73538E-07	4.64692E-07	5.56909E-07	3.54818E-07	0.000000407	6.53545E-07	1.14267E-07	0.000000295	2.428E-07	2.20267E-07
Acidimicrobium_ferrooxidans	3.21364E-07	9.01364E-08	8.94E-08	0.000000131	1.25462E-07	8.44615E-08	1.32769E-07	1.78846E-07	2.04727E-07	2.31545E-07	7.33727E-07	4.79545E-07	1.82667E-07	9.56E-08	1.432E-07	1.76467E-07
Ilumatobacter_coccineus	1.91273E-07	3.23182E-08	8.81818E-07	2.23455E-07	0	2.70769E-07	4.56231E-07	6.18077E-07	2.66545E-07	9.92727E-08	0.000000196	4.23273E-07	2.08E-08	2.032E-07	0.00000013	1.74533E-07
Actinomarinicola_tropica	8.76909E-07	1.11727E-07	3.32818E-07	8.01909E-07	6.29462E-07	3.05585E-06	0.000000467	5.86769E-07	0.000000563	4.16182E-07	6.94727E-07	1.02164E-06	2.274E-07	2.774E-07	6.02467E-07	3.60467E-07
Baekduia_soli	4.04364E-07	0.000000112	0.000000184	0.000000163	2.64923E-07	0.000000155	3.10923E-07	1.60754E-06	1.04918E-06	9.64455E-07	1.54345E-06	0.00000106	4.97067E-07	5.16733E-07	1.05167E-06	1.61033E-06
Rubrobacter_xylanophilus	1.98018E-06	1.94473E-06	5.28727E-07	1.24009E-06	2.43231E-07	8.99769E-07	6.27308E-07	1.16085E-06	1.24691E-06	7.14273E-07	1.05873E-06	0.000002264	3.15733E-07	2.43867E-07	5.58467E-07	5.038E-07
Rubrobacter_indicoceani	5.31545E-07	2.71636E-07	2.035E-07	3.46091E-07	2.33231E-07	6.76923E-08	5.74615E-08	9.99231E-08	3.07545E-07	2.28273E-07	6.56636E-07	1.96182E-07	2.43733E-07	2.38533E-07	2.486E-07	0.000000154
Rubrobacter_radiotolerans	2.10909E-06	5.97545E-07	7.52182E-07	1.22409E-06	1.23554E-06	7.32077E-07	6.59692E-07	2.67446E-06	1.63564E-06	7.02182E-07	1.78091E-06	2.45127E-06	7.89267E-07	1.0964E-06	1.0974E-06	5.834E-07
Rubrobacter_sp._SCSIO_52915	4.73273E-07	2.41273E-07	9.86727E-08	3.31273E-07	2.75231E-07	7.09769E-07	4.92308E-07	3.77231E-07	4.53909E-07	4.02727E-07	6.12545E-07	4.15727E-07	2.876E-07	0.000000377	4.2084E-07	2.62333E-07
Rubrobacter_sp._SCSIO_52909	1.66809E-06	1.97364E-07	2.79582E-07	7.23364E-07	3.97231E-07	7.76462E-07	7.14462E-07	0.000001261	1.06882E-06	7.63818E-07	1.10891E-06	2.58745E-06	5.024E-07	7.696E-07	1.2876E-06	1.2104E-06
Egicoccus_halophilus	9.95182E-07	8.40545E-07	4.39864E-07	6.85727E-07	4.35462E-07	9.46154E-09	6.73462E-07	6.74462E-07	4.20545E-07	7.63182E-07	5.45364E-07	1.90709E-06	0.000000268	2.902E-07	1.5824E-07	2.386E-07
Euzebya_sp._DY32-46	1.05445E-06	0.000002005	5.50764E-07	2.78473E-06	3.23538E-07	1.20662E-06	2.78877E-06	2.38638E-06	2.73945E-06	2.27791E-06	1.28155E-06	1.38264E-06	2.73333E-07	3.93133E-07	6.16667E-07	6.876E-07
Egibacter_rhizosphaerae	3.30727E-07	3.11182E-07	3.11455E-07	7.36273E-07	2.46385E-07	3.15385E-08	2.99538E-07	1.04154E-06	2.60545E-07	1.10636E-07	4.62636E-07	5.54455E-07	2.088E-07	2.19333E-07	2.62667E-07	6.97667E-07
Coriobacterium_glomerans	3.21545E-06	3.10023E-06	1.55736E-06	2.91909E-06	1.46615E-06	6.42615E-07	1.91346E-06	7.86862E-06	2.06718E-06	2.71582E-06	4.14027E-06	4.14455E-06	1.69347E-06	1.80433E-06	2.62447E-06	4.8094E-06
Collinsella_aerofaciens	3.41455E-05	1.99607E-05	1.37155E-05	2.90345E-05	2.51015E-05	1.99484E-05	2.60062E-05	7.01818E-05	3.67273E-05	3.31364E-05	3.38455E-05	3.95182E-05	0.000019086	2.75533E-05	2.51267E-05	4.65276E-05
Lancefieldella_parvula	0.000001151	2.08273E-07	2.55364E-07	4.23182E-07	5.57538E-07	3.45615E-07	5.09923E-07	4.65077E-07	7.95273E-07	1.12336E-06	6.39182E-07	7.70182E-07	5.37267E-07	1.13533E-06	1.08547E-06	1.25127E-06
Parolsenella_catena	6.59727E-06	1.12445E-06	5.39327E-06	5.79009E-06	7.15615E-06	9.13769E-07	4.61692E-06	5.16788E-05	1.19918E-05	8.20364E-06	1.37473E-05	1.36518E-05	9.12333E-06	1.10607E-05	0.0000136	0.000031674
Libanicoccus_massiliensis	8.40455E-06	2.64573E-06	7.67182E-06	1.34281E-05	1.15692E-05	1.09322E-05	8.86538E-06	5.83214E-05	1.61627E-05	1.36182E-05	1.91791E-05	1.96218E-05	1.08613E-05	1.43393E-05	1.84253E-05	3.35055E-05
Olsenella_umbonata	7.84636E-06	3.71064E-06	5.60291E-06	8.19627E-06	6.37846E-06	3.68238E-06	5.41538E-06	4.49238E-05	1.47673E-05	1.03555E-05	1.25891E-05	1.28882E-05	6.82533E-06	8.63733E-06	1.12253E-05	2.53404E-05
Olsenella_uli	1.16655E-05	5.82018E-06	7.48509E-06	1.17854E-05	5.90692E-06	7.48492E-06	1.00492E-05	6.63993E-05	1.81673E-05	1.15564E-05	0.000020031	1.45082E-05	0.000010162	1.40007E-05	1.66953E-05	3.77431E-05
Olsenella_timonensis	1.26545E-05	0.000006829	1.22391E-05	0.000019037	1.26292E-05	1.32878E-05	1.28731E-05	0.000111248	2.37109E-05	1.86455E-05	3.03073E-05	2.57545E-05	1.45707E-05	2.22827E-05	2.85933E-05	6.22409E-05
Olsenella_sp._oral_taxon_807	0.000004419	1.62818E-06	2.64236E-06	5.01591E-06	3.31385E-06	4.97977E-06	3.05662E-06	2.17554E-05	4.14818E-06	0.000003544	7.16273E-06	5.98182E-06	3.94267E-06	4.97267E-06	6.54133E-06	1.46127E-05
Olsenella_sp._LZLJ-2	8.37727E-06	3.64909E-06	9.39055E-06	1.21063E-05	1.06231E-05	8.64638E-06	8.77692E-06	8.23712E-05	1.67155E-05	1.36382E-05	2.04327E-05	1.73364E-05	1.21493E-05	0.000015326	1.88487E-05	0.000047605
Olsenella_sp._GAM18	1.52755E-05	6.06491E-06	1.59709E-05	2.07709E-05	2.04131E-05	2.05545E-05	1.71754E-05	0.000145742	3.26727E-05	0.0000251	4.04786E-05	3.25491E-05	2.39147E-05	3.02351E-05	3.82093E-05	8.38712E-05
Denitrobacterium_detoxificans	9.61091E-06	2.19673E-06	3.22436E-06	4.68355E-06	0.00000514	1.25115E-06	6.90692E-06	8.91462E-06	5.42636E-06	6.73364E-06	5.26273E-06	7.31636E-06	3.93133E-06	4.76533E-06	4.49627E-06	0.00000512
Cryptobacterium_curtum	6.61273E-07	0.000000729	2.39091E-07	1.06882E-06	3.53615E-07	0	8.11154E-07	3.81538E-07	4.04182E-07	4.49909E-07	5.90364E-07	3.99727E-07	0.000000304	8.85267E-07	8.90467E-07	0.000000565
Phoenicibacter_congonensis	0.000001416	0.00000026	2.88273E-07	3.71455E-07	1.53723E-06	1.23077E-07	6.21769E-07	1.30923E-06	6.87182E-07	1.52673E-06	1.14973E-06	1.05509E-06	1.6596E-06	2.6176E-06	2.74333E-06	1.79453E-06
Arabia_massiliensis	2.73091E-05	3.04882E-06	4.53327E-06	1.30281E-05	1.08815E-05	0.000001241	1.02477E-05	2.29538E-05	1.56709E-05	1.74109E-05	1.48209E-05	1.66364E-05	1.23287E-05	1.29087E-05	0.000012636	0.000017236
Slackia_heliotrinireducens	1.64382E-05	6.67191E-06	3.91845E-06	1.22745E-05	9.59692E-06	9.52738E-06	8.34231E-06	1.17669E-05	9.74909E-06	1.20164E-05	0.00000941	1.53091E-05	0.000008384	1.06873E-05	0.000009728	9.15133E-06
Gordonibacter_urolithinfaciens	9.94636E-06	1.28509E-06	1.18166E-06	5.69791E-06	4.55969E-06	4.35308E-06	4.31654E-06	5.73385E-06	4.77818E-06	8.64273E-06	5.43636E-06	7.98636E-06	4.81867E-06	4.81067E-06	5.82133E-06	5.81467E-06
Gordonibacter_pamelaeae	1.58382E-05	2.04291E-06	3.19045E-06	8.76882E-06	5.89462E-06	1.07815E-06	6.08077E-06	1.10992E-05	1.15673E-05	8.87727E-06	7.47909E-06	0.00000794	7.22733E-06	0.000008476	7.29467E-06	0.000009602
Eggerthella_lenta	2.31909E-05	1.23232E-06	0.000003847	1.08664E-05	7.82692E-06	2.17246E-06	7.86615E-06	1.54254E-05	1.32227E-05	1.33091E-05	1.22245E-05	1.22136E-05	9.68867E-06	0.000008992	1.07993E-05	1.35613E-05
Eggerthella_sp._YY7918	5.78818E-06	8.91091E-07	1.59927E-06	3.62945E-06	2.38308E-06	0.000001265	2.89885E-06	4.45538E-06	3.08064E-06	4.87364E-06	0.00000389	4.04909E-06	2.3858E-06	3.57133E-06	3.06047E-06	3.18933E-06
Eggerthella_sp._HF-1101	0.0000202	2.34927E-06	3.26955E-06	1.05422E-05	7.92769E-06	5.20185E-06	1.00492E-05	1.73862E-05	1.07664E-05	1.39027E-05	1.03336E-05	1.28009E-05	1.00807E-05	0.00001007	9.53067E-06	1.41653E-05
Adlercreutzia_equolifaciens	0.000182423	9.27773E-06	1.06119E-05	8.02423E-05	6.25878E-05	5.72554E-06	9.38441E-05	0.000124616	9.30945E-05	0.00011648	0.000070564	0.000106322	7.61197E-05	6.51378E-05	5.61571E-05	6.84114E-05
Adlercreutzia_sp._8CFCBH1	0.000210557	1.23485E-05	1.26406E-05	9.49261E-05	7.41191E-05	2.58908E-06	0.000108847	0.000147007	0.000108901	0.000135041	7.99276E-05	0.000119074	8.70232E-05	7.40579E-05	6.54749E-05	7.97886E-05
Kineococcus_radiotolerans	2.96364E-08	2.70909E-08	0	9.97273E-09	2.88462E-08	1.14846E-06	3.70846E-07	1.63846E-07	2.75455E-08	2.85455E-08	0	9.98182E-08	2.37333E-08	0	1.12667E-08	3.72E-08
Nakamurella_multipartita	0	0	0	4.79091E-09	2.05385E-08	1.26769E-07	2.63077E-08	3.94615E-08	0	0	0	7.13636E-09	2.14667E-08	0.00000007	5.52667E-08	4.28E-08
Nakamurella_panacisegetis	3.11818E-08	0	0	1.03636E-08	0	1.14615E-07	0	0	0	0	0	1.42727E-08	0	0	0	0
Nakamurella_sp._PAMC28650	3.11818E-08	0	0	1.03636E-08	2.75385E-08	0	2.73846E-08	0	0	0	9.41818E-08	0.000000035	2.14E-08	0	4.44E-09	1.28533E-07
Nakamurella_sp._s14-144	2.68636E-07	1.31273E-07	3.70909E-08	2.79455E-07	1.34769E-07	0	2.63077E-08	1.57615E-07	3.46091E-07	3.83545E-07	0.000000345	3.76364E-07	6.08E-08	9.26E-08	2.78E-08	0.000000026
Jiangella_sp._DSM_45060	0	3.45455E-08	0	9.54545E-09	2.88462E-08	4.20462E-07	1.05692E-07	5.22308E-08	0	3.62727E-08	1.02727E-08	1.11818E-07	2.37333E-08	3.50667E-08	1.49333E-08	1.98667E-08
Jiangella_alkaliphila	1.09455E-07	6.76364E-08	9.04545E-08	1.08545E-07	8.79231E-08	0	1.76231E-07	0.000000022	1.32727E-07	3.03636E-08	1.12455E-07	1.325E-07	2.09333E-08	9.00667E-08	1.1404E-07	0
Acidothermus_cellulolyticus	1.02027E-06	2.50818E-07	5.49182E-07	9.79273E-07	2.70846E-07	4.48769E-07	5.53846E-07	2.83154E-07	1.03518E-06	5.54182E-07	1.21373E-06	8.36636E-07	4.44933E-07	4.38733E-07	3.37133E-07	4.42267E-07
Epidermidibacterium_keratini	0	2.32727E-08	1.18673E-07	2.96727E-07	1.38077E-07	0.0000005	0	6.45385E-08	7.50909E-08	1.22182E-07	1.16636E-07	4.59091E-08	1.046E-07	0.000000069	7.06667E-08	1.42333E-07
Catenulispora_acidiphila	1.43109E-06	1.23018E-06	4.81455E-07	1.01391E-06	1.00538E-07	6.69769E-07	3.28077E-07	2.42077E-07	1.06273E-06	9.34909E-07	1.76873E-06	2.29836E-06	9.39333E-08	3.488E-07	4.546E-07	1.17333E-07
Geodermatophilus_obscurus	2.49818E-07	9.54545E-09	0	4.35455E-08	1.07538E-07	1.75385E-07	5.96154E-08	1.06154E-07	1.70636E-07	5.73636E-08	3.48273E-07	3.00909E-07	2.298E-07	1.55533E-07	7.51067E-08	1.32933E-07
Modestobacter_marinus	0.000000284	2.07273E-08	0.000000208	0.000000263	3.88462E-08	1.00538E-07	7.45385E-08	2.19462E-07	1.93727E-07	1.66273E-07	3.96818E-07	2.52909E-07	4.23333E-08	1.25733E-07	9.31333E-08	9.98667E-08
Blastococcus_saxobsidens	0.000000423	3.15455E-08	2.16727E-08	1.55418E-06	5.98462E-08	1.73231E-06	2.76385E-07	1.85323E-06	0.000000581	7.02364E-07	9.00455E-07	3.59182E-07	1.90667E-07	3.11067E-07	3.52973E-07	2.34267E-07
Thermobispora_bispora	0.000001135	3.31136E-07	1.14182E-07	1.13909E-06	2.91846E-07	3.03538E-06	3.42692E-07	6.75077E-07	6.59818E-07	7.85545E-07	4.14364E-07	7.36364E-07	8.436E-07	5.91467E-07	7.92733E-07	1.0882E-06
Jatrophihabitans_sp._GAS493	2.86364E-07	0	0	1.19636E-07	4.54615E-08	0	0	5.81538E-08	4.91818E-08	1.03727E-07	4.81818E-08	1.15273E-07	1.026E-07	1.228E-07	4.30667E-08	1.07667E-07
Frankia_casuarinae	3.15455E-08	7.54545E-08	0	5.18182E-09	0	1.04462E-07	1.23692E-07	0.000000022	9.18182E-08	0.000000028	3.95455E-08	7.78182E-08	2.44E-08	1.58E-08	4.82E-08	9.15333E-08
Frankia_sp._EAN1pec	3.69091E-08	2.34545E-08	3.70909E-08	0	3.15385E-08	2.11385E-07	1.51077E-07	9.89231E-08	5.97273E-08	0	3.33636E-08	8.42727E-08	2.37333E-08	0	6.54E-08	9.33333E-09
Frankia_inefficax	8.68182E-08	1.94545E-07	0	2.61545E-07	1.64692E-07	0	2.38077E-07	1.30846E-07	0	0	1.15909E-07	9.51818E-08	0.000000097	2.12533E-07	5.44667E-08	6.52E-08
Candidatus_Frankia_datiscae	3.06727E-07	1.15273E-07	3.78909E-08	3.26455E-07	3.05923E-07	7.22077E-07	1.19231E-07	2.06538E-07	3.12545E-07	2.30545E-07	7.74545E-08	4.99545E-07	1.18467E-07	2.274E-07	2.59333E-07	1.37333E-07
Frankia_alni	2.03727E-07	9.03636E-08	0	1.60182E-07	0.000000104	5.96923E-08	8.59231E-08	5.13077E-08	1.02364E-07	1.56182E-07	4.23091E-07	1.93091E-07	1.456E-07	1.28267E-07	1.954E-07	7.52667E-08
Frankia_sp._QA3	1.68091E-07	2.69091E-08	4.12727E-09	0.000000226	5.96846E-07	2.87923E-07	3.95692E-07	3.41923E-07	5.62909E-07	3.43545E-07	4.60545E-07	5.84364E-07	1.60067E-07	2.722E-07	5.33867E-07	4.342E-07
Stackebrandtia_nassauensis	1.36464E-06	9.55455E-08	4.12182E-08	1.59491E-06	3.39046E-06	4.24308E-07	9.14308E-07	2.54554E-06	4.97655E-06	2.42927E-06	3.87355E-06	3.07527E-06	3.40407E-06	2.24667E-06	3.7774E-06	2.46633E-06
Candidatus_Nanopelagicus_hibericus	2.86455E-07	0	3.70909E-08	2.90909E-08	1.22385E-07	0.000000026	2.33308E-07	7.93385E-07	0.000000247	3.03636E-08	2.62727E-07	3.20818E-07	2.14E-08	7.74E-08	1.292E-07	1.22E-08
Candidatus_Nanopelagicus_abundans	5.27273E-08	3.24545E-07	0	1.22364E-07	1.16538E-07	0	0	2.93846E-08	3.94545E-08	6.43636E-08	6.68182E-08	8.55455E-08	1.93533E-07	2.602E-07	1.87307E-07	3.73333E-08
Candidatus_Planktophila_sulfonica	2.70909E-08	0	0	0	0	0	0	2.46923E-08	0	0	0	0	0	0	0	0
Candidatus_Planktophila_limnetica	5.57273E-08	0	0	5.76364E-08	2.75385E-08	0	0	0	5.97273E-08	0.000000264	0	1.18182E-07	1.242E-07	4.49333E-08	4.91067E-08	1.296E-07
Candidatus_Planktophila_dulcis	1.58182E-08	3.47636E-07	3.70909E-08	4.79091E-09	0	3.52308E-07	0	1.90769E-08	0	0	2.05455E-08	0	0	0	0	0
Candidatus_Planktophila_lacus	0	0	4.58455E-08	0	3.58462E-08	4.56077E-07	0	1.00154E-07	0	3.89091E-08	1.06545E-07	0	0	4.84667E-08	8.48E-08	0
Candidatus_Planktophila_versatilis	7.83636E-08	1.10045E-07	3.68545E-08	0	1.23769E-07	0	1.08077E-07	3.74769E-07	1.20818E-07	1.36091E-07	1.56364E-07	0.000000181	3.12333E-07	2.418E-07	2.52133E-07	2.75533E-07
Candidatus_Planktophila_vernalis	1.17836E-06	0.000000065	2.25855E-07	1.22764E-06	4.12077E-07	0	1.85462E-07	4.92692E-07	6.54091E-07	5.18091E-07	6.50545E-07	9.56182E-07	4.35733E-07	7.35667E-07	3.88333E-07	5.89267E-07
Actinopolyspora_erythraea	6.05364E-07	1.94545E-07	1.60545E-07	7.32182E-07	0.000001234	2.91177E-06	4.83385E-07	4.31538E-07	9.06364E-07	1.24373E-06	0.000001301	1.54791E-06	8.11733E-07	1.5634E-06	7.94667E-07	8.32933E-07
Streptomonospora_sp._M2	0.000000114	2.70909E-08	2.59818E-08	2.05545E-07	2.59231E-08	0	7.45385E-08	0	3.75455E-08	0	1.56091E-07	6.35455E-08	0	3.50667E-08	5.684E-08	6.33333E-08
Thermobifida_fusca	8.26364E-07	4.45455E-08	7.67636E-08	1.74591E-06	2.75769E-07	9.94923E-07	3.77308E-07	4.25308E-07	5.82727E-07	0.000000563	9.21818E-07	1.05109E-06	3.338E-07	5.53733E-07	2.476E-07	9.564E-07
Nocardiopsis_dassonvillei	2.33182E-07	1.59455E-07	1.61182E-07	2.18545E-07	2.11615E-07	0.000000041	0.000000263	3.67846E-07	1.89364E-07	2.51636E-07	2.12909E-07	3.29636E-07	7.91333E-08	6.52667E-08	6.13333E-08	1.85667E-07
Nocardiopsis_gilva	0.00000024	2.72727E-08	1.63636E-08	1.63191E-06	1.06538E-07	2.41915E-06	4.26115E-06	2.93992E-06	0.000000745	5.38455E-07	7.32455E-07	9.00364E-07	2.55267E-07	1.586E-07	6.06267E-07	1.852E-07
Nocardiopsis_alba	3.98364E-07	6.93182E-08	2.08364E-07	2.01273E-07	3.15385E-08	3.53538E-07	6.96923E-08	3.55615E-07	1.99182E-07	7.25455E-08	2.38273E-07	2.29818E-07	7.52667E-08	1.70867E-07	2.85333E-07	1.14333E-07
Thermomonospora_curvata	6.64818E-07	1.94545E-07	3.83636E-08	4.57909E-07	3.75846E-07	4.68462E-07	3.68154E-07	0.000000471	3.30818E-07	5.10091E-07	7.74091E-07	1.02182E-06	5.59533E-07	6.848E-07	6.37467E-07	6.63667E-07
Actinomadura_amylolytica	3.52909E-07	1.94545E-07	3.70909E-08	2.75336E-07	7.17692E-08	1.69846E-07	1.53308E-07	1.54692E-07	2.68727E-07	1.03727E-07	8.75455E-08	4.51909E-07	8.02667E-08	1.04533E-07	2.016E-07	2.03333E-07
Actinomadura_verrucosospora	8.96364E-08	0	1.10909E-07	9.19091E-08	3.34615E-08	1.57923E-07	0.000000338	0.000000022	1.34818E-07	0	0.000000061	9.91364E-08	4.38E-08	1.43467E-07	8.24667E-08	1.98667E-08
Actinomadura_sp._NAK00032	1.17091E-07	0.000000047	4.10909E-08	1.32455E-07	1.93538E-07	2.95385E-08	8.37692E-08	1.48769E-07	9.73636E-08	9.13636E-08	1.16273E-07	9.07273E-08	1.14533E-07	1.58467E-07	2.33267E-07	1.86E-08
Actinomadura_sp._WMMB_499	6.23636E-08	0	7.40909E-08	1.43636E-08	1.10769E-07	2.95385E-08	0	0	0	0	1.99727E-07	7.78182E-08	2.72E-08	6.74667E-08	1.7484E-07	4.28E-08
Actinomadura_sp._WMMA1423	0.000000382	1.09682E-06	1.10945E-07	6.42455E-07	3.01308E-07	0	5.45846E-07	3.74769E-07	2.73182E-07	0.000000364	3.23091E-07	6.01455E-07	2.46533E-07	7.16533E-07	5.566E-07	3.884E-07
Streptosporangium_roseum	2.09545E-07	7.23636E-08	1.51955E-07	5.19091E-07	2.84769E-07	1.65077E-07	4.27923E-07	1.64108E-06	3.58091E-07	3.73818E-07	5.20273E-07	9.11727E-07	1.47733E-07	4.89533E-07	5.54733E-07	9.82467E-07
Streptosporangium_sp._'caverna'	2.70909E-08	1.82273E-07	1.28818E-07	4.35727E-07	0	2.58769E-07	6.96923E-08	8.14615E-08	3.75455E-08	0.000000206	1.06182E-07	3.52273E-07	0	3.48E-08	9.35733E-08	5.10667E-08
Nonomuraea_sp._ATCC_55076	7.22909E-07	7.41909E-07	1.45918E-06	8.04909E-07	5.76154E-08	4.56923E-07	1.00577E-06	0.000000874	7.75727E-07	4.95545E-07	6.94364E-07	8.75727E-07	0.000000195	2.73733E-07	1.984E-07	1.46933E-07
Nonomuraea_nitratireducens	1.55645E-06	3.23455E-07	4.28364E-07	4.51182E-07	6.80462E-07	1.36231E-06	2.30615E-07	1.99923E-07	1.01518E-06	0.000001294	1.16145E-06	1.03736E-06	7.51933E-07	1.15913E-06	8.32333E-07	6.418E-07
Lentzea_guizhouensis	2.70909E-08	0	0	2.90909E-08	0	0	3.11538E-08	1.90769E-08	0	0	0	0	0	0	1.12667E-08	1.22E-08
Kutzneria_albida	4.55273E-07	1.53455E-07	1.43636E-07	3.69818E-07	7.46923E-08	0	2.63077E-08	1.29538E-07	0.000000223	1.97727E-07	1.28455E-07	3.93636E-07	4.48667E-08	6.10667E-08	7.19333E-08	8.62667E-08
Kibdelosporangium_phytohabitans	2.38818E-07	3.85545E-07	0	4.69364E-07	1.93692E-07	1.59385E-07	2.86692E-07	2.67385E-07	3.52364E-07	1.34909E-07	2.07273E-07	3.30364E-07	1.95867E-07	3.788E-07	1.314E-07	9.64E-08
Actinosynnema_pretiosum	0	0	0	0	0	3.15385E-08	0	0	0	0	0	4.79091E-08	2.14667E-08	0	0	0
Actinosynnema_mirum	0	0	0	0	0	0	0	0	0	0	0	0	0	0	1.65333E-08	0
Saccharothrix_syringae	0.000000722	2.93091E-07	1.63636E-08	1.85091E-07	1.13615E-07	2.33385E-07	3.69538E-07	1.56615E-07	1.34882E-06	8.27364E-07	1.29045E-06	1.19827E-06	2.64467E-07	2.84267E-07	3.38707E-07	2.86867E-07
Saccharothrix_espanaensis	2.28545E-07	0	2.87273E-08	1.55455E-08	1.09538E-07	7.84615E-08	1.23692E-07	1.54154E-07	3.33909E-07	2.23455E-07	2.93273E-07	2.95273E-07	1.19867E-07	3.29267E-07	1.30733E-07	2.048E-07
Alloactinosynnema_sp._L-07	3.11818E-08	0	4.12727E-09	9.97273E-09	0	0	0	8.57692E-08	0	0	1.06364E-07	7.89091E-08	0	0.000000014	0	0
Allokutzneria_albata	0	0	0.000000085	2.26636E-07	8.60769E-08	1.56769E-07	3.52769E-07	4.09385E-07	2.93182E-07	8.02727E-08	2.51727E-07	3.02182E-07	4.88E-08	0.00000012	6.53733E-08	1.13667E-07
Prauserella_marina	5.17091E-07	9.36364E-09	1.08164E-07	5.14273E-07	1.58462E-07	1.24738E-06	0.000000222	3.46769E-07	3.83636E-07	0.000000357	5.53273E-07	4.85636E-07	2.60733E-07	2.546E-07	3.77867E-07	2.034E-07
Actinoalloteichus_hoggarensis	4.51727E-07	8.84545E-08	2.38127E-07	2.95455E-07	7.47692E-08	0	1.05538E-07	2.00308E-07	6.62727E-08	8.85455E-08	1.02727E-08	3.70909E-07	0	0.000000014	4.44E-09	1.20667E-07
Actinoalloteichus_hymeniacidonis	3.11818E-08	0.00000013	0	5.18182E-09	1.06154E-07	0	5.26154E-08	2.29615E-07	1.15455E-07	0	1.08727E-07	3.57273E-08	1.678E-07	2.17533E-07	1.18267E-07	8.32667E-08
Saccharopolyspora_spinosa	5.27273E-08	0	4.10909E-08	0	3.58462E-08	0	0	0	0	5.22727E-08	6.88182E-08	4.65909E-08	0	0	0	0
Saccharopolyspora_sp._ASAGF58	2.46364E-07	0.000000065	3.70909E-08	9.54545E-09	2.75385E-08	3.15385E-08	0	0	0.000000027	1.22182E-07	0	2.14545E-08	5.56E-08	4.55333E-08	7.804E-08	3.67333E-08
Saccharopolyspora_erythraea	9.25455E-08	0	7.42727E-07	2.92636E-07	2.59231E-08	8.05385E-08	0.000001059	9.01923E-07	2.14364E-07	8.26364E-08	3.58909E-07	5.30909E-08	3.05067E-07	2.60467E-07	3.296E-07	2.94533E-07
Saccharopolyspora_coralli	1.18882E-06	0.000000065	0.000000499	7.11818E-07	2.21154E-07	5.49154E-06	1.62692E-06	1.98485E-06	5.48364E-07	5.27091E-07	8.86545E-07	1.00836E-06	6.276E-07	2.73667E-07	3.704E-07	4.432E-07
Saccharopolyspora_pogona	0.000000206	2.53182E-07	2.46182E-08	1.56727E-07	1.03846E-07	8.61538E-07	1.93077E-07	8.13846E-08	0.000000167	2.56364E-07	1.66818E-07	0.000000817	8.72E-08	0	1.28667E-07	2.92E-08
Saccharomonospora_cyanea	5.42727E-08	1.04545E-08	0	3.72455E-08	2.59231E-08	0	1.94923E-07	0	0	3.62727E-08	3.95455E-08	2.14545E-08	0	0	4.83067E-08	0
Saccharomonospora_viridis	8.90909E-08	1.40364E-07	8.40909E-08	0.000000125	2.05385E-08	0	1.92308E-07	3.93846E-08	0	1.38182E-07	5.39091E-08	4.28182E-08	2.14667E-08	6.16667E-08	8.13733E-08	6.86667E-08
Saccharomonospora_azurea	1.15091E-07	0.000000047	2.13273E-07	5.50909E-08	1.23846E-07	0	3.33077E-08	7.15385E-08	0.000000053	1.32818E-07	1.70455E-07	1.45182E-07	1.91467E-07	3.11333E-08	2.2484E-07	0.00000011
Saccharomonospora_sp._31sw	6.19091E-08	3.35455E-08	0	1.93636E-08	2.75385E-08	3.83077E-08	0	0	0	3.03636E-08	0	1.47364E-07	0	3.48E-08	0	0
Saccharomonospora_marina	2.06636E-07	0.000000066	5.875E-07	7.36727E-07	0	0	0	1.97692E-07	1.21545E-07	1.05364E-07	4.59818E-07	6.26636E-07	1.202E-07	1.668E-07	0.000000213	2.044E-07
Saccharomonospora_glauca	0.000000128	3.17545E-07	1.23364E-07	0.000000099	0.000000155	5.24615E-07	2.81538E-07	2.06077E-07	0.000000305	6.76636E-07	5.46273E-07	7.55455E-07	3.16467E-07	5.23667E-07	4.782E-07	1.922E-07
Pseudonocardia_autotrophica	5.57273E-08	1.16364E-08	5.74545E-08	7.16364E-08	3.50462E-07	1.67115E-06	3.62538E-07	7.72308E-08	9.77273E-08	8.58182E-08	2.06364E-07	2.83636E-07	7.81333E-08	2.378E-07	4.67573E-07	0.000000026
Pseudonocardia_sp._EC080610-09	0	0	5.34545E-08	4.79091E-09	0	0	0	1.90769E-08	0	0	0	7.13636E-09	2.72E-08	3.09333E-08	1.65333E-08	0
Pseudonocardia_sp._Gen01	5.05818E-07	2.12273E-07	3.43545E-07	6.35727E-07	2.15846E-07	3.34769E-07	1.58462E-07	0.000000248	5.79091E-08	1.22182E-07	2.76364E-08	1.23727E-07	7.96667E-08	4.67333E-08	5.07067E-08	1.14067E-07
Pseudonocardia_sp._HH130629-09	0	2.34545E-08	1.31818E-07	1.45909E-07	1.86923E-08	0	0	2.46923E-08	4.86364E-08	1.32364E-07	2.14273E-07	9.00455E-08	2.14E-08	0.000000014	1.20867E-07	0
Pseudonocardia_sp._CGMCC_4.1532	1.43036E-06	7.32273E-07	4.044E-07	0.000000975	2.01385E-07	6.27692E-08	9.56154E-08	2.30615E-07	4.23364E-07	2.25636E-07	3.33909E-07	1.16882E-06	1.76133E-07	1.39533E-07	2.446E-07	3.562E-07
Pseudonocardia_sp._HH130630-07	1.64318E-06	3.93545E-07	1.98295E-06	1.37755E-06	1.77077E-07	3.14308E-07	1.31462E-06	1.64338E-06	5.00636E-07	8.41818E-07	7.09727E-07	7.04091E-07	1.67067E-07	2.90733E-07	1.67373E-07	0.000000194
Pseudonocardia_dioxanivorans	3.11091E-07	3.35455E-08	0	7.35182E-08	0.000000286	1.49769E-07	2.73846E-08	1.69692E-07	2.65273E-07	2.68727E-07	1.18182E-07	3.25909E-07	2.12667E-07	1.30667E-07	0.000000048	9.87333E-08
Pseudonocardia_sp._AL041005-10	1.20818E-07	0	0	1.34791E-07	6.33846E-08	7.21538E-08	0	1.14846E-07	7.18182E-08	3.89091E-08	6.66364E-08	7.07727E-08	4.46667E-08	1.592E-07	1.84533E-07	0
Amycolatopsis_japonica	0	0	0	0	0	7.21538E-08	0	0	0	0	0	0	0	0	0	0
Amycolatopsis_sp._BJA-103	0	0	0	0	0	0	0	0	0	0	0	0	0	0	6.46667E-08	0
Amycolatopsis_keratiniphila	3.11818E-08	0	0	0	0	6.47692E-08	0	2.46923E-08	3.75455E-08	3.89091E-08	0	0.000000097	0	0	0	0
Amycolatopsis_orientalis	1.36545E-07	5.05455E-08	0	1.50909E-07	2.05385E-08	1.23846E-07	0	7.72308E-08	0	5.67273E-08	9.48182E-08	5.35636E-07	6.76E-08	3.50667E-08	6.31333E-08	4.28E-08
Amycolatopsis_sp._YIM_10	0	0	6.33636E-08	4.79091E-09	0	0	0	0	0	0	0	0	6.90667E-08	0	0	0

Amycolatopsis_albispora	5.68182E-08	0	0	9.54545E-09	6.73077E-08	0	0	0	2.17909E-07	6.48182E-08	1.86091E-07	3.94545E-08	0	7.42667E-08	7.44E-08	9.33333E-09
Amycolatopsis_sp._Hca4	6.26091E-07	8.49091E-08	2.37164E-07	4.53091E-07	6.76923E-08	2.87077E-07	0	1.57692E-07	2.83182E-07	1.22545E-07	3.03727E-07	4.84182E-07	0	3.09333E-08	9.93733E-08	1.346E-07
Amycolatopsis_sp._AA4	1.32455E-07	1.66909E-07	4.09818E-08	2.30455E-07	3.58462E-08	4.66154E-08	0	0.000000291	9.42727E-08	2.73455E-07	7.56364E-08	1.39455E-07	4.96E-08	0	0	9.33333E-09
Amycolatopsis_mediterranei	2.29636E-07	0	5.48182E-08	2.59245E-07	0	4.13846E-08	5.26154E-08	1.65308E-07	5.97273E-08	9.36364E-08	2.39727E-07	5.37909E-07	1.17333E-07	0.000000061	2.0944E-07	3.86933E-07
Amycolatopsis_methanolica	1.21991E-06	0.000000411	4.08909E-07	6.06091E-07	5.56308E-07	5.53462E-07	4.02846E-07	1.05308E-06	1.05627E-06	1.47255E-06	1.45873E-06	1.40236E-06	6.30333E-07	8.12133E-07	5.16933E-07	5.944E-07
Phytohabitans_suffuscus	9.05455E-08	1.10091E-07	7.40909E-08	1.67545E-07	0	5.09231E-08	1.10385E-07	2.92077E-07	0	0	1.02727E-08	1.99091E-07	0	5.71333E-08	4.38667E-08	2.29667E-07
Phytohabitans_flavus	1.84909E-07	1.68182E-08	3.70909E-08	5.09545E-07	1.20615E-07	8.80692E-07	1.74846E-07	1.06615E-07	0.000000106	0.000000231	1.00182E-07	9.59091E-08	2.23867E-07	1.27533E-07	0.000000213	9.96E-08
Plantactinospora_sp._BC1	0	0	0	0	0	3.39154E-07	0	0	0	0	0	0	0	0	1.12667E-08	0
Plantactinospora_sp._BB1	2.98182E-08	0	0	0	0	6.27692E-08	0	0	2.64545E-08	0	3.33636E-08	0	0	0	0	0
Plantactinospora_sp._KBS50	0.00000007	1.37273E-08	1.33636E-07	9.54545E-09	0	2.06154E-07	7.92308E-08	9.63077E-08	0	0.000000081	1.46273E-07	0.00000005	2.64E-08	3.09333E-08	1.93733E-08	2.79333E-08
Verrucosispora_maris	2.56364E-08	0	0	0	0	4.27692E-08	3.77692E-08	0	0	0	0	0	0	0	2.55333E-08	0
Verrucosispora_sp._NA02020	4.43909E-07	9.78182E-08	5.75818E-08	7.19091E-08	0.000000243	9.46154E-09	3.39769E-07	1.51846E-07	2.11636E-07	2.41818E-07	6.47909E-07	3.89455E-07	0.000000117	2.554E-07	1.42667E-07	3.466E-07
Salinispora_arenicola	1.57818E-07	7.66364E-08	0	1.37182E-07	0	1.03692E-07	1.40846E-07	5.42308E-08	1.39273E-07	3.89091E-08	0	2.08818E-07	2.14667E-08	5.71333E-08	4.44E-09	2.92E-08
Salinispora_tropica	5.34364E-07	1.94545E-07	2.04909E-08	2.74273E-07	0	4.41538E-07	2.63077E-08	2.65077E-07	2.06182E-07	3.89091E-08	9.64545E-08	1.62455E-07	7.25333E-08	2.18067E-07	4.44E-08	1.01133E-07
Micromonospora_coriariae	0	0	0	0	0	2.13846E-08	0	0	0	0	0	0	0	0	0	0
Micromonospora_coxensis	0	0.000000065	0	0	0	3.15385E-08	0	0	0	0	0	0	0	0	0	3.72E-08
Micromonospora_sp._WMMC415	0	0	0	0	1.09231E-07	0	0	2.46923E-08	0	0	0	0	0.000000064	1.09733E-07	0	0
Micromonospora_narathiwatensis	0	0	0	0	0	0	0	0	0	0	0	3.16364E-08	0	0	0	0
Micromonospora_siamensis	2.30909E-08	0	0	0	3.15385E-08	7.96923E-08	0	0	8.20909E-08	0	0	1.42727E-08	0	0	0	0
Micromonospora_sp._L5	3.11818E-08	0	0	0	0	0	0	2.46923E-08	3.98182E-08	0	0	0	0	0	1.12667E-08	0
Micromonospora_sagamiensis	1.08455E-07	0	0	0	3.15385E-08	0	0	1.90769E-08	5.79091E-08	1.56364E-07	6.88182E-08	3.16364E-08	0	0	0	0
Micromonospora_aurantiaca	0	0	0	0	0	0	0	0	0	0	1.02727E-08	0	0	0	0	0
Micromonospora_craniellae	0	0.000000065	0	1.47273E-08	0	6.59538E-07	3.33077E-08	1.16769E-07	0	0	3.07273E-08	1.42727E-08	0	0	0	4.60667E-08
Micromonospora_terminaliae	1.48818E-07	0	0	1.47609E-07	3.15385E-08	2.16923E-08	5.26154E-08	1.16154E-07	2.75455E-08	2.04273E-07	1.06364E-07	4.65909E-08	0	6.18E-08	6.82667E-08	9.33333E-09
Micromonospora_purpureochromogenes	1.02909E-07	0.000000065	3.70909E-08	8.09091E-08	2.59231E-08	3.15385E-08	9.88462E-08	0	4.45455E-08	9.77273E-08	0	1.07455E-07	6.94E-08	7.78667E-08	6.86E-08	6.33333E-08
Micromonospora_sp._28ISP2-46	2.17182E-07	8.86364E-09	0.000000222	3.58455E-07	1.71538E-07	3.15385E-08	1.71846E-07	1.34154E-07	6.45455E-08	2.09909E-07	1.06364E-07	2.96909E-07	4.28667E-08	0.000000168	1.496E-07	0.000000154
Micromonospora_auratinigra	1.58182E-08	0	0	0.000000062	1.21308E-07	0	0	0	0	0	1.30909E-07	0.000000132	2.72E-08	0.000000084	8.13733E-08	1.98667E-08
Micromonospora_echinaurantiaca	5.71818E-08	1.46818E-07	1.63636E-08	3.47273E-08	0	0	8.81538E-08	0.000000042	3.98182E-08	9.49091E-08	0	7.13636E-09	2.44E-08	0.000000041	0	0
Micromonospora_sp._B006	2.30909E-08	0	0	0	0	2.52308E-08	0	0	0	1.04545E-07	8.02727E-08	0	0	0	0	0
Micromonospora_echinofusca	1.04364E-07	0	5.34545E-08	3.35727E-07	0	1.54769E-07	0	0.000000042	1.85818E-07	0	2.35273E-07	1.14318E-07	2.72E-08	0	5.51333E-08	1.86E-08
Micromonospora_sp._HM134	2.96364E-08	2.11818E-08	0	0	3.58462E-08	0	5.74615E-08	0	2.14545E-07	0	0.000000039	1.04636E-07	0	0.000000014	3.41733E-08	0
Micromonospora_rifamycinica	3.69091E-08	0	1.77273E-08	3.42727E-08	2.05385E-08	0	5.36923E-08	6.85231E-07	0	5.65455E-08	2.66636E-07	3.87727E-08	8.12E-08	0	5.244E-08	2.772E-07
Micromonospora_krabiensis	0	0	1.77273E-08	0	5.85385E-08	7.21538E-08	3.11538E-08	5.22308E-08	3.98182E-08	6.08182E-08	0	7.51818E-08	7.68E-08	6.12667E-08	9.68E-08	6.62667E-08
Micromonospora_zamorensis	6.25455E-08	0.00000026	6.37273E-08	2.08273E-07	0	5.95385E-07	8.59231E-08	1.08538E-07	0	1.02636E-07	0	0	6.08E-08	0	0	0
Micromonospora_sp._WMMA2032	2.56364E-08	0	4.12727E-09	1.94427E-07	0	1.01692E-07	2.63077E-08	0	0	0	0	2.65182E-07	6.35333E-08	1.77533E-07	2.534E-07	6.75333E-08
Micromonospora_viridifaciens	1.15091E-07	1.91182E-07	7.82182E-08	2.31636E-07	1.00231E-07	1.66769E-07	1.31538E-07	0.000000157	4.46909E-07	2.09091E-07	0	3.40091E-07	1.06267E-07	1.042E-07	2.55333E-08	1.86667E-08
Micromonospora_carbonacea	0.000000246	0	8.25455E-09	4.57545E-07	0	3.72308E-07	0	0	2.62545E-07	1.96545E-07	1.57091E-07	5.57273E-07	1.144E-07	3.26667E-08	2.69067E-08	1.60867E-07
Micromonospora_chokoriensis	1.58182E-08	0	8.25455E-09	0	3.58462E-08	0	0	0	0	0.000000047	8.02727E-08	0	2.08E-08	0	2.97333E-08	0
Micromonospora_echinospora	2.56364E-08	0	0	9.54545E-09	2.75385E-08	4.85462E-07	6.23077E-08	0.000000119	9.20909E-08	2.85455E-08	0	2.08045E-07	2.14E-08	1.188E-07	2.55333E-08	0
Micromonospora_inositola	1.98545E-07	7.77455E-07	1.54127E-07	9.24545E-08	7.17692E-08	0	7.40769E-08	0	6.59091E-08	0	1.80273E-07	0.000000142	2.64667E-08	1.04667E-07	2.97333E-08	0
Actinoplanes_sp._N902-109	1.52636E-07	3.50909E-08	8.25455E-09	1.37609E-07	1.13308E-07	1.41062E-06	4.58077E-07	3.19923E-07	8.72727E-08	1.16545E-07	3.24455E-07	0.000000154	3.82533E-07	0.00000005	1.63933E-07	9.99333E-08
Actinoplanes_friuliensis	2.11909E-07	8.66818E-08	1.01027E-07	1.21545E-07	3.15385E-08	0.000000855	0.000000094	3.03615E-07	1.15455E-07	2.29455E-07	1.74818E-07	3.53909E-07	8.97333E-08	2.84867E-07	3.41733E-08	4.13933E-07
Actinoplanes_derwentensis	2.16545E-07	3.62545E-07	1.07982E-07	5.08636E-07	0	7.42146E-06	1.52692E-07	1.01077E-07	1.99818E-07	1.22182E-07	4.69364E-07	6.77727E-07	2.23733E-07	3.294E-07	4.93533E-07	2.898E-07
Actinoplanes_teichomyceticus	5.58364E-07	0.000000065	1.00455E-07	3.80455E-07	1.37154E-07	8.55385E-07	3.17692E-07	2.03077E-07	2.54636E-07	3.70545E-07	7.82909E-07	4.26818E-07	1.206E-07	2.31533E-07	3.172E-07	1.70533E-07
Actinoplanes_missouriensis	1.17182E-07	2.47818E-07	3.70909E-08	2.01273E-07	2.05385E-08	5.86462E-07	1.21923E-07	0	8.08182E-08	2.35455E-07	1.10273E-07	3.11091E-07	5.04E-08	1.87067E-07	9.43333E-08	1.054E-07
Actinoplanes_sp._OR16	3.43727E-07	7.42727E-08	2.04909E-08	6.85455E-07	1.81538E-07	9.13846E-07	0.000000458	1.28692E-07	0.000000471	7.75455E-07	2.84182E-07	6.54273E-07	2.22667E-07	5.77467E-07	3.46267E-07	3.326E-07
Winkia_sp._C64	2.96364E-08	0	5.74545E-08	5.18182E-09	9.09231E-08	4.69231E-08	0	0	2.64545E-08	0.000000065	0	8.29091E-08	0	8.82667E-08	9.12667E-08	9.33333E-09
Pauljensenia_hongkongensis	8.37455E-07	1.07791E-06	1.26909E-07	5.99182E-07	0.000000256	6.87923E-07	2.46692E-07	6.35308E-07	5.14909E-07	4.11636E-07	6.73818E-07	9.37636E-07	3.38333E-07	3.08533E-07	2.13733E-07	2.77133E-07
Actinobaculum_sp._313	1.50955E-06	2.93909E-07	1.41436E-07	2.86227E-06	6.39077E-07	2.95623E-06	4.44085E-06	3.29023E-06	9.32818E-07	1.21418E-06	9.81636E-07	7.88909E-07	3.66867E-07	2.59267E-07	3.212E-07	5.288E-07
Mobiluncus_curtisii	9.01364E-07	5.15182E-07	0.000000384	8.10364E-07	3.32308E-07	0	2.70231E-07	6.93308E-07	7.36545E-07	4.27455E-07	4.47545E-07	1.53555E-06	1.55533E-07	4.16667E-07	3.71467E-07	3.142E-07
Arcanobacterium_phocae	3.11818E-08	1.04545E-08	8.75455E-09	9.54545E-09	7.61538E-08	6.43077E-07	0	1.46923E-08	0	0	0	0	0	0	0	1.98667E-08
Arcanobacterium_sp._2701	1.58182E-08	9.36364E-09	4.12727E-09	2.62973E-07	5.76154E-08	0	5.26154E-08	0	0.000000084	1.04545E-07	9.99091E-08	1.79818E-07	2.37333E-08	0.000000033	1.33333E-08	0
Arcanobacterium_haemolyticum	9.04545E-08	3.00273E-07	1.65909E-07	0.000000122	6.51538E-08	4.13846E-08	1.31538E-07	1.26615E-07	6.48182E-08	0	9.48182E-08	1.87091E-07	4.46667E-08	8.36E-08	5.93333E-08	5.82667E-08
Arcanobacterium_sp._JY-X040	1.32636E-07	3.74636E-07	1.70727E-07	3.97818E-07	0.000000352	2.06923E-07	2.67846E-07	2.99077E-07	3.54273E-07	3.05364E-07	8.47273E-07	3.84727E-07	7.23333E-08	4.15733E-07	3.23533E-07	1.902E-07
Actinotignum_schaalii	1.37236E-06	4.14636E-07	3.19909E-07	1.06182E-06	5.02923E-07	2.66023E-06	1.08054E-06	1.74992E-06	6.42545E-07	8.74545E-07	1.17109E-06	1.51382E-06	5.23933E-07	9.284E-07	8.27733E-07	8.056E-07
Flaviflexus_salsibiostraticola	3.11818E-08	0	0	7.04182E-07	0	9.38462E-07	1.04546E-06	9.28769E-07	3.19909E-07	1.17091E-07	4.32909E-07	2.24591E-07	5.61333E-08	3.09333E-08	3.88667E-08	0.000000104
Flaviflexus_sp._dk850	2.72545E-07	1.68182E-08	0	3.39091E-08	2.26923E-08	0	1.90769E-07	6.82308E-08	2.75455E-08	1.33091E-07	0.000000218	0.000000107	0.000000111	4.80667E-08	1.65333E-08	1.92333E-07
Flaviflexus_sp._H23T48	8.82636E-07	1.68182E-08	1.293E-07	1.06727E-07	4.74385E-07	0	4.33846E-08	1.58846E-07	9.01727E-07	1.08391E-06	8.57727E-07	1.62555E-06	1.0466E-06	9.502E-07	6.23667E-07	7.37733E-07
Schaalia_odontolytica	1.39636E-07	0	8.75455E-09	2.23909E-07	0.000000061	9.02308E-08	4.33846E-08	3.26154E-07	0.000000187	2.85455E-08	1.16636E-07	3.09091E-07	0.000000136	1.894E-07	0.000000146	1.72533E-07
Schaalia_sp._JY-X169	3.11818E-08	1.24409E-07	9.45455E-08	1.37364E-07	2.05385E-08	1.61769E-07	6.44615E-08	4.84615E-08	5.91818E-08	1.25182E-07	2.76364E-08	5.35455E-07	0.000000053	1.04667E-07	2.83733E-08	4.02E-08
Schaalia_meyeri	8.88545E-07	1.36273E-07	0.000000827	6.91818E-07	3.22385E-07	0	9.52308E-08	5.23615E-07	8.29636E-07	2.89455E-07	7.07636E-07	1.29864E-06	4.88E-08	1.958E-07	1.85507E-07	3.05933E-07
Schaalia_cardiffensis	2.67855E-06	1.01864E-06	3.94636E-07	1.53145E-06	1.83277E-06	1.43462E-06	4.39754E-06	3.35885E-06	2.92391E-06	1.87736E-06	3.46545E-06	3.20491E-06	1.12753E-06	1.56007E-06	0.000002294	1.53873E-06
Actinomyces_naeslundii	2.96364E-08	0	0	2.43636E-08	0	0	0	0	0	0	0	0	0	0	0	0
Actinomyces_gaoshouyii	0	0	0	0	0	0	0	0	0	5.22727E-08	0	0	0	0	7.36E-08	0
Actinomyces_pacaensis	0	0	0	1.32182E-07	6.46923E-08	2.19231E-07	5.26154E-08	1.21769E-07	0	0	0	1.34455E-07	0	1.58E-08	2.09733E-08	4.76667E-08
Actinomyces_sp._Z16	1.86182E-07	0	0	1.65091E-07	3.58462E-08	2.53538E-07	1.76692E-07	3.61231E-07	2.52636E-07	3.66364E-07	6.62727E-07	3.52545E-07	1.73667E-07	8.71333E-08	1.33333E-08	1.83267E-07
Actinomyces_sp._Chiba101	1.08545E-07	2.00455E-07	0	1.05091E-07	2.05385E-08	6.76923E-08	0	3.93846E-08	5.79091E-08	1.33091E-07	3.77273E-08	2.02455E-07	1.89133E-07	1.40467E-07	1.87733E-07	0.000000118
Actinomyces_tangfeifanii	2.56364E-08	0	0	2.27091E-07	7.16923E-08	7.21538E-08	1.41154E-07	0.000000088	2.64545E-08	0	2.12091E-07	0.000000211	1.45067E-07	0	7.43333E-08	1.41333E-07
Actinomyces_sp._oral_taxon_171	1.95545E-07	3.28182E-08	0	6.11818E-08	1.85538E-07	0	2.73846E-08	8.28462E-08	2.19727E-07	5.65455E-08	1.10818E-07	0.000000243	4.51333E-08	0.000000014	1.54733E-07	5.70667E-08
Actinomyces_sp._432	0.00000033	2.19273E-07	0.000000315	2.64818E-07	0.000000119	7.83538E-07	8.37692E-08	3.92846E-07	1.46364E-07	2.71636E-07	1.19273E-07	1.79091E-07	2.24E-08	6.18E-08	6.30667E-08	5.38667E-08
Actinomyces_sp._2129	6.67091E-07	0	3.55273E-07	8.40636E-07	2.12154E-07	2.16923E-08	1.99462E-07	9.98692E-07	0.000000997	6.89364E-07	5.94364E-07	8.53364E-07	3.99267E-07	3.45067E-07	3.726E-07	5.65133E-07
Actinomyces_sp._oral_taxon_169	0	1.17273E-07	2.51182E-08	2.21455E-07	2.05385E-08	8.63077E-08	2.63077E-08	3.37692E-08	0.000000064	1.40818E-07	7.85455E-08	1.23091E-07	2.64667E-08	4.64667E-08	5.35333E-08	0
Actinomyces_qiguomingii	2.43873E-06	2.59091E-07	7.91855E-07	1.87927E-06	2.40385E-07	0.000000712	1.74577E-06	1.84208E-06	1.07191E-06	1.67718E-06	1.63945E-06	1.66255E-06	4.46933E-07	6.53467E-07	9.26533E-07	5.34133E-07
Actinomyces_howellii	0.000000213	2.06182E-07	7.62182E-08	4.89091E-08	4.16769E-07	3.11538E-08	2.48923E-07	3.64385E-07	2.04636E-07	3.62727E-08	3.30636E-07	4.09545E-07	1.60133E-07	1.50667E-07	2.754E-07	1.35267E-07
Actinomyces_israelii	1.54545E-07	1.27727E-07	4.12727E-09	3.55182E-08	9.83846E-08	0	9.07692E-08	1.51154E-07	2.10636E-07	1.60091E-07	3.05364E-07	2.04318E-07	1.02267E-07	5.60667E-08	1.218E-07	1.88667E-07
Actinomyces_slackii	1.18455E-06	1.01573E-06	5.99664E-07	1.18364E-06	2.37615E-07	1.03785E-06	6.25385E-07	6.52769E-07	8.17545E-07	9.14091E-07	9.22364E-07	5.85818E-07	5.456E-07	3.072E-07	4.42067E-07	5.514E-07
Actinomyces_radicidentis	0.00000049	7.54727E-07	0.000000229	5.97545E-07	3.92308E-07	4.03462E-07	4.42154E-07	0.000001563	7.41545E-07	5.94091E-07	6.97545E-07	7.05364E-07	2.75733E-07	3.19267E-07	5.156E-07	7.33667E-07
Actinomyces_sp._oral_taxon_897	4.49727E-07	1.57091E-07	6.63364E-08	2.03727E-07	1.36769E-07	9.02308E-08	1.21462E-07	6.04692E-07	1.56727E-07	0.00000009	2.09182E-07	2.47727E-07	5.08E-08	0	2.20933E-07	8.01333E-08
Actinomyces_sp._zg-325	0	2.70909E-08	4.52727E-08	9.39091E-08	0	0	0	0	0	0	0	7.44545E-08	0	0	4.38667E-08	0
Actinomyces_viscosus	0	0	3.30455E-08	2.87273E-08	0	0	0	0	2.16364E-08	0.000000047	8.02727E-08	7.13636E-09	0	6.80667E-08	8.86667E-09	6.86667E-08
Actinomyces_sp._ZJ34	5.41818E-08	0.000000112	0	3.74818E-07	8.36154E-08	1.44308E-07	0	0.000000161	3.07909E-07	2.85455E-08	1.85091E-07	2.65364E-07	1.33067E-07	3.32067E-07	3.224E-08	3.67333E-08
Actinomyces_sp._ZJ750	8.81818E-08	0	0.000000035	2.30091E-07	5.20769E-08	8.56154E-08	0	1.90769E-08	1.22455E-07	0	2.12727E-07	3.79955E-07	1.86667E-08	1.84467E-07	6.91733E-08	4.76667E-08
Actinomyces_sp._dk561	4.23818E-07	2.60364E-07	0	2.01364E-07	1.49923E-07	3.88538E-07	1.53538E-07	1.39231E-07	0.000000465	2.26818E-07	1.01409E-06	3.67182E-07	7.12667E-08	2.924E-07	2.9024E-07	2.17667E-07
Actinomyces_sp._299	6.15091E-07	3.66227E-07	1.93545E-07	7.91636E-07	2.16308E-07	2.58177E-06	4.42462E-07	6.34615E-07	3.02818E-07	1.95727E-07	2.73545E-07	6.29727E-07	1.14067E-07	1.14267E-07	2.01267E-07	4.86867E-07
Actinomyces_sp._oral_taxon_414	1.38727E-07	1.78545E-07	1.43636E-07	4.25727E-07	2.09385E-07	4.18077E-07	5.17231E-07	1.07308E-07	6.14818E-07	1.99818E-07	3.69636E-07	5.46091E-07	2.28467E-07	2.72867E-07	0.000000583	4.266E-07
Trueperella_bialowiezensis	0.00000176	3.75455E-08	3.18364E-07	0	4.83846E-07	1.96154E-07	1.01962E-06	1.20662E-06	1.87973E-06	3.13336E-06	0.000003239	1.08364E-06	6.62733E-07	2.40933E-07	1.77333E-07	1.20133E-06
Trueperella_sp._19M2397	1.53636E-07	2.46727E-07	1.63636E-08	4.20455E-07	2.08769E-07	2.69538E-07	2.20769E-07	3.56538E-07	3.16182E-07	1.74455E-07	1.15364E-07	5.51455E-07	1.722E-07	1.53733E-07	1.41373E-07	0.000000194
Trueperella_pyogenes	1.70318E-05	0.000010222	3.64403E-06	1.65209E-05	2.12738E-05	1.82577E-05	1.16785E-05	1.25138E-05	2.02245E-05	1.86636E-05	1.69955E-05	2.80182E-05	0.000017568	1.99033E-05	1.60013E-05	1.61013E-05
Kribbella_qitaiheensis	0.00000007	0	0	0	0	8.53846E-08	0	0	0	0	0	2.80091E-07	2.72E-08	0	0	1.98667E-08
Kribbella_flavida	6.08455E-07	5.30727E-07	2.31636E-07	5.92727E-08	2.13538E-07	1.78769E-07	1.39923E-07	1.03462E-07	3.42182E-07	6.42727E-08	4.68545E-07	6.96727E-07	2.63533E-07	2.21267E-07	1.16533E-07	1.684E-07
Pimelobacter_simplex	2.56364E-08	6.28636E-08	1.774E-07	0.000000242	0.000000092	3.18308E-07	1.14308E-07	2.46923E-08	0.000000217	5.22727E-08	8.93636E-08	1.89727E-07	1.538E-07	8.58E-08	1.14933E-07	1.86E-08
Friedmanniella_luteola	1.63636E-07	0	2.04909E-08	2.38636E-07	1.05154E-07	2.38538E-07	6.23077E-08	2.47692E-08	3.03909E-07	0.000000028	3.26909E-07	2.52818E-07	4.81333E-08	7.60667E-08	1.37867E-07	9.89333E-08
Friedmanniella_sagamiharensis	1.95273E-07	5.39818E-07	1.11182E-07	3.03727E-07	2.75385E-08	2.23231E-07	3.21154E-07	1.40385E-07	2.78091E-07	8.85455E-08	9.42727E-08	2.94727E-07	2.14E-08	0	2.39333E-08	2.88667E-08
Marmoricola_scoriae	2.33727E-07	1.91727E-07	2.73273E-07	1.12336E-07	1.52923E-07	3.42462E-07	1.11154E-07	2.55077E-07	0.000000098	7.51818E-08	5.95455E-08	1.82545E-07	0.000000093	5.88667E-08	0.000000187	1.34067E-07
Mumia_sp._ZJ1417	8.58182E-08	0	8.30909E-08	1.11909E-07	2.88462E-08	3.43077E-07	0	1.90769E-08	7.53636E-08	6.25455E-08	2.16182E-07	4.92727E-08	4.16E-08	7.68E-08	3.88667E-08	1.09067E-07
Micropruina_glycogenica	7.13636E-07	3.38182E-08	1.21455E-06	1.83682E-06	6.95308E-07	2.03392E-06	0.000003247	2.78238E-06	6.75636E-07	6.08636E-07	5.45818E-07	5.65455E-07	0.000000465	8.51267E-07	3.642E-07	6.95133E-07
Aeromicrobium_choanae	0	0	0	0	0	0	0	0	0	3.03636E-08	0	0	0	0	0	0
Aeromicrobium_sp._MF47	0	0	0	0	0	0	0	1.90769E-08	0	0	0	0	0	2.98E-08	0	3.67333E-08
Aeromicrobium_erythreum	2.70909E-08	0	0	1.43636E-08	0	0	2.63077E-08	2.56462E-07	2.64545E-08	0	0	0.000000219	0	0.000000014	1.12667E-08	4.66667E-08
Aeromicrobium_sp._592	0.000000047	0.000000065	3.70909E-08	0	0	0	0	0.000000062	0	0	6.68182E-08	1.44091E-07	7.18667E-08	0.00000007	0	1.98667E-08
Aeromicrobium_marinum	3.14818E-07	3.83455E-07	0.000000464	1.75818E-07	0.000000065	2.38769E-07	3.73154E-07	1.46846E-07	0.000000199	1.39364E-07	1.26182E-07	2.21364E-07	4.22E-08	4.98E-08	7.624E-08	2.87733E-07
Aeromicrobium_sp._zg-629	1.04727E-07	3.09909E-07	1.52309E-07	1.99727E-07	2.88462E-08	0	1.83846E-07	1.86462E-07	5.79091E-08	2.85455E-08	0	5.08545E-07	0	0	1.12667E-08	9.12667E-08
Aeromicrobium_sp._A1-2	1.74727E-07	1.97273E-08	3.70909E-08	5.92636E-07	0	0	0	4.93846E-08	3.98182E-08	0	2.41091E-07	9.98182E-08	0	1.09467E-07	2.08067E-07	1.142E-07
Actinopolymorpha_singaporensis	1.25673E-06	2.37636E-07	5.48182E-08	3.86427E-07	2.46923E-07	5.19462E-07	5.42077E-07	0.000001894	6.67364E-07	6.39545E-07	6.94727E-07	9.89545E-07	3.938E-07	3.406E-07	3.374E-07	9.40667E-07
Nocardioides_sp._S-713	2.56364E-08	0.000000065	0	0	0	3.15385E-08	0	0.000000011	5.50909E-08	0	0	0	0	0	0	0
Nocardioides_mesophilus	5.57273E-08	0	0	2.47791E-07	0	3.32769E-07	3.77692E-08	0	0	0	2.76364E-08	0.000000035	1.26867E-07	4.88E-08	3.27333E-08	0.000000043
Nocardioides_sp._S-1144	7.37273E-08	2.72727E-08	0	0.000000124	6.19231E-08	1.37308E-07	0	0	0	0.000000047	0.000000048	0	0	1.58E-08	0	5.88667E-08
Nocardioides_sp._KUDC_5002	0	0	0	5.16364E-08	5.74615E-08	3.15385E-08	3.11538E-08	4.32308E-08	0	8.02727E-08	7.90909E-08	1.42727E-08	0	4.69333E-08	7.45067E-08	4.76667E-08
Nocardioides_marinisabuli	0.000000172	4.39091E-08	3.70909E-08	1.94636E-07	2.05385E-08	3.15385E-08	5.74615E-08	2.47769E-07	2.19182E-07	1.32545E-07	1.02727E-08	3.13364E-07	1.22867E-07	6.51333E-08	0.000000194	9.33333E-09
Nocardioides_sp._dk884	2.70909E-08	0	7.40909E-08	4.40909E-08	0	2.16923E-08	2.73846E-08	1.90769E-08	0	0	0	0	2.37333E-08	3.15333E-08	4.38667E-08	0
Nocardioides_sp._zg-579	0	1.68182E-08	6.55455E-08	4.72727E-08	2.05385E-08	2.91538E-07	2.63077E-08	0	3.94545E-08	5.22727E-08	0	7.13636E-09	0	4.76667E-08	7.464E-08	3.40667E-08
Nocardioides_sp._CF8	5.42727E-08	2.71818E-07	0.000000046	7.48182E-08	3.58462E-08	0	0	1.35231E-07	0	0	0	7.89091E-08	0	4.67333E-08	1.65333E-08	0
Nocardioides_sp._603	8.80909E-08	7.87273E-08	5.93636E-08	2.71545E-07	0.000000143	7.71538E-08	0	5.03846E-08	7.61818E-08	2.94909E-07	2.09727E-07	5.58636E-07	1.48333E-07	1.35067E-07	1.53973E-07	1.98667E-08
Nocardioides_sp._WS12	9.26364E-08	0	2.47273E-08	2.41545E-08	1.01923E-07	9.26462E-07	2.63077E-08	6.01538E-08	1.87273E-07	0	0	2.79455E-07	4.27333E-08	1.042E-07	6.46667E-08	8.34E-08
Nocardioides_sp._dk3136	1.15182E-07	0	0	1.03636E-08	0	6.27692E-08	0	0	3.75455E-08	0	0	0	0	0	0	9.41333E-08
Nocardioides_sp._MC1495	1.18636E-07	0	2.96364E-08	1.22364E-07	5.50769E-08	3.09077E-07	1.82231E-07	0.000000011	0	8.02727E-08	0	4.79091E-08	0	3.50667E-08	7.59333E-08	1.11133E-07
Nocardioides_sp._HDW12B	6.84545E-08	5.47273E-08	0	3.39091E-08	0	0.000000283	3.33077E-08	2.88692E-07	0	0	0	0	2.24E-08	7.97333E-08	0	0.000000107
Nocardioides_sp._ZJ1313	5.69091E-08	9.20909E-08	7.97273E-08	3.13091E-07	0	0	6.41538E-08	8.87692E-08	0	0	6.88182E-08	4.59091E-08	2.91333E-08	1.58E-08	2.55333E-08	6.33333E-08
Nocardioides_baekrokdamisoli	6.62091E-07	1.1685E-06	2.22273E-07	3.99455E-07	2.16385E-07	0	0	4.96077E-07	1.75136E-06	0.000000527	0.000000618	1.09764E-06	2.56867E-07	0.000000324	3.74467E-07	6.69467E-07
Nocardioides_sp._HY056	4.83364E-07	0	0.000000222	5.36364E-08	0	0	6.44615E-08	4.46462E-07	3.74727E-07	1.03727E-07	3.33636E-08	6.08091E-07	2.94667E-08	3.50667E-08	4.49067E-08	2.92E-08
Nocardioides_sp._JS614	0	1.01818E-07	1.63636E-08	8.64545E-08	3.58462E-08	2.95385E-08	0	6.23077E-08	1.11545E-07	0.000000028	1.40091E-07	7.07273E-08	0	0	2.01333E-08	0
Nocardioides_euryhalodurans	3.63545E-07	0	3.70909E-08	2.99727E-07	1.07923E-07	8.26385E-07	3.53308E-07	4.65692E-07	2.16364E-08	2.85545E-07	0.000000596	4.73818E-07	4.81333E-08	1.55867E-07	8.34667E-08	2.75267E-07
Nocardioides_seonyuensis	7.99091E-08	2.84545E-08	9.18455E-08	9.35182E-08	0	1.26769E-07	3.33077E-08	7.40769E-08	8.67273E-08	0	8.71818E-08	9.82727E-08	0	0	8.36E-08	0
Nocardioides_ungokensis	2.03727E-07	1.39455E-07	1.19309E-07	5.50636E-08	0	4.73846E-08	3.11538E-08	7.87692E-08	2.75455E-08	3.62727E-08	6.88182E-08	1.02591E-07	0.000000088	1.58E-08	0	0
Nocardioides_humi	1.48545E-07	1.86545E-07	2.04909E-08	1.21336E-07	1.22154E-07	9.46154E-09	1.36077E-07	1.03538E-07	9.54545E-08	0.00000013	9.42727E-08	1.24182E-07	7.02667E-08	4.96667E-08	1.24867E-07	0
Nocardioides_daphniae	5.77091E-07	0.000000065	4.60945E-07	1.26482E-06	2.19769E-07	6.76923E-08	5.68923E-07	0.000000473	7.35818E-07	5.75455E-07	5.04364E-07	3.60364E-07	4.00067E-07	3.45667E-07	3.922E-07	5.782E-07
Nocardioides_dokdonensis	1.76909E-07	1.04545E-07	1.15073E-07	2.52545E-07	2.60538E-07	0	1.91308E-07	4.68538E-07	1.46909E-07	1.74727E-07	0.000000061	3.90909E-07	7.00667E-08	1.096E-07	1.77333E-07	4.70267E-07
Nocardioides_sp._JQ2195	3.16364E-07	0.000000298	1.89527E-07	1.80182E-07	1.30846E-07	1.15615E-07	0	2.29308E-07	0.000000106	8.85455E-08	1.56182E-07	1.70091E-07	6.88E-08	1.58E-08	1.86933E-07	1.11867E-07
Nocardioides_sp._SB3-45	1.91818E-07	7.45455E-08	1.00073E-07	5.18182E-09	0	0	8.11538E-08	5.69231E-08	0.00000015	1.01636E-07	2.03182E-07	4.05909E-07	9.30667E-08	1.77467E-07	1.436E-07	3.72E-08
Nocardioides_anomalus	7.37273E-08	1.39545E-07	1.77273E-08	1.36455E-07	1.52385E-07	1.51231E-07	8.61538E-08	4.94615E-08	1.02364E-07	9.11818E-08	0.0000001	2.93091E-07	1.018E-07	1.91267E-07	3.964E-08	4.76667E-08
Nocardioides_sp._HDW12A	5.57273E-08	0	0	9.37273E-08	0	2.13846E-08	3.77692E-08	2.46923E-08	0	0	0	5.64545E-08	0	5.06E-08	8.86667E-09	7.55333E-08
Nocardioides_sp._78	1.03082E-06	2.08182E-08	4.53455E-08	2.91727E-07	1.34308E-07	2.91385E-07	5.74615E-08	9.26923E-08	3.16455E-07	5.22727E-08	2.52636E-07	2.29091E-07	1.37867E-07	1.67667E-07	2.022E-07	1.628E-07
Auraticoccus_monumenti	2.64818E-07	7.66364E-08	0	8.06364E-08	1.39231E-07	1.15808E-06	2.43846E-07	2.74308E-07	2.72727E-07	1.16273E-07	1.45909E-07	2.85455E-08	8.03333E-08	6.36E-08	7.44E-08	6.87333E-08
Propioniciclava_sp._HDW11	3.97545E-07	2.93545E-07	0	3.50909E-07	1.72385E-07	1.66231E-07	5.86692E-07	2.92769E-07	3.57636E-07	7.11909E-07	4.98818E-07	9.33364E-07	0	1.21467E-07	4.228E-07	2.05067E-07
Pseudopropionibacterium_propionicum	1.34482E-06	1.30545E-06	3.20182E-07	1.25955E-06	6.14615E-07	8.55923E-07	3.84769E-07	1.15123E-06	9.34364E-07	5.97091E-07	1.42718E-06	1.53745E-06	4.73333E-07	5.528E-07	1.03353E-06	4.572E-07
Microlunatus_sp._Gsoil_973	1.12818E-07	0.000000047	7.40909E-08	2.39091E-08	1.52615E-07	7.58385E-07	2.46615E-07	4.49385E-07	1.71091E-07	9.92727E-08	4.11364E-07	0.000000591	1.36267E-07	2.13467E-07	1.822E-07	2.372E-07
Microlunatus_sp._KUDC0627	3.99455E-07	0.000000126	1.54582E-07	3.91455E-07	4.62308E-08	1.79231E-08	6.66154E-08	7.31538E-08	1.24727E-07	1.91182E-07	4.56182E-07	1.03727E-07	9.56E-08	3.32667E-08	1.93733E-08	2.292E-07
Microlunatus_soli	2.02427E-06	3.86818E-07	2.14027E-06	1.49891E-06	1.63385E-07	3.67231E-07	1.72777E-06	1.49238E-06	4.12273E-07	7.70364E-07	1.76436E-06	1.27436E-06	2.19067E-07	2.78133E-07	3.07467E-07	5.188E-07

Microlunatus_phosphovorus	1.17818E-07	1.68182E-08	0	4.79091E-09	0	0	0	0	0	3.62727E-08	0	1.59364E-07	0	3.48E-08	2.97333E-08	0
Raineyella_sp._CBA3103	8.42727E-07	7.99455E-07	2.93727E-07	4.09545E-07	1.91231E-07	1.89231E-08	4.25385E-07	3.62385E-07	2.17091E-07	1.87818E-07	4.02182E-07	6.72636E-07	9.95333E-08	0.000000187	1.742E-07	3.51933E-07
Tessaracoccus_defluvii	0.000000521	1.40455E-07	7.58545E-08	8.72091E-07	1.04769E-07	7.22308E-08	3.49462E-07	2.81308E-07	1.78273E-07	4.55182E-07	3.50091E-07	6.09091E-07	8.27333E-08	5.24E-08	3.34067E-07	1.74533E-07
Tessaracoccus_timonensis	9.05455E-08	1.91864E-07	5.89455E-08	1.39364E-07	2.17231E-07	1.93538E-07	3.58946E-06	2.50715E-06	0.000000027	0	2.76364E-08	2.36273E-07	4.99733E-07	4.21267E-07	0.000000175	0.000000392
Tessaracoccus_flavus	4.08818E-07	4.13273E-07	4.84909E-07	2.33545E-07	2.46385E-07	0	1.44692E-07	2.38769E-07	6.81818E-07	3.53818E-07	4.51545E-07	4.04909E-07	2.96067E-07	6.65067E-07	4.49773E-07	1.89733E-07
Tessaracoccus_aquimaris	1.59764E-06	8.00182E-07	6.42545E-07	1.55782E-06	3.20308E-07	0	3.11462E-07	5.83077E-07	1.07355E-06	9.31182E-07	7.05727E-07	5.21364E-07	6.42533E-07	5.67667E-07	3.394E-07	2.756E-07
Tessaracoccus_flavescens	5.95727E-07	5.52636E-07	1.07545E-07	6.31909E-07	2.64077E-07	5.06154E-07	1.25154E-07	4.10692E-07	3.81091E-07	4.40455E-07	4.94727E-07	8.32545E-07	1.574E-07	2.77067E-07	8.12E-08	1.558E-07
Propionimicrobium_sp._Marseille-P3275	5.82782E-06	8.66845E-06	1.42559E-06	3.55136E-06	2.42208E-06	4.01523E-06	4.18923E-06	1.79977E-06	4.27127E-06	3.18482E-06	6.35409E-06	5.98545E-06	3.4696E-06	5.24887E-06	3.70773E-06	2.42327E-06
Propionibacterium_sp._oral_taxon_193	6.62727E-07	1.64227E-07	6.59455E-08	0.000000478	4.13846E-08	3.57462E-07	1.22692E-07	1.07692E-07	2.67909E-07	2.11182E-07	4.11727E-07	2.16364E-07	1.898E-07	2.376E-07	2.11733E-07	4.90667E-08
Propionibacterium_australiense	9.57727E-07	1.82045E-07	1.60545E-07	6.94636E-07	4.46538E-07	2.72115E-06	3.77923E-07	2.08962E-06	6.05818E-07	8.97364E-07	4.64909E-07	0.000001439	5.52333E-07	4.084E-07	2.88533E-07	3.484E-07
Propionibacterium_freudenreichii	2.08336E-06	2.16636E-07	9.428E-07	8.66727E-07	3.55923E-07	1.01377E-06	7.40308E-07	0.000001345	1.32064E-06	0.000000763	9.26545E-07	2.41009E-06	3.78067E-07	3.144E-07	5.368E-07	5.30467E-07
Propionibacterium_acidifaciens	0.000000373	3.21091E-07	3.40909E-08	4.96636E-07	2.85231E-07	1.86154E-07	8.86154E-08	4.84615E-08	3.75091E-07	1.96818E-07	4.36182E-07	0.0000006	7.64667E-08	3.14667E-07	2.24667E-07	1.862E-07
Cutibacterium_granulosum	2.88182E-07	6.37273E-08	0	2.88818E-07	2.36615E-07	8.47769E-07	6.18462E-08	8.89231E-08	3.78455E-07	1.66273E-07	1.32727E-07	4.76455E-07	2.06867E-07	4.49267E-07	4.90267E-07	5.414E-07
Cutibacterium_avidum	4.45936E-06	1.04664E-06	3.66286E-06	3.45227E-06	5.80615E-07	7.64492E-06	4.84854E-06	6.08923E-06	2.42945E-06	2.10582E-06	2.69291E-06	2.54582E-06	9.104E-07	9.67933E-07	1.54467E-06	1.31613E-06
Cutibacterium_acnes	1.33836E-06	5.96818E-07	1.81944E-06	1.79991E-06	6.88846E-07	3.06992E-06	4.18546E-06	5.08531E-06	1.84073E-06	1.13955E-06	2.39245E-06	1.70036E-06	9.11067E-07	1.05253E-06	8.40533E-07	7.85467E-07
Acidipropionibacterium_jensenii	8.48727E-07	1.46818E-07	8.79818E-08	9.62545E-07	5.16615E-07	1.09777E-06	7.17385E-07	9.70231E-07	8.85727E-07	5.83364E-07	5.92545E-07	1.11682E-06	5.992E-07	6.83667E-07	8.774E-07	9.79333E-07
Acidipropionibacterium_acidipropionici	1.29782E-06	1.38745E-06	3.29909E-07	1.08882E-06	3.86308E-07	4.31292E-06	6.93846E-07	0.000000766	7.40364E-07	7.12727E-07	6.27455E-07	9.67273E-07	5.72333E-07	7.506E-07	5.35333E-07	9.244E-07
Acidipropionibacterium_virtanenii	7.79455E-07	5.84545E-07	1.36618E-07	7.62273E-07	2.57231E-07	5.43923E-07	1.27146E-06	1.61838E-06	7.18273E-07	5.07545E-07	8.17545E-07	9.53909E-07	0.000000563	7.44467E-07	2.30733E-07	4.54133E-07
Streptacidiphilus_bronchialis	4.32727E-07	7.56182E-07	0.00000017	4.64364E-07	3.34615E-08	8.32923E-07	9.38462E-08	2.32769E-07	1.71091E-07	1.72182E-07	1.02727E-08	2.40636E-07	5.32E-08	1.022E-07	2.0964E-07	3.97333E-08
Streptacidiphilus_sp._PB12-B1b	1.26818E-07	3.87182E-07	4.50909E-08	5.82727E-08	1.52923E-07	5.92154E-07	1.57692E-07	0	0	0	1.19273E-07	2.04273E-07	9.47333E-08	3.96E-08	8.86667E-09	1.98667E-08
Streptacidiphilus_sp._P02-A3a	1.69273E-07	6.70909E-08	4.12727E-09	1.24791E-07	0	0	0	0	2.18091E-07	0.000000324	2.50182E-07	3.68727E-07	4.17333E-08	3.09333E-08	0	8.53333E-08
Kitasatospora_aureofaciens	0	0	0	0	3.58462E-08	0	0	0	0	0	0	0	0	0	0	0
Kitasatospora_albolonga	0	0	0	0	3.58462E-08	0	0	0	0	0	0	0	2.24E-08	1.61333E-08	1.49333E-08	0
Kitasatospora_setae	0	0.000000065	0	0	9.46154E-08	2.13846E-08	1.45846E-07	5.04615E-08	0	0	0	0	2.91333E-08	7.86E-08	5.98667E-08	0
Kitasatospora_sp._MMS16-BH015	0.000000089	1.41636E-07	3.27273E-08	5.29091E-08	3.34615E-08	2.52308E-08	3.77692E-08	2.83769E-07	0	5.22727E-08	1.02727E-08	6.90909E-08	0.000000027	1.17267E-07	1.22933E-07	3.65333E-08
Kitasatospora_sp._NA04385	0.000000662	8.84545E-08	0.000000249	7.29091E-08	0.000000089	0.000000026	4.33846E-08	2.66846E-07	1.19091E-07	0.000000133	0.000000394	3.82727E-07	2.14E-08	0	0	0
Streptomyces_sp._Go-475	0	0	0	0	0	0	0	0	0	0	0	0	0	3.09333E-08	0	0
Streptomyces_malaysiensis	0	0	0	0	0	0	0	0	0	0	0	0	0	2.38E-08	0	0
Streptomyces_autolyticus	0	0	0	0	0	7.21538E-08	0	0	0	0	0	0	0	0	0	0
Streptomyces_viridosporus	0	0.00000013	0	0	0	0	0	0	0	0	0	0	0	0	0	0
Streptomyces_collinus	0	1.37273E-08	0	0	0	0	0	0	0	0	0	0	0	0	0	0
Streptomyces_vinaceus	0	0	0	0	0	0	0	0	0	0	0	0	2.09333E-08	0	0	0
Streptomyces_sp._P3	0	0	0	0	0	0	0	0	0	0	0	0	5.44E-08	0.000000033	0	3.40667E-08
Streptomyces_luteoverticillatus	0	0	0	0	0	0	0	2.97692E-08	0	0	0	0	2.94667E-08	0	0	0
Streptomyces_hawaiiensis	0	0	0	0	0	0	0	6.41538E-08	0	0	0	0	0	0	0	0
Streptomyces_sp._SYP-A7193	0	0	0	0	3.15385E-08	0	0	0	0	0	0	3.16364E-08	0	0	0	0
Streptomyces_fungicidicus	0	0	0	0	0	0	0	0	0	0	0	7.13636E-09	0	1.71333E-08	0	0
Streptomyces_sp._QMT-12	2.56364E-08	0	0	0	0	0	0	0	0	0	0	7.13636E-09	0	0	0	0
Streptomyces_ferrugineus	0	0	0	5.18182E-09	0	0	0	0	0	0	0	7.13636E-09	0	0	0	0
Streptomyces_sp._WAC00288	8.12727E-08	0.00000013	0	5.18182E-09	2.26923E-08	0	0	0	0	0	0	0	0	0	0	0
Streptomyces_galilaeus	3.11818E-08	9.54545E-09	0	3.55182E-08	0	3.83077E-08	0	0	0.000000027	0	0	7.13636E-09	6.08E-08	0	0	0
Streptomyces_sp._CNQ-509	0	0	0	1.93636E-08	0	0	0	0	1.17636E-07	0	0	0	0	0	0	1.98667E-08
Streptomyces_sp._WAC_06738	0	0	0	1.93636E-08	0	1.74154E-07	0	0	0	0	0	0	0	0	0	0
Streptomyces_peucetius	0	0	0	3.99091E-08	3.15385E-08	0	0	2.46923E-08	0	0	0	0.000000035	0	0	0	0
Streptomyces_leeuwenhoekii	0	0	0	5.55545E-07	0	0	0	0	0	0	0	4.21364E-08	0	0	0	0
Streptomyces_sp._CMB-StM0423	2.70909E-08	3.24545E-07	0	6.48182E-08	0	0	0	0	3.75455E-08	5.22727E-08	0	0.000000138	0	0	0	0
Streptomyces_sp._Tu6071	0	0	0	0	0	0	0	0	0	0	0	0	4.86E-08	0	2.97333E-08	0
Streptomyces_coeruleorubidus	0	0	0	0	0	0	0	0	0	0	0	0	0	1.71333E-08	2.39333E-08	0
Streptomyces_coelicolor	0	0	0	0	0	0	0	0	0	0	0	0	0	0	4.82E-08	0
Streptomyces_sp._CB04723	3.62727E-08	0.000000065	0	0	0	0	0	0	0	0	0	0	6.08E-08	1.318E-07	1.00667E-07	3.72E-08
Streptomyces_rubrolavendulae	0	0	0	0	0.000000039	0	3.06923E-08	0	0	0	0	0	2.91333E-08	0	0	0
Streptomyces_roseochromogenus	0	1.68182E-08	0	7.97273E-08	5.95385E-08	0	0	0	2.72727E-08	0	0	0	0	0	4.38667E-08	0
Streptomyces_sp._2323.1	2.56364E-08	1.37273E-08	0	6.96091E-08	2.05385E-08	3.83077E-08	0	6.10769E-08	0.000000054	2.99909E-07	0	0.00000007	2.65933E-07	6.83333E-08	4.38667E-08	4.76667E-08
Streptomyces_rectiverticillatus	0	8.86364E-09	0	0	0	0	0	2.97692E-08	0	0	0	0	2.14E-08	0	4.44E-09	0
Streptomyces_clavuligerus	5.57273E-08	0	0	5.18182E-09	3.15385E-08	0	6.44615E-08	1.46923E-08	5.97273E-08	0	0	5.17273E-08	0	1.58E-08	0	1.26667E-07
Streptomyces_pristinaespiralis	4.60909E-08	0	0	8.39091E-08	0	0	6.44615E-08	0	1.13182E-07	0	0	0	1.86667E-08	1.71333E-08	0	4.76667E-08
Streptomyces_lavendulae	0	0	0	0	0	0	0	0	7.97273E-08	0	0	2.255E-07	2.64667E-08	2.62E-08	1.12667E-08	3.40667E-08
Streptomyces_spongiicola	3.11818E-08	0	0	4.88182E-08	1.18154E-07	0.000000026	0	1.90769E-08	0	0	0	7.13636E-09	2.09333E-08	1.58E-08	1.12667E-08	9.33333E-09
Streptomyces_sp._TN58	0	0	0	4.79091E-09	0	0	0	0	0	0	0	0	0	0	1.12667E-08	0
Streptomyces_parvulus	0	0	0	0	3.58462E-08	0	0	3.29231E-08	0	0	0	0	4.18667E-08	0	7.35333E-08	1.32467E-07
Streptomyces_chromofuscus	0	0	0	1.84091E-07	1.21077E-07	1.23846E-07	1.50308E-07	1.18308E-07	0	0	0	2.50455E-07	2.976E-07	1.51667E-07	2.59933E-07	1.98067E-07
Streptomyces_atratus	0	0	0	0	2.88462E-08	0	0	0	0	0	0	0	0	0	2.55333E-08	0
Streptomyces_violaceoruber	0	0	0	2.91545E-08	2.48538E-07	0	6.23077E-08	0	0	0	0	0.000000035	4.498E-07	2.97667E-07	0.000000325	2.95733E-07
Streptomyces_nigra	5.12727E-08	0	0	7.09091E-08	7.17692E-08	2.95385E-08	8.69231E-08	3.22308E-08	0	0	0	0	6.08E-08	1.58E-08	1.14907E-07	1.87867E-07
Streptomyces_sp._SAT1	6.19091E-08	0.000000109	0	0.000000048	2.59231E-08	4.27692E-08	2.73846E-08	9.84615E-08	0.000000198	1.17091E-07	0	0	3.94667E-08	1.61333E-08	2.97333E-08	0
Streptomyces_sp._QHH-9511	0	0	0	4.20909E-08	0	0	2.73846E-08	1.90769E-08	0	0	0	5.50455E-08	2.37333E-08	0	2.55333E-08	0
Streptomyces_alboniger	2.70909E-08	0	0	3.76364E-08	0	4.27692E-08	1.51538E-07	0	0	0	0	0	0	0	0	1.22E-08
Streptomyces_formicae	3.69091E-08	2.32727E-08	0	8.89727E-08	4.11538E-08	5.53846E-07	2.63077E-08	0	2.75455E-08	0.000000047	0	1.34455E-07	0	1.58E-08	0	5.21333E-08
Streptomyces_griseorubiginosus	1.14182E-07	6.80909E-08	0	0	6.46923E-08	2.11538E-07	2.63077E-08	6.45385E-08	0	3.62727E-08	0	3.57273E-08	0	3.50667E-08	1.026E-07	3.08E-08
Streptomyces_broussonetiae	7.88182E-08	1.16364E-08	0	0	0	0	2.63077E-08	0	0	0	0	7.13636E-09	0	0	0	0
Streptomyces_sp._3211	0	0	0	0	0	0	2.63077E-08	0	0	0	0	0	0	0	2.97333E-08	0
Streptomyces_sp._MOE7	0	0	0	0	0	0	2.63077E-08	0	0	0	0	0	0	0	1.29333E-07	0
Streptomyces_sp._endophyte_N2	2.56364E-08	0	0	5.18182E-09	2.05385E-08	0	2.63077E-08	0	0	6.08182E-08	0	0	0	0	4.03333E-08	0
Streptomyces_sp._NEAU-S7GS2	0	0	0	4.68818E-08	2.05385E-08	1.19615E-07	2.30769E-07	0	0	0	0	0	0	0	1.49333E-08	0
Streptomyces_cacaoi	3.11818E-08	1.04545E-08	0	3.24545E-08	3.73846E-08	0	0	7.93846E-08	0	0	0	1.88045E-07	9.26667E-08	0	4.64E-08	4.95333E-08
Streptomyces_sp._11-1-2	5.68182E-08	1.49182E-07	0	9.97273E-09	4.80769E-08	6.76923E-08	0	3.00769E-08	1.37636E-07	0	0	4.21364E-08	1.232E-07	8.60667E-08	3.28E-08	1.266E-07
Streptomyces_fagopyri	0	0	0	9.54545E-09	0	0	0	0	5.79091E-08	5.72727E-08	1.35636E-07	3.94545E-08	0	0	0	0
Streptomyces_albireticuli	2.70909E-08	0	0	1.03636E-08	0	0	0	0	0	0	6.88182E-08	7.13636E-09	0	0	0	6.33333E-08
Streptomyces_sp._W1SF4	7.37273E-08	2.03545E-07	0	6.13636E-08	0	0	0	0	0	0	6.88182E-08	1.42727E-08	0	0	0	0
Streptomyces_sp._Tu_2975	0	0	0	8.40909E-08	3.15385E-08	0	4.33846E-08	0	0	0	6.88182E-08	7.13636E-09	2.44E-08	0	2.09733E-08	3.40667E-08
Streptomyces_sampsonii	0	0	0	0	0	0	4.33846E-08	0	0	5.22727E-08	1.02727E-08	4.79091E-08	0	0	0	0
Streptomyces_exfoliatus	0	0	0	0	0	0	3.33077E-08	0	1.19636E-07	5.22727E-08	2.05455E-08	2.14545E-08	0	1.58E-08	0	9.33333E-09
Streptomyces_sp._CB09001	0	0	0	0	0	6.35385E-08	0	0	0	0	1.02727E-08	7.13636E-09	0	0	0	0
Streptomyces_sp._Mg1	0	0	0	0	0	0	0	0	0	2.85455E-08	1.02727E-08	5.64545E-08	0	0	2.97333E-08	4.95333E-08
Streptomyces_sp._Sge12	3.11818E-08	2.70909E-08	0	0	0	0	0	0	0.000000054	0	1.02727E-08	7.13636E-09	8.10667E-08	0	1.33333E-08	0
Streptomyces_sp._NA00687	2.27545E-07	0.000000085	0	1.43636E-08	6.16923E-08	0	1.01923E-07	0.000000011	1.51273E-07	5.22727E-08	8.93636E-08	1.89636E-07	6.46667E-08	1.58E-08	6.22E-08	0
Streptomyces_albidoflavus	0	0	0	0	0	0	1.17077E-07	0	0	0	2.76364E-08	2.80091E-07	0	0	0	0
Streptomyces_rutgersensis	9.26364E-08	1.16364E-08	0	4.79091E-09	0	9.08462E-08	1.89308E-07	1.90769E-08	8.49091E-08	9.49091E-08	2.31727E-07	7.13636E-09	7.72667E-08	5.22E-08	2.09733E-08	0
Streptomyces_sp._SS52	1.43909E-07	1.16364E-08	0	0	0	3.91615E-07	4.80769E-08	0.000000042	1.17636E-07	1.04545E-07	2.76364E-08	1.12591E-07	2.24E-08	0	6.384E-08	0
Streptomyces_sp._CdTB01	1.62636E-07	0	0	0	0	0	0	1.90769E-08	0	0	2.76364E-08	2.18364E-07	2.72E-08	1.71333E-08	0	4.76667E-08
Streptomyces_sp._HM190	9.11818E-08	0	0	6.63636E-08	0	0	5.54615E-08	0	5.48182E-08	5.22727E-08	1.90909E-07	1.99045E-07	2.24E-08	5.81333E-08	4.65067E-08	7.76E-08
Streptomyces_nodosus	0	0	0	3.89091E-08	0	7.76769E-07	3.11538E-08	1.90769E-08	0	0	8.70909E-08	0	0	0	0	0.000000079
Streptomyces_glaucescens	1.15273E-07	0.000000065	0	1.86818E-07	3.58462E-08	0	8.33077E-08	0	5.97273E-08	0	1.21818E-07	2.44636E-07	2.08E-08	5.88667E-08	1.49333E-08	1.22E-08
Streptomyces_lincolnensis	1.11818E-07	0	0	4.50909E-08	1.68385E-07	1.44846E-07	3.11538E-08	2.39154E-07	8.12727E-08	7.77273E-08	2.01545E-07	2.31136E-07	4.23333E-08	9.54667E-08	6.144E-08	2.24867E-07
Streptomyces_avermitilis	0	0	0	0	0	0	0	2.97692E-08	5.79091E-08	2.89545E-07	3.77273E-08	1.16909E-07	2.24E-08	0	0	1.54933E-07
Streptomyces_sp._RLB1-33	0	0	0	0	0	0	0	0	0	0	3.77273E-08	5.50455E-08	0	0	0	0
Streptomyces_aureoverticillatus	0	0	0	1.93636E-08	0	1.41923E-07	3.11538E-08	8.38462E-08	2.25455E-07	0	3.95455E-08	9.03636E-08	0	0	0	0
Streptomyces_globosus	0	0	0	4.79091E-09	3.15385E-08	0	3.06923E-08	7.03846E-08	0	3.03636E-08	6.71818E-08	0	0	0.000000033	0	0.000000056
Streptomyces_qinzhouensis	9.45455E-08	0	3.70909E-08	3.59091E-08	1.10615E-07	1.79231E-08	0	0.000000042	2.16818E-07	0.000000047	6.88182E-08	1.16591E-07	1.24133E-07	1.71333E-08	4.12E-08	3.40667E-08
Streptomyces_glomeroaurantiacus	2.70909E-08	4.39091E-08	3.70909E-08	3.24545E-08	2.75385E-08	0	2.63077E-08	0	3.98182E-08	0	6.68182E-08	2.85455E-08	0	0	0	0
Streptomyces_dengpaensis	3.69091E-08	0	7.40909E-08	2.55182E-08	9.32308E-08	1.19154E-07	0	5.22308E-08	2.75455E-08	0	2.76364E-08	8.94545E-08	4.18667E-08	1.336E-07	4.44E-09	6.97333E-08
Streptomyces_asterosporus	0	7.04545E-08	9.04545E-08	1.47273E-08	0	0	0	0	0	2.87273E-08	0	8.66818E-08	0	0	0	0
Streptomyces_katrae	2.52273E-07	0.00000039	6.14545E-08	0.00000028	0	0	0	0	4.45455E-08	0	0	1.42727E-08	0	0	6.12E-08	0
Streptomyces_cyaneochromogenes	2.56364E-08	1.37273E-08	2.96364E-08	1.91818E-08	0	0	0	2.46923E-08	0	0	0	0	2.08E-08	3.09333E-08	0	3.67333E-08
Streptomyces_sp._Z423-1	3.11818E-08	0.000000065	2.47273E-08	1.07882E-07	2.05385E-08	2.52308E-08	5.26154E-08	0	4.45455E-08	1.73909E-07	3.95455E-08	7.13636E-09	4.33333E-08	9.58E-08	7.05333E-08	2.10333E-07
Streptomyces_lunaelactis	2.59091E-07	1.16364E-08	7.40909E-08	1.04364E-07	6.69231E-08	1.60123E-06	4.15692E-07	1.66923E-07	2.07273E-07	1.04545E-07	2.14364E-07	6.61545E-07	1.24333E-07	1.208E-07	1.14867E-07	8.48667E-08
Streptomyces_nitrosporeus	2.27545E-07	4.92727E-07	1.63636E-08	0.000000113	1.05923E-07	0	1.24769E-07	1.35231E-07	1.98727E-07	3.62727E-08	6.71818E-08	2.34909E-07	2.14E-08	8.31333E-08	1.43067E-07	6.04E-08
Streptomyces_gilvosporeus	1.01455E-07	0	0	3.95727E-07	0	4.23769E-07	0.000000096	1.47308E-07	5.97273E-08	3.62727E-08	0	0.00000007	6.82667E-08	0	6.23333E-08	3.97333E-08
Streptomyces_albulus	1.52545E-07	0	0	4.28182E-08	3.34615E-08	1.79923E-07	0	8.52308E-08	1.74182E-07	1.04545E-07	6.71818E-08	3.01455E-07	6.82667E-08	0.000000041	8.86667E-09	8.59333E-08
Streptomyces_cyaneogriseus	1.58182E-08	0.000000037	4.94545E-08	8.09727E-08	0	0	0	0	0	3.62727E-08	0	6.73636E-08	0	4.49333E-08	0	0
Streptomyces_noursei	1.19727E-07	4.77636E-07	0	1.30182E-07	0	0	0	1.46923E-08	3.75455E-08	0.000000028	6.88182E-08	4.56864E-07	0	0	0	0
Streptomyces_tirandamycinicus	1.42691E-06	0.00000039	2.59091E-07	9.56636E-07	1.46923E-07	0	9.77692E-08	9.42308E-08	5.31091E-07	5.33727E-07	1.55909E-07	6.26182E-07	2.852E-07	4.02467E-07	2.56533E-07	5.97133E-07
Streptomyces_sp._WAC_01529	2.72727E-07	2.32727E-08	0	6.63636E-08	8.73846E-08	0	2.85615E-07	4.32308E-08	1.34364E-07	1.58364E-07	3.52909E-07	4.82727E-07	2.37333E-08	8.48667E-08	1.12667E-08	1.26667E-07
Streptomyces_sp._CRPJ-33	7.25455E-08	4.09091E-08	1.63636E-08	4.88818E-08	0	0	0	1.46923E-08	0	0	0	0	0	2.38E-08	0	3.40667E-08
Streptomyces_sp._SirexAA-E	4.14545E-08	0	3.70909E-08	0.000000084	1.86923E-08	3.83077E-08	0	0	5.97273E-08	0.000000028	0	2.14545E-08	0	0	0	1.66667E-08
Streptomyces_sp._SM18	1.54727E-07	1.79136E-07	0	8.08182E-08	3.15385E-08	0	1.00846E-07	1.17923E-07	5.97273E-08	0	0	2.85455E-08	0	6.66E-08	6.85333E-08	1.22E-08
Streptomyces_sp._YIM_121038	4.04455E-07	1.82273E-07	0.000000298	0.000000248	5.95385E-08	0	1.13077E-07	3.07154E-07	1.76455E-07	4.69727E-07	1.52727E-07	2.86273E-07	2.08E-08	3.35333E-08	0	0
Streptomyces_spectabilis	1.54091E-07	2.17864E-07	5.05273E-07	1.27818E-07	0	9.46154E-09	1.13077E-07	2.33692E-07	1.20455E-07	0.000000056	1.04909E-07	4.59091E-08	1.86667E-08	7.79333E-08	6.78E-08	0.000000228
Streptomyces_sp._SCSIO_3032	7.37273E-08	0	1.26364E-07	6.16364E-08	0	0	2.73846E-08	3.00769E-08	2.75455E-08	1.63545E-07	0	7.84545E-08	5.58667E-08	7.39333E-08	1.10067E-07	9.33333E-09
Streptomyces_antibioticus	6.12636E-07	2.34545E-08	9.81818E-08	0.000000237	3.67692E-07	0	1.24077E-07	0.000000356	1.45545E-07	0	3.53818E-07	4.26818E-07	0	1.14867E-07	2.248E-07	9.54E-08
Streptomyces_sp._Tue_6075	1.39273E-07	1.16364E-08	3.70909E-08	1.01545E-07	0.000000065	3.55231E-07	2.68615E-07	5.13077E-08	5.79091E-08	0.000000034	2.38818E-07	2.21636E-07	2.44E-08	2.98E-08	7.42667E-08	3.08E-08
Streptomyces_paludis	3.59818E-07	9.34545E-08	8.40909E-08	2.95455E-08	1.48077E-07	9.72308E-08	9.04615E-08	0	1.06273E-07	1.44545E-07	2.19273E-07	8.43818E-07	1.42667E-07	1.41733E-07	0	4.89333E-08
Streptomyces_tendae	0	0	0	0	0	0	0	0	3.75455E-08	0.000000034	2.39727E-07	7.12727E-08	0	0	1.01107E-07	0
Streptomyces_anulatus	6.25455E-08	2.32727E-08	5.34545E-08	5.16364E-08	1.62615E-07	1.15154E-07	1.10385E-07	3.56385E-07	1.69182E-07	1.66182E-07	1.27818E-07	1.37591E-07	4.16E-08	1.61333E-08	1.05667E-07	3.97333E-08
Streptomyces_griseochromogenes	1.96091E-07	0	2.20909E-07	6.31818E-08	6.63846E-08	9.05385E-08	9.07692E-08	0.000000143	3.80091E-07	1.92818E-07	1.10273E-07	1.51636E-07	4.52E-08	0.000000066	5.98667E-08	2.352E-07
Streptomyces_sp._RPA4-2	0.000000242	9.79091E-08	3.70909E-08	1.32273E-07	1.28462E-07	0	6.51538E-08	0	0.000000098	0	1.39909E-07	3.07909E-07	1.03067E-07	3.26667E-08	1.65333E-08	0.000000168
Streptomyces_sp._CRXT-G-22	3.62727E-08	2.34545E-08	0	6.63636E-08	7.61538E-08	1.55385E-07	6.40769E-08	3.60692E-07	0.000000099	1.99364E-07	4.36364E-08	1.17273E-07	1.42067E-07	1.76733E-07	9.08667E-08	1.32667E-07
Streptomyces_hygroscopicus	2.59091E-07	0	3.70909E-08	9.41818E-08	1.30462E-07	1.36308E-07	8.37692E-08	9.11538E-08	5.48182E-08	2.01091E-07	0.000000156	0.000000517	1.132E-07	7.60667E-08	1.37533E-07	1.03667E-07
Streptomyces_qaidamensis	0	0	0	3.24545E-08	0	0	0	0	0	0	0.0000001	0	6.08E-08	0	0	0
Streptomyces_scabiei	5.01818E-08	0	0	5.18182E-09	0.000000065	0	4.33846E-08	0	3.75455E-08	3.89091E-08	1.78545E-07	6.09091E-08	7.73333E-08	0	0	0
Streptomyces_finlayi	0	0.000000065	0	7.28182E-08	0	0	0	0	0	0	1.05636E-07	2.14545E-08	5.88667E-08	0	0	1.98667E-08
Streptomyces_globisporus	0	0	0	3.24545E-08	7.03846E-08	0.000000026	1.10769E-07	5.13077E-08	8.43636E-08	9.40909E-08	7.23636E-08	8.42727E-08	1.28533E-07	1.20467E-07	1.282E-07	1.00067E-07
Streptomyces_ficellus	8.43273E-07	1.98182E-08	0	8.03909E-07	2.40846E-07	2.15231E-07	0	3.24615E-07	3.66727E-07	2.08182E-07	2.23727E-07	2.89455E-07	3.94133E-07	4.876E-07	4.87067E-07	8.51667E-07
Streptomyces_sp._Rer75	6.25455E-08	2.01909E-07	3.70909E-08	0.000000308	5.20769E-08	0	0	1.83538E-07	0.000000053	1.43455E-07	7.23636E-08	1.32636E-07	0	0	9.35733E-08	8.03333E-08
Streptomyces_sp._GS7	1.53636E-07	0	0	1.86818E-07	2.05385E-08	4.33846E-08	0	0	7.53636E-08	0	0.000000142	8.73636E-08	0	0	0	1.98667E-08
Streptomyces_sp._SUK_48	2.62091E-07	0.000000065	0	1.35909E-07	5.63846E-08	0	0	1.86692E-07	6.97273E-08	0	1.43364E-07	4.45909E-08	1.39733E-07	9.82E-08	9.94E-08	2.728E-07
Streptomyces_sp._Z022	9.04545E-08	0	0	0	1.29923E-07	3.58462E-08	0.000000541	4.90615E-07	1.36182E-07	1.02909E-07	4.01364E-07	1.79364E-07	1.018E-07	1.03867E-07	3.73733E-07	1.41667E-07
Streptomyces_kanamyceticus	1.18182E-07	1.21818E-07	2.51182E-08	5.76364E-08	1.72231E-07	0	0	1.09769E-07	0.000000027	1.02909E-07	1.02727E-08	4.45909E-08	2.16933E-07	1.08333E-07	9.69733E-08	1.21133E-07

Streptomyces_olivaceus	0	1.04545E-08	8.75455E-09	9.54545E-09	0	0	0	0	0	0	0	0	0	0	0	0
Streptomyces_alboflavus	0	0	8.75455E-09	0	0	0	3.33077E-08	0	2.16364E-08	0	6.55455E-08	2.85455E-08	1.00533E-07	0	0	0
Streptomyces_sp._NHF165	7.44455E-07	1.64364E-07	4.13118E-07	3.92545E-07	3.58462E-08	1.13538E-07	6.02538E-07	8.95077E-07	6.06182E-07	3.89636E-07	6.23364E-07	5.08545E-07	0	0	2.24667E-08	0
Streptomyces_sp._ZFG47	5.57273E-08	0	6.12727E-08	1.78336E-06	0.000000276	5.53154E-07	1.72462E-06	2.16485E-06	0.000000027	9.92727E-08	4.80909E-07	6.73727E-07	1.68667E-07	6.57667E-07	6.34107E-07	4.85867E-07
Streptomyces_fodineus	1.02909E-07	9.36364E-08	2.45455E-07	3.37273E-08	2.88462E-08	1.93538E-07	1.31769E-07	8.19231E-08	5.91818E-08	1.02636E-07	9.31818E-08	8.66818E-08	1.45667E-07	3.09333E-08	3.064E-08	6.33333E-08
Streptomyces_sp._DSM_40868	1.58182E-08	0	1.77273E-08	4.79091E-09	2.05385E-08	0	1.06615E-07	5.69231E-08	8.43636E-08	0.000000028	0	8.80909E-08	2.14E-08	0	0	4.95333E-08
Streptomyces_sp._M56	0.000000149	0	1.77273E-08	6.13636E-08	3.88462E-08	2.71077E-07	1.84846E-07	6.69231E-08	1.02091E-07	1.64364E-07	0	9.65455E-08	7.98667E-08	9.98667E-08	1.074E-07	2.92E-08
Streptomyces_sp._S1D4-11	8.80909E-08	4.63636E-08	9.89091E-08	5.63636E-08	1.27846E-07	8.10769E-08	1.13231E-07	0	8.07273E-08	2.64727E-07	1.70091E-07	2.72545E-07	2.92867E-07	1.48467E-07	3.224E-08	7.08E-08
Streptomyces_sp._RPA4-5	1.17636E-07	8.84545E-08	1.01091E-07	1.30636E-07	0	6.76923E-08	3.84154E-07	5.28462E-08	3.06727E-07	0.000000047	2.97364E-07	8.87273E-08	2.14E-08	1.30467E-07	8.35333E-08	9.02E-08
Streptomyces_niveus	1.55364E-07	4.55E-08	3.28545E-08	2.26364E-07	6.91538E-08	2.16923E-08	4.80769E-08	0.000000011	0	1.74636E-07	0	7.12727E-08	2.14667E-08	3.09333E-08	6.39067E-08	1.37333E-07
Streptomyces_ambofaciens	9.36364E-08	0	4.12727E-09	0	3.58462E-08	0	0	4.07692E-08	0	0.000000028	1.02727E-08	2.14545E-08	0	0.000000033	1.93733E-08	1.98667E-08
Streptomyces_rimosus	6.84545E-08	0	4.12182E-08	1.36245E-07	1.16231E-07	0	1.46538E-07	1.93692E-07	0.000000204	7.29091E-08	1.71364E-07	2.76091E-07	2.30867E-07	2.39067E-07	1.79933E-07	4.03467E-07
Streptomyces_pluripotens	4.85182E-07	8.18182E-08	4.12182E-08	4.29273E-07	9.16154E-08	0	5.48462E-08	3.00769E-08	0.000000219	0.000000075	1.55636E-07	2.14545E-08	0.000000563	1.22333E-07	9.57333E-08	3.00333E-07
Streptomyces_lividans	1.12455E-07	4.39091E-08	4.12727E-09	3.87273E-08	1.38462E-07	0	6.23077E-08	8.46154E-08	0.000000106	0	0.000000093	1.46273E-07	6.08E-08	5.08667E-08	1.46467E-07	1.38333E-07
Streptomyces_actuosus	1.16727E-07	0	8.25455E-09	6.12727E-08	2.88462E-08	0	0	0	1.75182E-07	0	0	4.92727E-08	8.76E-08	6.39333E-08	6.91067E-08	3.08E-08
Streptomyces_bingchenggensis	2.55455E-07	2.83455E-07	4.09818E-08	1.90545E-07	0	7.22308E-08	4.80769E-08	4.91538E-08	0.000000213	2.05091E-07	3.87364E-07	3.72182E-07	2.24E-08	1.27733E-07	2.59667E-07	1.66667E-07
Streptomyces_seoulensis	4.26636E-07	4.61273E-07	9.44364E-08	0.000000317	8.92308E-08	4.03923E-07	3.06692E-07	3.49538E-07	4.43909E-07	2.59545E-07	0.000000226	6.05091E-07	7.56E-08	7.39333E-08	8.77733E-08	2.666E-07
Streptomyces_olivoreticuli	9.51818E-08	3.52636E-07	2.46182E-08	1.69909E-07	3.58462E-08	1.68815E-06	5.11308E-07	2.93846E-08	9.96364E-08	2.87273E-08	8.02727E-08	1.01636E-07	2.14E-08	6.12667E-08	1.13973E-07	0.000000088
Streptomyces_xiamenensis	6.23636E-08	0	3.00909E-08	1.45545E-07	3.58462E-08	3.65692E-07	0.00000013	6.66923E-08	4.45455E-08	2.46818E-07	1.44636E-07	3.54818E-07	2.94667E-08	0	1.48133E-07	3.67333E-08
Streptomyces_albus	6.23091E-07	4.90455E-07	3.16273E-07	3.95818E-07	1.68538E-07	4.66692E-07	4.71154E-07	3.37462E-07	6.16909E-07	3.54273E-07	8.97455E-07	1.15855E-06	2.74533E-07	3.456E-07	1.85733E-07	1.93533E-07
Streptomyces_sp._WAC_01438	5.42727E-08	0	4.12727E-09	1.01909E-07	6.49231E-08	4.59615E-07	2.63077E-08	2.17308E-07	1.83182E-07	0	4.98182E-08	1.81818E-07	0	1.46933E-07	7.42E-08	1.408E-07
Streptomyces_sp._M2	1.05455E-07	3.24545E-07	4.12727E-09	9.54545E-09	1.78769E-07	0	0	3.22308E-08	1.15455E-07	0	1.33636E-07	1.18364E-07	1.02467E-07	1.142E-07	1.5284E-07	5.10667E-08
Streptomyces_sp._TLI_053	2.96364E-08	0	4.12727E-09	3.55182E-08	6.30769E-08	0	4.33846E-08	4.84615E-08	2.16364E-08	0	2.43636E-07	0.000000085	0	3.50667E-08	0	0
Streptomyces_sp._SCUT-3	2.83545E-07	0	2.64945E-07	0.000000078	1.15462E-07	0	2.94154E-07	3.34615E-07	1.33545E-07	1.38909E-07	1.62909E-07	2.60455E-07	2.91333E-08	1.67733E-07	1.49107E-07	0.000000054
Streptomyces_sp._INR7	0	0	4.12727E-09	0	0	4.27692E-08	0	0	4.45455E-08	1.51545E-07	6.68182E-08	0.000000035	0	0	0	0
Streptomyces_sp._C8S0	0	0	4.12727E-09	3.35455E-08	0	4.13846E-08	0	1.15769E-07	8.61818E-08	0	0.000000061	2.85182E-07	2.14E-08	0	1.49333E-08	4.02E-08
Streptomyces_sp._RTd22	3.00455E-07	0.000000047	3.03709E-07	1.65636E-07	2.88462E-08	5.09231E-08	0	1.40692E-07	1.06182E-07	0.000000065	1.69909E-07	2.86091E-07	5.56E-08	8.10667E-08	9.53333E-08	5.70667E-08
Streptomyces_sp._769	0	1.04545E-08	4.94545E-08	7.65455E-08	1.83154E-07	8.10769E-08	9.77692E-08	9.03846E-08	2.64545E-08	0	0.000000039	1.34636E-07	0	0.000000115	9.73733E-08	1.71333E-07
Streptomyces_sp._fd1-xmd	6.23636E-08	4.21455E-07	1.37764E-07	1.74091E-07	0	0	0	2.46923E-08	2.64545E-08	3.62727E-08	6.66364E-08	1.16591E-07	0	0	0	0
Streptomyces_koyangensis	1.68018E-06	3.29182E-07	1.69005E-06	1.12109E-06	7.02308E-08	2.06692E-07	1.15731E-06	0.000001242	5.20273E-07	5.50455E-07	8.34818E-07	1.89809E-06	9.06E-08	3.356E-07	3.65333E-07	0.000000236
Streptomyces_lydicus	9.57273E-08	0	8.31818E-09	4.30909E-08	1.34769E-07	8.23077E-07	4.80769E-08	3.65846E-07	4.91818E-08	3.03636E-08	1.06091E-07	8.21364E-08	4.27333E-08	1.24067E-07	2.112E-07	2.148E-07
Streptomyces_griseoviridis	0	1.28818E-07	4.99091E-08	2.89091E-08	3.15385E-08	2.47692E-07	1.81538E-07	1.46923E-08	0	3.89091E-08	2.76364E-08	7.84545E-08	0	0	4.38667E-08	0
Streptomyces_cattleya	1.49818E-07	0	8.25455E-09	6.65455E-08	4.11538E-08	4.13846E-08	1.00385E-07	6.55385E-08	7.94545E-08	1.39545E-07	2.24182E-07	1.42273E-07	2.14E-08	6.98667E-08	7.64667E-08	9.33333E-09
Streptomyces_griseus	1.77909E-07	5.41818E-08	5.01273E-08	2.14518E-07	2.29385E-07	6.53692E-07	1.77846E-07	8.30769E-08	1.21545E-07	1.03545E-07	1.49091E-07	2.52455E-07	1.98667E-07	3.66267E-07	2.28133E-07	1.122E-07
Streptomyces_tsukubensis	1.13636E-07	1.60182E-07	0	9.66091E-08	2.05385E-08	0.000000079	0	5.81538E-08	0.000000027	9.23636E-08	1.07818E-07	2.14545E-08	9.35333E-08	8.12667E-08	0	1.044E-07
Streptomyces_davaonensis	3.99545E-07	0	2.15091E-07	1.81245E-06	4.68846E-07	0	2.03846E-07	2.51154E-07	1.30309E-06	1.62518E-06	1.66445E-06	2.63018E-06	5.34267E-07	4.77133E-07	7.90067E-07	1.22293E-06
Streptomyces_prasinus	7.44545E-07	0	0	3.56091E-07	6.49231E-08	0	2.64615E-07	1.36454E-06	4.59818E-07	3.76909E-07	6.78091E-07	7.25727E-07	5.39267E-07	4.12333E-07	5.34773E-07	0.000000202
Streptomyces_subrutilus	3.37727E-07	3.76455E-07	1.03582E-07	1.72273E-07	0	9.81538E-08	0	5.22308E-08	1.44091E-07	1.19909E-07	0	0.000000495	4.10667E-08	1.61933E-07	0	0.00000009
Streptomyces_hundungensis	3.11818E-08	0.000000065	0	1.91818E-08	1.16231E-07	9.87692E-07	5.26154E-08	7.28462E-08	2.72727E-08	0	0	1.72818E-07	2.72E-08	3.48E-08	0.000000086	0
Streptomyces_violaceusniger	2.78182E-07	9.36364E-09	9.59455E-08	2.54636E-07	2.26615E-07	2.07231E-07	8.86154E-08	4.00077E-07	1.07091E-07	2.08364E-07	7.07091E-07	1.50727E-07	1.338E-07	4.314E-07	1.462E-07	1.76733E-07
Streptomyces_antimycoticus	7.88182E-08	8.18182E-08	0	0.000000118	3.15385E-08	0	1.89308E-07	0	1.14273E-07	2.64455E-07	1.24091E-07	3.11818E-07	2.24E-08	6.57333E-08	0.000000048	0
Streptomyces_chartreusis	5.33818E-07	3.47182E-07	1.14364E-06	5.92091E-07	1.11554E-06	0	1.09923E-07	0.000000198	1.15036E-06	1.32327E-06	1.17309E-06	1.06818E-06	9.97467E-07	1.37633E-06	9.866E-07	1.62573E-06
Streptomyces_phaeolivaceus	6.74545E-08	0	3.70909E-08	3.55182E-08	2.59231E-08	0	2.63077E-08	8.92308E-08	3.98182E-08	0	4.81818E-08	7.69091E-08	0	1.09533E-07	0	1.13667E-07
Streptomyces_aquilus	2.70909E-08	0	5.11273E-08	2.83273E-07	0	2.16923E-08	0	7.40769E-08	0	5.22727E-08	0	0	2.14E-08	3.48E-08	1.65333E-08	1.22E-08
Streptomyces_vietnamensis	3.69091E-08	0	4.12727E-09	9.54545E-09	0	1.79231E-08	5.26154E-08	1.16154E-07	5.34545E-08	1.27091E-07	0	9.51818E-08	0	3.48E-08	2.97333E-08	0
Streptomyces_fulvissimus	2.51418E-06	7.73455E-07	2.84909E-06	1.72809E-06	6.46923E-08	1.37169E-06	2.93308E-07	3.93846E-07	4.99182E-07	0.000001233	1.31709E-06	1.19909E-06	1.90867E-07	2.30467E-07	0.000000109	1.63467E-07
Streptomyces_xinghaiensis	8.97455E-07	3.25591E-07	1.63636E-08	2.51818E-07	4.45615E-07	2.06154E-07	0.000000183	1.08308E-07	8.00909E-07	3.84818E-07	3.85636E-07	5.34364E-07	5.938E-07	2.55067E-07	0.000000235	2.45267E-07
Streptomyces_venezuelae	7.85364E-07	9.16091E-07	3.42527E-07	0.000000829	3.30538E-07	1.54008E-06	7.37923E-07	5.79462E-07	3.80091E-07	5.00091E-07	4.50909E-07	5.77182E-07	2.006E-07	4.03133E-07	1.864E-07	2.72933E-07
Streptomyces_sp._jing01	0	9.54545E-09	0	9.37273E-08	3.58462E-08	0	0	5.81538E-08	0	9.11818E-08	3.33636E-08	0	4.38667E-08	3.48E-08	0.000000044	1.22E-08
Streptomyces_sp._CRXT-Y-14	5.56727E-07	0.00000013	3.70909E-08	9.39091E-08	2.50846E-07	0	1.10769E-07	6.75692E-07	4.19545E-07	0.00000072	9.51636E-07	4.08636E-07	1.71867E-07	3.408E-07	2.55507E-07	2.30667E-07
Streptomyces_sp._CCM_MD2014	6.44364E-07	0.000000065	3.70909E-08	2.29455E-07	1.27769E-07	6.76923E-08	1.48769E-07	0	1.90727E-07	3.17909E-07	0.000000132	2.96636E-07	1.944E-07	2.564E-07	4.64667E-08	1.04867E-07
Streptomyces_sp._S8	3.29455E-07	9.35455E-08	0	1.69273E-07	1.73231E-07	3.83077E-08	1.93077E-07	4.88462E-08	0.000000258	0.000000371	2.62182E-07	5.05273E-07	9.57333E-08	6.93333E-08	4.44E-09	0.00000009
Streptomyces_sp._So13.3	6.46455E-07	2.34545E-08	2.87273E-08	4.81273E-07	2.08769E-07	7.29462E-07	1.41385E-07	9.71538E-08	1.56636E-07	0.000000133	1.54364E-07	2.93273E-07	1.56867E-07	1.49067E-07	8.224E-08	3.15867E-07
Streptomyces_sp._NA04227	2.26909E-07	0.00000013	1.64727E-07	0.000000369	1.99923E-07	0.000000026	3.42385E-07	3.52077E-07	1.98182E-07	4.90273E-07	2.15727E-07	1.09455E-07	4.034E-07	2.99933E-07	3.20333E-07	2.24867E-07
Streptomyces_sp._JB150	1.31727E-07	0	3.70909E-08	5.62455E-08	2.05385E-08	2.13846E-08	0	1.10385E-07	2.25364E-07	0.000000028	2.49818E-07	0	7.05333E-08	1.70733E-07	1.48533E-07	2.10667E-07
Streptomyces_sp._ADI95-16	1.26636E-07	0	0	0.000000042	0	0	7.40769E-08	4.32308E-08	0	9.22727E-08	1.10273E-07	3.63727E-07	1.22933E-07	1.55533E-07	1.02067E-07	3.40667E-08
Streptomyces_sp._S4.7	3.42545E-07	0	2.96364E-08	6.17427E-07	5.74615E-08	0	2.73923E-07	1.72615E-07	4.16182E-07	9.40909E-08	9.44545E-08	2.02182E-07	2.068E-07	5.02067E-07	2.996E-07	2.056E-07
Streptomyces_sp._3214.6	8.53727E-07	0	8.75455E-09	5.52818E-06	7.77385E-07	1.04564E-05	1.74769E-05	1.17931E-05	2.27991E-06	0.00000377	3.65818E-06	0.00000272	6.374E-07	8.15533E-07	1.24067E-06	7.03667E-07
Streptomyces_harbinensis	7.30227E-06	1.87273E-07	5.34545E-08	5.20091E-07	1.43992E-05	2.62154E-07	1.44385E-07	9.72308E-08	1.26389E-05	1.39636E-05	6.26227E-06	7.89673E-06	2.18213E-05	1.89587E-05	1.50213E-05	1.47807E-05
Scardovia_inopinata	1.69782E-06	1.13836E-06	5.88409E-07	1.69636E-06	8.28692E-07	1.13623E-06	0.00000076	1.16792E-06	9.92182E-07	0.000001084	8.03455E-07	1.61664E-06	4.856E-07	7.23333E-07	5.43267E-07	8.86333E-07
Parascardovia_denticolens	4.60273E-06	8.96909E-07	6.46818E-07	1.60655E-06	3.16885E-06	1.63227E-05	4.83038E-06	4.20469E-06	4.67127E-06	9.06545E-06	5.38818E-06	5.25409E-06	2.52427E-06	2.9742E-06	3.4046E-06	1.96733E-06
Gardnerella_vaginalis	0.000001175	2.44845E-06	4.46491E-07	2.97118E-06	1.84262E-06	1.53315E-06	1.03846E-06	0.000000832	1.64173E-06	0.000001559	0.000001771	1.98818E-06	1.39167E-06	1.80873E-06	1.81273E-06	1.53887E-06
Bifidobacterium_coryneforme	0	0	0	0	0	0	0	5.81538E-08	0	0	0	7.13636E-09	2.14E-08	3.09333E-08	0	0
Bifidobacterium_subtile	7.65273E-07	8.12818E-07	2.561E-07	4.27273E-07	3.68615E-07	0.000000606	5.54462E-07	6.82538E-07	5.23182E-07	4.09091E-07	5.91636E-07	6.66091E-07	2.32067E-07	1.90333E-07	0.000000261	5.08867E-07
Bifidobacterium_asteroides	1.16918E-06	1.14164E-06	5.67182E-07	8.91636E-07	7.84923E-07	2.49808E-06	3.62769E-07	1.11762E-06	7.89727E-07	6.20091E-07	8.56545E-07	2.16409E-06	4.55933E-07	5.13067E-07	5.132E-07	6.756E-07
Bifidobacterium_actinocoloniiforme	1.49545E-06	1.44191E-06	2.17782E-06	2.09991E-06	7.85308E-07	2.77769E-07	3.89623E-06	0.000005564	1.30782E-06	2.05118E-06	2.11482E-06	2.01255E-06	5.498E-07	3.306E-07	1.2658E-06	5.54267E-07
Bifidobacterium_indicum	1.48818E-07	0	0	3.85455E-08	8.85385E-08	1.54077E-07	1.12538E-07	1.31846E-07	1.15455E-07	1.31182E-07	3.03091E-07	2.76227E-07	6.58E-08	1.134E-07	1.02867E-07	4.95333E-08
Bifidobacterium_choerinum	0.0001519	7.55136E-06	6.24968E-05	3.43517E-05	0.00025692	0.000044315	0.00032922	0.000329715	3.28991E-05	5.23162E-05	0.000126052	5.88889E-05	0.000198798	0.000265782	0.000329874	0.000261124
Bifidobacterium_bifidum	9.87182E-07	6.31273E-07	3.20364E-07	6.04273E-07	3.28492E-05	4.04538E-07	4.19542E-05	2.45425E-05	1.77527E-06	4.83182E-07	5.11245E-06	1.27945E-06	2.71553E-05	3.50675E-05	4.92656E-05	3.82067E-05
Bifidobacterium_pullorum	6.30809E-05	1.05127E-06	1.43245E-05	1.67272E-05	3.14759E-05	2.79382E-05	3.73752E-05	0.000112063	9.30836E-06	1.67114E-05	0.000033923	2.14663E-05	1.33007E-06	1.53807E-06	1.75753E-06	0.000002323
Bifidobacterium_lemurum	2.19773E-06	2.13145E-06	5.29036E-07	1.20991E-06	1.42054E-06	9.82308E-07	1.10931E-06	3.03823E-06	1.63645E-06	5.34909E-07	1.12573E-06	1.26273E-06	1.21427E-06	0.000001542	1.36633E-06	1.37193E-06
Bifidobacterium_angulatum	2.72582E-06	1.02055E-06	1.01691E-06	2.83936E-06	1.68208E-06	1.62954E-06	3.01354E-06	2.94023E-06	2.50318E-06	2.30364E-06	1.65145E-06	1.53418E-06	1.1448E-06	1.2834E-06	8.19267E-07	1.03387E-06
Bifidobacterium_thermophilum	0.000000524	0.00000199	5.64491E-07	9.14545E-07	1.41108E-06	6.76231E-07	7.97023E-06	5.48631E-06	3.01455E-07	2.26636E-07	7.72545E-07	0.000000752	8.76067E-07	2.37433E-06	0.000002699	2.12533E-06
Bifidobacterium_animalis	0.000179731	1.27598E-05	6.09654E-05	2.15538E-05	0.000479887	3.85229E-05	0.000624683	0.000465299	5.28337E-05	7.86665E-05	0.000257778	0.00010973	0.000387778	0.000535012	0.000649622	0.000549988
Bifidobacterium_eulemuris	1.51518E-06	6.70909E-07	2.00609E-06	1.93991E-06	1.98808E-06	1.21751E-05	2.00738E-06	1.55631E-06	1.55009E-06	3.89818E-06	1.41682E-06	9.51945E-06	4.414E-07	1.04867E-06	2.03527E-06	1.07867E-06
Bifidobacterium_pseudocatenulatum	1.57482E-05	1.26271E-05	4.36832E-06	1.39345E-05	9.69538E-06	8.69538E-06	5.27769E-06	4.95577E-06	1.80736E-05	2.03882E-05	1.81473E-05	2.78055E-05	0.000007052	0.000009364	7.66333E-06	7.98333E-06
Bifidobacterium_scardovii	0.0000025	9.88545E-07	5.69455E-07	1.98782E-06	1.61608E-06	2.53923E-06	1.00454E-06	1.14677E-06	1.38918E-06	2.41136E-06	2.17136E-06	3.43209E-06	1.19173E-06	1.89487E-06	9.278E-07	1.2242E-06
Bifidobacterium_dentium	1.20618E-06	5.03636E-08	7.82182E-08	7.23182E-07	1.00331E-06	4.38354E-06	1.72038E-06	5.67231E-07	1.31555E-06	1.78818E-06	1.77364E-06	1.50264E-06	9.69933E-07	0.000001567	8.47467E-07	1.26067E-06
Bifidobacterium_adolescentis	8.35818E-06	0.000002412	1.29509E-06	6.81455E-06	4.31485E-06	8.31923E-07	4.97085E-06	6.44846E-06	0.000004081	3.19773E-06	4.44555E-06	4.04364E-06	5.10533E-06	0.000006074	6.90933E-06	0.0000087
Bifidobacterium_catenulatum	6.53364E-06	1.40664E-06	2.27627E-06	4.83727E-06	6.88231E-06	1.65523E-06	6.71692E-06	5.25862E-06	6.49818E-06	0.000006676	5.98455E-06	0.00001094	6.92067E-06	5.49227E-06	4.54787E-06	3.23133E-06
Bifidobacterium_longum	2.85909E-05	7.07018E-06	7.03391E-06	1.23536E-05	5.54295E-05	3.03392E-05	7.93415E-05	6.49615E-05	1.80818E-05	0.00002167	2.74027E-05	2.17773E-05	4.79716E-05	6.32626E-05	7.83273E-05	6.54593E-05
Bifidobacterium_breve	4.10364E-05	3.27557E-05	1.03421E-05	2.39927E-05	0.0000609	3.05645E-05	7.98145E-05	5.70077E-05	3.91629E-05	3.65455E-05	3.59455E-05	3.38182E-05	4.95733E-05	6.71659E-05	7.82651E-05	5.93628E-05
Bifidobacterium_pseudolongum	0.022408152	0.000962467	0.008653322	0.0055758	0.041862237	0.007122431	0.05293217	0.053327859	0.004668242	0.007766568	0.024156857	0.011393041	0.030400174	0.041997434	0.051528272	0.041947704
Tomitella_sp._HY172	0	0	0	4.20909E-08	0	0	0	0	8.49091E-08	0	0	1.08773E-07	2.24E-08	0	4.38667E-08	0
Tomitella_sp._HY188	8.17273E-08	0	0	6.60636E-08	3.58462E-08	0.000000026	0	0	2.75455E-08	0.000000028	0	7.13636E-09	4.38E-08	0.000000014	4.46667E-08	6.14E-08
Lawsonella_clevelandensis	1.29364E-07	0.00000013	3.42364E-07	2.87273E-08	3.88462E-08	6.27692E-08	3.33077E-08	1.90769E-08	2.16364E-08	0.000000028	1.43455E-07	1.13909E-07	1.006E-07	6.80667E-08	1.682E-07	3.37333E-08
Tsukamurella_paurometabola	4.60909E-08	1.04545E-08	0	5.27909E-08	8.78462E-08	2.36846E-07	5.54615E-08	1.73385E-07	3.10364E-07	8.02727E-08	2.05455E-08	1.21727E-07	0.000000106	1.038E-07	6.83333E-08	1.85867E-07
Tsukamurella_tyrosinosolvens	2.60455E-07	0	0	1.06455E-07	0	9.48462E-08	0	0	1.57091E-07	2.85455E-08	2.18091E-07	1.45545E-07	0	0	4.64E-08	3.20667E-08
Dietzia_sp._oral_taxon_368	3.11818E-08	0	0	0	0	0	0	0	0	0	0	0	0	1.61333E-08	0	0
Dietzia_psychralcaliphila	8.80909E-08	0	3.27273E-08	3.49818E-07	0	1.31846E-07	0	8.01538E-08	2.64545E-08	5.22727E-08	1.41273E-07	1.41545E-07	2.44E-08	2.38E-08	0	9.33333E-09
Dietzia_lutea	5.08545E-07	1.60045E-07	1.83127E-07	3.19455E-07	2.92615E-07	2.16923E-08	2.54077E-07	0.000000121	2.18455E-07	6.97909E-07	3.06636E-07	0.000000375	4.00667E-08	1.78267E-07	6.384E-08	1.27533E-07
Dietzia_timorensis	1.52909E-07	2.32727E-08	1.37764E-07	2.54727E-07	3.15385E-08	3.21538E-07	6.41538E-08	9.16154E-08	0.000000216	2.11455E-07	1.22455E-07	4.64818E-07	6.53333E-08	5.12E-08	6.76E-08	9.74E-08
Segniliparus_rotundus	0	0	0	7.93364E-08	1.88846E-07	2.95385E-08	1.32769E-07	2.11923E-07	2.75455E-08	0.000000028	0	1.27818E-07	2.97533E-07	3.08867E-07	7.154E-07	4.226E-07
Gordonia_iterans	0	0	0	4.50909E-08	0	8.84615E-08	0	0	3.98182E-08	8.56364E-08	0	0	0	0.000000014	0	0
Gordonia_polyisoprenivorans	0	0	0	0	0	0	6.41538E-08	0	0	5.22727E-08	0	0	0	0	0	0
Gordonia_rubripertincta	1.85273E-07	0	0	3.35455E-08	2.75385E-08	1.79231E-08	2.73846E-08	7.03846E-08	3.75455E-08	1.88182E-07	0	3.94545E-08	8.47333E-08	8.12E-08	8.86667E-09	1.19133E-07
Gordonia_sp._JH63	0	0	0	5.16364E-08	0	0	5.74615E-08	3.57692E-08	0	0	0	0	0	0	0	4.28E-08
Gordonia_sp._HY186	5.57273E-08	1.16364E-08	0	0	4.11538E-08	6.67769E-07	1.10385E-07	1.46923E-08	0	0	1.30909E-07	0	0	0	4.44E-09	0
Gordonia_insulae	0	0	3.70909E-08	0	0	7.29231E-08	7.92308E-08	0	7.50909E-08	1.82545E-07	0.000000048	5.64545E-08	8.28E-08	5.47333E-08	5.04667E-08	0
Gordonia_phthalatica	0	0	3.70909E-08	3.24545E-08	0	0	0	0	0	0	0	7.13636E-09	0	0	6.46667E-08	0
Gordonia_bronchialis	1.07818E-07	3.87273E-08	3.70909E-08	1.89609E-07	1.41231E-07	0.000000115	4.08769E-07	7.69231E-08	1.98545E-07	2.19727E-07	3.77273E-08	2.76364E-07	1.06933E-07	2.76733E-07	1.98333E-07	1.674E-07
Gordonia_terrae	1.78818E-07	0.000000065	0	1.74636E-07	0	2.57308E-07	9.77692E-08	1.29308E-07	0	0	1.68818E-07	2.85455E-08	0	0	2.09733E-08	1.144E-07
Gordonia_sp._KTR9	1.65182E-07	0	0	0.000000081	3.73846E-08	8.30769E-08	0	3.57692E-08	6.45455E-08	0.000000028	1.40091E-07	1.13545E-07	2.37333E-08	4.59333E-08	8.33333E-08	1.98667E-08
Gordonia_sp._YC-JH1	2.51909E-07	0	1.84027E-06	2.26545E-06	4.48538E-07	0	5.45077E-06	5.18923E-06	1.15391E-06	0.000000748	6.63455E-07	4.70909E-07	4.074E-07	4.69333E-07	1.0368E-06	7.87267E-07
Nocardia_sp._CS682	6.80909E-08	0	0	1.64273E-07	2.88462E-08	1.65154E-07	1.75385E-07	9.03846E-08	5.79091E-08	6.37273E-08	0	4.21364E-08	2.64667E-08	2.32467E-07	3.81733E-08	2.92E-08
Nocardia_seriolae	0	2.84545E-08	0	4.79091E-09	1.23923E-07	3.10692E-07	5.26154E-08	2.93846E-08	7.73636E-08	3.62727E-08	2.05455E-08	2.85455E-08	0	0	1.65333E-08	4.28E-08
Nocardia_sp._WCH-YHL-001	3.62727E-08	0	0	5.68182E-08	0	5.41385E-07	1.97308E-07	1.73692E-07	6.62727E-08	0	6.00909E-08	1.04636E-07	6.86E-08	1.63067E-07	1.032E-07	1.284E-07
Nocardia_farcinica	5.82727E-08	0	0	2.07273E-08	1.32077E-07	0	7.92308E-08	0	0	1.02636E-07	1.70455E-07	5.37273E-08	1.48533E-07	3.50667E-08	5.61333E-08	5.70667E-08
Nocardia_nova	6.08182E-08	0	1.63636E-08	0	4.13846E-08	5.19231E-08	1.04769E-07	5.81538E-08	3.94545E-08	0.000000028	1.02727E-08	5.64545E-08	5.28667E-08	0.00000007	0	0
Nocardia_wallacei	1.43045E-06	0.00000039	0	6.32609E-07	3.41077E-07	7.36154E-08	4.90615E-07	1.57385E-06	6.08727E-07	4.46273E-07	8.30455E-07	9.31636E-07	0.000000592	4.78267E-07	0.000000524	5.20467E-07
Nocardia_otitidiscaviarum	1.58182E-08	1.77273E-08	3.55455E-08	5.71818E-08	0	0	0	7.28462E-08	0	2.85455E-08	0	0	2.14E-08	5.12E-08	6.46667E-08	1.86E-08
Nocardia_arthritidis	0.000000357	3.04091E-07	3.68545E-08	6.29091E-08	5.45385E-08	2.97615E-07	1.19308E-07	1.25385E-07	1.30727E-07	3.89091E-08	1.70636E-07	0.00000032	2.94667E-08	1.58E-08	1.0044E-07	7.95333E-08
Nocardia_cyriacigeorgica	1.65273E-07	0.000000065	9.45818E-08	2.23091E-07	2.51385E-07	2.13846E-08	3.11538E-08	1.59385E-07	3.75455E-08	8.26364E-08	3.01545E-07	1.23727E-07	5.04E-08	1.18067E-07	6.38E-08	1.956E-07
Nocardia_asteroides	0	0	2.91273E-08	0	0	0	0	0	7.50909E-08	0	0	0	0	0.000000033	0	1.98667E-08
Nocardia_yunnanensis	1.36636E-07	4.02727E-08	1.17182E-07	9.54545E-09	2.22308E-07	2.52308E-08	0	8.38462E-08	0.000000054	0	3.95455E-08	3.32091E-07	7.32E-08	2.92133E-07	2.562E-07	3.06467E-07
Nocardia_terpenica	3.69091E-08	0	0	0	0	0	0	0	2.75455E-08	0	1.02727E-08	7.12727E-08	0	1.29267E-07	6.54E-08	0
Nocardia_brasiliensis	4.29091E-08	0	3.70909E-08	4.99091E-08	3.88462E-08	4.82615E-07	6.96923E-08	1.27538E-07	2.07545E-07	2.06273E-07	1.51455E-07	3.16364E-08	1.17933E-07	0.000000069	4.40667E-08	0.000000128
Nocardia_mangyaensis	9.11818E-08	2.47364E-07	1.63636E-08	1.17791E-07	9.19231E-08	0	3.06923E-08	5.81538E-08	3.75455E-08	8.70909E-08	1.50364E-07	4.47091E-07	4.00667E-08	1.654E-07	1.93733E-08	0
Rhodococcus_sp._008	0	0	0	0	0	0	0	0	0	0	0	0	0	0	0	1.98667E-08
Rhodococcus_aetherivorans	0	1.68182E-08	0	0	0	0	0	0	0	0	0	0	0	0	0	1.22E-08
Rhodococcus_sp._BH4	0	0	0	5.18182E-09	0	0	0	0	0	0	0	1.42727E-08	0	0	0	0
Rhodococcus_imtechensis	0	0	0	4.20909E-08	0	0	0	0	0	0	0	0	0	0	0	0
Rhodococcus_sp._B7740	0	0	0	0	0	0	4.33846E-08	0	0	0	0	0	0	0	0	0
Rhodococcus_sp._YL-1	0	0	0	0	0	1.03692E-07	0	3.57692E-08	0	0	0	0	0	2.38E-08	3.27333E-08	0
Rhodococcus_sp._M8	0	0	0	0	0	0	3.33077E-08	0	0	0	0	0	0	0	0	0
Rhodococcus_sp._2G	0	0	0	4.79091E-09	0	0	0	0	0	0	0	3.87727E-08	0	0	5.10667E-08	0
Rhodococcus_jostii	0	0	0	2.43636E-08	6.73846E-08	6.53077E-08	2.63077E-08	1.46923E-08	0.000000071	2.87273E-08	0	0.000000154	2.64E-08	0	1.18667E-07	1.99267E-07
Rhodococcus_sp._S2-17	3.62727E-08	1.89364E-07	2.87273E-08	0	0	0	0	5.69231E-08	0	0	0	7.13636E-09	0	0	0	4.28E-08
Rhodococcus_sp._WAY2	3.11818E-08	0	0	4.02727E-08	0	0	0	0	2.92727E-07	3.89091E-08	1.30909E-07	2.51273E-07	4.17333E-08	4.86667E-08	1.8684E-07	1.33333E-07
Rhodococcus_sp._WB9	1.18091E-07	7.54545E-08	0	4.79091E-09	0	0	0	0	0.000000027	5.22727E-08	0	1.51091E-07	4.58667E-08	0	1.10867E-07	0
Rhodococcus_opacus	3.11818E-08	1.39545E-07	0	0	0	8.53846E-08	0	0	5.97273E-08	6.92727E-08	6.88182E-08	2.85455E-08	0	0	0	0
Rhodococcus_sp._H-CA8f	1.67273E-07	0	0	0.000000181	2.02692E-07	0	0	2.51538E-07	1.95182E-07	0	6.88182E-08	7.55455E-08	0.000000062	0.000000041	3.88E-08	1.63267E-07
Rhodococcus_sp._AQ5-07	0	0	0	0	0	0	0	0	5.79091E-08	0	2.76364E-08	5.50455E-08	2.14E-08	0	0	0
Rhodococcus_pyridinivorans	2.05636E-07	0	0	0.000000066	0	0	0	0	0	7.55455E-08	3.95455E-08	0	0.000000027	0	2.78E-08	0
Rhodococcus_hoagii	1.28909E-07	0.000000065	0	9.90909E-08	7.90769E-08	1.49923E-07	8.66154E-08	1.83923E-07	0	3.62727E-08	1.18636E-07	2.67727E-07	4.88667E-08	1.19533E-07	1.09467E-07	8.32E-08
Rhodococcus_sp._PBTS_2	0	0	0	0	4.13846E-08	7.43077E-08	0	5.81538E-08	5.97273E-08	0	3.95455E-08	0	0	0	0	0
Rhodococcus_sp._W8901	3.62727E-08	9.54545E-09	0	1.03636E-08	0	0	0	1.46923E-08	0	0.000000028	3.95455E-08	5.50455E-08	0	0	2.39333E-08	0
Rhodococcus_coprophilus	8.56364E-08	0	2.96364E-08	9.97273E-09	0	0	0	9.03846E-08	1.45636E-07	0	0.000000322	0.00000014	2.91333E-08	6.49333E-08	5.264E-08	1.51667E-07

Rhodococcus_ruber	2.73182E-07	0.000000065	4.23273E-07	8.55182E-08	1.11462E-07	5.00308E-07	4.02308E-07	0.000000932	5.46364E-08	2.87273E-08	9.42727E-08	1.43455E-07	1.086E-07	2.22867E-07	0.000000168	1.47733E-07
Rhodococcus_rhodochrous	2.01909E-07	0.000000022	4.92182E-08	9.97273E-09	0	3.11538E-07	0	0.000000107	3.13909E-07	1.27636E-07	0	7.44545E-08	1.14667E-07	0.000000028	3.79333E-08	4.04E-08
Rhodococcus_fascians	8.63636E-07	3.01818E-07	2.22309E-07	1.91273E-07	1.50923E-07	9.02308E-08	2.95769E-07	1.56615E-07	5.05545E-07	0.000000559	7.45364E-07	6.69818E-07	0	9.96667E-08	2.09333E-07	0.000000228
Rhodococcus_erythropolis	2.56364E-08	3.35455E-08	8.25455E-09	0	5.90769E-08	0	0	0	0	0	0	0	0	0	0	0
Rhodococcus_sp._MTM3W5.2	0.000000151	1.68182E-08	8.25455E-09	2.91545E-08	1.18769E-07	0	4.33846E-08	0	2.75455E-08	3.89091E-08	0	2.39273E-07	2.14667E-08	1.046E-07	7.43333E-08	1.86667E-08
Rhodococcus_triatomae	2.01545E-07	0	0	2.557E-07	7.99231E-08	0	4.33846E-08	4.64615E-08	0.000000177	1.99818E-07	1.42636E-07	0.000000414	0	2.43733E-07	8.27333E-08	8.32E-08
Rhodococcus_sp._PBTS_1	5.75273E-07	1.59818E-07	1.97636E-06	0.000001976	3.40692E-07	2.04154E-07	4.48331E-06	4.59477E-06	0.000000536	8.57455E-07	4.90455E-07	1.20191E-06	0.000000346	5.58133E-07	8.77467E-07	2.42333E-07
Rhodococcus_sp._WMMA185	5.67273E-08	0	0	5.18182E-09	5.63846E-08	6.53077E-08	4.33846E-08	0.000000088	1.12182E-07	7.25455E-08	3.77273E-08	1.47636E-07	4.48667E-08	6.78E-08	1.45933E-07	0.000000203
Rhodococcus_sp._ABRD24	0.000000104	8.42273E-08	0.000000035	2.95455E-08	3.58462E-08	9.46154E-09	3.77692E-08	2.56923E-08	9.77273E-08	1.74636E-07	3.67364E-07	8.60455E-08	2.24E-08	1.57067E-07	5.47067E-08	5.66E-08
Rhodococcus_sp._SGAir0479	1.04636E-06	0	2.41909E-06	3.14182E-06	5.10692E-07	4.13846E-08	6.14385E-06	6.57023E-06	1.24809E-06	8.55818E-07	1.01155E-06	1.08218E-06	6.68067E-07	5.644E-07	1.27833E-06	5.004E-07
Rhodococcus_sp._X156	3.06845E-06	8.52818E-07	2.33057E-06	3.02018E-06	6.04846E-07	3.73008E-06	4.66277E-06	5.48308E-06	2.03336E-06	2.14382E-06	3.05973E-06	3.65409E-06	4.43667E-07	1.2554E-06	1.09113E-06	9.322E-07
Rhodococcus_sp._P1Y	1.69364E-07	1.68182E-08	0.000000046	2.54636E-07	1.03308E-07	0	1.29538E-07	3.54231E-07	1.66727E-07	1.27727E-07	3.15182E-07	3.86909E-07	2.00933E-07	2.16667E-07	1.77707E-07	2.528E-07
Mycolicibacillus_koreensis	2.77727E-07	2.01045E-07	1.04455E-07	1.61182E-07	0	0	7.45385E-08	1.79769E-07	0.000000106	0.000000034	1.37273E-07	0.000000092	2.64E-08	0	4.44E-09	1.22E-08
Mycolicibacter_terrae	0	9.20909E-08	0	9.54545E-09	0	6.76923E-08	0	0	3.75455E-08	0	0	7.13636E-09	0	0	0	0
Mycolicibacter_minnesotensis	0	0.000000065	0	2.89091E-08	8.79231E-08	3.15385E-08	2.63077E-08	1.35231E-07	9.77273E-08	2.85455E-08	0	2.20636E-07	5.64667E-08	0	2.62E-08	1.19733E-07
Mycolicibacter_hiberniae	0	0	0	6.12727E-08	5.93846E-08	0	9.46923E-08	1.42462E-07	1.45545E-07	8.26364E-08	3.95455E-08	1.42727E-08	2.94667E-08	0	4.49067E-08	1.27667E-07
Mycolicibacter_sinensis	2.56364E-08	0	0	4.79091E-09	0	0	2.73846E-08	1.00154E-07	0	0	1.39182E-07	8.26818E-08	5.58667E-08	2.38E-08	2.01333E-08	5.96667E-08
Hoyosella_subflava	2.96364E-08	0	1.11818E-07	1.29909E-07	2.75385E-08	0	0	8.28462E-08	0	0	0	9.64545E-08	0	0.000000061	0	1.01933E-07
Mycobacteroides_salmoniphilum	0	5.52727E-08	0	1.47273E-08	0	0	0	0	8.23636E-08	0	0	0	0	0	0	4.95333E-08
[Mycobacterium]_stephanolepidis	3.11818E-08	0	0	3.43364E-08	0	9.46154E-09	0	0	0	0	0	0	0	0	0	1.22E-08
Mycobacteroides_saopaulense	0	0	0	0	0	0	0	3.22308E-08	9.21818E-08	3.89091E-08	6.71818E-08	1.17182E-07	2.14E-08	0.00000007	1.49333E-08	0
Mycobacteroides_immunogenum	1.20727E-07	1.02091E-07	1.02818E-07	1.50818E-07	1.07692E-07	6.41538E-08	0	9.39231E-08	0.000000027	1.14818E-07	1.75909E-07	1.42727E-08	7.46667E-08	3.50667E-08	1.12667E-08	9.33333E-09
Mycobacteroides_chelonae	1.08436E-06	0.000000065	4.76455E-07	1.66436E-06	1.68692E-07	0	1.63308E-07	5.48462E-07	1.14409E-06	4.41818E-07	0.000000721	1.39491E-06	2.76467E-07	0.000000685	5.21067E-07	0.000000415
Mycobacteroides_abscessus	0.00001018	3.27909E-06	1.21881E-05	9.61427E-06	1.38438E-06	1.20169E-06	1.09341E-05	9.92877E-06	3.78336E-06	6.06527E-06	6.06636E-06	8.55182E-06	1.10453E-06	3.9776E-06	3.05947E-06	2.33807E-06
Mycolicibacterium_monacense	0	0.00000013	0	0	0	3.83077E-08	0	0	0	0	0	0	0	1.61333E-08	0	0
Mycolicibacterium_chitae	0	0	0	0	0	0	0	3.22308E-08	0	0	0	1.42727E-08	0	0	0	0
Mycolicibacterium_celeriflavum	0	0	0	0	0	0	3.06923E-08	0	0	0	0	0	0	0	0	0
Mycolicibacterium_doricum	2.56364E-08	0	0	2.43636E-08	0	0	0	0	0	0	0	0	2.14667E-08	0	1.65333E-08	0
Mycolicibacterium_aichiense	0	0	0	7.11818E-08	3.58462E-08	0	0	0	0	0	0	0	2.72E-08	9.18667E-08	5.94E-08	9.33333E-09
Mycolicibacterium_anyangense	0	1.68182E-08	0	1.11182E-07	5.63846E-08	0	8.28462E-08	0	3.98182E-08	0	0	0	0	1.71333E-08	0	9.38E-08
Mycolicibacterium_psychrotolerans	0	1.16364E-08	0	7.25455E-08	0.000000065	5.05077E-07	2.63077E-08	0	0.000000104	6.37273E-08	0	7.07273E-08	0	1.29133E-07	7.26667E-08	4.76667E-08
Mycolicibacterium_pulveris	0	0	0	4.79091E-09	0	0	3.11538E-08	2.93846E-08	4.45455E-08	0	6.68182E-08	4.21364E-08	1.988E-07	1.00333E-07	1.45573E-07	1.104E-07
Mycolicibacterium_phocaicum	3.11818E-08	0	0	0	0	0	0	0	0	5.22727E-08	0	7.13636E-09	0	0	0	0
Mycolicibacterium_vaccae	1.15273E-07	0	0	0	0	0	0	0	1.49455E-07	0	0	9.27273E-08	0	0	1.49333E-08	0
Mycolicibacterium_mageritense	0	1.08182E-07	0	2.39091E-08	0	3.15385E-08	0	0	0	0.000000047	6.88182E-08	0	9.20667E-08	1.17867E-07	3.59067E-08	1.51067E-07
Mycolicibacterium_boenickei	3.11818E-08	0	0	1.43636E-08	0	0	0	0	3.75455E-08	0	1.37273E-07	0	2.09333E-08	0	0	0
Mycolicibacterium_insubricum	0	0	0	0	0	0	0	1.90769E-08	0	0	1.02727E-08	0	0	0	2.99333E-08	7.33333E-08
Mycolicibacterium_alvei	0	0	0	2.41545E-08	2.05385E-08	7.65385E-08	4.33846E-08	0	0	0	3.79091E-08	4.28182E-08	2.14667E-08	8.61333E-08	4.44E-09	1.86E-08
Mycolicibacterium_aubagnense	2.56364E-08	0	0	5.18182E-09	2.59231E-08	0	0	0.000000022	2.36091E-07	5.83636E-08	4.81818E-08	1.62818E-07	0	6.18E-08	1.372E-07	1.71467E-07
Mycolicibacterium_helvum	0.000000088	2.04091E-07	0	0.000000155	9.89231E-08	0	1.07538E-07	0	1.41545E-07	2.44364E-07	5.52727E-08	3.63636E-07	1.12333E-07	6.71333E-08	1.03933E-07	1.842E-07
Mycolicibacterium_phlei	8.49091E-08	0	0	0	8.36154E-08	0	2.63077E-08	0	0	5.72727E-08	6.65455E-08	0	0	0	4.93067E-08	5.58667E-08
Mycolicibacterium_arabiense	0	0	0	5.18182E-09	0	0	0	0	0	0	7.90909E-08	1.42727E-08	0	0	1.626E-07	0
Mycolicibacterium_vanbaalenii	9.97273E-08	0	3.70909E-08	2.03364E-08	0	7.21538E-08	2.63077E-08	0	0	5.22727E-08	0	1.39136E-07	0	0	8.86667E-09	0
Mycolicibacterium_parafortuitum	6.60909E-08	0	1.63636E-08	1.93636E-08	0	5.66923E-07	1.76538E-07	3.57692E-08	6.68182E-08	0	1.22455E-07	1.42727E-08	0	0.000000033	5.04667E-08	0
Mycolicibacterium_smegmatis	2.58909E-07	1.04545E-08	1.63636E-08	6.78818E-08	1.14923E-07	0.000000134	0.000000094	0.000000154	7.95455E-08	2.43182E-07	1.52818E-07	9.51818E-08	1.66133E-07	1.14867E-07	1.95867E-07	8.86667E-08
Mycolicibacterium_litorale	0.000000152	7.83636E-08	2.47273E-08	7.28182E-08	4.11538E-08	0	3.73462E-07	3.22308E-08	4.45455E-08	0	2.21545E-07	3.57273E-08	4.61333E-08	0.000000041	1.16933E-07	8.44E-08
Mycolicibacterium_fallax	9.27273E-08	0.000000335	3.70909E-08	1.38518E-07	1.02385E-07	0	0	2.46923E-08	3.11545E-07	0.00000014	1.85273E-07	1.42727E-08	4.00667E-08	0	2.90667E-08	8.66E-08
Mycolicibacterium_frederiksbergense	1.58182E-08	2.63636E-08	0	2.43636E-08	0	1.76615E-07	3.11538E-08	0	0	0	0	0	0	0	0	1.86E-08
Mycolicibacterium_hassiacum	5.31091E-07	0	1.10909E-07	1.44455E-07	9.46154E-08	0.00000072	1.10385E-07	2.46923E-08	0	8.26364E-08	1.55909E-07	2.16636E-07	6.97333E-08	1.61333E-08	1.226E-07	0.000000168
Mycolicibacterium_aurum	0	0	0	0	0	0	0	3.81538E-08	0	1.75545E-07	1.49091E-07	4.65909E-08	2.44E-08	0	1.49333E-08	1.22E-08
Mycolicibacterium_confluentis	0.000000128	8.86364E-09	4.94545E-08	1.93636E-08	0	1.79231E-08	0	2.46923E-08	2.64545E-08	0	1.57455E-07	9.75E-08	0	0	0	1.66667E-08
Mycolicibacterium_thermoresistibile	0	8.84545E-08	3.70909E-08	1.93636E-08	1.28077E-07	0	0	3.57692E-08	0	0	1.64273E-07	7.51818E-08	2.08E-08	3.50667E-08	5.264E-08	3.33333E-08
Mycolicibacterium_fluoranthenivorans	3.22636E-07	1.61909E-07	7.40909E-08	7.23636E-08	1.89308E-07	4.13846E-08	2.56923E-07	1.03692E-07	2.32182E-07	2.96182E-07	5.38091E-07	4.31545E-07	1.784E-07	1.07133E-07	4.93067E-08	2.328E-07
Mycolicibacterium_gilvum	1.80727E-07	0	0	1.92909E-07	1.26769E-07	1.04615E-07	2.93846E-07	4.08231E-07	6.61818E-08	3.12727E-07	0.000000416	3.29182E-07	6.52667E-08	1.86467E-07	1.93733E-08	1.39067E-07
Mycolicibacterium_moriokaense	9.17364E-07	0	4.61636E-07	7.67636E-07	3.88692E-07	1.83531E-06	2.01077E-07	9.22308E-08	6.36182E-07	2.29636E-07	4.53182E-07	3.54182E-07	5.104E-07	3.76933E-07	7.72333E-07	4.05333E-07
Mycolicibacterium_madagascariense	2.96364E-08	0	1.77273E-08	8.17273E-08	3.58462E-08	1.54385E-07	2.63077E-08	2.10077E-07	2.16364E-08	2.85455E-08	8.02727E-08	7.95909E-08	2.14E-08	1.71333E-08	2.39333E-08	1.346E-07
Mycolicibacterium_sediminis	2.00636E-07	8.84545E-08	5.04545E-08	0.000000256	1.46615E-07	1.26154E-07	2.44077E-07	0.000000263	2.64545E-08	0	6.53636E-08	3.85182E-07	1.27067E-07	1.71733E-07	2.41267E-07	1.338E-07
Mycolicibacterium_rutilum	5.22909E-07	8.49091E-08	1.80218E-07	0.000000775	4.87615E-07	1.03308E-07	4.33538E-07	8.69308E-07	1.21282E-06	5.18727E-07	8.93545E-07	1.16755E-06	7.34867E-07	9.69333E-07	4.28667E-07	2.94333E-07
Mycolicibacterium_poriferae	3.69091E-08	2.08182E-08	4.12182E-08	5.18182E-09	3.58462E-08	8.44615E-08	4.33846E-08	1.46923E-08	4.32727E-08	1.25182E-07	0.000000061	3.57273E-08	0	4.70667E-08	1.26867E-07	1.192E-07
Mycolicibacterium_tokaiense	1.59545E-07	0.000000065	3.59855E-07	0.000000199	5.63846E-08	0	2.65077E-07	5.63769E-07	0.000000081	2.85455E-08	1.16273E-07	2.07364E-07	1.362E-07	6.20667E-08	8.36E-08	1.44267E-07
Mycolicibacterium_rhodesiae	1.28273E-07	0	0	3.24545E-08	0	0	1.07462E-07	0.000000066	3.17727E-07	0	0	1.67273E-07	1.508E-07	1.41533E-07	9.46E-08	1.29867E-07
Mycolicibacterium_chubuense	1.48727E-07	0	0	3.42727E-08	5.63846E-08	7.21538E-08	1.69846E-07	1.11077E-07	1.74364E-07	2.85455E-08	6.88182E-08	5.17273E-08	0	0.000000086	1.79333E-07	1.002E-07
Mycolicibacterium_goodii	6.25455E-08	1.68182E-08	0	5.18182E-09	0	5.12308E-08	1.05385E-07	7.39231E-08	5.79091E-08	3.03636E-08	1.43273E-07	7.48636E-07	7.46E-08	8.18667E-08	5.99067E-08	1.534E-07
Mycolicibacterium_fortuitum	1.62273E-07	0	4.12727E-09	4.94545E-08	0.000000162	0	0	1.90769E-08	1.77545E-07	0.000000093	1.61364E-07	8.70455E-07	6.90667E-08	1.99867E-07	8.66667E-08	1.006E-07
Mycolicibacterium_sarraceniae	1.79273E-07	1.94545E-07	2.53909E-08	0.000001239	9.89231E-08	1.38154E-06	2.20392E-06	1.74223E-06	3.23273E-07	4.12636E-07	5.75909E-07	2.61455E-07	7.95333E-08	9.78667E-08	0.000000183	1.98667E-08
Mycolicibacterium_duvalii	6.24545E-08	3.45455E-08	1.11073E-07	6.48182E-08	8.52308E-08	6.54154E-07	2.08231E-07	1.06538E-07	3.27545E-07	1.25182E-07	2.56364E-07	2.33182E-07	6.94667E-08	3.47067E-07	2.36467E-07	0.00000034
Mycobacterium_seoulense	0	0	0	0	0	0	0	0	0	0	0	0	4.86E-08	6.80667E-08	0	0
Mycobacterium_marseillense	5.57273E-08	0	0	0	0	0	0	0	0	0	0	0	0	0	0	0
Mycobacterium_kubicae	0	0	0	9.54545E-09	1.26154E-07	0	0	1.68385E-07	0	0	0	1.86818E-07	0	0	0	0
Mycobacterium_simiae	0	0	0	5.16364E-08	0	0	0	0	0	0	0	0	0	0	0	0
Mycobacterium_marinum	1.11818E-07	0	0	0	0	0	0	2.47692E-08	0	0.000000047	0	0	2.09867E-07	3.09333E-08	1.49333E-08	0
Mycobacterium_conspicuum	0	0	0	0	0	0	0	4.10769E-08	2.16364E-08	0	0	4.65909E-08	2.09333E-08	3.09333E-08	4.83067E-08	3.37333E-08
Mycobacterium_basiliense	5.68182E-08	1.16364E-08	0	4.79091E-09	0	0	1.15077E-07	1.24846E-07	0	8.62727E-08	0	1.42727E-08	0	6.60667E-08	2.24667E-08	5.39333E-08
Mycobacterium_florentinum	0	0	0	1.03636E-08	3.15385E-08	3.15385E-08	0	7.72308E-08	5.79091E-08	0	6.68182E-08	1.29273E-07	0	0	0	0
Mycobacterium_cookii	5.82727E-08	0	0	0	0	0.000000145	0	0	0	0	0	0	2.24E-08	0	0	0
Mycobacterium_shigaense	3.11818E-08	0	0	0	0	0	0	0	0	3.62727E-08	0	0	0	0	0	0
Mycobacterium_canettii	6.80909E-08	0	0	9.97273E-09	6.93077E-08	0	0.00000008	5.22308E-08	0	3.62727E-08	6.88182E-08	1.17273E-07	1.074E-07	1.58E-08	7.96E-08	9.33333E-09
Mycobacterium_grossiae	6.74545E-08	0	0	1.91818E-08	0.000000125	0	0	1.01692E-07	0	0.000000028	2.76364E-08	8.87273E-08	0	0	6.91067E-08	0
Mycobacterium_sp._KMS	2.03364E-07	3.28182E-08	0	7.97273E-08	9.40769E-08	1.21523E-06	1.10385E-07	4.03846E-08	1.94091E-07	1.11818E-07	2.76364E-08	1.42727E-08	1.60267E-07	7.64667E-08	1.98867E-07	1.21267E-07
Mycobacterium_ulcerans	0	0	0	0	0	0	0	0	0	0	6.65455E-08	0	0	0	0	0
Mycobacterium_sp._DL440	0	0	0	0	0	0	5.26154E-08	0	9.95455E-08	0	6.65455E-08	0	0	4.64667E-08	0	0
Mycobacterium_pseudoshottsii	0	0	0	0	1.86923E-08	0	0	0	2.64545E-08	0	3.77273E-08	0	6.88667E-08	1.40333E-07	4.93067E-08	8.62667E-08
Mycobacterium_lepraemurium	0	0	0	0	0	0	3.11538E-08	0	0	8.59091E-08	3.95455E-08	0	2.14E-08	3.50667E-08	4.44E-09	9.33333E-09
Mycobacterium_kansasii	0	0	0	1.47273E-08	0	2.13846E-08	3.33077E-08	0.000000011	0	5.22727E-08	4.98182E-08	2.85455E-08	0	0	0	0
Mycobacterium_stomatepiae	0	0	0	0	0	0	0	0	0	0	7.90909E-08	0	0	0	0	0
Mycobacterium_shinjukuense	3.62727E-08	1.16364E-08	3.70909E-08	1.38182E-07	9.07692E-08	1.55231E-07	2.02769E-07	3.00769E-08	4.45455E-08	0	0	6.21818E-08	8.23333E-08	1.31667E-07	1.73067E-07	1.46267E-07
Mycobacterium_dioxanotrophicus	0.000000061	0	7.40909E-08	1.19455E-07	0.000000122	0.0000003	0	9.53077E-08	7.50909E-08	1.04545E-07	6.15455E-08	3.26909E-07	0	0	1.45333E-07	9.33333E-09
Mycobacterium_heidelbergense	0	0	1.48182E-07	4.14545E-08	1.60846E-07	0.000000136	6.41538E-08	3.99538E-07	1.12727E-07	1.88455E-07	3.07273E-08	8.48818E-07	0	0	1.44867E-07	9.04667E-08
Mycobacterium_paraintracellulare	0	0	1.63636E-08	0	0	0	0	0	0	0	0	0	0	0	0	0
Mycobacterium_tuberculosis	1.58182E-08	0	0	0	0	0	0	0	0	0	0	2.85455E-08	0	0	4.44E-09	0
Mycobacterium_saskatchewanense	7.66364E-08	1.31182E-07	4.90909E-08	1.51545E-08	0	0	6.23077E-08	7.51538E-08	0	6.43636E-08	2.76364E-08	2.14545E-08	0	0	0	1.66667E-08
Mycobacterium_parmense	1.54727E-07	2.07636E-07	0	3.60091E-07	2.59231E-08	2.52308E-08	0	0	0	1.30636E-07	9.64545E-08	7.07273E-08	0	1.58E-08	4.44E-09	3.40667E-08
Mycobacterium_sp._EPa45	2.96364E-08	8.86364E-09	0	2.95455E-08	6.19231E-08	0	2.63077E-08	1.90769E-08	7.15455E-08	5.22727E-08	1.07909E-07	3.16364E-08	0	3.26667E-08	1.93733E-08	9.12667E-08
Mycobacterium_xenopi	1.81182E-07	1.06955E-07	0	5.64273E-08	1.82769E-07	8.90769E-08	7.45385E-08	1.35385E-07	5.97273E-08	3.89091E-08	5.39091E-08	4.37909E-07	4.61333E-08	8.96667E-08	4.83067E-08	0
Mycobacterium_avium	8.10909E-08	1.92273E-08	3.70909E-08	3.24545E-08	0	2.13846E-08	0	0	1.84545E-07	0	1.16273E-07	1.20364E-07	0	3.50667E-08	0	0
Mycobacterium_haemophilum	9.93636E-08	0	0	4.99091E-08	9.87692E-08	1.08077E-07	2.06385E-07	5.40077E-07	7.46364E-08	1.21636E-07	6.66364E-08	0.000000035	1.17067E-07	1.94733E-07	3.10267E-07	1.05867E-07
Mycobacterium_sp._DL90	6.59909E-07	1.74182E-07	2.39091E-07	2.73545E-07	2.23692E-07	6.43077E-08	8.15385E-08	6.32308E-08	4.68545E-07	8.54455E-07	0.000001069	1.31009E-06	1.416E-07	1.76733E-07	7.45067E-08	7.26667E-08
Mycobacterium_gordonae	5.09636E-07	2.77727E-07	9.54545E-08	2.99909E-07	2.77923E-07	6.53077E-08	5.74615E-08	3.51231E-07	4.69364E-07	2.48091E-07	5.24818E-07	2.85545E-07	1.308E-07	1.58E-08	1.60267E-07	1.95533E-07
Mycobacterium_paraseoulense	2.56364E-08	0	0	4.79091E-09	0.000000099	1.27385E-07	0	0	0.000000027	6.08182E-08	1.46818E-07	3.57273E-08	0	0	5.95733E-08	0
Mycobacterium_sp._MS1601	1.50727E-07	0	0	1.66091E-07	2.19692E-07	1.21238E-06	2.87538E-07	4.41538E-08	2.16364E-08	3.89091E-08	2.31727E-07	2.95182E-07	5.59333E-08	1.62867E-07	3.21867E-07	4.484E-07
Mycobacterium_gallinarum	0	0	4.12727E-09	0	0	2.13846E-08	0	0	4.45455E-08	3.89091E-08	7.90909E-08	4.59091E-08	0	0	1.57067E-08	1.98667E-08
Mycobacterium_paragordonae	1.52727E-07	2.74364E-07	8.72182E-08	2.86727E-07	0	0	3.06923E-08	3.37692E-08	1.20818E-07	5.22727E-08	0	2.29455E-07	0	1.61333E-08	7.09733E-08	0
Mycobacterium_sp._ELW1	8.36364E-08	0.000000198	1.44727E-07	1.13727E-07	3.34615E-08	1.10462E-07	7.92308E-08	0.000000042	0	5.22727E-08	1.35455E-07	0.000000085	0	3.11333E-08	0	0
Mycobacterium_sp._DL592	6.37636E-07	0	1.87973E-06	2.16964E-06	5.52923E-07	0	4.81308E-06	4.90631E-06	8.97636E-07	1.14782E-06	7.62455E-07	4.44182E-07	3.926E-07	1.92267E-07	7.086E-07	3.34333E-07
Mycobacterium_leprae	0	0	0	2.91545E-08	2.05385E-08	2.38769E-07	0	3.57692E-08	2.75455E-08	0	6.88182E-08	0.000000035	2.37333E-08	1.22267E-07	8.32E-08	3.67333E-08
Mycobacterium_noviomagense	9.39091E-08	0	0	3.83636E-08	0.000000078	6.56154E-08	2.63077E-08	3.72538E-07	8.91818E-08	0.000000047	7.72727E-08	1.47455E-07	0	3.48E-08	1.49333E-08	2.40667E-07
Mycobacterium_lacus	3.26091E-07	5.51455E-07	1.35545E-07	2.70091E-07	4.04308E-07	9.43077E-08	3.64692E-07	5.54692E-07	6.26182E-07	5.52182E-07	7.89091E-07	6.11091E-07	2.22533E-07	2.67467E-07	2.838E-07	6.77133E-07
Mycobacterium_mantenii	2.70909E-08	9.54545E-09	0	4.37273E-08	0	0	0	0	0	0	0	0.000000035	2.24E-08	1.02533E-07	2.62E-08	1.98667E-08
Mycobacterium_colombiense	1.89727E-07	0.000000065	6.30727E-08	1.94636E-07	1.52692E-07	6.18769E-07	3.62308E-07	2.53462E-07	1.08818E-07	1.98636E-07	4.51273E-07	2.00545E-07	0.000000408	0.000000522	3.178E-07	3.20267E-07
Mycobacterium_branderi	9.17273E-08	0	0.000000246	6.00636E-08	3.34615E-08	0	5.26154E-08	8.28462E-08	2.26727E-07	2.04455E-07	2.19182E-07	1.26909E-07	5.09333E-08	2.138E-07	2.53467E-07	1.69533E-07
Mycobacterium_sp._YC-RL4	1.50909E-07	2.71636E-07	7.40909E-08	1.42727E-07	9.83846E-08	0	1.12308E-07	1.20462E-07	4.98455E-07	2.22727E-07	4.86727E-07	2.03273E-07	4.10133E-07	0.000000264	2.70573E-07	2.66667E-07
Mycobacterium_sp._JS623	4.54545E-08	0	0	2.43636E-08	1.87254E-06	0	0	2.06385E-07	2.51818E-07	1.66545E-07	2.76364E-08	6.01636E-07	8.11133E-07	1.21713E-06	6.20733E-07	1.32887E-06
Corynebacterium_sp._L2-79-05	0	0	0	0	0	0	0	0	5.79091E-08	0	0	0	0	0	4.38667E-08	1.98667E-08
Corynebacterium_testudinoris	0	4.82727E-08	0	6.12727E-08	0	0	0.000000135	3.22308E-08	0	0	0	1.90364E-07	0	1.61333E-08	0	0
Corynebacterium_humireducens	2.98182E-08	0	0	0	0	6.10769E-08	0	0	0	0	0	0	0	0	0	0
Corynebacterium_mycetoides	9.11818E-08	0	0	2.43636E-08	1.86923E-08	0	3.11538E-08	1.44154E-07	0.000000089	5.90909E-08	1.30909E-07	3.74545E-08	9.26E-08	7.19333E-08	0.000000165	1.762E-07
Corynebacterium_aquilae	8.69091E-08	0	0	6.44545E-08	5.34615E-08	2.54538E-07	1.80462E-07	1.08769E-07	4.59455E-07	5.22727E-08	1.33636E-07	5.64545E-08	1.038E-07	0.000000014	1.65333E-08	0.000000055
Corynebacterium_kutscheri	0	0	0	1.91818E-08	3.58462E-08	0	0	0.000000053	0	1.14455E-07	1.35636E-07	0	2.08E-08	0	4.44E-09	1.86E-08
Corynebacterium_epidermidicanis	1.11818E-07	0	0	2.45455E-08	0	0	0	8.79231E-08	8.13636E-08	2.87273E-08	6.88182E-08	1.42727E-08	4.48667E-08	0	0.000000041	3.67333E-08
Corynebacterium_renale	0	0	0	0	3.34615E-08	0	0	0	5.79091E-08	2.85455E-08	1.02727E-08	0	0	0	8.86667E-09	0
Corynebacterium_resistens	0	0	0	1.76273E-07	3.15385E-08	0	0	1.09462E-07	1.51182E-07	0	9.44545E-08	7.13636E-09	0	0	8.03733E-08	5.96667E-08
Corynebacterium_singulare	0	1.04545E-08	0	5.18182E-09	0	0	3.33077E-08	2.93846E-08	0	0	1.04545E-07	7.13636E-09	2.94667E-08	1.58E-08	4.82E-08	0
Corynebacterium_endometrii	2.79091E-07	1.53455E-07	0	1.88091E-07	0	0	5.74615E-08	1.90769E-08	1.95182E-07	1.45909E-07	4.09091E-07	0.000000115	0	0	4.44E-09	0
Corynebacterium_sp._zg-320	2.39182E-07	0	7.40909E-08	2.89091E-08	6.73846E-08	1.23846E-07	0	5.03846E-08	1.18273E-07	8.62727E-08	6.88182E-08	2.50909E-07	5.09333E-08	0	9.90667E-08	1.34267E-07
Corynebacterium_aurimucosum	3.69091E-08	0	2.96364E-08	3.07273E-08	8.15385E-08	3.83077E-08	3.01923E-07	0.000000011	4.94909E-07	0	2.76364E-08	2.22455E-07	9.58E-08	4.91333E-08	0.000000113	1.53067E-07
Corynebacterium_terpenotabidum	3.57636E-07	0	2.96364E-08	1.61091E-07	1.16308E-07	2.42538E-07	7.92308E-08	6.84615E-08	2.46182E-07	3.61727E-07	6.68182E-08	6.91273E-07	3.27333E-07	1.82533E-07	9.82E-08	2.92E-08
Corynebacterium_sanguinis	1.08182E-07	0	3.70909E-08	1.35636E-07	1.34231E-07	2.69846E-07	1.00385E-07	2.46923E-08	1.24364E-07	2.63636E-07	1.68818E-07	1.69818E-07	2.37333E-08	3.09333E-08	5.94E-08	6.86667E-08
Corynebacterium_simulans	1.58182E-08	1.66045E-07	3.70909E-08	4.76091E-08	2.05385E-08	2.13846E-08	6.23077E-08	3.26231E-07	1.76909E-07	0.000000181	1.32182E-07	1.57818E-07	6.60667E-08	1.12467E-07	9.82E-08	8.66667E-08
Corynebacterium_geronticis	1.96727E-07	1.03636E-08	0	2.80545E-07	2.26923E-08	0	4.33846E-08	0	0	0.000000047	8.29091E-08	2.85455E-08	1.02933E-07	1.58E-08	9.38667E-08	6.80667E-08
Corynebacterium_genitalium	2.04455E-07	0.000000065	1.26364E-07	4.61636E-07	0	0	6.44615E-08	9.04615E-08	0	5.22727E-08	2.76364E-08	4.92727E-08	2.64E-08	1.07667E-07	0	1.20467E-07
Corynebacterium_sp._2019	1.22727E-07	0.00000013	0.000000047	2.87818E-07	0	0	4.44231E-07	2.56923E-08	0	0	0	6.21818E-08	0	0	0	0
Corynebacterium_jeikeium	6.14727E-07	3.82136E-07	6.98182E-08	3.41909E-07	0.0000001	4.12615E-07	0.000000196	5.45154E-07	1.77273E-06	6.66364E-08	5.49455E-07	9.50818E-07	7.05333E-08	3.194E-07	4.73733E-07	4.04533E-07
Corynebacterium_sp._zg-917	1.23182E-07	0	0	3.87273E-08	1.58231E-07	0	9.29231E-08	1.77462E-07	4.45455E-08	0	8.02727E-08	0	2.64E-08	1.264E-07	1.276E-07	9.74E-08
Corynebacterium_glutamicum	2.98182E-08	7.38636E-08	7.66364E-08	1.05455E-07	1.74077E-07	6.41538E-08	1.36385E-07	9.35385E-08	6.53909E-07	7.51818E-08	2.44273E-07	1.98182E-07	0.00000008	6.59333E-08	2.99507E-07	2.59867E-07
Corynebacterium_deserti	0	0	0	1.18064E-07	0	0	0	6.45385E-08	1.06182E-07	1.99818E-07	3.33636E-08	0	2.14E-08	0	8.86667E-09	0
Corynebacterium_xerosis	0.00000006	3.79091E-08	0	7.75455E-08	1.44077E-07	3.15385E-08	6.96923E-08	0.000000042	1.15455E-07	1.16545E-07	4.93545E-07	3.57273E-08	1.86667E-08	1.58E-08	0	2.15333E-08
Corynebacterium_nuruki	0.000000156	1.43545E-07	0	2.03364E-07	2.04538E-07	2.48462E-07	7.40769E-08	1.46923E-08	2.65636E-07	1.22182E-07	1.29818E-07	0.000000157	2.09867E-07	0.000000154	1.51733E-07	1.86733E-07
Corynebacterium_ureicelerivorans	2.10818E-07	1.48727E-07	3.70909E-08	1.16873E-06	1.66154E-07	1.70415E-06	2.86162E-06	2.29269E-06	5.83909E-07	5.11182E-07	2.34727E-07	4.57545E-07	1.44333E-07	1.398E-07	2.47107E-07	9.33333E-09

Corynebacterium_callunae	1.58182E-08	1.68182E-08	3.70909E-08	1.11182E-07	3.34615E-08	3.83077E-08	1.51077E-07	0	4.26455E-07	2.41909E-07	5.10909E-07	4.57909E-07	4.23333E-08	0	0.000000265	1.546E-07
Corynebacterium_segmentosum	7.73273E-07	0.000000065	7.40909E-08	8.32364E-07	3.08462E-07	1.38231E-07	3.11538E-08	2.82846E-07	2.37182E-07	4.31364E-07	3.43273E-07	2.59455E-07	4.32E-08	1.87467E-07	2.308E-07	4.96267E-07
Corynebacterium_timonense	0.00000049	1.68182E-08	3.70909E-08	1.21455E-07	3.64385E-07	4.55692E-07	0.000000271	1.55308E-07	4.60273E-07	2.63909E-07	1.79091E-07	8.04545E-07	1.26933E-07	2.202E-07	8.60667E-08	7.56E-08
Corynebacterium_suranareeae	2.28545E-07	0.000000065	3.70909E-08	2.41545E-08	1.99154E-07	1.10292E-06	1.35077E-07	5.59615E-07	2.53364E-07	0.000000028	4.56455E-07	1.70909E-07	1.97267E-07	1.71467E-07	3.66867E-07	4.19333E-07
Corynebacterium_lactis	1.58182E-08	1.03636E-08	0	5.36364E-08	2.05385E-08	3.77462E-07	0	1.63923E-07	1.86909E-07	1.17909E-07	1.76364E-07	2.39636E-07	4.38E-08	1.52667E-07	1.72773E-07	9.14667E-08
Corynebacterium_urealyticum	3.11818E-08	0.000000065	0	8.99091E-08	2.70308E-07	0	1.80769E-07	1.41769E-07	9.77273E-08	3.62727E-08	0.000000078	2.18273E-07	1.58333E-07	1.59133E-07	9.87733E-08	2.02067E-07
Corynebacterium_mustelae	6.62364E-07	8.27273E-08	0	3.08182E-07	2.78231E-07	1.72869E-06	8.44615E-07	1.33838E-06	4.31727E-07	5.01364E-07	6.20545E-07	7.88636E-07	4.19133E-07	4.73467E-07	6.438E-07	6.088E-07
Corynebacterium_sp._1959	0.000000361	3.18636E-07	4.50909E-08	0	0	0	1.41154E-07	1.79077E-07	9.62727E-08	7.02727E-08	0.000000175	1.73682E-07	4.79333E-08	0	1.49333E-08	1.98667E-08
Corynebacterium_sp._LMM-1652	2.56364E-08	0	0	3.24545E-08	6.33846E-08	0	0	3.22308E-08	1.24273E-07	2.07182E-07	1.33182E-07	5.64545E-08	1.642E-07	0.000000033	4.91067E-08	2.79333E-08
Corynebacterium_sp._ATCC_6931	9.37273E-08	2.34545E-08	0	1.43636E-08	3.58462E-08	2.57692E-07	1.19615E-07	6.79231E-08	0.000000027	2.18455E-07	2.25182E-07	8.42727E-08	8.17333E-08	4.49333E-08	1.12667E-08	2.25667E-07
Corynebacterium_sp._NML93-0612	1.20073E-06	5.27727E-08	7.25545E-07	9.87364E-07	1.57154E-07	6.03846E-06	0.000001211	2.79646E-06	9.03455E-07	0.00000046	9.64727E-07	1.16473E-06	3.698E-07	4.176E-07	2.89133E-07	5.62733E-07
Corynebacterium_pseudopelargi	5.42727E-08	1.16364E-08	2.30209E-07	0.000000358	0	2.52308E-08	1.35538E-07	1.32692E-07	8.30909E-08	2.85455E-08	2.63909E-07	9.45455E-08	8.83333E-08	0.000000033	1.41267E-07	0.000000096
Corynebacterium_imitans	7.04091E-07	2.41455E-07	0.000000349	3.02091E-07	0.000000078	0	3.82769E-07	2.98846E-07	8.27273E-07	0	3.67455E-07	4.98182E-07	2.64E-08	0	6.30667E-08	0
Corynebacterium_sp._2039	1.58182E-08	0.000000065	8.75455E-09	4.79091E-09	0	2.25077E-07	0	0	3.98182E-08	0.000000028	0	3.31818E-07	0	3.50667E-08	2.99733E-08	6.81333E-08
Corynebacterium_sp._Marseille-Q3630	7.57273E-08	1.43409E-07	3.85455E-07	4.79091E-09	2.05385E-08	2.13846E-08	0	1.98462E-07	1.59182E-07	2.85455E-08	0.000000039	3.22636E-07	9.93333E-08	6.62667E-08	2.97333E-08	1.86E-08
Corynebacterium_minutissimum	0.000000482	2.43818E-07	9.59091E-08	1.83727E-07	8.09231E-08	1.79231E-08	3.24231E-07	3.96923E-07	4.06091E-07	1.11818E-07	2.06364E-07	0.0000003	6.08E-08	1.61333E-08	1.98067E-07	2.36067E-07
Corynebacterium_kroppenstedtii	6.11455E-07	0	1.00573E-07	4.38364E-07	3.07615E-07	0	5.88385E-07	4.41231E-07	6.89545E-07	0.000000328	2.96273E-07	4.33455E-07	1.464E-07	1.27733E-07	1.57067E-07	5.42067E-07
Corynebacterium_ulcerans	2.93545E-07	0.000000065	1.06727E-07	3.86273E-07	1.42538E-07	2.70923E-07	1.24615E-07	2.37923E-07	2.07364E-07	1.09636E-07	1.15364E-07	0.00000063	2.36667E-07	1.06933E-07	1.13267E-07	1.878E-07
Corynebacterium_frankenforstense	3.92909E-07	0	4.12182E-08	1.19636E-07	1.03231E-07	1.36992E-06	1.29385E-07	1.75692E-07	3.98182E-08	9.17273E-08	2.24727E-07	3.18182E-07	0	6.18E-08	1.93467E-07	1.26667E-07
Corynebacterium_phocae	2.23636E-07	2.79909E-07	2.11491E-07	1.71727E-07	9.80769E-08	7.15231E-07	2.91154E-07	0.000000639	0.00000025	1.86636E-07	2.16818E-07	2.93636E-07	1.232E-07	1.088E-07	1.08707E-07	2.69933E-07
Corynebacterium_marinum	1.53545E-07	2.11182E-07	2.18545E-08	1.90636E-07	2.05385E-08	7.66154E-08	0	2.02154E-07	7.94545E-08	1.61273E-07	1.02727E-08	2.47727E-07	7.87333E-08	0	2.55333E-08	6.33333E-08
Corynebacterium_pelargi	2.30909E-08	3.49091E-08	7.82182E-08	1.58909E-07	3.34615E-08	6.53077E-08	0	0.000000011	1.56182E-07	3.03636E-08	2.13091E-07	2.43545E-07	4.78E-08	2.138E-07	5.95733E-08	3.40667E-08
Corynebacterium_doosanense	1.18273E-07	0	8.25455E-09	4.79091E-09	3.34615E-08	3.83077E-08	2.26923E-07	4.67692E-08	0	2.85455E-08	1.37273E-07	0.000000035	4.94E-08	1.71333E-08	6.46667E-08	1.826E-07
Corynebacterium_pseudotuberculosis	0.000000198	0.000000065	8.23455E-08	3.24545E-08	2.05385E-08	0	4.13538E-07	8.70769E-08	1.17636E-07	8.02727E-08	3.95455E-08	1.42727E-08	0	6.66E-08	5.36667E-08	0
Corynebacterium_efficiens	5.16091E-07	7.66364E-08	4.53455E-08	1.43455E-07	5.63846E-08	2.71769E-07	2.08385E-07	8.76154E-08	0.000000184	1.87727E-07	6.71818E-08	1.50909E-07	1.23733E-07	1.244E-07	1.77773E-07	3.08933E-07
Corynebacterium_atypicum	4.95182E-07	1.36873E-06	2.14273E-07	6.18818E-07	1.67077E-07	2.78077E-07	9.84615E-08	3.33769E-07	1.90364E-07	3.21727E-07	3.92909E-07	1.21873E-06	6.59333E-08	7.35333E-08	2.966E-07	1.01533E-07
Corynebacterium_uterequi	1.91364E-07	1.03636E-08	1.35545E-07	1.00882E-07	0	0	1.34154E-07	2.05615E-07	5.97273E-08	0	1.29818E-07	3.47455E-07	2.14E-08	0.000000066	1.73067E-07	1.15333E-07
Corynebacterium_halotolerans	0.000000143	1.13273E-07	0.00000008	1.06727E-07	1.19077E-07	3.81538E-07	0.00000015	1.12231E-07	0	8.85455E-08	8.86364E-08	1.52273E-07	4.45333E-08	1.648E-07	8.11067E-08	4.46E-08
Corynebacterium_flavescens	9.22545E-07	9.22545E-07	2.01364E-07	7.89818E-07	3.88923E-07	5.46846E-07	9.50308E-07	1.03115E-06	1.13173E-06	5.09273E-07	1.00982E-06	1.75936E-06	2.59733E-07	4.11267E-07	4.04133E-07	6.664E-07
Corynebacterium_variabile	7.28818E-07	1.95727E-07	4.09664E-07	2.84909E-07	1.96231E-07	1.19615E-07	7.77923E-07	5.99615E-07	0.000000104	2.05909E-07	3.98182E-07	7.91727E-07	1.0976E-06	2.36667E-07	2.75933E-07	2.986E-07
Corynebacterium_sp._23H37-10	2.96364E-08	0	4.12727E-09	0	8.36154E-08	0	2.63077E-08	2.47692E-08	0	0.000000147	1.41182E-07	0	4.78667E-08	0	1.3644E-07	5.10667E-08
Corynebacterium_sp._2184	1.12991E-06	3.71818E-08	2.88582E-07	8.90273E-07	3.24692E-07	1.03538E-07	6.57846E-07	7.32846E-07	9.06273E-07	5.43273E-07	8.90545E-07	5.80545E-07	3.79933E-07	0.000000378	4.03533E-07	4.534E-07
Corynebacterium_sp._4H37-19	1.62373E-06	2.79364E-07	8.35455E-08	1.34409E-06	4.24154E-07	2.92923E-07	1.62308E-07	2.71923E-07	9.71909E-07	5.78818E-07	1.41773E-06	5.21636E-07	2.52267E-07	3.01867E-07	5.68667E-07	5.66467E-07
Corynebacterium_provencense	7.94455E-07	1.68455E-07	7.55091E-07	4.23727E-07	1.66154E-07	6.22692E-07	1.54608E-06	8.13154E-07	2.14364E-07	2.14636E-07	2.60909E-07	2.03818E-07	1.21333E-07	1.958E-07	4.44E-09	2.20533E-07
Corynebacterium_camporealensis	1.15182E-07	1.04545E-08	1.70727E-07	3.59091E-08	8.57692E-08	4.13846E-08	0	0.000000042	0.00000016	2.85455E-08	2.05455E-08	2.04273E-07	0	1.01733E-07	1.29733E-07	2.288E-07
Corynebacterium_rouxii	0.00000317	0.000000666	2.49373E-06	2.94409E-06	7.53077E-08	0.000000147	2.14962E-06	2.45508E-06	0.000000483	1.09218E-06	2.22755E-06	2.41509E-06	2.11333E-07	5.48667E-07	1.08573E-07	2.494E-07
Corynebacterium_matruchotii	6.16909E-07	3.59364E-07	1.06909E-07	4.47545E-07	2.38231E-07	1.49077E-07	1.60846E-07	2.58231E-07	0.000000266	2.85455E-08	2.56364E-07	4.66727E-07	4.61333E-08	2.114E-07	7.51067E-08	6.33333E-08
Corynebacterium_glyciniphilum	2.31909E-07	0.000000065	7.97273E-08	1.53545E-07	0	2.28615E-07	8.11538E-08	0	0	8.02727E-08	6.65455E-08	2.03364E-07	9.59333E-08	1.128E-07	6.15067E-08	9.67333E-08
Corynebacterium_riegelii	1.79455E-07	4.54545E-08	1.53945E-07	1.33909E-07	9.62308E-08	1.23692E-07	1.17769E-07	2.59846E-07	3.15818E-07	3.62727E-08	3.95455E-08	5.52818E-07	2.04867E-07	2.33333E-07	1.72733E-07	2.70333E-07
Corynebacterium_maris	0.00000011	5.81818E-08	5.25455E-08	2.61182E-07	2.11154E-07	0	1.94154E-07	0	6.62727E-08	1.04273E-07	4.75455E-07	6.82455E-07	2.308E-07	3.99533E-07	4.84133E-07	0.000000275
Corynebacterium_diphtheriae	1.22536E-06	0.000000047	6.87091E-07	1.65473E-06	2.39077E-07	2.81615E-07	4.19538E-07	7.10692E-07	1.42182E-06	3.76455E-07	1.12491E-06	5.88273E-07	2.75067E-07	3.248E-07	4.97467E-07	3.58333E-07
Corynebacterium_cystitidis	1.02364E-07	0	3.70909E-08	2.17882E-07	0	0	0	2.62538E-07	6.62727E-08	0.000000034	1.30909E-07	3.16909E-07	0.000000074	6.78E-08	4.48667E-08	0
Corynebacterium_glaucum	0.000002069	3.80273E-07	4.744E-07	1.62473E-06	2.34846E-07	2.13846E-08	1.76492E-06	1.90777E-06	8.18727E-07	1.07018E-06	3.75909E-07	7.69273E-07	2.81667E-07	2.49867E-07	5.10267E-07	5.81867E-07
Corynebacterium_choanis	3.96273E-07	8.60727E-07	1.84909E-07	9.29091E-08	2.05538E-07	7.66154E-08	4.10692E-07	2.33692E-07	2.30364E-07	1.40545E-07	4.10909E-07	2.70909E-07	1.81933E-07	3.214E-07	4.83333E-07	0.000000357
Corynebacterium_vitaeruminis	1.01027E-06	0	1.01527E-06	0.000002124	2.72769E-07	2.12769E-06	2.34954E-06	2.66569E-06	1.26091E-06	1.08782E-06	1.39027E-06	1.52773E-06	6.43733E-07	0.000000417	5.784E-07	7.20067E-07
Corynebacterium_striatum	2.07818E-07	0	1.23636E-08	0.000000094	1.13846E-07	0.000000564	2.14923E-07	4.79846E-07	2.78273E-06	1.63909E-07	5.00818E-07	3.05364E-07	5.88667E-08	2.01867E-07	1.27653E-06	3.268E-07
Corynebacterium_argentoratense	3.34364E-07	1.71091E-07	6.17091E-08	2.75636E-07	1.69615E-07	4.33308E-07	0.000000123	3.76231E-07	0.000000237	4.06273E-07	3.26273E-07	0.000000696	3.372E-07	5.55667E-07	9.87933E-07	8.016E-07
Corynebacterium_casei	5.92727E-08	0	0	5.55364E-07	0	0	4.59154E-06	0.000006206	3.99872E-05	1.14455E-07	5.55691E-06	3.67545E-06	0	9.22733E-07	1.11649E-05	7.69427E-06
Corynebacterium_sp._2183	3.69091E-08	0.000000047	8.07636E-08	2.43636E-08	5.63846E-08	2.95385E-08	3.39692E-07	7.79231E-08	0	0.000000028	1.30909E-07	2.64455E-07	6.97333E-08	1.17933E-07	0	0
Corynebacterium_sp._NML98-0116	4.95636E-07	1.04545E-08	9.20036E-07	1.44818E-07	3.46462E-07	8.08462E-08	7.80462E-07	1.06554E-06	4.16455E-07	1.79273E-07	0.000000609	4.14182E-07	1.65267E-07	3.01667E-07	2.94933E-07	2.03067E-07
Corynebacterium_sp._sy039	1.05455E-07	2.09091E-07	7.02727E-07	4.76364E-07	9.07308E-07	0	0	0.000000011	9.28364E-07	8.98909E-07	6.12545E-07	6.52273E-07	7.93667E-07	9.578E-07	5.244E-07	5.93267E-07
Corynebacterium_ammoniagenes	1.17909E-07	0	2.96364E-08	2.70455E-07	8.79231E-08	2.95385E-08	1.09777E-06	2.01438E-06	0.00001509	3.16091E-07	2.49364E-06	1.35545E-06	5.59333E-08	5.42467E-07	2.49867E-06	2.00747E-06
Corynebacterium_sphenisci	7.87818E-06	6.16864E-07	1.65091E-06	5.38336E-06	1.53415E-06	8.99462E-07	4.02846E-07	2.89154E-06	3.48909E-06	2.26455E-06	3.21636E-06	4.65009E-06	2.22053E-06	0.00000295	0.000001943	3.45633E-06
Corynebacterium_stationis	5.12455E-07	0	1.93773E-07	1.38531E-05	1.51462E-07	2.01308E-07	0.000174634	0.000216869	0.001433686	2.24827E-06	0.000209721	0.000137101	2.08E-08	3.05043E-05	0.000408265	0.000244585
Jonesia_denitrificans	1.44727E-07	0	1.48182E-07	5.18182E-08	5.76154E-08	6.51538E-07	6.41538E-08	1.14385E-07	0.00000015	0.000000128	1.47545E-07	1.01091E-06	2.14E-08	0.000000047	2.83733E-08	1.552E-07
Luteimicrobium_xylanilyticum	3.73909E-07	0.000000065	3.87727E-07	7.47273E-07	1.21077E-07	0	8.14923E-07	0.000000307	5.37727E-07	4.42182E-07	6.58091E-07	2.69273E-07	0.000000439	1.846E-07	2.278E-07	3.648E-07
Georgenia_faecalis	2.85727E-07	2.14909E-07	5.11273E-08	3.04636E-07	7.46923E-08	2.19538E-07	0.000000331	1.50462E-07	1.39636E-07	0	1.99364E-07	3.42909E-07	0	1.58E-08	2.09733E-08	6.80667E-08
Georgenia_sp._Z294	1.46455E-07	0.000000065	2.04909E-08	1.01909E-07	1.31769E-07	2.22077E-07	7.92308E-08	3.72154E-07	9.77273E-08	2.17636E-07	3.23636E-07	5.92909E-07	0.000000104	8.56E-08	2.89267E-07	1.74667E-07
Georgenia_sp._Z443	6.15182E-07	2.34545E-08	6.17091E-08	7.32636E-07	0	2.15923E-07	5.87923E-07	9.31538E-08	1.61455E-07	2.07727E-07	1.30636E-07	3.66636E-07	5.09333E-08	1.75467E-07	6.384E-08	1.378E-07
Miniimonas_sp._S16	2.61545E-06	4.24636E-07	1.99818E-07	4.99909E-07	6.15615E-07	7.21538E-08	3.77692E-08	3.42923E-07	1.33727E-06	4.26545E-07	2.04455E-07	1.03882E-06	4.676E-07	2.84067E-07	3.17533E-07	1.29467E-07
Beutenbergia_cavernae	3.11818E-08	1.57818E-07	0	6.32727E-08	1.86923E-08	2.86615E-07	8.81538E-08	1.23308E-07	0.000000221	1.43182E-07	3.33636E-08	1.82364E-07	6.08E-08	9.29333E-08	1.674E-07	3.226E-07
Dermatophilus_congolensis	4.54545E-08	8.18182E-08	0.000000047	1.03636E-08	1.02077E-07	0	0	3.22308E-08	1.06455E-07	1.03545E-07	0	0	2.37333E-08	0	4.49067E-08	4.60667E-08
Mobilicoccus_sp._NJES-13	2.20727E-07	8.86364E-09	2.04909E-08	5.82727E-08	9.22308E-08	0	9.03846E-08	1.63154E-07	4.86364E-08	1.38091E-07	1.67273E-07	3.24364E-07	6.92E-08	1.596E-07	3.494E-07	0.000000156
Austwickia_chelonae	5.57273E-08	0	0	6.34273E-07	8.79231E-08	4.27692E-08	1.13077E-07	1.23077E-07	4.45455E-08	0	0.000000039	6.93636E-08	9.68E-08	2.16533E-07	2.28867E-07	1.42133E-07
Sanguibacter_keddieii	1.12455E-07	0	5.89455E-08	2.90909E-08	6.46923E-08	1.18308E-07	6.41538E-08	2.37231E-07	1.82455E-07	1.29636E-07	3.07182E-07	0.00000005	1.49733E-07	0.000000028	1.11533E-07	4.28E-08
Sanguibacter_sp._HDW7	8.98727E-07	0	0	2.06727E-07	0.000000219	1.24308E-07	2.63077E-08	4.06538E-07	2.69273E-07	3.14091E-07	0.000000591	2.52909E-07	4.27333E-08	2.19267E-07	1.37533E-07	1.932E-07
Kytococcus_sedentarius	1.27155E-06	5.14045E-07	5.53982E-07	1.45864E-06	3.08231E-07	2.09231E-07	1.42962E-06	1.23269E-06	6.59182E-07	8.99727E-07	5.63455E-07	1.31182E-06	1.70733E-07	2.68467E-07	0.000000463	9.276E-07
Dermacoccus_sp._PAMC28757	7.98182E-08	2.65727E-07	5.74545E-08	1.44091E-07	0	0	8.92308E-08	0	0	6.43636E-08	7.53636E-08	9.75E-08	2.91333E-08	3.09333E-08	0	0
Dermacoccus_nishinomiyaensis	1.34909E-07	1.40364E-07	6.71818E-08	0.000000284	2.26308E-07	2.70769E-07	2.12308E-07	2.32923E-07	2.64545E-08	1.69364E-07	4.56182E-07	5.42455E-07	1.01867E-07	1.05333E-07	5.38667E-08	1.282E-07
Luteipulveratus_mongoliensis	1.34545E-07	0.000000065	4.12727E-09	2.43636E-08	1.16538E-07	0	0	0.00000019	1.61182E-07	0.000000028	1.34364E-07	4.46909E-07	1.71733E-07	1.53733E-07	4.64667E-08	1.186E-07
Brevibacterium_sp._PAMC23299	3.11818E-08	0	0	3.72455E-08	3.92308E-08	1.09231E-07	2.63077E-08	0.000000042	3.98182E-08	3.62727E-08	0	0	4.88E-08	0	4.44E-09	1.98667E-08
Brevibacterium_luteolum	0	0	2.96364E-08	9.54545E-09	3.58462E-08	7.25077E-07	2.63077E-08	2.90077E-07	6.73636E-08	0	0.000000048	1.56364E-07	1.162E-07	6.41333E-08	3.224E-08	2.77467E-07
Brevibacterium_sp._o2	1.42364E-07	2.08273E-07	2.47273E-08	1.93636E-08	0	0	3.33077E-08	0.000000011	2.75455E-08	2.87273E-08	1.30909E-07	0.000000097	2.94667E-08	0	0	4.28E-08
Brevibacterium_sp._WO024	3.82909E-07	1.68182E-08	8.40909E-08	1.05636E-07	3.58462E-08	0	0	0.000000011	2.72818E-07	2.51727E-07	8.56364E-08	3.58636E-07	0	0	0	0
Brevibacterium_sp._CS2	0	1.68182E-08	1.63636E-08	2.69091E-07	1.70692E-07	4.63538E-07	2.93846E-07	2.16615E-07	8.91818E-08	0.000000028	8.02727E-08	2.25818E-07	1.16733E-07	7.42667E-08	1.12733E-07	5.94667E-08
Brevibacterium_sp._JNUCC-42	0	0.000000065	0	0	2.38308E-07	0	0	0	3.98182E-08	0	3.33636E-08	1.27818E-07	8.45333E-08	7.94667E-08	1.49333E-08	2.40933E-07
Brevibacterium_sandarakinum	4.03545E-07	1.03636E-08	7.49818E-08	3.95273E-07	0	2.06923E-07	1.43154E-07	1.84538E-07	0.000000272	0	3.95455E-08	2.85455E-08	4.17333E-08	6.73333E-08	2.69867E-07	8.62E-08
Brevibacterium_linens	0.000000107	3.50909E-08	1.89455E-07	9.53636E-08	3.34615E-08	2.06077E-07	0.000000087	9.42308E-08	2.52273E-07	0.000000047	1.75182E-07	1.48273E-07	6.08E-08	3.26667E-08	1.14307E-07	6.26667E-08
Brevibacterium_sp._YB235	1.35764E-06	4.49636E-07	1.58845E-06	9.72545E-07	4.11538E-08	8.11846E-07	1.48192E-06	9.35923E-07	3.90909E-07	9.89545E-07	6.70909E-07	1.52427E-06	4.51333E-08	2.532E-07	3.41933E-07	3.59867E-07
Brevibacterium_aurantiacum	2.61818E-07	1.16364E-08	1.33818E-07	6.32727E-08	6.17692E-08	7.33154E-07	2.07692E-07	8.67692E-08	2.62091E-07	1.45909E-07	7.23636E-08	2.85455E-08	6.88667E-08	1.71333E-08	1.12667E-08	7.09333E-08
Brevibacterium_siliguriense	3.22182E-07	0	1.69436E-07	2.35273E-07	2.08615E-07	6.64615E-07	4.11769E-07	4.67692E-07	0.000000094	1.89091E-07	5.04182E-07	0.000000304	1.49933E-07	1.28533E-07	4.52667E-07	1.866E-07
Brevibacterium_sp._'Marine'	1.39791E-06	0	4.10545E-07	0.000001518	2.11923E-07	0	7.07692E-08	5.65308E-07	4.05182E-07	4.88091E-07	0.00000121	1.90182E-06	3.672E-07	4.36267E-07	4.00933E-07	7.52067E-07
Xylanimicrobium_sp._FW10M-9	8.80909E-08	0	0	1.37182E-07	5.42308E-08	2.28923E-07	5.96154E-08	0	9.42727E-08	1.56818E-07	0	2.17455E-07	6.48E-08	2.03667E-07	0.00000013	1.91267E-07
Isoptericola_dokdonensis	8.79091E-08	0	0	4.68818E-08	2.05923E-07	2.62615E-07	0	1.35231E-07	9.95455E-08	0	1.47545E-07	6.21818E-08	1.20133E-07	0.000000165	1.23307E-07	1.39467E-07
Isoptericola_variabilis	7.83636E-08	0	0	8.43636E-08	5.02308E-08	0	1.36615E-07	0	2.16364E-08	5.22727E-08	1.91909E-07	7.78182E-08	2.14E-08	1.71333E-08	1.49333E-08	1.634E-07
Xylanimonas_allomyrinae	6.33636E-08	0	0.000000047	6.68182E-08	9.04615E-08	1.75462E-07	2.75923E-07	0.000000096	2.14273E-07	2.28455E-07	9.96364E-08	2.63182E-07	1.56133E-07	4.23333E-08	8.86667E-09	1.86E-08
Xylanimonas_cellulosilytica	0.000000472	3.70727E-07	3.27273E-08	2.28091E-07	5.45385E-08	1.23231E-07	3.32308E-07	2.56923E-08	0.000000525	7.30909E-07	6.34273E-07	5.83545E-07	5.09333E-08	7.29333E-08	9.82E-08	6.62E-08
Cellulosimicrobium_cellulans	0	0	3.70909E-08	2.87273E-08	0	0	7.92308E-08	2.66231E-07	2.75455E-08	0	3.79091E-08	2.85455E-08	2.14E-08	3.09333E-08	4.38667E-08	1.98667E-08
Cellulosimicrobium_sp._BI34T	1.50636E-07	9.54545E-09	3.27273E-08	4.45909E-07	3.79308E-07	1.61615E-07	2.94154E-07	1.36846E-07	7.73636E-08	1.70364E-07	0.0000001	5.55364E-07	3.06733E-07	3.998E-07	2.38933E-07	4.522E-07
Cellulosimicrobium_sp._JZ28	1.73818E-07	0	0.000000381	2.78909E-07	4.64615E-08	0	1.21331E-06	8.62769E-07	9.13636E-08	7.02727E-08	3.95455E-08	1.81909E-07	4.17333E-08	8.59333E-08	0.000000139	7.20667E-08
Ruania_sp._HY168	3.11818E-08	0	7.40909E-08	0.000000042	3.58462E-08	0	2.56154E-07	2.37385E-07	1.58909E-07	0.000000081	3.30636E-07	0	1.044E-07	3.50667E-08	1.566E-07	1.61933E-07
Haloactinobacterium_sp._HY164	1.17455E-07	0	2.87273E-08	4.72727E-08	2.59231E-08	1.44538E-07	3.11538E-08	5.22308E-08	1.80727E-07	5.67273E-08	0	4.45909E-08	2.64667E-08	1.28333E-07	2.68667E-07	2.346E-07
Haloactinobacterium_sp._RN3S43	0.000002078	2.68182E-07	2.46673E-07	1.20427E-06	4.83231E-07	2.95385E-08	0.000000391	6.16154E-07	1.19945E-06	1.61764E-06	9.22909E-07	1.24327E-06	3.82533E-07	6.158E-07	3.81867E-07	3.91867E-07
Arsenicicoccus_sp._oral_taxon_190	7.81818E-08	0.000000065	4.12727E-09	6.27273E-08	1.00615E-07	7.18923E-07	5.74615E-08	1.46615E-07	9.91818E-08	2.15182E-07	2.62545E-07	4.57909E-07	6.93333E-08	9.32E-08	5.35267E-07	1.47933E-07
Serinicoccus_marinus	0	0	0	4.40909E-08	0	0	0.000000132	0	0	0	0	0	0	0	0	0
Serinicoccus_profundi	5.57273E-08	0	1.63636E-08	4.20909E-08	0	0	0	2.46923E-08	2.75455E-08	3.62727E-08	2.16636E-07	1.65909E-07	0	6.60667E-08	0	1.06133E-07
Serinicoccus_hydrothermalis	1.47818E-07	9.77909E-07	1.27218E-07	1.66091E-07	0	0	0	2.46923E-08	2.64545E-08	0.000000028	2.48636E-07	1.88136E-07	0	4.70667E-08	4.44E-09	0
Serinicoccus_chungangensis	1.19364E-07	0.00000026	4.12727E-09	0.000000138	5.93846E-08	1.57692E-07	1.31538E-07	1.74615E-07	7.62727E-08	1.38636E-07	1.78545E-07	1.28364E-07	1.96467E-07	2.78667E-07	4.19467E-07	0.000000255
Janibacter_sp._YB324	3.11818E-08	0	0	0	0.000000039	0	0	3.22308E-08	0	0	0	0	0	0	0	0
Janibacter_limosus	3.69091E-08	0	3.70909E-08	3.58818E-08	9.51538E-08	7.14462E-07	2.12462E-07	7.40769E-08	3.75455E-08	1.84364E-07	5.83636E-08	1.12545E-06	0	0	1.876E-07	1.29067E-07
Janibacter_melonis	0	0	1.63636E-08	2.39091E-08	2.05385E-08	0	0	1.25462E-07	0	3.03636E-08	6.66364E-08	0	4.74667E-08	1.04133E-07	1.54333E-07	5.27333E-08
Janibacter_indicus	1.80636E-07	0	1.32818E-07	6.38182E-07	0.000000203	0	7.87692E-07	1.01069E-06	1.52545E-07	0	3.09636E-07	9.96364E-08	4.46667E-08	1.036E-07	2.772E-07	4.66667E-08
Tetrasphaera_sp._HKS02	0.000000227	0	7.39455E-08	1.80091E-07	2.05385E-08	3.04615E-07	5.26154E-08	1.85462E-07	8.67273E-08	1.32545E-07	0	2.16455E-07	8.60667E-08	6.57333E-08	1.80773E-07	3.00067E-07
Intrasporangium_calvum	5.68182E-08	0	3.70909E-08	7.58182E-08	1.05154E-07	7.92308E-08	0	5.22308E-08	2.64545E-08	7.55455E-08	0	0	4.61333E-08	1.78133E-07	3.50667E-08	0
Phycicoccus_sp._HDW14	1.58182E-08	8.86364E-09	0	4.20909E-08	0	0	0	5.22308E-08	0	0	0	0	2.24E-08	0	0	0
Phycicoccus_endophyticus	3.69091E-08	0	0.000000047	1.49545E-07	7.29231E-08	0	0	0	0	0	1.41182E-07	7.12727E-08	0	6.93333E-08	1.13573E-07	3.37333E-08
Ornithinimicrobium_sp._AMA3305	0	7.54545E-08	0	0	0	0	0	3.57692E-08	0	0	0	5.17273E-08	0	0	0	0
Ornithinimicrobium_sp._H23M54	2.45909E-07	0	9.60909E-08	3.70364E-07	8.69231E-08	0	1.79769E-07	1.62385E-07	0	1.85364E-07	0	0.000000066	1.39667E-07	1.154E-07	0	6.33333E-08
Ornithinimicrobium_pratense	1.64182E-07	0.000000065	2.04909E-08	1.02818E-07	3.15385E-08	1.95154E-07	0	0.000000102	1.07818E-07	1.61091E-07	1.07818E-07	1.71273E-07	1.26733E-07	1.12867E-07	5.01333E-08	2.85067E-07
Ornithinimicrobium_sp._HY006	6.27909E-07	0.000000708	8.80909E-08	0.000000088	2.45692E-07	0	3.70692E-07	1.63154E-07	3.75455E-08	3.89091E-08	1.47545E-07	7.13636E-08	2.10133E-07	4.01867E-07	0.000000199	3.72E-08
Ornithinimicrobium_flavum	1.15727E-07	0.000000065	0.00000035	5.16364E-08	2.88462E-08	0	0	5.81538E-08	0	0	0	0.000000035	2.24E-08	8.12667E-08	2.98667E-08	3.40667E-08
Paraoerskovia_marina	1.46909E-07	0	0	0	0	0	4.33846E-08	0	0	0	3.95455E-08	7.13636E-09	0	6.80667E-08	4.564E-08	1.378E-07
Actinotalea_sp._JY-7876	5.82727E-08	0	0	7.93364E-08	3.58462E-08	0.000000212	3.33077E-08	2.97692E-08	0.000000054	0	6.71818E-08	2.85455E-08	2.24E-08	0	0	1.66667E-08
Oerskovia_sp._KBS0722	4.16364E-07	5.81273E-07	0.000000193	5.52545E-07	1.97154E-07	0	3.11538E-08	4.32308E-08	3.52545E-07	1.92636E-07	4.56091E-07	7.19455E-07	1.77667E-07	2.57067E-07	1.13333E-07	2.00667E-07
Pseudactinotalea_sp._HY158	0	1.51545E-07	0	5.36364E-08	0	9.39231E-08	0	5.81538E-08	0	0	1.30909E-07	3.74545E-08	4.51333E-08	8.54667E-08	0	2.79333E-08
Cellulomonas_flavigena	7.37273E-08	2.34545E-08	0	0	0	0	0	2.97692E-08	0	0.000000056	0	8.16364E-08	7.00667E-08	2.38E-08	4.44E-09	3.67333E-08
Cellulomonas_sp._H30R-01	6.23636E-08	2.70909E-08	0	0	6.30769E-08	0	3.77692E-08	6.69231E-08	0	0	2.76364E-08	2.14545E-08	0	3.50667E-08	2.55333E-08	9.33333E-09
Cellulomonas_shaoxiangyii	7.37273E-08	0	2.96364E-08	3.24091E-07	6.30769E-08	7.39077E-07	1.15538E-07	6.66923E-08	9.54545E-08	0.000000195	1.72909E-07	3.18455E-07	4.38E-08	6.39333E-08	2.18267E-07	9.56E-08
Cellulomonas_fimi	6.27273E-08	0	3.70909E-08	0	0	6.76923E-08	0	0	4.45455E-08	0	0.000000061	1.42727E-08	0	1.58E-08	0	0
Cellulomonas_gilvus	3.69091E-08	8.48182E-08	0	9.97273E-09	0	0	0	0	0	5.22727E-08	0.000000039	9.71818E-08	0	1.58E-08	0	0
Cellulomonas_sp._Y8	3.01273E-07	4.22182E-07	6.17091E-08	0.000000343	5.63846E-08	8.44615E-08	0	0	0	0	3.47545E-07	3.55182E-07	2.37333E-08	5.12E-08	2.39333E-08	0
Cellulomonas_sp._JZ18	2.30909E-08	0	6.17727E-08	0	0	9.71538E-08	0	0	0	0	0	7.55455E-08	0	3.09333E-08	0	1.86E-08
Cellulomonas_sp._40-2	3.79091E-07	2.11364E-07	1.77155E-07	9.63818E-07	1.52077E-07	3.83077E-08	7.10769E-08	1.64923E-07	2.75455E-08	0	1.82455E-07	7.09545E-07	1.342E-07	2.75467E-07	3.04933E-07	4.46533E-07
Devriesea_agamarum	0.000000133	0	0	6.12727E-08	0.000000054	3.51538E-07	6.44615E-08	1.46923E-08	9.45455E-08	2.85455E-08	6.68182E-08	7.13636E-09	4.22667E-08	1.044E-07	6.41067E-08	8.32E-08
Dermabacter_jinjuensis	0.000000047	0	3.70909E-08	2.47273E-08	6.17692E-08	0	2.63077E-08	8.28462E-08	2.64545E-08	6.69091E-08	2.76364E-08	9.85455E-08	0	0.000000014	0	9.33333E-09
Dermabacter_vaginalis	8.56182E-07	0	3.52545E-07	7.89909E-07	1.90077E-07	4.13846E-08	6.23077E-08	5.54615E-07	7.40727E-07	6.09182E-07	6.65091E-07	3.55636E-07	3.93733E-07	4.68533E-07	4.714E-07	8.298E-07
Brachybacterium_avium	7.58182E-08	3.49091E-08	1.63636E-08	1.06273E-07	1.39769E-07	1.51123E-06	7.39077E-07	2.59538E-07	0.000000084	0	0	2.46909E-07	1.474E-07	0.000000069	1.64773E-07	2.11667E-07
Brachybacterium_sp._SGAir0954	8.69091E-08	0	3.33182E-07	4.82727E-08	2.05385E-08	2.13846E-08	0	2.41538E-07	4.91818E-08	5.89091E-08	0.000000039	4.65909E-08	1.86667E-08	2.62E-08	2.13267E-07	9.42E-08
Brachybacterium_sp._P6-10-X1	2.90636E-07	0.00000013	8.25455E-09	1.52909E-07	1.96462E-07	2.33692E-07	1.60462E-07	5.19154E-07	6.64636E-07	3.39636E-07	7.98727E-07	6.82273E-07	1.43867E-07	2.91067E-07	3.24867E-07	2.37533E-07
Brachybacterium_faecium	1.60636E-07	0	1.23636E-08	5.18182E-09	0	0	2.73846E-08	0	1.73636E-07	1.29909E-07	0.0000002	8.22727E-08	5.32E-08	0	1.63533E-07	2.22867E-07
Brachybacterium_saurashtrense	1.47155E-06	8.73455E-07	5.72727E-07	1.53427E-06	4.64615E-07	3.02215E-06	5.51846E-07	7.57462E-07	9.80545E-07	7.64909E-07	8.39091E-07	1.11282E-06	3.28333E-07	0.000000199	6.712E-07	5.238E-07
Brachybacterium_vulturis	9.10909E-08	0	5.42545E-08	5.63636E-08	1.07923E-07	2.25615E-07	0.000000292	1.11769E-07	4.57091E-07	1.91545E-07	8.31818E-08	0.000000722	1.64067E-07	2.10133E-07	1.67733E-07	2.33867E-07
Brachybacterium_ginsengisoli	9.21818E-08	3.35455E-08	1.23636E-08	4.81818E-07	9.06615E-07	4.72308E-07	7.91615E-07	3.22308E-07	9.36182E-07	4.62818E-07	1.35818E-07	5.42909E-07	8.12267E-07	1.07293E-06	6.92467E-07	9.67933E-07
Paenarthrobacter_aurescens	0	0	0	0	0	0	0	0	0	0	0	0	0	0	0	3.67333E-08
Paenarthrobacter_ureafaciens	1.46364E-07	8.86364E-09	0	1.43636E-08	6.03846E-08	1.50769E-07	3.33077E-08	3.42154E-07	6.68182E-08	9.36364E-08	0	2.44818E-07	8.22667E-08	1.08467E-07	8.404E-08	2.79333E-08
Sinomonas_atrocyanea	5.92727E-08	0	0	6.13636E-08	1.11923E-07	0	1.30385E-07	5.04615E-08	0.000000081	0	0	1.64136E-07	4.23333E-08	7.97333E-08	2.11333E-07	1.68067E-07
Zhihengliuella_sp._ISTPL4	2.56364E-08	0.000000065	0.000000047	9.54545E-09	0	2.13846E-08	0.000000057	0	0	0	6.88182E-08	7.71364E-08	0	0	8.86667E-09	1.22E-08
Neomicrococcus_aestuarii	5.73727E-07	8.86364E-09	7.40909E-08	1.38091E-07	7.48462E-08	4.13846E-08	9.77692E-08	1.15154E-07	1.73273E-07	3.62727E-08	0.000000375	9.56727E-07	1.00867E-07	1.54267E-07	9.85333E-08	1.26533E-07

Citricoccus_sp._SGAir0253	2.49636E-07	3.03909E-07	8.40909E-08	3.23364E-07	7.02308E-08	0	7.45385E-08	1.26308E-07	0.000000064	0.000000047	2.22818E-07	0.000000491	4.51333E-08	8.88E-08	1.738E-07	0.000000057
Auritidibacter_sp._NML130574	1.50364E-07	0	4.00091E-07	4.37273E-08	1.67462E-07	3.92231E-07	8.42846E-07	3.60308E-07	1.56191E-06	1.43455E-07	5.57909E-07	4.59182E-07	4.78E-08	1.71333E-08	8.162E-07	1.18853E-06
Renibacterium_salmoninarum	5.99818E-07	7.66364E-08	2.16755E-07	0.000000394	1.48846E-07	5.91538E-08	4.63154E-07	3.00923E-07	4.16091E-07	3.21727E-07	6.03909E-07	3.50364E-07	2.31867E-07	2.81467E-07	2.07133E-07	2.84267E-07
Glutamicibacter_nicotianae	0	0	0	0	0	0	0	0	0	0	1.02727E-08	0	0	0.000000014	0	0
Glutamicibacter_arilaitensis	0	1.68182E-08	1.63636E-08	0	6.17692E-08	0	1.28923E-07	8.44615E-08	1.64727E-07	3.89091E-08	3.33636E-08	4.28182E-08	2.72E-08	0	3.75733E-08	1.22067E-07
Glutamicibacter_mishrai	1.07909E-07	2.83591E-07	7.40909E-08	0.000000078	5.20769E-08	1.23846E-07	1.87077E-07	4.37692E-08	0.00000025	9.11818E-08	1.83636E-07	4.21364E-08	2.72E-08	0	4.184E-08	1.86E-08
Glutamicibacter_creatinolyticus	4.50545E-07	2.14818E-07	3.10818E-07	3.70909E-07	3.28692E-07	9.06538E-07	2.73538E-07	6.69615E-07	4.37091E-07	5.27727E-07	1.06182E-07	9.92364E-07	2.04667E-07	1.94733E-07	4.516E-07	2.20333E-07
Glutamicibacter_halophytocola	8.69091E-08	7.38636E-08	8.07636E-08	1.43909E-07	2.05385E-08	0	0	2.97692E-08	1.44091E-07	0	0	1.39636E-07	9.47333E-08	1.20133E-07	3.88E-08	4.76667E-08
Micrococcus_sp._KBS0714	0	1.16364E-08	0	0	0	0	0	0	0	0	1.02727E-08	0	0	0	0	0
Micrococcus_luteus	4.64818E-07	8.86364E-09	4.28455E-08	2.46727E-07	9.23077E-08	5.84462E-07	2.59462E-07	2.53308E-07	1.84818E-07	4.01091E-07	0.000000386	3.17818E-07	1.12133E-07	1.826E-07	1.30533E-07	2.26133E-07
Pseudarthrobacter_sulfonivorans	0	0	0	0	0	0	0	0.000000011	0	0	0	0	0	0	0	8.53333E-08
Pseudarthrobacter_sp._NIBRBAC000502771	0	0	0	0	0	0	0	0	0	0	0	7.13636E-09	0	0	0	0
Pseudarthrobacter_sp._NIBRBAC000502772	0	0	0	4.35182E-08	0	0	0	0	5.79091E-08	0	0	0	4.88E-08	0	4.78667E-08	5.84E-08
Pseudarthrobacter_sp._NIBRBAC000502770	0	0	0	0	3.15385E-08	0	0	3.22308E-08	0	0	0	0	2.24E-08	0	2.99733E-08	0
Pseudarthrobacter_equi	3.38182E-07	1.41636E-07	9.02727E-08	3.21727E-07	0	0	2.41154E-07	2.00077E-07	1.15455E-07	0.000000047	1.37273E-07	1.42727E-08	0	3.22667E-08	0.000000072	1.20467E-07
Pseudarthrobacter_sp._YJ56	3.62727E-08	5.27727E-08	0.000000047	5.16364E-08	2.05385E-08	0	3.77692E-08	4.93846E-08	0	0	0	0	2.09333E-08	0	0	0
Pseudarthrobacter_phenanthrenivorans	0	3.49091E-08	1.73909E-07	2.97518E-07	3.58462E-08	2.95385E-08	0	9.46154E-08	3.75455E-08	8.02727E-08	6.66364E-08	1.49273E-07	4.86E-08	3.09333E-08	0	2.82867E-07
Pseudarthrobacter_sp._BIM_B-2242	1.60182E-07	4.42727E-08	2.04909E-08	9.85455E-08	1.56923E-07	1.26077E-07	6.23077E-08	2.40769E-07	1.82455E-07	2.47818E-07	2.54818E-07	3.09909E-07	2.164E-07	3.09333E-08	1.97907E-07	1.58867E-07
Pseudarthrobacter_sp._NBSH8	1.29455E-07	0	5.01273E-08	7.59727E-08	6.93077E-08	0	0	3.57692E-08	3.94545E-08	0	3.95455E-08	6.21818E-08	2.24E-08	1.61333E-08	6.54E-08	0
Pseudarthrobacter_chlorophenolicus	3.36618E-06	7.06636E-07	0.000003683	2.09564E-06	1.51015E-06	5.47692E-08	4.11577E-06	2.59031E-06	1.95709E-06	2.33927E-06	2.34473E-06	3.19645E-06	2.04473E-06	2.1116E-06	1.73193E-06	2.40407E-06
Nesterenkonia_sp._NBAIMH1	0.000001603	1.86273E-07	9.91273E-07	1.73945E-06	3.00692E-07	2.16923E-08	1.02446E-06	1.72354E-06	7.71545E-07	6.58909E-07	0.000000845	1.23136E-06	2.87667E-07	6.15267E-07	4.676E-07	4.33533E-07
Kocuria_indica	0	0	0	0	0	0	0	0	0	0	0	0	0	0	0	1.86E-08
Kocuria_rhizophila	0	0	0	0	0	2.13846E-08	0	0	0	0	0	1.18182E-07	0	0	0	0
Kocuria_sp._KD4	3.62727E-08	0	0	0	0	0	0	0	3.75455E-08	0	0	0	0	0	0	0
Kocuria_varians	7.37273E-08	5.41818E-08	0	9.97273E-09	5.45385E-08	1.28462E-07	0	0.000000084	0	0	1.41182E-07	0	0	6.30667E-08	8.03733E-08	4.97333E-08
Kocuria_palustris	1.61091E-07	5.81818E-08	2.00909E-07	1.667E-07	8.34615E-08	7.21538E-08	2.63077E-08	4.98385E-07	1.19091E-07	8.95455E-08	2.54364E-07	8.66818E-08	5.08E-08	1.58667E-07	1.12667E-08	1.15333E-07
Kocuria_rosea	5.67273E-08	0	8.25455E-09	8.91818E-08	1.07692E-07	1.10154E-07	3.33077E-08	0	1.75182E-07	2.87273E-08	0	2.35909E-07	7.48E-08	4.67333E-08	2.39333E-08	0
Kocuria_sp._TGY1120_3	2.06182E-07	8.18182E-08	1.19945E-07	1.04364E-07	1.75308E-07	9.89231E-08	0	1.72538E-07	1.21364E-07	8.02727E-08	0.000000134	8.26818E-08	4.73333E-08	1.464E-07	1.3084E-07	1.716E-07
Kocuria_flava	3.00636E-07	0	1.70855E-07	2.47545E-07	4.11538E-08	1.69538E-07	2.63077E-08	2.69615E-07	2.34273E-07	3.75727E-07	0	2.95727E-07	2.37333E-08	1.71333E-08	5.98667E-08	4.28E-08
Kocuria_turfanensis	1.87091E-07	1.63136E-07	1.63636E-08	1.18636E-07	9.33846E-08	8.97538E-07	1.71077E-07	0.000000203	5.34545E-08	0.000000303	3.00273E-07	4.36727E-07	1.606E-07	1.43133E-07	2.82667E-08	1.21667E-07
Kocuria_sp._TGY1127_2	4.72818E-07	6.73636E-08	1.93709E-07	1.13455E-06	1.63538E-07	0	0	7.40769E-08	8.78273E-07	1.04945E-06	1.20236E-06	1.34755E-06	3.74267E-07	6.52867E-07	2.40267E-07	7.46133E-07
Rothia_amarae	0	0	0	0	0	0	4.33846E-08	0	0	0	0	0	0	0	1.49333E-08	0
Rothia_terrae	2.98182E-08	0	0	1.93636E-08	0	0	2.63077E-08	1.08462E-07	7.97273E-08	9.77273E-08	3.95455E-08	8.26818E-08	4.88E-08	6.72E-08	0	7.95333E-08
Rothia_aeria	2.67909E-07	3.88909E-07	2.92455E-08	1.39182E-07	1.33462E-07	0	5.96154E-08	1.80615E-07	0.000000193	0	1.38455E-07	1.06091E-07	1.182E-07	1.24667E-07	2.38E-08	0.000000026
Rothia_nasimurium	1.70818E-07	0	3.37636E-08	3.01155E-07	1.03231E-07	8.70769E-08	2.60769E-07	5.22308E-08	5.79091E-08	1.44545E-07	0.000000078	1.325E-07	9.46E-08	1.17067E-07	1.33533E-07	3.206E-07
Rothia_dentocariosa	1.41818E-07	0.000000065	1.11618E-07	3.43636E-07	7.78462E-08	1.30923E-07	1.20231E-07	6.72077E-07	2.93727E-07	1.28727E-07	2.65545E-07	2.78364E-07	7.20667E-08	1.87067E-07	2.24667E-08	1.84667E-07
Rothia_mucilaginosa	5.37727E-07	0.00000013	1.19227E-07	2.07273E-07	2.12154E-07	2.38615E-07	5.16538E-07	9.22385E-07	2.61636E-07	3.92273E-07	0.000000039	2.01818E-07	2.268E-07	5.658E-07	4.70973E-07	4.02267E-07
Arthrobacter_sp._AQ5-05	0	1.04545E-08	0	0	0	0	0	0	0	0	0	0	0	0	0	0
Arthrobacter_sp._UKPF54-2	0	0.000000065	0	5.16364E-08	5.93846E-08	1.45077E-07	0	5.22308E-08	1.15455E-07	2.85455E-08	0	8.98182E-08	4.24E-08	0.00000009	1.93733E-08	2.286E-07
Arthrobacter_sp._KBS0702	0	4.64091E-08	0	4.79091E-09	2.88462E-08	4.13846E-08	5.26154E-08	0.000000011	0	0	0	7.13636E-09	0	0.000000014	8.84667E-08	0
Arthrobacter_sp._Rue61a	0	0	0	1.04091E-07	3.58462E-08	2.13846E-08	2.63077E-08	0	5.79091E-08	9.22727E-08	6.68182E-08	4.92727E-08	0	0	5.94E-08	3.67333E-08
Arthrobacter_agilis	1.39636E-07	0	0	4.50909E-08	0.000000039	8.63846E-07	2.80154E-07	2.56923E-08	2.72727E-08	1.00909E-07	1.06727E-07	2.70909E-07	0.000000108	1.40933E-07	1.904E-07	2.224E-07
Arthrobacter_sp._U41	2.96364E-08	0	3.70909E-08	1.51545E-08	6.30769E-08	0	7.92308E-08	7.72308E-08	9.77273E-08	1.04091E-07	1.75182E-07	1.48273E-07	2.91333E-08	0.000000028	1.02667E-07	7.26667E-08
Arthrobacter_sp._PGP41	0	3.23182E-08	6.23636E-08	3.62727E-08	0	4.91538E-07	6.66154E-08	8.73846E-08	8.08182E-08	8.02727E-08	3.07273E-08	4.90364E-07	2.72E-08	1.136E-07	0.000000066	2.568E-07
Arthrobacter_sp._24S4-2	1.58182E-08	0	0	4.79091E-09	0	2.16923E-08	0	0	0	0	0	7.13636E-09	0	0	0	0
Arthrobacter_sp._YC-RL1	1.75636E-07	0.00000013	0	0.000000258	2.05385E-08	0	0	8.28462E-08	8.51818E-08	0.000000116	2.33091E-07	1.38636E-07	2.72E-08	1.32267E-07	4.38667E-08	9.33333E-09
Arthrobacter_dokdonellae	2.66455E-07	3.50727E-07	1.24436E-07	1.32091E-07	1.66308E-07	2.59231E-07	8.58462E-08	1.24923E-07	0	1.73818E-07	1.00545E-07	7.29364E-07	4.46667E-08	3.29333E-08	8.84667E-08	9.79333E-08
Arthrobacter_alpinus	4.85818E-07	4.31727E-07	1.46636E-07	1.20955E-06	7.08846E-07	2.46146E-06	1.82408E-06	1.51223E-06	5.88455E-07	3.31818E-07	4.57636E-07	6.20273E-07	5.96867E-07	5.556E-07	8.716E-07	6.938E-07
Arthrobacter_sp._ERGS1:01	2.93636E-07	0.000000233	2.04909E-08	3.43909E-07	4.75385E-08	8.84615E-08	5.26154E-08	2.47692E-08	2.24727E-07	0.000000112	0	1.78818E-07	6.41333E-08	1.12733E-07	4.82E-08	0.000000026
Arthrobacter_sp._ZXY-2	0	0	4.12727E-09	0	0	0	0	0	0	0	0	0	0	0	0	0
Arthrobacter_sp._PAMC25564	1.14455E-07	4.81409E-07	9.87091E-08	4.79273E-07	1.00615E-07	1.89231E-08	2.63077E-08	1.08923E-07	1.25455E-07	5.89091E-08	9.64545E-08	3.24364E-07	1.08467E-07	4.49333E-08	4.02667E-08	7.76E-08
Arthrobacter_sp._PAMC_25486	0.000000745	3.88909E-07	4.94545E-08	3.62182E-07	1.14923E-07	5.35615E-07	6.93154E-07	3.51308E-07	5.92818E-07	7.31909E-07	9.24636E-07	1.28918E-06	5.09333E-08	2.106E-07	8.444E-08	1.976E-07
Arthrobacter_crystallopoietes	5.02727E-07	7.55682E-07	2.93364E-07	4.47636E-07	6.33231E-07	4.13846E-07	8.21077E-07	3.01231E-07	6.23636E-07	2.93636E-07	5.00364E-07	3.35364E-07	2.73267E-07	2.84533E-07	1.15573E-07	2.77067E-07
Arthrobacter_citreus	6.37109E-06	4.70364E-07	8.04727E-07	5.22182E-07	1.83285E-06	3.94615E-07	3.65615E-06	2.74038E-06	6.91018E-06	1.03074E-05	9.10564E-06	5.99836E-06	2.16787E-06	1.25153E-06	1.9766E-06	2.8606E-06
Arthrobacter_sp._YN	1.37455E-07	1.16364E-08	0	4.79091E-09	1.86923E-08	0	0	1.27846E-07	3.75455E-08	6.92727E-08	0	1.66273E-07	0	9.36667E-08	1.49333E-08	3.37333E-08
Arthrobacter_sp._QXT-31	8.45727E-07	3.04091E-07	4.50909E-07	1.65127E-06	2.49154E-07	6.76923E-08	0	4.03308E-07	9.41091E-07	1.01573E-06	1.09973E-06	0.000001145	5.29267E-07	6.06933E-07	7.92467E-07	8.58867E-07
Arthrobacter_sp._NEB_688	3.03818E-07	0	8.30909E-08	4.32727E-08	3.44308E-07	0	1.04146E-06	3.79615E-07	7.15455E-08	1.07818E-07	3.16455E-07	7.89091E-08	1.35867E-07	3.64933E-07	5.794E-07	3.91933E-07
Arthrobacter_sp._FB24	2.81264E-06	0.00000013	6.99091E-07	2.73718E-06	3.85462E-07	0	2.68415E-06	1.57669E-06	1.51027E-06	0.000001458	0.000001689	1.07227E-06	3.99533E-07	4.48467E-07	5.20267E-07	0.000000457
Arthrobacter_sp._TES	3.35636E-06	2.26727E-06	8.45364E-07	2.98836E-06	0.000002421	2.76077E-06	4.76815E-06	9.14538E-07	3.86273E-06	2.50009E-06	1.85891E-06	2.30564E-06	1.2914E-06	1.29933E-06	7.05267E-07	1.09907E-06
Microcella_alkaliphila	0	0	0	0	0	0	0	1.46923E-08	0	0	0	0	0	0	0	0
Cnuibacter_physcomitrellae	0	0	0	0	0	0	0	0	0	0	0	0	0	0	4.82E-08	1.22E-08
Gryllotalpicola_protaetiae	0	1.64773E-07	0	1.09882E-07	0	8.84615E-08	3.11538E-08	8.31538E-08	1.04545E-07	0.000000047	3.92545E-07	0.00000012	2.64E-08	0.000000033	0	6.33333E-08
Lysinimonas_sp._KACC_19322	1.70818E-07	0	1.63636E-08	5.88182E-08	1.32385E-07	2.16923E-08	5.26154E-08	0	0.000000143	5.72727E-08	0	2.27909E-07	8.17333E-08	3.11333E-08	1.01467E-07	6.02667E-08
Frigoribacterium_sp._NBH87	2.52727E-07	2.87273E-08	1.90727E-07	1.33636E-07	8.33846E-08	1.62154E-07	2.62615E-07	4.07769E-07	1.28818E-07	1.80273E-07	2.81364E-07	8.16364E-08	6.99333E-08	2.404E-07	4.56067E-07	9.18667E-08
Pontimonas_salivibrio	0	0	3.28545E-08	7.12455E-08	3.96769E-07	0.000000136	0	7.65385E-08	2.75455E-08	6.27273E-08	2.76364E-08	1.42455E-07	2.72E-08	0.000000033	3.72667E-08	5.58667E-08
Diaminobutyricimonas_sp._LJ205	1.58182E-08	0	4.12727E-09	0	0	1.79231E-08	0	2.93846E-08	0	0	6.68182E-08	1.42727E-08	0	0	0	0
Plantibacter_flavus	0.000000133	1.55182E-07	0	1.91818E-08	2.05385E-08	1.06154E-07	0	2.47692E-08	0	0.000000056	0.000000041	0	0	6.52667E-08	0	1.22E-08
Plantibacter_sp._M259	0	0	0	3.07273E-08	0	8.10769E-08	0	0	0	0	9.05455E-08	0	0	3.26667E-08	0	0
Plantibacter_sp._PA-3-X8	0	0	4.12727E-09	4.79091E-09	0	9.46154E-09	0	0.000000011	2.16364E-08	0	1.02727E-08	0	2.09333E-08	0.000000028	7.06667E-08	0
Humibacter_sp._WJ7-1	9.57091E-07	8.31364E-07	4.29945E-07	1.17764E-06	2.97462E-07	0.000000081	7.04846E-07	7.34154E-07	8.64091E-07	6.61818E-07	3.63455E-07	9.55091E-07	5.97133E-07	1.62467E-07	1.21733E-07	5.038E-07
Microterricola_viridarii	0	7.49091E-08	7.65273E-08	1.51545E-08	0	0	4.33846E-08	1.65077E-07	0	0	2.76364E-08	2.50909E-07	2.64667E-08	0	0	1.25067E-07
Herbiconiux_sp._SALV-R1	1.45364E-07	5.93545E-07	7.16182E-08	1.89818E-07	0	0	3.48385E-07	7.76923E-08	8.43636E-08	0	6.66364E-08	3.17273E-07	0	0	1.16533E-07	0
Protaetiibacter_sp._SSC-01	4.60909E-08	0	4.12727E-09	2.17364E-07	3.58462E-08	7.57692E-08	1.39615E-07	1.46308E-07	3.75455E-08	0.000000056	4.81818E-08	5.17273E-08	1.24933E-07	0	8.92E-08	1.79867E-07
Protaetiibacter_intestinalis	5.82727E-08	7.54545E-08	5.11273E-08	6.26364E-08	1.01308E-07	0	0	2.16154E-07	1.28636E-07	3.89091E-08	0.000000134	9.07273E-08	6.62667E-08	1.71333E-08	2.99733E-08	5.52667E-08
Rhodoluna_lacicola	0	0	0	0	0	0	0	0	0	0	0	7.13636E-09	0	0	0	0
Candidatus_Rhodoluna_planktonica	2.70909E-08	0	0	4.79091E-09	0	0	0	0	0	2.87273E-08	1.43364E-07	2.14545E-08	0	0	0	0
Rhodoluna_limnophila	6.91818E-08	0	1.31482E-07	1.38182E-07	1.86923E-08	0	0	0	1.02455E-07	0	6.66364E-08	3.16364E-08	2.44E-08	0.000000033	4.44E-09	0
Rathayibacter_sp._VKM_Ac-2762	0	0	0	0	0	0	0	0	0	0	0	7.13636E-09	0	1.71333E-08	0	0
Rathayibacter_sp._VKM_Ac-2801	0	0	0	0	3.58462E-08	0	0	0	0	0	0	0	0	0	4.82E-08	3.40667E-08
Rathayibacter_tritici	0	0	2.87273E-08	3.24545E-08	0	0	0	0	3.75455E-08	0	0	0	0	0.000000014	6.46667E-08	0
Rathayibacter_sp._VKM_Ac-2760	1.45727E-07	5.41818E-08	3.27273E-08	9.87273E-08	3.15385E-08	0	0	2.46923E-08	5.97273E-08	0	1.97455E-07	2.14545E-08	4.78E-08	0	0	0
Rathayibacter_toxicus	1.20727E-07	5.69545E-07	3.27273E-08	1.87091E-07	1.37923E-07	0.000000095	2.41538E-07	2.13385E-07	0.000000195	0	1.44818E-07	3.88909E-07	6.08667E-08	7.76E-08	1.49333E-08	7.98667E-08
Rathayibacter_sp._VKM_Ac-2804	0	0	1.77273E-08	5.18182E-09	3.34615E-08	1.04769E-07	0	0	0	0	6.88182E-08	0	0	1.71333E-08	0	0
Rathayibacter_tanaceti	3.11818E-08	5.74545E-07	1.49673E-07	4.02273E-07	0	2.95385E-08	0	0.000000011	2.64545E-08	5.22727E-08	0	5.50455E-08	0	0	0	0
Rathayibacter_festucae	0	5.05455E-08	0.000000042	3.90909E-08	5.63846E-08	1.03077E-07	3.06923E-08	1.46923E-08	5.79091E-08	8.85455E-08	2.24182E-07	2.14545E-08	1.376E-07	1.04267E-07	5.76667E-08	9.33333E-09
Rathayibacter_sp._VKM_Ac-2759	1.50455E-07	9.54545E-09	4.12182E-08	8.06364E-08	5.76154E-08	4.13846E-08	2.63077E-08	9.24615E-08	5.42727E-08	0	3.95455E-08	1.51636E-07	1.00867E-07	2.03133E-07	2.46E-08	6.86667E-08
Agromyces_protaetiae	0	0	0	0	0	0	0	0	0	0	0	4.79091E-08	0	0	0	0
Agromyces_aureus	0	0	0	0	3.58462E-08	0	0	0	0	0	0	5.70909E-08	2.14E-08	0	1.076E-07	0
Agromyces_sp._HY052	0	1.16364E-08	3.70909E-08	1.55455E-08	0	2.80923E-07	8.59231E-08	0	5.97273E-08	0	0	2.14545E-08	0	0.000000014	1.65333E-08	5.10667E-08
Agromyces_flavus	1.51273E-07	0.000000065	1.36455E-07	4.35182E-08	3.34615E-08	0	0	6.84615E-08	6.68182E-08	5.22727E-08	0	9.59091E-08	6.08E-08	2.38E-08	0	0
Agromyces_sp._LHK192	1.22273E-07	2.34545E-08	0	9.54545E-09	7.76923E-08	2.52308E-08	2.63077E-08	3.22308E-08	2.03727E-07	0	0.000000039	1.03727E-07	4.38E-08	0	1.49333E-08	1.244E-07
Agromyces_sp._MF30-A	3.05545E-07	0.000000065	1.31545E-07	6.58182E-08	0	4.94538E-07	1.12923E-07	4.93846E-08	2.56364E-07	0	0.000000039	1.42727E-08	4.22667E-08	0	5.62E-08	4.97333E-08
Agromyces_sp._KACC_19306	0.000002158	6.15091E-07	2.36009E-06	0.000001676	0	0	0	0.00000037	3.74545E-07	0.000000741	1.42745E-06	2.19255E-06	0	0	2.39333E-08	1.98667E-08
Marisediminicola_antarctica	1.76455E-07	3.34227E-07	9.04545E-08	5.32818E-07	3.00538E-07	0.000000364	2.47538E-07	0.000000166	1.37273E-07	8.63636E-08	6.71818E-08	3.15455E-07	1.97733E-07	1.14867E-07	1.12973E-07	5.10667E-08
Frondihabitans_sp._762G35	2.82909E-07	2.34545E-08	5.46364E-07	2.91818E-07	2.59231E-08	0	2.29769E-06	1.09631E-06	1.60364E-07	0.000000047	4.29545E-07	4.515E-07	6.11333E-08	4.33333E-08	2.36533E-07	7.47333E-07
Frondihabitans_sp._PAMC_28766	8.34455E-07	5.64545E-08	1.67355E-06	2.11618E-06	4.11385E-07	2.95385E-08	4.50923E-06	4.40469E-06	0.000000721	2.57455E-07	4.39182E-07	9.15818E-07	3.99467E-07	4.13667E-07	6.63333E-07	4.794E-07
Glaciihabitans_sp._INWT7	2.38455E-07	1.46818E-07	9.32727E-08	2.01091E-07	0.000000065	4.13846E-08	1.33077E-07	1.45154E-07	1.45636E-07	1.78727E-07	1.99727E-07	3.13409E-07	1.062E-07	2.354E-07	3.10733E-07	1.41733E-07
Candidatus_Aquiluna_sp._UB-MaderosW2red	6.80909E-08	0.000000112	2.20364E-07	2.89091E-08	1.45692E-07	0.000000198	1.43692E-07	0.000000068	1.92727E-07	0.000000165	7.67727E-07	1.79455E-07	1.51533E-07	8.31333E-08	5.07333E-07	2.24867E-07
Candidatus_Aquiluna_sp._15G-AUS-rot	1.06282E-06	3.27455E-06	5.80091E-07	1.98182E-07	1.54077E-07	7.16154E-08	2.79308E-07	5.38538E-07	2.09091E-07	7.64818E-07	6.67727E-07	1.84645E-06	0	4.88E-08	4.04667E-08	2.57733E-07
Aurantimicrobium_minutum	0	0	0	0	0	0	0	5.22308E-08	0	0.000000081	3.79091E-08	3.74545E-08	0	4.49333E-08	1.23107E-07	0
Aurantimicrobium_sp._MWH-Mo1	2.76818E-07	0	1.10909E-07	3.24818E-07	2.75385E-08	1.56769E-07	1.29615E-07	4.37692E-08	2.48727E-07	1.50636E-07	1.37273E-07	2.40636E-07	4.88667E-08	1.43533E-07	5.93333E-08	2.05467E-07
Aurantimicrobium_sp._MWH-Uga1	0	0	0	4.79091E-09	0	0	0	0	0	0	0	8.60455E-08	0	3.48E-08	0	0
Leucobacter_sp._HDW9A	0	1.68182E-08	0	0	2.88462E-08	0	2.63077E-08	0	0	0	0	7.13636E-09	0	0	1.49333E-08	0
Leucobacter_sp._HDW9C	2.47273E-07	0	0	7.79091E-08	0	0.00000027	0	0	1.81909E-07	0	1.03455E-07	2.02545E-07	7.16667E-08	1.524E-07	4.80667E-08	3.08E-08
Leucobacter_muris	9.92727E-08	0.000000065	0	0	0	5.29231E-08	0	0	0.000000071	0	2.81273E-07	4.59091E-08	4.18667E-08	0	0	1.22E-08
Leucobacter_triazinivorans	1.11818E-07	0	4.12182E-08	1.68273E-07	3.34615E-08	5.85538E-07	4.33846E-08	1.33692E-07	1.52364E-07	7.25455E-08	0.000000167	6.21818E-08	4.22E-08	5.08667E-08	2.01333E-08	0
Leucobacter_sp._HDW9B	4.55727E-07	2.34545E-08	5.73455E-08	4.55364E-07	8.03846E-08	2.52308E-08	0	1.08538E-07	1.99364E-07	1.89636E-07	0.00000026	0.000000601	8.78E-08	6.52E-08	8.79733E-08	3.40667E-08
Leucobacter_denitrificans	1.55636E-07	0	1.63636E-08	1.47273E-08	9.22308E-08	0	0	3.21231E-07	4.80909E-08	1.24818E-07	1.33182E-07	4.28182E-08	0	8.36E-08	5.93333E-08	9.82667E-08
Mycetocola_sp._449	8.69091E-08	5.41818E-08	7.97273E-08	2.26636E-07	0	9.46154E-09	4.33846E-08	3.22308E-08	1.37727E-07	2.46909E-07	1.64273E-07	2.06182E-07	0.000000112	5.68E-08	1.49333E-08	2.15333E-08
Mycetocola_sp._JXN-3	3.92182E-07	1.12818E-07	1.37455E-07	1.85091E-07	2.88462E-08	1.30769E-07	6.66154E-08	1.72385E-07	3.64636E-07	0.000000163	0.000000502	2.53636E-07	9.82E-08	6.93333E-08	0.000000084	0.000000116
Salinibacterium_sp._UTAS2018	0	0.000000065	0	0	0	0	0	0.000000011	0	0	0	0	6.08E-08	0	0	9.33333E-09
Salinibacterium_sp._dk2585	0	0	0	0	0	0	0	1.90769E-08	0	0	6.88182E-08	2.85455E-08	0	0	0	0
Salinibacterium_sp._ZJ450	0.000000213	0	7.40909E-08	0.000000124	0	0	0	0	0	0	0	0	2.14E-08	0	2.97333E-08	1.22E-08
Salinibacterium_sp._ZJ70	1.68455E-07	4.87182E-07	1.63636E-08	5.39091E-08	0	0	2.63077E-08	0	2.16364E-08	5.22727E-08	2.38727E-07	1.45955E-07	0	5.19333E-08	0	6.10667E-08
Salinibacterium_sp._CGMCC_1.16371	2.06273E-07	3.23182E-08	2.66855E-07	7.27273E-08	0.000000312	4.79538E-07	1.46231E-07	1.23769E-07	8.61818E-08	1.38364E-07	1.35455E-07	2.14545E-08	7.18667E-08	1.074E-07	1.214E-07	7.42E-08
Leifsonia_sp._PS1209	0	0	0	0	0	0	0	0	2.75455E-08	0	0	0	0	4.20667E-08	0	2.82267E-07
Leifsonia_shinshuensis	0	5.36364E-08	0	0	0	0	4.33846E-08	5.44615E-08	0	0	0	0	2.14667E-08	0	0	6.33333E-08
Leifsonia_sp._21MFCrub1.1	3.22364E-07	5.84545E-07	1.10636E-07	4.69909E-07	0	0	2.73846E-08	1.03462E-07	0	0	3.77273E-08	7.13636E-09	0	0	0	1.22E-08
Leifsonia_xyli	3.98182E-07	5.46818E-07	1.78764E-07	2.04273E-07	1.69462E-07	1.11615E-07	5.26154E-08	0.000000087	3.70727E-07	2.07182E-07	1.89182E-07	5.14636E-07	2.24E-08	0	1.51333E-07	3.97333E-08
Agrococcus_jejuensis	2.38909E-07	1.84091E-08	3.55455E-08	0	2.88462E-08	5.11077E-07	8.66154E-08	1.46923E-08	4.32727E-08	2.87273E-08	1.47455E-07	1.42727E-08	7.48E-08	3.78E-08	2.39333E-08	3.66E-08
Agrococcus_sp._SGAir0287	1.54545E-07	9.54545E-09	1.97309E-07	7.48182E-08	0	1.44615E-07	0	0	0	0	0	0	8.70667E-08	1.888E-07	0	0.00000013
Agrococcus_carbonis	3.30636E-07	1.16364E-08	1.23273E-07	2.23455E-07	3.82154E-07	0	4.42231E-07	8.70923E-07	1.32445E-06	4.17364E-07	6.56545E-07	5.31909E-07	5.864E-07	4.238E-07	8.086E-07	5.754E-07
Cryobacterium_soli	1.84818E-07	9.65909E-08	2.47273E-08	5.80909E-08	1.64615E-07	1.23277E-06	2.28231E-07	2.14462E-07	1.21909E-07	0	3.46273E-07	1.23727E-07	2.008E-07	1.36333E-07	1.03533E-07	2.01733E-07
Cryobacterium_arcticum	4.82545E-07	1.94545E-07	3.70909E-08	1.39545E-07	3.67769E-07	9.61538E-08	2.57538E-07	1.87769E-07	4.14455E-07	0.000000376	5.98545E-07	4.36909E-07	3.79133E-07	3.78533E-07	3.812E-07	2.18467E-07
Cryobacterium_sp._LW097	0.000000221	1.62727E-07	6.17091E-08	4.74818E-07	5.02308E-08	0	3.23846E-07	3.19538E-07	2.05364E-07	2.69455E-07	4.02273E-07	6.73545E-07	7.05333E-08	3.99333E-08	1.024E-07	0
Clavibacter_sp._DM1	0	0	0	4.79091E-09	0	0	3.06923E-08	0.000000062	0	0	0	7.55455E-08	0	0	0	0
Clavibacter_michiganensis	1.10691E-06	4.13182E-07	4.53455E-08	2.96545E-07	3.09385E-07	3.11231E-07	4.13692E-07	9.02077E-07	5.42727E-07	3.57091E-07	5.46455E-07	1.56573E-06	4.62533E-07	5.47133E-07	6.49467E-07	4.76667E-07
Microbacterium_sp._Nx66	0	0	0	0	0	0	0	0	0	0	0	0	0	3.50667E-08	0	0
Microbacterium_aurum	0	1.68182E-08	0	0	0	6.76923E-08	0	0	0	0	0	0	2.91333E-08	0	0	0
Microbacterium_sp._CGR1	0	0	0	0	0	0.000000026	0	0	0	0	0	0	2.14667E-08	0	0	0
Microbacterium_sp._NY27	0	0	0	0	3.58462E-08	4.63923E-07	0	7.52308E-08	0	5.22727E-08	0	0	9.28E-08	0	0	0
Microbacterium_sp._No._7	0	0	0	0	0	0	0	0	0	0	0	0	4.16E-08	4.90667E-08	0	1.75133E-07
Microbacterium_sp._str._'China'	0	0	0	2.43636E-08	0	0	0	0	0	0	0	0	0	0	0	0
Microbacterium_sp._L-031	5.57273E-08	0	0	6.92727E-08	0	0	0	3.81538E-08	0	0	0	7.71364E-08	4.38667E-08	3.26667E-08	0	9.33333E-09
Microbacterium_sp._1.5R	2.56364E-08	0	0	5.18182E-09	0	0	0	0	2.72727E-08	0	0	1.42727E-08	1.86667E-08	0	1.49333E-08	0
Microbacterium_sp._A18JL200	1.27818E-07	0	0	1.24273E-07	3.58462E-08	0	1.01923E-07	4.64615E-08	0	0	0	7.13636E-09	2.14E-08	0	0.000000098	5.10667E-08
Microbacterium_oxydans	0	0	0	4.20909E-08	0	4.94615E-08	0	0	1.15455E-07	0	1.02727E-08	0	0	4.76667E-08	0	0
Microbacterium_sp._HY82	3.62727E-08	1.16364E-08	0	4.20909E-08	0	0	7.40769E-08	1.32385E-07	0	0	1.02727E-08	9.03636E-08	0.000000053	3.50667E-08	5.94E-08	1.466E-07

Microbacterium_sp._PAMC_28756	0	0	0	6.34545E-08	0	0	0	1.90769E-08	0.000000027	0	2.76364E-08	7.13636E-09	0	0	0	0
Microbacterium_sp._XT11	2.70909E-08	7.04545E-08	0	2.39727E-08	2.88462E-08	4.27692E-08	8.11538E-08	1.90769E-08	0	0.000000028	9.48182E-08	0	0	0	0	6.75333E-08
Microbacterium_sp._Se63.02b	9.05455E-08	9.54545E-09	0	1.32364E-07	1.01769E-07	7.21538E-08	5.26154E-08	0	9.21818E-08	9.62727E-08	2.03182E-07	2.97455E-07	6.58E-08	9.58E-08	1.53173E-07	0.000000271
Microbacterium_sp._BH-3-3-3	9.20909E-08	3.59545E-08	0	5.18182E-09	1.41615E-07	0.00000039	0	0.000000062	2.72727E-08	0.000000028	0.000000162	1.42727E-08	1.80667E-07	1.13333E-07	3.41733E-08	9.12667E-08
Microbacterium_sediminis	5.27273E-08	2.70909E-08	1.63636E-08	1.95609E-07	0	0	0	5.22308E-08	0	0	0	3.16364E-08	0	0	0	0
Microbacterium_sp._ST-M6	3.11818E-08	0	2.96364E-08	4.79091E-09	0	0	0	0	0.000000103	0	6.68182E-08	0	2.24E-08	0	5.02667E-08	9.33333E-09
Microbacterium_sp._SGAir0570	1.58182E-08	1.16364E-08	0	0	0	0	0	0	0	0.000000028	0	0.000000097	0	0	0	0
Microbacterium_sp._ABRD_28	1.38909E-07	1.93818E-07	2.87273E-08	9.02727E-08	6.73846E-08	4.13846E-08	2.04769E-07	2.09846E-07	0	0	1.06545E-07	2.18364E-07	0	0	0	0
Microbacterium_sp._1S1	1.55818E-07	0	3.70909E-08	9.54545E-09	1.29077E-07	0	1.24846E-07	1.37692E-07	1.33818E-07	1.98182E-07	7.90909E-08	1.39682E-07	0.000000138	3.09333E-08	1.29933E-07	9.89333E-08
Microbacterium_schleiferi	3.11818E-08	0	9.36364E-08	3.24545E-08	0	0	0	3.57692E-08	0	0	1.30909E-07	4.92727E-08	2.09333E-08	0	2.24667E-08	1.86E-08
Microbacterium_sp._TPU_3598	1.27673E-06	0	3.64091E-07	4.29636E-07	8.26462E-07	5.27538E-07	8.06923E-07	2.65192E-06	9.96364E-08	0.000000388	6.24909E-07	0.000000239	0	0	0	0
Microbacterium_sp._LKL04	1.55545E-07	0.000000058	5.43636E-08	8.61818E-08	7.47692E-08	5.91538E-08	1.62692E-07	0.00000013	0.000000368	6.37273E-08	3.07636E-07	1.53909E-07	1.54067E-07	2.38E-08	0.000000065	1.81867E-07
Microbacterium_sp._Y-01	0.00000006	0	3.70909E-08	2.43636E-08	2.05385E-08	0	1.89308E-07	1.01615E-07	3.75455E-08	7.29091E-08	1.08727E-07	1.68182E-07	2.64667E-08	1.386E-07	2.16867E-07	0.00000018
Microbacterium_pygmaeum	1.56455E-06	0.000000065	8.89091E-07	1.26127E-06	1.84923E-07	6.76923E-08	2.10154E-06	1.61692E-06	5.16727E-07	1.29809E-06	7.96909E-07	8.38045E-07	3.56933E-07	3.15333E-07	2.87867E-07	4.416E-07
Microbacterium_oryzae	2.70909E-08	0	8.75455E-09	6.48182E-08	0	0	0	0	8.91818E-08	0	9.64545E-08	0	0	0	0	0
Microbacterium_sp._10M-3C3	0	0	8.75455E-09	0	0.000000039	0	0	0	0	0	0	0	0	0	0	0
Microbacterium_endophyticum	2.56364E-08	9.36364E-09	4.53455E-08	3.24545E-08	0	0	4.80769E-08	1.90769E-08	0.000000053	0	0	9.51818E-08	2.37333E-08	3.09333E-08	6.46667E-08	6.75333E-08
Microbacterium_sp._4R-513	0.000000217	0	4.12182E-08	1.04455E-07	0	0	0	0	0	5.22727E-08	9.90909E-08	5.37273E-08	0	0.000000014	1.49333E-08	5.26E-08
Microbacterium_sp._PM5	0	0	4.12727E-09	3.07273E-08	0	0	0	0	0	0	0	7.13636E-09	0	1.58E-08	4.44E-09	0
Microbacterium_sp._A18JL241	9.86364E-08	7.95455E-08	3.37636E-08	6.14545E-08	0	0.000000026	8.69231E-08	3.57692E-08	0.00000008	0	0	3.57273E-08	0	1.61333E-08	0	0
Microbacterium_sp._WY121	0	0	3.82182E-08	9.54545E-09	2.88462E-08	2.13846E-08	0	2.68846E-07	0	7.29091E-08	0	7.13636E-09	5.02E-08	2.38E-08	3.13333E-08	0.000000028
Microbacterium_foliorum	2.41455E-07	1.10455E-07	7.42636E-08	6.61545E-08	3.16923E-07	4.00923E-07	0.00000043	1.58385E-07	2.99273E-07	2.11091E-07	2.62455E-07	5.81727E-07	1.618E-07	1.25467E-07	2.25733E-07	2.82333E-07
Microbacterium_testaceum	3.11818E-08	5.81818E-08	2.08127E-07	3.44727E-07	2.41615E-07	0.000000462	5.15846E-07	6.27692E-07	1.14545E-07	2.47182E-07	3.34818E-07	4.21182E-07	5.72533E-07	2.59533E-07	5.672E-07	4.816E-07
Microbacterium_hominis	3.00273E-07	3.62545E-07	1.36073E-07	7.69909E-07	1.24231E-07	2.13462E-07	5.60923E-07	9.80846E-07	3.98182E-08	6.48182E-08	2.28727E-07	1.50773E-07	2.06467E-07	8.24667E-08	0.000000385	5.688E-07
Microbacterium_chocolatum	0	0	0	6.12727E-08	0	0	0	0	3.98182E-08	0	0	0	0	5.06E-08	1.49333E-08	0
Microbacterium_protaetiae	8.80909E-08	0	6.99455E-08	0.000000066	1.05231E-07	0	2.63077E-08	3.57692E-08	5.48182E-08	6.08182E-08	6.68182E-08	8.55455E-08	4.88E-08	1.38333E-07	1.24733E-07	5.26667E-08
Microbacterium_wangchenii	1.70909E-07	0.000000045	9.20909E-08	0.000000211	3.34615E-08	2.52308E-08	9.29231E-08	2.70615E-07	2.85909E-07	1.07818E-07	2.19818E-07	1.54727E-07	9.90667E-08	5.19333E-08	4.82E-08	0.000000235
Microbacterium_amylolyticum	9.93636E-08	0	2.96364E-08	4.19882E-07	3.58462E-08	0	7.07692E-08	0.000000143	8.21818E-08	1.13091E-07	3.95455E-08	3.63545E-07	8.00667E-08	1.044E-07	8.35333E-08	5.70667E-08
Microbacterium_esteraromaticum	7.98545E-07	0	1.71512E-06	2.57318E-06	0.000000598	0	5.04077E-06	5.40692E-06	0.000000903	7.87091E-07	8.00818E-07	0.00000079	4.57133E-07	4.60533E-07	1.28913E-06	4.65733E-07
Microbacterium_lemovicicum	1.43273E-07	0	3.00491E-07	1.30373E-06	4.33462E-07	8.30769E-08	9.32308E-08	3.22231E-07	7.40818E-07	1.23527E-06	5.57455E-07	2.20673E-06	2.56667E-07	6.68733E-07	7.21867E-07	7.38467E-07
Microbacterium_sp._CBA3102	1.36636E-07	3.28182E-08	2.59818E-08	2.02182E-07	9.51538E-08	2.25615E-07	1.28923E-07	1.26154E-07	3.47273E-07	1.39727E-07	2.13091E-07	3.01182E-07	1.298E-07	0.000000075	2.28467E-07	2.656E-07
Microbacterium_oleivorans	3.22182E-07	7.45455E-08	3.25591E-06	3.93727E-07	7.45615E-07	1.19308E-06	5.55623E-06	3.73485E-06	2.75091E-07	3.90455E-07	2.07909E-07	1.97545E-07	1.46827E-06	2.1366E-06	1.71673E-06	1.18853E-06
Curtobacterium_sp._SGAir0471	0	0	0	0	0	4.30769E-08	0	0	0	0	0	0	2.91333E-08	2.38E-08	1.49333E-08	1.98667E-08
Curtobacterium_sp._Csp2	5.42727E-08	5.41818E-08	0	0	0	2.16923E-08	0	0	3.94545E-08	2.87273E-08	0	3.31818E-07	7.38E-08	3.32667E-08	2.39333E-08	1.07067E-07
Curtobacterium_sp._csp3	0	8.86364E-09	0	0	0	2.16923E-08	0	0	0	0	0	0	2.08E-08	0	5.94667E-08	0
Curtobacterium_pusillum	2.98182E-08	8.86364E-09	0	4.79091E-09	0	4.27692E-08	0	0	7.18182E-08	3.62727E-08	5.82727E-08	0	0	0	1.65333E-08	0
Curtobacterium_sp._BH-2-1-1	0	0.000000033	0	1.50909E-07	6.69231E-08	6.47692E-08	0	0	5.46364E-08	0	1.70455E-07	5.64545E-08	4.51333E-08	4.76667E-08	0	1.98667E-08
Curtobacterium_sp._MR_MD2014	2.24818E-07	0.000000057	2.47273E-08	1.03755E-06	2.05385E-08	1.42238E-06	0.000002837	1.84308E-06	2.63727E-07	3.76364E-07	0.000000703	6.28273E-07	8.57333E-08	1.40667E-07	3.368E-07	1.66667E-08
Curtobacterium_flaccumfaciens	0.072833083	0.027560484	0.002907445	0.030846021	0.043761606	0.017707925	0.02006932	0.016355254	0.048845146	0.036582953	0.028541717	0.067434393	0.042587283	0.01287712	0.015138064	0.026121442
Ndongobacter_massiliensis	3.46782E-06	7.52855E-06	1.74545E-06	3.01627E-06	1.58285E-06	2.06369E-06	0.000002317	2.65077E-06	2.60055E-06	2.74064E-06	1.67427E-06	6.36273E-06	1.07587E-06	1.2008E-06	1.20007E-06	9.94933E-07
endosymbiont_'TC1'_of_Trimyema_compressum	5.14636E-07	5.05455E-08	2.15727E-07	4.30636E-07	1.86923E-08	0	3.89231E-07	0.000001006	1.90636E-07	9.05455E-08	1.24091E-07	7.20545E-07	0.000001216	6.13267E-07	1.48467E-06	1.0408E-06
Limnochorda_pilosa	3.02273E-06	2.19018E-06	5.05182E-07	2.48291E-06	1.75423E-06	3.55977E-06	1.63954E-06	1.71608E-06	5.28418E-06	3.06382E-06	3.78291E-06	4.38909E-06	1.49273E-06	2.29393E-06	1.6834E-06	0.000001988
Ezakiella_massiliensis	2.43036E-06	2.51255E-06	6.15091E-07	0.000000494	1.49769E-07	0.000000095	1.17885E-06	9.55923E-07	1.28927E-06	0.000000687	1.80909E-06	7.87182E-07	3.40067E-07	5.866E-07	5.13333E-07	0.000000155
Gudongella_oleilytica	0.000001287	8.21818E-07	2.70373E-06	0.000001248	1.21254E-06	7.30538E-07	6.23615E-07	2.02854E-06	6.59909E-07	8.63364E-07	1.02327E-06	1.08445E-06	9.93867E-07	1.2858E-06	1.25807E-06	1.31273E-06
Tissierellia_sp._JN-28	1.09964E-05	6.36182E-06	3.99182E-06	6.62636E-06	2.46792E-06	0.000004149	4.37862E-06	0.000004535	1.16427E-05	1.76109E-05	6.90282E-06	9.18455E-06	2.66293E-06	2.43467E-06	3.40307E-06	0.000002835
Gottschalkia_acidurici	1.17727E-06	1.24355E-06	6.39818E-07	1.18936E-06	8.56308E-07	0.000000624	1.59892E-06	1.61385E-06	0.000001312	9.84182E-07	2.36736E-06	0.000002468	1.15913E-06	2.74447E-06	1.8468E-06	1.56627E-06
Anaerococcus_mediterraneensis	3.73618E-06	4.33455E-07	4.63364E-07	6.90182E-07	1.14262E-06	4.79077E-07	2.90031E-06	1.97677E-06	0.000003841	0.000005338	5.99845E-06	0.000004098	9.23267E-07	1.27567E-06	2.16727E-06	2.07347E-06
Anaerococcus_prevotii	3.62455E-07	1.16364E-08	3.11727E-07	8.72727E-07	5.62308E-07	0.000000065	1.12331E-06	1.22108E-06	1.95909E-07	5.83818E-07	0.000000405	2.09909E-06	1.13087E-06	1.08927E-06	1.57313E-06	1.97153E-06
Parvimonas_micra	1.07055E-05	1.63155E-06	4.52882E-06	7.25973E-06	3.73769E-06	2.36223E-06	8.18546E-06	1.24231E-05	1.05691E-05	6.64564E-06	1.31936E-05	7.95727E-06	4.35447E-06	7.81987E-06	7.5956E-06	0.000004338
Murdochiella_vaginalis	7.31364E-06	6.1225E-06	3.21459E-06	9.96155E-06	4.67892E-06	3.24162E-06	5.99085E-06	7.66692E-06	1.16809E-05	1.21382E-05	6.29091E-06	7.29364E-06	5.24133E-06	8.47067E-06	6.55867E-06	5.88067E-06
Finegoldia_magna	3.65364E-06	1.55359E-06	1.48109E-06	1.96818E-06	4.43092E-06	1.71662E-06	2.49308E-06	3.44862E-06	4.40764E-06	5.02091E-06	0.000003521	3.73364E-06	3.1738E-06	4.61133E-06	4.74187E-06	4.51047E-06
Peptoniphilus_sp._ING2-D1G	3.29145E-05	2.17496E-05	1.20636E-05	1.00645E-05	1.51277E-06	1.01369E-06	1.15262E-05	1.33138E-05	1.15491E-05	0.00001543	2.10305E-05	6.98291E-06	1.6856E-06	2.8788E-06	2.26933E-06	2.12327E-06
Peptoniphilus_ivorii	5.89636E-06	1.88409E-06	7.87727E-07	3.17882E-06	1.76554E-06	3.61392E-06	4.58854E-06	7.93769E-06	0.000005228	0.00000671	7.36909E-06	7.35318E-06	2.21813E-06	2.18993E-06	2.0502E-06	2.95533E-06
Peptoniphilus_harei	8.49364E-07	2.35727E-07	1.11982E-07	9.25455E-08	4.11692E-07	8.57308E-07	6.93231E-07	1.84985E-06	1.30327E-06	0.000001316	7.14727E-07	7.45364E-07	9.59067E-07	2.78533E-06	1.49453E-06	5.86707E-06
Dialister_pneumosintes	9.92727E-08	6.18091E-07	1.04364E-07	4.11545E-07	5.93846E-08	3.82308E-07	6.96923E-08	2.79385E-07	1.26545E-07	3.62727E-08	1.02727E-08	5.54091E-07	1.91067E-07	2.066E-07	1.3124E-07	8.14667E-08
Dialister_hominis	6.99182E-07	4.97455E-06	4.25364E-07	1.14027E-06	5.97692E-07	1.56308E-07	5.64615E-07	4.96769E-07	7.46455E-07	1.01836E-06	7.52455E-07	2.02336E-06	4.38867E-07	2.922E-07	5.03173E-07	1.38733E-07
Dialister_massiliensis	1.25909E-07	3.53545E-07	3.68545E-08	1.88791E-07	4.47769E-07	2.32315E-06	4.83846E-07	7.04385E-07	2.95818E-07	3.04818E-07	4.17364E-07	2.80455E-07	1.20533E-07	4.70933E-07	5.448E-07	1.74333E-07
Veillonella_sp._T1-7	0	1.16364E-08	4.90909E-08	0	0	1.14615E-07	0	8.57692E-08	2.72727E-08	0	0	3.94545E-08	0	8.31333E-08	2.99333E-08	3.37333E-08
Veillonella_dispar	5.39364E-07	3.45727E-07	1.64545E-07	2.77636E-07	3.34615E-08	0	1.86923E-07	7.58692E-07	3.21273E-07	1.79527E-06	1.99018E-06	4.37909E-07	1.83267E-07	0	4.20733E-07	1.90467E-07
Veillonella_rodentium	1.11118E-06	2.04909E-07	1.04545E-06	4.97182E-07	5.51308E-07	2.53923E-07	1.52154E-06	2.04654E-06	9.00091E-07	7.64727E-07	1.23718E-06	1.03545E-06	5.32467E-07	8.508E-07	5.26467E-07	0.000000875
Veillonella_atypica	2.65364E-07	5.02636E-07	2.96364E-08	4.40909E-08	1.52154E-07	2.28923E-07	0.000000114	1.23692E-07	1.02455E-07	1.32545E-07	3.95455E-08	1.18909E-07	1.70667E-07	4.048E-07	9.31333E-08	2.50667E-07
Veillonella_parvula	0.000000692	5.64364E-07	4.55273E-07	1.85727E-07	1.07069E-06	2.23846E-07	1.78385E-07	6.86846E-07	7.80818E-07	4.17455E-07	1.11264E-06	4.84909E-07	6.176E-07	1.00413E-06	1.0262E-06	1.06667E-06
Megasphaera_elsdenii	9.17364E-06	3.86864E-06	5.67775E-06	6.20273E-06	0.00000575	4.52354E-06	8.91923E-06	8.66846E-06	9.18182E-06	7.69636E-06	7.83545E-06	1.19736E-05	4.55793E-06	0.000008132	0.000006148	5.75933E-06
Megasphaera_stantonii	1.32155E-05	8.90327E-06	5.46064E-06	1.02991E-05	5.29585E-06	8.14762E-06	7.95615E-06	7.35846E-06	1.10645E-05	0.0000127	1.17409E-05	0.00001264	6.04333E-06	6.77933E-06	6.15467E-06	7.05467E-06
Phascolarctobacterium_faecium	4.81727E-06	2.92568E-06	1.28936E-06	4.89364E-06	2.59977E-06	3.24831E-06	4.85692E-06	6.11615E-06	3.82727E-06	3.70245E-06	4.68709E-06	7.29364E-06	2.15027E-06	2.6884E-06	2.3976E-06	1.52587E-06
Acidaminococcus_intestini	0.00000728	8.32955E-06	2.69245E-06	7.52182E-06	4.48192E-06	0.000003257	4.35692E-06	9.57615E-06	8.65818E-06	6.16545E-06	5.68818E-06	1.01745E-05	3.8758E-06	6.0918E-06	5.18733E-06	0.000005584
Acidaminococcus_fermentans	1.42527E-05	0.000008685	3.71641E-06	1.03255E-05	8.29923E-06	3.18377E-05	1.29715E-05	1.04877E-05	1.44264E-05	1.61891E-05	1.58073E-05	0.00002294	8.24867E-06	1.14427E-05	1.16973E-05	1.00393E-05
Sporomusa_termitida	1.71064E-06	1.83409E-06	7.31091E-07	1.76909E-06	0.000001268	2.25715E-06	1.59177E-06	1.13477E-06	2.65055E-06	0.000002096	2.56309E-06	3.52182E-06	1.06267E-06	1.9838E-06	2.01573E-06	1.51587E-06
Methylomusa_anaerophila	3.51991E-06	2.22018E-06	1.37582E-06	0.000002625	3.37431E-06	1.97808E-06	2.95323E-06	3.24762E-06	2.82282E-06	2.67809E-06	3.67027E-06	5.93909E-06	2.22593E-06	4.88047E-06	3.69107E-06	0.00000308
Pelosinus_fermentans	9.05173E-06	0.000007599	4.19682E-06	5.50073E-06	5.04308E-06	9.39377E-06	5.94538E-06	6.26462E-06	4.77727E-06	4.81473E-06	5.11636E-06	1.16827E-05	2.3528E-06	2.4446E-06	2.72327E-06	2.70113E-06
Pelosinus_sp._UFO1	3.43355E-06	3.33409E-07	9.09364E-07	1.24591E-06	1.57323E-06	8.66692E-07	2.76615E-06	3.10469E-06	3.75527E-06	6.16727E-06	4.61818E-06	3.80527E-06	1.8968E-06	2.5288E-06	1.49887E-06	0.000002435
Megamonas_hypermegale	5.44909E-06	1.53718E-06	1.99868E-06	1.82127E-06	1.86008E-06	1.01038E-06	0.000003358	5.08085E-06	4.88091E-06	7.10545E-06	6.50373E-06	5.95182E-06	2.00627E-06	1.91247E-06	2.48087E-06	2.95453E-06
Megamonas_funiformis	5.40527E-06	1.10032E-06	1.31364E-06	3.87682E-06	3.26569E-06	1.77046E-06	5.60469E-06	9.24269E-06	5.01036E-06	0.00000825	8.99727E-06	9.94364E-06	2.84267E-06	3.14333E-06	3.2046E-06	3.08373E-06
Pectinatus_frisingensis	1.83818E-05	3.23545E-06	3.31545E-06	0.000005989	6.64669E-06	4.98192E-06	1.09146E-05	1.05623E-05	2.18655E-05	3.53916E-05	2.14973E-05	1.77118E-05	6.04773E-06	5.56727E-06	4.95533E-06	9.07907E-06
Selenomonas_sp._oral_taxon_478	4.02455E-07	1.63909E-07	1.72364E-07	4.94909E-07	3.81846E-07	4.65538E-07	0.000000277	5.99462E-07	1.85182E-07	3.17727E-07	2.05091E-07	0.000000631	2.14733E-07	5.17067E-07	3.82933E-07	5.57067E-07
Selenomonas_sputigena	4.12636E-06	0.000005528	2.38482E-06	3.24636E-06	2.20054E-06	0.000002177	4.88577E-06	4.58962E-06	3.01636E-06	4.78736E-06	4.72009E-06	4.83518E-06	2.0198E-06	2.9996E-06	2.30067E-06	1.94573E-06
Selenomonas_ruminantium	9.29364E-06	6.74618E-06	3.86018E-06	6.93091E-06	5.78077E-06	1.42978E-05	6.42231E-06	7.34077E-06	8.06818E-06	8.06636E-06	9.06291E-06	1.40736E-05	4.3076E-06	4.69267E-06	0.00000485	0.000005156
Selenomonas_sp._oral_taxon_136	2.92827E-06	4.90673E-06	1.73618E-06	2.65091E-06	1.21415E-06	1.92531E-06	1.98331E-06	2.26146E-06	1.42645E-06	2.56964E-06	2.18064E-06	4.32364E-06	7.25067E-07	1.05107E-06	6.206E-07	5.07733E-07
Selenomonas_sp._oral_taxon_920	3.15818E-06	1.27436E-06	5.77091E-07	1.65473E-06	1.54677E-06	9.86769E-07	2.22615E-06	1.69262E-06	2.00964E-06	3.00464E-06	3.08582E-06	5.62036E-06	2.09453E-06	2.64267E-06	1.62233E-06	2.3274E-06
Selenomonas_sp._Marseille-Q3039	4.38318E-06	3.65555E-06	9.65909E-07	3.78109E-06	3.48485E-06	3.42515E-06	5.58838E-06	3.24723E-06	4.09382E-06	4.00191E-06	4.57891E-06	5.80455E-06	2.70293E-06	3.14887E-06	2.80867E-06	3.33707E-06
Selenomonas_sp._oral_taxon_126	3.40909E-06	1.66673E-06	9.70455E-07	0.000001274	0.000001644	5.39923E-07	1.94754E-06	1.90531E-06	3.31991E-06	1.55336E-06	2.15118E-06	2.91909E-06	9.61733E-07	1.9424E-06	0.000001637	2.53887E-06
Turicibacter_sp._H121	7.27727E-06	1.51055E-06	0.000149941	0.000149051	2.01431E-06	6.41923E-07	0.000482216	0.000332827	5.86982E-06	1.03809E-05	0.000442199	0.000184633	1.84613E-06	9.52007E-06	0.000268603	0.000141731
Turicibacter_sanguinis	4.10773E-06	1.51173E-06	7.34081E-05	7.88143E-05	0.000001898	1.54523E-06	0.000215571	0.000175255	3.55455E-06	4.25582E-06	0.000225118	9.37023E-05	1.1718E-06	4.6824E-06	0.000136879	7.07939E-05
Intestinibaculum_porci	4.99727E-06	2.33827E-06	2.21636E-06	4.69727E-06	2.85692E-06	5.91723E-06	3.19038E-06	3.84254E-06	5.19636E-06	4.55636E-06	3.79636E-06	6.76091E-06	2.85107E-06	3.65293E-06	4.65493E-06	3.24933E-06
Amedibacterium_intestinale	3.53273E-05	3.46715E-05	1.07427E-05	2.03415E-05	2.04223E-05	3.41015E-05	2.45585E-05	2.19969E-05	0.00003293	3.80273E-05	3.38364E-05	3.72082E-05	1.64067E-05	2.01907E-05	0.000019888	2.09133E-05
Erysipelothrix_piscisicarius	1.35764E-06	1.65091E-07	4.21036E-07	6.32909E-07	3.47615E-07	8.90769E-08	6.77923E-07	4.14462E-07	2.65545E-07	6.31455E-07	5.61182E-07	1.18545E-06	2.19933E-07	9.92667E-08	3.71133E-07	1.41867E-07
Erysipelothrix_larvae	3.56727E-07	6.20273E-07	4.31982E-07	3.73455E-07	2.94308E-07	3.13308E-07	3.85231E-07	0.00000046	4.81545E-07	0.000000223	7.79818E-07	1.03327E-06	1.24933E-07	0.000000419	6.29533E-07	4.55933E-07
Erysipelothrix_rhusiopathiae	1.01718E-06	5.10455E-07	0.000000236	9.42091E-07	1.38662E-06	1.73692E-07	8.94538E-07	9.03462E-07	7.43455E-07	8.87364E-07	1.19391E-06	1.87018E-06	1.22613E-06	1.18873E-06	1.68333E-06	1.44373E-06
Erysipelothrix_inopinata	5.35727E-07	4.79727E-07	1.24545E-07	2.36345E-06	2.15438E-06	9.72308E-08	1.18246E-06	1.94669E-06	2.09455E-07	5.72727E-07	1.40782E-06	2.27636E-06	2.35807E-06	2.46393E-06	4.0042E-06	6.40753E-06
Erysipelothrix_sp._HDW6B	2.12364E-07	3.13773E-07	0.000000046	1.09064E-07	4.25077E-07	8.33692E-07	0.000000135	5.70154E-07	6.95182E-07	4.19182E-07	0.00000087	5.38091E-07	7.53267E-07	4.062E-07	7.03067E-07	0.000000658
Erysipelothrix_sp._HDW6C	8.16364E-07	4.53682E-07	4.80818E-07	1.45091E-06	2.01938E-06	6.47692E-07	9.33615E-07	1.04685E-06	5.73818E-07	3.62091E-07	1.39118E-06	7.04182E-07	1.74793E-06	3.66333E-06	2.9986E-06	3.0702E-06
Erysipelothrix_sp._HDW6A	2.43864E-06	6.75273E-07	5.01727E-07	1.10345E-06	2.23115E-06	1.38565E-05	2.87846E-06	2.79354E-06	2.66118E-06	3.16873E-06	3.32545E-06	2.97818E-06	2.52667E-06	2.85293E-06	4.21493E-06	4.57467E-06
Faecalibaculum_rodentium	0.001195089	0.000202933	0.000266502	0.000677935	0.001967304	0.025955141	0.003837339	0.000921285	0.001343975	0.00108254	0.002032945	0.001455452	0.002071725	0.002094948	0.003391592	0.002758441
Faecalitalea_cylindroides	0.000109759	0.000067879	2.72117E-05	5.46764E-05	4.05657E-05	6.22888E-05	5.12025E-05	5.52258E-05	6.54364E-05	8.51726E-05	8.46077E-05	8.16894E-05	3.78449E-05	5.83219E-05	4.35309E-05	5.94679E-05
Longibaculum_sp._KGMB06250	0.000247124	0.000243697	7.64351E-05	9.30827E-05	4.37769E-05	4.02008E-05	8.19228E-05	9.00595E-05	0.000118416	9.90934E-05	0.000136706	0.000113639	5.31088E-05	0.000079439	5.57231E-05	5.20315E-05
Erysipelatoclostridium_ramosum	9.58273E-06	2.18236E-06	2.9546E-05	6.23882E-06	3.74769E-06	2.73716E-05	0.000007213	4.96538E-06	7.44727E-06	5.94455E-06	0.00001027	0.00000946	0.000005538	6.88467E-06	0.000005964	7.69533E-06
[Clostridium]_innocuum	0.001450277	0.00085501	0.000342669	0.000729427	0.000629192	0.000670031	0.000767478	0.00074226	0.001420368	0.00185352	0.001340338	0.001180061	0.000601985	0.000764807	0.000607922	0.000641217
Sporolactobacillus_terrae	1.21982E-06	1.80818E-07	2.66218E-07	1.38582E-06	2.26462E-07	1.94446E-06	3.58077E-07	6.83462E-07	4.82727E-07	4.97909E-07	0.000000562	1.23282E-06	3.95333E-07	7.362E-07	9.546E-07	0.000001242
Alicyclobacillus_acidocaldarius	2.27791E-06	9.32182E-07	3.98818E-07	0.000002188	9.09846E-07	6.17008E-06	1.74492E-06	1.00946E-06	1.54791E-06	1.60545E-06	1.75218E-06	2.05009E-06	6.30533E-07	1.4322E-06	1.47107E-06	1.33813E-06
Tumebacillus_algifaecis	1.53936E-06	5.47727E-07	0.000000747	3.34818E-07	1.69615E-06	3.70077E-06	3.97108E-06	2.79138E-06	2.97545E-07	7.60455E-07	1.42473E-06	1.03818E-06	3.742E-07	7.656E-07	0.000001325	1.19553E-06
Tumebacillus_avium	3.77182E-06	2.52418E-06	2.09016E-06	1.70145E-06	1.32292E-06	2.01585E-06	9.53615E-07	1.65923E-06	1.21982E-06	1.11191E-06	2.80364E-06	2.78136E-06	8.88933E-07	7.51667E-07	9.78733E-07	5.83067E-07
Kyrpidia_tusciae	7.04182E-07	0.000000215	1.40582E-07	3.36182E-07	4.44231E-07	1.71923E-07	7.00385E-07	0.000000289	0.000000755	9.29545E-07	1.06955E-06	9.84909E-07	1.844E-07	4.48267E-07	3.27173E-07	1.23733E-07
Kyrpidia_spormannii	6.19636E-07	1.15818E-07	2.13727E-07	5.71455E-07	5.25538E-07	3.98462E-07	8.05077E-07	6.49923E-07	7.57364E-07	5.68909E-07	4.16455E-07	0.000000537	6.37267E-07	8.36733E-07	7.58333E-07	6.884E-07
Novibacillus_thermophilus	1.39982E-06	8.04273E-07	5.29582E-07	3.72791E-07	1.89846E-07	0.000000634	3.69769E-07	3.81923E-07	4.38818E-07	4.86909E-07	7.38636E-07	0.000000789	3.39267E-07	0.000000166	3.30133E-07	2.71133E-07
Staphylospora_marina	1.49391E-06	0.000001235	9.00036E-07	1.69164E-06	9.75615E-07	3.19923E-07	0.000001442	1.33608E-06	1.32345E-06	0.00000168	1.74064E-06	2.54273E-06	6.688E-07	1.5118E-06	1.4996E-06	7.90067E-07
Laceyella_sacchari	4.95364E-07	1.15264E-06	0.000000046	0.000000294	3.01231E-07	1.85854E-06	2.93308E-07	2.19769E-07	1.36327E-06	2.02818E-07	4.97273E-07	6.71818E-07	1.47667E-07	5.676E-07	6.87133E-07	4.04467E-07
Thermoactinomyces_vulgaris	0.000001348	1.14423E-06	3.90036E-07	1.52655E-06	7.84154E-07	3.56231E-07	6.65231E-07	1.26392E-06	2.54218E-06	0.000001213	2.08173E-06	2.07636E-06	1.27607E-06	8.79133E-07	1.66333E-06	1.47507E-06
Kroppenstedtia_eburnea	1.50727E-06	2.93918E-06	8.04936E-07	9.82273E-07	1.35015E-06	3.65346E-06	1.00546E-06	1.16315E-06	2.35655E-06	1.32718E-06	8.32182E-07	2.35236E-06	7.57867E-07	1.0174E-06	1.30867E-06	1.43953E-06
Kroppenstedtia_pulmonis	0.000000404	7.68273E-07	0.000000324	1.19936E-06	1.48385E-07	4.04231E-07	6.70846E-07	5.07231E-07	7.51545E-07	1.21791E-06	1.10345E-06	6.49909E-07	3.68267E-07	6.15933E-07	3.75867E-07	3.20067E-07
Brochothrix_thermosphacta	1.23227E-06	3.23818E-07	0.000000567	7.49818E-07	2.88462E-08	0	8.72231E-07	9.22846E-07	1.68327E-06	1.05145E-06	1.33136E-06	1.08555E-06	1.54733E-07	3.282E-07	6.146E-07	1.488E-07
Listeria_welshimeri	5.57273E-08	1.16364E-08	0	2.87273E-08	1.06615E-07	5.03308E-07	2.04769E-07	1.28231E-07	3.14273E-07	9.36364E-08	0	1.02591E-07	0	3.11333E-08	1.49333E-08	6.96667E-08
Listeria_innocua	5.92364E-07	7.78909E-07	3.52273E-07	0.000000172	2.12692E-07	5.91538E-08	1.05385E-07	5.97308E-07	3.44155E-06	1.83182E-07	0.00000109	5.87909E-07	7.14667E-08	1.71133E-07	5.17667E-07	4.88933E-07
Listeria_seeligeri	1.68273E-07	0.000000047	3.27273E-08	1.61545E-07	6.44154E-07	3.15923E-07	4.46154E-07	3.87462E-07	4.70909E-07	2.46455E-07	6.19273E-07	1.18873E-06	3.392E-07	6.80267E-07	4.56133E-07	4.62333E-07
Listeria_weihenstephanensis	1.30618E-06	0.000000307	4.97636E-07	9.35182E-07	1.13015E-06	4.77231E-07	1.52177E-06	8.22923E-07	1.02855E-06	4.02818E-07	8.55545E-07	1.69136E-06	5.302E-07	6.586E-07	7.37667E-07	6.33133E-07
Listeria_sp._PSOL-1	1.34036E-06	5.81091E-07	3.71727E-07	3.85091E-07	1.45823E-06	5.99692E-07	1.53462E-07	7.53846E-07	8.12455E-07	3.48091E-07	8.08091E-07	1.86473E-06	5.25267E-07	9.02067E-07	1.25347E-06	1.06733E-06
Listeria_grayi	6.00182E-07	0	1.89164E-07	1.00909E-07	2.83692E-07	2.25846E-07	0.000000203	5.39231E-07	3.65364E-07	1.05364E-07	9.22818E-07	7.58636E-07	2.04267E-07	7.55533E-07	5.622E-07	7.38933E-07
Listeria_ivanovii	2.39591E-06	9.30091E-07	2.50182E-07	1.12418E-06	1.70377E-06	2.32923E-07	1.61723E-06	7.52231E-07	2.24418E-06	1.95182E-06	1.02555E-06	0.000002494	2.34913E-06	1.79507E-06	1.91993E-06	1.73753E-06
Listeria_monocytogenes	5.88636E-06	3.69327E-06	0.000001715	4.76182E-06	4.37546E-06	8.72792E-06	7.49154E-06	2.75408E-06	1.30858E-05	5.27945E-06	4.97727E-06	4.31573E-06	3.40373E-06	4.67693E-06	3.80693E-06	1.43767E-06
Viridibacillus_sp._JNUCC-6	4.87273E-08	4.93136E-07	7.23636E-08	2.30909E-07	2.63769E-07	5.19231E-08	7.45385E-08	1.95846E-07	1.19627E-06	1.50273E-07	6.58636E-07	0.0000001	1.102E-07	0.000000075	1.074E-07	7.12667E-08
Planomicrobium_glaciei	1.30718E-06	8.91727E-07	3.04982E-07	0.000000364	7.26077E-07	0.000000252	7.43846E-07	6.54308E-07	8.56545E-07	6.59545E-07	9.04182E-07	1.16191E-06	1.87067E-07	3.92333E-07	5.00867E-07	7.872E-07
Rummeliibacillus_stabekisii	1.72455E-06	5.89273E-07	4.95982E-07	0.000001705	2.16692E-06	0	1.27585E-06	1.27215E-06	5.72091E-07	6.86273E-07	1.75827E-06	1.56109E-06	7.12933E-07	1.26867E-06	1.93993E-06	7.136E-07
Kurthia_sp._11kri321	3.23727E-07	5.03636E-08	8.25455E-09	1.51636E-07	1.93154E-07	1.84923E-07	1.50846E-07	6.94615E-07	1.16509E-06	4.84091E-07	1.01527E-06	6.92545E-07	9.67333E-08	8.69333E-08	2.33307E-07	3.45467E-07
Kurthia_zopfii	7.77273E-08	8.18182E-08	8.06364E-08	9.23636E-08	6.30769E-08	2.02923E-07	8.69231E-08	2.28846E-07	4.30091E-07	7.02727E-08	2.07727E-07	2.15182E-07	2.64667E-08	8.13333E-08	2.24667E-08	8.94E-08
Solibacillus_silvestris	6.73455E-07	1.03173E-06	0.000000328	3.53091E-07	6.43462E-07	1.04008E-06	2.38615E-07	3.42308E-07	0.000000856	3.64091E-07	1.55455E-06	1.20691E-06	1.358E-07	2.266E-07	5.04067E-07	3.17867E-07
Solibacillus_sp._R5-41	0	1.68182E-08	6.29E-08	1.23818E-07	5.19385E-07	2.52308E-08	7.19231E-07	3.55615E-07	8.59545E-07	0	5.06909E-07	5.88273E-07	2.576E-07	9.82867E-07	7.94067E-07	3.30333E-07
Paenisporosarcina_antarctica	6.00545E-07	1.16364E-08	1.16545E-07	2.10091E-07	1.93385E-07	8.27692E-08	1.29385E-07	7.17385E-07	7.25455E-07	2.15818E-07	7.34091E-07	1.70909E-07	2.376E-07	2.46067E-07	0.000000478	4.73667E-07
Paenisporosarcina_sp._K2R23-3	4.52273E-07	0	0	3.94545E-08	8.42846E-07	0	2.68154E-07	1.90769E-08	5.96091E-07	0.000000047	1.06545E-07	0.000000123	5.598E-07	7.02733E-07	8.67533E-07	6.21533E-07
Jeotgalibacillus_malaysiensis	5.82182E-07	4.38455E-07	4.65818E-08	1.96273E-06	1.63462E-06	2.11538E-07	4.19308E-07	1.56523E-06	5.55273E-07	4.92182E-07	0.000001081	1.97182E-06	1.8742E-06	1.9978E-06	3.09613E-06	3.67147E-06
Ureibacillus_thermosphaericus	8.46182E-07	3.48727E-07	3.33727E-07	4.00727E-07	0.000000377	8.92615E-07	8.05923E-07	1.07662E-06	1.41636E-06	7.21455E-07	4.91091E-07	1.03882E-06	4.116E-07	9.868E-07	5.062E-07	8.56933E-07
Sporosarcina_sp.	0	0	0	0	4.13846E-08	0	0	3.22308E-08	0	0	0	7.13636E-09	0	0	0	0
Sporosarcina_sp._P33	0	0	0	0	0	0	0	3.22308E-08	0.000001258	0	6.68182E-08	3.16364E-08	0	0	0	0
Sporosarcina_sp._resist	0	0	0	5.48818E-08	0	0	0	0	6.01545E-07	5.22727E-08	3.95455E-08	1.08091E-07	0	0	5.94E-08	0
Sporosarcina_psychrophila	0	0	1.48182E-07	2.59091E-08	7.02308E-08	9.16154E-07	3.77692E-08	1.52692E-07	4.08818E-07	0.000000028	1.32636E-07	8.59818E-07	0.000000053	0.000000047	2.55667E-07	0.000000106
Sporosarcina_pasteurii	1.94455E-07	1.55545E-07	1.65545E-07	1.58545E-07	1.55077E-07	6.53077E-08	1.26308E-07	6.88231E-07	0.00001087	2.64727E-07	1.79082E-06	7.73545E-07	0.000000169	1.84667E-07	6.026E-07	5.53333E-07
Sporosarcina_sp._PTS2304	1.00027E-06	2.47027E-06	4.04636E-07	8.39091E-07	5.91538E-07	2.27908E-06	9.94923E-07	2.19369E-06	2.81255E-06	8.78818E-07	1.72664E-06	1.30664E-06	9.94133E-07	7.40867E-07	0.000001191	0.000000836
Sporosarcina_sp._P37	0.000000047	0	0	1.91818E-08	0.000000112	0	1.95846E-07	0	1.15455E-07	0	1.04273E-07	2.14545E-08	2.72E-08	1.168E-07	2.02267E-07	5.77333E-08
Sporosarcina_ureilytica	1.05545E-06	8.57273E-08	0.000000387	1.14091E-06	0.000000604	1.73923E-07	1.13392E-06	1.34438E-06	1.37965E-05	2.89091E-07	0.000002412	9.86818E-07	7.61133E-07	7.91133E-07	8.79133E-07	1.07633E-06
Sporosarcina_ureae	7.26091E-07	4.91364E-08	4.94636E-07	3.23273E-07	4.79692E-07	1.14154E-06	9.82077E-07	2.15738E-06	1.08161E-05	5.62091E-07	1.15564E-06	1.06991E-06	6.712E-07	8.55333E-07	9.884E-07	6.368E-07

Planococcus_halocryophilus	5.92727E-08	0	0	0	1.49462E-07	3.33846E-07	0	4.44615E-08	1.55273E-07	0	0	0	2.09333E-08	1.19267E-07	1.19733E-07	0
Planococcus_donghaensis	0	0	0	0	0	0	0	7.66923E-08	3.11545E-07	3.62727E-08	1.33636E-07	3.94545E-08	2.09333E-08	0	3.75733E-08	3.67333E-08
Planococcus_sp._MB-3u-03	2.20909E-07	0	2.18182E-07	1.55909E-07	1.76385E-07	2.77923E-07	5.26154E-08	0.000000171	3.98909E-07	0.000000299	1.78636E-07	7.79273E-07	6.94E-08	6.80667E-08	1.60707E-07	9.12667E-08
Planococcus_faecalis	6.48545E-07	4.30273E-07	9.60909E-08	2.44364E-07	3.25615E-07	2.30923E-07	5.45923E-07	0.000000166	8.12727E-08	2.42909E-07	5.93364E-07	5.04545E-07	2.94667E-08	1.77533E-07	3.00867E-07	2.77933E-07
Planococcus_maritimus	0	0	4.12727E-09	1.33455E-07	0	4.13846E-08	5.36923E-08	0.000000077	2.33364E-07	0.000000047	7.29091E-08	7.07273E-08	0	0	2.99733E-08	1.98667E-08
Planococcus_kocurii	1.85273E-07	0	1.15036E-07	2.55182E-08	0	2.95385E-08	0	0	9.15455E-08	8.08182E-08	5.09455E-07	8.94545E-08	0	0	6.54E-08	0
Planococcus_plakortidis	5.41636E-07	5.57045E-07	3.65818E-07	7.34727E-07	1.10615E-07	1.79231E-08	4.40385E-07	3.51231E-07	4.28545E-07	2.40636E-07	4.01455E-07	9.81364E-07	1.97333E-07	1.26667E-07	1.27733E-07	1.77333E-07
Planococcus_rifietoensis	9.30909E-08	9.06364E-08	4.37273E-08	4.24273E-08	8.36154E-08	0	7.43846E-08	1.99385E-07	2.27727E-07	2.85455E-08	0	6.21818E-08	0	3.48E-08	6.91067E-08	1.18667E-07
Planococcus_antarcticus	2.22764E-06	1.65182E-07	0.000000214	3.72455E-08	5.40538E-07	5.73077E-07	2.23385E-06	1.91377E-06	1.79945E-06	0.000003771	3.08709E-06	2.36145E-06	0.000000484	3.66333E-07	4.378E-07	1.26933E-06
Planococcus_dechangensis	2.00545E-07	9.20909E-08	5.48182E-08	1.76636E-07	0.000000371	0	1.73231E-07	2.68538E-07	6.13091E-07	6.34182E-07	2.95636E-07	6.14182E-07	0.000000225	4.44533E-07	3.05933E-07	2.48267E-07
Planococcus_versutus	1.47273E-07	1.16364E-08	2.92455E-08	3.14818E-07	3.58462E-08	0	0	1.04615E-07	2.50273E-07	0.000000062	2.76364E-08	1.23091E-07	2.556E-07	3.524E-07	5.244E-08	3.02667E-07
Planococcus_sp._PAMC_21323	4.92727E-07	7.40909E-08	4.12727E-09	2.78182E-07	9.57308E-07	1.77692E-07	5.26154E-08	9.91538E-08	5.45818E-07	5.22727E-08	8.70909E-08	2.12364E-07	8.28533E-07	7.03333E-07	1.00513E-06	4.50467E-07
Planococcus_sp._Y42	1.37155E-06	1.79382E-06	5.00273E-07	1.29609E-06	1.19546E-06	1.70008E-06	8.23846E-07	1.16246E-06	1.22655E-06	1.02945E-06	9.67091E-07	1.14282E-06	7.54267E-07	1.72807E-06	1.25267E-06	1.34993E-06
Exiguobacterium_sp._U13-1	0	0	0	0	0	0	0	0	0	0	0	0	0	0	1.12667E-08	4.04E-08
Exiguobacterium_acetylicum	0	0	2.87273E-08	0	0	0	0	0	0	0	0	0	0	0	0	0
Exiguobacterium_sp._MH3	0	0	0	0	0	2.95385E-08	0	0	0	0	1.02727E-08	0	0	0	0	0
Exiguobacterium_mexicanum	0	0	3.70909E-08	4.89091E-08	3.58462E-08	1.99077E-07	2.63077E-08	1.06462E-07	0.000000125	6.66364E-08	1.85818E-07	1.43182E-07	0.000000088	8.17333E-08	4.44E-09	0
Exiguobacterium_sp._ZWU0009	0	0	2.96364E-08	0	0	0	0	0	0	5.22727E-08	0	0	0	0	0	0
Exiguobacterium_sp._S3-2	9.78636E-07	0.000000065	0	3.10909E-08	0	0	0	0	2.55909E-07	1.52909E-07	1.59182E-07	1.19864E-07	0	0	0	0
Exiguobacterium_sp._AT1b	9.37545E-07	0	4.17636E-07	6.41091E-07	0.00000023	0	5.26615E-07	5.14154E-07	3.22545E-07	3.96818E-07	9.35545E-07	7.72636E-07	3.064E-07	3.61533E-07	7.39907E-07	5.57933E-07
Exiguobacterium_sp._Helios	1.07136E-06	8.99091E-08	7.13636E-08	5.14545E-08	7.18077E-07	9.72308E-08	3.78077E-07	2.71692E-07	1.19327E-06	1.48355E-06	1.67845E-06	1.90345E-06	0.000000908	0.000000436	6.39333E-07	9.40333E-07
Exiguobacterium_sibiricum	0	0	2.07636E-08	0	0	0	2.63077E-08	0.000000011	0	0	0	7.13636E-09	4.48667E-08	0.000000014	4.44E-09	6.33333E-08
Exiguobacterium_antarcticum	5.93636E-08	9.80909E-08	0	6.74545E-08	0.000001205	1.21238E-06	1.16154E-07	9.73385E-07	3.92182E-07	3.06364E-07	3.77727E-07	5.81727E-07	8.95933E-07	5.59667E-07	1.35207E-06	1.0786E-06
Exiguobacterium_sp._N4-1P	3.07391E-05	1.10301E-05	1.79545E-06	1.39782E-05	1.61623E-05	2.30288E-05	7.85692E-06	8.21385E-06	1.76709E-05	1.18736E-05	0.0000192	3.25272E-05	1.45613E-05	7.86047E-06	0.000006558	0.000010306
Gemella_sp._oral_taxon_928	0	0	0	0	0	0	0	0	0	0	0	0	0	2.38E-08	4.82E-08	4.95333E-08
Gemella_morbillorum	1.64727E-07	0	1.77427E-07	6.36455E-07	0.000000581	9.04615E-08	5.01308E-07	4.90385E-07	0	2.13455E-07	0.000000721	2.785E-07	8.93667E-07	8.96133E-07	1.32853E-06	1.33573E-06
Gemella_haemolysans	1.61409E-06	2.805E-07	2.794E-07	3.50273E-07	4.02846E-07	0	6.31923E-07	1.25523E-06	1.75182E-06	8.53909E-07	2.16891E-06	3.10745E-06	1.45447E-06	9.19933E-07	0.000001368	9.94667E-07
Gemella_sanguinis	1.29973E-06	0.000000065	9.02591E-07	1.31018E-06	1.63808E-06	4.13846E-08	5.48385E-07	1.76769E-06	0.000000294	4.55545E-07	6.18273E-07	1.99486E-06	1.8834E-06	1.18167E-06	2.58453E-06	3.90867E-06
Salinicoccus_halodurans	1.66909E-07	8.39091E-08	0.000000302	7.24545E-08	8.61538E-08	4.30769E-08	8.87692E-08	2.57385E-07	4.43091E-07	8.70909E-08	2.95818E-07	2.81273E-07	8.00667E-08	6.714E-07	4.39667E-07	1.57533E-07
Macrococcus_sp._IME1552	0	0	7.87E-08	5.27909E-08	1.01846E-07	0	1.60692E-07	0.000000022	3.82636E-07	0.000000034	2.94364E-07	1.66727E-07	8.58E-08	0	2.75133E-07	1.98133E-07
Macrococcus_caseolyticus	0	0	2.47545E-07	3.09182E-07	2.88462E-08	1.10462E-07	5.61923E-07	9.14385E-07	0	0.000000034	5.19727E-07	2.85636E-07	0	0.000000028	2.6744E-07	3.24667E-07
Macrococcus_canis	7.80364E-07	0	2.22664E-07	2.38636E-07	4.77923E-07	1.94985E-06	6.44615E-07	3.43308E-07	6.68818E-07	1.46955E-06	1.68882E-06	1.40473E-06	3.65267E-07	7.55733E-07	7.68533E-07	5.43467E-07
Auricoccus_indicus	3.73636E-07	0.00000026	7.53182E-08	4.61909E-07	2.93769E-07	2.95385E-08	4.99846E-07	2.47846E-07	6.98182E-07	6.42545E-07	4.35545E-07	2.62364E-07	2.054E-07	7.166E-07	5.88733E-07	4.15467E-07
Jeotgalicoccus_saudimassiliensis	2.09609E-06	2.19545E-07	1.7499E-05	3.72991E-06	7.36108E-06	6.44769E-07	1.71655E-05	1.44832E-05	2.58453E-05	2.11036E-06	1.68506E-05	8.23545E-06	4.80907E-06	2.50913E-06	2.29193E-05	2.39539E-05
Staphylococcus_sp._693-2	8.23636E-08	1.16364E-08	0	0	0	0	3.11538E-08	2.47692E-08	9.54545E-08	0	0	0	1.86667E-08	3.50667E-08	1.65333E-08	1.86E-08
Staphylococcus_schweitzeri	1.58182E-08	0	1.63636E-08	0	1.21385E-07	0	0	3.22308E-08	3.94545E-08	0	2.76364E-08	6.41364E-08	7.12667E-08	1.43467E-07	4.48667E-08	7.33333E-08
Staphylococcus_delphini	4.29091E-08	0.000000065	4.50909E-08	5.18182E-09	0	4.13846E-08	6.89231E-08	0	4.51545E-07	0	8.73636E-08	3.16364E-08	1.86667E-08	0	0	0
Staphylococcus_sp._MI_10-1553	0	0	0	0	0	4.13846E-08	0	0	1.73636E-07	0	8.02727E-08	0	0	6.48E-08	0	0
Staphylococcus_auricularis	1.09045E-06	2.80909E-08	2.96364E-07	6.74545E-08	2.05154E-07	3.50923E-07	1.06615E-07	2.39846E-07	9.23636E-08	0	3.83364E-07	3.41273E-07	0	0	1.848E-07	0
Staphylococcus_hyicus	8.50909E-08	1.04545E-08	2.04909E-08	2.03273E-07	0	1.45385E-07	0	1.46923E-08	1.64091E-07	0.000000047	3.02727E-07	3.33182E-07	6.62667E-08	4.66667E-08	1.262E-07	0.000000138
Staphylococcus_schleiferi	0	0	2.88545E-08	0	3.41923E-07	2.94923E-07	0	1.16154E-07	3.94545E-08	1.36273E-07	2.37455E-07	5.30909E-08	0.000000248	1.41267E-07	3.36933E-07	2.268E-07
Staphylococcus_argenteus	0	0	2.04909E-08	0.000000103	8.79231E-08	3.83077E-08	5.96154E-08	3.47692E-07	1.02455E-07	5.22727E-08	1.39909E-07	8.26818E-08	0	1.61333E-08	4.86667E-08	3.84667E-08
Staphylococcus_simiae	1.06364E-06	2.03409E-07	4.01436E-07	1.00182E-07	4.31231E-07	3.44538E-07	0.000000744	7.63154E-07	3.80091E-07	1.34545E-07	8.39545E-07	8.80909E-07	2.434E-07	4.96667E-07	5.194E-07	2.04933E-07
Staphylococcus_kloosii	8.17727E-07	1.81273E-07	5.15436E-07	6.56091E-08	2.34385E-07	3.65692E-07	4.42308E-07	3.53615E-07	2.87545E-07	2.75091E-07	2.97273E-07	5.20545E-07	1.422E-07	1.32467E-07	2.07933E-07	3.36733E-07
Staphylococcus_felis	6.60909E-08	1.92773E-07	1.788E-07	3.87273E-08	0	1.18462E-07	0.00000013	5.03846E-08	4.36273E-07	0.0000001	0.000000078	1.46818E-07	4.86E-08	1.85467E-07	5.93333E-08	5.26E-08
Staphylococcus_piscifermentans	5.94545E-08	0	1.23636E-08	0	1.21692E-07	0	8.92308E-08	3.00769E-08	0	0	0	0	1.17133E-07	2.92067E-07	1.34667E-07	0
Staphylococcus_sp._SDB_2975	5.69091E-08	2.34545E-08	3.70909E-08	4.82727E-08	0	0	9.73846E-08	0.000000073	3.98182E-08	8.32727E-08	3.03273E-07	1.37091E-07	0	0	2.51307E-07	1.34933E-07
Staphylococcus_caprae	1.27636E-07	0	5.61818E-08	7.28182E-08	6.30769E-08	3.93077E-08	1.39385E-07	3.22308E-08	1.64909E-07	3.62727E-08	0.000000179	0.000000202	0	4.23333E-08	0	3.40667E-08
Staphylococcus_warneri	1.68909E-07	0.000000047	1.19455E-07	9.55455E-08	6.46923E-08	2.57538E-07	0	1.90769E-08	0.000000124	1.44545E-07	6.65455E-08	1.86273E-07	7.02667E-08	3.09333E-08	1.69907E-07	4.78E-08
Staphylococcus_sp._AntiMn-1	2.70909E-08	5.46818E-07	2.8478E-06	0	0	1.55227E-05	1.53508E-06	3.99385E-07	1.91464E-06	9.30636E-07	5.02818E-06	4.90591E-06	1.01747E-06	1.208E-07	2.26933E-06	3.62067E-06
Staphylococcus_nepalensis	0.000112849	0.000106044	0.000335576	9.78909E-07	0.000182257	0.00277907	0.00025133	8.07773E-05	0.000254669	0.000160494	0.000662329	0.000616701	0.000144402	2.86345E-05	0.000358835	0.000508816
Staphylococcus_muscae	6.24364E-07	0.000000506	1.71709E-07	1.48182E-07	3.09692E-07	0.000000101	0.00000022	6.48077E-07	3.59909E-07	3.00545E-07	8.42818E-07	4.80909E-07	1.35067E-07	2.35267E-07	2.85667E-07	2.99867E-07
Staphylococcus_saprophyticus	1.26691E-06	5.14909E-07	5.78818E-07	6.44909E-07	7.84846E-07	4.88046E-06	2.73385E-07	4.58462E-07	1.24191E-06	1.50873E-06	1.31591E-06	9.27091E-07	4.94867E-07	5.302E-07	9.48467E-07	9.53667E-07
Staphylococcus_pasteuri	1.02045E-06	7.20636E-07	3.96182E-07	1.80909E-07	2.84538E-07	7.94308E-07	5.56538E-07	0.000000405	1.96455E-07	2.61091E-07	0.000000168	9.50818E-07	5.18667E-08	0.000000117	1.964E-07	1.06133E-07
Staphylococcus_carnosus	8.23636E-08	1.68182E-08	4.54909E-08	2.90091E-07	1.86077E-07	5.80538E-07	2.83231E-07	3.90769E-07	3.33364E-07	2.22182E-07	4.36364E-08	2.13909E-07	4.99333E-08	1.24133E-07	2.56133E-07	4.222E-07
Staphylococcus_stepanovicii	1.82636E-07	0	4.92727E-08	1.51545E-08	2.88462E-08	8.38462E-07	2.44692E-07	2.56769E-07	4.21182E-07	4.66909E-07	8.45818E-07	3.73545E-07	1.47133E-07	1.16667E-07	1.356E-07	2.72733E-07
Staphylococcus_sp._17KM0847	3.08545E-07	0.000000069	3.91909E-07	1.95545E-07	1.60154E-07	7.18385E-07	4.10923E-07	3.24923E-07	1.26182E-07	1.85545E-07	2.47364E-07	1.75455E-07	1.27667E-07	0.000000188	1.9484E-07	2.75067E-07
Staphylococcus_fleurettii	2.96364E-08	0	1.41909E-07	1.86427E-07	0	8.83077E-08	5.96154E-08	1.23154E-07	1.09727E-07	0	1.35364E-07	1.58045E-07	0	3.48E-08	1.57067E-08	1.55267E-07
Staphylococcus_lutrae	2.18909E-07	0	3.70909E-08	0.000000452	4.21615E-07	7.42769E-07	2.03692E-07	4.56077E-07	7.33455E-07	4.44636E-07	8.20091E-07	3.11091E-07	4.39067E-07	2.53467E-07	2.25733E-07	5.236E-07
Staphylococcus_succinus	7.23273E-07	4.32182E-07	3.323E-07	4.24273E-07	7.51077E-07	1.03492E-06	1.34685E-06	4.39846E-07	8.11273E-07	7.29273E-07	9.44909E-07	6.30636E-07	0.000000363	0.000000287	3.43507E-07	5.878E-07
Staphylococcus_pettenkoferi	4.60909E-08	0	0.000000047	4.10909E-08	2.62846E-07	0	3.33077E-08	1.61538E-07	2.89909E-07	0.000000068	3.37455E-07	3.05364E-07	7.22E-08	2.74533E-07	3.83307E-07	5.31067E-07
Staphylococcus_agnetis	0.000001386	8.51364E-07	2.95345E-07	0.000001293	6.63692E-07	4.55385E-07	1.96115E-06	0.00000048	9.44909E-07	8.52182E-07	1.45445E-06	5.59818E-07	2.18867E-07	4.408E-07	4.592E-07	4.66933E-07
Staphylococcus_simulans	7.20636E-07	4.05182E-07	8.10818E-07	6.42727E-08	6.56692E-07	3.33385E-06	6.32769E-07	2.55923E-07	1.01273E-06	1.79545E-07	2.24855E-06	1.81436E-06	1.05467E-07	1.70667E-07	4.00867E-07	0.000000644
Staphylococcus_condimenti	0.000000382	1.95864E-07	3.84636E-07	1.28818E-07	3.75308E-07	3.30923E-07	2.75769E-07	1.26615E-07	6.36545E-07	0.000000424	1.26755E-06	1.85882E-06	3.03733E-07	3.95667E-07	6.73333E-07	5.57933E-07
Staphylococcus_aureus	2.10245E-06	2.78945E-06	5.05591E-06	1.27636E-06	1.86531E-06	1.56088E-05	3.65085E-06	3.24885E-06	5.81545E-06	4.12945E-06	1.04145E-05	9.38091E-06	2.50547E-06	1.71493E-06	5.17387E-06	0.000006286
Staphylococcus_hominis	4.09364E-07	0.000000145	2.39227E-07	5.40091E-07	2.67385E-07	9.54231E-07	8.72077E-07	1.58462E-06	4.25545E-07	6.56455E-07	0.000000732	2.79791E-06	1.54347E-06	5.856E-07	2.13033E-06	9.39133E-07
Staphylococcus_capitis	0	0.000000065	1.42845E-07	1.00209E-06	2.82308E-07	2.13077E-07	2.98308E-07	4.46846E-07	2.72727E-08	0.000000047	3.15727E-07	0.000000387	5.43133E-07	6.91067E-07	1.1922E-06	0.000001046
Staphylococcus_vitulinus	9.57273E-07	0.00000013	7.18845E-07	1.06436E-06	6.80615E-07	1.29108E-06	1.30231E-06	1.53654E-06	8.59545E-07	2.17791E-06	2.01618E-06	2.27764E-06	1.1048E-06	1.04033E-06	3.6938E-06	2.8422E-06
Staphylococcus_lugdunensis	2.67836E-06	1.60091E-07	5.71864E-07	0.000000437	1.12354E-06	2.61377E-06	3.53992E-06	1.88046E-06	1.54009E-06	3.94445E-06	2.44709E-06	1.22182E-06	1.18353E-06	9.03533E-07	1.84433E-06	1.8016E-06
Staphylococcus_sciuri	5.43666E-05	4.41856E-05	9.81182E-06	0.000192565	5.39846E-07	1.47702E-05	6.05385E-07	4.86308E-07	2.13709E-06	2.51864E-06	1.18309E-05	1.16998E-05	0.000000657	6.18067E-07	2.49627E-06	0.000001577
Staphylococcus_chromogenes	0	0	1.11182E-07	2.43636E-08	0.000002003	1.45231E-07	4.33846E-08	4.21308E-07	4.03364E-07	1.36364E-07	1.21909E-07	2.41818E-07	1.39413E-06	0.000001384	1.55091E-06	1.4232E-06
Staphylococcus_haemolyticus	1.78282E-06	1.48409E-07	5.06364E-07	1.76336E-06	7.33462E-07	4.14154E-07	4.83846E-07	1.49515E-06	1.61491E-06	1.36191E-06	1.35036E-06	1.29709E-06	9.886E-07	1.0644E-06	9.62133E-07	0.000000972
Staphylococcus_cohnii	2.22427E-06	1.08209E-06	1.48845E-06	0.000000871	0.000000807	4.26477E-06	2.29108E-06	7.79154E-07	1.50973E-06	1.55391E-06	0.000003294	3.49482E-06	4.68333E-07	7.34933E-07	1.7176E-06	1.61973E-06
Staphylococcus_equorum	1.01945E-06	1.85273E-07	5.32864E-07	1.05755E-06	1.33892E-06	2.60677E-06	1.31423E-06	9.92538E-07	0.000000506	1.66727E-06	1.16209E-06	0.00000162	1.39413E-06	1.1374E-06	1.8346E-06	3.1922E-06
Staphylococcus_epidermidis	1.34855E-06	1.14891E-06	1.89414E-06	5.62818E-07	2.03531E-06	2.58862E-06	1.70115E-06	1.75146E-06	1.66782E-06	6.55727E-07	4.15736E-06	2.66382E-06	1.2944E-06	1.70313E-06	2.1422E-06	2.51353E-06
Staphylococcus_arlettae	6.21182E-07	3.66955E-07	2.09091E-07	6.75909E-07	7.24615E-07	7.80308E-07	3.90692E-07	2.56077E-07	2.22455E-07	2.62182E-07	4.93636E-07	1.85636E-07	1.234E-07	8.21333E-07	5.154E-07	5.466E-07
Staphylococcus_pseudintermedius	1.59409E-06	1.29455E-07	1.87545E-07	5.09364E-07	2.26969E-06	1.45854E-06	3.76077E-07	6.00846E-07	0.000001319	1.23173E-06	1.41355E-06	2.47918E-06	2.63587E-06	2.81247E-06	2.3444E-06	2.60033E-06
Staphylococcus_xylosus	6.17091E-05	0.000121746	2.52109E-06	7.22325E-05	4.67538E-06	0.000164237	1.56792E-06	3.29485E-06	7.75053E-05	0.00052653	0.00000605	2.26795E-05	2.94725E-05	0.000032131	4.02135E-05	4.22357E-05
Staphylococcus_lentus	4.40915E-05	2.69455E-07	0.000102016	1.97155E-07	5.51733E-05	0.001506077	0.000247614	0.000138582	0.000175744	0.000555323	0.000217959	0.000309318	5.73029E-05	0.000041267	0.000445259	0.000340883
Saccharibacillus_brassicae	2.10555E-06	2.31027E-06	1.18882E-06	1.63873E-06	8.48308E-07	0.000001804	0.00000142	9.27923E-07	1.38927E-06	1.13782E-06	2.05618E-06	1.68609E-06	5.41533E-07	4.86133E-07	7.60867E-07	1.18747E-06
Aneurinibacillus_sp._XH2	3.81364E-07	0.000000136	1.36455E-07	3.39091E-07	4.74462E-07	1.06423E-06	6.20385E-07	0.000001341	4.84182E-07	7.09273E-07	1.14909E-07	1.12309E-06	3.52933E-07	6.24067E-07	5.84133E-07	9.49533E-07
Aneurinibacillus_soli	8.49364E-07	4.25409E-07	2.12227E-07	4.36364E-07	5.34077E-07	3.34315E-06	3.14769E-07	6.00769E-07	8.51273E-07	5.25909E-07	5.94636E-07	1.17382E-06	3.59933E-07	7.82933E-07	3.17067E-07	6.12533E-07
Thermobacillus_composti	4.81818E-06	2.24045E-06	3.35798E-06	3.69064E-06	2.00369E-06	3.61985E-06	8.72923E-06	5.75438E-06	2.14773E-06	2.96945E-06	2.97564E-06	3.66064E-06	1.14213E-06	1.43073E-06	0.000001736	8.50933E-07
Cohnella_abietis	7.71909E-07	1.42009E-06	1.27273E-07	3.87182E-07	7.48923E-07	9.37538E-07	3.37615E-07	5.79308E-07	6.34545E-07	4.80364E-07	9.59818E-07	1.06609E-06	0.00000073	4.784E-07	7.8944E-07	7.01933E-07
Cohnella_candidum	2.68473E-06	4.41164E-06	5.33364E-07	1.86455E-06	2.20154E-06	1.57223E-06	2.72431E-06	1.52277E-06	1.35327E-06	1.91818E-06	1.94427E-06	1.89945E-06	1.58653E-06	1.9582E-06	1.35593E-06	1.3964E-06
Cohnella_sp._MFER-1	5.24636E-07	1.37473E-06	1.49127E-07	3.75273E-07	2.99769E-07	1.13154E-06	0.00000044	2.80385E-07	1.11955E-06	5.47818E-07	4.51364E-07	7.88273E-07	2.05867E-07	3.77933E-07	3.29773E-07	3.48667E-07
Cohnella_sp._KS_22	0.000001503	2.42636E-06	6.97436E-07	2.11582E-06	0.000000902	1.31754E-06	1.49008E-06	4.66692E-07	6.92909E-07	9.07636E-07	9.16455E-07	1.41218E-06	4.77267E-07	1.24447E-06	5.09467E-07	6.70267E-07
Brevibacillus_sp._7WMA2	0	0	7.40909E-08	0	0	0	0	0	0	2.85455E-08	0	3.94545E-08	0	0	0	0
Brevibacillus_sp._JNUCC-41	1.14964E-06	7.11091E-07	3.41127E-07	1.70336E-07	2.39615E-07	5.25462E-07	4.07308E-07	9.58615E-07	4.58182E-07	2.70727E-07	9.04091E-07	9.59364E-07	3.02933E-07	0.000000121	2.368E-07	4.782E-07
Brevibacillus_formosus	1.04727E-07	0.000000065	0	1.03636E-08	1.27154E-07	0	1.42385E-07	0.000000062	0	0.000000047	1.45545E-07	1.88955E-07	5.08667E-08	9.19333E-08	1.34533E-07	7.52E-08
Brevibacillus_sp._SCSIO_07484	1.07882E-06	2.47955E-07	1.0314E-06	1.36209E-06	0.000000379	7.97846E-07	2.76169E-06	3.21569E-06	4.14636E-07	5.95182E-07	7.61636E-07	1.35782E-06	2.284E-07	0.000000615	1.29433E-06	8.012E-07
Brevibacillus_brevis	1.74555E-06	8.66909E-07	6.41182E-07	0.000001748	5.53308E-07	2.54477E-06	6.93769E-07	7.85154E-07	1.14045E-06	2.50109E-06	3.99491E-06	3.11464E-06	6.69867E-07	1.51293E-06	8.56267E-07	7.23333E-07
Brevibacillus_laterosporus	0.000001229	6.79682E-07	3.53709E-07	1.81382E-06	1.72223E-06	1.14462E-07	9.09231E-07	1.44569E-06	2.72364E-07	3.25455E-07	1.05755E-06	0.000000982	1.68847E-06	1.7876E-06	2.65413E-06	3.22613E-06
Brevibacillus_agri	3.17573E-06	2.80436E-06	1.24391E-06	2.50573E-06	2.49015E-06	1.62531E-06	3.32469E-06	1.93308E-06	2.12873E-06	2.34491E-06	2.09964E-06	3.05291E-06	1.09627E-06	2.24713E-06	1.45473E-06	1.4896E-06
Paenibacillus_peoriae	0	0	0	0	3.34615E-08	0	2.53538E-07	7.99231E-08	5.79091E-08	7.55455E-08	0	1.50909E-07	1.10067E-07	1.344E-07	3.378E-07	2.49933E-07
Paenibacillus_sp._Cedars	6.23636E-08	2.34545E-08	0	5.18182E-09	3.15385E-08	0	0	0	0	1.19182E-07	6.68182E-08	8.56364E-08	0	6.60667E-08	0	0
Paenibacillus_kribbensis	1.72636E-07	3.54364E-07	0	6.46364E-08	2.75385E-08	0	3.36231E-07	1.27615E-07	1.15455E-07	5.65455E-08	6.71818E-08	2.05727E-07	1.094E-07	6.22667E-08	5.70667E-08	9.74E-08
Paenibacillus_sp._Izh-N1	0	0	2.47273E-08	0	0	0	2.53231E-07	2.84846E-07	0	0	0	1.42727E-08	1.08067E-07	1.56733E-07	1.57467E-07	1.56067E-07
Paenibacillus_sp._DCT19	3.29455E-07	3.99091E-07	5.34545E-08	2.45455E-08	1.63846E-07	7.04231E-07	7.92308E-08	6.04615E-08	2.36455E-07	1.84364E-07	3.33636E-08	5.05909E-07	1.07133E-07	7.07333E-08	5.94E-08	4.01333E-08
Paenibacillus_cellulosilyticus	5.18727E-07	2.04341E-06	1.62818E-07	4.52364E-07	1.63538E-07	4.59231E-07	4.65538E-07	1.07523E-06	5.20273E-07	3.78273E-07	3.22273E-07	7.75364E-07	2.73733E-07	3.072E-07	2.42267E-07	3.01133E-07
Paenibacillus_lautus	1.45273E-07	6.97818E-07	1.28309E-07	1.99818E-07	3.60231E-07	1.75992E-06	5.26154E-08	1.21769E-07	5.76545E-07	1.78182E-07	1.16818E-07	8.61182E-07	1.30267E-07	1.32533E-07	1.57533E-07	9.66E-08
Paenibacillus_antarcticus	1.43182E-07	1.31727E-07	2.23709E-07	1.03873E-06	2.12538E-07	4.25615E-07	9.19769E-07	0.000002437	3.41091E-07	1.95727E-07	1.19209E-06	1.59064E-06	4.304E-07	3.346E-07	8.016E-07	0.000000696
Paenibacillus_brasilensis	8.61455E-07	0.000000917	5.50936E-07	5.72455E-07	3.22462E-07	7.18615E-07	2.51077E-07	9.09692E-07	6.01182E-07	6.51273E-07	5.61182E-07	1.24982E-06	2.80133E-07	2.02467E-07	2.83267E-07	1.16267E-07
Paenibacillus_glucanolyticus	5.03545E-07	1.36318E-06	1.53273E-07	6.06909E-07	1.49385E-07	0	3.05385E-07	0.000000057	4.00455E-07	3.49636E-07	6.40273E-07	1.07164E-06	1.20133E-07	2.666E-07	2.8504E-07	3.034E-07
Paenibacillus_protaetiae	0.000001309	3.91636E-06	4.17182E-07	1.46727E-06	0.000000474	9.12769E-07	6.57769E-07	1.05946E-06	1.13064E-06	7.56091E-07	7.78364E-07	1.53736E-06	1.46533E-07	5.45133E-07	5.24533E-07	2.40333E-07
Paenibacillus_sp._37	3.22545E-07	8.44909E-07	2.634E-07	9.90909E-08	1.97692E-07	1.48969E-06	2.53308E-07	0.000000265	3.72818E-07	0.00000065	4.20091E-07	1.73145E-06	1.33667E-07	8.66E-08	2.71933E-07	1.41933E-07
Paenibacillus_sp._JZ16	9.52455E-07	2.04555E-06	1.00922E-06	1.84345E-06	3.57077E-07	1.83846E-07	6.59923E-07	0.000001507	8.75182E-07	1.15945E-06	1.07945E-06	3.50436E-06	3.98267E-07	3.876E-07	4.29333E-07	0.000000409
Paenibacillus_sp._BIHB4019	1.40909E-06	1.16036E-06	7.70764E-07	1.07482E-06	7.69308E-07	2.14838E-06	8.83846E-07	8.56692E-07	9.45091E-07	1.44582E-06	2.17855E-06	4.05682E-06	4.706E-07	8.93467E-07	8.858E-07	5.93533E-07
Paenibacillus_sp._IHB_B_3084	3.50818E-07	1.19591E-06	2.08091E-07	0.000000276	2.79769E-07	4.35462E-07	2.96615E-07	5.62923E-07	1.03727E-07	3.47455E-07	5.34818E-07	8.30091E-07	1.86667E-08	1.34467E-07	1.16107E-07	9.74E-08
Paenibacillus_sp._JNUCC-31	2.87573E-06	1.08614E-06	3.35909E-07	2.16545E-07	6.28308E-07	6.40769E-07	2.05523E-06	1.93908E-06	2.88318E-06	5.08109E-06	5.53018E-06	2.80536E-06	0.000000508	2.354E-07	6.97667E-07	1.07753E-06
Paenibacillus_sp._FSL_P4-0081	3.42545E-07	1.43773E-06	1.52991E-07	1.03891E-06	0.000000465	5.21923E-07	2.10769E-07	8.37385E-07	3.73273E-07	0.000000959	4.56818E-07	1.05582E-06	1.93067E-07	4.48333E-07	1.86067E-07	1.47933E-07
Paenibacillus_sp._JDR-2	7.69091E-07	3.16559E-06	8.24773E-07	2.28791E-06	4.64769E-07	1.25154E-06	4.25615E-07	6.01769E-07	8.31455E-07	5.50273E-07	5.80273E-07	1.77064E-06	3.93467E-07	2.968E-07	6.49267E-07	4.86133E-07
Paenibacillus_sp._JNUCC-32	1.04909E-07	1.92636E-07	8.31818E-09	2.91545E-08	2.74615E-07	7.19231E-08	2.27923E-07	4.40462E-07	2.94909E-07	2.73909E-07	2.58909E-07	7.80455E-07	4.11533E-07	0.000000293	4.084E-07	8.54533E-07
Paenibacillus_riograndensis	7.48455E-07	4.32509E-06	3.42364E-07	9.90909E-07	1.33431E-06	0.000001298	9.37154E-07	1.15185E-06	9.28455E-07	1.66727E-06	8.95091E-07	2.19591E-06	1.13567E-06	1.6956E-06	1.10267E-06	1.1876E-06
Paenibacillus_albus	4.80636E-07	2.23391E-06	2.08036E-07	1.28427E-06	4.31462E-07	9.41923E-07	5.04615E-07	1.06708E-06	6.77091E-07	3.37273E-07	0.000000485	9.08818E-07	5.096E-07	1.03467E-06	3.71067E-07	5.76267E-07
Paenibacillus_beijingensis	2.47455E-06	2.06809E-06	1.48857E-06	2.50409E-06	1.47115E-06	7.92231E-07	2.03262E-06	1.45054E-06	1.52736E-06	1.56309E-06	1.64509E-06	2.56191E-06	1.01647E-06	0.000000895	1.1294E-06	6.75533E-07
Paenibacillus_rhizovicinus	6.77091E-07	0.00000239	3.875E-07	4.57273E-07	4.56769E-07	9.26538E-07	0.000000413	2.30538E-07	3.13455E-07	5.72545E-07	6.32273E-07	1.41945E-06	2.99267E-07	3.42133E-07	0.000000653	3.918E-07
Paenibacillus_borealis	8.38273E-07	1.29032E-06	2.72436E-07	1.19145E-06	3.29538E-07	6.59692E-07	8.32385E-07	1.01785E-06	0.000000573	0.000000491	1.24282E-06	9.56636E-07	1.59467E-07	3.686E-07	6.28573E-07	1.61733E-07
Paenibacillus_xylanexedens	1.96909E-07	2.17318E-07	1.61073E-07	4.57545E-07	1.21154E-07	4.76462E-07	1.97077E-07	3.40154E-07	3.96818E-07	2.41364E-07	6.84091E-07	6.14455E-07	7.22E-08	3.008E-07	2.068E-07	0.00000014
Paenibacillus_cellulositrophicus	3.51373E-06	5.84391E-06	6.82818E-07	0.000001073	1.30754E-06	6.28923E-07	2.38646E-06	2.05415E-06	3.52091E-06	5.48127E-06	4.70491E-06	2.35718E-06	1.11507E-06	1.29047E-06	1.51977E-06	2.0516E-06
Paenibacillus_barcinonensis	4.27364E-07	9.51091E-07	4.54273E-07	1.45664E-06	1.23369E-06	3.51577E-06	8.64615E-07	0.00000052	1.18664E-06	2.56227E-06	0.000000495	0.000002596	6.342E-07	4.98667E-07	8.93533E-07	5.28133E-07
Paenibacillus_yonginensis	5.53545E-06	6.47273E-06	3.39045E-06	3.13455E-06	0.000000496	2.79654E-06	3.93192E-06	3.69408E-06	1.25927E-06	2.02364E-06	2.41464E-06	2.55555E-06	7.25733E-07	9.816E-07	1.20033E-06	4.82267E-07
Paenibacillus_guangzhouensis	4.20182E-07	0.000001302	1.48945E-07	2.95455E-07	4.92231E-07	0.00000069	5.81077E-07	0.000000673	4.03636E-07	3.07636E-07	8.61273E-07	1.77709E-06	1.84333E-07	1.30267E-07	3.78333E-07	1.85467E-07
Paenibacillus_bovis	1.00718E-06	0.000001261	2.93364E-07	4.87364E-07	6.46923E-07	5.09231E-08	5.96077E-07	7.14462E-07	1.04564E-06	5.61455E-07	8.70455E-07	1.96336E-06	4.492E-07	8.224E-07	9.47867E-07	6.52933E-07
Paenibacillus_psychroresistens	7.02182E-07	5.09273E-07	6.22818E-07	9.47091E-07	4.71846E-07	2.37923E-07	0.000001121	1.12646E-06	9.65182E-07	5.63727E-07	1.04755E-06	7.57182E-07	6.42867E-07	3.44067E-07	5.636E-07	0.000000519
Paenibacillus_ihbetae	1.12882E-06	0.000003189	2.30845E-07	9.38545E-07	5.58692E-07	6.79769E-07	8.90615E-07	9.33846E-07	1.05664E-06	1.00955E-06	1.23509E-06	1.19618E-06	4.81467E-07	7.278E-07	6.7824E-07	5.28667E-07
Paenibacillus_lutimineralis	1.03427E-06	1.52607E-05	3.03091E-07	1.45545E-06	7.12231E-07	0.000000573	8.93154E-07	0.000000912	0.00000046	5.21727E-07	6.67727E-07	1.80927E-06	5.31067E-07	6.044E-07	6.14133E-07	5.69067E-07
Paenibacillus_lentus	2.33382E-06	1.43735E-05	6.94636E-07	2.68864E-06	1.43831E-06	1.85238E-06	2.61485E-06	2.59954E-06	2.96455E-06	0.000002742	3.57464E-06	4.22091E-06	1.51933E-06	1.62073E-06	1.4502E-06	1.84213E-06
Paenibacillus_naphthalenovorans	1.43964E-06	3.68818E-07	1.00355E-06	2.41682E-06	1.14854E-06	5.01077E-07	8.19154E-07	1.15092E-06	2.01064E-06	1.57727E-06	2.47155E-06	2.26545E-06	1.23453E-06	1.03707E-06	8.79467E-07	0.000001555
Paenibacillus_jilunlii	1.29073E-06	1.94245E-06	4.06818E-07	9.86364E-07	8.31385E-07	8.46846E-07	1.49362E-06	5.91769E-07	1.16818E-06	8.77545E-07	6.37182E-07	1.16327E-06	4.34267E-07	5.68067E-07	3.53267E-07	6.21133E-07
Paenibacillus_lycopersici	2.12836E-06	0.00000279	9.86818E-07	0.000003042	1.54746E-06	1.96977E-06	0.000001165	1.03454E-06	1.94618E-06	2.22018E-06	0.000002666	3.96273E-06	8.21667E-07	1.3146E-06	1.34773E-06	1.04567E-06
Paenibacillus_odorifer	5.27636E-07	6.15955E-07	2.05636E-07	1.23455E-06	1.81685E-06	2.13592E-06	1.31308E-06	2.13631E-06	0.000000581	7.68091E-07	1.00564E-06	1.94464E-06	1.10833E-06	1.4214E-06	1.32673E-06	9.776E-07
Paenibacillus_sabinae	1.31891E-06	1.91236E-06	6.81909E-07	8.63636E-07	5.30308E-07	2.85308E-07	6.94231E-07	7.81846E-07	1.26382E-06	0.000001654	1.49045E-06	2.02245E-06	5.66467E-07	8.35867E-07	5.56667E-07	3.55467E-07
Paenibacillus_physcomitrellae	0.000001481	7.08164E-06	2.84818E-07	9.14091E-07	6.50308E-07	9.63769E-07	1.52677E-06	9.20923E-07	9.71818E-07	7.56818E-07	1.14336E-06	1.23473E-06	8.02267E-07	1.03487E-06	9.28067E-07	6.506E-07
Paenibacillus_donghaensis	6.65273E-06	7.23073E-06	8.24364E-06	3.77909E-06	2.16746E-06	0.000006415	4.21454E-06	5.24577E-06	2.66636E-06	3.40009E-06	3.57264E-06	5.51364E-06	1.59453E-06	1.81067E-06	2.58787E-06	2.0194E-06
Paenibacillus_xylanilyticus	1.39864E-06	1.82973E-06	5.06345E-07	1.03136E-06	8.58538E-07	9.88077E-07	2.48246E-06	6.31154E-07	1.64564E-06	8.46455E-07	6.35364E-07	1.60727E-06	7.216E-07	1.18447E-06	0.000000748	4.66133E-07
Paenibacillus_alvei	2.24336E-06	9.78636E-07	3.41545E-07	7.35364E-07	3.59308E-07	4.19538E-07	6.67385E-07	1.76154E-07	3.34909E-07	2.96636E-07	3.04636E-07	7.07364E-07	3.76667E-07	5.698E-07	6.94267E-07	2.69267E-07
Paenibacillus_stellifer	1.58273E-06	8.31409E-06	1.60636E-06	2.68218E-06	0.000001532	6.57992E-06	5.31338E-06	6.33377E-06	1.26227E-06	1.16773E-06	1.93173E-06	2.24936E-06	0.000002851	2.35867E-06	3.47007E-06	1.77533E-06

Paenibacillus_polymyxa	1.32873E-05	1.16015E-05	2.27695E-06	6.28727E-06	1.18882E-05	4.21608E-06	6.45292E-06	9.11538E-06	7.47464E-06	1.02373E-05	7.47818E-06	1.51427E-05	3.5258E-06	5.37993E-06	3.81067E-06	3.51413E-06
Paenibacillus_larvae	1.21964E-06	2.70445E-06	5.09591E-07	1.28618E-06	7.28692E-07	9.20692E-07	0.000001336	1.65185E-06	1.01527E-06	1.10736E-06	2.09355E-06	2.93682E-06	2.13667E-07	7.554E-07	9.66267E-07	0.000000464
Paenibacillus_terrae	1.77909E-07	7.01636E-07	2.01091E-07	6.61182E-07	1.68231E-07	5.47692E-08	4.32538E-07	3.10692E-07	4.80455E-07	4.17273E-07	1.21909E-07	2.29455E-07	1.37533E-07	4.002E-07	1.46733E-07	1.93133E-07
Paenibacillus_thiaminolyticus	2.18382E-06	2.31909E-06	3.95091E-07	1.87645E-06	1.50238E-06	1.75969E-06	2.84508E-06	1.68646E-06	0.000001797	1.85564E-06	1.29355E-06	2.41518E-06	1.01647E-06	1.3406E-06	1.02613E-06	8.132E-07
Paenibacillus_durus	4.24545E-06	4.56509E-06	1.17045E-06	4.08873E-06	2.93677E-06	0.000003082	2.25208E-06	3.31185E-06	3.90236E-06	3.25955E-06	3.34091E-06	3.61955E-06	0.000003304	4.66773E-06	3.45733E-06	3.03773E-06
Paenibacillus_baekrokdamisoli	1.54218E-06	5.32364E-07	9.45818E-08	1.88218E-06	3.02346E-06	6.69769E-07	1.29908E-06	0.000001661	4.60545E-06	3.47036E-06	3.73218E-06	2.12336E-06	2.29253E-06	1.68987E-06	2.69053E-06	1.95473E-06
Paenibacillus_mucilaginosus	6.50545E-06	0.000005378	2.07782E-06	6.97909E-06	2.18762E-06	0.00000387	6.75385E-06	8.71769E-06	3.90273E-06	4.41182E-06	6.85909E-06	8.16091E-06	1.64887E-06	2.6388E-06	2.2778E-06	3.11367E-06
Paenibacillus_crassostreae	7.03182E-07	6.15045E-07	3.95636E-07	3.73636E-07	1.08177E-06	9.64692E-07	1.03431E-06	7.50846E-07	6.37545E-07	5.41727E-07	9.37545E-07	1.02682E-06	3.71667E-07	1.10247E-06	5.648E-07	7.08667E-07
Paenibacillus_graminis	2.07364E-06	4.77173E-06	0.000000974	0.000001798	1.47223E-06	0.00000377	1.93554E-06	1.64638E-06	1.07545E-06	1.33236E-06	1.10445E-06	2.57609E-06	6.45733E-07	1.2374E-06	0.000001664	6.49933E-07
Paenibacillus_swuensis	5.17155E-06	2.91227E-06	1.33291E-06	2.76273E-06	0.000003866	3.44077E-06	4.67915E-06	1.68908E-06	9.25464E-06	4.73945E-06	8.34345E-06	0.00000542	2.81713E-06	3.5424E-06	1.9586E-06	1.79427E-06
Paenibacillus_chitinolyticus	8.55364E-06	6.48782E-06	2.10607E-06	6.44336E-06	7.24038E-06	6.17085E-06	9.95231E-06	3.46354E-06	9.45091E-06	8.44909E-06	4.26909E-06	7.16064E-06	4.12327E-06	6.09133E-06	2.92573E-06	2.99067E-06
Paenibacillus_sp._Y412MC10	4.39727E-07	3.90909E-07	1.59636E-07	4.07273E-07	1.13538E-07	5.63077E-07	5.74615E-08	3.33615E-07	1.42355E-06	2.85455E-08	3.54818E-07	3.24091E-07	4.38E-08	1.70867E-07	1.66973E-07	0.000000302
Paenibacillus_sp._FSL_R7-0273	1.81664E-06	1.55614E-06	7.17436E-07	1.23336E-06	6.41308E-07	9.61462E-07	0.000000853	9.12692E-07	7.48909E-07	1.04336E-06	1.00527E-06	1.27673E-06	5.99467E-07	8.42733E-07	0.00000085	9.614E-07
Paenibacillus_sp._IHBB_10380	3.16909E-07	8.89273E-07	4.90909E-08	1.68727E-07	3.99846E-07	9.72308E-08	2.59615E-07	0.000000179	3.98091E-07	3.30364E-07	5.50273E-07	2.62091E-07	3.12333E-07	3.36067E-07	1.94733E-07	2.11933E-07
Paenibacillus_sp._URB8-2	1.34282E-06	1.95173E-06	1.82591E-07	1.76973E-06	8.11231E-07	3.83077E-07	7.32846E-07	1.02338E-06	7.57727E-07	7.28818E-07	0.000000935	1.21555E-06	8.26067E-07	1.20207E-06	9.81933E-07	1.19227E-06
Paenibacillus_sp._32O-W	1.22209E-06	2.93855E-06	5.28182E-07	7.32364E-07	4.66385E-07	0.000000216	6.25077E-07	6.16231E-07	9.63545E-07	0.000000722	7.13545E-07	1.63373E-06	3.802E-07	7.336E-07	4.76933E-07	2.624E-07
Paenibacillus_sp._FSL_R5-0912	6.21364E-07	1.13882E-06	1.62945E-07	4.37727E-07	0.000000077	1.48923E-06	4.43846E-07	3.33231E-07	2.80273E-07	2.12545E-07	5.29636E-07	6.94727E-07	1.19067E-07	5.434E-07	3.86133E-07	2.24533E-07
Paenibacillus_sp._E222	1.78936E-06	0.00000404	2.43718E-06	2.29591E-06	7.25692E-07	9.09385E-07	6.26923E-07	8.94538E-07	7.89818E-07	3.35818E-07	7.04727E-07	1.48682E-06	7.886E-07	8.414E-07	1.04927E-06	8.42267E-07
Paenibacillus_sp._UniB2	3.49918E-06	1.95873E-06	2.44918E-06	3.35409E-06	9.46769E-07	8.66615E-07	2.57331E-06	2.37108E-06	1.97782E-06	2.32618E-06	2.54909E-06	0.00000302	3.89267E-07	9.22133E-07	1.00133E-06	9.27733E-07
Paenibacillus_sp._B01	1.00864E-06	3.40109E-06	7.81818E-07	9.97273E-07	3.91615E-07	1.70677E-06	1.20215E-06	9.64462E-07	9.19182E-07	1.44209E-06	0.000001582	2.73045E-06	5.44133E-07	7.376E-07	6.83667E-07	8.584E-07
Paenibacillus_sp._HB172198	1.25473E-06	0.000004091	7.50818E-07	1.98745E-06	4.27615E-07	7.27231E-07	1.69538E-06	1.88815E-06	0.000001588	7.18727E-07	4.09364E-07	1.83109E-06	1.18827E-06	1.25547E-06	8.72733E-07	1.13033E-06
Paenibacillus_sp._FSL_H7-0737	6.22455E-07	9.27727E-07	1.69436E-07	1.08882E-06	9.18615E-07	6.66385E-07	1.41346E-06	6.45231E-07	5.80455E-07	7.86455E-07	0.000001075	1.05291E-06	3.76533E-07	9.68867E-07	3.76133E-07	4.51267E-07
Paenibacillus_sp._CAA11	1.10536E-06	9.31655E-06	2.58273E-07	1.10518E-06	1.40185E-06	2.22692E-06	8.00308E-07	1.51446E-06	2.61336E-06	1.65573E-06	1.94691E-06	2.29618E-06	1.45893E-06	1.26273E-06	1.4736E-06	1.35373E-06
Paenibacillus_sp._PAMC21692	1.72927E-06	1.50218E-06	6.49491E-07	2.14009E-06	3.87446E-06	1.35331E-06	2.00615E-06	3.86615E-06	1.63491E-06	1.70145E-06	3.56855E-06	3.41136E-06	0.000002449	7.2182E-06	0.00000401	2.39233E-06
Paenibacillus_sp._FSL_R7-0331	0.000001799	1.34345E-06	4.14727E-07	1.38536E-06	2.06277E-06	2.28785E-06	1.46762E-06	9.55154E-07	0.000002286	2.14745E-06	2.11773E-06	2.89318E-06	3.36307E-06	2.87133E-06	2.9292E-06	2.9034E-06
Paenibacillus_sp._RUD330	4.46355E-06	2.37536E-06	1.06295E-05	0.000011687	3.04646E-06	2.08231E-06	2.54788E-05	2.59615E-05	5.72736E-06	6.48055E-06	3.97818E-06	4.76855E-06	3.14013E-06	3.3644E-06	6.04793E-06	2.55953E-06
Paenibacillus_sp._FSL_R5-0345	3.79318E-06	5.52182E-07	5.84273E-07	8.09909E-07	3.03831E-06	6.29846E-07	3.69115E-06	3.31138E-06	3.67009E-06	6.56336E-06	4.53145E-06	2.45573E-06	2.31093E-06	2.53687E-06	2.2224E-06	2.8386E-06
Paenibacillus_sp._FSL_H7-0357	4.52909E-06	2.61627E-06	1.50464E-06	1.67845E-06	8.28231E-06	2.46277E-06	2.44177E-06	2.51669E-06	2.20445E-06	2.75255E-06	2.79364E-06	3.99536E-06	5.60567E-06	4.98933E-06	4.9058E-06	4.2994E-06
Pontibacillus_sp._HMF3514	1.87636E-07	0.000000065	1.17182E-07	1.05636E-07	5.48538E-07	8.88462E-08	1.09923E-07	1.45769E-07	4.63091E-07	8.62727E-08	1.34182E-07	4.69182E-07	2.29733E-07	5.16067E-07	2.88333E-07	2.95533E-07
Terribacillus_goriensis	1.40727E-07	8.48273E-07	1.04036E-07	6.65455E-08	1.08538E-07	1.79231E-08	9.47692E-08	0.000000119	5.79091E-08	1.01091E-07	2.67818E-07	7.59909E-07	2.768E-07	9.44E-08	1.71667E-07	1.00133E-07
Aeribacillus_pallidus	1.95727E-07	1.66182E-07	3.37636E-08	4.85818E-07	1.38846E-07	8.20769E-08	1.89615E-07	3.08769E-07	3.35455E-07	0.000000196	4.08273E-07	2.21273E-07	5.04E-08	3.07667E-07	8.98E-08	1.558E-07
Fictibacillus_arsenicus	1.17664E-06	4.59091E-07	2.16309E-07	6.86818E-07	2.61308E-07	4.19077E-07	0.000000562	5.72385E-07	1.12682E-06	0.000000709	8.07818E-07	7.20818E-07	4.726E-07	5.39267E-07	4.46667E-07	7.928E-07
Fictibacillus_phosphorivorans	2.75273E-07	3.52364E-07	1.53545E-07	3.59455E-07	1.85846E-07	4.76923E-08	3.11538E-08	2.93846E-08	4.68273E-07	6.12727E-07	3.01364E-07	1.99818E-07	1.12533E-07	5.68E-08	1.402E-07	2.34467E-07
Parageobacillus_toebii	6.33636E-08	3.89545E-07	8.30909E-08	2.02909E-07	0	2.95385E-08	0	1.80923E-07	2.75455E-08	8.85455E-08	1.35636E-07	2.49818E-07	0	0	0	0.000000168
Parageobacillus_genomosp._1	7.15455E-08	4.39091E-08	3.70909E-08	1.75455E-07	5.02308E-08	4.52462E-07	1.59385E-07	1.05308E-07	2.22455E-07	0	0.000000255	1.42727E-08	2.93333E-07	1.12467E-07	3.86E-08	1.49667E-07
Parageobacillus_thermoglucosidasius	0	0	0.000000025	0	7.03846E-08	1.23846E-07	0	1.46923E-08	0	0	0	0	2.24E-08	4.49333E-08	4.46667E-08	5.34E-08
Paraliobacillus_sp._X-1125	2.82818E-07	1.37273E-08	3.70909E-08	2.22182E-07	1.42231E-07	0	4.26769E-07	4.75615E-07	2.22218E-06	3.79636E-07	5.73273E-07	3.28455E-07	2.84133E-07	9.06467E-07	5.96133E-07	2.84467E-07
Anaerobacillus_isosaccharinicus	4.13545E-07	1.01582E-06	4.69182E-07	4.59909E-07	5.55462E-07	1.76615E-07	1.15977E-06	0.000000786	1.83827E-06	4.09091E-07	5.61727E-07	7.11182E-07	5.20133E-07	0.000000534	1.4048E-06	1.0444E-06
Cytobacillus_oceanisediminis	8.20364E-07	2.83409E-06	0.00000074	1.26655E-06	8.52846E-07	1.52323E-06	9.97923E-07	7.73923E-07	1.36655E-06	2.07536E-06	1.54091E-06	1.21236E-06	1.23067E-07	7.50133E-07	7.216E-07	0.000000885
Cytobacillus_kochii	3.03636E-07	9.20909E-08	9.96364E-08	1.66091E-07	1.39231E-07	0.00000025	4.04462E-07	5.59308E-07	1.42718E-06	2.26818E-07	0.000000701	4.00545E-07	1.266E-07	3.77333E-07	2.65707E-07	3.522E-07
Pradoshia_sp._D12	5.27909E-07	0.000000021	4.26591E-07	2.09545E-07	0.00000021	0.000000081	6.01846E-07	3.13308E-07	9.95364E-07	4.97545E-07	9.74455E-07	7.13636E-07	2.224E-07	5.65533E-07	1.394E-07	3.43067E-07
Lentibacillus_sp._CBA3610	1.69455E-07	0	0	2.35091E-07	4.27385E-07	1.89231E-08	6.06923E-08	4.26692E-07	5.06182E-07	7.58636E-07	3.06909E-07	4.64364E-07	4.016E-07	2.62133E-07	3.33933E-07	3.34867E-07
Lentibacillus_amyloliquefaciens	5.75727E-07	8.73182E-08	1.13264E-07	4.16364E-07	5.54923E-07	1.33738E-06	1.55923E-07	2.94769E-07	1.58382E-06	3.30273E-07	9.35455E-07	7.55545E-07	2.93867E-07	4.902E-07	4.96267E-07	0.000000548
Mesobacillus_jeotgali	1.52445E-06	0.00000013	5.64491E-07	6.01091E-07	3.08462E-07	1.40615E-07	7.63231E-07	5.57846E-07	9.64091E-07	6.02818E-07	1.18973E-06	1.03236E-06	2.03933E-07	1.99867E-07	3.112E-07	3.59667E-07
Mesobacillus_foraminis	1.25382E-06	2.57545E-07	2.32727E-07	9.87182E-07	1.01646E-06	0.000000143	9.15692E-07	0.00000094	1.04709E-06	1.42691E-06	9.52909E-07	2.53455E-06	1.44187E-06	1.4132E-06	1.63567E-06	9.97133E-07
Salicibibacter_halophilus	0.000000212	1.32909E-07	8.00909E-08	7.98364E-07	1.94615E-07	2.15923E-06	2.14231E-07	3.22308E-08	1.31545E-07	2.08364E-07	4.82273E-07	4.68273E-07	1.35867E-07	3.294E-07	3.39773E-07	2.52267E-07
Salicibibacter_kimchii	4.67818E-07	3.89455E-07	6.57091E-08	7.28364E-07	3.32385E-07	2.28615E-07	1.03692E-06	1.60308E-07	4.35455E-07	2.56818E-07	1.93182E-07	7.16182E-07	2.22067E-07	2.652E-07	1.76667E-07	1.484E-07
Amphibacillus_xylanus	0.000000455	2.81818E-07	1.45636E-07	4.82818E-07	5.84231E-07	6.93846E-08	4.78615E-07	1.61392E-06	1.93055E-06	3.39455E-07	8.29727E-07	1.61036E-06	2.67867E-07	9.82133E-07	6.44933E-07	6.29867E-07
Gracilibacillus_sp._SCU50	3.05809E-06	0.000000181	5.63091E-07	3.58455E-07	1.27315E-06	6.53077E-08	1.45777E-06	2.40692E-06	4.48782E-06	4.27427E-06	6.13027E-06	3.19164E-06	1.18293E-06	1.80213E-06	2.09447E-06	2.41707E-06
Salimicrobium_jeotgali	2.55164E-06	1.99555E-06	1.44009E-06	1.89055E-06	9.87615E-07	5.44231E-07	1.71177E-06	2.14292E-06	1.13127E-06	1.32318E-06	2.19709E-06	1.96773E-06	1.77453E-06	2.38007E-06	2.34773E-06	2.64927E-06
Radiobacillus_deserti	1.23273E-06	0.000000245	2.828E-07	0.000001386	2.99846E-07	2.71154E-07	0.00000059	8.01077E-07	1.29664E-06	4.92636E-07	7.61364E-07	1.19355E-06	5.29733E-07	9.48533E-07	1.1548E-06	1.32133E-06
Peribacillus_butanolivorans	1.05545E-07	1.25636E-07	6.76182E-08	2.04909E-07	1.57462E-07	0	1.28538E-07	6.26923E-08	5.92636E-07	2.25182E-07	4.37909E-07	5.34091E-07	2.31333E-07	4.71067E-07	2.98867E-07	9.56E-08
Peribacillus_simplex	1.39636E-07	0.000000065	1.63636E-08	9.79091E-08	3.58462E-08	4.39077E-07	1.04385E-07	4.12308E-07	2.62727E-07	7.25455E-08	1.61364E-07	7.85455E-08	1.72667E-07	1.252E-07	9.51333E-08	6.80467E-07
Peribacillus_muralis	4.71364E-07	4.84455E-07	1.04709E-07	3.65727E-07	2.57846E-07	4.72077E-07	3.06462E-07	3.23385E-07	2.49455E-07	2.83636E-07	2.81545E-07	5.57091E-07	0.000000136	2.28267E-07	1.43133E-07	0.00000018
Peribacillus_asahii	1.24755E-06	3.10364E-07	5.43273E-07	1.17055E-06	2.36046E-06	4.96846E-07	1.46469E-06	0.000001228	2.42355E-06	6.69273E-07	1.93282E-06	4.29491E-06	9.15333E-07	1.15387E-06	1.3956E-06	1.5376E-06
Halobacillus_mangrovi	8.19091E-08	2.34545E-08	7.49818E-08	0.000000135	7.26923E-08	5.05385E-08	6.51538E-08	2.56923E-07	9.54545E-08	2.12818E-07	3.06273E-07	1.17273E-07	1.60133E-07	1.182E-07	9.764E-08	3.15933E-07
Halobacillus_halophilus	2.27118E-06	1.84364E-07	2.38273E-07	2.38091E-07	8.53077E-07	0.000000843	1.68423E-06	0.000001245	2.28364E-06	3.31645E-06	2.79645E-06	1.00414E-06	0.000001046	1.19473E-06	5.10907E-07	0.000000825
Halobacillus_litoralis	0.00000008	1.39091E-07	0	1.95455E-08	2.56962E-06	8.82385E-07	1.74846E-07	7.69923E-07	7.39182E-07	3.84364E-07	0.000000162	5.97273E-07	1.37327E-06	1.65173E-06	1.88913E-06	1.64833E-06
Metabacillus_sp._KUDC1714	9.54273E-07	3.52818E-07	1.81764E-07	7.90182E-07	7.36769E-07	6.76923E-08	7.10154E-07	7.47462E-07	0.000001581	3.78909E-07	1.03564E-06	1.67464E-06	4.08933E-07	4.69067E-07	8.64733E-07	7.132E-07
Metabacillus_litoralis	2.94818E-06	4.05727E-07	2.26545E-07	6.16273E-07	3.31385E-07	2.61538E-07	2.04308E-06	1.93915E-06	5.67291E-06	3.81073E-06	3.09045E-06	1.73418E-06	6.32533E-07	6.99267E-07	1.16587E-06	1.65647E-06
Psychrobacillus_sp._AK_1817	5.27273E-08	8.28182E-08	2.04909E-08	2.43636E-08	0	0	1.07846E-07	0	0.00000008	0	3.95455E-08	2.14545E-08	4.10667E-08	0	2.99333E-08	3.84667E-08
Psychrobacillus_glaciei	1.78327E-06	2.03682E-07	5.49818E-07	1.24355E-06	1.42477E-06	3.38923E-07	1.56762E-06	2.34662E-06	3.18491E-06	4.12336E-06	3.57664E-06	4.58364E-06	2.15227E-06	1.83973E-06	2.72247E-06	4.64773E-06
Oceanobacillus_kimchii	8.24545E-08	0	7.40727E-08	2.22727E-07	1.69308E-07	0	2.94615E-07	0.000000355	1.94818E-06	2.43182E-07	3.08455E-07	2.13909E-07	1.06467E-07	3.05133E-07	5.112E-07	3.27733E-07
Oceanobacillus_iheyensis	4.87636E-07	5.75455E-08	3.65409E-07	2.35273E-07	5.85846E-07	1.24308E-06	7.80769E-07	1.32246E-06	1.25809E-06	7.43909E-07	1.51691E-06	1.37355E-06	5.35533E-07	7.464E-07	7.16067E-07	8.49733E-07
Oceanobacillus_sp._160	0.000002289	7.53636E-08	1.69909E-07	1.11682E-06	1.64031E-06	5.76769E-07	1.40431E-06	2.57423E-06	1.95327E-06	3.29264E-06	2.47082E-06	1.28373E-06	2.1556E-06	1.37993E-06	2.67633E-06	4.08173E-06
Neobacillus_thermocopriae	4.56364E-08	0.000000065	1.93455E-07	1.35818E-07	2.05385E-08	7.21538E-08	1.58462E-07	4.24231E-07	2.16364E-08	1.68818E-07	6.55455E-08	7.84545E-08	1.35067E-07	0	4.884E-08	1.216E-07
Neobacillus_mesonae	3.17182E-06	4.24182E-07	1.34573E-06	2.11809E-06	5.01462E-06	0.000000536	1.11046E-06	0.000003182	2.08355E-06	2.23855E-06	1.49718E-06	3.84564E-06	3.16267E-06	4.50593E-06	5.0906E-06	5.55507E-06
Geobacillus_kaustophilus	5.92727E-08	2.34545E-08	0	0	1.86923E-08	8.30769E-08	0	0	0	0	0	0	0	0.000000014	0	0
Geobacillus_sp._GHH01	0	3.23182E-08	0	0	8.13077E-08	0.000000185	0	0	0	0	0	3.16364E-08	0.000000027	0	2.24667E-08	1.22E-08
Geobacillus_sp._Y4.1MC1	0.000000061	2.98545E-07	8.61818E-08	1.32182E-07	3.58462E-08	9.46154E-09	1.14923E-07	1.11615E-07	5.97273E-08	1.14455E-07	1.97727E-07	2.29818E-07	2.64667E-08	0	0	0
Geobacillus_sp._E263	2.56364E-08	1.68182E-08	0	2.95455E-08	0	0	0	0	0	0	1.99727E-07	8.73636E-08	0	0	0	0
Geobacillus_thermoleovorans	0	0	0	4.20909E-08	2.88462E-08	9.06923E-08	1.12538E-07	5.13846E-08	1.73636E-07	0	1.47545E-07	4.28182E-08	2.08E-08	3.29333E-08	1.33333E-08	0.000000059
[Bacillus]_caldolyticus	0	0	0	3.99091E-08	0	2.13846E-08	0	0	0	0	1.02727E-08	0	0	0	0	0
Geobacillus_sp._JS12	0	0	0	0	0	2.95385E-08	0	0	0	0	1.02727E-08	0	0	0	0	0
Geobacillus_sp._44C	5.57273E-08	1.16364E-08	0	0.00000012	1.86923E-08	0	0	0	1.62091E-07	1.44818E-07	9.64545E-08	9.64545E-08	0	7.60667E-08	5.98667E-08	0
Geobacillus_sp._WCH70	0	0	0	0	3.34615E-08	2.32846E-07	3.33077E-08	0	0	0	2.76364E-08	7.55455E-08	5.34E-08	0.000000067	1.49333E-08	6.33333E-08
Geobacillus_zalihae	0	9.54545E-09	3.70909E-08	0	0	0	0	0	0	0	1.07818E-07	0	0	0	0	0
Geobacillus_sp._46C-IIa	1.58545E-07	7.54545E-08	1.33091E-07	2.25609E-07	2.66308E-07	0.000000804	7.95615E-07	9.73077E-08	8.20909E-08	1.11273E-07	2.75273E-07	4.48727E-07	2.56333E-07	1.638E-07	1.898E-07	1.392E-07
Geobacillus_thermocatenulatus	0	2.63636E-08	4.12727E-09	0	3.15385E-08	2.11308E-07	0	2.47692E-08	0	0	9.99091E-08	7.13636E-09	2.44E-08	0	0	4.95333E-08
Geobacillus_sp._44B	7.33273E-07	6.51727E-07	2.39982E-07	2.82818E-07	1.14462E-07	0	6.44615E-08	2.68538E-07	4.28455E-07	2.22182E-07	0.000000371	0.000000347	1.01133E-07	3.646E-07	1.55133E-07	2.60467E-07
Geobacillus_genomosp._3	1.96636E-07	0	0	9.44545E-08	1.83923E-07	8.90769E-08	0	8.50154E-07	1.02455E-07	1.35455E-07	1.35636E-07	0	6.15333E-08	2.29067E-07	7.04867E-07	4.542E-07
Geobacillus_subterraneus	6.59455E-07	6.12091E-07	3.98336E-07	1.72173E-06	7.32077E-07	7.52538E-07	3.70077E-07	7.40846E-07	1.52036E-06	1.17709E-06	1.76782E-06	2.68973E-06	0.000000782	1.44027E-06	1.1544E-06	0.000000732
Geobacillus_thermodenitrificans	1.30545E-07	0	0	2.89455E-07	2.17154E-07	0	0.000000207	0.000000327	9.04909E-07	3.12727E-07	4.93273E-07	5.37682E-07	2.806E-07	0.000000248	6.94267E-07	2.682E-07
Geobacillus_stearothermophilus	1.94236E-06	9.21455E-07	7.32164E-07	1.54464E-06	3.33154E-07	1.92669E-06	5.45538E-07	1.42662E-06	8.08909E-07	1.02955E-06	1.27064E-06	1.47591E-06	1.33833E-06	1.21313E-06	7.12933E-07	0.00000141
Alkalihalobacillus_halodurans	1.31273E-06	2.43109E-06	3.19909E-07	6.34636E-07	1.41254E-06	1.46154E-07	1.73138E-06	6.35154E-07	1.54545E-06	3.99364E-07	2.51764E-06	1.35591E-06	0.00000016	1.14753E-06	1.93833E-06	1.85407E-06
Alkalihalobacillus_lehensis	3.53909E-07	0	9.98909E-08	2.52727E-07	3.46615E-07	0	3.89231E-07	5.05154E-07	1.24727E-07	1.74909E-07	4.04455E-07	3.00818E-07	1.06133E-07	1.75067E-07	2.20707E-07	2.29867E-07
Alkalihalobacillus_clausii	7.55909E-07	3.61455E-07	0.000000289	1.29891E-06	7.20846E-07	1.01669E-06	1.49531E-06	1.54585E-06	1.80936E-06	6.74455E-07	1.51891E-06	1.77018E-06	3.05733E-07	7.97867E-07	1.05007E-06	9.144E-07
Alkalihalobacillus_krulwichiae	9.76636E-07	2.70455E-07	1.50727E-07	1.18145E-06	7.19615E-07	4.77308E-07	3.54692E-07	6.37538E-07	1.19291E-06	9.68182E-07	6.79364E-07	6.00091E-07	7.54333E-07	9.87867E-07	9.43933E-07	9.522E-07
Alkalihalobacillus_pseudofirmus	2.04091E-07	0	6.67273E-08	0.000000758	1.01792E-06	7.89231E-07	3.74077E-07	7.63923E-07	3.02909E-07	2.66727E-07	5.68182E-07	6.35091E-07	1.13547E-06	8.026E-07	1.53853E-06	2.26567E-06
Anoxybacillus_sp._PDR2	0	6.61636E-07	7.66364E-08	2.03273E-07	0	0	0	0	7.97273E-08	0	7.90909E-08	3.94545E-08	0	0	0	0
Anoxybacillus_kamchatkensis	1.65818E-07	1.37273E-08	4.12727E-09	5.18182E-09	0	0	7.54615E-08	0	0.000000077	0.000000116	0	0	1.82667E-07	2.06267E-07	2.99333E-08	1.21333E-07
Anoxybacillus_amylolyticus	3.29364E-07	5.41818E-08	7.53727E-08	2.94364E-07	2.30538E-07	3.66154E-07	1.86615E-07	4.59538E-07	5.19727E-07	2.47818E-07	3.42364E-07	5.43545E-07	2.91333E-08	1.194E-07	1.19067E-07	2.74333E-07
Anoxybacillus_flavithermus	2.99545E-07	1.71182E-07	0	0.000000058	0.000000136	3.37538E-07	3.42538E-07	2.49692E-07	4.63636E-07	3.08455E-07	4.45182E-07	0.000000235	1.15933E-07	2.006E-07	1.10133E-07	1.51533E-07
Anoxybacillus_caldiproteolyticus	4.12364E-07	9.75455E-08	1.04945E-07	1.28518E-07	7.59615E-07	9.42923E-07	8.26692E-07	8.21769E-07	5.69909E-07	4.17364E-07	2.24091E-07	3.99727E-07	1.91533E-07	2.81667E-07	6.28733E-07	0.000000561
Anoxybacillus_gonensis	1.36091E-07	4.40364E-07	0.000000185	8.12182E-07	2.87692E-07	0	5.10538E-07	6.38692E-07	2.44636E-07	3.36364E-07	2.97455E-07	3.23636E-07	4.86E-08	5.416E-07	3.604E-07	2.73867E-07
Virgibacillus_sp._MSP4-1	7.45364E-07	2.07273E-07	1.00455E-07	4.98273E-07	3.34615E-08	2.95385E-08	3.30231E-07	2.83231E-07	2.63455E-07	1.17273E-07	2.41091E-07	4.17455E-07	2.91333E-08	8.36667E-08	1.562E-07	5.83333E-08
Virgibacillus_dokdonensis	2.14545E-07	0	9.16727E-08	1.94545E-07	1.47615E-07	1.46154E-07	1.05692E-07	2.03385E-07	0.000000736	2.76727E-07	1.26727E-07	1.28636E-07	1.97467E-07	3.50667E-08	2.97333E-08	7.82667E-08
Virgibacillus_halodenitrificans	7.99636E-07	2.83818E-07	2.484E-07	2.79636E-07	4.36769E-07	0	1.16615E-07	0.000000111	7.67727E-07	2.54909E-07	1.41727E-07	2.46636E-07	2.636E-07	0.000000482	5.29667E-07	3.568E-07
Virgibacillus_phasianinus	3.66091E-07	8.32091E-07	2.59455E-07	1.31427E-06	5.30923E-07	3.70462E-07	1.37815E-06	2.01246E-06	8.53455E-07	2.55273E-07	1.67927E-06	1.80827E-06	2.59733E-07	8.094E-07	1.16647E-06	0.000000801
Virgibacillus_sp._Bac332	1.18527E-06	2.08273E-07	5.92036E-07	1.83518E-07	1.85769E-07	0	3.79462E-07	6.66923E-07	2.22236E-06	0.000000204	9.95091E-07	6.48455E-07	1.112E-07	1.908E-07	5.53533E-07	2.69267E-07
Virgibacillus_necropolis	0.000000243	7.87273E-08	1.07709E-07	2.11182E-07	2.59231E-08	0	5.06692E-07	4.89692E-07	8.54364E-07	0.000000198	6.40182E-07	3.35091E-07	9.30667E-08	3.48E-08	1.934E-07	2.67733E-07
Virgibacillus_sp._Bac330	4.27818E-07	0.00000039	3.01727E-08	6.35091E-07	2.93462E-07	2.06923E-07	0.000000448	1.72308E-07	6.47818E-07	2.80182E-07	6.90455E-07	9.59091E-08	2.614E-07	4.06133E-07	7.46E-08	2.32867E-07
Virgibacillus_sp._6R	5.75545E-07	8.84545E-08	2.33273E-07	6.50818E-07	4.60615E-07	4.94615E-08	7.26385E-07	5.99538E-07	5.44545E-06	0.000000384	7.42545E-07	5.66545E-07	6.80667E-07	1.44127E-06	1.08833E-06	1.05753E-06
Virgibacillus_sp._SK37	9.41182E-07	6.73273E-07	9.31273E-08	9.72818E-07	1.62238E-06	0	7.12308E-07	7.04231E-07	2.22273E-07	2.84364E-07	6.65636E-07	3.13909E-07	1.39687E-06	1.70527E-06	2.68093E-06	2.4688E-06
Lysinibacillus_varians	0	0	0	0	1.48923E-07	0	0	1.10385E-07	5.97273E-08	0	0	9.98182E-08	6.73333E-08	0.000000186	4.48667E-08	2.41333E-07
Lysinibacillus_sp._2017	8.09091E-08	0	1.29982E-07	7.38182E-08	1.29769E-07	3.33154E-07	3.08923E-07	9.07308E-07	0.000001584	1.49364E-07	9.51727E-07	5.53182E-07	7.24E-08	3.21267E-07	8.58533E-07	5.04133E-07
Lysinibacillus_sp._YS11	2.07455E-07	2.72727E-08	8.25455E-09	6.13636E-08	0.000000469	0	2.45077E-07	2.57846E-07	2.98091E-07	1.09545E-07	7.68182E-08	1.96318E-07	1.54333E-07	1.26533E-07	2.1624E-07	0.000000143
Lysinibacillus_fusiformis	2.39364E-06	4.16364E-08	1.77455E-07	5.50636E-08	4.88923E-07	4.17538E-07	1.22123E-06	2.06638E-06	2.94645E-06	3.41918E-06	3.40291E-06	2.16727E-06	5.22867E-07	4.37133E-07	2.98933E-07	9.992E-07
Lysinibacillus_timonensis	1.77727E-07	0.00000013	3.00527E-07	2.21182E-07	3.30692E-07	1.04231E-07	3.06846E-07	2.36308E-07	2.77818E-07	2.30455E-07	6.92727E-07	8.22545E-07	1.96467E-07	1.50267E-07	4.464E-07	1.16533E-07
Lysinibacillus_sphaericus	9.87273E-07	3.24545E-07	4.42818E-07	3.26427E-07	5.39769E-07	0	2.72615E-07	3.83077E-07	6.57531E-05	1.95727E-07	2.77636E-07	5.83636E-07	4.12733E-07	6.79467E-07	8.034E-07	3.74533E-07
Lysinibacillus_sp._SGAir0095	1.24527E-06	1.87364E-07	5.63709E-07	3.24727E-07	1.24523E-06	0	2.53308E-07	9.15462E-07	6.96273E-07	9.82727E-08	6.79182E-07	9.26818E-07	1.23387E-06	1.36053E-06	1.71047E-06	1.29527E-06
Bacillus_aryabhattai	0	0	0	0	0	0	0	3.22308E-08	0	5.22727E-08	0	0	0	0	0	0
Bacillus_sp._24	0	0	0	0	0	0	0	1.90769E-08	0	0	0	0	0	0	0	0
Bacillus_sp._IHB_B_7164	0	0	0	9.54545E-09	0	0	0	0	0	0	0	0	0	0	0	0
Bacillus_sp._ABP14	0	1.04545E-08	0	1.93636E-08	0	0	0	3.57692E-08	0	0	0	0.000000057	0	0	0	3.67333E-08
Bacillus_sp._Lzh-5	0	2.34545E-08	0	4.40909E-08	0	5.09231E-08	0	3.22308E-08	0	0	0	0	0	1.61333E-08	0	0
Bacillus_paranthracis	0	0.000000033	0	0.00000007	6.46923E-08	1.95692E-07	0	0	3.98182E-08	0	0	0	0	0	0	2.62733E-07
Bacillus_sp._Cs-700	0	9.44545E-08	0	1.30545E-07	0	1.23846E-07	3.33077E-08	3.37692E-08	1.55273E-07	0	0	2.14545E-08	5.04E-08	0	1.12667E-08	0
Bacillus_sp._SYJ	0	0	0	6.62455E-08	5.50769E-08	0	0	0	0	0	0	0.000000035	0	0	1.12667E-08	6.81333E-08
Bacillus_sp._PAMC28748	0	0	0	0	0	0	2.63077E-08	0	0	0	0	0	2.64667E-08	0	0	1.86E-08
Bacillus_sp._MD-5	0	0	0	0	0	0	7.92308E-08	2.47692E-08	0	0	0	0	0	0	4.48667E-08	3.97333E-08
Bacillus_sp._N3536	0	1.00909E-07	2.96364E-08	2.39727E-08	0	1.18462E-07	0	0	5.79091E-08	0	0	7.13636E-09	2.14667E-08	0.000000041	0	0
Bacillus_sp._WP8	4.53636E-07	5.52727E-07	1.43909E-07	5.24455E-07	0	0	3.11538E-08	1.70846E-07	0.000000595	5.01636E-07	0.000000258	4.93636E-07	1.22067E-07	1.58E-08	4.44E-09	1.514E-07
Bacillus_albus	3.62727E-08	0	0	4.79091E-09	7.81538E-08	0	0	0	0	0	3.33636E-08	0	0	0	0	0
Bacillus_mobilis	1.92545E-07	4.41818E-07	2.03545E-07	7.42182E-07	7.16923E-08	0	2.70769E-07	2.13615E-07	0.00000014	0.000000056	3.41455E-07	3.27909E-07	0	0.000000317	3.69707E-07	1.90467E-07
Bacillus_paralicheniformis	4.66273E-07	3.84227E-07	1.07818E-07	6.69364E-07	4.25846E-07	1.41385E-06	5.84385E-07	4.47769E-07	8.11364E-07	4.05091E-07	5.96818E-07	1.19555E-06	2.898E-07	1.69133E-07	2.95267E-07	1.28867E-07
Bacillus_sp._1NLA3E	1.84818E-07	8.26364E-08	3.27273E-08	4.43364E-07	1.32077E-07	1.06546E-06	4.46077E-07	1.85308E-07	3.91091E-07	2.85636E-07	2.70455E-07	4.84818E-07	3.57067E-07	2.04533E-07	5.536E-07	2.96933E-07
Bacillus_haynesii	0	0	1.77273E-08	3.59091E-08	0	0	0	1.90769E-08	1.15455E-07	0.000000047	0	9.79091E-08	0	6.39333E-08	2.99333E-08	4.95333E-08
[Brevibacterium]_frigoritolerans	4.90091E-07	1.39545E-07	4.12727E-09	1.44245E-07	4.35923E-07	0	3.88692E-07	4.93615E-07	1.15455E-07	0	0	3.10091E-07	8.22E-08	0.000000104	0	0
Bacillus_gobiensis	2.52364E-07	3.26364E-07	1.49673E-07	1.79545E-07	1.78923E-07	4.94615E-08	2.63077E-08	9.39231E-08	2.10909E-07	2.36273E-07	4.48545E-07	3.15273E-07	9.24667E-08	9.22E-08	1.5304E-07	0
Bacillus_thermoamylovorans	4.40636E-07	2.39182E-07	4.214E-07	6.94182E-07	5.89769E-07	2.50769E-07	5.97538E-07	1.53923E-06	1.59036E-06	0.000000592	0.000000946	9.51636E-07	8.07667E-07	8.002E-07	8.37333E-07	7.104E-07
Bacillus_lentus	3.01636E-07	6.93455E-07	9.18182E-08	4.49818E-07	3.09462E-07	2.34538E-07	3.35462E-07	3.03385E-07	1.15392E-05	2.71909E-07	0.000000976	8.59818E-07	3.026E-07	0.00000052	2.732E-07	5.04933E-07
Bacillus_safensis	4.61618E-06	1.56636E-07	0.000000355	0.000000176	0.000001201	9.02769E-07	4.18508E-06	3.19285E-06	4.81345E-06	5.89909E-06	5.95182E-06	4.11545E-06	9.992E-07	5.06133E-07	8.09933E-07	2.60527E-06
Bacillus_vallismortis	3.21636E-07	0.000000065	4.12182E-08	1.55818E-07	1.06231E-07	3.11538E-08	7.92308E-08	1.50615E-07	1.12727E-07	2.24455E-07	2.76364E-08	2.98091E-07	5.09333E-08	0.000000033	7.82667E-08	0
Bacillus_sp._BS98	0	0	4.12727E-09	0	0	0	0	0	3.98182E-08	0	0	0	0	0	0	0
Bacillus_sp._FJAT-22090	2.09273E-07	5.86364E-07	1.29182E-07	2.66909E-07	5.71462E-07	5.35769E-07	1.13092E-06	6.55385E-07	8.36727E-07	1.37545E-07	5.55091E-07	2.91818E-07	0.000000445	1.57333E-07	6.738E-07	2.61267E-07
Bacillus_sp._FJAT-42376	3.40545E-07	2.58355E-06	1.31727E-07	5.59909E-07	7.25462E-07	1.79585E-06	5.97462E-07	5.82231E-07	4.95636E-07	6.18636E-07	6.51455E-07	1.31391E-06	5.33067E-07	5.49667E-07	6.05733E-07	5.15667E-07
Bacillus_sp._Y-01	2.86455E-06	3.12727E-08	1.49545E-07	1.93636E-08	6.27846E-07	1.96154E-07	1.13731E-06	1.58654E-06	1.96055E-06	0.000004169	0.0000041	1.11077E-06	7.526E-07	1.37333E-07	1.66667E-07	1.7402E-06

Bacillus_pacificus	0	0	8.25455E-09	7.97273E-08	0	0	0	4.64615E-08	0	7.57273E-08	0	7.13636E-09	2.24E-08	0	4.44E-09	9.33333E-09
Bacillus_luti	2.02273E-07	0	1.23636E-08	6.61545E-08	6.73846E-08	0	1.55846E-07	1.50462E-07	1.73636E-07	0.000000034	6.68182E-08	7.13636E-09	7.46E-08	1.48667E-07	1.288E-07	1.50867E-07
Bacillus_marisflavi	1.42518E-06	6.95636E-07	7.83436E-07	6.88091E-07	1.03146E-06	5.54077E-07	9.35077E-07	7.54154E-07	1.18336E-06	8.50545E-07	9.66909E-07	1.97155E-06	5.49133E-07	6.26067E-07	7.07467E-07	8.54467E-07
Bacillus_sp._PAMC26568	1.63909E-07	2.34545E-08	9.10909E-08	6.72727E-08	1.61077E-07	0	4.33846E-08	2.46923E-08	0.000002218	1.06091E-07	1.47291E-06	7.53636E-07	1.33867E-07	0.00000007	0	6.33333E-08
Bacillus_mycoides	1.23809E-06	5.71273E-07	4.175E-07	0.000000702	7.56615E-07	1.22523E-06	1.10369E-06	4.81231E-07	6.32273E-07	5.59545E-07	1.65727E-06	5.56818E-07	0.00000138	0.000002123	1.57773E-06	2.4914E-06
Bacillus_methanolicus	2.88727E-07	0.000000065	1.48182E-07	4.65273E-07	2.71769E-07	2.95385E-08	8.69231E-08	3.48692E-07	7.07636E-07	0.000000404	4.66818E-07	4.98636E-07	1.304E-07	2.27867E-07	2.598E-07	2.91067E-07
Bacillus_vietnamensis	2.57455E-06	3.76409E-07	6.85545E-07	5.50909E-07	7.20308E-07	5.47077E-07	2.87285E-06	2.38285E-06	3.09727E-06	4.66791E-06	4.70882E-06	2.60773E-06	9.84267E-07	3.494E-07	6.37267E-07	1.6676E-06
Bacillus_filamentosus	0.000000657	4.88273E-07	4.14855E-07	6.64182E-07	0.000000854	5.87538E-07	3.61923E-07	3.77154E-07	1.03891E-06	4.74455E-07	1.27127E-06	1.58955E-06	6.29333E-07	8.69933E-07	1.27447E-06	7.376E-07
Bacillus_beveridgei	3.03909E-07	2.01364E-07	8.40455E-08	1.21091E-07	2.21846E-07	1.14462E-07	4.39923E-07	0.000000188	4.45545E-07	4.23455E-07	5.20091E-07	2.47636E-07	1.21333E-07	3.35133E-07	1.838E-07	0
Bacillus_weihaiensis	3.39727E-07	4.17318E-07	1.62182E-07	1.67273E-07	3.07923E-07	8.23846E-08	4.38154E-07	1.34615E-07	1.17627E-06	0.000000309	2.37273E-07	3.94545E-07	2.14E-08	0.000000197	4.00533E-07	2.174E-07
Bacillus_altitudinis	5.14182E-07	3.28182E-07	8.23091E-08	2.14273E-07	2.79769E-07	6.44462E-07	6.90538E-07	1.97923E-07	7.90636E-07	3.31909E-07	4.39182E-07	3.67273E-07	1.76533E-07	3.438E-07	1.498E-07	5.358E-07
Bacillus_infantis	8.64273E-07	3.31545E-07	3.188E-07	7.72636E-07	4.51692E-07	4.72077E-07	6.22538E-07	6.65846E-07	7.42636E-07	0.00000067	7.91727E-07	0.0000015	6.19133E-07	1.0704E-06	1.12507E-06	6.182E-07
Bacillus_glycinifermentans	8.22545E-07	7.25864E-07	3.03636E-07	6.69909E-07	1.26585E-06	2.01815E-06	6.33154E-07	8.97231E-07	5.01182E-07	3.91364E-07	6.26182E-07	1.09318E-06	0.000000605	8.68067E-07	8.92067E-07	4.57133E-07
Bacillus_ciccensis	2.23909E-07	1.26473E-06	0.00000048	1.44436E-06	6.01615E-07	2.16923E-08	1.27154E-07	4.18846E-07	1.05082E-06	3.93727E-07	0.000000667	1.07445E-06	2.55667E-07	7.53533E-07	7.28107E-07	6.016E-07
Bacillus_circulans	1.70873E-06	4.56727E-07	0.000000236	8.92182E-07	6.33615E-07	2.79692E-07	1.45354E-06	1.83469E-06	1.94527E-06	1.85473E-06	0.000001742	2.11973E-06	4.786E-07	5.73733E-07	1.43473E-06	1.38193E-06
Bacillus_smithii	0.00000039	3.67636E-07	1.21273E-07	1.50455E-07	4.42846E-07	1.10154E-07	1.23769E-07	2.25615E-07	4.68727E-07	3.04364E-07	3.55545E-07	1.07455E-06	2.334E-07	2.014E-07	1.52933E-07	1.97333E-07
Bacillus_horikoshii	0.000004201	6.08045E-07	6.28455E-07	7.70364E-07	7.54769E-07	1.18892E-06	1.88854E-06	3.39923E-06	0.000004664	6.39009E-06	0.000007415	3.15473E-06	1.47007E-06	7.90133E-07	1.19933E-06	2.06467E-06
Bacillus_freudenreichii	0.000000486	3.34727E-07	3.85364E-07	2.06091E-07	3.57308E-07	9.40846E-07	5.97308E-07	6.57538E-07	3.26064E-06	3.75545E-07	0.000000668	7.21818E-07	2.87467E-07	4.14467E-07	5.4364E-07	2.31933E-07
Bacillus_megaterium	3.16091E-06	2.02736E-06	0.000000751	3.80018E-06	2.38038E-06	1.16654E-06	2.09092E-06	2.57977E-06	2.46345E-06	3.10491E-06	4.58473E-06	4.19636E-06	1.36233E-06	2.16553E-06	2.8798E-06	2.43173E-06
Bacillus_coagulans	3.30291E-05	2.87596E-05	1.32532E-05	1.40373E-05	1.98385E-06	3.36846E-06	1.12715E-05	1.50292E-05	1.11055E-05	9.19364E-06	1.78818E-05	0.00001004	5.45433E-06	5.4658E-06	5.0102E-06	2.79813E-06
Bacillus_flexus	1.54818E-06	4.34091E-07	6.75527E-07	1.05436E-06	1.34192E-06	2.30769E-07	9.86538E-07	1.54462E-06	1.06782E-06	5.58818E-07	1.24773E-06	2.13491E-06	1.1026E-06	9.33867E-07	1.95713E-06	2.32853E-06
Bacillus_cellulosilyticus	4.82636E-07	4.15909E-07	2.94545E-07	2.51091E-07	1.02831E-06	0	2.64769E-07	2.09923E-07	7.72818E-07	7.50455E-07	5.74636E-07	9.70182E-07	6.60467E-07	1.97493E-06	1.26107E-06	4.68467E-07
Bacillus_cohnii	1.47036E-06	4.87818E-07	2.39345E-07	0.000001075	3.97246E-06	0	9.73231E-07	2.90954E-06	8.41645E-06	2.01355E-06	3.90382E-06	1.52218E-06	3.9618E-06	2.4358E-06	0.000004114	2.17333E-06
Bacillus_dafuensis	0.000000399	0	1.95909E-07	8.63636E-08	1.52846E-06	4.45231E-07	1.48769E-07	4.68154E-07	6.70909E-07	0.000000075	6.74182E-07	4.32727E-07	7.31933E-07	9.282E-07	1.45711E-06	8.56333E-07
Bacillus_pumilus	2.97455E-06	1.18391E-06	3.83545E-07	6.86545E-07	6.66923E-07	3.12769E-07	2.17777E-06	1.73538E-06	3.06264E-06	0.000004145	4.10627E-06	2.56791E-06	8.96333E-07	9.996E-07	1.05597E-06	1.69513E-06
Bacillus_miscanthi	8.86818E-07	1.20273E-06	1.04727E-07	9.15909E-07	3.23462E-07	3.78923E-07	9.00538E-07	7.57308E-07	2.94455E-07	2.60364E-07	7.00545E-07	4.12455E-07	9.62667E-08	9.45667E-07	7.59333E-07	5.148E-07
Bacillus_wiedmannii	0	0	0	0	4.25615E-07	0.000000026	0	9.03846E-08	2.62818E-07	0.000000034	2.76364E-08	1.39727E-07	1.754E-07	2.09133E-07	0.000000604	2.57333E-07
Bacillus_anthracis	6.60909E-08	0.000000033	5.93636E-08	4.40909E-08	4.11538E-08	3.06923E-07	3.11538E-08	6.04615E-08	0.000000027	7.77273E-08	1.14818E-07	0.000000092	1.49067E-07	1.17533E-07	1.91267E-07	4.01333E-08
Bacillus_sp._JAS24-2	2.96364E-08	0	0	0	7.11154E-07	2.21538E-07	0	1.46923E-08	2.16364E-08	5.22727E-08	0	4.28182E-08	5.46533E-07	2.886E-07	1.2886E-06	6.648E-07
Bacillus_pseudomycoides	3.02455E-07	0	4.90909E-08	3.32091E-07	5.44769E-07	0	1.92308E-07	1.12569E-06	2.18182E-06	6.72909E-07	1.62518E-06	1.04545E-06	1.01187E-06	4.89267E-07	1.1408E-06	6.54867E-07
Bacillus_cytotoxicus	2.31182E-07	1.37273E-08	8.28455E-08	8.38636E-07	7.38462E-07	9.20846E-07	3.79462E-07	6.56538E-07	7.03545E-07	2.22818E-07	2.20182E-07	7.46273E-07	8.64667E-07	1.08233E-06	1.2002E-06	2.58527E-06
Bacillus_thuringiensis	3.43091E-06	1.26318E-06	9.81318E-07	2.45336E-06	0.000002191	1.13608E-06	2.31746E-06	2.67238E-06	3.04627E-06	2.06918E-06	2.62636E-06	3.15709E-06	0.000002501	2.72647E-06	2.5018E-06	2.3664E-06
Bacillus_cereus	4.64818E-06	9.85273E-07	1.99118E-06	2.35045E-06	5.62385E-06	1.20338E-06	3.17046E-06	2.51838E-06	5.59455E-06	4.70182E-06	3.95991E-06	4.90455E-06	0.000003984	6.49553E-06	0.00000468	3.5058E-06
Bacillus_tequilensis	4.28818E-07	0	0	5.92455E-08	1.05846E-07	4.87077E-07	5.26154E-08	7.13077E-08	0.000000027	1.69818E-07	1.49818E-07	3.57273E-08	1.69867E-07	1.46733E-07	1.34467E-07	1.66667E-08
Bacillus_licheniformis	3.83091E-07	1.97091E-07	1.05073E-07	6.01545E-07	5.20538E-07	1.26154E-06	0.000000318	2.21077E-07	4.17818E-07	5.02455E-07	5.55636E-07	1.07064E-06	3.53867E-07	5.21333E-07	4.776E-07	3.49533E-07
Bacillus_mojavensis	0	0	0	5.18182E-09	2.88462E-08	0	6.40769E-08	1.46923E-08	0	0	0	2.50636E-07	2.14E-08	1.258E-07	0	0
Bacillus_sonorensis	0.000001421	5.21545E-07	1.01982E-06	0.000001391	1.42646E-06	2.71462E-07	0.000000237	5.99692E-07	1.35273E-06	2.56545E-06	1.64564E-06	2.45227E-06	0.000000774	1.00613E-06	1.18353E-06	1.2826E-06
Bacillus_atrophaeus	1.96364E-07	7.38182E-08	4.53455E-08	5.27545E-07	0.000000224	1.18462E-07	4.39308E-07	5.54615E-08	3.27727E-07	1.48273E-07	1.94909E-07	2.92636E-07	0.000000171	7.68267E-07	3.52307E-07	5.39333E-08
Bacillus_velezensis	7.42455E-07	3.70045E-07	3.748E-07	5.34364E-07	2.46769E-07	0.000001926	5.72538E-07	4.99923E-07	1.14673E-06	8.15818E-07	4.38636E-07	1.01227E-06	3.80267E-07	6.03133E-07	8.63133E-07	9.59933E-07
Bacillus_amyloliquefaciens	1.53245E-06	6.88955E-07	8.58709E-07	2.79973E-06	2.52292E-06	8.33923E-07	2.70385E-07	7.80462E-07	2.87727E-06	1.76291E-06	1.73245E-06	2.41864E-06	2.27633E-06	0.0000021	2.53353E-06	0.000002438
Bacillus_subtilis	3.35464E-06	8.88727E-07	8.00091E-07	3.61182E-06	2.56177E-06	8.88077E-07	1.68192E-06	2.35392E-06	0.00000485	0.00000405	4.03818E-06	5.10091E-06	2.93873E-06	0.000003802	3.1614E-06	0.000003056
Bacillus_sp._DSL-17	1.20136E-06	0.00000026	7.82309E-07	3.98336E-07	4.69538E-07	0	4.40538E-07	1.08338E-06	8.42545E-07	1.81636E-07	5.82727E-07	7.09818E-07	0.00000027	2.46133E-07	4.86267E-07	2.66533E-07
Bacillus_sp._M4U3P1	0.000000124	0	9.88182E-08	1.00636E-07	1.23308E-07	1.14615E-07	8.92308E-08	2.33846E-07	3.32909E-07	4.54818E-07	6.38182E-07	1.31955E-06	1.19333E-07	1.07467E-07	2.86133E-07	5.626E-07
Bacillus_sp._KH172YL63	3.84909E-07	3.50909E-08	1.32591E-07	3.01636E-07	3.96231E-07	9.72308E-07	2.41154E-07	3.32308E-07	4.06182E-07	3.77182E-07	0.000000426	3.32636E-07	2.314E-07	1.97667E-07	3.24867E-07	4.596E-07
Bacillus_sp._HD4P25	2.84545E-07	3.01818E-08	8.64545E-08	0.000000423	1.62538E-07	5.12308E-08	7.60462E-07	0.000000967	1.22418E-06	0.000000189	8.70455E-07	4.37545E-07	6.86133E-07	0.000000637	9.81867E-07	4.282E-07
Bacillus_sp._OxB-1	0.000001366	0.000000922	1.56182E-07	1.21191E-06	6.17308E-07	1.35985E-06	2.39085E-06	0.000000744	2.38118E-06	1.12155E-06	1.03227E-06	1.06391E-06	0.000000472	0.000000444	7.20107E-07	5.81867E-07
Bacillus_sp._NSP9.1	1.01055E-06	3.61045E-07	7.76273E-07	6.12818E-07	4.22692E-07	8.19923E-07	5.11923E-07	1.21785E-06	7.42636E-07	3.58909E-07	8.80818E-07	1.29282E-06	3.58333E-07	6.66533E-07	8.40733E-07	6.682E-07
Bacillus_sp._FJAT-45348	2.25264E-06	1.65091E-07	7.18091E-07	4.85545E-07	5.43692E-07	9.36923E-08	1.13731E-06	6.01615E-07	1.11091E-06	5.08636E-07	7.04636E-07	1.74009E-06	2.64667E-08	2.622E-07	1.936E-07	2.58667E-07
Bacillus_sp._CS13	5.21091E-07	5.41455E-07	1.63127E-07	3.00545E-07	9.46154E-08	0	4.59462E-07	5.15231E-07	2.24909E-07	1.20273E-07	4.83455E-07	3.33455E-07	2.19533E-07	1.75267E-07	2.7544E-07	1.77867E-07
Bacillus_sp._X1(2014)	1.45845E-06	3.74091E-07	5.024E-07	6.97636E-07	2.53154E-07	0.000001387	8.29538E-07	1.01238E-06	9.20909E-07	7.66455E-07	8.31818E-07	9.79091E-07	1.73933E-07	7.508E-07	3.97933E-07	0.000000367
Bacillus_sp._FJAT-18017	0.000009961	1.89973E-06	5.24364E-07	3.46091E-07	2.94108E-06	1.89008E-06	4.86915E-06	3.68954E-06	1.02374E-05	2.02709E-05	8.44645E-06	3.41309E-06	3.14927E-06	1.5976E-06	1.25571E-06	3.83407E-06
Bacillus_sp._N1-1	2.18727E-07	1.53182E-07	2.04909E-08	6.30909E-07	1.31023E-06	0	4.89154E-07	7.74308E-07	2.34909E-07	9.49091E-08	4.88636E-07	3.28455E-07	1.04007E-06	7.50867E-07	0.000002169	3.0324E-06
Bacillus_sp._THAF10	2.26545E-07	0	7.40909E-08	1.13455E-06	1.45323E-06	1.35385E-07	7.35077E-07	5.63769E-07	3.92818E-07	2.57182E-07	4.61364E-07	3.83182E-07	9.52733E-07	1.09887E-06	1.30707E-06	1.9088E-06
Bacillus_sp._S3	9.63455E-07	0.000000904	3.53455E-07	1.15709E-06	2.79308E-07	0	3.19538E-07	4.99385E-07	1.99545E-06	7.91455E-07	9.20364E-07	6.75636E-07	1.80333E-07	6.322E-07	5.73133E-07	3.018E-07
Bacillus_sp._SB49	1.01827E-06	7.65455E-07	4.46364E-07	7.40636E-07	6.24308E-07	8.46462E-07	8.11692E-07	5.49231E-07	6.55182E-07	4.70364E-07	9.61818E-07	7.90545E-07	2.87533E-07	8.068E-07	2.974E-07	0.000000318
Bacillus_sp._BD59S	1.92727E-07	0.00000026	2.06364E-08	5.33909E-07	1.34846E-07	0	4.31538E-07	6.55385E-08	5.79091E-08	0	2.61818E-07	7.64545E-08	4.51333E-08	4.09533E-07	2.578E-07	1.144E-07
Bacillus_sp._Y1	1.00764E-06	2.53818E-07	2.43182E-07	9.91545E-07	6.76769E-07	5.93077E-08	2.80769E-07	7.96615E-07	1.54473E-06	1.19709E-06	1.92291E-06	3.07545E-06	6.06267E-07	1.06807E-06	5.60067E-07	7.68933E-07
Abiotrophia_defectiva	1.94909E-07	0.000000117	3.70818E-07	4.94909E-07	1.65538E-07	1.17146E-06	1.99615E-07	3.57846E-07	3.82091E-07	2.74727E-07	5.49909E-07	6.56818E-07	2.92667E-07	8.51333E-08	2.74107E-07	4.94E-08
Suicoccus_acidiformans	8.99273E-07	0.000000065	4.57091E-07	1.62382E-06	4.35692E-07	8.52846E-07	1.08077E-07	4.47692E-07	0.000001166	1.38145E-06	1.59764E-06	1.35064E-06	4.64533E-07	6.026E-07	7.158E-07	5.65933E-07
Aerococcus_sanguinicola	4.24455E-07	0	7.08545E-08	1.76727E-07	2.98692E-07	3.07077E-07	1.67692E-07	1.46608E-06	4.10545E-07	3.93545E-07	0.00000066	4.38545E-07	9.34E-08	2.18733E-07	5.99933E-07	3.23267E-07
Aerococcus_viridans	5.11364E-07	3.75227E-07	2.02127E-07	1.13445E-06	0.000000112	1.10462E-07	3.25385E-07	1.68462E-07	4.06727E-07	3.18636E-07	7.43182E-07	7.22818E-07	7.49333E-08	1.66267E-07	2.134E-07	1.982E-07
Aerococcus_urinaehominis	2.70909E-08	5.05455E-08	8.25455E-09	3.37882E-07	0	3.83077E-08	3.11538E-08	2.47692E-08	0	0	1.06545E-07	1.80364E-07	2.64E-08	3.48E-08	1.49333E-08	6.83333E-08
Aerococcus_urinae	1.13391E-06	6.13455E-07	4.21791E-07	6.63273E-07	8.25615E-07	5.06385E-07	2.78615E-07	0.00000097	0.000000799	8.04818E-07	5.46364E-07	1.80882E-06	6.126E-07	8.28467E-07	4.264E-07	5.98667E-07
Aerococcus_christensenii	1.09909E-06	4.42818E-07	1.97273E-07	9.83909E-07	2.65408E-06	1.60646E-06	3.09677E-06	6.05308E-07	2.03409E-06	1.33764E-06	7.21818E-07	2.55191E-06	1.4316E-06	2.1836E-06	1.55267E-06	0.000001537
Aerococcus_urinaeequi	1.16091E-07	1.68182E-08	3.71455E-07	2.34545E-07	7.90769E-08	3.96154E-08	8.86308E-07	6.90231E-07	2.40182E-07	0	1.07418E-06	3.11091E-07	8.50667E-08	2.046E-07	8.74933E-07	5.29333E-07
Marinilactibacillus_sp._15R	3.48182E-07	2.70909E-08	1.46455E-07	1.76427E-07	2.61154E-07	0.000000427	3.40769E-07	6.59077E-07	5.53636E-07	2.86364E-07	7.45818E-07	7.35727E-07	4.22067E-07	7.398E-07	6.896E-07	5.44733E-07
Jeotgalibaca_porci	1.12091E-07	1.90727E-07	0.000000153	6.74545E-08	1.29846E-07	0	1.76615E-07	2.23308E-07	0.000000734	1.33091E-07	9.17727E-07	1.90182E-07	1.538E-07	2.04467E-07	3.28067E-07	2.31467E-07
Jeotgalibaca_dankookensis	8.90364E-07	2.70455E-07	7.87182E-07	9.17091E-07	3.11154E-07	0	1.17346E-06	2.06492E-06	2.57273E-07	2.99182E-07	2.41782E-06	9.09727E-07	3.32533E-07	3.21267E-07	0.000001086	1.1966E-06
Jeotgalibaca_arthritidis	4.19273E-06	1.04818E-06	8.55364E-07	1.67582E-06	2.75769E-07	6.84769E-07	2.31246E-06	1.84685E-06	4.55673E-06	4.53118E-06	5.69091E-06	3.04155E-06	7.476E-07	3.134E-07	7.98533E-07	1.72073E-06
Jeotgalibaca_sp._PTS2502	2.54273E-07	9.36364E-08	1.40545E-07	3.71545E-07	2.88462E-08	3.61308E-07	2.73846E-08	1.20923E-07	1.19391E-06	1.65909E-07	4.42455E-07	9.45455E-08	8.99333E-08	9.58667E-08	1.184E-07	8.90667E-08
Jeotgalibaca_ciconiae	2.11273E-07	1.68182E-08	1.00073E-07	8.26091E-07	1.28038E-06	4.38923E-07	5.32615E-07	1.10392E-06	1.43745E-06	3.33364E-07	8.63364E-07	1.49591E-06	9.634E-07	0.000000817	2.16847E-06	2.49527E-06
Dolosigranulum_pigrum	1.13155E-06	5.38818E-07	0.000000672	0.000002551	0.00000193	5.13308E-07	2.24423E-06	1.83338E-06	1.67027E-06	6.76273E-07	1.71973E-06	1.38945E-06	1.50153E-06	1.4818E-06	2.20347E-06	0.000003728
Carnobacterium_sp._CP1	4.59155E-06	7.72909E-07	8.17545E-07	4.04727E-07	1.54231E-06	1.13862E-06	2.25008E-06	2.34546E-06	0.000002222	2.06018E-06	2.49091E-06	2.32364E-06	5.63333E-07	5.34067E-07	5.108E-07	1.52167E-06
Carnobacterium_sp._17-4	1.76909E-07	0.000000065	1.10855E-07	4.79091E-09	0.000000176	5.29231E-08	0	2.46923E-08	0.000000167	3.96455E-07	2.01545E-07	7.84545E-08	6.70667E-08	1.39333E-07	2.318E-07	2.05533E-07
Carnobacterium_inhibens	1.23036E-06	4.93182E-07	6.91164E-07	3.38636E-07	8.40154E-07	2.61538E-07	1.86385E-07	4.23769E-07	7.35364E-07	0.000000528	4.94818E-07	8.52182E-07	1.65667E-07	4.462E-07	4.28933E-07	4.43267E-07
Carnobacterium_maltaromaticum	1.31291E-06	6.24182E-07	3.09727E-07	1.08109E-06	6.02769E-07	4.50385E-07	1.38185E-06	6.82846E-07	7.96182E-07	4.50364E-07	1.43318E-06	1.81045E-06	4.30533E-07	4.38133E-07	1.06233E-06	8.67333E-07
Carnobacterium_divergens	1.69436E-06	0.00000083	3.86545E-07	1.70155E-06	7.69846E-07	5.27308E-07	0.00000175	6.11923E-07	1.85155E-06	7.44636E-07	1.17436E-06	0.000000809	5.774E-07	1.3546E-06	6.85067E-07	1.09373E-06
Oenococcus_sp._UCMA_16435	0	0	3.70909E-08	9.54545E-09	2.75385E-08	0	0	0	0	1.57273E-07	3.79091E-08	0	0	3.50667E-08	1.49333E-08	0
Oenococcus_oeni	3.74091E-07	0	5.34545E-08	1.50182E-07	2.58846E-07	0	0.000000541	0.000000265	2.64545E-08	5.22727E-08	0	1.06364E-07	2.09333E-08	1.104E-07	1.12667E-08	0
Oenococcus_kitaharae	1.23091E-07	1.18545E-07	4.12182E-08	0.000000196	4.66077E-07	2.16923E-08	5.36923E-08	3.73769E-07	1.14545E-07	1.60545E-07	5.21545E-07	5.75455E-07	2.72E-08	3.29333E-08	6.67333E-08	2.94733E-07
Oenococcus_sicerae	3.69091E-08	0.00000013	0	0.000000176	3.15385E-08	0	1.31538E-07	1.90769E-08	6.68182E-08	0	7.29091E-08	4.79091E-08	4.74667E-08	2.22667E-07	1.54867E-07	4.34E-08
Weissella_cryptocerci	5.27273E-08	0	0	0	0	1.59692E-07	6.41538E-08	3.70077E-07	0	0	0	0.000000035	5.58667E-08	0	2.05933E-07	0
Weissella_soli	0.00000006	0	0	1.12882E-07	3.40846E-07	4.06154E-08	3.43154E-07	2.92308E-07	7.95455E-08	2.86364E-07	0	2.545E-07	3.758E-07	8.91333E-08	1.71867E-07	2.49667E-07
Weissella_ceti	1.12091E-07	0	0	4.79091E-09	3.34615E-08	0.000000127	0	3.81538E-08	1.09182E-07	0	6.68182E-08	1.25545E-07	2.24E-08	1.71333E-08	7.26667E-08	2.64467E-07
Weissella_jogaejeotgali	0	1.03636E-08	0	5.18182E-09	1.06385E-07	1.44615E-07	3.11538E-08	2.25692E-07	0	0	1.02727E-08	8.26818E-08	1.582E-07	6.61333E-08	4.09467E-07	4.95333E-08
Weissella_sp._HDW19	3.61636E-07	3.42455E-07	7.66364E-08	1.27636E-07	0.000000434	0	4.37538E-07	1.51308E-07	8.20909E-08	1.88182E-07	4.82636E-07	3.47091E-07	1.53467E-07	3.57067E-07	8.216E-07	7.91067E-07
Weissella_diestrammenae	0.000000089	0.000000065	4.12727E-09	1.06909E-07	2.88462E-08	0	2.73846E-08	3.66923E-07	0	0	1.09818E-07	7.12727E-08	1.49067E-07	2.074E-07	1.47107E-07	7.81933E-07
Weissella_hellenica	1.88182E-07	2.34545E-08	7.54545E-08	8.88818E-08	4.38462E-07	1.39077E-07	5.54923E-07	6.91077E-07	7.95455E-08	9.22727E-08	6.53818E-07	7.35818E-07	1.96067E-07	2.70733E-07	9.22E-08	2.46267E-07
Weissella_confusa	2.56364E-08	3.59545E-08	3.48E-08	9.23636E-08	1.30462E-07	2.13846E-08	2.63077E-08	7.98462E-08	2.07545E-07	5.22727E-08	3.07091E-07	7.55455E-08	2.14667E-08	0	2.99333E-08	7.83333E-08
Weissella_cibaria	7.10455E-07	4.39636E-07	2.35455E-07	1.01782E-06	5.20385E-07	8.32077E-07	2.49154E-07	1.46169E-06	4.65364E-07	3.10182E-07	9.22364E-07	0.000000705	5.164E-07	5.05467E-07	7.14267E-07	2.39313E-06
Weissella_viridescens	5.92727E-08	0	0	1.43545E-07	1.44077E-07	0	3.11538E-08	6.55385E-08	1.99727E-07	1.61273E-07	3.95455E-08	1.01591E-07	5.36E-08	0.00000027	7.59333E-08	9.08E-08
Weissella_paramesenteroides	2.41636E-07	8.04091E-08	8.25455E-09	9.28091E-07	7.83385E-07	8.53846E-08	3.44615E-07	9.12154E-07	0.000000027	2.92818E-07	4.04636E-07	2.30636E-07	8.33867E-07	7.05667E-07	2.02713E-06	2.25967E-06
Weissella_koreensis	1.12091E-07	1.39364E-07	1.10909E-07	2.16955E-06	0.000001174	0	1.13769E-06	1.01169E-06	3.79545E-07	3.36727E-07	5.21364E-07	1.26218E-06	1.09387E-06	1.6758E-06	1.5308E-06	2.8608E-06
Leuconostoc_sp._C2	0	0	0	0	0	0	0	0	0	0	0	0	0	1.02533E-07	0	0
Leuconostoc_kimchii	0	0	0	0	0	0	0	0	0	0	0	0	6.08E-08	0	0	0
Leuconostoc_lactis	2.96364E-08	0	0	0	2.26923E-08	3.96154E-08	0	0	5.34545E-08	0	6.68182E-08	0	1.25933E-07	3.50667E-08	3.41733E-08	1.37067E-07
Leuconostoc_carnosum	0	0	0	2.43636E-08	0	0	0	6.41538E-08	3.94545E-08	0	2.76364E-08	4.45909E-08	0	0	4.44E-09	0
Leuconostoc_pseudomesenteroides	2.96364E-08	2.63636E-08	0	2.41545E-08	0.000000091	2.16923E-08	0.00000008	3.56923E-08	8.67273E-08	0	1.04909E-07	1.42727E-08	7.11333E-08	0.000000098	7.11333E-08	8.06E-08
Leuconostoc_mesenteroides	1.52336E-06	2.72727E-08	1.27545E-07	6.42727E-08	1.89077E-07	1.41308E-07	1.29462E-06	1.17238E-06	1.40736E-06	2.90536E-06	1.94336E-06	9.45364E-07	4.35333E-07	2.53133E-07	1.12533E-07	5.884E-07
Leuconostoc_suionicum	2.22836E-06	1.98182E-08	0.00000014	1.03636E-08	2.28308E-07	2.61538E-07	1.33108E-06	1.69877E-06	2.54827E-06	3.27745E-06	4.37727E-06	0.000001667	9.20267E-07	7.11333E-08	2.03533E-07	6.63333E-07
Leuconostoc_gelidum	2.10818E-07	2.95955E-07	4.93455E-08	2.91545E-08	6.03923E-07	6.27692E-08	3.33077E-08	0.00000025	0.000000077	7.57273E-08	3.63364E-07	0.000000636	1.38467E-06	1.5538E-06	1.42227E-06	9.09867E-07
Leuconostoc_citreum	0.000004365	0.00000039	2.32818E-06	5.06809E-06	3.16923E-06	0	3.95885E-06	3.89692E-06	2.47845E-06	3.02818E-06	5.00182E-06	4.92727E-06	2.0058E-06	4.40067E-06	4.14933E-06	2.53087E-06
Lactococcus_sp._TMW21615	0	0	0	0	0	5.96923E-08	0	0	0	0	0	0	0	0	0	0
Lactococcus_petauri	5.92727E-08	0	0	0	0	4.27692E-08	6.41538E-08	3.22308E-08	0	0	1.02727E-08	7.13636E-09	0	0	0	0
Lactococcus_sp._TMW21612	1.48182E-07	0	0	0	0	6.56154E-08	0.000000127	0.000000011	7.15455E-08	0	2.76364E-08	1.64182E-07	0.000000119	0	5.94E-08	1.86667E-08
Lactococcus_sp._1JSPR-7	1.52818E-07	2.81455E-07	0.000000228	2.30364E-07	5.20769E-08	0	0	7.72308E-08	4.05818E-07	0.000000333	0	1.49182E-07	1.03733E-07	1.376E-07	2.084E-07	3.19333E-07
Lactococcus_sp._KACC_19320	0.000001052	2.23818E-07	0	3.78973E-07	1.47069E-06	1.52077E-07	6.82385E-07	1.06377E-06	1.07218E-06	4.81727E-07	5.83727E-07	4.69818E-07	5.27533E-07	2.29067E-07	4.84933E-07	2.94933E-07
Lactococcus_raffinolactis	8.44091E-07	1.40364E-07	5.48182E-08	2.18636E-07	3.61692E-07	3.87538E-07	1.28538E-07	1.71846E-07	2.64364E-07	1.97636E-07	2.31545E-07	9.98273E-07	5.98933E-07	1.80867E-07	5.26733E-07	4.06533E-07
Lactococcus_garvieae	3.69182E-07	0.000000136	9.64545E-08	1.16727E-07	2.88462E-08	4.36923E-07	3.11538E-08	3.94615E-08	7.50909E-08	1.11273E-07	3.97273E-07	2.91273E-07	0	1.034E-07	1.3344E-07	1.37867E-07
Lactococcus_piscium	1.33182E-07	5.05273E-07	4.94545E-08	8.02091E-07	7.37769E-07	2.13846E-08	7.81154E-07	1.04062E-06	5.58364E-07	5.05273E-07	4.07182E-07	1.15532E-06	8.498E-07	1.00293E-06	2.13847E-06	2.2068E-06
Lactococcus_lactis	8.34336E-06	2.85664E-06	1.39027E-06	1.26136E-06	1.63392E-06	3.47969E-06	3.93423E-06	4.60569E-06	5.72918E-06	1.11294E-05	1.30685E-05	5.82318E-06	1.85773E-06	1.70373E-06	1.51247E-06	2.80073E-06
Streptococcus_sp._FDAARGOS_521	0	0	0	0	0	0	0	0	0	3.89091E-08	0	0	0	0	0	0
Streptococcus_gwangjuense	0	8.86364E-09	0	0	0	4.13846E-08	0	0	0	0	0	0	0	0	0	0
Streptococcus_ruminantium	0	0	0	0	0	0	0	0	0	0	0	0	0	3.50667E-08	0	9.33333E-09
Streptococcus_sp._HSISB1	0	0	0	0	2.88462E-08	0	0	0	0	0	0	0	2.94667E-08	0.000000014	0	8.19333E-08
Streptococcus_sp._CNU_G2	0	0	0	0	0	0	0	0	0	0	0	0.000000057	0	0	0	0
Streptococcus_sp._NPS_308	0	0	0	0	0	0	0	0	0	0.000000047	0	0	2.09333E-08	0	7.26667E-08	0
Streptococcus_sp._HSISS2	0	0	0	0	0	0	0	0	0	0	0	0	0	0	4.82E-08	0
Streptococcus_sp._NCTC_11567	0	0	0	0	0	0	0	0	0	0	6.68182E-08	0	0	0	0	0
Streptococcus_constellatus	0	3.35455E-08	0	6.64545E-08	3.15385E-08	7.43077E-08	2.88769E-07	3.57692E-08	2.16364E-08	0	1.02727E-08	7.13636E-09	0	0	0	1.98667E-08
Streptococcus_sp._I-G2	0	0	0	0	0	0	0	0	0	0	1.02727E-08	0	0	0	0	0
Streptococcus_milleri	1.11273E-07	0	0	1.28636E-07	3.63231E-07	5.71231E-07	6.14077E-07	1.04231E-07	1.56727E-07	0.000000308	4.81818E-08	3.60364E-07	4.90333E-07	4.88867E-07	4.01267E-07	3.85667E-07
Streptococcus_sp._I-P16	2.30909E-08	1.16364E-08	0	3.99091E-08	4.13846E-08	1.40623E-06	8.13846E-08	1.46923E-08	2.72727E-08	0.000000096	1.34182E-07	9.03636E-08	2.91333E-08	9.24667E-08	9.70667E-08	1.83933E-07
Streptococcus_koreensis	1.58182E-08	0	0	0	0	0	0	0	0	0	6.65455E-08	0	0	3.09333E-08	2.97333E-08	0
Streptococcus_macedonicus	1.58182E-08	9.54545E-09	0	0	0	4.27692E-08	0	0	0	3.03636E-08	0	0	0	3.35333E-08	0	0
Streptococcus_anginosus	2.75727E-07	2.56636E-07	1.89273E-07	0.000000142	2.92692E-07	1.61608E-06	3.82846E-07	3.99769E-07	0.000000243	2.90091E-07	3.30818E-07	7.99091E-07	2.66867E-07	3.124E-07	1.30773E-07	3.32133E-07
Streptococcus_sp._oral_taxon_431	2.95455E-07	0	1.03727E-07	9.61818E-08	1.63846E-07	5.19231E-08	0	6.69231E-08	9.62727E-08	7.29091E-08	0	5.30909E-08	1.57933E-07	1.204E-07	4.714E-07	4.07533E-07
Streptococcus_urinalis	4.48545E-07	1.16364E-08	1.58545E-07	1.91818E-08	1.43231E-07	2.13846E-08	2.63077E-08	3.05462E-07	0.000000183	4.10727E-07	2.97545E-07	0.000000117	2.40267E-07	4.15533E-07	2.508E-07	4.41467E-07
Streptococcus_pseudopneumoniae	8.23636E-08	0	0	5.18182E-09	2.05385E-08	2.13846E-08	0	0	8.20909E-08	1.25273E-07	0.000000154	0	1.144E-07	1.58E-08	0	0
Streptococcus_sp._1643	1.11818E-07	0	0	4.88182E-08	0	0	2.63077E-08	0	1.94909E-07	9.36364E-08	8.71818E-08	0.000000097	9.34667E-08	0.000000066	4.184E-08	1.43333E-07
Streptococcus_ferus	0.000000246	0	1.63636E-08	2.33427E-07	1.83923E-07	1.52462E-07	1.44462E-07	2.42615E-07	2.60364E-07	5.09818E-07	3.99364E-07	2.93909E-07	4.17933E-07	4.67267E-07	1.8604E-07	2.02133E-07
Streptococcus_ratti	2.12836E-06	7.62273E-08	4.43818E-07	4.54455E-07	6.54846E-07	4.80462E-07	8.27308E-07	0.000000233	1.07145E-06	0.000000552	9.35364E-07	0.000000615	4.226E-07	3.26267E-07	3.556E-07	5.83333E-07
Streptococcus_sp._116-D4	1.22545E-07	0	0	1.21636E-07	5.95385E-08	1.22462E-07	2.63077E-08	4.32308E-08	2.96091E-07	1.40091E-07	2.32636E-07	9.25E-08	3.19933E-07	7.62E-08	0.000000274	1.534E-07
Streptococcus_sp._HSISM1	0	0	0	2.27727E-07	3.15385E-08	0	0	2.12769E-07	5.12909E-07	1.04709E-06	3.33545E-07	0	0	8.10667E-08	1.65333E-08	6.86667E-08
Streptococcus_vestibularis	8.53636E-08	0	8.75455E-09	0	3.58462E-08	1.54846E-07	0	0	0	0.000000028	1.02727E-08	0	2.72E-08	1.11467E-07	8.86667E-09	0
Streptococcus_sp._A12	5.92545E-07	2.34545E-08	2.30755E-07	3.96909E-07	0	4.30769E-08	4.40923E-07	8.70154E-07	8.57636E-07	4.49455E-07	2.13273E-07	4.68364E-07	0	6.72E-08	1.526E-07	1.86E-08
Streptococcus_sp._oral_taxon_061	6.19091E-08	0	8.75455E-09	2.41545E-08	1.03308E-07	0	5.36923E-08	5.22308E-08	0	0	6.88182E-08	3.94545E-08	1.61933E-07	1.30133E-07	2.55333E-08	3.72E-08
Streptococcus_sp._CNU_G3	0	0	2.62727E-08	0	0	0	0	0	0	0	0	0	0	0	0	0

Streptococcus_infantarius	8.32727E-08	0.000000057	4.12727E-09	0	0	0.000000256	0	0	0	0	3.33636E-08	0	4.86E-08	0	1.49333E-08	0
Streptococcus_sobrinus	2.87727E-07	1.95182E-07	2.04909E-08	2.07609E-07	2.09846E-07	3.45846E-07	7.43846E-08	7.72308E-08	1.25909E-07	6.43636E-08	0.000000345	1.45455E-07	2.77933E-07	3.89733E-07	8.444E-08	9.88667E-08
Streptococcus_mutans	1.84455E-07	7.46818E-08	1.12309E-07	1.32182E-07	2.50692E-07	2.13846E-08	2.64385E-07	1.23923E-07	4.10091E-07	1.66182E-07	4.98182E-08	2.42727E-07	2.22667E-07	2.36467E-07	3.04333E-07	9.56E-08
Streptococcus_pseudoporcinus	9.36818E-07	6.01364E-07	2.10073E-07	0.000000162	1.43077E-07	2.13846E-08	2.32231E-07	2.08615E-07	0.000000117	0.000000028	0.000000416	7.46455E-07	1.62267E-07	8.23333E-08	2.71933E-07	4.77667E-07
Streptococcus_gordonii	5.31636E-07	1.55636E-07	9.11636E-08	3.28636E-07	2.82692E-07	1.08154E-07	2.61154E-07	1.53923E-07	0.000000349	1.43527E-06	3.79091E-07	4.72455E-07	4.53267E-07	1.84887E-06	6.83533E-07	3.65533E-07
Streptococcus_pluranimalium	0.000001163	1.21518E-06	3.72455E-07	4.78909E-07	1.93462E-07	1.53231E-07	4.84769E-07	6.97154E-07	3.77364E-07	6.13636E-07	7.18818E-07	8.39727E-07	3.052E-07	5.73067E-07	4.45533E-07	4.10067E-07
Streptococcus_sp._FDAARGOS_192	0	2.74545E-08	4.12727E-09	0	0	0	3.06923E-08	0	0	0	2.05455E-08	1.42727E-08	0	0	1.49333E-08	1.98667E-08
Streptococcus_sp._LPB0220	5.57273E-08	0	1.28818E-08	7.65455E-08	0	1.21385E-07	7.66923E-08	2.47692E-08	0	8.50909E-08	1.87727E-07	0.0000001	6.51333E-08	1.57933E-07	2.99333E-08	3.52667E-08
Streptococcus_troglodytae	2.30991E-06	1.56182E-07	2.71818E-07	2.43636E-08	4.68385E-07	3.26923E-07	1.06546E-06	1.49908E-06	2.24873E-06	4.20545E-06	5.73091E-06	2.54391E-06	8.84533E-07	1.47267E-07	3.15667E-07	1.0288E-06
Streptococcus_oralis	3.14427E-06	1.20364E-07	2.11818E-07	1.96182E-07	6.72231E-07	6.30769E-08	1.65338E-06	0.000002035	2.85118E-06	4.68818E-06	2.59818E-06	2.51464E-06	9.35933E-07	3.898E-07	3.754E-07	1.41353E-06
Streptococcus_halotolerans	5.72636E-07	3.06136E-06	1.92545E-07	2.91545E-08	2.81692E-07	1.41923E-07	2.11231E-07	3.60615E-07	3.41636E-07	1.97455E-07	6.54909E-07	0.000000057	1.538E-07	2.61933E-07	8.48667E-08	2.52733E-07
Streptococcus_pneumoniae	2.53545E-07	0.000000428	1.70727E-08	3.27273E-07	6.21692E-07	8.90769E-08	2.69308E-07	0.000000194	3.30273E-07	4.02273E-07	4.64727E-07	0.000000406	1.85787E-06	8.59333E-07	8.424E-07	9.71267E-07
Streptococcus_parasanguinis	1.25364E-07	0	7.04545E-07	3.96636E-07	3.20692E-07	2.13846E-08	1.80769E-07	2.73462E-07	1.07491E-06	6.54091E-07	1.65455E-06	1.12355E-06	3.97533E-07	4.938E-07	5.46933E-07	1.22533E-07
Streptococcus_periodonticum	5.54545E-08	0	0	6.24273E-08	3.15385E-08	0	6.96923E-08	1.90769E-08	2.75455E-08	0.000000028	5.12727E-08	1.99545E-07	9.47333E-08	1.79867E-07	1.07333E-07	2.03867E-07
Streptococcus_equinus	4.31636E-07	1.03577E-06	1.41518E-07	3.14182E-07	1.15769E-07	1.19692E-07	2.32538E-07	0.000000235	3.79727E-07	6.67273E-07	5.15455E-07	5.92455E-07	1.14067E-07	2.36933E-07	4.956E-07	3.25267E-07
Streptococcus_salivarius	5.36455E-07	0.000000525	3.52727E-07	2.23727E-07	7.26923E-08	2.64462E-07	2.30385E-07	1.37308E-07	1.02636E-07	2.13273E-07	3.68727E-07	3.95636E-07	1.14467E-07	2.858E-07	1.66267E-07	3.20667E-08
Streptococcus_himalayensis	2.68909E-07	4.81409E-07	3.27273E-08	1.62273E-07	1.61615E-07	3.90615E-07	0	9.35385E-08	2.98545E-07	0.000000333	3.97273E-07	7.85455E-08	4.122E-07	4.69733E-07	2.97267E-07	2.954E-07
Streptococcus_uberis	1.32364E-07	0	1.14545E-07	1.91818E-08	2.89462E-07	0	3.11538E-08	2.58231E-07	1.50273E-07	7.29091E-08	1.57364E-07	2.03727E-07	2.11533E-07	1.534E-07	2.13107E-07	1.81267E-07
Streptococcus_merionis	5.69455E-07	8.09273E-07	7.40909E-08	4.20818E-07	6.76308E-07	5.58462E-07	0.000000474	5.68538E-07	0.00000081	9.93636E-07	2.38909E-07	6.66182E-07	1.0298E-06	8.31867E-07	7.76867E-07	6.692E-07
Streptococcus_australis	7.98182E-08	4.44545E-08	1.15036E-07	4.39091E-08	9.75385E-08	2.09154E-07	2.24385E-07	4.05154E-07	7.15455E-08	0.000000112	3.48182E-07	1.18636E-07	9.51333E-08	3.27867E-07	1.93733E-08	1.31933E-07
Streptococcus_mitis	3.28727E-07	4.18636E-08	0	4.47E-08	9.82231E-07	4.45308E-07	1.43615E-07	4.21615E-07	3.48091E-07	0.00000052	2.48182E-07	2.50909E-07	7.44733E-07	5.34867E-07	6.80467E-07	7.28667E-07
Streptococcus_parauberis	4.86818E-07	3.66682E-07	2.12545E-07	1.87455E-07	0.000001074	2.25154E-07	1.51538E-07	1.59538E-07	1.09091E-07	1.53727E-07	2.32273E-07	5.58545E-07	5.472E-07	1.24493E-06	1.16847E-06	8.97733E-07
Streptococcus_marmotae	3.54636E-07	2.63891E-06	1.10818E-07	0.000000152	2.16769E-07	1.60615E-06	2.66077E-07	9.29154E-07	4.57364E-07	3.62545E-07	5.93545E-07	2.42182E-07	4.99467E-07	3.50867E-07	5.442E-07	5.08333E-07
Streptococcus_cristatus	1.18964E-06	1.10918E-06	2.84518E-07	1.43591E-06	0.000000837	9.84308E-07	1.00292E-06	1.13569E-06	2.15336E-06	2.08564E-06	0.000001387	1.38209E-06	1.33387E-06	1.12313E-06	1.57533E-06	1.8528E-06
Streptococcus_intermedius	3.40373E-06	1.40464E-06	1.7323E-06	1.69364E-06	5.29769E-07	2.86923E-07	3.19831E-06	2.69377E-06	3.25336E-06	1.51909E-06	2.50018E-06	2.12009E-06	4.30867E-07	1.2572E-06	5.24933E-07	5.094E-07
Streptococcus_iniae	1.09273E-06	4.80909E-08	7.12727E-08	8.23636E-08	6.80308E-07	1.72154E-07	6.36231E-07	1.25246E-06	1.71627E-06	2.21191E-06	3.53473E-06	1.70855E-06	8.396E-07	5.89333E-07	6.67467E-07	5.77067E-07
Streptococcus_lutetiensis	6.08182E-08	0	5.39727E-08	0	4.30846E-07	2.26615E-07	1.36538E-07	7.15385E-07	1.53182E-07	2.13727E-07	3.79091E-08	3.57273E-07	4.84067E-07	5.71267E-07	6.24667E-07	5.63867E-07
Streptococcus_respiraculi	0.000004224	3.76618E-06	9.00727E-07	1.30582E-06	1.54554E-06	5.13985E-06	3.79931E-06	3.25292E-06	3.39618E-06	4.84718E-06	5.19955E-06	4.38364E-06	2.32307E-06	0.000002076	1.3924E-06	2.98973E-06
Streptococcus_dysgalactiae	4.17955E-06	8.83336E-06	1.07109E-06	6.37427E-06	0.0000033	5.07462E-06	0.000003353	4.35492E-06	4.44582E-06	2.51845E-06	3.91273E-06	7.44909E-06	3.14473E-06	4.93667E-06	2.87093E-06	3.03033E-06
Streptococcus_equi	3.16636E-06	9.41182E-07	6.18136E-07	1.36091E-06	2.03162E-06	1.45869E-06	1.75392E-06	3.47223E-06	3.00627E-06	4.53473E-06	3.99545E-06	0.000004052	1.7488E-06	2.3076E-06	1.83827E-06	0.00000187
Streptococcus_porcinus	5.85091E-07	1.05727E-07	1.15545E-07	6.30818E-07	9.92769E-07	3.72154E-07	4.34923E-07	4.29846E-07	3.50273E-07	2.81727E-07	9.58545E-07	1.25827E-06	8.182E-07	9.528E-07	1.31293E-06	0.000001707
Streptococcus_sanguinis	2.61636E-06	4.25955E-06	1.04282E-06	2.66045E-06	2.12285E-06	1.60108E-06	1.47392E-06	2.77862E-06	3.13727E-06	2.07355E-06	0.00000231	2.62009E-06	1.7048E-06	2.06113E-06	2.0776E-06	1.98007E-06
Streptococcus_gallolyticus	0.00000834	2.69455E-06	2.10821E-06	3.20155E-06	2.53077E-06	1.99969E-06	5.12908E-06	0.00000455	6.57455E-06	1.00027E-05	1.10292E-05	0.00000938	2.50227E-06	2.47107E-06	1.94453E-06	2.84027E-06
Streptococcus_canis	2.26273E-06	2.29636E-06	5.14364E-07	1.50427E-06	1.68838E-06	1.96254E-05	1.79292E-06	1.73277E-06	0.0000018	1.75909E-06	2.59836E-06	1.72427E-06	1.8404E-06	2.14927E-06	1.30033E-06	2.21467E-06
Streptococcus_thermophilus	2.13909E-06	2.52827E-06	1.47866E-06	1.80082E-06	2.06538E-06	6.35577E-06	8.05615E-07	1.67715E-06	1.76627E-06	7.56091E-07	1.48291E-06	2.64364E-06	3.53133E-06	2.8224E-06	2.5344E-06	4.54707E-06
Streptococcus_agalactiae	8.06545E-06	7.60973E-06	3.01818E-06	5.75791E-06	5.96385E-06	2.11111E-05	6.69538E-06	5.79838E-06	7.56945E-06	5.01455E-06	0.0000098	7.83182E-06	5.57533E-06	7.4064E-06	7.02987E-06	4.45667E-06
Streptococcus_pasteurianus	2.25527E-06	1.60118E-06	1.49282E-06	3.06136E-06	4.22938E-06	2.54015E-06	1.55492E-06	2.03462E-06	1.52382E-06	1.87673E-06	2.00245E-06	3.47673E-06	2.8752E-06	3.22287E-06	3.60113E-06	2.05807E-06
Streptococcus_sp._NSJ-72	0.000000286	1.68182E-08	4.12182E-08	3.36455E-07	0.000000557	0	8.13308E-07	3.86769E-07	5.97545E-07	8.94818E-07	4.35364E-07	6.68636E-07	2.898E-07	1.06987E-06	2.096E-07	3.562E-07
Streptococcus_sp._DAT741	1.11182E-07	4.73591E-07	1.53527E-07	4.46427E-07	0	2.62308E-07	1.57462E-07	1.25615E-07	1.13182E-07	2.23818E-07	2.18091E-07	9.25E-08	2.24E-08	6.57333E-08	2.24667E-08	0.000000057
Streptococcus_sp._Z15	1.34827E-06	5.64636E-07	1.25109E-07	1.08664E-06	4.90231E-07	5.69538E-07	3.11538E-08	4.55308E-07	7.79818E-07	4.98818E-07	1.00391E-06	8.82909E-07	6.67667E-07	7.772E-07	4.85067E-07	6.06333E-07
Streptococcus_sp._KS_6	2.05391E-06	1.65727E-06	6.71909E-07	1.70291E-06	1.24892E-06	7.15231E-07	0.00000227	9.70846E-07	1.64345E-06	1.09227E-06	1.00245E-06	1.36327E-06	4.25533E-07	1.21107E-06	7.67333E-07	8.69733E-07
Streptococcus_pyogenes	5.91273E-06	1.10487E-05	4.85566E-06	1.02864E-05	6.86023E-06	6.97654E-06	8.71231E-06	7.11923E-06	9.99364E-06	1.13718E-05	7.98091E-06	1.86082E-05	0.000005128	0.000007216	6.52653E-06	6.72467E-06
Streptococcus_pantholopis	4.32273E-06	7.61909E-07	4.65273E-07	1.81545E-06	1.32515E-06	1.37769E-06	4.00738E-06	0.000002524	4.03909E-06	7.37636E-06	6.61473E-06	3.66773E-06	1.3456E-06	1.9708E-06	1.38747E-06	2.05667E-06
Streptococcus_suis	1.24645E-05	8.75127E-06	0.000004293	1.31509E-05	1.03585E-05	1.25531E-05	1.14838E-05	1.01415E-05	1.05536E-05	1.41591E-05	1.27264E-05	1.34327E-05	0.00000905	1.23287E-05	1.01133E-05	1.09027E-05
Streptococcus_acidominimus	3.36727E-05	6.36445E-05	1.35083E-05	7.36551E-05	3.52546E-05	2.32785E-05	3.50978E-05	4.60607E-05	4.10591E-05	4.12636E-05	0.000040472	5.31039E-05	0.000027223	4.51325E-05	2.99367E-05	2.55747E-05
Melissococcus_plutonius	2.96364E-08	3.29091E-08	1.90909E-07	1.34455E-07	1.98615E-07	2.71308E-07	3.85692E-07	3.60077E-07	1.12545E-07	1.83636E-07	4.48182E-07	4.21182E-07	1.568E-07	3.59867E-07	2.96667E-07	2.778E-07
Vagococcus_sp._MN-17	3.11818E-08	0	0	2.66091E-07	0	0	2.63077E-08	5.22308E-08	0	0	6.88182E-08	1.31636E-07	0	0	2.38533E-07	0
Vagococcus_sp._HDW17B	3.55182E-07	1.35636E-07	0.000000199	2.23182E-07	0	2.34692E-07	4.33846E-08	5.69231E-08	5.82182E-07	1.53382E-06	1.05636E-07	0.000000343	3.88067E-07	1.65133E-07	1.376E-07	4.57933E-07
Vagococcus_sp._HDW17A	6.87273E-08	1.19864E-07	1.91218E-07	6.64545E-08	2.59231E-08	1.47692E-07	1.73846E-07	0	1.04909E-07	1.13909E-07	3.81636E-07	0.000000134	0	0	1.68133E-07	1.98667E-08
Vagococcus_sp._CF-49	2.33727E-07	0	6.67273E-08	8.25455E-08	2.78385E-07	2.56769E-07	0	2.09231E-07	3.33182E-07	1.11273E-07	0.000000668	1.93909E-07	6.63333E-08	1.88667E-07	0.000000665	0.000000332
Vagococcus_teuberi	0.000000092	1.38864E-07	4.12727E-09	2.04727E-07	6.69231E-08	0	1.05385E-07	2.30231E-07	2.87636E-07	2.20091E-07	0.00000031	1.93455E-07	0.000000075	1.75733E-07	1.12573E-07	1.17267E-07
Vagococcus_penaei	3.72909E-07	0.000000065	2.36636E-07	4.10636E-08	4.04923E-07	0	0	3.69077E-07	3.15636E-07	0.000000062	3.56818E-07	4.44818E-07	3.98267E-07	6.74867E-07	4.60533E-07	1.0054E-06
Vagococcus_carniphilus	2.86182E-07	3.68182E-08	7.49818E-08	2.89455E-07	4.87385E-07	2.47846E-07	1.94154E-07	5.72154E-07	0.000000615	4.24364E-07	1.75627E-06	4.71818E-07	5.67067E-07	1.04373E-06	4.16533E-07	2.916E-07
Tetragenococcus_osmophilus	1.88091E-07	0	8.75455E-09	0	0.000000065	0	0	1.84154E-07	0	3.89091E-08	7.58182E-08	7.13636E-09	1.64067E-07	2.73267E-07	1.65333E-07	3.15067E-07
Tetragenococcus_koreensis	3.49755E-06	1.66545E-06	5.89364E-07	1.43873E-06	1.05762E-06	1.37185E-06	2.52415E-06	1.23723E-06	1.32045E-06	1.19618E-06	5.19545E-07	1.70655E-06	6.308E-07	9.03133E-07	6.732E-07	7.36267E-07
Tetragenococcus_halophilus	7.77727E-07	3.77318E-07	6.07273E-07	1.10055E-06	1.82531E-06	9.25462E-07	1.10323E-06	2.50246E-06	4.89273E-07	7.97636E-07	1.43464E-06	1.55364E-06	1.8046E-06	2.78927E-06	3.54627E-06	3.91127E-06
Enterococcus_sp._DA9	0	2.56818E-08	8.75455E-09	0	3.34615E-08	0	0	0	0	5.22727E-08	1.35636E-07	2.85455E-08	0	3.50667E-08	0	0
Enterococcus_sp._FDAARGOS_375	3.11818E-08	0	0.00000021	2.42609E-07	1.45077E-07	1.83876E-05	0	5.41538E-08	0	3.03636E-08	6.68182E-08	7.13636E-09	1.304E-07	0.00000008	6.46667E-08	1.19333E-07
Enterococcus_thailandicus	1.55564E-06	4.71364E-07	3.63091E-07	1.40518E-07	4.06462E-07	4.42146E-06	1.00469E-06	1.15408E-06	1.52255E-06	3.49109E-06	3.48036E-06	1.63545E-06	6.712E-07	6.07067E-07	4.762E-07	1.00933E-06
Enterococcus_sp._CR-Ec1	2.49091E-07	1.53345E-06	0.000000438	5.26545E-07	0	0.000010726	7.07692E-08	3.07154E-07	0.000000233	1.64273E-07	3.60636E-07	1.62545E-07	1.39133E-07	3.50667E-08	0	0
Enterococcus_sp._HSIEG1	0	0.000031755	8.07482E-06	5.63455E-06	0	0.000242874	0	0	2.72727E-08	4.80909E-07	0	1.42727E-08	6.08E-08	1.61333E-08	6.67333E-08	1.392E-07
Enterococcus_gallinarum	1.89636E-07	0.000236941	5.82345E-05	4.51249E-05	9.07077E-07	0.001890182	3.97385E-07	8.85308E-07	3.29182E-07	6.69782E-06	3.45455E-07	8.99818E-07	6.18067E-07	5.964E-07	4.24467E-07	3.05867E-07
Enterococcus_durans	0.000002561	3.23745E-06	1.59591E-06	1.29927E-06	2.18269E-06	2.41601E-05	1.09331E-06	0.000002272	0.000000786	1.09445E-06	0.000000949	3.79091E-06	4.11067E-07	1.02173E-06	9.36667E-07	3.60867E-07
Enterococcus_cecorum	3.46755E-06	2.55618E-06	1.12148E-06	1.23463E-05	0.00000113	1.51308E-05	1.24438E-06	3.78669E-06	1.58492E-05	5.08025E-05	1.35106E-05	6.29409E-06	0.00000131	2.21587E-06	3.26013E-06	1.9524E-06
Enterococcus_sp._FDAARGOS_553	3.11818E-08	6.75727E-06	1.33273E-06	1.32655E-06	0	5.77919E-05	3.33077E-08	1.90769E-08	0	6.37273E-08	0	0	2.14667E-08	3.48E-08	0	0
Enterococcus_sp._M190262	2.18091E-07	0.000496218	0.000130068	9.24492E-05	4.80462E-07	0.004140283	1.26885E-06	1.43554E-06	3.07455E-07	1.05568E-05	7.46364E-07	5.46909E-07	8.518E-07	5.704E-07	5.51467E-07	5.20267E-07
Enterococcus_wangshanyuanii	6.60455E-07	1.89023E-06	1.06391E-06	2.20809E-06	1.49938E-06	1.27723E-05	1.25238E-06	1.66108E-06	5.77545E-07	6.93636E-07	1.42464E-06	1.77509E-06	9.584E-07	1.07033E-06	0.000002572	2.87167E-06
Enterococcus_gilvus	4.95182E-07	1.00127E-06	1.34227E-07	0.000000736	5.27385E-07	1.39546E-06	5.66462E-07	7.11231E-07	4.12455E-07	3.53364E-07	6.72636E-07	0.000001226	4.93667E-07	9.596E-07	8.03667E-07	7.854E-07
Enterococcus_avium	2.25645E-06	1.16618E-06	8.10318E-07	1.89818E-06	1.95246E-06	3.87331E-06	1.74123E-06	1.72654E-06	2.42909E-06	1.84273E-06	1.30309E-06	1.44355E-06	1.14793E-06	1.63387E-06	1.77667E-06	2.0308E-06
Enterococcus_mundtii	1.24391E-05	2.92055E-06	4.46991E-06	1.11227E-05	2.95785E-06	3.53592E-05	1.26523E-05	1.01883E-05	5.85373E-06	1.11655E-05	6.20091E-06	5.42418E-06	2.77427E-06	3.43947E-06	3.82387E-06	3.01273E-06
Enterococcus_casseliflavus	5.51064E-06	6.06494E-05	6.79482E-06	7.35991E-06	3.06615E-06	0.000467636	2.86423E-06	3.34362E-06	7.62636E-06	7.87273E-06	4.02627E-06	5.00727E-06	2.8942E-06	4.38953E-06	2.52113E-06	2.7028E-06
Enterococcus_hirae	3.77364E-07	4.66409E-07	1.52818E-07	0.000000141	1.66792E-06	1.21046E-06	0.00000046	8.60462E-07	6.93909E-07	2.76818E-07	4.97545E-07	1.19973E-06	1.13893E-06	1.43147E-06	1.73213E-06	1.06527E-06
Enterococcus_saigonensis	2.99227E-05	1.37125E-05	1.01536E-05	0.00002928	1.63254E-05	5.64496E-05	2.10423E-05	2.05892E-05	2.58473E-05	2.67636E-05	2.23582E-05	2.95273E-05	0.000010172	1.32407E-05	1.25113E-05	9.22933E-06
Enterococcus_faecium	0.000311371	0.000331392	0.000104483	0.00019323	8.39727E-05	0.000247483	0.00014095	0.000166475	0.000176505	0.000266836	0.000184623	0.000314605	9.96899E-05	0.000132704	0.000126854	9.60515E-05
Enterococcus_faecalis	0.000527149	0.003445864	0.000159144	0.000763956	0.000601636	0.000636039	0.000342345	0.000380935	0.000780302	0.000971931	0.000577286	0.000876066	0.000498766	0.000661656	0.00062238	0.000452912
Lactobacillus_bombi	7.50909E-08	6.30455E-08	4.10909E-08	1.22727E-07	5.47692E-08	9.26154E-08	2.54385E-07	1.06308E-07	3.31636E-07	3.52727E-07	2.83182E-07	0.000000187	2.39267E-07	6.52E-08	2.99333E-08	2.81333E-07
Lactobacillus_hordei	6.27273E-08	0	0	8.88818E-08	1.12231E-07	0.0000002	0	3.37692E-08	0	0.000000264	1.35636E-07	5.37273E-08	9.32667E-08	0.00000008	4.44E-09	3.67333E-08
Lactobacillus_mali	4.47909E-07	0.000000157	8.21818E-08	1.47273E-08	2.50769E-07	1.29462E-07	1.38231E-07	8.18231E-07	5.73909E-07	6.48182E-08	4.60455E-07	3.38364E-07	2.502E-07	3.20733E-07	4.744E-07	0.000000054
Liquorilactobacillus_nagelii	1.51718E-06	1.84391E-06	3.69636E-07	1.37818E-06	1.69769E-07	2.16923E-08	0	2.83077E-07	3.17764E-06	2.93027E-06	1.05827E-06	8.86636E-07	3.42533E-07	2.35267E-07	4.42933E-07	4.452E-07
Lactobacillus_oligofermentans	0	0	0	0	2.52923E-07	4.13846E-08	0	1.08538E-07	1.61909E-07	0.000000034	2.76364E-08	7.13636E-09	6.08E-08	1.25667E-07	1.774E-07	2.872E-07
Lactobacillus_nenjiangensis	4.43545E-07	0	0	8.27273E-08	9.84154E-07	4.40923E-07	8.10769E-07	4.83308E-07	6.41364E-07	0.000000536	3.90909E-07	5.61273E-07	1.09893E-06	2.60267E-07	0.000000859	9.328E-07
Lactobacillus_hokkaidonensis	9.71291E-06	1.02091E-07	7.45455E-07	2.07273E-08	2.25354E-06	1.72923E-06	5.21277E-06	0.000003154	1.10374E-05	2.31973E-05	9.17445E-06	5.33155E-06	2.9554E-06	1.57807E-06	1.58093E-06	4.8012E-06
Lactobacillus_kefiri	1.18182E-07	1.34545E-07	0	9.72727E-08	8.55385E-08	7.26923E-08	2.63077E-08	0	1.78273E-07	0	1.02727E-08	2.14545E-08	1.21133E-07	0.000000055	1.12667E-08	8.62667E-08
Lactobacillus_hilgardii	1.00727E-07	0	6.67273E-08	1.95455E-08	1.59615E-07	4.40923E-07	1.16769E-07	1.41769E-07	3.94545E-08	9.49091E-08	4.61364E-07	0.000000068	5.08E-08	9.48E-08	1.28973E-07	1.446E-07
Lactobacillus_parabuchneri	0	0.000000047	5.11273E-08	2.93364E-08	0	0	0	4.67692E-08	3.94545E-08	0	3.95455E-08	1.42727E-08	2.24E-08	3.09333E-08	0	1.664E-07
Lactobacillus_buchneri	1.54455E-07	1.77273E-08	8.31818E-09	0.000000204	1.28846E-07	0	6.40769E-08	2.47692E-08	7.18182E-08	3.89091E-08	0	2.13091E-07	7.58E-08	6.76E-08	5.02667E-08	1.63467E-07
Lactobacillus_curieae	1.15909E-07	0.000000065	1.99091E-07	2.25545E-07	1.06077E-07	0	3.77692E-08	8.21538E-08	1.02364E-07	1.71182E-07	0.000000215	2.69409E-07	0.0000002	1.77933E-07	2.38E-08	9.43333E-08
Lactobacillus_kunkeei	6.10545E-07	0.00000026	1.09636E-07	3.38155E-07	2.76846E-07	0	3.30769E-08	6.72077E-07	8.54545E-07	4.02909E-07	8.69273E-07	6.99455E-07	4.54267E-07	1.94267E-07	5.21467E-07	3.50867E-07
Lactobacillus_rossiae	2.43691E-06	4.80909E-08	2.43364E-07	2.08818E-07	7.48769E-07	4.36846E-07	2.01438E-06	0.000001545	0.000001637	3.99573E-06	0.000004128	1.58745E-06	1.33007E-06	0.000000545	6.84333E-07	1.7366E-06
Pediococcus_inopinatus	1.99091E-07	5.60273E-07	0.000000047	1.61909E-07	0	0.00000006	2.63077E-08	8.02308E-08	0	0	1.04545E-07	4.50955E-07	2.24E-08	3.09333E-08	4.44E-09	9.41333E-08
Pediococcus_claussenii	1.58182E-08	0	9.60909E-08	1.43636E-08	3.15385E-08	2.33308E-07	0	0	2.72727E-08	8.26364E-08	4.92727E-08	8.73636E-08	8.95333E-08	1.43667E-07	4.44E-09	5.88E-08
Pediococcus_pentosaceus	0.000000694	7.10909E-08	9.84909E-08	1.17455E-07	1.07992E-06	8.08231E-07	5.67231E-07	4.33923E-07	1.01073E-06	6.12182E-07	9.78909E-07	7.66727E-07	8.804E-07	3.91867E-07	6.56267E-07	8.22467E-07
Pediococcus_acidilactici	5.37818E-07	5.50545E-07	1.04709E-07	1.46273E-07	7.86077E-07	1.07462E-06	3.20923E-07	3.72462E-07	3.89909E-07	1.91455E-07	9.92727E-08	2.85455E-08	2.172E-07	6.84133E-07	5.292E-07	2.198E-07
Pediococcus_damnosus	3.74091E-07	1.40909E-07	1.11818E-07	0.000000593	0	0	2.06923E-07	2.93846E-08	5.60909E-07	0.000000373	4.62091E-07	4.80636E-07	2.25467E-07	3.48E-08	2.54E-08	1.732E-07
Lactobacillus_paracollinoides	7.71364E-07	2.32727E-08	1.60664E-06	2.00364E-06	2.70846E-07	1.30769E-07	4.27846E-06	5.17615E-06	1.01064E-06	3.56636E-07	4.02455E-07	2.59818E-07	6.35933E-07	3.65867E-07	5.85333E-07	2.576E-07
Lactobacillus_malefermentans	5.82727E-08	1.68182E-08	1.15073E-07	1.44182E-07	7.46923E-08	0.000000026	5.33615E-07	4.07923E-07	0	3.62727E-08	9.48182E-08	1.50273E-07	2.44267E-07	7.62867E-07	5.366E-07	8.40667E-08
Lactobacillus_harbinensis	0.000001138	2.47455E-07	1.71073E-07	9.43636E-07	1.66731E-06	1.20223E-06	5.68077E-07	7.15308E-07	5.16818E-07	2.84273E-07	4.46182E-07	1.99455E-07	1.72947E-06	2.38033E-06	2.5384E-06	0.000002262
Amylolactobacillus_amylophilus	2.96364E-08	0	4.24545E-08	1.85273E-07	1.28692E-07	1.26769E-07	3.77692E-08	1.97308E-07	1.81727E-07	2.82818E-07	2.27364E-07	1.39455E-07	1.08333E-07	3.156E-07	2.48133E-07	4.16067E-07
Lactobacillus_fuchuensis	4.61364E-07	7.54545E-08	0	0.000000516	3.38769E-07	6.78462E-08	2.09538E-07	2.05385E-07	2.39182E-07	1.66455E-07	1.54364E-07	4.75273E-07	5.07067E-07	2.64067E-07	2.94133E-07	8.02867E-07
Lactobacillus_curvatus	0.000000061	3.23182E-08	2.89545E-08	7.75455E-08	4.86154E-08	2.32308E-07	1.11846E-07	6.41231E-07	2.72727E-08	3.62727E-08	1.61091E-07	2.95455E-07	1.07267E-07	9.64667E-08	0.000000198	4.97333E-08
Lactobacillus_graminis	1.52091E-07	9.75455E-08	1.01364E-07	2.14727E-07	3.34615E-08	5.16615E-07	1.08538E-07	7.92308E-08	1.88091E-07	5.22727E-08	3.74273E-07	0.000000173	4.29333E-08	1.24667E-07	1.47773E-07	1.76733E-07
Lactobacillus_sakei	2.84545E-07	1.14545E-07	1.63636E-08	4.40545E-07	5.16769E-07	4.21769E-07	5.26154E-07	6.86385E-07	3.28909E-07	1.95091E-07	4.14364E-07	1.00018E-06	1.586E-07	2.93533E-07	2.23867E-07	1.72133E-07
Lactiplantibacillus_plantarum	1.35545E-06	1.76818E-07	6.90755E-07	1.04518E-06	5.74077E-07	1.10763E-05	1.07808E-06	9.86846E-07	1.37955E-06	8.17909E-07	1.50864E-06	9.50182E-07	1.0426E-06	7.47667E-07	6.51533E-07	8.56733E-07
Lactobacillus_pentosus	1.29691E-06	3.87273E-07	0.000000468	5.94636E-07	5.80923E-07	2.37769E-07	5.53846E-07	4.81846E-07	6.44636E-07	5.49182E-07	8.59909E-07	1.28827E-06	4.77867E-07	3.40933E-07	3.75533E-07	5.01667E-07
Lactobacillus_paraplantarum	1.02818E-07	0	0	0	0.000000151	6.73846E-08	1.23923E-07	0.000000099	1.90636E-07	0.000000028	1.58545E-07	7.71364E-08	1.016E-07	1.75333E-07	6.91067E-08	9.33333E-09
Lactobacillus_sanfranciscensis	1.69273E-07	1.16364E-08	3.70909E-08	0.000000313	3.83769E-07	1.66923E-07	1.20385E-07	2.70077E-07	1.02355E-06	2.91182E-07	4.44455E-07	1.86091E-07	4.73067E-07	3.114E-07	2.08933E-07	1.79133E-07
Lactobacillus_fructivorans	7.93091E-07	7.82182E-07	1.70309E-07	1.25755E-06	4.22462E-07	3.49462E-07	1.34492E-06	2.32923E-07	6.84818E-07	2.88818E-07	1.15927E-06	5.81909E-07	5.994E-07	0.000000943	4.79933E-07	2.678E-07
Lactobacillus_lindneri	0.000000192	1.16364E-08	0	1.74545E-07	3.29615E-07	2.13846E-08	4.33846E-08	2.57154E-07	2.25273E-07	3.82091E-07	3.52091E-07	5.79545E-07	3.17933E-07	3.68333E-07	3.424E-07	4.19267E-07
Lacticaseibacillus_paracasei	1.58636E-07	0.000000065	1.10909E-07	1.84273E-07	3.01769E-07	7.08154E-07	1.18077E-07	3.22462E-07	1.30182E-07	1.93364E-07	3.37545E-07	2.24636E-07	4.35733E-07	6.63267E-07	8.79867E-07	8.58333E-07
Lactobacillus_casei	9.86364E-08	7.40909E-08	1.15073E-07	1.91818E-08	2.90692E-07	0.00000051	1.97231E-07	8.16923E-08	0.000000217	1.04545E-07	3.82182E-07	4.19909E-07	1.19667E-07	1.11267E-07	1.278E-07	2.60133E-07
Lactobacillus_manihotivorans	1.05018E-05	7.96273E-06	2.89027E-06	7.13818E-06	6.37623E-06	0.000011616	6.69623E-06	7.95923E-06	4.20273E-06	6.00982E-06	4.88173E-06	8.10982E-06	2.3742E-06	2.82987E-06	3.8336E-06	2.2438E-06
Lactobacillus_rhamnosus	5.24545E-07	1.77818E-07	1.63636E-08	6.91727E-07	1.23292E-06	1.27231E-06	8.85308E-07	4.23154E-07	0.000001	7.59182E-07	1.14782E-06	1.13164E-06	1.37547E-06	1.18547E-06	1.7564E-06	2.1716E-06
Lactobacillus_ginsenosidimutans	5.27273E-08	1.03636E-08	0	4.79091E-09	0.000000104	4.39231E-08	0	5.74615E-08	6.45455E-08	0	3.33636E-08	6.41364E-08	1.022E-07	6.60667E-08	1.49933E-07	1.54867E-07
Lactobacillus_allii	5.91818E-07	3.24545E-07	1.06818E-07	2.22545E-07	4.76846E-07	2.52308E-08	0	0.000000087	1.71273E-07	1.22182E-07	0.000000039	1.75364E-07	2.09667E-07	1.964E-07	0.00000028	2.16667E-07
Lactobacillus_farciminis	1.39718E-06	0	0	5.18182E-09	1.19085E-06	1.76077E-06	9.25077E-07	0.000000373	8.67727E-07	1.97273E-07	3.89364E-07	1.74309E-06	1.52433E-06	6.54067E-07	0.000000929	1.36073E-06
Lactobacillus_crustorum	2.95545E-07	8.87273E-08	4.12727E-09	9.66091E-08	3.74462E-07	3.30154E-07	6.51538E-08	1.36308E-07	2.30364E-07	3.29091E-07	2.91273E-07	6.72182E-07	2.042E-07	4.196E-07	4.116E-07	1.56933E-07
Lactobacillus_zhachilii	0.000001177	0	1.51636E-07	1.59091E-07	1.47115E-06	6.94077E-07	7.66769E-07	1.04008E-06	0.000000919	7.96727E-07	0.000000962	4.70091E-07	2.31033E-06	1.17633E-06	1.45447E-06	1.46493E-06
Lactobacillus_heilongjiangensis	1.06545E-06	0	4.12727E-09	9.97273E-09	7.66231E-07	6.10769E-07	6.54846E-07	3.74077E-07	5.47091E-07	4.76273E-07	4.90909E-07	5.92682E-07	1.37927E-06	9.842E-07	0.00000087	8.53533E-07
Lactobacillus_alimentarius	4.02636E-07	0	0	0	3.50538E-07	2.99154E-07	2.29846E-07	5.54769E-07	1.09727E-07	2.86455E-07	2.82273E-07	1.00364E-07	3.11667E-07	3.30067E-07	3.99867E-07	0.000000529
Lactobacillus_futsaii	3.90909E-07	0	2.88545E-08	4.07E-08	6.33923E-07	1.34154E-07	1.67923E-07	2.42692E-07	2.88273E-07	1.62909E-07	4.04818E-07	5.74455E-07	4.888E-07	0.000000237	2.918E-07	3.064E-07
Lactobacillus_paralimentarius	1.94455E-07	8.58182E-08	9.36364E-08	4.79091E-09	0.000000153	7.92308E-08	5.26154E-08	4.64615E-08	1.09091E-07	0	6.00909E-08	4.65909E-08	2.36333E-07	1.48467E-07	1.35267E-07	4.074E-07
Acetilactobacillus_jinshanensis	5.36909E-06	3.24545E-07	2.15049E-06	3.85455E-06	3.77846E-06	9.06154E-08	5.08885E-06	3.41946E-06	2.68282E-06	3.90145E-06	0.00000535	5.62409E-06	2.20353E-06	4.16267E-06	3.5144E-06	0.000002416
Lactobacillus_backii	2.71818E-07	0	8.25455E-09	1.51727E-07	1.90462E-07	1.31538E-07	5.05538E-07	2.88692E-07	0.000000054	0.000000241	5.53273E-07	2.69909E-07	4.90467E-07	2.468E-07	1.23733E-07	4.788E-07
Lactobacillus_bifermentans	1.06036E-06	0.000000065	2.62336E-07	1.23818E-07	0.000000697	1.53046E-06	1.12585E-06	0.000000765	1.50445E-06	1.18645E-06	9.23364E-07	9.18182E-07	1.32153E-06	5.874E-07	1.02333E-06	1.0982E-06
Lactobacillus_coryniformis	1.52136E-06	2.08018E-06	7.77273E-07	2.79873E-06	2.30485E-06	7.46923E-08	9.71077E-07	1.97815E-06	1.82673E-06	1.27027E-06	1.54718E-06	3.50318E-06	2.06227E-06	1.99373E-06	2.9472E-06	0.000005642
Lactobacillus_zymae	4.88091E-07	8.84545E-08	7.80727E-08	1.16182E-07	1.16769E-07	5.13769E-07	4.34923E-07	2.29538E-07	1.24091E-07	1.90455E-07	2.25182E-07	3.18364E-07	1.19867E-07	1.43533E-07	2.906E-07	3.65733E-07
Lactobacillus_suantsaii	2.88182E-07	3.26909E-07	3.29818E-08	6.46364E-08	9.33077E-08	4.24846E-07	2.10385E-07	4.34846E-07	1.40273E-07	6.74545E-08	0.000000448	0.000001478	0.000000185	2.36733E-07	2.7604E-07	4.774E-07
Lactobacillus_koreensis	3.65455E-07	1.62318E-07	1.33182E-07	4.46909E-07	1.99769E-07	4.13846E-08	4.41462E-07	1.34308E-07	4.24909E-07	0	5.87091E-07	7.28773E-07	9.48E-08	2.19867E-07	2.006E-07	1.11533E-07
Lactobacillus_brevis	6.49818E-06	1.33618E-06	1.39035E-06	2.56755E-06	5.52131E-06	2.53589E-05	0.00000712	3.69615E-06	7.56855E-06	4.62991E-06	6.62991E-06	8.39427E-06	5.53693E-06	5.45433E-06	1.06513E-05	9.62133E-06
Lactobacillus_gastricus	1.41727E-07	0	0	6.31818E-08	2.88462E-08	8.35385E-08	1.02846E-07	0	5.48182E-08	1.16636E-07	2.61818E-07	8.87273E-08	5.34667E-08	2.62E-08	3.30667E-08	1.266E-07
Lactobacillus_vaginalis	6.15636E-07	2.09727E-07	4.25845E-07	1.81182E-07	4.31231E-07	2.51592E-06	3.59231E-07	2.99154E-07	4.85455E-07	4.36909E-07	4.88091E-07	2.61045E-07	5.332E-07	4.51533E-07	3.228E-07	4.17267E-07
Lactobacillus_pontis	0.000000573	2.41545E-07	7.40909E-08	9.64818E-07	1.45385E-07	1.26077E-07	1.33692E-07	2.97769E-07	1.94182E-07	3.00818E-07	4.24727E-07	3.32636E-07	0.000000271	1.96267E-07	0.00000039	1.91933E-07
Lactobacillus_mucosae	1.94918E-06	4.41845E-06	3.20909E-07	0.000000262	1.40131E-06	1.16331E-06	2.56462E-07	8.73462E-07	1.02564E-06	9.52273E-07	1.02536E-06	1.65164E-06	1.43393E-06	1.12373E-06	9.41667E-07	1.55587E-06
Limosilactobacillus_fermentum	2.21927E-06	1.54373E-06	0.000000633	1.60364E-06	1.07531E-06	1.20335E-05	0.00000184	1.60308E-06	1.82173E-06	0.00000088	2.01155E-06	2.76973E-06	1.59067E-06	2.24867E-06	2.4096E-06	1.56653E-06
Lactobacillus_frumenti	5.54091E-07	0.000000671	1.15955E-07	5.45455E-07	2.63462E-07	7.54462E-07	6.20308E-07	4.57385E-07	1.45636E-07	2.84909E-07	3.84091E-07	1.41364E-07	3.94267E-07	4.62467E-07	2.06733E-07	1.728E-07
Limosilactobacillus_reuteri	0.003022578	0.004264291	0.001378114	0.002262241	0.004550351	0.023368223	0.002256285	0.001897837	0.002638955	0.003230416	0.002232982	0.002340682	0.003737638	0.001966527	0.002146015	0.002655982
Lactobacillus_sp._JM1	0	7.74545E-08	3.27273E-08	0	0	0	0	0	0	0	0	7.13636E-09	0	0	0	0
Lactobacillus_helsingborgensis	9.37818E-07	3.53636E-07	2.96364E-08	1.93727E-07	1.02438E-06	4.11538E-07	7.73385E-07	4.69692E-07	6.98909E-07	4.81727E-07	1.10627E-06	1.17209E-06	1.0696E-06	6.75867E-07	9.93333E-07	1.41953E-06

Lactobacillus_sp._BHWM-4	1.43364E-07	1.84091E-08	8.40909E-08	5.18182E-09	3.58462E-08	0	7.26154E-07	2.50231E-07	6.48182E-08	7.51818E-08	1.35545E-07	0.000000411	1.188E-07	1.65133E-07	3.35533E-07	3.36733E-07
Lactobacillus_terrae	2.07273E-07	5.05455E-08	1.21182E-07	3.07273E-08	2.84769E-07	6.93846E-08	3.11538E-08	3.00231E-07	4.62182E-07	2.93273E-07	1.61118E-06	1.18627E-06	2.31733E-07	9.46667E-08	3.33933E-07	6.52867E-07
Lactobacillus_kullabergensis	4.11318E-06	7.33182E-07	1.29982E-07	2.13727E-07	2.70869E-06	1.84077E-06	1.75223E-06	1.20654E-06	1.37164E-06	1.16064E-06	1.31636E-06	2.28545E-06	3.6864E-06	1.73827E-06	2.7168E-06	2.92213E-06
Lactobacillus_sp._NFFJ11	8.23636E-08	0	2.46182E-08	5.50909E-08	0.000000088	1.79231E-08	0	1.41846E-07	1.46727E-07	3.03636E-08	3.22727E-07	0	9.32667E-08	1.10533E-07	9.03333E-08	2.118E-07
Lactobacillus_sp._CBA3605	8.49091E-08	1.37273E-08	3.78909E-08	5.46364E-08	5.63846E-08	9.89231E-08	6.84615E-08	2.59538E-07	1.33545E-07	3.60455E-07	6.15909E-07	1.65318E-07	9.58E-08	6.60667E-08	8.95333E-08	1.18333E-07
Lactobacillus_sp._3B(2020)	9.86818E-07	7.64273E-07	4.13182E-07	3.90818E-07	7.06308E-07	4.02146E-06	7.38385E-07	3.83154E-07	7.05455E-07	5.90273E-07	2.59455E-07	5.91545E-07	1.0964E-06	3.64667E-07	5.484E-07	7.188E-07
Lactobacillus_delbrueckii	0.00000608	1.91436E-06	8.42909E-07	2.58718E-06	3.62846E-06	4.66154E-06	2.49446E-06	2.38631E-06	4.09891E-06	2.76009E-06	3.60273E-06	5.28464E-06	0.00000542	2.8898E-06	3.62207E-06	4.09007E-06
Lactobacillus_zeae	1.61091E-07	1.16364E-08	2.44582E-07	1.60455E-07	5.90769E-08	0	4.16615E-07	2.64692E-07	5.97273E-08	1.74364E-07	1.05636E-07	8.22727E-08	0.000000139	1.52533E-07	1.868E-07	2.15333E-08
Lactobacillus_gallinarum	5.98773E-06	0.0000005	1.33455E-07	1.07727E-07	7.35462E-06	3.66731E-06	3.40223E-06	3.72662E-06	3.73609E-06	0.000003224	2.99736E-06	2.94909E-06	0.000009374	3.74073E-06	5.53133E-06	6.9376E-06
Lactobacillus_apis	1.50307E-05	1.61727E-07	9.27091E-08	4.23273E-07	1.19169E-05	9.48054E-06	7.69385E-06	5.97254E-06	6.52345E-06	5.41909E-06	6.13391E-06	7.34064E-06	1.74893E-05	7.78873E-06	9.87667E-06	1.43307E-05
Lactobacillus_gasseri	9.95055E-06	9.86727E-07	7.40909E-08	4.83364E-08	0.00000832	4.90769E-06	5.92123E-06	2.66677E-06	3.81518E-06	3.46645E-06	4.40609E-06	6.31545E-06	1.15907E-05	5.09313E-06	6.86093E-06	1.03513E-05
Lactobacillus_dextrinicus	0.000001058	2.05909E-07	1.80764E-07	1.61455E-07	1.07262E-06	1.73923E-07	0	1.60846E-07	5.17455E-07	7.51364E-07	4.66545E-07	1.58364E-07	1.11273E-06	7.718E-07	9.71773E-07	7.446E-07
Lactobacillus_helveticus	1.95866E-05	9.50182E-07	3.28545E-08	6.43636E-08	0.00001632	0.000011886	1.11385E-05	7.10931E-06	9.25791E-06	8.35145E-06	1.09525E-05	8.63364E-06	2.50267E-05	9.66467E-06	1.54393E-05	1.94373E-05
Lactobacillus_iners	5.18364E-06	1.59318E-06	6.08045E-07	1.05109E-06	4.33154E-06	4.14308E-06	2.61423E-06	2.36892E-06	2.74182E-06	2.08891E-06	1.84055E-06	3.32927E-06	0.000005728	3.4538E-06	0.000004672	5.6144E-06
Lactobacillus_jensenii	4.23794E-05	7.62545E-07	1.65727E-08	3.28091E-07	4.59692E-05	2.60028E-05	2.56626E-05	2.41446E-05	2.54627E-05	2.11255E-05	0.000017577	1.98527E-05	6.23889E-05	0.000025104	0.000032296	4.17359E-05
Lactobacillus_acetotolerans	3.90225E-05	5.12364E-07	8.94545E-08	3.83818E-07	3.41431E-05	2.52409E-05	2.26577E-05	0.0000161	1.96673E-05	1.54355E-05	2.01775E-05	1.99925E-05	5.27138E-05	0.000021184	0.000029652	3.77787E-05
Lactobacillus_kefiranofaciens	2.91609E-05	8.06409E-07	2.34127E-07	3.03088E-06	3.38308E-05	1.52892E-05	1.88108E-05	2.12423E-05	1.95682E-05	1.49736E-05	1.42755E-05	1.55706E-05	4.36919E-05	1.90053E-05	0.000026704	3.81997E-05
Lactobacillus_amylovorus	7.26155E-05	1.12882E-06	7.56773E-07	7.40273E-07	6.46087E-05	4.55977E-05	4.39021E-05	2.84535E-05	3.88467E-05	3.35937E-05	3.75378E-05	4.02134E-05	9.98736E-05	3.80113E-05	5.37407E-05	7.68869E-05
Lactobacillus_paragasseri	4.52091E-06	6.81909E-07	3.44455E-07	1.74927E-06	3.01046E-06	2.49377E-06	2.17623E-06	2.06085E-06	2.78855E-06	1.91282E-06	0.000002585	2.51309E-06	4.60333E-06	2.36747E-06	3.20647E-06	0.000003852
Lactobacillus_amylolyticus	9.39219E-05	8.20182E-07	1.10909E-07	2.28909E-07	9.28991E-05	5.81349E-05	5.43228E-05	3.99319E-05	5.00584E-05	4.28736E-05	4.40807E-05	4.44965E-05	0.000133831	5.21759E-05	7.33651E-05	9.78317E-05
Lactobacillus_crispatus	0.00010817	3.35617E-05	3.09545E-06	3.46364E-06	0.00010621	5.86839E-05	5.64568E-05	5.34042E-05	5.51022E-05	4.75066E-05	5.06708E-05	5.31175E-05	0.000149313	5.45937E-05	8.43358E-05	0.000106422
Lactobacillus_sp._CBA3606	2.42818E-07	0	7.40909E-08	3.13909E-07	3.34615E-08	0	5.85385E-08	1.67462E-07	1.19091E-07	0	0	9.65455E-08	4.10667E-08	3.48E-08	0	3.67333E-08
Lactobacillus_sp._wkB8	2.08736E-06	0	4.12727E-09	0	1.89546E-06	1.17692E-06	7.52769E-07	8.42077E-07	1.10564E-06	8.56364E-07	1.07736E-06	1.15473E-06	2.36293E-06	9.58933E-07	1.38047E-06	1.91987E-06
Lactobacillus_sp.	7.24482E-06	6.01273E-06	1.27364E-06	1.04364E-06	5.77154E-06	0.000589083	0.0000035	3.40054E-06	1.09691E-05	8.11645E-06	1.33227E-05	2.92755E-06	1.04315E-05	5.82053E-06	3.6914E-06	5.50733E-06
Lactobacillus_acidophilus	0.000127003	7.06818E-06	1.65573E-06	2.70427E-06	0.000128314	0.000106516	7.19374E-05	5.83895E-05	6.89855E-05	6.13611E-05	6.13545E-05	5.97565E-05	0.000179906	7.16993E-05	0.000104649	0.000135146
Lactobacillus_johnsonii	0.023380416	0.056605637	0.004589233	0.003876731	0.014909908	0.001183818	0.005893924	0.014139548	0.007868196	0.007976912	0.005278352	0.00546458	0.013569755	0.007344425	0.009575799	0.01334234
Lactobacillus_saerimneri	8.53636E-08	1.97273E-08	3.70909E-08	4.79091E-08	3.58462E-08	0.000000202	0	3.53538E-07	8.78182E-08	1.22091E-07	3.25636E-07	3.98818E-07	9.88667E-08	4.64667E-08	1.77733E-07	1.962E-07
Lactobacillus_acidipiscis	1.96545E-07	1.53818E-07	1.36673E-07	1.61364E-07	8.13077E-08	2.04692E-07	1.46923E-07	3.56692E-07	1.32909E-07	9.40909E-08	3.16909E-07	1.72909E-07	1.72867E-07	5.92E-08	1.8724E-07	2.988E-07
Ligilactobacillus_agilis	6.46818E-07	3.34909E-07	1.91727E-07	0.000000763	1.00546E-06	3.07172E-05	6.91385E-07	2.39923E-07	8.26818E-07	7.54364E-07	0.000001733	1.25436E-06	1.04887E-06	1.20573E-06	0.000000755	4.748E-07
Ligilactobacillus_salivarius	6.50455E-06	2.28645E-06	1.66139E-06	0.000004742	0.000004189	4.09977E-06	5.35023E-06	4.75962E-06	5.42182E-06	3.35182E-06	5.12527E-06	5.66909E-06	0.000003882	5.58867E-06	0.00000691	8.68067E-06
Ligilactobacillus_ruminis	1.59527E-05	1.83436E-05	6.86882E-06	8.79545E-06	1.30423E-05	1.36929E-05	7.35231E-06	9.28154E-06	1.74791E-05	1.64227E-05	1.60527E-05	2.79057E-05	8.68067E-06	0.000015262	0.000011904	0.000009472
Lactobacillus_animalis	4.23234E-05	0.000034879	6.26357E-06	6.23227E-06	4.43908E-05	0.002443541	2.56308E-05	1.57654E-05	5.69693E-05	5.96989E-05	0.000102053	3.58703E-05	5.91431E-05	4.41055E-05	2.78847E-05	0.000037337
Lactobacillus_murinus	0.002767988	0.002260578	0.000372783	0.000437606	0.002977733	0.182349296	0.001784386	0.001051424	0.003669369	0.004054207	0.006684084	0.002330146	0.004031532	0.003129775	0.001948847	0.002527167
Natranaerobius_thermophilus	2.58545E-07	1.28818E-07	2.904E-07	2.67636E-07	6.57077E-07	2.19231E-07	1.41923E-07	4.53538E-07	3.19727E-07	2.04818E-07	3.25455E-07	3.12364E-07	5.86067E-07	8.58333E-07	1.1006E-06	6.65867E-07
Thermosediminibacter_oceani	1.04327E-06	1.81409E-06	3.06345E-07	1.67909E-06	3.72231E-07	1.05538E-07	1.13538E-06	1.38969E-06	7.30909E-07	9.15182E-07	9.19727E-07	6.50818E-07	2.04267E-07	6.30133E-07	3.54867E-07	7.476E-07
Biomaibacter_acetigenes	5.26364E-06	1.38964E-06	6.69273E-07	2.21218E-06	0.000001454	8.62077E-07	2.68154E-06	2.56885E-06	3.62455E-06	4.46636E-06	4.44073E-06	4.72273E-06	2.1192E-06	1.64067E-06	1.92253E-06	2.67553E-06
Tepidanaerobacter_acetatoxydans	1.85873E-06	1.04591E-06	7.42364E-07	8.87091E-07	8.05231E-07	2.27408E-06	2.55069E-06	2.42592E-06	0.000000746	0.000000989	0.000001804	1.66873E-06	5.546E-07	1.70367E-06	0.000001514	0.000001561
Acetohalobium_arabaticum	3.14182E-06	8.24636E-07	4.80727E-07	2.56909E-07	1.07392E-06	8.47923E-07	2.46138E-06	1.83154E-06	0.000001555	0.000003947	3.52118E-06	2.36964E-06	1.08193E-06	6.92467E-07	1.00533E-06	1.8956E-06
Halobacteroides_halobius	1.24727E-06	5.16909E-07	7.76364E-07	6.34455E-07	9.49462E-07	0	1.72208E-06	1.31831E-06	4.98273E-07	8.63727E-07	8.66636E-07	0.000000896	2.55133E-07	8.16867E-07	7.902E-07	8.85333E-07
Anoxybacter_fermentans	1.72136E-06	4.20273E-07	6.21455E-07	7.01273E-07	1.68131E-06	1.15831E-06	6.61462E-07	0.000000637	0.000000645	2.80909E-07	6.91182E-07	7.82273E-07	7.27733E-07	1.30793E-06	1.70307E-06	7.90867E-07
Halanaerobium_praevalens	2.77045E-06	2.36636E-07	6.34091E-07	5.54273E-07	8.24692E-07	2.54615E-07	1.70146E-06	1.82354E-06	0.000002819	4.43036E-06	5.40727E-06	2.48373E-06	9.716E-07	7.76333E-07	1.08984E-06	1.4106E-06
Halanaerobium_hydrogeniformans	1.89227E-06	6.75682E-07	3.22545E-07	0.000000929	1.14723E-06	0	6.76846E-07	6.19923E-07	9.23273E-07	1.14527E-06	8.38455E-07	1.09455E-06	9.34867E-07	1.84353E-06	1.2782E-06	8.93067E-07
Halothermothrix_orenii	7.57909E-07	1.01441E-06	1.00364E-07	0.000000978	6.11308E-07	2.78462E-07	4.48846E-07	3.79923E-07	2.61727E-07	7.48182E-07	7.11182E-07	0.000000506	0.000000257	1.44513E-06	1.04387E-06	4.14733E-07
Halocella_sp._SP3-1	4.38627E-06	3.62791E-06	1.97564E-06	4.07091E-06	5.17908E-06	7.87538E-07	4.91985E-06	4.73846E-06	3.92727E-06	3.40327E-06	5.51645E-06	5.44009E-06	3.40893E-06	4.1668E-06	7.31467E-06	0.00000643
Thermodesulfobium_acidiphilum	1.39182E-07	0.000000065	8.23455E-08	7.44545E-08	1.26154E-07	0	1.66154E-07	5.13077E-08	0.000000104	3.68727E-07	0	1.04636E-07	1.06667E-07	2.55133E-07	1.51333E-07	1.98667E-08
Thermodesulfobium_narugense	2.66636E-07	0	5.36364E-08	1.05091E-07	2.05385E-08	0	7.54615E-08	1.88846E-07	8.43636E-08	2.05818E-07	1.92545E-07	1.83455E-07	2.94667E-08	4.49333E-08	0	0
Mahella_australiensis	1.97836E-05	1.74105E-05	4.63509E-06	4.74636E-06	1.36269E-05	2.44646E-06	1.07916E-05	9.93692E-06	1.80209E-05	3.50405E-05	1.60218E-05	1.66791E-05	1.07613E-05	1.23087E-05	1.00566E-05	1.12411E-05
Thermanaeromonas_toyohensis	4.40545E-07	2.69409E-06	1.16436E-07	2.02182E-07	1.90538E-07	6.71077E-07	6.12692E-07	6.08615E-07	3.22727E-07	7.35091E-07	3.96091E-07	6.66818E-07	1.84467E-07	1.29133E-07	1.88467E-07	7.06267E-07
Carboxydothermus_hydrogenoformans	2.07809E-06	7.56182E-07	2.96091E-07	7.97818E-07	0.000000508	8.09077E-07	2.24692E-07	3.55769E-07	1.03845E-06	1.25991E-06	8.32091E-07	7.42182E-07	3.86267E-07	4.28333E-07	6.80067E-07	4.356E-07
Thermacetogenium_phaeum	9.64182E-07	0.000000505	2.12436E-07	1.42036E-06	8.77846E-07	1.44446E-06	1.16031E-06	0.000001534	1.72555E-06	1.51555E-06	1.27609E-06	3.10473E-06	1.10893E-06	1.03987E-06	1.21407E-06	1.59787E-06
Thermoanaerobacter_wiegelii	0	0	0	1.03636E-08	3.11077E-07	6.76923E-08	0.0000001	1.21538E-07	0.000000053	2.17273E-07	1.71455E-07	0.000000711	1.49667E-07	2.98267E-07	3.93307E-07	3.39933E-07
Thermoanaerobacter_mathranii	0	0	0	0	0	0	0	8.52308E-08	0	0	1.43545E-07	0	0	0	0	1.86E-08
Thermoanaerobacter_kivui	9.33636E-07	7.38636E-08	1.10945E-07	1.25636E-07	5.39231E-07	1.23231E-07	4.41923E-07	0.000000395	3.99364E-07	4.05727E-07	3.11545E-07	5.07455E-07	9.64E-08	0.000000467	4.66533E-07	3.73067E-07
Thermoanaerobacter_italicus	2.53909E-07	0	7.57273E-08	0.000000607	5.45385E-08	0	3.07385E-07	1.73692E-07	2.64091E-07	4.29091E-07	3.58091E-07	2.38273E-07	2.74867E-07	3.39733E-07	3.11867E-07	2.98067E-07
Thermoanaerobacter_siderophilus	3.16636E-07	0	1.85455E-07	1.25455E-06	0.000000147	0	0	1.97692E-07	1.04591E-06	8.39909E-07	5.13364E-07	1.29918E-06	2.086E-07	4.084E-07	5.55133E-07	4.75533E-07
Thermoanaerobacter_ethanolicus	5.92727E-08	0	1.10909E-07	2.07273E-08	1.67538E-07	4.91538E-07	2.46077E-07	2.91385E-07	0	0.000000028	2.05455E-08	6.76636E-07	2.872E-07	1.93333E-07	1.03267E-06	5.11133E-07
Caldanaerobacter_subterraneus	2.09136E-06	1.65673E-06	5.60036E-07	1.04582E-06	1.36708E-06	1.05769E-06	3.15238E-06	7.39692E-07	1.98536E-06	1.44645E-06	1.25955E-06	2.45427E-06	9.19533E-07	1.23353E-06	1.12927E-06	4.65533E-07
Ammonifex_degensii	1.90255E-06	1.03327E-06	3.678E-07	1.08709E-06	7.76769E-07	1.78077E-06	1.08485E-06	7.50538E-07	1.20764E-06	1.08155E-06	9.86727E-07	1.77318E-06	8.44267E-07	8.18467E-07	6.92733E-07	5.88067E-07
Moorella_glycerini	2.27845E-06	2.14527E-06	7.09818E-07	2.66191E-06	1.14123E-06	1.50454E-06	6.32308E-07	7.26615E-07	1.74945E-06	1.55327E-06	1.41891E-06	2.40364E-06	1.4698E-06	1.40253E-06	1.8822E-06	1.83467E-06
Moorella_thermoacetica	5.71364E-06	1.29445E-06	0.000003943	0.00001173	6.59846E-06	5.31023E-06	8.39615E-06	1.35108E-05	1.16464E-05	9.68727E-06	0.00001481	1.33336E-05	0.000006978	8.37267E-06	7.25267E-06	0.000007114
Caldicellulosiruptor_kristjanssonii	1.30182E-07	9.75455E-08	0	1.55455E-08	0	0	0	2.46923E-08	2.12545E-07	0.000000028	7.89091E-08	1.47364E-07	0	4.64667E-08	1.00667E-07	0
Caldicellulosiruptor_obsidiansis	0.000000262	4.71818E-08	7.53091E-08	2.27455E-07	0.000000189	8.49077E-07	2.92462E-07	5.21538E-07	2.97727E-07	3.80727E-07	0.00000076	2.25182E-07	2.776E-07	3.46067E-07	0.000000143	0.000000128
Caldicellulosiruptor_kronotskyensis	1.42182E-07	4.58182E-08	3.78909E-08	8.27273E-08	3.34615E-08	0	6.23077E-08	0.000000158	2.33909E-07	3.71364E-07	1.72527E-06	8.34545E-07	7.83333E-08	0.000000066	1.372E-07	1.22E-08
Caldicellulosiruptor_saccharolyticus	2.30091E-07	1.55182E-07	1.31436E-07	8.31818E-08	5.32231E-07	3.58462E-07	1.99615E-07	3.54538E-07	3.99909E-07	5.71091E-07	8.23818E-07	1.04973E-06	3.02867E-07	2.95667E-07	4.58133E-07	3.84333E-07
Caldicellulosiruptor_lactoaceticus	1.87818E-06	3.75455E-08	4.23091E-07	0	4.83846E-07	6.53077E-08	0.00000149	1.49338E-06	2.37645E-06	3.84782E-06	3.22564E-06	1.37123E-06	6.136E-07	2.23333E-07	3.97267E-07	1.21467E-06
Caldicellulosiruptor_changbaiensis	4.71364E-07	1.53455E-07	2.04182E-07	2.40455E-07	1.38462E-07	0	4.03308E-07	6.55538E-07	2.62818E-07	3.89091E-08	3.95455E-08	2.47909E-07	1.782E-07	2.57933E-07	3.3984E-07	2.17933E-07
Caldicellulosiruptor_hydrothermalis	7.72727E-08	0	1.63636E-08	1.43636E-08	6.30769E-08	7.21538E-08	4.08923E-07	0.000000282	0	1.77455E-07	1.41727E-07	7.13636E-09	1.136E-07	0.000000089	1.22467E-07	3.21667E-07
Caldicellulosiruptor_bescii	0	0	1.63636E-08	5.16364E-08	3.74846E-07	0	2.63077E-08	2.51538E-07	3.98182E-08	0.000000056	1.19091E-07	5.10182E-07	0	1.488E-07	3.14667E-08	2.11933E-07
Caldicellulosiruptor_owensensis	0.000000885	6.90273E-07	1.65909E-07	2.79091E-07	1.41108E-06	0	4.22769E-07	2.68231E-07	3.88182E-07	0.000000235	5.30909E-07	4.98636E-07	5.76467E-07	8.18667E-07	1.09933E-06	1.30073E-06
Thermoanaerobacterium_aotearoense	2.03882E-06	1.04905E-06	8.92118E-07	9.42364E-07	1.10008E-06	9.46154E-09	1.39546E-06	1.50731E-06	1.13773E-06	5.83818E-07	2.16955E-06	1.41536E-06	6.906E-07	1.58807E-06	1.72053E-06	1.4366E-06
Thermoanaerobacterium_xylanolyticum	3.22455E-07	6.65091E-07	1.17818E-07	6.73636E-07	4.03692E-07	4.09308E-07	1.65138E-06	1.20462E-06	3.06455E-07	5.78727E-07	5.22727E-07	6.45273E-07	3.25267E-07	6.54267E-07	6.44067E-07	7.35333E-07
Thermoanaerobacterium_sp._RBIITD	4.05182E-06	2.56655E-06	1.39509E-06	2.07927E-06	1.75408E-06	1.62008E-06	3.57685E-06	3.33046E-06	4.78218E-06	2.68518E-06	3.10445E-06	3.58936E-06	1.4016E-06	3.9206E-06	1.86787E-06	1.84807E-06
Thermoanaerobacterium_thermosaccharolyticum	1.32073E-06	1.72536E-06	1.00309E-06	1.64609E-06	3.91969E-06	5.66462E-07	1.42608E-06	1.89938E-06	1.16882E-06	1.28573E-06	1.95855E-06	0.00000199	2.53533E-06	8.92313E-06	4.91467E-06	2.4966E-06
Symbiobacterium_thermophilum	4.89818E-06	3.45045E-06	1.48882E-06	3.26773E-06	2.73231E-06	5.42069E-06	2.60677E-06	0.000004005	4.88255E-06	5.68273E-06	6.13018E-06	8.68182E-06	2.37273E-06	2.29107E-06	2.6558E-06	2.87227E-06
Syntrophothermus_lipocalidus	3.41455E-07	6.31818E-08	1.79309E-07	0.000000325	9.38462E-08	2.52308E-08	1.39923E-07	4.10923E-07	1.76455E-07	2.39636E-07	9.44545E-08	2.18545E-07	0.000000094	2.08133E-07	9.15067E-08	1.86667E-08
Syntrophomonas_wolfei	2.09336E-06	3.70773E-07	9.19545E-07	1.29018E-06	2.98369E-06	3.23615E-07	2.78231E-06	1.61369E-06	1.16736E-06	2.08582E-06	8.80364E-07	0.000003688	2.3674E-06	2.0988E-06	1.55747E-06	1.01213E-06
Candidatus_Syntrophocurvum_alkaliphilum	2.61373E-06	1.22091E-06	5.16455E-07	9.30545E-07	5.10769E-07	6.53077E-08	9.03923E-07	1.38046E-06	1.97555E-06	2.28382E-06	1.61491E-06	1.46564E-06	5.19133E-07	5.49533E-07	6.76933E-07	1.3408E-06
Heliorestis_convoluta	5.31727E-07	5.73409E-07	1.67073E-07	0.000000421	5.39692E-07	0.000000682	4.31846E-07	7.80462E-07	5.14182E-07	2.70364E-07	7.46091E-07	8.57909E-07	0.000000308	4.03733E-07	7.86267E-07	1.19793E-06
Heliobacterium_modesticaldum	3.16473E-06	4.96236E-06	1.82982E-06	5.96909E-06	2.32823E-06	4.08238E-06	6.00385E-06	5.86692E-06	3.39891E-06	0.000003681	5.13955E-06	6.86182E-06	2.10573E-06	3.85773E-06	4.36713E-06	3.29733E-06
Petrocella_atlantisensis	9.56455E-06	8.08345E-06	3.63455E-06	4.77545E-06	4.00023E-06	4.42915E-06	4.87315E-06	6.68385E-06	8.43909E-06	1.28955E-05	9.54182E-06	1.26736E-05	3.60833E-06	6.56367E-06	5.05933E-06	0.000004882
Clostridiales_bacterium_Choco116	2.42873E-05	1.44213E-05	4.59327E-06	1.21306E-05	8.55615E-06	2.53193E-05	0.000022202	3.00652E-05	1.89745E-05	1.27464E-05	1.71155E-05	2.43045E-05	7.34873E-06	0.000009722	8.20313E-06	7.65067E-06
Mageeibacillus_indolicus	7.22782E-06	2.71882E-06	6.45273E-07	2.95291E-06	0.000002102	0.000001301	2.11454E-06	0.000002733	5.01427E-06	4.16509E-06	4.01273E-06	6.10545E-06	2.0516E-06	2.8302E-06	2.0844E-06	2.5882E-06
Pseudoclostridium_thermosuccinogenes	7.65909E-06	6.02518E-06	2.90291E-06	8.29455E-06	6.47615E-06	4.15408E-06	6.74754E-06	8.70923E-06	7.23091E-06	1.16282E-05	1.34291E-05	0.00001406	5.26967E-06	8.7996E-06	0.00000893	6.32067E-06
Fastidiosipila_sanguinis	2.26245E-06	3.50455E-07	5.17273E-07	2.10727E-06	1.38777E-06	6.19869E-06	4.86877E-06	7.00685E-06	1.63591E-06	1.36636E-06	2.80755E-06	0.000003622	3.23927E-06	4.2382E-06	0.000004992	0.000004376
Thermoclostridium_stercorarium	7.21909E-06	5.72364E-06	4.08636E-06	5.85882E-06	6.72162E-06	8.56846E-06	6.46769E-06	6.28108E-06	8.81545E-06	1.05036E-05	7.51364E-06	9.49909E-06	7.25267E-06	7.29133E-06	7.75533E-06	0.000008046
Ruminiclostridium_cellulolyticum	3.89973E-06	3.91509E-06	1.55155E-06	2.81027E-06	3.55438E-06	4.91538E-07	2.43538E-06	2.52123E-06	2.46991E-06	2.70691E-06	5.31091E-06	4.59036E-06	2.87067E-06	4.99327E-06	6.34713E-06	4.42307E-06
Ruminiclostridium_sp._MA18	5.81091E-06	2.39736E-06	1.45657E-06	3.57682E-06	4.09354E-06	0.000003417	2.23577E-06	4.96385E-06	3.98545E-06	5.13827E-06	6.15909E-06	6.02455E-06	3.1878E-06	0.000006582	5.27467E-06	0.000003116
Hungateiclostridium_thermocellum	6.00636E-06	3.77445E-06	2.60882E-06	5.63791E-06	3.98538E-06	2.99915E-06	6.36769E-06	4.15646E-06	5.67182E-06	5.06945E-06	0.00000524	9.72545E-06	0.000003113	3.4202E-06	0.000004542	0.000003593
Hungateiclostridium_saccincola	5.66273E-06	0.000009868	2.95145E-06	0.00000642	2.86323E-06	4.46077E-06	4.11077E-06	5.16069E-06	4.78273E-06	4.26436E-06	5.81745E-06	9.34636E-06	2.5702E-06	4.65573E-06	3.76013E-06	3.39947E-06
Hungateiclostridium_clariflavum	5.59273E-06	3.00209E-06	1.86185E-06	3.00818E-06	0.000004931	2.23046E-06	2.72277E-06	2.47685E-06	5.16909E-06	5.44091E-06	4.83082E-06	6.69273E-06	0.000003472	6.34553E-06	5.70007E-06	5.46667E-06
Candidatus_Desulforudis_audaxviator	1.80873E-06	0.000000633	1.49836E-06	1.31427E-06	1.03854E-06	1.12454E-06	1.73115E-06	2.52231E-06	2.04073E-06	1.53145E-06	2.64318E-06	2.48055E-06	1.1954E-06	1.21653E-06	1.67593E-06	9.32933E-07
Thermoanaerosceptrum_fracticalcis	7.63545E-06	4.70891E-06	0.000003109	4.52273E-06	4.06277E-06	0.000005821	7.38231E-06	5.85538E-06	4.95455E-06	6.67727E-06	6.55636E-06	7.08636E-06	5.01333E-06	4.50867E-06	6.04667E-06	0.000004844
Thermincola_potens	2.12664E-06	0.000001496	9.21409E-07	2.67473E-06	1.62977E-06	9.89154E-07	2.33908E-06	1.65769E-06	2.13482E-06	1.97118E-06	1.84436E-06	2.25664E-06	1.00707E-06	1.4314E-06	1.32627E-06	1.06593E-06
Syntrophobotulus_glycolicus	2.04273E-05	2.74017E-05	6.09373E-06	0.000007387	1.23155E-05	7.09723E-06	7.40646E-06	9.09208E-06	1.43882E-05	1.36036E-05	9.04636E-06	1.65771E-05	0.00000707	0.000012604	8.79133E-06	7.38047E-06
Dehalobacter_sp._CF	2.16273E-07	3.16273E-07	2.47273E-08	8.45182E-08	4.93846E-08	5.12308E-07	0	5.81538E-08	0.000000121	0.000000124	1.02727E-08	7.51818E-08	2.64E-08	0.000000047	2.97333E-08	0.000000168
Dehalobacter_sp._DCA	2.44545E-07	2.11364E-07	0	7.43364E-08	6.23077E-08	2.72923E-07	2.21692E-07	1.42692E-07	2.16364E-08	8.08182E-08	0	4.79091E-08	2.37333E-08	0	1.892E-07	0
Dehalobacter_restrictus	4.99182E-06	1.05391E-06	1.11218E-06	2.19445E-06	2.00523E-06	1.46769E-06	3.98654E-06	4.17577E-06	3.92555E-06	5.60818E-06	4.91182E-06	5.64909E-06	2.0214E-06	2.49827E-06	2.90307E-06	2.75853E-06
Desulfosporosinus_acidiphilus	1.44145E-06	8.47455E-07	6.02909E-07	1.21027E-06	1.16385E-06	2.29362E-06	9.37385E-07	1.45131E-06	1.54482E-06	1.27809E-06	1.39936E-06	3.01436E-06	7.71067E-07	7.89867E-07	1.2398E-06	9.33667E-07
Desulfosporosinus_orientis	2.12782E-06	1.36082E-06	7.35364E-07	3.75527E-06	2.12608E-06	2.30069E-06	2.85738E-06	1.54831E-06	0.000001838	1.17591E-06	1.96909E-06	2.55082E-06	1.3336E-06	2.51567E-06	0.000001623	1.52047E-06
Desulfosporosinus_meridiei	3.27218E-06	1.34527E-06	7.12773E-07	3.37573E-06	1.40285E-06	3.75069E-06	4.32108E-06	5.46338E-06	5.96909E-06	3.78382E-06	7.38018E-06	6.23727E-06	1.64807E-06	1.5732E-06	1.46507E-06	2.17727E-06
Desulfosporosinus_youngiae	1.79555E-06	1.98355E-06	0.000004689	3.18164E-06	5.84462E-06	8.44615E-07	5.38615E-07	1.14685E-06	4.50127E-06	5.05218E-06	5.04545E-06	5.05364E-06	4.88393E-06	0.000007126	5.83533E-06	3.86333E-06
Desulfofarcimen_acetoxidans	8.35764E-06	4.82818E-06	1.62064E-06	4.44609E-06	5.82938E-06	4.71369E-06	5.41808E-06	0.000002417	6.68873E-06	6.76645E-06	6.20273E-06	6.54555E-06	3.34073E-06	0.000004364	2.9094E-06	1.69847E-06
Desulfallas_gibsoniae	7.01909E-06	3.73518E-06	1.81709E-06	4.63091E-06	3.25769E-06	1.00722E-05	5.33469E-06	5.03923E-06	0.000005668	6.21882E-06	7.35909E-06	8.52636E-06	2.49187E-06	3.96967E-06	3.30093E-06	4.04967E-06
Dehalobacterium_formicoaceticum	1.02382E-05	1.05807E-05	2.72236E-06	6.01273E-06	5.91615E-06	0.000004819	5.90415E-06	7.03846E-06	0.00001021	1.03736E-05	1.20664E-05	1.01464E-05	4.41267E-06	7.18333E-06	6.75187E-06	5.58173E-06
Desulfotomaculum_nigrificans	9.10455E-07	4.81182E-07	1.43818E-07	7.87273E-07	0.00000071	8.62008E-06	1.17469E-06	1.31085E-06	0.000000726	7.23182E-07	1.33091E-06	0.00000147	3.96333E-07	5.446E-07	9.976E-07	6.62933E-07
Desulfotomaculum_ferrireducens	1.93718E-06	4.43727E-07	6.53818E-07	2.16773E-06	1.21423E-06	3.06754E-06	2.92838E-06	3.23031E-06	1.63827E-06	2.15282E-06	1.94236E-06	2.57091E-06	6.78067E-07	1.22493E-06	1.40027E-06	1.09233E-06
Desulfotomaculum_reducens	3.68727E-06	6.36709E-06	2.47618E-06	4.69818E-06	2.63385E-06	2.41523E-06	2.22092E-06	3.76869E-06	3.25718E-06	2.93364E-06	2.28618E-06	4.16273E-06	2.5426E-06	2.6204E-06	2.5594E-06	1.82047E-06
Desulfotomaculum_ruminis	4.19182E-06	7.24864E-06	1.25227E-06	4.01155E-06	2.09177E-06	2.47462E-06	2.61077E-06	2.43438E-06	2.43709E-06	2.42282E-06	3.78818E-06	5.58091E-06	1.9364E-06	2.13447E-06	1.87013E-06	1.55367E-06
Desulfitobacterium_metallireducens	2.21009E-06	1.11145E-06	2.93409E-07	1.84227E-06	1.78515E-06	1.03405E-05	3.10308E-06	0.000002319	1.29691E-06	1.34782E-06	1.84318E-06	1.55164E-06	1.98753E-06	1.81767E-06	2.7758E-06	1.81107E-06
Desulfitobacterium_dehalogenans	3.05018E-06	2.12427E-06	0.000001132	2.80955E-06	2.77777E-06	3.05792E-06	2.76731E-06	2.19892E-06	5.50564E-06	2.93727E-06	4.15509E-06	3.07182E-06	1.1554E-06	2.52893E-06	2.2934E-06	1.8538E-06
Desulfitobacterium_dichloroeliminans	2.61282E-06	1.70036E-06	6.91545E-07	7.30627E-06	9.67115E-06	1.30738E-06	4.62769E-06	5.36315E-06	2.14836E-06	2.38827E-06	0.00000383	7.10727E-06	8.41633E-06	9.48667E-06	1.52273E-05	1.50633E-05
Desulfitobacterium_hafniense	1.17245E-05	7.24291E-06	2.84173E-06	9.15182E-06	7.00808E-06	4.07046E-06	6.34077E-06	6.12485E-06	9.15364E-06	1.29818E-05	1.28218E-05	1.36464E-05	4.2866E-06	1.00793E-05	7.7516E-06	5.4084E-06
Christensenella_minuta	7.39148E-05	1.62037E-05	1.03945E-05	0.00001518	2.37552E-05	2.38922E-05	4.15561E-05	3.35208E-05	8.98983E-05	0.000138299	6.87255E-05	4.40904E-05	0.000026579	0.000021834	0.000017864	4.03013E-05
Christensenella_sp._Marseille-P3954	0.000113243	8.74725E-05	2.93605E-05	8.70376E-05	7.24712E-05	9.80982E-05	7.92782E-05	7.95408E-05	9.99735E-05	9.17964E-05	9.09125E-05	0.000115846	6.74823E-05	9.55783E-05	7.21697E-05	5.55017E-05
Acetobacterium_woodii	7.58636E-06	4.16173E-06	0.000001611	4.27445E-06	2.33892E-06	1.56723E-06	4.25377E-06	4.63054E-06	6.94182E-06	1.02136E-05	9.34727E-06	7.53273E-06	2.4548E-06	3.20287E-06	3.33067E-06	3.32947E-06
Acetobacterium_sp._KB-1	4.29645E-06	2.97691E-06	2.07245E-06	3.13182E-06	3.81462E-06	2.82246E-06	4.15923E-06	4.33392E-06	4.94818E-06	5.42109E-06	5.59727E-06	8.26182E-06	2.78387E-06	4.29047E-06	4.13733E-06	3.95067E-06
Eubacterium_callanderi	3.00636E-05	0.000014824	1.11041E-05	2.40536E-05	3.22692E-05	4.24504E-05	2.06131E-05	2.42615E-05	2.54782E-05	0.0000354	2.54555E-05	4.57855E-05	0.000022994	2.97289E-05	2.30873E-05	2.40647E-05
[Eubacterium]_cellulosolvens	8.07266E-05	7.14579E-05	2.65657E-05	5.61648E-05	4.99968E-05	0.000078894	5.77993E-05	5.00465E-05	0.000077348	9.23024E-05	7.09421E-05	9.27653E-05	3.65807E-05	5.48127E-05	4.24774E-05	4.04747E-05
Eubacterium_limosum	7.45118E-05	9.00264E-05	1.80366E-05	5.95914E-05	4.93187E-05	5.50158E-05	4.94609E-05	4.59385E-05	0.000097664	0.000111688	7.20491E-05	9.47157E-05	4.05333E-05	4.95755E-05	4.84475E-05	4.43399E-05
Eubacterium_sp._NSJ-61	0.000163873	9.14083E-05	4.28689E-05	8.68614E-05	0.000134159	9.40785E-05	0.00012052	0.000154664	0.000169353	0.000226155	0.000161292	0.00029276	0.000124691	0.000146784	0.000126821	0.0001458
Eubacterium_maltosivorans	6.23396E-05	3.24325E-05	1.32994E-05	6.46522E-05	5.06082E-05	0.000052306	5.50498E-05	4.46441E-05	4.42031E-05	7.61653E-05	5.26411E-05	0.000056584	2.47913E-05	7.37336E-05	5.15743E-05	3.22813E-05
Peptacetobacter_hiranonis	1.53036E-05	0.000016186	5.64364E-06	6.44091E-06	0.000005286	3.38546E-06	6.63492E-06	8.35654E-06	9.99545E-06	8.57455E-06	9.06636E-06	5.98309E-06	3.00447E-06	5.40333E-06	5.66913E-06	5.51867E-06
Filifactor_alocis	6.81818E-06	5.60955E-06	3.40241E-06	5.63818E-06	1.57738E-06	3.43292E-06	3.49769E-06	9.31846E-06	1.04324E-05	1.05282E-05	9.02727E-06	6.70545E-06	2.8338E-06	0.000002794	3.7158E-06	0.000003708
Acetoanaerobium_sticklandii	1.67857E-05	3.91355E-06	0.000002305	1.93164E-06	5.33938E-06	1.05692E-06	8.24162E-06	7.56769E-06	0.00001552	2.62727E-05	1.65167E-05	1.08855E-05	4.27767E-06	3.70453E-06	4.9854E-06	9.30573E-06
Peptoclostridium_acidaminophilum	0.00001349	7.43236E-06	4.34589E-06	9.40636E-06	5.63462E-06	3.09546E-06	1.17124E-05	1.17931E-05	8.06182E-06	1.05191E-05	1.10873E-05	1.50609E-05	0.000006522	1.10787E-05	1.01027E-05	9.88267E-06
Paraclostridium_bifermentans	2.64755E-06	4.33891E-06	1.11973E-06	7.82091E-07	0.000002001	1.44831E-06	3.75954E-06	4.95854E-06	7.78455E-07	1.40791E-06	1.63027E-06	1.31564E-06	0.00000236	3.56627E-06	4.6196E-06	0.00000393
Paeniclostridium_sordellii	3.96627E-06	2.25982E-06	1.62591E-06	2.29645E-06	3.31908E-06	1.24377E-06	6.21923E-06	0.000005	2.64555E-06	0.000002411	3.78255E-06	3.25745E-06	4.23287E-06	5.66733E-06	5.31893E-06	5.8708E-06
Romboutsia_sp._CE17	0.000001402	1.06545E-06	1.76527E-06	6.33909E-07	8.63077E-07	6.24692E-07	9.78846E-07	2.62477E-06	1.35427E-06	1.27218E-06	1.81655E-06	3.02427E-06	0.0000016	2.0996E-06	3.62667E-06	4.32747E-06
Romboutsia_hominis	2.08591E-06	2.32118E-06	1.61845E-06	1.30545E-06	3.25108E-06	0.000002128	2.16538E-06	1.33792E-06	1.83836E-06	2.48182E-06	1.74655E-06	0.000005517	2.37267E-06	2.8236E-06	5.05533E-06	5.02947E-06
Romboutsia_ilealis	4.23491E-06	1.18318E-06	0.000686777	2.64409E-06	3.93469E-06	0.000001386	4.78508E-06	0.00000304	2.88845E-06	2.55136E-06	4.03818E-06	4.29309E-06	0.000560187	0.000388042	0.001169502	0.001414828
Clostridioides_difficile	0.001264928	0.001393952	0.000428832	0.000839744	0.000555595	0.000976873	0.000753145	0.00086075	0.001075116	0.000953296	0.001161533	0.001134078	0.000523057	0.000734799	0.000572257	0.000475404
Butyricicoccus_sp._GAM44	2.33964E-06	3.24545E-07	4.87727E-07	0.000001266	8.01538E-07	8.75385E-07	6.68923E-07	5.51308E-07	2.09036E-06	1.02427E-06	1.08255E-06	2.45464E-06	7.45533E-07	6.10133E-07	7.45267E-07	8.01533E-07
Hathewaya_histolytica	2.20827E-06	6.28045E-07	9.42182E-07	0.00000112	6.48462E-07	7.56077E-07	9.92231E-07	1.62246E-06	1.04764E-06	9.23182E-07	1.80636E-06	0.000001816	1.2344E-06	0.000001603	2.01613E-06	1.34067E-06
Caloranaerobacter_azorensis	5.83182E-06	2.22518E-06	1.79055E-06	1.85982E-06	2.56015E-06	0.00000103	0.000002995	3.62185E-06	5.03491E-06	6.89782E-06	5.45818E-06	4.07318E-06	2.51687E-06	3.39473E-06	3.08767E-06	2.78247E-06
Caloramator_sp._E03	5.97727E-06	4.34718E-06	1.62845E-06	3.45445E-06	2.05469E-06	2.26877E-06	2.99185E-06	3.84046E-06	2.88818E-06	2.07445E-06	3.61336E-06	4.42918E-06	2.48853E-06	3.44967E-06	3.3876E-06	2.57973E-06
Geosporobacter_ferrireducens	9.83727E-06	4.90564E-06	3.02709E-06	5.70273E-06	0.000004723	7.34408E-06	8.06777E-06	7.43569E-06	9.30364E-06	1.20664E-05	1.31345E-05	1.04909E-05	4.60687E-06	5.73533E-06	6.89733E-06	0.00000531
Crassaminicella_sp._SY095	0.0000047	2.60959E-06	1.27518E-06	3.22245E-06	5.52538E-06	1.62546E-06	4.62923E-06	5.65923E-06	3.60009E-06	3.98745E-06	4.89818E-06	4.17727E-06	2.9422E-06	6.12907E-06	0.000006876	3.87333E-06
Alkaliphilus_oremlandii	1.63245E-06	1.58545E-06	7.73118E-07	8.39182E-07	9.81308E-07	0.000000854	6.79769E-07	8.94846E-07	7.30091E-07	7.53909E-07	1.67636E-06	2.23182E-06	3.854E-07	9.954E-07	1.0072E-06	1.10107E-06

Alkaliphilus_metalliredigens	2.64645E-05	3.23632E-06	0.00000377	2.27188E-05	9.55231E-06	1.23554E-06	4.21462E-06	1.05211E-05	1.69091E-05	1.48664E-05	1.46073E-05	2.14909E-05	1.52747E-05	0.00001686	1.15233E-05	1.20013E-05
Hungatella_hathewayi	0.000358048	0.000446011	0.000125423	0.000258801	0.000218809	0.000310464	0.000262116	0.000220056	0.000361305	0.000449821	0.000291237	0.000365976	0.000169937	0.00023219	0.000181366	0.000164328
Candidatus_Arthromitus_sp._SFB-rat-Yit	8.23636E-06	3.62091E-07	1.11294E-06	1.46791E-06	3.82023E-06	0.00000255	2.15692E-06	2.34585E-06	2.12391E-06	3.28582E-06	4.05864E-06	2.61309E-06	3.5722E-06	4.50567E-06	3.11413E-06	2.31927E-06
Candidatus_Arthromitus_sp._SFB-mouse-NL	0.000139628	7.14545E-07	1.02348E-05	1.16633E-05	0.000028143	0.000000866	0.00001353	0.0000011	2.60559E-05	3.75912E-05	4.77285E-05	1.88218E-05	4.19272E-05	4.79509E-05	5.18853E-06	1.14647E-05
Candidatus_Arthromitus_sp._SFB-mouse	0.000186686	2.01818E-06	1.46727E-05	1.56414E-05	3.82813E-05	1.01308E-06	0.000017651	0.000001593	3.46203E-05	4.79028E-05	6.39039E-05	2.40683E-05	5.54849E-05	5.91912E-05	7.5192E-06	1.36453E-05
Clostridium_sp._MF28	1.19636E-07	1.14818E-07	1.63636E-08	0	0	0.000000026	0	0	0	0	1.02727E-08	4.65909E-08	0	0	0	0
Clostridium_autoethanogenum	1.69882E-06	1.24918E-06	3.81273E-07	8.94545E-07	1.39231E-07	2.95385E-08	8.26154E-07	6.57692E-07	0.000000371	7.32818E-07	9.89364E-07	8.26727E-07	2.38533E-07	0.000000894	4.09733E-07	2.80667E-07
Clostridium_ljungdahlii	4.08909E-07	2.69818E-07	2.52636E-07	3.07636E-07	0	0	4.33846E-08	0	5.07273E-07	6.32364E-07	4.28545E-07	2.08909E-07	4.27333E-08	0	2.39333E-08	2.79333E-08
Clostridium_drakei	4.77455E-07	4.66364E-07	2.73864E-07	4.25727E-07	3.80769E-07	3.35462E-07	5.17692E-07	3.08077E-07	3.80545E-07	8.39091E-08	7.43727E-07	6.79091E-07	4.33733E-07	2.234E-07	6.86667E-07	5.07733E-07
Clostridium_sporogenes	0	0	0	0	0.000000315	0	0	0	1.68455E-07	5.22727E-08	1.30909E-07	2.78818E-07	1.152E-07	1.338E-07	9.86E-08	1.04867E-07
Clostridium_diolis	1.44918E-06	2.59618E-06	5.11364E-07	1.01909E-06	1.53892E-06	0	1.29723E-06	0.00000113	4.29182E-07	6.90636E-07	1.68864E-06	0.000001355	1.826E-07	1.02333E-06	1.7686E-06	1.10613E-06
Clostridium_scatologenes	2.58455E-07	1.01336E-06	3.36818E-07	1.00764E-06	4.34154E-07	8.22462E-07	8.59615E-07	6.44308E-07	7.15455E-08	2.65818E-07	5.50909E-07	1.79873E-06	5.624E-07	0.000000587	7.25533E-07	7.56333E-07
Clostridium_saccharobutylicum	2.25091E-06	3.47782E-06	8.86727E-07	1.44418E-06	3.37454E-06	5.42462E-07	1.71646E-06	1.25092E-06	1.58491E-06	0.000000977	1.85473E-06	2.77227E-06	1.75513E-06	4.36867E-06	0.000004513	0.00000368
Clostridium_acetobutylicum	3.14936E-06	3.20541E-06	1.48164E-06	1.72564E-06	3.32831E-06	0	3.51123E-06	0.000002503	1.16082E-06	1.34164E-06	0.00000544	4.48182E-06	1.02647E-06	2.45767E-06	3.75593E-06	3.78153E-06
Clostridium_novyi	3.95909E-06	1.93282E-06	1.86345E-06	2.27427E-06	2.49154E-06	1.47554E-06	2.23277E-06	3.81208E-06	3.82745E-06	5.14727E-06	5.03745E-06	6.20455E-06	2.23213E-06	2.44693E-06	0.000002638	2.72667E-06
Clostridium_argentinense	2.15045E-06	0.000002368	1.15691E-06	1.69445E-06	1.64185E-06	1.90154E-07	6.67692E-07	9.07769E-07	1.86409E-06	2.02655E-06	1.85091E-06	1.68827E-06	1.04567E-06	1.88327E-06	3.31713E-06	0.000001845
Clostridium_chauvoei	8.18182E-07	4.13455E-07	3.52273E-07	7.01273E-07	0.0000016	5.12692E-07	2.36962E-06	2.97854E-06	1.40036E-06	9.88909E-07	0.000002382	1.76182E-06	0.000001894	2.21113E-06	2.8182E-06	2.42053E-06
Clostridium_fermenticellae	1.75545E-06	9.12545E-07	8.78427E-07	7.57091E-07	2.67992E-06	7.41385E-07	0.000001824	3.57562E-06	9.21455E-07	1.70464E-06	2.87318E-06	2.40818E-06	2.27467E-06	3.31447E-06	4.43173E-06	5.18433E-06
Clostridium_aceticum	0.00000607	5.10964E-06	1.69382E-06	2.08727E-06	3.63746E-06	2.40092E-06	4.36231E-06	3.66192E-06	3.89464E-06	6.18882E-06	7.74818E-06	5.98327E-06	2.23607E-06	3.13113E-06	0.000003896	4.48073E-06
Clostridium_cellulovorans	4.83864E-06	6.70455E-07	1.72677E-06	1.29073E-06	4.22046E-06	8.94923E-07	4.23146E-06	5.45577E-06	0.000004758	0.00000642	1.08075E-05	7.14455E-06	3.2176E-06	4.34553E-06	4.7644E-06	4.1164E-06
Clostridium_formicaceticum	1.93709E-06	1.53877E-06	1.10973E-06	2.51936E-06	2.91277E-06	2.38077E-07	1.18862E-06	1.55531E-06	1.64764E-06	2.11591E-06	3.30827E-06	2.36418E-06	2.76867E-06	2.82747E-06	3.47327E-06	3.93687E-06
Clostridium_bornimense	4.48684E-05	2.05692E-05	2.08316E-05	5.24545E-06	2.16923E-06	1.25046E-06	4.90538E-06	5.07092E-06	3.16064E-06	3.30727E-06	9.72973E-06	1.64818E-05	2.86633E-06	3.97647E-06	4.39693E-06	2.7088E-06
Clostridium_intestinale	1.79255E-06	7.96955E-07	5.70136E-07	1.01027E-06	1.14008E-06	1.29077E-07	9.91308E-07	2.20631E-06	1.05627E-06	0.000001748	2.71218E-06	0.000002137	2.21947E-06	2.23233E-06	2.01667E-06	2.02273E-06
Clostridium_taeniosporum	3.05155E-06	5.92182E-07	1.05686E-06	6.59636E-07	1.10692E-06	0.000001371	3.73792E-06	5.61238E-06	2.92718E-06	3.68118E-06	4.55418E-06	4.77391E-06	0.000003134	4.57287E-06	4.55727E-06	3.38333E-06
Clostridium_septicum	1.64191E-06	1.01314E-06	1.24709E-06	1.00927E-06	8.80231E-07	7.92154E-07	1.74838E-06	1.56792E-06	7.21909E-07	9.45727E-07	9.62364E-07	1.35636E-06	6.166E-07	1.85467E-06	2.17153E-06	1.41053E-06
Clostridium_carboxidivorans	1.67664E-06	1.36836E-06	7.17436E-07	1.62227E-06	1.33846E-06	8.26154E-07	1.13054E-06	2.99215E-06	0.000002614	1.69582E-06	2.30727E-06	2.31455E-06	1.5324E-06	2.3274E-06	2.42267E-06	1.8032E-06
Clostridium_cochlearium	1.44164E-06	9.14818E-07	1.05205E-06	1.63382E-06	0.000001786	7.60462E-07	1.58977E-06	3.31792E-06	1.08345E-06	2.33036E-06	1.82018E-06	1.48818E-06	2.73807E-06	4.17967E-06	0.000004646	0.00000403
Clostridium_tyrobutyricum	4.33555E-06	1.62536E-06	1.26871E-06	0.00000165	7.77623E-06	6.21692E-07	0.000001615	4.08892E-06	1.70782E-06	1.69355E-06	0.00000303	2.65673E-06	5.56233E-06	5.90867E-06	6.91847E-06	4.69973E-06
Clostridium_isatidis	2.05027E-06	7.76045E-07	1.22995E-06	2.07664E-06	3.24954E-06	6.86462E-07	2.90446E-06	4.28238E-06	2.94091E-06	2.37055E-06	3.48591E-06	5.10736E-06	2.04653E-06	2.79667E-06	2.88913E-06	3.8718E-06
Clostridium_beijerinckii	3.62227E-06	1.85136E-06	9.47391E-07	2.15509E-06	1.94023E-06	9.12923E-07	0.000004428	4.41069E-06	3.00791E-06	0.000003811	4.43273E-06	4.76145E-06	2.4126E-06	3.81193E-06	0.000002504	2.23607E-06
Clostridium_baratii	3.56636E-06	1.55291E-06	1.69505E-06	2.59727E-06	1.41462E-06	2.11185E-06	4.11385E-06	3.92062E-06	2.95664E-06	0.000003269	4.72973E-06	4.19545E-06	2.26927E-06	4.7928E-06	4.68993E-06	0.000003992
Clostridium_saccharoperbutylacetonicum	7.77364E-06	2.38886E-06	1.10559E-06	2.21773E-06	0.000004067	0.000002896	4.99008E-06	7.78154E-06	5.82436E-06	9.75545E-06	1.10765E-05	6.86818E-06	3.89173E-06	5.22493E-06	5.37533E-06	5.56067E-06
Clostridium_estertheticum	5.64091E-06	0.000001091	1.07127E-06	0.00000337	0.00000743	1.09254E-06	5.66238E-06	6.16615E-06	7.65182E-06	7.27727E-06	7.47636E-06	6.25818E-06	5.58533E-06	0.000007118	7.47133E-06	0.000009636
Clostridium_tetani	7.08182E-06	0.000005326	3.31645E-06	7.29955E-06	5.92538E-06	2.11042E-05	0.00000754	5.72462E-06	4.55436E-06	6.24636E-06	0.00001455	7.77182E-06	1.10427E-05	1.19093E-05	1.26876E-05	0.000014958
Clostridium_thermarum	8.43545E-06	5.87282E-06	1.74757E-06	6.13364E-06	7.09385E-06	3.50846E-06	8.12238E-06	3.93492E-06	7.61418E-06	8.40364E-06	6.46182E-06	9.65182E-06	5.75867E-06	6.39173E-06	0.000004998	5.36867E-06
Clostridium_perfringens	0.00001337	1.09486E-05	4.56455E-06	8.65636E-06	9.36923E-06	8.85685E-06	1.32692E-05	1.51242E-05	0.00000966	1.51991E-05	1.71482E-05	1.49618E-05	9.05267E-06	0.000012302	1.07893E-05	8.97333E-06
Clostridium_butyricum	1.13355E-05	6.59773E-06	2.68482E-06	7.77682E-06	7.16262E-06	2.83869E-06	8.50846E-06	9.67846E-06	0.00001208	1.47336E-05	1.78218E-05	1.34591E-05	0.000009026	1.16607E-05	1.24173E-05	1.26047E-05
Clostridium_botulinum	2.27282E-05	1.02391E-05	7.60191E-06	1.23364E-05	1.44335E-05	1.29909E-05	2.00662E-05	2.01769E-05	1.59691E-05	1.89436E-05	2.35545E-05	2.44682E-05	1.28033E-05	1.92453E-05	0.000017406	1.98553E-05
Clostridium_pasteurianum	0.000113313	7.75965E-05	2.51375E-05	2.99636E-05	3.39392E-05	2.22837E-05	5.51378E-05	4.94715E-05	8.21865E-05	0.000132114	8.51183E-05	0.000069122	0.000034544	3.58667E-05	2.98633E-05	3.85254E-05
Clostridium_kluyveri	3.94891E-05	3.67386E-05	1.38255E-05	3.89704E-05	1.54262E-05	1.24251E-05	1.99446E-05	1.61823E-05	7.75305E-05	7.74341E-05	3.85297E-05	3.15518E-05	1.44173E-05	2.09767E-05	0.000016142	1.55907E-05
Clostridium_sp._JN-1	2.45991E-06	5.77173E-06	9.72818E-07	2.52273E-06	1.68485E-06	2.16046E-06	1.90385E-06	3.42738E-06	1.85773E-06	2.09082E-06	2.42727E-06	4.76191E-06	1.3238E-06	2.2654E-06	2.4576E-06	2.84233E-06
Clostridium_sp._AWRP	4.04727E-06	2.32727E-06	1.38473E-06	2.35418E-06	2.05615E-06	5.87608E-06	2.29062E-06	2.13162E-06	0.000003197	4.12564E-06	0.00000381	8.31364E-06	1.3978E-06	2.1646E-06	2.7706E-06	3.38353E-06
Clostridium_sp._'deep_sea'	1.26864E-06	7.76591E-07	6.49364E-07	1.96345E-06	1.72123E-06	1.62762E-06	1.14869E-06	2.40592E-06	0.000001951	2.43836E-06	0.00000375	3.29364E-06	0.000002009	1.6154E-06	3.43987E-06	3.54353E-06
Clostridium_sp._JN-9	4.22455E-06	2.13773E-06	1.63764E-06	2.49291E-06	1.53292E-06	1.21931E-06	3.22923E-06	4.13954E-06	4.60818E-06	3.74909E-06	3.11355E-06	4.40818E-06	1.71827E-06	3.3534E-06	2.9238E-06	2.23787E-06
Clostridium_sp._CT4	3.72336E-06	4.92727E-06	1.66582E-06	3.04855E-06	0.000001799	4.39292E-06	3.15046E-06	4.57115E-06	2.82809E-06	3.15791E-06	4.32764E-06	3.24136E-06	1.36873E-06	3.20753E-06	2.60767E-06	2.24127E-06
Clostridium_sp._DL-VIII	4.08909E-06	1.51355E-06	1.43091E-06	2.20273E-06	2.52331E-06	5.01154E-07	3.87531E-06	2.94931E-06	2.75464E-06	2.71709E-06	0.000003743	4.73182E-06	1.7978E-06	3.2878E-06	3.35933E-06	1.8234E-06
Clostridium_sp._BNL1100	8.54909E-06	4.18091E-06	2.32609E-06	6.01909E-06	4.57946E-06	2.24854E-06	7.90692E-06	7.20692E-06	7.46545E-06	8.68455E-06	8.84364E-06	0.00000974	2.45933E-06	5.7908E-06	4.76533E-06	0.000005116
Clostridium_sp._SY8519	0.000164059	0.000121004	4.04311E-05	0.000092874	0.000112112	0.000157533	0.000116229	9.67659E-05	0.000171335	0.000233184	0.000132642	0.000175993	9.35403E-05	0.000110729	9.30083E-05	8.88865E-05
Dysosmobacter_welbionis	0.000384571	0.000132363	7.05873E-05	0.000211853	0.000229701	0.00028042	0.000204585	0.000221182	0.000450659	0.000328251	0.000377366	0.000442729	0.000175347	0.000223823	0.000201463	0.000182606
Oscillibacter_valericigenes	0.00049051	0.0001691	0.000099708	0.000258805	0.000257214	0.000271423	0.000247048	0.000305368	0.000599515	0.000348631	0.000359369	0.00049512	0.000213017	0.000263651	0.000229444	0.000204957
Oscillibacter_sp._PEA192	9.26345E-05	3.84569E-05	2.15037E-05	4.77117E-05	5.03664E-05	5.30635E-05	4.63357E-05	6.03897E-05	0.000118494	6.43937E-05	7.07506E-05	0.000093744	0.000043406	0.000052632	4.88091E-05	3.99752E-05
Oscillibacter_sp._NSJ-62	0.001132627	0.000537938	0.000239314	0.000624938	0.000562632	0.001011526	0.00052301	0.000611665	0.001308312	0.000794326	0.000810082	0.001110114	0.000485026	0.000625326	0.000547713	0.000477281
Carboxydocella_thermautotrophica	1.20445E-06	1.00836E-06	3.05636E-07	1.80955E-06	9.56462E-07	2.34638E-06	1.17762E-06	1.08515E-06	1.15445E-06	5.52909E-07	1.09536E-06	1.62245E-06	0.000000604	1.00447E-06	6.89867E-07	1.11747E-06
Thermaerobacter_marianensis	2.77745E-06	1.22736E-06	9.10727E-07	1.68709E-06	8.09769E-07	2.50523E-06	0.00000087	1.70085E-06	0.000001792	1.54545E-06	1.48591E-06	2.41809E-06	5.65467E-07	1.00573E-06	9.74267E-07	1.10867E-06
Thermaerobacter_sp._FW80	2.36245E-06	1.24936E-06	2.65741E-06	2.66727E-06	7.85692E-07	1.32354E-06	2.40423E-06	2.31177E-06	1.51409E-06	1.75109E-06	2.38273E-06	4.18682E-06	4.58333E-07	9.216E-07	1.0782E-06	9.77733E-07
Thermaerobacter_sp._PB12/4term	2.06245E-06	1.11264E-06	7.25182E-07	1.90964E-06	1.11185E-06	1.72785E-06	1.69731E-06	1.20308E-06	1.54218E-06	1.19991E-06	1.24127E-06	2.68809E-06	1.1274E-06	1.34527E-06	2.31707E-06	1.03847E-06
Monoglobus_pectinilyticus	0.00001743	1.28572E-05	4.61195E-06	1.25073E-05	1.48785E-05	2.36508E-05	1.32685E-05	1.34215E-05	1.58118E-05	1.94864E-05	1.84227E-05	2.23855E-05	1.34713E-05	0.000019946	0.000016728	1.16247E-05
Mogibacterium_pumilum	2.36709E-06	2.96745E-06	7.05364E-07	2.27464E-06	2.31608E-06	2.09162E-06	2.22923E-06	1.13715E-06	1.38855E-06	1.89073E-06	0.000002766	2.83227E-06	0.000001673	0.000003176	1.74833E-06	1.29613E-06
Mogibacterium_diversum	4.62727E-06	3.77773E-06	1.67018E-06	4.08864E-06	0.000003276	0.000002886	5.97231E-06	3.44254E-06	2.13373E-06	2.41273E-06	3.58382E-06	5.91364E-06	3.98393E-06	4.41613E-06	3.28807E-06	2.99787E-06
Aminipila_butyrica	1.24409E-05	1.18345E-05	7.18909E-06	7.83091E-06	3.89815E-06	9.47923E-06	6.20538E-06	5.41538E-06	6.32818E-06	0.00000623	6.77818E-06	8.48091E-06	4.22067E-06	5.24467E-06	4.97267E-06	3.3504E-06
Aminipila_sp._JN-18	2.42291E-05	2.01865E-05	9.12818E-06	1.90745E-05	9.94692E-06	2.26935E-05	1.58038E-05	1.72415E-05	2.02773E-05	1.83473E-05	2.40536E-05	2.97991E-05	0.000009402	1.20747E-05	1.12573E-05	1.04713E-05
Aminipila_sp._CBA3637	1.11673E-05	8.30355E-06	3.51264E-06	6.09364E-06	1.06162E-05	9.74077E-06	0.00000683	9.23231E-06	1.24627E-05	1.07273E-05	1.26418E-05	1.57264E-05	5.78867E-06	0.000008138	0.000008658	0.000007198
Massilistercora_timonensis	0.000647127	0.000642466	0.000178633	0.000382209	0.000428704	0.00080847	0.000474152	0.000397654	0.000643703	0.000813073	0.000537849	0.000672297	0.000345839	0.000462667	0.000385696	0.000346876
Intestinimonas_butyriciproducens	0.000783441	0.000439558	0.000173723	0.000527705	0.000512027	0.001150138	0.000437368	0.000496556	0.000878378	0.000796569	0.000769884	0.001014829	0.000422022	0.000585535	0.000493201	0.000430016
Flintibacter_sp._KGMB00164	0.001413457	0.000911271	0.000327254	0.000914338	0.000895924	0.003784714	0.000916352	0.000981797	0.001523784	0.001346315	0.001261374	0.001770415	0.000764603	0.000971949	0.000895004	0.000783826
Ethanoligenens_harbinense	3.53364E-05	2.40239E-05	1.29632E-05	3.13364E-05	1.83669E-05	3.11411E-05	2.98362E-05	0.00002671	3.37909E-05	3.11182E-05	3.06909E-05	4.80518E-05	1.71433E-05	2.35907E-05	2.07107E-05	2.12153E-05
Caproiciproducens_sp._NJN-50	3.53182E-05	1.74042E-05	9.12818E-06	2.18645E-05	1.44838E-05	2.67969E-05	1.76762E-05	2.14177E-05	3.65736E-05	2.11127E-05	2.75682E-05	4.26218E-05	0.00001391	0.000016186	1.61407E-05	1.21733E-05
Caproiciproducens_sp._7D4C2	4.97185E-05	3.04477E-05	1.30327E-05	4.08994E-05	3.29308E-05	5.77666E-05	0.000036066	3.60146E-05	0.00005258	5.06364E-05	5.46593E-05	7.44875E-05	2.91947E-05	3.73043E-05	0.00003348	3.04339E-05
Ruminococcus_albus	2.35636E-05	2.04369E-05	1.01574E-05	1.48173E-05	1.21415E-05	1.40588E-05	1.87431E-05	1.84123E-05	3.54227E-05	2.05536E-05	3.44842E-05	3.42705E-05	0.000008898	0.000011946	1.14507E-05	1.07073E-05
Ruminococcus_sp._JE7A12	1.53818E-05	1.02985E-05	3.99845E-06	1.06655E-05	6.96692E-06	7.31915E-06	0.00001086	9.75846E-06	1.78773E-05	1.04636E-05	1.92227E-05	4.56155E-05	1.68826E-05	9.34733E-06	9.30067E-06	0.000008818
Ruminococcus_bicirculans	3.05818E-05	1.91412E-05	9.79636E-06	3.80909E-05	0.00002697	2.67592E-05	3.59509E-05	3.97462E-05	6.16629E-05	0.0000309	6.51592E-05	4.43364E-05	2.27913E-05	3.34127E-05	2.73227E-05	0.000023052
Ruminococcus_champanellensis	6.27048E-05	4.93753E-05	2.17057E-05	4.97398E-05	3.69077E-05	3.99875E-05	3.79675E-05	3.87563E-05	0.00017334	5.80824E-05	0.000178639	0.000110987	0.00004182	4.12235E-05	0.00003544	0.00003246
Ruthenibacterium_lactatiformans	0.000265218	0.0001937	6.89035E-05	0.000752493	0.000257	0.0001087	0.000149824	0.000335358	0.000198937	0.000168935	0.00019147	0.000221015	0.000223762	0.000275735	0.000286139	0.000390467
Faecalibacterium_prausnitzii	0.001551444	0.001220889	0.000405454	0.000855611	0.0008233	0.001072277	0.000937384	0.000899307	0.001433068	0.001833027	0.001230467	0.001501824	0.00070428	0.000943125	0.000751174	0.00070336
Flavonifractor_plautii	0.002898753	0.00203478	0.000604648	0.001854728	0.001840656	0.004622069	0.001615081	0.001779282	0.003122773	0.002902971	0.002722438	0.003668463	0.001515872	0.002121985	0.001757358	0.001520359
Acutalibacter_muris	0.011996653	0.007812326	0.002637852	0.007492774	0.007613117	0.009869133	0.00718178	0.005845681	0.011235354	0.011279357	0.010478208	0.012197504	0.006241902	0.007983858	0.006666386	0.005713904
Cellulosilyticum_lentocellum	1.02973E-05	3.73845E-06	2.62109E-06	5.99091E-06	8.48692E-06	4.37585E-06	8.65231E-06	4.59569E-06	1.29936E-05	1.15927E-05	7.54182E-06	9.06818E-06	4.67093E-06	5.21907E-06	3.89687E-06	3.6472E-06
Cellulosilyticum_sp._WCF-2	2.61382E-06	2.95182E-06	0.000000345	2.66091E-06	0.000003277	1.53962E-06	2.20546E-06	2.64208E-06	2.09055E-06	2.31155E-06	2.50955E-06	3.24555E-06	2.31433E-06	7.78933E-06	0.00000348	1.81373E-06
Pseudobutyrivibrio_xylanivorans	5.36585E-05	3.62766E-05	1.53751E-05	3.46364E-05	3.90289E-05	2.55178E-05	4.51382E-05	3.29631E-05	5.34858E-05	5.07593E-05	4.69852E-05	0.000065335	0.000023102	4.31565E-05	3.28155E-05	2.68267E-05
Lachnoanaerobaculum_umeaense	0.000155641	0.000107696	2.81536E-05	4.15345E-05	5.86792E-05	4.01435E-05	9.47248E-05	7.78654E-05	0.000173809	0.000282022	0.000135109	0.000107285	5.92879E-05	5.28768E-05	4.47565E-05	7.93553E-05
Herbinix_luporum	9.37879E-05	5.18458E-05	1.75327E-05	4.20455E-05	5.50223E-05	5.67434E-05	6.26769E-05	5.38473E-05	0.000104849	0.000149957	8.30442E-05	8.95028E-05	3.93065E-05	5.49643E-05	4.21378E-05	4.76665E-05
Anaerotignum_propionicum	0.000115951	5.70867E-05	2.44373E-05	4.44752E-05	9.18997E-05	6.22499E-05	7.57053E-05	6.22498E-05	0.000111198	0.000145882	0.000106373	9.89246E-05	6.77711E-05	7.54521E-05	0.000110732	8.15764E-05
Lacrimispora_saccharolytica	0.000101503	0.000106188	2.95285E-05	0.000070387	4.30157E-05	0.000063298	6.16393E-05	5.76843E-05	8.44015E-05	0.000104493	7.34619E-05	0.000101898	3.47587E-05	5.10334E-05	0.00004358	4.02985E-05
Lacrimispora_sphenoides	9.51224E-05	8.38968E-05	2.71615E-05	6.27545E-05	4.74858E-05	5.98615E-05	6.70407E-05	5.82314E-05	8.88096E-05	0.000116388	8.34965E-05	9.56911E-05	0.000037416	5.21839E-05	4.48485E-05	4.56872E-05
Lachnospira_eligens	0.000241858	0.000149198	6.58665E-05	0.000102239	0.000147207	0.000113218	0.000183182	0.000175481	0.000272436	0.000342735	0.000233769	0.000207086	0.000122181	0.000171598	0.000132244	0.000133841
Anaerocolumna_cellulosilytica	3.66182E-05	2.97495E-05	1.15064E-05	2.47882E-05	0.00002449	1.98731E-05	0.000024486	2.05638E-05	3.51827E-05	4.02984E-05	3.53983E-05	4.83645E-05	1.64387E-05	2.14113E-05	1.92407E-05	1.71453E-05
Anaerocolumna_sp._CTTW	6.40495E-05	7.71679E-05	1.63106E-05	3.01655E-05	3.46654E-05	2.79062E-05	3.92353E-05	3.62908E-05	0.000059128	8.42935E-05	6.69935E-05	6.33289E-05	0.00002652	4.19983E-05	3.22606E-05	3.16679E-05
Anaerocolumna_sp._CBA3638	8.44777E-05	4.25225E-05	1.61145E-05	3.95364E-05	3.89665E-05	0.00002431	5.50056E-05	5.18668E-05	0.00008787	0.000124202	7.83741E-05	7.38909E-05	3.29421E-05	0.000042913	4.09691E-05	4.96297E-05
Anaerobutyricum_hallii	0.000494455	0.000355901	0.000107081	0.000250908	0.000199469	0.00026494	0.000269929	0.000227837	0.000647045	0.000908382	0.000452313	0.000379989	0.000202718	0.000214252	0.000191972	0.000252249
Anaerostipes_rhamnosivorans	6.32666E-05	6.11748E-05	0.000100917	0.00012656	4.70913E-05	5.28763E-05	4.70651E-05	3.83538E-05	5.96292E-05	0.000079844	5.54525E-05	6.98723E-05	3.03407E-05	4.81493E-05	4.63125E-05	4.29732E-05
Anaerostipes_caccae	8.73099E-05	9.17303E-05	0.000609832	0.00061159	5.64775E-05	9.92954E-05	6.09556E-05	0.000058881	8.13214E-05	0.000100636	7.87833E-05	0.000100407	4.05399E-05	5.47582E-05	4.83643E-05	4.76079E-05
Anaerostipes_hadrus	0.000249561	0.000127879	4.55624E-05	7.64415E-05	0.000120197	0.000096621	0.000128364	0.000115622	0.000265046	0.000455973	0.000224519	0.000211379	0.00010215	0.000119851	9.98635E-05	0.00013343
[Ruminococcus]_gnavus	0.000482464	0.00049506	0.000158608	0.00026393	0.00022115	0.000389664	0.000303265	0.00027692	0.000393225	0.000459516	0.000347674	0.000400548	0.000202231	0.000298953	0.000221834	0.000183843
Butyrivibrio_hungatei	5.23117E-05	2.63603E-05	1.3578E-05	2.62027E-05	2.74154E-05	3.73216E-05	3.42164E-05	3.28615E-05	5.04007E-05	7.62098E-05	4.36702E-05	6.23029E-05	2.08253E-05	2.58213E-05	2.17367E-05	2.62375E-05
Butyrivibrio_proteoclasticus	5.13904E-05	0.000039924	1.3071E-05	2.57815E-05	4.28885E-05	2.30609E-05	3.97572E-05	3.28138E-05	4.94422E-05	5.84696E-05	4.27273E-05	5.70599E-05	0.00003794	4.16265E-05	0.00003532	3.43533E-05
Butyrivibrio_fibrisolvens	0.000160328	9.59738E-05	3.06295E-05	7.15302E-05	9.97806E-05	7.03283E-05	0.000129976	7.84632E-05	0.000185931	0.000240319	0.000129185	0.00013055	7.27122E-05	8.55252E-05	6.38498E-05	7.76455E-05
Roseburia_hominis	0.000556352	0.00059689	0.000162594	0.000388057	0.000431241	0.000415392	0.000473961	0.000346497	0.000470669	0.000620143	0.000462337	0.000614974	0.000288943	0.000420864	0.0003353	0.000284494
Roseburia_intestinalis	0.000885278	0.000832962	0.000247086	0.000531686	0.000677538	0.000637681	0.000710718	0.000523589	0.000880815	0.001082745	0.000719273	0.001007264	0.000462011	0.000650959	0.000495332	0.000445005
Blautia_coccoides	0	5.08182E-07	0.000000312	2.79091E-07	2.26923E-08	1.74615E-07	0	0	0	0	1.02727E-08	0	5.36E-08	0	0	3.67333E-08
Blautia_sp._YL58	1.08082E-06	5.38227E-06	3.62673E-06	2.66118E-06	0	0	1.13077E-07	1.96692E-07	0.000000658	7.49182E-07	4.54636E-07	0.000000371	0	0	1.12667E-08	1.66667E-08
Blautia_argi	0.000334771	0.000476747	0.000106557	0.000169821	0.000133879	0.000276206	0.000192048	0.000179424	0.000207389	0.000260569	0.00022955	0.000288814	9.89111E-05	0.000142107	0.000114875	9.84807E-05
Blautia_hansenii	0.000301574	0.000215209	7.38259E-05	0.000154238	0.000129694	0.000158294	0.000172494	0.000144557	0.00024357	0.000344791	0.000259613	0.000281369	0.000091143	0.000130195	0.000112908	0.000110041
Blautia_sp._LZLJ-3	0.000110676	8.10975E-05	3.08799E-05	6.16173E-05	6.10922E-05	8.20577E-05	8.26499E-05	7.30358E-05	0.00012765	0.000183205	9.61926E-05	0.000118673	5.79954E-05	7.09725E-05	5.60159E-05	6.51715E-05
Blautia_sp._SC05B48	0.000358311	0.000307163	0.000109729	0.000208059	0.000159278	0.000267	0.000224767	0.000242254	0.000290287	0.00040146	0.000312259	0.000322893	0.000121672	0.000164183	0.00016112	0.000164645
Blautia_producta	0.002375855	0.003458674	0.001049111	0.001434329	0.001062008	0.003106935	0.001516858	0.001394558	0.001819736	0.002390149	0.001754941	0.002206009	0.000825759	0.001200883	0.001001548	0.000909767
Enterocloster_clostridioformis	0.000913494	0.000978243	0.000259348	0.000438616	0.000480206	0.000523021	0.000530065	0.000473924	0.000890354	0.001239103	0.000684842	0.00074067	0.000399356	0.000523903	0.000398273	0.000403831
Enterocloster_bolteae	0.007006538	0.005853909	0.001726201	0.004156142	0.004903366	0.005046323	0.004259816	0.004139282	0.008297972	0.009898365	0.005794968	0.007949083	0.003754884	0.005111855	0.003856881	0.003670982
Lachnoclostridium_phytofermentans	8.70642E-05	4.26977E-05	1.93018E-05	4.21621E-05	5.04242E-05	4.82886E-05	6.21083E-05	5.01036E-05	8.77004E-05	0.00010869	8.59748E-05	8.54777E-05	4.00595E-05	5.89948E-05	4.55707E-05	4.38237E-05
[Clostridium]_hylemonae	0.000212241	0.000385617	7.44481E-05	0.00012921	0.000113242	0.000467447	0.000177094	0.000149222	0.000186667	0.000263845	0.000179761	0.000217496	9.37997E-05	0.00013664	0.000116603	0.000106984
[Clostridium]_scindens	0.001863562	0.002542513	0.000574733	0.001201607	0.001177523	0.002111811	0.001193495	0.001035227	0.001623327	0.00203286	0.001417703	0.002145705	0.000893382	0.00121981	0.001069373	0.000911151
Lachnoclostridium_phocaeense	0.002023532	0.001678459	0.000491939	0.000957116	0.001305354	0.001554932	0.001446027	0.001147298	0.002105642	0.003252304	0.001697244	0.001945294	0.000994605	0.001402494	0.001112986	0.0010508
Lachnoclostridium_sp._YL32	0.01354607	0.013503917	0.003794938	0.007768246	0.008627126	0.011046005	0.00904931	0.007427111	0.013161368	0.017641698	0.010813486	0.014010761	0.006282837	0.009223585	0.006764546	0.006498915
Candidatus_Cloacimonas_acidaminovorans	1.63018E-06	1.125E-07	8.50909E-08	1.24427E-07	4.71538E-07	1.06154E-07	1.36823E-06	9.33769E-07	1.18036E-06	1.91009E-06	1.30882E-06	0.000000573	4.72733E-07	9.002E-07	5.06667E-07	1.2686E-06
Gemmatimonas_phototrophica	7.25455E-08	2.34545E-08	4.12727E-09	0	1.15154E-07	6.76923E-08	6.23077E-08	8.95385E-08	0	1.86909E-07	1.97727E-07	2.21636E-07	2.64667E-08	3.09333E-08	1.93733E-08	2.346E-07
Gemmatimonas_sp._TET16	7.79727E-07	7.53636E-08	2.96491E-07	1.02555E-06	4.65462E-07	2.41231E-07	0.000001263	6.34692E-07	0.000000851	2.76818E-07	5.55818E-07	4.16455E-07	5.23067E-07	5.804E-07	3.34867E-07	5.17267E-07
Gemmatimonas_aurantiaca	2.99909E-06	0.000000065	7.40909E-08	1.38473E-06	2.92231E-07	6.76923E-08	4.32615E-07	1.86631E-06	1.11664E-06	1.03564E-06	9.20909E-07	9.78636E-07	1.3298E-06	0.000000463	1.06187E-06	1.57573E-06
Gemmatirosa_kalamazoonesis	0.000005704	0.000000654	5.31664E-07	3.37173E-06	3.55308E-06	1.59738E-06	3.05085E-06	3.35285E-06	3.82682E-06	3.58055E-06	1.18291E-05	3.64645E-06	1.5884E-06	2.0556E-06	4.4362E-06	2.25553E-06
Fibrobacter_succinogenes	1.36618E-05	4.99091E-07	3.59694E-06	8.93364E-06	0.00001233	1.42692E-06	5.74692E-06	5.77462E-06	1.47727E-05	0.00001425	0.00001032	9.79909E-06	1.87133E-05	0.000015404	1.29147E-05	0.00001275
Candidatus_Cyclonatronum_proteinivorum	2.05418E-06	1.23182E-07	6.49482E-07	1.19636E-06	1.47677E-06	0.000005122	1.70154E-06	2.33615E-06	2.38064E-06	1.92455E-06	1.78491E-06	2.23673E-06	0.00000256	0.00000237	0.000002066	2.79067E-06
Melioribacter_roseus	1.23873E-06	4.06364E-07	1.42309E-07	5.59818E-07	1.07438E-06	2.86769E-07	8.96154E-07	1.41031E-06	1.28845E-06	0.000000636	1.20727E-06	0.00000174	1.0176E-06	1.0016E-06	8.896E-07	8.05733E-07
Ignavibacterium_album	1.18518E-06	2.38818E-06	4.02855E-07	6.73545E-07	1.60015E-06	0	1.01385E-06	7.15385E-07	4.20727E-07	4.11182E-07	0.000001199	0.00000135	4.43867E-07	1.03067E-06	2.15467E-06	1.92987E-06
Chloroherpeton_thalassium	8.27818E-07	3.29136E-07	1.57073E-07	5.81909E-07	2.83692E-07	1.13923E-07	2.50538E-07	4.73154E-07	0.000000284	2.67909E-07	3.61727E-07	3.81636E-07	4.57867E-07	5.00333E-07	2.6184E-07	4.11267E-07
Prosthecochloris_sp._CIB_2401	3.51909E-07	2.15909E-07	3.66091E-07	4.41364E-07	4.14308E-07	2.95385E-08	2.63077E-08	4.48462E-07	3.69909E-07	2.63182E-07	7.17364E-07	7.53818E-07	2.88467E-07	1.43667E-07	3.13267E-07	4.59533E-07
Prosthecochloris_sp._HL-130-GSB	1.21818E-07	2.65455E-07	3.91818E-07	3.48091E-07	0	1.79231E-08	1.25615E-07	4.14538E-07	1.71545E-07	0	2.53455E-07	1.83909E-07	7.75333E-08	7.74E-08	1.33333E-08	9.43333E-08
Prosthecochloris_aestuarii	3.97182E-07	3.28182E-08	2.56245E-07	1.14455E-06	9.81538E-08	9.82077E-07	1.54846E-06	1.57377E-06	5.81636E-07	5.38727E-07	9.15545E-07	9.76091E-07	3.20467E-07	4.59067E-07	1.726E-07	3.832E-07
Prosthecochloris_sp._GSB1	9.48091E-07	3.85636E-07	4.36818E-07	1.86791E-06	0.000000516	1.41538E-07	5.43154E-07	1.23669E-06	1.45009E-06	1.31782E-06	1.53509E-06	1.16027E-06	0.000000916	9.33867E-07	9.354E-07	8.40267E-07
Pelodictyon_phaeoclathratiforme	6.97636E-07	1.24273E-07	4.10764E-07	7.59364E-07	2.57385E-07	1.26846E-07	1.50769E-07	3.15923E-07	5.85636E-07	0.000001013	3.78455E-07	5.43364E-07	3.22667E-07	5.82267E-07	4.17533E-07	2.86133E-07
Pelodictyon_luteolum	2.38855E-06	1.72727E-07	0.00000307	4.29155E-06	1.85862E-06	0	0.00000956	7.79308E-06	3.40255E-06	2.37782E-06	2.62155E-06	3.35727E-06	2.65253E-06	2.51267E-06	0.000002746	3.44927E-06
Chlorobium_limicola	8.09182E-07	5.65455E-07	6.08727E-07	4.52455E-07	6.20923E-07	7.22769E-07	1.17731E-06	1.03846E-06	0.000001359	7.73545E-07	1.12682E-06	1.16809E-06	3.37667E-07	5.61667E-07	0.000000679	1.19527E-06
Chlorobium_phaeovibrioides	2.41009E-06	0.000000614	6.78273E-07	3.94545E-07	7.91462E-07	0.000000696	1.73285E-06	1.17885E-06	2.83091E-06	2.23582E-06	3.00009E-06	1.56655E-06	6.254E-07	2.15333E-07	4.87067E-07	1.8002E-06
Chlorobium_phaeobacteroides	3.71455E-06	1.34964E-06	7.62636E-07	3.84727E-06	1.63685E-06	4.55385E-07	2.37292E-06	1.29431E-06	0.000002357	1.79455E-06	1.89273E-06	2.18164E-06	1.66307E-06	1.27393E-06	2.05413E-06	1.96007E-06
Chlorobium_chlorochromatii	6.99636E-07	0.00000026	2.58945E-07	3.54673E-06	5.43692E-06	3.38154E-07	2.52715E-06	3.20169E-06	4.85273E-07	6.31545E-07	1.99082E-06	3.12082E-06	5.7798E-06	6.34093E-06	8.49933E-06	0.000008208
Chlorobaculum_tepidum	1.84845E-06	2.38455E-07	0.000000271	1.56109E-06	7.76154E-07	0	3.13154E-07	7.87923E-07	1.24973E-06	8.68364E-07	7.39455E-07	0.000001206	3.39067E-07	1.0538E-06	7.77333E-07	1.3122E-06
Chlorobaculum_limnaeum	1.57655E-05	6.42636E-07	5.33436E-06	1.51141E-05	3.82677E-06	0.000000403	1.56354E-05	1.18181E-05	8.22636E-06	1.43573E-05	8.53909E-06	9.92636E-06	3.6944E-06	4.78247E-06	4.51313E-06	5.22867E-06
Chlorobaculum_parvum	1.44448E-05	6.17636E-07	7.14145E-06	7.70545E-06	6.09385E-06	8.52734E-05	2.17854E-05	2.33438E-05	1.20891E-05	7.21727E-06	9.65909E-06	9.10818E-06	7.27867E-06	0.000007968	1.01433E-05	8.97733E-06
Haliscomenobacter_hydrossis	1.88955E-06	1.33636E-07	8.98491E-07	1.90545E-06	6.78308E-07	0.000000322	5.69538E-07	8.93308E-07	1.51582E-06	5.16636E-07	6.94364E-07	5.60364E-07	1.2612E-06	7.57467E-07	9.68867E-07	1.8534E-06
Saprospira_grandis	0.000000295	0	6.18182E-08	3.20727E-07	2.71231E-07	1.79231E-08	4.64846E-07	6.56077E-07	3.20091E-07	2.04273E-07	0.000000883	8.07273E-07	1.91667E-07	3.84467E-07	3.46067E-07	4.644E-07
Salinibacter_ruber	1.74109E-06	1.14118E-06	0.000001381	3.04136E-06	3.75462E-07	3.44831E-06	5.31869E-06	3.79077E-06	1.16627E-06	9.41364E-07	2.60227E-06	1.98564E-06	1.0402E-06	1.05233E-06	1.23213E-06	9.778E-07

Rhodothermus_marinus	0.00000888	1.60441E-06	4.74603E-06	0.00000982	3.03769E-06	5.14215E-06	1.38969E-05	1.59938E-05	6.72182E-06	4.96318E-06	8.15364E-06	7.82091E-06	4.39213E-06	4.42667E-06	5.28533E-06	5.05267E-06
Panacibacter_ginsenosidivorans	3.09182E-07	1.16364E-08	4.24545E-08	2.87273E-08	2.88462E-08	6.10769E-08	1.06615E-07	0.000000237	1.01364E-07	3.62727E-08	1.05818E-07	2.11818E-07	5.09333E-08	1.222E-07	1.23267E-07	2.16533E-07
Niastella_koreensis	3.89773E-06	0.000000117	1.70337E-06	3.95273E-06	0.000000858	1.57692E-07	1.44331E-06	1.65823E-06	2.87164E-06	5.26273E-06	3.42727E-06	2.77855E-06	1.33533E-06	0.000001777	2.4004E-06	2.00713E-06
Lacibacter_sp._S13-6-6	2.80055E-06	1.45636E-06	1.37327E-06	0.00000305	9.45462E-07	2.95385E-08	9.34154E-07	2.18923E-06	2.66973E-06	2.79255E-06	2.14791E-06	2.88991E-06	6.03333E-07	1.14013E-06	0.000001101	1.33253E-06
Filimonas_lacunae	2.09636E-06	0	1.05104E-06	2.77827E-06	7.63308E-07	1.26077E-06	3.93523E-06	4.01462E-06	1.97155E-06	1.54936E-06	2.56027E-06	2.54518E-06	8.042E-07	1.22813E-06	8.514E-07	1.24653E-06
Niabella_soli	1.99945E-06	4.63909E-07	2.34468E-06	0.000001946	1.15823E-06	2.19385E-07	2.05854E-06	2.71277E-06	1.55236E-06	1.47536E-06	2.70545E-06	2.36536E-06	1.20733E-06	1.34347E-06	0.000001404	1.57733E-06
Niabella_ginsenosidivorans	2.69845E-06	3.20636E-07	8.61909E-07	3.15091E-06	6.42615E-07	1.35077E-07	4.72923E-07	1.83662E-06	2.79273E-06	1.75209E-06	2.58209E-06	3.44245E-06	1.2722E-06	7.96733E-07	1.2708E-06	1.71427E-06
Arachidicoccus_ginsenosidivorans	2.36091E-07	0	4.12727E-09	9.54545E-09	0.000000268	1.11615E-07	9.35615E-07	6.14231E-07	2.32364E-07	1.11273E-07	5.94455E-07	8.43545E-07	7.56E-08	1.54867E-07	1.27067E-07	2.424E-07
Arachidicoccus_soli	2.25509E-06	0.000000065	2.83273E-07	1.20836E-06	3.31615E-07	2.95385E-08	1.34692E-07	1.10923E-06	1.09509E-06	6.23364E-07	8.91545E-07	8.80364E-07	3.30933E-07	5.44333E-07	5.09133E-07	6.892E-07
Arachidicoccus_sp._BS20	5.21909E-07	3.64682E-07	0.000000156	1.00236E-06	6.49231E-08	2.13846E-08	4.33846E-08	1.08769E-07	4.19182E-07	3.14091E-07	2.34182E-07	2.79727E-07	8.12667E-08	3.48E-08	1.38133E-07	3.26733E-07
Arachidicoccus_sp._B3-10	1.07709E-06	2.68636E-07	1.26091E-06	2.46473E-06	9.65462E-07	0	1.30969E-06	1.11585E-06	0.000000677	5.74636E-07	1.15845E-06	1.46927E-06	1.22993E-06	1.20953E-06	1.60513E-06	2.2296E-06
Pseudobacter_ginsenosidimutans	5.68818E-06	1.14736E-06	3.76727E-06	3.99364E-06	1.36169E-06	2.21562E-06	2.06338E-06	4.95608E-06	4.71727E-06	3.26555E-06	5.11273E-06	3.83818E-06	2.33687E-06	0.000003215	2.71247E-06	3.63533E-06
Paraflavitalea_soli	4.09182E-06	3.33636E-07	3.97455E-06	6.72636E-06	0.000001347	1.08769E-07	4.52369E-06	6.77685E-06	3.94155E-06	3.44909E-06	4.62364E-06	4.86764E-06	0.000001842	2.62027E-06	2.86533E-06	0.000003636
Flavihumibacter_sp._SB-02	6.53636E-06	2.83091E-07	6.63918E-06	1.06818E-05	2.70769E-06	2.95385E-08	9.88846E-06	1.13269E-05	6.07091E-06	0.00000636	5.95336E-06	0.00000491	2.00607E-06	3.0656E-06	0.000003142	3.91353E-06
Flavisolibacter_ginsenosidimutans	1.56909E-06	7.66364E-08	1.02818E-07	5.78909E-07	6.92154E-07	0	4.32538E-07	9.71692E-07	4.14273E-07	4.19545E-07	5.77273E-07	3.92818E-07	7.95333E-07	7.48867E-07	6.278E-07	1.0262E-06
Flavisolibacter_tropicus	1.39909E-06	7.64545E-08	2.62545E-07	6.23636E-07	1.64069E-06	2.95385E-08	0	2.62769E-07	6.24545E-07	4.43727E-07	8.07636E-07	0.000000464	2.1336E-06	2.08693E-06	3.13773E-06	2.5398E-06
Flavisolibacter_sp._17J28-1	5.41573E-06	2.77545E-06	4.88182E-06	0.00000603	2.28015E-06	6.21385E-07	3.89723E-06	0.000003614	4.09973E-06	3.37273E-06	3.64091E-06	3.42818E-06	0.000004588	3.85233E-06	4.41873E-06	5.53467E-06
Chitinophaga_sp._1303	0	0	0	0	4.58154E-07	2.95385E-08	0	0	0	0	0	0	4.562E-07	5.246E-07	1.2338E-06	7.87467E-07
Chitinophaga_caeni	3.44509E-06	7.11727E-07	9.13282E-06	8.58364E-06	0.000003093	9.86154E-07	0.00002271	2.16017E-05	4.66882E-06	4.81736E-06	3.84818E-06	6.41727E-06	2.98047E-06	0.000002469	3.83933E-06	2.68573E-06
Chitinophaga_pinensis	0.00002381	2.12991E-06	1.70927E-05	2.55509E-05	1.22123E-05	6.36362E-06	3.12608E-05	3.47885E-05	2.24727E-05	0.00002159	2.02036E-05	1.34518E-05	1.35747E-05	1.45647E-05	1.52427E-05	1.32513E-05
Chitinophaga_sp._H33E-04	0.000016794	4.32909E-07	2.97882E-06	4.91436E-06	5.10631E-06	1.16123E-06	1.06474E-05	7.81615E-06	1.89609E-05	3.96155E-05	1.45873E-05	8.48545E-06	5.24187E-06	4.53307E-06	4.2432E-06	9.29687E-06
Chitinophaga_sp._XS-30	5.02818E-06	6.08318E-07	4.05545E-06	4.01091E-06	2.88923E-06	4.15262E-06	6.49077E-06	7.80308E-06	5.58636E-06	3.73364E-06	4.64364E-06	4.97364E-06	3.53527E-06	4.02733E-06	3.78133E-06	0.000004546
Chitinophaga_sp._MD30	2.71945E-06	4.65455E-08	7.82909E-07	1.00582E-06	1.54315E-06	4.19615E-07	3.73385E-07	5.63231E-07	3.07882E-06	2.40764E-06	1.91991E-06	3.09364E-06	2.4734E-06	2.21053E-06	2.32747E-06	3.13673E-06
Pseudopedobacter_saltans	2.84545E-06	1.59273E-06	1.15358E-06	2.09164E-06	3.92462E-07	0.00000053	1.51808E-06	1.17146E-06	1.41445E-06	1.19873E-06	1.96673E-06	2.28582E-06	4.54933E-07	7.39867E-07	6.18867E-07	8.434E-07
Olivibacter_sp._SDN3	2.44036E-06	3.57273E-07	1.47027E-06	1.16755E-06	5.35231E-07	0.000000189	6.35538E-07	6.60538E-07	0.000001466	7.05364E-07	1.69182E-06	2.13055E-06	6.71867E-07	9.16333E-07	1.29433E-06	1.10353E-06
Olivibacter_sp._LS-1	7.75364E-07	0	3.70909E-08	4.97818E-07	4.97154E-07	0	0	0	7.40455E-07	6.43273E-07	1.57491E-06	1.06755E-06	0.0000006	4.38933E-07	4.14133E-07	8.16933E-07
Solitalea_canadensis	1.07509E-06	2.13864E-07	4.12727E-09	2.28909E-07	9.77846E-07	2.95385E-08	6.83308E-07	4.28077E-07	1.68409E-06	1.00445E-06	9.72455E-07	1.33673E-06	1.3756E-06	1.23933E-06	1.2616E-06	1.3396E-06
Anseongella_ginsenosidimutans	3.12545E-06	2.79545E-07	2.18473E-06	0.00000459	1.29646E-06	7.52308E-07	1.85208E-06	3.17946E-06	2.71218E-06	0.000002487	3.28927E-06	2.76636E-06	1.4996E-06	2.05853E-06	2.71187E-06	0.00000317
Mucilaginibacter_mallensis	9.15455E-08	0	1.08636E-07	2.62909E-07	1.48308E-07	1.96462E-07	6.62462E-07	4.55692E-07	0.000000328	2.54636E-07	7.40364E-07	7.01364E-07	4.39333E-07	3.198E-07	8.13467E-07	6.56867E-07
Mucilaginibacter_ginsenosidivorax	0.000001281	0.00000013	4.02727E-07	0.000001911	5.00769E-07	9.76154E-08	1.52908E-06	1.09308E-06	1.28855E-06	0.000000357	8.06545E-07	6.27727E-07	7.52067E-07	5.726E-07	5.932E-07	6.488E-07
Mucilaginibacter_xinganensis	1.97309E-06	6.12364E-07	1.02745E-06	3.08382E-06	7.99154E-07	3.51692E-07	6.06923E-08	4.08538E-07	2.41627E-06	2.16309E-06	2.62645E-06	9.73545E-07	9.07333E-07	1.1464E-06	1.3436E-06	1.31133E-06
Mucilaginibacter_paludis	6.10636E-06	1.36673E-06	1.81136E-06	2.70273E-06	7.01538E-07	1.96154E-07	2.59531E-06	3.45654E-06	3.65255E-06	5.28473E-06	5.97218E-06	4.47136E-06	0.000001688	1.09233E-06	1.41627E-06	1.73693E-06
Mucilaginibacter_ginsenosidivorans	1.76709E-05	3.36182E-07	6.93745E-06	1.85555E-05	3.27538E-06	1.19762E-06	1.58977E-05	1.20885E-05	9.91673E-06	1.50627E-05	0.00001022	9.27945E-06	3.61267E-06	4.66067E-06	4.30513E-06	4.75853E-06
Mucilaginibacter_rubeus	1.64009E-06	6.52727E-08	5.75945E-07	1.21509E-06	1.04246E-06	1.75769E-07	2.17692E-07	0.000000481	1.57882E-06	1.88182E-06	1.14018E-06	2.20136E-06	2.3544E-06	2.2624E-06	0.000001672	2.1446E-06
Mucilaginibacter_gotjawali	2.48618E-06	1.94727E-07	6.44909E-07	7.42091E-07	2.52969E-06	0	1.08031E-06	2.59308E-06	2.72545E-06	1.74236E-06	2.58573E-06	3.50273E-06	3.6368E-06	0.000003257	0.000003198	0.000003816
Mucilaginibacter_sp._G2-14	0.00000211	6.66818E-07	0.000000682	1.21109E-06	5.16231E-07	8.35385E-08	3.15846E-07	6.39385E-07	6.63364E-07	0.000000779	1.54391E-06	5.88091E-07	9.39533E-07	8.54667E-07	1.10333E-06	1.48807E-06
Mucilaginibacter_sp._PAMC_26640	0.000000314	1.67909E-07	1.35909E-07	1.09082E-06	7.96154E-08	1.61254E-06	2.26538E-06	2.20477E-06	0.00000063	6.58364E-07	5.43455E-07	3.74182E-07	1.632E-07	2.16867E-07	1.84533E-07	1.992E-07
Mucilaginibacter_sp._HYN0043	2.39364E-06	4.11455E-07	1.62167E-06	1.98755E-06	5.96077E-07	4.27692E-08	2.92385E-06	3.35892E-06	1.23918E-06	1.56127E-06	1.22255E-06	7.91545E-07	7.616E-07	5.32133E-07	5.04867E-07	1.22713E-06
Mucilaginibacter_sp._14171R-50	1.48091E-07	0.000000065	5.43636E-08	3.12455E-07	1.48769E-07	0	0	1.36846E-07	1.95909E-07	0.000000283	5.96909E-07	6.20636E-07	5.426E-07	0.000000375	7.84467E-07	7.19933E-07
Mucilaginibacter_sp._F39-2	1.18309E-05	0.00000073	5.86091E-06	7.07973E-06	4.77508E-06	7.64251E-05	1.81054E-05	1.85746E-05	8.71545E-06	7.83818E-06	8.11909E-06	8.34455E-06	6.43933E-06	6.39067E-06	6.69733E-06	6.99333E-06
Pedobacter_steynii	7.67818E-07	1.23182E-07	1.39273E-07	7.98455E-07	9.91538E-08	0	0	4.23615E-07	4.20909E-07	8.48545E-07	8.72727E-07	1.19773E-06	2.366E-07	3.87733E-07	3.556E-07	5.45067E-07
Pedobacter_suwonensis	2.92773E-06	1.63545E-07	6.67818E-07	1.86164E-06	3.88231E-07	0	1.69054E-06	1.76069E-06	1.00891E-06	8.01182E-07	0.000001348	1.17764E-06	3.95267E-07	4.90533E-07	8.928E-07	3.60267E-07
Pedobacter_sp._SW-16	7.09727E-07	4.93636E-07	6.14182E-07	9.69364E-07	0.000000208	0	0.000000497	3.03154E-07	1.07918E-06	1.03773E-06	6.05364E-07	5.14636E-07	2.52733E-07	3.328E-07	2.66933E-07	1.01587E-06
Pedobacter_cryoconitis	4.93545E-07	8.84545E-08	4.12727E-09	1.00727E-07	5.34615E-08	2.95385E-08	1.41462E-07	2.63923E-07	3.63455E-07	7.55455E-08	1.82909E-07	2.06045E-07	1.23533E-07	3.58467E-07	1.31267E-07	0.000000183
Pedobacter_ginsengisoli	1.37973E-06	4.19727E-07	4.89273E-07	8.43455E-07	3.47462E-07	4.13846E-07	8.96462E-07	2.47692E-07	9.33273E-07	6.24455E-07	5.90273E-07	8.95182E-07	0.000000298	7.764E-07	3.25667E-07	2.51267E-07
Pedobacter_roseus	2.81727E-06	1.04582E-06	4.94636E-07	2.39809E-06	1.50485E-06	1.55738E-06	2.16254E-06	7.52385E-07	2.00227E-06	1.48227E-06	1.21155E-06	2.17818E-06	8.146E-07	6.01733E-07	0.000000985	6.15733E-07
Pedobacter_heparinus	5.31436E-06	5.79364E-07	1.48573E-05	0.0000176	5.69462E-06	2.66154E-07	3.88826E-05	3.51798E-05	8.93273E-06	1.14064E-05	8.22636E-06	6.56545E-06	5.64013E-06	5.53467E-06	9.50933E-06	0.000005712
Pedobacter_sp._HDW13	1.49827E-06	5.03636E-08	6.12491E-07	7.45273E-07	3.20769E-07	4.14385E-07	6.10615E-07	4.91692E-07	9.38909E-07	5.92091E-07	9.91909E-07	0.000000611	2.14733E-07	3.564E-07	2.58333E-07	4.98933E-07
Pedobacter_sp._KBS0701	1.03364E-07	4.11818E-08	1.56436E-07	1.06073E-06	5.74538E-07	0	2.73846E-08	4.93846E-08	1.23836E-06	1.17573E-06	1.66573E-06	1.79382E-06	4.58067E-07	0.000000931	6.854E-07	4.342E-07
Pedobacter_sp._CJ43	6.24455E-07	2.93182E-07	9.03091E-08	2.74091E-07	2.12154E-07	0	3.05154E-07	6.49308E-07	0.000000404	2.53182E-07	3.35455E-07	6.32727E-07	3.12733E-07	7.42733E-07	5.78067E-07	3.69533E-07
Pedobacter_sp._eg	1.25645E-06	0	5.91364E-07	9.62273E-07	0.000000788	3.48154E-07	1.18615E-06	2.94131E-06	1.46336E-06	8.50909E-07	2.22018E-06	2.63764E-06	7.84133E-07	1.20913E-06	1.43247E-06	1.00413E-06
Pedobacter_sp._PACM_27299	5.81455E-07	1.16364E-08	0	2.34273E-07	2.72385E-07	0.000000095	3.16769E-07	7.86538E-07	6.56364E-07	3.96091E-07	6.15364E-07	5.53545E-07	0.00000015	0.00000033	1.24733E-07	4.578E-07
Pedobacter_sp._G11	1.36345E-06	2.15909E-07	7.52545E-07	1.74864E-06	6.31385E-07	3.81692E-07	4.77692E-07	6.56308E-07	1.15655E-06	8.59727E-07	0.00000137	1.34945E-06	1.0898E-06	1.4742E-06	7.90867E-07	8.34867E-07
Pedobacter_sp._JBR3-12	9.21182E-06	5.65991E-06	4.95464E-06	6.32909E-06	5.38085E-06	3.28031E-06	8.39469E-06	3.84754E-06	7.83709E-06	9.34745E-06	4.32073E-06	4.56982E-06	3.11487E-06	5.36807E-06	2.31833E-06	0.000001908
Sphingobacterium_sp._G1-14	0	0	0	4.79091E-09	0	0	0	0	0	0	0	2.85455E-08	0	0.000000014	4.44E-09	0
Sphingobacterium_multivorum	5.82727E-08	3.00545E-07	3.27273E-08	1.55455E-08	0	4.92231E-07	1.08769E-07	0	0	0	0	3.48409E-07	1.03067E-07	0	0	0
Sphingobacterium_sp._21	0	0	8.75455E-09	0	5.47692E-08	0	0	1.10385E-07	0	0	8.02727E-08	1.08727E-07	2.72E-08	1.58E-08	1.12667E-08	4.95333E-08
Sphingobacterium_psychroaquaticum	7.78182E-07	0	5.01273E-08	3.15818E-07	2.71154E-07	3.23615E-07	1.95692E-07	1.69231E-07	2.54455E-07	2.39182E-07	0.000000415	1.72091E-07	1.05533E-07	2.89533E-07	3.27467E-07	1.828E-07
Sphingobacterium_mizutaii	1.28773E-06	8.18182E-08	4.81818E-07	4.07818E-07	5.15154E-07	6.53846E-08	0	4.53385E-07	1.51173E-06	1.52364E-06	1.83418E-06	1.81645E-06	6.92933E-07	6.75533E-07	1.06147E-06	1.09807E-06
Sphingobacterium_daejeonense	1.55245E-06	2.87273E-08	6.98182E-08	4.89091E-08	9.53538E-07	6.78462E-08	9.26154E-08	1.19846E-07	1.32727E-06	1.16236E-06	9.39273E-07	1.83955E-06	1.84333E-06	1.4626E-06	1.6296E-06	2.12647E-06
Sphingobacterium_thalpophilum	0.00000344	0	1.64909E-06	3.91891E-06	1.30554E-06	1.53769E-07	1.29777E-06	2.05231E-06	3.21255E-06	3.64909E-06	3.97727E-06	4.33364E-06	1.63507E-06	1.9486E-06	1.62813E-06	0.000002003
Sphingobacterium_hotanense	2.05545E-07	1.04409E-07	5.34545E-08	2.19909E-07	1.13715E-06	0.000000026	0.00000008	8.29231E-08	1.04945E-06	7.00636E-07	7.62545E-07	0.000000233	8.02067E-07	7.16933E-07	1.40613E-06	1.5194E-06
Sphingobacterium_lactis	3.34091E-06	4.06455E-07	1.61685E-06	3.51382E-06	4.06308E-06	8.84615E-08	1.13969E-06	0.000002078	6.41545E-06	5.39291E-06	7.19182E-06	6.21364E-06	0.000004712	3.78113E-06	0.000003792	3.84573E-06
Sphingobacterium_sp._DR205	2.90364E-06	0.00000013	1.35364E-06	2.18409E-06	7.36462E-07	2.27538E-07	1.71392E-06	1.74538E-06	0.00000172	2.24709E-06	2.16291E-06	2.08945E-06	4.90667E-07	6.788E-07	6.45267E-07	7.622E-07
Sphingobacterium_sp._dk4302	4.20636E-07	4.41364E-07	2.52573E-07	4.20545E-07	1.11308E-07	4.13846E-08	1.29538E-07	2.00077E-07	6.09545E-07	4.10545E-07	1.96273E-07	9.31818E-08	9.47333E-08	2.614E-07	3.21467E-07	0.000000153
Sphingobacterium_sp._ML3W	3.33045E-06	0.000000065	8.91818E-07	3.06264E-06	2.15362E-06	4.13846E-08	0.000000271	7.25538E-07	3.72636E-06	3.18364E-06	2.31936E-06	4.30818E-06	2.8054E-06	2.8074E-06	2.44047E-06	2.84467E-06
Sphingobacterium_sp._B29	1.25209E-06	1.68182E-08	0	0.000000071	1.26846E-06	6.78462E-08	7.92308E-08	3.81538E-08	1.30609E-06	1.05764E-06	8.30636E-07	1.49591E-06	1.59167E-06	1.3792E-06	1.6982E-06	1.6986E-06
Sphingobacterium_sp._CZ-2	2.63482E-06	2.42536E-06	4.56182E-06	5.32545E-06	1.97046E-06	1.20615E-07	2.16577E-06	3.36846E-06	2.50164E-06	1.90036E-06	2.36082E-06	2.53191E-06	2.4028E-06	2.2502E-06	2.49047E-06	2.38953E-06
Marivirga_tractuosa	1.86909E-07	0	0	2.43636E-08	6.73846E-08	2.52308E-08	0.0000001	6.32308E-08	0	0	0	7.03636E-07	1.268E-07	0.000000033	4.44E-09	6.02667E-08
Flammeovirga_sp._MY04	0.000000452	0	1.69273E-07	1.50545E-07	3.30308E-07	1.35385E-07	6.41538E-08	1.54308E-07	1.62091E-07	8.85455E-08	0.0000004	2.16318E-07	1.204E-07	2.246E-07	4.338E-07	3.624E-07
Flammeovirga_pectinis	9.22455E-07	1.09545E-07	8.42818E-07	0.000001063	3.50846E-07	0.000000091	5.46154E-07	5.00538E-07	8.86909E-07	7.61636E-07	1.36582E-06	6.40818E-07	6.972E-07	4.74267E-07	0.000000515	8.84667E-07
Bernardetia_litoralis	1.58173E-06	9.24545E-08	1.02955E-06	1.38282E-06	2.73769E-07	1.04462E-07	1.89692E-06	1.50754E-06	6.90909E-07	1.13409E-06	8.34273E-07	1.10382E-06	1.58933E-07	3.84667E-07	4.51733E-07	2.78267E-07
Roseivirga_pacifica	1.04473E-06	3.87182E-07	3.28764E-07	6.20818E-07	6.83692E-07	2.13846E-08	8.81615E-07	1.26323E-06	7.98727E-07	6.02364E-07	1.14273E-06	1.16355E-06	4.036E-07	6.13133E-07	1.35893E-06	9.896E-07
Candidatus_Amoebophilus_asiaticus	1.80273E-07	3.75455E-08	5.34545E-08	1.14273E-07	8.78462E-08	4.26923E-07	3.84769E-07	1.74538E-07	1.70091E-07	2.44273E-07	2.92455E-07	3.12909E-07	1.33733E-07	1.08533E-07	3.974E-07	1.968E-07
Cardinium_endosymbiont_of_Encarsia_pergandiella	2.70909E-08	0	0	0	0	0	0	0	0.000000027	0	0	0	4.88667E-08	0.000000014	0	0
Cardinium_endosymbiont_of_Sogatella_furcifera	0	0	0	0	3.58462E-08	0	0	0	0	0	0	0	0	0	0	0
Candidatus_Cardinium_hertigii	6.37727E-07	1.48636E-07	1.43636E-07	1.46364E-07	1.52185E-06	3.44615E-06	0.00000132	1.29308E-06	1.50436E-06	1.92718E-06	1.85473E-06	1.56691E-06	1.8894E-06	2.18853E-06	2.28287E-06	2.55533E-06
Chryseolinea_soli	1.30655E-05	1.25745E-06	1.16826E-05	1.85109E-05	4.19846E-06	5.92292E-06	2.44292E-05	2.41446E-05	9.09364E-06	8.48818E-06	1.07918E-05	1.07991E-05	4.79867E-06	0.000005856	6.94733E-06	7.62667E-06
Belliella_baltica	2.83909E-07	0.000000065	0	0.000000214	1.73769E-07	1.35385E-07	1.21923E-07	1.89231E-07	2.64545E-07	3.37818E-07	0	0.00000012	4.78667E-08	1.522E-07	4.44E-09	1.62667E-07
Aquiflexum_balticum	3.16273E-07	2.44909E-07	1.66591E-07	1.34182E-07	0.0000002	5.54077E-07	3.58077E-07	3.19846E-07	3.65636E-07	0.000000434	8.85727E-07	1.22491E-06	0.000000176	1.27733E-07	5.80733E-07	4.88733E-07
Echinicola_sp._LN3S3	7.24636E-07	4.07364E-07	1.63636E-08	6.03364E-07	2.37923E-07	3.57692E-07	3.98923E-07	1.67462E-07	4.63909E-07	5.80091E-07	7.03364E-07	0.00000054	2.826E-07	1.598E-07	1.58373E-07	1.94133E-07
Echinicola_rosea	3.04109E-06	4.01636E-07	7.01727E-07	0.000001406	1.27662E-06	4.17538E-07	2.79777E-06	3.02815E-06	2.72682E-06	5.03709E-06	3.67709E-06	0.000002042	1.2782E-06	1.6058E-06	1.8826E-06	2.6038E-06
Echinicola_strongylocentroti	9.38818E-07	7.66364E-08	5.40036E-07	1.24418E-06	6.29231E-07	4.07385E-07	0.000000435	5.26231E-07	7.97818E-07	4.54455E-07	9.40455E-07	8.75727E-07	0.000000495	6.206E-07	4.992E-07	3.926E-07
Echinicola_vietnamensis	7.89455E-07	2.33455E-07	2.39182E-07	6.30727E-07	1.39254E-06	1.04438E-06	0.000000087	5.11385E-07	2.26727E-07	2.98909E-07	5.90091E-07	5.57636E-07	9.10467E-07	8.64667E-07	1.26407E-06	7.77533E-07
Cyclobacterium_amurskyense	7.22818E-07	2.32727E-08	2.14182E-07	7.33273E-07	5.25692E-07	7.96923E-08	4.60846E-07	9.99077E-07	1.44173E-06	3.79636E-07	1.26536E-06	1.19473E-06	5.93933E-07	2.95467E-07	1.1358E-06	5.32133E-07
Cyclobacterium_marinum	1.02045E-05	1.22364E-06	6.25818E-06	8.32273E-06	4.96923E-06	5.22185E-06	1.98685E-06	2.07854E-06	1.09909E-05	6.84727E-06	9.51636E-06	8.49273E-06	5.6188E-06	6.02533E-06	4.94867E-06	4.59233E-06
Algoriphagus_machipongonensis	1.41273E-07	0	0	1.43636E-08	1.12154E-07	4.13846E-08	1.11154E-07	1.46923E-08	6.59091E-08	8.58182E-08	1.84364E-07	5.50455E-08	2.27867E-07	2.226E-07	1.644E-07	2.25867E-07
Algoriphagus_sp._Y33	4.39455E-07	2.83455E-07	1.70091E-08	2.71182E-07	1.19846E-07	0.000000026	3.11538E-08	3.22615E-07	6.30182E-07	6.63091E-07	0.000001152	0.000000485	1.84467E-07	3.42267E-07	4.74067E-07	3.41667E-07
Algoriphagus_sanaruensis	6.70545E-06	0.000000065	1.66255E-06	4.04182E-06	8.75846E-06	1.79231E-08	2.15108E-06	3.15385E-06	4.55682E-06	4.72273E-06	3.77545E-06	4.27091E-06	0.000008076	6.55067E-06	7.73533E-06	6.71867E-06
Arcticibacterium_luteifluviistationis	8.47727E-07	1.14364E-07	1.12455E-07	6.45364E-07	1.06308E-07	9.50769E-08	4.35077E-07	6.04385E-07	3.84455E-07	2.79273E-07	1.10973E-06	7.54182E-07	2.24333E-07	2.28933E-07	2.28333E-07	1.992E-07
Allopseudarcicella_aquatilis	2.75136E-06	3.30909E-07	3.41682E-07	3.37273E-08	5.67308E-07	0	7.53462E-07	1.44731E-06	2.89827E-06	3.53764E-06	4.70045E-06	2.01645E-06	1.14573E-06	4.06733E-07	5.474E-07	1.93967E-06
Rhodocytophaga_sp._172606-1	6.85091E-07	8.49091E-08	4.42582E-07	3.86909E-07	3.50154E-07	3.03431E-06	8.14385E-07	1.08115E-06	5.92909E-07	2.88182E-07	5.00455E-07	5.68455E-07	3.47067E-07	4.44067E-07	3.20267E-07	7.434E-07
Leadbetterella_byssophila	1.14973E-06	0	2.40273E-07	1.64845E-06	3.57769E-07	9.68462E-08	8.11538E-08	8.69923E-07	1.68455E-06	1.59491E-06	1.21745E-06	1.98955E-06	8.116E-07	0.000000977	8.866E-07	1.3176E-06
Cytophaga_hutchinsonii	2.53173E-06	0.000000065	7.68036E-07	2.20855E-06	1.26269E-06	2.03385E-07	5.56154E-07	1.98877E-06	1.42109E-06	1.04373E-06	2.32609E-06	2.43545E-06	1.1828E-06	1.5924E-06	2.32047E-06	1.60213E-06
Dyadobacter_fermentans	1.25482E-05	1.59518E-06	4.95455E-06	1.04982E-05	4.11769E-06	7.76792E-06	4.00077E-06	6.93231E-06	7.08182E-06	5.49636E-06	7.14545E-06	7.72545E-06	5.24347E-06	0.000004432	5.0458E-06	0.000006036
Fibrella_sp._ES10-3-2-2	2.44364E-06	1.41636E-07	2.23091E-06	4.46527E-06	6.64846E-07	0	5.64654E-06	0.000005245	1.47164E-06	1.47345E-06	1.71718E-06	0.00000196	1.17707E-06	0.000000925	1.04433E-06	1.5232E-06
Fibrella_aestuarina	7.21364E-06	7.57273E-07	4.29495E-06	7.32818E-06	2.27992E-06	2.27531E-06	8.24608E-06	8.99077E-06	5.30364E-06	4.78818E-06	6.78727E-06	6.18182E-06	0.000004758	4.3306E-06	3.92873E-06	3.92067E-06
Runella_sp._HYN0085	2.42973E-06	3.28273E-07	1.20682E-06	2.23373E-06	2.75154E-07	0	8.05385E-07	1.16192E-06	6.52909E-07	8.22545E-07	8.73091E-07	1.32927E-06	2.47133E-07	0.000000604	0.000000555	6.766E-07
Runella_slithyformis	2.48927E-06	2.67273E-07	1.49327E-06	2.58327E-06	0.000001004	4.27846E-07	6.42308E-07	1.54577E-06	2.20036E-06	2.12409E-06	2.21091E-06	2.32218E-06	1.12413E-06	1.37907E-06	1.56133E-06	1.61787E-06
Runella_sp._SP2	3.29727E-06	0.00000026	2.67409E-07	1.48136E-06	1.82646E-06	2.92692E-07	3.77385E-07	2.16077E-06	2.66591E-06	1.73464E-06	3.81545E-06	3.29864E-06	1.4838E-06	1.14187E-06	1.41727E-06	2.1764E-06
Spirosoma_sp._KCTC_42546	0.000000111	5.81818E-08	1.72727E-07	4.45545E-07	1.70385E-07	1.60154E-07	2.80692E-07	7.26846E-07	2.10455E-07	0	9.41818E-08	1.11455E-07	2.09333E-08	1.10933E-07	3.044E-07	4.784E-07
Spirosoma_sp._KUDC1026	2.48718E-06	1.18273E-06	1.29545E-06	2.30636E-06	6.46615E-07	5.33538E-07	1.77462E-06	1.44615E-06	9.90455E-07	1.79791E-06	1.81627E-06	1.50991E-06	2.83867E-07	6.188E-07	6.13467E-07	5.74933E-07
Spirosoma_sp._I-24	1.16636E-07	2.11818E-07	2.87273E-08	2.95273E-07	6.93077E-08	0	0	8.28462E-08	3.54636E-07	1.42455E-07	1.75182E-07	4.65909E-08	6.96E-08	0	9.83067E-08	0
Spirosoma_sp._PL0136	5.40455E-07	2.79091E-07	1.56336E-06	1.11436E-06	9.06846E-07	0	9.18462E-08	1.90769E-08	1.11627E-06	0.000001109	1.10027E-06	1.05818E-06	7.35533E-07	9.07133E-07	6.954E-07	1.22647E-06
Spirosoma_pollinicola	2.97318E-06	1.71727E-07	1.32455E-06	2.47182E-06	1.70277E-06	2.80369E-06	2.25477E-06	2.04923E-06	1.60418E-06	2.36073E-06	1.73291E-06	1.88927E-06	1.33467E-06	1.48087E-06	1.48907E-06	1.57367E-06
Spirosoma_aerolatum	2.95845E-06	5.92273E-08	1.18818E-05	1.08764E-05	3.82046E-06	2.05385E-07	3.38282E-05	3.16308E-05	7.09136E-06	0.000007986	4.09364E-06	4.92673E-06	3.56513E-06	3.2718E-06	7.25573E-06	3.7122E-06
Spirosoma_radiotolerans	1.48818E-07	1.98182E-08	6.208E-07	3.68818E-07	3.14846E-07	7.52462E-07	4.54069E-06	3.53285E-06	3.29455E-07	3.10545E-07	5.73545E-07	0.000000555	2.676E-07	8.08733E-07	4.54467E-07	7.82333E-07
Spirosoma_montaniterrae	9.73818E-06	9.78818E-07	2.51536E-06	5.87818E-06	4.36731E-06	2.55154E-06	7.59923E-06	1.37485E-05	6.83636E-06	6.85636E-06	8.17545E-06	6.66909E-06	7.32867E-06	5.05533E-06	5.36067E-06	5.53133E-06
Spirosoma_rigui	1.68164E-05	4.53955E-06	1.13309E-05	1.33209E-05	5.48538E-06	5.55077E-06	8.62231E-06	1.02792E-05	1.20245E-05	8.74364E-06	1.11227E-05	8.77182E-06	7.92067E-06	0.000006608	6.69667E-06	0.00000697
Spirosoma_sp._BT328	2.87091E-07	0	8.69727E-08	0.000000456	7.46154E-08	0	1.76538E-07	2.53846E-07	0.000000071	1.90545E-07	1.67182E-07	3.89273E-07	1.90133E-07	2.95933E-07	1.23107E-07	0.000000443
Spirosoma_sp._CJU-R4	8.63182E-06	8.06364E-07	1.64955E-05	1.91315E-05	4.99008E-06	1.78923E-07	3.95803E-05	4.23122E-05	9.84864E-06	1.06527E-05	7.09364E-06	9.18727E-06	5.79533E-06	4.65867E-06	0.000008146	6.5562E-06
Spirosoma_sp._TS118	4.61545E-06	1.93364E-07	5.92918E-06	7.13709E-06	0.000002674	1.20062E-06	2.90538E-06	0.000004208	7.18091E-06	7.25545E-06	6.28727E-06	4.54636E-06	3.53527E-06	5.09733E-06	0.000003792	4.67067E-06
Adhaeribacter_sp._KUDC8001	0.000000274	3.87636E-07	1.02818E-07	2.67273E-07	9.66154E-08	0	2.45923E-07	2.82462E-07	5.77818E-07	4.58909E-07	4.97636E-07	4.39727E-07	1.484E-07	2.98533E-07	1.038E-07	2.67133E-07
Adhaeribacter_swui	3.80091E-06	4.57545E-07	2.70282E-06	3.73427E-06	1.40754E-06	4.57415E-06	3.95877E-06	4.51769E-06	1.43836E-06	2.25455E-06	2.85727E-06	2.48818E-06	1.32327E-06	2.1434E-06	1.9302E-06	1.77107E-06
Nibribacter_sp._BT10	1.40773E-06	9.05E-08	2.95273E-07	1.08291E-06	3.33462E-07	7.70769E-08	2.01769E-07	4.50077E-07	9.72455E-07	4.31909E-07	7.35182E-07	1.36727E-06	4.516E-07	6.098E-07	8.20467E-07	9.58333E-07
Rufibacter_tibetensis	1.65273E-07	0.000000202	3.62364E-07	4.21909E-07	1.76815E-06	7.67385E-07	1.22954E-05	8.60292E-06	0.000000545	1.47909E-07	9.56364E-07	4.97818E-07	8.20867E-07	0.000002951	2.86827E-06	1.66333E-06
Rufibacter_sp._DG31D	4.19273E-06	4.98455E-07	3.40455E-06	4.71082E-06	8.65615E-07	0	5.91415E-06	6.57869E-06	2.80273E-06	1.91018E-06	2.62791E-06	2.04273E-06	1.49787E-06	1.29727E-06	2.05907E-06	2.2656E-06
Rufibacter_sp._DG15C	2.01745E-06	3.42909E-07	8.39909E-07	9.36727E-07	1.29462E-06	3.17215E-06	9.76769E-07	9.92615E-07	2.56291E-06	2.97391E-06	4.23745E-06	2.35409E-06	1.66253E-06	2.60013E-06	2.54933E-06	1.66247E-06
Pontibacter_korlensis	3.02727E-06	0.00000013	8.34091E-07	2.89745E-06	7.59462E-07	4.13846E-08	3.12692E-07	1.46823E-06	1.52409E-06	1.12891E-06	1.32536E-06	2.19464E-06	8.98467E-07	7.11067E-07	0.000000776	0.000001245
Pontibacter_sp._SGAir0037	4.58091E-06	2.17091E-07	3.23559E-06	4.64636E-06	8.72077E-07	1.18923E-06	2.72369E-06	2.61446E-06	2.82855E-06	2.45773E-06	0.000004017	2.78945E-06	1.24167E-06	0.000001738	1.9186E-06	0.000002428
Pontibacter_akesuensis	2.53455E-06	7.36727E-07	3.43045E-06	6.07355E-06	5.05138E-06	2.15692E-07	5.52769E-06	8.22185E-06	2.42336E-06	2.23882E-06	3.54691E-06	7.41464E-06	1.86387E-06	1.64287E-06	2.78267E-06	2.48233E-06
Pontibacter_pudoricolor	1.15864E-06	4.09364E-07	1.17818E-06	1.86418E-06	2.10962E-06	0.000001121	1.70338E-06	8.88385E-07	0.00000241	1.89555E-06	0.00000218	1.86736E-06	1.3268E-06	2.42973E-06	2.72573E-06	0.00000279
Pontibacter_russatus	2.00418E-05	1.92382E-06	1.07418E-05	1.13464E-05	7.92246E-06	8.67042E-05	1.70531E-05	1.91092E-05	1.28591E-05	1.01827E-05	0.00001428	9.79455E-06	0.000009846	1.01993E-05	1.01547E-05	1.05627E-05
Pontibacter_actiniarum	1.09018E-05	3.13373E-06	8.66795E-06	1.63236E-05	3.49238E-06	5.51169E-06	5.88769E-06	7.94462E-06	7.70182E-06	5.81636E-06	0.00000995	8.80364E-06	3.89133E-06	0.000004762	5.57333E-06	0.000007396
Hymenobacter_qilianensis	7.29545E-07	0	5.48182E-08	1.92545E-07	0.000000153	2.30692E-07	3.44462E-07	1.73308E-07	3.34273E-07	1.48091E-07	3.67636E-07	9.43182E-07	2.828E-07	2.39933E-07	0.000000243	4.06667E-07
Hymenobacter_sp._HDW8	2.56364E-08	0	2.58636E-07	0.000000094	1.33615E-07	0	4.96615E-07	8.86462E-07	1.19636E-07	1.18909E-07	1.74727E-07	8.55455E-08	1.286E-07	4.86667E-08	7.07333E-08	6.97333E-08
Hymenobacter_baengnokdamensis	9.96727E-07	0	3.78273E-07	1.54855E-06	1.38154E-07	1.19615E-07	1.54154E-07	3.74846E-07	3.57636E-07	3.37909E-07	8.22182E-07	5.81091E-07	0.000000391	5.52133E-07	5.71867E-07	6.78933E-07
Hymenobacter_sedentarius	4.36364E-06	5.53364E-07	0.000002387	4.87364E-06	7.40154E-07	2.10769E-06	3.52923E-06	5.08869E-06	2.17545E-06	1.68845E-06	2.25973E-06	3.38273E-06	1.34973E-06	1.65173E-06	1.93707E-06	0.000001787
Hymenobacter_oligotrophus	0.00000537	6.90364E-07	3.56682E-06	5.13636E-06	6.92077E-07	1.88308E-06	3.28385E-06	2.98092E-06	2.72136E-06	2.57318E-06	3.93818E-06	3.31736E-06	1.19753E-06	7.994E-07	1.02887E-06	1.2854E-06
Hymenobacter_jejuensis	4.24909E-06	2.22727E-07	1.63057E-06	3.94273E-06	1.48469E-06	2.07285E-06	5.46385E-06	3.94331E-06	2.39436E-06	2.68636E-06	3.24455E-06	2.82545E-06	2.4296E-06	2.0658E-06	2.1058E-06	0.000002958
Hymenobacter_swuensis	0.00001041	4.75545E-07	8.27039E-06	1.28797E-05	4.99846E-06	3.29385E-07	1.70808E-05	1.91746E-05	1.04691E-05	9.47364E-06	8.50091E-06	6.40545E-06	7.42067E-06	5.89373E-06	6.27933E-06	5.89267E-06
Hymenobacter_nivis	4.63318E-06	5.26455E-07	2.16264E-06	2.44618E-06	1.29162E-06	3.06923E-07	5.12723E-06	6.17215E-06	2.38118E-06	0.00000256	3.56818E-06	3.20109E-06	1.5674E-06	2.0876E-06	2.20767E-06	2.12127E-06
Hymenobacter_sp._PAMC_26554	5.15455E-06	5.30455E-07	2.84818E-06	5.65273E-06	2.21538E-06	1.41538E-06	6.04731E-06	4.66846E-06	6.22618E-06	5.49818E-06	5.86091E-06	2.82818E-06	5.0012E-06	3.14467E-06	2.66567E-06	0.000003882
Hymenobacter_sp._DG25B	3.45664E-06	3.64545E-07	1.31364E-06	2.14991E-06	9.13846E-07	4.13692E-07	7.58462E-07	2.81915E-06	2.08945E-06	1.16518E-06	2.42064E-06	2.28818E-06	1.8612E-06	1.27593E-06	1.40267E-06	2.30313E-06
Hymenobacter_sp._DG25A	1.44227E-06	4.65455E-08	2.18455E-06	2.53955E-06	6.16308E-07	2.95385E-08	4.93538E-06	5.52354E-06	1.54245E-06	1.04445E-06	1.37255E-06	1.07382E-06	7.34267E-07	9.374E-07	2.0144E-06	1.52253E-06
Hymenobacter_sp._S2-20-2	1.43273E-06	1.34727E-07	7.17036E-07	1.20727E-06	4.86308E-07	0	6.39769E-07	1.90869E-06	1.23727E-06	9.52091E-07	1.40827E-06	1.56782E-06	8.29667E-07	0.000000707	1.1472E-06	1.18767E-06
Hymenobacter_sp._DG01	3.69545E-06	3.29727E-07	1.89053E-06	3.16727E-06	7.67615E-07	0.00000562	2.99685E-06	4.51223E-06	0.00000246	1.52318E-06	2.30518E-06	2.54245E-06	9.93467E-07	1.0434E-06	1.69727E-06	2.12453E-06
Hymenobacter_sp._TS19	7.34182E-06	1.67818E-06	7.44312E-06	7.09882E-06	1.03823E-06	6.92538E-07	5.59846E-06	5.96877E-06	2.81264E-06	3.51136E-06	5.43109E-06	4.74091E-06	1.35213E-06	1.86033E-06	1.30933E-06	2.04073E-06

Hymenobacter_sp._NBH84	4.93027E-06	2.17818E-07	2.61418E-06	5.86409E-06	1.10362E-06	2.16923E-08	2.60538E-06	3.04154E-06	3.15336E-06	2.84545E-06	0.00000376	3.99545E-06	1.90927E-06	2.01353E-06	1.78153E-06	0.000003056
Hymenobacter_sp._PAMC_26628	2.95855E-06	1.54455E-07	2.22432E-06	6.11636E-06	9.74308E-07	2.98985E-06	0.000010638	1.00618E-05	1.96082E-06	2.19945E-06	2.40455E-06	2.65773E-06	1.20573E-06	1.33133E-06	1.49427E-06	1.0692E-06
Hymenobacter_sp._BRD128	5.49091E-06	3.20636E-07	2.19091E-06	3.64182E-06	2.56292E-06	5.42154E-07	6.09708E-06	8.76246E-06	5.35273E-06	4.95164E-06	6.86091E-06	4.92091E-06	3.59087E-06	2.71447E-06	4.77667E-06	3.64687E-06
Hymenobacter_sp._BT182	0.00001028	1.39764E-06	6.22027E-06	0.00000713	1.75708E-06	6.76085E-06	6.68415E-06	9.30077E-06	0.00000377	4.33909E-06	6.93727E-06	0.00000595	2.66333E-06	4.05687E-06	0.000003314	0.000004302
Hymenobacter_sp._APR13	6.40209E-06	7.79364E-07	3.70918E-06	1.03036E-05	2.33008E-06	6.63331E-06	1.98795E-05	1.91142E-05	4.74273E-06	7.10336E-06	7.94545E-06	5.44364E-06	2.95713E-06	2.74333E-06	3.90067E-06	3.25533E-06
Hymenobacter_sp._17J36-26	1.06464E-06	1.12073E-06	7.81182E-07	0.000000839	2.06308E-07	0	9.44231E-07	5.69077E-07	7.27636E-07	6.91364E-07	8.36364E-07	1.14827E-06	9.63333E-08	5.97667E-07	3.10733E-07	0.000000202
Hymenobacter_sp._BT18	6.03909E-06	8.86636E-07	4.29345E-06	7.52364E-06	1.98823E-06	4.79231E-07	3.40692E-06	0.000004238	0.00000409	3.40182E-06	4.83455E-06	5.12645E-06	2.16813E-06	3.16167E-06	2.70933E-06	2.85207E-06
Hymenobacter_sp._BRD67	6.05273E-06	4.12364E-06	1.19503E-06	5.17891E-06	4.88362E-06	5.84085E-06	9.36308E-06	6.22615E-06	9.47182E-06	0.00000802	5.00755E-06	5.95636E-06	0.00000414	4.4358E-06	2.55933E-06	2.75273E-06
Ichthyobacterium_seriolicida	3.24091E-07	0	0	2.72545E-07	0.000000039	0	1.32462E-07	1.00846E-07	0.000000107	2.85455E-08	0	1.60364E-07	6.08E-08	5.71333E-08	6.61733E-08	2.17867E-07
Candidatus_Walczuchella_monophlebidarum	3.69636E-07	8.18182E-08	7.40909E-08	1.88727E-07	2.13308E-07	4.39231E-08	4.27231E-07	1.95615E-07	3.69182E-07	1.48455E-07	5.09909E-07	1.23091E-07	1.786E-07	1.74333E-07	2.87867E-07	1.074E-07
Fluviicola_taffensis	3.11455E-07	0	8.80909E-08	5.46364E-08	8.36923E-08	5.36923E-08	3.57385E-07	2.90308E-07	5.28364E-07	0.0000001	4.98455E-07	0.000000625	4.78E-08	2.79133E-07	1.8444E-07	5.14533E-07
Candidatus_Sulcia_muelleri	7.10909E-08	2.08182E-08	7.83636E-08	1.14364E-07	4.12462E-07	1.74769E-07	3.79308E-07	5.72846E-07	4.69273E-07	2.76364E-07	9.75455E-08	5.29909E-07	7.15867E-07	6.91667E-07	7.54667E-07	8.87067E-07
Owenweeksia_hongkongensis	4.37455E-06	0	2.39245E-06	3.07564E-06	2.47631E-06	0	2.77623E-06	3.49154E-06	3.41818E-06	3.07745E-06	3.85636E-06	0.000003575	2.04473E-06	2.61173E-06	0.000002888	2.91953E-06
Blattabacterium_punctulatus	0	0	0	0	0	3.15385E-08	0	0	0	0	0	0	0	0	0	0
Blattabacterium_clevelandi	0	0	0	0	0	0	0	0	0	0	0	7.13636E-09	0	0	0	0
Blattabacterium_sp._(Cryptocercus_kyebangensis)	0	0	0	0	3.15385E-08	0.000000026	0	0	0	0	1.98182E-07	3.30455E-07	0	0	0	0
Blattabacterium_sp._(Blaberus_giganteus)	0	0	3.27273E-08	0	0	0	0	0	0	0	0	3.74545E-08	0	0	0	0
Blattabacterium_sp._DPU	0	0	0	0.000000275	1.28692E-07	0	1.19615E-07	3.37308E-07	0	9.36364E-08	6.88182E-08	6.29455E-07	4.03933E-07	5.368E-07	0.000000397	6.16067E-07
Blattabacterium_sp._(Blattella_germanica)	2.40636E-07	1.66182E-06	1.44373E-06	5.20727E-07	1.33154E-07	4.73077E-08	2.73154E-07	4.30462E-07	4.71455E-07	9.40909E-08	3.32545E-07	1.02909E-07	3.35667E-07	2.882E-07	1.93667E-07	4.18467E-07
Blattabacterium_cuenoti	5.72264E-06	8.21591E-07	1.52775E-06	2.74336E-06	6.16231E-06	1.20454E-06	5.78154E-06	9.56846E-06	5.35182E-06	8.07818E-06	1.01255E-05	7.17273E-06	7.04133E-06	7.52667E-06	9.43667E-06	9.60133E-06
Weeksella_virosa	0	0	2.45455E-07	1.43636E-08	1.41769E-07	0	6.66154E-08	2.91077E-07	2.15091E-07	0.000000068	0	0	1.86667E-07	3.48E-08	1.29573E-07	1.968E-07
Cloacibacterium_normanense	1.55909E-07	2.87773E-07	1.76855E-07	2.22818E-07	9.25385E-08	1.40154E-07	1.44231E-07	2.42231E-07	8.43636E-08	9.40909E-08	0.000000202	1.99455E-07	9.46E-08	0.000000215	3.25133E-07	2.14067E-07
Ornithobacterium_rhinotracheale	1.06764E-06	0	4.08127E-07	2.86091E-07	5.28923E-07	0	1.02162E-06	0.000001245	9.91636E-07	7.18909E-07	1.08064E-06	0.00000138	7.99733E-07	6.60133E-07	5.774E-07	1.7236E-06
Cruoricaptor_ignavus	2.68727E-06	7.66364E-08	1.49091E-06	2.70091E-06	5.07154E-07	2.41615E-07	2.95138E-06	2.90946E-06	1.65718E-06	1.06491E-06	1.76155E-06	7.43727E-07	1.09073E-06	7.63133E-07	9.97133E-07	6.57133E-07
Bergeyella_cardium	2.54336E-06	3.09136E-07	1.48091E-06	1.93273E-06	5.65077E-07	2.08077E-06	1.45008E-06	0.000002833	1.03773E-06	1.86391E-06	1.81318E-06	1.99836E-06	9.06333E-07	8.97933E-07	9.82667E-07	1.12773E-06
Empedobacter_falsenii	3.49364E-07	0	8.40909E-08	3.39064E-07	6.02615E-07	0	0.00000013	3.64462E-07	7.90182E-07	2.60636E-07	7.06818E-07	7.60364E-07	4.47733E-07	2.31667E-07	4.928E-07	4.45267E-07
Empedobacter_stercoris	1.57636E-07	1.92545E-06	2.13727E-06	1.09636E-06	9.03846E-08	3.78462E-08	1.50769E-07	4.93846E-08	4.22545E-07	3.36455E-07	7.62909E-07	5.10818E-07	3.254E-07	9.48E-08	2.506E-07	6.384E-07
Empedobacter_brevis	2.06327E-06	0.000000065	7.08364E-07	2.48845E-06	6.10231E-07	0	1.89846E-06	1.53662E-06	1.01591E-06	1.42436E-06	1.97473E-06	1.53955E-06	0.000000203	6.52133E-07	5.31933E-07	5.50667E-07
Riemerella_anatipestifer	0.000004042	4.18818E-07	8.44091E-07	2.32455E-06	3.60154E-07	0.000000139	5.34077E-07	1.63323E-06	1.89336E-06	1.16027E-06	1.35545E-06	2.11536E-06	8.756E-07	9.784E-07	9.278E-07	1.28707E-06
Apibacter_sp._B3706	0	0	0	0	0	0	0	0	0	0	0	0	0	0	1.49333E-08	0
Apibacter_sp._B2966	0	0	0	9.13636E-08	0	0	0	5.72308E-08	0	0	3.33636E-08	7.13636E-09	0	7.94E-08	4.44E-09	0
Apibacter_raozihei	1.04818E-07	0	0	1.43636E-08	2.29077E-07	1.99385E-07	1.17615E-07	0	6.45455E-08	0.000000028	3.79091E-08	3.34409E-07	5.28667E-08	1.12733E-07	1.832E-07	2.15667E-07
Elizabethkingia_miricola	1.58273E-07	0	0	9.54545E-09	5.47692E-08	0.000000068	2.63385E-07	4.30077E-07	1.09182E-07	5.22727E-08	1.18636E-07	1.31955E-07	2.64E-08	5.06667E-08	8.39333E-08	3.40667E-08
Elizabethkingia_meningoseptica	4.51545E-07	1.00955E-07	1.10909E-07	2.82909E-07	3.05846E-07	2.95385E-08	0	1.54308E-07	6.92545E-07	0.000000132	4.80091E-07	9.78455E-07	1.01333E-07	2.06667E-07	2.52333E-07	4.99867E-07
Elizabethkingia_sp._JS20170427COW	9.28182E-07	0	0	3.54545E-08	2.04746E-06	4.95923E-07	2.78769E-07	3.40538E-07	1.02855E-06	0.000000391	1.02673E-06	9.99091E-07	1.7184E-06	1.3416E-06	2.13093E-06	1.80287E-06
Elizabethkingia_bruuniana	2.18336E-06	0.00000013	4.99182E-07	2.29455E-06	2.46154E-07	8.41692E-07	9.58692E-07	1.71723E-06	1.03764E-06	6.46455E-07	8.29182E-07	1.53836E-06	4.16267E-07	7.76467E-07	4.55333E-07	1.59227E-06
Elizabethkingia_anophelis	2.39118E-06	3.35909E-07	9.96818E-07	0.000001777	2.69846E-06	0.00000586	2.00315E-06	3.06638E-06	1.29455E-06	0.000001249	2.15691E-06	2.68291E-06	1.97147E-06	0.000002055	0.000002248	0.000003302
Elizabethkingia_ursingii	4.59636E-07	0	0	3.81455E-07	3.49615E-07	0	0	0	1.02091E-07	2.95455E-07	3.99727E-07	1.65636E-07	9.50733E-07	1.6192E-06	9.31067E-07	7.88067E-07
Chryseobacterium_carnis	0	0	0	4.79091E-09	2.75385E-08	0	0	0	2.72727E-08	3.62727E-08	0	0	0	4.84667E-08	4.82E-08	0
Chryseobacterium_antarcticum	0.00000006	0	3.70909E-08	5.18182E-09	1.24923E-07	0	0	5.22308E-08	7.61818E-08	0.000000081	0	1.42727E-08	0.000000111	0.000000075	4.344E-08	0
Chryseobacterium_haifense	1.96091E-07	0	2.01818E-07	2.59091E-08	3.15385E-08	0	0	4.93846E-08	3.98182E-08	0	1.04545E-07	6.01818E-08	4.30667E-08	0	1.12667E-08	0
Chryseobacterium_jeonii	2.00673E-06	1.32091E-06	7.72773E-07	5.51909E-07	1.26769E-07	5.32308E-08	9.65385E-07	9.09846E-07	7.03182E-07	4.50818E-07	1.03345E-06	4.25091E-07	1.916E-07	1.28467E-07	4.544E-08	1.144E-07
Kaistella_daneshvariae	1.83409E-06	0.00000026	6.36982E-07	1.19773E-06	7.59769E-07	4.99769E-07	6.45231E-07	1.32346E-06	1.29627E-06	7.19727E-07	1.22027E-06	1.50764E-06	7.00733E-07	7.17133E-07	1.30867E-06	7.27733E-07
Chryseobacterium_lactis	3.11818E-08	9.54545E-09	0	0.000000777	4.13846E-08	1.10769E-06	1.90462E-06	1.52908E-06	1.83273E-07	4.92636E-07	2.95091E-07	2.69182E-07	2.83667E-07	0.000000448	1.288E-07	1.68667E-07
Chryseobacterium_taklimakanense	3.83727E-06	8.32273E-08	9.10909E-07	2.73827E-06	1.42446E-06	5.19692E-07	2.95892E-06	3.47762E-06	0.00000266	1.86209E-06	2.56091E-06	2.28227E-06	1.4698E-06	1.98427E-06	0.000001516	1.9582E-06
Chryseobacterium_sp._SNU_WT5	0	0	0	5.16364E-08	0	0	0	0	0	6.37273E-08	0	7.13636E-09	0	0	0	3.67333E-08
Chryseobacterium_arthrosphaerae	2.96364E-08	0	0	7.12455E-08	1.62769E-07	0	0	4.38462E-08	0	6.08182E-08	0	1.39136E-07	1.09133E-07	7.72E-08	8.86667E-09	2.15333E-08
Chryseobacterium_sp._POL2	5.98182E-07	2.70909E-08	2.31455E-07	1.35427E-07	1.26385E-07	0	1.07538E-07	6.03846E-08	0.000000481	6.08727E-07	6.73909E-07	0.000000384	1.382E-07	1.79733E-07	8.464E-08	1.96667E-07
Chryseobacterium_indoltheticum	5.43636E-07	1.04545E-08	0	1.03636E-08	1.82308E-07	1.79231E-08	1.58923E-07	3.55538E-07	2.01545E-07	2.23545E-07	5.35909E-07	7.10273E-07	9.32E-08	1.052E-07	1.388E-07	1.29533E-07
Chryseobacterium_manosquense	2.05873E-06	2.17682E-07	1.27236E-06	1.76591E-06	1.42692E-07	4.41077E-06	2.29315E-06	3.88538E-06	0.000001247	6.24182E-07	1.44345E-06	2.14809E-06	5.284E-07	3.55333E-07	7.71667E-07	1.33273E-06
Chryseobacterium_sp._JV274	0.00000006	1.26136E-07	5.83636E-08	3.55182E-08	0.000000061	8.43846E-08	0	3.56923E-08	8.75455E-08	0	1.72727E-07	6.35455E-08	8.84667E-08	1.58E-08	1.352E-07	7.48667E-08
Chryseobacterium_sp._G0186	2.48727E-07	0	2.87273E-08	0	1.00846E-07	3.58462E-08	2.33308E-07	1.81846E-07	2.99182E-07	1.08818E-07	4.48364E-07	4.51273E-07	1.38267E-07	6.07333E-08	2.15067E-07	2.116E-07
Chryseobacterium_sp._3008163	0.000001091	5.03091E-07	3.30091E-07	9.60818E-07	4.92923E-07	0	2.81385E-07	2.27692E-07	1.07382E-06	7.52091E-07	1.69927E-06	1.80191E-06	7.28267E-07	5.366E-07	0.000000499	6.052E-07
Chryseobacterium_balustinum	2.42636E-06	1.64909E-07	7.47273E-07	2.83727E-06	3.83308E-07	0	0.000000447	2.46185E-06	8.35091E-07	1.43427E-06	1.06691E-06	1.89918E-06	8.18467E-07	6.28667E-07	8.49867E-07	0.000001234
Chryseobacterium_glaciei	6.26909E-07	1.94545E-07	3.41273E-07	4.31727E-07	0	0	4.08308E-07	4.68538E-07	4.59818E-07	2.90818E-07	2.17273E-07	4.05818E-07	0	0	8.39333E-08	1.096E-07
Chryseobacterium_joostei	6.26182E-07	1.16364E-08	6.95945E-07	7.87818E-07	1.96154E-07	1.99077E-07	3.22308E-07	3.50769E-07	9.22545E-07	4.39091E-07	9.20182E-07	4.29455E-07	4.89133E-07	2.83333E-07	7.87333E-07	3.17933E-07
Chryseobacterium_gleum	5.78909E-07	7.40909E-08	9.45818E-08	5.46727E-07	0.000000098	0	3.18231E-07	1.37769E-07	3.68727E-07	0.000000263	3.32455E-07	1.95909E-07	8.92E-08	2.44733E-07	1.6084E-07	1.79133E-07
Chryseobacterium_sp._7-3A	8.52818E-07	9.39909E-07	2.68909E-07	6.89364E-07	0.000000448	9.39308E-07	6.12385E-07	2.88538E-07	1.09791E-06	6.78636E-07	8.87818E-07	1.57227E-06	4.116E-07	4.25867E-07	5.40373E-07	9.49333E-07
Chryseobacterium_aureum	2.08182E-07	9.90909E-07	8.38182E-08	7.15455E-08	1.11154E-07	5.99231E-07	6.06923E-08	5.72308E-08	5.45455E-08	1.68273E-07	1.04545E-07	2.85455E-08	8.77333E-08	0.000000033	1.762E-07	1.388E-07
Chryseobacterium_camelliae	8.88818E-07	2.20955E-07	7.40091E-07	4.65818E-07	7.65077E-07	2.04077E-07	7.48923E-07	1.06608E-06	8.29909E-07	1.00255E-06	1.18682E-06	1.35327E-06	6.052E-07	6.62933E-07	7.89533E-07	1.10413E-06
Chryseobacterium_sp._T16E-39	1.36364E-07	0.000000065	2.64182E-07	2.85273E-07	8.79231E-08	0	0	1.90769E-08	5.91818E-08	0.000000028	2.63455E-07	1.42727E-08	1.37667E-07	1.16133E-07	2.57867E-07	3.37333E-08
Chryseobacterium_nakagawai	4.15455E-07	1.06182E-07	9.88182E-08	1.03636E-08	1.05231E-07	0	4.33846E-08	3.45385E-07	0	0.0000001	0.000000061	2.17636E-07	8.42E-08	1.81667E-07	1.93733E-08	1.96067E-07
Chryseobacterium_gallinarum	1.18318E-06	8.53636E-07	0.000000816	4.58636E-07	5.64462E-07	8.06408E-06	1.37754E-06	1.88054E-06	7.53091E-07	9.02818E-07	1.41664E-06	6.30273E-07	5.75933E-07	7.14267E-07	6.85067E-07	1.06093E-06
Chryseobacterium_carnipullorum	1.46318E-06	1.94545E-07	7.68636E-07	8.08545E-07	5.02231E-07	7.86769E-07	2.46769E-07	1.97462E-07	1.30464E-06	5.26909E-07	1.30736E-06	5.25182E-07	3.10467E-07	7.182E-07	0.000000469	6.56333E-07
Chryseobacterium_piperi	4.51091E-07	0	0.000000122	9.92455E-08	7.69385E-07	0	0	1.18077E-07	7.95455E-08	6.42727E-08	2.22636E-07	2.02409E-07	4.51267E-07	7.63467E-07	8.022E-07	7.472E-07
Chryseobacterium_bernardetii	1.66818E-06	5.84545E-07	1.91182E-07	1.10845E-06	4.80615E-07	4.93462E-07	1.47846E-07	7.13154E-07	8.03636E-07	4.36727E-07	1.36764E-06	1.40736E-06	5.05533E-07	6.686E-07	7.456E-07	1.0146E-06
Chryseobacterium_indologenes	2.23182E-06	4.66818E-07	0.000002123	2.62182E-06	2.53231E-06	1.59077E-07	7.63769E-07	1.74408E-06	1.95909E-06	2.23009E-06	2.05255E-06	3.29164E-06	2.30067E-06	2.8764E-06	2.79647E-06	2.62067E-06
Chryseobacterium_shandongense	1.19118E-05	6.24364E-07	1.01627E-05	8.97909E-06	5.28077E-06	0.000058748	1.33608E-05	1.94346E-05	1.00491E-05	8.67909E-06	8.90091E-06	0.00000952	0.000007306	6.83067E-06	7.21733E-06	0.000008248
Chryseobacterium_taihuense	3.86809E-06	1.41168E-06	1.03082E-06	1.99736E-06	2.18108E-06	8.12615E-07	2.78023E-06	1.97592E-06	6.15364E-06	6.95818E-06	0.000004722	2.58927E-06	1.5884E-06	2.21767E-06	1.92427E-06	2.3624E-06
Chryseobacterium_sp._IHB_B_17019	5.87727E-07	3.11182E-07	1.57909E-07	3.66091E-07	1.61077E-07	0	1.68923E-07	5.01462E-07	1.11445E-06	7.59364E-07	3.94727E-07	3.91682E-07	3.098E-07	1.33267E-07	4.97867E-07	3.24867E-07
Chryseobacterium_sp._F5649	2.98182E-08	9.22727E-08	0	5.18182E-09	0	2.13846E-08	0	1.46923E-08	0	0	0	7.13636E-09	2.72E-08	3.48E-08	1.886E-07	0
Chryseobacterium_sp._G0201	2.13355E-06	1.14518E-06	7.47545E-07	2.25255E-06	6.01538E-07	0	2.16923E-06	1.24092E-06	1.28991E-06	1.63427E-06	1.30009E-06	1.19736E-06	4.97533E-07	4.19467E-07	6.47933E-07	6.732E-07
Chryseobacterium_sp._NBC_122	9.77273E-08	5.99364E-07	6.68455E-07	4.48727E-07	1.39538E-07	2.82385E-07	1.01923E-07	1.61154E-07	2.79727E-07	2.67636E-07	2.30909E-07	3.86909E-07	1.04333E-07	1.51533E-07	1.06267E-07	0.000000384
Chryseobacterium_sp._StRB126	6.73182E-07	1.80364E-07	0	6.95455E-08	8.06154E-07	9.46154E-09	2.73846E-08	4.98692E-07	9.75455E-08	0	6.88182E-08	7.12727E-08	5.10267E-07	4.814E-07	8.90667E-07	1.05573E-06
Chryseobacterium_sp._G0162	6.52636E-07	0	2.31636E-07	1.45045E-06	2.89846E-07	0	0	1.73385E-07	0.000000417	4.68727E-07	5.56818E-07	4.14909E-07	1.94067E-07	3.06933E-07	2.87933E-07	7.132E-07
Chryseobacterium_sp._NEB161	7.91818E-07	3.49091E-08	1.00455E-07	1.70036E-06	3.53154E-07	5.35308E-07	1.99077E-07	9.43846E-07	7.54273E-07	5.44636E-07	7.65909E-07	5.59727E-07	5.22133E-07	7.00267E-07	8.224E-07	1.8262E-06
Chryseobacterium_sp._cx-624	3.93273E-06	4.78182E-08	1.85239E-06	2.63664E-06	0.000000797	6.76554E-06	3.72392E-06	4.71538E-06	2.04436E-06	2.33591E-06	2.65473E-06	2.18127E-06	1.2592E-06	1.31633E-06	1.18487E-06	0.000001501
Chryseobacterium_sp._6424	3.14727E-06	2.14818E-07	1.80313E-06	2.95209E-06	1.18923E-06	0.000011871	3.47646E-06	4.48046E-06	2.22336E-06	1.89764E-06	0.000002807	2.77273E-06	1.14233E-06	1.55713E-06	1.54953E-06	1.91667E-06
Chryseobacterium_sp.	1.44836E-06	0.000000423	8.93273E-07	1.89155E-06	6.28923E-07	7.53846E-08	7.27462E-07	1.07715E-06	1.32736E-06	1.18473E-06	2.05945E-06	1.72427E-06	9.20533E-07	1.29007E-06	1.10527E-06	1.29507E-06
Leeuwenhoekiella_sp._ZYFB001	2.04182E-07	0	7.40909E-08	2.27091E-07	1.09385E-07	0	9.48462E-08	2.54923E-07	2.56364E-07	1.66182E-07	6.68182E-08	7.10909E-08	6.36667E-08	8.66E-08	1.79267E-07	0.000000154
Psychroserpens_sp._NJDZ02	5.20909E-08	0	0	0	3.34615E-08	0	0	1.16154E-07	0	0	3.33636E-08	8.86364E-08	0	0	0	0
Oceanihabitans_sp._IOP_32	1.27855E-06	1.94545E-07	2.59091E-07	5.14273E-08	2.58385E-07	0	3.05462E-07	4.82769E-07	2.19818E-07	1.37091E-07	4.74273E-07	6.77682E-07	1.82667E-07	1.312E-07	1.202E-07	2.79333E-08
Changchengzhania_lutea	1.39364E-07	3.39091E-08	6.67273E-08	7.38182E-08	5.21538E-08	7.21538E-08	2.07385E-07	2.08769E-07	3.11636E-07	5.22727E-08	8.52818E-07	3.03091E-07	2.366E-07	2.98E-08	4.2584E-07	2.88867E-07
Flagellimonas_maritima	2.99182E-07	0.000000065	7.40909E-08	3.09909E-07	2.59231E-08	0	0	1.39538E-07	9.32727E-08	2.87273E-08	2.12545E-07	3.40909E-07	9.18667E-08	1.70667E-07	7.26E-08	6.90667E-08
Costertonia_aggregata	1.67418E-06	0.000000803	1.28636E-07	1.39673E-06	8.60769E-08	5.16769E-07	8.43846E-08	5.57077E-07	3.73727E-07	9.17636E-07	1.12427E-06	6.55273E-07	2.77867E-07	1.60067E-07	1.956E-07	3.31667E-07
Zunongwangia_profunda	8.93091E-07	4.16364E-07	5.96273E-07	9.61455E-07	1.63538E-07	5.12308E-08	9.39538E-07	1.12415E-06	7.13727E-07	3.01545E-07	9.04636E-07	7.79545E-07	1.07667E-07	1.902E-07	1.60173E-07	7.83333E-08
Arenibacter_algicola	9.04364E-07	0.000000065	4.52036E-07	8.90727E-07	4.04462E-07	1.64231E-07	3.82308E-07	1.06615E-07	8.22636E-07	2.28545E-07	1.13518E-06	7.55727E-07	0.000000304	2.74733E-07	2.47067E-07	2.18333E-07
Salegentibacter_sp._T436	2.87818E-07	0	1.01364E-07	0	1.78462E-07	1.42538E-07	2.95846E-07	7.65154E-07	1.29455E-07	2.37636E-07	1.42455E-07	2.51182E-07	1.36733E-07	2.00267E-07	4.14467E-07	2.16067E-07
Salegentibacter_salegens	8.42727E-08	5.05455E-08	4.12727E-09	1.43636E-08	2.34077E-07	1.40308E-07	2.63077E-08	0	1.17273E-07	0.000000075	3.45636E-07	4.31545E-07	2.14E-08	2.62E-08	4.70667E-08	1.16133E-07
Muriicola_sp._MMS17-SY002	1.80636E-07	1.16364E-08	1.43764E-07	0.000000186	3.72308E-07	3.44385E-07	1.83923E-07	2.92231E-07	2.92091E-07	1.86636E-07	4.85727E-07	2.79455E-07	2.20533E-07	3.174E-07	3.94067E-07	6.84667E-07
Amniculibacterium_sp._G2-70	1.66455E-07	2.09091E-07	8.86182E-07	3.30609E-07	5.83308E-07	4.13846E-08	0	1.48538E-07	5.99909E-07	8.69182E-07	7.06273E-07	6.42636E-07	5.40867E-07	0.000000833	9.098E-07	8.75267E-07
Algibacter_sp._L1A34	4.87273E-08	0	0	0	4.33923E-07	0	0	1.10385E-07	2.32636E-07	0	0	1.06091E-07	3.01733E-07	1.87467E-07	3.19133E-07	4.53733E-07
Algibacter_sp._L3A6	9.25273E-07	0.000000065	6.19364E-07	5.41273E-07	2.17077E-07	4.71538E-07	8.42231E-07	6.44769E-07	8.62545E-07	5.29636E-07	1.08482E-06	1.34545E-06	4.78267E-07	2.80133E-07	4.90067E-07	5.36733E-07
Algibacter_alginicilyticus	1.74455E-07	0	4.12182E-08	2.87273E-08	5.76154E-08	5.05385E-08	8.81538E-08	1.49385E-07	0	1.17273E-07	1.42455E-07	4.45909E-08	2.568E-07	9.98667E-08	1.668E-07	2.64867E-07
Kordia_sp._SMS9	0	0.000000065	0	1.47273E-08	5.50769E-08	0	0	4.88462E-08	0	2.27182E-07	0	3.57273E-08	2.72E-08	3.35333E-08	1.18667E-07	0
Kordia_antarctica	1.25364E-07	2.32727E-07	1.39585E-06	5.28182E-07	1.01677E-06	0.000000376	2.50308E-07	1.80923E-07	8.18909E-07	8.60182E-07	9.01182E-07	1.38709E-06	1.06153E-06	1.30173E-06	9.66467E-07	1.10207E-06
Olleya_sp._Bg11-27	1.16091E-07	2.63636E-08	2.11673E-07	7.22727E-08	1.22538E-07	0	0	1.90769E-08	1.95818E-07	2.43273E-07	1.64727E-07	1.09864E-07	1.416E-07	1.33333E-07	3.13333E-08	2.214E-07
Olleya_aquimaris	2.09845E-06	2.49955E-07	1.22273E-07	0	6.15846E-07	1.12769E-07	1.11115E-06	1.81523E-06	1.95164E-06	2.52509E-06	3.25182E-06	1.55482E-06	8.87533E-07	5.67667E-07	4.106E-07	1.07007E-06
Lutibacter_profundi	2.15527E-06	1.94545E-07	2.94818E-07	1.86673E-06	1.13154E-07	0	7.66923E-08	2.87615E-07	4.60182E-07	6.24364E-07	6.88455E-07	6.05909E-07	3.46467E-07	2.71267E-07	3.09267E-07	7.28733E-07
Galbibacter_sp._BG1	2.32364E-07	0	4.12727E-09	6.22727E-08	6.73846E-08	2.23385E-07	1.19615E-07	9.20769E-08	1.28909E-07	0	1.41182E-07	1.46818E-07	5.32E-08	1.00867E-07	1.57067E-08	1.216E-07
Tamlana_sp._UJ94	2.56364E-08	0	0	3.70818E-07	2.33846E-07	5.05385E-08	5.54615E-08	5.81538E-08	1.50091E-07	1.19727E-07	1.19091E-07	4.26364E-07	5.44E-08	5.06E-08	2.83733E-08	1.078E-07
Mariniflexile_sp._TRM1-10	2.73809E-06	2.79818E-07	2.38636E-07	6.21727E-07	4.86615E-07	6.53077E-08	8.11615E-07	1.43538E-06	1.64582E-06	2.69518E-06	3.25009E-06	3.03864E-06	5.59467E-07	2.67667E-07	9.89267E-07	1.37053E-06
Aureitalea_sp._RR4-38	8.53636E-08	0	0	4.79091E-09	8.91538E-08	0	1.19615E-07	2.65769E-07	2.72727E-08	0	1.14545E-07	8.15909E-08	6.42E-08	3.48E-08	4.152E-07	4.626E-07
Flavivirga_eckloniae	2.84591E-06	4.04545E-07	4.33455E-07	1.23182E-07	6.75385E-07	6.89385E-07	1.70038E-06	2.03446E-06	2.33136E-06	4.00427E-06	4.19209E-06	2.06773E-06	8.12733E-07	0.000000666	8.22973E-07	1.1346E-06
Zobellia_galactanivorans	5.46364E-07	3.10273E-07	2.623E-07	1.78664E-06	6.07615E-07	7.86538E-07	7.43846E-08	2.37692E-07	1.58018E-06	1.05827E-06	1.50464E-06	1.32655E-06	5.02133E-07	3.97867E-07	0.000000532	8.052E-07
Wenyingzhuangia_fucanilytica	0	0	0	0	0.000000196	0	2.63077E-08	2.12846E-07	4.45455E-08	0	0	1.42727E-08	2.44E-08	3.48E-08	7.46667E-08	7.71333E-08
Sediminicola_sp._YIK13	3.24182E-07	0	0	1.47273E-08	6.23077E-08	0	4.80769E-08	9.63077E-08	1.64364E-07	0.00000013	2.59909E-07	3.20636E-07	6.47333E-08	3.48E-08	2.368E-07	5.42267E-07
Psychroflexus_torquis	1.20364E-07	0	4.50909E-08	0.000000071	1.24231E-07	5.01846E-07	9.77692E-08	4.62615E-07	4.19182E-07	2.07545E-07	0.000000232	3.53636E-07	1.17733E-07	4.014E-07	3.15267E-07	1.58333E-07
Gilvibacter_sp._SZ-19	1.08418E-06	0.00000013	4.23909E-07	6.74364E-07	0.000000289	4.26538E-07	4.97692E-07	2.35615E-07	7.99818E-07	2.32182E-07	6.53364E-07	0.000001262	2.068E-07	3.23667E-07	4.97667E-07	6.72733E-07
Cellulophaga_baltica	0	9.36364E-09	0	5.94545E-08	0	0	0	0.0000002	5.79091E-08	0	0	2.14545E-08	0	1.71333E-08	4.78667E-08	2.908E-07
Cellulophaga_lytica	6.65455E-08	0	0	2.41545E-08	4.44769E-07	0	0	2.70385E-07	0	0.000000068	2.76364E-08	4.06273E-07	2.06333E-07	1.87467E-07	0.000000288	3.872E-07
Cellulophaga_sp._L1A9	2.53727E-07	4.87364E-07	5.74545E-08	3.41727E-07	2.20615E-07	0.000000026	1.98154E-07	2.95385E-07	2.75455E-08	0	0.00000035	4.28182E-07	0	1.228E-07	1.61133E-07	0.000000314
Cellulophaga_algicola	1.29182E-07	0	0	0	1.36769E-07	0	1.79692E-07	7.30077E-07	2.73727E-07	1.36273E-07	3.87182E-07	3.08273E-07	1.848E-07	3.13067E-07	1.78507E-07	3.31267E-07
Siansivirga_zeaxanthinifaciens	6.55818E-07	2.32727E-08	7.40909E-08	8.02818E-07	1.42515E-06	0	1.41462E-07	4.87923E-07	6.96364E-07	6.25182E-07	1.16545E-06	1.38791E-06	9.174E-07	1.42267E-06	0.000001917	9.328E-07
Croceibacter_atlanticus	2.90182E-07	0.000000065	3.70909E-08	1.03636E-08	2.05385E-08	0	1.60692E-07	2.60615E-07	1.15636E-07	6.66364E-08	3.44364E-07	6.09091E-07	7.83333E-08	3.30333E-07	1.32067E-07	4.17933E-07
Lacinutrix_venerupis	6.27273E-08	0	0	5.72455E-08	1.86923E-08	0	1.17077E-07	1.00923E-07	0	0	3.77273E-08	2.64364E-07	9.57333E-08	5.06E-08	1.344E-07	1.68067E-07
Lacinutrix_sp._Bg11-31	9.57273E-08	0	1.42182E-07	5.64545E-08	2.88462E-08	0	0	6.82308E-08	0	0	0	2.85455E-08	4.28667E-08	1.668E-07	2.754E-07	2.39667E-07
Lacinutrix_sp._5H-3-7-4	4.13727E-07	2.35091E-07	1.86636E-07	1.80455E-07	2.10077E-07	0	0	0	4.32455E-07	4.29273E-07	6.17636E-07	1.51636E-07	3.60533E-07	2.25933E-07	1.1624E-07	3.20667E-08
Robiginitalea_biformata	8.74909E-06	1.19155E-06	5.43091E-06	7.26455E-06	2.42077E-06	1.47969E-06	5.26385E-06	5.70769E-06	4.33636E-06	3.66955E-06	5.02364E-06	4.67455E-06	3.21733E-06	3.7478E-06	2.98133E-06	3.80353E-06
Dokdonia_sp._4H-3-7-5	0	2.34545E-08	0	0	0	0	0	1.80923E-07	8.15455E-08	0	2.05455E-08	0	2.09333E-08	3.01333E-08	3.86E-08	9.30667E-08
Dokdonia_sp._PRO95	0	0	3.70909E-08	1.03273E-07	8.23077E-08	1.79231E-08	0	3.81538E-08	1.96909E-07	0	0	1.42727E-08	2.14E-08	1.61333E-08	1.57067E-08	1.21533E-07
Dokdonia_donghaensis	1.58182E-08	0	4.90909E-08	0	3.34615E-08	0	0	0	0	0	0	0	0	0	0	0
Dokdonia_sp._Dokd-P16	2.70636E-07	0	0	3.31818E-07	2.05385E-08	0	0	0	2.62727E-07	3.20727E-07	2.12455E-07	3.59727E-07	6.62667E-08	8.50667E-08	2.39333E-08	7.52E-08
Dokdonia_sp._MED134	2.59227E-06	0.000000065	0.00000171	3.27182E-06	5.79538E-07	1.11062E-06	2.54154E-06	2.73577E-06	1.41309E-06	0.000001	2.54109E-06	1.87855E-06	7.78333E-07	1.23253E-06	1.8478E-06	1.9466E-06
Urechidicola_croceus	5.38727E-07	0	1.10909E-07	4.66364E-08	9.54538E-07	0	2.04769E-07	3.03154E-07	5.24909E-07	6.26727E-07	9.64273E-07	1.37136E-06	9.45867E-07	8.86933E-07	1.21693E-06	0.000001395
Euzebyella_marina	1.65273E-05	7.14545E-07	5.91545E-06	1.53518E-05	3.74654E-06	0	1.67923E-05	0.0000135	6.95455E-06	0.00001639	8.60727E-06	7.82064E-06	3.57507E-06	4.79467E-06	3.38713E-06	0.000004124
Tenacibaculum_sp._DSM_106434	1.11182E-07	0	0	0	3.88462E-08	0	8.59231E-08	2.30923E-07	2.72727E-08	1.61273E-07	5.71273E-07	3.48182E-07	2.688E-07	0.00000053	8.38E-08	1.656E-07
Tenacibaculum_sp._SZ-18	2.00964E-06	1.04545E-08	5.41455E-07	8.93636E-08	7.07385E-07	3.29231E-07	1.51669E-06	1.65492E-06	2.44655E-06	3.57364E-06	4.16845E-06	2.52036E-06	6.35533E-07	2.088E-07	3.752E-07	1.0854E-06
Tenacibaculum_todarodis	5.57273E-08	0	0	2.43636E-08	1.47308E-07	6.53077E-08	9.26615E-07	8.63769E-07	3.94545E-08	0.000000028	1.33727E-07	1.40364E-07	1.59533E-07	0.000000751	4.72067E-07	6.654E-07
Tenacibaculum_dicentrarchi	7.65182E-07	0	1.10909E-07	1.16791E-06	6.69385E-07	0	2.15385E-07	5.13846E-07	1.60818E-06	8.86273E-07	8.83727E-07	6.59455E-07	9.20667E-07	5.678E-07	5.93133E-07	6.46467E-07
Tenacibaculum_maritimum	0.000000334	1.86545E-07	3.70909E-08	2.45545E-07	2.75385E-08	3.96154E-08	0	3.56923E-08	5.96091E-07	3.68273E-07	1.52636E-07	1.25745E-06	1.33267E-07	1.11133E-07	8.67333E-08	1.476E-07
Tenacibaculum_mesophilum	4.37091E-07	1.84364E-07	0	2.38273E-07	1.85692E-07	0	1.17615E-07	3.33077E-07	7.83182E-07	3.70273E-07	6.54182E-07	3.55045E-07	4.63467E-07	5.622E-07	3.24467E-07	3.26467E-07
Tenacibaculum_jejuense	1.62636E-07	0	4.12727E-09	4.79091E-09	9.01462E-07	3.58462E-08	5.77308E-07	5.83077E-07	9.91091E-07	4.17455E-07	6.65636E-07	9.34455E-07	4.59667E-07	9.64933E-07	7.772E-07	8.27533E-07
Maribacter_dokdonensis	3.11818E-08	0	0	1.91818E-08	0	0	0	0	0	0	2.76364E-08	0	0	0	0	0
Maribacter_cobaltidurans	2.19727E-07	2.93455E-07	1.15182E-07	1.72182E-07	0	0	0	4.93846E-08	3.44818E-07	1.88818E-07	2.76364E-08	2.14545E-08	8.98E-08	0	3.59067E-08	0.00000006
Maribacter_sp._MJ134	1.80727E-07	1.87273E-08	3.39091E-08	2.29727E-07	1.88692E-07	2.62385E-07	4.50769E-07	6.43462E-07	1.58909E-07	1.86818E-07	4.46909E-07	7.47636E-07	0.000000246	9.68667E-08	1.916E-07	1.45267E-07
Maribacter_sp._T28	1.23364E-07	0	5.87091E-08	2.39727E-08	0.000000039	0	2.07154E-07	1.83462E-07	6.14545E-08	2.16364E-07	8.06636E-07	4.91727E-07	7.66E-08	2.22733E-07	8.93333E-08	8.96667E-08
Maribacter_sp._1_2014MBL_MicDiv	0.000001597	0.00000013	2.22727E-07	4.66364E-08	1.27331E-06	0	5.26154E-08	6.32538E-07	1.65182E-06	0.000001496	7.59273E-07	1.41127E-06	1.6504E-06	1.34047E-06	8.85267E-07	1.3586E-06

Maribacter_sp._HTCC2170	5.62818E-07	0	3.70909E-08	0.00000052	0.000000301	0	8.33077E-08	1.09162E-06	6.18455E-07	6.63091E-07	7.86364E-07	9.87091E-07	2.80733E-07	5.29067E-07	2.12933E-07	8.994E-07
Nonlabens_sediminis	0	0	0	2.91545E-08	0	0	0	0	0	0	0	0	0	0	0	0
Nonlabens_marinus	8.22091E-07	1.04545E-08	4.94545E-08	5.49455E-07	1.18923E-07	0	5.74615E-08	3.81538E-08	0.000000404	4.06182E-07	2.09455E-07	1.52545E-07	0	2.10933E-07	2.02133E-07	1.61267E-07
Nonlabens_spongiae	4.56818E-07	0.000000065	3.35309E-07	1.68918E-06	5.29077E-07	2.13431E-06	2.13538E-06	2.67354E-06	7.90364E-07	1.11382E-06	1.41191E-06	1.07282E-06	7.40867E-07	4.53667E-07	7.818E-07	7.04467E-07
Nonlabens_dokdonensis	1.57091E-07	0	2.04909E-08	1.06909E-07	1.59846E-07	1.00615E-07	1.44077E-07	9.25615E-07	6.45455E-08	0	4.34455E-07	3.35909E-07	9.41333E-08	7.68E-08	2.13067E-07	7.10733E-07
Nonlabens_sp._Ci31	4.60909E-08	0	0	9.54545E-09	4.93846E-08	0	0	2.51538E-07	0	2.85455E-08	0	5.50455E-08	1.164E-07	2.16933E-07	2.0744E-07	1.03067E-07
Nonlabens_sp._MJ115	1.25245E-06	9.54545E-09	3.93636E-07	9.87273E-07	2.35077E-07	0	2.73846E-08	5.58769E-07	6.67364E-07	2.45364E-07	6.93364E-07	7.94818E-07	0.00000041	4.344E-07	3.208E-07	7.25533E-07
Nonlabens_sp._MB-3u-79	3.11818E-08	0	0	4.79091E-09	0.000000061	2.95385E-08	2.73846E-08	4.37769E-07	1.28091E-07	0	1.20545E-07	1.42727E-08	1.82067E-07	2.96133E-07	1.64533E-07	5.07333E-08
Nonlabens_sp._Hel1_33_55	1.52527E-06	8.82727E-08	1.62891E-07	4.59182E-07	3.62462E-07	0	1.10915E-06	1.23738E-06	0.00000099	7.50636E-07	1.09373E-06	7.36273E-07	1.82267E-07	4.066E-07	2.63507E-07	4.88333E-07
Myroides_sp._A21	0	0	0	0	0	0	0	0	3.98182E-08	0	0	0	0	0	0	0
Myroides_odoratimimus	0	1.16364E-08	0	9.44545E-08	0	0	0	0	0	0	6.65455E-08	2.14545E-08	0	0	0	0
Myroides_profundi	3.69091E-08	9.54545E-09	4.10909E-08	4.94545E-08	0	0	0	2.47692E-08	0	3.03636E-08	0	0	0	3.26667E-08	0	0
Myroides_phaeus	3.95545E-07	0.000000065	1.28455E-07	0.00000007	8.83846E-08	0	1.60231E-07	0.000000312	1.35909E-07	7.25455E-08	1.36909E-07	3.96091E-07	1.01667E-07	9.84E-08	7.66667E-08	4.49667E-07
Myroides_odoratus	1.89182E-07	0	0	0	8.39308E-07	0	3.06923E-08	1.46923E-08	0	6.66364E-08	0	3.94545E-08	8.08933E-07	2.02733E-06	6.67333E-07	7.76E-08
Myroides_sp._ZB35	4.58091E-07	2.70909E-08	2.59091E-07	1.32818E-07	0.000000065	0	0	2.37615E-07	0	7.25455E-08	1.88545E-07	2.52364E-07	4.17333E-08	6.57333E-08	2.05333E-07	4.76667E-08
Myroides_sp._CJ210	1.34973E-06	0	2.30091E-07	5.64909E-07	2.37923E-07	0	8.61538E-08	2.70923E-07	3.43182E-07	6.58273E-07	4.23273E-07	8.63045E-07	5.15533E-07	4.732E-07	2.85467E-07	5.218E-07
Gramella_echinicola	0.000001062	1.40391E-06	1.41455E-06	1.85627E-06	5.20769E-08	4.36308E-07	9.60308E-07	1.11646E-06	6.57909E-07	4.18727E-07	9.42818E-07	2.58636E-07	2.42533E-07	5.09333E-07	3.79867E-07	3.442E-07
Gramella_sp._MAR_2010_147	4.28727E-07	1.57045E-06	0.000001928	1.21536E-06	3.07154E-07	8.79231E-08	3.89077E-07	9.13846E-07	3.63818E-07	0.000000127	3.86636E-07	3.90545E-07	2.72667E-07	1.774E-07	2.26733E-07	4.988E-07
Gramella_salexigens	1.43182E-07	5.07818E-07	3.06818E-07	1.91818E-08	6.80769E-08	0	0	3.81538E-08	0	2.87273E-08	1.02727E-08	6.00909E-07	2.32067E-07	2.092E-07	5.01333E-08	2.83467E-07
Gramella_forsetii	1.05036E-06	0.000000065	4.12727E-09	1.26273E-07	9.38462E-07	3.20385E-07	1.62354E-06	2.35592E-06	1.39809E-06	1.04509E-06	2.18791E-06	1.38709E-06	0.000000628	9.11333E-07	1.30553E-06	1.07633E-06
Gramella_fulva	3.76418E-06	1.75182E-07	2.37636E-06	0.000003858	6.54231E-07	3.24462E-07	4.05308E-06	0.000003022	2.11155E-06	2.74582E-06	2.00309E-06	2.57982E-06	7.13333E-07	1.29347E-06	9.96267E-07	1.12627E-06
Gramella_flava	1.02727E-07	1.15364E-07	9.60909E-08	7.57273E-08	1.52462E-07	1.24846E-07	6.40846E-07	4.44385E-07	0.000000433	0.000000075	5.44818E-07	4.96545E-07	2.01533E-07	0.000000272	2.836E-07	8.79333E-08
Muricauda_sp._501str8	1.01627E-06	4.65455E-08	0.000000742	1.08945E-06	5.89769E-07	5.21692E-07	0.00000023	7.77923E-07	8.91727E-07	0.000000339	1.03536E-06	3.03818E-07	4.798E-07	3.37333E-07	2.84573E-07	4.30867E-07
Muricauda_lutaonensis	5.36909E-07	7.88182E-08	4.51182E-07	1.03282E-06	1.74615E-06	1.07115E-06	1.17492E-06	4.48923E-07	1.98136E-06	1.37336E-06	4.48909E-07	1.21436E-06	1.67767E-06	0.000001699	2.1224E-06	8.07667E-07
Muricauda_ruestringensis	0.000001982	3.16364E-07	0.000003133	0.000002902	1.18969E-06	2.52308E-08	7.17308E-06	7.36385E-06	1.78455E-06	0.000001556	1.76591E-06	2.27255E-06	1.35887E-06	1.99187E-06	2.01047E-06	2.15987E-06
Gillisia_sp._Hel1_33_143	4.82364E-07	4.54545E-07	1.53818E-07	8.75455E-07	2.22385E-07	0	3.15385E-07	2.52923E-07	7.21818E-07	1.95455E-07	3.57545E-07	3.88091E-07	5.32E-08	7.338E-07	3.464E-07	3.11733E-07
Seonamhaeicola_sp._S2-3	1.63964E-06	0	4.12727E-09	1.537E-07	5.40962E-06	8.88462E-08	2.73846E-08	7.86077E-07	1.40182E-07	1.91545E-07	6.71818E-08	0.000000523	4.0856E-06	2.9706E-06	5.10827E-06	2.5866E-06
Antarcticibacterium_flavum	6.77545E-07	3.22455E-07	4.47545E-07	3.04727E-07	2.27077E-07	3.79231E-07	1.71615E-07	0	6.28455E-07	3.98273E-07	4.71545E-07	1.39727E-07	3.45867E-07	5.58067E-07	2.20467E-07	4.81467E-07
Antarcticibacterium_sp._PAMC_28998	2.58091E-06	0	1.22636E-06	1.73355E-06	1.11608E-06	7.17923E-06	1.88492E-06	4.02231E-06	0.000002418	1.34736E-06	2.02073E-06	1.86082E-06	1.6664E-06	0.000001166	1.35527E-06	1.61447E-06
Formosa_sp._Hel1_33_131	3.11818E-08	0	1.63636E-08	0	1.56538E-07	2.36923E-07	2.73846E-08	1.71388E-05	4.80909E-08	0	2.76364E-08	0.000000348	0.000014778	2.07687E-05	1.55406E-05	8.62193E-06
Formosa_agariphila	5.66364E-07	7.63636E-07	6.33636E-08	1.80909E-07	1.79231E-07	0	4.33462E-07	1.49385E-07	0	7.25455E-08	2.85273E-07	3.70909E-07	2.14E-08	3.35333E-08	3.23267E-07	5.83333E-08
Formosa_sediminum	0	0	4.12727E-09	0	1.01923E-07	0	4.33846E-08	0.00000016	0	0	6.53636E-08	7.86182E-07	1.626E-07	7.28667E-08	1.718E-07	0.0000002
Formosa_sp._Hel1_31_208	1.54364E-07	4.61818E-07	0.000001013	0.000000409	7.48462E-08	1.89231E-08	1.67769E-07	3.76308E-07	1.81091E-07	1.89545E-07	3.81364E-07	1.15909E-07	1.58067E-07	1.00267E-07	9.67067E-08	3.11133E-07
Formosa_sp._Hel3_A1_48	7.43636E-08	0	0	0	3.33385E-07	0	0	1.45769E-07	2.49818E-07	6.66364E-08	3.11182E-07	7.33182E-07	0.000000522	4.26133E-07	1.00973E-06	5.62867E-07
Formosa_sp._L2A11	2.24227E-06	8.14545E-08	0.000000349	2.85727E-07	6.78392E-06	6.50462E-07	1.23931E-06	3.55208E-06	3.53355E-06	3.98682E-06	0.000003906	3.65045E-06	3.10407E-06	3.7422E-06	5.15207E-06	4.78373E-06
Aequorivita_sublithincola	1.81855E-06	0.000000065	8.25455E-07	1.36009E-06	9.50308E-07	7.96923E-08	1.38408E-06	1.59115E-06	1.09427E-06	1.46009E-06	1.73891E-06	1.51555E-06	5.59467E-07	6.06267E-07	9.47733E-07	9.74667E-07
Aequorivita_sp._H23M31	0.00000374	7.65864E-07	1.58973E-06	4.47336E-06	8.00692E-07	5.44769E-07	2.67615E-06	1.85892E-06	1.38727E-06	0.000001687	2.17882E-06	2.01355E-06	7.30267E-07	1.31553E-06	1.00367E-06	8.42867E-07
Altibacter_sp._ALE3EI	2.00745E-06	1.93945E-06	5.92673E-07	1.41991E-06	1.08038E-06	4.39077E-07	1.87462E-06	1.81023E-06	1.87636E-06	1.13064E-06	1.23164E-06	1.49136E-06	9.47667E-07	1.1438E-06	1.12513E-06	1.23847E-06
Aquimarina_sp._AD10	1.40636E-07	0	7.40909E-08	9.37273E-08	2.82308E-07	0	0	2.05615E-07	1.74364E-07	0.000000028	3.16364E-07	6.18273E-07	6.59333E-08	2.28533E-07	2.15973E-07	1.92733E-07
Aquimarina_sp._AD1	1.33618E-06	1.03636E-08	9.10818E-07	5.62545E-07	6.02538E-07	1.79231E-07	9.28077E-07	1.99846E-06	9.93909E-07	5.69091E-07	1.87573E-06	1.41836E-06	5.512E-07	4.23067E-07	1.4156E-06	9.01133E-07
Aquimarina_sp._TRL1	1.13764E-06	1.16364E-08	3.08127E-07	1.11173E-06	3.43538E-07	3.50538E-07	2.20769E-07	6.37538E-07	6.70909E-07	0.000001423	9.60727E-07	1.69055E-06	7.27933E-07	8.66667E-07	0.000000517	9.71667E-07
Aquimarina_sp._BL5	3.20818E-07	1.16364E-08	2.23036E-07	2.22091E-06	1.01908E-06	0	6.94769E-07	1.78231E-06	6.85909E-07	5.66909E-07	1.11218E-06	1.83764E-06	0.000001538	1.51447E-06	2.1798E-06	3.78147E-06
Winogradskyella_schleiferi	6.08182E-08	0	0	1.11455E-07	3.03385E-07	2.13846E-08	4.33846E-08	4.32308E-08	2.08091E-07	0	6.65455E-08	1.20773E-07	8.32E-08	6.18E-08	1.358E-07	1.04867E-07
Winogradskyella_sp._PG-2	0.0000002	0	4.79455E-07	2.29818E-07	2.81077E-07	0	1.10808E-06	1.02785E-06	3.89636E-07	2.48545E-07	1.83364E-07	1.58455E-07	1.14733E-07	2.238E-07	9.96667E-08	1.90667E-07
Winogradskyella_sp._J14-2	1.44155E-06	1.94545E-07	6.33273E-07	9.81364E-07	0	0	1.46408E-06	9.32462E-07	1.71436E-06	9.37818E-07	9.66818E-07	1.26545E-06	0	0	3.964E-08	0
Winogradskyella_helgolandensis	3.70364E-07	0	8.75455E-09	1.10818E-07	2.27308E-07	2.16923E-08	7.92308E-08	1.24846E-07	1.69091E-07	1.08273E-07	1.46273E-07	1.60545E-07	1.112E-07	1.99467E-07	1.4924E-07	4.10667E-08
Winogradskyella_sp._PC-19	6.43091E-07	0	4.12182E-08	1.03636E-08	0.000000791	0	1.43692E-07	1.75538E-07	1.03164E-06	1.20127E-06	4.55364E-07	1.00909E-06	1.34347E-06	1.1546E-06	9.68267E-07	0.000001123
Winogradskyella_forsetii	1.54155E-06	0	1.19091E-07	5.45636E-07	2.13985E-06	1.35385E-07	5.50769E-07	1.15608E-06	0.000002719	2.42118E-06	1.45727E-06	1.87309E-06	2.3866E-06	2.01927E-06	2.2152E-06	2.12267E-06
Winogradskyella_sediminis	1.50482E-06	1.37273E-08	3.70909E-08	0.00000107	2.92877E-06	2.77385E-07	2.06392E-06	3.92962E-06	2.35182E-06	2.00145E-06	2.21273E-06	2.82036E-06	2.79373E-06	2.69933E-06	4.13693E-06	0.000004988
Polaribacter_sp._SA4-12	0	2.70909E-08	0	0	0	4.13846E-08	0	0	3.75455E-08	0.000000028	0	0	0	4.67333E-08	5.04667E-08	0
Polaribacter_sp._KT_15	0	0	0	0	9.63077E-08	2.95385E-08	0	1.62769E-07	7.97273E-08	0	0	9.54545E-08	5.55333E-08	0	8.50667E-08	0.000000154
Polaribacter_sp._BM10	6.08182E-08	0.000000065	0	1.91818E-08	0	0	1.27154E-07	0	0	0	3.77273E-08	0	4.10667E-08	0	0	0
Polaribacter_haliotis	2.32455E-07	1.68182E-08	0	1.50727E-07	3.44692E-07	1.24154E-07	1.02846E-07	0.000000076	0.000000186	8.62727E-08	1.70182E-07	2.19364E-07	4.32867E-07	4.80267E-07	3.81733E-07	2.15667E-07
Polaribacter_sp._L12M9	0	0	0	8.76091E-08	2.26923E-08	0	0	1.92154E-07	0	1.68273E-07	5.75364E-07	2.72818E-07	2.44E-08	0	2.33307E-07	4.95333E-08
Polaribacter_sp._Hel1_33_78	4.18636E-07	0	0	2.14818E-07	9.87692E-08	0	0	9.06154E-08	2.24273E-07	6.21364E-07	3.46273E-07	3.95455E-07	1.51467E-07	3.59867E-07	2.47533E-07	1.01133E-07
Polaribacter_reichenbachii	2.56364E-08	0	2.18545E-08	1.22636E-07	1.94077E-07	0	1.41846E-07	3.44846E-07	1.10818E-07	2.13545E-07	9.64545E-08	1.67818E-07	1.15867E-07	7.76E-08	5.16E-08	4.95333E-08
Polaribacter_vadi	1.58464E-06	1.04045E-06	0.000000443	4.76455E-07	1.12285E-06	9.84308E-07	2.42085E-06	5.32846E-07	1.08245E-06	6.17636E-07	6.54364E-07	1.45291E-06	7.63733E-07	6.08933E-07	6.39067E-07	9.18667E-08
Polaribacter_sp._L3A8	1.61091E-07	0	7.40909E-08	6.02727E-08	1.62692E-07	0	4.33846E-08	3.22308E-08	0.000000054	9.35455E-08	2.63455E-07	1.24818E-07	1.09133E-07	1.10867E-07	1.29933E-07	8.48667E-08
Polaribacter_sp._ALD11	4.37818E-07	0	0	0	1.15854E-06	7.16154E-08	5.36923E-08	0.000000586	2.03182E-07	2.22818E-07	1.37909E-07	0.000000208	9.232E-07	4.87133E-07	1.34387E-06	6.08067E-07
Polaribacter_sp._MED152	0	0	0	9.54545E-09	3.58462E-08	0	0	8.28462E-08	2.72727E-08	3.62727E-08	0	1.08818E-07	0	2.466E-07	7.96E-08	1.66667E-08
Polaribacter_sp._SA4-10	3.73455E-07	0.00000039	1.57909E-07	4.16545E-07	1.71615E-07	0	3.29615E-07	5.33538E-07	2.80273E-07	2.29273E-07	2.18091E-07	4.36182E-07	1.33667E-07	4.758E-07	4.36867E-07	8.02667E-08
Polaribacter_sp._KT25b	9.97091E-07	0.00000026	3.70909E-08	6.44273E-08	1.06685E-06	0	3.06923E-08	5.81538E-08	2.48636E-07	3.48273E-07	2.67455E-07	2.00545E-07	8.75933E-07	1.45347E-06	8.606E-07	8.53667E-07
Capnocytophaga_ochracea	1.48091E-07	0	4.10909E-08	1.91818E-08	0	0	3.77692E-08	0	2.75455E-08	0	1.02727E-08	7.95455E-08	1.86667E-08	3.01333E-08	5.04667E-08	4.97333E-08
Capnocytophaga_sp._H4358	3.20909E-07	0	0	6.74545E-08	4.94154E-07	0	0	0	0.000000728	5.87182E-07	8.82364E-07	1.35382E-06	5.43667E-07	3.79533E-07	8.508E-07	4.986E-07
Capnocytophaga_stomatis	0.000000156	1.16364E-08	0	6.90909E-08	5.63846E-08	4.13846E-08	0	0	1.92818E-07	1.04545E-07	1.33273E-07	1.15909E-07	7.63333E-08	3.09333E-08	8.69333E-08	1.20333E-07
Capnocytophaga_leadbetteri	0.000000214	0	0.000000047	1.31855E-06	5.15385E-08	1.72462E-06	4.11238E-06	2.27238E-06	5.45545E-07	4.68545E-07	1.24909E-06	2.57545E-07	6.90667E-08	7.76E-08	0.00000009	2.52067E-07
Capnocytophaga_sp._FDAARGOS_737	2.30909E-08	0	0	4.36364E-08	6.33846E-08	0	0	0	1.90909E-07	0.000000028	6.66364E-08	1.42727E-08	0.000000088	3.09333E-08	2.11133E-07	0
Capnocytophaga_sp._oral_taxon_902	1.66909E-07	0	0	0	1.47231E-07	1.79231E-08	2.86462E-07	2.97846E-07	2.21818E-07	6.92727E-08	4.08636E-07	5.38364E-07	5.02667E-08	7.88E-08	5.77933E-07	2.442E-07
Capnocytophaga_sp._oral_taxon_323	4.34182E-07	0	0	2.25091E-07	7.02308E-08	0	0	3.00769E-08	4.03727E-07	8.08182E-08	2.73364E-07	2.40909E-07	4.18667E-08	2.17667E-07	1.93067E-07	1.04733E-07
Capnocytophaga_cynodegmi	0.000001336	0	5.05636E-07	1.26418E-06	4.20538E-07	1.01154E-07	1.66862E-06	1.01377E-06	1.05036E-06	1.52791E-06	1.69418E-06	1.75927E-06	5.58533E-07	2.63067E-07	5.82467E-07	1.02413E-06
Capnocytophaga_sp._oral_taxon_878	9.93636E-08	4.11818E-08	1.23636E-08	5.96364E-08	0	0	0	0	8.91818E-08	1.04273E-07	3.33636E-08	0	4.27333E-08	1.93333E-07	1.30307E-07	6.33333E-08
Capnocytophaga_endodontalis	4.55818E-07	8.13636E-08	2.62727E-07	4.73336E-07	7.46923E-08	2.52308E-08	7.40769E-08	2.96769E-07	0	5.85818E-07	3.36364E-08	2.16818E-07	1.70533E-07	1.94333E-07	0.00000022	3.66733E-07
Capnocytophaga_haemolytica	2.36327E-06	1.16364E-08	1.11818E-07	2.03545E-06	1.40692E-07	5.23077E-08	3.13385E-07	3.45154E-07	1.81091E-06	4.16464E-06	1.68355E-06	0.000001801	7.90933E-07	2.25873E-06	1.84747E-06	1.47753E-06
Capnocytophaga_gingivalis	2.80636E-06	0	7.57855E-07	2.66109E-06	1.17785E-06	6.15615E-07	3.87408E-06	3.37077E-06	1.18364E-06	1.24482E-06	1.85555E-06	2.14427E-06	9.138E-07	1.49347E-06	1.4002E-06	2.01953E-06
Capnocytophaga_sputigena	5.32455E-07	2.13636E-07	9.15182E-07	6.28818E-07	1.16792E-06	1.08769E-07	8.92308E-08	5.48769E-07	0.0000011	1.06373E-06	1.04182E-06	1.08209E-06	8.07467E-07	1.3734E-06	1.0826E-06	1.40107E-06
Capnocytophaga_sp._oral_taxon_864	0.000001514	0.000000065	4.978E-07	2.94636E-07	2.70154E-06	2.95385E-08	1.12185E-06	1.12631E-06	2.06827E-06	2.33082E-06	2.12918E-06	1.60082E-06	0.000003592	2.69493E-06	2.29433E-06	2.73687E-06
Capnocytophaga_canimorsus	8.75727E-06	1.16364E-08	4.03636E-06	3.25727E-06	2.39925E-05	3.65231E-07	1.39615E-06	2.33908E-06	3.86636E-05	2.99055E-05	3.99727E-05	3.68455E-05	0.000022844	1.99893E-05	2.47093E-05	0.000021316
Flavobacterium_sp._M31R6	1.41091E-07	0	0	4.79091E-09	3.15385E-08	0	0	9.43077E-08	3.75455E-08	0	8.93636E-08	2.14545E-08	0	0	1.93733E-08	2.44E-08
Flavobacterium_arcticum	1.39727E-07	0	2.47273E-08	2.99091E-08	2.28769E-07	0	0	0	0	1.22182E-07	1.30909E-07	7.13636E-09	2.11467E-07	4.25133E-07	2.57507E-07	9.32E-08
Flavobacterium_branchiophilum	1.88273E-07	0	0	0.000000219	0	4.13846E-08	0	0.000000011	2.45455E-07	1.51091E-07	1.13636E-07	7.79636E-07	4.23333E-08	2.41667E-07	1.0984E-07	6.43333E-08
Flavobacterium_indicum	0	0	0	2.89091E-08	0	0	0	3.00769E-08	2.64545E-08	1.63545E-07	5.99091E-08	0	0	1.61333E-08	0	0
Flavobacterium_commune	3.71455E-07	0	1.72909E-07	0.000000083	3.80231E-07	0	0	0	6.10091E-07	2.46364E-07	1.08909E-06	1.03891E-06	1.43267E-07	5.214E-07	7.60173E-07	4.37933E-07
Flavobacterium_sp._HYN0086	1.82182E-07	0	0	0.000000177	2.09077E-07	8.90769E-08	0.00000033	3.62769E-07	1.60818E-07	1.28636E-07	2.64818E-07	3.23364E-07	2.186E-07	2.83667E-07	5.25973E-07	2.29533E-07
Flavobacterium_sp._CJ74	8.62818E-07	2.83273E-07	1.03082E-06	4.36727E-07	3.28538E-07	4.08846E-07	3.28308E-07	4.77615E-07	3.13727E-07	2.53545E-07	0.00000069	4.47273E-07	2.348E-07	4.78667E-07	1.48267E-07	5.22867E-07
Flavobacterium_sp._KBS0721	1.15445E-06	3.45455E-07	4.33391E-07	1.00745E-06	0.000000815	6.45385E-08	8.47846E-07	4.44308E-07	7.84455E-07	4.73545E-07	1.17818E-06	1.45818E-06	1.83933E-07	0.000000303	3.474E-07	7.38067E-07
Flavobacterium_sp._Sr18	2.50727E-07	2.09273E-07	1.288E-07	1.88727E-07	1.43538E-07	5.17538E-07	1.63923E-07	4.82538E-07	1.49091E-07	8.32727E-08	2.66909E-07	1.80545E-07	8.08667E-08	9.38E-08	1.118E-07	5.83333E-08
Flavobacterium_sp._I3-2	1.74545E-06	4.16364E-08	8.50909E-08	0	1.46231E-07	0	1.07815E-06	1.82954E-06	1.91027E-06	2.35127E-06	2.82609E-06	1.22573E-06	6.98533E-07	3.43067E-07	2.43933E-07	7.15733E-07
Flavobacterium_pallidum	5.48091E-07	1.34136E-07	4.41991E-07	0.000000571	1.32462E-07	2.06615E-07	3.52231E-07	3.86462E-07	5.30818E-07	2.24727E-07	2.90455E-07	2.15818E-07	1.63467E-07	3.384E-07	1.61733E-07	3.888E-07
Flavobacterium_nitrogenifigens	5.08909E-07	0.00000013	6.58182E-08	3.30636E-07	4.13308E-07	0	0	1.94538E-07	6.95727E-07	1.14364E-07	7.90091E-07	2.30909E-07	4.972E-07	2.89733E-07	2.68133E-07	2.942E-07
Flavobacterium_kingsejongi	0.000000123	0	2.67818E-07	1.39818E-07	8.33846E-08	2.38308E-07	1.96692E-07	1.90769E-08	2.59545E-07	0	3.42364E-07	1.85273E-07	1.77467E-07	1.178E-07	1.708E-07	9.87333E-08
Flavobacterium_crassostreae	5.19727E-07	1.16364E-07	1.80727E-07	6.17727E-07	2.51692E-07	9.33077E-08	1.51538E-07	0	3.05455E-07	2.85091E-07	1.92182E-07	1.17273E-07	6.84E-08	7.68667E-08	8.06E-08	2.89533E-07
Flavobacterium_psychrophilum	3.42909E-07	2.15436E-06	2.44345E-06	1.06036E-06	4.92077E-07	4.47692E-07	5.15692E-07	1.12423E-06	8.45818E-07	5.55455E-07	1.19091E-06	7.30545E-07	7.354E-07	4.784E-07	0.000000535	3.04733E-07
Flavobacterium_crocinum	2.81182E-07	0	4.12727E-09	1.03636E-08	1.72615E-07	4.20769E-07	2.91385E-07	4.69385E-07	5.83545E-07	0.000000318	3.79636E-07	1.20318E-06	1.762E-07	3.33933E-07	4.066E-07	0.000000667
Flavobacterium_anhuiense	9.53182E-07	0.000000065	3.70909E-08	3.87273E-07	6.12385E-07	1.92615E-07	7.19615E-07	1.97208E-06	5.45455E-07	6.37636E-07	1.47591E-06	1.44236E-06	6.604E-07	7.15333E-07	9.08333E-07	8.06733E-07
Flavobacterium_album	2.31355E-06	9.86091E-07	1.68003E-06	3.17455E-06	1.03931E-06	1.30462E-07	2.46538E-06	1.76138E-06	1.20818E-06	9.99455E-07	1.84609E-06	1.87218E-06	9.02733E-07	7.35333E-07	1.70793E-06	1.62633E-06
Flavobacterium_gilvum	2.17545E-07	1.39091E-07	1.06273E-06	6.60273E-07	8.65923E-07	5.34615E-07	0	0.000000011	0.000000649	1.16018E-06	6.60727E-07	1.06464E-06	6.154E-07	0.000001065	7.002E-07	1.07347E-06
Flavobacterium_faecale	3.03909E-07	4.65455E-08	3.26909E-07	2.13818E-07	1.34769E-07	3.58462E-08	7.40923E-07	6.47538E-07	2.71364E-07	6.31727E-07	2.04273E-07	6.86273E-07	1.244E-07	2.18533E-07	5.98533E-07	0.000000523
Flavobacterium_haoranii	5.42727E-08	1.37273E-08	0	4.79091E-09	7.35692E-07	0	4.46385E-07	6.00077E-07	5.55273E-07	1.18909E-07	2.44636E-07	6.23364E-07	0.00000063	0.000000748	8.56667E-07	7.57733E-07
Flavobacterium_sangjuense	1.79091E-07	0	0	0	3.74923E-07	9.72308E-08	0	0.000000011	1.27545E-07	0	2.52818E-07	1.42364E-07	3.76867E-07	4.364E-07	7.42933E-07	3.99333E-07
Flavobacterium_nackdongense	2.17909E-07	5.18364E-07	1.65455E-08	8.33091E-07	4.40769E-07	2.16923E-08	5.49308E-07	5.68385E-07	3.01455E-07	3.02182E-07	3.17636E-07	3.90364E-07	1.60933E-07	5.30933E-07	5.19867E-07	3.03533E-07
Flavobacterium_johnsoniae	4.15545E-07	2.51364E-07	1.10273E-06	4.41818E-07	6.06846E-07	0	0	4.93846E-08	8.18091E-07	8.54727E-07	0.000000458	9.07545E-07	7.28733E-07	1.27007E-06	1.4592E-06	6.282E-07
Flavobacterium_columnare	4.98182E-07	0	7.40909E-08	6.10545E-07	7.98615E-07	6.44615E-08	0.000000158	6.94615E-07	6.09909E-07	5.47182E-07	6.00364E-07	8.01455E-07	6.70867E-07	7.648E-07	7.564E-07	7.46867E-07
Flavobacterium_magnum	3.55909E-06	5.74409E-07	0.000003516	2.61455E-06	4.98923E-07	0.0000006	4.91085E-06	5.06492E-06	0.000001377	1.20536E-06	2.14882E-06	0.000002145	5.728E-07	1.4584E-06	1.2688E-06	1.42867E-06
Flavobacterium_sediminis	1.56136E-06	1.94545E-07	4.75455E-07	1.31791E-06	7.94923E-07	1.71385E-07	1.05923E-06	1.24646E-06	0.000001193	8.21727E-07	1.12755E-06	1.42545E-06	1.18987E-06	1.26207E-06	1.39493E-06	2.15547E-06
Flavobacterium_alkalisoli	1.56064E-05	0.00000039	5.36018E-06	1.33079E-05	3.87062E-06	5.91538E-08	1.44308E-05	1.00308E-05	7.72636E-06	5.00012818	3.39091E-06	6.18382E-06	3.11907E-06	0.000003786	3.03807E-06	0.00003
Flavobacterium_sp._xlx-214	8.97455E-07	1.08909E-07	0	5.90727E-07	3.46231E-07	9.36923E-08	0	8.44615E-08	6.26545E-07	5.03818E-07	6.18545E-07	0.000000785	2.29933E-07	3.70467E-07	4.17133E-07	5.888E-07
Flavobacterium_sp._P2-65	7.43727E-07	0	0	4.66818E-07	4.11231E-07	1.11615E-07	0.000000804	1.43423E-06	4.72364E-07	5.94909E-07	7.39091E-07	5.46545E-07	3.62133E-07	4.09267E-07	6.458E-07	3.92933E-07
Flavobacterium_sp._LPB0248	2.96364E-08	0	3.36455E-07	1.56545E-07	2.92462E-07	2.52308E-08	0	1.90769E-08	2.07091E-07	2.99455E-07	1.99273E-07	1.80091E-07	1.218E-07	1.84133E-07	3.1224E-07	2.80733E-07
Flavobacterium_sp._140616W15	2.11455E-07	0.000000065	0	4.79091E-09	2.79846E-07	0	5.54615E-08	1.15385E-07	1.20727E-07	5.22727E-08	3.33636E-08	8.36364E-08	3.18533E-07	3.37867E-07	3.928E-07	4.38133E-07
Flavobacterium_sp._MDT1-60	9.12909E-07	0	3.96091E-07	1.10491E-06	2.08946E-06	4.57154E-07	7.54615E-08	5.26385E-07	7.65909E-07	4.12818E-07	5.72273E-07	6.77273E-07	9.40333E-07	1.07593E-06	1.10307E-06	1.02647E-06
Salinivirga_cyanobacteriivorans	1.72209E-06	2.84545E-08	3.01127E-07	4.19973E-06	8.26154E-07	0	6.41538E-08	5.80615E-07	2.12455E-06	2.09327E-06	1.92518E-06	2.94955E-06	0.000001235	1.7826E-06	1.5786E-06	1.88673E-06
Labilibaculum_antarcticum	3.35864E-06	0.000000632	3.16503E-06	3.33818E-06	0.000003692	2.76308E-07	4.52538E-07	1.76546E-06	3.41182E-06	0.000003951	4.38727E-06	3.94909E-06	3.30667E-06	0.000004408	0.00000441	0.000002948
Alkalitalea_saponilacus	1.10718E-05	6.57227E-07	2.49391E-06	1.01309E-05	3.15585E-06	9.72308E-08	2.12477E-06	0.000004483	1.02155E-05	1.25973E-05	0.00000961	1.06827E-05	3.64087E-06	6.23827E-06	6.3234E-06	6.19267E-06
Draconibacterium_sp._M1	3.37109E-06	2.32364E-07	2.33073E-07	2.67927E-06	3.55277E-06	5.77077E-07	1.70185E-06	3.24377E-06	0.00000605	3.13818E-06	5.75636E-06	5.33909E-06	3.60353E-06	3.39333E-06	0.00000374	0.000003096
Draconibacterium_orientale	6.53364E-06	1.42727E-06	4.69473E-06	7.55545E-06	3.43308E-06	8.06154E-08	5.42154E-06	4.99846E-06	4.27545E-06	4.56091E-06	4.78545E-06	4.49364E-06	0.000003094	4.32267E-06	4.98867E-06	5.14467E-06
Tenuifilum_thalassicum	5.43818E-06	6.34182E-07	2.94997E-06	4.76091E-06	2.97323E-06	1.51838E-06	4.04808E-06	3.83077E-06	4.06818E-06	2.42509E-06	0.0000048	4.57364E-06	3.3186E-06	0.0000033	0.00000479	0.000006552
Paludibacter_propionicigenes	1.12291E-05	1.70773E-06	6.21818E-06	1.47391E-05	8.37615E-06	3.91231E-07	8.48615E-06	1.10415E-05	1.15255E-05	9.96182E-06	1.23282E-05	0.00001272	9.56267E-06	1.09673E-05	9.03267E-06	0.000012524
Proteiniphilum_saccharofermentans	2.54745E-05	1.38618E-06	1.03764E-05	2.77455E-05	1.31685E-05	1.82132E-05	2.38531E-05	2.90538E-05	1.98018E-05	1.80618E-05	2.39364E-05	2.47355E-05	1.65487E-05	1.89333E-05	2.45333E-05	2.62507E-05
Dysgonomonas_sp._HDW5A	3.21364E-07	0	0	1.03636E-08	3.78615E-07	0	0	0	4.94636E-07	2.45818E-07	6.31636E-07	3.72909E-07	6.48467E-07	3.45867E-07	2.88667E-07	6.82467E-07
Dysgonomonas_sp._HDW5B	3.13964E-06	2.39545E-07	2.79636E-06	4.62909E-06	2.15969E-06	9.16923E-08	1.89231E-06	1.84438E-06	3.31818E-06	3.13464E-06	4.23745E-06	3.69818E-06	1.99793E-06	0.000003075	2.55467E-06	2.59707E-06
Petrimonas_mucosa	8.49671E-05	4.78182E-06	2.27611E-05	6.63818E-05	0.000077765	8.54077E-06	4.23538E-05	0.0000525	9.34665E-05	9.11558E-05	7.84759E-05	8.68414E-05	0.000116172	0.000102741	8.59006E-05	9.82327E-05
Porphyromonas_cangingivalis	2.81182E-05	1.82218E-06	1.04762E-05	2.20664E-05	1.24608E-05	1.23035E-05	1.12408E-05	1.70638E-05	2.65273E-05	2.08836E-05	2.57909E-05	1.77345E-05	1.87073E-05	0.000014114	0.000016556	1.66013E-05
Porphyromonas_crevioricanis	1.47664E-05	1.35182E-06	2.70345E-05	3.28027E-05	9.51615E-06	1.51625E-05	7.07127E-05	6.74275E-05	1.80309E-05	1.82809E-05	1.51252E-05	1.34027E-05	1.10513E-05	1.10653E-05	0.000016444	1.37853E-05
Porphyromonas_gingivalis	7.61105E-05	2.57484E-05	4.70182E-05	7.01818E-05	6.26308E-05	2.65023E-05	0.000036	5.22923E-05	8.96635E-05	7.74488E-05	7.77177E-05	7.51492E-05	0.000086413	7.42119E-05	7.14357E-05	7.99409E-05
Porphyromonas_asaccharolytica	0.000280202	2.77708E-05	0.000189303	0.000296198	0.000104559	8.29501E-05	0.000287037	0.000321905	0.000190226	0.000207865	0.00023879	0.000218072	0.000150656	0.000125023	0.00013687	0.000195134
Barnesiella_viscericola	0.000605566	4.28857E-05	0.000282453	0.000515095	0.000236919	0.000482211	0.000496663	0.0005896	0.000458593	0.000450819	0.000551144	0.000433356	0.000322202	0.000313937	0.000316333	0.000372778
Butyricimonas_faecalis	0.000509054	0.000341434	0.00081642	0.000574041	0.000801408	0.000149956	0.000342374	0.000372358	0.000784206	0.000829969	0.000689412	0.000665919	0.00078057	0.00099169	0.0008137	0.000705027
Odoribacter_splanchnicus	0.000964669	0.000329318	0.000389248	0.000591693	0.000886578	0.000315092	0.00099118	0.001241355	0.001154479	0.001151697	0.001313638	0.001388553	0.000776683	0.001070774	0.001405282	0.001032894
Alloprevotella_sp._E39	0.000132583	0.000008095	6.84935E-05	0.000123744	0.000077593	0.000127168	6.13395E-05	8.47966E-05	0.000168911	0.000193174	0.000209541	0.000160421	0.000114338	0.000112895	0.00010084	0.000108459
Paraprevotella_xylaniphila	0.000314821	7.32068E-05	0.000283201	0.0002638	0.000369657	7.00458E-05	0.000293643	0.000282027	0.000552127	0.00050068	0.000503522	0.00046789	0.000468508	0.000440814	0.00040858	0.000432535
Prevotella_scopos	1.55491E-05	0.000000835	4.5633E-06	1.12136E-05	1.34054E-05	7.87069E-08	3.81946E-06	4.18385E-06	1.77327E-05	1.45427E-05	1.37773E-05	1.38436E-05	0.00001914	1.53553E-05	1.37907E-05	1.65013E-05
Prevotella_jejuni	9.24132E-05	0.000031814	6.43273E-05	8.94013E-05	4.96308E-05	6.00764E-05	0.00006621	7.80923E-05	8.33743E-05	8.68427E-05	7.84545E-05	0.0000685	6.97255E-05	6.30063E-05	6.19872E-05	7.03733E-05
Prevotella_sp._oral_taxon_299	0.00003319	3.70091E-07	1.62414E-05	5.28638E-05	4.73328E-05	8.15008E-06	1.79469E-05	1.92262E-05	7.66954E-05	8.67438E-05	5.86719E-05	5.97818E-05	6.33937E-05	6.43438E-05	5.62753E-05	5.14333E-05
Prevotella_oris	9.84126E-05	2.55332E-06	2.14854E-05	6.61853E-05	0.000135232	2.39179E-05	2.18477E-05	2.61215E-05	0.000158618	0.000175444	0.000109645	0.000125218	0.000197562	0.000172747	0.00014895	0.000151533
Prevotella_fusca	0.000130099	1.96664E-06	1.11175E-05	3.90791E-05	0.000189578	1.03028E-05	1.33338E-05	2.51646E-05	0.000177722	0.000190896	0.000108614	0.000134973	0.000272867	0.000232188	0.000188325	0.000194888
Prevotella_enoeca	0.000141534	1.61673E-06	4.49858E-05	0.000153146	0.000188114	1.87535E-05	2.62385E-05	0.000047252	0.00027291	0.000293278	0.00019204	0.00021081	0.000266025	0.000250101	0.000210948	0.000205493
Prevotella_denticola	0.000209866	6.14673E-06	7.14928E-05	0.000209857	0.00021615	2.30028E-05	6.10802E-05	8.46701E-05	0.00031285	0.000335975	0.000230874	0.00026356	0.000316283	0.000285247	0.000247734	0.000269394
Prevotella_intermedia	0.000266706	3.40062E-05	0.000202335	0.000308434	0.000448169	4.27173E-05	6.84288E-05	8.84331E-05	0.000541268	0.000592506	0.000402607	0.000438245	0.000549083	0.000559247	0.000447343	0.000416762

Prevotella_dentalis	0.000471317	1.51673E-05	0.000150262	0.000371412	0.000453146	0.000272011	0.000254607	0.000297272	0.000603123	0.000633948	0.000468182	0.000466885	0.000660728	0.000600541	0.000522091	0.000533959
Prevotella_melaninogenica	0.000294902	9.43045E-06	4.40091E-05	5.92003E-05	0.000513479	0.000160316	4.82781E-05	5.91248E-05	0.000442454	0.000469928	0.000252136	0.000306005	0.000722451	0.000604005	0.00048351	0.000480186
Prevotella_ruminicola	0.000347274	0.000004037	4.77341E-05	0.00016941	0.000516203	6.85758E-05	5.24231E-05	7.34605E-05	0.00053926	0.000586688	0.0003314	0.000402428	0.000760453	0.000642424	0.000526168	0.000549862
Mucinivorans_hirudinis	4.61655E-05	0.000006204	4.50927E-05	4.03973E-05	1.54292E-05	3.62097E-05	3.91462E-05	5.10538E-05	0.00002495	3.26182E-05	3.65091E-05	3.47273E-05	0.000017742	0.00002244	2.20933E-05	2.50733E-05
Alistipes_sp._dk3624	0.000459729	3.23436E-05	0.000287262	0.000529097	0.000321022	0.000166249	0.000624127	0.000590357	0.000555245	0.000498782	0.000591513	0.000500778	0.000388454	0.000392825	0.000433791	0.000400093
Alistipes_indistinctus	0.000463889	9.07948E-05	0.000392196	0.000474521	0.000375575	0.000144816	0.000547861	0.000557651	0.000516366	0.000520594	0.000542567	0.00047652	0.000448183	0.000472623	0.00044728	0.00041058
Alistipes_dispar	0.004134474	0.00092011	0.005400578	0.004134319	0.000907989	0.000873828	0.007005628	0.006389733	0.001791777	0.002980042	0.003181977	0.003160395	0.001072428	0.001492613	0.001772859	0.001150369
Alistipes_megaguti	0.002707187	0.000556692	0.003778409	0.003224178	0.000959242	0.000763769	0.006201625	0.005677936	0.001593204	0.002351406	0.002321025	0.002315459	0.001194984	0.001343941	0.001716295	0.001161868
Alistipes_finegoldii	0.003090051	0.000653321	0.004070334	0.003926842	0.000969088	0.001601032	0.007539191	0.006470997	0.001813946	0.002697104	0.002960132	0.002760262	0.001232222	0.001432806	0.001878149	0.001267209
Alistipes_onderdonkii	0.003149184	0.000642227	0.004356236	0.003887284	0.001109679	0.000798853	0.006264746	0.005700902	0.002120312	0.003012987	0.00313337	0.002934315	0.001462639	0.001740503	0.002242956	0.001654611
Alistipes_communis	0.004076476	0.000938393	0.005517731	0.005176538	0.001922592	0.002939346	0.010239596	0.008182969	0.002651794	0.003832544	0.004277323	0.004198563	0.00234201	0.002324311	0.002868707	0.002250796
Alistipes_shahii	0.004291397	0.001165366	0.006471961	0.006062893	0.002019727	0.001096	0.010040687	0.008678115	0.003002865	0.003993573	0.004803466	0.004676384	0.003280317	0.002806492	0.004547132	0.003222285
Tannerella_forsythia	0.000102647	6.70795E-06	3.79636E-05	8.58212E-05	4.30077E-05	4.09365E-05	6.13704E-05	9.52213E-05	7.92635E-05	7.42606E-05	8.37937E-05	7.50463E-05	7.66901E-05	0.00006284	6.08267E-05	7.24926E-05
Tannerella_sp._oral_taxon_HOT-286	0.000218867	4.33586E-05	0.000188478	0.000209288	0.000173159	0.000229815	0.000222323	0.000229822	0.000230234	0.000225537	0.000216152	0.000187964	0.000212263	0.000227013	0.000200794	0.000204169
Parabacteroides_sp._CT06	0.000206347	0.000059167	9.58292E-05	0.000164996	0.000271843	0.000438934	0.000108973	0.000180879	0.000379153	0.000380183	0.000376602	0.0003698	0.000379808	0.000409157	0.000411218	0.000390693
Parabacteroides_distasonis	0.009448961	0.003961252	0.006537079	0.006781155	0.018899134	0.040509165	0.011416547	0.014534387	0.026767526	0.027193238	0.02759558	0.025376757	0.026176566	0.028979022	0.029470535	0.024935471
Phocaeicola_salanitronis	0.000381839	7.69613E-05	0.000369984	0.000360118	0.000636801	0.000123856	0.000183926	0.000236579	0.000869401	0.000816372	0.000814747	0.000795875	0.000670279	0.000733058	0.000642208	0.000577454
Phocaeicola_dorei	0.007556506	0.008707358	0.038441602	0.014549398	0.042824503	0.000735925	0.000739477	0.000784616	0.033267958	0.037790629	0.02642617	0.028048566	0.033868373	0.049191848	0.035046019	0.027098993
Phocaeicola_vulgatus	0.007757308	0.009303731	0.043256256	0.015473741	0.048158278	0.000382154	0.000586169	0.000602318	0.036276736	0.041794081	0.028297977	0.03001276	0.037801337	0.055805193	0.039017901	0.029991432
Muribaculum_sp._TLL-A4	0.045265552	0.00149198	0.1035837	0.04464547	0.048663696	0.021505729	0.161110177	0.133346451	0.057302727	0.075790511	0.098451524	0.036640198	0.074286287	0.089360489	0.133271014	0.131691656
Muribaculum_intestinale	0.07273562	0.011611871	0.059654025	0.110216193	0.041644951	0.016928869	0.082399946	0.11504372	0.059933792	0.062689702	0.087142803	0.095045297	0.059837207	0.050427398	0.054124914	0.077752309
Duncaniella_dubosii	0.140265472	0.009395056	0.04552558	0.127802307	0.030311361	0.172166999	0.103411145	0.110864485	0.048645841	0.064817289	0.065120637	0.093388081	0.058271019	0.039158161	0.059578499	0.062440942
Duncaniella_sp._B8	0.000327179	2.07547E-05	0.000278699	0.000550401	0.000118071	6.05255E-05	0.000298636	0.000407343	0.000212664	0.000249014	0.000322538	0.000200523	0.000240841	0.000216553	0.000158073	0.000295823
Duncaniella_sp._C9	0.000971683	4.97444E-05	6.09513E-05	0.000886479	0.000310771	0.000187049	0.000592043	0.000909667	0.000539906	0.000427576	0.000710228	0.000404482	0.000429559	0.000544817	0.000444502	0.000621766
Bacteroides_heparinolyticus	0.000158225	7.39712E-05	0.000159035	0.000102856	0.000302058	4.18026E-05	4.52769E-05	4.98923E-05	0.000493022	0.000374948	0.000429101	0.000440639	0.000325376	0.000306884	0.000272509	0.000262673
Bacteroides_helcogenes	0.000255787	6.16263E-05	0.000187894	0.000141737	0.000499936	0.000101791	5.98656E-05	7.20385E-05	0.000874455	0.000654932	0.000747346	0.000780955	0.000515014	0.000467181	0.000447043	0.000428984
Bacteroides_cellulosilyticus	0.000250263	0.000143732	0.000302367	0.000248529	0.000357266	4.19336E-05	0.000152585	0.000137367	0.000536995	0.000475597	0.000453789	0.000457737	0.000396663	0.00038161	0.000340318	0.000335765
Bacteroides_zoogleoformans	0.000198041	0.000187921	0.000345268	0.000239667	0.000414058	0.000037156	7.23767E-05	0.000076055	0.000617686	0.000527339	0.000533144	0.000532438	0.000432847	0.0004502	0.000391746	0.000366798
Bacteroides_intestinalis	0.00042795	0.000303458	0.001048431	0.000545782	0.001358137	0.000590405	0.000354063	0.000185394	0.001259146	0.001229823	0.001076993	0.001085269	0.001180714	0.00148113	0.001199608	0.001015368
Bacteroides_thetaiotaomicron	0.017983423	0.008066322	0.008120416	0.004370557	0.001622419	0.001045664	0.001552764	0.001254868	0.00530656	0.003997244	0.006250615	0.004602002	0.002145615	0.001738513	0.001542982	0.001593656
Bacteroides_xylanisolvens	0.001292157	0.007314315	0.007694465	0.003852118	0.002018675	0.001288067	0.00202494	0.001640639	0.003135839	0.002073059	0.002315293	0.0018835	0.002478638	0.002200123	0.001695562	0.001757269
Bacteroides_ovatus	0.002005601	0.010070033	0.010179279	0.005231376	0.001988154	0.001448837	0.002330347	0.00189298	0.003795595	0.002379002	0.003171093	0.002502014	0.002739919	0.00208938	0.001791622	0.001964892
Bacteroides_caccae	0.001714245	0.010439183	0.010779475	0.005674681	0.002185676	0.000781568	0.002746228	0.002526485	0.004178821	0.002605192	0.003170329	0.002523459	0.003022453	0.002424311	0.0020052	0.002177651
Bacteroides_fragilis	0.00340106	0.002638842	0.00484347	0.003805342	0.005512242	0.00151656	0.002490042	0.002552076	0.006500266	0.006062508	0.005590831	0.00536696	0.006128257	0.006375473	0.005645942	0.005163258
Bacteroides_coprosuis	0.005064025	0.001952194	0.00425347	0.004486081	0.005390648	0.0007002	0.002865208	0.003213578	0.006142244	0.005604639	0.005094709	0.004293009	0.007154971	0.00673949	0.00572411	0.005824072
Bacteroides_uniformis	0.005597142	0.000329482	0.002270887	0.001679936	0.013618461	0.000367839	0.000312155	0.000241132	0.024463943	0.017535769	0.019959951	0.020460169	0.013758108	0.011531105	0.011286688	0.01052278
Bacteroides_caecimuris	0.020474464	0.115484466	0.118513383	0.06274859	0.029768058	0.036340936	0.038445679	0.02840055	0.04932972	0.031888459	0.037665543	0.030307167	0.039770431	0.03286866	0.026226002	0.028367952
Bacteroides_sp._PHL_2737	7.13891E-05	0.000009652	0.00003774	4.90109E-05	6.40462E-05	1.55285E-05	1.53701E-05	2.72169E-05	9.36286E-05	9.15501E-05	9.03522E-05	9.62704E-05	6.71561E-05	0.000078327	7.75493E-05	6.71212E-05
Bacteroides_sp._M10	0.000266998	0.001499975	0.001555977	0.000806355	0.000373384	0.000306645	0.000403993	0.000323952	0.000648431	0.000410096	0.000505845	0.000412497	0.000499048	0.000395951	0.000327219	0.000350856
Bacteroides_sp._A1C1	0.000818246	0.000242236	0.000618618	0.000364361	0.002022567	2.25604E-05	1.35346E-05	1.67431E-05	0.003465698	0.002592474	0.002958356	0.002942039	0.002017503	0.001771706	0.001692233	0.001559336
Bacteroides_sp._HF-162	0.00095111	3.10948E-05	0.000466418	0.000403695	0.002089304	0.000104164	0.000184486	0.000219649	0.003667591	0.002743195	0.003154455	0.003217242	0.002119635	0.001892266	0.001876277	0.001732853
Bacteroides_sp._CACC_737	0.00099353	7.36704E-05	0.000502574	0.000358773	0.002366272	7.15388E-05	6.09319E-05	7.23005E-05	0.00415291	0.003013532	0.003506333	0.003525594	0.002360307	0.002038923	0.001970738	0.001833006
Bacteroides_sp._HF-5141	0.000759664	0.004705707	0.004868303	0.002576911	0.001095184	0.00115753	0.001522358	0.001215177	0.001958997	0.001205853	0.001472013	0.001183325	0.001489785	0.001202875	0.000973087	0.001054171
Bacteroides_sp._HF-5287	0.001511277	0.004074828	0.00472862	0.002810304	0.001772372	0.000686604	0.001388577	0.001415717	0.002816493	0.002269815	0.002576961	0.002049399	0.002271812	0.002046608	0.001739365	0.001759375
Bacteroides_sp._CBA7301	0.013675174	0.098442636	0.098032493	0.049769124	0.017280524	0.00509326	0.023520906	0.02148684	0.035008013	0.020293759	0.02543993	0.019472364	0.025091611	0.018898199	0.014896656	0.016691745
