# Supplementary material for: Host circadian behaviors exert only weak selective pressure on the gut microbiome under stable conditions but are critical for recovery from antibiotic treatment
Source: PLoS Biol. 2022 Nov 9;20(11):e3001865. doi: 10.1371/journal.pbio.3001865 (PMC9645659; doi:10.1371/journal.pbio.3001865)
Supplement: S8 Table — Each value here is the mean relative abundance of the chosen genus from all the biological replicates at a given time point for the WT(T), Per(T), WT(UT), or Per(UT) samples, which were used to derive the volcano plots in S6 Fig. Columns are tabulated as in S7 Table. (PDF) [file pbio.3001865.s016.pdf]

S8 Table. Relative bacterial abundances at the genus level (total sequencing reads for each genus are divided by total sequencing reads per sample). Each value here is the mean relative abundance of the chosen genus from all the biological replicates at a given time point for the WT(T), Per(T), WT(UT) or Per(UT) samples, which were used to derive the volcano plots in S6 Fig. Columns are tabulated as in S7 Table.

S8 Table

Relative bacterial abundances(mean value for each genus at each time point) as genera that were used to derive the volcano plots in S6 Fig.

Genus	WT(T) Relative Abundance				Per(T) Relative Abundance				WT(UT) Relative Abundance				Per(UT) Relative Abundance			
	-14	11	154	238	-14	11	154	238	-14	11	154	238	-14	11	154	238
Acetobacterium	1.5494E-05	8.7321E-06	4.2241E-06	0.00000944	8.0269E-06	4.6128E-06	9.5054E-06	1.0579E-05	1.7521E-05	0.00001868	1.9979E-05	2.1257E-05	6.9053E-06	1.0035E-05	9.2547E-06	8.872E-06
Acholeplasma	4.2702E-05	1.0261E-05	1.0665E-05	4.5323E-05	7.4799E-05	1.3592E-06	4.3168E-05	5.86E-05	0.00000929	1.5096E-05	3.3599E-05	4.8558E-05	7.0424E-05	6.7673E-05	0.00010567	0.00012054
Acidaminococcus	2.2215E-05	1.8657E-05	7.0037E-06	1.9274E-05	1.3413E-05	3.6093E-05	1.7772E-05	2.0535E-05	2.3863E-05	2.2691E-05	2.1859E-05	3.4127E-05	0.0000126	0.00001819	1.7264E-05	1.6211E-05
Acinetobacter	2.0273E-05	9.882E-06	8.8609E-06	1.5102E-05	1.4436E-05	5.8935E-05	1.9067E-05	2.0929E-05	1.7023E-05	0.0000162	3.1691E-05	2.2345E-05	1.8037E-05	0.00002147	2.3478E-05	0.000028
Actinomyces	0.0000192	1.0148E-05	6.1537E-06	1.3822E-05	8.1985E-06	2.3959E-05	1.4305E-05	2.3915E-05	0.00001972	1.7748E-05	2.2578E-05	0.00001945	7.5447E-06	9.3347E-06	1.1496E-05	1.4683E-05
Acutalibacter	0.01199665	0.00781233	0.00263785	0.00749277	0.00761312	0.00986913	0.00718178	0.00584568	0.01123535	0.01127936	0.01047821	0.0121975	0.0062419	0.00798386	0.00666639	0.0057139
Adlercreutzia	0.00074185	4.1248E-05	4.7649E-05	0.00032833	0.00026178	1.1678E-05	0.00037571	0.00050432	0.00038216	0.00048328	0.00028264	0.00041353	0.0003085	0.00027297	0.0002339	0.00028529
Aeromonas	2.5773E-05	1.0383E-05	2.3388E-05	2.1594E-05	1.0998E-05	2.0523E-05	2.5946E-05	2.7654E-05	1.8279E-05	1.8624E-05	2.4027E-05	2.4908E-05	1.1012E-05	1.4331E-05	1.3418E-05	0.00001632
Akkermansia	0.01477394	0.35253087	0.0715679	0.01829554	0.00013357	0.00441157	0.0083385	0.00481901	0.0012384	0.00073626	0.00033855	0.00196553	0.00022746	9.4359E-05	9.7727E-05	0.00012435
Alcanivorax	5.1863E-05	1.7246E-05	3.7007E-06	0.00002554	3.0094E-05	1.1705E-05	1.4692E-05	0.00001339	0.00003365	2.1017E-05	2.4959E-05	5.5632E-05	2.6623E-05	8.3893E-06	1.1563E-05	2.1695E-05
Alistipes	0.04465338	0.00966337	0.06197917	0.05409288	0.01557105	0.016759	0.10274145	0.08898271	0.02647951	0.04025244	0.04112465	0.03941992	0.01943638	0.02206239	0.02841027	0.01925813
Alkaliphilus	2.8103E-05	4.8181E-06	4.5745E-06	2.3584E-05	1.0563E-05	2.0894E-06	4.8931E-06	1.1416E-05	1.7689E-05	1.5707E-05	1.6363E-05	2.3809E-05	1.5725E-05	1.7856E-05	1.2531E-05	1.3156E-05
Alloprevotella	0.00013258	8.095E-06	6.8494E-05	0.00012374	7.7593E-05	0.00012717	6.1339E-05	8.4797E-05	0.00016891	0.00019317	0.00020954	0.00016042	0.00011434	0.00011289	0.00010084	0.00010846
Amedibacterium	3.5327E-05	3.4672E-05	1.0743E-05	2.0342E-05	2.0422E-05	3.4101E-05	2.4558E-05	2.1997E-05	0.00003293	3.8027E-05	3.3836E-05	3.7208E-05	1.6407E-05	2.0191E-05	1.9888E-05	2.0913E-05
Aminipila	6.1258E-05	4.8217E-05	2.5853E-05	3.9764E-05	3.0969E-05	6.9201E-05	3.7769E-05	4.0838E-05	4.9436E-05	4.6491E-05	5.7293E-05	6.6993E-05	2.7627E-05	3.4762E-05	3.4513E-05	2.9863E-05
Anaerobutyricum	0.00049446	0.0003559	0.00010708	0.00025091	0.00019947	0.00026494	0.00026993	0.00022784	0.00064705	0.00090838	0.00045231	0.00037999	0.00020272	0.00021425	0.00019197	0.00025225
Anaerocolumna	0.00024509	0.00018347	0.00005881	0.00012243	0.000132	0.00010372	0.00015088	0.0001412	0.00024578	0.00033755	0.00024491	0.00024248	0.00010186	0.00014141	0.00011977	0.00012967
Anaerostipes	0.00044195	0.00032172	0.00078427	0.00085856	0.00024901	0.00028235	0.00025744	0.0002335	0.00043644	0.00067364	0.00039165	0.00042666	0.0001902	0.0002515	0.00021789	0.00024208
Anaerotignum	0.00011595	5.7087E-05	2.4437E-05	4.4475E-05	9.19E-05	6.225E-05	7.5705E-05	6.225E-05	0.0001112	0.00014588	0.00010637	9.8925E-05	6.7771E-05	7.5452E-05	0.00011073	8.1576E-05
Arabia	2.7309E-05	3.0488E-06	4.5333E-06	1.3028E-05	1.0882E-05	1.241E-06	1.0248E-05	2.2954E-05	1.5671E-05	1.7411E-05	1.4821E-05	1.6636E-05	1.2329E-05	1.2909E-05	1.2636E-05	1.7236E-05
Arcobacter	1.3572E-05	3.9498E-06	4.0984E-06	5.3018E-06	1.8236E-05	1.5535E-06	4.9054E-06	1.1372E-05	1.0389E-05	7.0582E-06	1.3096E-05	1.3674E-05	1.5953E-05	0.00001343	1.9759E-05	1.6679E-05
Arthrobacter	1.7578E-05	7.4678E-06	3.9965E-06	1.2158E-05	8.2869E-06	9.3728E-06	1.6868E-05	9.8746E-06	1.7049E-05	1.8707E-05	1.7559E-05	1.7176E-05	6.8899E-06	6.742E-06	7.6187E-06	8.9573E-06
Bacillus	0.00019539	0.00011653	6.467E-05	0.00012545	0.00012709	0.00011192	0.00014363	0.00015237	0.00029227	0.00018846	0.00021325	0.00021755	0.00011024	0.00012873	0.00014583	0.00013652
Bacteroides	0.10154641	0.31180842	0.33371876	0.18026388	0.13947885	0.0581296	0.09396419	0.07904107	0.24133157	0.16695371	0.19383297	0.17395719	0.16708489	0.14135133	0.12276552	0.12378393
Barnesiella	0.00060557	4.2886E-05	0.00028245	0.0005151	0.00023692	0.00048221	0.00049666	0.0005896	0.00045859	0.00045082	0.00055114	0.00043336	0.0003222	0.00031394	0.00031633	0.00037278
Bifidobacterium	0.02322658	0.0010826	0.00894325	0.00582556	0.04343249	0.00744305	0.05502008	0.05518203	0.0049689	0.00816816	0.02505075	0.01191056	0.03163097	0.04366004	0.05360969	0.04390584
Blattabacterium	1.0725E-05	3.4575E-06	3.9025E-06	6.5409E-06	9.8162E-06	1.4485E-06	8.1508E-06	1.3321E-05	0.000009	1.3906E-05	1.4175E-05	1.1208E-05	1.0859E-05	1.3181E-05	0.00001493	1.4271E-05
Blautia	0.00447936	0.02103479	0.00973958	0.01010777	0.00224349	0.00556276	0.00305234	0.00283289	0.00362371	0.00468397	0.00346426	0.00435993	0.00170862	0.00246541	0.0020278	0.00185303
Brachyspira	1.1842E-05	2.386E-06	2.0401E-06	2.9791E-06	3.0762E-05	3.5683E-06	8.2246E-06	1.1983E-05	7.8336E-06	5.9473E-06	8.9009E-06	1.0041E-05	2.1732E-05	2.2192E-05	2.5878E-05	0.00002091
Bradyrhizobium	1.4675E-05	3.9541E-06	4.2029E-06	1.2771E-05	7.8769E-06	1.0803E-05	0.00000826	1.2661E-05	1.3975E-05	1.1638E-05	1.5909E-05	1.6143E-05	8.4653E-06	9.3427E-06	1.0177E-05	1.0007E-05
Brevibacillus	1.3106E-05	1.0857E-05	4.8273E-06	9.9682E-06	9.1792E-06	8.0646E-06	1.1262E-05	1.0062E-05	7.4073E-06	8.0573E-06	1.2123E-05	1.3645E-05	5.6693E-06	9.0853E-06	9.358E-06	9.3513E-06
Burkholderia	2.0091E-05	4.4827E-06	9.1265E-06	1.4534E-05	8.9662E-06	1.4682E-05	1.9722E-05	2.4145E-05	1.5511E-05	1.3431E-05	1.5105E-05	1.8709E-05	1.0103E-05	9.994E-06	1.0831E-05	1.3113E-05
Butyricimonas	0.00050905	0.00034143	0.00081642	0.00057404	0.00080141	0.00014996	0.00034237	0.00037236	0.00078421	0.00082997	0.00068941	0.00066592	0.00078057	0.00099169	0.0008137	0.00070503
Butyrivibrio	0.00047643	0.00029179	0.00010447	0.0002131	0.00028376	0.00024246	0.00034078	0.00026554	0.00053709	0.00074249	0.00040829	0.00043372	0.00022077	0.00026033	0.00020415	0.00025244
Caldicellulosiruptor	1.6047E-05	4.9824E-06	4.2234E-06	7.0045E-06	7.0438E-06	3.0007E-06	0.00000958	1.1467E-05	1.3669E-05	1.7462E-05	2.0755E-05	1.4475E-05	7.1853E-06	7.9667E-06	8.1073E-06	1.0948E-05
Campylobacter	4.0104E-05	1.5531E-05	1.0925E-05	2.3054E-05	8.9859E-05	1.3047E-05	2.5905E-05	3.3408E-05	2.3414E-05	2.1664E-05	2.9764E-05	3.2882E-05	0.00012002	8.0327E-05	9.0115E-05	8.2483E-05
Candidatus_Arthromitus	0.00106843	1.1117E-05	7.9644E-05	8.8613E-05	0.00021457	9.8017E-06	0.00010442	1.6007E-05	0.00020546	0.0002646	0.00036154	0.00014796	0.00031035	0.00034038	4.3851E-05	8.3308E-05
Candidatus_Izimaplasma	0.00001358	2.2091E-06	2.6915E-06	1.2905E-05	1.5851E-05	6.3531E-07	7.7219E-06	1.157E-05	5.7115E-06	5.9314E-06	9.4736E-06	0.0000145	1.6589E-05	1.5798E-05	2.1514E-05	2.4968E-05
Candidatus_Nanosynbacter	3.0428E-05	1.8182E-06	1.2318E-05	2.4612E-05	1.9609E-05	8.8462E-08	2.5602E-05	2.1741E-05	1.8641E-05	2.3286E-05	3.6409E-05	3.3549E-05	1.6531E-05	2.5762E-05	2.4043E-05	1.7321E-05
Candidatus_Saccharimonas	3.4686E-05	1.5636E-06	1.4564E-05	2.5724E-05	2.2558E-05	1.8377E-07	2.4837E-05	2.7597E-05	2.1737E-05	2.5555E-05	3.7755E-05	3.5873E-05	1.8148E-05	2.7716E-05	0.00002894	2.0027E-05
Capnocytophaga	4.0791E-05	5.1432E-06	1.8955E-05	3.1564E-05	4.3177E-05	1.2791E-05	2.2717E-05	2.8985E-05	6.4345E-05	5.7718E-05	6.8582E-05	6.6155E-05	0.00004482	4.5113E-05	4.8381E-05	0.00004772
Caproiciproducens	9.8156E-05	5.3686E-05	2.6092E-05	7.128E-05	5.1529E-05	9.299E-05	6.2489E-05	6.7159E-05	0.00010553	8.1691E-05	9.5041E-05	0.00016242	6.0196E-05	6.0923E-05	5.4683E-05	4.6902E-05
Cellulosilyticum	0.00003039	1.8894E-05	7.6544E-06	1.7265E-05	0.00002401	1.0834E-05	2.2929E-05	0.00001966	2.7254E-05	2.9872E-05	0.00002935	2.8492E-05	1.5079E-05	2.9447E-05	2.2031E-05	1.7873E-05
Chitinophaga	6.401E-05	5.4327E-06	4.1294E-05	5.4486E-05	3.1277E-05	1.4065E-05	8.3455E-05	8.4379E-05	6.3131E-05	7.9784E-05	5.2055E-05	4.5791E-05	3.4887E-05	0.0000355	3.7433E-05	4.2493E-05
Chlorobaculum	3.3445E-05	1.6549E-06	1.3121E-05	2.5543E-05	1.2345E-05	8.608E-05	3.8192E-05	3.7277E-05	2.3441E-05	2.3565E-05	2.1336E-05	2.2195E-05	1.3033E-05	1.5313E-05	1.7631E-05	1.7767E-05
Christensenella	0.00019056	0.00010431	4.1446E-05	0.0001041	9.9552E-05	0.00012334	0.00012239	0.00011525	0.00019378	0.0002335	0.00016292	0.00016383	9.6255E-05	0.00012031	9.3955E-05	9.9944E-05
Chryseobacterium	7.5671E-05	1.5147E-05	4.2245E-05	6.0192E-05	3.6615E-05	0.00016306	6.7076E-05	8.4648E-05	6.1791E-05	5.7255E-05	0.00006612	6.4873E-05	4.2127E-05	4.7953E-05	5.1413E-05	0.0000565
Chryseolinea	1.3065E-05	1.2575E-06	1.1683E-05	1.8511E-05	4.1985E-06	5.9229E-06	2.4429E-05	2.4145E-05	9.0936E-06	8.4882E-06	1.0792E-05	1.0799E-05	4.7987E-06	5.856E-06	6.9473E-06	7.6267E-06
Citrobacter	1.2684E-05	3.9957E-06	2.1668E-05	8.9597E-06	5.2376E-06	1.4995E-05	6.0465E-06	6.5915E-06	0.00000589	4.6473E-06	7.6573E-06	9.1464E-06	3.8353E-06	4.3913E-06	4.9233E-06	4.9413E-06
Cloacibacillus	0.000029	2.1443E-05	5.8192E-06	2.1631E-05	2.2168E-05	3.4611E-05	1.9254E-05	2.1482E-05	3.1783E-05	2.4311E-05	2.6935E-05	0.0000301	1.5762E-05	2.4949E-05	1.8102E-05	1.7287E-05
Clostridioides	0.00126493	0.00139395	0.00042883	0.00083974	0.00055559	0.00097687	0.00075314	0.00086075	0.00107512	0.0009533	0.00116153	0.00113408	0.00052306	0.0007348	0.00057226	0.0004754
Clostridium	0.00073428	0.00048231	0.00024798	0.00039749	0.00051008	0.00035001	0.00056077	0.00063967	0.00063842	0.00082895	0.00070909	0.00071656	0.00051236	0.00072176	0.00066053	0.00061014
Collinsella	3.4145E-05	1.9961E-05	1.3715E-05	2.9035E-05	2.5102E-05	1.9948E-05	2.6006E-05	7.0182E-05	3.6727E-05	3.3136E-05	3.3845E-05	3.9518E-05	1.9086E-05	2.7553E-05	2.5127E-05	4.6528E-05
Corynebacterium	6.7491E-05	2.0586E-05	2.3718E-05	6.5083E-05	4.2262E-05	4.7657E-05	0.0002279	0.00028058	0.00157733	5.1355E-05	0.00027405	0.00020882	4.9133E-05	8.5015E-05	0.00048092	0.00031176
Curtobacterium	0.07283371	0.02756086	0.00290763	0.03084732	0.04376189	0.01770987	0.02007236	0.01635717	0.04884567	0.03658345	0.02854292	0.06743578	0.04258763	0.01287765	0.01513869	0.02612171
Cyclobacterium	0.0000122	1.8391E-06	0.00001118	1.1742E-05	1.0244E-05	5.3438E-06	2.4477E-06	3.2255E-06	1.6339E-05	1.2632E-05	1.4303E-05	1.2569E-05	1.0014E-05	1.1695E-05	9.6673E-06	7.476E-06
Dehalococcoides	8.0782E-06	8.0731E-06	2.7366E-06	1.7349E-05	5.3262E-06	8.3356E-06	8.6992E-06	1.0743E-05	7.6882E-06	6.1509E-06	1.1601E-05	2.7681E-05	9.0807E-06	1.1125E-05	1.1641E-05	0.00001036
Deinococcus	2.2055E-05	1.2183E-05	1.1912E-05	2.1698E-05	9.7638E-06	1.8856E-05	2.1766E-05	2.4698E-05	1.3559E-05	1.2716E-05	1.7592E-05	2.5545E-05	8.1533E-06	9.7353E-06	1.2002E-05	1.0972E-05
Desulfitobacterium	2.2336E-05	1.4458E-05	5.5216E-06	2.2395E-05	2.3097E-05	2.1747E-05	1.9525E-05	1.8715E-05	2.1325E-05	2.2445E-05	2.5719E-05	2.8345E-05	1.7355E-05	2.6182E-05	3.0853E-05	2.6651E-05
Desulfosarcina	1.4469E-05	8.4445E-06	6.6624E-06	1.3423E-05	5.5308E-06	1.2198E-05	8.9623E-06	7.9323E-06	0.00000981	8.4155E-06	1.2621E-05	1.4611E-05	4.7433E-06	6.618E-06	7.214E-06	0.000006
Desulfosporosinus	9.0555E-06	5.5687E-06	7.1902E-06	1.2165E-05	1.0954E-05	9.5572E-06	8.8554E-06	1.0227E-05	0.00001449	1.1712E-05	1.6346E-05	1.7126E-05	8.7987E-06	1.2417E-05	1.0845E-05	8.7407E-06
Desulfotomaculum	1.1409E-05	1.49E-05	4.6735E-06	1.5125E-05	9.4331E-06	1.7268E-05	1.0582E-05	1.3782E-05	8.3627E-06	8.7173E-06	1.1025E-05	1.5017E-05	8.5293E-06	1.0142E-05	1.2753E-05	1.2604E-05
Desulfovibrio	0.0003827	6.0268E-05	5.3713E-05	0.0002093	0.00036387	4.7245E-05	0.000101	0.00031648	0.00027908	0.00053312	0.00036849	0.00042754	0.00044537	0.00085318	0.00101601	0.00077616
Draconibacterium	1.2202E-05	1.6705E-06	4.9584E-06	1.1876E-05	8.6515E-06	9.3246E-07	8.8623E-06	9.9169E-06	1.2315E-05	0.00000977	1.2876E-05	1.2728E-05	0.00000774	9.9347E-06	1.0895E-05	1.0497E-05
Duncaniella	0.24443095	0.02017348	0.07307184	0.26076366	0.06875179	0.24742407	0.20910747	0.22325639	0.10440866	0.11930377	0.12232073	0.14935773	0.12709031	0.1017844	0.11226283	0.14556605
Dysgonomonas	1.7816E-05	2.0355E-06	8.1782E-06	1.4044E-05	1.9475E-05	1.2572E-06	5.483E-06	5.4938E-06	2.3782E-05	2.1045E-05	1.9918E-05	1.9518E-05	0.00002672	2.3633E-05	0.00002154	2.0821E-05
Dysosmobacter	0.00038457	0.00013236	7.0587E-05	0.00021185	0.0002297	0.00028042	0.00020458	0.00022118	0.00045066	0.00032825	0.00037737	0.00044273	0.00017535	0.00022382	0.00020146	0.00018261
Eggerthella	7.5131E-05	8.5378E-06	1.3792E-05	3.8721E-05	2.7696E-05	1.1587E-05	3.1615E-05	5.7008E-05	4.0227E-05	4.9764E-05	3.9373E-05	4.5964E-05	0.00003592	0.0000364	0.00003818	4.8367E-05
Enterobacter	1.4836E-05	5.2165E-06	0.00641677	0.00052773	6.5654E-06	4.6353E-06	1.4406E-05	1.9491E-05	1.2407E-05	1.3189E-05	1.3948E-05	1.3635E-05	5.7027E-06	8.816E-06	7.4553E-06	8.1564E-06
Enterocloster	0.00795584	0.00687571	0.00200929	0.00461459	0.00539779	0.00558886	0.00481074	0.00463382	0.00921191	0.01116572	0.0065036	0.00871487	0.00416534	0.005652	0.00426842	0.0040843
Enterococcus	0.00091817	0.00617607	0.00087909	0.00145591	0.00073232	0.01961568	0.00054272	0.00061328	0.0010413	0.00141687	0.00083722	0.00127339	0.00063486	0.00083886	0.0007936	0.00058626
Erysipelatoclostridium	0.00146013	0.00085732	0.00037227	0.00073569	0.00063305	0.00069744	0.00077473	0.00074729	0.00142816	0.00185971	0.00135084	0.00119003	0.00060766	0.00077181	0.00061404	0.00064891
Erysipelothrix	1.0921E-05	4.0615E-06	3.482E-06	1.2972E-05	1.8393E-05	1.7046E-05	1.1854E-05	1.4998E-05	7.9391E-06	9.8555E-06	1.5958E-05	1.6397E-05	2.2031E-05	2.1981E-05	3.0693E-05	3.5135E-05
Escherichia	3.4309E-06	1.0083E-06	3.9855E-05	7.0045E-06	2.1828E-06	1.0617E-06	3.7335E-06	4.1608E-06	4.1882E-06	3.1771E-06	3.8791E-06	3.3209E-06	3.0047E-06	2.3287E-06	2.8809E-06	4.1815E-06
Ethanoligenens	3.5336E-05	2.4024E-05	1.2963E-05	3.1336E-05	1.8367E-05	3.1141E-05	2.9836E-05	0.00002671	3.3791E-05	3.1118E-05	3.0691E-05	4.8052E-05	1.7143E-05	2.3591E-05	2.0711E-05	2.1215E-05
Eubacterium	0.00043917	0.00032201	0.00012222	0.00031309	0.00033093	0.00035462	0.00032411	0.00033706	0.00044304	0.0005683	0.00041133	0.00061963	0.00026117	0.00037197	0.0003066	0.0003016
Exiguobacterium	3.6542E-05	1.358E-05	3.9045E-06	1.6765E-05	1.9795E-05	2.5364E-05	1.1476E-05	1.1685E-05	2.1462E-05	1.6323E-05	2.4223E-05	3.926E-05	1.7597E-05	1.0887E-05	1.0893E-05	1.4538E-05
Faecalibacterium	0.00155144	0.00122089	0.00040545	0.00085561	0.0008233	0.00107228	0.00093738	0.00089931	0.00143307	0.00183303	0.00123047	0.00150182	0.00070428	0.00094313	0.00075117	0.00070336
Faecalibaculum	0.00119509	0.00020293	0.0002665	0.00067794	0.0019673	0.02595514	0.00383734	0.00092129	0.00134398	0.00108254	0.00203295	0.00145545	0.00207173	0.00209495	0.00339159	0.00275844
Faecalitalea	0.00010976	6.7879E-05	2.7212E-05	5.4676E-05	4.0566E-05	6.2289E-05	5.1203E-05	5.5226E-05	6.5436E-05	8.5173E-05	8.4608E-05	8.1689E-05	3.7845E-05	5.8322E-05	4.3531E-05	5.9468E-05
Fibrella	1.0265E-05	8.9909E-07	8.2883E-06	1.4639E-05	3.3402E-06	2.2753E-06	1.8827E-05	1.9852E-05	7.7818E-06	6.8318E-06	9.1418E-06	8.8345E-06	6.4333E-06	5.6027E-06	5.554E-06	6.1793E-06
Fibrobacter	1.3662E-05	4.9909E-07	3.5969E-06	8.9336E-06	0.00001233	1.4269E-06	5.7469E-06	5.7746E-06	1.4773E-05	0.00001425	0.00001032	9.7991E-06	1.8713E-05	1.5404E-05	1.2915E-05	0.00001275
Flavobacterium	5.063E-05	9.8151E-06	2.5536E-05	4.2336E-05	3.0869E-05	9.1454E-06	0.0000391	4.1862E-05	3.6127E-05	3.5855E-05	4.1027E-05	4.0891E-05	0.00002668	3.2207E-05	3.5127E-05	3.4853E-05
Flavonifractor	0.00289875	0.00203478	0.00060465	0.00185473	0.00184066	0.00462207	0.00161508	0.00177928	0.00312277	0.00290297	0.00272244	0.00366846	0.00151587	0.00212199	0.00175736	0.00152036
Flintibacter	0.00141346	0.00091127	0.00032725	0.00091434	0.00089592	0.00378471	0.00091635	0.0009818	0.00152378	0.00134631	0.00126137	0.00177041	0.0007646	0.00097195	0.000895	0.00078383
Formosa	5.2103E-06	1.3474E-06	1.4968E-06	8.8082E-07	8.2931E-06	9.7169E-07	2.9312E-06	2.2292E-05	6.5825E-06	8.7215E-06	6.0075E-06	6.3586E-06	1.9478E-05	2.5462E-05	2.2605E-05	0.00001533
Fusobacterium	2.9455E-05	1.3562E-05	1.3209E-05	2.3498E-05	4.8123E-05	8.5615E-06	2.5148E-05	3.7385E-05	2.0295E-05	2.4448E-05	3.3598E-05	3.7973E-05	4.6873E-05	5.8996E-05	6.6747E-05	6.3098E-05
Gemella	8.8173E-06	1.2347E-06	2.7639E-06	1.3821E-05	2.2163E-05	9.4123E-07	1.2088E-05	1.6574E-05	4.1319E-06	5.4427E-06	0.00001086	1.9265E-05	2.6592E-05	2.6019E-05	3.8729E-05	4.2802E-05
Geobacillus	9.5382E-06	6.5329E-06	4.5012E-06	1.2958E-05	5.7831E-06	1.1826E-05	7.7877E-06	1.0281E-05	1.0047E-05	7.9864E-06	1.1175E-05	1.4245E-05	5.942E-06	7.0647E-06	6.9207E-06	7.6993E-06
Geobacter	2.2682E-05	1.1899E-05	1.0659E-05	2.0865E-05	1.1975E-05	3.0092E-05	1.9952E-05	2.1638E-05	1.9925E-05	1.6334E-05	2.1565E-05	2.9445E-05	1.2141E-05	1.4918E-05	1.8397E-05	1.5161E-05
Gordonibacter	5.1273E-05	6.4944E-06	9.8166E-06	2.7523E-05	2.0324E-05	9.6068E-06	1.9608E-05	3.4531E-05	2.8791E-05	3.5618E-05	2.5109E-05	3.1827E-05	0.00002422	2.5667E-05	2.5667E-05	0.00003015
Haemophilus	2.4101E-06	1.1655E-07	5.8415E-07	1.6702E-06	1.0532E-06	0.00019306	7.9377E-07	1.698E-06	1.0599E-06	1.4421E-06	1.4219E-06	2.254E-06	1.3193E-06	1.8134E-06	9.9127E-07	1.6507E-06
Halomonas	0.00023351	8.6332E-05	1.4516E-05	0.00010728	0.00012262	6.6884E-05	7.6501E-05	6.8194E-05	0.00014954	0.00013014	9.2868E-05	0.00021034	0.00013276	5.0346E-05	5.8414E-05	8.8426E-05
Helicobacter	0.00014786	1.1456E-05	3.7532E-06	7.7818E-06	0.20763926	0.00032117	0.00000986	1.2916E-05	0.00011442	0.00001865	1.2464E-05	1.1857E-05	0.0925017	0.09356926	0.09166239	0.0757877
Herbinix	9.3788E-05	5.1846E-05	1.7533E-05	4.2045E-05	5.5022E-05	5.6743E-05	6.2677E-05	5.3847E-05	0.00010485	0.00014996	8.3044E-05	8.9503E-05	3.9306E-05	5.4964E-05	4.2138E-05	4.7667E-05
Hungateiclostridium	1.7991E-05	1.6733E-05	7.535E-06	1.5203E-05	1.3051E-05	9.6923E-06	1.3803E-05	1.2301E-05	1.6049E-05	0.00001482	1.7059E-05	2.6025E-05	1.0605E-05	1.5867E-05	0.00001517	1.2991E-05
Hungatella	0.00035805	0.00044601	0.00012542	0.0002588	0.00021881	0.00031046	0.00026212	0.00022006	0.0003613	0.00044982	0.00029124	0.00036598	0.00016994	0.00023219	0.00018137	0.00016433
Hymenobacter	0.00032722	3.8529E-05	0.0002184	0.00031798	0.00010928	0.00013931	0.00035814	0.00037751	0.00022161	0.00024077	0.00025532	0.00020822	0.00014923	0.0001486	0.00015322	0.00016365
Intestinimonas	0.00078344	0.00043956	0.00017372	0.00052771	0.00051203	0.00115014	0.00043737	0.00049656	0.00087838	0.00079657	0.00076988	0.00101483	0.00042202	0.00058554	0.0004932	0.00043002
Jeotgalicoccus	2.0961E-06	2.1955E-07	1.7499E-05	3.7299E-06	7.3611E-06	6.4477E-07	1.7165E-05	1.4483E-05	2.5845E-05	2.1104E-06	1.6851E-05	8.2355E-06	4.8091E-06	2.5091E-06	2.2919E-05	2.3954E-05
Klebsiella	0.00189757	7.6195E-06	0.00023995	1.6672E-05	5.9162E-06	1.4195E-05	4.1288E-05	1.7477E-05	0.00115801	1.6675E-05	4.259E-05	4.5721E-05	5.2393E-06	0.0001069	5.7607E-06	6.4401E-05
Lachnoanaerobaculum	0.00015564	0.0001077	2.8154E-05	4.1535E-05	5.8679E-05	4.0143E-05	9.4725E-05	7.7865E-05	0.00017381	0.00028202	0.00013511	0.00010729	5.9288E-05	5.2877E-05	4.4757E-05	7.9355E-05
Lachnoclostridium	0.01811122	0.01884014	0.00509086	0.01033331	0.01148956	0.01596296	0.01220979	0.01007703	0.01750835	0.023786	0.01450018	0.01881719	0.00847954	0.01230058	0.00932024	0.0088016
Lachnospira	0.00024186	0.0001492	6.5867E-05	0.00010224	0.00014721	0.00011322	0.00018318	0.00017548	0.00027244	0.00034273	0.00023377	0.00020709	0.00012218	0.0001716	0.00013224	0.00013384
Lacrimispora	0.00026351	0.00027247	7.8892E-05	0.00017933	0.00012481	0.00016767	0.00017229	0.00015715	0.00023867	0.00032273	0.00021746	0.000269	0.00010068	0.00014599	0.00012152	0.00011735
Lactobacillus	0.02545169	0.05779787	0.00469892	0.00399953	0.01678307	0.00281374	0.00695313	0.01519163	0.00889931	0.00889173	0.00616283	0.00637544	0.01612911	0.00840792	0.01106533	0.01530449
Leptotrichia	9.5782E-06	5.3412E-06	4.7883E-06	1.2026E-05	1.4802E-05	2.2192E-06	9.3469E-06	1.3486E-05	2.3375E-05	1.6121E-05	2.2218E-05	1.8058E-05	1.5599E-05	1.7695E-05	1.8627E-05	1.5681E-05
Libanicoccus	8.4045E-06	2.6457E-06	7.6718E-06	1.3428E-05	1.1569E-05	1.0932E-05	8.8654E-06	5.8321E-05	1.6163E-05	1.3618E-05	1.9179E-05	1.9622E-05	1.0861E-05	1.4339E-05	1.8425E-05	3.3506E-05
Ligilactobacillus	0.00290825	0.00237347	0.00039868	0.00047117	0.00311457	0.18933937	0.00187139	0.00111103	0.00384935	0.00423803	0.00698421	0.00246377	0.00420942	0.00327728	0.002046	0.00265534
Limosilactobacillus	0.00305604	0.0043094	0.00139167	0.00228594	0.00459161	0.0235832	0.00228125	0.00191923	0.0026663	0.00326064	0.00225811	0.00236688	0.00377784	0.00198891	0.0021691	0.0026845
Listeria	1.4596E-05	7.7045E-06	4.3393E-06	9.5255E-06	1.1165E-05	1.1826E-05	1.3351E-05	9.2185E-06	2.5609E-05	1.0965E-05	0.00001342	1.4925E-05	8.8573E-06	1.1559E-05	1.1471E-05	8.642E-06
Longibaculum	0.00024712	0.0002437	7.6435E-05	9.3083E-05	4.3777E-05	4.0201E-05	8.1923E-05	9.0059E-05	0.00011842	9.9093E-05	0.00013671	0.00011364	5.3109E-05	7.9439E-05	5.5723E-05	5.2032E-05
Mahella	1.9784E-05	1.7411E-05	4.6351E-06	4.7464E-06	1.3627E-05	2.4465E-06	1.0792E-05	9.9369E-06	1.8021E-05	3.504E-05	1.6022E-05	1.6679E-05	1.0761E-05	1.2309E-05	1.0057E-05	1.1241E-05
Massilia	9.8336E-06	2.5647E-06	5.4216E-06	1.0769E-05	5.4897E-06	2.8168E-05	1.4531E-05	2.3318E-05	9.3055E-06	6.1782E-06	8.0309E-06	1.0404E-05	6.826E-06	6.3887E-06	9.258E-06	1.0142E-05
Massilistercora	0.00064713	0.00064247	0.00017863	0.00038221	0.0004287	0.00080847	0.00047415	0.00039765	0.0006437	0.00081307	0.00053785	0.0006723	0.00034584	0.00046267	0.0003857	0.00034688
Mediterraneibacter	0.00048246	0.00049506	0.00015861	0.00026393	0.00022115	0.00038966	0.00030326	0.00027692	0.00039323	0.00045952	0.00034767	0.00040055	0.00020223	0.00029895	0.00022183	0.00018384

Megasphaera	0.0000236	1.4289E-05	1.1643E-05	1.7779E-05	1.1542E-05	1.5109E-05	1.7722E-05	1.6908E-05	2.1153E-05	2.1552E-05	2.0391E-05	2.6575E-05	1.0795E-05	1.5453E-05	1.3075E-05	1.3251E-05
Mesorhizobium	7.5655E-06	3.6194E-06	2.2548E-06	1.5369E-05	5.1954E-06	2.2223E-05	2.0214E-05	2.2381E-05	7.4582E-06	6.7191E-06	1.2775E-05	1.3248E-05	6.2373E-06	6.5387E-06	9.6273E-06	8.878E-06
Microbacterium	1.3979E-05	3.2845E-06	1.0281E-05	1.7754E-05	7.0308E-06	1.7643E-05	2.4521E-05	2.5992E-05	9.4418E-06	1.0345E-05	1.1696E-05	1.3867E-05	7.8833E-06	8.6033E-06	1.0613E-05	1.0989E-05
Monoglobus	0.00001743	1.2857E-05	4.612E-06	1.2507E-05	1.4878E-05	2.3651E-05	1.3268E-05	1.3422E-05	1.5812E-05	1.9486E-05	1.8423E-05	2.2385E-05	1.3471E-05	1.9946E-05	1.6728E-05	1.1625E-05
Moorella	1.2741E-05	6.1272E-06	1.0862E-05	1.8473E-05	8.3969E-06	7.945E-06	1.6608E-05	2.0441E-05	1.5157E-05	1.5718E-05	1.9968E-05	0.0000203	9.4073E-06	1.1921E-05	1.1323E-05	1.0391E-05
Mucilaginibacter	6.0491E-05	6.0117E-06	2.6595E-05	5.1036E-05	2.1515E-05	8.5543E-05	5.1346E-05	5.2554E-05	4.4891E-05	4.7945E-05	4.9609E-05	4.3236E-05	2.9393E-05	2.9107E-05	3.2347E-05	3.4373E-05
Mucinivorans	4.6165E-05	6.204E-06	4.5093E-05	4.0397E-05	1.5429E-05	3.621E-05	3.9146E-05	5.1054E-05	0.00002495	3.2618E-05	3.6509E-05	3.4727E-05	1.7742E-05	0.00002244	2.2093E-05	2.5073E-05
Muribaculum	0.12109593	0.01330937	0.1648302	0.15773384	0.09160707	0.04069261	0.24607959	0.2514493	0.11936895	0.14073629	0.18805206	0.13371452	0.1360833	0.14163849	0.18947409	0.21187814
Mycoplasma	1.9429E-05	2.8415E-06	6.5475E-06	1.4683E-05	6.5875E-05	3.1769E-05	1.8795E-05	3.7592E-05	2.3591E-05	1.5105E-05	2.4584E-05	2.8523E-05	5.5575E-05	5.1784E-05	6.3699E-05	6.6751E-05
Neisseria	2.1427E-05	6.6995E-06	9.7855E-06	0.00002038	1.3078E-05	2.4585E-05	2.2538E-05	2.7938E-05	0.0000213	1.7954E-05	2.0322E-05	2.5827E-05	1.4093E-05	1.4408E-05	1.8679E-05	0.00001779
Nitrosomonas	8.5291E-06	1.3578E-06	1.7207E-06	4.3625E-06	1.1595E-05	4.0151E-05	1.2314E-05	1.9965E-05	1.3085E-05	9.4191E-06	1.2716E-05	0.00001008	1.4261E-05	1.2242E-05	1.6709E-05	1.9317E-05
Odoribacter	0.00096467	0.00032932	0.00038925	0.00059169	0.00088658	0.00031509	0.00099118	0.00124135	0.00115448	0.0011517	0.00131364	0.00138855	0.00077668	0.00107077	0.00140528	0.00103289
Olsenella	8.8751E-05	3.7131E-05	9.823E-05	0.00012648	0.00011769	9.1269E-05	9.7875E-05	0.00090747	0.0001925	0.00014797	0.0002412	0.0001864	0.00014105	0.00018784	0.00023568	0.00053635
Oscillibacter	0.0020155	0.00084819	0.00041474	0.00106335	0.00101874	0.00147016	0.00093107	0.00113261	0.00241925	0.00140768	0.00142831	0.00194385	0.00086462	0.00110006	0.00095442	0.00083399
Paenibacillus	0.0002923	0.00062191	0.00011414	0.00023326	0.00019439	0.00024886	0.00025727	0.00025736	0.0002783	0.00028888	0.00026862	0.00035396	0.00015383	0.00019531	0.0001798	0.00015909
Paludibacter	1.1229E-05	1.7077E-06	6.2182E-06	1.4739E-05	8.3762E-06	3.9123E-07	8.4862E-06	1.1042E-05	1.1525E-05	9.9618E-06	1.2328E-05	0.00001272	9.5627E-06	1.0967E-05	9.0327E-06	1.2524E-05
Pandoraea	7.1573E-06	1.7968E-06	7.3818E-06	9.3907E-06	7.5346E-06	1.5524E-05	2.1709E-05	2.2523E-05	0.00001099	1.0048E-05	7.8227E-06	9.5691E-06	9.1507E-06	6.658E-06	1.2785E-05	1.2173E-05
Parabacteroides	0.01368066	0.00648866	0.00963566	0.00949767	0.02805369	0.06488741	0.01772911	0.02278477	0.03983806	0.04059517	0.0417812	0.03839432	0.03962848	0.04419048	0.045406	0.03842449
Paraprevotella	0.00031482	7.3207E-05	0.0002832	0.00024638	0.00036966	7.0046E-05	0.00029364	0.00028203	0.00055213	0.00050068	0.00050352	0.00046789	0.00046851	0.00044081	0.00040858	0.00043254
Parolsenella	6.5973E-06	1.1245E-06	5.3933E-06	5.7901E-06	7.1562E-06	9.1377E-07	4.6169E-06	5.1679E-05	1.1992E-05	8.2036E-06	1.3747E-05	1.3652E-05	9.1233E-06	1.1061E-05	0.0000136	3.1674E-05
Pasteurella	0.00082907	0.00028143	3.1151E-05	0.00034495	0.00043564	0.00019783	0.00022715	0.00018413	0.00051462	0.00042581	0.00029941	0.00072578	0.00045481	0.00013963	0.00016875	0.00029924
Pectinatus	1.8382E-05	3.2355E-06	3.3155E-06	5.989E-06	6.6467E-06	4.9819E-06	1.0915E-05	1.0562E-05	2.1865E-05	3.5392E-05	2.1497E-05	1.7712E-05	6.0477E-06	5.5673E-06	4.9553E-06	9.0791E-06
Pedobacter	3.2391E-05	9.2905E-06	2.4704E-05	3.7682E-05	1.9108E-05	7.2068E-06	5.8034E-05	5.0046E-05	3.1718E-05	3.3364E-05	2.7009E-05	0.0000295	1.7263E-05	2.2915E-05	2.1273E-05	1.8133E-05
Pelobacter	1.9233E-05	3.1676E-06	1.7843E-05	2.4304E-05	9.9023E-06	9.4805E-06	4.3618E-05	3.9298E-05	1.9562E-05	1.4919E-05	1.8612E-05	0.0000139	9.2033E-06	1.1259E-05	1.4399E-05	1.0897E-05
Peptoclostridium	0.00001349	7.4324E-06	4.3459E-06	9.4064E-06	5.6346E-06	3.0955E-06	1.1712E-05	1.1793E-05	8.0618E-06	1.0519E-05	1.1087E-05	1.5061E-05	6.522E-06	1.1079E-05	1.0103E-05	9.8827E-06
Peptoniphilus	3.9733E-05	2.3937E-05	1.3006E-05	1.3378E-05	3.7808E-06	6.3402E-06	1.7008E-05	2.3256E-05	1.8173E-05	2.3535E-05	2.9115E-05	1.5144E-05	5.0441E-06	7.9533E-06	5.902E-06	1.1149E-05
Petrimonas	8.4967E-05	4.7818E-06	2.2761E-05	6.6382E-05	7.7765E-05	8.5408E-06	4.2354E-05	0.0000525	9.3467E-05	9.1156E-05	7.8476E-05	8.6841E-05	0.00011617	0.00010274	8.5901E-05	9.8233E-05
Phocaeicola	0.02198153	0.02587318	0.11874284	0.04340443	0.13227749	0.00145456	0.00154665	0.00167061	0.1006062	0.11550286	0.07923558	0.0838759	0.10394211	0.15266514	0.10750307	0.08268269
Pontibacter	4.5564E-05	7.111E-06	3.1444E-05	4.6364E-05	2.1362E-05	9.522E-05	3.6723E-05	0.0000449	3.2055E-05	2.5064E-05	3.8618E-05	3.5182E-05	2.0233E-05	2.4087E-05	2.5893E-05	2.8473E-05
Porphyromonas	0.0004153	5.7348E-05	0.00027869	0.00043357	0.00019769	0.00014643	0.00041718	0.00047434	0.00033756	0.00033489	0.00037186	0.00033733	0.00027669	0.00023494	0.0002527	0.00031782
Prevotella	0.00392725	0.00012962	0.00100886	0.00257549	0.00570143	0.00100764	0.00085447	0.00112106	0.0063918	0.00691417	0.0042222	0.00475909	0.008106	0.00714451	0.00582688	0.00583216
Proteiniphilum	2.5475E-05	1.3862E-06	1.0376E-05	2.7745E-05	1.3168E-05	1.8213E-05	2.3853E-05	2.9054E-05	1.9802E-05	1.8062E-05	2.3936E-05	2.4735E-05	1.6549E-05	1.8933E-05	2.4533E-05	2.6251E-05
Pseudobutyrivibrio	5.3659E-05	3.6277E-05	1.5375E-05	3.4636E-05	3.9029E-05	2.5518E-05	4.5138E-05	3.2963E-05	5.3486E-05	5.0759E-05	4.6985E-05	6.5335E-05	2.3102E-05	4.3157E-05	3.2816E-05	2.6827E-05
Pseudomonas	0.00022379	7.6598E-05	0.00010013	0.00018214	9.0629E-05	0.00020102	0.00025762	0.00018304	0.00014924	0.00013283	0.00016085	0.00019484	0.00011692	0.00010579	0.00011815	0.0001245
Rhizobium	1.8923E-05	5.936E-06	1.3028E-05	0.0000208	8.7131E-06	1.011E-05	2.1548E-05	2.1807E-05	1.3919E-05	0.00001313	1.6589E-05	1.8115E-05	1.0738E-05	9.2447E-06	1.2031E-05	1.1649E-05
Rhodococcus	2.0164E-05	3.7628E-06	1.1735E-05	1.7384E-05	6.8346E-06	1.3175E-05	2.2495E-05	2.2587E-05	1.7295E-05	1.3104E-05	1.7491E-05	1.7095E-05	7.952E-06	9.4527E-06	9.6687E-06	8.884E-06
Rodentibacter	1.9066E-06	9.2332E-07	4.7655E-07	2.74E-07	6.3596E-06	0.02162705	1.4807E-05	1.8885E-05	7.8727E-07	7.7255E-07	1.3403E-06	1.8036E-06	1.1464E-05	2.1552E-05	2.8854E-05	3.628E-05
Romboutsia	8.2488E-06	4.6911E-06	0.00070232	5.0873E-06	0.00000835	4.8225E-06	8.1523E-06	8.4554E-06	6.8607E-06	6.8455E-06	8.1691E-06	1.3341E-05	0.00057282	0.00040078	0.00119909	0.00145018
Roseburia	0.00167111	0.00168636	0.0004708	0.00107279	0.00129888	0.00120194	0.00137128	0.00100612	0.00156879	0.00196653	0.00135474	0.00187759	0.00088886	0.00125749	0.00098247	0.00085353
Ruminococcus	0.000168	0.00011287	5.5343E-05	0.00013856	0.00010145	0.00011828	0.00012269	0.00012944	0.00037131	0.00016241	0.00037378	0.00027999	0.00011029	0.00012079	0.00010295	9.4174E-05
Runella	1.6232E-05	1.7698E-06	7.2064E-06	1.2615E-05	5.9677E-06	7.5008E-07	3.5468E-06	8.2346E-06	1.1059E-05	1.0298E-05	1.1928E-05	1.3695E-05	8.9993E-06	8.7947E-06	7.004E-06	8.154E-06
Ruthenibacterium	0.00026522	0.0001937	6.8904E-05	0.00075249	0.000257	0.0001087	0.00014982	0.00033536	0.00019894	0.00016894	0.00019147	0.00022102	0.00022376	0.00027573	0.00028614	0.00039047
Salmonella	5.6136E-06	1.0912E-06	0.00010355	1.1065E-05	7.4085E-06	5.3054E-06	2.7855E-06	3.5823E-06	1.0165E-05	8.2645E-06	8.3245E-06	9.6755E-06	7.2493E-06	5.556E-06	7.284E-06	7.9753E-06
Selenomonas	4.9355E-05	3.6295E-05	0.00001667	3.6555E-05	2.9932E-05	4.3382E-05	4.0544E-05	4.2592E-05	4.2904E-05	4.3809E-05	4.3167E-05	6.1298E-05	2.4357E-05	3.1073E-05	2.5863E-05	2.9323E-05
Serratia	1.6635E-05	6.3963E-06	1.2241E-05	1.1426E-05	6.3577E-06	2.046E-05	1.2115E-05	1.4925E-05	1.1882E-05	7.9836E-06	1.1954E-05	1.4555E-05	4.6993E-06	6.9327E-06	7.1893E-06	7.6687E-06
Shewanella	1.1018E-05	2.1078E-06	4.2054E-06	7.8727E-06	9.8177E-06	1.2253E-05	2.4876E-05	2.4473E-05	8.8691E-06	8.0873E-06	0.00001122	1.2595E-05	9.034E-06	1.3459E-05	1.3859E-05	1.1037E-05
Slackia	1.6438E-05	6.6719E-06	3.9185E-06	1.2275E-05	9.5969E-06	9.5274E-06	8.3423E-06	1.1767E-05	9.7491E-06	1.2016E-05	0.00000941	1.5309E-05	8.384E-06	1.0687E-05	9.728E-06	9.1513E-06
Sphingobacterium	3.3182E-05	6.3348E-06	1.5545E-05	0.0000254	2.9931E-05	2.3012E-06	9.0538E-06	1.4665E-05	3.9036E-05	3.5682E-05	0.0000332	3.6482E-05	4.0373E-05	3.8207E-05	3.5287E-05	3.6147E-05
Sphingomonas	1.6962E-05	4.6567E-06	1.5953E-05	2.1532E-05	9.4085E-06	9.8754E-06	3.0111E-05	3.3924E-05	1.4496E-05	1.1901E-05	1.4847E-05	1.7623E-05	8.014E-06	9.5073E-06	0.00001316	1.1705E-05
Spirosoma	6.9745E-05	9.7089E-06	7.6016E-05	9.4778E-05	3.6215E-05	0.00002644	0.00015021	0.00016027	0.00006325	6.0283E-05	5.8309E-05	5.6564E-05	0.0000423	4.0173E-05	5.0873E-05	4.6827E-05
Sporosarcina	1.1836E-05	3.9981E-06	3.7001E-06	3.878E-06	5.7465E-06	6.7637E-06	9.3646E-06	1.1939E-05	8.3428E-05	1.0855E-05	2.2522E-05	1.2181E-05	4.7613E-06	4.2691E-06	7.2027E-06	8.0413E-06
Staphylococcus	0.00034134	0.00029948	0.00049755	0.00030651	0.00030238	0.00464241	0.00056577	0.00028443	0.00058461	0.00132803	0.00100548	0.00106785	0.00028664	0.0001572	0.00094006	0.00099316
Streptococcus	0.00034499	0.0003402	0.00011742	0.00029556	0.0002299	0.00079654	0.00026824	0.00027814	0.00034259	0.00034444	0.00038856	0.00038053	0.00022364	0.00029666	0.00023068	0.00022482
Streptomyces	0.00014542	7.0895E-05	7.0872E-05	0.00013023	6.6678E-05	0.00010972	0.00012487	0.00014732	0.0001163	0.00010301	0.00011714	0.00015776	7.7375E-05	8.2466E-05	9.105E-05	8.9461E-05
Succinivibrio	1.3334E-05	2.567E-06	4.1538E-06	5.3772E-06	4.9715E-06	3.9903E-05	1.7273E-05	1.4718E-05	1.1655E-05	1.3096E-05	1.2449E-05	1.1347E-05	6.3093E-06	4.852E-06	5.8527E-06	7.4787E-06
Sutterella	3.2936E-05	3.3903E-06	2.2942E-06	7.3851E-06	7.7171E-05	0.00032514	8.7572E-05	0.00013881	9.1448E-05	5.4434E-05	6.0312E-05	7.0646E-05	0.00010523	6.921E-05	0.00010896	0.00014016
Synechococcus	3.1555E-05	9.4087E-06	1.1175E-05	2.6365E-05	1.6678E-05	1.8289E-05	0.00002259	0.0000294	2.4173E-05	2.2427E-05	2.8991E-05	3.1559E-05	1.5543E-05	1.9316E-05	2.2107E-05	0.00002258
Syntrophobotulus	2.0427E-05	2.7402E-05	6.0937E-06	7.387E-06	1.2315E-05	7.0972E-06	7.4065E-06	9.0921E-06	1.4388E-05	1.3604E-05	9.0464E-06	1.6577E-05	0.00000707	1.2604E-05	8.7913E-06	7.3805E-06
Tannerella	0.00032681	5.0516E-05	0.00023048	0.0002975	0.00022691	0.00027233	0.00028649	0.00032885	0.00032058	0.00030775	0.00030849	0.00027064	0.00030066	0.00030094	0.00027311	0.00028753
Thermaerobacter	1.2025E-05	5.9143E-06	5.8074E-06	0.00001012	5.9477E-06	1.4869E-05	8.8415E-06	9.3154E-06	9.5091E-06	8.3736E-06	1.0141E-05	1.7049E-05	4.7333E-06	7.2707E-06	7.3047E-06	6.856E-06
Thermoanaerobacterium	0.00001451	9.6325E-06	4.5935E-06	7.4245E-06	1.0217E-05	3.3497E-06	1.2222E-05	1.3297E-05	0.0000135	1.2414E-05	1.3798E-05	1.5437E-05	7.7353E-06	1.8985E-05	1.2693E-05	9.8707E-06
Thermus	1.2177E-05	8.3061E-06	4.3234E-06	9.5545E-06	6.2054E-06	1.827E-05	0.00000767	9.1969E-06	1.3616E-05	9.4964E-06	9.9245E-06	1.5775E-05	7.3033E-06	1.0809E-05	7.7973E-06	7.594E-06
Treponema	3.7836E-05	2.0374E-05	1.6213E-05	4.2145E-05	3.7646E-05	5.4133E-05	4.9816E-05	4.8962E-05	4.0164E-05	3.9127E-05	4.0318E-05	5.0232E-05	3.1493E-05	4.1167E-05	3.9042E-05	3.2493E-05
Trueperella	1.9272E-05	1.1119E-05	4.1787E-06	1.7176E-05	2.2092E-05	1.8772E-05	1.3158E-05	1.4483E-05	2.2565E-05	2.2064E-05	2.0475E-05	2.9991E-05	1.8477E-05	2.0515E-05	1.6407E-05	0.00001762
Turicibacter	1.6121E-05	4.1981E-06	0.00036285	0.00036669	5.5258E-06	3.1591E-06	0.00113947	0.00080186	1.2545E-05	1.8571E-05	0.00105046	0.00043956	4.0635E-06	2.257E-05	0.00064804	0.00034234
Vibrio	0.0000161	5.2891E-06	5.8309E-06	1.4176E-05	1.1225E-05	1.6582E-05	1.1774E-05	1.4449E-05	1.6525E-05	1.3979E-05	2.0709E-05	0.00001889	1.1995E-05	1.2276E-05	1.5645E-05	1.6624E-05
